# Identify the Key Active Ingredients and Pharmacological Mechanisms of Compound XiongShao Capsule in Treating Diabetic Peripheral Neuropathy by Network Pharmacology Approach

**DOI:** 10.1155/2019/5801591

**Published:** 2019-05-09

**Authors:** Meixiang Yu, Xin Song, Wanhua Yang, Ziwei Li, Xiaoqin Ma, Chenxia Hao

**Affiliations:** ^1^Department of Pharmacy, Ruijin Hospital, Shanghai Jiaotong University School of Medicine, Shanghai 200025, China; ^2^Renji Hospital, Shanghai Jiaotong University School of Medicine, Shanghai 200120, China

## Abstract

Compound XiongShao Capsule (CXSC), a traditional herb mixture, has shown significant clinical efficacy against diabetic peripheral neuropathy (DPN). However, its multicomponent and multitarget features cause difficulty in deciphering its molecular mechanisms. Our study aimed to identify the key active ingredients and potential pharmacological mechanisms of CXSC in treating DPN by network pharmacology and provide scientific evidence of its clinical efficacy. CXSC active ingredients were identified from both the Traditional Chinese Medicine Systems Pharmacology database, with parameters of oral bioavailability ≥ 30% and drug-likeness ≥ 0.18, and the Herbal Ingredients' Targets (HIT) database. The targets of those active ingredients were identified using ChemMapper based on 3D-structure similarity and using HIT database. DPN-related genes were acquired from microarray dataset GSE95849 and five widely used databases (TTD, Drugbank, KEGG, DisGeNET, and OMIM). Next, we obtained candidate targets with therapeutic effects against DPN by mapping active ingredient targets and DPN-related genes and identifying the proteins interacting with those candidate targets using STITCH 5.0. We constructed an “active ingredients-candidate targets-proteins” network using Cytoscape 3.61 and identified key active ingredients and key targets in the network. We identified 172 active ingredients in CXSC, 898 targets of the active ingredients, 110 DPN-related genes, and 38 candidate targets with therapeutic effects against DPN. Three key active ingredients, namely, quercetin, kaempferol, and baicalein, and 25 key targets were identified. Next, we input all key targets into ClueGO plugin for KEGG enrichment and molecular function analyses. The AGE-RAGE signaling pathway in diabetic complications and MAP kinase activity were determined as the main KEGG pathway and molecular function involved, respectively. We determined quercetin, kaempferol, and baicalein as the key active ingredients of CXSC and the AGE-RAGE signaling pathway and MAP kinase activity as the main pharmacological mechanisms of CXSC against DPN, proving the clinical efficacy of CXSC against DPN.

## 1. Introduction

Diabetic peripheral neuropathy (DPN) is one of the most common complications of diabetes, affecting approximately 50% of people with diabetes [1–3]. Characterized by numbness, pain, paresthesia, and sensory loss, it becomes a major cause of disability and mortality [4–6]. Given the estimated global prevalence of diabetes of 693 million by 2045 [4], DPN is likely to affect as many as 346.5 million people worldwide, at a tremendous cost.

The pathogenesis of DPN is complex and multifactorial [7, 8]. It is generally accepted that chronic hyperglycemia induces the generation of advanced glycation end products (AGEs) in peripheral nervous tissue, and AGEs frequently result in neurological dysfunction mainly by modifying nervous structural proteins or overexpressing their receptors (RAGE) [9–11]. In addition, high glucose level activates mitogen-activated protein kinase (MAPK) in sensory neurons [8]. Moreover, elevated p38 and ERK activities may cause neuron apoptosis, affecting neuronal functions and accelerating the progress of DPN [8, 12].

Despite advancements in the understanding of DPN pathogenesis, prevention and therapy of DPN mainly focus on glucose control and lifestyle modification [13]. There is still a lack of therapies against DPN pathogenesis other than aldose reductase inhibitors, i.e., epalrestat, and antioxidants, i.e., alpha-lipoic acid [14, 15]. In addition, these therapies show modest efficacy in clinical practice and exhibit certain adverse reactions, such as nausea, vomiting, and dizziness [14, 16]. Thus, safe and effective treatments of DPN that act on its mechanisms are urgently needed [13].

Recently, Traditional Chinese Medicine (TCM) has shown efficacy in treating DPN [17, 18]. Compound XiongShao Capsule (CXSC) was produced from Buyang Huanwu Decoction which has been known for hundreds of years to possess significant neuroprotective properties [19–21] and certain clinical effect against DPN [22, 23]. CXSC has also been approved to use to treat DPN in Ruijin Hospital, Shanghai Jiao Tong University by the Shanghai Food and Drug Administration. CXSC is composed of 12 traditional Chinese herbs: Radix Paeoniae (RP), Radix Cyathulae (RC), Rhizoma Chuanxiong (RCX), Cortex Lyci (CL), Radix Saposhnikoviae (RS), Cassia Twig (CT),* Sargassum pallidum *(SP),* Polygonatum sibiricum *(PG), Astragali Radix (AgR),* Ramulus mori* (RM),* Silybum marianum *(SM), and* Orostachys fimbriata *(OF). CXSC has shown significant efficacy in clinical practice over several years, including improving whole-blood high-shear viscosity and improving incubation period and amplitude in the median or peroneal nerve of patients suffering from DPN [24]. Furthermore, some herbs in CXSC have been confirmed to exhibit positive pharmacological effects against DPN. For example, CL, AgR, and SM are involved in blood glucose control and show anti-inflammatory and inhibitory effects against oxidative stress [25–27].

**Figure 1:**
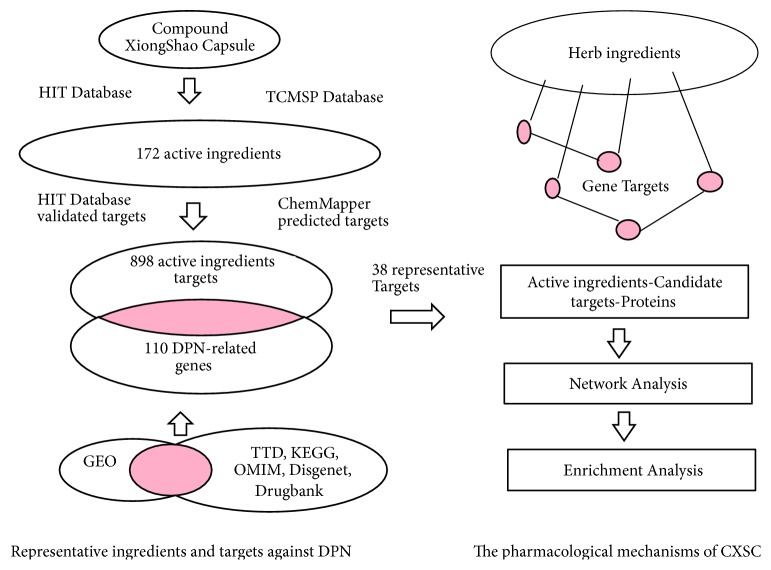
Flowcharts of the network pharmacology analysis. Left: summary of the identification of representative ingredients of CXSC and targets with therapeutic effects against DPN. Right: summary of the determination of the pharmacological mechanisms of CXSC.

However, the active ingredients of CXSC and their potential pharmacological mechanisms have not been fully studied. In a previous study, network pharmacology was used to discover the active ingredients and elucidate the mechanisms of herbal formulae [28]. Thus, our study aimed to use the network pharmacology approach to identify the key active ingredients of CXSC and their pharmacological mechanisms in treating DPN. A flowchart of the network pharmacology approach is presented in Figure 1.

## 2. Materials and Methods

### 2.1. Identification of Active Ingredients

Active ingredients of CXSC were collected both from the Traditional Chinese Medicine Systems Pharmacology (TCMSP) Database [29] (http://lsp.nwu.edu.cn/, updated on May 31, 2014) according to the ADME parameters: oral bioavailability (OB) > 30% and drug-likeness (DL) > 0.18 [30] and from the Herbal Ingredients' Targets (HIT) Database [31] (http://lifecenter.sgst.cn/hit/, downloaded on July 31, 2018). By combining these two databases and removing any overlapping data, the active ingredients of CXSC were identified.

The TCMSP database is a unique TCM platform for identifying the relationships between drugs, targets, and diseases [29]. HIT is a database containing herbal ingredients and validated protein targets derived from more than 3250 literatures [31].

### 2.2. Targets of Active Ingredients

The validated targets of the active ingredients of CXSC were collected from the HIT database [32, 33]. In addition, the predicted targets of these active ingredients were obtained by using ChemMapper (http://www.lilab-ecust.cn/chemmapper/index.html), a web server for predicting potential drug targets based on 3D structure similarity [34], with 3D structure similarity of above 1.0 and prediction score of above 0 [32, 33]. Duplicates of the validated and predicted targets were eliminated, and the targets of the active ingredients of CXSC were screened.

### 2.3. Genes Related to DPN Obtained

Known genes of DPN were identified from five currently available databases using “diabetic peripheral neuropathy” as the keyword. In addition, the main differentially expressed genes (DEGs) between DPN patients and diabetic patients were extracted from microarray data GSE95849 [35] in the Gene Expression Omnibus (GEO; http://www.ncbi.nlm.nih.gov/geo/) database [36], with a cut-off value of P < 0.05 and fold change |FC| of ≥ 1.5 [37]. DPN-related genes were identified after removal of duplicates.

The five databases were: the Therapeutic Target Database [38] (TTD, http://bidd.nus.edu.sg/group/cjttd/, last updated: 15th Sep. 2017); DrugBank [39] (http://www.drugbank.ca/, version:5.10); the Kyoto Encyclopedia of Genes and Genomes Pathway Database [40] (KEGG, https://www.kegg.jp/, downloaded:June. 2018); DisGeNET Database [41] (http://www.disgenet.org/web/DisGeNET/menu/home, version:5.0); Online Mendelian Inheritance in Man Database [42] (OMIM, http://www.omim.org/, last updated: 30th June. 2018).

### 2.4. Candidate Targets and Proteins Interacting with Candidate Targets

Candidate targets with therapeutic effects against DPN were identified by mapping the targets of the active ingredients of CXSC and the genes related to DPN [33]. The proteins that interacted with these candidate targets were screened by using STITCH 5.0 (http://stitch.embl.de/) [43], a database of known and predicted interactions between chemicals and proteins, with the species limited to “Homo Sapiens” and a confidence score of > 0.9.

### 2.5. Network Construction and Enrichment Analysis

A network of “active ingredients-candidate targets-proteins” was constructed using the Cytoscape 3.61 software [44]. The key active ingredients of CXSC and the key targets in this network were simultaneously identified by Network Analyzer plugin [45] using the following criterion: nodes with degree values exceeding twice the average value of all nodes in the network [46]. The degree value is the number of edges a node has in a network, which indicates how many active ingredients/targets/proteins one active ingredient/target/protein is related with. The larger the degree value, the more critical a role the node (active ingredient, target, or protein) is believed to play in the network [46]. A widely used visualization software, ClueGO [47], was used to explore the potential KEGG pathways and molecular functions of the key targets.

## 3. Results

### 3.1. Active Ingredients of CXSC

A total of 172 active ingredients (Supplementary Table 1) were identified in this study, of which 133 were collected from the TCMSP database and 39 were obtained from the HIT database. The numbers of active ingredients in RP, AgR, RCX, RS, CL, CT, PGS, SM, RM, RC, OF, and SP were 35, 22, 22, 21, 21, 13, 13, 13, 12, 7, 5, 5, and 4, respectively. The active ingredients that overlapped between herbs are shown in Table 1; for example, beta-sitosterol was identified in seven herbs, sitosterol was identified in six herbs, and quercetin was identified in five herbs.

### 3.2. Targets of the Active Ingredients of CXSC

**Figure 2:**
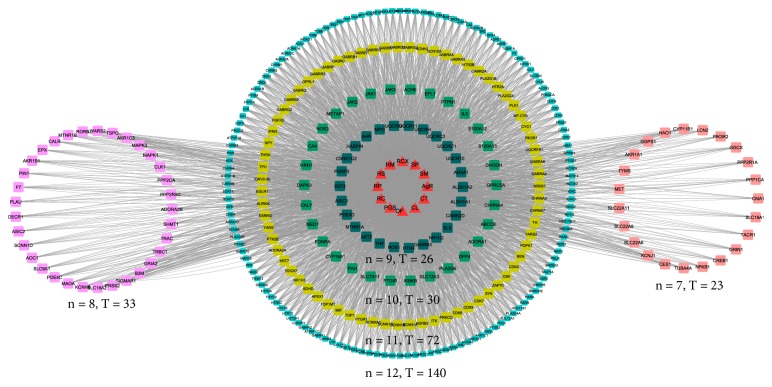
The targets that overlapped between at least 7 herbs. The triangle nodes represent the herbs, whereas the rectangle nodes represent the targets. The targets distributed in a circle are targeted by the same number of herbs. The number of herbs and number of targets are expressed as “n” and “T,” respectively.

A total of 898 targets of the active ingredients of CXSC (Supplementary Table 2) were identified in our study, including 229 validated targets and 669 predicted targets. Our analysis results showed that quercetin, baicalein, and kaempferol were the top three active ingredients targeting 308, 296, and 224 targets, respectively. Furthermore, there were 140 common targets of 12 herbs, 72 common targets of 11 herbs, 30 common targets of 10 herbs, 26 common targets of 9 herbs, 33 common targets of 8 herbs, and 23 common targets of 7 herbs. The common targets of at least 7 herbs are shown in Figure 2.

### 3.3. Genes Related to DPN

**Figure 3:**
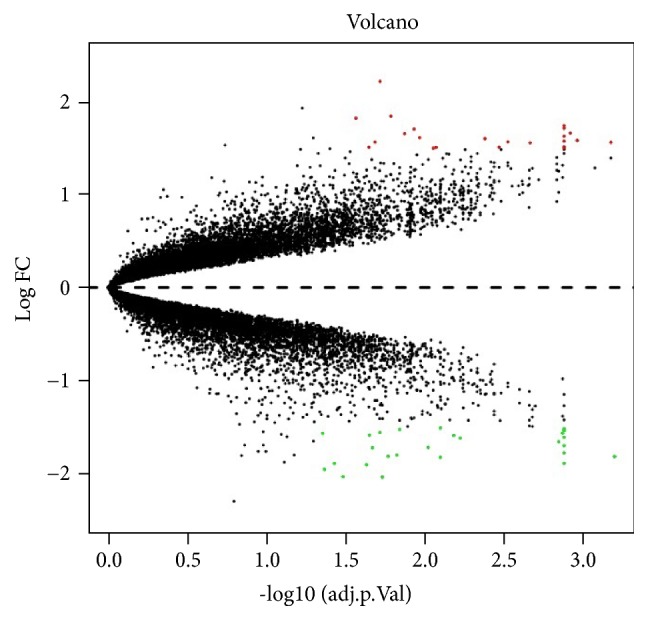
Volcano map of 51 DEGs. Red nodes represent upregulated genes and green nodes represent downregulated genes.

A total of 51 DEGs were extracted from microarray data GSE95849, including 23 upregulated genes and 28 downregulated genes, as shown in Figure 3. After combination with the 59 known targets from the other five databases, a total of 110 DPN-related genes were identified (Supplementary Table 3).

### 3.4. Candidate Targets and Interacting Proteins

A total of 38 candidate targets were shown to have potential pharmacological effects against DPN, including 11 predicted targets (INS, MAPK14, MMP2, NOS3, SCN9A, SLC6A4, SLC6A3, OPRM1, TUBB1, ABCC8, and KCNJ11) and 27 validated targets (AKT1, BAX, BCL2, CASP3, JUN, MAPK1, MAPK8, TNF, VEGFA, IL1B, IL6, PRKCA, PRKCB, RELA, SELE, STAT1, PLAU, CCND1, COL1A1, CXCL8, EDN1, F3, ICAM1, ACE, SLC6A2, and CYP1A2). Specifically, TNF, IL1B, IL6, and CXCL8 were involved in inflammatory reactions; AKT1, BAX, BCL2, CASP3, and MAPK8 were related to apoptosis; and MAPK1, MAPK8, and MAPK14 were associated with MAPK activity. In addition, there were 86 proteins that interacted with those candidate targets.

### 3.5. Key Active Ingredients and Key Targets in the Network

**Figure 4:**
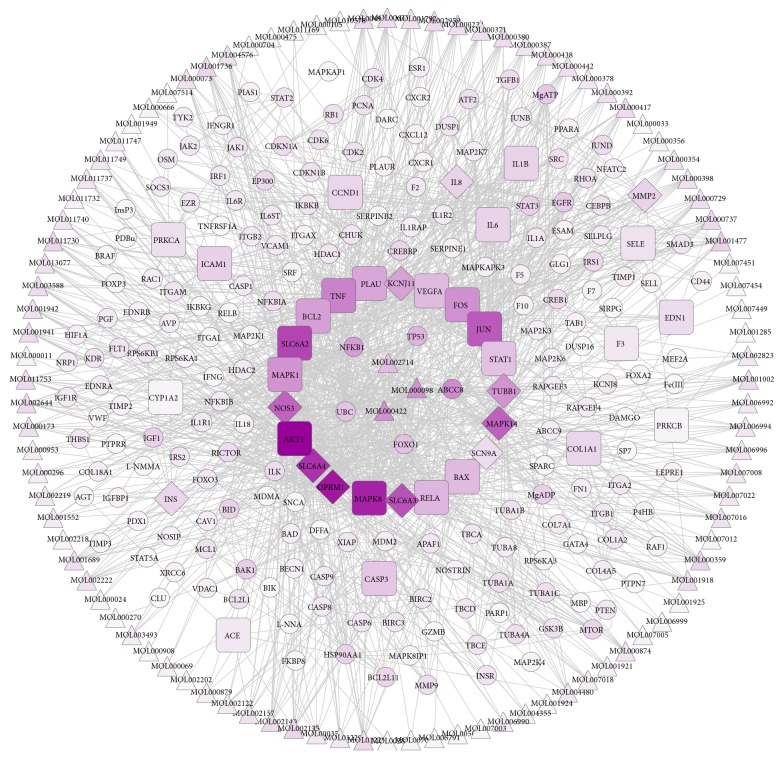
“Active ingredients-candidate targets-proteins” network. The triangle nodes represent active ingredients, the rectangle nodes represent validated targets, the diamond nodes represent predicted targets, and the circular nodes represent interacting proteins. The color of the nodes is shown in a gradient from purple to transparent according to descending order of the degree value. The three key active ingredients and 25 key targets are listed in the center circle.

**Figure 5:**
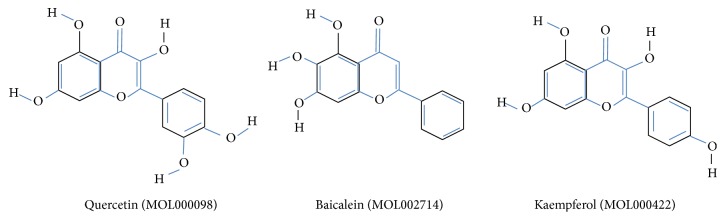
The chemical structure of the 3 key active ingredients.

An “active ingredients-candidate targets-proteins” network of 38 candidate targets and their interacting proteins was constructed using the Cytoscape 3.61 software (Figure 4). There were 328 nodes and 1396 edges in the network, with an average degree value of 8.767, as calculated by using the NetworkAnalyzer plugin. There were 28 nodes with degree values exceeding 18 (twice the average value of the degree), comprising of three active ingredients (quercetin, kaempferol, and baicalein) representing the key active ingredients (Figure 5) and 25 targets representing the key targets (Table 2).

### 3.6. KEGG Pathways and Molecular Functions Enriched

**Figure 6:**
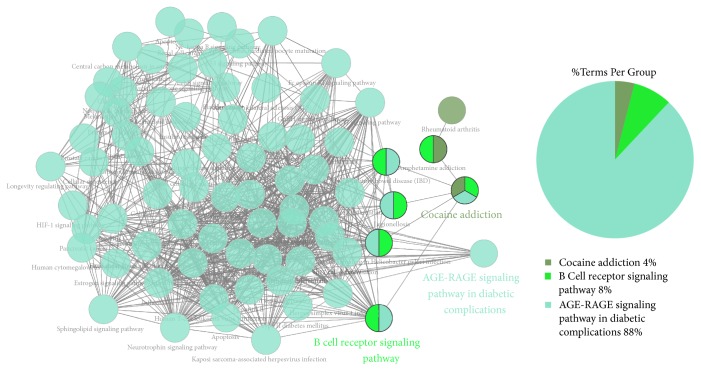
KEGG signaling pathways.

**Figure 7:**
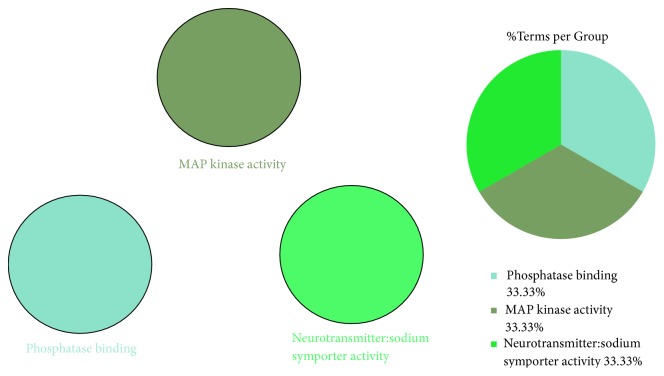
Molecular functions analysis.

The enrichment analysis results showed enrichment in the KEGG signaling pathways and molecular functions. As shown in Figure 6, the following three KEGG pathways were enriched: the AGE-RAGE signaling pathway in diabetic complications, B cell receptor signaling pathway, and cocaine addiction pathway, with the AGE-RAGE signaling pathway accounting for 88% of all the pathways enriched. The enriched molecular functions were divided into three groups: MAP kinase activity, phosphatase binding, and neurotransmitter: sodium symporter activity (Figure 7).

## 4. Discussion

TCM pharmacological mechanisms have always been associated with multiple components and multiple targets that are difficult to explain. However, the holistic ideas of TCM have been revealed by emerging network pharmacology approaches studying the relationships between drugs, targets, and diseases [28]. In our study, the active ingredients of CXSC, targets of these active ingredients, and genes related to DPN were identified comprehensively from the databases. Next, an “active ingredient-target-protein” network with 328 nodes and 1396 edges was constructed by the Cytoscape software. After analyzing the network, three key active ingredients and 25 key targets of CXSC were determined, and the pharmacological mechanisms of CXSC in treating DPN were elucidated by enrichment analysis of the 25 key targets.

### 4.1. Quercetin, Kaempferol, and Baicalein as the Key Active Ingredients of CXSC

**Figure 8:**
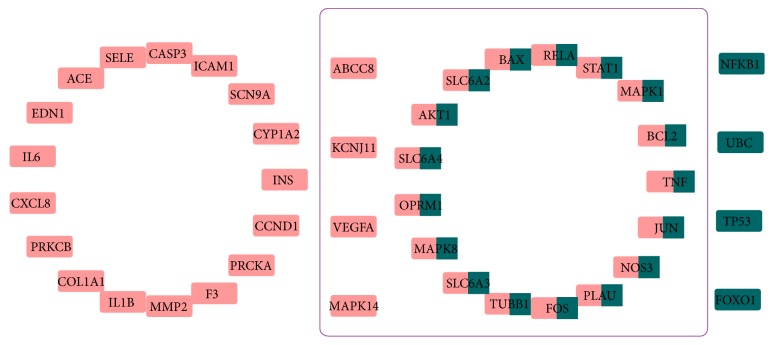
Data of the three key active ingredients (quercetin, kaempferol, and baicalein). Pink rectangles represent 38 candidate targets with therapeutic effects against DPN, whereas green rectangles represent the 25 key targets. Two-colored rectangles are targets that overlapped between the two categories. Targets in the purple box are targets of quercetin, kaempferol, and baicalein.

The “active ingredients-targets-proteins” network showed quercetin, kaempferol, and baicalein as the key active ingredients of CXSC that exhibit therapeutic effects against DPN. Moreover, these key ingredients targeted 308 (34.3%), 296 (33.0%), and 244 (27.2%) targets, respectively. Furthermore, these three key ingredients targeted 22 of the 38 candidate targets with therapeutic effects against DPN, as well as 21 of the 25 key targets of CXSC, as shown in Figure 8. Interestingly, several studies have previously shown that quercetin may be helpful against diabetic neuropathy by attenuating cold allodynia and hyperalgesia [48, 49], maintaining the density of the general neuronal population [50], and restoring sciatic nerves injuries [51] in streptozotocin- (STZ-) induced diabetic rats. Kaempferol [52] and baicalein [53] also showed neuroprotective effect against STZ-induced diabetic neuropathy. Thus, we speculated that CXSC exhibited significant therapeutic effect against DPN mainly through the synergistic actions of quercetin, kaempferol, and baicalein.

### 4.2. Regulations of the AGE-RAGE Pathway and MAPK Activity as the Pharmacological Mechanisms of CXSC Therapeutic Effect against DPN

Kaempferol and baicalein, the key active ingredients of CXSC, were previously confirmed to reduce the formation of AGEs, thereby reducing inflammatory responses in diabetic rat nerves [52, 54]; thus, these two compounds may exert therapeutic effect against DPN. Quercetin, another key active ingredient of CXSC, protected rat dorsal root ganglion neurons against high glucose-induced injury* in vitro* through dose-dependent inhibition of the NF-*κ*B signaling pathway; thus, this compound may be beneficial as a treatment of diabetic neuropathy [55]. Furthermore, activation and perpetuation of the AGE-RAGE signaling pathway have been reported in diabetic neuropathy [9]. AGEs act on RAGEs to allow sustained activation of NF-*κ*B, which aggravates inflammatory reactions and leads to neuronal dysfunction [9, 11]. Lukic I.K [10] identified the AGE-RAGE signaling pathway as a new therapeutic target of neuronal dysfunction. In our study, the AGE-RAGE signaling pathway of diabetic complications accounted for 88% of all the enriched pathways, suggesting that the therapeutic effect of CXSC against DPN was mainly related to reduction in AGE formation and inhibition of NF-*κ*B.

In addition, AGEs can activate p38 MAPK, leading to neuron apoptosis [56]. MAPK may play important roles in neuronal cell death or regeneration [57, 58]. For example, p38 MAPK was reported to aggravate oxidative stress and inflammation through induction of nitric oxide synthase, thereby resulting in NO production, as well as through regulation of the production of cytokines, such as TNF and IL-10, leading to neuronal cell death [59, 60]. Notably, increased phosphorylation of MAPK has been detected in diabetic animal models [61–63] and in the peripheral nerves of patients with DPN [12]. p38 MAPK, especially has been proved to be associated with DPN pathogenesis, such as increased mechanical hyperalgesia [64–66] and reduced nerve conduction velocity [67]. Thus, adjustment in the activity of MAPK may be the basis of preventive treatments of diabetic neuropathy [9]. Fortunately, tetramethylpyrazine, a main ingredient of RCX in CXSC, was found to block MAPK and suppress reactive oxygen species in N9 microglial cells, which may shed light on future treatments of neurodegenerative diseases [68]. Baicalein, one of the three key active ingredients identified in the current study, counteracts diabetes-associated p38 MAPK phosphorylation and oxidative-nitrosative stress, targeting several mechanisms implicated in DPN [53]. Therefore, the effects of CXSC on MAPK regulation against DPN may be attributed to tetramethylpyrazine and baicalein. Our molecular function analysis also indicated that the therapeutic effect of CXSC against DPN was related to MAPK activity. Our results were consistent with those of previous studies, which indicated that p38 MAPK activation mediates RAGE-induced, NF-*κ*B-dependent secretion of proinflammatory cytokines, leading to accelerated inflammation. Activation of the AGE-RAGE pathway and MAPK appeared to be present not only in DPN but also in diabetic nephropathy [69] and diabetic keratopathy [56]. Thus, further studies are needed to investigate whether CXSC is effective against other diabetic complications.

## 5. Conclusions

In conclusion, the multicomponent and multitarget features of the therapeutic effects of CXSC against DPN were effectively elucidated through network pharmacology approach. Quercetin, kaempferol, and baicalein were determined as the key active ingredients of CXSC. In addition, the AGE-RAGE signaling pathway and regulation of MAPK activity were shown as the main pharmacological mechanisms of the therapeutic effects of CXSC against DPN, thereby providing scientific evidence of the clinical efficacy of CXSC against DPN.

## Figures and Tables

**Table 1:** The number of overlapped active ingredients between herbs.

Active ingredients	Total	Herbs
beta-sitosterol	7	*CT, CL, OF, PGS, RC, RP, RS*
Sitosterol	6	*CT, OF, PGS, RP, RS, RCX*
quercetin	5	*SM, AgR, OF, RC, SP*
Stigmasterol	3	*SM, CL, RP*
acetic acid	3	*AgR, CT, RP*
kaempferol	3	*AgR, OF, RM*
(-)-taxifolin	2	*SM, CT*
CLR	2	*SM, CL*
hederagenin	2	*AgR, CL*
FA	2	*AgR, RCX*
(+)-catechin	2	*CT, RP*
baicalein	2	*PGS, RP*
ecdysterone	2	*RC, RM*
Mandanol	2	*RS, RCX*

**Table 2:** The information about the 25 key targets.

Gene	UniProt	Description	Degree	Source
AKT1	P31749	RAC-alpha serine/threonine-protein kinase	83	validated
OPRM1	P35372	Mu-type opioid receptor	76	predicted
MAPK8	P45983	Mitogen-activated protein kinase 8	72	validated
SLC6A4	P31645	Sodium-dependent serotonin transporter	67	predicted
SLC6A2	P23975	Sodium-dependent noradrenaline transporter	60	validated
SLC6A3	Q01959	Sodium-dependent dopamine transporter	56	predicted
JUN	P05412	Transcription factor AP-1	53	validated
MAPK14	Q16539	Mitogen-activated protein kinase 14	51	predicted
NOS3	P29474	Nitric oxide synthase	50	predicted
TNF	P01375	Tumor necrosis factor	40	validated
ABCC8	Q09428	ATP-binding cassette sub-family C member 8	37	proteins
FOS	P01100	Proto-oncogene c-Fos	35	validated
TUBB1	Q9H4B7	Tubulin beta-1 chain	34	predicted
MAPK1	P28482	Mitogen-activated protein kinase 1	34	validated
NFKB1	P19838	Nuclear factor NF-kappa-B p105 subunit	31	proteins
BCL2	P10415	Apoptosis regulator Bcl-2	30	validated
KCNJ11	Q14654	ATP-sensitive inward rectifier potassium channel 11	29	predicted
PLAU	P00749	Urokinase-type plasminogen activator	28	validated
RELA	Q04206	Transcription factor p65	23	validated
TP53	P04637	Cellular tumor antigen p53	23	proteins
BAX	Q07812	Apoptosis regulator BAX	21	validated
VEGFA	P15692	Vascular endothelial growth factor A	21	validated
STAT1	P42224	Signal transducer and activator of transcription 1-alpha/beta	19	validated
FOXO1	Q12778	Forkhead box protein O1	19	proteins
UBC	P0CG48	Polyubiquitin-C	18	proteins

## Data Availability

The data of our research can be acquired from the Supplementary Materials uploaded with this article.

## Supplementary Materials

Supplementary Materials Supplementary Table 1. 172 active ingredients in Compound XiongShao Capsule. 172 active ingredients in Compound XiongShao Capsule were downloaded from both the Traditional Chinese Medicine Systems Pharmacology (TCMSP) Database (http://lsp.nwu.edu.cn/, updated on May 31, 2014) according to an ADME principle: oral bioavailability (OB) > 30% and drug-likeness (DL) > 0.18 and the Herbal Ingredients' Targets (HIT) Database (downloaded on July 31, 2018). Supplementary Table 2. 898 targets of active ingredients in Compound XiongShao Capsule. The validated targets of the active ingredients in Compound XiongShao Capsule were extracted from HIT database. At the same time, the predicted targets of the active ingredients, which were saved as “MOL2” file format and then inputted into the ChemMapper (http://www.lilab-ecust.cn/chemmapper/index.html, downloaded in August 2018), were selected if the 3D similarity was above 1.0 and the prediction score was >0 as well. Eliminate duplicates, respectively, the targets of active ingredients were obtained. Supplementary Table 3. 110 diabetic peripheral neuropathy-related genes. The main differentially expressed genes (DEGs) of diabetic peripheral neuropathy (DPN) were extracted from Microarray data GSE95849 including six diabetic samples and six DPN samples in the Gene Expression Omnibus database (GEO, http://www.ncbi.nlm.nih.gov/geo/) with cut-off values of P < 0.05 and fold change |*FC*| ≥ 1.5. Simultaneously, known genes related to DPN were obtained from five currently available databases using “diabetic peripheral neuropathy” as the keyword: (1) the Therapeutic Target Database (TTD) (http://bidd.nus.edu.sg/group/cjttd/, last updated:15th Sep.2017). (2) DrugBank (http://www.drugbank.ca/, version:5.10). (3) the Kyoto Encyclopedia of Genes and Genomes (KEGG) Pathway Database (https://www.kegg.jp/, downloaded:June. 2018) (4) DisGeNET Database (http://www.disgenet.org/web/DisGeNET/menu/home, version:5.0). (5) Online Mendelian Inheritance in Man (OMIM) Database (http://www.omim.org/, last updated:30th June. 2018). At last, DPN-related genes were collected after eliminating duplicates.
