# Supplementary material for: Identify the Key Active Ingredients and Pharmacological Mechanisms of Compound XiongShao Capsule in Treating Diabetic Peripheral Neuropathy by Network Pharmacology Approach
Source: Evid Based Complement Alternat Med. 2019 May 9;2019:5801591. doi: 10.1155/2019/5801591 (PMC6532326; doi:10.1155/2019/5801591)
Supplement: Supplementary Materials — Supplementary Table 1. 172 active ingredients in Compound XiongShao Capsule. 172 active ingredients in Compound XiongShao Capsule were downloaded from both the Traditional Chinese Medicine Systems Pharmacology (TCMSP) Database (http://lsp.nwu.edu.cn/, updated on May 31, 2014) according to an ADME principle: oral bioavailability (OB) > 30% and drug-likeness (DL) > 0.18 and the Herbal Ingredients' Targets (HIT) Database (downloaded on July 31, 2018). Supplementary Table 2. 898 targets of active ingredients in Compound XiongShao Capsule. The validated targets of the active ingredients in Compound XiongShao Capsule were extracted from HIT database. At the same time, the predicted targets of the active ingredients, which were saved as “MOL2” file format and then inputted into the ChemMapper (http://www.lilab-ecust.cn/chemmapper/index.html, downloaded in August 2018), were selected if the 3D similarity was above 1.0 and the prediction score was >0 as well. Eliminate duplicates, respectively, the targets of active ingredients were obtained. Supplementary Table 3. 110 diabetic peripheral neuropathy-related genes. The main differentially expressed genes (DEGs) of diabetic peripheral neuropathy (DPN) were extracted from Microarray data GSE95849 including six diabetic samples and six DPN samples in the Gene Expression Omnibus database (GEO, http://www.ncbi.nlm.nih.gov/geo/) with cut-off values of P < 0.05 and fold change |FC| ≥ 1.5. Simultaneously, known genes related to DPN were obtained from five currently available databases using “diabetic peripheral neuropathy” as the keyword: (1) the Therapeutic Target Database (TTD) (http://bidd.nus.edu.sg/group/cjttd/, last updated:15th Sep.2017). (2) DrugBank (http://www.drugbank.ca/, version:5.10). (3) the Kyoto Encyclopedia of Genes and Genomes (KEGG) Pathway Database (https://www.kegg.jp/, downloaded:June. 2018) (4) DisGeNET Database (http://www.disgenet.org/web/DisGeNET/menu/home, version:5.0). (5) Online Mendelian Inheritance in [file 5801591.f1.pdf]

Supplementary Tab1. 172 active ingredients in CXSC.

Source	English Name	Mol ID	Ingredients	pubchem ID	OB(%)	DL
TCMSP	Radix Paeoniae（RP）	MOL002714	baicalein	NA	33.52	0.21
TCMSP	Radix Paeoniae（RP）	MOL000492	(+)-catechin	9064	54.83	0.24
TCMSP	Radix Paeoniae（RP）	MOL001002	ellagic acid	NA	43.06	0.43
TCMSP	Radix Paeoniae（RP）	MOL006994	1-o-beta-d-glucopyranosyl-8-o-benzoylpaeonisuffrone_qt	NA	36.01	0.3
TCMSP	Radix Paeoniae（RP）	MOL002883	Ethyl oleate (NF)	5363269	32.4	0.19
TCMSP	Radix Paeoniae（RP）	MOL006992	(2R,3R)-4-methoxyl-distylin	NA	59.98	0.3
TCMSP	Radix Paeoniae（RP）	MOL001918	paeoniflorgenone	NA	87.59	0.37
TCMSP	Radix Paeoniae（RP）	MOL001925	paeoniflorin_qt	NA	68.18	0.4
TCMSP	Radix Paeoniae（RP）	MOL007005	Albiflorin_qt	NA	48.7	0.33
TCMSP	Radix Paeoniae（RP）	MOL007016	Paeoniflorigenone	NA	65.33	0.37
TCMSP	Radix Paeoniae（RP）	MOL007022	evofolinB	NA	64.74	0.22
TCMSP	Radix Paeoniae（RP）	MOL006996	1-o-beta-d-glucopyranosylpaeonisuffrone_qt	NA	65.08	0.35
TCMSP	Radix Paeoniae（RP）	MOL007008	4-ethyl-paeoniflorin_qt	NA	56.87	0.44
TCMSP	Radix Paeoniae（RP）	MOL007012	4-o-methyl-paeoniflorin_qt	NA	56.7	0.43
TCMSP	Radix Paeoniae（RP）	MOL006990	(1S,2S,4R)-trans-2-hydroxy-1,8-cineole-B-D-glucopyranoside	NA	30.25	0.27
TCMSP	Radix Paeoniae（RP）	MOL007018	9-ethyl-neo-paeoniaflorin A_qt	NA	64.42	0.3
TCMSP	Radix Paeoniae（RP）	MOL007014	8-debenzoylpaeonidanin	NA	31.74	0.45
TCMSP	Radix Paeoniae（RP）	MOL005043	campest-5-en-3beta-ol	173183	37.58	0.71
TCMSP	Radix Paeoniae（RP）	MOL000449	Stigmasterol	5280794	43.83	0.76
TCMSP	Radix Paeoniae（RP）	MOL004355	Spinasterol	5281331	42.98	0.76
TCMSP	Radix Paeoniae（RP）	MOL000358	beta-sitosterol	222284	36.91	0.75
TCMSP	Radix Paeoniae（RP）	MOL000359	sitosterol	12303645	36.91	0.75
TCMSP	Radix Paeoniae（RP）	MOL006999	stigmast-7-en-3-ol	NA	37.42	0.75
TCMSP	Radix Paeoniae（RP）	MOL002776	Baicalin	5281605	40.12	0.75
TCMSP	Radix Paeoniae（RP）	MOL001921	Lactiflorin	NA	49.12	0.8
TCMSP	Radix Paeoniae（RP）	MOL001924	paeoniflorin	NA	53.87	0.79
TCMSP	Radix Paeoniae（RP）	MOL007004	Albiflorin	NA	30.25	0.77
TCMSP	Radix Paeoniae（RP）	MOL007003	benzoyl paeoniflorin	NA	31.14	0.54
TCMSP	Radix Paeoniae（RP）	MOL007025	isobenzoylpaeoniflorin	NA	31.14	0.54
TCMSP	Radix Cyathulae（RC）	MOL000098	quercetin	NA	46.43	0.28
TCMSP	Radix Cyathulae（RC）	MOL012286	Betavulgarin	442668	68.75	0.39
TCMSP	Radix Cyathulae（RC）	MOL012298	Rubrosterone	NA	32.69	0.47
TCMSP	Radix Cyathulae（RC）	MOL000358	beta-sitosterol	NA	36.91	0.75
TCMSP	Rhizoma Chuanxio(RCX)	MOL002140	Perlolyrine	160179	65.95	0.27
TCMSP	Rhizoma Chuanxio(RCX)	MOL001494	Mandenol	5282184	42	0.19
TCMSP	Rhizoma Chuanxio(RCX)	MOL002151	senkyunone	NA	47.66	0.24
TCMSP	Rhizoma Chuanxio(RCX)	MOL002135	Myricanone	NA	40.6	0.51
TCMSP	Rhizoma Chuanxio(RCX)	MOL002157	wallichilide	NA	42.31	0.71
TCMSP	Rhizoma Chuanxio(RCX)	MOL000359	sitosterol	NA	36.91	0.75
TCMSP	Rhizoma Chuanxio(RCX)	MOL000433	FA	6037	68.96	0.71
TCMSP	Cortex Lycii(CL)	MOL001689	acacetin	5280442	34.97	0.24
TCMSP	Cortex Lycii(CL)	MOL001552	OIN	154417	45.97	0.19
TCMSP	Cortex Lycii(CL)	MOL002219	Atropine	NA	34.53	0.21
TCMSP	Cortex Lycii(CL)	MOL002222	sugiol	NA	36.11	0.28
TCMSP	Cortex Lycii(CL)	MOL001645	Linoleyl acetate	5319042	42.1	0.2
TCMSP	Cortex Lycii(CL)	MOL002218	scopolin	25791637	56.45	0.39
TCMSP	Cortex Lycii(CL)	MOL000953	CLR	5997	37.87	0.68
TCMSP	Cortex Lycii(CL)	MOL000449	Stigmasterol	NA	43.83	0.76

TCMSP	Cortex Lycii(CL)	MOL000296	hederagenin	NA	36.91	0.75
TCMSP	Cortex Lycii(CL)	MOL000358	beta-sitosterol	NA	36.91	0.75
TCMSP	Cortex Lycii(CL)	MOL002224	aurantiamide acetate	NA	58.38	0.59
TCMSP	Cortex Lycii(CL)	MOL002228	Kulactone	NA	45.44	0.82
TCMSP	Cortex Lycii(CL)	MOL001790	Linarin	5317025	39.84	0.71
TCMSP	Radix Saposhnikoviae（RS）	MOL001941	Ammidin	10212	34.55	0.22
TCMSP	Radix Saposhnikoviae（RS）	MOL001942	isoimperatorin	68081	45.46	0.23
TCMSP	Radix Saposhnikoviae（RS）	MOL003588	Prangenidin	69502	36.31	0.22
TCMSP	Radix Saposhnikoviae（RS）	MOL000173	wogonin	NA	30.68	0.23
TCMSP	Radix Saposhnikoviae（RS）	MOL011753	5-O-Methylvisamminol	441970	37.99	0.25
TCMSP	Radix Saposhnikoviae（RS）	MOL011730	11-hydroxy-sec-o-beta-d-glucosylhamaudol_qt	NA	50.24	0.27
TCMSP	Radix Saposhnikoviae（RS）	MOL002644	Phellopterin	98608	40.19	0.28
TCMSP	Radix Saposhnikoviae（RS）	MOL011749	phelloptorin	NA	43.39	0.28
TCMSP	Radix Saposhnikoviae（RS）	MOL001494	Mandenol	NA	42	0.19
TCMSP	Radix Saposhnikoviae（RS）	MOL011737	divaricatacid	NA	87	0.32
TCMSP	Radix Saposhnikoviae（RS）	MOL007514	methyl icosa-11,14-dienoate	5365566	39.67	0.23
TCMSP	Radix Saposhnikoviae（RS）	MOL013077	Decursin	442126	39.27	0.38
TCMSP	Radix Saposhnikoviae（RS）	MOL011740	divaricatol	NA	31.65	0.38
TCMSP	Radix Saposhnikoviae（RS）	MOL011747	ledebouriellol	NA	32.05	0.51
TCMSP	Radix Saposhnikoviae（RS）	MOL000011	(2R,3R)-3-(4-hydroxy-3-methoxy-phenyl)-5-methoxy-2-methylol-2,3-dihydropyrano[5,6-h][1,4]benzodioxin-9-one	NA	68.83	0.66
TCMSP	Radix Saposhnikoviae（RS）	MOL000358	beta-sitosterol	NA	36.91	0.75
TCMSP	Radix Saposhnikoviae（RS）	MOL000359	sitosterol	NA	36.91	0.75
TCMSP	Radix Saposhnikoviae（RS）	MOL011732	anomalin	6859585	59.65	0.66
TCMSP	CassiaTwig（CT）	MOL000073	ent-Epicatechin	NA	48.96	0.24
TCMSP	CassiaTwig（CT）	MOL000492	(+)-catechin	NA	54.83	0.24
TCMSP	CassiaTwig（CT）	MOL001736	(-)-taxifolin	712316	60.51	0.27
TCMSP	CassiaTwig（CT）	MOL004576	taxifolin	439533	57.84	0.27
TCMSP	CassiaTwig（CT）	MOL000358	beta-sitosterol	NA	36.91	0.75
TCMSP	CassiaTwig（CT）	MOL000359	sitosterol	NA	36.91	0.75
TCMSP	CassiaTwig（CT）	MOL011169	Peroxyergosterol	NA	44.39	0.82
TCMSP	Sargassum pallidum（SP）	MOL000098	quercetin	NA	46.43	0.28
TCMSP	Sargassum pallidum（SP）	MOL010580	Diglycol dibenzoate	8437	59.22	0.27
TCMSP	Sargassum pallidum（SP）	MOL010578	N-[(1S)-1-(benzyl)-2-[[(1S)-1-(benzyl)-2-hydroxy-ethyl]amino]-2-keto-ethyl]benzamide	185904	45.76	0.43
TCMSP	Sargassum pallidum（SP）	MOL005440	Isofucosterol	5281326	43.78	0.76
TCMSP	Polygonatum sibiricum(PGS)	MOL006331	4',5-Dihydroxyflavone	165521	48.55	0.19
TCMSP	Polygonatum sibiricum(PGS)	MOL001792	DFV	114829	32.76	0.18
TCMSP	Polygonatum sibiricum(PGS)	MOL004941	(2R)-7-hydroxy-2-(4-hydroxyphenyl)chroman-4-one	928837	71.12	0.18
TCMSP	Polygonatum sibiricum(PGS)	MOL002714	baicalein	NA	33.52	0.21

TCMSP	Polygonatum sibiricum(PGS)	MOL002959	3'-Methoxydaidzein	5319422	48.57	0.24
TCMSP	Polygonatum sibiricum(PGS)	MOL000546	diosgenin	NA	80.88	0.81
TCMSP	Polygonatum sibiricum(PGS)	MOL000358	beta-sitosterol	NA	36.91	0.75
TCMSP	Polygonatum sibiricum(PGS)	MOL000359	sitosterol	NA	36.91	0.75
TCMSP	Polygonatum sibiricum(PGS)	MOL009760	sibiricoside A_qt	NA	35.26	0.86
TCMSP	Polygonatum sibiricum(PGS)	MOL003889	methylprotodioscin_qt	NA	35.12	0.86
TCMSP	Polygonatum sibiricum(PGS)	MOL009766	zhonghualiaoine 1	NA	34.72	0.78
TCMSP	Polygonatum sibiricum(PGS)	MOL009763	(+)-Syringaresinol-O-beta-D-glucoside	NA	43.35	0.77
TCMSP	Astragali Radix（AgR）	MOL000392	formononetin	5280378	69.67	0.21
TCMSP	Astragali Radix（AgR）	MOL000417	Calycosin	5280448	47.75	0.24
TCMSP	Astragali Radix（AgR）	MOL000422	kaempferol	NA	41.88	0.24
TCMSP	Astragali Radix（AgR）	MOL000380	(6aR,11aR)-9,10-dimethoxy-6a,11a-dihydro-6H-benzofurano[3,2-c]chromen-3-ol	14077830	64.26	0.42
TCMSP	Astragali Radix（AgR）	MOL000098	quercetin	NA	46.43	0.28
TCMSP	Astragali Radix（AgR）	MOL000438	(3R)-3-(2-hydroxy-3,4-dimethoxyphenyl)chroman-7-ol	10380176	67.67	0.26
TCMSP	Astragali Radix（AgR）	MOL000239	Jaranol	NA	50.83	0.29
TCMSP	Astragali Radix（AgR）	MOL000442	1,7-Dihydroxy-3,9-dimethoxy pterocarpene	5316760	39.05	0.48
TCMSP	Astragali Radix（AgR）	MOL000371	3,9-di-O-methylnissolin	NA	53.74	0.48
TCMSP	Astragali Radix（AgR）	MOL000354	isorhamnetin	NA	49.6	0.31
TCMSP	Astragali Radix（AgR）	MOL000398	isoflavanone	NA	109.99	0.3
TCMSP	Astragali Radix（AgR）	MOL000378	7-O-methylisomucronulatol	15689652	74.69	0.3
TCMSP	Astragali Radix（AgR）	MOL000296	hederagenin	NA	36.91	0.75
TCMSP	Astragali Radix（AgR）	MOL000387	Bifendate	108213	31.1	0.67
TCMSP	Astragali Radix（AgR）	MOL000033	(3S,8S,9S,10R,13R,14S,17R)-10,13-dimethyl-17-[(2R,5S)-5-propan-2-yloctan-2-yl]-2,3,4,7,8,9,11,12,14,15,16,17-dodecahydro-1H-cyclopenta[a]phenanthren-3-ol	NA	36.23	0.78
TCMSP	Astragali Radix（AgR）	MOL000433	FA	NA	68.96	0.71
TCMSP	Astragali Radix（AgR）	MOL000211	Mairin	NA	55.38	0.78
TCMSP	Astragali Radix（AgR）	MOL000379	9,10-dimethoxypterocarpan-3-O-β-D-glucoside	NA	36.74	0.92
TCMSP	Astragali Radix（AgR）	MOL000439	isomucronulatol-7,2'-di-O-glucosiole	15689653	49.28	0.62
TCMSP	Astragali Radix（AgR）	MOL000374	5'-hydroxyiso-muronulatol-2',5'-di-O-glucoside	NA	41.72	0.69
TCMSP	Ramulus Mori(RM)	MOL000422	kaempferol	5280863	41.88	0.24
TCMSP	Ramulus Mori(RM)	MOL000737	morin	5281670	46.23	0.27
TCMSP	Ramulus Mori(RM)	MOL000729	Oxysanguinarine	NA	46.97	0.87
TCMSP	Silybum marianum(SM)	MOL000098	quercetin	NA	46.43	0.28
TCMSP	Silybum marianum(SM)	MOL001736	(-)-taxifolin	NA	60.51	0.27
TCMSP	Silybum marianum(SM)	MOL001439	arachidonic acid	444899	45.57	0.2
TCMSP	Silybum marianum(SM)	MOL000953	CLR	NA	37.87	0.68
TCMSP	Silybum marianum(SM)	MOL000449	Stigmasterol	NA	43.83	0.76
TCMSP	Silybum marianum(SM)	MOL007449	24-methylidenelophenol	5283640	44.19	0.75
TCMSP	Silybum marianum(SM)	MOL007454	silymonin	NA	81.81	0.8
TCMSP	Silybum marianum(SM)	MOL007455	silandrin	NA	64.14	0.94
TCMSP	Silybum marianum(SM)	MOL007451	silydianin	NA	59.65	0.76
TCMSP	Silybum marianum(SM)	MOL007180	vitamin-e	3476	32.29	0.7
TCMSP	Silybum marianum(SM)	MOL007457	isosilychristin	NA	30.32	0.4

TCMSP	Orostachys fimbriata（OF）	MOL000422	kaempferol	NA	41.88	0.24
TCMSP	Orostachys fimbriata（OF）	MOL000098	quercetin	NA	46.43	0.28
TCMSP	Orostachys fimbriata（OF）	MOL002823	Herbacetin	5280544	36.07	0.27
TCMSP	Orostachys fimbriata（OF）	MOL000358	beta-sitosterol	NA	36.91	0.75
TCMSP	Orostachys fimbriata（OF）	MOL000359	sitosterol	NA	36.91	0.75
HIT	Radix Paeoniae（RP）	MOL000096	(-)-catechin	NA		
HIT	Radix Paeoniae（RP）	MOL000874	paeonol	11092		
HIT	Radix Paeoniae（RP）	MOL002249	gallocatechin	NA		
HIT	Radix Paeoniae（RP）	MOL004480	acetic acid	176		
HIT	Radix Paeoniae（RP）	MOL006791	epigallocatechin	72277		
HIT	Radix Paeoniae（RP）	MOL007002	paeoniflorin	442534		
HIT	Radix Cyathulae（RC）	MOL002212	ecdysterone	5459840		
HIT	Rhizoma Chuanxio(RCX)	MOL000024	alpha-humulene	5281520		
HIT	Rhizoma Chuanxio(RCX)	MOL000069	palmitic acid	985		
HIT	Rhizoma Chuanxio(RCX)	MOL000270	3-carene	26049		
HIT	Rhizoma Chuanxio(RCX)	MOL000669	camphor	2537		
HIT	Rhizoma Chuanxio(RCX)	MOL000879	methyl palmitate	8181		
HIT	Rhizoma Chuanxio(RCX)	MOL000908	beta-elemene	10583		
HIT	Rhizoma Chuanxio(RCX)	MOL001285	octanol	957		
HIT	Rhizoma Chuanxio(RCX)	MOL002042	thymol	6989		
HIT	Rhizoma Chuanxio(RCX)	MOL002122	z-ligustilide	642376		
HIT	Rhizoma Chuanxio(RCX)	MOL002189	butylphthalide	61361		
HIT	Rhizoma Chuanxio(RCX)	MOL002202	tetramethylpyrazine	14296		
HIT	Rhizoma Chuanxio(RCX)	MOL003493	naphthalene	931		
HIT	Rhizoma Chuanxio(RCX)	MOL004358	linalool	6549		
HIT	Rhizoma Chuanxio(RCX)	MOL004479	o-cresol	335		
HIT	Rhizoma Chuanxio(RCX)	MOL011846	menthyl acetate	62335		
HIT	Radix Saposhnikoviae（RS）	MOL000666	hexanal	6184		
HIT	Radix Saposhnikoviae（RS）	MOL000667	1-hexanol	8103		
HIT	Radix Saposhnikoviae（RS）	MOL001949	panaxynol	5281149		
HIT	CassiaTwig（CT）	MOL000105	protocatechuic acid	72		
HIT	CassiaTwig（CT）	MOL000475	anethole	637563		
HIT	CassiaTwig（CT）	MOL000704	styrene	7501		
HIT	CassiaTwig（CT）	MOL000991	cinnamaldehyde	637511		
HIT	CassiaTwig（CT）	MOL002295	trans-cinnamic acid	无		
HIT	CassiaTwig（CT）	MOL004480	acetic acid	176		
HIT	Polygonatum sibiricum(PGS)	MOL006670	aucubigenin	163040		
HIT	Astragali Radix（AgR）	MOL000356	lupeol	259846		
HIT	Astragali Radix（AgR）	MOL004480	acetic acid	176		
HIT	Ramulus Mori(RM)	MOL000738	kuwanon c	5481958		
HIT	Ramulus Mori(RM)	MOL001788	adenine	190		
HIT	Ramulus Mori(RM)	MOL002212	ecdysterone	5459840		
HIT	Ramulus Mori(RM)	MOL005570	myoinositol	892		
HIT	Silybum marianum(SM)	MOL001477	silymarin	1548994		

Supplementary Tab.2 898 targets of active ingredients in CXSC

Herb Name	MOL ID	Ingredients	Description	Uniprot	Gene symbol	Predicting score / Validated
Radix Paeoniae（RP）	MOL000096	(-)-catechin	Krueppel-like factor 7	O75840	KLF7	Validated
Radix Paeoniae（RP）	MOL000096	(-)-catechin	Peroxisome proliferator-activated receptor gamma	P37231	PPARG	Validated
Radix Paeoniae（RP）	MOL000096	(-)-catechin	Fatty acid synthase	P49327	FASN	Validated
Radix Paeoniae（RP）	MOL000096	(-)-catechin	Hyaluronan synthase 2	Q92819	HAS2	Validated
Radix Paeoniae（RP）	MOL000358	beta-sitosterol	Retinoic acid-induced protein 3	Q8NFJ5	GPRC5A	0.01
Radix Paeoniae（RP）	MOL000359	sitosterol	Retinoic acid receptor RXR-alpha	P19793	RXRA	0.016
Radix Paeoniae（RP）	MOL000359	sitosterol	Nuclear receptor subfamily 1 group I member 3	Q14994	NR1I3	0.016
Radix Paeoniae（RP）	MOL000359	sitosterol	Elongation factor Tu GTP-binding domain-containing protein 1	Q7Z2Z2	EFL1	0.026
Radix Paeoniae（RP）	MOL000359	sitosterol	Potassium channel subfamily K member 1	O00180	KCNK1	0.032
Radix Paeoniae（RP）	MOL000359	sitosterol	D-HSCDK2	O75100	CA11	0.032
Radix Paeoniae（RP）	MOL000359	sitosterol	Cell division control protein 2 homolog	P06493	CDK1	0.032
Radix Paeoniae（RP）	MOL000359	sitosterol	Cell division protein kinase 5	Q00535	CDK5	0.032
Radix Paeoniae（RP）	MOL000359	sitosterol	Sodium channel protein type 5 subunit alpha	Q14524	SCN5A	0.032
Radix Paeoniae（RP）	MOL000359	sitosterol	Potassium channel subfamily K member 6	Q9Y257	KCNK6	0.032
Radix Paeoniae（RP）	MOL000359	sitosterol	Neuronal acetylcholine receptor subunit alpha-3	P32297	CHRNA3	0.033
Radix Paeoniae（RP）	MOL000359	sitosterol	Neuronal acetylcholine receptor subunit alpha-7	P36544	CHRNA7	0.033
Radix Paeoniae（RP）	MOL000359	sitosterol	Neuronal acetylcholine receptor subunit alpha-4	P43681	CHRNA4	0.033
Radix Paeoniae（RP）	MOL000359	sitosterol	Phosphatidylinositol 3-kinase regulatory subunit alpha	P27986	PIK3R1	0.034
Radix Paeoniae（RP）	MOL000359	sitosterol	Phosphatidylinositol-4,5-bisphosphate 3-kinase catalytic subunit gamma isoform	P48736	PIK3CG	0.034
Radix Paeoniae（RP）	MOL000359	sitosterol	Serine/threonine-protein kinase PLK1	P53350	PLK1	0.034
Radix Paeoniae（RP）	MOL000359	sitosterol	Dehydrogenase/reductase SDR family member 8	Q8NBQ5	HSD17B11	0.041
Radix Paeoniae（RP）	MOL000359	sitosterol	Ig kappa chain C region	P01834	IGKC	0.042
Radix Paeoniae（RP）	MOL000359	sitosterol	Ig gamma-1 chain C region	P01857	IGHG1	0.042
Radix Paeoniae（RP）	MOL000359	sitosterol	Ig gamma-2 chain C region	P01859	IGHG2	0.042
Radix Paeoniae（RP）	MOL000359	sitosterol	Platelet glycoprotein IX	P14770	GP9	0.054
Radix Paeoniae（RP）	MOL000359	sitosterol	Phospholipase A2	P04054	PLA2G1B	0.057
Radix Paeoniae（RP）	MOL000359	sitosterol	Phospholipase A2, membrane associated	P14555	PLA2G2A	0.057
Radix Paeoniae（RP）	MOL000359	sitosterol	Annexin A1	P04083	ANXA1	0.062
Radix Paeoniae（RP）	MOL000359	sitosterol	Nuclear receptor 0B1	P51843	NR0B1	0.062
Radix Paeoniae（RP）	MOL000359	sitosterol	3 beta-hydroxysteroid dehydrogenase/Delta 5-->4-isomerase type II	P26439	HSD3B2	0.064
Radix Paeoniae（RP）	MOL000359	sitosterol	Cytosolic phospholipase A2	P47712	PLA2G4A	0.066
Radix Paeoniae（RP）	MOL000359	sitosterol	Corticosteroid 11-beta-dehydrogenase isozyme 1	P28845	HSD11B1	0.067
Radix Paeoniae（RP）	MOL000359	sitosterol	3-oxo-5-alpha-steroid 4-dehydrogenase 2	P31213	SRD5A2	0.067
Radix Paeoniae（RP）	MOL000359	sitosterol	Microtubule-associated protein 2	P11137	MAP2	0.068
Radix Paeoniae（RP）	MOL000359	sitosterol	Prolactin receptor	P16471	PRLR	0.068
Radix Paeoniae（RP）	MOL000359	sitosterol	Gonadotropin-releasing hormone receptor	P30968	GNRHR	0.068
Radix Paeoniae（RP）	MOL000359	sitosterol	Microtubule-associated protein 1A	P78559	MAP1A	0.068
Radix Paeoniae（RP）	MOL000359	sitosterol	Gonadotropin-releasing hormone II receptor	Q96P88	GNRHR2	0.068
Radix Paeoniae（RP）	MOL000359	sitosterol	Nuclear receptor coactivator 1	Q15788	NCOA1	0.069
Radix Paeoniae（RP）	MOL000359	sitosterol	Bile salt sulfotransferase	Q06520	SULT2A1	0.091
Radix Paeoniae（RP）	MOL000359	sitosterol	Prostaglandin G/H synthase 2	P35354	PTGS2	0.115
Radix Paeoniae（RP）	MOL000359	sitosterol	DNA polymerase kappa	Q9UBT6	POLK	0.115
Radix Paeoniae（RP）	MOL000359	sitosterol	Nuclear receptor coactivator 2	Q15596	NCOA2	0.116
Radix Paeoniae（RP）	MOL000359	sitosterol	Aldo-keto reductase family 1 member C1	Q04828	AKR1C1	0.125
Radix Paeoniae（RP）	MOL000359	sitosterol	ATP-binding cassette transporter sub-family C member 8	Q09428	ABCC8	0.127
Radix Paeoniae（RP）	MOL000359	sitosterol	3 beta-hydroxysteroid dehydrogenase/Delta 5-->4-isomerase type I	P14060	HSD3B1	0.128
Radix Paeoniae（RP）	MOL000359	sitosterol	Nitric-oxide synthase, endothelial	P29474	NOS3	0.134
Radix Paeoniae（RP）	MOL000359	sitosterol	Cannabinoid receptor 2	P34972	CNR2	0.136
Radix Paeoniae（RP）	MOL000359	sitosterol	3-oxo-5-alpha-steroid 4-dehydrogenase 1	P18405	SRD5A1	0.145
Radix Paeoniae（RP）	MOL000359	sitosterol	Nuclear receptor coactivator 5	Q9HCD5	NCOA5	0.15

Radix Paeoniae（RP）	MOL000359	sitosterol	Estradiol 17-beta-dehydrogenase 1	P14061	HSD17B1	0.221
Radix Paeoniae（RP）	MOL000359	sitosterol	Androgen receptor	P10275	AR	0.224
Radix Paeoniae（RP）	MOL000359	sitosterol	Estrogen receptor beta	Q92731	ESR2	0.246
Radix Paeoniae（RP）	MOL000359	sitosterol	Glucocorticoid receptor	P04150	NR3C1	0.288
Radix Paeoniae（RP）	MOL000359	sitosterol	Mineralocorticoid receptor	P08235	NR3C2	0.376
Radix Paeoniae（RP）	MOL000359	sitosterol	Estrogen receptor	P03372	ESR1	0.93
Radix Paeoniae（RP）	MOL000359	sitosterol	Progesterone receptor	P06401	PGR	1
Radix Paeoniae（RP）	MOL000449	Stigmasterol	5-hydroxytryptamine 1A receptor	P08908	HTR1A	0.012
Radix Paeoniae（RP）	MOL000449	Stigmasterol	Alpha-2A adrenergic receptor	P08913	ADRA2A	0.012
Radix Paeoniae（RP）	MOL000449	Stigmasterol	D(2) dopamine receptor	P14416	DRD2	0.012
Radix Paeoniae（RP）	MOL000449	Stigmasterol	Alpha-2B adrenergic receptor	P18089	ADRA2B	0.012
Radix Paeoniae（RP）	MOL000449	Stigmasterol	Alpha-2C adrenergic receptor	P18825	ADRA2C	0.012
Radix Paeoniae（RP）	MOL000449	Stigmasterol	5-hydroxytryptamine 1D receptor	P28221	HTR1D	0.012
Radix Paeoniae（RP）	MOL000449	Stigmasterol	5-hydroxytryptamine 1B receptor	P28222	HTR1B	0.012
Radix Paeoniae（RP）	MOL000449	Stigmasterol	5-hydroxytryptamine 2C receptor	P28335	HTR2C	0.012
Radix Paeoniae（RP）	MOL000449	Stigmasterol	D(3) dopamine receptor	P35462	DRD3	0.012
Radix Paeoniae（RP）	MOL000449	Stigmasterol	Elongation factor Tu GTP-binding domain-containing protein 1	Q7Z2Z2	EFL1	0.024
Radix Paeoniae（RP）	MOL000449	Stigmasterol	Phosphatidylinositol 3-kinase regulatory subunit alpha	P27986	PIK3R1	0.031
Radix Paeoniae（RP）	MOL000449	Stigmasterol	Neuronal acetylcholine receptor subunit alpha-3	P32297	CHRNA3	0.031
Radix Paeoniae（RP）	MOL000449	Stigmasterol	Neuronal acetylcholine receptor subunit alpha-7	P36544	CHRNA7	0.031
Radix Paeoniae（RP）	MOL000449	Stigmasterol	Neuronal acetylcholine receptor subunit alpha-4	P43681	CHRNA4	0.031
Radix Paeoniae（RP）	MOL000449	Stigmasterol	Phosphatidylinositol-4,5-bisphosphate 3-kinase catalytic subunit gamma isoform	P48736	PIK3CG	0.031
Radix Paeoniae（RP）	MOL000449	Stigmasterol	Serine/threonine-protein kinase PLK1	P53350	PLK1	0.031
Radix Paeoniae（RP）	MOL000449	Stigmasterol	Dehydrogenase/reductase SDR family member 8	Q8NBQ5	HSD17B11	0.037
Radix Paeoniae（RP）	MOL000449	Stigmasterol	Ig kappa chain C region	P01834	IGKC	0.04
Radix Paeoniae（RP）	MOL000449	Stigmasterol	Ig gamma-1 chain C region	P01857	IGHG1	0.04
Radix Paeoniae（RP）	MOL000449	Stigmasterol	Ig gamma-2 chain C region	P01859	IGHG2	0.04
Radix Paeoniae（RP）	MOL000449	Stigmasterol	Retinoic acid receptor alpha	P10276	RARA	0.041
Radix Paeoniae（RP）	MOL000449	Stigmasterol	Retinoic acid receptor gamma-1	P13631	RARG	0.041
Radix Paeoniae（RP）	MOL000449	Stigmasterol	Nuclear receptor coactivator 2	Q15596	NCOA2	0.041
Radix Paeoniae（RP）	MOL000449	Stigmasterol	3 beta-hydroxysteroid dehydrogenase/Delta 5-->4-isomerase type II	P26439	HSD3B2	0.06
Radix Paeoniae（RP）	MOL000449	Stigmasterol	Annexin A1	P04083	ANXA1	0.063
Radix Paeoniae（RP）	MOL000449	Stigmasterol	Nuclear receptor 0B1	P51843	NR0B1	0.063
Radix Paeoniae（RP）	MOL000449	Stigmasterol	Microtubule-associated protein 2	P11137	MAP2	0.064
Radix Paeoniae（RP）	MOL000449	Stigmasterol	Microtubule-associated protein 1A	P78559	MAP1A	0.064
Radix Paeoniae（RP）	MOL000449	Stigmasterol	Prolactin receptor	P16471	PRLR	0.065
Radix Paeoniae（RP）	MOL000449	Stigmasterol	3-oxo-5-alpha-steroid 4-dehydrogenase 2	P31213	SRD5A2	0.065
Radix Paeoniae（RP）	MOL000449	Stigmasterol	Cytosolic phospholipase A2	P47712	PLA2G4A	0.065
Radix Paeoniae（RP）	MOL000449	Stigmasterol	Estrogen receptor beta	Q92731	ESR2	0.065
Radix Paeoniae（RP）	MOL000449	Stigmasterol	Gonadotropin-releasing hormone receptor	P30968	GNRHR	0.066
Radix Paeoniae（RP）	MOL000449	Stigmasterol	Gonadotropin-releasing hormone II receptor	Q96P88	GNRHR2	0.066
Radix Paeoniae（RP）	MOL000449	Stigmasterol	Corticosteroid 11-beta-dehydrogenase isozyme 1	P28845	HSD11B1	0.067
Radix Paeoniae（RP）	MOL000449	Stigmasterol	Retinoic acid receptor RXR-alpha	P19793	RXRA	0.072
Radix Paeoniae（RP）	MOL000449	Stigmasterol	Nuclear receptor subfamily 1 group I member 3	Q14994	NR1I3	0.072
Radix Paeoniae（RP）	MOL000449	Stigmasterol	Bile salt sulfotransferase	Q06520	SULT2A1	0.087
Radix Paeoniae（RP）	MOL000449	Stigmasterol	Retinoic acid receptor RXR-beta	P28702	RXRB	0.094
Radix Paeoniae（RP）	MOL000449	Stigmasterol	cAMP-dependent protein kinase catalytic subunit alpha	P17612	PRKACA	0.115
Radix Paeoniae（RP）	MOL000449	Stigmasterol	DNA polymerase kappa	Q9UBT6	POLK	0.118
Radix Paeoniae（RP）	MOL000449	Stigmasterol	NADPH oxidase organizer 1	Q8NFA2	NOXO1	0.122
Radix Paeoniae（RP）	MOL000449	Stigmasterol	3 beta-hydroxysteroid dehydrogenase/Delta 5-->4-isomerase type I	P14060	HSD3B1	0.123
Radix Paeoniae（RP）	MOL000449	Stigmasterol	Aldo-keto reductase family 1 member C1	Q04828	AKR1C1	0.128
Radix Paeoniae（RP）	MOL000449	Stigmasterol	Mediator of RNA polymerase II transcription subunit 1	Q15648	MED1	0.129
Radix Paeoniae（RP）	MOL000449	Stigmasterol	Cannabinoid receptor 1	P21554	CNR1	0.13

Radix Paeoniae（RP）	MOL000449	Stigmasterol	Cannabinoid receptor 2	P34972	CNR2	0.137
Radix Paeoniae（RP）	MOL000449	Stigmasterol	Nuclear receptor coactivator 5	Q9HCD5	NCOA5	0.137
Radix Paeoniae（RP）	MOL000449	Stigmasterol	3-oxo-5-alpha-steroid 4-dehydrogenase 1	P18405	SRD5A1	0.142
Radix Paeoniae（RP）	MOL000449	Stigmasterol	Estradiol 17-beta-dehydrogenase 1	P14061	HSD17B1	0.214
Radix Paeoniae（RP）	MOL000449	Stigmasterol	Androgen receptor	P10275	AR	0.221
Radix Paeoniae（RP）	MOL000449	Stigmasterol	Nuclear receptor coactivator 1	Q15788	NCOA1	0.241
Radix Paeoniae（RP）	MOL000449	Stigmasterol	Glucocorticoid receptor	P04150	NR3C1	0.292
Radix Paeoniae（RP）	MOL000449	Stigmasterol	Mineralocorticoid receptor	P08235	NR3C2	0.377
Radix Paeoniae（RP）	MOL000449	Stigmasterol	Estrogen receptor	P03372	ESR1	0.983
Radix Paeoniae（RP）	MOL000449	Stigmasterol	Progesterone receptor	P06401	PGR	1
Radix Paeoniae（RP）	MOL000492	(+)-catechin	Catalase	P04040	CAT	Validated
Radix Paeoniae（RP）	MOL000874	paeonol	Tumor necrosis factor	P01375	TNF	Validated
Radix Paeoniae（RP）	MOL000874	paeonol	Amine oxidase [flavin-containing] A	P21397	MAOA	Validated
Radix Paeoniae（RP）	MOL000874	paeonol	Amine oxidase [flavin-containing] B	P27338	MAOB	Validated
Radix Paeoniae（RP）	MOL000874	paeonol	Interleukin-2	P60568	IL2	Validated
Radix Paeoniae（RP）	MOL000874	paeonol	Activator of 90 kDa heat shock protein ATPase homolog 1	O95433	AHSA1	Validated
Radix Paeoniae（RP）	MOL000874	paeonol	Intercellular adhesion molecule 1	P05362	ICAM1	Validated
Radix Paeoniae（RP）	MOL000874	paeonol	Apoptosis regulator Bcl-2	P10415	BCL2	Validated
Radix Paeoniae（RP）	MOL000874	paeonol	Tyrosinase	P14679	TYR	Validated
Radix Paeoniae（RP）	MOL000874	paeonol	NF-kappa-B inhibitor alpha	P25963	NFKBIA	Validated
Radix Paeoniae（RP）	MOL000874	paeonol	Mitogen-activated protein kinase 1	P28482	MAPK1	Validated
Radix Paeoniae（RP）	MOL000874	paeonol	RAC-alpha serine/threonine-protein kinase	P31749	AKT1	Validated
Radix Paeoniae（RP）	MOL000874	paeonol	Phosphatidylinositol-3,4,5-trisphosphate 3-phosphatase and dual-specificity protein phosphatase PTEN	P60484	PTEN	Validated
Radix Paeoniae（RP）	MOL000874	paeonol	Transcription factor p65	Q04206	RELA	Validated
Radix Paeoniae（RP）	MOL000874	paeonol	Apoptosis regulator BAX	Q07812	BAX	Validated
Radix Paeoniae（RP）	MOL001002	ellagic acid	Cystathionine beta-synthase	P35520	CBS	0.002
Radix Paeoniae（RP）	MOL001002	ellagic acid	Pyridoxal phosphate phosphatase	Q96GD0	PDXP	0.002
Radix Paeoniae（RP）	MOL001002	ellagic acid	Acetylcholine receptor subunit alpha	P02708	CHRNA1	0.008
Radix Paeoniae（RP）	MOL001002	ellagic acid	Cholinesterase	P06276	BCHE	0.008
Radix Paeoniae（RP）	MOL001002	ellagic acid	Acetylcholine receptor subunit gamma	P07510	CHRNG	0.008
Radix Paeoniae（RP）	MOL001002	ellagic acid	Acetylcholine receptor subunit beta	P11230	CHRNB1	0.008
Radix Paeoniae（RP）	MOL001002	ellagic acid	Neuronal acetylcholine receptor subunit beta-2	P17787	CHRNB2	0.008
Radix Paeoniae（RP）	MOL001002	ellagic acid	Neuronal acetylcholine receptor subunit alpha-5	P30532	CHRNA5	0.008
Radix Paeoniae（RP）	MOL001002	ellagic acid	Neuronal acetylcholine receptor subunit beta-4	P30926	CHRNB4	0.008
Radix Paeoniae（RP）	MOL001002	ellagic acid	Neuronal acetylcholine receptor subunit alpha-4	P43681	CHRNA4	0.008
Radix Paeoniae（RP）	MOL001002	ellagic acid	Acetylcholine receptor subunit epsilon	Q04844	CHRNE	0.008
Radix Paeoniae（RP）	MOL001002	ellagic acid	Neuronal acetylcholine receptor subunit beta-3	Q05901	CHRNB3	0.008
Radix Paeoniae（RP）	MOL001002	ellagic acid	Acetylcholine receptor subunit delta	Q07001	CHRND	0.008
Radix Paeoniae（RP）	MOL001002	ellagic acid	Neuronal acetylcholine receptor subunit alpha-6	Q15825	KCNJ8	0.008
Radix Paeoniae（RP）	MOL001002	ellagic acid	Neuronal acetylcholine receptor subunit alpha-10	Q9GZZ6	CHRNA10	0.008
Radix Paeoniae（RP）	MOL001002	ellagic acid	Neuronal acetylcholine receptor subunit alpha-9	Q9UGM1	CHRNA9	0.008
Radix Paeoniae（RP）	MOL001002	ellagic acid	Translocator protein	P30536	TSPO	0.009
Radix Paeoniae（RP）	MOL001002	ellagic acid	Voltage-dependent T-type calcium channel subunit alpha-1G	O43497	CACNA1G	0.01
Radix Paeoniae（RP）	MOL001002	ellagic acid	Carbonic anhydrase-related protein 11	O75493	CA11	0.01
Radix Paeoniae（RP）	MOL001002	ellagic acid	Voltage-dependent T-type calcium channel subunit alpha-1H	O95180	CACNA1H	0.01
Radix Paeoniae（RP）	MOL001002	ellagic acid	Carbonic anhydrase 3	P07451	CA3	0.01
Radix Paeoniae（RP）	MOL001002	ellagic acid	Glutathione S-transferase P	P09211	GSTP1	0.01
Radix Paeoniae（RP）	MOL001002	ellagic acid	Sphingomyelin phosphodiesterase	P17405	SMPD1	0.01
Radix Paeoniae（RP）	MOL001002	ellagic acid	Carbonic anhydrase 6	P23280	CA6	0.01
Radix Paeoniae（RP）	MOL001002	ellagic acid	Histamine H2 receptor	P25021	HRH2	0.01
Radix Paeoniae（RP）	MOL001002	ellagic acid	Amine oxidase [flavin-containing] B	P27338	MAOB	0.01
Radix Paeoniae（RP）	MOL001002	ellagic acid	5-hydroxytryptamine 1E receptor	P28566	HTR1E	0.01

Radix Paeoniae（RP）	MOL001002	ellagic acid	5-hydroxytryptamine 7 receptor	P34969	HTR7	0.01
Radix Paeoniae（RP）	MOL001002	ellagic acid	Carbonic anhydrase 5A, mitochondrial	P35218	CA5A	0.01
Radix Paeoniae（RP）	MOL001002	ellagic acid	Carbonic anhydrase-related protein	P35219	CA8	0.01
Radix Paeoniae（RP）	MOL001002	ellagic acid	Carbonic anhydrase 7	P43166	CA7	0.01
Radix Paeoniae（RP）	MOL001002	ellagic acid	5-hydroxytryptamine 6 receptor	P50406	HTR6	0.01
Radix Paeoniae（RP）	MOL001002	ellagic acid	Sodium channel subunit beta-1	Q07699	SCN1B	0.01
Radix Paeoniae（RP）	MOL001002	ellagic acid	Sodium channel protein type 9 subunit alpha	Q15858	SCN9A	0.01
Radix Paeoniae（RP）	MOL001002	ellagic acid	Opioid receptor, sigma 1	Q5T1J1	SIGMAR1	0.01
Radix Paeoniae（RP）	MOL001002	ellagic acid	Sodium channel subunit beta-4	Q8IWT1	SCN4B	0.01
Radix Paeoniae（RP）	MOL001002	ellagic acid	Carbonic anhydrase 13	Q8N1Q1	CA13	0.01
Radix Paeoniae（RP）	MOL001002	ellagic acid	Sodium channel protein type 2 subunit alpha	Q99250	SCN2A	0.01
Radix Paeoniae（RP）	MOL001002	ellagic acid	Sigma 1-type opioid receptor	Q99720	SIGMAR1	0.01
Radix Paeoniae（RP）	MOL001002	ellagic acid	Carbonic anhydrase-related protein 10	Q9NS85	CA10	0.01
Radix Paeoniae（RP）	MOL001002	ellagic acid	Sodium channel protein type 3 subunit alpha	Q9NY46	SCN3A	0.01
Radix Paeoniae（RP）	MOL001002	ellagic acid	Sodium channel subunit beta-3	Q9NY72	SCN3B	0.01
Radix Paeoniae（RP）	MOL001002	ellagic acid	D1 dopamine receptor-interacting protein calcyon	Q9NYX4	CALY	0.01
Radix Paeoniae（RP）	MOL001002	ellagic acid	Potassium voltage-gated channel subfamily D member 2	Q9NZV8	KCND2	0.01
Radix Paeoniae（RP）	MOL001002	ellagic acid	Voltage-dependent T-type calcium channel subunit alpha-1I	Q9P0X4	CACNA1I	0.01
Radix Paeoniae（RP）	MOL001002	ellagic acid	Sodium channel protein type 11 subunit alpha	Q9UI33	SCN11A	0.01
Radix Paeoniae（RP）	MOL001002	ellagic acid	Potassium voltage-gated channel subfamily D member 3	Q9UK17	KCND3	0.01
Radix Paeoniae（RP）	MOL001002	ellagic acid	Carbonic anhydrase 14	Q9ULX7	CA14	0.01
Radix Paeoniae（RP）	MOL001002	ellagic acid	Carbonic anhydrase 5B, mitochondrial	Q9Y2D0	CA5B	0.01
Radix Paeoniae（RP）	MOL001002	ellagic acid	Solute carrier family 12 member 2	P55011	SLC12A2	0.011
Radix Paeoniae（RP）	MOL001002	ellagic acid	Sodium/hydrogen exchanger 1	P19634	SLC9A1	0.012
Radix Paeoniae（RP）	MOL001002	ellagic acid	Amiloride-sensitive amine oxidase [copper-containing]	P19801	AOC1	0.012
Radix Paeoniae（RP）	MOL001002	ellagic acid	Amiloride-sensitive sodium channel subunit delta	P51172	SCNN1D	0.012
Radix Paeoniae（RP）	MOL001002	ellagic acid	Amiloride-sensitive cation channel 2, neuronal	P78348	ASIC1	0.012
Radix Paeoniae（RP）	MOL001002	ellagic acid	Amiloride-sensitive cation channel 1, neuronal	Q16515	ASIC2	0.012
Radix Paeoniae（RP）	MOL001002	ellagic acid	Phosphatidylinositol 3-kinase regulatory subunit beta	O00459	PIK3R2	0.013
Radix Paeoniae（RP）	MOL001002	ellagic acid	Mitogen-activated protein kinase 1	P28482	MAPK1	0.013
Radix Paeoniae（RP）	MOL001002	ellagic acid	Sodium channel protein type 1 subunit alpha	P35498	SCN1A	0.015
Radix Paeoniae（RP）	MOL001002	ellagic acid	Sodium channel protein type 4 subunit alpha	P35499	SCN4A	0.017
Radix Paeoniae（RP）	MOL001002	ellagic acid	5-hydroxytryptamine 4 receptor	Q13639	HTR4	0.017
Radix Paeoniae（RP）	MOL001002	ellagic acid	High affinity cAMP-specific and IBMX-insensitive 3',5'-cyclic phosphodiesterase 8A	O60658	PDE8A	0.018
Radix Paeoniae（RP）	MOL001002	ellagic acid	fMet-Leu-Phe receptor	P21462	FPR1	0.018
Radix Paeoniae（RP）	MOL001002	ellagic acid	Neuronal acetylcholine receptor subunit alpha-3	P32297	CHRNA3	0.018
Radix Paeoniae（RP）	MOL001002	ellagic acid	Neuronal acetylcholine receptor subunit alpha-7	P36544	CHRNA7	0.018
Radix Paeoniae（RP）	MOL001002	ellagic acid	6-phosphogluconate dehydrogenase, decarboxylating	P52209	PGD	0.018
Radix Paeoniae（RP）	MOL001002	ellagic acid	cAMP-specific 3',5'-cyclic phosphodiesterase 4C	Q08493	PDE4C	0.018
Radix Paeoniae（RP）	MOL001002	ellagic acid	Prostaglandin D2 receptor	Q13258	PTGDR	0.018
Radix Paeoniae（RP）	MOL001002	ellagic acid	High-affinity cAMP-specific 3',5'-cyclic phosphodiesterase 7A	Q13946	PDE7A	0.018
Radix Paeoniae（RP）	MOL001002	ellagic acid	cAMP-specific 3',5'-cyclic phosphodiesterase 7B	Q9NP56	PDE7B	0.018
Radix Paeoniae（RP）	MOL001002	ellagic acid	Cysteinyl leukotriene receptor 2	Q9NS75	CYSLTR2	0.018
Radix Paeoniae（RP）	MOL001002	ellagic acid	Cysteinyl leukotriene receptor 1	Q9Y271	CYSLTR1	0.018
Radix Paeoniae（RP）	MOL001002	ellagic acid	Elongation factor 2	P13639	EEF2	0.019
Radix Paeoniae（RP）	MOL001002	ellagic acid	Serine/threonine-protein kinase PLK1	P53350	PLK1	0.019
Radix Paeoniae（RP）	MOL001002	ellagic acid	Poly [ADP-ribose] polymerase 3	Q9Y6F1	PARP3	0.019
Radix Paeoniae（RP）	MOL001002	ellagic acid	Inosine-5'-monophosphate dehydrogenase 1	P20839	IMPDH1	0.02
Radix Paeoniae（RP）	MOL001002	ellagic acid	Acetylcholinesterase	P22303	ACHE	0.02
Radix Paeoniae（RP）	MOL001002	ellagic acid	Cytosolic purine 5'-nucleotidase	P49902	NT5C2	0.02
Radix Paeoniae（RP）	MOL001002	ellagic acid	Adenosine kinase	P55263	ADK	0.02
Radix Paeoniae（RP）	MOL001002	ellagic acid	Neuronal acetylcholine receptor subunit alpha-2	Q15822	CHRNA2	0.02

Radix Paeoniae（RP）	MOL001002	ellagic acid	DNA polymerase	P04293	UL30	0.021
Radix Paeoniae（RP）	MOL001002	ellagic acid	Thymidylate synthase	P04818	TYMS	0.021
Radix Paeoniae（RP）	MOL001002	ellagic acid	Ribonucleoside-diphosphate reductase large subunit	P23921	RRM1	0.021
Radix Paeoniae（RP）	MOL001002	ellagic acid	Amine oxidase [flavin-containing] A	P21397	MAOA	0.022
Radix Paeoniae（RP）	MOL001002	ellagic acid	5-hydroxytryptamine 1F receptor	P30939	HTR1F	0.022
Radix Paeoniae（RP）	MOL001002	ellagic acid	Sodium-dependent dopamine transporter	Q01959	SLC6A3	0.022
Radix Paeoniae（RP）	MOL001002	ellagic acid	Sodium channel protein type 5 subunit alpha	Q14524	SCN5A	0.022
Radix Paeoniae（RP）	MOL001002	ellagic acid	Glutamate [NMDA] receptor subunit 3A	Q8TCU5	GRIN3A	0.022
Radix Paeoniae（RP）	MOL001002	ellagic acid	cAMP and cAMP-inhibited cGMP 3',5'-cyclic phosphodiesterase 10A	Q9Y233	PDE10A	0.022
Radix Paeoniae（RP）	MOL001002	ellagic acid	Carbonic anhydrase 12	O43570	CA12	0.023
Radix Paeoniae（RP）	MOL001002	ellagic acid	Coagulation factor IX	P00740	F9	0.023
Radix Paeoniae（RP）	MOL001002	ellagic acid	Coagulation factor X	P00742	F10	0.023
Radix Paeoniae（RP）	MOL001002	ellagic acid	Osteocalcin	P02818	BGLAP	0.023
Radix Paeoniae（RP）	MOL001002	ellagic acid	Vitamin K-dependent protein C	P04070	PROC	0.023
Radix Paeoniae（RP）	MOL001002	ellagic acid	Coagulation factor VII	P08709	F7	0.023
Radix Paeoniae（RP）	MOL001002	ellagic acid	NAD(P)H dehydrogenase [quinone] 1	P15559	NQO1	0.023
Radix Paeoniae（RP）	MOL001002	ellagic acid	Vitamin K-dependent protein Z	P22891	PROZ	0.023
Radix Paeoniae（RP）	MOL001002	ellagic acid	Vitamin K-dependent gamma-carboxylase	P38435	GGCX	0.023
Radix Paeoniae（RP）	MOL001002	ellagic acid	Pterin-4-alpha-carbinolamine dehydratase	P61457	PCBD1	0.023
Radix Paeoniae（RP）	MOL001002	ellagic acid	Carbonic anhydrase 9	Q16790	CA9	0.023
Radix Paeoniae（RP）	MOL001002	ellagic acid	Vitamin K epoxide reductase complex subunit 1-like protein 1	Q8N0U8	VKORC1L1	0.023
Radix Paeoniae（RP）	MOL001002	ellagic acid	Vitamin K epoxide reductase complex subunit 1	Q9BQB6	VKORC1	0.023
Radix Paeoniae（RP）	MOL001002	ellagic acid	Serine/threonine-protein kinase 17B	O94768	STK17B	0.024
Radix Paeoniae（RP）	MOL001002	ellagic acid	ATP synthase subunit gamma, mitochondrial	P36542	ATP5F1C	0.024
Radix Paeoniae（RP）	MOL001002	ellagic acid	UDP-glucuronosyltransferase 3A1	Q6NUS8	UGT3A1	0.024
Radix Paeoniae（RP）	MOL001002	ellagic acid	Neurocan core protein	O14594	NCAN	0.025
Radix Paeoniae（RP）	MOL001002	ellagic acid	Sodium/potassium-transporting ATPase alpha-1 chain	P05023	ATP1A1	0.025
Radix Paeoniae（RP）	MOL001002	ellagic acid	Gamma-aminobutyric-acid receptor subunit alpha-4	P48169	GABRA4	0.025
Radix Paeoniae（RP）	MOL001002	ellagic acid	Solute carrier family 12 member 1	Q13621	SLC12A1	0.025
Radix Paeoniae（RP）	MOL001002	ellagic acid	Gamma-aminobutyric-acid receptor subunit alpha-6	Q16445	GABRA6	0.025
Radix Paeoniae（RP）	MOL001002	ellagic acid	Beta-3 adrenergic receptor	P13945	ADRB3	0.026
Radix Paeoniae（RP）	MOL001002	ellagic acid	Macrophage migration inhibitory factor	P14174	MIF	0.026
Radix Paeoniae（RP）	MOL001002	ellagic acid	Amiloride-sensitive sodium channel subunit alpha	P37088	SCNN1A	0.026
Radix Paeoniae（RP）	MOL001002	ellagic acid	Amiloride-sensitive sodium channel subunit beta	P51168	SCNN1B	0.026
Radix Paeoniae（RP）	MOL001002	ellagic acid	Amiloride-sensitive sodium channel subunit gamma	P51170	SCNN1G	0.026
Radix Paeoniae（RP）	MOL001002	ellagic acid	Tyrosyl-tRNA synthetase, mitochondrial	Q9Y2Z4	YARS2	0.026
Radix Paeoniae（RP）	MOL001002	ellagic acid	Gamma-aminobutyric-acid receptor subunit beta-3	P28472	GABRB3	0.027
Radix Paeoniae（RP）	MOL001002	ellagic acid	cAMP response element-binding protein	P16220	CREB1	0.028
Radix Paeoniae（RP）	MOL001002	ellagic acid	Nociceptin receptor	P41146	OPRL1	0.028
Radix Paeoniae（RP）	MOL001002	ellagic acid	Cyclin-dependent kinase 5 activator 1	Q15078	CDK5R1	0.028
Radix Paeoniae（RP）	MOL001002	ellagic acid	Tyrosine-protein kinase JAK2	O60674	JAK2	0.03
Radix Paeoniae（RP）	MOL001002	ellagic acid	Tyrosine-protein kinase JAK1	P23458	JAK1	0.03
Radix Paeoniae（RP）	MOL001002	ellagic acid	5-hydroxytryptamine 3 receptor	P46098	HTR3A	0.03
Radix Paeoniae（RP）	MOL001002	ellagic acid	Tyrosine-protein kinase JAK3	P52333	JAK3	0.03
Radix Paeoniae（RP）	MOL001002	ellagic acid	Dehydrogenase/reductase SDR family member 8	Q8NBQ5	HSD17B11	0.03
Radix Paeoniae（RP）	MOL001002	ellagic acid	Myeloperoxidase	P05164	MPO	0.031
Radix Paeoniae（RP）	MOL001002	ellagic acid	Eosinophil peroxidase	P11678	EPX	0.031
Radix Paeoniae（RP）	MOL001002	ellagic acid	Calreticulin	P27797	CALR	0.031
Radix Paeoniae（RP）	MOL001002	ellagic acid	Melatonin receptor type 1B	P49286	MTNR1B	0.031
Radix Paeoniae（RP）	MOL001002	ellagic acid	Nuclear receptor ROR-beta	Q92753	RORB	0.031
Radix Paeoniae（RP）	MOL001002	ellagic acid	Gamma-aminobutyric acid receptor subunit theta	Q9UN88	GABRQ	0.031
Radix Paeoniae（RP）	MOL001002	ellagic acid	Tyrosine-protein kinase Lyn	P07948	LYN	0.033
Radix Paeoniae（RP）	MOL001002	ellagic acid	Phosphatidylinositol 3-kinase regulatory subunit alpha	P27986	PIK3R1	0.033

Radix Paeoniae（RP）	MOL001002	ellagic acid	3-phosphoinositide-dependent protein kinase 1	O15530	PDPK1	0.034
Radix Paeoniae（RP）	MOL001002	ellagic acid	Tyrosine-protein kinase CSK	P41240	CSK	0.034
Radix Paeoniae（RP）	MOL001002	ellagic acid	Tyrosine-protein kinase ZAP-70	P43403	ZAP70	0.034
Radix Paeoniae（RP）	MOL001002	ellagic acid	Tyrosine-protein kinase SYK	P43405	SYK	0.034
Radix Paeoniae（RP）	MOL001002	ellagic acid	Protein kinase C theta type	Q04759	PRKCQ	0.034
Radix Paeoniae（RP）	MOL001002	ellagic acid	Tyrosine-protein kinase ITK/TSK	Q08881	ITK	0.034
Radix Paeoniae（RP）	MOL001002	ellagic acid	Alcohol dehydrogenase [NADP+]	P14550	AKR1A1	0.035
Radix Paeoniae（RP）	MOL001002	ellagic acid	Tryptophan 5-hydroxylase 1	P17752	TPH1	0.035
Radix Paeoniae（RP）	MOL001002	ellagic acid	3-oxo-5-alpha-steroid 4-dehydrogenase 1	P18405	SRD5A1	0.035
Radix Paeoniae（RP）	MOL001002	ellagic acid	3-oxo-5-alpha-steroid 4-dehydrogenase 2	P31213	SRD5A2	0.035
Radix Paeoniae（RP）	MOL001002	ellagic acid	Prostaglandin reductase 1	Q14914	PTGR1	0.035
Radix Paeoniae（RP）	MOL001002	ellagic acid	Mitogen-activated protein kinase 3	P27361	MAPK3	0.036
Radix Paeoniae（RP）	MOL001002	ellagic acid	Peroxisome proliferator-activated receptor delta	Q03181	PPARD	0.036
Radix Paeoniae（RP）	MOL001002	ellagic acid	Sterol O-acyltransferase 2	O75908	SOAT2	0.038
Radix Paeoniae（RP）	MOL001002	ellagic acid	Phospholipase A2	P04054	PLA2G1B	0.038
Radix Paeoniae（RP）	MOL001002	ellagic acid	Sterol O-acyltransferase 1	P35610	SOAT1	0.038
Radix Paeoniae（RP）	MOL001002	ellagic acid	Gamma-aminobutyric-acid receptor subunit beta-2	P47870	GABRB2	0.038
Radix Paeoniae（RP）	MOL001002	ellagic acid	Inhibitor of nuclear factor kappa-B kinase subunit alpha	O15111	CHUK	0.039
Radix Paeoniae（RP）	MOL001002	ellagic acid	Arachidonate 5-lipoxygenase	P09917	ALOX5	0.039
Radix Paeoniae（RP）	MOL001002	ellagic acid	Methionine aminopeptidase 1	P53582	METAP1	0.039
Radix Paeoniae（RP）	MOL001002	ellagic acid	Mitogen-activated protein kinase 8	P45983	MAPK8	0.04
Radix Paeoniae（RP）	MOL001002	ellagic acid	Mitogen-activated protein kinase 10	P53779	MAPK10	0.04
Radix Paeoniae（RP）	MOL001002	ellagic acid	cAMP-specific 3',5'-cyclic phosphodiesterase 4D	Q08499	PDE4D	0.04
Radix Paeoniae（RP）	MOL001002	ellagic acid	Peptidyl-prolyl cis-trans isomerase, mitochondrial	P30405	PPIF	0.041
Radix Paeoniae（RP）	MOL001002	ellagic acid	Gamma-aminobutyric acid receptor subunit gamma-2	P18507	GABRG2	0.042
Radix Paeoniae（RP）	MOL001002	ellagic acid	3 beta-hydroxysteroid dehydrogenase/Delta 5-->4-isomerase type II	P26439	HSD3B2	0.044
Radix Paeoniae（RP）	MOL001002	ellagic acid	Gonadotropin-releasing hormone receptor	P30968	GNRHR	0.045
Radix Paeoniae（RP）	MOL001002	ellagic acid	Calmodulin	P62158		0.045
Radix Paeoniae（RP）	MOL001002	ellagic acid	Gonadotropin-releasing hormone II receptor	Q96P88	GNRHR2	0.045
Radix Paeoniae（RP）	MOL001002	ellagic acid	cGMP-inhibited 3',5'-cyclic phosphodiesterase A	Q14432	PDE3A	0.046
Radix Paeoniae（RP）	MOL001002	ellagic acid	Gamma-aminobutyric acid receptor subunit rho-3	A8MPY1	GABRR3	0.047
Radix Paeoniae（RP）	MOL001002	ellagic acid	Gamma-aminobutyric acid receptor subunit pi	O00591	GABRP	0.047
Radix Paeoniae（RP）	MOL001002	ellagic acid	Gamma-aminobutyric acid receptor subunit delta	O14764	GABRD	0.047
Radix Paeoniae（RP）	MOL001002	ellagic acid	Microtubule-associated protein 2	P11137	MAP2	0.047
Radix Paeoniae（RP）	MOL001002	ellagic acid	Gamma-aminobutyric-acid receptor subunit beta-1	P18505	GABRB1	0.047
Radix Paeoniae（RP）	MOL001002	ellagic acid	Gamma-aminobutyric-acid receptor subunit rho-1	P24046	GABRR1	0.047
Radix Paeoniae（RP）	MOL001002	ellagic acid	Gamma-aminobutyric acid receptor subunit rho-2	P28476	GABRR2	0.047
Radix Paeoniae（RP）	MOL001002	ellagic acid	Nitric-oxide synthase, brain	P29475	NOS1	0.047
Radix Paeoniae（RP）	MOL001002	ellagic acid	Gamma-aminobutyric-acid receptor subunit alpha-5	P31644	GABRA5	0.047
Radix Paeoniae（RP）	MOL001002	ellagic acid	Estrogen-related receptor gamma	P62508	ESRRG	0.047
Radix Paeoniae（RP）	MOL001002	ellagic acid	Tubulin beta-2C chain	P68371	TUBB4B	0.047
Radix Paeoniae（RP）	MOL001002	ellagic acid	Gamma-aminobutyric acid receptor subunit epsilon	P78334	GABRE	0.047
Radix Paeoniae（RP）	MOL001002	ellagic acid	Microtubule-associated protein 1A	P78559	MAP1A	0.047
Radix Paeoniae（RP）	MOL001002	ellagic acid	Gamma-aminobutyric acid receptor subunit gamma-1	Q8N1C3	GABRG1	0.047
Radix Paeoniae（RP）	MOL001002	ellagic acid	Gamma-aminobutyric acid receptor subunit gamma-3	Q99928	GABRG3	0.047
Radix Paeoniae（RP）	MOL001002	ellagic acid	Phenylalanine-4-hydroxylase	P00439	PAH	0.049
Radix Paeoniae（RP）	MOL001002	ellagic acid	Carbonic anhydrase 4	P22748	CA4	0.049
Radix Paeoniae（RP）	MOL001002	ellagic acid	ATP synthase subunit beta, mitochondrial	P06576	ATP5F1B	0.05
Radix Paeoniae（RP）	MOL001002	ellagic acid	Tyrosine-protein kinase HCK	P08631	HCK	0.05
Radix Paeoniae（RP）	MOL001002	ellagic acid	ATP synthase subunit alpha, mitochondrial	P25705	ATP5F1A	0.05
Radix Paeoniae（RP）	MOL001002	ellagic acid	Tyrosine 3-monooxygenase	P07101	TH	0.051
Radix Paeoniae（RP）	MOL001002	ellagic acid	Keratin, type II cytoskeletal 7	P08729	KRT7	0.051
Radix Paeoniae（RP）	MOL001002	ellagic acid	Gamma-aminobutyric-acid receptor subunit alpha-1	P14867	GABRA1	0.053

Radix Paeoniae（RP）	MOL001002	ellagic acid	Gamma-aminobutyric-acid receptor subunit alpha-3	P34903	GABRA3	0.053
Radix Paeoniae（RP）	MOL001002	ellagic acid	Gamma-aminobutyric-acid receptor subunit alpha-2	P47869	GABRA2	0.053
Radix Paeoniae（RP）	MOL001002	ellagic acid	Trypsin-1	P07477	PRSS1	0.054
Radix Paeoniae（RP）	MOL001002	ellagic acid	Tryptophanyl-tRNA synthetase, mitochondrial	Q9UGM6	WARS2	0.054
Radix Paeoniae（RP）	MOL001002	ellagic acid	Tyrosyl-tRNA synthetase, cytoplasmic	P54577	YARS	0.055
Radix Paeoniae（RP）	MOL001002	ellagic acid	Tripartite motif-containing protein 13	O60858	TRIM13	0.058
Radix Paeoniae（RP）	MOL001002	ellagic acid	D-HSCDK2	O75100	CA11	0.058
Radix Paeoniae（RP）	MOL001002	ellagic acid	Cell division control protein 2 homolog	P06493	CDK1	0.058
Radix Paeoniae（RP）	MOL001002	ellagic acid	Death-associated protein kinase 3	O43293	DAPK3	0.062
Radix Paeoniae（RP）	MOL001002	ellagic acid	Carbonic anhydrase 1	P00915	CA1	0.063
Radix Paeoniae（RP）	MOL001002	ellagic acid	Casein kinase II subunit alpha	P68400	CSNK2A1	0.063
Radix Paeoniae（RP）	MOL001002	ellagic acid	Melatonin receptor type 1A	P48039	MTNR1A	0.064
Radix Paeoniae（RP）	MOL001002	ellagic acid	Glycogen synthase kinase-3 beta	P49841	GSK3B	0.064
Radix Paeoniae（RP）	MOL001002	ellagic acid	Bile salt sulfotransferase	Q06520	SULT2A1	0.065
Radix Paeoniae（RP）	MOL001002	ellagic acid	cAMP-specific 3',5'-cyclic phosphodiesterase 4A	P27815	PDE4A	0.066
Radix Paeoniae（RP）	MOL001002	ellagic acid	Proto-oncogene tyrosine-protein kinase LCK	P06239	LCK	0.068
Radix Paeoniae（RP）	MOL001002	ellagic acid	Phosphatidylinositol-4,5-bisphosphate 3-kinase catalytic subunit gamma isoform	P48736	PIK3CG	0.068
Radix Paeoniae（RP）	MOL001002	ellagic acid	Phospholipase A2, membrane associated	P14555	PLA2G2A	0.072
Radix Paeoniae（RP）	MOL001002	ellagic acid	ATP-sensitive inward rectifier potassium channel 1	P48048	KCNJ1	0.072
Radix Paeoniae（RP）	MOL001002	ellagic acid	Glucocorticoid receptor	P04150	NR3C1	0.073
Radix Paeoniae（RP）	MOL001002	ellagic acid	Poly [ADP-ribose] polymerase 1	P09874	PARP1	0.073
Radix Paeoniae（RP）	MOL001002	ellagic acid	Aldose reductase	P15121	AKR1B1	0.073
Radix Paeoniae（RP）	MOL001002	ellagic acid	Cannabinoid receptor 1	P21554	CNR1	0.073
Radix Paeoniae（RP）	MOL001002	ellagic acid	Endothelin-1 receptor	P25101	EDNRA	0.073
Radix Paeoniae（RP）	MOL001002	ellagic acid	Calcium/calmodulin-dependent protein kinase type II alpha chain	Q9UQM7	CAMK2A	0.073
Radix Paeoniae（RP）	MOL001002	ellagic acid	Purine nucleoside phosphorylase	P00491	PNP	0.074
Radix Paeoniae（RP）	MOL001002	ellagic acid	Thyroid hormone receptor beta-1	P10828	THRB	0.075
Radix Paeoniae（RP）	MOL001002	ellagic acid	Cytochrome P450 19A1	P11511	CYP19A1	0.075
Radix Paeoniae（RP）	MOL001002	ellagic acid	Cannabinoid receptor 2	P34972	CNR2	0.075
Radix Paeoniae（RP）	MOL001002	ellagic acid	Tubulin beta-1 chain	Q9H4B7	TUBB1	0.075
Radix Paeoniae（RP）	MOL001002	ellagic acid	Carbonic anhydrase 2	P00918	CA2	0.076
Radix Paeoniae（RP）	MOL001002	ellagic acid	Nuclear receptor coactivator 2	Q15596	NCOA2	0.076
Radix Paeoniae（RP）	MOL001002	ellagic acid	Hepatocyte growth factor receptor	P08581	MET	0.077
Radix Paeoniae（RP）	MOL001002	ellagic acid	Membrane copper amine oxidase	Q16853	AOC3	0.077
Radix Paeoniae（RP）	MOL001002	ellagic acid	NADPH oxidase organizer 1	Q8NFA2	NOXO1	0.077
Radix Paeoniae（RP）	MOL001002	ellagic acid	Egl nine homolog 1	Q9GZT9	EGLN1	0.077
Radix Paeoniae（RP）	MOL001002	ellagic acid	Dual specificity protein kinase CLK1	P49759	CLK1	0.079
Radix Paeoniae（RP）	MOL001002	ellagic acid	Group IIE secretory phospholipase A2	Q9NZK7	PLA2G2E	0.079
Radix Paeoniae（RP）	MOL001002	ellagic acid	Nitric oxide synthase, inducible	P35228	NOS2	0.08
Radix Paeoniae（RP）	MOL001002	ellagic acid	Neuropeptide Y	P01303	NPY	0.081
Radix Paeoniae（RP）	MOL001002	ellagic acid	RAC-alpha serine/threonine-protein kinase	P31749	AKT1	0.081
Radix Paeoniae（RP）	MOL001002	ellagic acid	S-methyl-5-thioadenosine phosphorylase	Q13126	MTAP	0.081
Radix Paeoniae（RP）	MOL001002	ellagic acid	5-hydroxytryptamine 1D receptor	P28221	HTR1D	0.082
Radix Paeoniae（RP）	MOL001002	ellagic acid	Hydroxyacid oxidase 1	Q9UJM8	HAO1	0.082
Radix Paeoniae（RP）	MOL001002	ellagic acid	C-jun-amino-terminal kinase-interacting protein 1	Q9UQF2	MAPK8IP1	0.082
Radix Paeoniae（RP）	MOL001002	ellagic acid	5-hydroxytryptamine 1B receptor	P28222	HTR1B	0.083
Radix Paeoniae（RP）	MOL001002	ellagic acid	Sodium-dependent serotonin transporter	P31645	SLC6A4	0.083
Radix Paeoniae（RP）	MOL001002	ellagic acid	Toll-like receptor 7	Q9NYK1	TLR7	0.083
Radix Paeoniae（RP）	MOL001002	ellagic acid	Sodium-dependent noradrenaline transporter	P23975	SLC6A2	0.084
Radix Paeoniae（RP）	MOL001002	ellagic acid	Triosephosphate isomerase	P60174	TPI1	0.084
Radix Paeoniae（RP）	MOL001002	ellagic acid	3 beta-hydroxysteroid dehydrogenase/Delta 5-->4-isomerase type I	P14060	HSD3B1	0.085
Radix Paeoniae（RP）	MOL001002	ellagic acid	Ig kappa chain V-II region RPMI 6410	P06310	IGKV2-30	0.086
Radix Paeoniae（RP）	MOL001002	ellagic acid	Cell division protein kinase 5	Q00535	CDK5	0.087

Radix Paeoniae（RP）	MOL001002	ellagic acid	Nuclear receptor coactivator 5	Q9HCD5	NCOA5	0.088
Radix Paeoniae（RP）	MOL001002	ellagic acid	Ribosyldihydronicotinamide dehydrogenase [quinone]	P16083	NQO2	0.089
Radix Paeoniae（RP）	MOL001002	ellagic acid	cAMP-specific 3',5'-cyclic phosphodiesterase 4B	Q07343	PDE4B	0.089
Radix Paeoniae（RP）	MOL001002	ellagic acid	DNA polymerase kappa	Q9UBT6	POLK	0.09
Radix Paeoniae（RP）	MOL001002	ellagic acid	Proto-oncogene serine/threonine-protein kinase Pim-1	P11309	PIM1	0.091
Radix Paeoniae（RP）	MOL001002	ellagic acid	Mineralocorticoid receptor	P08235	NR3C2	0.093
Radix Paeoniae（RP）	MOL001002	ellagic acid	Nitric-oxide synthase, endothelial	P29474	NOS3	0.096
Radix Paeoniae（RP）	MOL001002	ellagic acid	Tubulin alpha-3 chain	Q71U36	TUBA1A	0.097
Radix Paeoniae（RP）	MOL001002	ellagic acid	Muscarinic acetylcholine receptor M2	P08172	CHRM2	0.098
Radix Paeoniae（RP）	MOL001002	ellagic acid	Alpha-1D adrenergic receptor	P25100	ADRA1D	0.098
Radix Paeoniae（RP）	MOL001002	ellagic acid	cAMP-dependent protein kinase inhibitor alpha	P61925	PKIA	0.102
Radix Paeoniae（RP）	MOL001002	ellagic acid	Rho-associated protein kinase 1	Q13464	ROCK1	0.102
Radix Paeoniae（RP）	MOL001002	ellagic acid	Beta-1 adrenergic receptor	P08588	ADRB1	0.103
Radix Paeoniae（RP）	MOL001002	ellagic acid	MAP kinase-activated protein kinase 2	P49137	MAPKAPK2	0.106
Radix Paeoniae（RP）	MOL001002	ellagic acid	D(1B) dopamine receptor	P21918	DRD5	0.107
Radix Paeoniae（RP）	MOL001002	ellagic acid	Prostaglandin G/H synthase 1	P23219	PTGS1	0.107
Radix Paeoniae（RP）	MOL001002	ellagic acid	5-hydroxytryptamine 2B receptor	P41595	HTR2B	0.107
Radix Paeoniae（RP）	MOL001002	ellagic acid	D(4) dopamine receptor	P21917	DRD4	0.108
Radix Paeoniae（RP）	MOL001002	ellagic acid	D(3) dopamine receptor	P35462	DRD3	0.108
Radix Paeoniae（RP）	MOL001002	ellagic acid	Muscarinic acetylcholine receptor M1	P11229	CHRM1	0.11
Radix Paeoniae（RP）	MOL001002	ellagic acid	Muscarinic acetylcholine receptor M3	P20309	CHRM3	0.11
Radix Paeoniae（RP）	MOL001002	ellagic acid	Beta-2 adrenergic receptor	P07550	ADRB2	0.116
Radix Paeoniae（RP）	MOL001002	ellagic acid	Muscarinic acetylcholine receptor M4	P08173	CHRM4	0.122
Radix Paeoniae（RP）	MOL001002	ellagic acid	Muscarinic acetylcholine receptor M5	P08912	CHRM5	0.122
Radix Paeoniae（RP）	MOL001002	ellagic acid	Alpha-2A adrenergic receptor	P08913	ADRA2A	0.122
Radix Paeoniae（RP）	MOL001002	ellagic acid	Alpha-2C adrenergic receptor	P18825	ADRA2C	0.122
Radix Paeoniae（RP）	MOL001002	ellagic acid	Alpha-1B adrenergic receptor	P35368	ADRA1B	0.135
Radix Paeoniae（RP）	MOL001002	ellagic acid	Androgen receptor	P10275	AR	0.139
Radix Paeoniae（RP）	MOL001002	ellagic acid	5-hydroxytryptamine 2C receptor	P28335	HTR2C	0.144
Radix Paeoniae（RP）	MOL001002	ellagic acid	Estradiol 17-beta-dehydrogenase 1	P14061	HSD17B1	0.149
Radix Paeoniae（RP）	MOL001002	ellagic acid	Histamine H1 receptor	P35367	HRH1	0.155
Radix Paeoniae（RP）	MOL001002	ellagic acid	Alpha-2B adrenergic receptor	P18089	ADRA2B	0.159
Radix Paeoniae（RP）	MOL001002	ellagic acid	5-hydroxytryptamine 1A receptor	P08908	HTR1A	0.166
Radix Paeoniae（RP）	MOL001002	ellagic acid	Prothrombin	P00734	F2	0.187
Radix Paeoniae（RP）	MOL001002	ellagic acid	5-hydroxytryptamine 2A receptor	P28223	HTR2A	0.206
Radix Paeoniae（RP）	MOL001002	ellagic acid	Delta-type opioid receptor	P41143	OPRD1	0.206
Radix Paeoniae（RP）	MOL001002	ellagic acid	D(1A) dopamine receptor	P21728	DRD1	0.217
Radix Paeoniae（RP）	MOL001002	ellagic acid	Alpha-1A adrenergic receptor	P35348	ADRA1A	0.222
Radix Paeoniae（RP）	MOL001002	ellagic acid	Progesterone receptor	P06401	PGR	0.234
Radix Paeoniae（RP）	MOL001002	ellagic acid	Hemoglobin subunit alpha	P69905	HBA1	0.241
Radix Paeoniae（RP）	MOL001002	ellagic acid	D(2) dopamine receptor	P14416	DRD2	0.255
Radix Paeoniae（RP）	MOL001002	ellagic acid	Kappa-type opioid receptor	P41145	OPRK1	0.265
Radix Paeoniae（RP）	MOL001002	ellagic acid	Nuclear receptor coactivator 1	Q15788	NCOA1	0.283
Radix Paeoniae（RP）	MOL001002	ellagic acid	Cell division protein kinase 2	P24941	CDK2	0.298
Radix Paeoniae（RP）	MOL001002	ellagic acid	Cyclin-A2	P20248	CCNA2	0.33
Radix Paeoniae（RP）	MOL001002	ellagic acid	Mu-type opioid receptor	P35372	OPRM1	0.371
Radix Paeoniae（RP）	MOL001002	ellagic acid	cAMP-dependent protein kinase catalytic subunit alpha	P17612	PRKACA	0.412
Radix Paeoniae（RP）	MOL001002	ellagic acid	Prostaglandin G/H synthase 2	P35354	PTGS2	0.437
Radix Paeoniae（RP）	MOL001002	ellagic acid	Estrogen receptor beta	Q92731	ESR2	0.594
Radix Paeoniae（RP）	MOL001002	ellagic acid	Estrogen receptor	P03372	ESR1	1
Radix Paeoniae（RP）	MOL001918	paeoniflorgenone	Actin-related protein 2/3 complex subunit 1B	O15143	ARPC1B	0.01
Radix Paeoniae（RP）	MOL001918	paeoniflorgenone	Actin-related protein 2/3 complex subunit 2	O15144	ARPC2	0.01
Radix Paeoniae（RP）	MOL001918	paeoniflorgenone	Actin-related protein 2/3 complex subunit 3	O15145	ARPC3	0.01

Radix Paeoniae（RP）	MOL001918	paeoniflorgenone	Coagulation factor IX	P00740	F9	0.01
Radix Paeoniae（RP）	MOL001918	paeoniflorgenone	Coagulation factor X	P00742	F10	0.01
Radix Paeoniae（RP）	MOL001918	paeoniflorgenone	Osteocalcin	P02818	BGLAP	0.01
Radix Paeoniae（RP）	MOL001918	paeoniflorgenone	Vitamin K-dependent protein C	P04070	PROC	0.01
Radix Paeoniae（RP）	MOL001918	paeoniflorgenone	Coagulation factor VII	P08709	F7	0.01
Radix Paeoniae（RP）	MOL001918	paeoniflorgenone	NAD(P)H dehydrogenase [quinone] 1	P15559	NQO1	0.01
Radix Paeoniae（RP）	MOL001918	paeoniflorgenone	Ribosyldihydronicotinamide dehydrogenase [quinone]	P16083	NQO2	0.01
Radix Paeoniae（RP）	MOL001918	paeoniflorgenone	Vitamin K-dependent protein Z	P22891	PROZ	0.01
Radix Paeoniae（RP）	MOL001918	paeoniflorgenone	Vitamin K-dependent gamma-carboxylase	P38435	GGCX	0.01
Radix Paeoniae（RP）	MOL001918	paeoniflorgenone	Actin-related protein 2/3 complex subunit 4	P59998	ARPC4	0.01
Radix Paeoniae（RP）	MOL001918	paeoniflorgenone	Actin-related protein 3	P61158	ACTR3	0.01
Radix Paeoniae（RP）	MOL001918	paeoniflorgenone	Actin-related protein 2	P61160	ACTR2	0.01
Radix Paeoniae（RP）	MOL001918	paeoniflorgenone	Vitamin K epoxide reductase complex subunit 1-like protein 1	Q8N0U8	VKORC1L1	0.01
Radix Paeoniae（RP）	MOL001918	paeoniflorgenone	Vitamin K epoxide reductase complex subunit 1	Q9BQB6	VKORC1	0.01
Radix Paeoniae（RP）	MOL001918	paeoniflorgenone	Acetylcholine receptor subunit alpha	P02708	CHRNA1	0.011
Radix Paeoniae（RP）	MOL001918	paeoniflorgenone	Cholinesterase	P06276	BCHE	0.011
Radix Paeoniae（RP）	MOL001918	paeoniflorgenone	Acetylcholine receptor subunit gamma	P07510	CHRNG	0.011
Radix Paeoniae（RP）	MOL001918	paeoniflorgenone	Acetylcholine receptor subunit beta	P11230	CHRNB1	0.011
Radix Paeoniae（RP）	MOL001918	paeoniflorgenone	Neuronal acetylcholine receptor subunit beta-2	P17787	CHRNB2	0.011
Radix Paeoniae（RP）	MOL001918	paeoniflorgenone	Succinate dehydrogenase [ubiquinone] iron-sulfur subunit, mitochondrial	P21912	SDHB	0.011
Radix Paeoniae（RP）	MOL001918	paeoniflorgenone	Acetylcholinesterase	P22303	ACHE	0.011
Radix Paeoniae（RP）	MOL001918	paeoniflorgenone	Neuronal acetylcholine receptor subunit alpha-5	P30532	CHRNA5	0.011
Radix Paeoniae（RP）	MOL001918	paeoniflorgenone	Succinate dehydrogenase [ubiquinone] flavoprotein subunit, mitochondrial	P31040	SDHA	0.011
Radix Paeoniae（RP）	MOL001918	paeoniflorgenone	Acetylcholine receptor subunit epsilon	Q04844	CHRNE	0.011
Radix Paeoniae（RP）	MOL001918	paeoniflorgenone	Neuronal acetylcholine receptor subunit beta-3	Q05901	CHRNB3	0.011
Radix Paeoniae（RP）	MOL001918	paeoniflorgenone	Acetylcholine receptor subunit delta	Q07001	CHRND	0.011
Radix Paeoniae（RP）	MOL001918	paeoniflorgenone	Neuronal acetylcholine receptor subunit alpha-6	Q15825	KCNJ8	0.011
Radix Paeoniae（RP）	MOL001918	paeoniflorgenone	2,4-dienoyl-CoA reductase, mitochondrial	Q16698	DECR1	0.011
Radix Paeoniae（RP）	MOL001918	paeoniflorgenone	Succinate dehydrogenase cytochrome b560 subunit, mitochondrial	Q99643	SDHC	0.011
Radix Paeoniae（RP）	MOL001918	paeoniflorgenone	Neuronal acetylcholine receptor subunit alpha-9	Q9UGM1	CHRNA9	0.011
Radix Paeoniae（RP）	MOL001918	paeoniflorgenone	Potassium channel subfamily K member 6	Q9Y257	KCNK6	0.011
Radix Paeoniae（RP）	MOL001918	paeoniflorgenone	cGMP-specific 3',5'-cyclic phosphodiesterase	O76074	PDE5A	0.012
Radix Paeoniae（RP）	MOL001918	paeoniflorgenone	Macrophage migration inhibitory factor	P14174	MIF	0.012
Radix Paeoniae（RP）	MOL001918	paeoniflorgenone	Liver carboxylesterase 1	P23141	CES1	0.012
Radix Paeoniae（RP）	MOL001918	paeoniflorgenone	5-hydroxytryptamine 1E receptor	P28566	HTR1E	0.012
Radix Paeoniae（RP）	MOL001918	paeoniflorgenone	Adenosine A2a receptor	P29274	ADORA2A	0.012
Radix Paeoniae（RP）	MOL001918	paeoniflorgenone	Adenosine A1 receptor	P30542	ADORA1	0.012
Radix Paeoniae（RP）	MOL001918	paeoniflorgenone	cGMP-inhibited 3',5'-cyclic phosphodiesterase A	Q14432	PDE3A	0.012
Radix Paeoniae（RP）	MOL001918	paeoniflorgenone	Histamine H4 receptor	Q9H3N8	HRH4	0.012
Radix Paeoniae（RP）	MOL001918	paeoniflorgenone	Potassium voltage-gated channel subfamily D member 2	Q9NZV8	KCND2	0.012
Radix Paeoniae（RP）	MOL001918	paeoniflorgenone	cAMP and cAMP-inhibited cGMP 3',5'-cyclic phosphodiesterase 10A	Q9Y233	PDE10A	0.012
Radix Paeoniae（RP）	MOL001918	paeoniflorgenone	High affinity cAMP-specific and IBMX-insensitive 3',5'-cyclic phosphodiesterase 8A	O60658	PDE8A	0.013
Radix Paeoniae（RP）	MOL001918	paeoniflorgenone	6-phosphogluconate dehydrogenase, decarboxylating	P52209	PGD	0.013
Radix Paeoniae（RP）	MOL001918	paeoniflorgenone	cAMP-specific 3',5'-cyclic phosphodiesterase 4C	Q08493	PDE4C	0.013
Radix Paeoniae（RP）	MOL001918	paeoniflorgenone	cAMP-specific 3',5'-cyclic phosphodiesterase 4D	Q08499	PDE4D	0.013
Radix Paeoniae（RP）	MOL001918	paeoniflorgenone	High-affinity cAMP-specific 3',5'-cyclic phosphodiesterase 7A	Q13946	PDE7A	0.013
Radix Paeoniae（RP）	MOL001918	paeoniflorgenone	cAMP-specific 3',5'-cyclic phosphodiesterase 7B	Q9NP56	PDE7B	0.013
Radix Paeoniae（RP）	MOL001918	paeoniflorgenone	Cytochrome b	P00156	MT-CYB	0.014
Radix Paeoniae（RP）	MOL001918	paeoniflorgenone	Cytochrome c1, heme protein, mitochondrial	P08574	CYC1	0.014
Radix Paeoniae（RP）	MOL001918	paeoniflorgenone	Cytochrome b-c1 complex subunit 7	P14927	UQCRB	0.014
Radix Paeoniae（RP）	MOL001918	paeoniflorgenone	Cytochrome b-c1 complex subunit Rieske, mitochondrial	P47985	UQCRFS1	0.014
Radix Paeoniae（RP）	MOL001918	paeoniflorgenone	Sodium/potassium-transporting ATPase alpha-1 chain	P05023	ATP1A1	0.015

Radix Paeoniae（RP）	MOL001918	paeoniflorgenone	Sodium/potassium-transporting ATPase gamma chain	P54710	FXYD2	0.015
Radix Paeoniae（RP）	MOL001918	paeoniflorgenone	Opioid receptor, sigma 1	Q5T1J1	SIGMAR1	0.015
Radix Paeoniae（RP）	MOL001918	paeoniflorgenone	Sigma 1-type opioid receptor	Q99720	SIGMAR1	0.015
Radix Paeoniae（RP）	MOL001918	paeoniflorgenone	Glutamate [NMDA] receptor subunit epsilon-4	O15399	GRIN2D	0.016
Radix Paeoniae（RP）	MOL001918	paeoniflorgenone	Glutamate [NMDA] receptor subunit 3B	O60391	GRIN3B	0.016
Radix Paeoniae（RP）	MOL001918	paeoniflorgenone	Glutamate [NMDA] receptor subunit zeta-1	Q05586	GRIN1	0.016
Radix Paeoniae（RP）	MOL001918	paeoniflorgenone	Glutamate [NMDA] receptor subunit epsilon-1	Q12879	GRIN2A	0.016
Radix Paeoniae（RP）	MOL001918	paeoniflorgenone	Neuronal acetylcholine receptor subunit alpha-4	P43681	CHRNA4	0.017
Radix Paeoniae（RP）	MOL001918	paeoniflorgenone	72 kDa type IV collagenase	P08253	MMP2	0.018
Radix Paeoniae（RP）	MOL001918	paeoniflorgenone	Substance-P receptor	P25103	TACR1	0.018
Radix Paeoniae（RP）	MOL001918	paeoniflorgenone	Potassium channel subfamily K member 1	O00180	KCNK1	0.019
Radix Paeoniae（RP）	MOL001918	paeoniflorgenone	Retinoic acid receptor alpha	P10276	RARA	0.019
Radix Paeoniae（RP）	MOL001918	paeoniflorgenone	Retinoic acid receptor beta	P10826	RARB	0.019
Radix Paeoniae（RP）	MOL001918	paeoniflorgenone	Retinoic acid receptor gamma-1	P13631	RARG	0.019
Radix Paeoniae（RP）	MOL001918	paeoniflorgenone	Neuronal acetylcholine receptor subunit alpha-3	P32297	CHRNA3	0.019
Radix Paeoniae（RP）	MOL001918	paeoniflorgenone	Retinoic acid receptor RXR-gamma	P48443	RXRG	0.019
Radix Paeoniae（RP）	MOL001918	paeoniflorgenone	Tyrosine 3-monooxygenase	P07101	TH	0.021
Radix Paeoniae（RP）	MOL001918	paeoniflorgenone	Solute carrier family 22 member 6	Q4U2R8	SLC22A6	0.021
Radix Paeoniae（RP）	MOL001918	paeoniflorgenone	Solute carrier family 22 member 8	Q8TCC7	SLC22A8	0.021
Radix Paeoniae（RP）	MOL001918	paeoniflorgenone	Solute carrier family 22 member 11	Q9NSA0	SLC22A11	0.021
Radix Paeoniae（RP）	MOL001918	paeoniflorgenone	Tyrosyl-tRNA synthetase, mitochondrial	Q9Y2Z4	YARS2	0.021
Radix Paeoniae（RP）	MOL001918	paeoniflorgenone	Tyrosine-protein kinase JAK2	O60674	JAK2	0.022
Radix Paeoniae（RP）	MOL001918	paeoniflorgenone	Tyrosine-protein kinase JAK1	P23458	JAK1	0.022
Radix Paeoniae（RP）	MOL001918	paeoniflorgenone	Tyrosine-protein kinase JAK3	P52333	JAK3	0.022
Radix Paeoniae（RP）	MOL001918	paeoniflorgenone	Oxysterols receptor LXR-beta	P55055	NR1H2	0.022
Radix Paeoniae（RP）	MOL001918	paeoniflorgenone	Oxysterols receptor LXR-alpha	Q13133	NR1H3	0.022
Radix Paeoniae（RP）	MOL001918	paeoniflorgenone	Phenylalanine-4-hydroxylase	P00439	PAH	0.024
Radix Paeoniae（RP）	MOL001918	paeoniflorgenone	Retinoic acid receptor RXR-alpha	P19793	RXRA	0.024
Radix Paeoniae（RP）	MOL001918	paeoniflorgenone	Bile salt sulfotransferase	Q06520	SULT2A1	0.024
Radix Paeoniae（RP）	MOL001918	paeoniflorgenone	Nuclear receptor subfamily 1 group I member 3	Q14994	NR1I3	0.024
Radix Paeoniae（RP）	MOL001918	paeoniflorgenone	cAMP response element-binding protein	P16220	CREB1	0.025
Radix Paeoniae（RP）	MOL001918	paeoniflorgenone	5-hydroxytryptamine 7 receptor	P34969	HTR7	0.025
Radix Paeoniae（RP）	MOL001918	paeoniflorgenone	Nociceptin receptor	P41146	OPRL1	0.025
Radix Paeoniae（RP）	MOL001918	paeoniflorgenone	5-hydroxytryptamine 6 receptor	P50406	HTR6	0.025
Radix Paeoniae（RP）	MOL001918	paeoniflorgenone	D1 dopamine receptor-interacting protein calcyon	Q9NYX4	CALY	0.025
Radix Paeoniae（RP）	MOL001918	paeoniflorgenone	Succinate dehydrogenase [ubiquinone] cytochrome b small subunit, mitochondrial	O14521	SDHD	0.026
Radix Paeoniae（RP）	MOL001918	paeoniflorgenone	cAMP-specific 3',5'-cyclic phosphodiesterase 4B	Q07343	PDE4B	0.027
Radix Paeoniae（RP）	MOL001918	paeoniflorgenone	Neuronal acetylcholine receptor subunit alpha-2	Q15822	CHRNA2	0.028
Radix Paeoniae（RP）	MOL001918	paeoniflorgenone	Ig gamma-1 chain C region	P01857	IGHG1	0.03
Radix Paeoniae（RP）	MOL001918	paeoniflorgenone	Ig gamma-2 chain C region	P01859	IGHG2	0.03
Radix Paeoniae（RP）	MOL001918	paeoniflorgenone	Peptidyl-prolyl cis-trans isomerase, mitochondrial	P30405	PPIF	0.03
Radix Paeoniae（RP）	MOL001918	paeoniflorgenone	Neuronal acetylcholine receptor subunit alpha-7	P36544	CHRNA7	0.03
Radix Paeoniae（RP）	MOL001918	paeoniflorgenone	DNA topoisomerase 2-alpha	P11388	TOP2A	0.031
Radix Paeoniae（RP）	MOL001918	paeoniflorgenone	Mitogen-activated protein kinase 8	P45983	MAPK8	0.031
Radix Paeoniae（RP）	MOL001918	paeoniflorgenone	Mitogen-activated protein kinase 10	P53779	MAPK10	0.031
Radix Paeoniae（RP）	MOL001918	paeoniflorgenone	Tubulin alpha-1 chain	P68366	TUBA4A	0.031
Radix Paeoniae（RP）	MOL001918	paeoniflorgenone	Casein kinase II subunit alpha	P68400	CSNK2A1	0.031
Radix Paeoniae（RP）	MOL001918	paeoniflorgenone	Casein kinase I isoform gamma-2	P78368	CSNK1G2	0.031
Radix Paeoniae（RP）	MOL001918	paeoniflorgenone	Serine/threonine-protein kinase haspin	Q8TF76	HASPIN	0.031
Radix Paeoniae（RP）	MOL001918	paeoniflorgenone	Nitric-oxide synthase, endothelial	P29474	NOS3	0.032
Radix Paeoniae（RP）	MOL001918	paeoniflorgenone	Nitric-oxide synthase, brain	P29475	NOS1	0.032
Radix Paeoniae（RP）	MOL001918	paeoniflorgenone	Neuronal acetylcholine receptor subunit alpha-10	Q9GZZ6	CHRNA10	0.032
Radix Paeoniae（RP）	MOL001918	paeoniflorgenone	Carbonic anhydrase 12	O43570	CA12	0.033

Radix Paeoniae（RP）	MOL001918	paeoniflorgenone	Platelet glycoprotein IX	P14770	GP9	0.033
Radix Paeoniae（RP）	MOL001918	paeoniflorgenone	Carbonic anhydrase 4	P22748	CA4	0.033
Radix Paeoniae（RP）	MOL001918	paeoniflorgenone	Phosphatidylinositol 3-kinase regulatory subunit alpha	P27986	PIK3R1	0.033
Radix Paeoniae（RP）	MOL001918	paeoniflorgenone	Neuronal acetylcholine receptor subunit beta-4	P30926	CHRNB4	0.033
Radix Paeoniae（RP）	MOL001918	paeoniflorgenone	Serine/threonine-protein kinase PLK1	P53350	PLK1	0.033
Radix Paeoniae（RP）	MOL001918	paeoniflorgenone	Solute carrier family 12 member 3	P55017	SLC12A3	0.033
Radix Paeoniae（RP）	MOL001918	paeoniflorgenone	Hemoglobin subunit alpha	P69905	HBA1	0.033
Radix Paeoniae（RP）	MOL001918	paeoniflorgenone	Solute carrier family 12 member 1	Q13621	SLC12A1	0.033
Radix Paeoniae（RP）	MOL001918	paeoniflorgenone	Carbonic anhydrase 9	Q16790	CA9	0.033
Radix Paeoniae（RP）	MOL001918	paeoniflorgenone	Glutamate [NMDA] receptor subunit epsilon-2	Q13224	GRIN2B	0.035
Radix Paeoniae（RP）	MOL001918	paeoniflorgenone	Proto-oncogene tyrosine-protein kinase LCK	P06239	LCK	0.036
Radix Paeoniae（RP）	MOL001918	paeoniflorgenone	Tyrosine-protein kinase Lyn	P07948	LYN	0.036
Radix Paeoniae（RP）	MOL001918	paeoniflorgenone	Beta-2 adrenergic receptor	P07550	ADRB2	0.037
Radix Paeoniae（RP）	MOL001918	paeoniflorgenone	Beta-1 adrenergic receptor	P08588	ADRB1	0.037
Radix Paeoniae（RP）	MOL001918	paeoniflorgenone	5-hydroxytryptamine 2B receptor	P41595	HTR2B	0.037
Radix Paeoniae（RP）	MOL001918	paeoniflorgenone	Angiotensin-converting enzyme	P12821	ACE	0.038
Radix Paeoniae（RP）	MOL001918	paeoniflorgenone	Translocator protein	P30536	TSPO	0.038
Radix Paeoniae（RP）	MOL001918	paeoniflorgenone	Prothrombin	P00734	F2	0.04
Radix Paeoniae（RP）	MOL001918	paeoniflorgenone	Muscarinic acetylcholine receptor M5	P08912	CHRM5	0.04
Radix Paeoniae（RP）	MOL001918	paeoniflorgenone	cAMP-specific 3',5'-cyclic phosphodiesterase 4A	P27815	PDE4A	0.042
Radix Paeoniae（RP）	MOL001918	paeoniflorgenone	Cell division control protein 2 homolog	P06493	CDK1	0.043
Radix Paeoniae（RP）	MOL001918	paeoniflorgenone	Retinoic acid receptor RXR-beta	P28702	RXRB	0.043
Radix Paeoniae（RP）	MOL001918	paeoniflorgenone	Corticosteroid 11-beta-dehydrogenase isozyme 1	P28845	HSD11B1	0.043
Radix Paeoniae（RP）	MOL001918	paeoniflorgenone	5-hydroxytryptamine 3 receptor	P46098	HTR3A	0.043
Radix Paeoniae（RP）	MOL001918	paeoniflorgenone	Cell division protein kinase 5	Q00535	CDK5	0.043
Radix Paeoniae（RP）	MOL001918	paeoniflorgenone	Gonadotropin-releasing hormone receptor	P30968	GNRHR	0.044
Radix Paeoniae（RP）	MOL001918	paeoniflorgenone	Tyrosyl-tRNA synthetase, cytoplasmic	P54577	YARS	0.044
Radix Paeoniae（RP）	MOL001918	paeoniflorgenone	Gonadotropin-releasing hormone II receptor	Q96P88	GNRHR2	0.044
Radix Paeoniae（RP）	MOL001918	paeoniflorgenone	3-oxo-5-alpha-steroid 4-dehydrogenase 1	P18405	SRD5A1	0.045
Radix Paeoniae（RP）	MOL001918	paeoniflorgenone	Microtubule-associated protein 2	P11137	MAP2	0.046
Radix Paeoniae（RP）	MOL001918	paeoniflorgenone	3 beta-hydroxysteroid dehydrogenase/Delta 5-->4-isomerase type I	P14060	HSD3B1	0.046
Radix Paeoniae（RP）	MOL001918	paeoniflorgenone	3 beta-hydroxysteroid dehydrogenase/Delta 5-->4-isomerase type II	P26439	HSD3B2	0.046
Radix Paeoniae（RP）	MOL001918	paeoniflorgenone	Microtubule-associated protein 1A	P78559	MAP1A	0.046
Radix Paeoniae（RP）	MOL001918	paeoniflorgenone	Nuclear receptor coactivator 2	Q15596	NCOA2	0.047
Radix Paeoniae（RP）	MOL001918	paeoniflorgenone	Gamma-aminobutyric-acid receptor subunit beta-3	P28472	GABRB3	0.048
Radix Paeoniae（RP）	MOL001918	paeoniflorgenone	Estradiol 17-beta-dehydrogenase 1	P14061	HSD17B1	0.05
Radix Paeoniae（RP）	MOL001918	paeoniflorgenone	Alpha-1D adrenergic receptor	P25100	ADRA1D	0.05
Radix Paeoniae（RP）	MOL001918	paeoniflorgenone	5-hydroxytryptamine 1D receptor	P28221	HTR1D	0.051
Radix Paeoniae（RP）	MOL001918	paeoniflorgenone	Tripartite motif-containing protein 13	O60858	TRIM13	0.052
Radix Paeoniae（RP）	MOL001918	paeoniflorgenone	5-hydroxytryptamine 2A receptor	P28223	HTR2A	0.052
Radix Paeoniae（RP）	MOL001918	paeoniflorgenone	RAC-beta serine/threonine-protein kinase	P31751	AKT2	0.052
Radix Paeoniae（RP）	MOL001918	paeoniflorgenone	Aldo-keto reductase family 1 member C3	P42330	AKR1C3	0.055
Radix Paeoniae（RP）	MOL001918	paeoniflorgenone	Gamma-aminobutyric acid receptor subunit theta	Q9UN88	GABRQ	0.058
Radix Paeoniae（RP）	MOL001918	paeoniflorgenone	Sodium channel protein type 5 subunit alpha	Q14524	SCN5A	0.06
Radix Paeoniae（RP）	MOL001918	paeoniflorgenone	Gamma-aminobutyric-acid receptor subunit alpha-6	Q16445	GABRA6	0.06
Radix Paeoniae（RP）	MOL001918	paeoniflorgenone	Death-associated protein kinase 3	O43293	DAPK3	0.061
Radix Paeoniae（RP）	MOL001918	paeoniflorgenone	Proto-oncogene serine/threonine-protein kinase Pim-1	P11309	PIM1	0.062
Radix Paeoniae（RP）	MOL001918	paeoniflorgenone	Mediator of RNA polymerase II transcription subunit 1	Q15648	MED1	0.062
Radix Paeoniae（RP）	MOL001918	paeoniflorgenone	Dihydrofolate reductase	P00374	DHFR	0.063
Radix Paeoniae（RP）	MOL001918	paeoniflorgenone	Ig kappa chain C region	P01834	IGKC	0.063
Radix Paeoniae（RP）	MOL001918	paeoniflorgenone	Gamma-aminobutyric-acid receptor subunit beta-2	P47870	GABRB2	0.063
Radix Paeoniae（RP）	MOL001918	paeoniflorgenone	S-methyl-5-thioadenosine phosphorylase	Q13126	MTAP	0.064
Radix Paeoniae（RP）	MOL001918	paeoniflorgenone	C-jun-amino-terminal kinase-interacting protein 1	Q9UQF2	MAPK8IP1	0.064

Radix Paeoniae（RP）	MOL001918	paeoniflorgenone	5-hydroxytryptamine 1A receptor	P08908	HTR1A	0.065
Radix Paeoniae（RP）	MOL001918	paeoniflorgenone	D(4) dopamine receptor	P21917	DRD4	0.065
Radix Paeoniae（RP）	MOL001918	paeoniflorgenone	5-hydroxytryptamine 1B receptor	P28222	HTR1B	0.065
Radix Paeoniae（RP）	MOL001918	paeoniflorgenone	5-hydroxytryptamine 2C receptor	P28335	HTR2C	0.065
Radix Paeoniae（RP）	MOL001918	paeoniflorgenone	RAC-alpha serine/threonine-protein kinase	P31749	AKT1	0.065
Radix Paeoniae（RP）	MOL001918	paeoniflorgenone	Aldo-keto reductase family 1 member C1	Q04828	AKR1C1	0.065
Radix Paeoniae（RP）	MOL001918	paeoniflorgenone	Tyrosine-protein phosphatase non-receptor type 1	P18031	PTPN1	0.066
Radix Paeoniae（RP）	MOL001918	paeoniflorgenone	NADPH oxidase organizer 1	Q8NFA2	NOXO1	0.066
Radix Paeoniae（RP）	MOL001918	paeoniflorgenone	DNA polymerase kappa	Q9UBT6	POLK	0.066
Radix Paeoniae（RP）	MOL001918	paeoniflorgenone	Thyroid hormone receptor alpha	P10827	THRA	0.067
Radix Paeoniae（RP）	MOL001918	paeoniflorgenone	Cytochrome P450 19A1	P11511	CYP19A1	0.067
Radix Paeoniae（RP）	MOL001918	paeoniflorgenone	Thymidylate synthase	P04818	TYMS	0.068
Radix Paeoniae（RP）	MOL001918	paeoniflorgenone	Peroxisome proliferator-activated receptor alpha	Q07869	PPARA	0.068
Radix Paeoniae（RP）	MOL001918	paeoniflorgenone	Neuropeptide Y	P01303	NPY	0.069
Radix Paeoniae（RP）	MOL001918	paeoniflorgenone	Phosphatidylinositol-4,5-bisphosphate 3-kinase catalytic subunit gamma isoform	P48736	PIK3CG	0.069
Radix Paeoniae（RP）	MOL001918	paeoniflorgenone	ATP-binding cassette transporter sub-family C member 8	Q09428	ABCC8	0.069
Radix Paeoniae（RP）	MOL001918	paeoniflorgenone	Ig kappa chain V-II region RPMI 6410	P06310	IGKV2-30	0.07
Radix Paeoniae（RP）	MOL001918	paeoniflorgenone	Gamma-aminobutyric-acid receptor subunit alpha-4	P48169	GABRA4	0.07
Radix Paeoniae（RP）	MOL001918	paeoniflorgenone	Thyroid hormone receptor beta-1	P10828	THRB	0.071
Radix Paeoniae（RP）	MOL001918	paeoniflorgenone	Macrophage metalloelastase	P39900	MMP12	0.072
Radix Paeoniae（RP）	MOL001918	paeoniflorgenone	Thiamin pyrophosphokinase 1	Q9H3S4	TPK1	0.072
Radix Paeoniae（RP）	MOL001918	paeoniflorgenone	Nuclear receptor coactivator 5	Q9HCD5	NCOA5	0.073
Radix Paeoniae（RP）	MOL001918	paeoniflorgenone	Nuclear receptor coactivator 1	Q15788	NCOA1	0.074
Radix Paeoniae（RP）	MOL001918	paeoniflorgenone	Toll-like receptor 7	Q9NYK1	TLR7	0.074
Radix Paeoniae（RP）	MOL001918	paeoniflorgenone	Tyrosine-protein kinase HCK	P08631	HCK	0.075
Radix Paeoniae（RP）	MOL001918	paeoniflorgenone	Gamma-aminobutyric acid type B receptor, subunit 1	Q9UBS5	GABBR1	0.075
Radix Paeoniae（RP）	MOL001918	paeoniflorgenone	D(1B) dopamine receptor	P21918	DRD5	0.077
Radix Paeoniae（RP）	MOL001918	paeoniflorgenone	Alpha-1B adrenergic receptor	P35368	ADRA1B	0.078
Radix Paeoniae（RP）	MOL001918	paeoniflorgenone	D(3) dopamine receptor	P35462	DRD3	0.078
Radix Paeoniae（RP）	MOL001918	paeoniflorgenone	Muscarinic acetylcholine receptor M4	P08173	CHRM4	0.085
Radix Paeoniae（RP）	MOL001918	paeoniflorgenone	Carbonic anhydrase 1	P00915	CA1	0.086
Radix Paeoniae（RP）	MOL001918	paeoniflorgenone	Gamma-aminobutyric acid receptor subunit gamma-2	P18507	GABRG2	0.089
Radix Paeoniae（RP）	MOL001918	paeoniflorgenone	Gamma-aminobutyric acid receptor subunit gamma-1	Q8N1C3	GABRG1	0.089
Radix Paeoniae（RP）	MOL001918	paeoniflorgenone	Gamma-aminobutyric acid receptor subunit rho-3	A8MPY1	GABRR3	0.094
Radix Paeoniae（RP）	MOL001918	paeoniflorgenone	Gamma-aminobutyric acid receptor subunit pi	O00591	GABRP	0.094
Radix Paeoniae（RP）	MOL001918	paeoniflorgenone	Gamma-aminobutyric acid receptor subunit delta	O14764	GABRD	0.094
Radix Paeoniae（RP）	MOL001918	paeoniflorgenone	Gamma-aminobutyric-acid receptor subunit beta-1	P18505	GABRB1	0.094
Radix Paeoniae（RP）	MOL001918	paeoniflorgenone	Gamma-aminobutyric-acid receptor subunit rho-1	P24046	GABRR1	0.094
Radix Paeoniae（RP）	MOL001918	paeoniflorgenone	Gamma-aminobutyric acid receptor subunit rho-2	P28476	GABRR2	0.094
Radix Paeoniae（RP）	MOL001918	paeoniflorgenone	Gamma-aminobutyric acid receptor subunit epsilon	P78334	GABRE	0.094
Radix Paeoniae（RP）	MOL001918	paeoniflorgenone	Gamma-aminobutyric acid receptor subunit gamma-3	Q99928	GABRG3	0.094
Radix Paeoniae（RP）	MOL001918	paeoniflorgenone	Muscarinic acetylcholine receptor M2	P08172	CHRM2	0.099
Radix Paeoniae（RP）	MOL001918	paeoniflorgenone	Carbonic anhydrase 2	P00918	CA2	0.103
Radix Paeoniae（RP）	MOL001918	paeoniflorgenone	Alpha-2C adrenergic receptor	P18825	ADRA2C	0.104
Radix Paeoniae（RP）	MOL001918	paeoniflorgenone	Gamma-aminobutyric-acid receptor subunit alpha-3	P34903	GABRA3	0.105
Radix Paeoniae（RP）	MOL001918	paeoniflorgenone	cAMP-dependent protein kinase inhibitor alpha	P61925	PKIA	0.106
Radix Paeoniae（RP）	MOL001918	paeoniflorgenone	D(2) dopamine receptor	P14416	DRD2	0.107
Radix Paeoniae（RP）	MOL001918	paeoniflorgenone	Gamma-aminobutyric-acid receptor subunit alpha-5	P31644	GABRA5	0.11
Radix Paeoniae（RP）	MOL001918	paeoniflorgenone	Alpha-1A adrenergic receptor	P35348	ADRA1A	0.11
Radix Paeoniae（RP）	MOL001918	paeoniflorgenone	Sodium-dependent dopamine transporter	Q01959	SLC6A3	0.11
Radix Paeoniae（RP）	MOL001918	paeoniflorgenone	Gamma-aminobutyric-acid receptor subunit alpha-1	P14867	GABRA1	0.114
Radix Paeoniae（RP）	MOL001918	paeoniflorgenone	Alpha-2B adrenergic receptor	P18089	ADRA2B	0.117
Radix Paeoniae（RP）	MOL001918	paeoniflorgenone	Gamma-aminobutyric-acid receptor subunit alpha-2	P47869	GABRA2	0.119

Radix Paeoniae（RP）	MOL001918	paeoniflorgenone	Alpha-2A adrenergic receptor	P08913	ADRA2A	0.12
Radix Paeoniae（RP）	MOL001918	paeoniflorgenone	D(1A) dopamine receptor	P21728	DRD1	0.12
Radix Paeoniae（RP）	MOL001918	paeoniflorgenone	Sodium-dependent serotonin transporter	P31645	SLC6A4	0.122
Radix Paeoniae（RP）	MOL001918	paeoniflorgenone	Histamine H1 receptor	P35367	HRH1	0.124
Radix Paeoniae（RP）	MOL001918	paeoniflorgenone	Trypsin-1	P07477	PRSS1	0.13
Radix Paeoniae（RP）	MOL001918	paeoniflorgenone	Glutamate [NMDA] receptor subunit 3A	Q8TCU5	GRIN3A	0.131
Radix Paeoniae（RP）	MOL001918	paeoniflorgenone	Mineralocorticoid receptor	P08235	NR3C2	0.134
Radix Paeoniae（RP）	MOL001918	paeoniflorgenone	Nitric oxide synthase, inducible	P35228	NOS2	0.134
Radix Paeoniae（RP）	MOL001918	paeoniflorgenone	Androgen receptor	P10275	AR	0.135
Radix Paeoniae（RP）	MOL001918	paeoniflorgenone	Muscarinic acetylcholine receptor M3	P20309	CHRM3	0.145
Radix Paeoniae（RP）	MOL001918	paeoniflorgenone	Cytochrome P450 11B1, mitochondrial	P15538	CYP11B1	0.153
Radix Paeoniae（RP）	MOL001918	paeoniflorgenone	Cell division protein kinase 2	P24941	CDK2	0.177
Radix Paeoniae（RP）	MOL001918	paeoniflorgenone	Sodium-dependent noradrenaline transporter	P23975	SLC6A2	0.182
Radix Paeoniae（RP）	MOL001918	paeoniflorgenone	Glucocorticoid receptor	P04150	NR3C1	0.191
Radix Paeoniae（RP）	MOL001918	paeoniflorgenone	Muscarinic acetylcholine receptor M1	P11229	CHRM1	0.21
Radix Paeoniae（RP）	MOL001918	paeoniflorgenone	Delta-type opioid receptor	P41143	OPRD1	0.241
Radix Paeoniae（RP）	MOL001918	paeoniflorgenone	Estrogen receptor beta	Q92731	ESR2	0.264
Radix Paeoniae（RP）	MOL001918	paeoniflorgenone	Kappa-type opioid receptor	P41145	OPRK1	0.348
Radix Paeoniae（RP）	MOL001918	paeoniflorgenone	Prostaglandin G/H synthase 1	P23219	PTGS1	0.4
Radix Paeoniae（RP）	MOL001918	paeoniflorgenone	Progesterone receptor	P06401	PGR	0.502
Radix Paeoniae（RP）	MOL001918	paeoniflorgenone	Mu-type opioid receptor	P35372	OPRM1	0.524
Radix Paeoniae（RP）	MOL001918	paeoniflorgenone	cAMP-dependent protein kinase catalytic subunit alpha	P17612	PRKACA	0.595
Radix Paeoniae（RP）	MOL001918	paeoniflorgenone	Prostaglandin G/H synthase 2	P35354	PTGS2	0.805
Radix Paeoniae（RP）	MOL001918	paeoniflorgenone	Estrogen receptor	P03372	ESR1	1
Radix Paeoniae（RP）	MOL001921	Lactiflorin	Gamma-aminobutyric acid receptor subunit rho-3	A8MPY1	GABRR3	0.01
Radix Paeoniae（RP）	MOL001921	Lactiflorin	Gamma-aminobutyric acid receptor subunit pi	O00591	GABRP	0.01
Radix Paeoniae（RP）	MOL001921	Lactiflorin	Gamma-aminobutyric acid receptor subunit delta	O14764	GABRD	0.01
Radix Paeoniae（RP）	MOL001921	Lactiflorin	Gamma-aminobutyric-acid receptor subunit beta-1	P18505	GABRB1	0.01
Radix Paeoniae（RP）	MOL001921	Lactiflorin	Gamma-aminobutyric acid receptor subunit gamma-2	P18507	GABRG2	0.01
Radix Paeoniae（RP）	MOL001921	Lactiflorin	Gamma-aminobutyric-acid receptor subunit rho-1	P24046	GABRR1	0.01
Radix Paeoniae（RP）	MOL001921	Lactiflorin	Gamma-aminobutyric acid receptor subunit rho-2	P28476	GABRR2	0.01
Radix Paeoniae（RP）	MOL001921	Lactiflorin	Gamma-aminobutyric-acid receptor subunit alpha-5	P31644	GABRA5	0.01
Radix Paeoniae（RP）	MOL001921	Lactiflorin	Gamma-aminobutyric-acid receptor subunit alpha-3	P34903	GABRA3	0.01
Radix Paeoniae（RP）	MOL001921	Lactiflorin	Gamma-aminobutyric-acid receptor subunit beta-2	P47870	GABRB2	0.01
Radix Paeoniae（RP）	MOL001921	Lactiflorin	Gamma-aminobutyric-acid receptor subunit alpha-4	P48169	GABRA4	0.01
Radix Paeoniae（RP）	MOL001921	Lactiflorin	Gamma-aminobutyric acid receptor subunit epsilon	P78334	GABRE	0.01
Radix Paeoniae（RP）	MOL001921	Lactiflorin	Gamma-aminobutyric-acid receptor subunit alpha-6	Q16445	GABRA6	0.01
Radix Paeoniae（RP）	MOL001921	Lactiflorin	Gamma-aminobutyric acid receptor subunit gamma-1	Q8N1C3	GABRG1	0.01
Radix Paeoniae（RP）	MOL001921	Lactiflorin	Gamma-aminobutyric acid receptor subunit gamma-3	Q99928	GABRG3	0.01
Radix Paeoniae（RP）	MOL001921	Lactiflorin	Gamma-aminobutyric acid receptor subunit theta	Q9UN88	GABRQ	0.01
Radix Paeoniae（RP）	MOL001921	Lactiflorin	Potassium channel subfamily K member 1	O00180	KCNK1	0.031
Radix Paeoniae（RP）	MOL001921	Lactiflorin	Sodium channel protein type 5 subunit alpha	Q14524	SCN5A	0.031
Radix Paeoniae（RP）	MOL001921	Lactiflorin	Gamma-aminobutyric-acid receptor subunit alpha-1	P14867	GABRA1	0.041
Radix Paeoniae（RP）	MOL001921	Lactiflorin	Gamma-aminobutyric-acid receptor subunit alpha-2	P47869	GABRA2	0.041
Radix Paeoniae（RP）	MOL001921	Lactiflorin	Carbonic anhydrase 1	P00915	CA1	0.058
Radix Paeoniae（RP）	MOL001921	Lactiflorin	Carbonic anhydrase 2	P00918	CA2	0.058
Radix Paeoniae（RP）	MOL001921	Lactiflorin	Sodium/potassium-transporting ATPase gamma chain	P54710	FXYD2	0.058
Radix Paeoniae（RP）	MOL001921	Lactiflorin	Muscarinic acetylcholine receptor M2	P08172	CHRM2	0.079
Radix Paeoniae（RP）	MOL001921	Lactiflorin	Muscarinic acetylcholine receptor M4	P08173	CHRM4	0.079
Radix Paeoniae（RP）	MOL001921	Lactiflorin	Histone deacetylase 8	Q9BY41	HDAC8	0.101
Radix Paeoniae（RP）	MOL001921	Lactiflorin	Platelet glycoprotein IX	P14770	GP9	0.104
Radix Paeoniae（RP）	MOL001921	Lactiflorin	Muscarinic acetylcholine receptor M3	P20309	CHRM3	0.195
Radix Paeoniae（RP）	MOL001921	Lactiflorin	S-methyl-5-thioadenosine phosphorylase	Q13126	MTAP	0.211

Radix Paeoniae（RP）	MOL001921	Lactiflorin	Sodium-dependent noradrenaline transporter	P23975	SLC6A2	0.216
Radix Paeoniae（RP）	MOL001921	Lactiflorin	Sodium-dependent dopamine transporter	Q01959	SLC6A3	0.216
Radix Paeoniae（RP）	MOL001921	Lactiflorin	DNA polymerase kappa	Q9UBT6	POLK	0.218
Radix Paeoniae（RP）	MOL001921	Lactiflorin	Mu-type opioid receptor	P35372	OPRM1	0.224
Radix Paeoniae（RP）	MOL001921	Lactiflorin	Kappa-type opioid receptor	P41145	OPRK1	0.224
Radix Paeoniae（RP）	MOL001921	Lactiflorin	Hemoglobin subunit alpha	P69905	HBA1	0.234
Radix Paeoniae（RP）	MOL001921	Lactiflorin	Muscarinic acetylcholine receptor M1	P11229	CHRM1	0.311
Radix Paeoniae（RP）	MOL001924	paeoniflorin	Retinoic acid receptor alpha	P10276	RARA	0.062
Radix Paeoniae（RP）	MOL001924	paeoniflorin	Retinoic acid receptor beta	P10826	RARB	0.062
Radix Paeoniae（RP）	MOL001924	paeoniflorin	Retinoic acid receptor gamma-1	P13631	RARG	0.062
Radix Paeoniae（RP）	MOL001924	paeoniflorin	Retinoic acid receptor RXR-beta	P28702	RXRB	0.062
Radix Paeoniae（RP）	MOL001924	paeoniflorin	Retinoic acid receptor RXR-gamma	P48443	RXRG	0.062
Radix Paeoniae（RP）	MOL001924	paeoniflorin	3 beta-hydroxysteroid dehydrogenase/Delta 5-->4-isomerase type I	P14060	HSD3B1	0.11
Radix Paeoniae（RP）	MOL001924	paeoniflorin	Estradiol 17-beta-dehydrogenase 1	P14061	HSD17B1	0.11
Radix Paeoniae（RP）	MOL001924	paeoniflorin	Nociceptin receptor	P41146	OPRL1	0.121
Radix Paeoniae（RP）	MOL001924	paeoniflorin	5-hydroxytryptamine 3 receptor	P46098	HTR3A	0.122
Radix Paeoniae（RP）	MOL001924	paeoniflorin	Glutamate [NMDA] receptor subunit epsilon-1	Q12879	GRIN2A	0.122
Radix Paeoniae（RP）	MOL001924	paeoniflorin	Glutamate [NMDA] receptor subunit epsilon-2	Q13224	GRIN2B	0.122
Radix Paeoniae（RP）	MOL001924	paeoniflorin	Muscarinic acetylcholine receptor M2	P08172	CHRM2	0.15
Radix Paeoniae（RP）	MOL001924	paeoniflorin	Muscarinic acetylcholine receptor M4	P08173	CHRM4	0.15
Radix Paeoniae（RP）	MOL001924	paeoniflorin	Muscarinic acetylcholine receptor M3	P20309	CHRM3	0.15
Radix Paeoniae（RP）	MOL001924	paeoniflorin	Progesterone receptor	P06401	PGR	0.175
Radix Paeoniae（RP）	MOL001924	paeoniflorin	Mineralocorticoid receptor	P08235	NR3C2	0.175
Radix Paeoniae（RP）	MOL001924	paeoniflorin	Tripartite motif-containing protein 13	O60858	TRIM13	0.268
Radix Paeoniae（RP）	MOL001924	paeoniflorin	Glutamate [NMDA] receptor subunit 3A	Q8TCU5	GRIN3A	0.271
Radix Paeoniae（RP）	MOL001924	paeoniflorin	Muscarinic acetylcholine receptor M1	P11229	CHRM1	0.326
Radix Paeoniae（RP）	MOL001924	paeoniflorin	S-methyl-5-thioadenosine phosphorylase	Q13126	MTAP	0.376
Radix Paeoniae（RP）	MOL001924	paeoniflorin	Estrogen receptor beta	Q92731	ESR2	0.385
Radix Paeoniae（RP）	MOL001924	paeoniflorin	Serine/threonine-protein kinase 6	O14965	AURKA	0.402
Radix Paeoniae（RP）	MOL001924	paeoniflorin	Delta-type opioid receptor	P41143	OPRD1	0.414
Radix Paeoniae（RP）	MOL001924	paeoniflorin	Estrogen receptor	P03372	ESR1	0.809
Radix Paeoniae（RP）	MOL001924	paeoniflorin	Mu-type opioid receptor	P35372	OPRM1	1
Radix Paeoniae（RP）	MOL001924	paeoniflorin	Kappa-type opioid receptor	P41145	OPRK1	1
Radix Paeoniae（RP）	MOL001925	paeoniflorin_qt	Alpha-1D adrenergic receptor	P25100	ADRA1D	0.021
Radix Paeoniae（RP）	MOL001925	paeoniflorin_qt	Alpha-1A adrenergic receptor	P35348	ADRA1A	0.021
Radix Paeoniae（RP）	MOL001925	paeoniflorin_qt	Alpha-1B adrenergic receptor	P35368	ADRA1B	0.021
Radix Paeoniae（RP）	MOL001925	paeoniflorin_qt	Potassium voltage-gated channel subfamily H member 2	Q12809	KCNH2	0.021
Radix Paeoniae（RP）	MOL001925	paeoniflorin_qt	Potassium voltage-gated channel subfamily H member 6	Q9H252	KCNH6	0.021
Radix Paeoniae（RP）	MOL001925	paeoniflorin_qt	Lactotransferrin	P02788	LTF	0.023
Radix Paeoniae（RP）	MOL001925	paeoniflorin_qt	Mannose-binding protein C	P11226	MBL2	0.023
Radix Paeoniae（RP）	MOL001925	paeoniflorin_qt	Lactase-phlorizin hydrolase	P09848	LCT	0.025
Radix Paeoniae（RP）	MOL001925	paeoniflorin_qt	Ig gamma-1 chain C region	P01857	IGHG1	0.03
Radix Paeoniae（RP）	MOL001925	paeoniflorin_qt	5-hydroxytryptamine 3 receptor	P46098	HTR3A	0.032
Radix Paeoniae（RP）	MOL001925	paeoniflorin_qt	Sodium-dependent dopamine transporter	Q01959	SLC6A3	0.032
Radix Paeoniae（RP）	MOL001925	paeoniflorin_qt	Glutamate [NMDA] receptor subunit 3A	Q8TCU5	GRIN3A	0.032
Radix Paeoniae（RP）	MOL001925	paeoniflorin_qt	Sodium channel protein type 10 subunit alpha	Q9Y5Y9	SCN10A	0.032
Radix Paeoniae（RP）	MOL001925	paeoniflorin_qt	Gamma-aminobutyric-acid receptor subunit alpha-5	P31644	GABRA5	0.036
Radix Paeoniae（RP）	MOL001925	paeoniflorin_qt	Gamma-aminobutyric-acid receptor subunit alpha-3	P34903	GABRA3	0.036
Radix Paeoniae（RP）	MOL001925	paeoniflorin_qt	Neuronal acetylcholine receptor subunit alpha-7	P36544	CHRNA7	0.036
Radix Paeoniae（RP）	MOL001925	paeoniflorin_qt	Neuronal acetylcholine receptor subunit alpha-4	P43681	CHRNA4	0.036
Radix Paeoniae（RP）	MOL001925	paeoniflorin_qt	Gamma-aminobutyric-acid receptor subunit alpha-4	P48169	GABRA4	0.036
Radix Paeoniae（RP）	MOL001925	paeoniflorin_qt	Gamma-aminobutyric-acid receptor subunit alpha-6	Q16445	GABRA6	0.036
Radix Paeoniae（RP）	MOL001925	paeoniflorin_qt	Trypsin-2	P07478	PRSS2	0.043

Radix Paeoniae（RP）	MOL001925	paeoniflorin_qt	Muscarinic acetylcholine receptor M2	P08172	CHRM2	0.055
Radix Paeoniae（RP）	MOL001925	paeoniflorin_qt	Muscarinic acetylcholine receptor M4	P08173	CHRM4	0.055
Radix Paeoniae（RP）	MOL001925	paeoniflorin_qt	Muscarinic acetylcholine receptor M1	P11229	CHRM1	0.055
Radix Paeoniae（RP）	MOL001925	paeoniflorin_qt	Amiloride-sensitive sodium channel subunit alpha	P37088	SCNN1A	0.055
Radix Paeoniae（RP）	MOL001925	paeoniflorin_qt	Amiloride-sensitive sodium channel subunit beta	P51168	SCNN1B	0.055
Radix Paeoniae（RP）	MOL001925	paeoniflorin_qt	Amiloride-sensitive sodium channel subunit gamma	P51170	SCNN1G	0.055
Radix Paeoniae（RP）	MOL001925	paeoniflorin_qt	Estrogen receptor	P03372	ESR1	0.057
Radix Paeoniae（RP）	MOL001925	paeoniflorin_qt	DNA topoisomerase 2-alpha	P11388	TOP2A	0.057
Radix Paeoniae（RP）	MOL001925	paeoniflorin_qt	Protein tyrosine kinase 2 beta	Q14289	PTK2B	0.057
Radix Paeoniae（RP）	MOL001925	paeoniflorin_qt	Nuclear receptor coactivator 1	Q15788	NCOA1	0.057
Radix Paeoniae（RP）	MOL001925	paeoniflorin_qt	Beta-1 adrenergic receptor	P08588	ADRB1	0.06
Radix Paeoniae（RP）	MOL001925	paeoniflorin_qt	Glutamate receptor, ionotropic kainate 2	Q13002	GRIK2	0.06
Radix Paeoniae（RP）	MOL001925	paeoniflorin_qt	Gamma-aminobutyric-acid receptor subunit alpha-1	P14867	GABRA1	0.067
Radix Paeoniae（RP）	MOL001925	paeoniflorin_qt	Gamma-aminobutyric-acid receptor subunit alpha-2	P47869	GABRA2	0.067
Radix Paeoniae（RP）	MOL001925	paeoniflorin_qt	Pancreatic alpha-amylase	P04746	AMY2A	0.092
Radix Paeoniae（RP）	MOL001925	paeoniflorin_qt	NAD(P)H dehydrogenase [quinone] 1	P15559	NQO1	0.103
Radix Paeoniae（RP）	MOL001925	paeoniflorin_qt	Vitamin K epoxide reductase complex subunit 1	Q9BQB6	VKORC1	0.103
Radix Paeoniae（RP）	MOL001925	paeoniflorin_qt	Estrogen receptor beta	Q92731	ESR2	0.137
Radix Paeoniae（RP）	MOL001925	paeoniflorin_qt	Group IIE secretory phospholipase A2	Q9NZK7	PLA2G2E	0.181
Radix Paeoniae（RP）	MOL001925	paeoniflorin_qt	cAMP-dependent protein kinase catalytic subunit alpha	P17612	PRKACA	0.194
Radix Paeoniae（RP）	MOL001925	paeoniflorin_qt	Methionine aminopeptidase 2	P50579	METAP2	0.202
Radix Paeoniae（RP）	MOL001925	paeoniflorin_qt	Serine/threonine-protein kinase 6	O14965	AURKA	0.205
Radix Paeoniae（RP）	MOL001925	paeoniflorin_qt	Hemoglobin subunit alpha	P69905	HBA1	0.207
Radix Paeoniae（RP）	MOL001925	paeoniflorin_qt	Tubulin alpha-3 chain	Q71U36	TUBA1A	0.213
Radix Paeoniae（RP）	MOL001925	paeoniflorin_qt	ATP-binding cassette transporter sub-family C member 8	Q09428	ABCC8	0.223
Radix Paeoniae（RP）	MOL001925	paeoniflorin_qt	Sodium channel protein type 5 subunit alpha	Q14524	SCN5A	0.232
Radix Paeoniae（RP）	MOL001925	paeoniflorin_qt	Prostaglandin G/H synthase 1	P23219	PTGS1	0.714
Radix Paeoniae（RP）	MOL001925	paeoniflorin_qt	Prostaglandin G/H synthase 2	P35354	PTGS2	1
Radix Paeoniae（RP）	MOL002249	gallocatechin	Matrix metalloproteinase-9	P14780	MMP9	Validated
Radix Paeoniae（RP）	MOL002714	baicalein	Voltage-dependent T-type calcium channel subunit alpha-1G	O43497	CACNA1G	0.01
Radix Paeoniae（RP）	MOL002714	baicalein	Carbonic anhydrase 12	O43570	CA12	0.01
Radix Paeoniae（RP）	MOL002714	baicalein	Carbonic anhydrase-related protein 11	O75493	CA11	0.01
Radix Paeoniae（RP）	MOL002714	baicalein	Voltage-dependent T-type calcium channel subunit alpha-1H	O95180	CACNA1H	0.01
Radix Paeoniae（RP）	MOL002714	baicalein	Carbonic anhydrase 3	P07451	CA3	0.01
Radix Paeoniae（RP）	MOL002714	baicalein	Sodium/hydrogen exchanger 1	P19634	SLC9A1	0.01
Radix Paeoniae（RP）	MOL002714	baicalein	Amiloride-sensitive amine oxidase [copper-containing]	P19801	AOC1	0.01
Radix Paeoniae（RP）	MOL002714	baicalein	Carbonic anhydrase 6	P23280	CA6	0.01
Radix Paeoniae（RP）	MOL002714	baicalein	Amine oxidase [flavin-containing] B	P27338	MAOB	0.01
Radix Paeoniae（RP）	MOL002714	baicalein	Carbonic anhydrase 5A, mitochondrial	P35218	CA5A	0.01
Radix Paeoniae（RP）	MOL002714	baicalein	Carbonic anhydrase-related protein	P35219	CA8	0.01
Radix Paeoniae（RP）	MOL002714	baicalein	Sodium channel protein type 1 subunit alpha	P35498	SCN1A	0.01
Radix Paeoniae（RP）	MOL002714	baicalein	Sodium channel protein type 4 subunit alpha	P35499	SCN4A	0.01
Radix Paeoniae（RP）	MOL002714	baicalein	Carbonic anhydrase 7	P43166	CA7	0.01
Radix Paeoniae（RP）	MOL002714	baicalein	Amiloride-sensitive sodium channel subunit delta	P51172	SCNN1D	0.01
Radix Paeoniae（RP）	MOL002714	baicalein	Amiloride-sensitive cation channel 2, neuronal	P78348	ASIC1	0.01
Radix Paeoniae（RP）	MOL002714	baicalein	Sodium channel subunit beta-1	Q07699	SCN1B	0.01
Radix Paeoniae（RP）	MOL002714	baicalein	Sodium channel protein type 9 subunit alpha	Q15858	SCN9A	0.01
Radix Paeoniae（RP）	MOL002714	baicalein	Amiloride-sensitive cation channel 1, neuronal	Q16515	ASIC2	0.01
Radix Paeoniae（RP）	MOL002714	baicalein	Carbonic anhydrase 9	Q16790	CA9	0.01
Radix Paeoniae（RP）	MOL002714	baicalein	Opioid receptor, sigma 1	Q5T1J1	SIGMAR1	0.01
Radix Paeoniae（RP）	MOL002714	baicalein	Sodium channel subunit beta-4	Q8IWT1	SCN4B	0.01
Radix Paeoniae（RP）	MOL002714	baicalein	Carbonic anhydrase 13	Q8N1Q1	CA13	0.01
Radix Paeoniae（RP）	MOL002714	baicalein	Sodium channel protein type 2 subunit alpha	Q99250	SCN2A	0.01

Radix Paeoniae（RP）	MOL002714	baicalein	Sigma 1-type opioid receptor	Q99720	SIGMAR1	0.01
Radix Paeoniae（RP）	MOL002714	baicalein	Carbonic anhydrase-related protein 10	Q9NS85	CA10	0.01
Radix Paeoniae（RP）	MOL002714	baicalein	Sodium channel protein type 3 subunit alpha	Q9NY46	SCN3A	0.01
Radix Paeoniae（RP）	MOL002714	baicalein	Sodium channel subunit beta-3	Q9NY72	SCN3B	0.01
Radix Paeoniae（RP）	MOL002714	baicalein	Voltage-dependent T-type calcium channel subunit alpha-1I	Q9P0X4	CACNA1I	0.01
Radix Paeoniae（RP）	MOL002714	baicalein	Sodium channel protein type 11 subunit alpha	Q9UI33	SCN11A	0.01
Radix Paeoniae（RP）	MOL002714	baicalein	Carbonic anhydrase 14	Q9ULX7	CA14	0.01
Radix Paeoniae（RP）	MOL002714	baicalein	Potassium channel subfamily K member 6	Q9Y257	KCNK6	0.01
Radix Paeoniae（RP）	MOL002714	baicalein	Carbonic anhydrase 5B, mitochondrial	Q9Y2D0	CA5B	0.01
Radix Paeoniae（RP）	MOL002714	baicalein	Retinoic acid receptor alpha	P10276	RARA	0.011
Radix Paeoniae（RP）	MOL002714	baicalein	Retinoic acid receptor beta	P10826	RARB	0.011
Radix Paeoniae（RP）	MOL002714	baicalein	Retinoic acid receptor gamma-1	P13631	RARG	0.011
Radix Paeoniae（RP）	MOL002714	baicalein	Retinoic acid receptor RXR-beta	P28702	RXRB	0.011
Radix Paeoniae（RP）	MOL002714	baicalein	Retinoic acid receptor RXR-gamma	P48443	RXRG	0.011
Radix Paeoniae（RP）	MOL002714	baicalein	Furin	P09958	FURIN	0.012
Radix Paeoniae（RP）	MOL002714	baicalein	Sodium/potassium-transporting ATPase gamma chain	P54710	FXYD2	0.012
Radix Paeoniae（RP）	MOL002714	baicalein	D1 dopamine receptor-interacting protein calcyon	Q9NYX4	CALY	0.012
Radix Paeoniae（RP）	MOL002714	baicalein	Glycolipid transfer protein	Q9NZD2	GLTP	0.012
Radix Paeoniae（RP）	MOL002714	baicalein	Gamma-aminobutyric acid receptor subunit rho-3	A8MPY1	GABRR3	0.013
Radix Paeoniae（RP）	MOL002714	baicalein	Phosphatidylinositol 3-kinase regulatory subunit beta	O00459	PIK3R2	0.013
Radix Paeoniae（RP）	MOL002714	baicalein	Gamma-aminobutyric acid receptor subunit pi	O00591	GABRP	0.013
Radix Paeoniae（RP）	MOL002714	baicalein	Gamma-aminobutyric acid receptor subunit delta	O14764	GABRD	0.013
Radix Paeoniae（RP）	MOL002714	baicalein	Beta-3 adrenergic receptor	P13945	ADRB3	0.013
Radix Paeoniae（RP）	MOL002714	baicalein	Gamma-aminobutyric-acid receptor subunit beta-1	P18505	GABRB1	0.013
Radix Paeoniae（RP）	MOL002714	baicalein	Gamma-aminobutyric acid receptor subunit gamma-2	P18507	GABRG2	0.013
Radix Paeoniae（RP）	MOL002714	baicalein	Gamma-aminobutyric-acid receptor subunit rho-1	P24046	GABRR1	0.013
Radix Paeoniae（RP）	MOL002714	baicalein	Phosphatidylinositol 3-kinase regulatory subunit alpha	P27986	PIK3R1	0.013
Radix Paeoniae（RP）	MOL002714	baicalein	Gamma-aminobutyric acid receptor subunit rho-2	P28476	GABRR2	0.013
Radix Paeoniae（RP）	MOL002714	baicalein	Mitogen-activated protein kinase 1	P28482	MAPK1	0.013
Radix Paeoniae（RP）	MOL002714	baicalein	Solute carrier family 12 member 2	P55011	SLC12A2	0.013
Radix Paeoniae（RP）	MOL002714	baicalein	Gamma-aminobutyric acid receptor subunit epsilon	P78334	GABRE	0.013
Radix Paeoniae（RP）	MOL002714	baicalein	Gamma-aminobutyric acid receptor subunit gamma-1	Q8N1C3	GABRG1	0.013
Radix Paeoniae（RP）	MOL002714	baicalein	Gamma-aminobutyric acid receptor subunit gamma-3	Q99928	GABRG3	0.013
Radix Paeoniae（RP）	MOL002714	baicalein	Neuronal acetylcholine receptor subunit alpha-3	P32297	CHRNA3	0.014
Radix Paeoniae（RP）	MOL002714	baicalein	Neuronal acetylcholine receptor subunit alpha-4	P43681	CHRNA4	0.014
Radix Paeoniae（RP）	MOL002714	baicalein	Gamma-aminobutyric-acid receptor subunit alpha-4	P48169	GABRA4	0.014
Radix Paeoniae（RP）	MOL002714	baicalein	Chromaffin granule amine transporter	P54219	SLC18A1	0.014
Radix Paeoniae（RP）	MOL002714	baicalein	Synaptic vesicular amine transporter	Q05940	SLC18A2	0.014
Radix Paeoniae（RP）	MOL002714	baicalein	Gamma-aminobutyric-acid receptor subunit alpha-6	Q16445	GABRA6	0.014
Radix Paeoniae（RP）	MOL002714	baicalein	Protein S100-B	P04271	S100B	0.015
Radix Paeoniae（RP）	MOL002714	baicalein	Trypsin-2	P07478	PRSS2	0.015
Radix Paeoniae（RP）	MOL002714	baicalein	Interleukin-3	P08700	IL3	0.015
Radix Paeoniae（RP）	MOL002714	baicalein	Protein S100-A1	P23297	S100A1	0.015
Radix Paeoniae（RP）	MOL002714	baicalein	Histamine H1 receptor	P35367	HRH1	0.015
Radix Paeoniae（RP）	MOL002714	baicalein	Muscarinic acetylcholine receptor M4	P08173	CHRM4	0.016
Radix Paeoniae（RP）	MOL002714	baicalein	Muscarinic acetylcholine receptor M1	P11229	CHRM1	0.016
Radix Paeoniae（RP）	MOL002714	baicalein	Elongation factor 2	P13639	EEF2	0.016
Radix Paeoniae（RP）	MOL002714	baicalein	Muscarinic acetylcholine receptor M3	P20309	CHRM3	0.016
Radix Paeoniae（RP）	MOL002714	baicalein	Poly [ADP-ribose] polymerase 3	Q9Y6F1	PARP3	0.016
Radix Paeoniae（RP）	MOL002714	baicalein	Purine nucleoside phosphorylase	P00491	PNP	0.018
Radix Paeoniae（RP）	MOL002714	baicalein	DNA polymerase alpha catalytic subunit	P09884	POLA1	0.018
Radix Paeoniae（RP）	MOL002714	baicalein	Ribonucleoside-diphosphate reductase large subunit	P23921	RRM1	0.018
Radix Paeoniae（RP）	MOL002714	baicalein	Ribonucleoside-diphosphate reductase M2 subunit	P31350	RRM2	0.018

Radix Paeoniae（RP）	MOL002714	baicalein	DNA polymerase epsilon subunit 2	P56282	POLE2	0.018
Radix Paeoniae（RP）	MOL002714	baicalein	DNA polymerase epsilon catalytic subunit A	Q07864	POLE	0.018
Radix Paeoniae（RP）	MOL002714	baicalein	Ribonucleoside-diphosphate reductase subunit M2 B	Q7LG56	RRM2B	0.018
Radix Paeoniae（RP）	MOL002714	baicalein	DNA polymerase epsilon subunit 3	Q9NRF9	POLE3	0.018
Radix Paeoniae（RP）	MOL002714	baicalein	Cytochrome b	P00156	MT-CYB	0.019
Radix Paeoniae（RP）	MOL002714	baicalein	Cytochrome c1, heme protein, mitochondrial	P08574	CYC1	0.019
Radix Paeoniae（RP）	MOL002714	baicalein	Cytochrome b-c1 complex subunit Rieske, mitochondrial	P47985	UQCRFS1	0.019
Radix Paeoniae（RP）	MOL002714	baicalein	Neuronal acetylcholine receptor subunit alpha-2	Q15822	CHRNA2	0.019
Radix Paeoniae（RP）	MOL002714	baicalein	5-hydroxytryptamine 4 receptor	Q13639	HTR4	0.02
Radix Paeoniae（RP）	MOL002714	baicalein	Cell division protein kinase 4	P11802	CDK4	0.021
Radix Paeoniae（RP）	MOL002714	baicalein	Cell division protein kinase 7	P50613	CDK7	0.021
Radix Paeoniae（RP）	MOL002714	baicalein	Cell division protein kinase 9	P50750	CDK9	0.021
Radix Paeoniae（RP）	MOL002714	baicalein	Cell division protein kinase 6	Q00534	CDK6	0.021
Radix Paeoniae（RP）	MOL002714	baicalein	Bile salt sulfotransferase	Q06520	SULT2A1	0.021
Radix Paeoniae（RP）	MOL002714	baicalein	Dehydrogenase/reductase SDR family member 8	Q8NBQ5	HSD17B11	0.021
Radix Paeoniae（RP）	MOL002714	baicalein	Potassium channel subfamily K member 1	O00180	KCNK1	0.022
Radix Paeoniae（RP）	MOL002714	baicalein	Serine/threonine-protein kinase 17B	O94768	STK17B	0.022
Radix Paeoniae（RP）	MOL002714	baicalein	Gamma-aminobutyric-acid receptor subunit alpha-5	P31644	GABRA5	0.022
Radix Paeoniae（RP）	MOL002714	baicalein	Gamma-aminobutyric-acid receptor subunit alpha-3	P34903	GABRA3	0.022
Radix Paeoniae（RP）	MOL002714	baicalein	ATP synthase subunit gamma, mitochondrial	P36542	ATP5F1C	0.022
Radix Paeoniae（RP）	MOL002714	baicalein	UDP-glucuronosyltransferase 3A1	Q6NUS8	UGT3A1	0.022
Radix Paeoniae（RP）	MOL002714	baicalein	Neuronal acetylcholine receptor subunit alpha-7	P36544	CHRNA7	0.023
Radix Paeoniae（RP）	MOL002714	baicalein	Cyclin-dependent kinase 5 activator 1	Q15078	CDK5R1	0.023
Radix Paeoniae（RP）	MOL002714	baicalein	3-phosphoinositide-dependent protein kinase 1	O15530	PDPK1	0.024
Radix Paeoniae（RP）	MOL002714	baicalein	Tyrosine-protein kinase Lyn	P07948	LYN	0.024
Radix Paeoniae（RP）	MOL002714	baicalein	Tyrosine-protein kinase CSK	P41240	CSK	0.024
Radix Paeoniae（RP）	MOL002714	baicalein	Tyrosine-protein kinase ZAP-70	P43403	ZAP70	0.024
Radix Paeoniae（RP）	MOL002714	baicalein	Tyrosine-protein kinase SYK	P43405	SYK	0.024
Radix Paeoniae（RP）	MOL002714	baicalein	Protein kinase C theta type	Q04759	PRKCQ	0.024
Radix Paeoniae（RP）	MOL002714	baicalein	Tyrosine-protein kinase ITK/TSK	Q08881	ITK	0.024
Radix Paeoniae（RP）	MOL002714	baicalein	85 kDa calcium-independent phospholipase A2	O60733	PLA2G6	0.025
Radix Paeoniae（RP）	MOL002714	baicalein	Amiloride-sensitive sodium channel subunit alpha	P37088	SCNN1A	0.025
Radix Paeoniae（RP）	MOL002714	baicalein	Cytosolic phospholipase A2	P47712	PLA2G4A	0.025
Radix Paeoniae（RP）	MOL002714	baicalein	Amiloride-sensitive sodium channel subunit beta	P51168	SCNN1B	0.025
Radix Paeoniae（RP）	MOL002714	baicalein	Amiloride-sensitive sodium channel subunit gamma	P51170	SCNN1G	0.025
Radix Paeoniae（RP）	MOL002714	baicalein	Carbonic anhydrase 4	P22748	CA4	0.026
Radix Paeoniae（RP）	MOL002714	baicalein	Tyrosine-protein kinase JAK2	O60674	JAK2	0.027
Radix Paeoniae（RP）	MOL002714	baicalein	Tyrosine-protein kinase JAK1	P23458	JAK1	0.027
Radix Paeoniae（RP）	MOL002714	baicalein	Tyrosine-protein kinase JAK3	P52333	JAK3	0.027
Radix Paeoniae（RP）	MOL002714	baicalein	Tripartite motif-containing protein 13	O60858	TRIM13	0.028
Radix Paeoniae（RP）	MOL002714	baicalein	cAMP response element-binding protein	P16220	CREB1	0.028
Radix Paeoniae（RP）	MOL002714	baicalein	Glutamate [NMDA] receptor subunit 3A	Q8TCU5	GRIN3A	0.028
Radix Paeoniae（RP）	MOL002714	baicalein	Phenylalanine-4-hydroxylase	P00439	PAH	0.029
Radix Paeoniae（RP）	MOL002714	baicalein	Amine oxidase [flavin-containing] A	P21397	MAOA	0.029
Radix Paeoniae（RP）	MOL002714	baicalein	Potassium voltage-gated channel subfamily H member 2	Q12809	KCNH2	0.029
Radix Paeoniae（RP）	MOL002714	baicalein	Potassium voltage-gated channel subfamily H member 6	Q9H252	KCNH6	0.029
Radix Paeoniae（RP）	MOL002714	baicalein	Sodium channel protein type 10 subunit alpha	Q9Y5Y9	SCN10A	0.03
Radix Paeoniae（RP）	MOL002714	baicalein	Solute carrier family 12 member 1	Q13621	SLC12A1	0.031
Radix Paeoniae（RP）	MOL002714	baicalein	Methionine aminopeptidase 1	P53582	METAP1	0.032
Radix Paeoniae（RP）	MOL002714	baicalein	Calcium/calmodulin-dependent protein kinase type II subunit delta	Q13557	CAMK2D	0.032
Radix Paeoniae（RP）	MOL002714	baicalein	STE20-like serine/threonine-protein kinase	Q9H2G2	SLK	0.032
Radix Paeoniae（RP）	MOL002714	baicalein	DNA-(apurinic or apyrimidinic site) lyase	P27695	APEX1	0.033
Radix Paeoniae（RP）	MOL002714	baicalein	Gamma-aminobutyric-acid receptor subunit alpha-1	P14867	GABRA1	0.034

Radix Paeoniae（RP）	MOL002714	baicalein	Mitogen-activated protein kinase 8	P45983	MAPK8	0.034
Radix Paeoniae（RP）	MOL002714	baicalein	Gamma-aminobutyric-acid receptor subunit alpha-2	P47869	GABRA2	0.034
Radix Paeoniae（RP）	MOL002714	baicalein	Mitogen-activated protein kinase 10	P53779	MAPK10	0.034
Radix Paeoniae（RP）	MOL002714	baicalein	Inhibitor of nuclear factor kappa-B kinase subunit beta	O14920	IKBKB	0.035
Radix Paeoniae（RP）	MOL002714	baicalein	Phospholipase A2, membrane associated	P14555	PLA2G2A	0.035
Radix Paeoniae（RP）	MOL002714	baicalein	Casein kinase I isoform gamma-2	P78368	CSNK1G2	0.035
Radix Paeoniae（RP）	MOL002714	baicalein	Protein S100-A12	P80511	S100A12	0.035
Radix Paeoniae（RP）	MOL002714	baicalein	Lactoylglutathione lyase	Q04760	GLO1	0.035
Radix Paeoniae（RP）	MOL002714	baicalein	Prostaglandin reductase 2	Q8N8N7	PTGR2	0.035
Radix Paeoniae（RP）	MOL002714	baicalein	Serine/threonine-protein kinase haspin	Q8TF76	HASPIN	0.035
Radix Paeoniae（RP）	MOL002714	baicalein	Protein S100-A13	Q99584	S100A13	0.035
Radix Paeoniae（RP）	MOL002714	baicalein	Group IIE secretory phospholipase A2	Q9NZK7	PLA2G2E	0.035
Radix Paeoniae（RP）	MOL002714	baicalein	Cystine/glutamate transporter	Q9UPY5	SLC7A11	0.035
Radix Paeoniae（RP）	MOL002714	baicalein	Ig kappa chain C region	P01834	IGKC	0.036
Radix Paeoniae（RP）	MOL002714	baicalein	Ig gamma-1 chain C region	P01857	IGHG1	0.036
Radix Paeoniae（RP）	MOL002714	baicalein	Myeloperoxidase	P05164	MPO	0.036
Radix Paeoniae（RP）	MOL002714	baicalein	Muscarinic acetylcholine receptor M2	P08172	CHRM2	0.036
Radix Paeoniae（RP）	MOL002714	baicalein	DNA topoisomerase 1	P11387	TOP1	0.036
Radix Paeoniae（RP）	MOL002714	baicalein	Eosinophil peroxidase	P11678	EPX	0.036
Radix Paeoniae（RP）	MOL002714	baicalein	Calreticulin	P27797	CALR	0.036
Radix Paeoniae（RP）	MOL002714	baicalein	Sepiapterin reductase	P35270	SPR	0.036
Radix Paeoniae（RP）	MOL002714	baicalein	Melatonin receptor type 1B	P49286	MTNR1B	0.036
Radix Paeoniae（RP）	MOL002714	baicalein	Calmodulin	P62158		0.036
Radix Paeoniae（RP）	MOL002714	baicalein	6-pyruvoyl tetrahydrobiopterin synthase	Q03393	PTS	0.036
Radix Paeoniae（RP）	MOL002714	baicalein	Nuclear receptor ROR-beta	Q92753	RORB	0.036
Radix Paeoniae（RP）	MOL002714	baicalein	DNA topoisomerase I, mitochondrial	Q969P6	TOP1MT	0.036
Radix Paeoniae（RP）	MOL002714	baicalein	Tyrosyl-tRNA synthetase, cytoplasmic	P54577	YARS	0.037
Radix Paeoniae（RP）	MOL002714	baicalein	Peptidyl-prolyl cis-trans isomerase NIMA-interacting 1	Q13526	PIN1	0.037
Radix Paeoniae（RP）	MOL002714	baicalein	Urokinase-type plasminogen activator	P00749	PLAU	0.038
Radix Paeoniae（RP）	MOL002714	baicalein	5-hydroxytryptamine 3 receptor	P46098	HTR3A	0.038
Radix Paeoniae（RP）	MOL002714	baicalein	Glycogen phosphorylase, muscle form	P11217	PYGM	0.039
Radix Paeoniae（RP）	MOL002714	baicalein	Peptidyl-prolyl cis-trans isomerase, mitochondrial	P30405	PPIF	0.039
Radix Paeoniae（RP）	MOL002714	baicalein	Tubulin alpha-1 chain	P68366	TUBA4A	0.04
Radix Paeoniae（RP）	MOL002714	baicalein	3-oxo-5-alpha-steroid 4-dehydrogenase 2	P31213	SRD5A2	0.041
Radix Paeoniae（RP）	MOL002714	baicalein	Sterol O-acyltransferase 2	O75908	SOAT2	0.043
Radix Paeoniae（RP）	MOL002714	baicalein	Gonadotropin-releasing hormone receptor	P30968	GNRHR	0.043
Radix Paeoniae（RP）	MOL002714	baicalein	Sterol O-acyltransferase 1	P35610	SOAT1	0.043
Radix Paeoniae（RP）	MOL002714	baicalein	Gonadotropin-releasing hormone II receptor	Q96P88	GNRHR2	0.043
Radix Paeoniae（RP）	MOL002714	baicalein	Microtubule-associated protein 2	P11137	MAP2	0.044
Radix Paeoniae（RP）	MOL002714	baicalein	3 beta-hydroxysteroid dehydrogenase/Delta 5-->4-isomerase type I	P14060	HSD3B1	0.044
Radix Paeoniae（RP）	MOL002714	baicalein	3 beta-hydroxysteroid dehydrogenase/Delta 5-->4-isomerase type II	P26439	HSD3B2	0.044
Radix Paeoniae（RP）	MOL002714	baicalein	Nitric-oxide synthase, brain	P29475	NOS1	0.044
Radix Paeoniae（RP）	MOL002714	baicalein	Estrogen-related receptor gamma	P62508	ESRRG	0.044
Radix Paeoniae（RP）	MOL002714	baicalein	Microtubule-associated protein 1A	P78559	MAP1A	0.044
Radix Paeoniae（RP）	MOL002714	baicalein	Keratin, type II cytoskeletal 7	P08729	KRT7	0.045
Radix Paeoniae（RP）	MOL002714	baicalein	Platelet glycoprotein IX	P14770	GP9	0.045
Radix Paeoniae（RP）	MOL002714	baicalein	cAMP-specific 3',5'-cyclic phosphodiesterase 4C	Q08493	PDE4C	0.045
Radix Paeoniae（RP）	MOL002714	baicalein	cGMP-inhibited 3',5'-cyclic phosphodiesterase A	Q14432	PDE3A	0.045
Radix Paeoniae（RP）	MOL002714	baicalein	Epidermal growth factor receptor	P00533	EGFR	0.046
Radix Paeoniae（RP）	MOL002714	baicalein	5-hydroxytryptamine 1B receptor	P28222	HTR1B	0.046
Radix Paeoniae（RP）	MOL002714	baicalein	5-hydroxytryptamine 2C receptor	P28335	HTR2C	0.046
Radix Paeoniae（RP）	MOL002714	baicalein	5-hydroxytryptamine 2B receptor	P41595	HTR2B	0.046
Radix Paeoniae（RP）	MOL002714	baicalein	ATP synthase subunit beta, mitochondrial	P06576	ATP5F1B	0.047

Radix Paeoniae（RP）	MOL002714	baicalein	Tyrosine-protein kinase HCK	P08631	HCK	0.047
Radix Paeoniae（RP）	MOL002714	baicalein	ATP synthase subunit alpha, mitochondrial	P25705	ATP5F1A	0.047
Radix Paeoniae（RP）	MOL002714	baicalein	Phosphatidylinositol-4,5-bisphosphate 3-kinase catalytic subunit gamma isoform	P48736	PIK3CG	0.047
Radix Paeoniae（RP）	MOL002714	baicalein	Protein tyrosine kinase 2 beta	Q14289	PTK2B	0.047
Radix Paeoniae（RP）	MOL002714	baicalein	Proto-oncogene serine/threonine-protein kinase Pim-1	P11309	PIM1	0.049
Radix Paeoniae（RP）	MOL002714	baicalein	Sodium-dependent dopamine transporter	Q01959	SLC6A3	0.049
Radix Paeoniae（RP）	MOL002714	baicalein	Glycogen synthase kinase-3 beta	P49841	GSK3B	0.05
Radix Paeoniae（RP）	MOL002714	baicalein	Tubulin beta-2C chain	P68371	TUBB4B	0.05
Radix Paeoniae（RP）	MOL002714	baicalein	Proto-oncogene tyrosine-protein kinase LCK	P06239	LCK	0.051
Radix Paeoniae（RP）	MOL002714	baicalein	DNA (cytosine-5)-methyltransferase 1	P26358	DNMT1	0.056
Radix Paeoniae（RP）	MOL002714	baicalein	Toll-like receptor 7	Q9NYK1	TLR7	0.057
Radix Paeoniae（RP）	MOL002714	baicalein	Death-associated protein kinase 3	O43293	DAPK3	0.058
Radix Paeoniae（RP）	MOL002714	baicalein	Carbonic anhydrase 1	P00915	CA1	0.058
Radix Paeoniae（RP）	MOL002714	baicalein	5-hydroxytryptamine 1D receptor	P28221	HTR1D	0.062
Radix Paeoniae（RP）	MOL002714	baicalein	Serine/threonine-protein kinase 6	O14965	AURKA	0.067
Radix Paeoniae（RP）	MOL002714	baicalein	Hepatocyte growth factor receptor	P08581	MET	0.067
Radix Paeoniae（RP）	MOL002714	baicalein	Mannose-binding protein C	P11226	MBL2	0.068
Radix Paeoniae（RP）	MOL002714	baicalein	5-hydroxytryptamine 2A receptor	P28223	HTR2A	0.068
Radix Paeoniae（RP）	MOL002714	baicalein	Coagulation factor VII	P08709	F7	0.069
Radix Paeoniae（RP）	MOL002714	baicalein	Tyrosine-protein phosphatase non-receptor type 1	P18031	PTPN1	0.069
Radix Paeoniae（RP）	MOL002714	baicalein	Aldo-keto reductase family 1 member C1	Q04828	AKR1C1	0.069
Radix Paeoniae（RP）	MOL002714	baicalein	5-hydroxytryptamine 1A receptor	P08908	HTR1A	0.07
Radix Paeoniae（RP）	MOL002714	baicalein	Dihydrofolate reductase	P00374	DHFR	0.071
Radix Paeoniae（RP）	MOL002714	baicalein	Ig kappa chain V-II region RPMI 6410	P06310	IGKV2-30	0.071
Radix Paeoniae（RP）	MOL002714	baicalein	Thyroid hormone receptor alpha	P10827	THRA	0.071
Radix Paeoniae（RP）	MOL002714	baicalein	Casein kinase II subunit alpha	P68400	CSNK2A1	0.071
Radix Paeoniae（RP）	MOL002714	baicalein	cAMP-specific 3',5'-cyclic phosphodiesterase 4D	Q08499	PDE4D	0.071
Radix Paeoniae（RP）	MOL002714	baicalein	L-amino-acid oxidase	Q96RQ9	IL4I1	0.071
Radix Paeoniae（RP）	MOL002714	baicalein	Cannabinoid receptor 1	P21554	CNR1	0.073
Radix Paeoniae（RP）	MOL002714	baicalein	Endothelin-1 receptor	P25101	EDNRA	0.073
Radix Paeoniae（RP）	MOL002714	baicalein	C-jun-amino-terminal kinase-interacting protein 1	Q9UQF2	MAPK8IP1	0.073
Radix Paeoniae（RP）	MOL002714	baicalein	Calcium/calmodulin-dependent protein kinase type II alpha chain	Q9UQM7	CAMK2A	0.073
Radix Paeoniae（RP）	MOL002714	baicalein	Carbonic anhydrase 2	P00918	CA2	0.074
Radix Paeoniae（RP）	MOL002714	baicalein	Ribosyldihydronicotinamide dehydrogenase [quinone]	P16083	NQO2	0.074
Radix Paeoniae（RP）	MOL002714	baicalein	Sodium-dependent noradrenaline transporter	P23975	SLC6A2	0.074
Radix Paeoniae（RP）	MOL002714	baicalein	Melatonin receptor type 1A	P48039	MTNR1A	0.074
Radix Paeoniae（RP）	MOL002714	baicalein	Triosephosphate isomerase	P60174	TPI1	0.074
Radix Paeoniae（RP）	MOL002714	baicalein	S-methyl-5-thioadenosine phosphorylase	Q13126	MTAP	0.074
Radix Paeoniae（RP）	MOL002714	baicalein	Membrane copper amine oxidase	Q16853	AOC3	0.075
Radix Paeoniae（RP）	MOL002714	baicalein	Phospholipase A2	P04054	PLA2G1B	0.078
Radix Paeoniae（RP）	MOL002714	baicalein	Beta-2 adrenergic receptor	P07550	ADRB2	0.079
Radix Paeoniae（RP）	MOL002714	baicalein	D(4) dopamine receptor	P21917	DRD4	0.079
Radix Paeoniae（RP）	MOL002714	baicalein	MAP kinase-activated protein kinase 2	P49137	MAPKAPK2	0.08
Radix Paeoniae（RP）	MOL002714	baicalein	Sodium channel protein type 5 subunit alpha	Q14524	SCN5A	0.082
Radix Paeoniae（RP）	MOL002714	baicalein	Nuclear receptor coactivator 5	Q9HCD5	NCOA5	0.083
Radix Paeoniae（RP）	MOL002714	baicalein	Interferon gamma	P01579	IFNG	0.085
Radix Paeoniae（RP）	MOL002714	baicalein	3-oxo-5-alpha-steroid 4-dehydrogenase 1	P18405	SRD5A1	0.087
Radix Paeoniae（RP）	MOL002714	baicalein	Androgen receptor	P10275	AR	0.089
Radix Paeoniae（RP）	MOL002714	baicalein	Proto-oncogene tyrosine-protein kinase Src	P12931	SRC	0.089
Radix Paeoniae（RP）	MOL002714	baicalein	Nitric-oxide synthase, endothelial	P29474	NOS3	0.092
Radix Paeoniae（RP）	MOL002714	baicalein	Sodium-dependent serotonin transporter	P31645	SLC6A4	0.093
Radix Paeoniae（RP）	MOL002714	baicalein	cAMP-specific 3',5'-cyclic phosphodiesterase 4A	P27815	PDE4A	0.094
Radix Paeoniae（RP）	MOL002714	baicalein	Alpha-2A adrenergic receptor	P08913	ADRA2A	0.095

Radix Paeoniae（RP）	MOL002714	baicalein	Alpha-2C adrenergic receptor	P18825	ADRA2C	0.095
Radix Paeoniae（RP）	MOL002714	baicalein	D(3) dopamine receptor	P35462	DRD3	0.095
Radix Paeoniae（RP）	MOL002714	baicalein	DNA polymerase kappa	Q9UBT6	POLK	0.095
Radix Paeoniae（RP）	MOL002714	baicalein	Beta-1 adrenergic receptor	P08588	ADRB1	0.096
Radix Paeoniae（RP）	MOL002714	baicalein	Alpha-1D adrenergic receptor	P25100	ADRA1D	0.096
Radix Paeoniae（RP）	MOL002714	baicalein	D-HSCDK2	O75100	CA11	0.1
Radix Paeoniae（RP）	MOL002714	baicalein	Cell division control protein 2 homolog	P06493	CDK1	0.1
Radix Paeoniae（RP）	MOL002714	baicalein	Tubulin alpha-3 chain	Q71U36	TUBA1A	0.103
Radix Paeoniae（RP）	MOL002714	baicalein	cAMP-dependent protein kinase inhibitor alpha	P61925	PKIA	0.107
Radix Paeoniae（RP）	MOL002714	baicalein	Rho-associated protein kinase 1	Q13464	ROCK1	0.107
Radix Paeoniae（RP）	MOL002714	baicalein	Inhibitor of nuclear factor kappa-B kinase subunit alpha	O15111	CHUK	0.112
Radix Paeoniae（RP）	MOL002714	baicalein	Arachidonate 5-lipoxygenase	P09917	ALOX5	0.112
Radix Paeoniae（RP）	MOL002714	baicalein	Alpha-2B adrenergic receptor	P18089	ADRA2B	0.112
Radix Paeoniae（RP）	MOL002714	baicalein	D(1B) dopamine receptor	P21918	DRD5	0.112
Radix Paeoniae（RP）	MOL002714	baicalein	Cell division protein kinase 5	Q00535	CDK5	0.127
Radix Paeoniae（RP）	MOL002714	baicalein	DNA topoisomerase 2-alpha	P11388	TOP2A	0.128
Radix Paeoniae（RP）	MOL002714	baicalein	Alpha-1B adrenergic receptor	P35368	ADRA1B	0.129
Radix Paeoniae（RP）	MOL002714	baicalein	Progesterone receptor	P06401	PGR	0.136
Radix Paeoniae（RP）	MOL002714	baicalein	Estradiol 17-beta-dehydrogenase 1	P14061	HSD17B1	0.142
Radix Paeoniae（RP）	MOL002714	baicalein	D(2) dopamine receptor	P14416	DRD2	0.145
Radix Paeoniae（RP）	MOL002714	baicalein	Hemoglobin subunit alpha	P69905	HBA1	0.146
Radix Paeoniae（RP）	MOL002714	baicalein	Peroxisome proliferator-activated receptor gamma	P37231	PPARG	0.151
Radix Paeoniae（RP）	MOL002714	baicalein	Delta-type opioid receptor	P41143	OPRD1	0.156
Radix Paeoniae（RP）	MOL002714	baicalein	RAC-alpha serine/threonine-protein kinase	P31749	AKT1	0.158
Radix Paeoniae（RP）	MOL002714	baicalein	Alpha-1A adrenergic receptor	P35348	ADRA1A	0.162
Radix Paeoniae（RP）	MOL002714	baicalein	D(1A) dopamine receptor	P21728	DRD1	0.178
Radix Paeoniae（RP）	MOL002714	baicalein	cAMP-specific 3',5'-cyclic phosphodiesterase 4B	Q07343	PDE4B	0.192
Radix Paeoniae（RP）	MOL002714	baicalein	Prothrombin	P00734	F2	0.197
Radix Paeoniae（RP）	MOL002714	baicalein	Kappa-type opioid receptor	P41145	OPRK1	0.22
Radix Paeoniae（RP）	MOL002714	baicalein	Nitric oxide synthase, inducible	P35228	NOS2	0.237
Radix Paeoniae（RP）	MOL002714	baicalein	Cell division protein kinase 2	P24941	CDK2	0.241
Radix Paeoniae（RP）	MOL002714	baicalein	Nuclear receptor coactivator 1	Q15788	NCOA1	0.271
Radix Paeoniae（RP）	MOL002714	baicalein	Trypsin-1	P07477	PRSS1	0.332
Radix Paeoniae（RP）	MOL002714	baicalein	Mu-type opioid receptor	P35372	OPRM1	0.402
Radix Paeoniae（RP）	MOL002714	baicalein	Prostaglandin G/H synthase 1	P23219	PTGS1	0.422
Radix Paeoniae（RP）	MOL002714	baicalein	cAMP-dependent protein kinase catalytic subunit alpha	P17612	PRKACA	0.441
Radix Paeoniae（RP）	MOL002714	baicalein	Estrogen receptor beta	Q92731	ESR2	0.491
Radix Paeoniae（RP）	MOL002714	baicalein	Cyclin-A2	P20248	CCNA2	0.677
Radix Paeoniae（RP）	MOL002714	baicalein	Prostaglandin G/H synthase 2	P35354	PTGS2	0.778
Radix Paeoniae（RP）	MOL002714	baicalein	Estrogen receptor	P03372	ESR1	1
Radix Paeoniae（RP）	MOL002776	Baicalin	Ribosyldihydronicotinamide dehydrogenase [quinone]	P16083	NQO2	0.094
Radix Paeoniae（RP）	MOL002776	Baicalin	Prostaglandin G/H synthase 1	P23219	PTGS1	0.094
Radix Paeoniae（RP）	MOL002776	Baicalin	Casein kinase II subunit alpha	P68400	CSNK2A1	0.094
Radix Paeoniae（RP）	MOL002776	Baicalin	ATP synthase subunit beta, mitochondrial	P06576	ATP5F1B	0.148
Radix Paeoniae（RP）	MOL002776	Baicalin	ATP synthase subunit alpha, mitochondrial	P25705	ATP5F1A	0.148
Radix Paeoniae（RP）	MOL002776	Baicalin	Estrogen receptor	P03372	ESR1	0.231
Radix Paeoniae（RP）	MOL002776	Baicalin	Glycogen phosphorylase, muscle form	P11217	PYGM	0.323
Radix Paeoniae（RP）	MOL002776	Baicalin	DNA polymerase kappa	Q9UBT6	POLK	0.343
Radix Paeoniae（RP）	MOL002776	Baicalin	Nuclear receptor coactivator 1	Q15788	NCOA1	0.489
Radix Paeoniae（RP）	MOL002776	Baicalin	Estrogen receptor beta	Q92731	ESR2	1
Radix Paeoniae（RP）	MOL002883	Ethyl oleate (NF)	Toll-like receptor 4	O00206	TLR4	0.018
Radix Paeoniae（RP）	MOL002883	Ethyl oleate (NF)	Peroxisomal 3,2-trans-enoyl-CoA isomerase	O75521	ECI2	0.018
Radix Paeoniae（RP）	MOL002883	Ethyl oleate (NF)	Proto-oncogene tyrosine-protein kinase ABL1	P00519	ABL1	0.018

Radix Paeoniae（RP）	MOL002883	Ethyl oleate (NF)	Insulin	P01308	INS	0.018
Radix Paeoniae（RP）	MOL002883	Ethyl oleate (NF)	Poliovirus receptor	P15151	PVR	0.018
Radix Paeoniae（RP）	MOL002883	Ethyl oleate (NF)	cAMP-dependent protein kinase catalytic subunit alpha	P17612	PRKACA	0.018
Radix Paeoniae（RP）	MOL002883	Ethyl oleate (NF)	Ganglioside GM2 activator	P17900	GM2A	0.018
Radix Paeoniae（RP）	MOL002883	Ethyl oleate (NF)	Recoverin	P35243	RCVRN	0.018
Radix Paeoniae（RP）	MOL002883	Ethyl oleate (NF)	Hepatocyte nuclear factor 4-alpha	P41235	HNF4A	0.018
Radix Paeoniae（RP）	MOL002883	Ethyl oleate (NF)	Guanylyl cyclase-activating protein 1	P43080	GUCA1A	0.018
Radix Paeoniae（RP）	MOL002883	Ethyl oleate (NF)	cAMP-dependent protein kinase inhibitor alpha	P61925	PKIA	0.018
Radix Paeoniae（RP）	MOL002883	Ethyl oleate (NF)	Calmodulin	P62158		0.018
Radix Paeoniae（RP）	MOL002883	Ethyl oleate (NF)	ADP-ribosylation factor 6	P62330	ARF6	0.018
Radix Paeoniae（RP）	MOL002883	Ethyl oleate (NF)	FK506-binding protein 1A	P62942	FKBP1A	0.018
Radix Paeoniae（RP）	MOL002883	Ethyl oleate (NF)	Calcineurin subunit B isoform 1	P63098	PPP3R1	0.018
Radix Paeoniae（RP）	MOL002883	Ethyl oleate (NF)	ADP-ribosylation factor 1	P84077	ARF1	0.018
Radix Paeoniae（RP）	MOL002883	Ethyl oleate (NF)	Serine/threonine-protein phosphatase 2B catalytic subunit alpha isoform	Q08209	PPP3CA	0.018
Radix Paeoniae（RP）	MOL002883	Ethyl oleate (NF)	Nuclear receptor coactivator 1	Q15788	NCOA1	0.018
Radix Paeoniae（RP）	MOL002883	Ethyl oleate (NF)	SEC14-like protein 2	O76054	SEC14L2	0.027
Radix Paeoniae（RP）	MOL002883	Ethyl oleate (NF)	Alpha-lactalbumin	P00709	LALBA	0.027
Radix Paeoniae（RP）	MOL002883	Ethyl oleate (NF)	Glycodelin	P09466	PAEP	0.027
Radix Paeoniae（RP）	MOL002883	Ethyl oleate (NF)	Cytochrome P450 2C8	P10632	CYP2C8	0.027
Radix Paeoniae（RP）	MOL002883	Ethyl oleate (NF)	Hepatocyte nuclear factor 4-gamma	Q14541	HNF4G	0.027
Radix Paeoniae（RP）	MOL002883	Ethyl oleate (NF)	Trafficking protein particle complex subunit 6A	O75865	TRAPPC6A	0.05
Radix Paeoniae（RP）	MOL002883	Ethyl oleate (NF)	Trafficking protein particle complex subunit 6B	Q86SZ2	TRAPPC6B	0.05
Radix Paeoniae（RP）	MOL002883	Ethyl oleate (NF)	Trafficking protein particle complex subunit 5	Q8IUR0	TRAPPC5	0.05
Radix Paeoniae（RP）	MOL002883	Ethyl oleate (NF)	Trafficking protein particle complex subunit 4	Q9Y296	TRAPPC4	0.05
Radix Paeoniae（RP）	MOL002883	Ethyl oleate (NF)	Trafficking protein particle complex subunit 1	Q9Y5R8	TRAPPC1	0.05
Radix Paeoniae（RP）	MOL002883	Ethyl oleate (NF)	Dihydroorotate dehydrogenase, mitochondrial	Q02127	DHODH	0.071
Radix Paeoniae（RP）	MOL002883	Ethyl oleate (NF)	Retinoic acid receptor alpha	P10276	RARA	0.089
Radix Paeoniae（RP）	MOL002883	Ethyl oleate (NF)	Retinoic acid receptor beta	P10826	RARB	0.089
Radix Paeoniae（RP）	MOL002883	Ethyl oleate (NF)	Retinoic acid receptor gamma-1	P13631	RARG	0.089
Radix Paeoniae（RP）	MOL002883	Ethyl oleate (NF)	Retinoic acid receptor RXR-beta	P28702	RXRB	0.089
Radix Paeoniae（RP）	MOL002883	Ethyl oleate (NF)	Retinoic acid receptor RXR-gamma	P48443	RXRG	0.089
Radix Paeoniae（RP）	MOL002883	Ethyl oleate (NF)	Myelin P2 protein	P02689	PMP2	0.092
Radix Paeoniae（RP）	MOL002883	Ethyl oleate (NF)	Lymphocyte antigen 96	Q9Y6Y9	LY96	0.093
Radix Paeoniae（RP）	MOL002883	Ethyl oleate (NF)	BC269730_2	O60427	FADS1	0.172
Radix Paeoniae（RP）	MOL002883	Ethyl oleate (NF)	Delta-6 fatty acid desaturase	O95864	FADS2	0.172
Radix Paeoniae（RP）	MOL002883	Ethyl oleate (NF)	Rhodopsin	P08100	RHO	0.172
Radix Paeoniae（RP）	MOL002883	Ethyl oleate (NF)	Sodium/calcium exchanger 1	P32418	SLC8A1	0.172
Radix Paeoniae（RP）	MOL002883	Ethyl oleate (NF)	Transient receptor potential cation channel subfamily V member 1	Q8NER1	TRPV1	0.172
Radix Paeoniae（RP）	MOL002883	Ethyl oleate (NF)	Furin	P09958	FURIN	0.178
Radix Paeoniae（RP）	MOL002883	Ethyl oleate (NF)	Trafficking protein particle complex subunit 3	O43617	TRAPPC3	0.225
Radix Paeoniae（RP）	MOL002883	Ethyl oleate (NF)	Phospholipase A2	P04054	PLA2G1B	0.362
Radix Paeoniae（RP）	MOL002883	Ethyl oleate (NF)	Phospholipase A2, membrane associated	P14555	PLA2G2A	0.362
Radix Paeoniae（RP）	MOL002883	Ethyl oleate (NF)	Hemoglobin subunit alpha	P69905	HBA1	0.688
Radix Paeoniae（RP）	MOL002883	Ethyl oleate (NF)	Glycolipid transfer protein	Q9NZD2	GLTP	0.739
Radix Paeoniae（RP）	MOL002883	Ethyl oleate (NF)	Cannabinoid receptor 2	P34972	CNR2	0.746
Radix Paeoniae（RP）	MOL002883	Ethyl oleate (NF)	Neutrophil gelatinase-associated lipocalin	P80188	LCN2	0.812
Radix Paeoniae（RP）	MOL002883	Ethyl oleate (NF)	Lysozyme C	P61626	LYZ	0.873
Radix Paeoniae（RP）	MOL002883	Ethyl oleate (NF)	Vitamin K-dependent protein C	P04070	PROC	0.96
Radix Paeoniae（RP）	MOL002883	Ethyl oleate (NF)	Colipase	P04118	CLPS	0.983
Radix Paeoniae（RP）	MOL004355	Spinasterol	Muscarinic acetylcholine receptor M2	P08172	CHRM2	0.038
Radix Paeoniae（RP）	MOL004355	Spinasterol	Muscarinic acetylcholine receptor M4	P08173	CHRM4	0.038
Radix Paeoniae（RP）	MOL004355	Spinasterol	Muscarinic acetylcholine receptor M5	P08912	CHRM5	0.038
Radix Paeoniae（RP）	MOL004355	Spinasterol	Muscarinic acetylcholine receptor M1	P11229	CHRM1	0.038

Radix Paeoniae（RP）	MOL004355	Spinasterol	Sodium-dependent noradrenaline transporter	P23975	SLC6A2	0.038
Radix Paeoniae（RP）	MOL004355	Spinasterol	Histamine H1 receptor	P35367	HRH1	0.038
Radix Paeoniae（RP）	MOL004355	Spinasterol	Potassium channel subfamily K member 1	O00180	KCNK1	0.056
Radix Paeoniae（RP）	MOL004355	Spinasterol	Sodium channel protein type 5 subunit alpha	Q14524	SCN5A	0.056
Radix Paeoniae（RP）	MOL004355	Spinasterol	Potassium channel subfamily K member 6	Q9Y257	KCNK6	0.056
Radix Paeoniae（RP）	MOL004355	Spinasterol	Ig kappa chain C region	P01834	IGKC	0.058
Radix Paeoniae（RP）	MOL004355	Spinasterol	Ig gamma-1 chain C region	P01857	IGHG1	0.058
Radix Paeoniae（RP）	MOL004355	Spinasterol	Ig gamma-2 chain C region	P01859	IGHG2	0.058
Radix Paeoniae（RP）	MOL004355	Spinasterol	Proto-oncogene tyrosine-protein kinase LCK	P06239	LCK	0.085
Radix Paeoniae（RP）	MOL004355	Spinasterol	Tyrosine-protein kinase Lyn	P07948	LYN	0.085
Radix Paeoniae（RP）	MOL004355	Spinasterol	Muscarinic acetylcholine receptor M3	P20309	CHRM3	0.086
Radix Paeoniae（RP）	MOL004355	Spinasterol	Mu-type opioid receptor	P35372	OPRM1	0.086
Radix Paeoniae（RP）	MOL004355	Spinasterol	Kappa-type opioid receptor	P41145	OPRK1	0.086
Radix Paeoniae（RP）	MOL004355	Spinasterol	Platelet glycoprotein IX	P14770	GP9	0.088
Radix Paeoniae（RP）	MOL004355	Spinasterol	3-oxo-5-alpha-steroid 4-dehydrogenase 1	P18405	SRD5A1	0.089
Radix Paeoniae（RP）	MOL004355	Spinasterol	3-oxo-5-alpha-steroid 4-dehydrogenase 2	P31213	SRD5A2	0.089
Radix Paeoniae（RP）	MOL004355	Spinasterol	Bile salt sulfotransferase	Q06520	SULT2A1	0.095
Radix Paeoniae（RP）	MOL004355	Spinasterol	Microtubule-associated protein 2	P11137	MAP2	0.109
Radix Paeoniae（RP）	MOL004355	Spinasterol	Microtubule-associated protein 1A	P78559	MAP1A	0.109
Radix Paeoniae（RP）	MOL004355	Spinasterol	Prolactin receptor	P16471	PRLR	0.111
Radix Paeoniae（RP）	MOL004355	Spinasterol	Retinoic acid receptor RXR-alpha	P19793	RXRA	0.113
Radix Paeoniae（RP）	MOL004355	Spinasterol	Gonadotropin-releasing hormone receptor	P30968	GNRHR	0.113
Radix Paeoniae（RP）	MOL004355	Spinasterol	Nuclear receptor subfamily 1 group I member 3	Q14994	NR1I3	0.113
Radix Paeoniae（RP）	MOL004355	Spinasterol	Gonadotropin-releasing hormone II receptor	Q96P88	GNRHR2	0.113
Radix Paeoniae（RP）	MOL004355	Spinasterol	Corticosteroid 11-beta-dehydrogenase isozyme 1	P28845	HSD11B1	0.123
Radix Paeoniae（RP）	MOL004355	Spinasterol	Sodium-dependent serotonin transporter	P31645	SLC6A4	0.184
Radix Paeoniae（RP）	MOL004355	Spinasterol	Death-associated protein kinase 3	O43293	DAPK3	0.186
Radix Paeoniae（RP）	MOL004355	Spinasterol	Nuclear receptor coactivator 5	Q9HCD5	NCOA5	0.193
Radix Paeoniae（RP）	MOL004355	Spinasterol	Mediator of RNA polymerase II transcription subunit 1	Q15648	MED1	0.195
Radix Paeoniae（RP）	MOL004355	Spinasterol	Aldo-keto reductase family 1 member C1	Q04828	AKR1C1	0.2
Radix Paeoniae（RP）	MOL004355	Spinasterol	Androgen receptor	P10275	AR	0.233
Radix Paeoniae（RP）	MOL004355	Spinasterol	Cannabinoid receptor 2	P34972	CNR2	0.233
Radix Paeoniae（RP）	MOL004355	Spinasterol	Estradiol 17-beta-dehydrogenase 1	P14061	HSD17B1	0.303
Radix Paeoniae（RP）	MOL004355	Spinasterol	Nuclear receptor coactivator 1	Q15788	NCOA1	0.36
Radix Paeoniae（RP）	MOL004355	Spinasterol	Glucocorticoid receptor	P04150	NR3C1	0.409
Radix Paeoniae（RP）	MOL004355	Spinasterol	Mineralocorticoid receptor	P08235	NR3C2	0.646
Radix Paeoniae（RP）	MOL004355	Spinasterol	Estrogen receptor	P03372	ESR1	0.962
Radix Paeoniae（RP）	MOL004355	Spinasterol	Progesterone receptor	P06401	PGR	1
Radix Paeoniae（RP）	MOL004480	acetic acid	Ras-specific guanine nucleotide-releasing factor 2	O14827	RASGRF2	Validated
Radix Paeoniae（RP）	MOL004480	acetic acid	Superoxide dismutase [Cu-Zn]	P00441	SOD1	Validated
Radix Paeoniae（RP）	MOL004480	acetic acid	Urokinase-type plasminogen activator	P00749	PLAU	Validated
Radix Paeoniae（RP）	MOL004480	acetic acid	Tissue-type plasminogen activator	P00750	PLAT	Validated
Radix Paeoniae（RP）	MOL004480	acetic acid	Interstitial collagenase	P03956	MMP1	Validated
Radix Paeoniae（RP）	MOL004480	acetic acid	Granulocyte-macrophage colony-stimulating factor	P04141	CSF2	Validated
Radix Paeoniae（RP）	MOL004480	acetic acid	Superoxide dismutase [Mn], mitochondrial	P04179	SOD2	Validated
Radix Paeoniae（RP）	MOL004480	acetic acid	Interleukin-6	P05231	IL6	Validated
Radix Paeoniae（RP）	MOL004480	acetic acid	Lipoprotein lipase	P06858	LPL	Validated
Radix Paeoniae（RP）	MOL004480	acetic acid	Arachidonate 5-lipoxygenase	P09917	ALOX5	Validated
Radix Paeoniae（RP）	MOL004480	acetic acid	Cytochrome P450 19A1	P11511	CYP19A1	Validated
Radix Paeoniae（RP）	MOL004480	acetic acid	Neutrophil cytosol factor 1	P14598	NCF1	Validated
Radix Paeoniae（RP）	MOL004480	acetic acid	G2/mitotic-specific cyclin-B1	P14635	CCNB1	Validated
Radix Paeoniae（RP）	MOL004480	acetic acid	Pancreatic triacylglycerol lipase	P16233	PNLIP	Validated
Radix Paeoniae（RP）	MOL004480	acetic acid	Cyclin-A2	P20248	CCNA2	Validated

Radix Paeoniae（RP）	MOL004480	acetic acid	Catechol O-methyltransferase	P21964	COMT	Validated
Radix Paeoniae（RP）	MOL004480	acetic acid	Cell division protein kinase 2	P24941	CDK2	Validated
Radix Paeoniae（RP）	MOL004480	acetic acid	Caspase-3	P42574	CASP3	Validated
Radix Paeoniae（RP）	MOL004480	acetic acid	Type I iodothyronine deiodinase	P49895	DIO1	Validated
Radix Paeoniae（RP）	MOL004480	acetic acid	High affinity immunoglobulin epsilon receptor subunit beta	Q01362	MS4A2	Validated
Radix Paeoniae（RP）	MOL004480	acetic acid	Serine/threonine-protein kinase Sgk3	Q96BR1	SGK3	Validated
Radix Paeoniae（RP）	MOL005043	campest-5-en-3beta-ol	Ig kappa chain C region	P01834	IGKC	0.013
Radix Paeoniae（RP）	MOL005043	campest-5-en-3beta-ol	Ig gamma-1 chain C region	P01857	IGHG1	0.013
Radix Paeoniae（RP）	MOL005043	campest-5-en-3beta-ol	Ig gamma-2 chain C region	P01859	IGHG2	0.013
Radix Paeoniae（RP）	MOL005043	campest-5-en-3beta-ol	Dehydrogenase/reductase SDR family member 8	Q8NBQ5	HSD17B11	0.013
Radix Paeoniae（RP）	MOL005043	campest-5-en-3beta-ol	Muscarinic acetylcholine receptor M2	P08172	CHRM2	0.022
Radix Paeoniae（RP）	MOL005043	campest-5-en-3beta-ol	Neuronal acetylcholine receptor subunit alpha-2	Q15822	CHRNA2	0.022
Radix Paeoniae（RP）	MOL005043	campest-5-en-3beta-ol	Serine/threonine-protein phosphatase 2A catalytic subunit alpha isoform	P67775	PPP2CA	0.029
Radix Paeoniae（RP）	MOL005043	campest-5-en-3beta-ol	Serine/threonine-protein phosphatase 2A 56 kDa regulatory subunit gamma isoform	Q13362	PPP2R5C	0.029
Radix Paeoniae（RP）	MOL005043	campest-5-en-3beta-ol	Annexin A1	P04083	ANXA1	0.03
Radix Paeoniae（RP）	MOL005043	campest-5-en-3beta-ol	Nuclear receptor 0B1	P51843	NR0B1	0.03
Radix Paeoniae（RP）	MOL005043	campest-5-en-3beta-ol	Cytosolic phospholipase A2	P47712	PLA2G4A	0.033
Radix Paeoniae（RP）	MOL005043	campest-5-en-3beta-ol	3 beta-hydroxysteroid dehydrogenase/Delta 5-->4-isomerase type II	P26439	HSD3B2	0.034
Radix Paeoniae（RP）	MOL005043	campest-5-en-3beta-ol	Corticosteroid 11-beta-dehydrogenase isozyme 1	P28845	HSD11B1	0.035
Radix Paeoniae（RP）	MOL005043	campest-5-en-3beta-ol	Gonadotropin-releasing hormone receptor	P30968	GNRHR	0.037
Radix Paeoniae（RP）	MOL005043	campest-5-en-3beta-ol	Gonadotropin-releasing hormone II receptor	Q96P88	GNRHR2	0.037
Radix Paeoniae（RP）	MOL005043	campest-5-en-3beta-ol	Microtubule-associated protein 2	P11137	MAP2	0.038
Radix Paeoniae（RP）	MOL005043	campest-5-en-3beta-ol	Prolactin receptor	P16471	PRLR	0.038
Radix Paeoniae（RP）	MOL005043	campest-5-en-3beta-ol	Microtubule-associated protein 1A	P78559	MAP1A	0.038
Radix Paeoniae（RP）	MOL005043	campest-5-en-3beta-ol	Retinoic acid receptor RXR-alpha	P19793	RXRA	0.042
Radix Paeoniae（RP）	MOL005043	campest-5-en-3beta-ol	Nuclear receptor subfamily 1 group I member 3	Q14994	NR1I3	0.042
Radix Paeoniae（RP）	MOL005043	campest-5-en-3beta-ol	3-oxo-5-alpha-steroid 4-dehydrogenase 1	P18405	SRD5A1	0.044
Radix Paeoniae（RP）	MOL005043	campest-5-en-3beta-ol	Bile salt sulfotransferase	Q06520	SULT2A1	0.056
Radix Paeoniae（RP）	MOL005043	campest-5-en-3beta-ol	cAMP-dependent protein kinase catalytic subunit alpha	P17612	PRKACA	0.071
Radix Paeoniae（RP）	MOL005043	campest-5-en-3beta-ol	Cannabinoid receptor 1	P21554	CNR1	0.077
Radix Paeoniae（RP）	MOL005043	campest-5-en-3beta-ol	DNA polymerase kappa	Q9UBT6	POLK	0.08
Radix Paeoniae（RP）	MOL005043	campest-5-en-3beta-ol	NADPH oxidase organizer 1	Q8NFA2	NOXO1	0.082
Radix Paeoniae（RP）	MOL005043	campest-5-en-3beta-ol	Mediator of RNA polymerase II transcription subunit 1	Q15648	MED1	0.085
Radix Paeoniae（RP）	MOL005043	campest-5-en-3beta-ol	Nitric-oxide synthase, endothelial	P29474	NOS3	0.086
Radix Paeoniae（RP）	MOL005043	campest-5-en-3beta-ol	Cannabinoid receptor 2	P34972	CNR2	0.087
Radix Paeoniae（RP）	MOL005043	campest-5-en-3beta-ol	3 beta-hydroxysteroid dehydrogenase/Delta 5-->4-isomerase type I	P14060	HSD3B1	0.089
Radix Paeoniae（RP）	MOL005043	campest-5-en-3beta-ol	Nuclear receptor coactivator 5	Q9HCD5	NCOA5	0.101
Radix Paeoniae（RP）	MOL005043	campest-5-en-3beta-ol	3-oxo-5-alpha-steroid 4-dehydrogenase 2	P31213	SRD5A2	0.119
Radix Paeoniae（RP）	MOL005043	campest-5-en-3beta-ol	Estradiol 17-beta-dehydrogenase 1	P14061	HSD17B1	0.166
Radix Paeoniae（RP）	MOL005043	campest-5-en-3beta-ol	Androgen receptor	P10275	AR	0.173
Radix Paeoniae（RP）	MOL005043	campest-5-en-3beta-ol	Nuclear receptor coactivator 1	Q15788	NCOA1	0.189
Radix Paeoniae（RP）	MOL005043	campest-5-en-3beta-ol	Estrogen receptor beta	Q92731	ESR2	0.194
Radix Paeoniae（RP）	MOL005043	campest-5-en-3beta-ol	Glucocorticoid receptor	P04150	NR3C1	0.221
Radix Paeoniae（RP）	MOL005043	campest-5-en-3beta-ol	Prostaglandin G/H synthase 2	P35354	PTGS2	0.292
Radix Paeoniae（RP）	MOL005043	campest-5-en-3beta-ol	Mineralocorticoid receptor	P08235	NR3C2	0.298
Radix Paeoniae（RP）	MOL005043	campest-5-en-3beta-ol	Progesterone receptor	P06401	PGR	0.702
Radix Paeoniae（RP）	MOL005043	campest-5-en-3beta-ol	Estrogen receptor	P03372	ESR1	1
Radix Paeoniae（RP）	MOL006791	epigallocatechin	Interleukin-8	P10145	IL8	Validated
Radix Paeoniae（RP）	MOL006990	(1S,2S,4R)-trans-2-hydroxy-1,8-cineole-B-D-glucopyranoside	Acetylcholine receptor subunit alpha	P02708	CHRNA1	0.006
Radix Paeoniae（RP）	MOL006990	(1S,2S,4R)-trans-2-hydroxy-1,8-cineole-B-D-glucopyranoside	Cholinesterase	P06276	BCHE	0.006
Radix Paeoniae（RP）	MOL006990	(1S,2S,4R)-trans-2-hydroxy-1,8-cineole-B-D-glucopyranoside	Acetylcholine receptor subunit gamma	P07510	CHRNG	0.006
Radix Paeoniae（RP）	MOL006990	(1S,2S,4R)-trans-2-hydroxy-1,8-cineole-B-D-glucopyranoside	Acetylcholine receptor subunit beta	P11230	CHRNB1	0.006

Radix Paeoniae（RP）	MOL006990	(1S,2S,4R)-trans-2-hydroxy-1,8-cineole-B-D-glucopyranoside	Neuronal acetylcholine receptor subunit beta-2	P17787	CHRNB2	0.006
Radix Paeoniae（RP）	MOL006990	(1S,2S,4R)-trans-2-hydroxy-1,8-cineole-B-D-glucopyranoside	Acetylcholinesterase	P22303	ACHE	0.006
Radix Paeoniae（RP）	MOL006990	(1S,2S,4R)-trans-2-hydroxy-1,8-cineole-B-D-glucopyranoside	Neuronal acetylcholine receptor subunit alpha-5	P30532	CHRNA5	0.006
Radix Paeoniae（RP）	MOL006990	(1S,2S,4R)-trans-2-hydroxy-1,8-cineole-B-D-glucopyranoside	Neuronal acetylcholine receptor subunit beta-4	P30926	CHRNB4	0.006
Radix Paeoniae（RP）	MOL006990	(1S,2S,4R)-trans-2-hydroxy-1,8-cineole-B-D-glucopyranoside	Acetylcholine receptor subunit epsilon	Q04844	CHRNE	0.006
Radix Paeoniae（RP）	MOL006990	(1S,2S,4R)-trans-2-hydroxy-1,8-cineole-B-D-glucopyranoside	Neuronal acetylcholine receptor subunit beta-3	Q05901	CHRNB3	0.006
Radix Paeoniae（RP）	MOL006990	(1S,2S,4R)-trans-2-hydroxy-1,8-cineole-B-D-glucopyranoside	Acetylcholine receptor subunit delta	Q07001	CHRND	0.006
Radix Paeoniae（RP）	MOL006990	(1S,2S,4R)-trans-2-hydroxy-1,8-cineole-B-D-glucopyranoside	Neuronal acetylcholine receptor subunit alpha-2	Q15822	CHRNA2	0.006
Radix Paeoniae（RP）	MOL006990	(1S,2S,4R)-trans-2-hydroxy-1,8-cineole-B-D-glucopyranoside	Neuronal acetylcholine receptor subunit alpha-6	Q15825	KCNJ8	0.006
Radix Paeoniae（RP）	MOL006990	(1S,2S,4R)-trans-2-hydroxy-1,8-cineole-B-D-glucopyranoside	Neuronal acetylcholine receptor subunit alpha-10	Q9GZZ6	CHRNA10	0.006
Radix Paeoniae（RP）	MOL006990	(1S,2S,4R)-trans-2-hydroxy-1,8-cineole-B-D-glucopyranoside	Neuronal acetylcholine receptor subunit alpha-9	Q9UGM1	CHRNA9	0.006
Radix Paeoniae（RP）	MOL006990	(1S,2S,4R)-trans-2-hydroxy-1,8-cineole-B-D-glucopyranoside	D1 dopamine receptor-interacting protein calcyon	Q9NYX4	CALY	0.008
Radix Paeoniae（RP）	MOL006990	(1S,2S,4R)-trans-2-hydroxy-1,8-cineole-B-D-glucopyranoside	5-hydroxytryptamine 2A receptor	P28223	HTR2A	0.009
Radix Paeoniae（RP）	MOL006990	(1S,2S,4R)-trans-2-hydroxy-1,8-cineole-B-D-glucopyranoside	cGMP-dependent 3',5'-cyclic phosphodiesterase	O00408	PDE2A	0.01
Radix Paeoniae（RP）	MOL006990	(1S,2S,4R)-trans-2-hydroxy-1,8-cineole-B-D-glucopyranoside	High affinity cAMP-specific and IBMX-insensitive 3',5'-cyclic phosphodiesterase 8A	O60658	PDE8A	0.01
Radix Paeoniae（RP）	MOL006990	(1S,2S,4R)-trans-2-hydroxy-1,8-cineole-B-D-glucopyranoside	cGMP-specific 3',5'-cyclic phosphodiesterase	O76074	PDE5A	0.01
Radix Paeoniae（RP）	MOL006990	(1S,2S,4R)-trans-2-hydroxy-1,8-cineole-B-D-glucopyranoside	High-affinity cGMP-specific 3',5'-cyclic phosphodiesterase 9A	O76083	PDE9A	0.01
Radix Paeoniae（RP）	MOL006990	(1S,2S,4R)-trans-2-hydroxy-1,8-cineole-B-D-glucopyranoside	cAMP-specific 3',5'-cyclic phosphodiesterase 4D	Q08499	PDE4D	0.01
Radix Paeoniae（RP）	MOL006990	(1S,2S,4R)-trans-2-hydroxy-1,8-cineole-B-D-glucopyranoside	cGMP-inhibited 3',5'-cyclic phosphodiesterase B	Q13370	PDE3B	0.01
Radix Paeoniae（RP）	MOL006990	(1S,2S,4R)-trans-2-hydroxy-1,8-cineole-B-D-glucopyranoside	High-affinity cAMP-specific 3',5'-cyclic phosphodiesterase 7A	Q13946	PDE7A	0.01
Radix Paeoniae（RP）	MOL006990	(1S,2S,4R)-trans-2-hydroxy-1,8-cineole-B-D-glucopyranoside	Homeobox protein engrailed-2	P19622	EN2	0.02
Radix Paeoniae（RP）	MOL006990	(1S,2S,4R)-trans-2-hydroxy-1,8-cineole-B-D-glucopyranoside	Geranylgeranyl pyrophosphate synthetase	O95749	GGPS1	0.024
Radix Paeoniae（RP）	MOL006990	(1S,2S,4R)-trans-2-hydroxy-1,8-cineole-B-D-glucopyranoside	2,4-dienoyl-CoA reductase, mitochondrial	Q16698	DECR1	0.024
Radix Paeoniae（RP）	MOL006990	(1S,2S,4R)-trans-2-hydroxy-1,8-cineole-B-D-glucopyranoside	5-hydroxytryptamine 3 receptor	P46098	HTR3A	0.025
Radix Paeoniae（RP）	MOL006990	(1S,2S,4R)-trans-2-hydroxy-1,8-cineole-B-D-glucopyranoside	5-hydroxytryptamine 4 receptor	Q13639	HTR4	0.025
Radix Paeoniae（RP）	MOL006990	(1S,2S,4R)-trans-2-hydroxy-1,8-cineole-B-D-glucopyranoside	Isopentenyl-diphosphate Delta-isomerase 1	Q13907	IDI1	0.026
Radix Paeoniae（RP）	MOL006990	(1S,2S,4R)-trans-2-hydroxy-1,8-cineole-B-D-glucopyranoside	Potassium channel subfamily K member 6	Q9Y257	KCNK6	0.029
Radix Paeoniae（RP）	MOL006990	(1S,2S,4R)-trans-2-hydroxy-1,8-cineole-B-D-glucopyranoside	D(1A) dopamine receptor	P21728	DRD1	0.03
Radix Paeoniae（RP）	MOL006990	(1S,2S,4R)-trans-2-hydroxy-1,8-cineole-B-D-glucopyranoside	D(4) dopamine receptor	P21917	DRD4	0.03
Radix Paeoniae（RP）	MOL006990	(1S,2S,4R)-trans-2-hydroxy-1,8-cineole-B-D-glucopyranoside	D(1B) dopamine receptor	P21918	DRD5	0.03
Radix Paeoniae（RP）	MOL006990	(1S,2S,4R)-trans-2-hydroxy-1,8-cineole-B-D-glucopyranoside	Neuronal acetylcholine receptor subunit alpha-3	P32297	CHRNA3	0.034
Radix Paeoniae（RP）	MOL006990	(1S,2S,4R)-trans-2-hydroxy-1,8-cineole-B-D-glucopyranoside	Nociceptin receptor	P41146	OPRL1	0.04
Radix Paeoniae（RP）	MOL006990	(1S,2S,4R)-trans-2-hydroxy-1,8-cineole-B-D-glucopyranoside	Retinoic acid receptor RXR-alpha	P19793	RXRA	0.041
Radix Paeoniae（RP）	MOL006990	(1S,2S,4R)-trans-2-hydroxy-1,8-cineole-B-D-glucopyranoside	Nuclear receptor subfamily 1 group I member 3	Q14994	NR1I3	0.041
Radix Paeoniae（RP）	MOL006990	(1S,2S,4R)-trans-2-hydroxy-1,8-cineole-B-D-glucopyranoside	Nuclear receptor coactivator 1	Q15788	NCOA1	0.041
Radix Paeoniae（RP）	MOL006990	(1S,2S,4R)-trans-2-hydroxy-1,8-cineole-B-D-glucopyranoside	Arachidonate 5-lipoxygenase	P09917	ALOX5	0.046
Radix Paeoniae（RP）	MOL006990	(1S,2S,4R)-trans-2-hydroxy-1,8-cineole-B-D-glucopyranoside	Aryl hydrocarbon receptor	P35869	AHR	0.046
Radix Paeoniae（RP）	MOL006990	(1S,2S,4R)-trans-2-hydroxy-1,8-cineole-B-D-glucopyranoside	Dihydroorotate dehydrogenase, mitochondrial	Q02127	DHODH	0.046
Radix Paeoniae（RP）	MOL006990	(1S,2S,4R)-trans-2-hydroxy-1,8-cineole-B-D-glucopyranoside	Muscarinic acetylcholine receptor M5	P08912	CHRM5	0.048
Radix Paeoniae（RP）	MOL006990	(1S,2S,4R)-trans-2-hydroxy-1,8-cineole-B-D-glucopyranoside	Histamine H1 receptor	P35367	HRH1	0.048
Radix Paeoniae（RP）	MOL006990	(1S,2S,4R)-trans-2-hydroxy-1,8-cineole-B-D-glucopyranoside	Sodium-dependent serotonin transporter	P31645	SLC6A4	0.049
Radix Paeoniae（RP）	MOL006990	(1S,2S,4R)-trans-2-hydroxy-1,8-cineole-B-D-glucopyranoside	Carbonic anhydrase 1	P00915	CA1	0.05
Radix Paeoniae（RP）	MOL006990	(1S,2S,4R)-trans-2-hydroxy-1,8-cineole-B-D-glucopyranoside	Carbonic anhydrase 2	P00918	CA2	0.05
Radix Paeoniae（RP）	MOL006990	(1S,2S,4R)-trans-2-hydroxy-1,8-cineole-B-D-glucopyranoside	Sodium/potassium-transporting ATPase gamma chain	P54710	FXYD2	0.05
Radix Paeoniae（RP）	MOL006990	(1S,2S,4R)-trans-2-hydroxy-1,8-cineole-B-D-glucopyranoside	Rhodopsin	P08100	RHO	0.051
Radix Paeoniae（RP）	MOL006990	(1S,2S,4R)-trans-2-hydroxy-1,8-cineole-B-D-glucopyranoside	Alpha-2A adrenergic receptor	P08913	ADRA2A	0.052
Radix Paeoniae（RP）	MOL006990	(1S,2S,4R)-trans-2-hydroxy-1,8-cineole-B-D-glucopyranoside	D(2) dopamine receptor	P14416	DRD2	0.052

Radix Paeoniae（RP）	MOL006990	(1S,2S,4R)-trans-2-hydroxy-1,8-cineole-B-D-glucopyranoside	Alpha-2B adrenergic receptor	P18089	ADRA2B	0.052
Radix Paeoniae（RP）	MOL006990	(1S,2S,4R)-trans-2-hydroxy-1,8-cineole-B-D-glucopyranoside	Alpha-2C adrenergic receptor	P18825	ADRA2C	0.052
Radix Paeoniae（RP）	MOL006990	(1S,2S,4R)-trans-2-hydroxy-1,8-cineole-B-D-glucopyranoside	5-hydroxytryptamine 1D receptor	P28221	HTR1D	0.052
Radix Paeoniae（RP）	MOL006990	(1S,2S,4R)-trans-2-hydroxy-1,8-cineole-B-D-glucopyranoside	5-hydroxytryptamine 1B receptor	P28222	HTR1B	0.052
Radix Paeoniae（RP）	MOL006990	(1S,2S,4R)-trans-2-hydroxy-1,8-cineole-B-D-glucopyranoside	Gamma-aminobutyric-acid receptor subunit alpha-5	P31644	GABRA5	0.052
Radix Paeoniae（RP）	MOL006990	(1S,2S,4R)-trans-2-hydroxy-1,8-cineole-B-D-glucopyranoside	D(3) dopamine receptor	P35462	DRD3	0.052
Radix Paeoniae（RP）	MOL006990	(1S,2S,4R)-trans-2-hydroxy-1,8-cineole-B-D-glucopyranoside	Gamma-aminobutyric-acid receptor subunit alpha-4	P48169	GABRA4	0.052
Radix Paeoniae（RP）	MOL006990	(1S,2S,4R)-trans-2-hydroxy-1,8-cineole-B-D-glucopyranoside	Glutamate receptor, ionotropic kainate 2	Q13002	GRIK2	0.052
Radix Paeoniae（RP）	MOL006990	(1S,2S,4R)-trans-2-hydroxy-1,8-cineole-B-D-glucopyranoside	Gamma-aminobutyric-acid receptor subunit alpha-6	Q16445	GABRA6	0.052
Radix Paeoniae（RP）	MOL006990	(1S,2S,4R)-trans-2-hydroxy-1,8-cineole-B-D-glucopyranoside	5-hydroxytryptamine 2B receptor	P41595	HTR2B	0.053
Radix Paeoniae（RP）	MOL006990	(1S,2S,4R)-trans-2-hydroxy-1,8-cineole-B-D-glucopyranoside	Farnesyl pyrophosphate synthetase	P14324	FDPS	0.064
Radix Paeoniae（RP）	MOL006990	(1S,2S,4R)-trans-2-hydroxy-1,8-cineole-B-D-glucopyranoside	Potassium channel subfamily K member 1	O00180	KCNK1	0.067
Radix Paeoniae（RP）	MOL006990	(1S,2S,4R)-trans-2-hydroxy-1,8-cineole-B-D-glucopyranoside	Sodium channel protein type 5 subunit alpha	Q14524	SCN5A	0.067
Radix Paeoniae（RP）	MOL006990	(1S,2S,4R)-trans-2-hydroxy-1,8-cineole-B-D-glucopyranoside	Annexin A5	P08758	ANXA5	0.068
Radix Paeoniae（RP）	MOL006990	(1S,2S,4R)-trans-2-hydroxy-1,8-cineole-B-D-glucopyranoside	Heparin-binding growth factor 2	P09038	FGF2	0.068
Radix Paeoniae（RP）	MOL006990	(1S,2S,4R)-trans-2-hydroxy-1,8-cineole-B-D-glucopyranoside	Guanine nucleotide-binding protein G(s) subunit alpha isoforms short	P63092	GNAS	0.07
Radix Paeoniae（RP）	MOL006990	(1S,2S,4R)-trans-2-hydroxy-1,8-cineole-B-D-glucopyranoside	Adenylate cyclase type 2	Q08462	ADCY2	0.07
Radix Paeoniae（RP）	MOL006990	(1S,2S,4R)-trans-2-hydroxy-1,8-cineole-B-D-glucopyranoside	Glucosamine-6-phosphate isomerase	P46926	GNPDA1	0.071
Radix Paeoniae（RP）	MOL006990	(1S,2S,4R)-trans-2-hydroxy-1,8-cineole-B-D-glucopyranoside	5-hydroxytryptamine 2C receptor	P28335	HTR2C	0.075
Radix Paeoniae（RP）	MOL006990	(1S,2S,4R)-trans-2-hydroxy-1,8-cineole-B-D-glucopyranoside	Liver carboxylesterase 1	P23141	CES1	0.078
Radix Paeoniae（RP）	MOL006990	(1S,2S,4R)-trans-2-hydroxy-1,8-cineole-B-D-glucopyranoside	Platelet-activating factor acetylhydrolase IB subunit gamma	Q15102	PAFAH1B3	0.078
Radix Paeoniae（RP）	MOL006990	(1S,2S,4R)-trans-2-hydroxy-1,8-cineole-B-D-glucopyranoside	Estradiol 17-beta-dehydrogenase 1	P14061	HSD17B1	0.08
Radix Paeoniae（RP）	MOL006990	(1S,2S,4R)-trans-2-hydroxy-1,8-cineole-B-D-glucopyranoside	Neuronal acetylcholine receptor subunit alpha-4	P43681	CHRNA4	0.08
Radix Paeoniae（RP）	MOL006990	(1S,2S,4R)-trans-2-hydroxy-1,8-cineole-B-D-glucopyranoside	Thymidine kinase 2, mitochondrial	O00142	TK2	0.081
Radix Paeoniae（RP）	MOL006990	(1S,2S,4R)-trans-2-hydroxy-1,8-cineole-B-D-glucopyranoside	Gamma-aminobutyric-acid receptor subunit alpha-3	P34903	GABRA3	0.087
Radix Paeoniae（RP）	MOL006990	(1S,2S,4R)-trans-2-hydroxy-1,8-cineole-B-D-glucopyranoside	3 beta-hydroxysteroid dehydrogenase/Delta 5-->4-isomerase type II	P26439	HSD3B2	0.092
Radix Paeoniae（RP）	MOL006990	(1S,2S,4R)-trans-2-hydroxy-1,8-cineole-B-D-glucopyranoside	Tripartite motif-containing protein 13	O60858	TRIM13	0.095
Radix Paeoniae（RP）	MOL006990	(1S,2S,4R)-trans-2-hydroxy-1,8-cineole-B-D-glucopyranoside	Delta-type opioid receptor	P41143	OPRD1	0.095
Radix Paeoniae（RP）	MOL006990	(1S,2S,4R)-trans-2-hydroxy-1,8-cineole-B-D-glucopyranoside	Prolactin receptor	P16471	PRLR	0.1
Radix Paeoniae（RP）	MOL006990	(1S,2S,4R)-trans-2-hydroxy-1,8-cineole-B-D-glucopyranoside	Neuronal acetylcholine receptor subunit alpha-7	P36544	CHRNA7	0.101
Radix Paeoniae（RP）	MOL006990	(1S,2S,4R)-trans-2-hydroxy-1,8-cineole-B-D-glucopyranoside	Platelet glycoprotein IX	P14770	GP9	0.105
Radix Paeoniae（RP）	MOL006990	(1S,2S,4R)-trans-2-hydroxy-1,8-cineole-B-D-glucopyranoside	Tryptophanyl-tRNA synthetase, mitochondrial	Q9UGM6	WARS2	0.105
Radix Paeoniae（RP）	MOL006990	(1S,2S,4R)-trans-2-hydroxy-1,8-cineole-B-D-glucopyranoside	Muscarinic acetylcholine receptor M2	P08172	CHRM2	0.111
Radix Paeoniae（RP）	MOL006990	(1S,2S,4R)-trans-2-hydroxy-1,8-cineole-B-D-glucopyranoside	Muscarinic acetylcholine receptor M4	P08173	CHRM4	0.111
Radix Paeoniae（RP）	MOL006990	(1S,2S,4R)-trans-2-hydroxy-1,8-cineole-B-D-glucopyranoside	Muscarinic acetylcholine receptor M3	P20309	CHRM3	0.111
Radix Paeoniae（RP）	MOL006990	(1S,2S,4R)-trans-2-hydroxy-1,8-cineole-B-D-glucopyranoside	Sodium-dependent noradrenaline transporter	P23975	SLC6A2	0.111
Radix Paeoniae（RP）	MOL006990	(1S,2S,4R)-trans-2-hydroxy-1,8-cineole-B-D-glucopyranoside	5-hydroxytryptamine 1A receptor	P08908	HTR1A	0.114
Radix Paeoniae（RP）	MOL006990	(1S,2S,4R)-trans-2-hydroxy-1,8-cineole-B-D-glucopyranoside	Gamma-aminobutyric-acid receptor subunit alpha-2	P47869	GABRA2	0.126
Radix Paeoniae（RP）	MOL006990	(1S,2S,4R)-trans-2-hydroxy-1,8-cineole-B-D-glucopyranoside	Thyroid hormone receptor, alpha isoform 1 variant	Q59FW3	SIGMAR1	0.153
Radix Paeoniae（RP）	MOL006990	(1S,2S,4R)-trans-2-hydroxy-1,8-cineole-B-D-glucopyranoside	Nuclear receptor coactivator 5	Q9HCD5	NCOA5	0.155
Radix Paeoniae（RP）	MOL006990	(1S,2S,4R)-trans-2-hydroxy-1,8-cineole-B-D-glucopyranoside	MAP kinase-activated protein kinase 2	P49137	MAPKAPK2	0.158
Radix Paeoniae（RP）	MOL006990	(1S,2S,4R)-trans-2-hydroxy-1,8-cineole-B-D-glucopyranoside	Triosephosphate isomerase	P60174	TPI1	0.158
Radix Paeoniae（RP）	MOL006990	(1S,2S,4R)-trans-2-hydroxy-1,8-cineole-B-D-glucopyranoside	Egl nine homolog 1	Q9GZT9	EGLN1	0.158
Radix Paeoniae（RP）	MOL006990	(1S,2S,4R)-trans-2-hydroxy-1,8-cineole-B-D-glucopyranoside	S-methyl-5-thioadenosine phosphorylase	Q13126	MTAP	0.159
Radix Paeoniae（RP）	MOL006990	(1S,2S,4R)-trans-2-hydroxy-1,8-cineole-B-D-glucopyranoside	Estrogen receptor beta	Q92731	ESR2	0.159
Radix Paeoniae（RP）	MOL006990	(1S,2S,4R)-trans-2-hydroxy-1,8-cineole-B-D-glucopyranoside	RAC-alpha serine/threonine-protein kinase	P31749	AKT1	0.16
Radix Paeoniae（RP）	MOL006990	(1S,2S,4R)-trans-2-hydroxy-1,8-cineole-B-D-glucopyranoside	Gamma-aminobutyric-acid receptor subunit alpha-1	P14867	GABRA1	0.161

Radix Paeoniae（RP）	MOL006990	(1S,2S,4R)-trans-2-hydroxy-1,8-cineole-B-D-glucopyranoside	cAMP-dependent protein kinase catalytic subunit alpha	P17612	PRKACA	0.162
Radix Paeoniae（RP）	MOL006990	(1S,2S,4R)-trans-2-hydroxy-1,8-cineole-B-D-glucopyranoside	Renin	P00797	REN	0.163
Radix Paeoniae（RP）	MOL006990	(1S,2S,4R)-trans-2-hydroxy-1,8-cineole-B-D-glucopyranoside	Inosine-5'-monophosphate dehydrogenase 1	P20839	IMPDH1	0.164
Radix Paeoniae（RP）	MOL006990	(1S,2S,4R)-trans-2-hydroxy-1,8-cineole-B-D-glucopyranoside	Cyclin-A2	P20248	CCNA2	0.166
Radix Paeoniae（RP）	MOL006990	(1S,2S,4R)-trans-2-hydroxy-1,8-cineole-B-D-glucopyranoside	DNA polymerase kappa	Q9UBT6	POLK	0.17
Radix Paeoniae（RP）	MOL006990	(1S,2S,4R)-trans-2-hydroxy-1,8-cineole-B-D-glucopyranoside	Hydroxyacid oxidase 1	Q9UJM8	HAO1	0.171
Radix Paeoniae（RP）	MOL006990	(1S,2S,4R)-trans-2-hydroxy-1,8-cineole-B-D-glucopyranoside	Dihydrofolate reductase	P00374	DHFR	0.174
Radix Paeoniae（RP）	MOL006990	(1S,2S,4R)-trans-2-hydroxy-1,8-cineole-B-D-glucopyranoside	Muscarinic acetylcholine receptor M1	P11229	CHRM1	0.174
Radix Paeoniae（RP）	MOL006990	(1S,2S,4R)-trans-2-hydroxy-1,8-cineole-B-D-glucopyranoside	Cathepsin D	P07339	CTSD	0.18
Radix Paeoniae（RP）	MOL006990	(1S,2S,4R)-trans-2-hydroxy-1,8-cineole-B-D-glucopyranoside	Cell division protein kinase 2	P24941	CDK2	0.183
Radix Paeoniae（RP）	MOL006990	(1S,2S,4R)-trans-2-hydroxy-1,8-cineole-B-D-glucopyranoside	3 beta-hydroxysteroid dehydrogenase/Delta 5-->4-isomerase type I	P14060	HSD3B1	0.186
Radix Paeoniae（RP）	MOL006990	(1S,2S,4R)-trans-2-hydroxy-1,8-cineole-B-D-glucopyranoside	Androgen receptor	P10275	AR	0.216
Radix Paeoniae（RP）	MOL006990	(1S,2S,4R)-trans-2-hydroxy-1,8-cineole-B-D-glucopyranoside	Glucocorticoid receptor	P04150	NR3C1	0.217
Radix Paeoniae（RP）	MOL006990	(1S,2S,4R)-trans-2-hydroxy-1,8-cineole-B-D-glucopyranoside	Mineralocorticoid receptor	P08235	NR3C2	0.217
Radix Paeoniae（RP）	MOL006990	(1S,2S,4R)-trans-2-hydroxy-1,8-cineole-B-D-glucopyranoside	Kappa-type opioid receptor	P41145	OPRK1	0.31
Radix Paeoniae（RP）	MOL006990	(1S,2S,4R)-trans-2-hydroxy-1,8-cineole-B-D-glucopyranoside	Mu-type opioid receptor	P35372	OPRM1	0.35
Radix Paeoniae（RP）	MOL006990	(1S,2S,4R)-trans-2-hydroxy-1,8-cineole-B-D-glucopyranoside	Progesterone receptor	P06401	PGR	0.449
Radix Paeoniae（RP）	MOL006990	(1S,2S,4R)-trans-2-hydroxy-1,8-cineole-B-D-glucopyranoside	Estrogen receptor	P03372	ESR1	1
Radix Paeoniae（RP）	MOL006992	(2R,3R)-4-methoxyl-distylin	Cytochrome b-c1 complex subunit 8	O14949	UQCRQ	0.014
Radix Paeoniae（RP）	MOL006992	(2R,3R)-4-methoxyl-distylin	Cytochrome b-c1 complex subunit 10	O14957	UQCR11	0.014
Radix Paeoniae（RP）	MOL006992	(2R,3R)-4-methoxyl-distylin	Cytochrome b-c1 complex subunit 6, mitochondrial	P07919	UQCRH	0.014
Radix Paeoniae（RP）	MOL006992	(2R,3R)-4-methoxyl-distylin	Cytochrome b-c1 complex subunit 7	P14927	UQCRB	0.014
Radix Paeoniae（RP）	MOL006992	(2R,3R)-4-methoxyl-distylin	Cytochrome b-c1 complex subunit 2, mitochondrial	P22695	UQCRC2	0.014
Radix Paeoniae（RP）	MOL006992	(2R,3R)-4-methoxyl-distylin	Ubiquinol-cytochrome-c reductase complex core protein 1, mitochondrial	P31930	UQCRC1	0.014
Radix Paeoniae（RP）	MOL006992	(2R,3R)-4-methoxyl-distylin	Cytochrome b-c1 complex subunit 9	Q9UDW1	UQCR10	0.014
Radix Paeoniae（RP）	MOL006992	(2R,3R)-4-methoxyl-distylin	Sodium/hydrogen exchanger 1	P19634	SLC9A1	0.016
Radix Paeoniae（RP）	MOL006992	(2R,3R)-4-methoxyl-distylin	Amiloride-sensitive amine oxidase [copper-containing]	P19801	AOC1	0.016
Radix Paeoniae（RP）	MOL006992	(2R,3R)-4-methoxyl-distylin	Amiloride-sensitive sodium channel subunit delta	P51172	SCNN1D	0.016
Radix Paeoniae（RP）	MOL006992	(2R,3R)-4-methoxyl-distylin	Amiloride-sensitive cation channel 2, neuronal	P78348	ASIC1	0.016
Radix Paeoniae（RP）	MOL006992	(2R,3R)-4-methoxyl-distylin	Amiloride-sensitive cation channel 1, neuronal	Q16515	ASIC2	0.016
Radix Paeoniae（RP）	MOL006992	(2R,3R)-4-methoxyl-distylin	Phosphatidylinositol 3-kinase regulatory subunit beta	O00459	PIK3R2	0.017
Radix Paeoniae（RP）	MOL006992	(2R,3R)-4-methoxyl-distylin	Beta-3 adrenergic receptor	P13945	ADRB3	0.017
Radix Paeoniae（RP）	MOL006992	(2R,3R)-4-methoxyl-distylin	Phosphatidylinositol 3-kinase regulatory subunit alpha	P27986	PIK3R1	0.017
Radix Paeoniae（RP）	MOL006992	(2R,3R)-4-methoxyl-distylin	Mitogen-activated protein kinase 1	P28482	MAPK1	0.017
Radix Paeoniae（RP）	MOL006992	(2R,3R)-4-methoxyl-distylin	Purine nucleoside phosphorylase	P00491	PNP	0.023
Radix Paeoniae（RP）	MOL006992	(2R,3R)-4-methoxyl-distylin	D(4) dopamine receptor	P21917	DRD4	0.023
Radix Paeoniae（RP）	MOL006992	(2R,3R)-4-methoxyl-distylin	Ribonucleoside-diphosphate reductase M2 subunit	P31350	RRM2	0.023
Radix Paeoniae（RP）	MOL006992	(2R,3R)-4-methoxyl-distylin	DNA polymerase epsilon subunit 2	P56282	POLE2	0.023
Radix Paeoniae（RP）	MOL006992	(2R,3R)-4-methoxyl-distylin	DNA polymerase epsilon catalytic subunit A	Q07864	POLE	0.023
Radix Paeoniae（RP）	MOL006992	(2R,3R)-4-methoxyl-distylin	Ribonucleoside-diphosphate reductase subunit M2 B	Q7LG56	RRM2B	0.023
Radix Paeoniae（RP）	MOL006992	(2R,3R)-4-methoxyl-distylin	DNA polymerase epsilon subunit 3	Q9NRF9	POLE3	0.023
Radix Paeoniae（RP）	MOL006992	(2R,3R)-4-methoxyl-distylin	Epidermal growth factor receptor	P00533	EGFR	0.025
Radix Paeoniae（RP）	MOL006992	(2R,3R)-4-methoxyl-distylin	Cell division protein kinase 4	P11802	CDK4	0.025
Radix Paeoniae（RP）	MOL006992	(2R,3R)-4-methoxyl-distylin	5-hydroxytryptamine 2A receptor	P28223	HTR2A	0.025
Radix Paeoniae（RP）	MOL006992	(2R,3R)-4-methoxyl-distylin	Cell division protein kinase 7	P50613	CDK7	0.025
Radix Paeoniae（RP）	MOL006992	(2R,3R)-4-methoxyl-distylin	Cell division protein kinase 9	P50750	CDK9	0.025
Radix Paeoniae（RP）	MOL006992	(2R,3R)-4-methoxyl-distylin	Cell division protein kinase 6	Q00534	CDK6	0.025
Radix Paeoniae（RP）	MOL006992	(2R,3R)-4-methoxyl-distylin	Trypsin-2	P07478	PRSS2	0.026
Radix Paeoniae（RP）	MOL006992	(2R,3R)-4-methoxyl-distylin	Toll-like receptor 7	Q9NYK1	TLR7	0.026
Radix Paeoniae（RP）	MOL006992	(2R,3R)-4-methoxyl-distylin	Mu-type opioid receptor	P35372	OPRM1	0.027

Radix Paeoniae（RP）	MOL006992	(2R,3R)-4-methoxyl-distylin	5-hydroxytryptamine 4 receptor	Q13639	HTR4	0.027
Radix Paeoniae（RP）	MOL006992	(2R,3R)-4-methoxyl-distylin	Cyclin-dependent kinase 5 activator 1	Q15078	CDK5R1	0.028
Radix Paeoniae（RP）	MOL006992	(2R,3R)-4-methoxyl-distylin	Glutamate [NMDA] receptor subunit 3A	Q8TCU5	GRIN3A	0.028
Radix Paeoniae（RP）	MOL006992	(2R,3R)-4-methoxyl-distylin	Cytochrome b	P00156	MT-CYB	0.033
Radix Paeoniae（RP）	MOL006992	(2R,3R)-4-methoxyl-distylin	Cytochrome c1, heme protein, mitochondrial	P08574	CYC1	0.033
Radix Paeoniae（RP）	MOL006992	(2R,3R)-4-methoxyl-distylin	Macrophage migration inhibitory factor	P14174	MIF	0.033
Radix Paeoniae（RP）	MOL006992	(2R,3R)-4-methoxyl-distylin	Cytochrome b-c1 complex subunit Rieske, mitochondrial	P47985	UQCRFS1	0.033
Radix Paeoniae（RP）	MOL006992	(2R,3R)-4-methoxyl-distylin	Potassium channel subfamily K member 1	O00180	KCNK1	0.035
Radix Paeoniae（RP）	MOL006992	(2R,3R)-4-methoxyl-distylin	Muscarinic acetylcholine receptor M4	P08173	CHRM4	0.035
Radix Paeoniae（RP）	MOL006992	(2R,3R)-4-methoxyl-distylin	Muscarinic acetylcholine receptor M1	P11229	CHRM1	0.035
Radix Paeoniae（RP）	MOL006992	(2R,3R)-4-methoxyl-distylin	Sucrase-isomaltase, intestinal	P14410	SI	0.035
Radix Paeoniae（RP）	MOL006992	(2R,3R)-4-methoxyl-distylin	Muscarinic acetylcholine receptor M3	P20309	CHRM3	0.035
Radix Paeoniae（RP）	MOL006992	(2R,3R)-4-methoxyl-distylin	Potassium channel subfamily K member 6	Q9Y257	KCNK6	0.035
Radix Paeoniae（RP）	MOL006992	(2R,3R)-4-methoxyl-distylin	Tyrosine 3-monooxygenase	P07101	TH	0.036
Radix Paeoniae（RP）	MOL006992	(2R,3R)-4-methoxyl-distylin	Tyrosyl-tRNA synthetase, mitochondrial	Q9Y2Z4	YARS2	0.036
Radix Paeoniae（RP）	MOL006992	(2R,3R)-4-methoxyl-distylin	Amiloride-sensitive sodium channel subunit alpha	P37088	SCNN1A	0.037
Radix Paeoniae（RP）	MOL006992	(2R,3R)-4-methoxyl-distylin	Amiloride-sensitive sodium channel subunit beta	P51168	SCNN1B	0.037
Radix Paeoniae（RP）	MOL006992	(2R,3R)-4-methoxyl-distylin	Amiloride-sensitive sodium channel subunit gamma	P51170	SCNN1G	0.037
Radix Paeoniae（RP）	MOL006992	(2R,3R)-4-methoxyl-distylin	Phenylalanine-4-hydroxylase	P00439	PAH	0.04
Radix Paeoniae（RP）	MOL006992	(2R,3R)-4-methoxyl-distylin	Sodium/potassium-transporting ATPase gamma chain	P54710	FXYD2	0.041
Radix Paeoniae（RP）	MOL006992	(2R,3R)-4-methoxyl-distylin	Solute carrier family 12 member 3	P55017	SLC12A3	0.041
Radix Paeoniae（RP）	MOL006992	(2R,3R)-4-methoxyl-distylin	Calcium-activated potassium channel subunit alpha 1	Q12791	KCNMA1	0.041
Radix Paeoniae（RP）	MOL006992	(2R,3R)-4-methoxyl-distylin	Prostaglandin reductase 1	Q14914	PTGR1	0.041
Radix Paeoniae（RP）	MOL006992	(2R,3R)-4-methoxyl-distylin	cGMP-specific 3',5'-cyclic phosphodiesterase	O76074	PDE5A	0.042
Radix Paeoniae（RP）	MOL006992	(2R,3R)-4-methoxyl-distylin	Adenosine A2a receptor	P29274	ADORA2A	0.042
Radix Paeoniae（RP）	MOL006992	(2R,3R)-4-methoxyl-distylin	Adenosine A1 receptor	P30542	ADORA1	0.042
Radix Paeoniae（RP）	MOL006992	(2R,3R)-4-methoxyl-distylin	cAMP-specific 3',5'-cyclic phosphodiesterase 4C	Q08493	PDE4C	0.042
Radix Paeoniae（RP）	MOL006992	(2R,3R)-4-methoxyl-distylin	cGMP-inhibited 3',5'-cyclic phosphodiesterase A	Q14432	PDE3A	0.042
Radix Paeoniae（RP）	MOL006992	(2R,3R)-4-methoxyl-distylin	cAMP and cAMP-inhibited cGMP 3',5'-cyclic phosphodiesterase 10A	Q9Y233	PDE10A	0.042
Radix Paeoniae（RP）	MOL006992	(2R,3R)-4-methoxyl-distylin	Serine/threonine-protein kinase 17B	O94768	STK17B	0.043
Radix Paeoniae（RP）	MOL006992	(2R,3R)-4-methoxyl-distylin	Proto-oncogene serine/threonine-protein kinase Pim-1	P11309	PIM1	0.043
Radix Paeoniae（RP）	MOL006992	(2R,3R)-4-methoxyl-distylin	ATP synthase subunit gamma, mitochondrial	P36542	ATP5F1C	0.043
Radix Paeoniae（RP）	MOL006992	(2R,3R)-4-methoxyl-distylin	UDP-glucuronosyltransferase 3A1	Q6NUS8	UGT3A1	0.043
Radix Paeoniae（RP）	MOL006992	(2R,3R)-4-methoxyl-distylin	Glycogen phosphorylase, muscle form	P11217	PYGM	0.045
Radix Paeoniae（RP）	MOL006992	(2R,3R)-4-methoxyl-distylin	DNA polymerase alpha catalytic subunit	P09884	POLA1	0.049
Radix Paeoniae（RP）	MOL006992	(2R,3R)-4-methoxyl-distylin	Ribonucleoside-diphosphate reductase large subunit	P23921	RRM1	0.049
Radix Paeoniae（RP）	MOL006992	(2R,3R)-4-methoxyl-distylin	D(3) dopamine receptor	P35462	DRD3	0.05
Radix Paeoniae（RP）	MOL006992	(2R,3R)-4-methoxyl-distylin	Inhibitor of nuclear factor kappa-B kinase subunit beta	O14920	IKBKB	0.051
Radix Paeoniae（RP）	MOL006992	(2R,3R)-4-methoxyl-distylin	Phospholipase A2	P04054	PLA2G1B	0.051
Radix Paeoniae（RP）	MOL006992	(2R,3R)-4-methoxyl-distylin	Proto-oncogene tyrosine-protein kinase LCK	P06239	LCK	0.051
Radix Paeoniae（RP）	MOL006992	(2R,3R)-4-methoxyl-distylin	Tyrosine-protein kinase Lyn	P07948	LYN	0.051
Radix Paeoniae（RP）	MOL006992	(2R,3R)-4-methoxyl-distylin	Casein kinase I isoform gamma-2	P78368	CSNK1G2	0.051
Radix Paeoniae（RP）	MOL006992	(2R,3R)-4-methoxyl-distylin	Serine/threonine-protein kinase haspin	Q8TF76	HASPIN	0.051
Radix Paeoniae（RP）	MOL006992	(2R,3R)-4-methoxyl-distylin	Group IIE secretory phospholipase A2	Q9NZK7	PLA2G2E	0.051
Radix Paeoniae（RP）	MOL006992	(2R,3R)-4-methoxyl-distylin	Cystine/glutamate transporter	Q9UPY5	SLC7A11	0.051
Radix Paeoniae（RP）	MOL006992	(2R,3R)-4-methoxyl-distylin	Ribosyldihydronicotinamide dehydrogenase [quinone]	P16083	NQO2	0.052
Radix Paeoniae（RP）	MOL006992	(2R,3R)-4-methoxyl-distylin	Ig kappa chain C region	P01834	IGKC	0.054
Radix Paeoniae（RP）	MOL006992	(2R,3R)-4-methoxyl-distylin	Ig gamma-1 chain C region	P01857	IGHG1	0.054
Radix Paeoniae（RP）	MOL006992	(2R,3R)-4-methoxyl-distylin	Keratin, type II cytoskeletal 7	P08729	KRT7	0.055
Radix Paeoniae（RP）	MOL006992	(2R,3R)-4-methoxyl-distylin	5-hydroxytryptamine 1A receptor	P08908	HTR1A	0.055
Radix Paeoniae（RP）	MOL006992	(2R,3R)-4-methoxyl-distylin	Platelet glycoprotein IX	P14770	GP9	0.055
Radix Paeoniae（RP）	MOL006992	(2R,3R)-4-methoxyl-distylin	Peptidyl-prolyl cis-trans isomerase, mitochondrial	P30405	PPIF	0.055
Radix Paeoniae（RP）	MOL006992	(2R,3R)-4-methoxyl-distylin	Sodium-dependent dopamine transporter	Q01959	SLC6A3	0.055

Radix Paeoniae（RP）	MOL006992	(2R,3R)-4-methoxyl-distylin	5-hydroxytryptamine 3 receptor	P46098	HTR3A	0.059
Radix Paeoniae（RP）	MOL006992	(2R,3R)-4-methoxyl-distylin	Alpha-1D adrenergic receptor	P25100	ADRA1D	0.062
Radix Paeoniae（RP）	MOL006992	(2R,3R)-4-methoxyl-distylin	Alpha-1B adrenergic receptor	P35368	ADRA1B	0.062
Radix Paeoniae（RP）	MOL006992	(2R,3R)-4-methoxyl-distylin	Glycogen synthase kinase-3 beta	P49841	GSK3B	0.062
Radix Paeoniae（RP）	MOL006992	(2R,3R)-4-methoxyl-distylin	Alpha-2C adrenergic receptor	P18825	ADRA2C	0.064
Radix Paeoniae（RP）	MOL006992	(2R,3R)-4-methoxyl-distylin	Sodium channel protein type 10 subunit alpha	Q9Y5Y9	SCN10A	0.064
Radix Paeoniae（RP）	MOL006992	(2R,3R)-4-methoxyl-distylin	Sterol O-acyltransferase 2	O75908	SOAT2	0.066
Radix Paeoniae（RP）	MOL006992	(2R,3R)-4-methoxyl-distylin	Prothrombin	P00734	F2	0.066
Radix Paeoniae（RP）	MOL006992	(2R,3R)-4-methoxyl-distylin	Niemann-Pick C1-like protein 1	Q9UHC9	NPC1L1	0.066
Radix Paeoniae（RP）	MOL006992	(2R,3R)-4-methoxyl-distylin	Retinoic acid receptor RXR-beta	P28702	RXRB	0.068
Radix Paeoniae（RP）	MOL006992	(2R,3R)-4-methoxyl-distylin	Oxysterols receptor LXR-beta	P55055	NR1H2	0.068
Radix Paeoniae（RP）	MOL006992	(2R,3R)-4-methoxyl-distylin	Nuclear receptor coactivator 2	Q15596	NCOA2	0.068
Radix Paeoniae（RP）	MOL006992	(2R,3R)-4-methoxyl-distylin	Sodium channel protein type 5 subunit alpha	Q14524	SCN5A	0.071
Radix Paeoniae（RP）	MOL006992	(2R,3R)-4-methoxyl-distylin	Rho-associated protein kinase 1	Q13464	ROCK1	0.073
Radix Paeoniae（RP）	MOL006992	(2R,3R)-4-methoxyl-distylin	D(1A) dopamine receptor	P21728	DRD1	0.074
Radix Paeoniae（RP）	MOL006992	(2R,3R)-4-methoxyl-distylin	D(1B) dopamine receptor	P21918	DRD5	0.074
Radix Paeoniae（RP）	MOL006992	(2R,3R)-4-methoxyl-distylin	Muscarinic acetylcholine receptor M2	P08172	CHRM2	0.075
Radix Paeoniae（RP）	MOL006992	(2R,3R)-4-methoxyl-distylin	Tyrosyl-tRNA synthetase, cytoplasmic	P54577	YARS	0.076
Radix Paeoniae（RP）	MOL006992	(2R,3R)-4-methoxyl-distylin	D(2) dopamine receptor	P14416	DRD2	0.078
Radix Paeoniae（RP）	MOL006992	(2R,3R)-4-methoxyl-distylin	Beta-1 adrenergic receptor	P08588	ADRB1	0.083
Radix Paeoniae（RP）	MOL006992	(2R,3R)-4-methoxyl-distylin	Alpha-2A adrenergic receptor	P08913	ADRA2A	0.083
Radix Paeoniae（RP）	MOL006992	(2R,3R)-4-methoxyl-distylin	DNA topoisomerase 2-alpha	P11388	TOP2A	0.083
Radix Paeoniae（RP）	MOL006992	(2R,3R)-4-methoxyl-distylin	Protein tyrosine kinase 2 beta	Q14289	PTK2B	0.083
Radix Paeoniae（RP）	MOL006992	(2R,3R)-4-methoxyl-distylin	Alpha-2B adrenergic receptor	P18089	ADRA2B	0.086
Radix Paeoniae（RP）	MOL006992	(2R,3R)-4-methoxyl-distylin	Carbonic anhydrase 1	P00915	CA1	0.087
Radix Paeoniae（RP）	MOL006992	(2R,3R)-4-methoxyl-distylin	Cell division protein kinase 2	P24941	CDK2	0.087
Radix Paeoniae（RP）	MOL006992	(2R,3R)-4-methoxyl-distylin	D-HSCDK2	O75100	CA11	0.088
Radix Paeoniae（RP）	MOL006992	(2R,3R)-4-methoxyl-distylin	Cell division control protein 2 homolog	P06493	CDK1	0.088
Radix Paeoniae（RP）	MOL006992	(2R,3R)-4-methoxyl-distylin	Estrogen-related receptor gamma	P62508	ESRRG	0.088
Radix Paeoniae（RP）	MOL006992	(2R,3R)-4-methoxyl-distylin	ATP synthase subunit beta, mitochondrial	P06576	ATP5F1B	0.09
Radix Paeoniae（RP）	MOL006992	(2R,3R)-4-methoxyl-distylin	Tyrosine-protein kinase HCK	P08631	HCK	0.09
Radix Paeoniae（RP）	MOL006992	(2R,3R)-4-methoxyl-distylin	ATP synthase subunit alpha, mitochondrial	P25705	ATP5F1A	0.09
Radix Paeoniae（RP）	MOL006992	(2R,3R)-4-methoxyl-distylin	Casein kinase I isoform gamma-1	Q9HCP0	CSNK1G1	0.103
Radix Paeoniae（RP）	MOL006992	(2R,3R)-4-methoxyl-distylin	Death-associated protein kinase 3	O43293	DAPK3	0.104
Radix Paeoniae（RP）	MOL006992	(2R,3R)-4-methoxyl-distylin	Nitric oxide synthase, inducible	P35228	NOS2	0.104
Radix Paeoniae（RP）	MOL006992	(2R,3R)-4-methoxyl-distylin	MAP kinase-activated protein kinase 2	P49137	MAPKAPK2	0.104
Radix Paeoniae（RP）	MOL006992	(2R,3R)-4-methoxyl-distylin	Beta-2 adrenergic receptor	P07550	ADRB2	0.105
Radix Paeoniae（RP）	MOL006992	(2R,3R)-4-methoxyl-distylin	Coagulation factor VII	P08709	F7	0.105
Radix Paeoniae（RP）	MOL006992	(2R,3R)-4-methoxyl-distylin	Alpha-1A adrenergic receptor	P35348	ADRA1A	0.105
Radix Paeoniae（RP）	MOL006992	(2R,3R)-4-methoxyl-distylin	Dihydrofolate reductase	P00374	DHFR	0.106
Radix Paeoniae（RP）	MOL006992	(2R,3R)-4-methoxyl-distylin	Cannabinoid receptor 1	P21554	CNR1	0.106
Radix Paeoniae（RP）	MOL006992	(2R,3R)-4-methoxyl-distylin	Peroxisome proliferator-activated receptor gamma	P37231	PPARG	0.106
Radix Paeoniae（RP）	MOL006992	(2R,3R)-4-methoxyl-distylin	Calcium/calmodulin-dependent protein kinase type II alpha chain	Q9UQM7	CAMK2A	0.106
Radix Paeoniae（RP）	MOL006992	(2R,3R)-4-methoxyl-distylin	Serine/threonine-protein kinase 6	O14965	AURKA	0.107
Radix Paeoniae（RP）	MOL006992	(2R,3R)-4-methoxyl-distylin	Cytohesin-2	Q99418	CYTH2	0.108
Radix Paeoniae（RP）	MOL006992	(2R,3R)-4-methoxyl-distylin	Casein kinase II subunit alpha	P68400	CSNK2A1	0.109
Radix Paeoniae（RP）	MOL006992	(2R,3R)-4-methoxyl-distylin	S-methyl-5-thioadenosine phosphorylase	Q13126	MTAP	0.109
Radix Paeoniae（RP）	MOL006992	(2R,3R)-4-methoxyl-distylin	Sodium-dependent serotonin transporter	P31645	SLC6A4	0.11
Radix Paeoniae（RP）	MOL006992	(2R,3R)-4-methoxyl-distylin	Cytochrome P450 19A1	P11511	CYP19A1	0.113
Radix Paeoniae（RP）	MOL006992	(2R,3R)-4-methoxyl-distylin	RAC-alpha serine/threonine-protein kinase	P31749	AKT1	0.113
Radix Paeoniae（RP）	MOL006992	(2R,3R)-4-methoxyl-distylin	Calmodulin	P62158		0.114
Radix Paeoniae（RP）	MOL006992	(2R,3R)-4-methoxyl-distylin	Mitogen-activated protein kinase 14	Q16539	MAPK14	0.115
Radix Paeoniae（RP）	MOL006992	(2R,3R)-4-methoxyl-distylin	Proto-oncogene tyrosine-protein kinase Src	P12931	SRC	0.116

Radix Paeoniae（RP）	MOL006992	(2R,3R)-4-methoxyl-distylin	Tubulin alpha-3 chain	Q71U36	TUBA1A	0.116
Radix Paeoniae（RP）	MOL006992	(2R,3R)-4-methoxyl-distylin	Cell division protein kinase 5	Q00535	CDK5	0.12
Radix Paeoniae（RP）	MOL006992	(2R,3R)-4-methoxyl-distylin	DNA polymerase kappa	Q9UBT6	POLK	0.12
Radix Paeoniae（RP）	MOL006992	(2R,3R)-4-methoxyl-distylin	Fibroblast growth factor receptor 2	P21802	FGFR2	0.121
Radix Paeoniae（RP）	MOL006992	(2R,3R)-4-methoxyl-distylin	Neuropeptide Y	P01303	NPY	0.124
Radix Paeoniae（RP）	MOL006992	(2R,3R)-4-methoxyl-distylin	Carbonic anhydrase 2	P00918	CA2	0.132
Radix Paeoniae（RP）	MOL006992	(2R,3R)-4-methoxyl-distylin	cAMP-specific 3',5'-cyclic phosphodiesterase 4B	Q07343	PDE4B	0.134
Radix Paeoniae（RP）	MOL006992	(2R,3R)-4-methoxyl-distylin	Interferon gamma	P01579	IFNG	0.135
Radix Paeoniae（RP）	MOL006992	(2R,3R)-4-methoxyl-distylin	Sterol O-acyltransferase 1	P35610	SOAT1	0.136
Radix Paeoniae（RP）	MOL006992	(2R,3R)-4-methoxyl-distylin	Oxysterols receptor LXR-alpha	Q13133	NR1H3	0.136
Radix Paeoniae（RP）	MOL006992	(2R,3R)-4-methoxyl-distylin	Phosphatidylinositol-4,5-bisphosphate 3-kinase catalytic subunit gamma isoform	P48736	PIK3CG	0.138
Radix Paeoniae（RP）	MOL006992	(2R,3R)-4-methoxyl-distylin	cAMP-dependent protein kinase catalytic subunit alpha	P17612	PRKACA	0.151
Radix Paeoniae（RP）	MOL006992	(2R,3R)-4-methoxyl-distylin	Inhibitor of nuclear factor kappa-B kinase subunit alpha	O15111	CHUK	0.16
Radix Paeoniae（RP）	MOL006992	(2R,3R)-4-methoxyl-distylin	Arachidonate 5-lipoxygenase	P09917	ALOX5	0.16
Radix Paeoniae（RP）	MOL006992	(2R,3R)-4-methoxyl-distylin	cAMP-specific 3',5'-cyclic phosphodiesterase 4A	P27815	PDE4A	0.18
Radix Paeoniae（RP）	MOL006992	(2R,3R)-4-methoxyl-distylin	C-jun-amino-terminal kinase-interacting protein 1	Q9UQF2	MAPK8IP1	0.232
Radix Paeoniae（RP）	MOL006992	(2R,3R)-4-methoxyl-distylin	Hemoglobin subunit alpha	P69905	HBA1	0.257
Radix Paeoniae（RP）	MOL006992	(2R,3R)-4-methoxyl-distylin	Cyclin-A2	P20248	CCNA2	0.34
Radix Paeoniae（RP）	MOL006992	(2R,3R)-4-methoxyl-distylin	Trypsin-1	P07477	PRSS1	0.357
Radix Paeoniae（RP）	MOL006992	(2R,3R)-4-methoxyl-distylin	Prostaglandin G/H synthase 1	P23219	PTGS1	0.382
Radix Paeoniae（RP）	MOL006992	(2R,3R)-4-methoxyl-distylin	Nuclear receptor coactivator 1	Q15788	NCOA1	0.4
Radix Paeoniae（RP）	MOL006992	(2R,3R)-4-methoxyl-distylin	Prostaglandin G/H synthase 2	P35354	PTGS2	0.655
Radix Paeoniae（RP）	MOL006992	(2R,3R)-4-methoxyl-distylin	Estrogen receptor beta	Q92731	ESR2	0.788
Radix Paeoniae（RP）	MOL006992	(2R,3R)-4-methoxyl-distylin	Estrogen receptor	P03372	ESR1	1
Radix Paeoniae（RP）	MOL006994	1-o-beta-d-glucopyranosyl-8-o-benzoylpaeonisuffrone_qt	Cytochrome b-c1 complex subunit 8	O14949	UQCRQ	0.018
Radix Paeoniae（RP）	MOL006994	1-o-beta-d-glucopyranosyl-8-o-benzoylpaeonisuffrone_qt	Cytochrome b-c1 complex subunit 10	O14957	UQCR11	0.018
Radix Paeoniae（RP）	MOL006994	1-o-beta-d-glucopyranosyl-8-o-benzoylpaeonisuffrone_qt	Cytochrome b-c1 complex subunit 6, mitochondrial	P07919	UQCRH	0.018
Radix Paeoniae（RP）	MOL006994	1-o-beta-d-glucopyranosyl-8-o-benzoylpaeonisuffrone_qt	Cytochrome b-c1 complex subunit 2, mitochondrial	P22695	UQCRC2	0.018
Radix Paeoniae（RP）	MOL006994	1-o-beta-d-glucopyranosyl-8-o-benzoylpaeonisuffrone_qt	Ubiquinol-cytochrome-c reductase complex core protein 1, mitochondrial	P31930	UQCRC1	0.018
Radix Paeoniae（RP）	MOL006994	1-o-beta-d-glucopyranosyl-8-o-benzoylpaeonisuffrone_qt	Cytochrome b-c1 complex subunit 9	Q9UDW1	UQCR10	0.018
Radix Paeoniae（RP）	MOL006994	1-o-beta-d-glucopyranosyl-8-o-benzoylpaeonisuffrone_qt	Acetylcholinesterase	P22303	ACHE	0.019
Radix Paeoniae（RP）	MOL006994	1-o-beta-d-glucopyranosyl-8-o-benzoylpaeonisuffrone_qt	Glycine receptor subunit alpha-1	P23415	GLRA1	0.019
Radix Paeoniae（RP）	MOL006994	1-o-beta-d-glucopyranosyl-8-o-benzoylpaeonisuffrone_qt	ATP synthase delta chain, mitochondrial	P30049	ATP5F1D	0.019
Radix Paeoniae（RP）	MOL006994	1-o-beta-d-glucopyranosyl-8-o-benzoylpaeonisuffrone_qt	Neuronal acetylcholine receptor subunit alpha-3	P32297	CHRNA3	0.019
Radix Paeoniae（RP）	MOL006994	1-o-beta-d-glucopyranosyl-8-o-benzoylpaeonisuffrone_qt	Neuronal acetylcholine receptor subunit alpha-7	P36544	CHRNA7	0.019
Radix Paeoniae（RP）	MOL006994	1-o-beta-d-glucopyranosyl-8-o-benzoylpaeonisuffrone_qt	Glutamate receptor 1	P42261	GRIA1	0.019
Radix Paeoniae（RP）	MOL006994	1-o-beta-d-glucopyranosyl-8-o-benzoylpaeonisuffrone_qt	Calcium-transporting ATPase type 2C member 1	P98194	ATP2C1	0.019
Radix Paeoniae（RP）	MOL006994	1-o-beta-d-glucopyranosyl-8-o-benzoylpaeonisuffrone_qt	Potassium voltage-gated channel subfamily A member 1	Q09470	KCNA1	0.019
Radix Paeoniae（RP）	MOL006994	1-o-beta-d-glucopyranosyl-8-o-benzoylpaeonisuffrone_qt	Glutamate [NMDA] receptor subunit epsilon-1	Q12879	GRIN2A	0.02
Radix Paeoniae（RP）	MOL006994	1-o-beta-d-glucopyranosyl-8-o-benzoylpaeonisuffrone_qt	Glutamate [NMDA] receptor subunit epsilon-2	Q13224	GRIN2B	0.02
Radix Paeoniae（RP）	MOL006994	1-o-beta-d-glucopyranosyl-8-o-benzoylpaeonisuffrone_qt	5-hydroxytryptamine 4 receptor	Q13639	HTR4	0.02
Radix Paeoniae（RP）	MOL006994	1-o-beta-d-glucopyranosyl-8-o-benzoylpaeonisuffrone_qt	Glutamate [NMDA] receptor subunit 3A	Q8TCU5	GRIN3A	0.02
Radix Paeoniae（RP）	MOL006994	1-o-beta-d-glucopyranosyl-8-o-benzoylpaeonisuffrone_qt	Gamma-aminobutyric-acid receptor subunit beta-3	P28472	GABRB3	0.021
Radix Paeoniae（RP）	MOL006994	1-o-beta-d-glucopyranosyl-8-o-benzoylpaeonisuffrone_qt	Sodium channel protein type 4 subunit alpha	P35499	SCN4A	0.021
Radix Paeoniae（RP）	MOL006994	1-o-beta-d-glucopyranosyl-8-o-benzoylpaeonisuffrone_qt	Gamma-aminobutyric-acid receptor subunit beta-2	P47870	GABRB2	0.021
Radix Paeoniae（RP）	MOL006994	1-o-beta-d-glucopyranosyl-8-o-benzoylpaeonisuffrone_qt	Ig kappa chain C region	P01834	IGKC	0.022
Radix Paeoniae（RP）	MOL006994	1-o-beta-d-glucopyranosyl-8-o-benzoylpaeonisuffrone_qt	Ig gamma-1 chain C region	P01857	IGHG1	0.022
Radix Paeoniae（RP）	MOL006994	1-o-beta-d-glucopyranosyl-8-o-benzoylpaeonisuffrone_qt	Ig gamma-2 chain C region	P01859	IGHG2	0.022
Radix Paeoniae（RP）	MOL006994	1-o-beta-d-glucopyranosyl-8-o-benzoylpaeonisuffrone_qt	Interleukin-3	P08700	IL3	0.022

Radix Paeoniae（RP）	MOL006994	1-o-beta-d-glucopyranosyl-8-o-benzoylpaeonisuffrone_qt	Amiloride-sensitive sodium channel subunit alpha	P37088	SCNN1A	0.022
Radix Paeoniae（RP）	MOL006994	1-o-beta-d-glucopyranosyl-8-o-benzoylpaeonisuffrone_qt	Amiloride-sensitive sodium channel subunit beta	P51168	SCNN1B	0.022
Radix Paeoniae（RP）	MOL006994	1-o-beta-d-glucopyranosyl-8-o-benzoylpaeonisuffrone_qt	Amiloride-sensitive sodium channel subunit gamma	P51170	SCNN1G	0.022
Radix Paeoniae（RP）	MOL006994	1-o-beta-d-glucopyranosyl-8-o-benzoylpaeonisuffrone_qt	Protein S100-A12	P80511	S100A12	0.022
Radix Paeoniae（RP）	MOL006994	1-o-beta-d-glucopyranosyl-8-o-benzoylpaeonisuffrone_qt	Protein S100-A13	Q99584	S100A13	0.022
Radix Paeoniae（RP）	MOL006994	1-o-beta-d-glucopyranosyl-8-o-benzoylpaeonisuffrone_qt	Tyrosine-protein kinase JAK2	O60674	JAK2	0.023
Radix Paeoniae（RP）	MOL006994	1-o-beta-d-glucopyranosyl-8-o-benzoylpaeonisuffrone_qt	Muscarinic acetylcholine receptor M5	P08912	CHRM5	0.023
Radix Paeoniae（RP）	MOL006994	1-o-beta-d-glucopyranosyl-8-o-benzoylpaeonisuffrone_qt	Sucrase-isomaltase, intestinal	P14410	SI	0.023
Radix Paeoniae（RP）	MOL006994	1-o-beta-d-glucopyranosyl-8-o-benzoylpaeonisuffrone_qt	Tyrosine-protein kinase JAK1	P23458	JAK1	0.023
Radix Paeoniae（RP）	MOL006994	1-o-beta-d-glucopyranosyl-8-o-benzoylpaeonisuffrone_qt	5-hydroxytryptamine 1D receptor	P28221	HTR1D	0.023
Radix Paeoniae（RP）	MOL006994	1-o-beta-d-glucopyranosyl-8-o-benzoylpaeonisuffrone_qt	5-hydroxytryptamine 1B receptor	P28222	HTR1B	0.023
Radix Paeoniae（RP）	MOL006994	1-o-beta-d-glucopyranosyl-8-o-benzoylpaeonisuffrone_qt	Adenosine A2a receptor	P29274	ADORA2A	0.023
Radix Paeoniae（RP）	MOL006994	1-o-beta-d-glucopyranosyl-8-o-benzoylpaeonisuffrone_qt	Adenosine A1 receptor	P30542	ADORA1	0.023
Radix Paeoniae（RP）	MOL006994	1-o-beta-d-glucopyranosyl-8-o-benzoylpaeonisuffrone_qt	Histamine H1 receptor	P35367	HRH1	0.023
Radix Paeoniae（RP）	MOL006994	1-o-beta-d-glucopyranosyl-8-o-benzoylpaeonisuffrone_qt	5-hydroxytryptamine 2B receptor	P41595	HTR2B	0.023
Radix Paeoniae（RP）	MOL006994	1-o-beta-d-glucopyranosyl-8-o-benzoylpaeonisuffrone_qt	Tyrosine-protein kinase JAK3	P52333	JAK3	0.023
Radix Paeoniae（RP）	MOL006994	1-o-beta-d-glucopyranosyl-8-o-benzoylpaeonisuffrone_qt	cAMP-specific 3',5'-cyclic phosphodiesterase 4C	Q08493	PDE4C	0.023
Radix Paeoniae（RP）	MOL006994	1-o-beta-d-glucopyranosyl-8-o-benzoylpaeonisuffrone_qt	ATP synthase subunit beta, mitochondrial	P06576	ATP5F1B	0.024
Radix Paeoniae（RP）	MOL006994	1-o-beta-d-glucopyranosyl-8-o-benzoylpaeonisuffrone_qt	Stromelysin-1	P08254	MMP3	0.024
Radix Paeoniae（RP）	MOL006994	1-o-beta-d-glucopyranosyl-8-o-benzoylpaeonisuffrone_qt	Stromelysin-2	P09238	MMP10	0.024
Radix Paeoniae（RP）	MOL006994	1-o-beta-d-glucopyranosyl-8-o-benzoylpaeonisuffrone_qt	Arachidonate 5-lipoxygenase	P09917	ALOX5	0.024
Radix Paeoniae（RP）	MOL006994	1-o-beta-d-glucopyranosyl-8-o-benzoylpaeonisuffrone_qt	ATP synthase subunit alpha, mitochondrial	P25705	ATP5F1A	0.024
Radix Paeoniae（RP）	MOL006994	1-o-beta-d-glucopyranosyl-8-o-benzoylpaeonisuffrone_qt	5-hydroxytryptamine 2A receptor	P28223	HTR2A	0.024
Radix Paeoniae（RP）	MOL006994	1-o-beta-d-glucopyranosyl-8-o-benzoylpaeonisuffrone_qt	Alpha-1A adrenergic receptor	P35348	ADRA1A	0.024
Radix Paeoniae（RP）	MOL006994	1-o-beta-d-glucopyranosyl-8-o-benzoylpaeonisuffrone_qt	Aryl hydrocarbon receptor	P35869	AHR	0.024
Radix Paeoniae（RP）	MOL006994	1-o-beta-d-glucopyranosyl-8-o-benzoylpaeonisuffrone_qt	Macrophage metalloelastase	P39900	MMP12	0.024
Radix Paeoniae（RP）	MOL006994	1-o-beta-d-glucopyranosyl-8-o-benzoylpaeonisuffrone_qt	Dihydroorotate dehydrogenase, mitochondrial	Q02127	DHODH	0.024
Radix Paeoniae（RP）	MOL006994	1-o-beta-d-glucopyranosyl-8-o-benzoylpaeonisuffrone_qt	Solute carrier family 22 member 6	Q4U2R8	SLC22A6	0.024
Radix Paeoniae（RP）	MOL006994	1-o-beta-d-glucopyranosyl-8-o-benzoylpaeonisuffrone_qt	Solute carrier family 22 member 8	Q8TCC7	SLC22A8	0.024
Radix Paeoniae（RP）	MOL006994	1-o-beta-d-glucopyranosyl-8-o-benzoylpaeonisuffrone_qt	Solute carrier family 22 member 11	Q9NSA0	SLC22A11	0.024
Radix Paeoniae（RP）	MOL006994	1-o-beta-d-glucopyranosyl-8-o-benzoylpaeonisuffrone_qt	Cyclin-dependent kinase 5 activator 1	Q15078	CDK5R1	0.027
Radix Paeoniae（RP）	MOL006994	1-o-beta-d-glucopyranosyl-8-o-benzoylpaeonisuffrone_qt	Tripartite motif-containing protein 13	O60858	TRIM13	0.028
Radix Paeoniae（RP）	MOL006994	1-o-beta-d-glucopyranosyl-8-o-benzoylpaeonisuffrone_qt	Potassium channel subfamily K member 6	Q9Y257	KCNK6	0.028
Radix Paeoniae（RP）	MOL006994	1-o-beta-d-glucopyranosyl-8-o-benzoylpaeonisuffrone_qt	Cytochrome b	P00156	MT-CYB	0.031
Radix Paeoniae（RP）	MOL006994	1-o-beta-d-glucopyranosyl-8-o-benzoylpaeonisuffrone_qt	Cytochrome c1, heme protein, mitochondrial	P08574	CYC1	0.031
Radix Paeoniae（RP）	MOL006994	1-o-beta-d-glucopyranosyl-8-o-benzoylpaeonisuffrone_qt	Retinoic acid receptor beta	P10826	RARB	0.031
Radix Paeoniae（RP）	MOL006994	1-o-beta-d-glucopyranosyl-8-o-benzoylpaeonisuffrone_qt	Retinoic acid receptor gamma-1	P13631	RARG	0.031
Radix Paeoniae（RP）	MOL006994	1-o-beta-d-glucopyranosyl-8-o-benzoylpaeonisuffrone_qt	Cytochrome b-c1 complex subunit Rieske, mitochondrial	P47985	UQCRFS1	0.031
Radix Paeoniae（RP）	MOL006994	1-o-beta-d-glucopyranosyl-8-o-benzoylpaeonisuffrone_qt	Retinoic acid receptor RXR-gamma	P48443	RXRG	0.031
Radix Paeoniae（RP）	MOL006994	1-o-beta-d-glucopyranosyl-8-o-benzoylpaeonisuffrone_qt	3-hydroxy-3-methylglutaryl-coenzyme A reductase	P04035	HMGCR	0.036
Radix Paeoniae（RP）	MOL006994	1-o-beta-d-glucopyranosyl-8-o-benzoylpaeonisuffrone_qt	Annexin A1	P04083	ANXA1	0.036
Radix Paeoniae（RP）	MOL006994	1-o-beta-d-glucopyranosyl-8-o-benzoylpaeonisuffrone_qt	Integrin alpha-L	P20701	ITGAL	0.036
Radix Paeoniae（RP）	MOL006994	1-o-beta-d-glucopyranosyl-8-o-benzoylpaeonisuffrone_qt	Nuclear receptor 0B1	P51843	NR0B1	0.036
Radix Paeoniae（RP）	MOL006994	1-o-beta-d-glucopyranosyl-8-o-benzoylpaeonisuffrone_qt	Calcium/calmodulin-dependent protein kinase type II subunit delta	Q13557	CAMK2D	0.037
Radix Paeoniae（RP）	MOL006994	1-o-beta-d-glucopyranosyl-8-o-benzoylpaeonisuffrone_qt	STE20-like serine/threonine-protein kinase	Q9H2G2	SLK	0.037
Radix Paeoniae（RP）	MOL006994	1-o-beta-d-glucopyranosyl-8-o-benzoylpaeonisuffrone_qt	Peroxisome proliferator-activated receptor gamma	P37231	PPARG	0.038
Radix Paeoniae（RP）	MOL006994	1-o-beta-d-glucopyranosyl-8-o-benzoylpaeonisuffrone_qt	Serine/threonine-protein phosphatase 2A catalytic subunit alpha isoform	P67775	PPP2CA	0.038

Radix Paeoniae（RP）	MOL006994	1-o-beta-d-glucopyranosyl-8-o-benzoylpaeonisuffrone_qt	Serine/threonine-protein phosphatase 2A 56 kDa regulatory subunit gamma isoform	Q13362	PPP2R5C	0.038
Radix Paeoniae（RP）	MOL006994	1-o-beta-d-glucopyranosyl-8-o-benzoylpaeonisuffrone_qt	Oxysterols receptor LXR-beta	P55055	NR1H2	0.04
Radix Paeoniae（RP）	MOL006994	1-o-beta-d-glucopyranosyl-8-o-benzoylpaeonisuffrone_qt	Sterol O-acyltransferase 2	O75908	SOAT2	0.043
Radix Paeoniae（RP）	MOL006994	1-o-beta-d-glucopyranosyl-8-o-benzoylpaeonisuffrone_qt	Sterol O-acyltransferase 1	P35610	SOAT1	0.043
Radix Paeoniae（RP）	MOL006994	1-o-beta-d-glucopyranosyl-8-o-benzoylpaeonisuffrone_qt	5-hydroxytryptamine 3 receptor	P46098	HTR3A	0.047
Radix Paeoniae（RP）	MOL006994	1-o-beta-d-glucopyranosyl-8-o-benzoylpaeonisuffrone_qt	Platelet glycoprotein IX	P14770	GP9	0.049
Radix Paeoniae（RP）	MOL006994	1-o-beta-d-glucopyranosyl-8-o-benzoylpaeonisuffrone_qt	5-hydroxytryptamine 1A receptor	P08908	HTR1A	0.05
Radix Paeoniae（RP）	MOL006994	1-o-beta-d-glucopyranosyl-8-o-benzoylpaeonisuffrone_qt	Gamma-aminobutyric-acid receptor subunit alpha-5	P31644	GABRA5	0.052
Radix Paeoniae（RP）	MOL006994	1-o-beta-d-glucopyranosyl-8-o-benzoylpaeonisuffrone_qt	Gamma-aminobutyric-acid receptor subunit alpha-3	P34903	GABRA3	0.052
Radix Paeoniae（RP）	MOL006994	1-o-beta-d-glucopyranosyl-8-o-benzoylpaeonisuffrone_qt	DNA topoisomerase 2-alpha	P11388	TOP2A	0.053
Radix Paeoniae（RP）	MOL006994	1-o-beta-d-glucopyranosyl-8-o-benzoylpaeonisuffrone_qt	cAMP-specific 3',5'-cyclic phosphodiesterase 4A	P27815	PDE4A	0.053
Radix Paeoniae（RP）	MOL006994	1-o-beta-d-glucopyranosyl-8-o-benzoylpaeonisuffrone_qt	cAMP-specific 3',5'-cyclic phosphodiesterase 4B	Q07343	PDE4B	0.053
Radix Paeoniae（RP）	MOL006994	1-o-beta-d-glucopyranosyl-8-o-benzoylpaeonisuffrone_qt	Protein tyrosine kinase 2 beta	Q14289	PTK2B	0.053
Radix Paeoniae（RP）	MOL006994	1-o-beta-d-glucopyranosyl-8-o-benzoylpaeonisuffrone_qt	cGMP-specific 3',5'-cyclic phosphodiesterase	O76074	PDE5A	0.054
Radix Paeoniae（RP）	MOL006994	1-o-beta-d-glucopyranosyl-8-o-benzoylpaeonisuffrone_qt	Ribosyldihydronicotinamide dehydrogenase [quinone]	P16083	NQO2	0.057
Radix Paeoniae（RP）	MOL006994	1-o-beta-d-glucopyranosyl-8-o-benzoylpaeonisuffrone_qt	Potassium channel subfamily K member 1	O00180	KCNK1	0.06
Radix Paeoniae（RP）	MOL006994	1-o-beta-d-glucopyranosyl-8-o-benzoylpaeonisuffrone_qt	Troponin C, slow skeletal and cardiac muscles	P63316	TNNC1	0.06
Radix Paeoniae（RP）	MOL006994	1-o-beta-d-glucopyranosyl-8-o-benzoylpaeonisuffrone_qt	D-HSCDK2	O75100	CA11	0.061
Radix Paeoniae（RP）	MOL006994	1-o-beta-d-glucopyranosyl-8-o-benzoylpaeonisuffrone_qt	Glycogen synthase kinase-3 beta	P49841	GSK3B	0.061
Radix Paeoniae（RP）	MOL006994	1-o-beta-d-glucopyranosyl-8-o-benzoylpaeonisuffrone_qt	Aldo-keto reductase family 1 member C3	P42330	AKR1C3	0.064
Radix Paeoniae（RP）	MOL006994	1-o-beta-d-glucopyranosyl-8-o-benzoylpaeonisuffrone_qt	Retinoic acid receptor alpha	P10276	RARA	0.068
Radix Paeoniae（RP）	MOL006994	1-o-beta-d-glucopyranosyl-8-o-benzoylpaeonisuffrone_qt	Retinoic acid receptor RXR-beta	P28702	RXRB	0.078
Radix Paeoniae（RP）	MOL006994	1-o-beta-d-glucopyranosyl-8-o-benzoylpaeonisuffrone_qt	Tubulin alpha-3 chain	Q71U36	TUBA1A	0.08
Radix Paeoniae（RP）	MOL006994	1-o-beta-d-glucopyranosyl-8-o-benzoylpaeonisuffrone_qt	Geranylgeranyl pyrophosphate synthetase	O95749	GGPS1	0.081
Radix Paeoniae（RP）	MOL006994	1-o-beta-d-glucopyranosyl-8-o-benzoylpaeonisuffrone_qt	Epidermal growth factor receptor	P00533	EGFR	0.082
Radix Paeoniae（RP）	MOL006994	1-o-beta-d-glucopyranosyl-8-o-benzoylpaeonisuffrone_qt	Androgen receptor	P10275	AR	0.082
Radix Paeoniae（RP）	MOL006994	1-o-beta-d-glucopyranosyl-8-o-benzoylpaeonisuffrone_qt	Carbonic anhydrase 2	P00918	CA2	0.083
Radix Paeoniae（RP）	MOL006994	1-o-beta-d-glucopyranosyl-8-o-benzoylpaeonisuffrone_qt	Glucocorticoid receptor	P04150	NR3C1	0.083
Radix Paeoniae（RP）	MOL006994	1-o-beta-d-glucopyranosyl-8-o-benzoylpaeonisuffrone_qt	Endothelin-1 receptor	P25101	EDNRA	0.083
Radix Paeoniae（RP）	MOL006994	1-o-beta-d-glucopyranosyl-8-o-benzoylpaeonisuffrone_qt	Solute carrier family 12 member 1	Q13621	SLC12A1	0.083
Radix Paeoniae（RP）	MOL006994	1-o-beta-d-glucopyranosyl-8-o-benzoylpaeonisuffrone_qt	Nuclear receptor coactivator 2	Q15596	NCOA2	0.084
Radix Paeoniae（RP）	MOL006994	1-o-beta-d-glucopyranosyl-8-o-benzoylpaeonisuffrone_qt	Glycogen phosphorylase, muscle form	P11217	PYGM	0.085
Radix Paeoniae（RP）	MOL006994	1-o-beta-d-glucopyranosyl-8-o-benzoylpaeonisuffrone_qt	Serine/threonine-protein kinase 6	O14965	AURKA	0.086
Radix Paeoniae（RP）	MOL006994	1-o-beta-d-glucopyranosyl-8-o-benzoylpaeonisuffrone_qt	Beta-2 adrenergic receptor	P07550	ADRB2	0.086
Radix Paeoniae（RP）	MOL006994	1-o-beta-d-glucopyranosyl-8-o-benzoylpaeonisuffrone_qt	Beta-1 adrenergic receptor	P08588	ADRB1	0.086
Radix Paeoniae（RP）	MOL006994	1-o-beta-d-glucopyranosyl-8-o-benzoylpaeonisuffrone_qt	Sodium-dependent dopamine transporter	Q01959	SLC6A3	0.086
Radix Paeoniae（RP）	MOL006994	1-o-beta-d-glucopyranosyl-8-o-benzoylpaeonisuffrone_qt	Oxysterols receptor LXR-alpha	Q13133	NR1H3	0.086
Radix Paeoniae（RP）	MOL006994	1-o-beta-d-glucopyranosyl-8-o-benzoylpaeonisuffrone_qt	Potassium voltage-gated channel subfamily KQT member 1	P51787	KCNQ1	0.087
Radix Paeoniae（RP）	MOL006994	1-o-beta-d-glucopyranosyl-8-o-benzoylpaeonisuffrone_qt	ATP-sensitive inward rectifier potassium channel 1	P48048	KCNJ1	0.088
Radix Paeoniae（RP）	MOL006994	1-o-beta-d-glucopyranosyl-8-o-benzoylpaeonisuffrone_qt	Proto-oncogene tyrosine-protein kinase Src	P12931	SRC	0.091
Radix Paeoniae（RP）	MOL006994	1-o-beta-d-glucopyranosyl-8-o-benzoylpaeonisuffrone_qt	Mineralocorticoid receptor	P08235	NR3C2	0.093
Radix Paeoniae（RP）	MOL006994	1-o-beta-d-glucopyranosyl-8-o-benzoylpaeonisuffrone_qt	Fibroblast growth factor receptor 2	P21802	FGFR2	0.093
Radix Paeoniae（RP）	MOL006994	1-o-beta-d-glucopyranosyl-8-o-benzoylpaeonisuffrone_qt	RAC-alpha serine/threonine-protein kinase	P31749	AKT1	0.093
Radix Paeoniae（RP）	MOL006994	1-o-beta-d-glucopyranosyl-8-o-benzoylpaeonisuffrone_qt	ATP-binding cassette transporter sub-family C member 8	Q09428	ABCC8	0.093
Radix Paeoniae（RP）	MOL006994	1-o-beta-d-glucopyranosyl-8-o-benzoylpaeonisuffrone_qt	C-jun-amino-terminal kinase-interacting protein 1	Q9UQF2	MAPK8IP1	0.093
Radix Paeoniae（RP）	MOL006994	1-o-beta-d-glucopyranosyl-8-o-benzoylpaeonisuffrone_qt	Cell division protein kinase 2	P24941	CDK2	0.095
Radix Paeoniae（RP）	MOL006994	1-o-beta-d-glucopyranosyl-8-o-benzoylpaeonisuffrone_qt	Peroxisome proliferator-activated receptor alpha	Q07869	PPARA	0.097

Radix Paeoniae（RP）	MOL006994	1-o-beta-d-glucopyranosyl-8-o-benzoylpaeonisuffrone_qt	Tyrosine-protein phosphatase non-receptor type 1	P18031	PTPN1	0.1
Radix Paeoniae（RP）	MOL006994	1-o-beta-d-glucopyranosyl-8-o-benzoylpaeonisuffrone_qt	Mitogen-activated protein kinase 14	Q16539	MAPK14	0.1
Radix Paeoniae（RP）	MOL006994	1-o-beta-d-glucopyranosyl-8-o-benzoylpaeonisuffrone_qt	Liver carboxylesterase 1	P23141	CES1	0.102
Radix Paeoniae（RP）	MOL006994	1-o-beta-d-glucopyranosyl-8-o-benzoylpaeonisuffrone_qt	Muscarinic acetylcholine receptor M3	P20309	CHRM3	0.114
Radix Paeoniae（RP）	MOL006994	1-o-beta-d-glucopyranosyl-8-o-benzoylpaeonisuffrone_qt	Gamma-aminobutyric-acid receptor subunit alpha-2	P47869	GABRA2	0.114
Radix Paeoniae（RP）	MOL006994	1-o-beta-d-glucopyranosyl-8-o-benzoylpaeonisuffrone_qt	Casein kinase II subunit alpha	P68400	CSNK2A1	0.115
Radix Paeoniae（RP）	MOL006994	1-o-beta-d-glucopyranosyl-8-o-benzoylpaeonisuffrone_qt	Sodium-dependent serotonin transporter	P31645	SLC6A4	0.117
Radix Paeoniae（RP）	MOL006994	1-o-beta-d-glucopyranosyl-8-o-benzoylpaeonisuffrone_qt	Calmodulin	P62158		0.126
Radix Paeoniae（RP）	MOL006994	1-o-beta-d-glucopyranosyl-8-o-benzoylpaeonisuffrone_qt	Cell division control protein 2 homolog	P06493	CDK1	0.129
Radix Paeoniae（RP）	MOL006994	1-o-beta-d-glucopyranosyl-8-o-benzoylpaeonisuffrone_qt	Cell division protein kinase 5	Q00535	CDK5	0.129
Radix Paeoniae（RP）	MOL006994	1-o-beta-d-glucopyranosyl-8-o-benzoylpaeonisuffrone_qt	Sodium channel protein type 5 subunit alpha	Q14524	SCN5A	0.13
Radix Paeoniae（RP）	MOL006994	1-o-beta-d-glucopyranosyl-8-o-benzoylpaeonisuffrone_qt	Delta-type opioid receptor	P41143	OPRD1	0.133
Radix Paeoniae（RP）	MOL006994	1-o-beta-d-glucopyranosyl-8-o-benzoylpaeonisuffrone_qt	Gamma-aminobutyric-acid receptor subunit alpha-1	P14867	GABRA1	0.14
Radix Paeoniae（RP）	MOL006994	1-o-beta-d-glucopyranosyl-8-o-benzoylpaeonisuffrone_qt	Muscarinic acetylcholine receptor M2	P08172	CHRM2	0.145
Radix Paeoniae（RP）	MOL006994	1-o-beta-d-glucopyranosyl-8-o-benzoylpaeonisuffrone_qt	Muscarinic acetylcholine receptor M4	P08173	CHRM4	0.145
Radix Paeoniae（RP）	MOL006994	1-o-beta-d-glucopyranosyl-8-o-benzoylpaeonisuffrone_qt	Sodium-dependent noradrenaline transporter	P23975	SLC6A2	0.148
Radix Paeoniae（RP）	MOL006994	1-o-beta-d-glucopyranosyl-8-o-benzoylpaeonisuffrone_qt	Progesterone receptor	P06401	PGR	0.154
Radix Paeoniae（RP）	MOL006994	1-o-beta-d-glucopyranosyl-8-o-benzoylpaeonisuffrone_qt	Trypsin-1	P07477	PRSS1	0.176
Radix Paeoniae（RP）	MOL006994	1-o-beta-d-glucopyranosyl-8-o-benzoylpaeonisuffrone_qt	Hemoglobin subunit alpha	P69905	HBA1	0.199
Radix Paeoniae（RP）	MOL006994	1-o-beta-d-glucopyranosyl-8-o-benzoylpaeonisuffrone_qt	Kappa-type opioid receptor	P41145	OPRK1	0.203
Radix Paeoniae（RP）	MOL006994	1-o-beta-d-glucopyranosyl-8-o-benzoylpaeonisuffrone_qt	Muscarinic acetylcholine receptor M1	P11229	CHRM1	0.206
Radix Paeoniae（RP）	MOL006994	1-o-beta-d-glucopyranosyl-8-o-benzoylpaeonisuffrone_qt	Nuclear receptor coactivator 1	Q15788	NCOA1	0.268
Radix Paeoniae（RP）	MOL006994	1-o-beta-d-glucopyranosyl-8-o-benzoylpaeonisuffrone_qt	cAMP-dependent protein kinase catalytic subunit alpha	P17612	PRKACA	0.271
Radix Paeoniae（RP）	MOL006994	1-o-beta-d-glucopyranosyl-8-o-benzoylpaeonisuffrone_qt	Mu-type opioid receptor	P35372	OPRM1	0.334
Radix Paeoniae（RP）	MOL006994	1-o-beta-d-glucopyranosyl-8-o-benzoylpaeonisuffrone_qt	Prostaglandin G/H synthase 1	P23219	PTGS1	0.405
Radix Paeoniae（RP）	MOL006994	1-o-beta-d-glucopyranosyl-8-o-benzoylpaeonisuffrone_qt	Estrogen receptor beta	Q92731	ESR2	0.479
Radix Paeoniae（RP）	MOL006994	1-o-beta-d-glucopyranosyl-8-o-benzoylpaeonisuffrone_qt	Estrogen receptor	P03372	ESR1	0.563
Radix Paeoniae（RP）	MOL006994	1-o-beta-d-glucopyranosyl-8-o-benzoylpaeonisuffrone_qt	Prostaglandin G/H synthase 2	P35354	PTGS2	1
Radix Paeoniae（RP）	MOL006996	1-o-beta-d-glucopyranosylpaeonisuffrone_qt	D-HSCDK2	O75100	CA11	0.01
Radix Paeoniae（RP）	MOL006996	1-o-beta-d-glucopyranosylpaeonisuffrone_qt	Cell division protein kinase 2	P24941	CDK2	0.01
Radix Paeoniae（RP）	MOL006996	1-o-beta-d-glucopyranosylpaeonisuffrone_qt	Glycogen synthase kinase-3 beta	P49841	GSK3B	0.01
Radix Paeoniae（RP）	MOL006996	1-o-beta-d-glucopyranosylpaeonisuffrone_qt	Cyclin-dependent kinase 5 activator 1	Q15078	CDK5R1	0.01
Radix Paeoniae（RP）	MOL006996	1-o-beta-d-glucopyranosylpaeonisuffrone_qt	Potassium channel subfamily K member 6	Q9Y257	KCNK6	0.01
Radix Paeoniae（RP）	MOL006996	1-o-beta-d-glucopyranosylpaeonisuffrone_qt	Retinoic acid receptor alpha	P10276	RARA	0.015
Radix Paeoniae（RP）	MOL006996	1-o-beta-d-glucopyranosylpaeonisuffrone_qt	Retinoic acid receptor beta	P10826	RARB	0.015
Radix Paeoniae（RP）	MOL006996	1-o-beta-d-glucopyranosylpaeonisuffrone_qt	Retinoic acid receptor gamma-1	P13631	RARG	0.015
Radix Paeoniae（RP）	MOL006996	1-o-beta-d-glucopyranosylpaeonisuffrone_qt	Retinoic acid receptor RXR-gamma	P48443	RXRG	0.015
Radix Paeoniae（RP）	MOL006996	1-o-beta-d-glucopyranosylpaeonisuffrone_qt	Interleukin-3	P08700	IL3	0.016
Radix Paeoniae（RP）	MOL006996	1-o-beta-d-glucopyranosylpaeonisuffrone_qt	Protein S100-A12	P80511	S100A12	0.016
Radix Paeoniae（RP）	MOL006996	1-o-beta-d-glucopyranosylpaeonisuffrone_qt	Protein S100-A13	Q99584	S100A13	0.016
Radix Paeoniae（RP）	MOL006996	1-o-beta-d-glucopyranosylpaeonisuffrone_qt	Tyrosine-protein kinase JAK2	O60674	JAK2	0.017
Radix Paeoniae（RP）	MOL006996	1-o-beta-d-glucopyranosylpaeonisuffrone_qt	ATP synthase subunit beta, mitochondrial	P06576	ATP5F1B	0.017
Radix Paeoniae（RP）	MOL006996	1-o-beta-d-glucopyranosylpaeonisuffrone_qt	Tyrosine-protein kinase JAK1	P23458	JAK1	0.017
Radix Paeoniae（RP）	MOL006996	1-o-beta-d-glucopyranosylpaeonisuffrone_qt	ATP synthase subunit alpha, mitochondrial	P25705	ATP5F1A	0.017
Radix Paeoniae（RP）	MOL006996	1-o-beta-d-glucopyranosylpaeonisuffrone_qt	Tyrosine-protein kinase JAK3	P52333	JAK3	0.017
Radix Paeoniae（RP）	MOL006996	1-o-beta-d-glucopyranosylpaeonisuffrone_qt	Arachidonate 5-lipoxygenase	P09917	ALOX5	0.018
Radix Paeoniae（RP）	MOL006996	1-o-beta-d-glucopyranosylpaeonisuffrone_qt	Mu-type opioid receptor	P35372	OPRM1	0.018

Radix Paeoniae（RP）	MOL006996	1-o-beta-d-glucopyranosylpaeonisuffrone_qt	Aryl hydrocarbon receptor	P35869	AHR	0.018
Radix Paeoniae（RP）	MOL006996	1-o-beta-d-glucopyranosylpaeonisuffrone_qt	Amiloride-sensitive sodium channel subunit alpha	P37088	SCNN1A	0.018
Radix Paeoniae（RP）	MOL006996	1-o-beta-d-glucopyranosylpaeonisuffrone_qt	Delta-type opioid receptor	P41143	OPRD1	0.018
Radix Paeoniae（RP）	MOL006996	1-o-beta-d-glucopyranosylpaeonisuffrone_qt	Kappa-type opioid receptor	P41145	OPRK1	0.018
Radix Paeoniae（RP）	MOL006996	1-o-beta-d-glucopyranosylpaeonisuffrone_qt	Amiloride-sensitive sodium channel subunit beta	P51168	SCNN1B	0.018
Radix Paeoniae（RP）	MOL006996	1-o-beta-d-glucopyranosylpaeonisuffrone_qt	Amiloride-sensitive sodium channel subunit gamma	P51170	SCNN1G	0.018
Radix Paeoniae（RP）	MOL006996	1-o-beta-d-glucopyranosylpaeonisuffrone_qt	Dihydroorotate dehydrogenase, mitochondrial	Q02127	DHODH	0.018
Radix Paeoniae（RP）	MOL006996	1-o-beta-d-glucopyranosylpaeonisuffrone_qt	Gamma-aminobutyric-acid receptor subunit alpha-1	P14867	GABRA1	0.019
Radix Paeoniae（RP）	MOL006996	1-o-beta-d-glucopyranosylpaeonisuffrone_qt	Gamma-aminobutyric-acid receptor subunit alpha-2	P47869	GABRA2	0.019
Radix Paeoniae（RP）	MOL006996	1-o-beta-d-glucopyranosylpaeonisuffrone_qt	Muscarinic acetylcholine receptor M5	P08912	CHRM5	0.022
Radix Paeoniae（RP）	MOL006996	1-o-beta-d-glucopyranosylpaeonisuffrone_qt	Histamine H1 receptor	P35367	HRH1	0.022
Radix Paeoniae（RP）	MOL006996	1-o-beta-d-glucopyranosylpaeonisuffrone_qt	Carbonic anhydrase 1	P00915	CA1	0.024
Radix Paeoniae（RP）	MOL006996	1-o-beta-d-glucopyranosylpaeonisuffrone_qt	Carbonic anhydrase 2	P00918	CA2	0.024
Radix Paeoniae（RP）	MOL006996	1-o-beta-d-glucopyranosylpaeonisuffrone_qt	Sodium/potassium-transporting ATPase gamma chain	P54710	FXYD2	0.024
Radix Paeoniae（RP）	MOL006996	1-o-beta-d-glucopyranosylpaeonisuffrone_qt	Oxysterols receptor LXR-beta	P55055	NR1H2	0.024
Radix Paeoniae（RP）	MOL006996	1-o-beta-d-glucopyranosylpaeonisuffrone_qt	Oxysterols receptor LXR-alpha	Q13133	NR1H3	0.024
Radix Paeoniae（RP）	MOL006996	1-o-beta-d-glucopyranosylpaeonisuffrone_qt	Potassium channel subfamily K member 1	O00180	KCNK1	0.029
Radix Paeoniae（RP）	MOL006996	1-o-beta-d-glucopyranosylpaeonisuffrone_qt	Glucocorticoid receptor	P04150	NR3C1	0.03
Radix Paeoniae（RP）	MOL006996	1-o-beta-d-glucopyranosylpaeonisuffrone_qt	Progesterone receptor	P06401	PGR	0.03
Radix Paeoniae（RP）	MOL006996	1-o-beta-d-glucopyranosylpaeonisuffrone_qt	Cell division control protein 2 homolog	P06493	CDK1	0.032
Radix Paeoniae（RP）	MOL006996	1-o-beta-d-glucopyranosylpaeonisuffrone_qt	NAD(P)H dehydrogenase [quinone] 1	P15559	NQO1	0.032
Radix Paeoniae（RP）	MOL006996	1-o-beta-d-glucopyranosylpaeonisuffrone_qt	Cell division protein kinase 5	Q00535	CDK5	0.032
Radix Paeoniae（RP）	MOL006996	1-o-beta-d-glucopyranosylpaeonisuffrone_qt	Sterol O-acyltransferase 2	O75908	SOAT2	0.035
Radix Paeoniae（RP）	MOL006996	1-o-beta-d-glucopyranosylpaeonisuffrone_qt	Sterol O-acyltransferase 1	P35610	SOAT1	0.035
Radix Paeoniae（RP）	MOL006996	1-o-beta-d-glucopyranosylpaeonisuffrone_qt	DNA topoisomerase 2-alpha	P11388	TOP2A	0.039
Radix Paeoniae（RP）	MOL006996	1-o-beta-d-glucopyranosylpaeonisuffrone_qt	Protein tyrosine kinase 2 beta	Q14289	PTK2B	0.039
Radix Paeoniae（RP）	MOL006996	1-o-beta-d-glucopyranosylpaeonisuffrone_qt	Platelet glycoprotein IX	P14770	GP9	0.041
Radix Paeoniae（RP）	MOL006996	1-o-beta-d-glucopyranosylpaeonisuffrone_qt	Retinoic acid receptor RXR-beta	P28702	RXRB	0.05
Radix Paeoniae（RP）	MOL006996	1-o-beta-d-glucopyranosylpaeonisuffrone_qt	Sodium channel protein type 5 subunit alpha	Q14524	SCN5A	0.051
Radix Paeoniae（RP）	MOL006996	1-o-beta-d-glucopyranosylpaeonisuffrone_qt	Muscarinic acetylcholine receptor M3	P20309	CHRM3	0.056
Radix Paeoniae（RP）	MOL006996	1-o-beta-d-glucopyranosylpaeonisuffrone_qt	Nuclear receptor coactivator 2	Q15596	NCOA2	0.058
Radix Paeoniae（RP）	MOL006996	1-o-beta-d-glucopyranosylpaeonisuffrone_qt	Ribosyldihydronicotinamide dehydrogenase [quinone]	P16083	NQO2	0.059
Radix Paeoniae（RP）	MOL006996	1-o-beta-d-glucopyranosylpaeonisuffrone_qt	Casein kinase II subunit alpha	P68400	CSNK2A1	0.059
Radix Paeoniae（RP）	MOL006996	1-o-beta-d-glucopyranosylpaeonisuffrone_qt	Epidermal growth factor receptor	P00533	EGFR	0.071
Radix Paeoniae（RP）	MOL006996	1-o-beta-d-glucopyranosylpaeonisuffrone_qt	cAMP-dependent protein kinase catalytic subunit alpha	P17612	PRKACA	0.071
Radix Paeoniae（RP）	MOL006996	1-o-beta-d-glucopyranosylpaeonisuffrone_qt	Glycogen phosphorylase, muscle form	P11217	PYGM	0.072
Radix Paeoniae（RP）	MOL006996	1-o-beta-d-glucopyranosylpaeonisuffrone_qt	Mineralocorticoid receptor	P08235	NR3C2	0.073
Radix Paeoniae（RP）	MOL006996	1-o-beta-d-glucopyranosylpaeonisuffrone_qt	Nuclear receptor coactivator 1	Q15788	NCOA1	0.075
Radix Paeoniae（RP）	MOL006996	1-o-beta-d-glucopyranosylpaeonisuffrone_qt	Tubulin alpha-3 chain	Q71U36	TUBA1A	0.075
Radix Paeoniae（RP）	MOL006996	1-o-beta-d-glucopyranosylpaeonisuffrone_qt	RAC-alpha serine/threonine-protein kinase	P31749	AKT1	0.079
Radix Paeoniae（RP）	MOL006996	1-o-beta-d-glucopyranosylpaeonisuffrone_qt	ATP-sensitive inward rectifier potassium channel 1	P48048	KCNJ1	0.081
Radix Paeoniae（RP）	MOL006996	1-o-beta-d-glucopyranosylpaeonisuffrone_qt	Fibroblast growth factor receptor 2	P21802	FGFR2	0.082
Radix Paeoniae（RP）	MOL006996	1-o-beta-d-glucopyranosylpaeonisuffrone_qt	ATP-binding cassette transporter sub-family C member 8	Q09428	ABCC8	0.084
Radix Paeoniae（RP）	MOL006996	1-o-beta-d-glucopyranosylpaeonisuffrone_qt	Calmodulin	P62158		0.085
Radix Paeoniae（RP）	MOL006996	1-o-beta-d-glucopyranosylpaeonisuffrone_qt	Prolyl endopeptidase	P48147	PREP	0.086
Radix Paeoniae（RP）	MOL006996	1-o-beta-d-glucopyranosylpaeonisuffrone_qt	Peroxisome proliferator-activated receptor alpha	Q07869	PPARA	0.086
Radix Paeoniae（RP）	MOL006996	1-o-beta-d-glucopyranosylpaeonisuffrone_qt	Mitogen-activated protein kinase 14	Q16539	MAPK14	0.087

Radix Paeoniae（RP）	MOL006996	1-o-beta-d-glucopyranosylpaeonisuffrone_qt	Liver carboxylesterase 1	P23141	CES1	0.088
Radix Paeoniae（RP）	MOL006996	1-o-beta-d-glucopyranosylpaeonisuffrone_qt	Muscarinic acetylcholine receptor M2	P08172	CHRM2	0.09
Radix Paeoniae（RP）	MOL006996	1-o-beta-d-glucopyranosylpaeonisuffrone_qt	Muscarinic acetylcholine receptor M4	P08173	CHRM4	0.09
Radix Paeoniae（RP）	MOL006996	1-o-beta-d-glucopyranosylpaeonisuffrone_qt	Sodium-dependent dopamine transporter	Q01959	SLC6A3	0.091
Radix Paeoniae（RP）	MOL006996	1-o-beta-d-glucopyranosylpaeonisuffrone_qt	Sodium-dependent serotonin transporter	P31645	SLC6A4	0.093
Radix Paeoniae（RP）	MOL006996	1-o-beta-d-glucopyranosylpaeonisuffrone_qt	Muscarinic acetylcholine receptor M1	P11229	CHRM1	0.124
Radix Paeoniae（RP）	MOL006996	1-o-beta-d-glucopyranosylpaeonisuffrone_qt	Sodium-dependent noradrenaline transporter	P23975	SLC6A2	0.143
Radix Paeoniae（RP）	MOL006996	1-o-beta-d-glucopyranosylpaeonisuffrone_qt	Serine/threonine-protein kinase 6	O14965	AURKA	0.157
Radix Paeoniae（RP）	MOL006996	1-o-beta-d-glucopyranosylpaeonisuffrone_qt	Cyclin-A2	P20248	CCNA2	0.16
Radix Paeoniae（RP）	MOL006996	1-o-beta-d-glucopyranosylpaeonisuffrone_qt	Trypsin-1	P07477	PRSS1	0.165
Radix Paeoniae（RP）	MOL006996	1-o-beta-d-glucopyranosylpaeonisuffrone_qt	Hemoglobin subunit alpha	P69905	HBA1	0.183
Radix Paeoniae（RP）	MOL006996	1-o-beta-d-glucopyranosylpaeonisuffrone_qt	Estrogen receptor beta	Q92731	ESR2	0.198
Radix Paeoniae（RP）	MOL006996	1-o-beta-d-glucopyranosylpaeonisuffrone_qt	Estrogen receptor	P03372	ESR1	0.317
Radix Paeoniae（RP）	MOL006996	1-o-beta-d-glucopyranosylpaeonisuffrone_qt	Prostaglandin G/H synthase 1	P23219	PTGS1	0.43
Radix Paeoniae（RP）	MOL006996	1-o-beta-d-glucopyranosylpaeonisuffrone_qt	Prostaglandin G/H synthase 2	P35354	PTGS2	1
Radix Paeoniae（RP）	MOL006999	stigmast-7-en-3-ol	Elongation factor Tu GTP-binding domain-containing protein 1	Q7Z2Z2	EFL1	0.014
Radix Paeoniae（RP）	MOL006999	stigmast-7-en-3-ol	Neuronal acetylcholine receptor subunit alpha-3	P32297	CHRNA3	0.027
Radix Paeoniae（RP）	MOL006999	stigmast-7-en-3-ol	Neuronal acetylcholine receptor subunit alpha-7	P36544	CHRNA7	0.027
Radix Paeoniae（RP）	MOL006999	stigmast-7-en-3-ol	Neuronal acetylcholine receptor subunit alpha-4	P43681	CHRNA4	0.027
Radix Paeoniae（RP）	MOL006999	stigmast-7-en-3-ol	Dehydrogenase/reductase SDR family member 8	Q8NBQ5	HSD17B11	0.035
Radix Paeoniae（RP）	MOL006999	stigmast-7-en-3-ol	Ig kappa chain C region	P01834	IGKC	0.036
Radix Paeoniae（RP）	MOL006999	stigmast-7-en-3-ol	Ig gamma-1 chain C region	P01857	IGHG1	0.036
Radix Paeoniae（RP）	MOL006999	stigmast-7-en-3-ol	Ig gamma-2 chain C region	P01859	IGHG2	0.036
Radix Paeoniae（RP）	MOL006999	stigmast-7-en-3-ol	Nuclear receptor subfamily 1 group I member 3	Q14994	NR1I3	0.037
Radix Paeoniae（RP）	MOL006999	stigmast-7-en-3-ol	Prothrombin	P00734	F2	0.045
Radix Paeoniae（RP）	MOL006999	stigmast-7-en-3-ol	Trypsin-1	P07477	PRSS1	0.045
Radix Paeoniae（RP）	MOL006999	stigmast-7-en-3-ol	Retinoic acid receptor gamma-1	P13631	RARG	0.054
Radix Paeoniae（RP）	MOL006999	stigmast-7-en-3-ol	3 beta-hydroxysteroid dehydrogenase/Delta 5-->4-isomerase type II	P26439	HSD3B2	0.057
Radix Paeoniae（RP）	MOL006999	stigmast-7-en-3-ol	Annexin A1	P04083	ANXA1	0.059
Radix Paeoniae（RP）	MOL006999	stigmast-7-en-3-ol	Nuclear receptor 0B1	P51843	NR0B1	0.059
Radix Paeoniae（RP）	MOL006999	stigmast-7-en-3-ol	Microtubule-associated protein 2	P11137	MAP2	0.061
Radix Paeoniae（RP）	MOL006999	stigmast-7-en-3-ol	Microtubule-associated protein 1A	P78559	MAP1A	0.061
Radix Paeoniae（RP）	MOL006999	stigmast-7-en-3-ol	Prolactin receptor	P16471	PRLR	0.062
Radix Paeoniae（RP）	MOL006999	stigmast-7-en-3-ol	Gonadotropin-releasing hormone receptor	P30968	GNRHR	0.062
Radix Paeoniae（RP）	MOL006999	stigmast-7-en-3-ol	Gonadotropin-releasing hormone II receptor	Q96P88	GNRHR2	0.062
Radix Paeoniae（RP）	MOL006999	stigmast-7-en-3-ol	Corticosteroid 11-beta-dehydrogenase isozyme 1	P28845	HSD11B1	0.063
Radix Paeoniae（RP）	MOL006999	stigmast-7-en-3-ol	Bile salt sulfotransferase	Q06520	SULT2A1	0.079
Radix Paeoniae（RP）	MOL006999	stigmast-7-en-3-ol	Retinoic acid receptor alpha	P10276	RARA	0.087
Radix Paeoniae（RP）	MOL006999	stigmast-7-en-3-ol	Retinoic acid receptor beta	P10826	RARB	0.087
Radix Paeoniae（RP）	MOL006999	stigmast-7-en-3-ol	Retinoic acid receptor RXR-beta	P28702	RXRB	0.087
Radix Paeoniae（RP）	MOL006999	stigmast-7-en-3-ol	Retinoic acid receptor RXR-gamma	P48443	RXRG	0.087
Radix Paeoniae（RP）	MOL006999	stigmast-7-en-3-ol	cAMP-dependent protein kinase catalytic subunit alpha	P17612	PRKACA	0.101
Radix Paeoniae（RP）	MOL006999	stigmast-7-en-3-ol	Nuclear receptor coactivator 2	Q15596	NCOA2	0.101
Radix Paeoniae（RP）	MOL006999	stigmast-7-en-3-ol	DNA polymerase kappa	Q9UBT6	POLK	0.101
Radix Paeoniae（RP）	MOL006999	stigmast-7-en-3-ol	Retinoic acid receptor RXR-alpha	P19793	RXRA	0.104
Radix Paeoniae（RP）	MOL006999	stigmast-7-en-3-ol	ATP-binding cassette transporter sub-family C member 8	Q09428	ABCC8	0.105
Radix Paeoniae（RP）	MOL006999	stigmast-7-en-3-ol	3 beta-hydroxysteroid dehydrogenase/Delta 5-->4-isomerase type I	P14060	HSD3B1	0.114
Radix Paeoniae（RP）	MOL006999	stigmast-7-en-3-ol	Aldo-keto reductase family 1 member C1	Q04828	AKR1C1	0.115
Radix Paeoniae（RP）	MOL006999	stigmast-7-en-3-ol	Nitric-oxide synthase, endothelial	P29474	NOS3	0.118
Radix Paeoniae（RP）	MOL006999	stigmast-7-en-3-ol	Cannabinoid receptor 2	P34972	CNR2	0.123
Radix Paeoniae（RP）	MOL006999	stigmast-7-en-3-ol	Nuclear receptor coactivator 5	Q9HCD5	NCOA5	0.129

Radix Paeoniae（RP）	MOL006999	stigmast-7-en-3-ol	3-oxo-5-alpha-steroid 4-dehydrogenase 1	P18405	SRD5A1	0.14
Radix Paeoniae（RP）	MOL006999	stigmast-7-en-3-ol	3-oxo-5-alpha-steroid 4-dehydrogenase 2	P31213	SRD5A2	0.144
Radix Paeoniae（RP）	MOL006999	stigmast-7-en-3-ol	Estrogen receptor beta	Q92731	ESR2	0.191
Radix Paeoniae（RP）	MOL006999	stigmast-7-en-3-ol	Estradiol 17-beta-dehydrogenase 1	P14061	HSD17B1	0.194
Radix Paeoniae（RP）	MOL006999	stigmast-7-en-3-ol	Androgen receptor	P10275	AR	0.21
Radix Paeoniae（RP）	MOL006999	stigmast-7-en-3-ol	Nuclear receptor coactivator 1	Q15788	NCOA1	0.217
Radix Paeoniae（RP）	MOL006999	stigmast-7-en-3-ol	Glucocorticoid receptor	P04150	NR3C1	0.272
Radix Paeoniae（RP）	MOL006999	stigmast-7-en-3-ol	Mineralocorticoid receptor	P08235	NR3C2	0.356
Radix Paeoniae（RP）	MOL006999	stigmast-7-en-3-ol	Progesterone receptor	P06401	PGR	0.955
Radix Paeoniae（RP）	MOL006999	stigmast-7-en-3-ol	Estrogen receptor	P03372	ESR1	1
Radix Paeoniae（RP）	MOL007002	paeoniflorin	Tumor necrosis factor	P01375	TNF	Validated
Radix Paeoniae（RP）	MOL007002	paeoniflorin	Interleukin-6	P05231	IL6	Validated
Radix Paeoniae（RP）	MOL007002	paeoniflorin	Monocyte differentiation antigen CD14	P08571	CD14	Validated
Radix Paeoniae（RP）	MOL007002	paeoniflorin	Lipopolysaccharide-binding protein	P18428	LBP	Validated
Radix Paeoniae（RP）	MOL007003	benzoyl paeoniflorin	5-hydroxytryptamine 3 receptor	P46098	HTR3A	0.014
Radix Paeoniae（RP）	MOL007003	benzoyl paeoniflorin	Glutamate [NMDA] receptor subunit epsilon-1	Q12879	GRIN2A	0.014
Radix Paeoniae（RP）	MOL007003	benzoyl paeoniflorin	Glutamate [NMDA] receptor subunit epsilon-2	Q13224	GRIN2B	0.014
Radix Paeoniae（RP）	MOL007003	benzoyl paeoniflorin	Glutamate [NMDA] receptor subunit 3A	Q8TCU5	GRIN3A	0.014
Radix Paeoniae（RP）	MOL007003	benzoyl paeoniflorin	Muscarinic acetylcholine receptor M2	P08172	CHRM2	0.023
Radix Paeoniae（RP）	MOL007003	benzoyl paeoniflorin	Muscarinic acetylcholine receptor M4	P08173	CHRM4	0.023
Radix Paeoniae（RP）	MOL007003	benzoyl paeoniflorin	Muscarinic acetylcholine receptor M1	P11229	CHRM1	0.023
Radix Paeoniae（RP）	MOL007003	benzoyl paeoniflorin	Muscarinic acetylcholine receptor M3	P20309	CHRM3	0.023
Radix Paeoniae（RP）	MOL007003	benzoyl paeoniflorin	Carbonic anhydrase 1	P00915	CA1	0.106
Radix Paeoniae（RP）	MOL007003	benzoyl paeoniflorin	Carbonic anhydrase 2	P00918	CA2	0.106
Radix Paeoniae（RP）	MOL007003	benzoyl paeoniflorin	Sodium/potassium-transporting ATPase gamma chain	P54710	FXYD2	0.106
Radix Paeoniae（RP）	MOL007003	benzoyl paeoniflorin	Potassium channel subfamily K member 1	O00180	KCNK1	0.126
Radix Paeoniae（RP）	MOL007003	benzoyl paeoniflorin	Sodium channel protein type 5 subunit alpha	Q14524	SCN5A	0.126
Radix Paeoniae（RP）	MOL007003	benzoyl paeoniflorin	Potassium channel subfamily K member 6	Q9Y257	KCNK6	0.126
Radix Paeoniae（RP）	MOL007003	benzoyl paeoniflorin	Tripartite motif-containing protein 13	O60858	TRIM13	0.149
Radix Paeoniae（RP）	MOL007003	benzoyl paeoniflorin	Delta-type opioid receptor	P41143	OPRD1	0.149
Radix Paeoniae（RP）	MOL007003	benzoyl paeoniflorin	Platelet glycoprotein IX	P14770	GP9	0.327
Radix Paeoniae（RP）	MOL007003	benzoyl paeoniflorin	Estrogen receptor	P03372	ESR1	0.841
Radix Paeoniae（RP）	MOL007003	benzoyl paeoniflorin	Mu-type opioid receptor	P35372	OPRM1	1
Radix Paeoniae（RP）	MOL007003	benzoyl paeoniflorin	Kappa-type opioid receptor	P41145	OPRK1	1
Radix Paeoniae（RP）	MOL007004	Albiflorin	Platelet glycoprotein IX	P14770	GP9	0.285
Radix Paeoniae（RP）	MOL007004	Albiflorin	Estrogen receptor	P03372	ESR1	1
Radix Paeoniae（RP）	MOL007005	Albiflorin_qt	Tumor necrosis factor	P01375	TNF	0.018
Radix Paeoniae（RP）	MOL007005	Albiflorin_qt	Nuclear factor NF-kappa-B p105 subunit	P19838	NFKB1	0.018
Radix Paeoniae（RP）	MOL007005	Albiflorin_qt	Fibroblast growth factor receptor 2	P21802	FGFR2	0.018
Radix Paeoniae（RP）	MOL007005	Albiflorin_qt	Trypsin-2	P07478	PRSS2	0.024
Radix Paeoniae（RP）	MOL007005	Albiflorin_qt	Cell division control protein 2 homolog	P06493	CDK1	0.031
Radix Paeoniae（RP）	MOL007005	Albiflorin_qt	Cell division protein kinase 2	P24941	CDK2	0.031
Radix Paeoniae（RP）	MOL007005	Albiflorin_qt	Cell division protein kinase 5	Q00535	CDK5	0.031
Radix Paeoniae（RP）	MOL007005	Albiflorin_qt	Arachidonate 5-lipoxygenase	P09917	ALOX5	0.034
Radix Paeoniae（RP）	MOL007005	Albiflorin_qt	Aryl hydrocarbon receptor	P35869	AHR	0.034
Radix Paeoniae（RP）	MOL007005	Albiflorin_qt	Dihydroorotate dehydrogenase, mitochondrial	Q02127	DHODH	0.034
Radix Paeoniae（RP）	MOL007005	Albiflorin_qt	Muscarinic acetylcholine receptor M1	P11229	CHRM1	0.041
Radix Paeoniae（RP）	MOL007005	Albiflorin_qt	Prostaglandin G/H synthase 1	P23219	PTGS1	0.156
Radix Paeoniae（RP）	MOL007005	Albiflorin_qt	Calmodulin	P62158		0.158
Radix Paeoniae（RP）	MOL007005	Albiflorin_qt	Egl nine homolog 1	Q9GZT9	EGLN1	0.158
Radix Paeoniae（RP）	MOL007005	Albiflorin_qt	Mannose-binding protein C	P11226	MBL2	0.162
Radix Paeoniae（RP）	MOL007005	Albiflorin_qt	Tubulin alpha-3 chain	Q71U36	TUBA1A	0.164
Radix Paeoniae（RP）	MOL007005	Albiflorin_qt	ATP-binding cassette transporter sub-family C member 8	Q09428	ABCC8	0.176

Radix Paeoniae（RP）	MOL007005	Albiflorin_qt	Methionine aminopeptidase 2	P50579	METAP2	0.179
Radix Paeoniae（RP）	MOL007005	Albiflorin_qt	Prostaglandin G/H synthase 2	P35354	PTGS2	1
Radix Paeoniae（RP）	MOL007008	4-ethyl-paeoniflorin_qt	Actin-related protein 2/3 complex subunit 1B	O15143	ARPC1B	0.011
Radix Paeoniae（RP）	MOL007008	4-ethyl-paeoniflorin_qt	Actin-related protein 2/3 complex subunit 2	O15144	ARPC2	0.011
Radix Paeoniae（RP）	MOL007008	4-ethyl-paeoniflorin_qt	Actin-related protein 2/3 complex subunit 3	O15145	ARPC3	0.011
Radix Paeoniae（RP）	MOL007008	4-ethyl-paeoniflorin_qt	D-HSCDK2	O75100	CA11	0.011
Radix Paeoniae（RP）	MOL007008	4-ethyl-paeoniflorin_qt	Cell division control protein 2 homolog	P06493	CDK1	0.011
Radix Paeoniae（RP）	MOL007008	4-ethyl-paeoniflorin_qt	Glycogen synthase kinase-3 beta	P49841	GSK3B	0.011
Radix Paeoniae（RP）	MOL007008	4-ethyl-paeoniflorin_qt	Actin-related protein 2/3 complex subunit 4	P59998	ARPC4	0.011
Radix Paeoniae（RP）	MOL007008	4-ethyl-paeoniflorin_qt	Actin-related protein 3	P61158	ACTR3	0.011
Radix Paeoniae（RP）	MOL007008	4-ethyl-paeoniflorin_qt	Actin-related protein 2	P61160	ACTR2	0.011
Radix Paeoniae（RP）	MOL007008	4-ethyl-paeoniflorin_qt	Cell division protein kinase 5	Q00535	CDK5	0.011
Radix Paeoniae（RP）	MOL007008	4-ethyl-paeoniflorin_qt	Cyclin-dependent kinase 5 activator 1	Q15078	CDK5R1	0.011
Radix Paeoniae（RP）	MOL007008	4-ethyl-paeoniflorin_qt	Alpha-2A adrenergic receptor	P08913	ADRA2A	0.013
Radix Paeoniae（RP）	MOL007008	4-ethyl-paeoniflorin_qt	D(2) dopamine receptor	P14416	DRD2	0.013
Radix Paeoniae（RP）	MOL007008	4-ethyl-paeoniflorin_qt	Alpha-2B adrenergic receptor	P18089	ADRA2B	0.013
Radix Paeoniae（RP）	MOL007008	4-ethyl-paeoniflorin_qt	Alpha-2C adrenergic receptor	P18825	ADRA2C	0.013
Radix Paeoniae（RP）	MOL007008	4-ethyl-paeoniflorin_qt	5-hydroxytryptamine 2C receptor	P28335	HTR2C	0.013
Radix Paeoniae（RP）	MOL007008	4-ethyl-paeoniflorin_qt	D(3) dopamine receptor	P35462	DRD3	0.013
Radix Paeoniae（RP）	MOL007008	4-ethyl-paeoniflorin_qt	5-hydroxytryptamine 2B receptor	P41595	HTR2B	0.013
Radix Paeoniae（RP）	MOL007008	4-ethyl-paeoniflorin_qt	Opioid receptor, sigma 1	Q5T1J1	SIGMAR1	0.013
Radix Paeoniae（RP）	MOL007008	4-ethyl-paeoniflorin_qt	Sigma 1-type opioid receptor	Q99720	SIGMAR1	0.013
Radix Paeoniae（RP）	MOL007008	4-ethyl-paeoniflorin_qt	Potassium channel subfamily K member 6	Q9Y257	KCNK6	0.019
Radix Paeoniae（RP）	MOL007008	4-ethyl-paeoniflorin_qt	5-hydroxytryptamine 3 receptor	P46098	HTR3A	0.02
Radix Paeoniae（RP）	MOL007008	4-ethyl-paeoniflorin_qt	Glutamate [NMDA] receptor subunit epsilon-1	Q12879	GRIN2A	0.02
Radix Paeoniae（RP）	MOL007008	4-ethyl-paeoniflorin_qt	Glutamate [NMDA] receptor subunit epsilon-2	Q13224	GRIN2B	0.02
Radix Paeoniae（RP）	MOL007008	4-ethyl-paeoniflorin_qt	Dihydrofolate reductase	P00374	DHFR	0.023
Radix Paeoniae（RP）	MOL007008	4-ethyl-paeoniflorin_qt	Serine/threonine-protein phosphatase 2A 65 kDa regulatory subunit A alpha isoform	P30153	PPP2R1A	0.023
Radix Paeoniae（RP）	MOL007008	4-ethyl-paeoniflorin_qt	Serine/threonine-protein phosphatase PP1-alpha catalytic subunit	P62136	PPP1CA	0.023
Radix Paeoniae（RP）	MOL007008	4-ethyl-paeoniflorin_qt	Carbonic anhydrase 1	P00915	CA1	0.024
Radix Paeoniae（RP）	MOL007008	4-ethyl-paeoniflorin_qt	Stromelysin-1	P08254	MMP3	0.024
Radix Paeoniae（RP）	MOL007008	4-ethyl-paeoniflorin_qt	5-hydroxytryptamine 1A receptor	P08908	HTR1A	0.024
Radix Paeoniae（RP）	MOL007008	4-ethyl-paeoniflorin_qt	Stromelysin-2	P09238	MMP10	0.024
Radix Paeoniae（RP）	MOL007008	4-ethyl-paeoniflorin_qt	Arachidonate 5-lipoxygenase	P09917	ALOX5	0.024
Radix Paeoniae（RP）	MOL007008	4-ethyl-paeoniflorin_qt	Amine oxidase [flavin-containing] A	P21397	MAOA	0.024
Radix Paeoniae（RP）	MOL007008	4-ethyl-paeoniflorin_qt	5-hydroxytryptamine 1D receptor	P28221	HTR1D	0.024
Radix Paeoniae（RP）	MOL007008	4-ethyl-paeoniflorin_qt	5-hydroxytryptamine 1B receptor	P28222	HTR1B	0.024
Radix Paeoniae（RP）	MOL007008	4-ethyl-paeoniflorin_qt	Aryl hydrocarbon receptor	P35869	AHR	0.024
Radix Paeoniae（RP）	MOL007008	4-ethyl-paeoniflorin_qt	Macrophage metalloelastase	P39900	MMP12	0.024
Radix Paeoniae（RP）	MOL007008	4-ethyl-paeoniflorin_qt	Dihydroorotate dehydrogenase, mitochondrial	Q02127	DHODH	0.024
Radix Paeoniae（RP）	MOL007008	4-ethyl-paeoniflorin_qt	Neuronal acetylcholine receptor subunit alpha-2	Q15822	CHRNA2	0.024
Radix Paeoniae（RP）	MOL007008	4-ethyl-paeoniflorin_qt	Solute carrier family 22 member 6	Q4U2R8	SLC22A6	0.024
Radix Paeoniae（RP）	MOL007008	4-ethyl-paeoniflorin_qt	Solute carrier family 22 member 8	Q8TCC7	SLC22A8	0.024
Radix Paeoniae（RP）	MOL007008	4-ethyl-paeoniflorin_qt	Solute carrier family 22 member 11	Q9NSA0	SLC22A11	0.024
Radix Paeoniae（RP）	MOL007008	4-ethyl-paeoniflorin_qt	Transient receptor potential cation channel subfamily A member 1	O75762	TRPA1	0.025
Radix Paeoniae（RP）	MOL007008	4-ethyl-paeoniflorin_qt	Transient receptor potential cation channel subfamily M member 8	Q7Z2W7	TRPM8	0.025
Radix Paeoniae（RP）	MOL007008	4-ethyl-paeoniflorin_qt	Transient receptor potential cation channel subfamily V member 3	Q8NET8	TRPV3	0.025
Radix Paeoniae（RP）	MOL007008	4-ethyl-paeoniflorin_qt	Retinoic acid receptor beta	P10826	RARB	0.027
Radix Paeoniae（RP）	MOL007008	4-ethyl-paeoniflorin_qt	Retinoic acid receptor gamma-1	P13631	RARG	0.027
Radix Paeoniae（RP）	MOL007008	4-ethyl-paeoniflorin_qt	Retinoic acid receptor RXR-beta	P28702	RXRB	0.027
Radix Paeoniae（RP）	MOL007008	4-ethyl-paeoniflorin_qt	Retinoic acid receptor RXR-gamma	P48443	RXRG	0.027
Radix Paeoniae（RP）	MOL007008	4-ethyl-paeoniflorin_qt	Nociceptin receptor	P41146	OPRL1	0.031
Radix Paeoniae（RP）	MOL007008	4-ethyl-paeoniflorin_qt	Annexin A1	P04083	ANXA1	0.038

Radix Paeoniae（RP）	MOL007008	4-ethyl-paeoniflorin_qt	Nuclear receptor 0B1	P51843	NR0B1	0.038
Radix Paeoniae（RP）	MOL007008	4-ethyl-paeoniflorin_qt	NAD(P)H dehydrogenase [quinone] 1	P15559	NQO1	0.039
Radix Paeoniae（RP）	MOL007008	4-ethyl-paeoniflorin_qt	Prolactin receptor	P16471	PRLR	0.039
Radix Paeoniae（RP）	MOL007008	4-ethyl-paeoniflorin_qt	Retinoic acid receptor RXR-alpha	P19793	RXRA	0.039
Radix Paeoniae（RP）	MOL007008	4-ethyl-paeoniflorin_qt	Gonadotropin-releasing hormone receptor	P30968	GNRHR	0.039
Radix Paeoniae（RP）	MOL007008	4-ethyl-paeoniflorin_qt	Estrogen-related receptor gamma	P62508	ESRRG	0.039
Radix Paeoniae（RP）	MOL007008	4-ethyl-paeoniflorin_qt	Nuclear receptor subfamily 1 group I member 3	Q14994	NR1I3	0.039
Radix Paeoniae（RP）	MOL007008	4-ethyl-paeoniflorin_qt	Gonadotropin-releasing hormone II receptor	Q96P88	GNRHR2	0.039
Radix Paeoniae（RP）	MOL007008	4-ethyl-paeoniflorin_qt	Carbonic anhydrase 2	P00918	CA2	0.04
Radix Paeoniae（RP）	MOL007008	4-ethyl-paeoniflorin_qt	DNA topoisomerase 2-alpha	P11388	TOP2A	0.04
Radix Paeoniae（RP）	MOL007008	4-ethyl-paeoniflorin_qt	Protein tyrosine kinase 2 beta	Q14289	PTK2B	0.04
Radix Paeoniae（RP）	MOL007008	4-ethyl-paeoniflorin_qt	Potassium channel subfamily K member 1	O00180	KCNK1	0.046
Radix Paeoniae（RP）	MOL007008	4-ethyl-paeoniflorin_qt	Gamma-aminobutyric-acid receptor subunit alpha-1	P14867	GABRA1	0.049
Radix Paeoniae（RP）	MOL007008	4-ethyl-paeoniflorin_qt	Gamma-aminobutyric-acid receptor subunit alpha-2	P47869	GABRA2	0.049
Radix Paeoniae（RP）	MOL007008	4-ethyl-paeoniflorin_qt	Platelet glycoprotein IX	P14770	GP9	0.05
Radix Paeoniae（RP）	MOL007008	4-ethyl-paeoniflorin_qt	Serine/threonine-protein phosphatase 2A catalytic subunit alpha isoform	P67775	PPP2CA	0.053
Radix Paeoniae（RP）	MOL007008	4-ethyl-paeoniflorin_qt	Serine/threonine-protein phosphatase 2A 56 kDa regulatory subunit gamma isoform	Q13362	PPP2R5C	0.053
Radix Paeoniae（RP）	MOL007008	4-ethyl-paeoniflorin_qt	Ribosyldihydronicotinamide dehydrogenase [quinone]	P16083	NQO2	0.055
Radix Paeoniae（RP）	MOL007008	4-ethyl-paeoniflorin_qt	Casein kinase II subunit alpha	P68400	CSNK2A1	0.055
Radix Paeoniae（RP）	MOL007008	4-ethyl-paeoniflorin_qt	Sodium-dependent dopamine transporter	Q01959	SLC6A3	0.055
Radix Paeoniae（RP）	MOL007008	4-ethyl-paeoniflorin_qt	Phospholipase A2, membrane associated	P14555	PLA2G2A	0.057
Radix Paeoniae（RP）	MOL007008	4-ethyl-paeoniflorin_qt	Lactoylglutathione lyase	Q04760	GLO1	0.057
Radix Paeoniae（RP）	MOL007008	4-ethyl-paeoniflorin_qt	Prostaglandin reductase 2	Q8N8N7	PTGR2	0.057
Radix Paeoniae（RP）	MOL007008	4-ethyl-paeoniflorin_qt	Aldo-keto reductase family 1 member C3	P42330	AKR1C3	0.058
Radix Paeoniae（RP）	MOL007008	4-ethyl-paeoniflorin_qt	Platelet-activating factor acetylhydrolase IB subunit gamma	Q15102	PAFAH1B3	0.06
Radix Paeoniae（RP）	MOL007008	4-ethyl-paeoniflorin_qt	Retinoic acid receptor alpha	P10276	RARA	0.061
Radix Paeoniae（RP）	MOL007008	4-ethyl-paeoniflorin_qt	Glutamate [NMDA] receptor subunit 3A	Q8TCU5	GRIN3A	0.067
Radix Paeoniae（RP）	MOL007008	4-ethyl-paeoniflorin_qt	Tripartite motif-containing protein 13	O60858	TRIM13	0.07
Radix Paeoniae（RP）	MOL007008	4-ethyl-paeoniflorin_qt	Estradiol 17-beta-dehydrogenase 1	P14061	HSD17B1	0.082
Radix Paeoniae（RP）	MOL007008	4-ethyl-paeoniflorin_qt	Thymidylate synthase	P04818	TYMS	0.084
Radix Paeoniae（RP）	MOL007008	4-ethyl-paeoniflorin_qt	Cell division protein kinase 2	P24941	CDK2	0.084
Radix Paeoniae（RP）	MOL007008	4-ethyl-paeoniflorin_qt	Sodium channel protein type 5 subunit alpha	Q14524	SCN5A	0.086
Radix Paeoniae（RP）	MOL007008	4-ethyl-paeoniflorin_qt	Proto-oncogene tyrosine-protein kinase Src	P12931	SRC	0.087
Radix Paeoniae（RP）	MOL007008	4-ethyl-paeoniflorin_qt	Fibroblast growth factor receptor 2	P21802	FGFR2	0.087
Radix Paeoniae（RP）	MOL007008	4-ethyl-paeoniflorin_qt	ATP-sensitive inward rectifier potassium channel 1	P48048	KCNJ1	0.087
Radix Paeoniae（RP）	MOL007008	4-ethyl-paeoniflorin_qt	Glycogen phosphorylase, muscle form	P11217	PYGM	0.088
Radix Paeoniae（RP）	MOL007008	4-ethyl-paeoniflorin_qt	Cannabinoid receptor 2	P34972	CNR2	0.088
Radix Paeoniae（RP）	MOL007008	4-ethyl-paeoniflorin_qt	Potassium voltage-gated channel subfamily KQT member 1	P51787	KCNQ1	0.09
Radix Paeoniae（RP）	MOL007008	4-ethyl-paeoniflorin_qt	Aldo-keto reductase family 1 member B10	O60218	AKR1B10	0.092
Radix Paeoniae（RP）	MOL007008	4-ethyl-paeoniflorin_qt	Calmodulin	P62158		0.092
Radix Paeoniae（RP）	MOL007008	4-ethyl-paeoniflorin_qt	Sodium-dependent serotonin transporter	P31645	SLC6A4	0.093
Radix Paeoniae（RP）	MOL007008	4-ethyl-paeoniflorin_qt	Cyclin-A2	P20248	CCNA2	0.097
Radix Paeoniae（RP）	MOL007008	4-ethyl-paeoniflorin_qt	Mitogen-activated protein kinase 14	Q16539	MAPK14	0.099
Radix Paeoniae（RP）	MOL007008	4-ethyl-paeoniflorin_qt	Tyrosine-protein phosphatase non-receptor type 1	P18031	PTPN1	0.1
Radix Paeoniae（RP）	MOL007008	4-ethyl-paeoniflorin_qt	Hemoglobin subunit alpha	P69905	HBA1	0.1
Radix Paeoniae（RP）	MOL007008	4-ethyl-paeoniflorin_qt	ATP-binding cassette transporter sub-family C member 8	Q09428	ABCC8	0.1
Radix Paeoniae（RP）	MOL007008	4-ethyl-paeoniflorin_qt	C-jun-amino-terminal kinase-interacting protein 1	Q9UQF2	MAPK8IP1	0.103
Radix Paeoniae（RP）	MOL007008	4-ethyl-paeoniflorin_qt	Peroxisome proliferator-activated receptor alpha	Q07869	PPARA	0.109
Radix Paeoniae（RP）	MOL007008	4-ethyl-paeoniflorin_qt	Nuclear receptor coactivator 2	Q15596	NCOA2	0.115
Radix Paeoniae（RP）	MOL007008	4-ethyl-paeoniflorin_qt	Sodium-dependent noradrenaline transporter	P23975	SLC6A2	0.118
Radix Paeoniae（RP）	MOL007008	4-ethyl-paeoniflorin_qt	Liver carboxylesterase 1	P23141	CES1	0.127
Radix Paeoniae（RP）	MOL007008	4-ethyl-paeoniflorin_qt	Muscarinic acetylcholine receptor M2	P08172	CHRM2	0.128
Radix Paeoniae（RP）	MOL007008	4-ethyl-paeoniflorin_qt	Muscarinic acetylcholine receptor M4	P08173	CHRM4	0.128

Radix Paeoniae（RP）	MOL007008	4-ethyl-paeoniflorin_qt	Muscarinic acetylcholine receptor M3	P20309	CHRM3	0.128
Radix Paeoniae（RP）	MOL007008	4-ethyl-paeoniflorin_qt	Glucocorticoid receptor	P04150	NR3C1	0.13
Radix Paeoniae（RP）	MOL007008	4-ethyl-paeoniflorin_qt	Mineralocorticoid receptor	P08235	NR3C2	0.13
Radix Paeoniae（RP）	MOL007008	4-ethyl-paeoniflorin_qt	Androgen receptor	P10275	AR	0.131
Radix Paeoniae（RP）	MOL007008	4-ethyl-paeoniflorin_qt	Progesterone receptor	P06401	PGR	0.177
Radix Paeoniae（RP）	MOL007008	4-ethyl-paeoniflorin_qt	Nuclear receptor coactivator 1	Q15788	NCOA1	0.181
Radix Paeoniae（RP）	MOL007008	4-ethyl-paeoniflorin_qt	Peroxisome proliferator-activated receptor gamma	P37231	PPARG	0.182
Radix Paeoniae（RP）	MOL007008	4-ethyl-paeoniflorin_qt	Estrogen receptor beta	Q92731	ESR2	0.182
Radix Paeoniae（RP）	MOL007008	4-ethyl-paeoniflorin_qt	Trypsin-1	P07477	PRSS1	0.185
Radix Paeoniae（RP）	MOL007008	4-ethyl-paeoniflorin_qt	Delta-type opioid receptor	P41143	OPRD1	0.187
Radix Paeoniae（RP）	MOL007008	4-ethyl-paeoniflorin_qt	Muscarinic acetylcholine receptor M1	P11229	CHRM1	0.218
Radix Paeoniae（RP）	MOL007008	4-ethyl-paeoniflorin_qt	Kappa-type opioid receptor	P41145	OPRK1	0.265
Radix Paeoniae（RP）	MOL007008	4-ethyl-paeoniflorin_qt	Mu-type opioid receptor	P35372	OPRM1	0.381
Radix Paeoniae（RP）	MOL007008	4-ethyl-paeoniflorin_qt	cAMP-dependent protein kinase catalytic subunit alpha	P17612	PRKACA	0.382
Radix Paeoniae（RP）	MOL007008	4-ethyl-paeoniflorin_qt	Prostaglandin G/H synthase 1	P23219	PTGS1	0.444
Radix Paeoniae（RP）	MOL007008	4-ethyl-paeoniflorin_qt	Estrogen receptor	P03372	ESR1	0.506
Radix Paeoniae（RP）	MOL007008	4-ethyl-paeoniflorin_qt	Prostaglandin G/H synthase 2	P35354	PTGS2	1
Radix Paeoniae（RP）	MOL007012	4-o-methyl-paeoniflorin_qt	Gamma-aminobutyric-acid receptor subunit alpha-2	P47869	GABRA2	0.01
Radix Paeoniae（RP）	MOL007012	4-o-methyl-paeoniflorin_qt	Opioid receptor, sigma 1	Q5T1J1	SIGMAR1	0.014
Radix Paeoniae（RP）	MOL007012	4-o-methyl-paeoniflorin_qt	Sigma 1-type opioid receptor	Q99720	SIGMAR1	0.014
Radix Paeoniae（RP）	MOL007012	4-o-methyl-paeoniflorin_qt	Trypsin-2	P07478	PRSS2	0.017
Radix Paeoniae（RP）	MOL007012	4-o-methyl-paeoniflorin_qt	5-hydroxytryptamine 1B receptor	P28222	HTR1B	0.021
Radix Paeoniae（RP）	MOL007012	4-o-methyl-paeoniflorin_qt	5-hydroxytryptamine 3 receptor	P46098	HTR3A	0.021
Radix Paeoniae（RP）	MOL007012	4-o-methyl-paeoniflorin_qt	Glutamate [NMDA] receptor subunit epsilon-1	Q12879	GRIN2A	0.021
Radix Paeoniae（RP）	MOL007012	4-o-methyl-paeoniflorin_qt	Glutamate [NMDA] receptor subunit epsilon-2	Q13224	GRIN2B	0.021
Radix Paeoniae（RP）	MOL007012	4-o-methyl-paeoniflorin_qt	Acetylcholinesterase	P22303	ACHE	0.022
Radix Paeoniae（RP）	MOL007012	4-o-methyl-paeoniflorin_qt	Vascular endothelial growth factor	A2A2V4	VEGFA	0.026
Radix Paeoniae（RP）	MOL007012	4-o-methyl-paeoniflorin_qt	Tumor necrosis factor ligand superfamily member 11	O14788	TNFSF11	0.026
Radix Paeoniae（RP）	MOL007012	4-o-methyl-paeoniflorin_qt	Cadherin-5	P33151	CDH5	0.026
Radix Paeoniae（RP）	MOL007012	4-o-methyl-paeoniflorin_qt	Gamma-aminobutyric-acid receptor subunit beta-3	P28472	GABRB3	0.027
Radix Paeoniae（RP）	MOL007012	4-o-methyl-paeoniflorin_qt	Sodium channel protein type 4 subunit alpha	P35499	SCN4A	0.027
Radix Paeoniae（RP）	MOL007012	4-o-methyl-paeoniflorin_qt	Gamma-aminobutyric-acid receptor subunit beta-2	P47870	GABRB2	0.027
Radix Paeoniae（RP）	MOL007012	4-o-methyl-paeoniflorin_qt	Transient receptor potential cation channel subfamily A member 1	O75762	TRPA1	0.029
Radix Paeoniae（RP）	MOL007012	4-o-methyl-paeoniflorin_qt	Interleukin-3	P08700	IL3	0.029
Radix Paeoniae（RP）	MOL007012	4-o-methyl-paeoniflorin_qt	Protein S100-A12	P80511	S100A12	0.029
Radix Paeoniae（RP）	MOL007012	4-o-methyl-paeoniflorin_qt	Transient receptor potential cation channel subfamily M member 8	Q7Z2W7	TRPM8	0.029
Radix Paeoniae（RP）	MOL007012	4-o-methyl-paeoniflorin_qt	Transient receptor potential cation channel subfamily V member 3	Q8NET8	TRPV3	0.029
Radix Paeoniae（RP）	MOL007012	4-o-methyl-paeoniflorin_qt	Protein S100-A13	Q99584	S100A13	0.029
Radix Paeoniae（RP）	MOL007012	4-o-methyl-paeoniflorin_qt	Retinoic acid receptor beta	P10826	RARB	0.03
Radix Paeoniae（RP）	MOL007012	4-o-methyl-paeoniflorin_qt	Retinoic acid receptor gamma-1	P13631	RARG	0.03
Radix Paeoniae（RP）	MOL007012	4-o-methyl-paeoniflorin_qt	Retinoic acid receptor RXR-beta	P28702	RXRB	0.03
Radix Paeoniae（RP）	MOL007012	4-o-methyl-paeoniflorin_qt	Retinoic acid receptor RXR-gamma	P48443	RXRG	0.03
Radix Paeoniae（RP）	MOL007012	4-o-methyl-paeoniflorin_qt	Sodium channel protein type 9 subunit alpha	Q15858	SCN9A	0.032
Radix Paeoniae（RP）	MOL007012	4-o-methyl-paeoniflorin_qt	Sodium channel protein type 10 subunit alpha	Q9Y5Y9	SCN10A	0.032
Radix Paeoniae（RP）	MOL007012	4-o-methyl-paeoniflorin_qt	Potassium channel subfamily K member 1	O00180	KCNK1	0.033
Radix Paeoniae（RP）	MOL007012	4-o-methyl-paeoniflorin_qt	Arachidonate 5-lipoxygenase	P09917	ALOX5	0.033
Radix Paeoniae（RP）	MOL007012	4-o-methyl-paeoniflorin_qt	Aryl hydrocarbon receptor	P35869	AHR	0.033
Radix Paeoniae（RP）	MOL007012	4-o-methyl-paeoniflorin_qt	Dihydroorotate dehydrogenase, mitochondrial	Q02127	DHODH	0.033
Radix Paeoniae（RP）	MOL007012	4-o-methyl-paeoniflorin_qt	Potassium channel subfamily K member 6	Q9Y257	KCNK6	0.033
Radix Paeoniae（RP）	MOL007012	4-o-methyl-paeoniflorin_qt	Neuronal acetylcholine receptor subunit alpha-2	Q15822	CHRNA2	0.035
Radix Paeoniae（RP）	MOL007012	4-o-methyl-paeoniflorin_qt	Beta-nerve growth factor	P01138	NGF	0.037
Radix Paeoniae（RP）	MOL007012	4-o-methyl-paeoniflorin_qt	Beta-1 adrenergic receptor	P08588	ADRB1	0.037
Radix Paeoniae（RP）	MOL007012	4-o-methyl-paeoniflorin_qt	Beta-3 adrenergic receptor	P13945	ADRB3	0.037

Radix Paeoniae（RP）	MOL007012	4-o-methyl-paeoniflorin_qt	Nociceptin receptor	P41146	OPRL1	0.038
Radix Paeoniae（RP）	MOL007012	4-o-methyl-paeoniflorin_qt	Glycine receptor subunit alpha-1	P23415	GLRA1	0.039
Radix Paeoniae（RP）	MOL007012	4-o-methyl-paeoniflorin_qt	ATP synthase delta chain, mitochondrial	P30049	ATP5F1D	0.039
Radix Paeoniae（RP）	MOL007012	4-o-methyl-paeoniflorin_qt	Glutamate receptor 1	P42261	GRIA1	0.039
Radix Paeoniae（RP）	MOL007012	4-o-methyl-paeoniflorin_qt	Calcium-transporting ATPase type 2C member 1	P98194	ATP2C1	0.039
Radix Paeoniae（RP）	MOL007012	4-o-methyl-paeoniflorin_qt	Potassium voltage-gated channel subfamily A member 1	Q09470	KCNA1	0.039
Radix Paeoniae（RP）	MOL007012	4-o-methyl-paeoniflorin_qt	Methionine aminopeptidase 1	P53582	METAP1	0.048
Radix Paeoniae（RP）	MOL007012	4-o-methyl-paeoniflorin_qt	RAC-beta serine/threonine-protein kinase	P31751	AKT2	0.05
Radix Paeoniae（RP）	MOL007012	4-o-methyl-paeoniflorin_qt	cAMP-dependent protein kinase inhibitor alpha	P61925	PKIA	0.05
Radix Paeoniae（RP）	MOL007012	4-o-methyl-paeoniflorin_qt	Carbonic anhydrase 1	P00915	CA1	0.055
Radix Paeoniae（RP）	MOL007012	4-o-methyl-paeoniflorin_qt	Progesterone receptor	P06401	PGR	0.055
Radix Paeoniae（RP）	MOL007012	4-o-methyl-paeoniflorin_qt	Corticosteroid 11-beta-dehydrogenase isozyme 1	P28845	HSD11B1	0.055
Radix Paeoniae（RP）	MOL007012	4-o-methyl-paeoniflorin_qt	Sodium/potassium-transporting ATPase gamma chain	P54710	FXYD2	0.055
Radix Paeoniae（RP）	MOL007012	4-o-methyl-paeoniflorin_qt	Platelet glycoprotein IX	P14770	GP9	0.069
Radix Paeoniae（RP）	MOL007012	4-o-methyl-paeoniflorin_qt	Gamma-aminobutyric-acid receptor subunit alpha-1	P14867	GABRA1	0.069
Radix Paeoniae（RP）	MOL007012	4-o-methyl-paeoniflorin_qt	Muscarinic acetylcholine receptor M2	P08172	CHRM2	0.074
Radix Paeoniae（RP）	MOL007012	4-o-methyl-paeoniflorin_qt	Muscarinic acetylcholine receptor M4	P08173	CHRM4	0.074
Radix Paeoniae（RP）	MOL007012	4-o-methyl-paeoniflorin_qt	Integrin alpha-L	P20701	ITGAL	0.074
Radix Paeoniae（RP）	MOL007012	4-o-methyl-paeoniflorin_qt	Retinoic acid receptor alpha	P10276	RARA	0.079
Radix Paeoniae（RP）	MOL007012	4-o-methyl-paeoniflorin_qt	Sodium channel protein type 5 subunit alpha	Q14524	SCN5A	0.085
Radix Paeoniae（RP）	MOL007012	4-o-methyl-paeoniflorin_qt	Beta-2 adrenergic receptor	P07550	ADRB2	0.094
Radix Paeoniae（RP）	MOL007012	4-o-methyl-paeoniflorin_qt	Glutamate [NMDA] receptor subunit 3A	Q8TCU5	GRIN3A	0.094
Radix Paeoniae（RP）	MOL007012	4-o-methyl-paeoniflorin_qt	Tripartite motif-containing protein 13	O60858	TRIM13	0.097
Radix Paeoniae（RP）	MOL007012	4-o-methyl-paeoniflorin_qt	Nuclear receptor coactivator 1	Q15788	NCOA1	0.098
Radix Paeoniae（RP）	MOL007012	4-o-methyl-paeoniflorin_qt	Aldo-keto reductase family 1 member C3	P42330	AKR1C3	0.106
Radix Paeoniae（RP）	MOL007012	4-o-methyl-paeoniflorin_qt	Histamine H1 receptor	P35367	HRH1	0.117
Radix Paeoniae（RP）	MOL007012	4-o-methyl-paeoniflorin_qt	Solute carrier family 12 member 1	Q13621	SLC12A1	0.12
Radix Paeoniae（RP）	MOL007012	4-o-methyl-paeoniflorin_qt	cAMP-dependent protein kinase catalytic subunit alpha	P17612	PRKACA	0.121
Radix Paeoniae（RP）	MOL007012	4-o-methyl-paeoniflorin_qt	Tubulin alpha-3 chain	Q71U36	TUBA1A	0.121
Radix Paeoniae（RP）	MOL007012	4-o-methyl-paeoniflorin_qt	Glycogen phosphorylase, muscle form	P11217	PYGM	0.125
Radix Paeoniae（RP）	MOL007012	4-o-methyl-paeoniflorin_qt	Proto-oncogene tyrosine-protein kinase Src	P12931	SRC	0.125
Radix Paeoniae（RP）	MOL007012	4-o-methyl-paeoniflorin_qt	Peroxisome proliferator-activated receptor gamma	P37231	PPARG	0.128
Radix Paeoniae（RP）	MOL007012	4-o-methyl-paeoniflorin_qt	Nuclear receptor coactivator 2	Q15596	NCOA2	0.128
Radix Paeoniae（RP）	MOL007012	4-o-methyl-paeoniflorin_qt	Glucocorticoid receptor	P04150	NR3C1	0.129
Radix Paeoniae（RP）	MOL007012	4-o-methyl-paeoniflorin_qt	Trypsin-1	P07477	PRSS1	0.129
Radix Paeoniae（RP）	MOL007012	4-o-methyl-paeoniflorin_qt	Mineralocorticoid receptor	P08235	NR3C2	0.13
Radix Paeoniae（RP）	MOL007012	4-o-methyl-paeoniflorin_qt	Peroxisome proliferator-activated receptor alpha	Q07869	PPARA	0.13
Radix Paeoniae（RP）	MOL007012	4-o-methyl-paeoniflorin_qt	Carbonic anhydrase 2	P00918	CA2	0.131
Radix Paeoniae（RP）	MOL007012	4-o-methyl-paeoniflorin_qt	Potassium voltage-gated channel subfamily KQT member 1	P51787	KCNQ1	0.133
Radix Paeoniae（RP）	MOL007012	4-o-methyl-paeoniflorin_qt	Liver carboxylesterase 1	P23141	CES1	0.138
Radix Paeoniae（RP）	MOL007012	4-o-methyl-paeoniflorin_qt	Muscarinic acetylcholine receptor M3	P20309	CHRM3	0.167
Radix Paeoniae（RP）	MOL007012	4-o-methyl-paeoniflorin_qt	3-hydroxy-3-methylglutaryl-coenzyme A reductase	P04035	HMGCR	0.169
Radix Paeoniae（RP）	MOL007012	4-o-methyl-paeoniflorin_qt	Delta-type opioid receptor	P41143	OPRD1	0.213
Radix Paeoniae（RP）	MOL007012	4-o-methyl-paeoniflorin_qt	Muscarinic acetylcholine receptor M1	P11229	CHRM1	0.261
Radix Paeoniae（RP）	MOL007012	4-o-methyl-paeoniflorin_qt	Cell division protein kinase 2	P24941	CDK2	0.27
Radix Paeoniae（RP）	MOL007012	4-o-methyl-paeoniflorin_qt	Cyclin-A2	P20248	CCNA2	0.275
Radix Paeoniae（RP）	MOL007012	4-o-methyl-paeoniflorin_qt	Mu-type opioid receptor	P35372	OPRM1	0.447
Radix Paeoniae（RP）	MOL007012	4-o-methyl-paeoniflorin_qt	Kappa-type opioid receptor	P41145	OPRK1	0.447
Radix Paeoniae（RP）	MOL007012	4-o-methyl-paeoniflorin_qt	Prostaglandin G/H synthase 1	P23219	PTGS1	0.476
Radix Paeoniae（RP）	MOL007012	4-o-methyl-paeoniflorin_qt	Estrogen receptor beta	Q92731	ESR2	0.569
Radix Paeoniae（RP）	MOL007012	4-o-methyl-paeoniflorin_qt	Prostaglandin G/H synthase 2	P35354	PTGS2	1
Radix Paeoniae（RP）	MOL007014	8-debenzoylpaeonidanin	Nuclear receptor coactivator 2	Q15596	NCOA2	0.355
Radix Paeoniae（RP）	MOL007014	8-debenzoylpaeonidanin	Estrogen receptor	P03372	ESR1	1

Radix Paeoniae（RP）	MOL007016	Paeoniflorigenone	Peptide deformylase, mitochondrial	Q9HBH1	PDF	0.003
Radix Paeoniae（RP）	MOL007016	Paeoniflorigenone	Acetylcholine receptor subunit alpha	P02708	CHRNA1	0.005
Radix Paeoniae（RP）	MOL007016	Paeoniflorigenone	Cholinesterase	P06276	BCHE	0.005
Radix Paeoniae（RP）	MOL007016	Paeoniflorigenone	Acetylcholine receptor subunit gamma	P07510	CHRNG	0.005
Radix Paeoniae（RP）	MOL007016	Paeoniflorigenone	Acetylcholine receptor subunit beta	P11230	CHRNB1	0.005
Radix Paeoniae（RP）	MOL007016	Paeoniflorigenone	Neuronal acetylcholine receptor subunit beta-2	P17787	CHRNB2	0.005
Radix Paeoniae（RP）	MOL007016	Paeoniflorigenone	Acetylcholinesterase	P22303	ACHE	0.005
Radix Paeoniae（RP）	MOL007016	Paeoniflorigenone	Neuronal acetylcholine receptor subunit alpha-5	P30532	CHRNA5	0.005
Radix Paeoniae（RP）	MOL007016	Paeoniflorigenone	Neuronal acetylcholine receptor subunit beta-4	P30926	CHRNB4	0.005
Radix Paeoniae（RP）	MOL007016	Paeoniflorigenone	Neuronal acetylcholine receptor subunit alpha-3	P32297	CHRNA3	0.005
Radix Paeoniae（RP）	MOL007016	Paeoniflorigenone	Neuronal acetylcholine receptor subunit alpha-7	P36544	CHRNA7	0.005
Radix Paeoniae（RP）	MOL007016	Paeoniflorigenone	Acetylcholine receptor subunit epsilon	Q04844	CHRNE	0.005
Radix Paeoniae（RP）	MOL007016	Paeoniflorigenone	Neuronal acetylcholine receptor subunit beta-3	Q05901	CHRNB3	0.005
Radix Paeoniae（RP）	MOL007016	Paeoniflorigenone	Acetylcholine receptor subunit delta	Q07001	CHRND	0.005
Radix Paeoniae（RP）	MOL007016	Paeoniflorigenone	Neuronal acetylcholine receptor subunit alpha-2	Q15822	CHRNA2	0.005
Radix Paeoniae（RP）	MOL007016	Paeoniflorigenone	Neuronal acetylcholine receptor subunit alpha-6	Q15825	KCNJ8	0.005
Radix Paeoniae（RP）	MOL007016	Paeoniflorigenone	Thiosulfate sulfurtransferase	Q16762	TST	0.005
Radix Paeoniae（RP）	MOL007016	Paeoniflorigenone	Neuronal acetylcholine receptor subunit alpha-9	Q9UGM1	CHRNA9	0.005
Radix Paeoniae（RP）	MOL007016	Paeoniflorigenone	Cytochrome b-c1 complex subunit 8	O14949	UQCRQ	0.007
Radix Paeoniae（RP）	MOL007016	Paeoniflorigenone	Cytochrome b-c1 complex subunit 10	O14957	UQCR11	0.007
Radix Paeoniae（RP）	MOL007016	Paeoniflorigenone	Cytochrome b	P00156	MT-CYB	0.007
Radix Paeoniae（RP）	MOL007016	Paeoniflorigenone	Cytochrome b-c1 complex subunit 6, mitochondrial	P07919	UQCRH	0.007
Radix Paeoniae（RP）	MOL007016	Paeoniflorigenone	Cytochrome c1, heme protein, mitochondrial	P08574	CYC1	0.007
Radix Paeoniae（RP）	MOL007016	Paeoniflorigenone	Cytochrome b-c1 complex subunit 2, mitochondrial	P22695	UQCRC2	0.007
Radix Paeoniae（RP）	MOL007016	Paeoniflorigenone	Ubiquinol-cytochrome-c reductase complex core protein 1, mitochondrial	P31930	UQCRC1	0.007
Radix Paeoniae（RP）	MOL007016	Paeoniflorigenone	Cytochrome b-c1 complex subunit Rieske, mitochondrial	P47985	UQCRFS1	0.007
Radix Paeoniae（RP）	MOL007016	Paeoniflorigenone	Cytochrome b-c1 complex subunit 9	Q9UDW1	UQCR10	0.007
Radix Paeoniae（RP）	MOL007016	Paeoniflorigenone	D(1A) dopamine receptor	P21728	DRD1	0.021
Radix Paeoniae（RP）	MOL007016	Paeoniflorigenone	D(1B) dopamine receptor	P21918	DRD5	0.021
Radix Paeoniae（RP）	MOL007016	Paeoniflorigenone	Gamma-aminobutyric-acid receptor subunit beta-3	P28472	GABRB3	0.021
Radix Paeoniae（RP）	MOL007016	Paeoniflorigenone	Gamma-aminobutyric-acid receptor subunit beta-2	P47870	GABRB2	0.021
Radix Paeoniae（RP）	MOL007016	Paeoniflorigenone	Sodium/potassium-transporting ATPase gamma chain	P54710	FXYD2	0.021
Radix Paeoniae（RP）	MOL007016	Paeoniflorigenone	Voltage-dependent T-type calcium channel subunit alpha-1G	O43497	CACNA1G	0.022
Radix Paeoniae（RP）	MOL007016	Paeoniflorigenone	Carbonic anhydrase 12	O43570	CA12	0.022
Radix Paeoniae（RP）	MOL007016	Paeoniflorigenone	Carbonic anhydrase-related protein 11	O75493	CA11	0.022
Radix Paeoniae（RP）	MOL007016	Paeoniflorigenone	Voltage-dependent T-type calcium channel subunit alpha-1H	O95180	CACNA1H	0.022
Radix Paeoniae（RP）	MOL007016	Paeoniflorigenone	Carbonic anhydrase 3	P07451	CA3	0.022
Radix Paeoniae（RP）	MOL007016	Paeoniflorigenone	Carbonic anhydrase 6	P23280	CA6	0.022
Radix Paeoniae（RP）	MOL007016	Paeoniflorigenone	Amine oxidase [flavin-containing] B	P27338	MAOB	0.022
Radix Paeoniae（RP）	MOL007016	Paeoniflorigenone	Carbonic anhydrase 5A, mitochondrial	P35218	CA5A	0.022
Radix Paeoniae（RP）	MOL007016	Paeoniflorigenone	Carbonic anhydrase-related protein	P35219	CA8	0.022
Radix Paeoniae（RP）	MOL007016	Paeoniflorigenone	Sodium channel protein type 1 subunit alpha	P35498	SCN1A	0.022
Radix Paeoniae（RP）	MOL007016	Paeoniflorigenone	Carbonic anhydrase 7	P43166	CA7	0.022
Radix Paeoniae（RP）	MOL007016	Paeoniflorigenone	Sodium channel subunit beta-1	Q07699	SCN1B	0.022
Radix Paeoniae（RP）	MOL007016	Paeoniflorigenone	Sodium channel protein type 9 subunit alpha	Q15858	SCN9A	0.022
Radix Paeoniae（RP）	MOL007016	Paeoniflorigenone	Carbonic anhydrase 9	Q16790	CA9	0.022
Radix Paeoniae（RP）	MOL007016	Paeoniflorigenone	Sodium channel subunit beta-4	Q8IWT1	SCN4B	0.022
Radix Paeoniae（RP）	MOL007016	Paeoniflorigenone	Carbonic anhydrase 13	Q8N1Q1	CA13	0.022
Radix Paeoniae（RP）	MOL007016	Paeoniflorigenone	Sodium channel protein type 2 subunit alpha	Q99250	SCN2A	0.022
Radix Paeoniae（RP）	MOL007016	Paeoniflorigenone	Carbonic anhydrase-related protein 10	Q9NS85	CA10	0.022
Radix Paeoniae（RP）	MOL007016	Paeoniflorigenone	Sodium channel protein type 3 subunit alpha	Q9NY46	SCN3A	0.022
Radix Paeoniae（RP）	MOL007016	Paeoniflorigenone	Sodium channel subunit beta-3	Q9NY72	SCN3B	0.022
Radix Paeoniae（RP）	MOL007016	Paeoniflorigenone	Voltage-dependent T-type calcium channel subunit alpha-1I	Q9P0X4	CACNA1I	0.022

Radix Paeoniae（RP）	MOL007016	Paeoniflorigenone	Carbonic anhydrase 14	Q9ULX7	CA14	0.022
Radix Paeoniae（RP）	MOL007016	Paeoniflorigenone	Carbonic anhydrase 5B, mitochondrial	Q9Y2D0	CA5B	0.022
Radix Paeoniae（RP）	MOL007016	Paeoniflorigenone	5-hydroxytryptamine 2C receptor	P28335	HTR2C	0.023
Radix Paeoniae（RP）	MOL007016	Paeoniflorigenone	Calmodulin	P62158		0.023
Radix Paeoniae（RP）	MOL007016	Paeoniflorigenone	Phenylalanine-4-hydroxylase	P00439	PAH	0.024
Radix Paeoniae（RP）	MOL007016	Paeoniflorigenone	Alpha-1D adrenergic receptor	P25100	ADRA1D	0.024
Radix Paeoniae（RP）	MOL007016	Paeoniflorigenone	5-hydroxytryptamine 1E receptor	P28566	HTR1E	0.024
Radix Paeoniae（RP）	MOL007016	Paeoniflorigenone	5-hydroxytryptamine 1F receptor	P30939	HTR1F	0.024
Radix Paeoniae（RP）	MOL007016	Paeoniflorigenone	Alpha-1B adrenergic receptor	P35368	ADRA1B	0.024
Radix Paeoniae（RP）	MOL007016	Paeoniflorigenone	5-hydroxytryptamine 2B receptor	P41595	HTR2B	0.024
Radix Paeoniae（RP）	MOL007016	Paeoniflorigenone	Phosphatidylinositol 3-kinase regulatory subunit beta	O00459	PIK3R2	0.025
Radix Paeoniae（RP）	MOL007016	Paeoniflorigenone	Beta-3 adrenergic receptor	P13945	ADRB3	0.025
Radix Paeoniae（RP）	MOL007016	Paeoniflorigenone	Phosphatidylinositol 3-kinase regulatory subunit alpha	P27986	PIK3R1	0.025
Radix Paeoniae（RP）	MOL007016	Paeoniflorigenone	Mitogen-activated protein kinase 1	P28482	MAPK1	0.025
Radix Paeoniae（RP）	MOL007016	Paeoniflorigenone	Potassium channel subfamily K member 6	Q9Y257	KCNK6	0.035
Radix Paeoniae（RP）	MOL007016	Paeoniflorigenone	Gamma-aminobutyric-acid receptor subunit alpha-1	P14867	GABRA1	0.036
Radix Paeoniae（RP）	MOL007016	Paeoniflorigenone	Gamma-aminobutyric-acid receptor subunit alpha-2	P47869	GABRA2	0.036
Radix Paeoniae（RP）	MOL007016	Paeoniflorigenone	5-hydroxytryptamine 4 receptor	Q13639	HTR4	0.036
Radix Paeoniae（RP）	MOL007016	Paeoniflorigenone	Voltage-dependent calcium channel subunit alpha-2/delta-1	P54289	CACNA2D1	0.039
Radix Paeoniae（RP）	MOL007016	Paeoniflorigenone	Voltage-dependent N-type calcium channel subunit alpha-1B	Q00975	CACNA1B	0.039
Radix Paeoniae（RP）	MOL007016	Paeoniflorigenone	Voltage-dependent calcium channel subunit alpha-2/delta-2	Q9NY47	CACNA2D2	0.039
Radix Paeoniae（RP）	MOL007016	Paeoniflorigenone	Tyrosine 3-monooxygenase	P07101	TH	0.042
Radix Paeoniae（RP）	MOL007016	Paeoniflorigenone	Tyrosyl-tRNA synthetase, mitochondrial	Q9Y2Z4	YARS2	0.042
Radix Paeoniae（RP）	MOL007016	Paeoniflorigenone	Thymidylate synthase	P04818	TYMS	0.043
Radix Paeoniae（RP）	MOL007016	Paeoniflorigenone	Stromelysin-1	P08254	MMP3	0.044
Radix Paeoniae（RP）	MOL007016	Paeoniflorigenone	Stromelysin-2	P09238	MMP10	0.044
Radix Paeoniae（RP）	MOL007016	Paeoniflorigenone	Macrophage metalloelastase	P39900	MMP12	0.044
Radix Paeoniae（RP）	MOL007016	Paeoniflorigenone	Solute carrier family 22 member 6	Q4U2R8	SLC22A6	0.044
Radix Paeoniae（RP）	MOL007016	Paeoniflorigenone	Solute carrier family 22 member 8	Q8TCC7	SLC22A8	0.044
Radix Paeoniae（RP）	MOL007016	Paeoniflorigenone	Neuronal acetylcholine receptor subunit alpha-10	Q9GZZ6	CHRNA10	0.044
Radix Paeoniae（RP）	MOL007016	Paeoniflorigenone	Solute carrier family 22 member 11	Q9NSA0	SLC22A11	0.044
Radix Paeoniae（RP）	MOL007016	Paeoniflorigenone	Tumor necrosis factor	P01375	TNF	0.046
Radix Paeoniae（RP）	MOL007016	Paeoniflorigenone	Nuclear factor NF-kappa-B p105 subunit	P19838	NFKB1	0.046
Radix Paeoniae（RP）	MOL007016	Paeoniflorigenone	Vascular endothelial growth factor	A2A2V4	VEGFA	0.047
Radix Paeoniae（RP）	MOL007016	Paeoniflorigenone	Tumor necrosis factor ligand superfamily member 11	O14788	TNFSF11	0.047
Radix Paeoniae（RP）	MOL007016	Paeoniflorigenone	Cadherin-5	P33151	CDH5	0.047
Radix Paeoniae（RP）	MOL007016	Paeoniflorigenone	Muscarinic acetylcholine receptor M5	P08912	CHRM5	0.049
Radix Paeoniae（RP）	MOL007016	Paeoniflorigenone	Delta-type opioid receptor	P41143	OPRD1	0.051
Radix Paeoniae（RP）	MOL007016	Paeoniflorigenone	Amine oxidase [flavin-containing] A	P21397	MAOA	0.056
Radix Paeoniae（RP）	MOL007016	Paeoniflorigenone	Carbonic anhydrase 4	P22748	CA4	0.056
Radix Paeoniae（RP）	MOL007016	Paeoniflorigenone	Sodium channel protein type 4 subunit alpha	P35499	SCN4A	0.056
Radix Paeoniae（RP）	MOL007016	Paeoniflorigenone	D(2) dopamine receptor	P14416	DRD2	0.057
Radix Paeoniae（RP）	MOL007016	Paeoniflorigenone	D(3) dopamine receptor	P35462	DRD3	0.057
Radix Paeoniae（RP）	MOL007016	Paeoniflorigenone	5-hydroxytryptamine 1D receptor	P28221	HTR1D	0.06
Radix Paeoniae（RP）	MOL007016	Paeoniflorigenone	5-hydroxytryptamine 1B receptor	P28222	HTR1B	0.06
Radix Paeoniae（RP）	MOL007016	Paeoniflorigenone	cAMP-dependent protein kinase inhibitor alpha	P61925	PKIA	0.06
Radix Paeoniae（RP）	MOL007016	Paeoniflorigenone	Peroxiredoxin-5, mitochondrial	P30044	PRDX5	0.061
Radix Paeoniae（RP）	MOL007016	Paeoniflorigenone	Serine/threonine-protein phosphatase 2A catalytic subunit alpha isoform	P67775	PPP2CA	0.068
Radix Paeoniae（RP）	MOL007016	Paeoniflorigenone	Serine/threonine-protein phosphatase 2A 56 kDa regulatory subunit gamma isoform	Q13362	PPP2R5C	0.068
Radix Paeoniae（RP）	MOL007016	Paeoniflorigenone	cAMP-specific 3',5'-cyclic phosphodiesterase 4A	P27815	PDE4A	0.069
Radix Paeoniae（RP）	MOL007016	Paeoniflorigenone	cAMP-specific 3',5'-cyclic phosphodiesterase 4B	Q07343	PDE4B	0.069
Radix Paeoniae（RP）	MOL007016	Paeoniflorigenone	Prothrombin	P00734	F2	0.07

Radix Paeoniae（RP）	MOL007016	Paeoniflorigenone	Macrophage migration inhibitory factor	P14174	MIF	0.07
Radix Paeoniae（RP）	MOL007016	Paeoniflorigenone	Glutamate [NMDA] receptor subunit 3A	Q8TCU5	GRIN3A	0.07
Radix Paeoniae（RP）	MOL007016	Paeoniflorigenone	Sodium channel protein type 11 subunit alpha	Q9UI33	SCN11A	0.07
Radix Paeoniae（RP）	MOL007016	Paeoniflorigenone	Progesterone receptor	P06401	PGR	0.075
Radix Paeoniae（RP）	MOL007016	Paeoniflorigenone	Mineralocorticoid receptor	P08235	NR3C2	0.075
Radix Paeoniae（RP）	MOL007016	Paeoniflorigenone	Peroxisome proliferator-activated receptor gamma	P37231	PPARG	0.079
Radix Paeoniae（RP）	MOL007016	Paeoniflorigenone	Sodium-dependent dopamine transporter	Q01959	SLC6A3	0.079
Radix Paeoniae（RP）	MOL007016	Paeoniflorigenone	Nuclear receptor coactivator 2	Q15596	NCOA2	0.079
Radix Paeoniae（RP）	MOL007016	Paeoniflorigenone	Ribosyldihydronicotinamide dehydrogenase [quinone]	P16083	NQO2	0.082
Radix Paeoniae（RP）	MOL007016	Paeoniflorigenone	Sodium-dependent noradrenaline transporter	P23975	SLC6A2	0.083
Radix Paeoniae（RP）	MOL007016	Paeoniflorigenone	Sodium-dependent serotonin transporter	P31645	SLC6A4	0.083
Radix Paeoniae（RP）	MOL007016	Paeoniflorigenone	Potassium channel subfamily K member 1	O00180	KCNK1	0.084
Radix Paeoniae（RP）	MOL007016	Paeoniflorigenone	Carbonic anhydrase 1	P00915	CA1	0.09
Radix Paeoniae（RP）	MOL007016	Paeoniflorigenone	Platelet glycoprotein IX	P14770	GP9	0.091
Radix Paeoniae（RP）	MOL007016	Paeoniflorigenone	Alpha-2A adrenergic receptor	P08913	ADRA2A	0.095
Radix Paeoniae（RP）	MOL007016	Paeoniflorigenone	Alpha-2B adrenergic receptor	P18089	ADRA2B	0.095
Radix Paeoniae（RP）	MOL007016	Paeoniflorigenone	Alpha-2C adrenergic receptor	P18825	ADRA2C	0.095
Radix Paeoniae（RP）	MOL007016	Paeoniflorigenone	Alpha-1A adrenergic receptor	P35348	ADRA1A	0.095
Radix Paeoniae（RP）	MOL007016	Paeoniflorigenone	Tyrosyl-tRNA synthetase, cytoplasmic	P54577	YARS	0.096
Radix Paeoniae（RP）	MOL007016	Paeoniflorigenone	Beta-1 adrenergic receptor	P08588	ADRB1	0.099
Radix Paeoniae（RP）	MOL007016	Paeoniflorigenone	DNA topoisomerase 2-alpha	P11388	TOP2A	0.104
Radix Paeoniae（RP）	MOL007016	Paeoniflorigenone	Protein tyrosine kinase 2 beta	Q14289	PTK2B	0.104
Radix Paeoniae（RP）	MOL007016	Paeoniflorigenone	Fibroblast growth factor receptor 2	P21802	FGFR2	0.105
Radix Paeoniae（RP）	MOL007016	Paeoniflorigenone	Platelet-activating factor acetylhydrolase IB subunit gamma	Q15102	PAFAH1B3	0.105
Radix Paeoniae（RP）	MOL007016	Paeoniflorigenone	Cell division control protein 2 homolog	P06493	CDK1	0.108
Radix Paeoniae（RP）	MOL007016	Paeoniflorigenone	5-hydroxytryptamine 1A receptor	P08908	HTR1A	0.108
Radix Paeoniae（RP）	MOL007016	Paeoniflorigenone	Cell division protein kinase 5	Q00535	CDK5	0.108
Radix Paeoniae（RP）	MOL007016	Paeoniflorigenone	Muscarinic acetylcholine receptor M3	P20309	CHRM3	0.114
Radix Paeoniae（RP）	MOL007016	Paeoniflorigenone	Sodium channel protein type 10 subunit alpha	Q9Y5Y9	SCN10A	0.118
Radix Paeoniae（RP）	MOL007016	Paeoniflorigenone	5-hydroxytryptamine 3 receptor	P46098	HTR3A	0.119
Radix Paeoniae（RP）	MOL007016	Paeoniflorigenone	Carbonic anhydrase 2	P00918	CA2	0.123
Radix Paeoniae（RP）	MOL007016	Paeoniflorigenone	cAMP-dependent protein kinase catalytic subunit alpha	P17612	PRKACA	0.133
Radix Paeoniae（RP）	MOL007016	Paeoniflorigenone	Beta-2 adrenergic receptor	P07550	ADRB2	0.136
Radix Paeoniae（RP）	MOL007016	Paeoniflorigenone	Cannabinoid receptor 2	P34972	CNR2	0.14
Radix Paeoniae（RP）	MOL007016	Paeoniflorigenone	ATP-sensitive inward rectifier potassium channel 1	P48048	KCNJ1	0.142
Radix Paeoniae（RP）	MOL007016	Paeoniflorigenone	Phosphatidylinositol-4,5-bisphosphate 3-kinase catalytic subunit gamma isoform	P48736	PIK3CG	0.142
Radix Paeoniae（RP）	MOL007016	Paeoniflorigenone	Vesicle-fusing ATPase	P46459	NSF	0.143
Radix Paeoniae（RP）	MOL007016	Paeoniflorigenone	Dual specificity tyrosine-phosphorylation-regulated kinase 1A	Q13627	DYRK1A	0.144
Radix Paeoniae（RP）	MOL007016	Paeoniflorigenone	Mitogen-activated protein kinase 14	Q16539	MAPK14	0.144
Radix Paeoniae（RP）	MOL007016	Paeoniflorigenone	Geranylgeranyl pyrophosphate synthetase	O95749	GGPS1	0.145
Radix Paeoniae（RP）	MOL007016	Paeoniflorigenone	Solute carrier family 12 member 1	Q13621	SLC12A1	0.146
Radix Paeoniae（RP）	MOL007016	Paeoniflorigenone	Neuropeptide Y	P01303	NPY	0.151
Radix Paeoniae（RP）	MOL007016	Paeoniflorigenone	Leukotriene A-4 hydrolase	P09960	LTA4H	0.152
Radix Paeoniae（RP）	MOL007016	Paeoniflorigenone	Serine/threonine-protein kinase 6	O14965	AURKA	0.161
Radix Paeoniae（RP）	MOL007016	Paeoniflorigenone	Heat shock protein HSP 90-beta	P08238	HSP90AB1	0.161
Radix Paeoniae（RP）	MOL007016	Paeoniflorigenone	C-jun-amino-terminal kinase-interacting protein 1	Q9UQF2	MAPK8IP1	0.164
Radix Paeoniae（RP）	MOL007016	Paeoniflorigenone	Glycogen phosphorylase, muscle form	P11217	PYGM	0.165
Radix Paeoniae（RP）	MOL007016	Paeoniflorigenone	Hemoglobin subunit alpha	P69905	HBA1	0.168
Radix Paeoniae（RP）	MOL007016	Paeoniflorigenone	Prolyl endopeptidase	P48147	PREP	0.171
Radix Paeoniae（RP）	MOL007016	Paeoniflorigenone	Muscarinic acetylcholine receptor M2	P08172	CHRM2	0.173
Radix Paeoniae（RP）	MOL007016	Paeoniflorigenone	Muscarinic acetylcholine receptor M4	P08173	CHRM4	0.173
Radix Paeoniae（RP）	MOL007016	Paeoniflorigenone	Casein kinase II subunit alpha	P68400	CSNK2A1	0.174
Radix Paeoniae（RP）	MOL007016	Paeoniflorigenone	Nuclear receptor coactivator 1	Q15788	NCOA1	0.22

Radix Paeoniae（RP）	MOL007016	Paeoniflorigenone	Liver carboxylesterase 1	P23141	CES1	0.223
Radix Paeoniae（RP）	MOL007016	Paeoniflorigenone	Sodium channel protein type 5 subunit alpha	Q14524	SCN5A	0.256
Radix Paeoniae（RP）	MOL007016	Paeoniflorigenone	Prostaglandin G/H synthase 1	P23219	PTGS1	0.273
Radix Paeoniae（RP）	MOL007016	Paeoniflorigenone	Muscarinic acetylcholine receptor M1	P11229	CHRM1	0.279
Radix Paeoniae（RP）	MOL007016	Paeoniflorigenone	Trypsin-1	P07477	PRSS1	0.28
Radix Paeoniae（RP）	MOL007016	Paeoniflorigenone	Kappa-type opioid receptor	P41145	OPRK1	0.301
Radix Paeoniae（RP）	MOL007016	Paeoniflorigenone	Mu-type opioid receptor	P35372	OPRM1	0.41
Radix Paeoniae（RP）	MOL007016	Paeoniflorigenone	Estrogen receptor beta	Q92731	ESR2	0.454
Radix Paeoniae（RP）	MOL007016	Paeoniflorigenone	Estrogen receptor	P03372	ESR1	0.574
Radix Paeoniae（RP）	MOL007016	Paeoniflorigenone	Cell division protein kinase 2	P24941	CDK2	0.59
Radix Paeoniae（RP）	MOL007016	Paeoniflorigenone	Prostaglandin G/H synthase 2	P35354	PTGS2	0.704
Radix Paeoniae（RP）	MOL007016	Paeoniflorigenone	Cyclin-A2	P20248	CCNA2	1
Radix Paeoniae（RP）	MOL007018	9-ethyl-neo-paeoniaflorin A_qt	Cytochrome b-c1 complex subunit 8	O14949	UQCRQ	0.01
Radix Paeoniae（RP）	MOL007018	9-ethyl-neo-paeoniaflorin A_qt	Cytochrome b-c1 complex subunit 10	O14957	UQCR11	0.01
Radix Paeoniae（RP）	MOL007018	9-ethyl-neo-paeoniaflorin A_qt	Cytochrome b	P00156	MT-CYB	0.01
Radix Paeoniae（RP）	MOL007018	9-ethyl-neo-paeoniaflorin A_qt	Cytochrome b-c1 complex subunit 6, mitochondrial	P07919	UQCRH	0.01
Radix Paeoniae（RP）	MOL007018	9-ethyl-neo-paeoniaflorin A_qt	Cytochrome c1, heme protein, mitochondrial	P08574	CYC1	0.01
Radix Paeoniae（RP）	MOL007018	9-ethyl-neo-paeoniaflorin A_qt	Cytochrome b-c1 complex subunit 2, mitochondrial	P22695	UQCRC2	0.01
Radix Paeoniae（RP）	MOL007018	9-ethyl-neo-paeoniaflorin A_qt	Ubiquinol-cytochrome-c reductase complex core protein 1, mitochondrial	P31930	UQCRC1	0.01
Radix Paeoniae（RP）	MOL007018	9-ethyl-neo-paeoniaflorin A_qt	Cytochrome b-c1 complex subunit Rieske, mitochondrial	P47985	UQCRFS1	0.01
Radix Paeoniae（RP）	MOL007018	9-ethyl-neo-paeoniaflorin A_qt	Cytochrome b-c1 complex subunit 9	Q9UDW1	UQCR10	0.01
Radix Paeoniae（RP）	MOL007018	9-ethyl-neo-paeoniaflorin A_qt	Glutamate [NMDA] receptor subunit epsilon-4	O15399	GRIN2D	0.011
Radix Paeoniae（RP）	MOL007018	9-ethyl-neo-paeoniaflorin A_qt	Glutamate [NMDA] receptor subunit 3B	O60391	GRIN3B	0.011
Radix Paeoniae（RP）	MOL007018	9-ethyl-neo-paeoniaflorin A_qt	Glutamate [NMDA] receptor subunit epsilon-1	Q12879	GRIN2A	0.011
Radix Paeoniae（RP）	MOL007018	9-ethyl-neo-paeoniaflorin A_qt	Glutamate [NMDA] receptor subunit epsilon-2	Q13224	GRIN2B	0.011
Radix Paeoniae（RP）	MOL007018	9-ethyl-neo-paeoniaflorin A_qt	Glutamate [NMDA] receptor subunit epsilon-3	Q14957	GRIN2C	0.011
Radix Paeoniae（RP）	MOL007018	9-ethyl-neo-paeoniaflorin A_qt	Glutamate [NMDA] receptor subunit 3A	Q8TCU5	GRIN3A	0.011
Radix Paeoniae（RP）	MOL007018	9-ethyl-neo-paeoniaflorin A_qt	Actin-related protein 2/3 complex subunit 1B	O15143	ARPC1B	0.014
Radix Paeoniae（RP）	MOL007018	9-ethyl-neo-paeoniaflorin A_qt	Actin-related protein 2/3 complex subunit 2	O15144	ARPC2	0.014
Radix Paeoniae（RP）	MOL007018	9-ethyl-neo-paeoniaflorin A_qt	Actin-related protein 2/3 complex subunit 3	O15145	ARPC3	0.014
Radix Paeoniae（RP）	MOL007018	9-ethyl-neo-paeoniaflorin A_qt	Actin-related protein 2/3 complex subunit 4	P59998	ARPC4	0.014
Radix Paeoniae（RP）	MOL007018	9-ethyl-neo-paeoniaflorin A_qt	Actin-related protein 3	P61158	ACTR3	0.014
Radix Paeoniae（RP）	MOL007018	9-ethyl-neo-paeoniaflorin A_qt	Actin-related protein 2	P61160	ACTR2	0.014
Radix Paeoniae（RP）	MOL007018	9-ethyl-neo-paeoniaflorin A_qt	Beta-2 adrenergic receptor	P07550	ADRB2	0.018
Radix Paeoniae（RP）	MOL007018	9-ethyl-neo-paeoniaflorin A_qt	Beta-1 adrenergic receptor	P08588	ADRB1	0.018
Radix Paeoniae（RP）	MOL007018	9-ethyl-neo-paeoniaflorin A_qt	Retinoic acid receptor alpha	P10276	RARA	0.018
Radix Paeoniae（RP）	MOL007018	9-ethyl-neo-paeoniaflorin A_qt	Retinoic acid receptor beta	P10826	RARB	0.018
Radix Paeoniae（RP）	MOL007018	9-ethyl-neo-paeoniaflorin A_qt	Retinoic acid receptor gamma-1	P13631	RARG	0.018
Radix Paeoniae（RP）	MOL007018	9-ethyl-neo-paeoniaflorin A_qt	Retinoic acid receptor RXR-beta	P28702	RXRB	0.018
Radix Paeoniae（RP）	MOL007018	9-ethyl-neo-paeoniaflorin A_qt	5-hydroxytryptamine 1F receptor	P30939	HTR1F	0.018
Radix Paeoniae（RP）	MOL007018	9-ethyl-neo-paeoniaflorin A_qt	Retinoic acid receptor RXR-gamma	P48443	RXRG	0.018
Radix Paeoniae（RP）	MOL007018	9-ethyl-neo-paeoniaflorin A_qt	Muscarinic acetylcholine receptor M5	P08912	CHRM5	0.02
Radix Paeoniae（RP）	MOL007018	9-ethyl-neo-paeoniaflorin A_qt	5-hydroxytryptamine 7 receptor	P34969	HTR7	0.02
Radix Paeoniae（RP）	MOL007018	9-ethyl-neo-paeoniaflorin A_qt	Histamine H1 receptor	P35367	HRH1	0.02
Radix Paeoniae（RP）	MOL007018	9-ethyl-neo-paeoniaflorin A_qt	Alpha-1B adrenergic receptor	P35368	ADRA1B	0.02
Radix Paeoniae（RP）	MOL007018	9-ethyl-neo-paeoniaflorin A_qt	5-hydroxytryptamine 6 receptor	P50406	HTR6	0.02
Radix Paeoniae（RP）	MOL007018	9-ethyl-neo-paeoniaflorin A_qt	5-hydroxytryptamine 4 receptor	Q13639	HTR4	0.02
Radix Paeoniae（RP）	MOL007018	9-ethyl-neo-paeoniaflorin A_qt	Kappa-type opioid receptor	P41145	OPRK1	0.022
Radix Paeoniae（RP）	MOL007018	9-ethyl-neo-paeoniaflorin A_qt	Carbonic anhydrase 1	P00915	CA1	0.034
Radix Paeoniae（RP）	MOL007018	9-ethyl-neo-paeoniaflorin A_qt	Carbonic anhydrase 2	P00918	CA2	0.034
Radix Paeoniae（RP）	MOL007018	9-ethyl-neo-paeoniaflorin A_qt	Lactase-phlorizin hydrolase	P09848	LCT	0.034
Radix Paeoniae（RP）	MOL007018	9-ethyl-neo-paeoniaflorin A_qt	Sodium/potassium-transporting ATPase gamma chain	P54710	FXYD2	0.034
Radix Paeoniae（RP）	MOL007018	9-ethyl-neo-paeoniaflorin A_qt	D-HSCDK2	O75100	CA11	0.035

Radix Paeoniae（RP）	MOL007018	9-ethyl-neo-paeoniaflorin A_qt	Cell division control protein 2 homolog	P06493	CDK1	0.035
Radix Paeoniae（RP）	MOL007018	9-ethyl-neo-paeoniaflorin A_qt	Cell division protein kinase 5	Q00535	CDK5	0.035
Radix Paeoniae（RP）	MOL007018	9-ethyl-neo-paeoniaflorin A_qt	Cyclin-dependent kinase 5 activator 1	Q15078	CDK5R1	0.035
Radix Paeoniae（RP）	MOL007018	9-ethyl-neo-paeoniaflorin A_qt	5-hydroxytryptamine 1D receptor	P28221	HTR1D	0.047
Radix Paeoniae（RP）	MOL007018	9-ethyl-neo-paeoniaflorin A_qt	5-hydroxytryptamine 2B receptor	P41595	HTR2B	0.047
Radix Paeoniae（RP）	MOL007018	9-ethyl-neo-paeoniaflorin A_qt	5-hydroxytryptamine 2A receptor	P28223	HTR2A	0.048
Radix Paeoniae（RP）	MOL007018	9-ethyl-neo-paeoniaflorin A_qt	5-hydroxytryptamine 1E receptor	P28566	HTR1E	0.048
Radix Paeoniae（RP）	MOL007018	9-ethyl-neo-paeoniaflorin A_qt	Calmodulin	P62158		0.048
Radix Paeoniae（RP）	MOL007018	9-ethyl-neo-paeoniaflorin A_qt	D(1A) dopamine receptor	P21728	DRD1	0.049
Radix Paeoniae（RP）	MOL007018	9-ethyl-neo-paeoniaflorin A_qt	D(4) dopamine receptor	P21917	DRD4	0.049
Radix Paeoniae（RP）	MOL007018	9-ethyl-neo-paeoniaflorin A_qt	D(1B) dopamine receptor	P21918	DRD5	0.049
Radix Paeoniae（RP）	MOL007018	9-ethyl-neo-paeoniaflorin A_qt	Alpha-1A adrenergic receptor	P35348	ADRA1A	0.049
Radix Paeoniae（RP）	MOL007018	9-ethyl-neo-paeoniaflorin A_qt	5-hydroxytryptamine 3 receptor	P46098	HTR3A	0.05
Radix Paeoniae（RP）	MOL007018	9-ethyl-neo-paeoniaflorin A_qt	Gamma-aminobutyric-acid receptor subunit alpha-1	P14867	GABRA1	0.051
Radix Paeoniae（RP）	MOL007018	9-ethyl-neo-paeoniaflorin A_qt	Mu-type opioid receptor	P35372	OPRM1	0.051
Radix Paeoniae（RP）	MOL007018	9-ethyl-neo-paeoniaflorin A_qt	Gamma-aminobutyric-acid receptor subunit alpha-2	P47869	GABRA2	0.051
Radix Paeoniae（RP）	MOL007018	9-ethyl-neo-paeoniaflorin A_qt	Sodium-dependent noradrenaline transporter	P23975	SLC6A2	0.052
Radix Paeoniae（RP）	MOL007018	9-ethyl-neo-paeoniaflorin A_qt	Sodium-dependent dopamine transporter	Q01959	SLC6A3	0.052
Radix Paeoniae（RP）	MOL007018	9-ethyl-neo-paeoniaflorin A_qt	cGMP-specific 3',5'-cyclic phosphodiesterase	O76074	PDE5A	0.053
Radix Paeoniae（RP）	MOL007018	9-ethyl-neo-paeoniaflorin A_qt	Retinal rod rhodopsin-sensitive cGMP 3',5'-cyclic phosphodiesterase subunit gamma	P18545	PDE6G	0.053
Radix Paeoniae（RP）	MOL007018	9-ethyl-neo-paeoniaflorin A_qt	NAD(P)H dehydrogenase [quinone] 1	P15559	NQO1	0.054
Radix Paeoniae（RP）	MOL007018	9-ethyl-neo-paeoniaflorin A_qt	Peroxisome proliferator-activated receptor gamma	P37231	PPARG	0.063
Radix Paeoniae（RP）	MOL007018	9-ethyl-neo-paeoniaflorin A_qt	Nuclear receptor coactivator 2	Q15596	NCOA2	0.063
Radix Paeoniae（RP）	MOL007018	9-ethyl-neo-paeoniaflorin A_qt	Mitogen-activated protein kinase 8	P45983	MAPK8	0.07
Radix Paeoniae（RP）	MOL007018	9-ethyl-neo-paeoniaflorin A_qt	Mitogen-activated protein kinase 10	P53779	MAPK10	0.07
Radix Paeoniae（RP）	MOL007018	9-ethyl-neo-paeoniaflorin A_qt	Potassium channel subfamily K member 1	O00180	KCNK1	0.073
Radix Paeoniae（RP）	MOL007018	9-ethyl-neo-paeoniaflorin A_qt	Platelet glycoprotein IX	P14770	GP9	0.073
Radix Paeoniae（RP）	MOL007018	9-ethyl-neo-paeoniaflorin A_qt	Potassium channel subfamily K member 6	Q9Y257	KCNK6	0.073
Radix Paeoniae（RP）	MOL007018	9-ethyl-neo-paeoniaflorin A_qt	Alpha-2A adrenergic receptor	P08913	ADRA2A	0.077
Radix Paeoniae（RP）	MOL007018	9-ethyl-neo-paeoniaflorin A_qt	Alpha-2B adrenergic receptor	P18089	ADRA2B	0.077
Radix Paeoniae（RP）	MOL007018	9-ethyl-neo-paeoniaflorin A_qt	Alpha-2C adrenergic receptor	P18825	ADRA2C	0.077
Radix Paeoniae（RP）	MOL007018	9-ethyl-neo-paeoniaflorin A_qt	5-hydroxytryptamine 2C receptor	P28335	HTR2C	0.077
Radix Paeoniae（RP）	MOL007018	9-ethyl-neo-paeoniaflorin A_qt	D(2) dopamine receptor	P14416	DRD2	0.078
Radix Paeoniae（RP）	MOL007018	9-ethyl-neo-paeoniaflorin A_qt	D(3) dopamine receptor	P35462	DRD3	0.078
Radix Paeoniae（RP）	MOL007018	9-ethyl-neo-paeoniaflorin A_qt	Glycogen synthase kinase-3 beta	P49841	GSK3B	0.08
Radix Paeoniae（RP）	MOL007018	9-ethyl-neo-paeoniaflorin A_qt	Muscarinic acetylcholine receptor M2	P08172	CHRM2	0.086
Radix Paeoniae（RP）	MOL007018	9-ethyl-neo-paeoniaflorin A_qt	Muscarinic acetylcholine receptor M4	P08173	CHRM4	0.086
Radix Paeoniae（RP）	MOL007018	9-ethyl-neo-paeoniaflorin A_qt	Aldo-keto reductase family 1 member C3	P42330	AKR1C3	0.101
Radix Paeoniae（RP）	MOL007018	9-ethyl-neo-paeoniaflorin A_qt	Phospholipase A2	P04054	PLA2G1B	0.103
Radix Paeoniae（RP）	MOL007018	9-ethyl-neo-paeoniaflorin A_qt	5-hydroxytryptamine 1B receptor	P28222	HTR1B	0.105
Radix Paeoniae（RP）	MOL007018	9-ethyl-neo-paeoniaflorin A_qt	S-methyl-5-thioadenosine phosphorylase	Q13126	MTAP	0.112
Radix Paeoniae（RP）	MOL007018	9-ethyl-neo-paeoniaflorin A_qt	Tyrosine-protein kinase HCK	P08631	HCK	0.114
Radix Paeoniae（RP）	MOL007018	9-ethyl-neo-paeoniaflorin A_qt	Serine/threonine-protein kinase 6	O14965	AURKA	0.116
Radix Paeoniae（RP）	MOL007018	9-ethyl-neo-paeoniaflorin A_qt	Estrogen receptor	P03372	ESR1	0.116
Radix Paeoniae（RP）	MOL007018	9-ethyl-neo-paeoniaflorin A_qt	Salivary alpha-amylase	P04745	AMY1A	0.116
Radix Paeoniae（RP）	MOL007018	9-ethyl-neo-paeoniaflorin A_qt	Thromboxane A2 receptor	P21731	TBXA2R	0.118
Radix Paeoniae（RP）	MOL007018	9-ethyl-neo-paeoniaflorin A_qt	High affinity immunoglobulin epsilon receptor gamma-subunit	P30273	FCER1G	0.12
Radix Paeoniae（RP）	MOL007018	9-ethyl-neo-paeoniaflorin A_qt	Oxysterols receptor LXR-alpha	Q13133	NR1H3	0.12
Radix Paeoniae（RP）	MOL007018	9-ethyl-neo-paeoniaflorin A_qt	Glycogen phosphorylase, muscle form	P11217	PYGM	0.121
Radix Paeoniae（RP）	MOL007018	9-ethyl-neo-paeoniaflorin A_qt	Type-1 angiotensin II receptor	P30556	AGTR1	0.123
Radix Paeoniae（RP）	MOL007018	9-ethyl-neo-paeoniaflorin A_qt	Calcium/calmodulin-dependent protein kinase type II alpha chain	Q9UQM7	CAMK2A	0.123
Radix Paeoniae（RP）	MOL007018	9-ethyl-neo-paeoniaflorin A_qt	Trypsin-1	P07477	PRSS1	0.125
Radix Paeoniae（RP）	MOL007018	9-ethyl-neo-paeoniaflorin A_qt	Cell division protein kinase 2	P24941	CDK2	0.126

Radix Paeoniae（RP）	MOL007018	9-ethyl-neo-paeoniaflorin A_qt	Cell division protein kinase 6	Q00534	CDK6	0.126
Radix Paeoniae（RP）	MOL007018	9-ethyl-neo-paeoniaflorin A_qt	Aldehyde dehydrogenase, mitochondrial	P05091	ALDH2	0.128
Radix Paeoniae（RP）	MOL007018	9-ethyl-neo-paeoniaflorin A_qt	Amine oxidase [flavin-containing] A	P21397	MAOA	0.129
Radix Paeoniae（RP）	MOL007018	9-ethyl-neo-paeoniaflorin A_qt	Retinoic acid receptor RXR-alpha	P19793	RXRA	0.133
Radix Paeoniae（RP）	MOL007018	9-ethyl-neo-paeoniaflorin A_qt	Sodium channel protein type 10 subunit alpha	Q9Y5Y9	SCN10A	0.133
Radix Paeoniae（RP）	MOL007018	9-ethyl-neo-paeoniaflorin A_qt	Peroxisome proliferator-activated receptor alpha	Q07869	PPARA	0.139
Radix Paeoniae（RP）	MOL007018	9-ethyl-neo-paeoniaflorin A_qt	Hemoglobin subunit alpha	P69905	HBA1	0.158
Radix Paeoniae（RP）	MOL007018	9-ethyl-neo-paeoniaflorin A_qt	5-hydroxytryptamine 1A receptor	P08908	HTR1A	0.163
Radix Paeoniae（RP）	MOL007018	9-ethyl-neo-paeoniaflorin A_qt	Muscarinic acetylcholine receptor M3	P20309	CHRM3	0.22
Radix Paeoniae（RP）	MOL007018	9-ethyl-neo-paeoniaflorin A_qt	C-jun-amino-terminal kinase-interacting protein 1	Q9UQF2	MAPK8IP1	0.231
Radix Paeoniae（RP）	MOL007018	9-ethyl-neo-paeoniaflorin A_qt	Sodium channel protein type 5 subunit alpha	Q14524	SCN5A	0.238
Radix Paeoniae（RP）	MOL007018	9-ethyl-neo-paeoniaflorin A_qt	Estrogen receptor beta	Q92731	ESR2	0.239
Radix Paeoniae（RP）	MOL007018	9-ethyl-neo-paeoniaflorin A_qt	Progesterone receptor	P06401	PGR	0.248
Radix Paeoniae（RP）	MOL007018	9-ethyl-neo-paeoniaflorin A_qt	Muscarinic acetylcholine receptor M1	P11229	CHRM1	0.254
Radix Paeoniae（RP）	MOL007018	9-ethyl-neo-paeoniaflorin A_qt	Prostaglandin G/H synthase 1	P23219	PTGS1	0.321
Radix Paeoniae（RP）	MOL007018	9-ethyl-neo-paeoniaflorin A_qt	cAMP-dependent protein kinase catalytic subunit alpha	P17612	PRKACA	0.382
Radix Paeoniae（RP）	MOL007018	9-ethyl-neo-paeoniaflorin A_qt	Prostaglandin G/H synthase 2	P35354	PTGS2	1
Radix Paeoniae（RP）	MOL007022	evofolinB	Phosphatidylinositol 3-kinase regulatory subunit beta	O00459	PIK3R2	0.014
Radix Paeoniae（RP）	MOL007022	evofolinB	Beta-3 adrenergic receptor	P13945	ADRB3	0.014
Radix Paeoniae（RP）	MOL007022	evofolinB	Phosphatidylinositol 3-kinase regulatory subunit alpha	P27986	PIK3R1	0.014
Radix Paeoniae（RP）	MOL007022	evofolinB	Mitogen-activated protein kinase 1	P28482	MAPK1	0.014
Radix Paeoniae（RP）	MOL007022	evofolinB	5-hydroxytryptamine 1A receptor	P08908	HTR1A	0.016
Radix Paeoniae（RP）	MOL007022	evofolinB	5-hydroxytryptamine 2C receptor	P28335	HTR2C	0.016
Radix Paeoniae（RP）	MOL007022	evofolinB	Translocator protein	P30536	TSPO	0.016
Radix Paeoniae（RP）	MOL007022	evofolinB	5-hydroxytryptamine 2B receptor	P41595	HTR2B	0.016
Radix Paeoniae（RP）	MOL007022	evofolinB	D1 dopamine receptor-interacting protein calcyon	Q9NYX4	CALY	0.016
Radix Paeoniae（RP）	MOL007022	evofolinB	Histamine H2 receptor	P25021	HRH2	0.019
Radix Paeoniae（RP）	MOL007022	evofolinB	Glutamate receptor 2	P42262	GRIA2	0.021
Radix Paeoniae（RP）	MOL007022	evofolinB	Voltage-dependent calcium channel subunit alpha-2/delta-1	P54289	CACNA2D1	0.021
Radix Paeoniae（RP）	MOL007022	evofolinB	Voltage-dependent L-type calcium channel subunit alpha-1D	Q01668	CACNA1D	0.021
Radix Paeoniae（RP）	MOL007022	evofolinB	Voltage-dependent L-type calcium channel subunit beta-2	Q08289	CACNB2	0.021
Radix Paeoniae（RP）	MOL007022	evofolinB	Voltage-dependent L-type calcium channel subunit alpha-1C	Q13936	CACNA1C	0.021
Radix Paeoniae（RP）	MOL007022	evofolinB	Voltage-dependent calcium channel subunit alpha-2/delta-2	Q9NY47	CACNA2D2	0.021
Radix Paeoniae（RP）	MOL007022	evofolinB	High affinity cAMP-specific and IBMX-insensitive 3',5'-cyclic phosphodiesterase 8A	O60658	PDE8A	0.022
Radix Paeoniae（RP）	MOL007022	evofolinB	6-phosphogluconate dehydrogenase, decarboxylating	P52209	PGD	0.022
Radix Paeoniae（RP）	MOL007022	evofolinB	cAMP-specific 3',5'-cyclic phosphodiesterase 4C	Q08493	PDE4C	0.022
Radix Paeoniae（RP）	MOL007022	evofolinB	cAMP-specific 3',5'-cyclic phosphodiesterase 4D	Q08499	PDE4D	0.022
Radix Paeoniae（RP）	MOL007022	evofolinB	High-affinity cAMP-specific 3',5'-cyclic phosphodiesterase 7A	Q13946	PDE7A	0.022
Radix Paeoniae（RP）	MOL007022	evofolinB	cGMP-inhibited 3',5'-cyclic phosphodiesterase A	Q14432	PDE3A	0.022
Radix Paeoniae（RP）	MOL007022	evofolinB	cAMP-specific 3',5'-cyclic phosphodiesterase 7B	Q9NP56	PDE7B	0.022
Radix Paeoniae（RP）	MOL007022	evofolinB	cAMP and cAMP-inhibited cGMP 3',5'-cyclic phosphodiesterase 10A	Q9Y233	PDE10A	0.022
Radix Paeoniae（RP）	MOL007022	evofolinB	Gamma-aminobutyric-acid receptor subunit beta-3	P28472	GABRB3	0.024
Radix Paeoniae（RP）	MOL007022	evofolinB	Gamma-aminobutyric-acid receptor subunit beta-2	P47870	GABRB2	0.024
Radix Paeoniae（RP）	MOL007022	evofolinB	Tyrosyl-tRNA synthetase, cytoplasmic	P54577	YARS	0.024
Radix Paeoniae（RP）	MOL007022	evofolinB	Gamma-aminobutyric-acid receptor subunit alpha-6	Q16445	GABRA6	0.024
Radix Paeoniae（RP）	MOL007022	evofolinB	Gamma-aminobutyric acid receptor subunit theta	Q9UN88	GABRQ	0.024
Radix Paeoniae（RP）	MOL007022	evofolinB	Neuronal acetylcholine receptor subunit alpha-2	Q15822	CHRNA2	0.025
Radix Paeoniae（RP）	MOL007022	evofolinB	Opioid receptor, sigma 1	Q5T1J1	SIGMAR1	0.025
Radix Paeoniae（RP）	MOL007022	evofolinB	Sigma 1-type opioid receptor	Q99720	SIGMAR1	0.025
Radix Paeoniae（RP）	MOL007022	evofolinB	Glutamate [NMDA] receptor subunit epsilon-4	O15399	GRIN2D	0.027
Radix Paeoniae（RP）	MOL007022	evofolinB	Glutamate [NMDA] receptor subunit 3B	O60391	GRIN3B	0.027
Radix Paeoniae（RP）	MOL007022	evofolinB	Glutamate [NMDA] receptor subunit zeta-1	Q05586	GRIN1	0.027

Radix Paeoniae（RP）	MOL007022	evofolinB	5-hydroxytryptamine 3 receptor	P46098	HTR3A	0.028
Radix Paeoniae（RP）	MOL007022	evofolinB	Sodium channel protein type 10 subunit alpha	Q9Y5Y9	SCN10A	0.028
Radix Paeoniae（RP）	MOL007022	evofolinB	Stromelysin-1	P08254	MMP3	0.029
Radix Paeoniae（RP）	MOL007022	evofolinB	Stromelysin-2	P09238	MMP10	0.029
Radix Paeoniae（RP）	MOL007022	evofolinB	Macrophage metalloelastase	P39900	MMP12	0.029
Radix Paeoniae（RP）	MOL007022	evofolinB	Amine oxidase [flavin-containing] A	P21397	MAOA	0.03
Radix Paeoniae（RP）	MOL007022	evofolinB	Chromaffin granule amine transporter	P54219	SLC18A1	0.03
Radix Paeoniae（RP）	MOL007022	evofolinB	Synaptic vesicular amine transporter	Q05940	SLC18A2	0.03
Radix Paeoniae（RP）	MOL007022	evofolinB	Gamma-aminobutyric-acid receptor subunit alpha-4	P48169	GABRA4	0.032
Radix Paeoniae（RP）	MOL007022	evofolinB	cAMP response element-binding protein	P16220	CREB1	0.036
Radix Paeoniae（RP）	MOL007022	evofolinB	Phenylalanine-4-hydroxylase	P00439	PAH	0.038
Radix Paeoniae（RP）	MOL007022	evofolinB	DNA topoisomerase 2-alpha	P11388	TOP2A	0.038
Radix Paeoniae（RP）	MOL007022	evofolinB	Sepiapterin reductase	P35270	SPR	0.038
Radix Paeoniae（RP）	MOL007022	evofolinB	Tubulin alpha-1 chain	P68366	TUBA4A	0.038
Radix Paeoniae（RP）	MOL007022	evofolinB	6-pyruvoyl tetrahydrobiopterin synthase	Q03393	PTS	0.038
Radix Paeoniae（RP）	MOL007022	evofolinB	Tryptophanyl-tRNA synthetase, mitochondrial	Q9UGM6	WARS2	0.038
Radix Paeoniae（RP）	MOL007022	evofolinB	Succinate dehydrogenase [ubiquinone] cytochrome b small subunit, mitochondrial	O14521	SDHD	0.039
Radix Paeoniae（RP）	MOL007022	evofolinB	5-hydroxytryptamine 1D receptor	P28221	HTR1D	0.039
Radix Paeoniae（RP）	MOL007022	evofolinB	5-hydroxytryptamine 1B receptor	P28222	HTR1B	0.039
Radix Paeoniae（RP）	MOL007022	evofolinB	Peroxisome proliferator-activated receptor delta	Q03181	PPARD	0.04
Radix Paeoniae（RP）	MOL007022	evofolinB	Peroxisome proliferator-activated receptor alpha	Q07869	PPARA	0.04
Radix Paeoniae（RP）	MOL007022	evofolinB	D(4) dopamine receptor	P21917	DRD4	0.043
Radix Paeoniae（RP）	MOL007022	evofolinB	Sterol O-acyltransferase 1	P35610	SOAT1	0.045
Radix Paeoniae（RP）	MOL007022	evofolinB	Niemann-Pick C1-like protein 1	Q9UHC9	NPC1L1	0.045
Radix Paeoniae（RP）	MOL007022	evofolinB	Muscarinic acetylcholine receptor M2	P08172	CHRM2	0.048
Radix Paeoniae（RP）	MOL007022	evofolinB	Gamma-aminobutyric acid receptor subunit gamma-1	Q8N1C3	GABRG1	0.048
Radix Paeoniae（RP）	MOL007022	evofolinB	Potassium voltage-gated channel subfamily D member 2	Q9NZV8	KCND2	0.048
Radix Paeoniae（RP）	MOL007022	evofolinB	cAMP-specific 3',5'-cyclic phosphodiesterase 4A	P27815	PDE4A	0.052
Radix Paeoniae（RP）	MOL007022	evofolinB	cAMP-specific 3',5'-cyclic phosphodiesterase 4B	Q07343	PDE4B	0.052
Radix Paeoniae（RP）	MOL007022	evofolinB	Gamma-aminobutyric acid receptor subunit rho-3	A8MPY1	GABRR3	0.056
Radix Paeoniae（RP）	MOL007022	evofolinB	Gamma-aminobutyric acid receptor subunit pi	O00591	GABRP	0.056
Radix Paeoniae（RP）	MOL007022	evofolinB	Gamma-aminobutyric acid receptor subunit delta	O14764	GABRD	0.056
Radix Paeoniae（RP）	MOL007022	evofolinB	Gamma-aminobutyric-acid receptor subunit beta-1	P18505	GABRB1	0.056
Radix Paeoniae（RP）	MOL007022	evofolinB	Gamma-aminobutyric acid receptor subunit gamma-2	P18507	GABRG2	0.056
Radix Paeoniae（RP）	MOL007022	evofolinB	Gamma-aminobutyric-acid receptor subunit rho-1	P24046	GABRR1	0.056
Radix Paeoniae（RP）	MOL007022	evofolinB	Gamma-aminobutyric acid receptor subunit rho-2	P28476	GABRR2	0.056
Radix Paeoniae（RP）	MOL007022	evofolinB	Gamma-aminobutyric acid receptor subunit epsilon	P78334	GABRE	0.056
Radix Paeoniae（RP）	MOL007022	evofolinB	Gamma-aminobutyric acid receptor subunit gamma-3	Q99928	GABRG3	0.056
Radix Paeoniae（RP）	MOL007022	evofolinB	Aldo-keto reductase family 1 member C3	P42330	AKR1C3	0.06
Radix Paeoniae（RP）	MOL007022	evofolinB	Group IIE secretory phospholipase A2	Q9NZK7	PLA2G2E	0.06
Radix Paeoniae（RP）	MOL007022	evofolinB	Alpha-1D adrenergic receptor	P25100	ADRA1D	0.061
Radix Paeoniae（RP）	MOL007022	evofolinB	Alpha-1B adrenergic receptor	P35368	ADRA1B	0.061
Radix Paeoniae（RP）	MOL007022	evofolinB	D(3) dopamine receptor	P35462	DRD3	0.069
Radix Paeoniae（RP）	MOL007022	evofolinB	Gamma-aminobutyric-acid receptor subunit alpha-3	P34903	GABRA3	0.072
Radix Paeoniae（RP）	MOL007022	evofolinB	Gamma-aminobutyric-acid receptor subunit alpha-1	P14867	GABRA1	0.081
Radix Paeoniae（RP）	MOL007022	evofolinB	Gamma-aminobutyric-acid receptor subunit alpha-5	P31644	GABRA5	0.081
Radix Paeoniae（RP）	MOL007022	evofolinB	Gamma-aminobutyric-acid receptor subunit alpha-2	P47869	GABRA2	0.081
Radix Paeoniae（RP）	MOL007022	evofolinB	Beta-1 adrenergic receptor	P08588	ADRB1	0.082
Radix Paeoniae（RP）	MOL007022	evofolinB	Carbonic anhydrase 2	P00918	CA2	0.083
Radix Paeoniae（RP）	MOL007022	evofolinB	Phospholipase A2	P04054	PLA2G1B	0.084
Radix Paeoniae（RP）	MOL007022	evofolinB	Estradiol 17-beta-dehydrogenase 1	P14061	HSD17B1	0.084
Radix Paeoniae（RP）	MOL007022	evofolinB	Nitric oxide synthase, inducible	P35228	NOS2	0.084
Radix Paeoniae（RP）	MOL007022	evofolinB	Triosephosphate isomerase	P60174	TPI1	0.084

Radix Paeoniae（RP）	MOL007022	evofolinB	Estrogen-related receptor gamma	P62508	ESRRG	0.084
Radix Paeoniae（RP）	MOL007022	evofolinB	Aldo-keto reductase family 1 member C1	Q04828	AKR1C1	0.084
Radix Paeoniae（RP）	MOL007022	evofolinB	Toll-like receptor 7	Q9NYK1	TLR7	0.084
Radix Paeoniae（RP）	MOL007022	evofolinB	Alpha-2C adrenergic receptor	P18825	ADRA2C	0.086
Radix Paeoniae（RP）	MOL007022	evofolinB	5-hydroxytryptamine 2A receptor	P28223	HTR2A	0.086
Radix Paeoniae（RP）	MOL007022	evofolinB	Phosphatidylinositol-4,5-bisphosphate 3-kinase catalytic subunit gamma isoform	P48736	PIK3CG	0.086
Radix Paeoniae（RP）	MOL007022	evofolinB	Potassium channel subfamily K member 3	O14649	KCNK3	0.087
Radix Paeoniae（RP）	MOL007022	evofolinB	Nuclear receptor coactivator 1	Q15788	NCOA1	0.088
Radix Paeoniae（RP）	MOL007022	evofolinB	Ig kappa chain V-II region RPMI 6410	P06310	IGKV2-30	0.09
Radix Paeoniae（RP）	MOL007022	evofolinB	Cytochrome P450 19A1	P11511	CYP19A1	0.09
Radix Paeoniae（RP）	MOL007022	evofolinB	Thyroid hormone receptor alpha	P10827	THRA	0.092
Radix Paeoniae（RP）	MOL007022	evofolinB	D(1A) dopamine receptor	P21728	DRD1	0.093
Radix Paeoniae（RP）	MOL007022	evofolinB	D(1B) dopamine receptor	P21918	DRD5	0.093
Radix Paeoniae（RP）	MOL007022	evofolinB	RAC-alpha serine/threonine-protein kinase	P31749	AKT1	0.093
Radix Paeoniae（RP）	MOL007022	evofolinB	Thymidylate synthase	P04818	TYMS	0.094
Radix Paeoniae（RP）	MOL007022	evofolinB	Mitogen-activated protein kinase 14	Q16539	MAPK14	0.094
Radix Paeoniae（RP）	MOL007022	evofolinB	Neuropeptide Y	P01303	NPY	0.095
Radix Paeoniae（RP）	MOL007022	evofolinB	Thyroid hormone receptor, alpha isoform 1 variant	Q59FW3	SIGMAR1	0.096
Radix Paeoniae（RP）	MOL007022	evofolinB	Glutamate [NMDA] receptor subunit 3A	Q8TCU5	GRIN3A	0.096
Radix Paeoniae（RP）	MOL007022	evofolinB	Thyroid hormone receptor beta-1	P10828	THRB	0.097
Radix Paeoniae（RP）	MOL007022	evofolinB	D(2) dopamine receptor	P14416	DRD2	0.097
Radix Paeoniae（RP）	MOL007022	evofolinB	Nuclear receptor coactivator 2	Q15596	NCOA2	0.097
Radix Paeoniae（RP）	MOL007022	evofolinB	Cell division protein kinase 5	Q00535	CDK5	0.098
Radix Paeoniae（RP）	MOL007022	evofolinB	Beta-2 adrenergic receptor	P07550	ADRB2	0.105
Radix Paeoniae（RP）	MOL007022	evofolinB	Alpha-2B adrenergic receptor	P18089	ADRA2B	0.109
Radix Paeoniae（RP）	MOL007022	evofolinB	Heat shock protein HSP 90-beta	P08238	HSP90AB1	0.111
Radix Paeoniae（RP）	MOL007022	evofolinB	Cytochrome P450 11B1, mitochondrial	P15538	CYP11B1	0.112
Radix Paeoniae（RP）	MOL007022	evofolinB	Alpha-1A adrenergic receptor	P35348	ADRA1A	0.112
Radix Paeoniae（RP）	MOL007022	evofolinB	Delta-type opioid receptor	P41143	OPRD1	0.123
Radix Paeoniae（RP）	MOL007022	evofolinB	Prostaglandin G/H synthase 1	P23219	PTGS1	0.129
Radix Paeoniae（RP）	MOL007022	evofolinB	Alpha-2A adrenergic receptor	P08913	ADRA2A	0.136
Radix Paeoniae（RP）	MOL007022	evofolinB	Histamine H1 receptor	P35367	HRH1	0.146
Radix Paeoniae（RP）	MOL007022	evofolinB	Muscarinic acetylcholine receptor M3	P20309	CHRM3	0.158
Radix Paeoniae（RP）	MOL007022	evofolinB	Sodium channel protein type 5 subunit alpha	Q14524	SCN5A	0.163
Radix Paeoniae（RP）	MOL007022	evofolinB	Kappa-type opioid receptor	P41145	OPRK1	0.167
Radix Paeoniae（RP）	MOL007022	evofolinB	cAMP-dependent protein kinase catalytic subunit alpha	P17612	PRKACA	0.192
Radix Paeoniae（RP）	MOL007022	evofolinB	Sodium-dependent serotonin transporter	P31645	SLC6A4	0.196
Radix Paeoniae（RP）	MOL007022	evofolinB	Estrogen receptor beta	Q92731	ESR2	0.198
Radix Paeoniae（RP）	MOL007022	evofolinB	Mu-type opioid receptor	P35372	OPRM1	0.21
Radix Paeoniae（RP）	MOL007022	evofolinB	Sodium-dependent dopamine transporter	Q01959	SLC6A3	0.291
Radix Paeoniae（RP）	MOL007022	evofolinB	Sodium-dependent noradrenaline transporter	P23975	SLC6A2	0.297
Radix Paeoniae（RP）	MOL007022	evofolinB	Muscarinic acetylcholine receptor M1	P11229	CHRM1	0.306
Radix Paeoniae（RP）	MOL007022	evofolinB	Prostaglandin G/H synthase 2	P35354	PTGS2	0.54
Radix Paeoniae（RP）	MOL007022	evofolinB	Estrogen receptor	P03372	ESR1	1
Radix Paeoniae（RP）	MOL007025	isobenzoylpaeoniflorin	Muscarinic acetylcholine receptor M2	P08172	CHRM2	0.132
Radix Paeoniae（RP）	MOL007025	isobenzoylpaeoniflorin	Muscarinic acetylcholine receptor M4	P08173	CHRM4	0.132
Radix Paeoniae（RP）	MOL007025	isobenzoylpaeoniflorin	Muscarinic acetylcholine receptor M1	P11229	CHRM1	0.132
Radix Paeoniae（RP）	MOL007025	isobenzoylpaeoniflorin	Muscarinic acetylcholine receptor M3	P20309	CHRM3	0.132
Radix Paeoniae（RP）	MOL007025	isobenzoylpaeoniflorin	Gamma-aminobutyric-acid receptor subunit alpha-1	P14867	GABRA1	0.155
Radix Paeoniae（RP）	MOL007025	isobenzoylpaeoniflorin	Gamma-aminobutyric-acid receptor subunit alpha-2	P47869	GABRA2	0.155
Radix Paeoniae（RP）	MOL007025	isobenzoylpaeoniflorin	Potassium channel subfamily K member 6	Q9Y257	KCNK6	0.155
Radix Paeoniae（RP）	MOL007025	isobenzoylpaeoniflorin	Carbonic anhydrase 1	P00915	CA1	0.215
Radix Paeoniae（RP）	MOL007025	isobenzoylpaeoniflorin	Carbonic anhydrase 2	P00918	CA2	0.215

Radix Paeoniae（RP）	MOL007025	isobenzoylpaeoniflorin	Sodium/potassium-transporting ATPase gamma chain	P54710	FXYD2	0.215
Radix Paeoniae（RP）	MOL007025	isobenzoylpaeoniflorin	Progesterone receptor	P06401	PGR	0.71
Radix Paeoniae（RP）	MOL007025	isobenzoylpaeoniflorin	Mineralocorticoid receptor	P08235	NR3C2	0.71
Radix Paeoniae（RP）	MOL007025	isobenzoylpaeoniflorin	Platelet glycoprotein IX	P14770	GP9	0.762
Radix Paeoniae（RP）	MOL007025	isobenzoylpaeoniflorin	Potassium channel subfamily K member 1	O00180	KCNK1	1
Radix Paeoniae（RP）	MOL007025	isobenzoylpaeoniflorin	Sodium channel protein type 5 subunit alpha	Q14524	SCN5A	1
Radix Cyathulae（RC）	MOL000098	quercetin	Adenosine A2b receptor	P29275	ADORA2B	0.011
Radix Cyathulae（RC）	MOL000098	quercetin	Adenosine A3 receptor	P33765	SHMT1	0.011
Radix Cyathulae（RC）	MOL000098	quercetin	cGMP-specific 3',5'-cyclic phosphodiesterase	O76074	PDE5A	0.012
Radix Cyathulae（RC）	MOL000098	quercetin	Adenosine A1 receptor	P30542	ADORA1	0.012
Radix Cyathulae（RC）	MOL000098	quercetin	cAMP-specific 3',5'-cyclic phosphodiesterase 4B	Q07343	PDE4B	0.012
Radix Cyathulae（RC）	MOL000098	quercetin	Neuronal acetylcholine receptor subunit alpha-2	Q15822	CHRNA2	0.012
Radix Cyathulae（RC）	MOL000098	quercetin	Potassium channel subfamily K member 1	O00180	KCNK1	0.013
Radix Cyathulae（RC）	MOL000098	quercetin	Tyrosine 3-monooxygenase	P07101	TH	0.013
Radix Cyathulae（RC）	MOL000098	quercetin	cGMP-inhibited 3',5'-cyclic phosphodiesterase A	Q14432	PDE3A	0.013
Radix Cyathulae（RC）	MOL000098	quercetin	cAMP and cAMP-inhibited cGMP 3',5'-cyclic phosphodiesterase 10A	Q9Y233	PDE10A	0.013
Radix Cyathulae（RC）	MOL000098	quercetin	Tyrosyl-tRNA synthetase, mitochondrial	Q9Y2Z4	YARS2	0.013
Radix Cyathulae（RC）	MOL000098	quercetin	3-phosphoinositide-dependent protein kinase 1	O15530	PDPK1	0.014
Radix Cyathulae（RC）	MOL000098	quercetin	Serine/threonine-protein kinase 17B	O94768	STK17B	0.014
Radix Cyathulae（RC）	MOL000098	quercetin	Pepsin A	P00790	REN	0.014
Radix Cyathulae（RC）	MOL000098	quercetin	Tyrosine-protein kinase Lyn	P07948	LYN	0.014
Radix Cyathulae（RC）	MOL000098	quercetin	Muscarinic acetylcholine receptor M5	P08912	CHRM5	0.014
Radix Cyathulae（RC）	MOL000098	quercetin	Cell division protein kinase 4	P11802	CDK4	0.014
Radix Cyathulae（RC）	MOL000098	quercetin	Gamma-aminobutyric-acid receptor subunit alpha-1	P14867	GABRA1	0.014
Radix Cyathulae（RC）	MOL000098	quercetin	Dipeptidyl peptidase 4	P27487	DPP4	0.014
Radix Cyathulae（RC）	MOL000098	quercetin	ATP synthase subunit gamma, mitochondrial	P36542	ATP5F1C	0.014
Radix Cyathulae（RC）	MOL000098	quercetin	Tyrosine-protein kinase CSK	P41240	CSK	0.014
Radix Cyathulae（RC）	MOL000098	quercetin	Tyrosine-protein kinase ZAP-70	P43403	ZAP70	0.014
Radix Cyathulae（RC）	MOL000098	quercetin	Tyrosine-protein kinase SYK	P43405	SYK	0.014
Radix Cyathulae（RC）	MOL000098	quercetin	Gamma-aminobutyric-acid receptor subunit alpha-2	P47869	GABRA2	0.014
Radix Cyathulae（RC）	MOL000098	quercetin	Cell division protein kinase 7	P50613	CDK7	0.014
Radix Cyathulae（RC）	MOL000098	quercetin	Cell division protein kinase 9	P50750	CDK9	0.014
Radix Cyathulae（RC）	MOL000098	quercetin	Cell division protein kinase 6	Q00534	CDK6	0.014
Radix Cyathulae（RC）	MOL000098	quercetin	Protein kinase C theta type	Q04759	PRKCQ	0.014
Radix Cyathulae（RC）	MOL000098	quercetin	Tyrosine-protein kinase ITK/TSK	Q08881	ITK	0.014
Radix Cyathulae（RC）	MOL000098	quercetin	Cyclin-dependent kinase 5 activator 1	Q15078	CDK5R1	0.014
Radix Cyathulae（RC）	MOL000098	quercetin	UDP-glucuronosyltransferase 3A1	Q6NUS8	UGT3A1	0.014
Radix Cyathulae（RC）	MOL000098	quercetin	Dehydrogenase/reductase SDR family member 8	Q8NBQ5	HSD17B11	0.014
Radix Cyathulae（RC）	MOL000098	quercetin	85 kDa calcium-independent phospholipase A2	O60733	PLA2G6	0.015
Radix Cyathulae（RC）	MOL000098	quercetin	Ig gamma-2 chain C region	P01859	IGHG2	0.015
Radix Cyathulae（RC）	MOL000098	quercetin	Retinoic acid receptor alpha	P10276	RARA	0.015
Radix Cyathulae（RC）	MOL000098	quercetin	Retinoic acid receptor beta	P10826	RARB	0.015
Radix Cyathulae（RC）	MOL000098	quercetin	Retinoic acid receptor gamma-1	P13631	RARG	0.015
Radix Cyathulae（RC）	MOL000098	quercetin	Beta-3 adrenergic receptor	P13945	ADRB3	0.015
Radix Cyathulae（RC）	MOL000098	quercetin	5-hydroxytryptamine 1D receptor	P28221	HTR1D	0.015
Radix Cyathulae（RC）	MOL000098	quercetin	5-hydroxytryptamine 1B receptor	P28222	HTR1B	0.015
Radix Cyathulae（RC）	MOL000098	quercetin	5-hydroxytryptamine 2C receptor	P28335	HTR2C	0.015

Radix Cyathulae（RC）	MOL000098	quercetin	Amiloride-sensitive sodium channel subunit alpha	P37088	SCNN1A	0.015
Radix Cyathulae（RC）	MOL000098	quercetin	Cytosolic phospholipase A2	P47712	PLA2G4A	0.015
Radix Cyathulae（RC）	MOL000098	quercetin	Retinoic acid receptor RXR-gamma	P48443	RXRG	0.015
Radix Cyathulae（RC）	MOL000098	quercetin	Amiloride-sensitive sodium channel subunit beta	P51168	SCNN1B	0.015
Radix Cyathulae（RC）	MOL000098	quercetin	Amiloride-sensitive sodium channel subunit gamma	P51170	SCNN1G	0.015
Radix Cyathulae（RC）	MOL000098	quercetin	Sodium/potassium-transporting ATPase gamma chain	P54710	FXYD2	0.015
Radix Cyathulae（RC）	MOL000098	quercetin	Solute carrier family 12 member 3	P55017	SLC12A3	0.015
Radix Cyathulae（RC）	MOL000098	quercetin	Calcium-activated potassium channel subunit alpha 1	Q12791	KCNMA1	0.015
Radix Cyathulae（RC）	MOL000098	quercetin	5-hydroxytryptamine 3 receptor	P46098	HTR3A	0.017
Radix Cyathulae（RC）	MOL000098	quercetin	Prostaglandin reductase 1	Q14914	PTGR1	0.017
Radix Cyathulae（RC）	MOL000098	quercetin	Glutamate [NMDA] receptor subunit 3A	Q8TCU5	GRIN3A	0.017
Radix Cyathulae（RC）	MOL000098	quercetin	DNA topoisomerase 1	P11387	TOP1	0.02
Radix Cyathulae（RC）	MOL000098	quercetin	Macrophage migration inhibitory factor	P14174	MIF	0.02
Radix Cyathulae（RC）	MOL000098	quercetin	Calcium/calmodulin-dependent protein kinase type II subunit delta	Q13557	CAMK2D	0.02
Radix Cyathulae（RC）	MOL000098	quercetin	DNA topoisomerase I, mitochondrial	Q969P6	TOP1MT	0.02
Radix Cyathulae（RC）	MOL000098	quercetin	STE20-like serine/threonine-protein kinase	Q9H2G2	SLK	0.02
Radix Cyathulae（RC）	MOL000098	quercetin	Tripartite motif-containing protein 13	O60858	TRIM13	0.021
Radix Cyathulae（RC）	MOL000098	quercetin	Chymase	P23946	CMA1	0.021
Radix Cyathulae（RC）	MOL000098	quercetin	DNA-(apurinic or apyrimidinic site) lyase	P27695	APEX1	0.021
Radix Cyathulae（RC）	MOL000098	quercetin	Succinate dehydrogenase [ubiquinone] cytochrome b small subunit, mitochondrial	O14521	SDHD	0.022
Radix Cyathulae（RC）	MOL000098	quercetin	Inhibitor of nuclear factor kappa-B kinase subunit beta	O14920	IKBKB	0.022
Radix Cyathulae（RC）	MOL000098	quercetin	Ribosyldihydronicotinamide dehydrogenase [quinone]	P16083	NQO2	0.022
Radix Cyathulae（RC）	MOL000098	quercetin	Peptidyl-prolyl cis-trans isomerase, mitochondrial	P30405	PPIF	0.022
Radix Cyathulae（RC）	MOL000098	quercetin	Prostacyclin receptor	P43119	PTGIR	0.022
Radix Cyathulae（RC）	MOL000098	quercetin	Mitogen-activated protein kinase 8	P45983	MAPK8	0.022
Radix Cyathulae（RC）	MOL000098	quercetin	Mitogen-activated protein kinase 10	P53779	MAPK10	0.022
Radix Cyathulae（RC）	MOL000098	quercetin	Peroxisome proliferator-activated receptor delta	Q03181	PPARD	0.022
Radix Cyathulae（RC）	MOL000098	quercetin	Group IIE secretory phospholipase A2	Q9NZK7	PLA2G2E	0.022
Radix Cyathulae（RC）	MOL000098	quercetin	Cystine/glutamate transporter	Q9UPY5	SLC7A11	0.022
Radix Cyathulae（RC）	MOL000098	quercetin	Oxysterols receptor LXR-beta	P55055	NR1H2	0.023
Radix Cyathulae（RC）	MOL000098	quercetin	Oxysterols receptor LXR-alpha	Q13133	NR1H3	0.023
Radix Cyathulae（RC）	MOL000098	quercetin	Sodium channel protein type 5 subunit alpha	Q14524	SCN5A	0.023
Radix Cyathulae（RC）	MOL000098	quercetin	Phenylalanine-4-hydroxylase	P00439	PAH	0.024
Radix Cyathulae（RC）	MOL000098	quercetin	Rho-associated protein kinase 1	Q13464	ROCK1	0.024
Radix Cyathulae（RC）	MOL000098	quercetin	Alpha-1D adrenergic receptor	P25100	ADRA1D	0.025
Radix Cyathulae（RC）	MOL000098	quercetin	D(4) dopamine receptor	P21917	DRD4	0.026
Radix Cyathulae（RC）	MOL000098	quercetin	Keratin, type II cytoskeletal 7	P08729	KRT7	0.028
Radix Cyathulae（RC）	MOL000098	quercetin	Platelet glycoprotein IX	P14770	GP9	0.028
Radix Cyathulae（RC）	MOL000098	quercetin	Adenosine A2a receptor	P29274	ADORA2A	0.029
Radix Cyathulae（RC）	MOL000098	quercetin	Sterol O-acyltransferase 2	O75908	SOAT2	0.031
Radix Cyathulae（RC）	MOL000098	quercetin	Sterol O-acyltransferase 1	P35610	SOAT1	0.031
Radix Cyathulae（RC）	MOL000098	quercetin	Tubulin beta-2C chain	P68371	TUBB4B	0.031
Radix Cyathulae（RC）	MOL000098	quercetin	3 beta-hydroxysteroid dehydrogenase/Delta 5-->4-isomerase type II	P26439	HSD3B2	0.032
Radix Cyathulae（RC）	MOL000098	quercetin	Protein tyrosine kinase 2 beta	Q14289	PTK2B	0.032
Radix Cyathulae（RC）	MOL000098	quercetin	Tyrosyl-tRNA synthetase, cytoplasmic	P54577	YARS	0.033
Radix Cyathulae（RC）	MOL000098	quercetin	ATP synthase subunit beta, mitochondrial	P06576	ATP5F1B	0.034
Radix Cyathulae（RC）	MOL000098	quercetin	Muscarinic acetylcholine receptor M4	P08173	CHRM4	0.034

Radix Cyathulae（RC）	MOL000098	quercetin	Tyrosine-protein kinase HCK	P08631	HCK	0.034
Radix Cyathulae（RC）	MOL000098	quercetin	Proto-oncogene serine/threonine-protein kinase Pim-1	P11309	PIM1	0.034
Radix Cyathulae（RC）	MOL000098	quercetin	Muscarinic acetylcholine receptor M3	P20309	CHRM3	0.034
Radix Cyathulae（RC）	MOL000098	quercetin	ATP synthase subunit alpha, mitochondrial	P25705	ATP5F1A	0.034
Radix Cyathulae（RC）	MOL000098	quercetin	Phosphatidylinositol-4,5-bisphosphate 3-kinase catalytic subunit gamma isoform	P48736	PIK3CG	0.034
Radix Cyathulae（RC）	MOL000098	quercetin	Epidermal growth factor receptor	P00533	EGFR	0.035
Radix Cyathulae（RC）	MOL000098	quercetin	Carbonic anhydrase 1	P00915	CA1	0.035
Radix Cyathulae（RC）	MOL000098	quercetin	Proto-oncogene tyrosine-protein kinase LCK	P06239	LCK	0.035
Radix Cyathulae（RC）	MOL000098	quercetin	5-hydroxytryptamine 1A receptor	P08908	HTR1A	0.035
Radix Cyathulae（RC）	MOL000098	quercetin	Glycogen phosphorylase, muscle form	P11217	PYGM	0.035
Radix Cyathulae（RC）	MOL000098	quercetin	Alpha-1B adrenergic receptor	P35368	ADRA1B	0.035
Radix Cyathulae（RC）	MOL000098	quercetin	Bile salt sulfotransferase	Q06520	SULT2A1	0.036
Radix Cyathulae（RC）	MOL000098	quercetin	Ig kappa chain C region	P01834	IGKC	0.037
Radix Cyathulae（RC）	MOL000098	quercetin	Ig gamma-1 chain C region	P01857	IGHG1	0.037
Radix Cyathulae（RC）	MOL000098	quercetin	D(2) dopamine receptor	P14416	DRD2	0.037
Radix Cyathulae（RC）	MOL000098	quercetin	D(3) dopamine receptor	P35462	DRD3	0.037
Radix Cyathulae（RC）	MOL000098	quercetin	Estrogen-related receptor gamma	P62508	ESRRG	0.037
Radix Cyathulae（RC）	MOL000098	quercetin	Muscarinic acetylcholine receptor M2	P08172	CHRM2	0.044
Radix Cyathulae（RC）	MOL000098	quercetin	Muscarinic acetylcholine receptor M1	P11229	CHRM1	0.044
Radix Cyathulae（RC）	MOL000098	quercetin	Retinoic acid receptor RXR-beta	P28702	RXRB	0.044
Radix Cyathulae（RC）	MOL000098	quercetin	Aldo-keto reductase family 1 member C1	Q04828	AKR1C1	0.044
Radix Cyathulae（RC）	MOL000098	quercetin	Alpha-2A adrenergic receptor	P08913	ADRA2A	0.045
Radix Cyathulae（RC）	MOL000098	quercetin	Cytochrome P450 19A1	P11511	CYP19A1	0.045
Radix Cyathulae（RC）	MOL000098	quercetin	Alpha-2C adrenergic receptor	P18825	ADRA2C	0.045
Radix Cyathulae（RC）	MOL000098	quercetin	Alpha-1A adrenergic receptor	P35348	ADRA1A	0.045
Radix Cyathulae（RC）	MOL000098	quercetin	Kynurenine--oxoglutarate transaminase 1	Q16773	KYAT1	0.045
Radix Cyathulae（RC）	MOL000098	quercetin	Membrane copper amine oxidase	Q16853	AOC3	0.045
Radix Cyathulae（RC）	MOL000098	quercetin	NADPH oxidase organizer 1	Q8NFA2	NOXO1	0.045
Radix Cyathulae（RC）	MOL000098	quercetin	Serine/threonine-protein kinase 6	O14965	AURKA	0.046
Radix Cyathulae（RC）	MOL000098	quercetin	Thyroid hormone receptor beta-1	P10828	THRB	0.046
Radix Cyathulae（RC）	MOL000098	quercetin	Egl nine homolog 1	Q9GZT9	EGLN1	0.046
Radix Cyathulae（RC）	MOL000098	quercetin	Tubulin beta-1 chain	Q9H4B7	TUBB1	0.046
Radix Cyathulae（RC）	MOL000098	quercetin	Cannabinoid receptor 1	P21554	CNR1	0.047
Radix Cyathulae（RC）	MOL000098	quercetin	D(1A) dopamine receptor	P21728	DRD1	0.047
Radix Cyathulae（RC）	MOL000098	quercetin	D(1B) dopamine receptor	P21918	DRD5	0.047
Radix Cyathulae（RC）	MOL000098	quercetin	Endothelin-1 receptor	P25101	EDNRA	0.047
Radix Cyathulae（RC）	MOL000098	quercetin	Thiamin pyrophosphokinase 1	Q9H3S4	TPK1	0.047
Radix Cyathulae（RC）	MOL000098	quercetin	Glucocorticoid receptor	P04150	NR3C1	0.048
Radix Cyathulae（RC）	MOL000098	quercetin	Ig kappa chain V-II region RPMI 6410	P06310	IGKV2-30	0.048
Radix Cyathulae（RC）	MOL000098	quercetin	Leukotriene A-4 hydrolase	P09960	LTA4H	0.048
Radix Cyathulae（RC）	MOL000098	quercetin	RAC-alpha serine/threonine-protein kinase	P31749	AKT1	0.048
Radix Cyathulae（RC）	MOL000098	quercetin	Nitric oxide synthase, inducible	P35228	NOS2	0.048
Radix Cyathulae（RC）	MOL000098	quercetin	S-methyl-5-thioadenosine phosphorylase	Q13126	MTAP	0.048
Radix Cyathulae（RC）	MOL000098	quercetin	cAMP-specific 3',5'-cyclic phosphodiesterase 4A	P27815	PDE4A	0.049
Radix Cyathulae（RC）	MOL000098	quercetin	Triosephosphate isomerase	P60174	TPI1	0.049
Radix Cyathulae（RC）	MOL000098	quercetin	Solute carrier family 12 member 1	Q13621	SLC12A1	0.049
Radix Cyathulae（RC）	MOL000098	quercetin	Thyroid hormone receptor alpha	P10827	THRA	0.05

Radix Cyathulae（RC）	MOL000098	quercetin	Calmodulin	P62158		0.05
Radix Cyathulae（RC）	MOL000098	quercetin	Casein kinase II subunit alpha	P68400	CSNK2A1	0.05
Radix Cyathulae（RC）	MOL000098	quercetin	Neuropeptide Y	P01303	NPY	0.051
Radix Cyathulae（RC）	MOL000098	quercetin	Peroxisome proliferator-activated receptor gamma	P37231	PPARG	0.051
Radix Cyathulae（RC）	MOL000098	quercetin	Nuclear receptor coactivator 2	Q15596	NCOA2	0.051
Radix Cyathulae（RC）	MOL000098	quercetin	Toll-like receptor 7	Q9NYK1	TLR7	0.051
Radix Cyathulae（RC）	MOL000098	quercetin	C-jun-amino-terminal kinase-interacting protein 1	Q9UQF2	MAPK8IP1	0.051
Radix Cyathulae（RC）	MOL000098	quercetin	cAMP-dependent protein kinase inhibitor alpha	P61925	PKIA	0.053
Radix Cyathulae（RC）	MOL000098	quercetin	Interferon gamma	P01579	IFNG	0.055
Radix Cyathulae（RC）	MOL000098	quercetin	Fibroblast growth factor receptor 2	P21802	FGFR2	0.055
Radix Cyathulae（RC）	MOL000098	quercetin	Sodium-dependent noradrenaline transporter	P23975	SLC6A2	0.055
Radix Cyathulae（RC）	MOL000098	quercetin	MAP kinase-activated protein kinase 2	P49137	MAPKAPK2	0.055
Radix Cyathulae（RC）	MOL000098	quercetin	Glycogen synthase kinase-3 beta	P49841	GSK3B	0.055
Radix Cyathulae（RC）	MOL000098	quercetin	Sodium-dependent dopamine transporter	Q01959	SLC6A3	0.055
Radix Cyathulae（RC）	MOL000098	quercetin	Nuclear receptor coactivator 5	Q9HCD5	NCOA5	0.055
Radix Cyathulae（RC）	MOL000098	quercetin	Carbonic anhydrase 2	P00918	CA2	0.056
Radix Cyathulae（RC）	MOL000098	quercetin	Alpha-2B adrenergic receptor	P18089	ADRA2B	0.056
Radix Cyathulae（RC）	MOL000098	quercetin	Proto-oncogene tyrosine-protein kinase Src	P12931	SRC	0.057
Radix Cyathulae（RC）	MOL000098	quercetin	DNA polymerase kappa	Q9UBT6	POLK	0.058
Radix Cyathulae（RC）	MOL000098	quercetin	DNA topoisomerase 2-alpha	P11388	TOP2A	0.059
Radix Cyathulae（RC）	MOL000098	quercetin	3 beta-hydroxysteroid dehydrogenase/Delta 5-->4-isomerase type I	P14060	HSD3B1	0.061
Radix Cyathulae（RC）	MOL000098	quercetin	Beta-2 adrenergic receptor	P07550	ADRB2	0.066
Radix Cyathulae（RC）	MOL000098	quercetin	Beta-1 adrenergic receptor	P08588	ADRB1	0.066
Radix Cyathulae（RC）	MOL000098	quercetin	Tubulin alpha-3 chain	Q71U36	TUBA1A	0.067
Radix Cyathulae（RC）	MOL000098	quercetin	Prothrombin	P00734	F2	0.07
Radix Cyathulae（RC）	MOL000098	quercetin	Estradiol 17-beta-dehydrogenase 1	P14061	HSD17B1	0.071
Radix Cyathulae（RC）	MOL000098	quercetin	Delta-type opioid receptor	P41143	OPRD1	0.073
Radix Cyathulae（RC）	MOL000098	quercetin	Kappa-type opioid receptor	P41145	OPRK1	0.073
Radix Cyathulae（RC）	MOL000098	quercetin	D-HSCDK2	O75100	CA11	0.075
Radix Cyathulae（RC）	MOL000098	quercetin	Sodium-dependent serotonin transporter	P31645	SLC6A4	0.075
Radix Cyathulae（RC）	MOL000098	quercetin	Mineralocorticoid receptor	P08235	NR3C2	0.077
Radix Cyathulae（RC）	MOL000098	quercetin	Inhibitor of nuclear factor kappa-B kinase subunit alpha	O15111	CHUK	0.079
Radix Cyathulae（RC）	MOL000098	quercetin	Arachidonate 5-lipoxygenase	P09917	ALOX5	0.079
Radix Cyathulae（RC）	MOL000098	quercetin	cAMP-dependent protein kinase catalytic subunit alpha	P17612	PRKACA	0.083
Radix Cyathulae（RC）	MOL000098	quercetin	Dihydrofolate reductase	P00374	DHFR	0.091
Radix Cyathulae（RC）	MOL000098	quercetin	Cell division control protein 2 homolog	P06493	CDK1	0.095
Radix Cyathulae（RC）	MOL000098	quercetin	Cell division protein kinase 5	Q00535	CDK5	0.115
Radix Cyathulae（RC）	MOL000098	quercetin	Trypsin-1	P07477	PRSS1	0.138
Radix Cyathulae（RC）	MOL000098	quercetin	Cyclin-A2	P20248	CCNA2	0.151
Radix Cyathulae（RC）	MOL000098	quercetin	Cell division protein kinase 2	P24941	CDK2	0.156
Radix Cyathulae（RC）	MOL000098	quercetin	Hemoglobin subunit alpha	P69905	HBA1	0.169
Radix Cyathulae（RC）	MOL000098	quercetin	Mu-type opioid receptor	P35372	OPRM1	0.179
Radix Cyathulae（RC）	MOL000098	quercetin	Progesterone receptor	P06401	PGR	0.2
Radix Cyathulae（RC）	MOL000098	quercetin	Nuclear receptor coactivator 1	Q15788	NCOA1	0.205
Radix Cyathulae（RC）	MOL000098	quercetin	Prostaglandin G/H synthase 1	P23219	PTGS1	0.22
Radix Cyathulae（RC）	MOL000098	quercetin	Estrogen receptor beta	Q92731	ESR2	0.357
Radix Cyathulae（RC）	MOL000098	quercetin	Prostaglandin G/H synthase 2	P35354	PTGS2	0.39

Radix Cyathulae（RC）	MOL000098	quercetin	Estrogen receptor	P03372	ESR1	1
Radix Cyathulae（RC）	MOL000358	beta-sitosterol	Retinoic acid receptor RXR-gamma	P48443	RXRG	0.01
Radix Cyathulae（RC）	MOL000358	beta-sitosterol	Nuclear receptor 0B1	P51843	NR0B1	0.01
Radix Cyathulae（RC）	MOL000358	beta-sitosterol	Retinoic acid-induced protein 3	Q8NFJ5	GPRC5A	0.01
Radix Cyathulae（RC）	MOL000358	beta-sitosterol	Retinoic acid receptor alpha	P10276	RARA	0.023
Radix Cyathulae（RC）	MOL000358	beta-sitosterol	Retinoic acid receptor beta	P10826	RARB	0.023
Radix Cyathulae（RC）	MOL000358	beta-sitosterol	Retinal dehydrogenase 2	O94788	ALDH1A2	0.029
Radix Cyathulae（RC）	MOL000358	beta-sitosterol	Retinal dehydrogenase 1	P00352	ALDH1A1	0.029
Radix Cyathulae（RC）	MOL000358	beta-sitosterol	Potassium channel subfamily K member 1	O00180	KCNK1	0.036
Radix Cyathulae（RC）	MOL000358	beta-sitosterol	Sodium channel protein type 5 subunit alpha	Q14524	SCN5A	0.036
Radix Cyathulae（RC）	MOL000358	beta-sitosterol	Potassium channel subfamily K member 6	Q9Y257	KCNK6	0.036
Radix Cyathulae（RC）	MOL000358	beta-sitosterol	Serine/threonine-protein phosphatase 2A 65 kDa regulatory subunit A alpha isoform	P30153	PPP2R1A	0.038
Radix Cyathulae（RC）	MOL000358	beta-sitosterol	Serine/threonine-protein phosphatase PP1-alpha catalytic subunit	P62136	PPP1CA	0.038
Radix Cyathulae（RC）	MOL000358	beta-sitosterol	Ig kappa chain C region	P01834	IGKC	0.043
Radix Cyathulae（RC）	MOL000358	beta-sitosterol	Ig gamma-1 chain C region	P01857	IGHG1	0.043
Radix Cyathulae（RC）	MOL000358	beta-sitosterol	Ig gamma-2 chain C region	P01859	IGHG2	0.043
Radix Cyathulae（RC）	MOL000358	beta-sitosterol	Nuclear receptor subfamily 1 group I member 3	Q14994	NR1I3	0.05
Radix Cyathulae（RC）	MOL000358	beta-sitosterol	Retinoic acid receptor gamma-1	P13631	RARG	0.052
Radix Cyathulae（RC）	MOL000358	beta-sitosterol	Retinoic acid receptor RXR-beta	P28702	RXRB	0.052
Radix Cyathulae（RC）	MOL000358	beta-sitosterol	Bile salt sulfotransferase	Q06520	SULT2A1	0.056
Radix Cyathulae（RC）	MOL000358	beta-sitosterol	Dehydrogenase/reductase SDR family member 8	Q8NBQ5	HSD17B11	0.056
Radix Cyathulae（RC）	MOL000358	beta-sitosterol	Platelet glycoprotein IX	P14770	GP9	0.063
Radix Cyathulae（RC）	MOL000358	beta-sitosterol	Phospholipase A2	P04054	PLA2G1B	0.069
Radix Cyathulae（RC）	MOL000358	beta-sitosterol	Phospholipase A2, membrane associated	P14555	PLA2G2A	0.069
Radix Cyathulae（RC）	MOL000358	beta-sitosterol	3-oxo-5-alpha-steroid 4-dehydrogenase 1	P18405	SRD5A1	0.074
Radix Cyathulae（RC）	MOL000358	beta-sitosterol	3-oxo-5-alpha-steroid 4-dehydrogenase 2	P31213	SRD5A2	0.074
Radix Cyathulae（RC）	MOL000358	beta-sitosterol	3 beta-hydroxysteroid dehydrogenase/Delta 5-->4-isomerase type II	P26439	HSD3B2	0.079
Radix Cyathulae（RC）	MOL000358	beta-sitosterol	Microtubule-associated protein 2	P11137	MAP2	0.08
Radix Cyathulae（RC）	MOL000358	beta-sitosterol	Microtubule-associated protein 1A	P78559	MAP1A	0.08
Radix Cyathulae（RC）	MOL000358	beta-sitosterol	Gonadotropin-releasing hormone receptor	P30968	GNRHR	0.082
Radix Cyathulae（RC）	MOL000358	beta-sitosterol	Gonadotropin-releasing hormone II receptor	Q96P88	GNRHR2	0.082
Radix Cyathulae（RC）	MOL000358	beta-sitosterol	Corticosteroid 11-beta-dehydrogenase isozyme 1	P28845	HSD11B1	0.084
Radix Cyathulae（RC）	MOL000358	beta-sitosterol	Cytosolic phospholipase A2	P47712	PLA2G4A	0.084
Radix Cyathulae（RC）	MOL000358	beta-sitosterol	Retinoic acid receptor RXR-alpha	P19793	RXRA	0.092
Radix Cyathulae（RC）	MOL000358	beta-sitosterol	Serine/threonine-protein phosphatase 2A catalytic subunit alpha isoform	P67775	PPP2CA	0.095
Radix Cyathulae（RC）	MOL000358	beta-sitosterol	Serine/threonine-protein phosphatase 2A 56 kDa regulatory subunit gamma isoform	Q13362	PPP2R5C	0.095
Radix Cyathulae（RC）	MOL000358	beta-sitosterol	Sodium-dependent serotonin transporter	P31645	SLC6A4	0.146
Radix Cyathulae（RC）	MOL000358	beta-sitosterol	Aldo-keto reductase family 1 member C1	Q04828	AKR1C1	0.147
Radix Cyathulae（RC）	MOL000358	beta-sitosterol	Prostaglandin G/H synthase 2	P35354	PTGS2	0.15
Radix Cyathulae（RC）	MOL000358	beta-sitosterol	Estrogen receptor beta	Q92731	ESR2	0.163
Radix Cyathulae（RC）	MOL000358	beta-sitosterol	Mediator of RNA polymerase II transcription subunit 1	Q15648	MED1	0.164
Radix Cyathulae（RC）	MOL000358	beta-sitosterol	Estradiol 17-beta-dehydrogenase 1	P14061	HSD17B1	0.173
Radix Cyathulae（RC）	MOL000358	beta-sitosterol	3 beta-hydroxysteroid dehydrogenase/Delta 5-->4-isomerase type I	P14060	HSD3B1	0.175
Radix Cyathulae（RC）	MOL000358	beta-sitosterol	Nuclear receptor coactivator 5	Q9HCD5	NCOA5	0.178
Radix Cyathulae（RC）	MOL000358	beta-sitosterol	Androgen receptor	P10275	AR	0.184
Radix Cyathulae（RC）	MOL000358	beta-sitosterol	Glucocorticoid receptor	P04150	NR3C1	0.187
Radix Cyathulae（RC）	MOL000358	beta-sitosterol	Cannabinoid receptor 2	P34972	CNR2	0.191

Radix Cyathulae（RC）	MOL000358	beta-sitosterol	Nuclear receptor coactivator 1	Q15788	NCOA1	0.296
Radix Cyathulae（RC）	MOL000358	beta-sitosterol	Mineralocorticoid receptor	P08235	NR3C2	0.39
Radix Cyathulae（RC）	MOL000358	beta-sitosterol	Estrogen receptor	P03372	ESR1	0.971
Radix Cyathulae（RC）	MOL000358	beta-sitosterol	Progesterone receptor	P06401	PGR	1
Radix Cyathulae（RC）	MOL002212	ecdysterone	Superoxide dismutase [Cu-Zn]	P00441	SOD1	Validated
Radix Cyathulae（RC）	MOL012286	Betavulgarin	2,4-dienoyl-CoA reductase, mitochondrial	Q16698	DECR1	0.01
Radix Cyathulae（RC）	MOL012286	Betavulgarin	Chromaffin granule amine transporter	P54219	SLC18A1	0.011
Radix Cyathulae（RC）	MOL012286	Betavulgarin	5-hydroxytryptamine 4 receptor	Q13639	HTR4	0.011
Radix Cyathulae（RC）	MOL012286	Betavulgarin	Pepsin A	P00790	REN	0.012
Radix Cyathulae（RC）	MOL012286	Betavulgarin	Alanine aminotransferase 1	P24298	GPT	0.012
Radix Cyathulae（RC）	MOL012286	Betavulgarin	Amine oxidase [flavin-containing] B	P27338	MAOB	0.012
Radix Cyathulae（RC）	MOL012286	Betavulgarin	Dipeptidyl peptidase 4	P27487	DPP4	0.012
Radix Cyathulae（RC）	MOL012286	Betavulgarin	Nociceptin receptor	P41146	OPRL1	0.012
Radix Cyathulae（RC）	MOL012286	Betavulgarin	4-aminobutyrate aminotransferase, mitochondrial	P80404	ABAT	0.012
Radix Cyathulae（RC）	MOL012286	Betavulgarin	Glutamate [NMDA] receptor subunit zeta-1	Q05586	GRIN1	0.012
Radix Cyathulae（RC）	MOL012286	Betavulgarin	Glutamate [NMDA] receptor subunit epsilon-1	Q12879	GRIN2A	0.012
Radix Cyathulae（RC）	MOL012286	Betavulgarin	Glutamate [NMDA] receptor subunit epsilon-2	Q13224	GRIN2B	0.012
Radix Cyathulae（RC）	MOL012286	Betavulgarin	Glutamate [NMDA] receptor subunit epsilon-3	Q14957	GRIN2C	0.012
Radix Cyathulae（RC）	MOL012286	Betavulgarin	Membrane copper amine oxidase	Q16853	AOC3	0.012
Radix Cyathulae（RC）	MOL012286	Betavulgarin	Alanine aminotransferase 2	Q8TD30	GPT2	0.012
Radix Cyathulae（RC）	MOL012286	Betavulgarin	Cytochrome b-c1 complex subunit 8	O14949	UQCRQ	0.013
Radix Cyathulae（RC）	MOL012286	Betavulgarin	Cytochrome b-c1 complex subunit 10	O14957	UQCR11	0.013
Radix Cyathulae（RC）	MOL012286	Betavulgarin	Cytochrome b	P00156	MT-CYB	0.013
Radix Cyathulae（RC）	MOL012286	Betavulgarin	Cytochrome b-c1 complex subunit 6, mitochondrial	P07919	UQCRH	0.013
Radix Cyathulae（RC）	MOL012286	Betavulgarin	Cytochrome c1, heme protein, mitochondrial	P08574	CYC1	0.013
Radix Cyathulae（RC）	MOL012286	Betavulgarin	Cytochrome b-c1 complex subunit 2, mitochondrial	P22695	UQCRC2	0.013
Radix Cyathulae（RC）	MOL012286	Betavulgarin	Ubiquinol-cytochrome-c reductase complex core protein 1, mitochondrial	P31930	UQCRC1	0.013
Radix Cyathulae（RC）	MOL012286	Betavulgarin	Cytochrome b-c1 complex subunit Rieske, mitochondrial	P47985	UQCRFS1	0.013
Radix Cyathulae（RC）	MOL012286	Betavulgarin	Glutamate [NMDA] receptor subunit 3A	Q8TCU5	GRIN3A	0.013
Radix Cyathulae（RC）	MOL012286	Betavulgarin	Cytochrome b-c1 complex subunit 9	Q9UDW1	UQCR10	0.013
Radix Cyathulae（RC）	MOL012286	Betavulgarin	Potassium channel subfamily K member 6	Q9Y257	KCNK6	0.013
Radix Cyathulae（RC）	MOL012286	Betavulgarin	Neuronal acetylcholine receptor subunit alpha-3	P32297	CHRNA3	0.015
Radix Cyathulae（RC）	MOL012286	Betavulgarin	Ig kappa chain C region	P01834	IGKC	0.017
Radix Cyathulae（RC）	MOL012286	Betavulgarin	Ig gamma-1 chain C region	P01857	IGHG1	0.017
Radix Cyathulae（RC）	MOL012286	Betavulgarin	Ig gamma-2 chain C region	P01859	IGHG2	0.017
Radix Cyathulae（RC）	MOL012286	Betavulgarin	Acetylcholinesterase	P22303	ACHE	0.017
Radix Cyathulae（RC）	MOL012286	Betavulgarin	Stromelysin-1	P08254	MMP3	0.018
Radix Cyathulae（RC）	MOL012286	Betavulgarin	Interleukin-3	P08700	IL3	0.018
Radix Cyathulae（RC）	MOL012286	Betavulgarin	Stromelysin-2	P09238	MMP10	0.018
Radix Cyathulae（RC）	MOL012286	Betavulgarin	Adenosine A2a receptor	P29274	ADORA2A	0.018
Radix Cyathulae（RC）	MOL012286	Betavulgarin	Adenosine A2b receptor	P29275	ADORA2B	0.018
Radix Cyathulae（RC）	MOL012286	Betavulgarin	Adenosine A3 receptor	P33765	SHMT1	0.018
Radix Cyathulae（RC）	MOL012286	Betavulgarin	Macrophage metalloelastase	P39900	MMP12	0.018
Radix Cyathulae（RC）	MOL012286	Betavulgarin	Neuronal acetylcholine receptor subunit alpha-4	P43681	CHRNA4	0.018
Radix Cyathulae（RC）	MOL012286	Betavulgarin	Protein S100-A12	P80511	S100A12	0.018
Radix Cyathulae（RC）	MOL012286	Betavulgarin	Protein S100-A13	Q99584	S100A13	0.018
Radix Cyathulae（RC）	MOL012286	Betavulgarin	Gamma-aminobutyric acid receptor subunit rho-3	A8MPY1	GABRR3	0.019

Radix Cyathulae（RC）	MOL012286	Betavulgarin	Gamma-aminobutyric acid receptor subunit pi	O00591	GABRP	0.019
Radix Cyathulae（RC）	MOL012286	Betavulgarin	Gamma-aminobutyric acid receptor subunit delta	O14764	GABRD	0.019
Radix Cyathulae（RC）	MOL012286	Betavulgarin	Gamma-aminobutyric-acid receptor subunit beta-1	P18505	GABRB1	0.019
Radix Cyathulae（RC）	MOL012286	Betavulgarin	Gamma-aminobutyric acid receptor subunit gamma-2	P18507	GABRG2	0.019
Radix Cyathulae（RC）	MOL012286	Betavulgarin	Gamma-aminobutyric-acid receptor subunit rho-1	P24046	GABRR1	0.019
Radix Cyathulae（RC）	MOL012286	Betavulgarin	Gamma-aminobutyric-acid receptor subunit beta-3	P28472	GABRB3	0.019
Radix Cyathulae（RC）	MOL012286	Betavulgarin	Gamma-aminobutyric acid receptor subunit rho-2	P28476	GABRR2	0.019
Radix Cyathulae（RC）	MOL012286	Betavulgarin	Amiloride-sensitive sodium channel subunit alpha	P37088	SCNN1A	0.019
Radix Cyathulae（RC）	MOL012286	Betavulgarin	Gamma-aminobutyric-acid receptor subunit beta-2	P47870	GABRB2	0.019
Radix Cyathulae（RC）	MOL012286	Betavulgarin	Gamma-aminobutyric-acid receptor subunit alpha-4	P48169	GABRA4	0.019
Radix Cyathulae（RC）	MOL012286	Betavulgarin	Amiloride-sensitive sodium channel subunit beta	P51168	SCNN1B	0.019
Radix Cyathulae（RC）	MOL012286	Betavulgarin	Amiloride-sensitive sodium channel subunit gamma	P51170	SCNN1G	0.019
Radix Cyathulae（RC）	MOL012286	Betavulgarin	Gamma-aminobutyric acid receptor subunit epsilon	P78334	GABRE	0.019
Radix Cyathulae（RC）	MOL012286	Betavulgarin	Gamma-aminobutyric-acid receptor subunit alpha-6	Q16445	GABRA6	0.019
Radix Cyathulae（RC）	MOL012286	Betavulgarin	Gamma-aminobutyric acid receptor subunit gamma-1	Q8N1C3	GABRG1	0.019
Radix Cyathulae（RC）	MOL012286	Betavulgarin	Gamma-aminobutyric acid receptor subunit gamma-3	Q99928	GABRG3	0.019
Radix Cyathulae（RC）	MOL012286	Betavulgarin	Arachidonate 5-lipoxygenase	P09917	ALOX5	0.022
Radix Cyathulae（RC）	MOL012286	Betavulgarin	Aryl hydrocarbon receptor	P35869	AHR	0.022
Radix Cyathulae（RC）	MOL012286	Betavulgarin	Dihydroorotate dehydrogenase, mitochondrial	Q02127	DHODH	0.022
Radix Cyathulae（RC）	MOL012286	Betavulgarin	Serine/threonine-protein kinase 17B	O94768	STK17B	0.023
Radix Cyathulae（RC）	MOL012286	Betavulgarin	Alpha-1A adrenergic receptor	P35348	ADRA1A	0.023
Radix Cyathulae（RC）	MOL012286	Betavulgarin	Alpha-1B adrenergic receptor	P35368	ADRA1B	0.023
Radix Cyathulae（RC）	MOL012286	Betavulgarin	ATP synthase subunit gamma, mitochondrial	P36542	ATP5F1C	0.023
Radix Cyathulae（RC）	MOL012286	Betavulgarin	Bile salt sulfotransferase	Q06520	SULT2A1	0.023
Radix Cyathulae（RC）	MOL012286	Betavulgarin	UDP-glucuronosyltransferase 3A1	Q6NUS8	UGT3A1	0.023
Radix Cyathulae（RC）	MOL012286	Betavulgarin	Glycogen phosphorylase, muscle form	P11217	PYGM	0.024
Radix Cyathulae（RC）	MOL012286	Betavulgarin	Cell division protein kinase 4	P11802	CDK4	0.024
Radix Cyathulae（RC）	MOL012286	Betavulgarin	Cell division protein kinase 7	P50613	CDK7	0.024
Radix Cyathulae（RC）	MOL012286	Betavulgarin	Cell division protein kinase 9	P50750	CDK9	0.024
Radix Cyathulae（RC）	MOL012286	Betavulgarin	Cell division protein kinase 6	Q00534	CDK6	0.024
Radix Cyathulae（RC）	MOL012286	Betavulgarin	Cyclin-dependent kinase 5 activator 1	Q15078	CDK5R1	0.024
Radix Cyathulae（RC）	MOL012286	Betavulgarin	Coagulation factor IX	P00740	F9	0.025
Radix Cyathulae（RC）	MOL012286	Betavulgarin	Coagulation factor X	P00742	F10	0.025
Radix Cyathulae（RC）	MOL012286	Betavulgarin	Tumor necrosis factor	P01375	TNF	0.025
Radix Cyathulae（RC）	MOL012286	Betavulgarin	Osteocalcin	P02818	BGLAP	0.025
Radix Cyathulae（RC）	MOL012286	Betavulgarin	Vitamin K-dependent protein C	P04070	PROC	0.025
Radix Cyathulae（RC）	MOL012286	Betavulgarin	NAD(P)H dehydrogenase [quinone] 1	P15559	NQO1	0.025
Radix Cyathulae（RC）	MOL012286	Betavulgarin	Nuclear factor NF-kappa-B p105 subunit	P19838	NFKB1	0.025
Radix Cyathulae（RC）	MOL012286	Betavulgarin	Fibroblast growth factor receptor 2	P21802	FGFR2	0.025
Radix Cyathulae（RC）	MOL012286	Betavulgarin	Vitamin K-dependent protein Z	P22891	PROZ	0.025
Radix Cyathulae（RC）	MOL012286	Betavulgarin	Vitamin K-dependent gamma-carboxylase	P38435	GGCX	0.025
Radix Cyathulae（RC）	MOL012286	Betavulgarin	Vitamin K epoxide reductase complex subunit 1-like protein 1	Q8N0U8	VKORC1L1	0.025
Radix Cyathulae（RC）	MOL012286	Betavulgarin	Vitamin K epoxide reductase complex subunit 1	Q9BQB6	VKORC1	0.025
Radix Cyathulae（RC）	MOL012286	Betavulgarin	T-cell receptor alpha chain C region	P01848	TRAC	0.026
Radix Cyathulae（RC）	MOL012286	Betavulgarin	T-cell receptor beta chain C region	P01850	TRBC1	0.026
Radix Cyathulae（RC）	MOL012286	Betavulgarin	Beta-2-microglobulin	P61769	B2M	0.026
Radix Cyathulae（RC）	MOL012286	Betavulgarin	Synaptic vesicular amine transporter	Q05940	SLC18A2	0.026

Radix Cyathulae（RC）	MOL012286	Betavulgarin	Neuronal acetylcholine receptor subunit alpha-7	P36544	CHRNA7	0.027
Radix Cyathulae（RC）	MOL012286	Betavulgarin	Potassium channel subfamily K member 1	O00180	KCNK1	0.028
Radix Cyathulae（RC）	MOL012286	Betavulgarin	Tripartite motif-containing protein 13	O60858	TRIM13	0.029
Radix Cyathulae（RC）	MOL012286	Betavulgarin	Carboxypeptidase A1	P15085	CPA1	0.029
Radix Cyathulae（RC）	MOL012286	Betavulgarin	Peptidyl-prolyl cis-trans isomerase, mitochondrial	P30405	PPIF	0.029
Radix Cyathulae（RC）	MOL012286	Betavulgarin	Delta-type opioid receptor	P41143	OPRD1	0.029
Radix Cyathulae（RC）	MOL012286	Betavulgarin	Methionine aminopeptidase 1	P53582	METAP1	0.029
Radix Cyathulae（RC）	MOL012286	Betavulgarin	Tubulin beta-2C chain	P68371	TUBB4B	0.029
Radix Cyathulae（RC）	MOL012286	Betavulgarin	Tubulin alpha-3 chain	Q71U36	TUBA1A	0.029
Radix Cyathulae（RC）	MOL012286	Betavulgarin	Urokinase-type plasminogen activator	P00749	PLAU	0.03
Radix Cyathulae（RC）	MOL012286	Betavulgarin	Myosin light chain 6B	P14649	MYL6B	0.03
Radix Cyathulae（RC）	MOL012286	Betavulgarin	Myosin regulatory light chain 12A	P19105	MYL12A	0.03
Radix Cyathulae（RC）	MOL012286	Betavulgarin	5-hydroxytryptamine 3 receptor	P46098	HTR3A	0.03
Radix Cyathulae（RC）	MOL012286	Betavulgarin	Casein kinase I isoform gamma-2	P78368	CSNK1G2	0.03
Radix Cyathulae（RC）	MOL012286	Betavulgarin	Calcium/calmodulin-dependent protein kinase type II subunit delta	Q13557	CAMK2D	0.03
Radix Cyathulae（RC）	MOL012286	Betavulgarin	Serine/threonine-protein kinase haspin	Q8TF76	HASPIN	0.03
Radix Cyathulae（RC）	MOL012286	Betavulgarin	STE20-like serine/threonine-protein kinase	Q9H2G2	SLK	0.03
Radix Cyathulae（RC）	MOL012286	Betavulgarin	Tryptophanyl-tRNA synthetase, mitochondrial	Q9UGM6	WARS2	0.03
Radix Cyathulae（RC）	MOL012286	Betavulgarin	DNA-(apurinic or apyrimidinic site) lyase	P27695	APEX1	0.031
Radix Cyathulae（RC）	MOL012286	Betavulgarin	Retinoic acid receptor gamma-1	P13631	RARG	0.032
Radix Cyathulae（RC）	MOL012286	Betavulgarin	Retinoic acid receptor RXR-beta	P28702	RXRB	0.032
Radix Cyathulae（RC）	MOL012286	Betavulgarin	Tyrosyl-tRNA synthetase, cytoplasmic	P54577	YARS	0.032
Radix Cyathulae（RC）	MOL012286	Betavulgarin	Peptidyl-prolyl cis-trans isomerase NIMA-interacting 1	Q13526	PIN1	0.032
Radix Cyathulae（RC）	MOL012286	Betavulgarin	Myeloperoxidase	P05164	MPO	0.033
Radix Cyathulae（RC）	MOL012286	Betavulgarin	Eosinophil peroxidase	P11678	EPX	0.033
Radix Cyathulae（RC）	MOL012286	Betavulgarin	Calreticulin	P27797	CALR	0.033
Radix Cyathulae（RC）	MOL012286	Betavulgarin	Melatonin receptor type 1A	P48039	MTNR1A	0.033
Radix Cyathulae（RC）	MOL012286	Betavulgarin	Melatonin receptor type 1B	P49286	MTNR1B	0.033
Radix Cyathulae（RC）	MOL012286	Betavulgarin	Nuclear receptor ROR-beta	Q92753	RORB	0.033
Radix Cyathulae（RC）	MOL012286	Betavulgarin	Sodium channel protein type 10 subunit alpha	Q9Y5Y9	SCN10A	0.033
Radix Cyathulae（RC）	MOL012286	Betavulgarin	Proto-oncogene tyrosine-protein kinase LCK	P06239	LCK	0.034
Radix Cyathulae（RC）	MOL012286	Betavulgarin	Tyrosine-protein kinase Lyn	P07948	LYN	0.034
Radix Cyathulae（RC）	MOL012286	Betavulgarin	5-hydroxytryptamine 1D receptor	P28221	HTR1D	0.036
Radix Cyathulae（RC）	MOL012286	Betavulgarin	Tubulin alpha-1 chain	P68366	TUBA4A	0.036
Radix Cyathulae（RC）	MOL012286	Betavulgarin	D(4) dopamine receptor	P21917	DRD4	0.037
Radix Cyathulae（RC）	MOL012286	Betavulgarin	D(3) dopamine receptor	P35462	DRD3	0.037
Radix Cyathulae（RC）	MOL012286	Betavulgarin	Casein kinase II subunit alpha	P68400	CSNK2A1	0.037
Radix Cyathulae（RC）	MOL012286	Betavulgarin	5-hydroxytryptamine 2B receptor	P41595	HTR2B	0.038
Radix Cyathulae（RC）	MOL012286	Betavulgarin	Carbonic anhydrase 4	P22748	CA4	0.039
Radix Cyathulae（RC）	MOL012286	Betavulgarin	5-hydroxytryptamine 1A receptor	P08908	HTR1A	0.04
Radix Cyathulae（RC）	MOL012286	Betavulgarin	Mitogen-activated protein kinase 8	P45983	MAPK8	0.04
Radix Cyathulae（RC）	MOL012286	Betavulgarin	Mitogen-activated protein kinase 10	P53779	MAPK10	0.04
Radix Cyathulae（RC）	MOL012286	Betavulgarin	Retinoic acid receptor RXR-alpha	P19793	RXRA	0.041
Radix Cyathulae（RC）	MOL012286	Betavulgarin	Nuclear receptor subfamily 1 group I member 3	Q14994	NR1I3	0.041
Radix Cyathulae（RC）	MOL012286	Betavulgarin	Sodium-dependent noradrenaline transporter	P23975	SLC6A2	0.042
Radix Cyathulae（RC）	MOL012286	Betavulgarin	Protein tyrosine kinase 2 beta	Q14289	PTK2B	0.042
Radix Cyathulae（RC）	MOL012286	Betavulgarin	Microtubule-associated protein 2	P11137	MAP2	0.043

Radix Cyathulae（RC）	MOL012286	Betavulgarin	Estrogen-related receptor gamma	P62508	ESRRG	0.043
Radix Cyathulae（RC）	MOL012286	Betavulgarin	Microtubule-associated protein 1A	P78559	MAP1A	0.043
Radix Cyathulae（RC）	MOL012286	Betavulgarin	Mineralocorticoid receptor	P08235	NR3C2	0.045
Radix Cyathulae（RC）	MOL012286	Betavulgarin	Androgen receptor	P10275	AR	0.045
Radix Cyathulae（RC）	MOL012286	Betavulgarin	Amine oxidase [flavin-containing] A	P21397	MAOA	0.045
Radix Cyathulae（RC）	MOL012286	Betavulgarin	Sodium channel protein type 5 subunit alpha	Q14524	SCN5A	0.046
Radix Cyathulae（RC）	MOL012286	Betavulgarin	Platelet glycoprotein IX	P14770	GP9	0.047
Radix Cyathulae（RC）	MOL012286	Betavulgarin	Aldo-keto reductase family 1 member C3	P42330	AKR1C3	0.047
Radix Cyathulae（RC）	MOL012286	Betavulgarin	5-hydroxytryptamine 1B receptor	P28222	HTR1B	0.05
Radix Cyathulae（RC）	MOL012286	Betavulgarin	Nitric-oxide synthase, brain	P29475	NOS1	0.05
Radix Cyathulae（RC）	MOL012286	Betavulgarin	ATP synthase subunit beta, mitochondrial	P06576	ATP5F1B	0.051
Radix Cyathulae（RC）	MOL012286	Betavulgarin	Tyrosine-protein kinase HCK	P08631	HCK	0.051
Radix Cyathulae（RC）	MOL012286	Betavulgarin	Alpha-2A adrenergic receptor	P08913	ADRA2A	0.051
Radix Cyathulae（RC）	MOL012286	Betavulgarin	Proto-oncogene serine/threonine-protein kinase Pim-1	P11309	PIM1	0.051
Radix Cyathulae（RC）	MOL012286	Betavulgarin	Estradiol 17-beta-dehydrogenase 1	P14061	HSD17B1	0.051
Radix Cyathulae（RC）	MOL012286	Betavulgarin	Alpha-2C adrenergic receptor	P18825	ADRA2C	0.051
Radix Cyathulae（RC）	MOL012286	Betavulgarin	D(1B) dopamine receptor	P21918	DRD5	0.051
Radix Cyathulae（RC）	MOL012286	Betavulgarin	ATP synthase subunit alpha, mitochondrial	P25705	ATP5F1A	0.051
Radix Cyathulae（RC）	MOL012286	Betavulgarin	RAC-beta serine/threonine-protein kinase	P31751	AKT2	0.051
Radix Cyathulae（RC）	MOL012286	Betavulgarin	cAMP-dependent protein kinase inhibitor alpha	P61925	PKIA	0.051
Radix Cyathulae（RC）	MOL012286	Betavulgarin	Phospholipase A2	P04054	PLA2G1B	0.052
Radix Cyathulae（RC）	MOL012286	Betavulgarin	5-hydroxytryptamine 2C receptor	P28335	HTR2C	0.052
Radix Cyathulae（RC）	MOL012286	Betavulgarin	Muscarinic acetylcholine receptor M4	P08173	CHRM4	0.053
Radix Cyathulae（RC）	MOL012286	Betavulgarin	Muscarinic acetylcholine receptor M3	P20309	CHRM3	0.053
Radix Cyathulae（RC）	MOL012286	Betavulgarin	Epidermal growth factor receptor	P00533	EGFR	0.054
Radix Cyathulae（RC）	MOL012286	Betavulgarin	Muscarinic acetylcholine receptor M1	P11229	CHRM1	0.054
Radix Cyathulae（RC）	MOL012286	Betavulgarin	Glycogen synthase kinase-3 beta	P49841	GSK3B	0.054
Radix Cyathulae（RC）	MOL012286	Betavulgarin	Gamma-aminobutyric-acid receptor subunit alpha-5	P31644	GABRA5	0.057
Radix Cyathulae（RC）	MOL012286	Betavulgarin	Sodium-dependent serotonin transporter	P31645	SLC6A4	0.057
Radix Cyathulae（RC）	MOL012286	Betavulgarin	DNA polymerase	P04293	UL30	0.058
Radix Cyathulae（RC）	MOL012286	Betavulgarin	Sodium-dependent dopamine transporter	Q01959	SLC6A3	0.062
Radix Cyathulae（RC）	MOL012286	Betavulgarin	Dual specificity protein kinase CLK1	P49759	CLK1	0.063
Radix Cyathulae（RC）	MOL012286	Betavulgarin	Tubulin beta-1 chain	Q9H4B7	TUBB1	0.063
Radix Cyathulae（RC）	MOL012286	Betavulgarin	Thyroid hormone receptor alpha	P10827	THRA	0.064
Radix Cyathulae（RC）	MOL012286	Betavulgarin	Mu-type opioid receptor	P35372	OPRM1	0.064
Radix Cyathulae（RC）	MOL012286	Betavulgarin	Kappa-type opioid receptor	P41145	OPRK1	0.064
Radix Cyathulae（RC）	MOL012286	Betavulgarin	Sulfotransferase 1A1	P50225	SULT1A1	0.064
Radix Cyathulae（RC）	MOL012286	Betavulgarin	Triosephosphate isomerase	P60174	TPI1	0.064
Radix Cyathulae（RC）	MOL012286	Betavulgarin	Kynurenine--oxoglutarate transaminase 1	Q16773	KYAT1	0.064
Radix Cyathulae（RC）	MOL012286	Betavulgarin	Neprilysin	P08473	MME	0.065
Radix Cyathulae（RC）	MOL012286	Betavulgarin	Alpha-2B adrenergic receptor	P18089	ADRA2B	0.065
Radix Cyathulae（RC）	MOL012286	Betavulgarin	D(1A) dopamine receptor	P21728	DRD1	0.065
Radix Cyathulae（RC）	MOL012286	Betavulgarin	Nuclear receptor coactivator 5	Q9HCD5	NCOA5	0.065
Radix Cyathulae（RC）	MOL012286	Betavulgarin	Calcium/calmodulin-dependent protein kinase type II alpha chain	Q9UQM7	CAMK2A	0.065
Radix Cyathulae（RC）	MOL012286	Betavulgarin	Trypsin-2	P07478	PRSS2	0.066
Radix Cyathulae（RC）	MOL012286	Betavulgarin	Thyroid hormone receptor beta-1	P10828	THRB	0.066
Radix Cyathulae（RC）	MOL012286	Betavulgarin	D(2) dopamine receptor	P14416	DRD2	0.066

Radix Cyathulae（RC）	MOL012286	Betavulgarin	BAG family molecular chaperone regulator 1	Q99933	BAG1	0.066
Radix Cyathulae（RC）	MOL012286	Betavulgarin	Cannabinoid receptor 2	P34972	CNR2	0.067
Radix Cyathulae（RC）	MOL012286	Betavulgarin	Potassium voltage-gated channel subfamily KQT member 1	P51787	KCNQ1	0.067
Radix Cyathulae（RC）	MOL012286	Betavulgarin	NADPH oxidase organizer 1	Q8NFA2	NOXO1	0.067
Radix Cyathulae（RC）	MOL012286	Betavulgarin	Endothelin-1 receptor	P25101	EDNRA	0.068
Radix Cyathulae（RC）	MOL012286	Betavulgarin	Neutrophil gelatinase-associated lipocalin	P80188	LCN2	0.068
Radix Cyathulae（RC）	MOL012286	Betavulgarin	DNA polymerase kappa	Q9UBT6	POLK	0.068
Radix Cyathulae（RC）	MOL012286	Betavulgarin	Retinoic acid receptor alpha	P10276	RARA	0.069
Radix Cyathulae（RC）	MOL012286	Betavulgarin	Tyrosine-protein phosphatase non-receptor type 1	P18031	PTPN1	0.069
Radix Cyathulae（RC）	MOL012286	Betavulgarin	Cannabinoid receptor 1	P21554	CNR1	0.069
Radix Cyathulae（RC）	MOL012286	Betavulgarin	Serine/threonine-protein kinase 6	O14965	AURKA	0.07
Radix Cyathulae（RC）	MOL012286	Betavulgarin	Dihydrofolate reductase	P00374	DHFR	0.07
Radix Cyathulae（RC）	MOL012286	Betavulgarin	Gamma-aminobutyric-acid receptor subunit alpha-3	P34903	GABRA3	0.07
Radix Cyathulae（RC）	MOL012286	Betavulgarin	Calmodulin	P62158		0.07
Radix Cyathulae（RC）	MOL012286	Betavulgarin	Interferon gamma	P01579	IFNG	0.071
Radix Cyathulae（RC）	MOL012286	Betavulgarin	Proto-oncogene tyrosine-protein kinase Src	P12931	SRC	0.071
Radix Cyathulae（RC）	MOL012286	Betavulgarin	S-methyl-5-thioadenosine phosphorylase	Q13126	MTAP	0.071
Radix Cyathulae（RC）	MOL012286	Betavulgarin	Muscarinic acetylcholine receptor M2	P08172	CHRM2	0.08
Radix Cyathulae（RC）	MOL012286	Betavulgarin	Liver carboxylesterase 1	P23141	CES1	0.08
Radix Cyathulae（RC）	MOL012286	Betavulgarin	Phosphatidylinositol-4,5-bisphosphate 3-kinase catalytic subunit gamma isoform	P48736	PIK3CG	0.08
Radix Cyathulae（RC）	MOL012286	Betavulgarin	Coagulation factor VII	P08709	F7	0.082
Radix Cyathulae（RC）	MOL012286	Betavulgarin	Carbonic anhydrase 1	P00915	CA1	0.083
Radix Cyathulae（RC）	MOL012286	Betavulgarin	Carbonic anhydrase 2	P00918	CA2	0.083
Radix Cyathulae（RC）	MOL012286	Betavulgarin	5-hydroxytryptamine 2A receptor	P28223	HTR2A	0.084
Radix Cyathulae（RC）	MOL012286	Betavulgarin	Peroxisome proliferator-activated receptor gamma	P37231	PPARG	0.087
Radix Cyathulae（RC）	MOL012286	Betavulgarin	Gamma-aminobutyric-acid receptor subunit alpha-2	P47869	GABRA2	0.098
Radix Cyathulae（RC）	MOL012286	Betavulgarin	Ribosyldihydronicotinamide dehydrogenase [quinone]	P16083	NQO2	0.107
Radix Cyathulae（RC）	MOL012286	Betavulgarin	Beta-2 adrenergic receptor	P07550	ADRB2	0.109
Radix Cyathulae（RC）	MOL012286	Betavulgarin	Gamma-aminobutyric-acid receptor subunit alpha-1	P14867	GABRA1	0.111
Radix Cyathulae（RC）	MOL012286	Betavulgarin	D-HSCDK2	O75100	CA11	0.113
Radix Cyathulae（RC）	MOL012286	Betavulgarin	DNA topoisomerase 2-alpha	P11388	TOP2A	0.12
Radix Cyathulae（RC）	MOL012286	Betavulgarin	C-jun-amino-terminal kinase-interacting protein 1	Q9UQF2	MAPK8IP1	0.129
Radix Cyathulae（RC）	MOL012286	Betavulgarin	Nuclear receptor coactivator 2	Q15596	NCOA2	0.134
Radix Cyathulae（RC）	MOL012286	Betavulgarin	MAP kinase-activated protein kinase 2	P49137	MAPKAPK2	0.141
Radix Cyathulae（RC）	MOL012286	Betavulgarin	cAMP-specific 3',5'-cyclic phosphodiesterase 4A	P27815	PDE4A	0.142
Radix Cyathulae（RC）	MOL012286	Betavulgarin	ATP-binding cassette transporter sub-family C member 8	Q09428	ABCC8	0.142
Radix Cyathulae（RC）	MOL012286	Betavulgarin	Cell division control protein 2 homolog	P06493	CDK1	0.143
Radix Cyathulae（RC）	MOL012286	Betavulgarin	ATP-sensitive inward rectifier potassium channel 1	P48048	KCNJ1	0.148
Radix Cyathulae（RC）	MOL012286	Betavulgarin	Mitogen-activated protein kinase 14	Q16539	MAPK14	0.15
Radix Cyathulae（RC）	MOL012286	Betavulgarin	Cyclin-A2	P20248	CCNA2	0.151
Radix Cyathulae（RC）	MOL012286	Betavulgarin	Nitric oxide synthase, inducible	P35228	NOS2	0.152
Radix Cyathulae（RC）	MOL012286	Betavulgarin	Nitric-oxide synthase, endothelial	P29474	NOS3	0.161
Radix Cyathulae（RC）	MOL012286	Betavulgarin	Prothrombin	P00734	F2	0.166
Radix Cyathulae（RC）	MOL012286	Betavulgarin	Beta-1 adrenergic receptor	P08588	ADRB1	0.167
Radix Cyathulae（RC）	MOL012286	Betavulgarin	Cell division protein kinase 5	Q00535	CDK5	0.173
Radix Cyathulae（RC）	MOL012286	Betavulgarin	Progesterone receptor	P06401	PGR	0.196
Radix Cyathulae（RC）	MOL012286	Betavulgarin	Trypsin-1	P07477	PRSS1	0.21

Radix Cyathulae（RC）	MOL012286	Betavulgarin	Hemoglobin subunit alpha	P69905	HBA1	0.223
Radix Cyathulae（RC）	MOL012286	Betavulgarin	Nuclear receptor coactivator 1	Q15788	NCOA1	0.229
Radix Cyathulae（RC）	MOL012286	Betavulgarin	Cell division protein kinase 2	P24941	CDK2	0.235
Radix Cyathulae（RC）	MOL012286	Betavulgarin	Prostaglandin G/H synthase 1	P23219	PTGS1	0.241
Radix Cyathulae（RC）	MOL012286	Betavulgarin	cAMP-dependent protein kinase catalytic subunit alpha	P17612	PRKACA	0.389
Radix Cyathulae（RC）	MOL012286	Betavulgarin	Estrogen receptor beta	Q92731	ESR2	0.402
Radix Cyathulae（RC）	MOL012286	Betavulgarin	Prostaglandin G/H synthase 2	P35354	PTGS2	0.778
Radix Cyathulae（RC）	MOL012286	Betavulgarin	Estrogen receptor	P03372	ESR1	1
Radix Cyathulae（RC）	MOL012298	Rubrosterone	Carbonic anhydrase 12	O43570	CA12	0.009
Radix Cyathulae（RC）	MOL012298	Rubrosterone	Carbonic anhydrase-related protein 11	O75493	CA11	0.009
Radix Cyathulae（RC）	MOL012298	Rubrosterone	Carbonic anhydrase 3	P07451	CA3	0.009
Radix Cyathulae（RC）	MOL012298	Rubrosterone	Amine oxidase [flavin-containing] A	P21397	MAOA	0.009
Radix Cyathulae（RC）	MOL012298	Rubrosterone	Carbonic anhydrase 6	P23280	CA6	0.009
Radix Cyathulae（RC）	MOL012298	Rubrosterone	Amine oxidase [flavin-containing] B	P27338	MAOB	0.009
Radix Cyathulae（RC）	MOL012298	Rubrosterone	Carbonic anhydrase 5A, mitochondrial	P35218	CA5A	0.009
Radix Cyathulae（RC）	MOL012298	Rubrosterone	Sodium channel protein type 4 subunit alpha	P35499	SCN4A	0.009
Radix Cyathulae（RC）	MOL012298	Rubrosterone	Carbonic anhydrase 7	P43166	CA7	0.009
Radix Cyathulae（RC）	MOL012298	Rubrosterone	Sodium channel protein type 9 subunit alpha	Q15858	SCN9A	0.009
Radix Cyathulae（RC）	MOL012298	Rubrosterone	Carbonic anhydrase 13	Q8N1Q1	CA13	0.009
Radix Cyathulae（RC）	MOL012298	Rubrosterone	Sodium channel subunit beta-3	Q9NY72	SCN3B	0.009
Radix Cyathulae（RC）	MOL012298	Rubrosterone	Carbonic anhydrase 5B, mitochondrial	Q9Y2D0	CA5B	0.009
Radix Cyathulae（RC）	MOL012298	Rubrosterone	Acetylcholine receptor subunit alpha	P02708	CHRNA1	0.01
Radix Cyathulae（RC）	MOL012298	Rubrosterone	Cholinesterase	P06276	BCHE	0.01
Radix Cyathulae（RC）	MOL012298	Rubrosterone	Acetylcholine receptor subunit gamma	P07510	CHRNG	0.01
Radix Cyathulae（RC）	MOL012298	Rubrosterone	Acetylcholine receptor subunit beta	P11230	CHRNB1	0.01
Radix Cyathulae（RC）	MOL012298	Rubrosterone	Neuronal acetylcholine receptor subunit beta-2	P17787	CHRNB2	0.01
Radix Cyathulae（RC）	MOL012298	Rubrosterone	Acetylcholinesterase	P22303	ACHE	0.01
Radix Cyathulae（RC）	MOL012298	Rubrosterone	Neuronal acetylcholine receptor subunit alpha-5	P30532	CHRNA5	0.01
Radix Cyathulae（RC）	MOL012298	Rubrosterone	Neuronal acetylcholine receptor subunit beta-4	P30926	CHRNB4	0.01
Radix Cyathulae（RC）	MOL012298	Rubrosterone	Neuronal acetylcholine receptor subunit alpha-4	P43681	CHRNA4	0.01
Radix Cyathulae（RC）	MOL012298	Rubrosterone	Acetylcholine receptor subunit epsilon	Q04844	CHRNE	0.01
Radix Cyathulae（RC）	MOL012298	Rubrosterone	Neuronal acetylcholine receptor subunit beta-3	Q05901	CHRNB3	0.01
Radix Cyathulae（RC）	MOL012298	Rubrosterone	Acetylcholine receptor subunit delta	Q07001	CHRND	0.01
Radix Cyathulae（RC）	MOL012298	Rubrosterone	Neuronal acetylcholine receptor subunit alpha-6	Q15825	KCNJ8	0.01
Radix Cyathulae（RC）	MOL012298	Rubrosterone	Neuronal acetylcholine receptor subunit alpha-10	Q9GZZ6	CHRNA10	0.01
Radix Cyathulae（RC）	MOL012298	Rubrosterone	Neuronal acetylcholine receptor subunit alpha-9	Q9UGM1	CHRNA9	0.01
Radix Cyathulae（RC）	MOL012298	Rubrosterone	D(2) dopamine receptor	P14416	DRD2	0.016
Radix Cyathulae（RC）	MOL012298	Rubrosterone	D(1A) dopamine receptor	P21728	DRD1	0.016
Radix Cyathulae（RC）	MOL012298	Rubrosterone	D(4) dopamine receptor	P21917	DRD4	0.016
Radix Cyathulae（RC）	MOL012298	Rubrosterone	D(1B) dopamine receptor	P21918	DRD5	0.016
Radix Cyathulae（RC）	MOL012298	Rubrosterone	D(3) dopamine receptor	P35462	DRD3	0.016
Radix Cyathulae（RC）	MOL012298	Rubrosterone	Opioid receptor, sigma 1	Q5T1J1	SIGMAR1	0.02
Radix Cyathulae（RC）	MOL012298	Rubrosterone	Sigma 1-type opioid receptor	Q99720	SIGMAR1	0.02
Radix Cyathulae（RC）	MOL012298	Rubrosterone	Translocator protein	P30536	TSPO	0.021
Radix Cyathulae（RC）	MOL012298	Rubrosterone	Phenylalanine-4-hydroxylase	P00439	PAH	0.022
Radix Cyathulae（RC）	MOL012298	Rubrosterone	Beta-2 adrenergic receptor	P07550	ADRB2	0.022
Radix Cyathulae（RC）	MOL012298	Rubrosterone	Alpha-2C adrenergic receptor	P18825	ADRA2C	0.022

Radix Cyathulae（RC）	MOL012298	Rubrosterone	Potassium voltage-gated channel subfamily KQT member 2	O43526	KCNQ2	0.024
Radix Cyathulae（RC）	MOL012298	Rubrosterone	High affinity nerve growth factor receptor	P04629	NTRK1	0.024
Radix Cyathulae（RC）	MOL012298	Rubrosterone	Muscarinic acetylcholine receptor M2	P08172	CHRM2	0.024
Radix Cyathulae（RC）	MOL012298	Rubrosterone	Muscarinic acetylcholine receptor M4	P08173	CHRM4	0.024
Radix Cyathulae（RC）	MOL012298	Rubrosterone	5-hydroxytryptamine 1A receptor	P08908	HTR1A	0.024
Radix Cyathulae（RC）	MOL012298	Rubrosterone	Muscarinic acetylcholine receptor M5	P08912	CHRM5	0.024
Radix Cyathulae（RC）	MOL012298	Rubrosterone	Muscarinic acetylcholine receptor M3	P20309	CHRM3	0.024
Radix Cyathulae（RC）	MOL012298	Rubrosterone	fMet-Leu-Phe receptor	P21462	FPR1	0.024
Radix Cyathulae（RC）	MOL012298	Rubrosterone	5-hydroxytryptamine 2A receptor	P28223	HTR2A	0.024
Radix Cyathulae（RC）	MOL012298	Rubrosterone	Histamine H1 receptor	P35367	HRH1	0.024
Radix Cyathulae（RC）	MOL012298	Rubrosterone	Solute carrier family 12 member 2	P55011	SLC12A2	0.024
Radix Cyathulae（RC）	MOL012298	Rubrosterone	Potassium voltage-gated channel subfamily A member 1	Q09470	KCNA1	0.024
Radix Cyathulae（RC）	MOL012298	Rubrosterone	Prostaglandin D2 receptor	Q13258	PTGDR	0.024
Radix Cyathulae（RC）	MOL012298	Rubrosterone	Solute carrier family 12 member 1	Q13621	SLC12A1	0.024
Radix Cyathulae（RC）	MOL012298	Rubrosterone	BDNF/NT-3 growth factors receptor	Q16620	NTRK2	0.024
Radix Cyathulae（RC）	MOL012298	Rubrosterone	Solute carrier family 12 member 5	Q9H2X9	SLC12A5	0.024
Radix Cyathulae（RC）	MOL012298	Rubrosterone	Cysteinyl leukotriene receptor 2	Q9NS75	CYSLTR2	0.024
Radix Cyathulae（RC）	MOL012298	Rubrosterone	Potassium voltage-gated channel subfamily D member 2	Q9NZV8	KCND2	0.024
Radix Cyathulae（RC）	MOL012298	Rubrosterone	Potassium voltage-gated channel subfamily D member 3	Q9UK17	KCND3	0.024
Radix Cyathulae（RC）	MOL012298	Rubrosterone	Solute carrier family 12 member 4	Q9UP95	SLC12A4	0.024
Radix Cyathulae（RC）	MOL012298	Rubrosterone	Cysteinyl leukotriene receptor 1	Q9Y271	CYSLTR1	0.024
Radix Cyathulae（RC）	MOL012298	Rubrosterone	Potassium voltage-gated channel subfamily H member 2	Q12809	KCNH2	0.026
Radix Cyathulae（RC）	MOL012298	Rubrosterone	T-cell receptor alpha chain C region	P01848	TRAC	0.027
Radix Cyathulae（RC）	MOL012298	Rubrosterone	T-cell receptor beta chain C region	P01850	TRBC1	0.027
Radix Cyathulae（RC）	MOL012298	Rubrosterone	5-hydroxytryptamine 3 receptor	P46098	HTR3A	0.027
Radix Cyathulae（RC）	MOL012298	Rubrosterone	Beta-2-microglobulin	P61769	B2M	0.027
Radix Cyathulae（RC）	MOL012298	Rubrosterone	Glutamate [NMDA] receptor subunit epsilon-1	Q12879	GRIN2A	0.027
Radix Cyathulae（RC）	MOL012298	Rubrosterone	Glutamate [NMDA] receptor subunit epsilon-2	Q13224	GRIN2B	0.027
Radix Cyathulae（RC）	MOL012298	Rubrosterone	Carbonic anhydrase 4	P22748	CA4	0.028
Radix Cyathulae（RC）	MOL012298	Rubrosterone	Elongation factor Tu GTP-binding domain-containing protein 1	Q7Z2Z2	EFL1	0.028
Radix Cyathulae（RC）	MOL012298	Rubrosterone	Neuronal acetylcholine receptor subunit alpha-3	P32297	CHRNA3	0.029
Radix Cyathulae（RC）	MOL012298	Rubrosterone	Neuronal acetylcholine receptor subunit alpha-7	P36544	CHRNA7	0.029
Radix Cyathulae（RC）	MOL012298	Rubrosterone	Gamma-aminobutyric-acid receptor subunit beta-2	P47870	GABRB2	0.029
Radix Cyathulae（RC）	MOL012298	Rubrosterone	Gamma-aminobutyric-acid receptor subunit alpha-4	P48169	GABRA4	0.029
Radix Cyathulae（RC）	MOL012298	Rubrosterone	Gamma-aminobutyric-acid receptor subunit alpha-6	Q16445	GABRA6	0.029
Radix Cyathulae（RC）	MOL012298	Rubrosterone	Gamma-aminobutyric acid receptor subunit theta	Q9UN88	GABRQ	0.029
Radix Cyathulae（RC）	MOL012298	Rubrosterone	Eukaryotic translation initiation factor 4E	P06730	EIF4E	0.035
Radix Cyathulae（RC）	MOL012298	Rubrosterone	Carcinoembryonic antigen-related cell adhesion molecule 5	P06731	CEACAM5	0.035
Radix Cyathulae（RC）	MOL012298	Rubrosterone	cAMP response element-binding protein	P16220	CREB1	0.035
Radix Cyathulae（RC）	MOL012298	Rubrosterone	Nociceptin receptor	P41146	OPRL1	0.035
Radix Cyathulae（RC）	MOL012298	Rubrosterone	Phosphatidylinositol 3-kinase regulatory subunit alpha	P27986	PIK3R1	0.037
Radix Cyathulae（RC）	MOL012298	Rubrosterone	Phosphatidylinositol-4,5-bisphosphate 3-kinase catalytic subunit gamma isoform	P48736	PIK3CG	0.037
Radix Cyathulae（RC）	MOL012298	Rubrosterone	Serine/threonine-protein kinase PLK1	P53350	PLK1	0.037
Radix Cyathulae（RC）	MOL012298	Rubrosterone	Tyrosine 3-monooxygenase	P07101	TH	0.039
Radix Cyathulae（RC）	MOL012298	Rubrosterone	Retinoic acid receptor RXR-alpha	P19793	RXRA	0.039
Radix Cyathulae（RC）	MOL012298	Rubrosterone	5-hydroxytryptamine 1D receptor	P28221	HTR1D	0.039
Radix Cyathulae（RC）	MOL012298	Rubrosterone	5-hydroxytryptamine 1B receptor	P28222	HTR1B	0.039

Radix Cyathulae（RC）	MOL012298	Rubrosterone	5-hydroxytryptamine 1F receptor	P30939	HTR1F	0.039
Radix Cyathulae（RC）	MOL012298	Rubrosterone	Tyrosyl-tRNA synthetase, cytoplasmic	P54577	YARS	0.039
Radix Cyathulae（RC）	MOL012298	Rubrosterone	Nuclear receptor subfamily 1 group I member 3	Q14994	NR1I3	0.039
Radix Cyathulae（RC）	MOL012298	Rubrosterone	Nuclear receptor coactivator 1	Q15788	NCOA1	0.039
Radix Cyathulae（RC）	MOL012298	Rubrosterone	Tyrosyl-tRNA synthetase, mitochondrial	Q9Y2Z4	YARS2	0.039
Radix Cyathulae（RC）	MOL012298	Rubrosterone	Neuronal acetylcholine receptor subunit alpha-2	Q15822	CHRNA2	0.04
Radix Cyathulae（RC）	MOL012298	Rubrosterone	Gamma-aminobutyric acid receptor subunit rho-3	A8MPY1	GABRR3	0.044
Radix Cyathulae（RC）	MOL012298	Rubrosterone	Gamma-aminobutyric acid receptor subunit pi	O00591	GABRP	0.044
Radix Cyathulae（RC）	MOL012298	Rubrosterone	Gamma-aminobutyric acid receptor subunit delta	O14764	GABRD	0.044
Radix Cyathulae（RC）	MOL012298	Rubrosterone	Gamma-aminobutyric-acid receptor subunit alpha-1	P14867	GABRA1	0.044
Radix Cyathulae（RC）	MOL012298	Rubrosterone	Gamma-aminobutyric-acid receptor subunit beta-1	P18505	GABRB1	0.044
Radix Cyathulae（RC）	MOL012298	Rubrosterone	Gamma-aminobutyric acid receptor subunit gamma-2	P18507	GABRG2	0.044
Radix Cyathulae（RC）	MOL012298	Rubrosterone	Gamma-aminobutyric-acid receptor subunit rho-1	P24046	GABRR1	0.044
Radix Cyathulae（RC）	MOL012298	Rubrosterone	Gamma-aminobutyric acid receptor subunit rho-2	P28476	GABRR2	0.044
Radix Cyathulae（RC）	MOL012298	Rubrosterone	Gamma-aminobutyric-acid receptor subunit alpha-5	P31644	GABRA5	0.044
Radix Cyathulae（RC）	MOL012298	Rubrosterone	Gamma-aminobutyric-acid receptor subunit alpha-3	P34903	GABRA3	0.044
Radix Cyathulae（RC）	MOL012298	Rubrosterone	Gamma-aminobutyric-acid receptor subunit alpha-2	P47869	GABRA2	0.044
Radix Cyathulae（RC）	MOL012298	Rubrosterone	Gamma-aminobutyric acid receptor subunit epsilon	P78334	GABRE	0.044
Radix Cyathulae（RC）	MOL012298	Rubrosterone	Gamma-aminobutyric acid receptor subunit gamma-1	Q8N1C3	GABRG1	0.044
Radix Cyathulae（RC）	MOL012298	Rubrosterone	Gamma-aminobutyric acid receptor subunit gamma-3	Q99928	GABRG3	0.044
Radix Cyathulae（RC）	MOL012298	Rubrosterone	Ig kappa chain C region	P01834	IGKC	0.045
Radix Cyathulae（RC）	MOL012298	Rubrosterone	Ig gamma-1 chain C region	P01857	IGHG1	0.045
Radix Cyathulae（RC）	MOL012298	Rubrosterone	Ig gamma-2 chain C region	P01859	IGHG2	0.045
Radix Cyathulae（RC）	MOL012298	Rubrosterone	Sodium-dependent dopamine transporter	Q01959	SLC6A3	0.045
Radix Cyathulae（RC）	MOL012298	Rubrosterone	Dehydrogenase/reductase SDR family member 8	Q8NBQ5	HSD17B11	0.045
Radix Cyathulae（RC）	MOL012298	Rubrosterone	Carbonic anhydrase 2	P00918	CA2	0.047
Radix Cyathulae（RC）	MOL012298	Rubrosterone	Prostaglandin reductase 1	Q14914	PTGR1	0.048
Radix Cyathulae（RC）	MOL012298	Rubrosterone	Alpha-2B adrenergic receptor	P18089	ADRA2B	0.052
Radix Cyathulae（RC）	MOL012298	Rubrosterone	Alpha-1B adrenergic receptor	P35368	ADRA1B	0.052
Radix Cyathulae（RC）	MOL012298	Rubrosterone	Muscarinic acetylcholine receptor M1	P11229	CHRM1	0.054
Radix Cyathulae（RC）	MOL012298	Rubrosterone	Sodium-dependent serotonin transporter	P31645	SLC6A4	0.054
Radix Cyathulae（RC）	MOL012298	Rubrosterone	cAMP-specific 3',5'-cyclic phosphodiesterase 4B	Q07343	PDE4B	0.056
Radix Cyathulae（RC）	MOL012298	Rubrosterone	cGMP-inhibited 3',5'-cyclic phosphodiesterase A	Q14432	PDE3A	0.056
Radix Cyathulae（RC）	MOL012298	Rubrosterone	Nitric-oxide synthase, endothelial	P29474	NOS3	0.057
Radix Cyathulae（RC）	MOL012298	Rubrosterone	Nitric-oxide synthase, brain	P29475	NOS1	0.057
Radix Cyathulae（RC）	MOL012298	Rubrosterone	Peptidyl-prolyl cis-trans isomerase, mitochondrial	P30405	PPIF	0.058
Radix Cyathulae（RC）	MOL012298	Rubrosterone	Annexin A1	P04083	ANXA1	0.059
Radix Cyathulae（RC）	MOL012298	Rubrosterone	Nuclear receptor 0B1	P51843	NR0B1	0.059
Radix Cyathulae（RC）	MOL012298	Rubrosterone	Carbonic anhydrase 1	P00915	CA1	0.066
Radix Cyathulae（RC）	MOL012298	Rubrosterone	3 beta-hydroxysteroid dehydrogenase/Delta 5-->4-isomerase type II	P26439	HSD3B2	0.071
Radix Cyathulae（RC）	MOL012298	Rubrosterone	Prolactin receptor	P16471	PRLR	0.075
Radix Cyathulae（RC）	MOL012298	Rubrosterone	Corticosteroid 11-beta-dehydrogenase isozyme 1	P28845	HSD11B1	0.075
Radix Cyathulae（RC）	MOL012298	Rubrosterone	Gonadotropin-releasing hormone receptor	P30968	GNRHR	0.075
Radix Cyathulae（RC）	MOL012298	Rubrosterone	Gonadotropin-releasing hormone II receptor	Q96P88	GNRHR2	0.075
Radix Cyathulae（RC）	MOL012298	Rubrosterone	Tripartite motif-containing protein 13	O60858	TRIM13	0.079
Radix Cyathulae（RC）	MOL012298	Rubrosterone	Alpha-1A adrenergic receptor	P35348	ADRA1A	0.081
Radix Cyathulae（RC）	MOL012298	Rubrosterone	Sodium-dependent noradrenaline transporter	P23975	SLC6A2	0.084

Radix Cyathulae（RC）	MOL012298	Rubrosterone	Alpha-2A adrenergic receptor	P08913	ADRA2A	0.085
Radix Cyathulae（RC）	MOL012298	Rubrosterone	Alpha-1D adrenergic receptor	P25100	ADRA1D	0.085
Radix Cyathulae（RC）	MOL012298	Rubrosterone	Beta-1 adrenergic receptor	P08588	ADRB1	0.089
Radix Cyathulae（RC）	MOL012298	Rubrosterone	Glutamate [NMDA] receptor subunit 3A	Q8TCU5	GRIN3A	0.09
Radix Cyathulae（RC）	MOL012298	Rubrosterone	Bile salt sulfotransferase	Q06520	SULT2A1	0.099
Radix Cyathulae（RC）	MOL012298	Rubrosterone	C-jun-amino-terminal kinase-interacting protein 1	Q9UQF2	MAPK8IP1	0.121
Radix Cyathulae（RC）	MOL012298	Rubrosterone	Ig kappa chain V-II region RPMI 6410	P06310	IGKV2-30	0.123
Radix Cyathulae（RC）	MOL012298	Rubrosterone	Triosephosphate isomerase	P60174	TPI1	0.124
Radix Cyathulae（RC）	MOL012298	Rubrosterone	Cytochrome c	P99999	CYCS	0.124
Radix Cyathulae（RC）	MOL012298	Rubrosterone	Hemoglobin subunit alpha	P69905	HBA1	0.125
Radix Cyathulae（RC）	MOL012298	Rubrosterone	Glucocorticoid receptor	P04150	NR3C1	0.126
Radix Cyathulae（RC）	MOL012298	Rubrosterone	MAP kinase-activated protein kinase 2	P49137	MAPKAPK2	0.127
Radix Cyathulae（RC）	MOL012298	Rubrosterone	Cytochrome P450 19A1	P11511	CYP19A1	0.128
Radix Cyathulae（RC）	MOL012298	Rubrosterone	Dual specificity protein kinase CLK1	P49759	CLK1	0.132
Radix Cyathulae（RC）	MOL012298	Rubrosterone	Nuclear receptor coactivator 5	Q9HCD5	NCOA5	0.137
Radix Cyathulae（RC）	MOL012298	Rubrosterone	3 beta-hydroxysteroid dehydrogenase/Delta 5-->4-isomerase type I	P14060	HSD3B1	0.139
Radix Cyathulae（RC）	MOL012298	Rubrosterone	Aldo-keto reductase family 1 member C1	Q04828	AKR1C1	0.143
Radix Cyathulae（RC）	MOL012298	Rubrosterone	Prothrombin	P00734	F2	0.189
Radix Cyathulae（RC）	MOL012298	Rubrosterone	Trypsin-1	P07477	PRSS1	0.189
Radix Cyathulae（RC）	MOL012298	Rubrosterone	Estradiol 17-beta-dehydrogenase 1	P14061	HSD17B1	0.236
Radix Cyathulae（RC）	MOL012298	Rubrosterone	Androgen receptor	P10275	AR	0.243
Radix Cyathulae（RC）	MOL012298	Rubrosterone	Estrogen receptor beta	Q92731	ESR2	0.26
Radix Cyathulae（RC）	MOL012298	Rubrosterone	Prostaglandin G/H synthase 2	P35354	PTGS2	0.27
Radix Cyathulae（RC）	MOL012298	Rubrosterone	Delta-type opioid receptor	P41143	OPRD1	0.288
Radix Cyathulae（RC）	MOL012298	Rubrosterone	Mineralocorticoid receptor	P08235	NR3C2	0.327
Radix Cyathulae（RC）	MOL012298	Rubrosterone	Cell division protein kinase 2	P24941	CDK2	0.387
Radix Cyathulae（RC）	MOL012298	Rubrosterone	Kappa-type opioid receptor	P41145	OPRK1	0.465
Radix Cyathulae（RC）	MOL012298	Rubrosterone	Mu-type opioid receptor	P35372	OPRM1	0.61
Radix Cyathulae（RC）	MOL012298	Rubrosterone	Progesterone receptor	P06401	PGR	0.662
Radix Cyathulae（RC）	MOL012298	Rubrosterone	Estrogen receptor	P03372	ESR1	1
Rhizoma Chuanxio(RCX)	MOL000024	alpha-humulene	Tumor necrosis factor	P01375	TNF	Validated
Rhizoma Chuanxio(RCX)	MOL000024	alpha-humulene [C0557]	Interleukin-1 beta	P01584	IL1B	Validated
Rhizoma Chuanxio(RCX)	MOL000069	palmitic acid	Solute carrier family 22 member 5	O76082	SLC22A5	Validated
Rhizoma Chuanxio(RCX)	MOL000069	palmitic acid	Tumor necrosis factor	P01375	TNF	Validated
Rhizoma Chuanxio(RCX)	MOL000069	palmitic acid	Collagen alpha-1(I) chain	P02452	COL1A1	Validated
Rhizoma Chuanxio(RCX)	MOL000069	palmitic acid	Apoptosis regulator Bcl-2	P10415	BCL2	Validated
Rhizoma Chuanxio(RCX)	MOL000069	palmitic acid	Interleukin-10	P22301	IL10	Validated
Rhizoma Chuanxio(RCX)	MOL000069	palmitic acid	Choline-phosphate cytidylyltransferase A	P49585	PCYT1A	Validated
Rhizoma Chuanxio(RCX)	MOL000069	palmitic acid	Phosphatidylinositol-3,4,5-trisphosphate 3-phosphatase and dual-specificity protein phosphatase PTEN	P60484	PTEN	Validated
Rhizoma Chuanxio(RCX)	MOL000069	palmitic acid	Putative beta-glucuronidase-like protein SMA3	Q15486	GUSBP1	Validated
Rhizoma Chuanxio(RCX)	MOL000270	3-carene	Collagen alpha-1(I) chain	P02452	COL1A1	Validated
Rhizoma Chuanxio(RCX)	MOL000270	3-carene	Osteopontin	P10451	SPP1	Validated
Rhizoma Chuanxio(RCX)	MOL000359	sitosterol	Retinoic acid receptor RXR-alpha	P19793	RXRA	0.016
Rhizoma Chuanxio(RCX)	MOL000359	sitosterol	Nuclear receptor subfamily 1 group I member 3	Q14994	NR1I3	0.016
Rhizoma Chuanxio(RCX)	MOL000359	sitosterol	Elongation factor Tu GTP-binding domain-containing protein 1	Q7Z2Z2	EFL1	0.026
Rhizoma Chuanxio(RCX)	MOL000359	sitosterol	Potassium channel subfamily K member 1	O00180	KCNK1	0.032

Rhizoma Chuanxio(RCX)	MOL000359	sitosterol	D-HSCDK2	O75100	CA11	0.032
Rhizoma Chuanxio(RCX)	MOL000359	sitosterol	Cell division control protein 2 homolog	P06493	CDK1	0.032
Rhizoma Chuanxio(RCX)	MOL000359	sitosterol	Cell division protein kinase 5	Q00535	CDK5	0.032
Rhizoma Chuanxio(RCX)	MOL000359	sitosterol	Sodium channel protein type 5 subunit alpha	Q14524	SCN5A	0.032
Rhizoma Chuanxio(RCX)	MOL000359	sitosterol	Potassium channel subfamily K member 6	Q9Y257	KCNK6	0.032
Rhizoma Chuanxio(RCX)	MOL000359	sitosterol	Neuronal acetylcholine receptor subunit alpha-3	P32297	CHRNA3	0.033
Rhizoma Chuanxio(RCX)	MOL000359	sitosterol	Neuronal acetylcholine receptor subunit alpha-7	P36544	CHRNA7	0.033
Rhizoma Chuanxio(RCX)	MOL000359	sitosterol	Neuronal acetylcholine receptor subunit alpha-4	P43681	CHRNA4	0.033
Rhizoma Chuanxio(RCX)	MOL000359	sitosterol	Phosphatidylinositol 3-kinase regulatory subunit alpha	P27986	PIK3R1	0.034
Rhizoma Chuanxio(RCX)	MOL000359	sitosterol	Phosphatidylinositol-4,5-bisphosphate 3-kinase catalytic subunit gamma isoform	P48736	PIK3CG	0.034
Rhizoma Chuanxio(RCX)	MOL000359	sitosterol	Serine/threonine-protein kinase PLK1	P53350	PLK1	0.034
Rhizoma Chuanxio(RCX)	MOL000359	sitosterol	Dehydrogenase/reductase SDR family member 8	Q8NBQ5	HSD17B11	0.041
Rhizoma Chuanxio(RCX)	MOL000359	sitosterol	Ig kappa chain C region	P01834	IGKC	0.042
Rhizoma Chuanxio(RCX)	MOL000359	sitosterol	Ig gamma-1 chain C region	P01857	IGHG1	0.042
Rhizoma Chuanxio(RCX)	MOL000359	sitosterol	Ig gamma-2 chain C region	P01859	IGHG2	0.042
Rhizoma Chuanxio(RCX)	MOL000359	sitosterol	Platelet glycoprotein IX	P14770	GP9	0.054
Rhizoma Chuanxio(RCX)	MOL000359	sitosterol	Phospholipase A2	P04054	PLA2G1B	0.057
Rhizoma Chuanxio(RCX)	MOL000359	sitosterol	Phospholipase A2, membrane associated	P14555	PLA2G2A	0.057
Rhizoma Chuanxio(RCX)	MOL000359	sitosterol	Annexin A1	P04083	ANXA1	0.062
Rhizoma Chuanxio(RCX)	MOL000359	sitosterol	Nuclear receptor 0B1	P51843	NR0B1	0.062
Rhizoma Chuanxio(RCX)	MOL000359	sitosterol	3 beta-hydroxysteroid dehydrogenase/Delta 5-->4-isomerase type II	P26439	HSD3B2	0.064
Rhizoma Chuanxio(RCX)	MOL000359	sitosterol	Cytosolic phospholipase A2	P47712	PLA2G4A	0.066
Rhizoma Chuanxio(RCX)	MOL000359	sitosterol	Corticosteroid 11-beta-dehydrogenase isozyme 1	P28845	HSD11B1	0.067
Rhizoma Chuanxio(RCX)	MOL000359	sitosterol	3-oxo-5-alpha-steroid 4-dehydrogenase 2	P31213	SRD5A2	0.067
Rhizoma Chuanxio(RCX)	MOL000359	sitosterol	Microtubule-associated protein 2	P11137	MAP2	0.068
Rhizoma Chuanxio(RCX)	MOL000359	sitosterol	Prolactin receptor	P16471	PRLR	0.068
Rhizoma Chuanxio(RCX)	MOL000359	sitosterol	Gonadotropin-releasing hormone receptor	P30968	GNRHR	0.068
Rhizoma Chuanxio(RCX)	MOL000359	sitosterol	Microtubule-associated protein 1A	P78559	MAP1A	0.068
Rhizoma Chuanxio(RCX)	MOL000359	sitosterol	Gonadotropin-releasing hormone II receptor	Q96P88	GNRHR2	0.068
Rhizoma Chuanxio(RCX)	MOL000359	sitosterol	Nuclear receptor coactivator 1	Q15788	NCOA1	0.069
Rhizoma Chuanxio(RCX)	MOL000359	sitosterol	Bile salt sulfotransferase	Q06520	SULT2A1	0.091
Rhizoma Chuanxio(RCX)	MOL000359	sitosterol	Prostaglandin G/H synthase 2	P35354	PTGS2	0.115
Rhizoma Chuanxio(RCX)	MOL000359	sitosterol	DNA polymerase kappa	Q9UBT6	POLK	0.115
Rhizoma Chuanxio(RCX)	MOL000359	sitosterol	Nuclear receptor coactivator 2	Q15596	NCOA2	0.116
Rhizoma Chuanxio(RCX)	MOL000359	sitosterol	Aldo-keto reductase family 1 member C1	Q04828	AKR1C1	0.125
Rhizoma Chuanxio(RCX)	MOL000359	sitosterol	ATP-binding cassette transporter sub-family C member 8	Q09428	ABCC8	0.127
Rhizoma Chuanxio(RCX)	MOL000359	sitosterol	3 beta-hydroxysteroid dehydrogenase/Delta 5-->4-isomerase type I	P14060	HSD3B1	0.128
Rhizoma Chuanxio(RCX)	MOL000359	sitosterol	Nitric-oxide synthase, endothelial	P29474	NOS3	0.134
Rhizoma Chuanxio(RCX)	MOL000359	sitosterol	Cannabinoid receptor 2	P34972	CNR2	0.136
Rhizoma Chuanxio(RCX)	MOL000359	sitosterol	3-oxo-5-alpha-steroid 4-dehydrogenase 1	P18405	SRD5A1	0.145
Rhizoma Chuanxio(RCX)	MOL000359	sitosterol	Nuclear receptor coactivator 5	Q9HCD5	NCOA5	0.15
Rhizoma Chuanxio(RCX)	MOL000359	sitosterol	Estradiol 17-beta-dehydrogenase 1	P14061	HSD17B1	0.221
Rhizoma Chuanxio(RCX)	MOL000359	sitosterol	Androgen receptor	P10275	AR	0.224
Rhizoma Chuanxio(RCX)	MOL000359	sitosterol	Estrogen receptor beta	Q92731	ESR2	0.246
Rhizoma Chuanxio(RCX)	MOL000359	sitosterol	Glucocorticoid receptor	P04150	NR3C1	0.288
Rhizoma Chuanxio(RCX)	MOL000359	sitosterol	Mineralocorticoid receptor	P08235	NR3C2	0.376
Rhizoma Chuanxio(RCX)	MOL000359	sitosterol	Estrogen receptor	P03372	ESR1	0.93

Rhizoma Chuanxio(RCX)	MOL000359	sitosterol	Progesterone receptor	P06401	PGR	1
Rhizoma Chuanxio(RCX)	MOL000433	FA	Prothrombin	P00734	F2	0.08
Rhizoma Chuanxio(RCX)	MOL000433	FA	Trypsin-1	P07477	PRSS1	0.08
Rhizoma Chuanxio(RCX)	MOL000433	FA	Sepiapterin reductase	P35270	SPR	0.104
Rhizoma Chuanxio(RCX)	MOL000433	FA	6-pyruvoyl tetrahydrobiopterin synthase	Q03393	PTS	0.104
Rhizoma Chuanxio(RCX)	MOL000433	FA	Folate receptor beta	P14207	FOLR2	0.47
Rhizoma Chuanxio(RCX)	MOL000433	FA	Cyclin-A2	P20248	CCNA2	1
Rhizoma Chuanxio(RCX)	MOL000669	camphor	Neuronal acetylcholine receptor subunit alpha-4	P43681	CHRNA4	Validated
Rhizoma Chuanxio(RCX)	MOL000879	methyl palmitate	Tumor necrosis factor	P01375	TNF	Validated
Rhizoma Chuanxio(RCX)	MOL000879	methyl palmitate [C0121]	Interleukin-6	P05231	IL6	Validated
Rhizoma Chuanxio(RCX)	MOL000879	methyl palmitate [C0121]	Interleukin-10	P22301	IL10	Validated
Rhizoma Chuanxio(RCX)	MOL000879	methyl palmitate [C0121]	Prostaglandin G/H synthase 2	P35354	PTGS2	Validated
Rhizoma Chuanxio(RCX)	MOL000879	methyl palmitate	Prostaglandin E2 receptor EP3 subtype	P43115	PTGER3	Validated
Rhizoma Chuanxio(RCX)	MOL000879	methyl palmitate	Transcription factor p65	Q04206	RELA	Validated
Rhizoma Chuanxio(RCX)	MOL000908	methyl palmitate	Cellular tumor antigen p53	P04637	TP53	Validated
Rhizoma Chuanxio(RCX)	MOL000908	methyl palmitate	Retinoblastoma-associated protein	P06400	RB1	Validated
Rhizoma Chuanxio(RCX)	MOL000908	methyl palmitate	Cell division control protein 2 homolog	P06493	CDC2	Validated
Rhizoma Chuanxio(RCX)	MOL000908	methyl palmitate	Apoptosis regulator Bcl-2	P10415	BCL2	Validated
Rhizoma Chuanxio(RCX)	MOL000908	methyl palmitate	G2/mitotic-specific cyclin-B1	P14635	CCNB1	Validated
Rhizoma Chuanxio(RCX)	MOL000908	methyl palmitate	Cyclin-dependent kinase inhibitor 1	P38936	CDKN1A	Validated
Rhizoma Chuanxio(RCX)	MOL000908	methyl palmitate	Eukaryotic translation initiation factor 6	P56537	EIF6	Validated
Rhizoma Chuanxio(RCX)	MOL000908	methyl palmitate	Transforming protein RhoA	P61586	RHOA	Validated
Rhizoma Chuanxio(RCX)	MOL000908	methyl palmitate	Protein CBFA2T1	Q06455	RUNX1T1	Validated
Rhizoma Chuanxio(RCX)	MOL000908	beta-elemene	Telomerase protein component 1	Q99973	TEP1	Validated
Rhizoma Chuanxio(RCX)	MOL001494	Mandenol	GTPase KRas	P01116	KRAS	0.056
Rhizoma Chuanxio(RCX)	MOL001494	Mandenol	Phosducin	P20941	PDC	0.056
Rhizoma Chuanxio(RCX)	MOL001494	Mandenol	Protein farnesyltransferase/geranylgeranyltransferase type I alpha subunit	P49354	FNTA	0.056
Rhizoma Chuanxio(RCX)	MOL001494	Mandenol	Protein farnesyltransferase subunit beta	P49356	FNTB	0.056
Rhizoma Chuanxio(RCX)	MOL001494	Mandenol	Geranylgeranyl transferase type-2 subunit beta	P53611	RABGGTB	0.056
Rhizoma Chuanxio(RCX)	MOL001494	Mandenol	Guanine nucleotide-binding protein G(I)/G(S)/G(T) subunit beta-1	P62873	GNB1	0.056
Rhizoma Chuanxio(RCX)	MOL001494	Mandenol	Guanine nucleotide-binding protein G(T) subunit gamma-T1	P63211	GNGT1	0.056
Rhizoma Chuanxio(RCX)	MOL001494	Mandenol	Geranylgeranyl transferase type-2 subunit alpha	Q92696	RABGGTA	0.056
Rhizoma Chuanxio(RCX)	MOL001494	Mandenol	Lymphocyte antigen 96	Q9Y6Y9	LY96	0.097
Rhizoma Chuanxio(RCX)	MOL001494	Mandenol	SEC14-like protein 2	O76054	SEC14L2	0.108
Rhizoma Chuanxio(RCX)	MOL001494	Mandenol	Alpha-lactalbumin	P00709	LALBA	0.108
Rhizoma Chuanxio(RCX)	MOL001494	Mandenol	Myelin P2 protein	P02689	PMP2	0.108
Rhizoma Chuanxio(RCX)	MOL001494	Mandenol	Glycodelin	P09466	PAEP	0.108
Rhizoma Chuanxio(RCX)	MOL001494	Mandenol	Cytochrome P450 2C8	P10632	CYP2C8	0.108
Rhizoma Chuanxio(RCX)	MOL001494	Mandenol	Hepatocyte nuclear factor 4-gamma	Q14541	HNF4G	0.108
Rhizoma Chuanxio(RCX)	MOL001494	Mandenol	Trafficking protein particle complex subunit 3	O43617	TRAPPC3	0.201
Rhizoma Chuanxio(RCX)	MOL001494	Mandenol	Furin	P09958	FURIN	0.283
Rhizoma Chuanxio(RCX)	MOL001494	Mandenol	Glycolipid transfer protein	Q9NZD2	GLTP	0.283
Rhizoma Chuanxio(RCX)	MOL001494	Mandenol	Rhodopsin	P08100	RHO	0.32
Rhizoma Chuanxio(RCX)	MOL001494	Mandenol	Progesterone receptor	P06401	PGR	0.447
Rhizoma Chuanxio(RCX)	MOL001494	Mandenol	Mineralocorticoid receptor	P08235	NR3C2	0.447
Rhizoma Chuanxio(RCX)	MOL001494	Mandenol	Phospholipase A2	P04054	PLA2G1B	0.563

Rhizoma Chuanxio(RCX)	MOL001494	Mandenol	Phospholipase A2, membrane associated	P14555	PLA2G2A	0.563
Rhizoma Chuanxio(RCX)	MOL001494	Mandenol	Hemoglobin subunit alpha	P69905	HBA1	1
Rhizoma Chuanxio(RCX)	MOL002042	thymol	Neutrophil elastase	P08246	ELANE	Validated
Rhizoma Chuanxio(RCX)	MOL002122	z-ligustilide	Superoxide dismutase [Cu-Zn]	P00441	SOD1	Validated
Rhizoma Chuanxio(RCX)	MOL002122	z-ligustilide	Tumor necrosis factor	P01375	TNF	Validated
Rhizoma Chuanxio(RCX)	MOL002122	z-ligustilide	Apoptosis regulator Bcl-2	P10415	BCL2	Validated
Rhizoma Chuanxio(RCX)	MOL002122	z-ligustilide	Acetylcholinesterase	P22303	ACHE	Validated
Rhizoma Chuanxio(RCX)	MOL002122	z-ligustilide	Choline O-acetyltransferase	P28329	CHAT	Validated
Rhizoma Chuanxio(RCX)	MOL002122	z-ligustilide	Caspase-3	P42574	CASP3	Validated
Rhizoma Chuanxio(RCX)	MOL002122	z-ligustilide	Transcription factor p65	Q04206	RELA	Validated
Rhizoma Chuanxio(RCX)	MOL002122	z-ligustilide	Apoptosis regulator BAX	Q07812	BAX	Validated
Rhizoma Chuanxio(RCX)	MOL002135	Myricanone	Amine oxidase [flavin-containing] A	P21397	MAOA	0.01
Rhizoma Chuanxio(RCX)	MOL002135	Myricanone	Chromaffin granule amine transporter	P54219	SLC18A1	0.01
Rhizoma Chuanxio(RCX)	MOL002135	Myricanone	Synaptic vesicular amine transporter	Q05940	SLC18A2	0.01
Rhizoma Chuanxio(RCX)	MOL002135	Myricanone	2,4-dienoyl-CoA reductase, mitochondrial	Q16698	DECR1	0.01
Rhizoma Chuanxio(RCX)	MOL002135	Myricanone	Elongation factor 2	P13639	EEF2	0.011
Rhizoma Chuanxio(RCX)	MOL002135	Myricanone	Tubulin alpha-3 chain	Q71U36	TUBA1A	0.011
Rhizoma Chuanxio(RCX)	MOL002135	Myricanone	Tubulin alpha-6 chain	Q9BQE3	TUBA1C	0.011
Rhizoma Chuanxio(RCX)	MOL002135	Myricanone	Tubulin beta-2B chain	Q9BVA1	TUBB2B	0.011
Rhizoma Chuanxio(RCX)	MOL002135	Myricanone	Stathmin-4	Q9H169	STMN4	0.011
Rhizoma Chuanxio(RCX)	MOL002135	Myricanone	Poly [ADP-ribose] polymerase 3	Q9Y6F1	PARP3	0.011
Rhizoma Chuanxio(RCX)	MOL002135	Myricanone	Serine/threonine-protein kinase 17B	O94768	STK17B	0.013
Rhizoma Chuanxio(RCX)	MOL002135	Myricanone	ATP synthase subunit gamma, mitochondrial	P36542	ATP5F1C	0.013
Rhizoma Chuanxio(RCX)	MOL002135	Myricanone	Phosphatidylinositol-4,5-bisphosphate 3-kinase catalytic subunit gamma isoform	P48736	PIK3CG	0.013
Rhizoma Chuanxio(RCX)	MOL002135	Myricanone	UDP-glucuronosyltransferase 3A1	Q6NUS8	UGT3A1	0.013
Rhizoma Chuanxio(RCX)	MOL002135	Myricanone	Tyrosyl-tRNA synthetase, cytoplasmic	P54577	YARS	0.017
Rhizoma Chuanxio(RCX)	MOL002135	Myricanone	Glutamate [NMDA] receptor subunit zeta-1	Q05586	GRIN1	0.017
Rhizoma Chuanxio(RCX)	MOL002135	Myricanone	Glutamate [NMDA] receptor subunit epsilon-1	Q12879	GRIN2A	0.017
Rhizoma Chuanxio(RCX)	MOL002135	Myricanone	Glutamate [NMDA] receptor subunit epsilon-2	Q13224	GRIN2B	0.017
Rhizoma Chuanxio(RCX)	MOL002135	Myricanone	Glutamate [NMDA] receptor subunit epsilon-3	Q14957	GRIN2C	0.017
Rhizoma Chuanxio(RCX)	MOL002135	Myricanone	Delta-type opioid receptor	P41143	OPRD1	0.019
Rhizoma Chuanxio(RCX)	MOL002135	Myricanone	Beta-2 adrenergic receptor	P07550	ADRB2	0.021
Rhizoma Chuanxio(RCX)	MOL002135	Myricanone	Beta-1 adrenergic receptor	P08588	ADRB1	0.021
Rhizoma Chuanxio(RCX)	MOL002135	Myricanone	D(4) dopamine receptor	P21917	DRD4	0.021
Rhizoma Chuanxio(RCX)	MOL002135	Myricanone	5-hydroxytryptamine 1D receptor	P28221	HTR1D	0.021
Rhizoma Chuanxio(RCX)	MOL002135	Myricanone	5-hydroxytryptamine 1B receptor	P28222	HTR1B	0.021
Rhizoma Chuanxio(RCX)	MOL002135	Myricanone	Histamine H1 receptor	P35367	HRH1	0.021
Rhizoma Chuanxio(RCX)	MOL002135	Myricanone	5-hydroxytryptamine 1A receptor	P08908	HTR1A	0.022
Rhizoma Chuanxio(RCX)	MOL002135	Myricanone	Muscarinic acetylcholine receptor M5	P08912	CHRM5	0.022
Rhizoma Chuanxio(RCX)	MOL002135	Myricanone	Muscarinic acetylcholine receptor M1	P11229	CHRM1	0.022
Rhizoma Chuanxio(RCX)	MOL002135	Myricanone	Muscarinic acetylcholine receptor M3	P20309	CHRM3	0.022
Rhizoma Chuanxio(RCX)	MOL002135	Myricanone	Alpha-1D adrenergic receptor	P25100	ADRA1D	0.022
Rhizoma Chuanxio(RCX)	MOL002135	Myricanone	5-hydroxytryptamine 2C receptor	P28335	HTR2C	0.022
Rhizoma Chuanxio(RCX)	MOL002135	Myricanone	Alpha-1B adrenergic receptor	P35368	ADRA1B	0.022
Rhizoma Chuanxio(RCX)	MOL002135	Myricanone	Sodium-dependent dopamine transporter	Q01959	SLC6A3	0.022
Rhizoma Chuanxio(RCX)	MOL002135	Myricanone	5-hydroxytryptamine 2A receptor	P28223	HTR2A	0.023
Rhizoma Chuanxio(RCX)	MOL002135	Myricanone	Macrophage migration inhibitory factor	P14174	MIF	0.024

Rhizoma Chuanxio(RCX)	MOL002135	Myricanone	Urokinase-type plasminogen activator	P00749	PLAU	0.025
Rhizoma Chuanxio(RCX)	MOL002135	Myricanone	72 kDa type IV collagenase	P08253	MMP2	0.025
Rhizoma Chuanxio(RCX)	MOL002135	Myricanone	Angiotensin-converting enzyme	P12821	ACE	0.025
Rhizoma Chuanxio(RCX)	MOL002135	Myricanone	Mitogen-activated protein kinase 8	P45983	MAPK8	0.026
Rhizoma Chuanxio(RCX)	MOL002135	Myricanone	Mitogen-activated protein kinase 10	P53779	MAPK10	0.026
Rhizoma Chuanxio(RCX)	MOL002135	Myricanone	Nitric-oxide synthase, endothelial	P29474	NOS3	0.027
Rhizoma Chuanxio(RCX)	MOL002135	Myricanone	Nitric-oxide synthase, brain	P29475	NOS1	0.027
Rhizoma Chuanxio(RCX)	MOL002135	Myricanone	Tryptophanyl-tRNA synthetase, mitochondrial	Q9UGM6	WARS2	0.027
Rhizoma Chuanxio(RCX)	MOL002135	Myricanone	cAMP and cAMP-inhibited cGMP 3',5'-cyclic phosphodiesterase 10A	Q9Y233	PDE10A	0.027
Rhizoma Chuanxio(RCX)	MOL002135	Myricanone	Proto-oncogene tyrosine-protein kinase LCK	P06239	LCK	0.028
Rhizoma Chuanxio(RCX)	MOL002135	Myricanone	Tyrosine-protein kinase Lyn	P07948	LYN	0.028
Rhizoma Chuanxio(RCX)	MOL002135	Myricanone	Peptidyl-prolyl cis-trans isomerase, mitochondrial	P30405	PPIF	0.028
Rhizoma Chuanxio(RCX)	MOL002135	Myricanone	Translocator protein	P30536	TSPO	0.029
Rhizoma Chuanxio(RCX)	MOL002135	Myricanone	Succinate dehydrogenase [ubiquinone] cytochrome b small subunit, mitochondrial	O14521	SDHD	0.031
Rhizoma Chuanxio(RCX)	MOL002135	Myricanone	ATP synthase subunit beta, mitochondrial	P06576	ATP5F1B	0.031
Rhizoma Chuanxio(RCX)	MOL002135	Myricanone	Tyrosine-protein kinase HCK	P08631	HCK	0.031
Rhizoma Chuanxio(RCX)	MOL002135	Myricanone	Proto-oncogene serine/threonine-protein kinase Pim-1	P11309	PIM1	0.031
Rhizoma Chuanxio(RCX)	MOL002135	Myricanone	ATP synthase subunit alpha, mitochondrial	P25705	ATP5F1A	0.031
Rhizoma Chuanxio(RCX)	MOL002135	Myricanone	Casein kinase I isoform gamma-2	P78368	CSNK1G2	0.031
Rhizoma Chuanxio(RCX)	MOL002135	Myricanone	Serine/threonine-protein kinase haspin	Q8TF76	HASPIN	0.031
Rhizoma Chuanxio(RCX)	MOL002135	Myricanone	Inhibitor of nuclear factor kappa-B kinase subunit alpha	O15111	CHUK	0.033
Rhizoma Chuanxio(RCX)	MOL002135	Myricanone	Arachidonate 5-lipoxygenase	P09917	ALOX5	0.033
Rhizoma Chuanxio(RCX)	MOL002135	Myricanone	Gamma-aminobutyric-acid receptor subunit beta-3	P28472	GABRB3	0.033
Rhizoma Chuanxio(RCX)	MOL002135	Myricanone	Alpha-2A adrenergic receptor	P08913	ADRA2A	0.034
Rhizoma Chuanxio(RCX)	MOL002135	Myricanone	D(3) dopamine receptor	P35462	DRD3	0.034
Rhizoma Chuanxio(RCX)	MOL002135	Myricanone	5-hydroxytryptamine 2B receptor	P41595	HTR2B	0.035
Rhizoma Chuanxio(RCX)	MOL002135	Myricanone	Cell division control protein 2 homolog	P06493	CDK1	0.042
Rhizoma Chuanxio(RCX)	MOL002135	Myricanone	Gamma-aminobutyric-acid receptor subunit alpha-6	Q16445	GABRA6	0.042
Rhizoma Chuanxio(RCX)	MOL002135	Myricanone	Gamma-aminobutyric-acid receptor subunit alpha-4	P48169	GABRA4	0.046
Rhizoma Chuanxio(RCX)	MOL002135	Myricanone	Gamma-aminobutyric acid receptor subunit theta	Q9UN88	GABRQ	0.046
Rhizoma Chuanxio(RCX)	MOL002135	Myricanone	D(2) dopamine receptor	P14416	DRD2	0.047
Rhizoma Chuanxio(RCX)	MOL002135	Myricanone	Alpha-2B adrenergic receptor	P18089	ADRA2B	0.047
Rhizoma Chuanxio(RCX)	MOL002135	Myricanone	Alpha-2C adrenergic receptor	P18825	ADRA2C	0.047
Rhizoma Chuanxio(RCX)	MOL002135	Myricanone	D(1A) dopamine receptor	P21728	DRD1	0.047
Rhizoma Chuanxio(RCX)	MOL002135	Myricanone	D(1B) dopamine receptor	P21918	DRD5	0.047
Rhizoma Chuanxio(RCX)	MOL002135	Myricanone	Alpha-1A adrenergic receptor	P35348	ADRA1A	0.047
Rhizoma Chuanxio(RCX)	MOL002135	Myricanone	Sodium-dependent noradrenaline transporter	P23975	SLC6A2	0.05
Rhizoma Chuanxio(RCX)	MOL002135	Myricanone	Gamma-aminobutyric-acid receptor subunit beta-2	P47870	GABRB2	0.05
Rhizoma Chuanxio(RCX)	MOL002135	Myricanone	Oxysterols receptor LXR-alpha	Q13133	NR1H3	0.054
Rhizoma Chuanxio(RCX)	MOL002135	Myricanone	Acetylcholinesterase	P22303	ACHE	0.055
Rhizoma Chuanxio(RCX)	MOL002135	Myricanone	Tubulin beta-1 chain	Q9H4B7	TUBB1	0.055
Rhizoma Chuanxio(RCX)	MOL002135	Myricanone	DNA polymerase kappa	Q9UBT6	POLK	0.055
Rhizoma Chuanxio(RCX)	MOL002135	Myricanone	C-jun-amino-terminal kinase-interacting protein 1	Q9UQF2	MAPK8IP1	0.055
Rhizoma Chuanxio(RCX)	MOL002135	Myricanone	Cannabinoid receptor 1	P21554	CNR1	0.056
Rhizoma Chuanxio(RCX)	MOL002135	Myricanone	Ig kappa chain V-II region RPMI 6410	P06310	IGKV2-30	0.058
Rhizoma Chuanxio(RCX)	MOL002135	Myricanone	Cytochrome P450 11B1, mitochondrial	P15538	CYP11B1	0.058
Rhizoma Chuanxio(RCX)	MOL002135	Myricanone	cAMP-specific 3',5'-cyclic phosphodiesterase 4A	P27815	PDE4A	0.058

Rhizoma Chuanxio(RCX)	MOL002135	Myricanone	cGMP-inhibited 3',5'-cyclic phosphodiesterase A	Q14432	PDE3A	0.058
Rhizoma Chuanxio(RCX)	MOL002135	Myricanone	Nuclear receptor coactivator 2	Q15596	NCOA2	0.058
Rhizoma Chuanxio(RCX)	MOL002135	Myricanone	Mitogen-activated protein kinase 14	Q16539	MAPK14	0.058
Rhizoma Chuanxio(RCX)	MOL002135	Myricanone	Cyclin-A2	P20248	CCNA2	0.059
Rhizoma Chuanxio(RCX)	MOL002135	Myricanone	Dihydrofolate reductase	P00374	DHFR	0.06
Rhizoma Chuanxio(RCX)	MOL002135	Myricanone	Neuropeptide Y	P01303	NPY	0.06
Rhizoma Chuanxio(RCX)	MOL002135	Myricanone	Triosephosphate isomerase	P60174	TPI1	0.06
Rhizoma Chuanxio(RCX)	MOL002135	Myricanone	S-methyl-5-thioadenosine phosphorylase	Q13126	MTAP	0.06
Rhizoma Chuanxio(RCX)	MOL002135	Myricanone	Endothelin-1 receptor	P25101	EDNRA	0.063
Rhizoma Chuanxio(RCX)	MOL002135	Myricanone	Kappa-type opioid receptor	P41145	OPRK1	0.063
Rhizoma Chuanxio(RCX)	MOL002135	Myricanone	Mu-type opioid receptor	P35372	OPRM1	0.065
Rhizoma Chuanxio(RCX)	MOL002135	Myricanone	Thyroid hormone receptor alpha	P10827	THRA	0.066
Rhizoma Chuanxio(RCX)	MOL002135	Myricanone	Thyroid hormone receptor beta-1	P10828	THRB	0.067
Rhizoma Chuanxio(RCX)	MOL002135	Myricanone	Group IIE secretory phospholipase A2	Q9NZK7	PLA2G2E	0.07
Rhizoma Chuanxio(RCX)	MOL002135	Myricanone	Gamma-aminobutyric acid receptor subunit gamma-2	P18507	GABRG2	0.071
Rhizoma Chuanxio(RCX)	MOL002135	Myricanone	Prostaglandin G/H synthase 1	P23219	PTGS1	0.072
Rhizoma Chuanxio(RCX)	MOL002135	Myricanone	Gamma-aminobutyric acid receptor subunit rho-3	A8MPY1	GABRR3	0.075
Rhizoma Chuanxio(RCX)	MOL002135	Myricanone	Gamma-aminobutyric acid receptor subunit pi	O00591	GABRP	0.075
Rhizoma Chuanxio(RCX)	MOL002135	Myricanone	Gamma-aminobutyric-acid receptor subunit rho-1	P24046	GABRR1	0.075
Rhizoma Chuanxio(RCX)	MOL002135	Myricanone	Gamma-aminobutyric acid receptor subunit rho-2	P28476	GABRR2	0.075
Rhizoma Chuanxio(RCX)	MOL002135	Myricanone	Gamma-aminobutyric-acid receptor subunit alpha-3	P34903	GABRA3	0.075
Rhizoma Chuanxio(RCX)	MOL002135	Myricanone	Gamma-aminobutyric acid receptor subunit epsilon	P78334	GABRE	0.075
Rhizoma Chuanxio(RCX)	MOL002135	Myricanone	Gamma-aminobutyric acid receptor subunit gamma-3	Q99928	GABRG3	0.075
Rhizoma Chuanxio(RCX)	MOL002135	Myricanone	Sodium-dependent serotonin transporter	P31645	SLC6A4	0.077
Rhizoma Chuanxio(RCX)	MOL002135	Myricanone	Gamma-aminobutyric acid receptor subunit delta	O14764	GABRD	0.079
Rhizoma Chuanxio(RCX)	MOL002135	Myricanone	Gamma-aminobutyric-acid receptor subunit alpha-1	P14867	GABRA1	0.079
Rhizoma Chuanxio(RCX)	MOL002135	Myricanone	Gamma-aminobutyric-acid receptor subunit beta-1	P18505	GABRB1	0.079
Rhizoma Chuanxio(RCX)	MOL002135	Myricanone	Gamma-aminobutyric-acid receptor subunit alpha-5	P31644	GABRA5	0.079
Rhizoma Chuanxio(RCX)	MOL002135	Myricanone	Gamma-aminobutyric-acid receptor subunit alpha-2	P47869	GABRA2	0.079
Rhizoma Chuanxio(RCX)	MOL002135	Myricanone	Gamma-aminobutyric acid receptor subunit gamma-1	Q8N1C3	GABRG1	0.079
Rhizoma Chuanxio(RCX)	MOL002135	Myricanone	Cell division protein kinase 5	Q00535	CDK5	0.089
Rhizoma Chuanxio(RCX)	MOL002135	Myricanone	cAMP-specific 3',5'-cyclic phosphodiesterase 4B	Q07343	PDE4B	0.089
Rhizoma Chuanxio(RCX)	MOL002135	Myricanone	Prothrombin	P00734	F2	0.107
Rhizoma Chuanxio(RCX)	MOL002135	Myricanone	Trypsin-1	P07477	PRSS1	0.113
Rhizoma Chuanxio(RCX)	MOL002135	Myricanone	RAC-alpha serine/threonine-protein kinase	P31749	AKT1	0.121
Rhizoma Chuanxio(RCX)	MOL002135	Myricanone	Nuclear receptor coactivator 1	Q15788	NCOA1	0.166
Rhizoma Chuanxio(RCX)	MOL002135	Myricanone	cAMP-dependent protein kinase catalytic subunit alpha	P17612	PRKACA	0.177
Rhizoma Chuanxio(RCX)	MOL002135	Myricanone	Cell division protein kinase 2	P24941	CDK2	0.182
Rhizoma Chuanxio(RCX)	MOL002135	Myricanone	Prostaglandin G/H synthase 2	P35354	PTGS2	0.22
Rhizoma Chuanxio(RCX)	MOL002135	Myricanone	Estrogen receptor beta	Q92731	ESR2	0.397
Rhizoma Chuanxio(RCX)	MOL002135	Myricanone	Estrogen receptor	P03372	ESR1	1
Rhizoma Chuanxio(RCX)	MOL002140	Perlolyrine	Sodium channel protein type 1 subunit alpha	P35498	SCN1A	0.01
Rhizoma Chuanxio(RCX)	MOL002140	Perlolyrine	2,4-dienoyl-CoA reductase, mitochondrial	Q16698	DECR1	0.01
Rhizoma Chuanxio(RCX)	MOL002140	Perlolyrine	5-hydroxytryptamine 1F receptor	P30939	HTR1F	0.011
Rhizoma Chuanxio(RCX)	MOL002140	Perlolyrine	Sodium channel protein type 4 subunit alpha	P35499	SCN4A	0.011
Rhizoma Chuanxio(RCX)	MOL002140	Perlolyrine	Glutathione S-transferase P	P09211	GSTP1	0.012
Rhizoma Chuanxio(RCX)	MOL002140	Perlolyrine	Alpha-1D adrenergic receptor	P25100	ADRA1D	0.012

Rhizoma Chuanxio(RCX)	MOL002140	Perlolyrine	5-hydroxytryptamine 1E receptor	P28566	HTR1E	0.012
Rhizoma Chuanxio(RCX)	MOL002140	Perlolyrine	5-hydroxytryptamine 7 receptor	P34969	HTR7	0.012
Rhizoma Chuanxio(RCX)	MOL002140	Perlolyrine	Kappa-type opioid receptor	P41145	OPRK1	0.012
Rhizoma Chuanxio(RCX)	MOL002140	Perlolyrine	Potassium voltage-gated channel subfamily H member 2	Q12809	KCNH2	0.012
Rhizoma Chuanxio(RCX)	MOL002140	Perlolyrine	D1 dopamine receptor-interacting protein calcyon	Q9NYX4	CALY	0.012
Rhizoma Chuanxio(RCX)	MOL002140	Perlolyrine	Sodium-dependent dopamine transporter	Q01959	SLC6A3	0.013
Rhizoma Chuanxio(RCX)	MOL002140	Perlolyrine	5-hydroxytryptamine 4 receptor	Q13639	HTR4	0.013
Rhizoma Chuanxio(RCX)	MOL002140	Perlolyrine	Neuronal acetylcholine receptor subunit alpha-2	Q15822	CHRNA2	0.013
Rhizoma Chuanxio(RCX)	MOL002140	Perlolyrine	Opioid receptor, sigma 1	Q5T1J1	SIGMAR1	0.013
Rhizoma Chuanxio(RCX)	MOL002140	Perlolyrine	Glutamate [NMDA] receptor subunit 3A	Q8TCU5	GRIN3A	0.013
Rhizoma Chuanxio(RCX)	MOL002140	Perlolyrine	Sigma 1-type opioid receptor	Q99720	SIGMAR1	0.013
Rhizoma Chuanxio(RCX)	MOL002140	Perlolyrine	Carbonic anhydrase 1	P00915	CA1	0.014
Rhizoma Chuanxio(RCX)	MOL002140	Perlolyrine	Carbonic anhydrase 2	P00918	CA2	0.014
Rhizoma Chuanxio(RCX)	MOL002140	Perlolyrine	Beta-1 adrenergic receptor	P08588	ADRB1	0.014
Rhizoma Chuanxio(RCX)	MOL002140	Perlolyrine	Delta-type opioid receptor	P41143	OPRD1	0.014
Rhizoma Chuanxio(RCX)	MOL002140	Perlolyrine	Elongation factor 2	P13639	EEF2	0.015
Rhizoma Chuanxio(RCX)	MOL002140	Perlolyrine	Poly [ADP-ribose] polymerase 3	Q9Y6F1	PARP3	0.015
Rhizoma Chuanxio(RCX)	MOL002140	Perlolyrine	Acetyl-CoA carboxylase 2	O00763	ACACB	0.017
Rhizoma Chuanxio(RCX)	MOL002140	Perlolyrine	Cytochrome b-c1 complex subunit 8	O14949	UQCRQ	0.017
Rhizoma Chuanxio(RCX)	MOL002140	Perlolyrine	Cytochrome b-c1 complex subunit 10	O14957	UQCR11	0.017
Rhizoma Chuanxio(RCX)	MOL002140	Perlolyrine	Cytochrome b	P00156	MT-CYB	0.017
Rhizoma Chuanxio(RCX)	MOL002140	Perlolyrine	Adenine phosphoribosyltransferase	P07741	APRT	0.017
Rhizoma Chuanxio(RCX)	MOL002140	Perlolyrine	Cytochrome b-c1 complex subunit 6, mitochondrial	P07919	UQCRH	0.017
Rhizoma Chuanxio(RCX)	MOL002140	Perlolyrine	Cytochrome c1, heme protein, mitochondrial	P08574	CYC1	0.017
Rhizoma Chuanxio(RCX)	MOL002140	Perlolyrine	Retinoic acid receptor alpha	P10276	RARA	0.017
Rhizoma Chuanxio(RCX)	MOL002140	Perlolyrine	Retinoic acid receptor beta	P10826	RARB	0.017
Rhizoma Chuanxio(RCX)	MOL002140	Perlolyrine	Retinoic acid receptor gamma-1	P13631	RARG	0.017
Rhizoma Chuanxio(RCX)	MOL002140	Perlolyrine	Retinoic acid receptor RXR-alpha	P19793	RXRA	0.017
Rhizoma Chuanxio(RCX)	MOL002140	Perlolyrine	Cytochrome b-c1 complex subunit 2, mitochondrial	P22695	UQCRC2	0.017
Rhizoma Chuanxio(RCX)	MOL002140	Perlolyrine	Low molecular weight phosphotyrosine protein phosphatase	P24666	ACP1	0.017
Rhizoma Chuanxio(RCX)	MOL002140	Perlolyrine	Ubiquinol-cytochrome-c reductase complex core protein 1, mitochondrial	P31930	UQCRC1	0.017
Rhizoma Chuanxio(RCX)	MOL002140	Perlolyrine	Cytochrome b-c1 complex subunit Rieske, mitochondrial	P47985	UQCRFS1	0.017
Rhizoma Chuanxio(RCX)	MOL002140	Perlolyrine	Retinoic acid receptor RXR-gamma	P48443	RXRG	0.017
Rhizoma Chuanxio(RCX)	MOL002140	Perlolyrine	Serine/threonine-protein kinase SRPK2	P78362	SRPK2	0.017
Rhizoma Chuanxio(RCX)	MOL002140	Perlolyrine	Peroxisomal trans-2-enoyl-CoA reductase	Q9BY49	PECR	0.017
Rhizoma Chuanxio(RCX)	MOL002140	Perlolyrine	Cytochrome b-c1 complex subunit 9	Q9UDW1	UQCR10	0.017
Rhizoma Chuanxio(RCX)	MOL002140	Perlolyrine	Potassium channel subfamily K member 1	O00180	KCNK1	0.018
Rhizoma Chuanxio(RCX)	MOL002140	Perlolyrine	Tyrosine 3-monooxygenase	P07101	TH	0.019
Rhizoma Chuanxio(RCX)	MOL002140	Perlolyrine	Neuronal acetylcholine receptor subunit alpha-3	P32297	CHRNA3	0.019
Rhizoma Chuanxio(RCX)	MOL002140	Perlolyrine	Neuronal acetylcholine receptor subunit alpha-7	P36544	CHRNA7	0.019
Rhizoma Chuanxio(RCX)	MOL002140	Perlolyrine	Neuronal acetylcholine receptor subunit alpha-4	P43681	CHRNA4	0.019
Rhizoma Chuanxio(RCX)	MOL002140	Perlolyrine	Tyrosyl-tRNA synthetase, cytoplasmic	P54577	YARS	0.019
Rhizoma Chuanxio(RCX)	MOL002140	Perlolyrine	Tyrosyl-tRNA synthetase, mitochondrial	Q9Y2Z4	YARS2	0.019
Rhizoma Chuanxio(RCX)	MOL002140	Perlolyrine	Vascular endothelial growth factor	A2A2V4	VEGFA	0.021
Rhizoma Chuanxio(RCX)	MOL002140	Perlolyrine	Tumor necrosis factor ligand superfamily member 11	O14788	TNFSF11	0.021
Rhizoma Chuanxio(RCX)	MOL002140	Perlolyrine	Serine/threonine-protein kinase 17B	O94768	STK17B	0.021
Rhizoma Chuanxio(RCX)	MOL002140	Perlolyrine	ATP synthase subunit beta, mitochondrial	P06576	ATP5F1B	0.021

Rhizoma Chuanxio(RCX)	MOL002140	Perlolyrine	Interleukin-3	P08700	IL3	0.021
Rhizoma Chuanxio(RCX)	MOL002140	Perlolyrine	Arachidonate 5-lipoxygenase	P09917	ALOX5	0.021
Rhizoma Chuanxio(RCX)	MOL002140	Perlolyrine	ATP synthase subunit alpha, mitochondrial	P25705	ATP5F1A	0.021
Rhizoma Chuanxio(RCX)	MOL002140	Perlolyrine	Cadherin-5	P33151	CDH5	0.021
Rhizoma Chuanxio(RCX)	MOL002140	Perlolyrine	Aryl hydrocarbon receptor	P35869	AHR	0.021
Rhizoma Chuanxio(RCX)	MOL002140	Perlolyrine	ATP synthase subunit gamma, mitochondrial	P36542	ATP5F1C	0.021
Rhizoma Chuanxio(RCX)	MOL002140	Perlolyrine	Protein S100-A12	P80511	S100A12	0.021
Rhizoma Chuanxio(RCX)	MOL002140	Perlolyrine	Dihydroorotate dehydrogenase, mitochondrial	Q02127	DHODH	0.021
Rhizoma Chuanxio(RCX)	MOL002140	Perlolyrine	UDP-glucuronosyltransferase 3A1	Q6NUS8	UGT3A1	0.021
Rhizoma Chuanxio(RCX)	MOL002140	Perlolyrine	Protein S100-A13	Q99584	S100A13	0.021
Rhizoma Chuanxio(RCX)	MOL002140	Perlolyrine	Amine oxidase [flavin-containing] A	P21397	MAOA	0.023
Rhizoma Chuanxio(RCX)	MOL002140	Perlolyrine	85 kDa calcium-independent phospholipase A2	O60733	PLA2G6	0.024
Rhizoma Chuanxio(RCX)	MOL002140	Perlolyrine	T-cell receptor alpha chain C region	P01848	TRAC	0.024
Rhizoma Chuanxio(RCX)	MOL002140	Perlolyrine	T-cell receptor beta chain C region	P01850	TRBC1	0.024
Rhizoma Chuanxio(RCX)	MOL002140	Perlolyrine	DNA topoisomerase 2-alpha	P11388	TOP2A	0.024
Rhizoma Chuanxio(RCX)	MOL002140	Perlolyrine	DNA-(apurinic or apyrimidinic site) lyase	P27695	APEX1	0.024
Rhizoma Chuanxio(RCX)	MOL002140	Perlolyrine	Cytosolic phospholipase A2	P47712	PLA2G4A	0.024
Rhizoma Chuanxio(RCX)	MOL002140	Perlolyrine	Beta-2-microglobulin	P61769	B2M	0.024
Rhizoma Chuanxio(RCX)	MOL002140	Perlolyrine	Cyclin-dependent kinase 5 activator 1	Q15078	CDK5R1	0.03
Rhizoma Chuanxio(RCX)	MOL002140	Perlolyrine	Acetylcholinesterase	P22303	ACHE	0.031
Rhizoma Chuanxio(RCX)	MOL002140	Perlolyrine	Muscarinic acetylcholine receptor M5	P08912	CHRM5	0.032
Rhizoma Chuanxio(RCX)	MOL002140	Perlolyrine	D(1B) dopamine receptor	P21918	DRD5	0.032
Rhizoma Chuanxio(RCX)	MOL002140	Perlolyrine	Myeloperoxidase	P05164	MPO	0.034
Rhizoma Chuanxio(RCX)	MOL002140	Perlolyrine	Tyrosine-protein kinase Lyn	P07948	LYN	0.034
Rhizoma Chuanxio(RCX)	MOL002140	Perlolyrine	Eosinophil peroxidase	P11678	EPX	0.034
Rhizoma Chuanxio(RCX)	MOL002140	Perlolyrine	Ribosyldihydronicotinamide dehydrogenase [quinone]	P16083	NQO2	0.034
Rhizoma Chuanxio(RCX)	MOL002140	Perlolyrine	Calreticulin	P27797	CALR	0.034
Rhizoma Chuanxio(RCX)	MOL002140	Perlolyrine	Melatonin receptor type 1A	P48039	MTNR1A	0.034
Rhizoma Chuanxio(RCX)	MOL002140	Perlolyrine	Melatonin receptor type 1B	P49286	MTNR1B	0.034
Rhizoma Chuanxio(RCX)	MOL002140	Perlolyrine	Methionine aminopeptidase 1	P53582	METAP1	0.034
Rhizoma Chuanxio(RCX)	MOL002140	Perlolyrine	Calmodulin	P62158		0.034
Rhizoma Chuanxio(RCX)	MOL002140	Perlolyrine	Nuclear receptor ROR-beta	Q92753	RORB	0.034
Rhizoma Chuanxio(RCX)	MOL002140	Perlolyrine	Succinate dehydrogenase [ubiquinone] cytochrome b small subunit, mitochondrial	O14521	SDHD	0.035
Rhizoma Chuanxio(RCX)	MOL002140	Perlolyrine	Mu-type opioid receptor	P35372	OPRM1	0.035
Rhizoma Chuanxio(RCX)	MOL002140	Perlolyrine	3-phosphoinositide-dependent protein kinase 1	O15530	PDPK1	0.036
Rhizoma Chuanxio(RCX)	MOL002140	Perlolyrine	Sterol O-acyltransferase 2	O75908	SOAT2	0.036
Rhizoma Chuanxio(RCX)	MOL002140	Perlolyrine	DNA topoisomerase 1	P11387	TOP1	0.036
Rhizoma Chuanxio(RCX)	MOL002140	Perlolyrine	Sterol O-acyltransferase 1	P35610	SOAT1	0.036
Rhizoma Chuanxio(RCX)	MOL002140	Perlolyrine	Tyrosine-protein kinase CSK	P41240	CSK	0.036
Rhizoma Chuanxio(RCX)	MOL002140	Perlolyrine	Tyrosine-protein kinase ZAP-70	P43403	ZAP70	0.036
Rhizoma Chuanxio(RCX)	MOL002140	Perlolyrine	Tyrosine-protein kinase SYK	P43405	SYK	0.036
Rhizoma Chuanxio(RCX)	MOL002140	Perlolyrine	Protein kinase C theta type	Q04759	PRKCQ	0.036
Rhizoma Chuanxio(RCX)	MOL002140	Perlolyrine	Tyrosine-protein kinase ITK/TSK	Q08881	ITK	0.036
Rhizoma Chuanxio(RCX)	MOL002140	Perlolyrine	DNA topoisomerase I, mitochondrial	Q969P6	TOP1MT	0.036
Rhizoma Chuanxio(RCX)	MOL002140	Perlolyrine	Sodium channel protein type 10 subunit alpha	Q9Y5Y9	SCN10A	0.036
Rhizoma Chuanxio(RCX)	MOL002140	Perlolyrine	Beta-2 adrenergic receptor	P07550	ADRB2	0.038
Rhizoma Chuanxio(RCX)	MOL002140	Perlolyrine	Translocator protein	P30536	TSPO	0.038

Rhizoma Chuanxio(RCX)	MOL002140	Perlolyrine	Casein kinase II subunit alpha	P68400	CSNK2A1	0.039
Rhizoma Chuanxio(RCX)	MOL002140	Perlolyrine	Nuclear receptor coactivator 2	Q15596	NCOA2	0.04
Rhizoma Chuanxio(RCX)	MOL002140	Perlolyrine	S-methyl-5-thioadenosine phosphorylase	Q13126	MTAP	0.041
Rhizoma Chuanxio(RCX)	MOL002140	Perlolyrine	Urokinase-type plasminogen activator	P00749	PLAU	0.043
Rhizoma Chuanxio(RCX)	MOL002140	Perlolyrine	Alcohol dehydrogenase [NADP+]	P14550	AKR1A1	0.043
Rhizoma Chuanxio(RCX)	MOL002140	Perlolyrine	Phospholipase A2, membrane associated	P14555	PLA2G2A	0.043
Rhizoma Chuanxio(RCX)	MOL002140	Perlolyrine	Retinoic acid receptor RXR-beta	P28702	RXRB	0.043
Rhizoma Chuanxio(RCX)	MOL002140	Perlolyrine	Casein kinase I isoform gamma-2	P78368	CSNK1G2	0.043
Rhizoma Chuanxio(RCX)	MOL002140	Perlolyrine	Lactoylglutathione lyase	Q04760	GLO1	0.043
Rhizoma Chuanxio(RCX)	MOL002140	Perlolyrine	Prostaglandin reductase 2	Q8N8N7	PTGR2	0.043
Rhizoma Chuanxio(RCX)	MOL002140	Perlolyrine	Serine/threonine-protein kinase haspin	Q8TF76	HASPIN	0.043
Rhizoma Chuanxio(RCX)	MOL002140	Perlolyrine	Aldo-keto reductase family 1 member C3	P42330	AKR1C3	0.044
Rhizoma Chuanxio(RCX)	MOL002140	Perlolyrine	S-phase kinase-associated protein 1	P63208	SKP1	0.044
Rhizoma Chuanxio(RCX)	MOL002140	Perlolyrine	cGMP-inhibited 3',5'-cyclic phosphodiesterase A	Q14432	PDE3A	0.044
Rhizoma Chuanxio(RCX)	MOL002140	Perlolyrine	cAMP and cAMP-inhibited cGMP 3',5'-cyclic phosphodiesterase 10A	Q9Y233	PDE10A	0.044
Rhizoma Chuanxio(RCX)	MOL002140	Perlolyrine	Phospholipase A2	P04054	PLA2G1B	0.045
Rhizoma Chuanxio(RCX)	MOL002140	Perlolyrine	Mitogen-activated protein kinase 3	P27361	MAPK3	0.045
Rhizoma Chuanxio(RCX)	MOL002140	Perlolyrine	Keratin, type II cytoskeletal 7	P08729	KRT7	0.047
Rhizoma Chuanxio(RCX)	MOL002140	Perlolyrine	Platelet glycoprotein IX	P14770	GP9	0.047
Rhizoma Chuanxio(RCX)	MOL002140	Perlolyrine	Tyrosine-protein kinase HCK	P08631	HCK	0.05
Rhizoma Chuanxio(RCX)	MOL002140	Perlolyrine	Mitogen-activated protein kinase 8	P45983	MAPK8	0.05
Rhizoma Chuanxio(RCX)	MOL002140	Perlolyrine	Phosphatidylinositol-4,5-bisphosphate 3-kinase catalytic subunit gamma isoform	P48736	PIK3CG	0.05
Rhizoma Chuanxio(RCX)	MOL002140	Perlolyrine	Mitogen-activated protein kinase 10	P53779	MAPK10	0.05
Rhizoma Chuanxio(RCX)	MOL002140	Perlolyrine	Muscarinic acetylcholine receptor M4	P08173	CHRM4	0.052
Rhizoma Chuanxio(RCX)	MOL002140	Perlolyrine	Alpha-2B adrenergic receptor	P18089	ADRA2B	0.052
Rhizoma Chuanxio(RCX)	MOL002140	Perlolyrine	Alpha-2C adrenergic receptor	P18825	ADRA2C	0.052
Rhizoma Chuanxio(RCX)	MOL002140	Perlolyrine	Muscarinic acetylcholine receptor M3	P20309	CHRM3	0.052
Rhizoma Chuanxio(RCX)	MOL002140	Perlolyrine	D(4) dopamine receptor	P21917	DRD4	0.052
Rhizoma Chuanxio(RCX)	MOL002140	Perlolyrine	D(3) dopamine receptor	P35462	DRD3	0.052
Rhizoma Chuanxio(RCX)	MOL002140	Perlolyrine	5-hydroxytryptamine 6 receptor	P50406	HTR6	0.052
Rhizoma Chuanxio(RCX)	MOL002140	Perlolyrine	Gamma-aminobutyric-acid receptor subunit alpha-6	Q16445	GABRA6	0.052
Rhizoma Chuanxio(RCX)	MOL002140	Perlolyrine	5-hydroxytryptamine 3 receptor	P46098	HTR3A	0.053
Rhizoma Chuanxio(RCX)	MOL002140	Perlolyrine	Tubulin beta-2C chain	P68371	TUBB4B	0.054
Rhizoma Chuanxio(RCX)	MOL002140	Perlolyrine	Nitric-oxide synthase, brain	P29475	NOS1	0.055
Rhizoma Chuanxio(RCX)	MOL002140	Perlolyrine	Tryptophanyl-tRNA synthetase, mitochondrial	Q9UGM6	WARS2	0.055
Rhizoma Chuanxio(RCX)	MOL002140	Perlolyrine	Death-associated protein kinase 3	O43293	DAPK3	0.058
Rhizoma Chuanxio(RCX)	MOL002140	Perlolyrine	Tyrosine-protein kinase JAK2	O60674	JAK2	0.058
Rhizoma Chuanxio(RCX)	MOL002140	Perlolyrine	Tyrosine-protein kinase JAK1	P23458	JAK1	0.058
Rhizoma Chuanxio(RCX)	MOL002140	Perlolyrine	Tyrosine-protein kinase JAK3	P52333	JAK3	0.058
Rhizoma Chuanxio(RCX)	MOL002140	Perlolyrine	Casein kinase I isoform gamma-3	Q9Y6M4	CSNK1G3	0.058
Rhizoma Chuanxio(RCX)	MOL002140	Perlolyrine	RAC-beta serine/threonine-protein kinase	P31751	AKT2	0.06
Rhizoma Chuanxio(RCX)	MOL002140	Perlolyrine	Rho-associated protein kinase 1	Q13464	ROCK1	0.06
Rhizoma Chuanxio(RCX)	MOL002140	Perlolyrine	Proto-oncogene serine/threonine-protein kinase Pim-1	P11309	PIM1	0.065
Rhizoma Chuanxio(RCX)	MOL002140	Perlolyrine	Gamma-aminobutyric-acid receptor subunit alpha-4	P48169	GABRA4	0.065
Rhizoma Chuanxio(RCX)	MOL002140	Perlolyrine	Gamma-aminobutyric acid receptor subunit theta	Q9UN88	GABRQ	0.065
Rhizoma Chuanxio(RCX)	MOL002140	Perlolyrine	Gamma-aminobutyric-acid receptor subunit beta-3	P28472	GABRB3	0.066
Rhizoma Chuanxio(RCX)	MOL002140	Perlolyrine	D-HSCDK2	O75100	CA11	0.069

Rhizoma Chuanxio(RCX)	MOL002140	Perlolyrine	Cell division control protein 2 homolog	P06493	CDK1	0.069
Rhizoma Chuanxio(RCX)	MOL002140	Perlolyrine	Muscarinic acetylcholine receptor M2	P08172	CHRM2	0.072
Rhizoma Chuanxio(RCX)	MOL002140	Perlolyrine	Alpha-1B adrenergic receptor	P35368	ADRA1B	0.072
Rhizoma Chuanxio(RCX)	MOL002140	Perlolyrine	Gamma-aminobutyric-acid receptor subunit beta-2	P47870	GABRB2	0.073
Rhizoma Chuanxio(RCX)	MOL002140	Perlolyrine	Leukotriene A-4 hydrolase	P09960	LTA4H	0.075
Rhizoma Chuanxio(RCX)	MOL002140	Perlolyrine	Endothelin-1 receptor	P25101	EDNRA	0.075
Rhizoma Chuanxio(RCX)	MOL002140	Perlolyrine	Dihydrofolate reductase	P00374	DHFR	0.076
Rhizoma Chuanxio(RCX)	MOL002140	Perlolyrine	Cytochrome P450 11B1, mitochondrial	P15538	CYP11B1	0.076
Rhizoma Chuanxio(RCX)	MOL002140	Perlolyrine	Dual specificity protein kinase CLK1	P49759	CLK1	0.076
Rhizoma Chuanxio(RCX)	MOL002140	Perlolyrine	Serine/threonine-protein kinase 6	O14965	AURKA	0.078
Rhizoma Chuanxio(RCX)	MOL002140	Perlolyrine	Proto-oncogene tyrosine-protein kinase LCK	P06239	LCK	0.078
Rhizoma Chuanxio(RCX)	MOL002140	Perlolyrine	Mitogen-activated protein kinase 1	P28482	MAPK1	0.078
Rhizoma Chuanxio(RCX)	MOL002140	Perlolyrine	Mitogen-activated protein kinase 14	Q16539	MAPK14	0.078
Rhizoma Chuanxio(RCX)	MOL002140	Perlolyrine	Poly [ADP-ribose] polymerase 1	P09874	PARP1	0.079
Rhizoma Chuanxio(RCX)	MOL002140	Perlolyrine	Epidermal growth factor receptor	P00533	EGFR	0.08
Rhizoma Chuanxio(RCX)	MOL002140	Perlolyrine	Protein farnesyltransferase/geranylgeranyltransferase type I alpha subunit	P49354	FNTA	0.08
Rhizoma Chuanxio(RCX)	MOL002140	Perlolyrine	cAMP-specific 3',5'-cyclic phosphodiesterase 4D	Q08499	PDE4D	0.08
Rhizoma Chuanxio(RCX)	MOL002140	Perlolyrine	Sodium channel protein type 5 subunit alpha	Q14524	SCN5A	0.081
Rhizoma Chuanxio(RCX)	MOL002140	Perlolyrine	DNA polymerase kappa	Q9UBT6	POLK	0.081
Rhizoma Chuanxio(RCX)	MOL002140	Perlolyrine	Hepatocyte growth factor receptor	P08581	MET	0.082
Rhizoma Chuanxio(RCX)	MOL002140	Perlolyrine	cGMP-specific 3',5'-cyclic phosphodiesterase	O76074	PDE5A	0.085
Rhizoma Chuanxio(RCX)	MOL002140	Perlolyrine	Triosephosphate isomerase	P60174	TPI1	0.086
Rhizoma Chuanxio(RCX)	MOL002140	Perlolyrine	Hydroxyacid oxidase 1	Q9UJM8	HAO1	0.087
Rhizoma Chuanxio(RCX)	MOL002140	Perlolyrine	Tyrosine-protein phosphatase non-receptor type 1	P18031	PTPN1	0.089
Rhizoma Chuanxio(RCX)	MOL002140	Perlolyrine	Membrane copper amine oxidase	Q16853	AOC3	0.089
Rhizoma Chuanxio(RCX)	MOL002140	Perlolyrine	Calcium/calmodulin-dependent protein kinase type II alpha chain	Q9UQM7	CAMK2A	0.089
Rhizoma Chuanxio(RCX)	MOL002140	Perlolyrine	5-hydroxytryptamine 1D receptor	P28221	HTR1D	0.091
Rhizoma Chuanxio(RCX)	MOL002140	Perlolyrine	Toll-like receptor 7	Q9NYK1	TLR7	0.091
Rhizoma Chuanxio(RCX)	MOL002140	Perlolyrine	Alpha-2A adrenergic receptor	P08913	ADRA2A	0.092
Rhizoma Chuanxio(RCX)	MOL002140	Perlolyrine	5-hydroxytryptamine 1B receptor	P28222	HTR1B	0.092
Rhizoma Chuanxio(RCX)	MOL002140	Perlolyrine	Peroxisome proliferator-activated receptor gamma	P37231	PPARG	0.092
Rhizoma Chuanxio(RCX)	MOL002140	Perlolyrine	5-hydroxytryptamine 2B receptor	P41595	HTR2B	0.093
Rhizoma Chuanxio(RCX)	MOL002140	Perlolyrine	Geranylgeranyl pyrophosphate synthetase	O95749	GGPS1	0.094
Rhizoma Chuanxio(RCX)	MOL002140	Perlolyrine	Muscarinic acetylcholine receptor M1	P11229	CHRM1	0.094
Rhizoma Chuanxio(RCX)	MOL002140	Perlolyrine	Histamine H1 receptor	P35367	HRH1	0.095
Rhizoma Chuanxio(RCX)	MOL002140	Perlolyrine	Glycogen phosphorylase, muscle form	P11217	PYGM	0.097
Rhizoma Chuanxio(RCX)	MOL002140	Perlolyrine	Aldose reductase	P15121	AKR1B1	0.097
Rhizoma Chuanxio(RCX)	MOL002140	Perlolyrine	Group IIE secretory phospholipase A2	Q9NZK7	PLA2G2E	0.098
Rhizoma Chuanxio(RCX)	MOL002140	Perlolyrine	cAMP-specific 3',5'-cyclic phosphodiesterase 4A	P27815	PDE4A	0.099
Rhizoma Chuanxio(RCX)	MOL002140	Perlolyrine	Cell division protein kinase 5	Q00535	CDK5	0.107
Rhizoma Chuanxio(RCX)	MOL002140	Perlolyrine	Peroxisome proliferator-activated receptor delta	Q03181	PPARD	0.108
Rhizoma Chuanxio(RCX)	MOL002140	Perlolyrine	Gamma-aminobutyric acid receptor subunit gamma-2	P18507	GABRG2	0.112
Rhizoma Chuanxio(RCX)	MOL002140	Perlolyrine	Gamma-aminobutyric acid receptor subunit gamma-1	Q8N1C3	GABRG1	0.112
Rhizoma Chuanxio(RCX)	MOL002140	Perlolyrine	Glycogen synthase kinase-3 beta	P49841	GSK3B	0.116
Rhizoma Chuanxio(RCX)	MOL002140	Perlolyrine	Peroxisome proliferator-activated receptor alpha	Q07869	PPARA	0.116
Rhizoma Chuanxio(RCX)	MOL002140	Perlolyrine	Tubulin alpha-3 chain	Q71U36	TUBA1A	0.116

Rhizoma Chuanxio(RCX)	MOL002140	Perlolyrine	Gamma-aminobutyric acid receptor subunit rho-3	A8MPY1	GABRR3	0.119
Rhizoma Chuanxio(RCX)	MOL002140	Perlolyrine	Gamma-aminobutyric acid receptor subunit pi	O00591	GABRP	0.119
Rhizoma Chuanxio(RCX)	MOL002140	Perlolyrine	Gamma-aminobutyric acid receptor subunit delta	O14764	GABRD	0.119
Rhizoma Chuanxio(RCX)	MOL002140	Perlolyrine	Gamma-aminobutyric-acid receptor subunit beta-1	P18505	GABRB1	0.119
Rhizoma Chuanxio(RCX)	MOL002140	Perlolyrine	Gamma-aminobutyric-acid receptor subunit rho-1	P24046	GABRR1	0.119
Rhizoma Chuanxio(RCX)	MOL002140	Perlolyrine	Gamma-aminobutyric acid receptor subunit rho-2	P28476	GABRR2	0.119
Rhizoma Chuanxio(RCX)	MOL002140	Perlolyrine	Nitric-oxide synthase, endothelial	P29474	NOS3	0.119
Rhizoma Chuanxio(RCX)	MOL002140	Perlolyrine	Gamma-aminobutyric-acid receptor subunit alpha-3	P34903	GABRA3	0.119
Rhizoma Chuanxio(RCX)	MOL002140	Perlolyrine	Gamma-aminobutyric acid receptor subunit epsilon	P78334	GABRE	0.119
Rhizoma Chuanxio(RCX)	MOL002140	Perlolyrine	Gamma-aminobutyric acid receptor subunit gamma-3	Q99928	GABRG3	0.119
Rhizoma Chuanxio(RCX)	MOL002140	Perlolyrine	Sodium-dependent noradrenaline transporter	P23975	SLC6A2	0.12
Rhizoma Chuanxio(RCX)	MOL002140	Perlolyrine	Gamma-aminobutyric-acid receptor subunit alpha-5	P31644	GABRA5	0.126
Rhizoma Chuanxio(RCX)	MOL002140	Perlolyrine	cAMP-dependent protein kinase inhibitor alpha	P61925	PKIA	0.129
Rhizoma Chuanxio(RCX)	MOL002140	Perlolyrine	Alpha-1A adrenergic receptor	P35348	ADRA1A	0.134
Rhizoma Chuanxio(RCX)	MOL002140	Perlolyrine	Gamma-aminobutyric-acid receptor subunit alpha-2	P47869	GABRA2	0.143
Rhizoma Chuanxio(RCX)	MOL002140	Perlolyrine	Gamma-aminobutyric-acid receptor subunit alpha-1	P14867	GABRA1	0.144
Rhizoma Chuanxio(RCX)	MOL002140	Perlolyrine	D(1A) dopamine receptor	P21728	DRD1	0.153
Rhizoma Chuanxio(RCX)	MOL002140	Perlolyrine	5-hydroxytryptamine 1A receptor	P08908	HTR1A	0.157
Rhizoma Chuanxio(RCX)	MOL002140	Perlolyrine	cAMP-specific 3',5'-cyclic phosphodiesterase 4B	Q07343	PDE4B	0.16
Rhizoma Chuanxio(RCX)	MOL002140	Perlolyrine	Sodium-dependent serotonin transporter	P31645	SLC6A4	0.162
Rhizoma Chuanxio(RCX)	MOL002140	Perlolyrine	RAC-alpha serine/threonine-protein kinase	P31749	AKT1	0.163
Rhizoma Chuanxio(RCX)	MOL002140	Perlolyrine	C-jun-amino-terminal kinase-interacting protein 1	Q9UQF2	MAPK8IP1	0.167
Rhizoma Chuanxio(RCX)	MOL002140	Perlolyrine	Coagulation factor VII	P08709	F7	0.171
Rhizoma Chuanxio(RCX)	MOL002140	Perlolyrine	MAP kinase-activated protein kinase 2	P49137	MAPKAPK2	0.172
Rhizoma Chuanxio(RCX)	MOL002140	Perlolyrine	5-hydroxytryptamine 2C receptor	P28335	HTR2C	0.173
Rhizoma Chuanxio(RCX)	MOL002140	Perlolyrine	Nitric oxide synthase, inducible	P35228	NOS2	0.179
Rhizoma Chuanxio(RCX)	MOL002140	Perlolyrine	Proto-oncogene tyrosine-protein kinase Src	P12931	SRC	0.192
Rhizoma Chuanxio(RCX)	MOL002140	Perlolyrine	D(2) dopamine receptor	P14416	DRD2	0.193
Rhizoma Chuanxio(RCX)	MOL002140	Perlolyrine	5-hydroxytryptamine 2A receptor	P28223	HTR2A	0.234
Rhizoma Chuanxio(RCX)	MOL002140	Perlolyrine	Hemoglobin subunit alpha	P69905	HBA1	0.27
Rhizoma Chuanxio(RCX)	MOL002140	Perlolyrine	Prothrombin	P00734	F2	0.284
Rhizoma Chuanxio(RCX)	MOL002140	Perlolyrine	Trypsin-1	P07477	PRSS1	0.301
Rhizoma Chuanxio(RCX)	MOL002140	Perlolyrine	Nuclear receptor coactivator 1	Q15788	NCOA1	0.328
Rhizoma Chuanxio(RCX)	MOL002140	Perlolyrine	Estrogen receptor beta	Q92731	ESR2	0.364
Rhizoma Chuanxio(RCX)	MOL002140	Perlolyrine	Estrogen receptor	P03372	ESR1	0.377
Rhizoma Chuanxio(RCX)	MOL002140	Perlolyrine	Cell division protein kinase 2	P24941	CDK2	0.411
Rhizoma Chuanxio(RCX)	MOL002140	Perlolyrine	Prostaglandin G/H synthase 1	P23219	PTGS1	0.415
Rhizoma Chuanxio(RCX)	MOL002140	Perlolyrine	cAMP-dependent protein kinase catalytic subunit alpha	P17612	PRKACA	0.957
Rhizoma Chuanxio(RCX)	MOL002140	Perlolyrine	Cyclin-A2	P20248	CCNA2	0.988
Rhizoma Chuanxio(RCX)	MOL002140	Perlolyrine	Prostaglandin G/H synthase 2	P35354	PTGS2	1
Rhizoma Chuanxio(RCX)	MOL002151	senkyunone	Geranylgeranyl pyrophosphate synthetase	O95749	GGPS1	0.065
Rhizoma Chuanxio(RCX)	MOL002151	senkyunone	Farnesyl pyrophosphate synthetase	P14324	FDPS	0.065
Rhizoma Chuanxio(RCX)	MOL002151	senkyunone	Phosducin	P20941	PDC	0.065
Rhizoma Chuanxio(RCX)	MOL002151	senkyunone	Guanine nucleotide-binding protein G(I)/G(S)/G(T) subunit beta-1	P62873	GNB1	0.065
Rhizoma Chuanxio(RCX)	MOL002151	senkyunone	Guanine nucleotide-binding protein G(T) subunit gamma-T1	P63211	GNGT1	0.065
Rhizoma Chuanxio(RCX)	MOL002151	senkyunone	Furin	P09958	FURIN	0.086
Rhizoma Chuanxio(RCX)	MOL002151	senkyunone	SEC14-like protein 2	O76054	SEC14L2	0.096

Rhizoma Chuanxio(RCX)	MOL002151	senkyunone	Alpha-lactalbumin	P00709	LALBA	0.096
Rhizoma Chuanxio(RCX)	MOL002151	senkyunone	Myelin P2 protein	P02689	PMP2	0.096
Rhizoma Chuanxio(RCX)	MOL002151	senkyunone	Glycodelin	P09466	PAEP	0.096
Rhizoma Chuanxio(RCX)	MOL002151	senkyunone	Cytochrome P450 2C8	P10632	CYP2C8	0.096
Rhizoma Chuanxio(RCX)	MOL002151	senkyunone	Hepatocyte nuclear factor 4-gamma	Q14541	HNF4G	0.096
Rhizoma Chuanxio(RCX)	MOL002151	senkyunone	Trafficking protein particle complex subunit 3	O43617	TRAPPC3	0.159
Rhizoma Chuanxio(RCX)	MOL002151	senkyunone	Glycolipid transfer protein	Q9NZD2	GLTP	0.17
Rhizoma Chuanxio(RCX)	MOL002151	senkyunone	GTPase KRas	P01116	KRAS	0.187
Rhizoma Chuanxio(RCX)	MOL002151	senkyunone	Protein farnesyltransferase/geranylgeranyltransferase type I alpha subunit	P49354	FNTA	0.187
Rhizoma Chuanxio(RCX)	MOL002151	senkyunone	Protein farnesyltransferase subunit beta	P49356	FNTB	0.187
Rhizoma Chuanxio(RCX)	MOL002151	senkyunone	Geranylgeranyl transferase type-2 subunit beta	P53611	RABGGTB	0.187
Rhizoma Chuanxio(RCX)	MOL002151	senkyunone	Geranylgeranyl transferase type-2 subunit alpha	Q92696	RABGGTA	0.187
Rhizoma Chuanxio(RCX)	MOL002151	senkyunone	Rhodopsin	P08100	RHO	0.257
Rhizoma Chuanxio(RCX)	MOL002151	senkyunone	Potassium channel subfamily K member 1	O00180	KCNK1	0.293
Rhizoma Chuanxio(RCX)	MOL002151	senkyunone	Sodium channel protein type 5 subunit alpha	Q14524	SCN5A	0.293
Rhizoma Chuanxio(RCX)	MOL002151	senkyunone	Potassium channel subfamily K member 6	Q9Y257	KCNK6	0.293
Rhizoma Chuanxio(RCX)	MOL002151	senkyunone	Platelet glycoprotein IX	P14770	GP9	0.468
Rhizoma Chuanxio(RCX)	MOL002151	senkyunone	Phospholipase A2	P04054	PLA2G1B	0.564
Rhizoma Chuanxio(RCX)	MOL002151	senkyunone	Phospholipase A2, membrane associated	P14555	PLA2G2A	0.564
Rhizoma Chuanxio(RCX)	MOL002151	senkyunone	Vitamin K-dependent gamma-carboxylase	P38435	GGCX	0.919
Rhizoma Chuanxio(RCX)	MOL002151	senkyunone	Cannabinoid receptor 1	P21554	CNR1	0.958
Rhizoma Chuanxio(RCX)	MOL002151	senkyunone	Proto-oncogene tyrosine-protein kinase Src	P12931	SRC	0.964
Rhizoma Chuanxio(RCX)	MOL002151	senkyunone	Cannabinoid receptor 2	P34972	CNR2	1
Rhizoma Chuanxio(RCX)	MOL002157	wallichilide	Neuronal acetylcholine receptor subunit alpha-4	P43681	CHRNA4	0.016
Rhizoma Chuanxio(RCX)	MOL002157	wallichilide	Gamma-aminobutyric-acid receptor subunit alpha-4	P48169	GABRA4	0.016
Rhizoma Chuanxio(RCX)	MOL002157	wallichilide	Glutamate receptor, ionotropic kainate 2	Q13002	GRIK2	0.016
Rhizoma Chuanxio(RCX)	MOL002157	wallichilide	Gamma-aminobutyric-acid receptor subunit alpha-6	Q16445	GABRA6	0.016
Rhizoma Chuanxio(RCX)	MOL002157	wallichilide	Opioid receptor, sigma 1	Q5T1J1	SIGMAR1	0.017
Rhizoma Chuanxio(RCX)	MOL002157	wallichilide	Glutamate [NMDA] receptor subunit 3A	Q8TCU5	GRIN3A	0.017
Rhizoma Chuanxio(RCX)	MOL002157	wallichilide	Sigma 1-type opioid receptor	Q99720	SIGMAR1	0.017
Rhizoma Chuanxio(RCX)	MOL002157	wallichilide	Elongation factor Tu GTP-binding domain-containing protein 1	Q7Z2Z2	EFL1	0.02
Rhizoma Chuanxio(RCX)	MOL002157	wallichilide	Sodium channel protein type 4 subunit alpha	P35499	SCN4A	0.023
Rhizoma Chuanxio(RCX)	MOL002157	wallichilide	Gamma-aminobutyric-acid receptor subunit beta-2	P47870	GABRB2	0.023
Rhizoma Chuanxio(RCX)	MOL002157	wallichilide	Alpha-2A adrenergic receptor	P08913	ADRA2A	0.025
Rhizoma Chuanxio(RCX)	MOL002157	wallichilide	Muscarinic acetylcholine receptor M5	P08912	CHRM5	0.026
Rhizoma Chuanxio(RCX)	MOL002157	wallichilide	D(1B) dopamine receptor	P21918	DRD5	0.026
Rhizoma Chuanxio(RCX)	MOL002157	wallichilide	Alpha-1D adrenergic receptor	P25100	ADRA1D	0.026
Rhizoma Chuanxio(RCX)	MOL002157	wallichilide	5-hydroxytryptamine 1D receptor	P28221	HTR1D	0.026
Rhizoma Chuanxio(RCX)	MOL002157	wallichilide	5-hydroxytryptamine 1B receptor	P28222	HTR1B	0.026
Rhizoma Chuanxio(RCX)	MOL002157	wallichilide	Histamine H1 receptor	P35367	HRH1	0.026
Rhizoma Chuanxio(RCX)	MOL002157	wallichilide	D(3) dopamine receptor	P35462	DRD3	0.026
Rhizoma Chuanxio(RCX)	MOL002157	wallichilide	5-hydroxytryptamine 2B receptor	P41595	HTR2B	0.026
Rhizoma Chuanxio(RCX)	MOL002157	wallichilide	Potassium channel subfamily K member 6	Q9Y257	KCNK6	0.028
Rhizoma Chuanxio(RCX)	MOL002157	wallichilide	Angiotensin-converting enzyme	P12821	ACE	0.035
Rhizoma Chuanxio(RCX)	MOL002157	wallichilide	Neuronal acetylcholine receptor subunit alpha-7	P36544	CHRNA7	0.047
Rhizoma Chuanxio(RCX)	MOL002157	wallichilide	Neuronal acetylcholine receptor subunit alpha-2	Q15822	CHRNA2	0.049

Rhizoma Chuanxio(RCX)	MOL002157	wallichilide	Voltage-dependent calcium channel subunit alpha-2/delta-1	P54289	CACNA2D1	0.056
Rhizoma Chuanxio(RCX)	MOL002157	wallichilide	Voltage-dependent L-type calcium channel subunit alpha-1D	Q01668	CACNA1D	0.056
Rhizoma Chuanxio(RCX)	MOL002157	wallichilide	Voltage-dependent L-type calcium channel subunit beta-2	Q08289	CACNB2	0.056
Rhizoma Chuanxio(RCX)	MOL002157	wallichilide	Voltage-dependent L-type calcium channel subunit alpha-1C	Q13936	CACNA1C	0.056
Rhizoma Chuanxio(RCX)	MOL002157	wallichilide	Gamma-aminobutyric acid receptor subunit rho-3	A8MPY1	GABRR3	0.057
Rhizoma Chuanxio(RCX)	MOL002157	wallichilide	Gamma-aminobutyric acid receptor subunit pi	O00591	GABRP	0.057
Rhizoma Chuanxio(RCX)	MOL002157	wallichilide	Gamma-aminobutyric acid receptor subunit delta	O14764	GABRD	0.057
Rhizoma Chuanxio(RCX)	MOL002157	wallichilide	Gamma-aminobutyric-acid receptor subunit beta-1	P18505	GABRB1	0.057
Rhizoma Chuanxio(RCX)	MOL002157	wallichilide	Gamma-aminobutyric acid receptor subunit gamma-2	P18507	GABRG2	0.057
Rhizoma Chuanxio(RCX)	MOL002157	wallichilide	Gamma-aminobutyric-acid receptor subunit rho-1	P24046	GABRR1	0.057
Rhizoma Chuanxio(RCX)	MOL002157	wallichilide	Gamma-aminobutyric acid receptor subunit rho-2	P28476	GABRR2	0.057
Rhizoma Chuanxio(RCX)	MOL002157	wallichilide	Gamma-aminobutyric acid receptor subunit epsilon	P78334	GABRE	0.057
Rhizoma Chuanxio(RCX)	MOL002157	wallichilide	Gamma-aminobutyric acid receptor subunit gamma-1	Q8N1C3	GABRG1	0.057
Rhizoma Chuanxio(RCX)	MOL002157	wallichilide	Gamma-aminobutyric acid receptor subunit gamma-3	Q99928	GABRG3	0.057
Rhizoma Chuanxio(RCX)	MOL002157	wallichilide	Sodium/potassium-transporting ATPase alpha-1 chain	P05023	ATP1A1	0.059
Rhizoma Chuanxio(RCX)	MOL002157	wallichilide	Sodium/potassium-transporting ATPase gamma chain	P54710	FXYD2	0.059
Rhizoma Chuanxio(RCX)	MOL002157	wallichilide	Solute carrier family 12 member 2	P55011	SLC12A2	0.059
Rhizoma Chuanxio(RCX)	MOL002157	wallichilide	Calcium-activated potassium channel subunit alpha 1	Q12791	KCNMA1	0.059
Rhizoma Chuanxio(RCX)	MOL002157	wallichilide	ATP-sensitive inward rectifier potassium channel 11	Q14654	KCNJ11	0.059
Rhizoma Chuanxio(RCX)	MOL002157	wallichilide	Gamma-aminobutyric-acid receptor subunit beta-3	P28472	GABRB3	0.063
Rhizoma Chuanxio(RCX)	MOL002157	wallichilide	Ig kappa chain C region	P01834	IGKC	0.072
Rhizoma Chuanxio(RCX)	MOL002157	wallichilide	Ig gamma-1 chain C region	P01857	IGHG1	0.072
Rhizoma Chuanxio(RCX)	MOL002157	wallichilide	Ig gamma-2 chain C region	P01859	IGHG2	0.072
Rhizoma Chuanxio(RCX)	MOL002157	wallichilide	Potassium channel subfamily K member 1	O00180	KCNK1	0.074
Rhizoma Chuanxio(RCX)	MOL002157	wallichilide	Alpha-2B adrenergic receptor	P18089	ADRA2B	0.074
Rhizoma Chuanxio(RCX)	MOL002157	wallichilide	Alpha-2C adrenergic receptor	P18825	ADRA2C	0.074
Rhizoma Chuanxio(RCX)	MOL002157	wallichilide	Sodium channel protein type 5 subunit alpha	Q14524	SCN5A	0.074
Rhizoma Chuanxio(RCX)	MOL002157	wallichilide	5-hydroxytryptamine 1A receptor	P08908	HTR1A	0.075
Rhizoma Chuanxio(RCX)	MOL002157	wallichilide	D(4) dopamine receptor	P21917	DRD4	0.075
Rhizoma Chuanxio(RCX)	MOL002157	wallichilide	5-hydroxytryptamine 2C receptor	P28335	HTR2C	0.075
Rhizoma Chuanxio(RCX)	MOL002157	wallichilide	Alpha-1B adrenergic receptor	P35368	ADRA1B	0.075
Rhizoma Chuanxio(RCX)	MOL002157	wallichilide	Muscarinic acetylcholine receptor M2	P08172	CHRM2	0.076
Rhizoma Chuanxio(RCX)	MOL002157	wallichilide	Muscarinic acetylcholine receptor M4	P08173	CHRM4	0.076
Rhizoma Chuanxio(RCX)	MOL002157	wallichilide	Muscarinic acetylcholine receptor M3	P20309	CHRM3	0.076
Rhizoma Chuanxio(RCX)	MOL002157	wallichilide	Phosphatidylinositol 3-kinase regulatory subunit alpha	P27986	PIK3R1	0.08
Rhizoma Chuanxio(RCX)	MOL002157	wallichilide	Phosphatidylinositol-4,5-bisphosphate 3-kinase catalytic subunit gamma isoform	P48736	PIK3CG	0.08
Rhizoma Chuanxio(RCX)	MOL002157	wallichilide	Serine/threonine-protein kinase PLK1	P53350	PLK1	0.08
Rhizoma Chuanxio(RCX)	MOL002157	wallichilide	Gamma-aminobutyric-acid receptor subunit alpha-5	P31644	GABRA5	0.096
Rhizoma Chuanxio(RCX)	MOL002157	wallichilide	Gamma-aminobutyric-acid receptor subunit alpha-3	P34903	GABRA3	0.096
Rhizoma Chuanxio(RCX)	MOL002157	wallichilide	Nociceptin receptor	P41146	OPRL1	0.096
Rhizoma Chuanxio(RCX)	MOL002157	wallichilide	Peptidyl-prolyl cis-trans isomerase, mitochondrial	P30405	PPIF	0.121
Rhizoma Chuanxio(RCX)	MOL002157	wallichilide	Phospholipase A2, membrane associated	P14555	PLA2G2A	0.122
Rhizoma Chuanxio(RCX)	MOL002157	wallichilide	Peroxisome proliferator-activated receptor gamma	P37231	PPARG	0.122
Rhizoma Chuanxio(RCX)	MOL002157	wallichilide	Lactoylglutathione lyase	Q04760	GLO1	0.122
Rhizoma Chuanxio(RCX)	MOL002157	wallichilide	Prostaglandin reductase 2	Q8N8N7	PTGR2	0.122
Rhizoma Chuanxio(RCX)	MOL002157	wallichilide	Phospholipase A2	P04054	PLA2G1B	0.123
Rhizoma Chuanxio(RCX)	MOL002157	wallichilide	ATP synthase subunit beta, mitochondrial	P06576	ATP5F1B	0.123

Rhizoma Chuanxio(RCX)	MOL002157	wallichilide	3-oxo-5-alpha-steroid 4-dehydrogenase 1	P18405	SRD5A1	0.123
Rhizoma Chuanxio(RCX)	MOL002157	wallichilide	ATP synthase subunit alpha, mitochondrial	P25705	ATP5F1A	0.123
Rhizoma Chuanxio(RCX)	MOL002157	wallichilide	3-oxo-5-alpha-steroid 4-dehydrogenase 2	P31213	SRD5A2	0.123
Rhizoma Chuanxio(RCX)	MOL002157	wallichilide	Aldo-keto reductase family 1 member C3	P42330	AKR1C3	0.123
Rhizoma Chuanxio(RCX)	MOL002157	wallichilide	5-hydroxytryptamine 2A receptor	P28223	HTR2A	0.124
Rhizoma Chuanxio(RCX)	MOL002157	wallichilide	Alpha-1A adrenergic receptor	P35348	ADRA1A	0.124
Rhizoma Chuanxio(RCX)	MOL002157	wallichilide	Muscarinic acetylcholine receptor M1	P11229	CHRM1	0.127
Rhizoma Chuanxio(RCX)	MOL002157	wallichilide	Platelet glycoprotein IX	P14770	GP9	0.134
Rhizoma Chuanxio(RCX)	MOL002157	wallichilide	Solute carrier family 12 member 1	Q13621	SLC12A1	0.142
Rhizoma Chuanxio(RCX)	MOL002157	wallichilide	Sodium-dependent serotonin transporter	P31645	SLC6A4	0.146
Rhizoma Chuanxio(RCX)	MOL002157	wallichilide	Progesterone receptor	P06401	PGR	0.148
Rhizoma Chuanxio(RCX)	MOL002157	wallichilide	Mineralocorticoid receptor	P08235	NR3C2	0.148
Rhizoma Chuanxio(RCX)	MOL002157	wallichilide	D(2) dopamine receptor	P14416	DRD2	0.173
Rhizoma Chuanxio(RCX)	MOL002157	wallichilide	D(1A) dopamine receptor	P21728	DRD1	0.173
Rhizoma Chuanxio(RCX)	MOL002157	wallichilide	Gamma-aminobutyric-acid receptor subunit alpha-1	P14867	GABRA1	0.183
Rhizoma Chuanxio(RCX)	MOL002157	wallichilide	Gamma-aminobutyric-acid receptor subunit alpha-2	P47869	GABRA2	0.183
Rhizoma Chuanxio(RCX)	MOL002157	wallichilide	Tripartite motif-containing protein 13	O60858	TRIM13	0.215
Rhizoma Chuanxio(RCX)	MOL002157	wallichilide	Endothelin-1 receptor	P25101	EDNRA	0.263
Rhizoma Chuanxio(RCX)	MOL002157	wallichilide	Oxysterols receptor LXR-alpha	Q13133	NR1H3	0.263
Rhizoma Chuanxio(RCX)	MOL002157	wallichilide	NADPH oxidase organizer 1	Q8NFA2	NOXO1	0.265
Rhizoma Chuanxio(RCX)	MOL002157	wallichilide	Cytochrome P450 11B1, mitochondrial	P15538	CYP11B1	0.267
Rhizoma Chuanxio(RCX)	MOL002157	wallichilide	Nitric oxide synthase, inducible	P35228	NOS2	0.274
Rhizoma Chuanxio(RCX)	MOL002157	wallichilide	cAMP-specific 3',5'-cyclic phosphodiesterase 4B	Q07343	PDE4B	0.274
Rhizoma Chuanxio(RCX)	MOL002157	wallichilide	Prothrombin	P00734	F2	0.277
Rhizoma Chuanxio(RCX)	MOL002157	wallichilide	Retinoic acid receptor RXR-alpha	P19793	RXRA	0.28
Rhizoma Chuanxio(RCX)	MOL002157	wallichilide	C-jun-amino-terminal kinase-interacting protein 1	Q9UQF2	MAPK8IP1	0.282
Rhizoma Chuanxio(RCX)	MOL002157	wallichilide	Cannabinoid receptor 1	P21554	CNR1	0.286
Rhizoma Chuanxio(RCX)	MOL002157	wallichilide	Thiamin pyrophosphokinase 1	Q9H3S4	TPK1	0.288
Rhizoma Chuanxio(RCX)	MOL002157	wallichilide	Liver carboxylesterase 1	P23141	CES1	0.293
Rhizoma Chuanxio(RCX)	MOL002157	wallichilide	Carbonic anhydrase 1	P00915	CA1	0.307
Rhizoma Chuanxio(RCX)	MOL002157	wallichilide	Carbonic anhydrase 2	P00918	CA2	0.307
Rhizoma Chuanxio(RCX)	MOL002157	wallichilide	ATP-binding cassette transporter sub-family C member 8	Q09428	ABCC8	0.32
Rhizoma Chuanxio(RCX)	MOL002157	wallichilide	Sodium-dependent noradrenaline transporter	P23975	SLC6A2	0.484
Rhizoma Chuanxio(RCX)	MOL002157	wallichilide	Sodium-dependent dopamine transporter	Q01959	SLC6A3	0.484
Rhizoma Chuanxio(RCX)	MOL002157	wallichilide	Nitric-oxide synthase, endothelial	P29474	NOS3	0.567
Rhizoma Chuanxio(RCX)	MOL002157	wallichilide	Delta-type opioid receptor	P41143	OPRD1	0.573
Rhizoma Chuanxio(RCX)	MOL002157	wallichilide	Kappa-type opioid receptor	P41145	OPRK1	0.812
Rhizoma Chuanxio(RCX)	MOL002157	wallichilide	Prostaglandin G/H synthase 1	P23219	PTGS1	0.853
Rhizoma Chuanxio(RCX)	MOL002157	wallichilide	Mu-type opioid receptor	P35372	OPRM1	0.931
Rhizoma Chuanxio(RCX)	MOL002157	wallichilide	Prostaglandin G/H synthase 2	P35354	PTGS2	1
Rhizoma Chuanxio(RCX)	MOL002189	butylphthalide	Beta-nerve growth factor	P01138	NGF	Validated
Rhizoma Chuanxio(RCX)	MOL002189	butylphthalide	Brain-derived neurotrophic factor	P23560	BDNF	Validated
Rhizoma Chuanxio(RCX)	MOL002202	tetramethylpyrazine	Integrin alpha-IIb	P08514	ITGA2B	Validated
Rhizoma Chuanxio(RCX)	MOL002202	tetramethylpyrazine	Vascular endothelial growth factor A	P15692	VEGFA	Validated
Rhizoma Chuanxio(RCX)	MOL002202	tetramethylpyrazine	Thromboxane-A synthase	P24557	TBXAS1	Validated
Rhizoma Chuanxio(RCX)	MOL002202	tetramethylpyrazine	Hypoxia-inducible factor 1-alpha	Q16665	HIF1A	Validated
Rhizoma Chuanxio(RCX)	MOL003493	naphthalene	Cytochrome P450 1A1	P04798	CYP1A1	Validated

Rhizoma Chuanxio(RCX)	MOL003493	naphthalene	Proto-oncogene c-Fos	P01100	FOS	Validated
Rhizoma Chuanxio(RCX)	MOL003493	naphthalene	RAF proto-oncogene serine/threonine-protein kinase	P04049	RAF1	Validated
Rhizoma Chuanxio(RCX)	MOL003493	naphthalene	Myeloperoxidase	P05164	MPO	Validated
Rhizoma Chuanxio(RCX)	MOL003493	naphthalene	Transcription factor AP-1	P05412	JUN	Validated
Rhizoma Chuanxio(RCX)	MOL003493	naphthalene	Ubiquitin carboxyl-terminal hydrolase isozyme L1	P09936	UCHL1	Validated
Rhizoma Chuanxio(RCX)	MOL003493	naphthalene	Interleukin-8	P10145	IL8	Validated
Rhizoma Chuanxio(RCX)	MOL003493	naphthalene	Apoptosis regulator Bcl-2	P10415	BCL2	Validated
Rhizoma Chuanxio(RCX)	MOL003493	naphthalene	Peroxiredoxin-5, mitochondrial	P30044	PRDX5	Validated
Rhizoma Chuanxio(RCX)	MOL003493	naphthalene	RNA-binding protein FUS	P35637	FUS	Validated
Rhizoma Chuanxio(RCX)	MOL003493	naphthalene	Eukaryotic translation initiation factor 6	P56537	EIF6	Validated
Rhizoma Chuanxio(RCX)	MOL004358	linalool	Katanin p60 ATPase-containing subunit A1	O75449	KATNA1	Validated
Rhizoma Chuanxio(RCX)	MOL004358	linalool	Adenosine receptor A2a	P29274	ADORA2A	Validated
Rhizoma Chuanxio(RCX)	MOL004358	linalool	Nitric oxide synthase, inducible	P35228	NOS2	Validated
Rhizoma Chuanxio(RCX)	MOL004358	linalool	Prostaglandin G/H synthase 2	P35354	PTGS2	Validated
Rhizoma Chuanxio(RCX)	MOL004479	o-cresol	Prostaglandin G/H synthase 1	P23219	PTGS1	Validated
Rhizoma Chuanxio(RCX)	MOL004479	o-cresol	Prostaglandin G/H synthase 2	P35354	PTGS2	Validated
Rhizoma Chuanxio(RCX)	MOL011846	menthyl acetate	Cytochrome P450 3A4	P08684	CYP3A4	Validated
Cortex Lycii(CL)	MOL000296	hederagenin	Nuclear receptor 0B1	P51843	NR0B1	0.013
Cortex Lycii(CL)	MOL000296	hederagenin	Retinoic acid-induced protein 3	Q8NFJ5	GPRC5A	0.013
Cortex Lycii(CL)	MOL000296	hederagenin	Elongation factor Tu GTP-binding domain-containing protein 1	Q7Z2Z2	EFL1	0.016
Cortex Lycii(CL)	MOL000296	hederagenin	Tripartite motif-containing protein 13	O60858	TRIM13	0.027
Cortex Lycii(CL)	MOL000296	hederagenin	Nociceptin receptor	P41146	OPRL1	0.027
Cortex Lycii(CL)	MOL000296	hederagenin	Retinal dehydrogenase 2	O94788	ALDH1A2	0.028
Cortex Lycii(CL)	MOL000296	hederagenin	Retinal dehydrogenase 1	P00352	ALDH1A1	0.028
Cortex Lycii(CL)	MOL000296	hederagenin	Neuronal acetylcholine receptor subunit alpha-3	P32297	CHRNA3	0.03
Cortex Lycii(CL)	MOL000296	hederagenin	Neuronal acetylcholine receptor subunit alpha-7	P36544	CHRNA7	0.03
Cortex Lycii(CL)	MOL000296	hederagenin	Neuronal acetylcholine receptor subunit alpha-4	P43681	CHRNA4	0.03
Cortex Lycii(CL)	MOL000296	hederagenin	Dehydrogenase/reductase SDR family member 8	Q8NBQ5	HSD17B11	0.036
Cortex Lycii(CL)	MOL000296	hederagenin	Ig kappa chain C region	P01834	IGKC	0.04
Cortex Lycii(CL)	MOL000296	hederagenin	Ig gamma-1 chain C region	P01857	IGHG1	0.04
Cortex Lycii(CL)	MOL000296	hederagenin	Ig gamma-2 chain C region	P01859	IGHG2	0.04
Cortex Lycii(CL)	MOL000296	hederagenin	Retinoic acid receptor RXR-gamma	P48443	RXRG	0.043
Cortex Lycii(CL)	MOL000296	hederagenin	Nuclear receptor subfamily 1 group I member 3	Q14994	NR1I3	0.043
Cortex Lycii(CL)	MOL000296	hederagenin	Retinoic acid receptor alpha	P10276	RARA	0.051
Cortex Lycii(CL)	MOL000296	hederagenin	Retinoic acid receptor beta	P10826	RARB	0.051
Cortex Lycii(CL)	MOL000296	hederagenin	Muscarinic acetylcholine receptor M2	P08172	CHRM2	0.056
Cortex Lycii(CL)	MOL000296	hederagenin	Neuronal acetylcholine receptor subunit alpha-2	Q15822	CHRNA2	0.056
Cortex Lycii(CL)	MOL000296	hederagenin	3 beta-hydroxysteroid dehydrogenase/Delta 5-->4-isomerase type II	P26439	HSD3B2	0.057
Cortex Lycii(CL)	MOL000296	hederagenin	Microtubule-associated protein 2	P11137	MAP2	0.061
Cortex Lycii(CL)	MOL000296	hederagenin	Microtubule-associated protein 1A	P78559	MAP1A	0.061
Cortex Lycii(CL)	MOL000296	hederagenin	Prolactin receptor	P16471	PRLR	0.062
Cortex Lycii(CL)	MOL000296	hederagenin	3-oxo-5-alpha-steroid 4-dehydrogenase 2	P31213	SRD5A2	0.062
Cortex Lycii(CL)	MOL000296	hederagenin	Estrogen receptor beta	Q92731	ESR2	0.062
Cortex Lycii(CL)	MOL000296	hederagenin	Gonadotropin-releasing hormone receptor	P30968	GNRHR	0.063
Cortex Lycii(CL)	MOL000296	hederagenin	Gonadotropin-releasing hormone II receptor	Q96P88	GNRHR2	0.063
Cortex Lycii(CL)	MOL000296	hederagenin	Corticosteroid 11-beta-dehydrogenase isozyme 1	P28845	HSD11B1	0.065
Cortex Lycii(CL)	MOL000296	hederagenin	Delta-type opioid receptor	P41143	OPRD1	0.065
Cortex Lycii(CL)	MOL000296	hederagenin	Retinoic acid receptor gamma-1	P13631	RARG	0.074
Cortex Lycii(CL)	MOL000296	hederagenin	Retinoic acid receptor RXR-beta	P28702	RXRB	0.074
Cortex Lycii(CL)	MOL000296	hederagenin	Bile salt sulfotransferase	Q06520	SULT2A1	0.081
Cortex Lycii(CL)	MOL000296	hederagenin	Mu-type opioid receptor	P35372	OPRM1	0.103

Cortex Lycii(CL)	MOL000296	hederagenin	Kappa-type opioid receptor	P41145	OPRK1	0.103
Cortex Lycii(CL)	MOL000296	hederagenin	Retinoic acid receptor RXR-alpha	P19793	RXRA	0.104
Cortex Lycii(CL)	MOL000296	hederagenin	DNA polymerase kappa	Q9UBT6	POLK	0.112
Cortex Lycii(CL)	MOL000296	hederagenin	NADPH oxidase organizer 1	Q8NFA2	NOXO1	0.114
Cortex Lycii(CL)	MOL000296	hederagenin	3 beta-hydroxysteroid dehydrogenase/Delta 5-->4-isomerase type I	P14060	HSD3B1	0.115
Cortex Lycii(CL)	MOL000296	hederagenin	ATP-binding cassette transporter sub-family C member 8	Q09428	ABCC8	0.115
Cortex Lycii(CL)	MOL000296	hederagenin	Glucocorticoid receptor	P04150	NR3C1	0.116
Cortex Lycii(CL)	MOL000296	hederagenin	Mediator of RNA polymerase II transcription subunit 1	Q15648	MED1	0.121
Cortex Lycii(CL)	MOL000296	hederagenin	Nuclear receptor coactivator 5	Q9HCD5	NCOA5	0.125
Cortex Lycii(CL)	MOL000296	hederagenin	Cannabinoid receptor 2	P34972	CNR2	0.128
Cortex Lycii(CL)	MOL000296	hederagenin	Aldo-keto reductase family 1 member C1	Q04828	AKR1C1	0.131
Cortex Lycii(CL)	MOL000296	hederagenin	Androgen receptor	P10275	AR	0.135
Cortex Lycii(CL)	MOL000296	hederagenin	3-oxo-5-alpha-steroid 4-dehydrogenase 1	P18405	SRD5A1	0.135
Cortex Lycii(CL)	MOL000296	hederagenin	Estradiol 17-beta-dehydrogenase 1	P14061	HSD17B1	0.198
Cortex Lycii(CL)	MOL000296	hederagenin	Mineralocorticoid receptor	P08235	NR3C2	0.215
Cortex Lycii(CL)	MOL000296	hederagenin	Prostaglandin G/H synthase 2	P35354	PTGS2	0.236
Cortex Lycii(CL)	MOL000296	hederagenin	Nuclear receptor coactivator 1	Q15788	NCOA1	0.307
Cortex Lycii(CL)	MOL000296	hederagenin	Progesterone receptor	P06401	PGR	0.811
Cortex Lycii(CL)	MOL000296	hederagenin	Estrogen receptor	P03372	ESR1	1
Cortex Lycii(CL)	MOL000358	beta-sitosterol	Retinoic acid receptor RXR-gamma	P48443	RXRG	0.01
Cortex Lycii(CL)	MOL000358	beta-sitosterol	Nuclear receptor 0B1	P51843	NR0B1	0.01
Cortex Lycii(CL)	MOL000358	beta-sitosterol	Retinoic acid-induced protein 3	Q8NFJ5	GPRC5A	0.01
Cortex Lycii(CL)	MOL000358	beta-sitosterol	Retinoic acid receptor alpha	P10276	RARA	0.023
Cortex Lycii(CL)	MOL000358	beta-sitosterol	Retinoic acid receptor beta	P10826	RARB	0.023
Cortex Lycii(CL)	MOL000358	beta-sitosterol	Retinal dehydrogenase 2	O94788	ALDH1A2	0.029
Cortex Lycii(CL)	MOL000358	beta-sitosterol	Retinal dehydrogenase 1	P00352	ALDH1A1	0.029
Cortex Lycii(CL)	MOL000358	beta-sitosterol	Potassium channel subfamily K member 1	O00180	KCNK1	0.036
Cortex Lycii(CL)	MOL000358	beta-sitosterol	Sodium channel protein type 5 subunit alpha	Q14524	SCN5A	0.036
Cortex Lycii(CL)	MOL000358	beta-sitosterol	Potassium channel subfamily K member 6	Q9Y257	KCNK6	0.036
Cortex Lycii(CL)	MOL000358	beta-sitosterol	Serine/threonine-protein phosphatase 2A 65 kDa regulatory subunit A alpha isoform	P30153	PPP2R1A	0.038
Cortex Lycii(CL)	MOL000358	beta-sitosterol	Serine/threonine-protein phosphatase PP1-alpha catalytic subunit	P62136	PPP1CA	0.038
Cortex Lycii(CL)	MOL000358	beta-sitosterol	Ig kappa chain C region	P01834	IGKC	0.043
Cortex Lycii(CL)	MOL000358	beta-sitosterol	Ig gamma-1 chain C region	P01857	IGHG1	0.043
Cortex Lycii(CL)	MOL000358	beta-sitosterol	Ig gamma-2 chain C region	P01859	IGHG2	0.043
Cortex Lycii(CL)	MOL000358	beta-sitosterol	Nuclear receptor subfamily 1 group I member 3	Q14994	NR1I3	0.05
Cortex Lycii(CL)	MOL000358	beta-sitosterol	Retinoic acid receptor gamma-1	P13631	RARG	0.052
Cortex Lycii(CL)	MOL000358	beta-sitosterol	Retinoic acid receptor RXR-beta	P28702	RXRB	0.052
Cortex Lycii(CL)	MOL000358	beta-sitosterol	Bile salt sulfotransferase	Q06520	SULT2A1	0.056
Cortex Lycii(CL)	MOL000358	beta-sitosterol	Dehydrogenase/reductase SDR family member 8	Q8NBQ5	HSD17B11	0.056
Cortex Lycii(CL)	MOL000358	beta-sitosterol	Platelet glycoprotein IX	P14770	GP9	0.063
Cortex Lycii(CL)	MOL000358	beta-sitosterol	Phospholipase A2	P04054	PLA2G1B	0.069
Cortex Lycii(CL)	MOL000358	beta-sitosterol	Phospholipase A2, membrane associated	P14555	PLA2G2A	0.069
Cortex Lycii(CL)	MOL000358	beta-sitosterol	3-oxo-5-alpha-steroid 4-dehydrogenase 1	P18405	SRD5A1	0.074
Cortex Lycii(CL)	MOL000358	beta-sitosterol	3-oxo-5-alpha-steroid 4-dehydrogenase 2	P31213	SRD5A2	0.074
Cortex Lycii(CL)	MOL000358	beta-sitosterol	3 beta-hydroxysteroid dehydrogenase/Delta 5-->4-isomerase type II	P26439	HSD3B2	0.079
Cortex Lycii(CL)	MOL000358	beta-sitosterol	Microtubule-associated protein 2	P11137	MAP2	0.08
Cortex Lycii(CL)	MOL000358	beta-sitosterol	Microtubule-associated protein 1A	P78559	MAP1A	0.08
Cortex Lycii(CL)	MOL000358	beta-sitosterol	Gonadotropin-releasing hormone receptor	P30968	GNRHR	0.082
Cortex Lycii(CL)	MOL000358	beta-sitosterol	Gonadotropin-releasing hormone II receptor	Q96P88	GNRHR2	0.082
Cortex Lycii(CL)	MOL000358	beta-sitosterol	Corticosteroid 11-beta-dehydrogenase isozyme 1	P28845	HSD11B1	0.084
Cortex Lycii(CL)	MOL000358	beta-sitosterol	Cytosolic phospholipase A2	P47712	PLA2G4A	0.084
Cortex Lycii(CL)	MOL000358	beta-sitosterol	Retinoic acid receptor RXR-alpha	P19793	RXRA	0.092
Cortex Lycii(CL)	MOL000358	beta-sitosterol	Serine/threonine-protein phosphatase 2A catalytic subunit alpha isoform	P67775	PPP2CA	0.095

Cortex Lycii(CL)	MOL000358	beta-sitosterol	Serine/threonine-protein phosphatase 2A 56 kDa regulatory subunit gamma isoform	Q13362	PPP2R5C	0.095
Cortex Lycii(CL)	MOL000358	beta-sitosterol	Sodium-dependent serotonin transporter	P31645	SLC6A4	0.146
Cortex Lycii(CL)	MOL000358	beta-sitosterol	Aldo-keto reductase family 1 member C1	Q04828	AKR1C1	0.147
Cortex Lycii(CL)	MOL000358	beta-sitosterol	Prostaglandin G/H synthase 2	P35354	PTGS2	0.15
Cortex Lycii(CL)	MOL000358	beta-sitosterol	Estrogen receptor beta	Q92731	ESR2	0.163
Cortex Lycii(CL)	MOL000358	beta-sitosterol	Mediator of RNA polymerase II transcription subunit 1	Q15648	MED1	0.164
Cortex Lycii(CL)	MOL000358	beta-sitosterol	Estradiol 17-beta-dehydrogenase 1	P14061	HSD17B1	0.173
Cortex Lycii(CL)	MOL000358	beta-sitosterol	3 beta-hydroxysteroid dehydrogenase/Delta 5-->4-isomerase type I	P14060	HSD3B1	0.175
Cortex Lycii(CL)	MOL000358	beta-sitosterol	Nuclear receptor coactivator 5	Q9HCD5	NCOA5	0.178
Cortex Lycii(CL)	MOL000358	beta-sitosterol	Androgen receptor	P10275	AR	0.184
Cortex Lycii(CL)	MOL000358	beta-sitosterol	Glucocorticoid receptor	P04150	NR3C1	0.187
Cortex Lycii(CL)	MOL000358	beta-sitosterol	Cannabinoid receptor 2	P34972	CNR2	0.191
Cortex Lycii(CL)	MOL000358	beta-sitosterol	Nuclear receptor coactivator 1	Q15788	NCOA1	0.296
Cortex Lycii(CL)	MOL000358	beta-sitosterol	Mineralocorticoid receptor	P08235	NR3C2	0.39
Cortex Lycii(CL)	MOL000358	beta-sitosterol	Estrogen receptor	P03372	ESR1	0.971
Cortex Lycii(CL)	MOL000358	beta-sitosterol	Progesterone receptor	P06401	PGR	1
Cortex Lycii(CL)	MOL000449	Stigmasterol	5-hydroxytryptamine 1A receptor	P08908	HTR1A	0.012
Cortex Lycii(CL)	MOL000449	Stigmasterol	Alpha-2A adrenergic receptor	P08913	ADRA2A	0.012
Cortex Lycii(CL)	MOL000449	Stigmasterol	D(2) dopamine receptor	P14416	DRD2	0.012
Cortex Lycii(CL)	MOL000449	Stigmasterol	Alpha-2B adrenergic receptor	P18089	ADRA2B	0.012
Cortex Lycii(CL)	MOL000449	Stigmasterol	Alpha-2C adrenergic receptor	P18825	ADRA2C	0.012
Cortex Lycii(CL)	MOL000449	Stigmasterol	5-hydroxytryptamine 1D receptor	P28221	HTR1D	0.012
Cortex Lycii(CL)	MOL000449	Stigmasterol	5-hydroxytryptamine 1B receptor	P28222	HTR1B	0.012
Cortex Lycii(CL)	MOL000449	Stigmasterol	5-hydroxytryptamine 2C receptor	P28335	HTR2C	0.012
Cortex Lycii(CL)	MOL000449	Stigmasterol	D(3) dopamine receptor	P35462	DRD3	0.012
Cortex Lycii(CL)	MOL000449	Stigmasterol	Elongation factor Tu GTP-binding domain-containing protein 1	Q7Z2Z2	EFL1	0.024
Cortex Lycii(CL)	MOL000449	Stigmasterol	Phosphatidylinositol 3-kinase regulatory subunit alpha	P27986	PIK3R1	0.031
Cortex Lycii(CL)	MOL000449	Stigmasterol	Neuronal acetylcholine receptor subunit alpha-3	P32297	CHRNA3	0.031
Cortex Lycii(CL)	MOL000449	Stigmasterol	Neuronal acetylcholine receptor subunit alpha-7	P36544	CHRNA7	0.031
Cortex Lycii(CL)	MOL000449	Stigmasterol	Neuronal acetylcholine receptor subunit alpha-4	P43681	CHRNA4	0.031
Cortex Lycii(CL)	MOL000449	Stigmasterol	Phosphatidylinositol-4,5-bisphosphate 3-kinase catalytic subunit gamma isoform	P48736	PIK3CG	0.031
Cortex Lycii(CL)	MOL000449	Stigmasterol	Serine/threonine-protein kinase PLK1	P53350	PLK1	0.031
Cortex Lycii(CL)	MOL000449	Stigmasterol	Dehydrogenase/reductase SDR family member 8	Q8NBQ5	HSD17B11	0.037
Cortex Lycii(CL)	MOL000449	Stigmasterol	Ig kappa chain C region	P01834	IGKC	0.04
Cortex Lycii(CL)	MOL000449	Stigmasterol	Ig gamma-1 chain C region	P01857	IGHG1	0.04
Cortex Lycii(CL)	MOL000449	Stigmasterol	Ig gamma-2 chain C region	P01859	IGHG2	0.04
Cortex Lycii(CL)	MOL000449	Stigmasterol	Retinoic acid receptor alpha	P10276	RARA	0.041
Cortex Lycii(CL)	MOL000449	Stigmasterol	Retinoic acid receptor gamma-1	P13631	RARG	0.041
Cortex Lycii(CL)	MOL000449	Stigmasterol	Nuclear receptor coactivator 2	Q15596	NCOA2	0.041
Cortex Lycii(CL)	MOL000449	Stigmasterol	3 beta-hydroxysteroid dehydrogenase/Delta 5-->4-isomerase type II	P26439	HSD3B2	0.06
Cortex Lycii(CL)	MOL000449	Stigmasterol	Annexin A1	P04083	ANXA1	0.063
Cortex Lycii(CL)	MOL000449	Stigmasterol	Nuclear receptor 0B1	P51843	NR0B1	0.063
Cortex Lycii(CL)	MOL000449	Stigmasterol	Microtubule-associated protein 2	P11137	MAP2	0.064
Cortex Lycii(CL)	MOL000449	Stigmasterol	Microtubule-associated protein 1A	P78559	MAP1A	0.064
Cortex Lycii(CL)	MOL000449	Stigmasterol	Prolactin receptor	P16471	PRLR	0.065
Cortex Lycii(CL)	MOL000449	Stigmasterol	3-oxo-5-alpha-steroid 4-dehydrogenase 2	P31213	SRD5A2	0.065
Cortex Lycii(CL)	MOL000449	Stigmasterol	Cytosolic phospholipase A2	P47712	PLA2G4A	0.065
Cortex Lycii(CL)	MOL000449	Stigmasterol	Estrogen receptor beta	Q92731	ESR2	0.065
Cortex Lycii(CL)	MOL000449	Stigmasterol	Gonadotropin-releasing hormone receptor	P30968	GNRHR	0.066
Cortex Lycii(CL)	MOL000449	Stigmasterol	Gonadotropin-releasing hormone II receptor	Q96P88	GNRHR2	0.066
Cortex Lycii(CL)	MOL000449	Stigmasterol	Corticosteroid 11-beta-dehydrogenase isozyme 1	P28845	HSD11B1	0.067
Cortex Lycii(CL)	MOL000449	Stigmasterol	Retinoic acid receptor RXR-alpha	P19793	RXRA	0.072
Cortex Lycii(CL)	MOL000449	Stigmasterol	Nuclear receptor subfamily 1 group I member 3	Q14994	NR1I3	0.072

Cortex Lycii(CL)	MOL000449	Stigmasterol	Bile salt sulfotransferase	Q06520	SULT2A1	0.087
Cortex Lycii(CL)	MOL000449	Stigmasterol	Retinoic acid receptor RXR-beta	P28702	RXRB	0.094
Cortex Lycii(CL)	MOL000449	Stigmasterol	cAMP-dependent protein kinase catalytic subunit alpha	P17612	PRKACA	0.115
Cortex Lycii(CL)	MOL000449	Stigmasterol	DNA polymerase kappa	Q9UBT6	POLK	0.118
Cortex Lycii(CL)	MOL000449	Stigmasterol	NADPH oxidase organizer 1	Q8NFA2	NOXO1	0.122
Cortex Lycii(CL)	MOL000449	Stigmasterol	3 beta-hydroxysteroid dehydrogenase/Delta 5-->4-isomerase type I	P14060	HSD3B1	0.123
Cortex Lycii(CL)	MOL000449	Stigmasterol	Aldo-keto reductase family 1 member C1	Q04828	AKR1C1	0.128
Cortex Lycii(CL)	MOL000449	Stigmasterol	Mediator of RNA polymerase II transcription subunit 1	Q15648	MED1	0.129
Cortex Lycii(CL)	MOL000449	Stigmasterol	Cannabinoid receptor 1	P21554	CNR1	0.13
Cortex Lycii(CL)	MOL000449	Stigmasterol	Cannabinoid receptor 2	P34972	CNR2	0.137
Cortex Lycii(CL)	MOL000449	Stigmasterol	Nuclear receptor coactivator 5	Q9HCD5	NCOA5	0.137
Cortex Lycii(CL)	MOL000449	Stigmasterol	3-oxo-5-alpha-steroid 4-dehydrogenase 1	P18405	SRD5A1	0.142
Cortex Lycii(CL)	MOL000449	Stigmasterol	Estradiol 17-beta-dehydrogenase 1	P14061	HSD17B1	0.214
Cortex Lycii(CL)	MOL000449	Stigmasterol	Androgen receptor	P10275	AR	0.221
Cortex Lycii(CL)	MOL000449	Stigmasterol	Nuclear receptor coactivator 1	Q15788	NCOA1	0.241
Cortex Lycii(CL)	MOL000449	Stigmasterol	Glucocorticoid receptor	P04150	NR3C1	0.292
Cortex Lycii(CL)	MOL000449	Stigmasterol	Mineralocorticoid receptor	P08235	NR3C2	0.377
Cortex Lycii(CL)	MOL000449	Stigmasterol	Estrogen receptor	P03372	ESR1	0.983
Cortex Lycii(CL)	MOL000449	Stigmasterol	Progesterone receptor	P06401	PGR	1
Cortex Lycii(CL)	MOL000953	CLR	Elongation factor Tu GTP-binding domain-containing protein 1	Q7Z2Z2	EFL1	0.013
Cortex Lycii(CL)	MOL000953	CLR	Retinoic acid-induced protein 3	Q8NFJ5	GPRC5A	0.016
Cortex Lycii(CL)	MOL000953	CLR	5-hydroxytryptamine 1A receptor	P08908	HTR1A	0.017
Cortex Lycii(CL)	MOL000953	CLR	5-hydroxytryptamine 2A receptor	P28223	HTR2A	0.018
Cortex Lycii(CL)	MOL000953	CLR	5-hydroxytryptamine 2C receptor	P28335	HTR2C	0.018
Cortex Lycii(CL)	MOL000953	CLR	D(3) dopamine receptor	P35462	DRD3	0.018
Cortex Lycii(CL)	MOL000953	CLR	Muscarinic acetylcholine receptor M4	P08173	CHRM4	0.019
Cortex Lycii(CL)	MOL000953	CLR	Muscarinic acetylcholine receptor M5	P08912	CHRM5	0.019
Cortex Lycii(CL)	MOL000953	CLR	Muscarinic acetylcholine receptor M3	P20309	CHRM3	0.019
Cortex Lycii(CL)	MOL000953	CLR	Histamine H1 receptor	P35367	HRH1	0.019
Cortex Lycii(CL)	MOL000953	CLR	Tyrosine-protein kinase JAK2	O60674	JAK2	0.023
Cortex Lycii(CL)	MOL000953	CLR	Muscarinic acetylcholine receptor M2	P08172	CHRM2	0.023
Cortex Lycii(CL)	MOL000953	CLR	Tyrosine-protein kinase JAK1	P23458	JAK1	0.023
Cortex Lycii(CL)	MOL000953	CLR	Tyrosine-protein kinase JAK3	P52333	JAK3	0.023
Cortex Lycii(CL)	MOL000953	CLR	Neuronal acetylcholine receptor subunit alpha-3	P32297	CHRNA3	0.024
Cortex Lycii(CL)	MOL000953	CLR	Neuronal acetylcholine receptor subunit alpha-7	P36544	CHRNA7	0.024
Cortex Lycii(CL)	MOL000953	CLR	Neuronal acetylcholine receptor subunit alpha-4	P43681	CHRNA4	0.024
Cortex Lycii(CL)	MOL000953	CLR	Nuclear receptor subfamily 1 group I member 3	Q14994	NR1I3	0.027
Cortex Lycii(CL)	MOL000953	CLR	Retinal dehydrogenase 2	O94788	ALDH1A2	0.03
Cortex Lycii(CL)	MOL000953	CLR	Retinal dehydrogenase 1	P00352	ALDH1A1	0.03
Cortex Lycii(CL)	MOL000953	CLR	Dehydrogenase/reductase SDR family member 8	Q8NBQ5	HSD17B11	0.03
Cortex Lycii(CL)	MOL000953	CLR	5-hydroxytryptamine 2B receptor	P41595	HTR2B	0.031
Cortex Lycii(CL)	MOL000953	CLR	Ig kappa chain C region	P01834	IGKC	0.032
Cortex Lycii(CL)	MOL000953	CLR	Ig gamma-1 chain C region	P01857	IGHG1	0.032
Cortex Lycii(CL)	MOL000953	CLR	Ig gamma-2 chain C region	P01859	IGHG2	0.032
Cortex Lycii(CL)	MOL000953	CLR	Muscarinic acetylcholine receptor M1	P11229	CHRM1	0.032
Cortex Lycii(CL)	MOL000953	CLR	Annexin A1	P04083	ANXA1	0.034
Cortex Lycii(CL)	MOL000953	CLR	Gamma-aminobutyric-acid receptor subunit alpha-1	P14867	GABRA1	0.039
Cortex Lycii(CL)	MOL000953	CLR	Gamma-aminobutyric-acid receptor subunit alpha-2	P47869	GABRA2	0.039
Cortex Lycii(CL)	MOL000953	CLR	D(2) dopamine receptor	P14416	DRD2	0.044
Cortex Lycii(CL)	MOL000953	CLR	D(1A) dopamine receptor	P21728	DRD1	0.044
Cortex Lycii(CL)	MOL000953	CLR	Cytosolic phospholipase A2	P47712	PLA2G4A	0.048
Cortex Lycii(CL)	MOL000953	CLR	3 beta-hydroxysteroid dehydrogenase/Delta 5-->4-isomerase type II	P26439	HSD3B2	0.051
Cortex Lycii(CL)	MOL000953	CLR	Corticosteroid 11-beta-dehydrogenase isozyme 1	P28845	HSD11B1	0.052

Cortex Lycii(CL)	MOL000953	CLR	Gonadotropin-releasing hormone receptor	P30968	GNRHR	0.054
Cortex Lycii(CL)	MOL000953	CLR	Gonadotropin-releasing hormone II receptor	Q96P88	GNRHR2	0.054
Cortex Lycii(CL)	MOL000953	CLR	Microtubule-associated protein 2	P11137	MAP2	0.055
Cortex Lycii(CL)	MOL000953	CLR	Prolactin receptor	P16471	PRLR	0.055
Cortex Lycii(CL)	MOL000953	CLR	Microtubule-associated protein 1A	P78559	MAP1A	0.055
Cortex Lycii(CL)	MOL000953	CLR	Nuclear receptor 0B1	P51843	NR0B1	0.058
Cortex Lycii(CL)	MOL000953	CLR	Bile salt sulfotransferase	Q06520	SULT2A1	0.066
Cortex Lycii(CL)	MOL000953	CLR	cAMP-dependent protein kinase catalytic subunit alpha	P17612	PRKACA	0.084
Cortex Lycii(CL)	MOL000953	CLR	Mu-type opioid receptor	P35372	OPRM1	0.084
Cortex Lycii(CL)	MOL000953	CLR	Retinoic acid receptor gamma-1	P13631	RARG	0.093
Cortex Lycii(CL)	MOL000953	CLR	Retinoic acid receptor RXR-gamma	P48443	RXRG	0.093
Cortex Lycii(CL)	MOL000953	CLR	Lysozyme C	P61626	LYZ	0.093
Cortex Lycii(CL)	MOL000953	CLR	ATP-binding cassette transporter sub-family C member 8	Q09428	ABCC8	0.093
Cortex Lycii(CL)	MOL000953	CLR	DNA polymerase kappa	Q9UBT6	POLK	0.093
Cortex Lycii(CL)	MOL000953	CLR	Retinoic acid receptor beta	P10826	RARB	0.095
Cortex Lycii(CL)	MOL000953	CLR	Aldo-keto reductase family 1 member C1	Q04828	AKR1C1	0.095
Cortex Lycii(CL)	MOL000953	CLR	Mediator of RNA polymerase II transcription subunit 1	Q15648	MED1	0.096
Cortex Lycii(CL)	MOL000953	CLR	3 beta-hydroxysteroid dehydrogenase/Delta 5-->4-isomerase type I	P14060	HSD3B1	0.1
Cortex Lycii(CL)	MOL000953	CLR	Nuclear receptor coactivator 5	Q9HCD5	NCOA5	0.102
Cortex Lycii(CL)	MOL000953	CLR	Retinoic acid receptor RXR-alpha	P19793	RXRA	0.104
Cortex Lycii(CL)	MOL000953	CLR	Cannabinoid receptor 2	P34972	CNR2	0.105
Cortex Lycii(CL)	MOL000953	CLR	Nuclear receptor coactivator 1	Q15788	NCOA1	0.108
Cortex Lycii(CL)	MOL000953	CLR	3-oxo-5-alpha-steroid 4-dehydrogenase 1	P18405	SRD5A1	0.118
Cortex Lycii(CL)	MOL000953	CLR	Retinoic acid receptor RXR-beta	P28702	RXRB	0.119
Cortex Lycii(CL)	MOL000953	CLR	3-oxo-5-alpha-steroid 4-dehydrogenase 2	P31213	SRD5A2	0.119
Cortex Lycii(CL)	MOL000953	CLR	Retinoic acid receptor alpha	P10276	RARA	0.12
Cortex Lycii(CL)	MOL000953	CLR	Estradiol 17-beta-dehydrogenase 1	P14061	HSD17B1	0.165
Cortex Lycii(CL)	MOL000953	CLR	Androgen receptor	P10275	AR	0.179
Cortex Lycii(CL)	MOL000953	CLR	Prostaglandin G/H synthase 2	P35354	PTGS2	0.189
Cortex Lycii(CL)	MOL000953	CLR	Glucocorticoid receptor	P04150	NR3C1	0.196
Cortex Lycii(CL)	MOL000953	CLR	Mineralocorticoid receptor	P08235	NR3C2	0.288
Cortex Lycii(CL)	MOL000953	CLR	Estrogen receptor beta	Q92731	ESR2	0.444
Cortex Lycii(CL)	MOL000953	CLR	Progesterone receptor	P06401	PGR	0.844
Cortex Lycii(CL)	MOL000953	CLR	Estrogen receptor	P03372	ESR1	1
Cortex Lycii(CL)	MOL001552	OIN	Gamma-aminobutyric acid receptor subunit rho-3	A8MPY1	GABRR3	0.014
Cortex Lycii(CL)	MOL001552	OIN	Gamma-aminobutyric acid receptor subunit pi	O00591	GABRP	0.014
Cortex Lycii(CL)	MOL001552	OIN	Gamma-aminobutyric acid receptor subunit delta	O14764	GABRD	0.014
Cortex Lycii(CL)	MOL001552	OIN	Gamma-aminobutyric-acid receptor subunit beta-1	P18505	GABRB1	0.014
Cortex Lycii(CL)	MOL001552	OIN	Gamma-aminobutyric acid receptor subunit gamma-2	P18507	GABRG2	0.014
Cortex Lycii(CL)	MOL001552	OIN	Gamma-aminobutyric-acid receptor subunit rho-1	P24046	GABRR1	0.014
Cortex Lycii(CL)	MOL001552	OIN	Gamma-aminobutyric acid receptor subunit rho-2	P28476	GABRR2	0.014
Cortex Lycii(CL)	MOL001552	OIN	Glutamate receptor 1	P42261	GRIA1	0.014
Cortex Lycii(CL)	MOL001552	OIN	Gamma-aminobutyric-acid receptor subunit beta-2	P47870	GABRB2	0.014
Cortex Lycii(CL)	MOL001552	OIN	Gamma-aminobutyric acid receptor subunit epsilon	P78334	GABRE	0.014
Cortex Lycii(CL)	MOL001552	OIN	Gamma-aminobutyric acid receptor subunit gamma-1	Q8N1C3	GABRG1	0.014
Cortex Lycii(CL)	MOL001552	OIN	Gamma-aminobutyric acid receptor subunit gamma-3	Q99928	GABRG3	0.014
Cortex Lycii(CL)	MOL001552	OIN	Gamma-aminobutyric acid receptor subunit theta	Q9UN88	GABRQ	0.014
Cortex Lycii(CL)	MOL001552	OIN	Serine/threonine-protein kinase 17B	O94768	STK17B	0.059
Cortex Lycii(CL)	MOL001552	OIN	ATP synthase subunit beta, mitochondrial	P06576	ATP5F1B	0.059
Cortex Lycii(CL)	MOL001552	OIN	Proto-oncogene serine/threonine-protein kinase Pim-1	P11309	PIM1	0.059
Cortex Lycii(CL)	MOL001552	OIN	ATP synthase subunit alpha, mitochondrial	P25705	ATP5F1A	0.059
Cortex Lycii(CL)	MOL001552	OIN	ATP synthase subunit gamma, mitochondrial	P36542	ATP5F1C	0.059

Cortex Lycii(CL)	MOL001552	OIN	Phosphatidylinositol-4,5-bisphosphate 3-kinase catalytic subunit gamma isoform	P48736	PIK3CG	0.059
Cortex Lycii(CL)	MOL001552	OIN	UDP-glucuronosyltransferase 3A1	Q6NUS8	UGT3A1	0.059
Cortex Lycii(CL)	MOL001552	OIN	Sodium/potassium-transporting ATPase alpha-1 chain	P05023	ATP1A1	0.116
Cortex Lycii(CL)	MOL001552	OIN	Solute carrier family 12 member 2	P55011	SLC12A2	0.116
Cortex Lycii(CL)	MOL001552	OIN	Calcium-activated potassium channel subunit alpha 1	Q12791	KCNMA1	0.116
Cortex Lycii(CL)	MOL001552	OIN	Solute carrier family 12 member 1	Q13621	SLC12A1	0.116
Cortex Lycii(CL)	MOL001552	OIN	ATP-sensitive inward rectifier potassium channel 11	Q14654	KCNJ11	0.116
Cortex Lycii(CL)	MOL001552	OIN	5-hydroxytryptamine 3 receptor	P46098	HTR3A	0.141
Cortex Lycii(CL)	MOL001552	OIN	Glutamate [NMDA] receptor subunit 3A	Q8TCU5	GRIN3A	0.141
Cortex Lycii(CL)	MOL001552	OIN	Neuronal acetylcholine receptor subunit alpha-10	Q9GZZ6	CHRNA10	0.141
Cortex Lycii(CL)	MOL001552	OIN	Amiloride-sensitive sodium channel subunit alpha	P37088	SCNN1A	0.148
Cortex Lycii(CL)	MOL001552	OIN	Amiloride-sensitive sodium channel subunit beta	P51168	SCNN1B	0.148
Cortex Lycii(CL)	MOL001552	OIN	Amiloride-sensitive sodium channel subunit gamma	P51170	SCNN1G	0.148
Cortex Lycii(CL)	MOL001552	OIN	Tyrosine-protein kinase HCK	P08631	HCK	0.153
Cortex Lycii(CL)	MOL001552	OIN	Neuronal acetylcholine receptor subunit alpha-7	P36544	CHRNA7	0.159
Cortex Lycii(CL)	MOL001552	OIN	Neuronal acetylcholine receptor subunit alpha-4	P43681	CHRNA4	0.159
Cortex Lycii(CL)	MOL001552	OIN	Glutamate receptor, ionotropic kainate 2	Q13002	GRIK2	0.159
Cortex Lycii(CL)	MOL001552	OIN	Sodium channel protein type 5 subunit alpha	Q14524	SCN5A	0.161
Cortex Lycii(CL)	MOL001552	OIN	Sodium channel protein type 11 subunit alpha	Q9UI33	SCN11A	0.161
Cortex Lycii(CL)	MOL001552	OIN	Muscarinic acetylcholine receptor M2	P08172	CHRM2	0.175
Cortex Lycii(CL)	MOL001552	OIN	Muscarinic acetylcholine receptor M4	P08173	CHRM4	0.175
Cortex Lycii(CL)	MOL001552	OIN	Sucrase-isomaltase, intestinal	P14410	SI	0.175
Cortex Lycii(CL)	MOL001552	OIN	D(4) dopamine receptor	P21917	DRD4	0.185
Cortex Lycii(CL)	MOL001552	OIN	5-hydroxytryptamine 2A receptor	P28223	HTR2A	0.185
Cortex Lycii(CL)	MOL001552	OIN	D(3) dopamine receptor	P35462	DRD3	0.185
Cortex Lycii(CL)	MOL001552	OIN	Gamma-aminobutyric-acid receptor subunit alpha-1	P14867	GABRA1	0.207
Cortex Lycii(CL)	MOL001552	OIN	Gamma-aminobutyric-acid receptor subunit alpha-5	P31644	GABRA5	0.207
Cortex Lycii(CL)	MOL001552	OIN	Gamma-aminobutyric-acid receptor subunit alpha-3	P34903	GABRA3	0.207
Cortex Lycii(CL)	MOL001552	OIN	Gamma-aminobutyric-acid receptor subunit alpha-2	P47869	GABRA2	0.207
Cortex Lycii(CL)	MOL001552	OIN	Gamma-aminobutyric-acid receptor subunit alpha-4	P48169	GABRA4	0.207
Cortex Lycii(CL)	MOL001552	OIN	Gamma-aminobutyric-acid receptor subunit alpha-6	Q16445	GABRA6	0.207
Cortex Lycii(CL)	MOL001552	OIN	Rho-associated protein kinase 1	Q13464	ROCK1	0.248
Cortex Lycii(CL)	MOL001552	OIN	cAMP-dependent protein kinase inhibitor alpha	P61925	PKIA	0.249
Cortex Lycii(CL)	MOL001552	OIN	Carbonic anhydrase 2	P00918	CA2	0.267
Cortex Lycii(CL)	MOL001552	OIN	Sodium channel protein type 10 subunit alpha	Q9Y5Y9	SCN10A	0.337
Cortex Lycii(CL)	MOL001552	OIN	Sodium-dependent serotonin transporter	P31645	SLC6A4	0.358
Cortex Lycii(CL)	MOL001552	OIN	Muscarinic acetylcholine receptor M3	P20309	CHRM3	0.384
Cortex Lycii(CL)	MOL001552	OIN	Carbonic anhydrase 1	P00915	CA1	0.417
Cortex Lycii(CL)	MOL001552	OIN	Nitric oxide synthase, inducible	P35228	NOS2	0.47
Cortex Lycii(CL)	MOL001552	OIN	MAP kinase-activated protein kinase 2	P49137	MAPKAPK2	0.48
Cortex Lycii(CL)	MOL001552	OIN	Dihydrofolate reductase	P00374	DHFR	0.484
Cortex Lycii(CL)	MOL001552	OIN	Lysozyme C	P61626	LYZ	0.531
Cortex Lycii(CL)	MOL001552	OIN	ATP-binding cassette transporter sub-family C member 8	Q09428	ABCC8	0.531
Cortex Lycii(CL)	MOL001552	OIN	cAMP-dependent protein kinase catalytic subunit alpha	P17612	PRKACA	0.532
Cortex Lycii(CL)	MOL001552	OIN	Sodium-dependent dopamine transporter	Q01959	SLC6A3	0.554
Cortex Lycii(CL)	MOL001552	OIN	D(2) dopamine receptor	P14416	DRD2	0.614
Cortex Lycii(CL)	MOL001552	OIN	Sodium-dependent noradrenaline transporter	P23975	SLC6A2	0.75
Cortex Lycii(CL)	MOL001552	OIN	Muscarinic acetylcholine receptor M1	P11229	CHRM1	1
Cortex Lycii(CL)	MOL001645	Linoleyl acetate	SEC14-like protein 2	O76054	SEC14L2	0.019
Cortex Lycii(CL)	MOL001645	Linoleyl acetate	Alpha-lactalbumin	P00709	LALBA	0.019
Cortex Lycii(CL)	MOL001645	Linoleyl acetate	Glycodelin	P09466	PAEP	0.019
Cortex Lycii(CL)	MOL001645	Linoleyl acetate	Cytochrome P450 2C8	P10632	CYP2C8	0.019
Cortex Lycii(CL)	MOL001645	Linoleyl acetate	Hepatocyte nuclear factor 4-gamma	Q14541	HNF4G	0.019

Cortex Lycii(CL)	MOL001645	Linoleyl acetate	Lymphocyte antigen 96	Q9Y6Y9	LY96	0.053
Cortex Lycii(CL)	MOL001645	Linoleyl acetate	Dihydroorotate dehydrogenase, mitochondrial	Q02127	DHODH	0.058
Cortex Lycii(CL)	MOL001645	Linoleyl acetate	Trafficking protein particle complex subunit 3	O43617	TRAPPC3	0.071
Cortex Lycii(CL)	MOL001645	Linoleyl acetate	Myelin P2 protein	P02689	PMP2	0.073
Cortex Lycii(CL)	MOL001645	Linoleyl acetate	Carbonic anhydrase 1	P00915	CA1	0.118
Cortex Lycii(CL)	MOL001645	Linoleyl acetate	Carbonic anhydrase 2	P00918	CA2	0.118
Cortex Lycii(CL)	MOL001645	Linoleyl acetate	Carbonic anhydrase 4	P22748	CA4	0.118
Cortex Lycii(CL)	MOL001645	Linoleyl acetate	Solute carrier family 12 member 3	P55017	SLC12A3	0.118
Cortex Lycii(CL)	MOL001645	Linoleyl acetate	Carbonic anhydrase 9	Q16790	CA9	0.118
Cortex Lycii(CL)	MOL001645	Linoleyl acetate	Retinoic acid receptor alpha	P10276	RARA	0.119
Cortex Lycii(CL)	MOL001645	Linoleyl acetate	Retinoic acid receptor beta	P10826	RARB	0.119
Cortex Lycii(CL)	MOL001645	Linoleyl acetate	Retinoic acid receptor gamma-1	P13631	RARG	0.119
Cortex Lycii(CL)	MOL001645	Linoleyl acetate	Retinoic acid receptor RXR-beta	P28702	RXRB	0.119
Cortex Lycii(CL)	MOL001645	Linoleyl acetate	Retinoic acid receptor RXR-gamma	P48443	RXRG	0.119
Cortex Lycii(CL)	MOL001645	Linoleyl acetate	Rhodopsin	P08100	RHO	0.138
Cortex Lycii(CL)	MOL001645	Linoleyl acetate	Furin	P09958	FURIN	0.207
Cortex Lycii(CL)	MOL001645	Linoleyl acetate	Beta-1 adrenergic receptor	P08588	ADRB1	0.234
Cortex Lycii(CL)	MOL001645	Linoleyl acetate	Progesterone receptor	P06401	PGR	0.36
Cortex Lycii(CL)	MOL001645	Linoleyl acetate	Mineralocorticoid receptor	P08235	NR3C2	0.36
Cortex Lycii(CL)	MOL001645	Linoleyl acetate	Gamma-aminobutyric-acid receptor subunit alpha-1	P14867	GABRA1	0.417
Cortex Lycii(CL)	MOL001645	Linoleyl acetate	Gamma-aminobutyric-acid receptor subunit alpha-2	P47869	GABRA2	0.417
Cortex Lycii(CL)	MOL001645	Linoleyl acetate	Phospholipase A2	P04054	PLA2G1B	0.426
Cortex Lycii(CL)	MOL001645	Linoleyl acetate	Phospholipase A2, membrane associated	P14555	PLA2G2A	0.426
Cortex Lycii(CL)	MOL001645	Linoleyl acetate	Glycolipid transfer protein	Q9NZD2	GLTP	0.469
Cortex Lycii(CL)	MOL001645	Linoleyl acetate	Cytohesin-2	Q99418	CYTH2	0.83
Cortex Lycii(CL)	MOL001645	Linoleyl acetate	Cannabinoid receptor 1	P21554	CNR1	0.835
Cortex Lycii(CL)	MOL001645	Linoleyl acetate	Colipase	P04118	CLPS	0.927
Cortex Lycii(CL)	MOL001645	Linoleyl acetate	Lysozyme C	P61626	LYZ	0.944
Cortex Lycii(CL)	MOL001645	Linoleyl acetate	Vitamin K-dependent protein C	P04070	PROC	0.984
Cortex Lycii(CL)	MOL001689	acacetin	5-hydroxytryptamine 7 receptor	P34969	HTR7	0.01
Cortex Lycii(CL)	MOL001689	acacetin	5-hydroxytryptamine 6 receptor	P50406	HTR6	0.01
Cortex Lycii(CL)	MOL001689	acacetin	Potassium voltage-gated channel subfamily H member 2	Q12809	KCNH2	0.01
Cortex Lycii(CL)	MOL001689	acacetin	Acetylcholine receptor subunit alpha	P02708	CHRNA1	0.011
Cortex Lycii(CL)	MOL001689	acacetin	Cholinesterase	P06276	BCHE	0.011
Cortex Lycii(CL)	MOL001689	acacetin	Acetylcholine receptor subunit gamma	P07510	CHRNG	0.011
Cortex Lycii(CL)	MOL001689	acacetin	Acetylcholine receptor subunit beta	P11230	CHRNB1	0.011
Cortex Lycii(CL)	MOL001689	acacetin	Neuronal acetylcholine receptor subunit beta-2	P17787	CHRNB2	0.011
Cortex Lycii(CL)	MOL001689	acacetin	Neuronal acetylcholine receptor subunit alpha-5	P30532	CHRNA5	0.011
Cortex Lycii(CL)	MOL001689	acacetin	Neuronal acetylcholine receptor subunit beta-4	P30926	CHRNB4	0.011
Cortex Lycii(CL)	MOL001689	acacetin	Neuronal acetylcholine receptor subunit alpha-3	P32297	CHRNA3	0.011
Cortex Lycii(CL)	MOL001689	acacetin	Neuronal acetylcholine receptor subunit alpha-7	P36544	CHRNA7	0.011
Cortex Lycii(CL)	MOL001689	acacetin	Acetylcholine receptor subunit epsilon	Q04844	CHRNE	0.011
Cortex Lycii(CL)	MOL001689	acacetin	Neuronal acetylcholine receptor subunit beta-3	Q05901	CHRNB3	0.011
Cortex Lycii(CL)	MOL001689	acacetin	Acetylcholine receptor subunit delta	Q07001	CHRND	0.011
Cortex Lycii(CL)	MOL001689	acacetin	Neuronal acetylcholine receptor subunit alpha-2	Q15822	CHRNA2	0.011
Cortex Lycii(CL)	MOL001689	acacetin	Neuronal acetylcholine receptor subunit alpha-6	Q15825	KCNJ8	0.011
Cortex Lycii(CL)	MOL001689	acacetin	2,4-dienoyl-CoA reductase, mitochondrial	Q16698	DECR1	0.011
Cortex Lycii(CL)	MOL001689	acacetin	Neuronal acetylcholine receptor subunit alpha-9	Q9UGM1	CHRNA9	0.011
Cortex Lycii(CL)	MOL001689	acacetin	Glutathione S-transferase P	P09211	GSTP1	0.012
Cortex Lycii(CL)	MOL001689	acacetin	Gamma-aminobutyric acid receptor subunit rho-3	A8MPY1	GABRR3	0.014
Cortex Lycii(CL)	MOL001689	acacetin	Gamma-aminobutyric acid receptor subunit pi	O00591	GABRP	0.014
Cortex Lycii(CL)	MOL001689	acacetin	Gamma-aminobutyric acid receptor subunit delta	O14764	GABRD	0.014
Cortex Lycii(CL)	MOL001689	acacetin	Death-associated protein kinase 3	O43293	DAPK3	0.014

Cortex Lycii(CL)	MOL001689	acacetin	Tyrosine-protein kinase JAK2	O60674	JAK2	0.014
Cortex Lycii(CL)	MOL001689	acacetin	Gamma-aminobutyric-acid receptor subunit beta-1	P18505	GABRB1	0.014
Cortex Lycii(CL)	MOL001689	acacetin	Gamma-aminobutyric acid receptor subunit gamma-2	P18507	GABRG2	0.014
Cortex Lycii(CL)	MOL001689	acacetin	Tyrosine-protein kinase JAK1	P23458	JAK1	0.014
Cortex Lycii(CL)	MOL001689	acacetin	Gamma-aminobutyric-acid receptor subunit rho-1	P24046	GABRR1	0.014
Cortex Lycii(CL)	MOL001689	acacetin	Gamma-aminobutyric acid receptor subunit rho-2	P28476	GABRR2	0.014
Cortex Lycii(CL)	MOL001689	acacetin	Gamma-aminobutyric-acid receptor subunit alpha-5	P31644	GABRA5	0.014
Cortex Lycii(CL)	MOL001689	acacetin	Gamma-aminobutyric-acid receptor subunit alpha-3	P34903	GABRA3	0.014
Cortex Lycii(CL)	MOL001689	acacetin	Tyrosine-protein kinase JAK3	P52333	JAK3	0.014
Cortex Lycii(CL)	MOL001689	acacetin	Gamma-aminobutyric acid receptor subunit epsilon	P78334	GABRE	0.014
Cortex Lycii(CL)	MOL001689	acacetin	Gamma-aminobutyric acid receptor subunit gamma-1	Q8N1C3	GABRG1	0.014
Cortex Lycii(CL)	MOL001689	acacetin	Gamma-aminobutyric acid receptor subunit gamma-3	Q99928	GABRG3	0.014
Cortex Lycii(CL)	MOL001689	acacetin	Potassium channel subfamily K member 6	Q9Y257	KCNK6	0.016
Cortex Lycii(CL)	MOL001689	acacetin	5-hydroxytryptamine 4 receptor	Q13639	HTR4	0.017
Cortex Lycii(CL)	MOL001689	acacetin	Interleukin-3	P08700	IL3	0.019
Cortex Lycii(CL)	MOL001689	acacetin	Protein S100-A12	P80511	S100A12	0.019
Cortex Lycii(CL)	MOL001689	acacetin	Protein S100-A13	Q99584	S100A13	0.019
Cortex Lycii(CL)	MOL001689	acacetin	Purine nucleoside phosphorylase	P00491	PNP	0.02
Cortex Lycii(CL)	MOL001689	acacetin	Ribonucleoside-diphosphate reductase M2 subunit	P31350	RRM2	0.02
Cortex Lycii(CL)	MOL001689	acacetin	DNA polymerase epsilon subunit 2	P56282	POLE2	0.02
Cortex Lycii(CL)	MOL001689	acacetin	DNA polymerase epsilon catalytic subunit A	Q07864	POLE	0.02
Cortex Lycii(CL)	MOL001689	acacetin	Ribonucleoside-diphosphate reductase subunit M2 B	Q7LG56	RRM2B	0.02
Cortex Lycii(CL)	MOL001689	acacetin	DNA polymerase epsilon subunit 3	Q9NRF9	POLE3	0.02
Cortex Lycii(CL)	MOL001689	acacetin	Serine/threonine-protein kinase 17B	O94768	STK17B	0.022
Cortex Lycii(CL)	MOL001689	acacetin	Coagulation factor IX	P00740	F9	0.022
Cortex Lycii(CL)	MOL001689	acacetin	Coagulation factor X	P00742	F10	0.022
Cortex Lycii(CL)	MOL001689	acacetin	Osteocalcin	P02818	BGLAP	0.022
Cortex Lycii(CL)	MOL001689	acacetin	Vitamin K-dependent protein C	P04070	PROC	0.022
Cortex Lycii(CL)	MOL001689	acacetin	Cell division protein kinase 4	P11802	CDK4	0.022
Cortex Lycii(CL)	MOL001689	acacetin	NAD(P)H dehydrogenase [quinone] 1	P15559	NQO1	0.022
Cortex Lycii(CL)	MOL001689	acacetin	Vitamin K-dependent protein Z	P22891	PROZ	0.022
Cortex Lycii(CL)	MOL001689	acacetin	ATP synthase subunit gamma, mitochondrial	P36542	ATP5F1C	0.022
Cortex Lycii(CL)	MOL001689	acacetin	Amiloride-sensitive sodium channel subunit alpha	P37088	SCNN1A	0.022
Cortex Lycii(CL)	MOL001689	acacetin	Vitamin K-dependent gamma-carboxylase	P38435	GGCX	0.022
Cortex Lycii(CL)	MOL001689	acacetin	Cell division protein kinase 7	P50613	CDK7	0.022
Cortex Lycii(CL)	MOL001689	acacetin	Cell division protein kinase 9	P50750	CDK9	0.022
Cortex Lycii(CL)	MOL001689	acacetin	Amiloride-sensitive sodium channel subunit beta	P51168	SCNN1B	0.022
Cortex Lycii(CL)	MOL001689	acacetin	Amiloride-sensitive sodium channel subunit gamma	P51170	SCNN1G	0.022
Cortex Lycii(CL)	MOL001689	acacetin	UDP-glucuronosyltransferase 3A1	Q6NUS8	UGT3A1	0.022
Cortex Lycii(CL)	MOL001689	acacetin	Vitamin K epoxide reductase complex subunit 1-like protein 1	Q8N0U8	VKORC1L1	0.022
Cortex Lycii(CL)	MOL001689	acacetin	Vitamin K epoxide reductase complex subunit 1	Q9BQB6	VKORC1	0.022
Cortex Lycii(CL)	MOL001689	acacetin	Retinoic acid receptor beta	P10826	RARB	0.023
Cortex Lycii(CL)	MOL001689	acacetin	cAMP response element-binding protein	P16220	CREB1	0.023
Cortex Lycii(CL)	MOL001689	acacetin	Carbonic anhydrase 4	P22748	CA4	0.023
Cortex Lycii(CL)	MOL001689	acacetin	Nociceptin receptor	P41146	OPRL1	0.023
Cortex Lycii(CL)	MOL001689	acacetin	Retinoic acid receptor RXR-gamma	P48443	RXRG	0.023
Cortex Lycii(CL)	MOL001689	acacetin	Sodium/potassium-transporting ATPase gamma chain	P54710	FXYD2	0.023
Cortex Lycii(CL)	MOL001689	acacetin	Solute carrier family 12 member 3	P55017	SLC12A3	0.023
Cortex Lycii(CL)	MOL001689	acacetin	Calcium-activated potassium channel subunit alpha 1	Q12791	KCNMA1	0.023
Cortex Lycii(CL)	MOL001689	acacetin	3-phosphoinositide-dependent protein kinase 1	O15530	PDPK1	0.024
Cortex Lycii(CL)	MOL001689	acacetin	Tyrosine-protein kinase Lyn	P07948	LYN	0.024
Cortex Lycii(CL)	MOL001689	acacetin	D(1A) dopamine receptor	P21728	DRD1	0.024
Cortex Lycii(CL)	MOL001689	acacetin	D(4) dopamine receptor	P21917	DRD4	0.024

Cortex Lycii(CL)	MOL001689	acacetin	D(1B) dopamine receptor	P21918	DRD5	0.024
Cortex Lycii(CL)	MOL001689	acacetin	Alpha-1D adrenergic receptor	P25100	ADRA1D	0.024
Cortex Lycii(CL)	MOL001689	acacetin	Tyrosine-protein kinase CSK	P41240	CSK	0.024
Cortex Lycii(CL)	MOL001689	acacetin	Tyrosine-protein kinase ZAP-70	P43403	ZAP70	0.024
Cortex Lycii(CL)	MOL001689	acacetin	Tyrosine-protein kinase SYK	P43405	SYK	0.024
Cortex Lycii(CL)	MOL001689	acacetin	Protein kinase C theta type	Q04759	PRKCQ	0.024
Cortex Lycii(CL)	MOL001689	acacetin	Tyrosine-protein kinase ITK/TSK	Q08881	ITK	0.024
Cortex Lycii(CL)	MOL001689	acacetin	Cyclin-dependent kinase 5 activator 1	Q15078	CDK5R1	0.024
Cortex Lycii(CL)	MOL001689	acacetin	Solute carrier family 12 member 2	P55011	SLC12A2	0.026
Cortex Lycii(CL)	MOL001689	acacetin	Solute carrier family 12 member 5	Q9H2X9	SLC12A5	0.026
Cortex Lycii(CL)	MOL001689	acacetin	Solute carrier family 12 member 4	Q9UP95	SLC12A4	0.026
Cortex Lycii(CL)	MOL001689	acacetin	DNA polymerase subunit alpha B	Q14181	POLA2	0.027
Cortex Lycii(CL)	MOL001689	acacetin	Prostaglandin reductase 1	Q14914	PTGR1	0.027
Cortex Lycii(CL)	MOL001689	acacetin	Thymidylate synthase	P04818	TYMS	0.028
Cortex Lycii(CL)	MOL001689	acacetin	Bifunctional purine biosynthesis protein PURH [Includes: Phosphoribosylaminoimidazolecarboxamide formyltransferase	P31939	ATIC	0.028
Cortex Lycii(CL)	MOL001689	acacetin	Gamma-aminobutyric-acid receptor subunit alpha-1	P14867	GABRA1	0.03
Cortex Lycii(CL)	MOL001689	acacetin	Gamma-aminobutyric-acid receptor subunit alpha-2	P47869	GABRA2	0.03
Cortex Lycii(CL)	MOL001689	acacetin	Potassium channel subfamily K member 1	O00180	KCNK1	0.031
Cortex Lycii(CL)	MOL001689	acacetin	Inhibitor of nuclear factor kappa-B kinase subunit beta	O14920	IKBKB	0.031
Cortex Lycii(CL)	MOL001689	acacetin	Lipoic acid synthetase, mitochondrial	O43766	LIAS	0.031
Cortex Lycii(CL)	MOL001689	acacetin	Urokinase-type plasminogen activator	P00749	PLAU	0.031
Cortex Lycii(CL)	MOL001689	acacetin	Phospholipase A2, membrane associated	P14555	PLA2G2A	0.031
Cortex Lycii(CL)	MOL001689	acacetin	Lactoylglutathione lyase	Q04760	GLO1	0.031
Cortex Lycii(CL)	MOL001689	acacetin	Prostaglandin reductase 2	Q8N8N7	PTGR2	0.031
Cortex Lycii(CL)	MOL001689	acacetin	Neuronal acetylcholine receptor subunit alpha-10	Q9GZZ6	CHRNA10	0.031
Cortex Lycii(CL)	MOL001689	acacetin	Group IIE secretory phospholipase A2	Q9NZK7	PLA2G2E	0.031
Cortex Lycii(CL)	MOL001689	acacetin	Cystine/glutamate transporter	Q9UPY5	SLC7A11	0.031
Cortex Lycii(CL)	MOL001689	acacetin	Lipoyltransferase 1, mitochondrial	Q9Y234	LIPT1	0.031
Cortex Lycii(CL)	MOL001689	acacetin	DNA topoisomerase 1	P11387	TOP1	0.032
Cortex Lycii(CL)	MOL001689	acacetin	Alcohol dehydrogenase [NADP+]	P14550	AKR1A1	0.032
Cortex Lycii(CL)	MOL001689	acacetin	Aldose reductase	P15121	AKR1B1	0.032
Cortex Lycii(CL)	MOL001689	acacetin	DNA topoisomerase I, mitochondrial	Q969P6	TOP1MT	0.032
Cortex Lycii(CL)	MOL001689	acacetin	Casein kinase I isoform gamma-2	P78368	CSNK1G2	0.033
Cortex Lycii(CL)	MOL001689	acacetin	Serine/threonine-protein kinase haspin	Q8TF76	HASPIN	0.033
Cortex Lycii(CL)	MOL001689	acacetin	DNA-(apurinic or apyrimidinic site) lyase	P27695	APEX1	0.036
Cortex Lycii(CL)	MOL001689	acacetin	cGMP-inhibited 3',5'-cyclic phosphodiesterase A	Q14432	PDE3A	0.036
Cortex Lycii(CL)	MOL001689	acacetin	cAMP and cAMP-inhibited cGMP 3',5'-cyclic phosphodiesterase 10A	Q9Y233	PDE10A	0.036
Cortex Lycii(CL)	MOL001689	acacetin	cAMP-specific 3',5'-cyclic phosphodiesterase 4C	Q08493	PDE4C	0.037
Cortex Lycii(CL)	MOL001689	acacetin	5-hydroxytryptamine 1B receptor	P28222	HTR1B	0.038
Cortex Lycii(CL)	MOL001689	acacetin	Alpha-1B adrenergic receptor	P35368	ADRA1B	0.038
Cortex Lycii(CL)	MOL001689	acacetin	Glutamate [NMDA] receptor subunit 3A	Q8TCU5	GRIN3A	0.038
Cortex Lycii(CL)	MOL001689	acacetin	Acetylcholinesterase	P22303	ACHE	0.04
Cortex Lycii(CL)	MOL001689	acacetin	Glycogen phosphorylase, muscle form	P11217	PYGM	0.041
Cortex Lycii(CL)	MOL001689	acacetin	5-hydroxytryptamine 2A receptor	P28223	HTR2A	0.041
Cortex Lycii(CL)	MOL001689	acacetin	5-hydroxytryptamine 2B receptor	P41595	HTR2B	0.041
Cortex Lycii(CL)	MOL001689	acacetin	DNA polymerase alpha catalytic subunit	P09884	POLA1	0.042
Cortex Lycii(CL)	MOL001689	acacetin	Ribonucleoside-diphosphate reductase large subunit	P23921	RRM1	0.042
Cortex Lycii(CL)	MOL001689	acacetin	Retinal rod rhodopsin-sensitive cGMP 3',5'-cyclic phosphodiesterase subunit gamma	P18545	PDE6G	0.044
Cortex Lycii(CL)	MOL001689	acacetin	Mitogen-activated protein kinase 8	P45983	MAPK8	0.045
Cortex Lycii(CL)	MOL001689	acacetin	Mitogen-activated protein kinase 10	P53779	MAPK10	0.045
Cortex Lycii(CL)	MOL001689	acacetin	Sterol O-acyltransferase 2	O75908	SOAT2	0.046
Cortex Lycii(CL)	MOL001689	acacetin	Keratin, type II cytoskeletal 7	P08729	KRT7	0.046

Cortex Lycii(CL)	MOL001689	acacetin	Platelet glycoprotein IX	P14770	GP9	0.046
Cortex Lycii(CL)	MOL001689	acacetin	Gonadotropin-releasing hormone receptor	P30968	GNRHR	0.046
Cortex Lycii(CL)	MOL001689	acacetin	Sterol O-acyltransferase 1	P35610	SOAT1	0.046
Cortex Lycii(CL)	MOL001689	acacetin	Gonadotropin-releasing hormone II receptor	Q96P88	GNRHR2	0.046
Cortex Lycii(CL)	MOL001689	acacetin	Coagulation factor VII	P08709	F7	0.047
Cortex Lycii(CL)	MOL001689	acacetin	Nitric-oxide synthase, brain	P29475	NOS1	0.047
Cortex Lycii(CL)	MOL001689	acacetin	Epidermal growth factor receptor	P00533	EGFR	0.048
Cortex Lycii(CL)	MOL001689	acacetin	ATP synthase subunit beta, mitochondrial	P06576	ATP5F1B	0.048
Cortex Lycii(CL)	MOL001689	acacetin	Tyrosine-protein kinase HCK	P08631	HCK	0.048
Cortex Lycii(CL)	MOL001689	acacetin	ATP synthase subunit alpha, mitochondrial	P25705	ATP5F1A	0.048
Cortex Lycii(CL)	MOL001689	acacetin	Phosphatidylinositol-4,5-bisphosphate 3-kinase catalytic subunit gamma isoform	P48736	PIK3CG	0.048
Cortex Lycii(CL)	MOL001689	acacetin	Cell division protein kinase 6	Q00534	CDK6	0.048
Cortex Lycii(CL)	MOL001689	acacetin	Sodium-dependent serotonin transporter	P31645	SLC6A4	0.05
Cortex Lycii(CL)	MOL001689	acacetin	Tubulin beta-2C chain	P68371	TUBB4B	0.05
Cortex Lycii(CL)	MOL001689	acacetin	Tripartite motif-containing protein 13	O60858	TRIM13	0.051
Cortex Lycii(CL)	MOL001689	acacetin	Retinoic acid receptor alpha	P10276	RARA	0.051
Cortex Lycii(CL)	MOL001689	acacetin	Proto-oncogene serine/threonine-protein kinase Pim-1	P11309	PIM1	0.051
Cortex Lycii(CL)	MOL001689	acacetin	Retinoic acid receptor gamma-1	P13631	RARG	0.051
Cortex Lycii(CL)	MOL001689	acacetin	RAC-beta serine/threonine-protein kinase	P31751	AKT2	0.051
Cortex Lycii(CL)	MOL001689	acacetin	Alpha-1A adrenergic receptor	P35348	ADRA1A	0.051
Cortex Lycii(CL)	MOL001689	acacetin	Alpha-2A adrenergic receptor	P08913	ADRA2A	0.052
Cortex Lycii(CL)	MOL001689	acacetin	3 beta-hydroxysteroid dehydrogenase/Delta 5-->4-isomerase type I	P14060	HSD3B1	0.052
Cortex Lycii(CL)	MOL001689	acacetin	Alpha-2C adrenergic receptor	P18825	ADRA2C	0.052
Cortex Lycii(CL)	MOL001689	acacetin	3 beta-hydroxysteroid dehydrogenase/Delta 5-->4-isomerase type II	P26439	HSD3B2	0.052
Cortex Lycii(CL)	MOL001689	acacetin	5-hydroxytryptamine 1D receptor	P28221	HTR1D	0.052
Cortex Lycii(CL)	MOL001689	acacetin	D(3) dopamine receptor	P35462	DRD3	0.052
Cortex Lycii(CL)	MOL001689	acacetin	Estrogen-related receptor gamma	P62508	ESRRG	0.052
Cortex Lycii(CL)	MOL001689	acacetin	Rho-associated protein kinase 1	Q13464	ROCK1	0.052
Cortex Lycii(CL)	MOL001689	acacetin	Protein tyrosine kinase 2 beta	Q14289	PTK2B	0.052
Cortex Lycii(CL)	MOL001689	acacetin	Proto-oncogene tyrosine-protein kinase LCK	P06239	LCK	0.053
Cortex Lycii(CL)	MOL001689	acacetin	Ribosyldihydronicotinamide dehydrogenase [quinone]	P16083	NQO2	0.054
Cortex Lycii(CL)	MOL001689	acacetin	Retinoic acid receptor RXR-alpha	P19793	RXRA	0.055
Cortex Lycii(CL)	MOL001689	acacetin	Solute carrier family 12 member 1	Q13621	SLC12A1	0.057
Cortex Lycii(CL)	MOL001689	acacetin	Sodium-dependent dopamine transporter	Q01959	SLC6A3	0.059
Cortex Lycii(CL)	MOL001689	acacetin	Sodium channel protein type 10 subunit alpha	Q9Y5Y9	SCN10A	0.059
Cortex Lycii(CL)	MOL001689	acacetin	5-hydroxytryptamine 1A receptor	P08908	HTR1A	0.06
Cortex Lycii(CL)	MOL001689	acacetin	5-hydroxytryptamine 3 receptor	P46098	HTR3A	0.06
Cortex Lycii(CL)	MOL001689	acacetin	Casein kinase II subunit alpha	P68400	CSNK2A1	0.06
Cortex Lycii(CL)	MOL001689	acacetin	Toll-like receptor 7	Q9NYK1	TLR7	0.06
Cortex Lycii(CL)	MOL001689	acacetin	Thyroid hormone receptor beta-1	P10828	THRB	0.066
Cortex Lycii(CL)	MOL001689	acacetin	D(2) dopamine receptor	P14416	DRD2	0.066
Cortex Lycii(CL)	MOL001689	acacetin	Alpha-2B adrenergic receptor	P18089	ADRA2B	0.066
Cortex Lycii(CL)	MOL001689	acacetin	Hepatocyte growth factor receptor	P08581	MET	0.067
Cortex Lycii(CL)	MOL001689	acacetin	Nuclear receptor coactivator 2	Q15596	NCOA2	0.068
Cortex Lycii(CL)	MOL001689	acacetin	Mitogen-activated protein kinase 14	Q16539	MAPK14	0.068
Cortex Lycii(CL)	MOL001689	acacetin	Membrane copper amine oxidase	Q16853	AOC3	0.068
Cortex Lycii(CL)	MOL001689	acacetin	Endothelin-1 receptor	P25101	EDNRA	0.069
Cortex Lycii(CL)	MOL001689	acacetin	5-hydroxytryptamine 2C receptor	P28335	HTR2C	0.069
Cortex Lycii(CL)	MOL001689	acacetin	Protein farnesyltransferase/geranylgeranyltransferase type I alpha subunit	P49354	FNTA	0.069
Cortex Lycii(CL)	MOL001689	acacetin	Leukotriene A-4 hydrolase	P09960	LTA4H	0.07
Cortex Lycii(CL)	MOL001689	acacetin	Cannabinoid receptor 2	P34972	CNR2	0.07
Cortex Lycii(CL)	MOL001689	acacetin	Cytochrome P450 11B1, mitochondrial	P15538	CYP11B1	0.071

Cortex Lycii(CL)	MOL001689	acacetin	Cannabinoid receptor 1	P21554	CNR1	0.071
Cortex Lycii(CL)	MOL001689	acacetin	L-amino-acid oxidase	Q96RQ9	IL4I1	0.071
Cortex Lycii(CL)	MOL001689	acacetin	Geranylgeranyl pyrophosphate synthetase	O95749	GGPS1	0.072
Cortex Lycii(CL)	MOL001689	acacetin	Ig kappa chain V-II region RPMI 6410	P06310	IGKV2-30	0.072
Cortex Lycii(CL)	MOL001689	acacetin	ATP-sensitive inward rectifier potassium channel 1	P48048	KCNJ1	0.072
Cortex Lycii(CL)	MOL001689	acacetin	Serine/threonine-protein kinase 6	O14965	AURKA	0.073
Cortex Lycii(CL)	MOL001689	acacetin	Sodium-dependent noradrenaline transporter	P23975	SLC6A2	0.073
Cortex Lycii(CL)	MOL001689	acacetin	RAC-alpha serine/threonine-protein kinase	P31749	AKT1	0.073
Cortex Lycii(CL)	MOL001689	acacetin	Activin receptor type-1	Q04771	ACVR1	0.074
Cortex Lycii(CL)	MOL001689	acacetin	Nuclear receptor coactivator 5	Q9HCD5	NCOA5	0.074
Cortex Lycii(CL)	MOL001689	acacetin	Thyroid hormone receptor alpha	P10827	THRA	0.075
Cortex Lycii(CL)	MOL001689	acacetin	Muscarinic acetylcholine receptor M2	P08172	CHRM2	0.076
Cortex Lycii(CL)	MOL001689	acacetin	S-methyl-5-thioadenosine phosphorylase	Q13126	MTAP	0.077
Cortex Lycii(CL)	MOL001689	acacetin	Calcium/calmodulin-dependent protein kinase type II alpha chain	Q9UQM7	CAMK2A	0.077
Cortex Lycii(CL)	MOL001689	acacetin	D-HSCDK2	O75100	CA11	0.078
Cortex Lycii(CL)	MOL001689	acacetin	Carbonic anhydrase 1	P00915	CA1	0.078
Cortex Lycii(CL)	MOL001689	acacetin	Tyrosine-protein phosphatase non-receptor type 1	P18031	PTPN1	0.079
Cortex Lycii(CL)	MOL001689	acacetin	Retinoic acid receptor RXR-beta	P28702	RXRB	0.08
Cortex Lycii(CL)	MOL001689	acacetin	Glycogen synthase kinase-3 beta	P49841	GSK3B	0.081
Cortex Lycii(CL)	MOL001689	acacetin	Interferon gamma	P01579	IFNG	0.082
Cortex Lycii(CL)	MOL001689	acacetin	Melatonin receptor type 1A	P48039	MTNR1A	0.082
Cortex Lycii(CL)	MOL001689	acacetin	MAP kinase-activated protein kinase 2	P49137	MAPKAPK2	0.083
Cortex Lycii(CL)	MOL001689	acacetin	DNA polymerase kappa	Q9UBT6	POLK	0.088
Cortex Lycii(CL)	MOL001689	acacetin	cGMP-specific 3',5'-cyclic phosphodiesterase	O76074	PDE5A	0.092
Cortex Lycii(CL)	MOL001689	acacetin	DNA topoisomerase 2-alpha	P11388	TOP2A	0.092
Cortex Lycii(CL)	MOL001689	acacetin	Sodium channel protein type 5 subunit alpha	Q14524	SCN5A	0.092
Cortex Lycii(CL)	MOL001689	acacetin	Dihydrofolate reductase	P00374	DHFR	0.094
Cortex Lycii(CL)	MOL001689	acacetin	Progesterone receptor	P06401	PGR	0.094
Cortex Lycii(CL)	MOL001689	acacetin	Nitric-oxide synthase, endothelial	P29474	NOS3	0.098
Cortex Lycii(CL)	MOL001689	acacetin	Inhibitor of nuclear factor kappa-B kinase subunit alpha	O15111	CHUK	0.101
Cortex Lycii(CL)	MOL001689	acacetin	Arachidonate 5-lipoxygenase	P09917	ALOX5	0.101
Cortex Lycii(CL)	MOL001689	acacetin	Peroxisome proliferator-activated receptor gamma	P37231	PPARG	0.101
Cortex Lycii(CL)	MOL001689	acacetin	Tubulin alpha-3 chain	Q71U36	TUBA1A	0.103
Cortex Lycii(CL)	MOL001689	acacetin	Carbonic anhydrase 2	P00918	CA2	0.106
Cortex Lycii(CL)	MOL001689	acacetin	Cell division control protein 2 homolog	P06493	CDK1	0.106
Cortex Lycii(CL)	MOL001689	acacetin	cAMP-dependent protein kinase inhibitor alpha	P61925	PKIA	0.107
Cortex Lycii(CL)	MOL001689	acacetin	Prothrombin	P00734	F2	0.124
Cortex Lycii(CL)	MOL001689	acacetin	Delta-type opioid receptor	P41143	OPRD1	0.132
Cortex Lycii(CL)	MOL001689	acacetin	Cell division protein kinase 5	Q00535	CDK5	0.134
Cortex Lycii(CL)	MOL001689	acacetin	Androgen receptor	P10275	AR	0.142
Cortex Lycii(CL)	MOL001689	acacetin	cAMP-specific 3',5'-cyclic phosphodiesterase 4D	Q08499	PDE4D	0.142
Cortex Lycii(CL)	MOL001689	acacetin	C-jun-amino-terminal kinase-interacting protein 1	Q9UQF2	MAPK8IP1	0.143
Cortex Lycii(CL)	MOL001689	acacetin	Nitric oxide synthase, inducible	P35228	NOS2	0.144
Cortex Lycii(CL)	MOL001689	acacetin	Proto-oncogene tyrosine-protein kinase Src	P12931	SRC	0.156
Cortex Lycii(CL)	MOL001689	acacetin	cAMP-specific 3',5'-cyclic phosphodiesterase 4A	P27815	PDE4A	0.158
Cortex Lycii(CL)	MOL001689	acacetin	Kappa-type opioid receptor	P41145	OPRK1	0.159
Cortex Lycii(CL)	MOL001689	acacetin	cAMP-specific 3',5'-cyclic phosphodiesterase 4B	Q07343	PDE4B	0.159
Cortex Lycii(CL)	MOL001689	acacetin	Trypsin-1	P07477	PRSS1	0.217
Cortex Lycii(CL)	MOL001689	acacetin	Cell division protein kinase 2	P24941	CDK2	0.248
Cortex Lycii(CL)	MOL001689	acacetin	Hemoglobin subunit alpha	P69905	HBA1	0.262
Cortex Lycii(CL)	MOL001689	acacetin	Mu-type opioid receptor	P35372	OPRM1	0.289
Cortex Lycii(CL)	MOL001689	acacetin	Prostaglandin G/H synthase 1	P23219	PTGS1	0.306
Cortex Lycii(CL)	MOL001689	acacetin	cAMP-dependent protein kinase catalytic subunit alpha	P17612	PRKACA	0.387

Cortex Lycii(CL)	MOL001689	acacetin	Nuclear receptor coactivator 1	Q15788	NCOA1	0.415
Cortex Lycii(CL)	MOL001689	acacetin	Prostaglandin G/H synthase 2	P35354	PTGS2	0.557
Cortex Lycii(CL)	MOL001689	acacetin	Estrogen receptor beta	Q92731	ESR2	0.595
Cortex Lycii(CL)	MOL001689	acacetin	Cyclin-A2	P20248	CCNA2	0.676
Cortex Lycii(CL)	MOL001689	acacetin	Estrogen receptor	P03372	ESR1	1
Cortex Lycii(CL)	MOL002218	scopolin	Acetyl-CoA carboxylase 2	O00763	ACACB	0.014
Cortex Lycii(CL)	MOL002218	scopolin	Propionyl-CoA carboxylase alpha chain, mitochondrial	P05165	PCCA	0.014
Cortex Lycii(CL)	MOL002218	scopolin	Propionyl-CoA carboxylase beta chain, mitochondrial	P05166	PCCB	0.014
Cortex Lycii(CL)	MOL002218	scopolin	Pyruvate carboxylase, mitochondrial	P11498	PC	0.014
Cortex Lycii(CL)	MOL002218	scopolin	Biotin--protein ligase	P50747	HLCS	0.014
Cortex Lycii(CL)	MOL002218	scopolin	Methylcrotonoyl-CoA carboxylase subunit alpha, mitochondrial	Q96RQ3	MCCC1	0.014
Cortex Lycii(CL)	MOL002218	scopolin	Methylcrotonoyl-CoA carboxylase beta chain, mitochondrial	Q9HCC0	MCCC2	0.014
Cortex Lycii(CL)	MOL002218	scopolin	Sodium-dependent multivitamin transporter	Q9Y289	SLC5A6	0.014
Cortex Lycii(CL)	MOL002218	scopolin	Alpha-1D adrenergic receptor	P25100	ADRA1D	0.043
Cortex Lycii(CL)	MOL002218	scopolin	Alpha-1A adrenergic receptor	P35348	ADRA1A	0.043
Cortex Lycii(CL)	MOL002218	scopolin	Alpha-1B adrenergic receptor	P35368	ADRA1B	0.043
Cortex Lycii(CL)	MOL002218	scopolin	Potassium voltage-gated channel subfamily H member 2	Q12809	KCNH2	0.043
Cortex Lycii(CL)	MOL002218	scopolin	Potassium voltage-gated channel subfamily H member 6	Q9H252	KCNH6	0.043
Cortex Lycii(CL)	MOL002218	scopolin	Alpha-2A adrenergic receptor	P08913	ADRA2A	0.05
Cortex Lycii(CL)	MOL002218	scopolin	D(2) dopamine receptor	P14416	DRD2	0.05
Cortex Lycii(CL)	MOL002218	scopolin	Alpha-2B adrenergic receptor	P18089	ADRA2B	0.05
Cortex Lycii(CL)	MOL002218	scopolin	Alpha-2C adrenergic receptor	P18825	ADRA2C	0.05
Cortex Lycii(CL)	MOL002218	scopolin	5-hydroxytryptamine 1D receptor	P28221	HTR1D	0.05
Cortex Lycii(CL)	MOL002218	scopolin	5-hydroxytryptamine 1B receptor	P28222	HTR1B	0.05
Cortex Lycii(CL)	MOL002218	scopolin	5-hydroxytryptamine 2C receptor	P28335	HTR2C	0.05
Cortex Lycii(CL)	MOL002218	scopolin	D(3) dopamine receptor	P35462	DRD3	0.05
Cortex Lycii(CL)	MOL002218	scopolin	Neuronal acetylcholine receptor subunit alpha-2	Q15822	CHRNA2	0.055
Cortex Lycii(CL)	MOL002218	scopolin	Opioid receptor, sigma 1	Q5T1J1	SIGMAR1	0.055
Cortex Lycii(CL)	MOL002218	scopolin	Glutamate [NMDA] receptor subunit 3A	Q8TCU5	GRIN3A	0.055
Cortex Lycii(CL)	MOL002218	scopolin	Sigma 1-type opioid receptor	Q99720	SIGMAR1	0.055
Cortex Lycii(CL)	MOL002218	scopolin	Estradiol 17-beta-dehydrogenase 1	P14061	HSD17B1	0.056
Cortex Lycii(CL)	MOL002218	scopolin	Bile salt sulfotransferase	Q06520	SULT2A1	0.056
Cortex Lycii(CL)	MOL002218	scopolin	Mu-type opioid receptor	P35372	OPRM1	0.069
Cortex Lycii(CL)	MOL002218	scopolin	5-hydroxytryptamine 3 receptor	P46098	HTR3A	0.069
Cortex Lycii(CL)	MOL002218	scopolin	5-hydroxytryptamine 4 receptor	Q13639	HTR4	0.069
Cortex Lycii(CL)	MOL002218	scopolin	Amiloride-sensitive sodium channel subunit alpha	P37088	SCNN1A	0.083
Cortex Lycii(CL)	MOL002218	scopolin	Amiloride-sensitive sodium channel subunit beta	P51168	SCNN1B	0.083
Cortex Lycii(CL)	MOL002218	scopolin	Amiloride-sensitive sodium channel subunit gamma	P51170	SCNN1G	0.083
Cortex Lycii(CL)	MOL002218	scopolin	Vascular endothelial growth factor	A2A2V4	VEGFA	0.084
Cortex Lycii(CL)	MOL002218	scopolin	Tumor necrosis factor ligand superfamily member 11	O14788	TNFSF11	0.084
Cortex Lycii(CL)	MOL002218	scopolin	Cadherin-5	P33151	CDH5	0.084
Cortex Lycii(CL)	MOL002218	scopolin	Maltase-glucoamylase, intestinal	O43451	MGAM	0.085
Cortex Lycii(CL)	MOL002218	scopolin	Lysosomal alpha-glucosidase	P10253	GAA	0.085
Cortex Lycii(CL)	MOL002218	scopolin	Neutral alpha-glucosidase AB	Q14697	GANAB	0.085
Cortex Lycii(CL)	MOL002218	scopolin	Beta-3 adrenergic receptor	P13945	ADRB3	0.089
Cortex Lycii(CL)	MOL002218	scopolin	Neuronal acetylcholine receptor subunit alpha-3	P32297	CHRNA3	0.093
Cortex Lycii(CL)	MOL002218	scopolin	Neuronal acetylcholine receptor subunit alpha-7	P36544	CHRNA7	0.093
Cortex Lycii(CL)	MOL002218	scopolin	Neuronal acetylcholine receptor subunit alpha-4	P43681	CHRNA4	0.093
Cortex Lycii(CL)	MOL002218	scopolin	DNA polymerase	P04293	UL30	0.139
Cortex Lycii(CL)	MOL002218	scopolin	Tubulin beta-2C chain	P68371	TUBB4B	0.141
Cortex Lycii(CL)	MOL002218	scopolin	Tubulin alpha-3 chain	Q71U36	TUBA1A	0.141
Cortex Lycii(CL)	MOL002218	scopolin	Carbonic anhydrase 1	P00915	CA1	0.146
Cortex Lycii(CL)	MOL002218	scopolin	Carbonic anhydrase 4	P22748	CA4	0.146

Cortex Lycii(CL)	MOL002218	scopolin	Beta-2 adrenergic receptor	P07550	ADRB2	0.212
Cortex Lycii(CL)	MOL002218	scopolin	Beta-1 adrenergic receptor	P08588	ADRB1	0.212
Cortex Lycii(CL)	MOL002218	scopolin	5-hydroxytryptamine 1A receptor	P08908	HTR1A	0.262
Cortex Lycii(CL)	MOL002218	scopolin	ATP-sensitive inward rectifier potassium channel 1	P48048	KCNJ1	0.304
Cortex Lycii(CL)	MOL002218	scopolin	Tyrosine-protein kinase HCK	P08631	HCK	0.308
Cortex Lycii(CL)	MOL002218	scopolin	Egl nine homolog 1	Q9GZT9	EGLN1	0.312
Cortex Lycii(CL)	MOL002218	scopolin	Prostaglandin E2 receptor, EP2 subtype	P43116	PTGER2	0.314
Cortex Lycii(CL)	MOL002218	scopolin	Sodium channel protein type 5 subunit alpha	Q14524	SCN5A	0.314
Cortex Lycii(CL)	MOL002218	scopolin	Cyclin-A2	P20248	CCNA2	0.317
Cortex Lycii(CL)	MOL002218	scopolin	Dual specificity protein kinase CLK1	P49759	CLK1	0.319
Cortex Lycii(CL)	MOL002218	scopolin	Carbonic anhydrase 2	P00918	CA2	0.32
Cortex Lycii(CL)	MOL002218	scopolin	Estrogen receptor beta	Q92731	ESR2	0.322
Cortex Lycii(CL)	MOL002218	scopolin	Mitogen-activated protein kinase 14	Q16539	MAPK14	0.323
Cortex Lycii(CL)	MOL002218	scopolin	MAP kinase-activated protein kinase 2	P49137	MAPKAPK2	0.325
Cortex Lycii(CL)	MOL002218	scopolin	Prostaglandin G/H synthase 2	P35354	PTGS2	0.667
Cortex Lycii(CL)	MOL002218	scopolin	Estrogen receptor	P03372	ESR1	1
Cortex Lycii(CL)	MOL002219	Atropine	Gamma-aminobutyric acid receptor subunit rho-3	A8MPY1	GABRR3	0.013
Cortex Lycii(CL)	MOL002219	Atropine	Gamma-aminobutyric acid receptor subunit pi	O00591	GABRP	0.013
Cortex Lycii(CL)	MOL002219	Atropine	Gamma-aminobutyric acid receptor subunit delta	O14764	GABRD	0.013
Cortex Lycii(CL)	MOL002219	Atropine	Gamma-aminobutyric-acid receptor subunit alpha-1	P14867	GABRA1	0.013
Cortex Lycii(CL)	MOL002219	Atropine	Gamma-aminobutyric-acid receptor subunit beta-1	P18505	GABRB1	0.013
Cortex Lycii(CL)	MOL002219	Atropine	Gamma-aminobutyric acid receptor subunit gamma-2	P18507	GABRG2	0.013
Cortex Lycii(CL)	MOL002219	Atropine	Gamma-aminobutyric-acid receptor subunit rho-1	P24046	GABRR1	0.013
Cortex Lycii(CL)	MOL002219	Atropine	Gamma-aminobutyric acid receptor subunit rho-2	P28476	GABRR2	0.013
Cortex Lycii(CL)	MOL002219	Atropine	Gamma-aminobutyric-acid receptor subunit alpha-5	P31644	GABRA5	0.013
Cortex Lycii(CL)	MOL002219	Atropine	Gamma-aminobutyric-acid receptor subunit alpha-3	P34903	GABRA3	0.013
Cortex Lycii(CL)	MOL002219	Atropine	Gamma-aminobutyric-acid receptor subunit alpha-2	P47869	GABRA2	0.013
Cortex Lycii(CL)	MOL002219	Atropine	Gamma-aminobutyric-acid receptor subunit beta-2	P47870	GABRB2	0.013
Cortex Lycii(CL)	MOL002219	Atropine	Gamma-aminobutyric-acid receptor subunit alpha-4	P48169	GABRA4	0.013
Cortex Lycii(CL)	MOL002219	Atropine	Gamma-aminobutyric acid receptor subunit epsilon	P78334	GABRE	0.013
Cortex Lycii(CL)	MOL002219	Atropine	Gamma-aminobutyric-acid receptor subunit alpha-6	Q16445	GABRA6	0.013
Cortex Lycii(CL)	MOL002219	Atropine	Gamma-aminobutyric acid receptor subunit gamma-1	Q8N1C3	GABRG1	0.013
Cortex Lycii(CL)	MOL002219	Atropine	Gamma-aminobutyric acid receptor subunit gamma-3	Q99928	GABRG3	0.013
Cortex Lycii(CL)	MOL002219	Atropine	Gamma-aminobutyric acid receptor subunit theta	Q9UN88	GABRQ	0.013
Cortex Lycii(CL)	MOL002219	Atropine	Cytochrome b-c1 complex subunit 8	O14949	UQCRQ	0.02
Cortex Lycii(CL)	MOL002219	Atropine	Cytochrome b-c1 complex subunit 10	O14957	UQCR11	0.02
Cortex Lycii(CL)	MOL002219	Atropine	Cytochrome b	P00156	MT-CYB	0.02
Cortex Lycii(CL)	MOL002219	Atropine	Cytochrome b-c1 complex subunit 6, mitochondrial	P07919	UQCRH	0.02
Cortex Lycii(CL)	MOL002219	Atropine	Cytochrome c1, heme protein, mitochondrial	P08574	CYC1	0.02
Cortex Lycii(CL)	MOL002219	Atropine	Cytochrome b-c1 complex subunit 2, mitochondrial	P22695	UQCRC2	0.02
Cortex Lycii(CL)	MOL002219	Atropine	Ubiquinol-cytochrome-c reductase complex core protein 1, mitochondrial	P31930	UQCRC1	0.02
Cortex Lycii(CL)	MOL002219	Atropine	Cytochrome b-c1 complex subunit Rieske, mitochondrial	P47985	UQCRFS1	0.02
Cortex Lycii(CL)	MOL002219	Atropine	Cytochrome b-c1 complex subunit 9	Q9UDW1	UQCR10	0.02
Cortex Lycii(CL)	MOL002219	Atropine	D(4) dopamine receptor	P21917	DRD4	0.025
Cortex Lycii(CL)	MOL002219	Atropine	D(1B) dopamine receptor	P21918	DRD5	0.025
Cortex Lycii(CL)	MOL002219	Atropine	5-hydroxytryptamine 2B receptor	P41595	HTR2B	0.025
Cortex Lycii(CL)	MOL002219	Atropine	D1 dopamine receptor-interacting protein calcyon	Q9NYX4	CALY	0.025
Cortex Lycii(CL)	MOL002219	Atropine	Amine oxidase [flavin-containing] A	P21397	MAOA	0.033
Cortex Lycii(CL)	MOL002219	Atropine	Alanine aminotransferase 1	P24298	GPT	0.033
Cortex Lycii(CL)	MOL002219	Atropine	Amine oxidase [flavin-containing] B	P27338	MAOB	0.033
Cortex Lycii(CL)	MOL002219	Atropine	4-aminobutyrate aminotransferase, mitochondrial	P80404	ABAT	0.033
Cortex Lycii(CL)	MOL002219	Atropine	Membrane copper amine oxidase	Q16853	AOC3	0.033
Cortex Lycii(CL)	MOL002219	Atropine	Alanine aminotransferase 2	Q8TD30	GPT2	0.033

Cortex Lycii(CL)	MOL002219	Atropine	Muscarinic acetylcholine receptor M1	P11229	CHRM1	0.037
Cortex Lycii(CL)	MOL002219	Atropine	Sodium-dependent noradrenaline transporter	P23975	SLC6A2	0.037
Cortex Lycii(CL)	MOL002219	Atropine	Sodium-dependent serotonin transporter	P31645	SLC6A4	0.037
Cortex Lycii(CL)	MOL002219	Atropine	Alpha-1A adrenergic receptor	P35348	ADRA1A	0.037
Cortex Lycii(CL)	MOL002219	Atropine	Sodium-dependent dopamine transporter	Q01959	SLC6A3	0.037
Cortex Lycii(CL)	MOL002219	Atropine	High affinity cAMP-specific and IBMX-insensitive 3',5'-cyclic phosphodiesterase 8A	O60658	PDE8A	0.042
Cortex Lycii(CL)	MOL002219	Atropine	cAMP-specific 3',5'-cyclic phosphodiesterase 4A	P27815	PDE4A	0.042
Cortex Lycii(CL)	MOL002219	Atropine	6-phosphogluconate dehydrogenase, decarboxylating	P52209	PGD	0.042
Cortex Lycii(CL)	MOL002219	Atropine	cAMP-specific 3',5'-cyclic phosphodiesterase 4C	Q08493	PDE4C	0.042
Cortex Lycii(CL)	MOL002219	Atropine	cAMP-specific 3',5'-cyclic phosphodiesterase 4D	Q08499	PDE4D	0.042
Cortex Lycii(CL)	MOL002219	Atropine	High-affinity cAMP-specific 3',5'-cyclic phosphodiesterase 7A	Q13946	PDE7A	0.042
Cortex Lycii(CL)	MOL002219	Atropine	cAMP-specific 3',5'-cyclic phosphodiesterase 7B	Q9NP56	PDE7B	0.042
Cortex Lycii(CL)	MOL002219	Atropine	Protein S100-B	P04271	S100B	0.045
Cortex Lycii(CL)	MOL002219	Atropine	Protein S100-A1	P23297	S100A1	0.045
Cortex Lycii(CL)	MOL002219	Atropine	Interleukin-3	P08700	IL3	0.046
Cortex Lycii(CL)	MOL002219	Atropine	Elongation factor Tu GTP-binding domain-containing protein 1	Q7Z2Z2	EFL1	0.048
Cortex Lycii(CL)	MOL002219	Atropine	Glutamate [NMDA] receptor subunit zeta-1	Q05586	GRIN1	0.049
Cortex Lycii(CL)	MOL002219	Atropine	Glutamate [NMDA] receptor subunit epsilon-1	Q12879	GRIN2A	0.049
Cortex Lycii(CL)	MOL002219	Atropine	Glutamate [NMDA] receptor subunit epsilon-2	Q13224	GRIN2B	0.049
Cortex Lycii(CL)	MOL002219	Atropine	Glutamate [NMDA] receptor subunit epsilon-3	Q14957	GRIN2C	0.049
Cortex Lycii(CL)	MOL002219	Atropine	Tripartite motif-containing protein 13	O60858	TRIM13	0.05
Cortex Lycii(CL)	MOL002219	Atropine	Nociceptin receptor	P41146	OPRL1	0.05
Cortex Lycii(CL)	MOL002219	Atropine	Carbonic anhydrase 12	O43570	CA12	0.061
Cortex Lycii(CL)	MOL002219	Atropine	Carbonic anhydrase 4	P22748	CA4	0.061
Cortex Lycii(CL)	MOL002219	Atropine	Calcium-activated potassium channel subunit alpha 1	Q12791	KCNMA1	0.061
Cortex Lycii(CL)	MOL002219	Atropine	Solute carrier family 12 member 1	Q13621	SLC12A1	0.061
Cortex Lycii(CL)	MOL002219	Atropine	ATP-sensitive inward rectifier potassium channel 11	Q14654	KCNJ11	0.061
Cortex Lycii(CL)	MOL002219	Atropine	Carbonic anhydrase 9	Q16790	CA9	0.061
Cortex Lycii(CL)	MOL002219	Atropine	5-hydroxytryptamine 1A receptor	P08908	HTR1A	0.063
Cortex Lycii(CL)	MOL002219	Atropine	Alpha-2A adrenergic receptor	P08913	ADRA2A	0.063
Cortex Lycii(CL)	MOL002219	Atropine	Alpha-2B adrenergic receptor	P18089	ADRA2B	0.063
Cortex Lycii(CL)	MOL002219	Atropine	Alpha-2C adrenergic receptor	P18825	ADRA2C	0.063
Cortex Lycii(CL)	MOL002219	Atropine	D(1A) dopamine receptor	P21728	DRD1	0.063
Cortex Lycii(CL)	MOL002219	Atropine	5-hydroxytryptamine 1B receptor	P28222	HTR1B	0.063
Cortex Lycii(CL)	MOL002219	Atropine	5-hydroxytryptamine 2C receptor	P28335	HTR2C	0.063
Cortex Lycii(CL)	MOL002219	Atropine	D(3) dopamine receptor	P35462	DRD3	0.063
Cortex Lycii(CL)	MOL002219	Atropine	Ig kappa chain C region	P01834	IGKC	0.067
Cortex Lycii(CL)	MOL002219	Atropine	Ig gamma-1 chain C region	P01857	IGHG1	0.067
Cortex Lycii(CL)	MOL002219	Atropine	Ig gamma-2 chain C region	P01859	IGHG2	0.067
Cortex Lycii(CL)	MOL002219	Atropine	D-HSCDK2	O75100	CA11	0.071
Cortex Lycii(CL)	MOL002219	Atropine	Cell division control protein 2 homolog	P06493	CDK1	0.071
Cortex Lycii(CL)	MOL002219	Atropine	Cell division protein kinase 5	Q00535	CDK5	0.071
Cortex Lycii(CL)	MOL002219	Atropine	Dehydrogenase/reductase SDR family member 8	Q8NBQ5	HSD17B11	0.071
Cortex Lycii(CL)	MOL002219	Atropine	Potassium channel subfamily K member 1	O00180	KCNK1	0.072
Cortex Lycii(CL)	MOL002219	Atropine	Farnesyl pyrophosphate synthetase	P14324	FDPS	0.072
Cortex Lycii(CL)	MOL002219	Atropine	Sodium channel protein type 5 subunit alpha	Q14524	SCN5A	0.072
Cortex Lycii(CL)	MOL002219	Atropine	Potassium channel subfamily K member 6	Q9Y257	KCNK6	0.072
Cortex Lycii(CL)	MOL002219	Atropine	Neuronal acetylcholine receptor subunit alpha-3	P32297	CHRNA3	0.076
Cortex Lycii(CL)	MOL002219	Atropine	Neuronal acetylcholine receptor subunit alpha-7	P36544	CHRNA7	0.076
Cortex Lycii(CL)	MOL002219	Atropine	Neuronal acetylcholine receptor subunit alpha-4	P43681	CHRNA4	0.076
Cortex Lycii(CL)	MOL002219	Atropine	Tyrosinase	P14679	TYR	0.087
Cortex Lycii(CL)	MOL002219	Atropine	3-oxo-5-beta-steroid 4-dehydrogenase	P51857	AKR1D1	0.087
Cortex Lycii(CL)	MOL002219	Atropine	cAMP-specific 3',5'-cyclic phosphodiesterase 4B	Q07343	PDE4B	0.097

Cortex Lycii(CL)	MOL002219	Atropine	D(2) dopamine receptor	P14416	DRD2	0.1
Cortex Lycii(CL)	MOL002219	Atropine	5-hydroxytryptamine 1D receptor	P28221	HTR1D	0.1
Cortex Lycii(CL)	MOL002219	Atropine	Histamine H1 receptor	P35367	HRH1	0.1
Cortex Lycii(CL)	MOL002219	Atropine	Protein S100-A12	P80511	S100A12	0.104
Cortex Lycii(CL)	MOL002219	Atropine	Protein S100-A13	Q99584	S100A13	0.104
Cortex Lycii(CL)	MOL002219	Atropine	Mu-type opioid receptor	P35372	OPRM1	0.112
Cortex Lycii(CL)	MOL002219	Atropine	Delta-type opioid receptor	P41143	OPRD1	0.112
Cortex Lycii(CL)	MOL002219	Atropine	Alcohol dehydrogenase [NADP+]	P14550	AKR1A1	0.113
Cortex Lycii(CL)	MOL002219	Atropine	Aldose reductase	P15121	AKR1B1	0.113
Cortex Lycii(CL)	MOL002219	Atropine	Prostacyclin receptor	P43119	PTGIR	0.114
Cortex Lycii(CL)	MOL002219	Atropine	Tubulin beta-2C chain	P68371	TUBB4B	0.114
Cortex Lycii(CL)	MOL002219	Atropine	Peroxisome proliferator-activated receptor delta	Q03181	PPARD	0.114
Cortex Lycii(CL)	MOL002219	Atropine	Tubulin alpha-3 chain	Q71U36	TUBA1A	0.114
Cortex Lycii(CL)	MOL002219	Atropine	Carboxypeptidase A1	P15085	CPA1	0.116
Cortex Lycii(CL)	MOL002219	Atropine	3 beta-hydroxysteroid dehydrogenase/Delta 5-->4-isomerase type II	P26439	HSD3B2	0.118
Cortex Lycii(CL)	MOL002219	Atropine	Gonadotropin-releasing hormone receptor	P30968	GNRHR	0.131
Cortex Lycii(CL)	MOL002219	Atropine	Gonadotropin-releasing hormone II receptor	Q96P88	GNRHR2	0.131
Cortex Lycii(CL)	MOL002219	Atropine	Carbonic anhydrase 2	P00918	CA2	0.136
Cortex Lycii(CL)	MOL002219	Atropine	Sodium/potassium-transporting ATPase alpha-1 chain	P05023	ATP1A1	0.136
Cortex Lycii(CL)	MOL002219	Atropine	Platelet glycoprotein IX	P14770	GP9	0.137
Cortex Lycii(CL)	MOL002219	Atropine	Bile salt sulfotransferase	Q06520	SULT2A1	0.156
Cortex Lycii(CL)	MOL002219	Atropine	Nitric-oxide synthase, brain	P29475	NOS1	0.158
Cortex Lycii(CL)	MOL002219	Atropine	RAC-beta serine/threonine-protein kinase	P31751	AKT2	0.173
Cortex Lycii(CL)	MOL002219	Atropine	cAMP-dependent protein kinase inhibitor alpha	P61925	PKIA	0.173
Cortex Lycii(CL)	MOL002219	Atropine	Kappa-type opioid receptor	P41145	OPRK1	0.174
Cortex Lycii(CL)	MOL002219	Atropine	3-oxo-5-alpha-steroid 4-dehydrogenase 2	P31213	SRD5A2	0.204
Cortex Lycii(CL)	MOL002219	Atropine	3 beta-hydroxysteroid dehydrogenase/Delta 5-->4-isomerase type I	P14060	HSD3B1	0.228
Cortex Lycii(CL)	MOL002219	Atropine	Cell division protein kinase 2	P24941	CDK2	0.231
Cortex Lycii(CL)	MOL002219	Atropine	Lysozyme C	P61626	LYZ	0.232
Cortex Lycii(CL)	MOL002219	Atropine	Glyceraldehyde-3-phosphate dehydrogenase	P04406	GAPDH	0.233
Cortex Lycii(CL)	MOL002219	Atropine	Endothelin-1 receptor	P25101	EDNRA	0.234
Cortex Lycii(CL)	MOL002219	Atropine	Cyclin-A2	P20248	CCNA2	0.237
Cortex Lycii(CL)	MOL002219	Atropine	3-oxo-5-alpha-steroid 4-dehydrogenase 1	P18405	SRD5A1	0.241
Cortex Lycii(CL)	MOL002219	Atropine	Tyrosine-protein phosphatase non-receptor type 1	P18031	PTPN1	0.245
Cortex Lycii(CL)	MOL002219	Atropine	Phosphatidylinositol-4,5-bisphosphate 3-kinase catalytic subunit gamma isoform	P48736	PIK3CG	0.246
Cortex Lycii(CL)	MOL002219	Atropine	Prothrombin	P00734	F2	0.249
Cortex Lycii(CL)	MOL002219	Atropine	NADPH oxidase organizer 1	Q8NFA2	NOXO1	0.25
Cortex Lycii(CL)	MOL002219	Atropine	Thymidylate synthase	P04818	TYMS	0.258
Cortex Lycii(CL)	MOL002219	Atropine	Aldo-keto reductase family 1 member C1	Q04828	AKR1C1	0.258
Cortex Lycii(CL)	MOL002219	Atropine	Neutrophil gelatinase-associated lipocalin	P80188	LCN2	0.263
Cortex Lycii(CL)	MOL002219	Atropine	Estrogen receptor beta	Q92731	ESR2	0.278
Cortex Lycii(CL)	MOL002219	Atropine	Trypsin-2	P07478	PRSS2	0.279
Cortex Lycii(CL)	MOL002219	Atropine	Carbonic anhydrase 1	P00915	CA1	0.284
Cortex Lycii(CL)	MOL002219	Atropine	Mineralocorticoid receptor	P08235	NR3C2	0.288
Cortex Lycii(CL)	MOL002219	Atropine	Hemoglobin subunit alpha	P69905	HBA1	0.289
Cortex Lycii(CL)	MOL002219	Atropine	5'-AMP-activated protein kinase catalytic subunit alpha-1	Q13131	PRKAA1	0.291
Cortex Lycii(CL)	MOL002219	Atropine	Nitric-oxide synthase, endothelial	P29474	NOS3	0.329
Cortex Lycii(CL)	MOL002219	Atropine	Estradiol 17-beta-dehydrogenase 1	P14061	HSD17B1	0.377
Cortex Lycii(CL)	MOL002219	Atropine	Androgen receptor	P10275	AR	0.423
Cortex Lycii(CL)	MOL002219	Atropine	Prostaglandin G/H synthase 2	P35354	PTGS2	0.516
Cortex Lycii(CL)	MOL002219	Atropine	cAMP-dependent protein kinase catalytic subunit alpha	P17612	PRKACA	0.918
Cortex Lycii(CL)	MOL002219	Atropine	Estrogen receptor	P03372	ESR1	0.966
Cortex Lycii(CL)	MOL002219	Atropine	Progesterone receptor	P06401	PGR	1

Cortex Lycii(CL)	MOL002222	sugiol	High affinity nerve growth factor receptor	P04629	NTRK1	0.01
Cortex Lycii(CL)	MOL002222	sugiol	Potassium voltage-gated channel subfamily A member 1	Q09470	KCNA1	0.01
Cortex Lycii(CL)	MOL002222	sugiol	BDNF/NT-3 growth factors receptor	Q16620	NTRK2	0.01
Cortex Lycii(CL)	MOL002222	sugiol	Potassium voltage-gated channel subfamily D member 2	Q9NZV8	KCND2	0.01
Cortex Lycii(CL)	MOL002222	sugiol	Potassium voltage-gated channel subfamily D member 3	Q9UK17	KCND3	0.01
Cortex Lycii(CL)	MOL002222	sugiol	Glutamate [NMDA] receptor subunit epsilon-4	O15399	GRIN2D	0.011
Cortex Lycii(CL)	MOL002222	sugiol	Glutamate [NMDA] receptor subunit 3B	O60391	GRIN3B	0.011
Cortex Lycii(CL)	MOL002222	sugiol	Epidermal growth factor receptor	P00533	EGFR	0.011
Cortex Lycii(CL)	MOL002222	sugiol	Protein S100-B	P04271	S100B	0.011
Cortex Lycii(CL)	MOL002222	sugiol	Interleukin-3	P08700	IL3	0.011
Cortex Lycii(CL)	MOL002222	sugiol	Cell division protein kinase 4	P11802	CDK4	0.011
Cortex Lycii(CL)	MOL002222	sugiol	Protein S100-A1	P23297	S100A1	0.011
Cortex Lycii(CL)	MOL002222	sugiol	Cell division protein kinase 7	P50613	CDK7	0.011
Cortex Lycii(CL)	MOL002222	sugiol	Cell division protein kinase 9	P50750	CDK9	0.011
Cortex Lycii(CL)	MOL002222	sugiol	Cell division protein kinase 6	Q00534	CDK6	0.011
Cortex Lycii(CL)	MOL002222	sugiol	5-hydroxytryptamine 4 receptor	Q13639	HTR4	0.011
Cortex Lycii(CL)	MOL002222	sugiol	Sodium channel protein type 10 subunit alpha	Q9Y5Y9	SCN10A	0.011
Cortex Lycii(CL)	MOL002222	sugiol	Coagulation factor IX	P00740	F9	0.012
Cortex Lycii(CL)	MOL002222	sugiol	Coagulation factor X	P00742	F10	0.012
Cortex Lycii(CL)	MOL002222	sugiol	Osteocalcin	P02818	BGLAP	0.012
Cortex Lycii(CL)	MOL002222	sugiol	Vitamin K-dependent protein C	P04070	PROC	0.012
Cortex Lycii(CL)	MOL002222	sugiol	Coagulation factor VII	P08709	F7	0.012
Cortex Lycii(CL)	MOL002222	sugiol	NAD(P)H dehydrogenase [quinone] 1	P15559	NQO1	0.012
Cortex Lycii(CL)	MOL002222	sugiol	fMet-Leu-Phe receptor	P21462	FPR1	0.012
Cortex Lycii(CL)	MOL002222	sugiol	Vitamin K-dependent protein Z	P22891	PROZ	0.012
Cortex Lycii(CL)	MOL002222	sugiol	Vitamin K-dependent gamma-carboxylase	P38435	GGCX	0.012
Cortex Lycii(CL)	MOL002222	sugiol	Glutamate [NMDA] receptor subunit epsilon-1	Q12879	GRIN2A	0.012
Cortex Lycii(CL)	MOL002222	sugiol	Glutamate [NMDA] receptor subunit epsilon-2	Q13224	GRIN2B	0.012
Cortex Lycii(CL)	MOL002222	sugiol	Prostaglandin D2 receptor	Q13258	PTGDR	0.012
Cortex Lycii(CL)	MOL002222	sugiol	Glutamate [NMDA] receptor subunit epsilon-3	Q14957	GRIN2C	0.012
Cortex Lycii(CL)	MOL002222	sugiol	Neuronal acetylcholine receptor subunit alpha-2	Q15822	CHRNA2	0.012
Cortex Lycii(CL)	MOL002222	sugiol	Opioid receptor, sigma 1	Q5T1J1	SIGMAR1	0.012
Cortex Lycii(CL)	MOL002222	sugiol	Vitamin K epoxide reductase complex subunit 1-like protein 1	Q8N0U8	VKORC1L1	0.012
Cortex Lycii(CL)	MOL002222	sugiol	Sigma 1-type opioid receptor	Q99720	SIGMAR1	0.012
Cortex Lycii(CL)	MOL002222	sugiol	Vitamin K epoxide reductase complex subunit 1	Q9BQB6	VKORC1	0.012
Cortex Lycii(CL)	MOL002222	sugiol	Cysteinyl leukotriene receptor 2	Q9NS75	CYSLTR2	0.012
Cortex Lycii(CL)	MOL002222	sugiol	Cysteinyl leukotriene receptor 1	Q9Y271	CYSLTR1	0.012
Cortex Lycii(CL)	MOL002222	sugiol	Pepsin A	P00790	REN	0.013
Cortex Lycii(CL)	MOL002222	sugiol	Elongation factor 2	P13639	EEF2	0.013
Cortex Lycii(CL)	MOL002222	sugiol	Dipeptidyl peptidase 4	P27487	DPP4	0.013
Cortex Lycii(CL)	MOL002222	sugiol	Nociceptin receptor	P41146	OPRL1	0.013
Cortex Lycii(CL)	MOL002222	sugiol	Cyclin-dependent kinase 5 activator 1	Q15078	CDK5R1	0.013
Cortex Lycii(CL)	MOL002222	sugiol	Poly [ADP-ribose] polymerase 3	Q9Y6F1	PARP3	0.013
Cortex Lycii(CL)	MOL002222	sugiol	3-phosphoinositide-dependent protein kinase 1	O15530	PDPK1	0.014
Cortex Lycii(CL)	MOL002222	sugiol	Tyrosine-protein kinase CSK	P41240	CSK	0.014
Cortex Lycii(CL)	MOL002222	sugiol	Tyrosine-protein kinase ZAP-70	P43403	ZAP70	0.014
Cortex Lycii(CL)	MOL002222	sugiol	Tyrosine-protein kinase SYK	P43405	SYK	0.014
Cortex Lycii(CL)	MOL002222	sugiol	MAP kinase-activated protein kinase 2	P49137	MAPKAPK2	0.014
Cortex Lycii(CL)	MOL002222	sugiol	Protein kinase C theta type	Q04759	PRKCQ	0.014
Cortex Lycii(CL)	MOL002222	sugiol	Tyrosine-protein kinase ITK/TSK	Q08881	ITK	0.014
Cortex Lycii(CL)	MOL002222	sugiol	Potassium channel subfamily K member 1	O00180	KCNK1	0.015
Cortex Lycii(CL)	MOL002222	sugiol	High affinity cAMP-specific and IBMX-insensitive 3',5'-cyclic phosphodiesterase 8A	O60658	PDE8A	0.015
Cortex Lycii(CL)	MOL002222	sugiol	Tyrosine-protein kinase Lyn	P07948	LYN	0.015

Cortex Lycii(CL)	MOL002222	sugiol	Glycogen phosphorylase, muscle form	P11217	PYGM	0.015
Cortex Lycii(CL)	MOL002222	sugiol	6-phosphogluconate dehydrogenase, decarboxylating	P52209	PGD	0.015
Cortex Lycii(CL)	MOL002222	sugiol	High-affinity cAMP-specific 3',5'-cyclic phosphodiesterase 7A	Q13946	PDE7A	0.015
Cortex Lycii(CL)	MOL002222	sugiol	Gamma-aminobutyric-acid receptor subunit alpha-6	Q16445	GABRA6	0.015
Cortex Lycii(CL)	MOL002222	sugiol	cAMP-specific 3',5'-cyclic phosphodiesterase 7B	Q9NP56	PDE7B	0.015
Cortex Lycii(CL)	MOL002222	sugiol	Serine/threonine-protein kinase 17B	O94768	STK17B	0.016
Cortex Lycii(CL)	MOL002222	sugiol	ATP synthase subunit gamma, mitochondrial	P36542	ATP5F1C	0.016
Cortex Lycii(CL)	MOL002222	sugiol	UDP-glucuronosyltransferase 3A1	Q6NUS8	UGT3A1	0.016
Cortex Lycii(CL)	MOL002222	sugiol	Phosphatidylinositol 3-kinase regulatory subunit alpha	P27986	PIK3R1	0.017
Cortex Lycii(CL)	MOL002222	sugiol	Serine/threonine-protein kinase PLK1	P53350	PLK1	0.017
Cortex Lycii(CL)	MOL002222	sugiol	cGMP-inhibited 3',5'-cyclic phosphodiesterase A	Q14432	PDE3A	0.017
Cortex Lycii(CL)	MOL002222	sugiol	cAMP and cAMP-inhibited cGMP 3',5'-cyclic phosphodiesterase 10A	Q9Y233	PDE10A	0.017
Cortex Lycii(CL)	MOL002222	sugiol	Bile salt sulfotransferase	Q06520	SULT2A1	0.019
Cortex Lycii(CL)	MOL002222	sugiol	Solute carrier family 22 member 6	Q4U2R8	SLC22A6	0.019
Cortex Lycii(CL)	MOL002222	sugiol	Dehydrogenase/reductase SDR family member 8	Q8NBQ5	HSD17B11	0.019
Cortex Lycii(CL)	MOL002222	sugiol	Solute carrier family 22 member 8	Q8TCC7	SLC22A8	0.019
Cortex Lycii(CL)	MOL002222	sugiol	Solute carrier family 22 member 11	Q9NSA0	SLC22A11	0.019
Cortex Lycii(CL)	MOL002222	sugiol	Neuronal acetylcholine receptor subunit alpha-3	P32297	CHRNA3	0.02
Cortex Lycii(CL)	MOL002222	sugiol	Neuronal acetylcholine receptor subunit alpha-7	P36544	CHRNA7	0.02
Cortex Lycii(CL)	MOL002222	sugiol	Neuronal acetylcholine receptor subunit alpha-4	P43681	CHRNA4	0.02
Cortex Lycii(CL)	MOL002222	sugiol	2,4-dienoyl-CoA reductase, mitochondrial	Q16698	DECR1	0.02
Cortex Lycii(CL)	MOL002222	sugiol	Translocator protein	P30536	TSPO	0.021
Cortex Lycii(CL)	MOL002222	sugiol	Solute carrier family 12 member 3	P55017	SLC12A3	0.021
Cortex Lycii(CL)	MOL002222	sugiol	D1 dopamine receptor-interacting protein calcyon	Q9NYX4	CALY	0.021
Cortex Lycii(CL)	MOL002222	sugiol	Potassium voltage-gated channel subfamily KQT member 3	O43525	KCNQ3	0.022
Cortex Lycii(CL)	MOL002222	sugiol	Histamine H2 receptor	P25021	HRH2	0.022
Cortex Lycii(CL)	MOL002222	sugiol	Amiloride-sensitive cation channel 2, neuronal	P78348	ASIC1	0.022
Cortex Lycii(CL)	MOL002222	sugiol	Tyrosine-protein kinase JAK2	O60674	JAK2	0.023
Cortex Lycii(CL)	MOL002222	sugiol	Tyrosine-protein kinase JAK1	P23458	JAK1	0.023
Cortex Lycii(CL)	MOL002222	sugiol	Tyrosine-protein kinase JAK3	P52333	JAK3	0.023
Cortex Lycii(CL)	MOL002222	sugiol	Protein S100-A12	P80511	S100A12	0.025
Cortex Lycii(CL)	MOL002222	sugiol	Protein S100-A13	Q99584	S100A13	0.025
Cortex Lycii(CL)	MOL002222	sugiol	Lipoic acid synthetase, mitochondrial	O43766	LIAS	0.026
Cortex Lycii(CL)	MOL002222	sugiol	DNA-(apurinic or apyrimidinic site) lyase	P27695	APEX1	0.026
Cortex Lycii(CL)	MOL002222	sugiol	Lipoyltransferase 1, mitochondrial	Q9Y234	LIPT1	0.026
Cortex Lycii(CL)	MOL002222	sugiol	Gamma-aminobutyric-acid receptor subunit alpha-4	P48169	GABRA4	0.027
Cortex Lycii(CL)	MOL002222	sugiol	Guanine nucleotide-binding protein G(s) subunit alpha isoforms short	P63092	GNAS	0.027
Cortex Lycii(CL)	MOL002222	sugiol	Casein kinase I isoform gamma-2	P78368	CSNK1G2	0.027
Cortex Lycii(CL)	MOL002222	sugiol	Glutamate [NMDA] receptor subunit zeta-1	Q05586	GRIN1	0.027
Cortex Lycii(CL)	MOL002222	sugiol	Adenylate cyclase type 2	Q08462	ADCY2	0.027
Cortex Lycii(CL)	MOL002222	sugiol	Calcium/calmodulin-dependent protein kinase type II subunit delta	Q13557	CAMK2D	0.027
Cortex Lycii(CL)	MOL002222	sugiol	Serine/threonine-protein kinase haspin	Q8TF76	HASPIN	0.027
Cortex Lycii(CL)	MOL002222	sugiol	STE20-like serine/threonine-protein kinase	Q9H2G2	SLK	0.027
Cortex Lycii(CL)	MOL002222	sugiol	Gamma-aminobutyric acid receptor subunit theta	Q9UN88	GABRQ	0.027
Cortex Lycii(CL)	MOL002222	sugiol	Inhibitor of nuclear factor kappa-B kinase subunit alpha	O15111	CHUK	0.028
Cortex Lycii(CL)	MOL002222	sugiol	Mitogen-activated protein kinase 3	P27361	MAPK3	0.028
Cortex Lycii(CL)	MOL002222	sugiol	Nitric-oxide synthase, endothelial	P29474	NOS3	0.028
Cortex Lycii(CL)	MOL002222	sugiol	Nitric-oxide synthase, brain	P29475	NOS1	0.028
Cortex Lycii(CL)	MOL002222	sugiol	Aldo-keto reductase family 1 member C3	P42330	AKR1C3	0.028
Cortex Lycii(CL)	MOL002222	sugiol	Tubulin beta-2C chain	P68371	TUBB4B	0.028
Cortex Lycii(CL)	MOL002222	sugiol	Peroxisome proliferator-activated receptor delta	Q03181	PPARD	0.028
Cortex Lycii(CL)	MOL002222	sugiol	Tubulin alpha-3 chain	Q71U36	TUBA1A	0.028

Cortex Lycii(CL)	MOL002222	sugiol	Glycolipid transfer protein	Q9NZD2	GLTP	0.028
Cortex Lycii(CL)	MOL002222	sugiol	Tryptophanyl-tRNA synthetase, mitochondrial	Q9UGM6	WARS2	0.028
Cortex Lycii(CL)	MOL002222	sugiol	Succinate dehydrogenase [ubiquinone] cytochrome b small subunit, mitochondrial	O14521	SDHD	0.029
Cortex Lycii(CL)	MOL002222	sugiol	Tripartite motif-containing protein 13	O60858	TRIM13	0.029
Cortex Lycii(CL)	MOL002222	sugiol	Alcohol dehydrogenase [NADP+]	P14550	AKR1A1	0.029
Cortex Lycii(CL)	MOL002222	sugiol	Methionine aminopeptidase 1	P53582	METAP1	0.029
Cortex Lycii(CL)	MOL002222	sugiol	Serine/threonine-protein phosphatase 2A catalytic subunit alpha isoform	P67775	PPP2CA	0.029
Cortex Lycii(CL)	MOL002222	sugiol	Serine/threonine-protein phosphatase 2A 56 kDa regulatory subunit gamma isoform	Q13362	PPP2R5C	0.029
Cortex Lycii(CL)	MOL002222	sugiol	Glycogen synthase kinase-3 beta	P49841	GSK3B	0.03
Cortex Lycii(CL)	MOL002222	sugiol	Sterol O-acyltransferase 2	O75908	SOAT2	0.031
Cortex Lycii(CL)	MOL002222	sugiol	Annexin A1	P04083	ANXA1	0.031
Cortex Lycii(CL)	MOL002222	sugiol	Sterol O-acyltransferase 1	P35610	SOAT1	0.031
Cortex Lycii(CL)	MOL002222	sugiol	Nuclear receptor 0B1	P51843	NR0B1	0.031
Cortex Lycii(CL)	MOL002222	sugiol	Tubulin alpha-1 chain	P68366	TUBA4A	0.031
Cortex Lycii(CL)	MOL002222	sugiol	Carbonic anhydrase 12	O43570	CA12	0.032
Cortex Lycii(CL)	MOL002222	sugiol	Ig gamma-2 chain C region	P01859	IGHG2	0.032
Cortex Lycii(CL)	MOL002222	sugiol	Proto-oncogene tyrosine-protein kinase LCK	P06239	LCK	0.032
Cortex Lycii(CL)	MOL002222	sugiol	Carbonic anhydrase 4	P22748	CA4	0.032
Cortex Lycii(CL)	MOL002222	sugiol	Peptidyl-prolyl cis-trans isomerase, mitochondrial	P30405	PPIF	0.032
Cortex Lycii(CL)	MOL002222	sugiol	Carbonic anhydrase 9	Q16790	CA9	0.032
Cortex Lycii(CL)	MOL002222	sugiol	Prothrombin	P00734	F2	0.033
Cortex Lycii(CL)	MOL002222	sugiol	Sodium/potassium-transporting ATPase alpha-1 chain	P05023	ATP1A1	0.033
Cortex Lycii(CL)	MOL002222	sugiol	Proto-oncogene serine/threonine-protein kinase Pim-1	P11309	PIM1	0.033
Cortex Lycii(CL)	MOL002222	sugiol	Mitogen-activated protein kinase 8	P45983	MAPK8	0.033
Cortex Lycii(CL)	MOL002222	sugiol	Mitogen-activated protein kinase 10	P53779	MAPK10	0.033
Cortex Lycii(CL)	MOL002222	sugiol	Platelet glycoprotein IX	P14770	GP9	0.034
Cortex Lycii(CL)	MOL002222	sugiol	5-hydroxytryptamine 1D receptor	P28221	HTR1D	0.034
Cortex Lycii(CL)	MOL002222	sugiol	5-hydroxytryptamine 1B receptor	P28222	HTR1B	0.034
Cortex Lycii(CL)	MOL002222	sugiol	Retinoic acid receptor RXR-beta	P28702	RXRB	0.034
Cortex Lycii(CL)	MOL002222	sugiol	Cytosolic phospholipase A2	P47712	PLA2G4A	0.034
Cortex Lycii(CL)	MOL002222	sugiol	cAMP-specific 3',5'-cyclic phosphodiesterase 4C	Q08493	PDE4C	0.034
Cortex Lycii(CL)	MOL002222	sugiol	cAMP-specific 3',5'-cyclic phosphodiesterase 4D	Q08499	PDE4D	0.034
Cortex Lycii(CL)	MOL002222	sugiol	Potassium voltage-gated channel subfamily H member 2	Q12809	KCNH2	0.034
Cortex Lycii(CL)	MOL002222	sugiol	ATP synthase subunit beta, mitochondrial	P06576	ATP5F1B	0.035
Cortex Lycii(CL)	MOL002222	sugiol	Trypsin-1	P07477	PRSS1	0.035
Cortex Lycii(CL)	MOL002222	sugiol	Tyrosine-protein kinase HCK	P08631	HCK	0.035
Cortex Lycii(CL)	MOL002222	sugiol	ATP synthase subunit alpha, mitochondrial	P25705	ATP5F1A	0.035
Cortex Lycii(CL)	MOL002222	sugiol	cAMP-dependent protein kinase inhibitor alpha	P61925	PKIA	0.035
Cortex Lycii(CL)	MOL002222	sugiol	Potassium voltage-gated channel subfamily KQT member 2	O43526	KCNQ2	0.036
Cortex Lycii(CL)	MOL002222	sugiol	3-oxo-5-alpha-steroid 4-dehydrogenase 2	P31213	SRD5A2	0.036
Cortex Lycii(CL)	MOL002222	sugiol	Nuclear receptor subfamily 1 group I member 3	Q14994	NR1I3	0.036
Cortex Lycii(CL)	MOL002222	sugiol	3 beta-hydroxysteroid dehydrogenase/Delta 5-->4-isomerase type I	P14060	HSD3B1	0.037
Cortex Lycii(CL)	MOL002222	sugiol	Ribosyldihydronicotinamide dehydrogenase [quinone]	P16083	NQO2	0.037
Cortex Lycii(CL)	MOL002222	sugiol	Gonadotropin-releasing hormone receptor	P30968	GNRHR	0.037
Cortex Lycii(CL)	MOL002222	sugiol	Gamma-aminobutyric-acid receptor subunit beta-2	P47870	GABRB2	0.037
Cortex Lycii(CL)	MOL002222	sugiol	Sodium-dependent dopamine transporter	Q01959	SLC6A3	0.037
Cortex Lycii(CL)	MOL002222	sugiol	Protein tyrosine kinase 2 beta	Q14289	PTK2B	0.037
Cortex Lycii(CL)	MOL002222	sugiol	Gonadotropin-releasing hormone II receptor	Q96P88	GNRHR2	0.037
Cortex Lycii(CL)	MOL002222	sugiol	Prolactin receptor	P16471	PRLR	0.038
Cortex Lycii(CL)	MOL002222	sugiol	Microtubule-associated protein 2	P11137	MAP2	0.039
Cortex Lycii(CL)	MOL002222	sugiol	Gamma-aminobutyric acid receptor subunit gamma-2	P18507	GABRG2	0.039
Cortex Lycii(CL)	MOL002222	sugiol	Estrogen-related receptor gamma	P62508	ESRRG	0.039

Cortex Lycii(CL)	MOL002222	sugiol	Microtubule-associated protein 1A	P78559	MAP1A	0.039
Cortex Lycii(CL)	MOL002222	sugiol	Gamma-aminobutyric acid receptor subunit gamma-1	Q8N1C3	GABRG1	0.039
Cortex Lycii(CL)	MOL002222	sugiol	Glutamate [NMDA] receptor subunit 3A	Q8TCU5	GRIN3A	0.041
Cortex Lycii(CL)	MOL002222	sugiol	D-HSCDK2	O75100	CA11	0.043
Cortex Lycii(CL)	MOL002222	sugiol	Cell division control protein 2 homolog	P06493	CDK1	0.043
Cortex Lycii(CL)	MOL002222	sugiol	Gamma-aminobutyric-acid receptor subunit beta-3	P28472	GABRB3	0.043
Cortex Lycii(CL)	MOL002222	sugiol	Sodium channel protein type 4 subunit alpha	P35499	SCN4A	0.043
Cortex Lycii(CL)	MOL002222	sugiol	Gamma-aminobutyric acid receptor subunit rho-3	A8MPY1	GABRR3	0.045
Cortex Lycii(CL)	MOL002222	sugiol	Gamma-aminobutyric acid receptor subunit pi	O00591	GABRP	0.045
Cortex Lycii(CL)	MOL002222	sugiol	Gamma-aminobutyric acid receptor subunit delta	O14764	GABRD	0.045
Cortex Lycii(CL)	MOL002222	sugiol	Gamma-aminobutyric-acid receptor subunit rho-1	P24046	GABRR1	0.045
Cortex Lycii(CL)	MOL002222	sugiol	Gamma-aminobutyric acid receptor subunit rho-2	P28476	GABRR2	0.045
Cortex Lycii(CL)	MOL002222	sugiol	Gamma-aminobutyric acid receptor subunit epsilon	P78334	GABRE	0.045
Cortex Lycii(CL)	MOL002222	sugiol	Gamma-aminobutyric acid receptor subunit gamma-3	Q99928	GABRG3	0.045
Cortex Lycii(CL)	MOL002222	sugiol	D(4) dopamine receptor	P21917	DRD4	0.047
Cortex Lycii(CL)	MOL002222	sugiol	Alpha-1B adrenergic receptor	P35368	ADRA1B	0.047
Cortex Lycii(CL)	MOL002222	sugiol	Sodium channel protein type 5 subunit alpha	Q14524	SCN5A	0.047
Cortex Lycii(CL)	MOL002222	sugiol	Casein kinase II subunit alpha	P68400	CSNK2A1	0.048
Cortex Lycii(CL)	MOL002222	sugiol	Death-associated protein kinase 3	O43293	DAPK3	0.049
Cortex Lycii(CL)	MOL002222	sugiol	5-hydroxytryptamine 3 receptor	P46098	HTR3A	0.051
Cortex Lycii(CL)	MOL002222	sugiol	Gamma-aminobutyric-acid receptor subunit beta-1	P18505	GABRB1	0.052
Cortex Lycii(CL)	MOL002222	sugiol	Gamma-aminobutyric-acid receptor subunit alpha-5	P31644	GABRA5	0.052
Cortex Lycii(CL)	MOL002222	sugiol	Gamma-aminobutyric-acid receptor subunit alpha-3	P34903	GABRA3	0.052
Cortex Lycii(CL)	MOL002222	sugiol	Arachidonate 5-lipoxygenase	P09917	ALOX5	0.053
Cortex Lycii(CL)	MOL002222	sugiol	cAMP-specific 3',5'-cyclic phosphodiesterase 4B	Q07343	PDE4B	0.053
Cortex Lycii(CL)	MOL002222	sugiol	Tyrosine-protein phosphatase non-receptor type 1	P18031	PTPN1	0.056
Cortex Lycii(CL)	MOL002222	sugiol	RAC-alpha serine/threonine-protein kinase	P31749	AKT1	0.056
Cortex Lycii(CL)	MOL002222	sugiol	Phosphatidylinositol-4,5-bisphosphate 3-kinase catalytic subunit gamma isoform	P48736	PIK3CG	0.056
Cortex Lycii(CL)	MOL002222	sugiol	Proto-oncogene tyrosine-protein kinase Src	P12931	SRC	0.057
Cortex Lycii(CL)	MOL002222	sugiol	Phospholipase A2, membrane associated	P14555	PLA2G2A	0.057
Cortex Lycii(CL)	MOL002222	sugiol	Solute carrier family 12 member 1	Q13621	SLC12A1	0.057
Cortex Lycii(CL)	MOL002222	sugiol	Tubulin beta-1 chain	Q9H4B7	TUBB1	0.057
Cortex Lycii(CL)	MOL002222	sugiol	Toll-like receptor 7	Q9NYK1	TLR7	0.057
Cortex Lycii(CL)	MOL002222	sugiol	DNA polymerase kappa	Q9UBT6	POLK	0.057
Cortex Lycii(CL)	MOL002222	sugiol	Fibroblast growth factor receptor 2	P21802	FGFR2	0.058
Cortex Lycii(CL)	MOL002222	sugiol	Potassium voltage-gated channel subfamily KQT member 1	P51787	KCNQ1	0.058
Cortex Lycii(CL)	MOL002222	sugiol	Cell division protein kinase 5	Q00535	CDK5	0.058
Cortex Lycii(CL)	MOL002222	sugiol	Mediator of RNA polymerase II transcription subunit 1	Q15648	MED1	0.058
Cortex Lycii(CL)	MOL002222	sugiol	Hydroxyacid oxidase 1	Q9UJM8	HAO1	0.058
Cortex Lycii(CL)	MOL002222	sugiol	Poly [ADP-ribose] polymerase 1	P09874	PARP1	0.059
Cortex Lycii(CL)	MOL002222	sugiol	Triosephosphate isomerase	P60174	TPI1	0.059
Cortex Lycii(CL)	MOL002222	sugiol	Retinoic acid receptor alpha	P10276	RARA	0.06
Cortex Lycii(CL)	MOL002222	sugiol	Aldose reductase	P15121	AKR1B1	0.06
Cortex Lycii(CL)	MOL002222	sugiol	Aldo-keto reductase family 1 member C1	Q04828	AKR1C1	0.06
Cortex Lycii(CL)	MOL002222	sugiol	Alpha-1D adrenergic receptor	P25100	ADRA1D	0.061
Cortex Lycii(CL)	MOL002222	sugiol	Endothelin-1 receptor	P25101	EDNRA	0.061
Cortex Lycii(CL)	MOL002222	sugiol	Dual specificity protein kinase CLK1	P49759	CLK1	0.061
Cortex Lycii(CL)	MOL002222	sugiol	S-methyl-5-thioadenosine phosphorylase	Q13126	MTAP	0.061
Cortex Lycii(CL)	MOL002222	sugiol	Group IIE secretory phospholipase A2	Q9NZK7	PLA2G2E	0.061
Cortex Lycii(CL)	MOL002222	sugiol	Ig kappa chain V-II region RPMI 6410	P06310	IGKV2-30	0.062
Cortex Lycii(CL)	MOL002222	sugiol	Melatonin receptor type 1A	P48039	MTNR1A	0.062
Cortex Lycii(CL)	MOL002222	sugiol	Thyroid hormone receptor alpha	P10827	THRA	0.063
Cortex Lycii(CL)	MOL002222	sugiol	Thyroid hormone receptor beta-1	P10828	THRB	0.063

Cortex Lycii(CL)	MOL002222	sugiol	Cannabinoid receptor 2	P34972	CNR2	0.064
Cortex Lycii(CL)	MOL002222	sugiol	Thyroid hormone receptor, alpha isoform 1 variant	Q59FW3	SIGMAR1	0.064
Cortex Lycii(CL)	MOL002222	sugiol	Thiamin pyrophosphokinase 1	Q9H3S4	TPK1	0.064
Cortex Lycii(CL)	MOL002222	sugiol	Inosine-5'-monophosphate dehydrogenase 1	P20839	IMPDH1	0.065
Cortex Lycii(CL)	MOL002222	sugiol	Thymidylate synthase	P04818	TYMS	0.066
Cortex Lycii(CL)	MOL002222	sugiol	Gamma-aminobutyric-acid receptor subunit alpha-1	P14867	GABRA1	0.066
Cortex Lycii(CL)	MOL002222	sugiol	Nuclear receptor coactivator 5	Q9HCD5	NCOA5	0.066
Cortex Lycii(CL)	MOL002222	sugiol	Dihydrofolate reductase	P00374	DHFR	0.067
Cortex Lycii(CL)	MOL002222	sugiol	Phospholipase A2	P04054	PLA2G1B	0.067
Cortex Lycii(CL)	MOL002222	sugiol	Liver carboxylesterase 1	P23141	CES1	0.067
Cortex Lycii(CL)	MOL002222	sugiol	C-jun-amino-terminal kinase-interacting protein 1	Q9UQF2	MAPK8IP1	0.069
Cortex Lycii(CL)	MOL002222	sugiol	Cannabinoid receptor 1	P21554	CNR1	0.07
Cortex Lycii(CL)	MOL002222	sugiol	Alpha-2C adrenergic receptor	P18825	ADRA2C	0.072
Cortex Lycii(CL)	MOL002222	sugiol	D(3) dopamine receptor	P35462	DRD3	0.072
Cortex Lycii(CL)	MOL002222	sugiol	5-hydroxytryptamine 2B receptor	P41595	HTR2B	0.072
Cortex Lycii(CL)	MOL002222	sugiol	Gamma-aminobutyric-acid receptor subunit alpha-2	P47869	GABRA2	0.072
Cortex Lycii(CL)	MOL002222	sugiol	Retinoic acid receptor RXR-alpha	P19793	RXRA	0.074
Cortex Lycii(CL)	MOL002222	sugiol	cAMP-specific 3',5'-cyclic phosphodiesterase 4A	P27815	PDE4A	0.074
Cortex Lycii(CL)	MOL002222	sugiol	Peroxisome proliferator-activated receptor gamma	P37231	PPARG	0.075
Cortex Lycii(CL)	MOL002222	sugiol	NADPH oxidase organizer 1	Q8NFA2	NOXO1	0.075
Cortex Lycii(CL)	MOL002222	sugiol	3-oxo-5-alpha-steroid 4-dehydrogenase 1	P18405	SRD5A1	0.077
Cortex Lycii(CL)	MOL002222	sugiol	Alpha-2B adrenergic receptor	P18089	ADRA2B	0.084
Cortex Lycii(CL)	MOL002222	sugiol	D(1B) dopamine receptor	P21918	DRD5	0.084
Cortex Lycii(CL)	MOL002222	sugiol	Muscarinic acetylcholine receptor M2	P08172	CHRM2	0.087
Cortex Lycii(CL)	MOL002222	sugiol	5-hydroxytryptamine 1A receptor	P08908	HTR1A	0.088
Cortex Lycii(CL)	MOL002222	sugiol	Cell division protein kinase 2	P24941	CDK2	0.097
Cortex Lycii(CL)	MOL002222	sugiol	Muscarinic acetylcholine receptor M5	P08912	CHRM5	0.099
Cortex Lycii(CL)	MOL002222	sugiol	Sodium-dependent serotonin transporter	P31645	SLC6A4	0.1
Cortex Lycii(CL)	MOL002222	sugiol	DNA topoisomerase 2-alpha	P11388	TOP2A	0.101
Cortex Lycii(CL)	MOL002222	sugiol	Ig kappa chain C region	P01834	IGKC	0.102
Cortex Lycii(CL)	MOL002222	sugiol	Ig gamma-1 chain C region	P01857	IGHG1	0.102
Cortex Lycii(CL)	MOL002222	sugiol	Carbonic anhydrase 1	P00915	CA1	0.105
Cortex Lycii(CL)	MOL002222	sugiol	Carbonic anhydrase 2	P00918	CA2	0.105
Cortex Lycii(CL)	MOL002222	sugiol	Glucocorticoid receptor	P04150	NR3C1	0.105
Cortex Lycii(CL)	MOL002222	sugiol	Delta-type opioid receptor	P41143	OPRD1	0.108
Cortex Lycii(CL)	MOL002222	sugiol	5-hydroxytryptamine 2C receptor	P28335	HTR2C	0.111
Cortex Lycii(CL)	MOL002222	sugiol	Muscarinic acetylcholine receptor M4	P08173	CHRM4	0.112
Cortex Lycii(CL)	MOL002222	sugiol	Muscarinic acetylcholine receptor M3	P20309	CHRM3	0.112
Cortex Lycii(CL)	MOL002222	sugiol	Nuclear receptor coactivator 2	Q15596	NCOA2	0.114
Cortex Lycii(CL)	MOL002222	sugiol	Estradiol 17-beta-dehydrogenase 1	P14061	HSD17B1	0.116
Cortex Lycii(CL)	MOL002222	sugiol	D(1A) dopamine receptor	P21728	DRD1	0.121
Cortex Lycii(CL)	MOL002222	sugiol	Cyclin-A2	P20248	CCNA2	0.122
Cortex Lycii(CL)	MOL002222	sugiol	Nitric oxide synthase, inducible	P35228	NOS2	0.123
Cortex Lycii(CL)	MOL002222	sugiol	Alpha-2A adrenergic receptor	P08913	ADRA2A	0.125
Cortex Lycii(CL)	MOL002222	sugiol	Cytochrome P450 11B1, mitochondrial	P15538	CYP11B1	0.127
Cortex Lycii(CL)	MOL002222	sugiol	Kappa-type opioid receptor	P41145	OPRK1	0.137
Cortex Lycii(CL)	MOL002222	sugiol	Sodium-dependent noradrenaline transporter	P23975	SLC6A2	0.141
Cortex Lycii(CL)	MOL002222	sugiol	Mu-type opioid receptor	P35372	OPRM1	0.141
Cortex Lycii(CL)	MOL002222	sugiol	D(2) dopamine receptor	P14416	DRD2	0.146
Cortex Lycii(CL)	MOL002222	sugiol	Alpha-1A adrenergic receptor	P35348	ADRA1A	0.147
Cortex Lycii(CL)	MOL002222	sugiol	Muscarinic acetylcholine receptor M1	P11229	CHRM1	0.15
Cortex Lycii(CL)	MOL002222	sugiol	Mineralocorticoid receptor	P08235	NR3C2	0.151
Cortex Lycii(CL)	MOL002222	sugiol	Histamine H1 receptor	P35367	HRH1	0.159
Cortex Lycii(CL)	MOL002222	sugiol	Androgen receptor	P10275	AR	0.16

Cortex Lycii(CL)	MOL002222	sugiol	5-hydroxytryptamine 2A receptor	P28223	HTR2A	0.163
Cortex Lycii(CL)	MOL002222	sugiol	cAMP-dependent protein kinase catalytic subunit alpha	P17612	PRKACA	0.231
Cortex Lycii(CL)	MOL002222	sugiol	Prostaglandin G/H synthase 1	P23219	PTGS1	0.232
Cortex Lycii(CL)	MOL002222	sugiol	Nuclear receptor coactivator 1	Q15788	NCOA1	0.242
Cortex Lycii(CL)	MOL002222	sugiol	Estrogen receptor beta	Q92731	ESR2	0.429
Cortex Lycii(CL)	MOL002222	sugiol	Progesterone receptor	P06401	PGR	0.471
Cortex Lycii(CL)	MOL002222	sugiol	Prostaglandin G/H synthase 2	P35354	PTGS2	0.635
Cortex Lycii(CL)	MOL002222	sugiol	Estrogen receptor	P03372	ESR1	1
Cortex Lycii(CL)	MOL002224	aurantiamide acetate	cAMP-dependent protein kinase catalytic subunit alpha	P17612	PRKACA	0.079
Cortex Lycii(CL)	MOL002224	aurantiamide acetate	cAMP-dependent protein kinase inhibitor alpha	P61925	PKIA	0.079
Cortex Lycii(CL)	MOL002228	Kulactone	Dehydrogenase/reductase SDR family member 8	Q8NBQ5	HSD17B11	0.012
Cortex Lycii(CL)	MOL002228	Kulactone	Ig kappa chain C region	P01834	IGKC	0.017
Cortex Lycii(CL)	MOL002228	Kulactone	Ig gamma-1 chain C region	P01857	IGHG1	0.017
Cortex Lycii(CL)	MOL002228	Kulactone	Ig gamma-2 chain C region	P01859	IGHG2	0.017
Cortex Lycii(CL)	MOL002228	Kulactone	Retinoic acid receptor RXR-alpha	P19793	RXRA	0.03
Cortex Lycii(CL)	MOL002228	Kulactone	Nuclear receptor subfamily 1 group I member 3	Q14994	NR1I3	0.03
Cortex Lycii(CL)	MOL002228	Kulactone	3 beta-hydroxysteroid dehydrogenase/Delta 5-->4-isomerase type II	P26439	HSD3B2	0.031
Cortex Lycii(CL)	MOL002228	Kulactone	Estrogen receptor beta	Q92731	ESR2	0.035
Cortex Lycii(CL)	MOL002228	Kulactone	Annexin A1	P04083	ANXA1	0.036
Cortex Lycii(CL)	MOL002228	Kulactone	Prolactin receptor	P16471	PRLR	0.036
Cortex Lycii(CL)	MOL002228	Kulactone	3-oxo-5-alpha-steroid 4-dehydrogenase 2	P31213	SRD5A2	0.036
Cortex Lycii(CL)	MOL002228	Kulactone	Nuclear receptor 0B1	P51843	NR0B1	0.036
Cortex Lycii(CL)	MOL002228	Kulactone	Gonadotropin-releasing hormone receptor	P30968	GNRHR	0.037
Cortex Lycii(CL)	MOL002228	Kulactone	Cytosolic phospholipase A2	P47712	PLA2G4A	0.037
Cortex Lycii(CL)	MOL002228	Kulactone	Gonadotropin-releasing hormone II receptor	Q96P88	GNRHR2	0.037
Cortex Lycii(CL)	MOL002228	Kulactone	Corticosteroid 11-beta-dehydrogenase isozyme 1	P28845	HSD11B1	0.038
Cortex Lycii(CL)	MOL002228	Kulactone	Muscarinic acetylcholine receptor M1	P11229	CHRM1	0.039
Cortex Lycii(CL)	MOL002228	Kulactone	Bile salt sulfotransferase	Q06520	SULT2A1	0.055
Cortex Lycii(CL)	MOL002228	Kulactone	Muscarinic acetylcholine receptor M2	P08172	CHRM2	0.075
Cortex Lycii(CL)	MOL002228	Kulactone	Neuronal acetylcholine receptor subunit alpha-2	Q15822	CHRNA2	0.076
Cortex Lycii(CL)	MOL002228	Kulactone	cAMP-dependent protein kinase catalytic subunit alpha	P17612	PRKACA	0.08
Cortex Lycii(CL)	MOL002228	Kulactone	Mediator of RNA polymerase II transcription subunit 1	Q15648	MED1	0.08
Cortex Lycii(CL)	MOL002228	Kulactone	NADPH oxidase organizer 1	Q8NFA2	NOXO1	0.08
Cortex Lycii(CL)	MOL002228	Kulactone	Inosine-5'-monophosphate dehydrogenase 1	P20839	IMPDH1	0.081
Cortex Lycii(CL)	MOL002228	Kulactone	3 beta-hydroxysteroid dehydrogenase/Delta 5-->4-isomerase type I	P14060	HSD3B1	0.085
Cortex Lycii(CL)	MOL002228	Kulactone	Nuclear receptor coactivator 5	Q9HCD5	NCOA5	0.086
Cortex Lycii(CL)	MOL002228	Kulactone	Thyroid hormone receptor, alpha isoform 1 variant	Q59FW3	SIGMAR1	0.091
Cortex Lycii(CL)	MOL002228	Kulactone	Nuclear receptor coactivator 1	Q15788	NCOA1	0.092
Cortex Lycii(CL)	MOL002228	Kulactone	3-oxo-5-alpha-steroid 4-dehydrogenase 1	P18405	SRD5A1	0.104
Cortex Lycii(CL)	MOL002228	Kulactone	Aldo-keto reductase family 1 member C1	Q04828	AKR1C1	0.115
Cortex Lycii(CL)	MOL002228	Kulactone	Estradiol 17-beta-dehydrogenase 1	P14061	HSD17B1	0.164
Cortex Lycii(CL)	MOL002228	Kulactone	Androgen receptor	P10275	AR	0.172
Cortex Lycii(CL)	MOL002228	Kulactone	Glucocorticoid receptor	P04150	NR3C1	0.309
Cortex Lycii(CL)	MOL002228	Kulactone	Mineralocorticoid receptor	P08235	NR3C2	0.314
Cortex Lycii(CL)	MOL002228	Kulactone	Estrogen receptor	P03372	ESR1	0.637
Cortex Lycii(CL)	MOL002228	Kulactone	Progesterone receptor	P06401	PGR	1
Radix Saposhnikoviae（RS）	MOL000011	(2R,3R)-3-(4-hydroxy-3-methoxy-phenyl)-5-methoxy-2-methylol-2,3-dihydropyrano[5,6-h][1,4]benzodioxin-9-one	Neuronal acetylcholine receptor subunit alpha-3	P32297	CHRNA3	0.012
Radix Saposhnikoviae（RS）	MOL000011	(2R,3R)-3-(4-hydroxy-3-methoxy-phenyl)-5-methoxy-2-methylol-2,3-dihydropyrano[5,6-h][1,4]benzodioxin-9-one	Neuronal acetylcholine receptor subunit alpha-7	P36544	CHRNA7	0.012
Radix Saposhnikoviae（RS）	MOL000011	(2R,3R)-3-(4-hydroxy-3-methoxy-phenyl)-5-methoxy-2-methylol-2,3-dihydropyrano[5,6-h][1,4]benzodioxin-9-one	Death-associated protein kinase 3	O43293	DAPK3	0.018

Radix Saposhnikoviae（RS）	MOL000011	(2R,3R)-3-(4-hydroxy-3-methoxy-phenyl)-5-methoxy-2-methylol-2,3-dihydropyrano[5,6-h][1,4]benzodioxin-9-one	Tyrosine-protein kinase JAK2	O60674	JAK2	0.018
Radix Saposhnikoviae（RS）	MOL000011	(2R,3R)-3-(4-hydroxy-3-methoxy-phenyl)-5-methoxy-2-methylol-2,3-dihydropyrano[5,6-h][1,4]benzodioxin-9-one	Tyrosine-protein kinase JAK1	P23458	JAK1	0.018
Radix Saposhnikoviae（RS）	MOL000011	(2R,3R)-3-(4-hydroxy-3-methoxy-phenyl)-5-methoxy-2-methylol-2,3-dihydropyrano[5,6-h][1,4]benzodioxin-9-one	Tyrosine-protein kinase JAK3	P52333	JAK3	0.018
Radix Saposhnikoviae（RS）	MOL000011	(2R,3R)-3-(4-hydroxy-3-methoxy-phenyl)-5-methoxy-2-methylol-2,3-dihydropyrano[5,6-h][1,4]benzodioxin-9-one	Serine/threonine-protein kinase 17B	O94768	STK17B	0.02
Radix Saposhnikoviae（RS）	MOL000011	(2R,3R)-3-(4-hydroxy-3-methoxy-phenyl)-5-methoxy-2-methylol-2,3-dihydropyrano[5,6-h][1,4]benzodioxin-9-one	ATP synthase subunit beta, mitochondrial	P06576	ATP5F1B	0.02
Radix Saposhnikoviae（RS）	MOL000011	(2R,3R)-3-(4-hydroxy-3-methoxy-phenyl)-5-methoxy-2-methylol-2,3-dihydropyrano[5,6-h][1,4]benzodioxin-9-one	ATP synthase subunit alpha, mitochondrial	P25705	ATP5F1A	0.02
Radix Saposhnikoviae（RS）	MOL000011	(2R,3R)-3-(4-hydroxy-3-methoxy-phenyl)-5-methoxy-2-methylol-2,3-dihydropyrano[5,6-h][1,4]benzodioxin-9-one	ATP synthase subunit gamma, mitochondrial	P36542	ATP5F1C	0.02
Radix Saposhnikoviae（RS）	MOL000011	(2R,3R)-3-(4-hydroxy-3-methoxy-phenyl)-5-methoxy-2-methylol-2,3-dihydropyrano[5,6-h][1,4]benzodioxin-9-one	Phosphatidylinositol-4,5-bisphosphate 3-kinase catalytic subunit gamma isoform	P48736	PIK3CG	0.02
Radix Saposhnikoviae（RS）	MOL000011	(2R,3R)-3-(4-hydroxy-3-methoxy-phenyl)-5-methoxy-2-methylol-2,3-dihydropyrano[5,6-h][1,4]benzodioxin-9-one	UDP-glucuronosyltransferase 3A1	Q6NUS8	UGT3A1	0.02
Radix Saposhnikoviae（RS）	MOL000011	(2R,3R)-3-(4-hydroxy-3-methoxy-phenyl)-5-methoxy-2-methylol-2,3-dihydropyrano[5,6-h][1,4]benzodioxin-9-one	Muscarinic acetylcholine receptor M5	P08912	CHRM5	0.024
Radix Saposhnikoviae（RS）	MOL000011	(2R,3R)-3-(4-hydroxy-3-methoxy-phenyl)-5-methoxy-2-methylol-2,3-dihydropyrano[5,6-h][1,4]benzodioxin-9-one	Muscarinic acetylcholine receptor M3	P20309	CHRM3	0.024
Radix Saposhnikoviae（RS）	MOL000011	(2R,3R)-3-(4-hydroxy-3-methoxy-phenyl)-5-methoxy-2-methylol-2,3-dihydropyrano[5,6-h][1,4]benzodioxin-9-one	Histamine H1 receptor	P35367	HRH1	0.024
Radix Saposhnikoviae（RS）	MOL000011	(2R,3R)-3-(4-hydroxy-3-methoxy-phenyl)-5-methoxy-2-methylol-2,3-dihydropyrano[5,6-h][1,4]benzodioxin-9-one	3-phosphoinositide-dependent protein kinase 1	O15530	PDPK1	0.028
Radix Saposhnikoviae（RS）	MOL000011	(2R,3R)-3-(4-hydroxy-3-methoxy-phenyl)-5-methoxy-2-methylol-2,3-dihydropyrano[5,6-h][1,4]benzodioxin-9-one	Proto-oncogene tyrosine-protein kinase LCK	P06239	LCK	0.028
Radix Saposhnikoviae（RS）	MOL000011	(2R,3R)-3-(4-hydroxy-3-methoxy-phenyl)-5-methoxy-2-methylol-2,3-dihydropyrano[5,6-h][1,4]benzodioxin-9-one	Retinoic acid receptor alpha	P10276	RARA	0.028
Radix Saposhnikoviae（RS）	MOL000011	(2R,3R)-3-(4-hydroxy-3-methoxy-phenyl)-5-methoxy-2-methylol-2,3-dihydropyrano[5,6-h][1,4]benzodioxin-9-one	Retinoic acid receptor gamma-1	P13631	RARG	0.028
Radix Saposhnikoviae（RS）	MOL000011	(2R,3R)-3-(4-hydroxy-3-methoxy-phenyl)-5-methoxy-2-methylol-2,3-dihydropyrano[5,6-h][1,4]benzodioxin-9-one	Retinoic acid receptor RXR-beta	P28702	RXRB	0.028
Radix Saposhnikoviae（RS）	MOL000011	(2R,3R)-3-(4-hydroxy-3-methoxy-phenyl)-5-methoxy-2-methylol-2,3-dihydropyrano[5,6-h][1,4]benzodioxin-9-one	Tyrosine-protein kinase CSK	P41240	CSK	0.028
Radix Saposhnikoviae（RS）	MOL000011	(2R,3R)-3-(4-hydroxy-3-methoxy-phenyl)-5-methoxy-2-methylol-2,3-dihydropyrano[5,6-h][1,4]benzodioxin-9-one	Tyrosine-protein kinase ZAP-70	P43403	ZAP70	0.028
Radix Saposhnikoviae（RS）	MOL000011	(2R,3R)-3-(4-hydroxy-3-methoxy-phenyl)-5-methoxy-2-methylol-2,3-dihydropyrano[5,6-h][1,4]benzodioxin-9-one	Tyrosine-protein kinase SYK	P43405	SYK	0.028
Radix Saposhnikoviae（RS）	MOL000011	(2R,3R)-3-(4-hydroxy-3-methoxy-phenyl)-5-methoxy-2-methylol-2,3-dihydropyrano[5,6-h][1,4]benzodioxin-9-one	Protein kinase C theta type	Q04759	PRKCQ	0.028
Radix Saposhnikoviae（RS）	MOL000011	(2R,3R)-3-(4-hydroxy-3-methoxy-phenyl)-5-methoxy-2-methylol-2,3-dihydropyrano[5,6-h][1,4]benzodioxin-9-one	Tyrosine-protein kinase ITK/TSK	Q08881	ITK	0.028
Radix Saposhnikoviae（RS）	MOL000011	(2R,3R)-3-(4-hydroxy-3-methoxy-phenyl)-5-methoxy-2-methylol-2,3-dihydropyrano[5,6-h][1,4]benzodioxin-9-one	Cyclin-dependent kinase 5 activator 1	Q15078	CDK5R1	0.03

Radix Saposhnikoviae（RS）	MOL000011	(2R,3R)-3-(4-hydroxy-3-methoxy-phenyl)-5-methoxy-2-methylol-2,3-dihydropyrano[5,6-h][1,4]benzodioxin-9-one	Potassium channel subfamily K member 6	Q9Y257	KCNK6	0.031
Radix Saposhnikoviae（RS）	MOL000011	(2R,3R)-3-(4-hydroxy-3-methoxy-phenyl)-5-methoxy-2-methylol-2,3-dihydropyrano[5,6-h][1,4]benzodioxin-9-one	Ribosyldihydronicotinamide dehydrogenase [quinone]	P16083	NQO2	0.034
Radix Saposhnikoviae（RS）	MOL000011	(2R,3R)-3-(4-hydroxy-3-methoxy-phenyl)-5-methoxy-2-methylol-2,3-dihydropyrano[5,6-h][1,4]benzodioxin-9-one	Prostaglandin G/H synthase 1	P23219	PTGS1	0.034
Radix Saposhnikoviae（RS）	MOL000011	(2R,3R)-3-(4-hydroxy-3-methoxy-phenyl)-5-methoxy-2-methylol-2,3-dihydropyrano[5,6-h][1,4]benzodioxin-9-one	Mu-type opioid receptor	P35372	OPRM1	0.034
Radix Saposhnikoviae（RS）	MOL000011	(2R,3R)-3-(4-hydroxy-3-methoxy-phenyl)-5-methoxy-2-methylol-2,3-dihydropyrano[5,6-h][1,4]benzodioxin-9-one	Amiloride-sensitive sodium channel subunit alpha	P37088	SCNN1A	0.034
Radix Saposhnikoviae（RS）	MOL000011	(2R,3R)-3-(4-hydroxy-3-methoxy-phenyl)-5-methoxy-2-methylol-2,3-dihydropyrano[5,6-h][1,4]benzodioxin-9-one	5-hydroxytryptamine 3 receptor	P46098	HTR3A	0.034
Radix Saposhnikoviae（RS）	MOL000011	(2R,3R)-3-(4-hydroxy-3-methoxy-phenyl)-5-methoxy-2-methylol-2,3-dihydropyrano[5,6-h][1,4]benzodioxin-9-one	Amiloride-sensitive sodium channel subunit beta	P51168	SCNN1B	0.034
Radix Saposhnikoviae（RS）	MOL000011	(2R,3R)-3-(4-hydroxy-3-methoxy-phenyl)-5-methoxy-2-methylol-2,3-dihydropyrano[5,6-h][1,4]benzodioxin-9-one	Amiloride-sensitive sodium channel subunit gamma	P51170	SCNN1G	0.034
Radix Saposhnikoviae（RS）	MOL000011	(2R,3R)-3-(4-hydroxy-3-methoxy-phenyl)-5-methoxy-2-methylol-2,3-dihydropyrano[5,6-h][1,4]benzodioxin-9-one	Casein kinase II subunit alpha	P68400	CSNK2A1	0.034
Radix Saposhnikoviae（RS）	MOL000011	(2R,3R)-3-(4-hydroxy-3-methoxy-phenyl)-5-methoxy-2-methylol-2,3-dihydropyrano[5,6-h][1,4]benzodioxin-9-one	5-hydroxytryptamine 4 receptor	Q13639	HTR4	0.034
Radix Saposhnikoviae（RS）	MOL000011	(2R,3R)-3-(4-hydroxy-3-methoxy-phenyl)-5-methoxy-2-methylol-2,3-dihydropyrano[5,6-h][1,4]benzodioxin-9-one	Alpha-2A adrenergic receptor	P08913	ADRA2A	0.036
Radix Saposhnikoviae（RS）	MOL000011	(2R,3R)-3-(4-hydroxy-3-methoxy-phenyl)-5-methoxy-2-methylol-2,3-dihydropyrano[5,6-h][1,4]benzodioxin-9-one	Alpha-1A adrenergic receptor	P35348	ADRA1A	0.036
Radix Saposhnikoviae（RS）	MOL000011	(2R,3R)-3-(4-hydroxy-3-methoxy-phenyl)-5-methoxy-2-methylol-2,3-dihydropyrano[5,6-h][1,4]benzodioxin-9-one	Beta-3 adrenergic receptor	P13945	ADRB3	0.037
Radix Saposhnikoviae（RS）	MOL000011	(2R,3R)-3-(4-hydroxy-3-methoxy-phenyl)-5-methoxy-2-methylol-2,3-dihydropyrano[5,6-h][1,4]benzodioxin-9-one	Peroxisome proliferator-activated receptor gamma	P37231	PPARG	0.044
Radix Saposhnikoviae（RS）	MOL000011	(2R,3R)-3-(4-hydroxy-3-methoxy-phenyl)-5-methoxy-2-methylol-2,3-dihydropyrano[5,6-h][1,4]benzodioxin-9-one	Nuclear receptor coactivator 2	Q15596	NCOA2	0.044
Radix Saposhnikoviae（RS）	MOL000011	(2R,3R)-3-(4-hydroxy-3-methoxy-phenyl)-5-methoxy-2-methylol-2,3-dihydropyrano[5,6-h][1,4]benzodioxin-9-one	Trypsin-1	P07477	PRSS1	0.05
Radix Saposhnikoviae（RS）	MOL000011	(2R,3R)-3-(4-hydroxy-3-methoxy-phenyl)-5-methoxy-2-methylol-2,3-dihydropyrano[5,6-h][1,4]benzodioxin-9-one	Tyrosine-protein kinase HCK	P08631	HCK	0.05
Radix Saposhnikoviae（RS）	MOL000011	(2R,3R)-3-(4-hydroxy-3-methoxy-phenyl)-5-methoxy-2-methylol-2,3-dihydropyrano[5,6-h][1,4]benzodioxin-9-one	Macrophage migration inhibitory factor	P14174	MIF	0.05
Radix Saposhnikoviae（RS）	MOL000011	(2R,3R)-3-(4-hydroxy-3-methoxy-phenyl)-5-methoxy-2-methylol-2,3-dihydropyrano[5,6-h][1,4]benzodioxin-9-one	Calcium/calmodulin-dependent protein kinase type II subunit delta	Q13557	CAMK2D	0.05
Radix Saposhnikoviae（RS）	MOL000011	(2R,3R)-3-(4-hydroxy-3-methoxy-phenyl)-5-methoxy-2-methylol-2,3-dihydropyrano[5,6-h][1,4]benzodioxin-9-one	STE20-like serine/threonine-protein kinase	Q9H2G2	SLK	0.05
Radix Saposhnikoviae（RS）	MOL000011	(2R,3R)-3-(4-hydroxy-3-methoxy-phenyl)-5-methoxy-2-methylol-2,3-dihydropyrano[5,6-h][1,4]benzodioxin-9-one	cGMP-inhibited 3',5'-cyclic phosphodiesterase A	Q14432	PDE3A	0.052
Radix Saposhnikoviae（RS）	MOL000011	(2R,3R)-3-(4-hydroxy-3-methoxy-phenyl)-5-methoxy-2-methylol-2,3-dihydropyrano[5,6-h][1,4]benzodioxin-9-one	cAMP and cAMP-inhibited cGMP 3',5'-cyclic phosphodiesterase 10A	Q9Y233	PDE10A	0.052
Radix Saposhnikoviae（RS）	MOL000011	(2R,3R)-3-(4-hydroxy-3-methoxy-phenyl)-5-methoxy-2-methylol-2,3-dihydropyrano[5,6-h][1,4]benzodioxin-9-one	Platelet glycoprotein IX	P14770	GP9	0.055

Radix Saposhnikoviae（RS）	MOL000011	(2R,3R)-3-(4-hydroxy-3-methoxy-phenyl)-5-methoxy-2-methylol-2,3-dihydropyrano[5,6-h][1,4]benzodioxin-9-one	Sterol O-acyltransferase 2	O75908	SOAT2	0.056
Radix Saposhnikoviae（RS）	MOL000011	(2R,3R)-3-(4-hydroxy-3-methoxy-phenyl)-5-methoxy-2-methylol-2,3-dihydropyrano[5,6-h][1,4]benzodioxin-9-one	Sterol O-acyltransferase 1	P35610	SOAT1	0.056
Radix Saposhnikoviae（RS）	MOL000011	(2R,3R)-3-(4-hydroxy-3-methoxy-phenyl)-5-methoxy-2-methylol-2,3-dihydropyrano[5,6-h][1,4]benzodioxin-9-one	Muscarinic acetylcholine receptor M4	P08173	CHRM4	0.06
Radix Saposhnikoviae（RS）	MOL000011	(2R,3R)-3-(4-hydroxy-3-methoxy-phenyl)-5-methoxy-2-methylol-2,3-dihydropyrano[5,6-h][1,4]benzodioxin-9-one	Muscarinic acetylcholine receptor M1	P11229	CHRM1	0.06
Radix Saposhnikoviae（RS）	MOL000011	(2R,3R)-3-(4-hydroxy-3-methoxy-phenyl)-5-methoxy-2-methylol-2,3-dihydropyrano[5,6-h][1,4]benzodioxin-9-one	Glycogen synthase kinase-3 beta	P49841	GSK3B	0.069
Radix Saposhnikoviae（RS）	MOL000011	(2R,3R)-3-(4-hydroxy-3-methoxy-phenyl)-5-methoxy-2-methylol-2,3-dihydropyrano[5,6-h][1,4]benzodioxin-9-one	Potassium channel subfamily K member 1	O00180	KCNK1	0.074
Radix Saposhnikoviae（RS）	MOL000011	(2R,3R)-3-(4-hydroxy-3-methoxy-phenyl)-5-methoxy-2-methylol-2,3-dihydropyrano[5,6-h][1,4]benzodioxin-9-one	Gamma-aminobutyric-acid receptor subunit alpha-1	P14867	GABRA1	0.074
Radix Saposhnikoviae（RS）	MOL000011	(2R,3R)-3-(4-hydroxy-3-methoxy-phenyl)-5-methoxy-2-methylol-2,3-dihydropyrano[5,6-h][1,4]benzodioxin-9-one	Gamma-aminobutyric-acid receptor subunit alpha-2	P47869	GABRA2	0.074
Radix Saposhnikoviae（RS）	MOL000011	(2R,3R)-3-(4-hydroxy-3-methoxy-phenyl)-5-methoxy-2-methylol-2,3-dihydropyrano[5,6-h][1,4]benzodioxin-9-one	Sodium channel protein type 5 subunit alpha	Q14524	SCN5A	0.074
Radix Saposhnikoviae（RS）	MOL000011	(2R,3R)-3-(4-hydroxy-3-methoxy-phenyl)-5-methoxy-2-methylol-2,3-dihydropyrano[5,6-h][1,4]benzodioxin-9-one	5-hydroxytryptamine 1A receptor	P08908	HTR1A	0.083
Radix Saposhnikoviae（RS）	MOL000011	(2R,3R)-3-(4-hydroxy-3-methoxy-phenyl)-5-methoxy-2-methylol-2,3-dihydropyrano[5,6-h][1,4]benzodioxin-9-one	Beta-2 adrenergic receptor	P07550	ADRB2	0.084
Radix Saposhnikoviae（RS）	MOL000011	(2R,3R)-3-(4-hydroxy-3-methoxy-phenyl)-5-methoxy-2-methylol-2,3-dihydropyrano[5,6-h][1,4]benzodioxin-9-one	Proto-oncogene serine/threonine-protein kinase Pim-1	P11309	PIM1	0.094
Radix Saposhnikoviae（RS）	MOL000011	(2R,3R)-3-(4-hydroxy-3-methoxy-phenyl)-5-methoxy-2-methylol-2,3-dihydropyrano[5,6-h][1,4]benzodioxin-9-one	Muscarinic acetylcholine receptor M2	P08172	CHRM2	0.095
Radix Saposhnikoviae（RS）	MOL000011	(2R,3R)-3-(4-hydroxy-3-methoxy-phenyl)-5-methoxy-2-methylol-2,3-dihydropyrano[5,6-h][1,4]benzodioxin-9-one	Estrogen-related receptor gamma	P62508	ESRRG	0.101
Radix Saposhnikoviae（RS）	MOL000011	(2R,3R)-3-(4-hydroxy-3-methoxy-phenyl)-5-methoxy-2-methylol-2,3-dihydropyrano[5,6-h][1,4]benzodioxin-9-one	Fibroblast growth factor receptor 2	P21802	FGFR2	0.102
Radix Saposhnikoviae（RS）	MOL000011	(2R,3R)-3-(4-hydroxy-3-methoxy-phenyl)-5-methoxy-2-methylol-2,3-dihydropyrano[5,6-h][1,4]benzodioxin-9-one	Dual specificity protein kinase CLK1	P49759	CLK1	0.103
Radix Saposhnikoviae（RS）	MOL000011	(2R,3R)-3-(4-hydroxy-3-methoxy-phenyl)-5-methoxy-2-methylol-2,3-dihydropyrano[5,6-h][1,4]benzodioxin-9-one	Toll-like receptor 7	Q9NYK1	TLR7	0.103
Radix Saposhnikoviae（RS）	MOL000011	(2R,3R)-3-(4-hydroxy-3-methoxy-phenyl)-5-methoxy-2-methylol-2,3-dihydropyrano[5,6-h][1,4]benzodioxin-9-one	Cannabinoid receptor 1	P21554	CNR1	0.107
Radix Saposhnikoviae（RS）	MOL000011	(2R,3R)-3-(4-hydroxy-3-methoxy-phenyl)-5-methoxy-2-methylol-2,3-dihydropyrano[5,6-h][1,4]benzodioxin-9-one	Ig heavy chain V-I region ND	P01744	IGKC	0.108
Radix Saposhnikoviae（RS）	MOL000011	(2R,3R)-3-(4-hydroxy-3-methoxy-phenyl)-5-methoxy-2-methylol-2,3-dihydropyrano[5,6-h][1,4]benzodioxin-9-one	Tyrosine-protein phosphatase non-receptor type 1	P18031	PTPN1	0.108
Radix Saposhnikoviae（RS）	MOL000011	(2R,3R)-3-(4-hydroxy-3-methoxy-phenyl)-5-methoxy-2-methylol-2,3-dihydropyrano[5,6-h][1,4]benzodioxin-9-one	ATP-sensitive inward rectifier potassium channel 1	P48048	KCNJ1	0.108
Radix Saposhnikoviae（RS）	MOL000011	(2R,3R)-3-(4-hydroxy-3-methoxy-phenyl)-5-methoxy-2-methylol-2,3-dihydropyrano[5,6-h][1,4]benzodioxin-9-one	MAP kinase-activated protein kinase 2	P49137	MAPKAPK2	0.109
Radix Saposhnikoviae（RS）	MOL000011	(2R,3R)-3-(4-hydroxy-3-methoxy-phenyl)-5-methoxy-2-methylol-2,3-dihydropyrano[5,6-h][1,4]benzodioxin-9-one	Prothrombin	P00734	F2	0.111

Radix Saposhnikoviae（RS）	MOL000011	(2R,3R)-3-(4-hydroxy-3-methoxy-phenyl)-5-methoxy-2-methylol-2,3-dihydropyrano[5,6-h][1,4]benzodioxin-9-one	Casein kinase I isoform gamma-1	Q9HCP0	CSNK1G1	0.112
Radix Saposhnikoviae（RS）	MOL000011	(2R,3R)-3-(4-hydroxy-3-methoxy-phenyl)-5-methoxy-2-methylol-2,3-dihydropyrano[5,6-h][1,4]benzodioxin-9-one	D-HSCDK2	O75100	CA11	0.113
Radix Saposhnikoviae（RS）	MOL000011	(2R,3R)-3-(4-hydroxy-3-methoxy-phenyl)-5-methoxy-2-methylol-2,3-dihydropyrano[5,6-h][1,4]benzodioxin-9-one	Epidermal growth factor receptor	P00533	EGFR	0.115
Radix Saposhnikoviae（RS）	MOL000011	(2R,3R)-3-(4-hydroxy-3-methoxy-phenyl)-5-methoxy-2-methylol-2,3-dihydropyrano[5,6-h][1,4]benzodioxin-9-one	S-methyl-5-thioadenosine phosphorylase	Q13126	MTAP	0.115
Radix Saposhnikoviae（RS）	MOL000011	(2R,3R)-3-(4-hydroxy-3-methoxy-phenyl)-5-methoxy-2-methylol-2,3-dihydropyrano[5,6-h][1,4]benzodioxin-9-one	cAMP-specific 3',5'-cyclic phosphodiesterase 4A	P27815	PDE4A	0.116
Radix Saposhnikoviae（RS）	MOL000011	(2R,3R)-3-(4-hydroxy-3-methoxy-phenyl)-5-methoxy-2-methylol-2,3-dihydropyrano[5,6-h][1,4]benzodioxin-9-one	cAMP-specific 3',5'-cyclic phosphodiesterase 4B	Q07343	PDE4B	0.118
Radix Saposhnikoviae（RS）	MOL000011	(2R,3R)-3-(4-hydroxy-3-methoxy-phenyl)-5-methoxy-2-methylol-2,3-dihydropyrano[5,6-h][1,4]benzodioxin-9-one	Mitogen-activated protein kinase 14	Q16539	MAPK14	0.118
Radix Saposhnikoviae（RS）	MOL000011	(2R,3R)-3-(4-hydroxy-3-methoxy-phenyl)-5-methoxy-2-methylol-2,3-dihydropyrano[5,6-h][1,4]benzodioxin-9-one	Calmodulin	P62158		0.119
Radix Saposhnikoviae（RS）	MOL000011	(2R,3R)-3-(4-hydroxy-3-methoxy-phenyl)-5-methoxy-2-methylol-2,3-dihydropyrano[5,6-h][1,4]benzodioxin-9-one	Activin receptor type-1	Q04771	ACVR1	0.123
Radix Saposhnikoviae（RS）	MOL000011	(2R,3R)-3-(4-hydroxy-3-methoxy-phenyl)-5-methoxy-2-methylol-2,3-dihydropyrano[5,6-h][1,4]benzodioxin-9-one	Beta-1 adrenergic receptor	P08588	ADRB1	0.138
Radix Saposhnikoviae（RS）	MOL000011	(2R,3R)-3-(4-hydroxy-3-methoxy-phenyl)-5-methoxy-2-methylol-2,3-dihydropyrano[5,6-h][1,4]benzodioxin-9-one	Cell division control protein 2 homolog	P06493	CDK1	0.154
Radix Saposhnikoviae（RS）	MOL000011	(2R,3R)-3-(4-hydroxy-3-methoxy-phenyl)-5-methoxy-2-methylol-2,3-dihydropyrano[5,6-h][1,4]benzodioxin-9-one	Cell division protein kinase 5	Q00535	CDK5	0.154
Radix Saposhnikoviae（RS）	MOL000011	(2R,3R)-3-(4-hydroxy-3-methoxy-phenyl)-5-methoxy-2-methylol-2,3-dihydropyrano[5,6-h][1,4]benzodioxin-9-one	Coagulation factor VII	P08709	F7	0.224
Radix Saposhnikoviae（RS）	MOL000011	(2R,3R)-3-(4-hydroxy-3-methoxy-phenyl)-5-methoxy-2-methylol-2,3-dihydropyrano[5,6-h][1,4]benzodioxin-9-one	cAMP-specific 3',5'-cyclic phosphodiesterase 4D	Q08499	PDE4D	0.246
Radix Saposhnikoviae（RS）	MOL000011	(2R,3R)-3-(4-hydroxy-3-methoxy-phenyl)-5-methoxy-2-methylol-2,3-dihydropyrano[5,6-h][1,4]benzodioxin-9-one	Hemoglobin subunit alpha	P69905	HBA1	0.269
Radix Saposhnikoviae（RS）	MOL000011	(2R,3R)-3-(4-hydroxy-3-methoxy-phenyl)-5-methoxy-2-methylol-2,3-dihydropyrano[5,6-h][1,4]benzodioxin-9-one	Cell division protein kinase 2	P24941	CDK2	0.277
Radix Saposhnikoviae（RS）	MOL000011	(2R,3R)-3-(4-hydroxy-3-methoxy-phenyl)-5-methoxy-2-methylol-2,3-dihydropyrano[5,6-h][1,4]benzodioxin-9-one	Prostaglandin G/H synthase 2	P35354	PTGS2	0.341
Radix Saposhnikoviae（RS）	MOL000011	(2R,3R)-3-(4-hydroxy-3-methoxy-phenyl)-5-methoxy-2-methylol-2,3-dihydropyrano[5,6-h][1,4]benzodioxin-9-one	Nuclear receptor coactivator 1	Q15788	NCOA1	0.461
Radix Saposhnikoviae（RS）	MOL000011	(2R,3R)-3-(4-hydroxy-3-methoxy-phenyl)-5-methoxy-2-methylol-2,3-dihydropyrano[5,6-h][1,4]benzodioxin-9-one	Cyclin-A2	P20248	CCNA2	0.605
Radix Saposhnikoviae（RS）	MOL000011	(2R,3R)-3-(4-hydroxy-3-methoxy-phenyl)-5-methoxy-2-methylol-2,3-dihydropyrano[5,6-h][1,4]benzodioxin-9-one	Estrogen receptor beta	Q92731	ESR2	0.826
Radix Saposhnikoviae（RS）	MOL000011	(2R,3R)-3-(4-hydroxy-3-methoxy-phenyl)-5-methoxy-2-methylol-2,3-dihydropyrano[5,6-h][1,4]benzodioxin-9-one	Estrogen receptor	P03372	ESR1	1
Radix Saposhnikoviae（RS）	MOL000173	wogonin	Sodium/hydrogen exchanger 1	P19634	SLC9A1	0.01
Radix Saposhnikoviae（RS）	MOL000173	wogonin	Amiloride-sensitive amine oxidase [copper-containing]	P19801	AOC1	0.01
Radix Saposhnikoviae（RS）	MOL000173	wogonin	Amiloride-sensitive sodium channel subunit delta	P51172	SCNN1D	0.01
Radix Saposhnikoviae（RS）	MOL000173	wogonin	Amiloride-sensitive cation channel 2, neuronal	P78348	ASIC1	0.01
Radix Saposhnikoviae（RS）	MOL000173	wogonin	Amiloride-sensitive cation channel 1, neuronal	Q16515	ASIC2	0.01

Radix Saposhnikoviae（RS）	MOL000173	wogonin	2,4-dienoyl-CoA reductase, mitochondrial	Q16698	DECR1	0.011
Radix Saposhnikoviae（RS）	MOL000173	wogonin	Amine oxidase [flavin-containing] A	P21397	MAOA	0.012
Radix Saposhnikoviae（RS）	MOL000173	wogonin	Chromaffin granule amine transporter	P54219	SLC18A1	0.012
Radix Saposhnikoviae（RS）	MOL000173	wogonin	Synaptic vesicular amine transporter	Q05940	SLC18A2	0.012
Radix Saposhnikoviae（RS）	MOL000173	wogonin	Lactase-phlorizin hydrolase	P09848	LCT	0.013
Radix Saposhnikoviae（RS）	MOL000173	wogonin	Gamma-aminobutyric-acid receptor subunit beta-2	P47870	GABRB2	0.013
Radix Saposhnikoviae（RS）	MOL000173	wogonin	Gamma-aminobutyric acid receptor subunit theta	Q9UN88	GABRQ	0.013
Radix Saposhnikoviae（RS）	MOL000173	wogonin	Neuronal acetylcholine receptor subunit alpha-4	P43681	CHRNA4	0.014
Radix Saposhnikoviae（RS）	MOL000173	wogonin	Glutamate receptor, ionotropic kainate 2	Q13002	GRIK2	0.014
Radix Saposhnikoviae（RS）	MOL000173	wogonin	Death-associated protein kinase 3	O43293	DAPK3	0.017
Radix Saposhnikoviae（RS）	MOL000173	wogonin	Tyrosine-protein kinase JAK2	O60674	JAK2	0.017
Radix Saposhnikoviae（RS）	MOL000173	wogonin	Tyrosine-protein kinase JAK1	P23458	JAK1	0.017
Radix Saposhnikoviae（RS）	MOL000173	wogonin	Tyrosine-protein kinase JAK3	P52333	JAK3	0.017
Radix Saposhnikoviae（RS）	MOL000173	wogonin	5-hydroxytryptamine 4 receptor	Q13639	HTR4	0.017
Radix Saposhnikoviae（RS）	MOL000173	wogonin	Potassium channel subfamily K member 6	Q9Y257	KCNK6	0.017
Radix Saposhnikoviae（RS）	MOL000173	wogonin	Cytochrome b-c1 complex subunit 8	O14949	UQCRQ	0.018
Radix Saposhnikoviae（RS）	MOL000173	wogonin	Cytochrome b-c1 complex subunit 10	O14957	UQCR11	0.018
Radix Saposhnikoviae（RS）	MOL000173	wogonin	Cytochrome b-c1 complex subunit 6, mitochondrial	P07919	UQCRH	0.018
Radix Saposhnikoviae（RS）	MOL000173	wogonin	Retinoic acid receptor alpha	P10276	RARA	0.018
Radix Saposhnikoviae（RS）	MOL000173	wogonin	Retinoic acid receptor beta	P10826	RARB	0.018
Radix Saposhnikoviae（RS）	MOL000173	wogonin	Retinoic acid receptor gamma-1	P13631	RARG	0.018
Radix Saposhnikoviae（RS）	MOL000173	wogonin	Retinoic acid receptor RXR-alpha	P19793	RXRA	0.018
Radix Saposhnikoviae（RS）	MOL000173	wogonin	Cytochrome b-c1 complex subunit 2, mitochondrial	P22695	UQCRC2	0.018
Radix Saposhnikoviae（RS）	MOL000173	wogonin	Ubiquinol-cytochrome-c reductase complex core protein 1, mitochondrial	P31930	UQCRC1	0.018
Radix Saposhnikoviae（RS）	MOL000173	wogonin	Retinoic acid receptor RXR-gamma	P48443	RXRG	0.018
Radix Saposhnikoviae（RS）	MOL000173	wogonin	Cytochrome b-c1 complex subunit 9	Q9UDW1	UQCR10	0.018
Radix Saposhnikoviae（RS）	MOL000173	wogonin	Purine nucleoside phosphorylase	P00491	PNP	0.02
Radix Saposhnikoviae（RS）	MOL000173	wogonin	DNA polymerase alpha catalytic subunit	P09884	POLA1	0.02
Radix Saposhnikoviae（RS）	MOL000173	wogonin	Ribonucleoside-diphosphate reductase large subunit	P23921	RRM1	0.02
Radix Saposhnikoviae（RS）	MOL000173	wogonin	Ribonucleoside-diphosphate reductase M2 subunit	P31350	RRM2	0.02
Radix Saposhnikoviae（RS）	MOL000173	wogonin	DNA polymerase epsilon subunit 2	P56282	POLE2	0.02
Radix Saposhnikoviae（RS）	MOL000173	wogonin	DNA polymerase epsilon catalytic subunit A	Q07864	POLE	0.02
Radix Saposhnikoviae（RS）	MOL000173	wogonin	Ribonucleoside-diphosphate reductase subunit M2 B	Q7LG56	RRM2B	0.02
Radix Saposhnikoviae（RS）	MOL000173	wogonin	DNA polymerase epsilon subunit 3	Q9NRF9	POLE3	0.02
Radix Saposhnikoviae（RS）	MOL000173	wogonin	Interleukin-3	P08700	IL3	0.021
Radix Saposhnikoviae（RS）	MOL000173	wogonin	Protein S100-A12	P80511	S100A12	0.021
Radix Saposhnikoviae（RS）	MOL000173	wogonin	Protein S100-A13	Q99584	S100A13	0.021
Radix Saposhnikoviae（RS）	MOL000173	wogonin	Gamma-aminobutyric acid receptor subunit rho-3	A8MPY1	GABRR3	0.022
Radix Saposhnikoviae（RS）	MOL000173	wogonin	Gamma-aminobutyric acid receptor subunit pi	O00591	GABRP	0.022
Radix Saposhnikoviae（RS）	MOL000173	wogonin	Gamma-aminobutyric acid receptor subunit delta	O14764	GABRD	0.022
Radix Saposhnikoviae（RS）	MOL000173	wogonin	Gamma-aminobutyric-acid receptor subunit beta-1	P18505	GABRB1	0.022
Radix Saposhnikoviae（RS）	MOL000173	wogonin	Gamma-aminobutyric acid receptor subunit gamma-2	P18507	GABRG2	0.022
Radix Saposhnikoviae（RS）	MOL000173	wogonin	Gamma-aminobutyric-acid receptor subunit rho-1	P24046	GABRR1	0.022
Radix Saposhnikoviae（RS）	MOL000173	wogonin	Gamma-aminobutyric acid receptor subunit rho-2	P28476	GABRR2	0.022
Radix Saposhnikoviae（RS）	MOL000173	wogonin	Gamma-aminobutyric acid receptor subunit epsilon	P78334	GABRE	0.022
Radix Saposhnikoviae（RS）	MOL000173	wogonin	Gamma-aminobutyric acid receptor subunit gamma-1	Q8N1C3	GABRG1	0.022
Radix Saposhnikoviae（RS）	MOL000173	wogonin	Gamma-aminobutyric acid receptor subunit gamma-3	Q99928	GABRG3	0.022

Radix Saposhnikoviae（RS）	MOL000173	wogonin	Phenylalanine-4-hydroxylase	P00439	PAH	0.023
Radix Saposhnikoviae（RS）	MOL000173	wogonin	Cell division protein kinase 4	P11802	CDK4	0.023
Radix Saposhnikoviae（RS）	MOL000173	wogonin	Cell division protein kinase 7	P50613	CDK7	0.023
Radix Saposhnikoviae（RS）	MOL000173	wogonin	Cell division protein kinase 9	P50750	CDK9	0.023
Radix Saposhnikoviae（RS）	MOL000173	wogonin	Potassium voltage-gated channel subfamily H member 2	Q12809	KCNH2	0.023
Radix Saposhnikoviae（RS）	MOL000173	wogonin	Serine/threonine-protein kinase 17B	O94768	STK17B	0.024
Radix Saposhnikoviae（RS）	MOL000173	wogonin	Beta-3 adrenergic receptor	P13945	ADRB3	0.024
Radix Saposhnikoviae（RS）	MOL000173	wogonin	ATP synthase subunit gamma, mitochondrial	P36542	ATP5F1C	0.024
Radix Saposhnikoviae（RS）	MOL000173	wogonin	Solute carrier family 22 member 6	Q4U2R8	SLC22A6	0.024
Radix Saposhnikoviae（RS）	MOL000173	wogonin	UDP-glucuronosyltransferase 3A1	Q6NUS8	UGT3A1	0.024
Radix Saposhnikoviae（RS）	MOL000173	wogonin	Solute carrier family 22 member 8	Q8TCC7	SLC22A8	0.024
Radix Saposhnikoviae（RS）	MOL000173	wogonin	Solute carrier family 22 member 11	Q9NSA0	SLC22A11	0.024
Radix Saposhnikoviae（RS）	MOL000173	wogonin	cAMP response element-binding protein	P16220	CREB1	0.025
Radix Saposhnikoviae（RS）	MOL000173	wogonin	Carbonic anhydrase 4	P22748	CA4	0.025
Radix Saposhnikoviae（RS）	MOL000173	wogonin	Amiloride-sensitive sodium channel subunit alpha	P37088	SCNN1A	0.025
Radix Saposhnikoviae（RS）	MOL000173	wogonin	Nociceptin receptor	P41146	OPRL1	0.025
Radix Saposhnikoviae（RS）	MOL000173	wogonin	Amiloride-sensitive sodium channel subunit beta	P51168	SCNN1B	0.025
Radix Saposhnikoviae（RS）	MOL000173	wogonin	Amiloride-sensitive sodium channel subunit gamma	P51170	SCNN1G	0.025
Radix Saposhnikoviae（RS）	MOL000173	wogonin	Sodium/potassium-transporting ATPase gamma chain	P54710	FXYD2	0.025
Radix Saposhnikoviae（RS）	MOL000173	wogonin	Cyclin-dependent kinase 5 activator 1	Q15078	CDK5R1	0.025
Radix Saposhnikoviae（RS）	MOL000173	wogonin	3-phosphoinositide-dependent protein kinase 1	O15530	PDPK1	0.026
Radix Saposhnikoviae（RS）	MOL000173	wogonin	Tyrosine-protein kinase Lyn	P07948	LYN	0.026
Radix Saposhnikoviae（RS）	MOL000173	wogonin	Tyrosine-protein kinase CSK	P41240	CSK	0.026
Radix Saposhnikoviae（RS）	MOL000173	wogonin	Tyrosine-protein kinase ZAP-70	P43403	ZAP70	0.026
Radix Saposhnikoviae（RS）	MOL000173	wogonin	Tyrosine-protein kinase SYK	P43405	SYK	0.026
Radix Saposhnikoviae（RS）	MOL000173	wogonin	Protein kinase C theta type	Q04759	PRKCQ	0.026
Radix Saposhnikoviae（RS）	MOL000173	wogonin	Tyrosine-protein kinase ITK/TSK	Q08881	ITK	0.026
Radix Saposhnikoviae（RS）	MOL000173	wogonin	85 kDa calcium-independent phospholipase A2	O60733	PLA2G6	0.028
Radix Saposhnikoviae（RS）	MOL000173	wogonin	Dihydrofolate reductase	P00374	DHFR	0.028
Radix Saposhnikoviae（RS）	MOL000173	wogonin	Cytosolic phospholipase A2	P47712	PLA2G4A	0.028
Radix Saposhnikoviae（RS）	MOL000173	wogonin	Neuronal acetylcholine receptor subunit alpha-7	P36544	CHRNA7	0.029
Radix Saposhnikoviae（RS）	MOL000173	wogonin	Solute carrier family 12 member 2	P55011	SLC12A2	0.029
Radix Saposhnikoviae（RS）	MOL000173	wogonin	Solute carrier family 12 member 5	Q9H2X9	SLC12A5	0.029
Radix Saposhnikoviae（RS）	MOL000173	wogonin	Solute carrier family 12 member 4	Q9UP95	SLC12A4	0.029
Radix Saposhnikoviae（RS）	MOL000173	wogonin	Cytochrome b	P00156	MT-CYB	0.03
Radix Saposhnikoviae（RS）	MOL000173	wogonin	Cytochrome c1, heme protein, mitochondrial	P08574	CYC1	0.03
Radix Saposhnikoviae（RS）	MOL000173	wogonin	Sodium-dependent noradrenaline transporter	P23975	SLC6A2	0.03
Radix Saposhnikoviae（RS）	MOL000173	wogonin	Sodium-dependent serotonin transporter	P31645	SLC6A4	0.03
Radix Saposhnikoviae（RS）	MOL000173	wogonin	Cytochrome b-c1 complex subunit Rieske, mitochondrial	P47985	UQCRFS1	0.03
Radix Saposhnikoviae（RS）	MOL000173	wogonin	Neuronal acetylcholine receptor subunit alpha-10	Q9GZZ6	CHRNA10	0.03
Radix Saposhnikoviae（RS）	MOL000173	wogonin	Gamma-aminobutyric-acid receptor subunit alpha-4	P48169	GABRA4	0.033
Radix Saposhnikoviae（RS）	MOL000173	wogonin	Gamma-aminobutyric-acid receptor subunit alpha-6	Q16445	GABRA6	0.033
Radix Saposhnikoviae（RS）	MOL000173	wogonin	Potassium channel subfamily K member 1	O00180	KCNK1	0.034
Radix Saposhnikoviae（RS）	MOL000173	wogonin	Muscarinic acetylcholine receptor M1	P11229	CHRM1	0.034
Radix Saposhnikoviae（RS）	MOL000173	wogonin	Muscarinic acetylcholine receptor M3	P20309	CHRM3	0.034
Radix Saposhnikoviae（RS）	MOL000173	wogonin	Calcium/calmodulin-dependent protein kinase type II subunit delta	Q13557	CAMK2D	0.034
Radix Saposhnikoviae（RS）	MOL000173	wogonin	STE20-like serine/threonine-protein kinase	Q9H2G2	SLK	0.034

Radix Saposhnikoviae（RS）	MOL000173	wogonin	Guanine nucleotide-binding protein G(s) subunit alpha isoforms short	P63092	GNAS	0.035
Radix Saposhnikoviae（RS）	MOL000173	wogonin	Adenylate cyclase type 2	Q08462	ADCY2	0.035
Radix Saposhnikoviae（RS）	MOL000173	wogonin	Casein kinase I isoform gamma-2	P78368	CSNK1G2	0.036
Radix Saposhnikoviae（RS）	MOL000173	wogonin	cGMP-inhibited 3',5'-cyclic phosphodiesterase A	Q14432	PDE3A	0.036
Radix Saposhnikoviae（RS）	MOL000173	wogonin	Glutamate [NMDA] receptor subunit 3A	Q8TCU5	GRIN3A	0.036
Radix Saposhnikoviae（RS）	MOL000173	wogonin	Serine/threonine-protein kinase haspin	Q8TF76	HASPIN	0.036
Radix Saposhnikoviae（RS）	MOL000173	wogonin	cAMP and cAMP-inhibited cGMP 3',5'-cyclic phosphodiesterase 10A	Q9Y233	PDE10A	0.036
Radix Saposhnikoviae（RS）	MOL000173	wogonin	DNA-(apurinic or apyrimidinic site) lyase	P27695	APEX1	0.037
Radix Saposhnikoviae（RS）	MOL000173	wogonin	5-hydroxytryptamine 2B receptor	P41595	HTR2B	0.038
Radix Saposhnikoviae（RS）	MOL000173	wogonin	Acetylcholinesterase	P22303	ACHE	0.04
Radix Saposhnikoviae（RS）	MOL000173	wogonin	Methionine aminopeptidase 1	P53582	METAP1	0.041
Radix Saposhnikoviae（RS）	MOL000173	wogonin	Retinoic acid receptor RXR-beta	P28702	RXRB	0.042
Radix Saposhnikoviae（RS）	MOL000173	wogonin	Peptidyl-prolyl cis-trans isomerase, mitochondrial	P30405	PPIF	0.042
Radix Saposhnikoviae（RS）	MOL000173	wogonin	Gamma-aminobutyric-acid receptor subunit alpha-5	P31644	GABRA5	0.042
Radix Saposhnikoviae（RS）	MOL000173	wogonin	Gamma-aminobutyric-acid receptor subunit alpha-3	P34903	GABRA3	0.042
Radix Saposhnikoviae（RS）	MOL000173	wogonin	Urokinase-type plasminogen activator	P00749	PLAU	0.043
Radix Saposhnikoviae（RS）	MOL000173	wogonin	Ribosyldihydronicotinamide dehydrogenase [quinone]	P16083	NQO2	0.045
Radix Saposhnikoviae（RS）	MOL000173	wogonin	Nuclear receptor coactivator 2	Q15596	NCOA2	0.045
Radix Saposhnikoviae（RS）	MOL000173	wogonin	Retinal rod rhodopsin-sensitive cGMP 3',5'-cyclic phosphodiesterase subunit gamma	P18545	PDE6G	0.047
Radix Saposhnikoviae（RS）	MOL000173	wogonin	Sodium-dependent dopamine transporter	Q01959	SLC6A3	0.048
Radix Saposhnikoviae（RS）	MOL000173	wogonin	Sterol O-acyltransferase 2	O75908	SOAT2	0.049
Radix Saposhnikoviae（RS）	MOL000173	wogonin	Ig gamma-1 chain C region	P01857	IGHG1	0.049
Radix Saposhnikoviae（RS）	MOL000173	wogonin	Sterol O-acyltransferase 1	P35610	SOAT1	0.049
Radix Saposhnikoviae（RS）	MOL000173	wogonin	Progesterone receptor	P06401	PGR	0.05
Radix Saposhnikoviae（RS）	MOL000173	wogonin	Gonadotropin-releasing hormone receptor	P30968	GNRHR	0.05
Radix Saposhnikoviae（RS）	MOL000173	wogonin	Gonadotropin-releasing hormone II receptor	Q96P88	GNRHR2	0.05
Radix Saposhnikoviae（RS）	MOL000173	wogonin	Mitogen-activated protein kinase 8	P45983	MAPK8	0.051
Radix Saposhnikoviae（RS）	MOL000173	wogonin	Mitogen-activated protein kinase 10	P53779	MAPK10	0.051
Radix Saposhnikoviae（RS）	MOL000173	wogonin	Epidermal growth factor receptor	P00533	EGFR	0.053
Radix Saposhnikoviae（RS）	MOL000173	wogonin	ATP synthase subunit beta, mitochondrial	P06576	ATP5F1B	0.053
Radix Saposhnikoviae（RS）	MOL000173	wogonin	Tyrosine-protein kinase HCK	P08631	HCK	0.053
Radix Saposhnikoviae（RS）	MOL000173	wogonin	3 beta-hydroxysteroid dehydrogenase/Delta 5-->4-isomerase type I	P14060	HSD3B1	0.053
Radix Saposhnikoviae（RS）	MOL000173	wogonin	D(4) dopamine receptor	P21917	DRD4	0.053
Radix Saposhnikoviae（RS）	MOL000173	wogonin	ATP synthase subunit alpha, mitochondrial	P25705	ATP5F1A	0.053
Radix Saposhnikoviae（RS）	MOL000173	wogonin	3 beta-hydroxysteroid dehydrogenase/Delta 5-->4-isomerase type II	P26439	HSD3B2	0.053
Radix Saposhnikoviae（RS）	MOL000173	wogonin	5-hydroxytryptamine 1D receptor	P28221	HTR1D	0.053
Radix Saposhnikoviae（RS）	MOL000173	wogonin	5-hydroxytryptamine 1B receptor	P28222	HTR1B	0.053
Radix Saposhnikoviae（RS）	MOL000173	wogonin	Phosphatidylinositol-4,5-bisphosphate 3-kinase catalytic subunit gamma isoform	P48736	PIK3CG	0.053
Radix Saposhnikoviae（RS）	MOL000173	wogonin	Cell division protein kinase 6	Q00534	CDK6	0.053
Radix Saposhnikoviae（RS）	MOL000173	wogonin	Estrogen-related receptor gamma	P62508	ESRRG	0.054
Radix Saposhnikoviae（RS）	MOL000173	wogonin	Protein tyrosine kinase 2 beta	Q14289	PTK2B	0.054
Radix Saposhnikoviae（RS）	MOL000173	wogonin	Carbonic anhydrase 1	P00915	CA1	0.055
Radix Saposhnikoviae（RS）	MOL000173	wogonin	Carbonic anhydrase 2	P00918	CA2	0.055
Radix Saposhnikoviae（RS）	MOL000173	wogonin	Proto-oncogene serine/threonine-protein kinase Pim-1	P11309	PIM1	0.055
Radix Saposhnikoviae（RS）	MOL000173	wogonin	Casein kinase I isoform gamma-3	Q9Y6M4	CSNK1G3	0.055
Radix Saposhnikoviae（RS）	MOL000173	wogonin	Tripartite motif-containing protein 13	O60858	TRIM13	0.056
Radix Saposhnikoviae（RS）	MOL000173	wogonin	5-hydroxytryptamine 2A receptor	P28223	HTR2A	0.056

Radix Saposhnikoviae（RS）	MOL000173	wogonin	Proto-oncogene tyrosine-protein kinase LCK	P06239	LCK	0.057
Radix Saposhnikoviae（RS）	MOL000173	wogonin	Keratin, type II cytoskeletal 7	P08729	KRT7	0.057
Radix Saposhnikoviae（RS）	MOL000173	wogonin	Platelet glycoprotein IX	P14770	GP9	0.057
Radix Saposhnikoviae（RS）	MOL000173	wogonin	Gamma-aminobutyric-acid receptor subunit alpha-1	P14867	GABRA1	0.058
Radix Saposhnikoviae（RS）	MOL000173	wogonin	5-hydroxytryptamine 3 receptor	P46098	HTR3A	0.058
Radix Saposhnikoviae（RS）	MOL000173	wogonin	Gamma-aminobutyric-acid receptor subunit alpha-2	P47869	GABRA2	0.058
Radix Saposhnikoviae（RS）	MOL000173	wogonin	Sodium channel protein type 10 subunit alpha	Q9Y5Y9	SCN10A	0.058
Radix Saposhnikoviae（RS）	MOL000173	wogonin	Rho-associated protein kinase 1	Q13464	ROCK1	0.063
Radix Saposhnikoviae（RS）	MOL000173	wogonin	DNA polymerase	P04293	UL30	0.064
Radix Saposhnikoviae（RS）	MOL000173	wogonin	Solute carrier family 12 member 1	Q13621	SLC12A1	0.064
Radix Saposhnikoviae（RS）	MOL000173	wogonin	Glycogen phosphorylase, muscle form	P11217	PYGM	0.065
Radix Saposhnikoviae（RS）	MOL000173	wogonin	Toll-like receptor 7	Q9NYK1	TLR7	0.065
Radix Saposhnikoviae（RS）	MOL000173	wogonin	Alpha-1D adrenergic receptor	P25100	ADRA1D	0.066
Radix Saposhnikoviae（RS）	MOL000173	wogonin	Beta-1 adrenergic receptor	P08588	ADRB1	0.067
Radix Saposhnikoviae（RS）	MOL000173	wogonin	5-hydroxytryptamine 2C receptor	P28335	HTR2C	0.067
Radix Saposhnikoviae（RS）	MOL000173	wogonin	Low molecular weight phosphotyrosine protein phosphatase	P24666	ACP1	0.075
Radix Saposhnikoviae（RS）	MOL000173	wogonin	Thymidylate synthase	P04818	TYMS	0.076
Radix Saposhnikoviae（RS）	MOL000173	wogonin	Coagulation factor VII	P08709	F7	0.076
Radix Saposhnikoviae（RS）	MOL000173	wogonin	Cannabinoid receptor 2	P34972	CNR2	0.076
Radix Saposhnikoviae（RS）	MOL000173	wogonin	Serine/threonine-protein kinase 6	O14965	AURKA	0.078
Radix Saposhnikoviae（RS）	MOL000173	wogonin	Geranylgeranyl pyrophosphate synthetase	O95749	GGPS1	0.078
Radix Saposhnikoviae（RS）	MOL000173	wogonin	cAMP-specific 3',5'-cyclic phosphodiesterase 4A	P27815	PDE4A	0.078
Radix Saposhnikoviae（RS）	MOL000173	wogonin	Membrane copper amine oxidase	Q16853	AOC3	0.078
Radix Saposhnikoviae（RS）	MOL000173	wogonin	L-amino-acid oxidase	Q96RQ9	IL4I1	0.078
Radix Saposhnikoviae（RS）	MOL000173	wogonin	Tubulin beta-1 chain	Q9H4B7	TUBB1	0.078
Radix Saposhnikoviae（RS）	MOL000173	wogonin	Mitogen-activated protein kinase 14	Q16539	MAPK14	0.079
Radix Saposhnikoviae（RS）	MOL000173	wogonin	Hepatocyte growth factor receptor	P08581	MET	0.08
Radix Saposhnikoviae（RS）	MOL000173	wogonin	Cannabinoid receptor 1	P21554	CNR1	0.08
Radix Saposhnikoviae（RS）	MOL000173	wogonin	Endothelin-1 receptor	P25101	EDNRA	0.08
Radix Saposhnikoviae（RS）	MOL000173	wogonin	Ig kappa chain V-II region RPMI 6410	P06310	IGKV2-30	0.081
Radix Saposhnikoviae（RS）	MOL000173	wogonin	Beta-2 adrenergic receptor	P07550	ADRB2	0.081
Radix Saposhnikoviae（RS）	MOL000173	wogonin	Leukotriene A-4 hydrolase	P09960	LTA4H	0.081
Radix Saposhnikoviae（RS）	MOL000173	wogonin	Alpha-1B adrenergic receptor	P35368	ADRA1B	0.081
Radix Saposhnikoviae（RS）	MOL000173	wogonin	S-methyl-5-thioadenosine phosphorylase	Q13126	MTAP	0.081
Radix Saposhnikoviae（RS）	MOL000173	wogonin	Heat shock protein HSP 90-beta	P08238	HSP90AB1	0.082
Radix Saposhnikoviae（RS）	MOL000173	wogonin	D(1A) dopamine receptor	P21728	DRD1	0.082
Radix Saposhnikoviae（RS）	MOL000173	wogonin	D(1B) dopamine receptor	P21918	DRD5	0.082
Radix Saposhnikoviae（RS）	MOL000173	wogonin	Interferon gamma	P01579	IFNG	0.083
Radix Saposhnikoviae（RS）	MOL000173	wogonin	Phospholipase A2	P04054	PLA2G1B	0.083
Radix Saposhnikoviae（RS）	MOL000173	wogonin	Thyroid hormone receptor alpha	P10827	THRA	0.084
Radix Saposhnikoviae（RS）	MOL000173	wogonin	Nuclear receptor coactivator 5	Q9HCD5	NCOA5	0.084
Radix Saposhnikoviae（RS）	MOL000173	wogonin	Calcium/calmodulin-dependent protein kinase type II alpha chain	Q9UQM7	CAMK2A	0.086
Radix Saposhnikoviae（RS）	MOL000173	wogonin	Muscarinic acetylcholine receptor M2	P08172	CHRM2	0.087
Radix Saposhnikoviae（RS）	MOL000173	wogonin	Tubulin alpha-3 chain	Q71U36	TUBA1A	0.087
Radix Saposhnikoviae（RS）	MOL000173	wogonin	Glycogen synthase kinase-3 beta	P49841	GSK3B	0.088
Radix Saposhnikoviae（RS）	MOL000173	wogonin	5-hydroxytryptamine 1A receptor	P08908	HTR1A	0.09
Radix Saposhnikoviae（RS）	MOL000173	wogonin	MAP kinase-activated protein kinase 2	P49137	MAPKAPK2	0.09

Radix Saposhnikoviae（RS）	MOL000173	wogonin	Melatonin receptor type 1A	P48039	MTNR1A	0.094
Radix Saposhnikoviae（RS）	MOL000173	wogonin	Alpha-2A adrenergic receptor	P08913	ADRA2A	0.095
Radix Saposhnikoviae（RS）	MOL000173	wogonin	Alpha-1A adrenergic receptor	P35348	ADRA1A	0.095
Radix Saposhnikoviae（RS）	MOL000173	wogonin	Casein kinase II subunit alpha	P68400	CSNK2A1	0.095
Radix Saposhnikoviae（RS）	MOL000173	wogonin	Alpha-2C adrenergic receptor	P18825	ADRA2C	0.096
Radix Saposhnikoviae（RS）	MOL000173	wogonin	Peroxisome proliferator-activated receptor gamma	P37231	PPARG	0.096
Radix Saposhnikoviae（RS）	MOL000173	wogonin	DNA topoisomerase 2-alpha	P11388	TOP2A	0.097
Radix Saposhnikoviae（RS）	MOL000173	wogonin	D(3) dopamine receptor	P35462	DRD3	0.097
Radix Saposhnikoviae（RS）	MOL000173	wogonin	Inhibitor of nuclear factor kappa-B kinase subunit alpha	O15111	CHUK	0.098
Radix Saposhnikoviae（RS）	MOL000173	wogonin	Arachidonate 5-lipoxygenase	P09917	ALOX5	0.098
Radix Saposhnikoviae（RS）	MOL000173	wogonin	Group IIE secretory phospholipase A2	Q9NZK7	PLA2G2E	0.099
Radix Saposhnikoviae（RS）	MOL000173	wogonin	cGMP-specific 3',5'-cyclic phosphodiesterase	O76074	PDE5A	0.1
Radix Saposhnikoviae（RS）	MOL000173	wogonin	DNA polymerase kappa	Q9UBT6	POLK	0.101
Radix Saposhnikoviae（RS）	MOL000173	wogonin	Androgen receptor	P10275	AR	0.103
Radix Saposhnikoviae（RS）	MOL000173	wogonin	Ig kappa chain C region	P01834	IGKC	0.104
Radix Saposhnikoviae（RS）	MOL000173	wogonin	D-HSCDK2	O75100	CA11	0.115
Radix Saposhnikoviae（RS）	MOL000173	wogonin	Proto-oncogene tyrosine-protein kinase Src	P12931	SRC	0.116
Radix Saposhnikoviae（RS）	MOL000173	wogonin	Alpha-2B adrenergic receptor	P18089	ADRA2B	0.124
Radix Saposhnikoviae（RS）	MOL000173	wogonin	D(2) dopamine receptor	P14416	DRD2	0.126
Radix Saposhnikoviae（RS）	MOL000173	wogonin	Sodium channel protein type 5 subunit alpha	Q14524	SCN5A	0.126
Radix Saposhnikoviae（RS）	MOL000173	wogonin	Prothrombin	P00734	F2	0.131
Radix Saposhnikoviae（RS）	MOL000173	wogonin	cAMP-dependent protein kinase inhibitor alpha	P61925	PKIA	0.131
Radix Saposhnikoviae（RS）	MOL000173	wogonin	Cell division control protein 2 homolog	P06493	CDK1	0.146
Radix Saposhnikoviae（RS）	MOL000173	wogonin	Cell division protein kinase 5	Q00535	CDK5	0.146
Radix Saposhnikoviae（RS）	MOL000173	wogonin	Protein farnesyltransferase/geranylgeranyltransferase type I alpha subunit	P49354	FNTA	0.16
Radix Saposhnikoviae（RS）	MOL000173	wogonin	C-jun-amino-terminal kinase-interacting protein 1	Q9UQF2	MAPK8IP1	0.163
Radix Saposhnikoviae（RS）	MOL000173	wogonin	RAC-alpha serine/threonine-protein kinase	P31749	AKT1	0.164
Radix Saposhnikoviae（RS）	MOL000173	wogonin	cAMP-specific 3',5'-cyclic phosphodiesterase 4D	Q08499	PDE4D	0.166
Radix Saposhnikoviae（RS）	MOL000173	wogonin	cAMP-specific 3',5'-cyclic phosphodiesterase 4B	Q07343	PDE4B	0.169
Radix Saposhnikoviae（RS）	MOL000173	wogonin	Delta-type opioid receptor	P41143	OPRD1	0.179
Radix Saposhnikoviae（RS）	MOL000173	wogonin	Hemoglobin subunit alpha	P69905	HBA1	0.191
Radix Saposhnikoviae（RS）	MOL000173	wogonin	Kappa-type opioid receptor	P41145	OPRK1	0.241
Radix Saposhnikoviae（RS）	MOL000173	wogonin	Nitric oxide synthase, inducible	P35228	NOS2	0.244
Radix Saposhnikoviae（RS）	MOL000173	wogonin	Cell division protein kinase 2	P24941	CDK2	0.271
Radix Saposhnikoviae（RS）	MOL000173	wogonin	Trypsin-1	P07477	PRSS1	0.291
Radix Saposhnikoviae（RS）	MOL000173	wogonin	Nuclear receptor coactivator 1	Q15788	NCOA1	0.376
Radix Saposhnikoviae（RS）	MOL000173	wogonin	Mu-type opioid receptor	P35372	OPRM1	0.385
Radix Saposhnikoviae（RS）	MOL000173	wogonin	Prostaglandin G/H synthase 1	P23219	PTGS1	0.409
Radix Saposhnikoviae（RS）	MOL000173	wogonin	cAMP-dependent protein kinase catalytic subunit alpha	P17612	PRKACA	0.541
Radix Saposhnikoviae（RS）	MOL000173	wogonin	Estrogen receptor beta	Q92731	ESR2	0.633
Radix Saposhnikoviae（RS）	MOL000173	wogonin	Estrogen receptor	P03372	ESR1	0.881
Radix Saposhnikoviae（RS）	MOL000173	wogonin	Prostaglandin G/H synthase 2	P35354	PTGS2	0.882
Radix Saposhnikoviae（RS）	MOL000173	wogonin	Cyclin-A2	P20248	CCNA2	1
Radix Saposhnikoviae（RS）	MOL000358	beta-sitosterol	Retinoic acid receptor RXR-gamma	P48443	RXRG	0.01
Radix Saposhnikoviae（RS）	MOL000358	beta-sitosterol	Nuclear receptor 0B1	P51843	NR0B1	0.01
Radix Saposhnikoviae（RS）	MOL000358	beta-sitosterol	Retinoic acid-induced protein 3	Q8NFJ5	GPRC5A	0.01

Radix Saposhnikoviae（RS）	MOL000358	beta-sitosterol	Retinoic acid receptor alpha	P10276	RARA	0.023
Radix Saposhnikoviae（RS）	MOL000358	beta-sitosterol	Retinoic acid receptor beta	P10826	RARB	0.023
Radix Saposhnikoviae（RS）	MOL000358	beta-sitosterol	Retinal dehydrogenase 2	O94788	ALDH1A2	0.029
Radix Saposhnikoviae（RS）	MOL000358	beta-sitosterol	Retinal dehydrogenase 1	P00352	ALDH1A1	0.029
Radix Saposhnikoviae（RS）	MOL000358	beta-sitosterol	Potassium channel subfamily K member 1	O00180	KCNK1	0.036
Radix Saposhnikoviae（RS）	MOL000358	beta-sitosterol	Sodium channel protein type 5 subunit alpha	Q14524	SCN5A	0.036
Radix Saposhnikoviae（RS）	MOL000358	beta-sitosterol	Potassium channel subfamily K member 6	Q9Y257	KCNK6	0.036
Radix Saposhnikoviae（RS）	MOL000358	beta-sitosterol	Serine/threonine-protein phosphatase 2A 65 kDa regulatory subunit A alpha isoform	P30153	PPP2R1A	0.038
Radix Saposhnikoviae（RS）	MOL000358	beta-sitosterol	Serine/threonine-protein phosphatase PP1-alpha catalytic subunit	P62136	PPP1CA	0.038
Radix Saposhnikoviae（RS）	MOL000358	beta-sitosterol	Ig kappa chain C region	P01834	IGKC	0.043
Radix Saposhnikoviae（RS）	MOL000358	beta-sitosterol	Ig gamma-1 chain C region	P01857	IGHG1	0.043
Radix Saposhnikoviae（RS）	MOL000358	beta-sitosterol	Ig gamma-2 chain C region	P01859	IGHG2	0.043
Radix Saposhnikoviae（RS）	MOL000358	beta-sitosterol	Nuclear receptor subfamily 1 group I member 3	Q14994	NR1I3	0.05
Radix Saposhnikoviae（RS）	MOL000358	beta-sitosterol	Retinoic acid receptor gamma-1	P13631	RARG	0.052
Radix Saposhnikoviae（RS）	MOL000358	beta-sitosterol	Retinoic acid receptor RXR-beta	P28702	RXRB	0.052
Radix Saposhnikoviae（RS）	MOL000358	beta-sitosterol	Bile salt sulfotransferase	Q06520	SULT2A1	0.056
Radix Saposhnikoviae（RS）	MOL000358	beta-sitosterol	Dehydrogenase/reductase SDR family member 8	Q8NBQ5	HSD17B11	0.056
Radix Saposhnikoviae（RS）	MOL000358	beta-sitosterol	Platelet glycoprotein IX	P14770	GP9	0.063
Radix Saposhnikoviae（RS）	MOL000358	beta-sitosterol	Phospholipase A2	P04054	PLA2G1B	0.069
Radix Saposhnikoviae（RS）	MOL000358	beta-sitosterol	Phospholipase A2, membrane associated	P14555	PLA2G2A	0.069
Radix Saposhnikoviae（RS）	MOL000358	beta-sitosterol	3-oxo-5-alpha-steroid 4-dehydrogenase 1	P18405	SRD5A1	0.074
Radix Saposhnikoviae（RS）	MOL000358	beta-sitosterol	3-oxo-5-alpha-steroid 4-dehydrogenase 2	P31213	SRD5A2	0.074
Radix Saposhnikoviae（RS）	MOL000358	beta-sitosterol	3 beta-hydroxysteroid dehydrogenase/Delta 5-->4-isomerase type II	P26439	HSD3B2	0.079
Radix Saposhnikoviae（RS）	MOL000358	beta-sitosterol	Microtubule-associated protein 2	P11137	MAP2	0.08
Radix Saposhnikoviae（RS）	MOL000358	beta-sitosterol	Microtubule-associated protein 1A	P78559	MAP1A	0.08
Radix Saposhnikoviae（RS）	MOL000358	beta-sitosterol	Gonadotropin-releasing hormone receptor	P30968	GNRHR	0.082
Radix Saposhnikoviae（RS）	MOL000358	beta-sitosterol	Gonadotropin-releasing hormone II receptor	Q96P88	GNRHR2	0.082
Radix Saposhnikoviae（RS）	MOL000358	beta-sitosterol	Corticosteroid 11-beta-dehydrogenase isozyme 1	P28845	HSD11B1	0.084
Radix Saposhnikoviae（RS）	MOL000358	beta-sitosterol	Cytosolic phospholipase A2	P47712	PLA2G4A	0.084
Radix Saposhnikoviae（RS）	MOL000358	beta-sitosterol	Retinoic acid receptor RXR-alpha	P19793	RXRA	0.092
Radix Saposhnikoviae（RS）	MOL000358	beta-sitosterol	Serine/threonine-protein phosphatase 2A catalytic subunit alpha isoform	P67775	PPP2CA	0.095
Radix Saposhnikoviae（RS）	MOL000358	beta-sitosterol	Serine/threonine-protein phosphatase 2A 56 kDa regulatory subunit gamma isoform	Q13362	PPP2R5C	0.095
Radix Saposhnikoviae（RS）	MOL000358	beta-sitosterol	Sodium-dependent serotonin transporter	P31645	SLC6A4	0.146
Radix Saposhnikoviae（RS）	MOL000358	beta-sitosterol	Aldo-keto reductase family 1 member C1	Q04828	AKR1C1	0.147
Radix Saposhnikoviae（RS）	MOL000358	beta-sitosterol	Prostaglandin G/H synthase 2	P35354	PTGS2	0.15
Radix Saposhnikoviae（RS）	MOL000358	beta-sitosterol	Estrogen receptor beta	Q92731	ESR2	0.163
Radix Saposhnikoviae（RS）	MOL000358	beta-sitosterol	Mediator of RNA polymerase II transcription subunit 1	Q15648	MED1	0.164
Radix Saposhnikoviae（RS）	MOL000358	beta-sitosterol	Estradiol 17-beta-dehydrogenase 1	P14061	HSD17B1	0.173
Radix Saposhnikoviae（RS）	MOL000358	beta-sitosterol	3 beta-hydroxysteroid dehydrogenase/Delta 5-->4-isomerase type I	P14060	HSD3B1	0.175
Radix Saposhnikoviae（RS）	MOL000358	beta-sitosterol	Nuclear receptor coactivator 5	Q9HCD5	NCOA5	0.178
Radix Saposhnikoviae（RS）	MOL000358	beta-sitosterol	Androgen receptor	P10275	AR	0.184
Radix Saposhnikoviae（RS）	MOL000358	beta-sitosterol	Glucocorticoid receptor	P04150	NR3C1	0.187
Radix Saposhnikoviae（RS）	MOL000358	beta-sitosterol	Cannabinoid receptor 2	P34972	CNR2	0.191
Radix Saposhnikoviae（RS）	MOL000358	beta-sitosterol	Nuclear receptor coactivator 1	Q15788	NCOA1	0.296
Radix Saposhnikoviae（RS）	MOL000358	beta-sitosterol	Mineralocorticoid receptor	P08235	NR3C2	0.39
Radix Saposhnikoviae（RS）	MOL000358	beta-sitosterol	Estrogen receptor	P03372	ESR1	0.971
Radix Saposhnikoviae（RS）	MOL000358	beta-sitosterol	Progesterone receptor	P06401	PGR	1

Radix Saposhnikoviae（RS）	MOL000359	sitosterol	Retinoic acid receptor RXR-alpha	P19793	RXRA	0.016
Radix Saposhnikoviae（RS）	MOL000359	sitosterol	Nuclear receptor subfamily 1 group I member 3	Q14994	NR1I3	0.016
Radix Saposhnikoviae（RS）	MOL000359	sitosterol	Elongation factor Tu GTP-binding domain-containing protein 1	Q7Z2Z2	EFL1	0.026
Radix Saposhnikoviae（RS）	MOL000359	sitosterol	Potassium channel subfamily K member 1	O00180	KCNK1	0.032
Radix Saposhnikoviae（RS）	MOL000359	sitosterol	D-HSCDK2	O75100	CA11	0.032
Radix Saposhnikoviae（RS）	MOL000359	sitosterol	Cell division control protein 2 homolog	P06493	CDK1	0.032
Radix Saposhnikoviae（RS）	MOL000359	sitosterol	Cell division protein kinase 5	Q00535	CDK5	0.032
Radix Saposhnikoviae（RS）	MOL000359	sitosterol	Sodium channel protein type 5 subunit alpha	Q14524	SCN5A	0.032
Radix Saposhnikoviae（RS）	MOL000359	sitosterol	Potassium channel subfamily K member 6	Q9Y257	KCNK6	0.032
Radix Saposhnikoviae（RS）	MOL000359	sitosterol	Neuronal acetylcholine receptor subunit alpha-3	P32297	CHRNA3	0.033
Radix Saposhnikoviae（RS）	MOL000359	sitosterol	Neuronal acetylcholine receptor subunit alpha-7	P36544	CHRNA7	0.033
Radix Saposhnikoviae（RS）	MOL000359	sitosterol	Neuronal acetylcholine receptor subunit alpha-4	P43681	CHRNA4	0.033
Radix Saposhnikoviae（RS）	MOL000359	sitosterol	Phosphatidylinositol 3-kinase regulatory subunit alpha	P27986	PIK3R1	0.034
Radix Saposhnikoviae（RS）	MOL000359	sitosterol	Phosphatidylinositol-4,5-bisphosphate 3-kinase catalytic subunit gamma isoform	P48736	PIK3CG	0.034
Radix Saposhnikoviae（RS）	MOL000359	sitosterol	Serine/threonine-protein kinase PLK1	P53350	PLK1	0.034
Radix Saposhnikoviae（RS）	MOL000359	sitosterol	Dehydrogenase/reductase SDR family member 8	Q8NBQ5	HSD17B11	0.041
Radix Saposhnikoviae（RS）	MOL000359	sitosterol	Ig kappa chain C region	P01834	IGKC	0.042
Radix Saposhnikoviae（RS）	MOL000359	sitosterol	Ig gamma-1 chain C region	P01857	IGHG1	0.042
Radix Saposhnikoviae（RS）	MOL000359	sitosterol	Ig gamma-2 chain C region	P01859	IGHG2	0.042
Radix Saposhnikoviae（RS）	MOL000359	sitosterol	Platelet glycoprotein IX	P14770	GP9	0.054
Radix Saposhnikoviae（RS）	MOL000359	sitosterol	Phospholipase A2	P04054	PLA2G1B	0.057
Radix Saposhnikoviae（RS）	MOL000359	sitosterol	Phospholipase A2, membrane associated	P14555	PLA2G2A	0.057
Radix Saposhnikoviae（RS）	MOL000359	sitosterol	Annexin A1	P04083	ANXA1	0.062
Radix Saposhnikoviae（RS）	MOL000359	sitosterol	Nuclear receptor 0B1	P51843	NR0B1	0.062
Radix Saposhnikoviae（RS）	MOL000359	sitosterol	3 beta-hydroxysteroid dehydrogenase/Delta 5-->4-isomerase type II	P26439	HSD3B2	0.064
Radix Saposhnikoviae（RS）	MOL000359	sitosterol	Cytosolic phospholipase A2	P47712	PLA2G4A	0.066
Radix Saposhnikoviae（RS）	MOL000359	sitosterol	Corticosteroid 11-beta-dehydrogenase isozyme 1	P28845	HSD11B1	0.067
Radix Saposhnikoviae（RS）	MOL000359	sitosterol	3-oxo-5-alpha-steroid 4-dehydrogenase 2	P31213	SRD5A2	0.067
Radix Saposhnikoviae（RS）	MOL000359	sitosterol	Microtubule-associated protein 2	P11137	MAP2	0.068
Radix Saposhnikoviae（RS）	MOL000359	sitosterol	Prolactin receptor	P16471	PRLR	0.068
Radix Saposhnikoviae（RS）	MOL000359	sitosterol	Gonadotropin-releasing hormone receptor	P30968	GNRHR	0.068
Radix Saposhnikoviae（RS）	MOL000359	sitosterol	Microtubule-associated protein 1A	P78559	MAP1A	0.068
Radix Saposhnikoviae（RS）	MOL000359	sitosterol	Gonadotropin-releasing hormone II receptor	Q96P88	GNRHR2	0.068
Radix Saposhnikoviae（RS）	MOL000359	sitosterol	Nuclear receptor coactivator 1	Q15788	NCOA1	0.069
Radix Saposhnikoviae（RS）	MOL000359	sitosterol	Bile salt sulfotransferase	Q06520	SULT2A1	0.091
Radix Saposhnikoviae（RS）	MOL000359	sitosterol	Prostaglandin G/H synthase 2	P35354	PTGS2	0.115
Radix Saposhnikoviae（RS）	MOL000359	sitosterol	DNA polymerase kappa	Q9UBT6	POLK	0.115
Radix Saposhnikoviae（RS）	MOL000359	sitosterol	Nuclear receptor coactivator 2	Q15596	NCOA2	0.116
Radix Saposhnikoviae（RS）	MOL000359	sitosterol	Aldo-keto reductase family 1 member C1	Q04828	AKR1C1	0.125
Radix Saposhnikoviae（RS）	MOL000359	sitosterol	ATP-binding cassette transporter sub-family C member 8	Q09428	ABCC8	0.127
Radix Saposhnikoviae（RS）	MOL000359	sitosterol	3 beta-hydroxysteroid dehydrogenase/Delta 5-->4-isomerase type I	P14060	HSD3B1	0.128
Radix Saposhnikoviae（RS）	MOL000359	sitosterol	Nitric-oxide synthase, endothelial	P29474	NOS3	0.134
Radix Saposhnikoviae（RS）	MOL000359	sitosterol	Cannabinoid receptor 2	P34972	CNR2	0.136
Radix Saposhnikoviae（RS）	MOL000359	sitosterol	3-oxo-5-alpha-steroid 4-dehydrogenase 1	P18405	SRD5A1	0.145
Radix Saposhnikoviae（RS）	MOL000359	sitosterol	Nuclear receptor coactivator 5	Q9HCD5	NCOA5	0.15
Radix Saposhnikoviae（RS）	MOL000359	sitosterol	Estradiol 17-beta-dehydrogenase 1	P14061	HSD17B1	0.221
Radix Saposhnikoviae（RS）	MOL000359	sitosterol	Androgen receptor	P10275	AR	0.224

Radix Saposhnikoviae（RS）	MOL000359	sitosterol	Estrogen receptor beta	Q92731	ESR2	0.246
Radix Saposhnikoviae（RS）	MOL000359	sitosterol	Glucocorticoid receptor	P04150	NR3C1	0.288
Radix Saposhnikoviae（RS）	MOL000359	sitosterol	Mineralocorticoid receptor	P08235	NR3C2	0.376
Radix Saposhnikoviae（RS）	MOL000359	sitosterol	Estrogen receptor	P03372	ESR1	0.93
Radix Saposhnikoviae（RS）	MOL000359	sitosterol	Progesterone receptor	P06401	PGR	1
Radix Saposhnikoviae（RS）	MOL000666	hexanal	Tumor necrosis factor	P01375	TNF	Validated
Radix Saposhnikoviae（RS）	MOL000666	hexanal	Transcription factor AP-1	P05412	JUN	Validated
Radix Saposhnikoviae（RS）	MOL000666	hexanal	Tissue factor	P13726	F3	Validated
Radix Saposhnikoviae（RS）	MOL000666	hexanal	Platelet glycoprotein 4	P16671	CD36	Validated
Radix Saposhnikoviae（RS）	MOL000666	hexanal	Mitogen-activated protein kinase 10	P53779	MAPK10	Validated
Radix Saposhnikoviae（RS）	MOL000666	hexanal	Abl interactor 1	Q8IZP0	ABI1	Validated
Radix Saposhnikoviae（RS）	MOL000667	1-hexanol	Calponin-3	Q15417	CNN3	Validated
Radix Saposhnikoviae（RS）	MOL001494	Mandenol	GTPase KRas	P01116	KRAS	0.056
Radix Saposhnikoviae（RS）	MOL001494	Mandenol	Phosducin	P20941	PDC	0.056
Radix Saposhnikoviae（RS）	MOL001494	Mandenol	Protein farnesyltransferase/geranylgeranyltransferase type I alpha subunit	P49354	FNTA	0.056
Radix Saposhnikoviae（RS）	MOL001494	Mandenol	Protein farnesyltransferase subunit beta	P49356	FNTB	0.056
Radix Saposhnikoviae（RS）	MOL001494	Mandenol	Geranylgeranyl transferase type-2 subunit beta	P53611	RABGGTB	0.056
Radix Saposhnikoviae（RS）	MOL001494	Mandenol	Guanine nucleotide-binding protein G(I)/G(S)/G(T) subunit beta-1	P62873	GNB1	0.056
Radix Saposhnikoviae（RS）	MOL001494	Mandenol	Guanine nucleotide-binding protein G(T) subunit gamma-T1	P63211	GNGT1	0.056
Radix Saposhnikoviae（RS）	MOL001494	Mandenol	Geranylgeranyl transferase type-2 subunit alpha	Q92696	RABGGTA	0.056
Radix Saposhnikoviae（RS）	MOL001494	Mandenol	Lymphocyte antigen 96	Q9Y6Y9	LY96	0.097
Radix Saposhnikoviae（RS）	MOL001494	Mandenol	SEC14-like protein 2	O76054	SEC14L2	0.108
Radix Saposhnikoviae（RS）	MOL001494	Mandenol	Alpha-lactalbumin	P00709	LALBA	0.108
Radix Saposhnikoviae（RS）	MOL001494	Mandenol	Myelin P2 protein	P02689	PMP2	0.108
Radix Saposhnikoviae（RS）	MOL001494	Mandenol	Glycodelin	P09466	PAEP	0.108
Radix Saposhnikoviae（RS）	MOL001494	Mandenol	Cytochrome P450 2C8	P10632	CYP2C8	0.108
Radix Saposhnikoviae（RS）	MOL001494	Mandenol	Hepatocyte nuclear factor 4-gamma	Q14541	HNF4G	0.108
Radix Saposhnikoviae（RS）	MOL001494	Mandenol	Trafficking protein particle complex subunit 3	O43617	TRAPPC3	0.201
Radix Saposhnikoviae（RS）	MOL001494	Mandenol	Furin	P09958	FURIN	0.283
Radix Saposhnikoviae（RS）	MOL001494	Mandenol	Glycolipid transfer protein	Q9NZD2	GLTP	0.283
Radix Saposhnikoviae（RS）	MOL001494	Mandenol	Rhodopsin	P08100	RHO	0.32
Radix Saposhnikoviae（RS）	MOL001494	Mandenol	Progesterone receptor	P06401	PGR	0.447
Radix Saposhnikoviae（RS）	MOL001494	Mandenol	Mineralocorticoid receptor	P08235	NR3C2	0.447
Radix Saposhnikoviae（RS）	MOL001494	Mandenol	Phospholipase A2	P04054	PLA2G1B	0.563
Radix Saposhnikoviae（RS）	MOL001494	Mandenol	Phospholipase A2, membrane associated	P14555	PLA2G2A	0.563
Radix Saposhnikoviae（RS）	MOL001494	Mandenol	Hemoglobin subunit alpha	P69905	HBA1	1
Radix Saposhnikoviae（RS）	MOL001941	Ammidin	Acetylcholine receptor subunit alpha	P02708	CHRNA1	0.011
Radix Saposhnikoviae（RS）	MOL001941	Ammidin	Cholinesterase	P06276	BCHE	0.011
Radix Saposhnikoviae（RS）	MOL001941	Ammidin	Acetylcholine receptor subunit gamma	P07510	CHRNG	0.011
Radix Saposhnikoviae（RS）	MOL001941	Ammidin	Acetylcholine receptor subunit beta	P11230	CHRNB1	0.011
Radix Saposhnikoviae（RS）	MOL001941	Ammidin	Neuronal acetylcholine receptor subunit beta-2	P17787	CHRNB2	0.011
Radix Saposhnikoviae（RS）	MOL001941	Ammidin	Neuronal acetylcholine receptor subunit alpha-5	P30532	CHRNA5	0.011
Radix Saposhnikoviae（RS）	MOL001941	Ammidin	Neuronal acetylcholine receptor subunit beta-4	P30926	CHRNB4	0.011
Radix Saposhnikoviae（RS）	MOL001941	Ammidin	Acetylcholine receptor subunit epsilon	Q04844	CHRNE	0.011
Radix Saposhnikoviae（RS）	MOL001941	Ammidin	Neuronal acetylcholine receptor subunit beta-3	Q05901	CHRNB3	0.011
Radix Saposhnikoviae（RS）	MOL001941	Ammidin	Acetylcholine receptor subunit delta	Q07001	CHRND	0.011

Radix Saposhnikoviae（RS）	MOL001941	Ammidin	Neuronal acetylcholine receptor subunit alpha-6	Q15825	KCNJ8	0.011
Radix Saposhnikoviae（RS）	MOL001941	Ammidin	Neuronal acetylcholine receptor subunit alpha-10	Q9GZZ6	CHRNA10	0.011
Radix Saposhnikoviae（RS）	MOL001941	Ammidin	Neuronal acetylcholine receptor subunit alpha-9	Q9UGM1	CHRNA9	0.011
Radix Saposhnikoviae（RS）	MOL001941	Ammidin	Sodium channel protein type 4 subunit alpha	P35499	SCN4A	0.013
Radix Saposhnikoviae（RS）	MOL001941	Ammidin	Actin-related protein 2/3 complex subunit 1B	O15143	ARPC1B	0.014
Radix Saposhnikoviae（RS）	MOL001941	Ammidin	Actin-related protein 2/3 complex subunit 2	O15144	ARPC2	0.014
Radix Saposhnikoviae（RS）	MOL001941	Ammidin	Actin-related protein 2/3 complex subunit 3	O15145	ARPC3	0.014
Radix Saposhnikoviae（RS）	MOL001941	Ammidin	Actin-related protein 2/3 complex subunit 4	P59998	ARPC4	0.014
Radix Saposhnikoviae（RS）	MOL001941	Ammidin	Actin-related protein 3	P61158	ACTR3	0.014
Radix Saposhnikoviae（RS）	MOL001941	Ammidin	Actin-related protein 2	P61160	ACTR2	0.014
Radix Saposhnikoviae（RS）	MOL001941	Ammidin	2,4-dienoyl-CoA reductase, mitochondrial	Q16698	DECR1	0.015
Radix Saposhnikoviae（RS）	MOL001941	Ammidin	Elongation factor 2	P13639	EEF2	0.02
Radix Saposhnikoviae（RS）	MOL001941	Ammidin	5-hydroxytryptamine 1F receptor	P30939	HTR1F	0.02
Radix Saposhnikoviae（RS）	MOL001941	Ammidin	Opioid receptor, sigma 1	Q5T1J1	SIGMAR1	0.02
Radix Saposhnikoviae（RS）	MOL001941	Ammidin	Sigma 1-type opioid receptor	Q99720	SIGMAR1	0.02
Radix Saposhnikoviae（RS）	MOL001941	Ammidin	Poly [ADP-ribose] polymerase 3	Q9Y6F1	PARP3	0.02
Radix Saposhnikoviae（RS）	MOL001941	Ammidin	Neuronal acetylcholine receptor subunit alpha-3	P32297	CHRNA3	0.021
Radix Saposhnikoviae（RS）	MOL001941	Ammidin	Solute carrier family 12 member 2	P55011	SLC12A2	0.021
Radix Saposhnikoviae（RS）	MOL001941	Ammidin	Solute carrier family 12 member 5	Q9H2X9	SLC12A5	0.021
Radix Saposhnikoviae（RS）	MOL001941	Ammidin	Solute carrier family 12 member 4	Q9UP95	SLC12A4	0.021
Radix Saposhnikoviae（RS）	MOL001941	Ammidin	Potassium channel subfamily K member 6	Q9Y257	KCNK6	0.021
Radix Saposhnikoviae（RS）	MOL001941	Ammidin	Carbonic anhydrase 12	O43570	CA12	0.022
Radix Saposhnikoviae（RS）	MOL001941	Ammidin	Beta-nerve growth factor	P01138	NGF	0.022
Radix Saposhnikoviae（RS）	MOL001941	Ammidin	Muscarinic acetylcholine receptor M5	P08912	CHRM5	0.022
Radix Saposhnikoviae（RS）	MOL001941	Ammidin	Calcium-activated potassium channel subunit alpha 1	Q12791	KCNMA1	0.022
Radix Saposhnikoviae（RS）	MOL001941	Ammidin	ATP-sensitive inward rectifier potassium channel 11	Q14654	KCNJ11	0.022
Radix Saposhnikoviae（RS）	MOL001941	Ammidin	Carbonic anhydrase 9	Q16790	CA9	0.022
Radix Saposhnikoviae（RS）	MOL001941	Ammidin	5-hydroxytryptamine 6 receptor	P50406	HTR6	0.023
Radix Saposhnikoviae（RS）	MOL001941	Ammidin	Glutamate receptor, ionotropic kainate 2	Q13002	GRIK2	0.023
Radix Saposhnikoviae（RS）	MOL001941	Ammidin	cGMP-dependent 3',5'-cyclic phosphodiesterase	O00408	PDE2A	0.024
Radix Saposhnikoviae（RS）	MOL001941	Ammidin	High-affinity cGMP-specific 3',5'-cyclic phosphodiesterase 9A	O76083	PDE9A	0.024
Radix Saposhnikoviae（RS）	MOL001941	Ammidin	6-phosphogluconate dehydrogenase, decarboxylating	P52209	PGD	0.024
Radix Saposhnikoviae（RS）	MOL001941	Ammidin	cGMP-inhibited 3',5'-cyclic phosphodiesterase B	Q13370	PDE3B	0.024
Radix Saposhnikoviae（RS）	MOL001941	Ammidin	cAMP-specific 3',5'-cyclic phosphodiesterase 7B	Q9NP56	PDE7B	0.024
Radix Saposhnikoviae（RS）	MOL001941	Ammidin	Acetylcholinesterase	P22303	ACHE	0.025
Radix Saposhnikoviae（RS）	MOL001941	Ammidin	Adenosine A2b receptor	P29275	ADORA2B	0.025
Radix Saposhnikoviae（RS）	MOL001941	Ammidin	Troponin C, slow skeletal and cardiac muscles	P63316	TNNC1	0.025
Radix Saposhnikoviae（RS）	MOL001941	Ammidin	Potassium voltage-gated channel subfamily D member 2	Q9NZV8	KCND2	0.025
Radix Saposhnikoviae（RS）	MOL001941	Ammidin	Glutamate [NMDA] receptor subunit zeta-1	Q05586	GRIN1	0.026
Radix Saposhnikoviae（RS）	MOL001941	Ammidin	Glutamate [NMDA] receptor subunit epsilon-1	Q12879	GRIN2A	0.026
Radix Saposhnikoviae（RS）	MOL001941	Ammidin	Glutamate [NMDA] receptor subunit epsilon-2	Q13224	GRIN2B	0.026
Radix Saposhnikoviae（RS）	MOL001941	Ammidin	Glutamate [NMDA] receptor subunit epsilon-3	Q14957	GRIN2C	0.026
Radix Saposhnikoviae（RS）	MOL001941	Ammidin	Interleukin-3	P08700	IL3	0.027
Radix Saposhnikoviae（RS）	MOL001941	Ammidin	Protein S100-A12	P80511	S100A12	0.027
Radix Saposhnikoviae（RS）	MOL001941	Ammidin	Cyclin-dependent kinase 5 activator 1	Q15078	CDK5R1	0.027
Radix Saposhnikoviae（RS）	MOL001941	Ammidin	Protein S100-A13	Q99584	S100A13	0.027
Radix Saposhnikoviae（RS）	MOL001941	Ammidin	Sodium channel protein type 11 subunit alpha	Q9UI33	SCN11A	0.028

Radix Saposhnikoviae（RS）	MOL001941	Ammidin	5-hydroxytryptamine 3 receptor	P46098	HTR3A	0.029
Radix Saposhnikoviae（RS）	MOL001941	Ammidin	5-hydroxytryptamine 4 receptor	Q13639	HTR4	0.029
Radix Saposhnikoviae（RS）	MOL001941	Ammidin	T-cell receptor alpha chain C region	P01848	TRAC	0.033
Radix Saposhnikoviae（RS）	MOL001941	Ammidin	T-cell receptor beta chain C region	P01850	TRBC1	0.033
Radix Saposhnikoviae（RS）	MOL001941	Ammidin	Beta-2-microglobulin	P61769	B2M	0.033
Radix Saposhnikoviae（RS）	MOL001941	Ammidin	3-phosphoinositide-dependent protein kinase 1	O15530	PDPK1	0.034
Radix Saposhnikoviae（RS）	MOL001941	Ammidin	Proto-oncogene tyrosine-protein kinase LCK	P06239	LCK	0.034
Radix Saposhnikoviae（RS）	MOL001941	Ammidin	Proto-oncogene serine/threonine-protein kinase Pim-1	P11309	PIM1	0.034
Radix Saposhnikoviae（RS）	MOL001941	Ammidin	Tyrosine-protein kinase CSK	P41240	CSK	0.034
Radix Saposhnikoviae（RS）	MOL001941	Ammidin	Tyrosine-protein kinase ZAP-70	P43403	ZAP70	0.034
Radix Saposhnikoviae（RS）	MOL001941	Ammidin	Tyrosine-protein kinase SYK	P43405	SYK	0.034
Radix Saposhnikoviae（RS）	MOL001941	Ammidin	Neuronal acetylcholine receptor subunit alpha-4	P43681	CHRNA4	0.034
Radix Saposhnikoviae（RS）	MOL001941	Ammidin	Protein kinase C theta type	Q04759	PRKCQ	0.034
Radix Saposhnikoviae（RS）	MOL001941	Ammidin	Tyrosine-protein kinase ITK/TSK	Q08881	ITK	0.034
Radix Saposhnikoviae（RS）	MOL001941	Ammidin	Prostaglandin reductase 1	Q14914	PTGR1	0.034
Radix Saposhnikoviae（RS）	MOL001941	Ammidin	Neuronal acetylcholine receptor subunit alpha-2	Q15822	CHRNA2	0.034
Radix Saposhnikoviae（RS）	MOL001941	Ammidin	Tripartite motif-containing protein 13	O60858	TRIM13	0.035
Radix Saposhnikoviae（RS）	MOL001941	Ammidin	cAMP response element-binding protein	P16220	CREB1	0.035
Radix Saposhnikoviae（RS）	MOL001941	Ammidin	Tyrosine-protein kinase JAK2	O60674	JAK2	0.037
Radix Saposhnikoviae（RS）	MOL001941	Ammidin	Tyrosine-protein kinase JAK1	P23458	JAK1	0.037
Radix Saposhnikoviae（RS）	MOL001941	Ammidin	Tyrosine-protein kinase JAK3	P52333	JAK3	0.037
Radix Saposhnikoviae（RS）	MOL001941	Ammidin	Potassium channel subfamily K member 1	O00180	KCNK1	0.038
Radix Saposhnikoviae（RS）	MOL001941	Ammidin	Tyrosine 3-monooxygenase	P07101	TH	0.038
Radix Saposhnikoviae（RS）	MOL001941	Ammidin	Peptidyl-prolyl cis-trans isomerase NIMA-interacting 1	Q13526	PIN1	0.038
Radix Saposhnikoviae（RS）	MOL001941	Ammidin	Tyrosyl-tRNA synthetase, mitochondrial	Q9Y2Z4	YARS2	0.038
Radix Saposhnikoviae（RS）	MOL001941	Ammidin	Sterol O-acyltransferase 2	O75908	SOAT2	0.04
Radix Saposhnikoviae（RS）	MOL001941	Ammidin	Sterol O-acyltransferase 1	P35610	SOAT1	0.04
Radix Saposhnikoviae（RS）	MOL001941	Ammidin	85 kDa calcium-independent phospholipase A2	O60733	PLA2G6	0.041
Radix Saposhnikoviae（RS）	MOL001941	Ammidin	Prothrombin	P00734	F2	0.041
Radix Saposhnikoviae（RS）	MOL001941	Ammidin	Retinoic acid receptor RXR-alpha	P19793	RXRA	0.041
Radix Saposhnikoviae（RS）	MOL001941	Ammidin	DNA-(apurinic or apyrimidinic site) lyase	P27695	APEX1	0.041
Radix Saposhnikoviae（RS）	MOL001941	Ammidin	Retinoic acid receptor RXR-beta	P28702	RXRB	0.041
Radix Saposhnikoviae（RS）	MOL001941	Ammidin	Cytosolic phospholipase A2	P47712	PLA2G4A	0.041
Radix Saposhnikoviae（RS）	MOL001941	Ammidin	Peptidyl-prolyl cis-trans isomerase, mitochondrial	P30405	PPIF	0.042
Radix Saposhnikoviae（RS）	MOL001941	Ammidin	Lipoic acid synthetase, mitochondrial	O43766	LIAS	0.043
Radix Saposhnikoviae（RS）	MOL001941	Ammidin	Myeloperoxidase	P05164	MPO	0.043
Radix Saposhnikoviae（RS）	MOL001941	Ammidin	Eosinophil peroxidase	P11678	EPX	0.043
Radix Saposhnikoviae（RS）	MOL001941	Ammidin	Farnesyl pyrophosphate synthetase	P14324	FDPS	0.043
Radix Saposhnikoviae（RS）	MOL001941	Ammidin	Calreticulin	P27797	CALR	0.043
Radix Saposhnikoviae（RS）	MOL001941	Ammidin	Melatonin receptor type 1A	P48039	MTNR1A	0.043
Radix Saposhnikoviae（RS）	MOL001941	Ammidin	Melatonin receptor type 1B	P49286	MTNR1B	0.043
Radix Saposhnikoviae（RS）	MOL001941	Ammidin	S-phase kinase-associated protein 1	P63208	SKP1	0.043
Radix Saposhnikoviae（RS）	MOL001941	Ammidin	Isopentenyl-diphosphate Delta-isomerase 1	Q13907	IDI1	0.043
Radix Saposhnikoviae（RS）	MOL001941	Ammidin	Nuclear receptor ROR-beta	Q92753	RORB	0.043
Radix Saposhnikoviae（RS）	MOL001941	Ammidin	Histone deacetylase 8	Q9BY41	HDAC8	0.043
Radix Saposhnikoviae（RS）	MOL001941	Ammidin	Group IIE secretory phospholipase A2	Q9NZK7	PLA2G2E	0.043
Radix Saposhnikoviae（RS）	MOL001941	Ammidin	Lipoyltransferase 1, mitochondrial	Q9Y234	LIPT1	0.043

Radix Saposhnikoviae（RS）	MOL001941	Ammidin	Tumor necrosis factor	P01375	TNF	0.045
Radix Saposhnikoviae（RS）	MOL001941	Ammidin	Glutathione S-transferase A2	P09210	GSTA2	0.045
Radix Saposhnikoviae（RS）	MOL001941	Ammidin	Keratin, type II cytoskeletal 7	P08729	KRT7	0.046
Radix Saposhnikoviae（RS）	MOL001941	Ammidin	Platelet glycoprotein IX	P14770	GP9	0.046
Radix Saposhnikoviae（RS）	MOL001941	Ammidin	Solute carrier family 12 member 1	Q13621	SLC12A1	0.046
Radix Saposhnikoviae（RS）	MOL001941	Ammidin	Sodium/potassium-transporting ATPase alpha-1 chain	P05023	ATP1A1	0.047
Radix Saposhnikoviae（RS）	MOL001941	Ammidin	Beta-3 adrenergic receptor	P13945	ADRB3	0.047
Radix Saposhnikoviae（RS）	MOL001941	Ammidin	Alpha-1D adrenergic receptor	P25100	ADRA1D	0.047
Radix Saposhnikoviae（RS）	MOL001941	Ammidin	Neuronal acetylcholine receptor subunit alpha-7	P36544	CHRNA7	0.048
Radix Saposhnikoviae（RS）	MOL001941	Ammidin	Methionine aminopeptidase 1	P53582	METAP1	0.048
Radix Saposhnikoviae（RS）	MOL001941	Ammidin	Casein kinase II subunit alpha	P68400	CSNK2A1	0.048
Radix Saposhnikoviae（RS）	MOL001941	Ammidin	D1 dopamine receptor-interacting protein calcyon	Q9NYX4	CALY	0.049
Radix Saposhnikoviae（RS）	MOL001941	Ammidin	Mitogen-activated protein kinase 8	P45983	MAPK8	0.051
Radix Saposhnikoviae（RS）	MOL001941	Ammidin	Mitogen-activated protein kinase 10	P53779	MAPK10	0.051
Radix Saposhnikoviae（RS）	MOL001941	Ammidin	High affinity cAMP-specific and IBMX-insensitive 3',5'-cyclic phosphodiesterase 8A	O60658	PDE8A	0.052
Radix Saposhnikoviae（RS）	MOL001941	Ammidin	cGMP-specific 3',5'-cyclic phosphodiesterase	O76074	PDE5A	0.052
Radix Saposhnikoviae（RS）	MOL001941	Ammidin	Phospholipase A2, membrane associated	P14555	PLA2G2A	0.052
Radix Saposhnikoviae（RS）	MOL001941	Ammidin	Aldose reductase	P15121	AKR1B1	0.052
Radix Saposhnikoviae（RS）	MOL001941	Ammidin	Mitogen-activated protein kinase 3	P27361	MAPK3	0.052
Radix Saposhnikoviae（RS）	MOL001941	Ammidin	Peroxisome proliferator-activated receptor gamma	P37231	PPARG	0.052
Radix Saposhnikoviae（RS）	MOL001941	Ammidin	Aldo-keto reductase family 1 member C3	P42330	AKR1C3	0.052
Radix Saposhnikoviae（RS）	MOL001941	Ammidin	Peroxisome proliferator-activated receptor delta	Q03181	PPARD	0.052
Radix Saposhnikoviae（RS）	MOL001941	Ammidin	Lactoylglutathione lyase	Q04760	GLO1	0.052
Radix Saposhnikoviae（RS）	MOL001941	Ammidin	Prostaglandin reductase 2	Q8N8N7	PTGR2	0.052
Radix Saposhnikoviae（RS）	MOL001941	Ammidin	RAC-beta serine/threonine-protein kinase	P31751	AKT2	0.053
Radix Saposhnikoviae（RS）	MOL001941	Ammidin	cGMP-inhibited 3',5'-cyclic phosphodiesterase A	Q14432	PDE3A	0.053
Radix Saposhnikoviae（RS）	MOL001941	Ammidin	Glutamate [NMDA] receptor subunit epsilon-4	O15399	GRIN2D	0.056
Radix Saposhnikoviae（RS）	MOL001941	Ammidin	Glutamate [NMDA] receptor subunit 3B	O60391	GRIN3B	0.056
Radix Saposhnikoviae（RS）	MOL001941	Ammidin	D-HSCDK2	O75100	CA11	0.056
Radix Saposhnikoviae（RS）	MOL001941	Ammidin	Cell division control protein 2 homolog	P06493	CDK1	0.056
Radix Saposhnikoviae（RS）	MOL001941	Ammidin	Translocator protein	P30536	TSPO	0.056
Radix Saposhnikoviae（RS）	MOL001941	Ammidin	Cell division protein kinase 5	Q00535	CDK5	0.056
Radix Saposhnikoviae（RS）	MOL001941	Ammidin	Tubulin beta-2C chain	P68371	TUBB4B	0.057
Radix Saposhnikoviae（RS）	MOL001941	Ammidin	Sodium channel protein type 10 subunit alpha	Q9Y5Y9	SCN10A	0.058
Radix Saposhnikoviae（RS）	MOL001941	Ammidin	Nitric-oxide synthase, brain	P29475	NOS1	0.061
Radix Saposhnikoviae（RS）	MOL001941	Ammidin	Platelet-activating factor acetylhydrolase IB subunit gamma	Q15102	PAFAH1B3	0.061
Radix Saposhnikoviae（RS）	MOL001941	Ammidin	Potassium voltage-gated channel subfamily H member 2	Q12809	KCNH2	0.064
Radix Saposhnikoviae（RS）	MOL001941	Ammidin	Tryptophanyl-tRNA synthetase, mitochondrial	Q9UGM6	WARS2	0.068
Radix Saposhnikoviae（RS）	MOL001941	Ammidin	5-hydroxytryptamine 1D receptor	P28221	HTR1D	0.069
Radix Saposhnikoviae（RS）	MOL001941	Ammidin	Alpha-2A adrenergic receptor	P08913	ADRA2A	0.071
Radix Saposhnikoviae（RS）	MOL001941	Ammidin	D(4) dopamine receptor	P21917	DRD4	0.071
Radix Saposhnikoviae（RS）	MOL001941	Ammidin	D(1B) dopamine receptor	P21918	DRD5	0.071
Radix Saposhnikoviae（RS）	MOL001941	Ammidin	D(3) dopamine receptor	P35462	DRD3	0.071
Radix Saposhnikoviae（RS）	MOL001941	Ammidin	Calmodulin	P62158		0.071
Radix Saposhnikoviae（RS）	MOL001941	Ammidin	Carbonic anhydrase 4	P22748	CA4	0.072
Radix Saposhnikoviae（RS）	MOL001941	Ammidin	Gamma-aminobutyric-acid receptor subunit beta-3	P28472	GABRB3	0.072
Radix Saposhnikoviae（RS）	MOL001941	Ammidin	5-hydroxytryptamine 2B receptor	P41595	HTR2B	0.072

Radix Saposhnikoviae（RS）	MOL001941	Ammidin	MAP kinase-activated protein kinase 2	P49137	MAPKAPK2	0.072
Radix Saposhnikoviae（RS）	MOL001941	Ammidin	Beta-2 adrenergic receptor	P07550	ADRB2	0.073
Radix Saposhnikoviae（RS）	MOL001941	Ammidin	Beta-1 adrenergic receptor	P08588	ADRB1	0.073
Radix Saposhnikoviae（RS）	MOL001941	Ammidin	Gamma-aminobutyric acid receptor subunit theta	Q9UN88	GABRQ	0.073
Radix Saposhnikoviae（RS）	MOL001941	Ammidin	Death-associated protein kinase 3	O43293	DAPK3	0.077
Radix Saposhnikoviae（RS）	MOL001941	Ammidin	Muscarinic acetylcholine receptor M4	P08173	CHRM4	0.079
Radix Saposhnikoviae（RS）	MOL001941	Ammidin	Tyrosyl-tRNA synthetase, cytoplasmic	P54577	YARS	0.079
Radix Saposhnikoviae（RS）	MOL001941	Ammidin	cAMP-specific 3',5'-cyclic phosphodiesterase 4C	Q08493	PDE4C	0.08
Radix Saposhnikoviae（RS）	MOL001941	Ammidin	High-affinity cAMP-specific 3',5'-cyclic phosphodiesterase 7A	Q13946	PDE7A	0.08
Radix Saposhnikoviae（RS）	MOL001941	Ammidin	3-oxo-5-alpha-steroid 4-dehydrogenase 2	P31213	SRD5A2	0.083
Radix Saposhnikoviae（RS）	MOL001941	Ammidin	Calcium/calmodulin-dependent protein kinase type II alpha chain	Q9UQM7	CAMK2A	0.083
Radix Saposhnikoviae（RS）	MOL001941	Ammidin	Cannabinoid receptor 1	P21554	CNR1	0.084
Radix Saposhnikoviae（RS）	MOL001941	Ammidin	DNA topoisomerase 2-alpha	P11388	TOP2A	0.085
Radix Saposhnikoviae（RS）	MOL001941	Ammidin	Potassium channel subfamily K member 3	O14649	KCNK3	0.087
Radix Saposhnikoviae（RS）	MOL001941	Ammidin	Poly [ADP-ribose] polymerase 1	P09874	PARP1	0.087
Radix Saposhnikoviae（RS）	MOL001941	Ammidin	Proto-oncogene tyrosine-protein kinase Src	P12931	SRC	0.087
Radix Saposhnikoviae（RS）	MOL001941	Ammidin	Oxysterols receptor LXR-alpha	Q13133	NR1H3	0.088
Radix Saposhnikoviae（RS）	MOL001941	Ammidin	Gamma-aminobutyric-acid receptor subunit beta-2	P47870	GABRB2	0.089
Radix Saposhnikoviae（RS）	MOL001941	Ammidin	Ig kappa chain V-II region RPMI 6410	P06310	IGKV2-30	0.09
Radix Saposhnikoviae（RS）	MOL001941	Ammidin	Phosphatidylinositol-4,5-bisphosphate 3-kinase catalytic subunit gamma isoform	P48736	PIK3CG	0.09
Radix Saposhnikoviae（RS）	MOL001941	Ammidin	Dual specificity protein kinase CLK1	P49759	CLK1	0.09
Radix Saposhnikoviae（RS）	MOL001941	Ammidin	Potassium voltage-gated channel subfamily KQT member 1	P51787	KCNQ1	0.09
Radix Saposhnikoviae（RS）	MOL001941	Ammidin	Tyrosine-protein phosphatase non-receptor type 1	P18031	PTPN1	0.091
Radix Saposhnikoviae（RS）	MOL001941	Ammidin	Peroxisome proliferator-activated receptor alpha	Q07869	PPARA	0.091
Radix Saposhnikoviae（RS）	MOL001941	Ammidin	Gamma-aminobutyric-acid receptor subunit alpha-6	Q16445	GABRA6	0.091
Radix Saposhnikoviae（RS）	MOL001941	Ammidin	5-hydroxytryptamine 1B receptor	P28222	HTR1B	0.092
Radix Saposhnikoviae（RS）	MOL001941	Ammidin	Egl nine homolog 1	Q9GZT9	EGLN1	0.092
Radix Saposhnikoviae（RS）	MOL001941	Ammidin	Hepatocyte growth factor receptor	P08581	MET	0.093
Radix Saposhnikoviae（RS）	MOL001941	Ammidin	Sodium-dependent dopamine transporter	Q01959	SLC6A3	0.093
Radix Saposhnikoviae（RS）	MOL001941	Ammidin	Ribosyldihydronicotinamide dehydrogenase [quinone]	P16083	NQO2	0.094
Radix Saposhnikoviae（RS）	MOL001941	Ammidin	Serine/threonine-protein kinase 6	O14965	AURKA	0.095
Radix Saposhnikoviae（RS）	MOL001941	Ammidin	Alpha-2B adrenergic receptor	P18089	ADRA2B	0.095
Radix Saposhnikoviae（RS）	MOL001941	Ammidin	Alpha-2C adrenergic receptor	P18825	ADRA2C	0.095
Radix Saposhnikoviae（RS）	MOL001941	Ammidin	Carbonic anhydrase 1	P00915	CA1	0.097
Radix Saposhnikoviae（RS）	MOL001941	Ammidin	Ig gamma-1 chain C region	P01857	IGHG1	0.097
Radix Saposhnikoviae（RS）	MOL001941	Ammidin	5-hydroxytryptamine 2C receptor	P28335	HTR2C	0.097
Radix Saposhnikoviae（RS）	MOL001941	Ammidin	Sodium-dependent serotonin transporter	P31645	SLC6A4	0.097
Radix Saposhnikoviae（RS）	MOL001941	Ammidin	Cytochrome P450 11B1, mitochondrial	P15538	CYP11B1	0.098
Radix Saposhnikoviae（RS）	MOL001941	Ammidin	RAC-alpha serine/threonine-protein kinase	P31749	AKT1	0.098
Radix Saposhnikoviae（RS）	MOL001941	Ammidin	Alpha-1B adrenergic receptor	P35368	ADRA1B	0.098
Radix Saposhnikoviae（RS）	MOL001941	Ammidin	Glycogen synthase kinase-3 beta	P49841	GSK3B	0.098
Radix Saposhnikoviae（RS）	MOL001941	Ammidin	DNA polymerase kappa	Q9UBT6	POLK	0.098
Radix Saposhnikoviae（RS）	MOL001941	Ammidin	Gamma-aminobutyric-acid receptor subunit alpha-4	P48169	GABRA4	0.099
Radix Saposhnikoviae（RS）	MOL001941	Ammidin	Triosephosphate isomerase	P60174	TPI1	0.099
Radix Saposhnikoviae（RS）	MOL001941	Ammidin	Amine oxidase [flavin-containing] B	P27338	MAOB	0.103
Radix Saposhnikoviae（RS）	MOL001941	Ammidin	C-jun-amino-terminal kinase-interacting protein 1	Q9UQF2	MAPK8IP1	0.104
Radix Saposhnikoviae（RS）	MOL001941	Ammidin	Hydroxyacid oxidase 1	Q9UJM8	HAO1	0.105

Radix Saposhnikoviae（RS）	MOL001941	Ammidin	Muscarinic acetylcholine receptor M2	P08172	CHRM2	0.107
Radix Saposhnikoviae（RS）	MOL001941	Ammidin	cAMP-specific 3',5'-cyclic phosphodiesterase 4D	Q08499	PDE4D	0.107
Radix Saposhnikoviae（RS）	MOL001941	Ammidin	Glutamate [NMDA] receptor subunit 3A	Q8TCU5	GRIN3A	0.107
Radix Saposhnikoviae（RS）	MOL001941	Ammidin	Trypsin-1	P07477	PRSS1	0.108
Radix Saposhnikoviae（RS）	MOL001941	Ammidin	Toll-like receptor 7	Q9NYK1	TLR7	0.108
Radix Saposhnikoviae（RS）	MOL001941	Ammidin	cAMP-dependent protein kinase inhibitor alpha	P61925	PKIA	0.109
Radix Saposhnikoviae（RS）	MOL001941	Ammidin	Gamma-aminobutyric acid receptor subunit gamma-2	P18507	GABRG2	0.115
Radix Saposhnikoviae（RS）	MOL001941	Ammidin	Tubulin alpha-3 chain	Q71U36	TUBA1A	0.118
Radix Saposhnikoviae（RS）	MOL001941	Ammidin	Carbonic anhydrase 2	P00918	CA2	0.122
Radix Saposhnikoviae（RS）	MOL001941	Ammidin	Gamma-aminobutyric acid receptor subunit rho-3	A8MPY1	GABRR3	0.123
Radix Saposhnikoviae（RS）	MOL001941	Ammidin	Gamma-aminobutyric acid receptor subunit pi	O00591	GABRP	0.123
Radix Saposhnikoviae（RS）	MOL001941	Ammidin	Gamma-aminobutyric-acid receptor subunit rho-1	P24046	GABRR1	0.123
Radix Saposhnikoviae（RS）	MOL001941	Ammidin	Gamma-aminobutyric acid receptor subunit rho-2	P28476	GABRR2	0.123
Radix Saposhnikoviae（RS）	MOL001941	Ammidin	Gamma-aminobutyric acid receptor subunit epsilon	P78334	GABRE	0.123
Radix Saposhnikoviae（RS）	MOL001941	Ammidin	Gamma-aminobutyric acid receptor subunit gamma-1	Q8N1C3	GABRG1	0.123
Radix Saposhnikoviae（RS）	MOL001941	Ammidin	Gamma-aminobutyric acid receptor subunit gamma-3	Q99928	GABRG3	0.123
Radix Saposhnikoviae（RS）	MOL001941	Ammidin	Sodium-dependent noradrenaline transporter	P23975	SLC6A2	0.125
Radix Saposhnikoviae（RS）	MOL001941	Ammidin	Liver carboxylesterase 1	P23141	CES1	0.126
Radix Saposhnikoviae（RS）	MOL001941	Ammidin	Nitric-oxide synthase, endothelial	P29474	NOS3	0.126
Radix Saposhnikoviae（RS）	MOL001941	Ammidin	Alpha-1A adrenergic receptor	P35348	ADRA1A	0.126
Radix Saposhnikoviae（RS）	MOL001941	Ammidin	Gamma-aminobutyric acid receptor subunit delta	O14764	GABRD	0.132
Radix Saposhnikoviae（RS）	MOL001941	Ammidin	Gamma-aminobutyric-acid receptor subunit beta-1	P18505	GABRB1	0.132
Radix Saposhnikoviae（RS）	MOL001941	Ammidin	Adenosine A2a receptor	P29274	ADORA2A	0.136
Radix Saposhnikoviae（RS）	MOL001941	Ammidin	Adenosine A1 receptor	P30542	ADORA1	0.137
Radix Saposhnikoviae（RS）	MOL001941	Ammidin	D(1A) dopamine receptor	P21728	DRD1	0.147
Radix Saposhnikoviae（RS）	MOL001941	Ammidin	5-hydroxytryptamine 2A receptor	P28223	HTR2A	0.148
Radix Saposhnikoviae（RS）	MOL001941	Ammidin	Delta-type opioid receptor	P41143	OPRD1	0.151
Radix Saposhnikoviae（RS）	MOL001941	Ammidin	5-hydroxytryptamine 1A receptor	P08908	HTR1A	0.154
Radix Saposhnikoviae（RS）	MOL001941	Ammidin	Gamma-aminobutyric-acid receptor subunit alpha-5	P31644	GABRA5	0.158
Radix Saposhnikoviae（RS）	MOL001941	Ammidin	Gamma-aminobutyric-acid receptor subunit alpha-3	P34903	GABRA3	0.159
Radix Saposhnikoviae（RS）	MOL001941	Ammidin	Histamine H1 receptor	P35367	HRH1	0.162
Radix Saposhnikoviae（RS）	MOL001941	Ammidin	Muscarinic acetylcholine receptor M3	P20309	CHRM3	0.166
Radix Saposhnikoviae（RS）	MOL001941	Ammidin	Protein farnesyltransferase/geranylgeranyltransferase type I alpha subunit	P49354	FNTA	0.175
Radix Saposhnikoviae（RS）	MOL001941	Ammidin	Sodium channel protein type 5 subunit alpha	Q14524	SCN5A	0.183
Radix Saposhnikoviae（RS）	MOL001941	Ammidin	Gamma-aminobutyric-acid receptor subunit alpha-2	P47869	GABRA2	0.184
Radix Saposhnikoviae（RS）	MOL001941	Ammidin	Gamma-aminobutyric-acid receptor subunit alpha-1	P14867	GABRA1	0.193
Radix Saposhnikoviae（RS）	MOL001941	Ammidin	Cell division protein kinase 2	P24941	CDK2	0.205
Radix Saposhnikoviae（RS）	MOL001941	Ammidin	cAMP-specific 3',5'-cyclic phosphodiesterase 4A	P27815	PDE4A	0.22
Radix Saposhnikoviae（RS）	MOL001941	Ammidin	cAMP-specific 3',5'-cyclic phosphodiesterase 4B	Q07343	PDE4B	0.22
Radix Saposhnikoviae（RS）	MOL001941	Ammidin	D(2) dopamine receptor	P14416	DRD2	0.226
Radix Saposhnikoviae（RS）	MOL001941	Ammidin	Hemoglobin subunit alpha	P69905	HBA1	0.233
Radix Saposhnikoviae（RS）	MOL001941	Ammidin	Kappa-type opioid receptor	P41145	OPRK1	0.267
Radix Saposhnikoviae（RS）	MOL001941	Ammidin	Mitogen-activated protein kinase 14	Q16539	MAPK14	0.284
Radix Saposhnikoviae（RS）	MOL001941	Ammidin	Muscarinic acetylcholine receptor M1	P11229	CHRM1	0.289
Radix Saposhnikoviae（RS）	MOL001941	Ammidin	Nuclear receptor coactivator 1	Q15788	NCOA1	0.289
Radix Saposhnikoviae（RS）	MOL001941	Ammidin	cAMP-dependent protein kinase catalytic subunit alpha	P17612	PRKACA	0.448

Radix Saposhnikoviae（RS）	MOL001941	Ammidin	Mu-type opioid receptor	P35372	OPRM1	0.453
Radix Saposhnikoviae（RS）	MOL001941	Ammidin	Estrogen receptor beta	Q92731	ESR2	0.47
Radix Saposhnikoviae（RS）	MOL001941	Ammidin	Cyclin-A2	P20248	CCNA2	0.508
Radix Saposhnikoviae（RS）	MOL001941	Ammidin	Prostaglandin G/H synthase 1	P23219	PTGS1	0.543
Radix Saposhnikoviae（RS）	MOL001941	Ammidin	Estrogen receptor	P03372	ESR1	0.621
Radix Saposhnikoviae（RS）	MOL001941	Ammidin	Prostaglandin G/H synthase 2	P35354	PTGS2	1
Radix Saposhnikoviae（RS）	MOL001942	isoimperatorin	Cytochrome b-c1 complex subunit 8	O14949	UQCRQ	0.003
Radix Saposhnikoviae（RS）	MOL001942	isoimperatorin	Cytochrome b-c1 complex subunit 10	O14957	UQCR11	0.003
Radix Saposhnikoviae（RS）	MOL001942	isoimperatorin	Cytochrome b	P00156	MT-CYB	0.003
Radix Saposhnikoviae（RS）	MOL001942	isoimperatorin	Cytochrome c1, heme protein, mitochondrial	P08574	CYC1	0.003
Radix Saposhnikoviae（RS）	MOL001942	isoimperatorin	Cytochrome b-c1 complex subunit 7	P14927	UQCRB	0.003
Radix Saposhnikoviae（RS）	MOL001942	isoimperatorin	Cytochrome b-c1 complex subunit 2, mitochondrial	P22695	UQCRC2	0.003
Radix Saposhnikoviae（RS）	MOL001942	isoimperatorin	Ubiquinol-cytochrome-c reductase complex core protein 1, mitochondrial	P31930	UQCRC1	0.003
Radix Saposhnikoviae（RS）	MOL001942	isoimperatorin	Cytochrome b-c1 complex subunit Rieske, mitochondrial	P47985	UQCRFS1	0.003
Radix Saposhnikoviae（RS）	MOL001942	isoimperatorin	Cytochrome b-c1 complex subunit 9	Q9UDW1	UQCR10	0.003
Radix Saposhnikoviae（RS）	MOL001942	isoimperatorin	Kynurenine--oxoglutarate transaminase 1	Q16773	KYAT1	0.007
Radix Saposhnikoviae（RS）	MOL001942	isoimperatorin	Sodium channel protein type 1 subunit alpha	P35498	SCN1A	0.008
Radix Saposhnikoviae（RS）	MOL001942	isoimperatorin	Sodium channel protein type 4 subunit alpha	P35499	SCN4A	0.017
Radix Saposhnikoviae（RS）	MOL001942	isoimperatorin	Glutamate receptor, ionotropic kainate 2	Q13002	GRIK2	0.02
Radix Saposhnikoviae（RS）	MOL001942	isoimperatorin	Acetylcholine receptor subunit alpha	P02708	CHRNA1	0.022
Radix Saposhnikoviae（RS）	MOL001942	isoimperatorin	Cholinesterase	P06276	BCHE	0.022
Radix Saposhnikoviae（RS）	MOL001942	isoimperatorin	Acetylcholine receptor subunit gamma	P07510	CHRNG	0.022
Radix Saposhnikoviae（RS）	MOL001942	isoimperatorin	Acetylcholine receptor subunit beta	P11230	CHRNB1	0.022
Radix Saposhnikoviae（RS）	MOL001942	isoimperatorin	Neuronal acetylcholine receptor subunit beta-2	P17787	CHRNB2	0.022
Radix Saposhnikoviae（RS）	MOL001942	isoimperatorin	Neuronal acetylcholine receptor subunit alpha-5	P30532	CHRNA5	0.022
Radix Saposhnikoviae（RS）	MOL001942	isoimperatorin	Acetylcholine receptor subunit epsilon	Q04844	CHRNE	0.022
Radix Saposhnikoviae（RS）	MOL001942	isoimperatorin	Neuronal acetylcholine receptor subunit beta-3	Q05901	CHRNB3	0.022
Radix Saposhnikoviae（RS）	MOL001942	isoimperatorin	Acetylcholine receptor subunit delta	Q07001	CHRND	0.022
Radix Saposhnikoviae（RS）	MOL001942	isoimperatorin	Neuronal acetylcholine receptor subunit alpha-6	Q15825	KCNJ8	0.022
Radix Saposhnikoviae（RS）	MOL001942	isoimperatorin	Neuronal acetylcholine receptor subunit alpha-10	Q9GZZ6	CHRNA10	0.022
Radix Saposhnikoviae（RS）	MOL001942	isoimperatorin	Neuronal acetylcholine receptor subunit alpha-9	Q9UGM1	CHRNA9	0.022
Radix Saposhnikoviae（RS）	MOL001942	isoimperatorin	5-hydroxytryptamine 1F receptor	P30939	HTR1F	0.025
Radix Saposhnikoviae（RS）	MOL001942	isoimperatorin	Calmodulin	P62158		0.025
Radix Saposhnikoviae（RS）	MOL001942	isoimperatorin	Troponin C, slow skeletal and cardiac muscles	P63316	TNNC1	0.025
Radix Saposhnikoviae（RS）	MOL001942	isoimperatorin	5-hydroxytryptamine 1E receptor	P28566	HTR1E	0.027
Radix Saposhnikoviae（RS）	MOL001942	isoimperatorin	5-hydroxytryptamine 7 receptor	P34969	HTR7	0.027
Radix Saposhnikoviae（RS）	MOL001942	isoimperatorin	Solute carrier family 12 member 2	P55011	SLC12A2	0.027
Radix Saposhnikoviae（RS）	MOL001942	isoimperatorin	Solute carrier family 12 member 1	Q13621	SLC12A1	0.027
Radix Saposhnikoviae（RS）	MOL001942	isoimperatorin	Solute carrier family 12 member 5	Q9H2X9	SLC12A5	0.027
Radix Saposhnikoviae（RS）	MOL001942	isoimperatorin	Solute carrier family 12 member 4	Q9UP95	SLC12A4	0.027
Radix Saposhnikoviae（RS）	MOL001942	isoimperatorin	Glutathione S-transferase P	P09211	GSTP1	0.028
Radix Saposhnikoviae（RS）	MOL001942	isoimperatorin	Beta-3 adrenergic receptor	P13945	ADRB3	0.028
Radix Saposhnikoviae（RS）	MOL001942	isoimperatorin	Opioid receptor, sigma 1	Q5T1J1	SIGMAR1	0.029
Radix Saposhnikoviae（RS）	MOL001942	isoimperatorin	Sigma 1-type opioid receptor	Q99720	SIGMAR1	0.029
Radix Saposhnikoviae（RS）	MOL001942	isoimperatorin	Glutamate [NMDA] receptor subunit zeta-1	Q05586	GRIN1	0.031
Radix Saposhnikoviae（RS）	MOL001942	isoimperatorin	5-hydroxytryptamine 4 receptor	Q13639	HTR4	0.031
Radix Saposhnikoviae（RS）	MOL001942	isoimperatorin	Potassium channel subfamily K member 6	Q9Y257	KCNK6	0.031

Radix Saposhnikoviae（RS）	MOL001942	isoimperatorin	Interleukin-3	P08700	IL3	0.032
Radix Saposhnikoviae（RS）	MOL001942	isoimperatorin	Substance-P receptor	P25103	TACR1	0.032
Radix Saposhnikoviae（RS）	MOL001942	isoimperatorin	Protein S100-A12	P80511	S100A12	0.032
Radix Saposhnikoviae（RS）	MOL001942	isoimperatorin	Protein S100-A13	Q99584	S100A13	0.032
Radix Saposhnikoviae（RS）	MOL001942	isoimperatorin	Dihydrofolate reductase	P00374	DHFR	0.033
Radix Saposhnikoviae（RS）	MOL001942	isoimperatorin	Glutamate [NMDA] receptor subunit epsilon-1	Q12879	GRIN2A	0.033
Radix Saposhnikoviae（RS）	MOL001942	isoimperatorin	Glutamate [NMDA] receptor subunit epsilon-2	Q13224	GRIN2B	0.033
Radix Saposhnikoviae（RS）	MOL001942	isoimperatorin	Prostaglandin reductase 1	Q14914	PTGR1	0.033
Radix Saposhnikoviae（RS）	MOL001942	isoimperatorin	Glutamate [NMDA] receptor subunit epsilon-3	Q14957	GRIN2C	0.033
Radix Saposhnikoviae（RS）	MOL001942	isoimperatorin	Actin-related protein 2/3 complex subunit 1B	O15143	ARPC1B	0.035
Radix Saposhnikoviae（RS）	MOL001942	isoimperatorin	Actin-related protein 2/3 complex subunit 2	O15144	ARPC2	0.035
Radix Saposhnikoviae（RS）	MOL001942	isoimperatorin	Actin-related protein 2/3 complex subunit 3	O15145	ARPC3	0.035
Radix Saposhnikoviae（RS）	MOL001942	isoimperatorin	Actin-related protein 2/3 complex subunit 4	P59998	ARPC4	0.035
Radix Saposhnikoviae（RS）	MOL001942	isoimperatorin	Actin-related protein 3	P61158	ACTR3	0.035
Radix Saposhnikoviae（RS）	MOL001942	isoimperatorin	Actin-related protein 2	P61160	ACTR2	0.035
Radix Saposhnikoviae（RS）	MOL001942	isoimperatorin	Sodium channel protein type 10 subunit alpha	Q9Y5Y9	SCN10A	0.035
Radix Saposhnikoviae（RS）	MOL001942	isoimperatorin	Neuronal acetylcholine receptor subunit alpha-4	P43681	CHRNA4	0.036
Radix Saposhnikoviae（RS）	MOL001942	isoimperatorin	Kappa-type opioid receptor	P41145	OPRK1	0.037
Radix Saposhnikoviae（RS）	MOL001942	isoimperatorin	Neuronal acetylcholine receptor subunit alpha-3	P32297	CHRNA3	0.038
Radix Saposhnikoviae（RS）	MOL001942	isoimperatorin	T-cell receptor alpha chain C region	P01848	TRAC	0.039
Radix Saposhnikoviae（RS）	MOL001942	isoimperatorin	T-cell receptor beta chain C region	P01850	TRBC1	0.039
Radix Saposhnikoviae（RS）	MOL001942	isoimperatorin	Beta-2-microglobulin	P61769	B2M	0.039
Radix Saposhnikoviae（RS）	MOL001942	isoimperatorin	3-phosphoinositide-dependent protein kinase 1	O15530	PDPK1	0.04
Radix Saposhnikoviae（RS）	MOL001942	isoimperatorin	Proto-oncogene tyrosine-protein kinase LCK	P06239	LCK	0.04
Radix Saposhnikoviae（RS）	MOL001942	isoimperatorin	Proto-oncogene serine/threonine-protein kinase Pim-1	P11309	PIM1	0.04
Radix Saposhnikoviae（RS）	MOL001942	isoimperatorin	Tyrosine-protein kinase CSK	P41240	CSK	0.04
Radix Saposhnikoviae（RS）	MOL001942	isoimperatorin	Tyrosine-protein kinase ZAP-70	P43403	ZAP70	0.04
Radix Saposhnikoviae（RS）	MOL001942	isoimperatorin	Tyrosine-protein kinase SYK	P43405	SYK	0.04
Radix Saposhnikoviae（RS）	MOL001942	isoimperatorin	Glycogen synthase kinase-3 beta	P49841	GSK3B	0.04
Radix Saposhnikoviae（RS）	MOL001942	isoimperatorin	Protein kinase C theta type	Q04759	PRKCQ	0.04
Radix Saposhnikoviae（RS）	MOL001942	isoimperatorin	Tyrosine-protein kinase ITK/TSK	Q08881	ITK	0.04
Radix Saposhnikoviae（RS）	MOL001942	isoimperatorin	Tyrosine-protein kinase JAK2	O60674	JAK2	0.045
Radix Saposhnikoviae（RS）	MOL001942	isoimperatorin	Tyrosine-protein kinase JAK1	P23458	JAK1	0.045
Radix Saposhnikoviae（RS）	MOL001942	isoimperatorin	Tyrosine-protein kinase JAK3	P52333	JAK3	0.045
Radix Saposhnikoviae（RS）	MOL001942	isoimperatorin	Tyrosine 3-monooxygenase	P07101	TH	0.046
Radix Saposhnikoviae（RS）	MOL001942	isoimperatorin	Peptidyl-prolyl cis-trans isomerase NIMA-interacting 1	Q13526	PIN1	0.046
Radix Saposhnikoviae（RS）	MOL001942	isoimperatorin	Tyrosyl-tRNA synthetase, mitochondrial	Q9Y2Z4	YARS2	0.046
Radix Saposhnikoviae（RS）	MOL001942	isoimperatorin	85 kDa calcium-independent phospholipase A2	O60733	PLA2G6	0.048
Radix Saposhnikoviae（RS）	MOL001942	isoimperatorin	Prothrombin	P00734	F2	0.048
Radix Saposhnikoviae（RS）	MOL001942	isoimperatorin	ATP synthase subunit beta, mitochondrial	P06576	ATP5F1B	0.048
Radix Saposhnikoviae（RS）	MOL001942	isoimperatorin	ATP synthase subunit alpha, mitochondrial	P25705	ATP5F1A	0.048
Radix Saposhnikoviae（RS）	MOL001942	isoimperatorin	Cytosolic phospholipase A2	P47712	PLA2G4A	0.048
Radix Saposhnikoviae（RS）	MOL001942	isoimperatorin	DNA-(apurinic or apyrimidinic site) lyase	P27695	APEX1	0.049
Radix Saposhnikoviae（RS）	MOL001942	isoimperatorin	Calcium/calmodulin-dependent protein kinase type II subunit delta	Q13557	CAMK2D	0.05
Radix Saposhnikoviae（RS）	MOL001942	isoimperatorin	STE20-like serine/threonine-protein kinase	Q9H2G2	SLK	0.05
Radix Saposhnikoviae（RS）	MOL001942	isoimperatorin	Potassium channel subfamily K member 1	O00180	KCNK1	0.051
Radix Saposhnikoviae（RS）	MOL001942	isoimperatorin	Lipoic acid synthetase, mitochondrial	O43766	LIAS	0.051

Radix Saposhnikoviae（RS）	MOL001942	isoimperatorin	Farnesyl pyrophosphate synthetase	P14324	FDPS	0.051
Radix Saposhnikoviae（RS）	MOL001942	isoimperatorin	Liver carboxylesterase 1	P23141	CES1	0.051
Radix Saposhnikoviae（RS）	MOL001942	isoimperatorin	Isopentenyl-diphosphate Delta-isomerase 1	Q13907	IDI1	0.051
Radix Saposhnikoviae（RS）	MOL001942	isoimperatorin	Platelet-activating factor acetylhydrolase IB subunit gamma	Q15102	PAFAH1B3	0.051
Radix Saposhnikoviae（RS）	MOL001942	isoimperatorin	Lipoyltransferase 1, mitochondrial	Q9Y234	LIPT1	0.051
Radix Saposhnikoviae（RS）	MOL001942	isoimperatorin	Peptidyl-prolyl cis-trans isomerase, mitochondrial	P30405	PPIF	0.053
Radix Saposhnikoviae（RS）	MOL001942	isoimperatorin	Neuronal acetylcholine receptor subunit alpha-2	Q15822	CHRNA2	0.053
Radix Saposhnikoviae（RS）	MOL001942	isoimperatorin	D1 dopamine receptor-interacting protein calcyon	Q9NYX4	CALY	0.053
Radix Saposhnikoviae（RS）	MOL001942	isoimperatorin	D-HSCDK2	O75100	CA11	0.054
Radix Saposhnikoviae（RS）	MOL001942	isoimperatorin	Cell division control protein 2 homolog	P06493	CDK1	0.054
Radix Saposhnikoviae（RS）	MOL001942	isoimperatorin	Alpha-1D adrenergic receptor	P25100	ADRA1D	0.054
Radix Saposhnikoviae（RS）	MOL001942	isoimperatorin	Neuronal acetylcholine receptor subunit beta-4	P30926	CHRNB4	0.055
Radix Saposhnikoviae（RS）	MOL001942	isoimperatorin	Nitric-oxide synthase, endothelial	P29474	NOS3	0.056
Radix Saposhnikoviae（RS）	MOL001942	isoimperatorin	Nitric-oxide synthase, brain	P29475	NOS1	0.056
Radix Saposhnikoviae（RS）	MOL001942	isoimperatorin	Translocator protein	P30536	TSPO	0.057
Radix Saposhnikoviae（RS）	MOL001942	isoimperatorin	5-hydroxytryptamine 6 receptor	P50406	HTR6	0.057
Radix Saposhnikoviae（RS）	MOL001942	isoimperatorin	Alcohol dehydrogenase [NADP+]	P14550	AKR1A1	0.058
Radix Saposhnikoviae（RS）	MOL001942	isoimperatorin	Beta-1 adrenergic receptor	P08588	ADRB1	0.059
Radix Saposhnikoviae（RS）	MOL001942	isoimperatorin	Ribosyldihydronicotinamide dehydrogenase [quinone]	P16083	NQO2	0.059
Radix Saposhnikoviae（RS）	MOL001942	isoimperatorin	5-hydroxytryptamine 3 receptor	P46098	HTR3A	0.059
Radix Saposhnikoviae（RS）	MOL001942	isoimperatorin	Casein kinase II subunit alpha	P68400	CSNK2A1	0.059
Radix Saposhnikoviae（RS）	MOL001942	isoimperatorin	cAMP-specific 3',5'-cyclic phosphodiesterase 4C	Q08493	PDE4C	0.059
Radix Saposhnikoviae（RS）	MOL001942	isoimperatorin	cAMP-specific 3',5'-cyclic phosphodiesterase 4D	Q08499	PDE4D	0.059
Radix Saposhnikoviae（RS）	MOL001942	isoimperatorin	Mitogen-activated protein kinase 3	P27361	MAPK3	0.06
Radix Saposhnikoviae（RS）	MOL001942	isoimperatorin	Neuronal acetylcholine receptor subunit alpha-7	P36544	CHRNA7	0.06
Radix Saposhnikoviae（RS）	MOL001942	isoimperatorin	Mitogen-activated protein kinase 8	P45983	MAPK8	0.06
Radix Saposhnikoviae（RS）	MOL001942	isoimperatorin	Mitogen-activated protein kinase 10	P53779	MAPK10	0.06
Radix Saposhnikoviae（RS）	MOL001942	isoimperatorin	Peroxisome proliferator-activated receptor delta	Q03181	PPARD	0.06
Radix Saposhnikoviae（RS）	MOL001942	isoimperatorin	Keratin, type II cytoskeletal 7	P08729	KRT7	0.061
Radix Saposhnikoviae（RS）	MOL001942	isoimperatorin	Phospholipase A2, membrane associated	P14555	PLA2G2A	0.061
Radix Saposhnikoviae（RS）	MOL001942	isoimperatorin	Platelet glycoprotein IX	P14770	GP9	0.061
Radix Saposhnikoviae（RS）	MOL001942	isoimperatorin	Peroxisome proliferator-activated receptor gamma	P37231	PPARG	0.061
Radix Saposhnikoviae（RS）	MOL001942	isoimperatorin	Lactoylglutathione lyase	Q04760	GLO1	0.061
Radix Saposhnikoviae（RS）	MOL001942	isoimperatorin	Prostaglandin reductase 2	Q8N8N7	PTGR2	0.061
Radix Saposhnikoviae（RS）	MOL001942	isoimperatorin	Group IIE secretory phospholipase A2	Q9NZK7	PLA2G2E	0.063
Radix Saposhnikoviae（RS）	MOL001942	isoimperatorin	Glutamate [NMDA] receptor subunit epsilon-4	O15399	GRIN2D	0.066
Radix Saposhnikoviae（RS）	MOL001942	isoimperatorin	Glutamate [NMDA] receptor subunit 3B	O60391	GRIN3B	0.066
Radix Saposhnikoviae（RS）	MOL001942	isoimperatorin	Sodium-dependent dopamine transporter	Q01959	SLC6A3	0.069
Radix Saposhnikoviae（RS）	MOL001942	isoimperatorin	Mineralocorticoid receptor	P08235	NR3C2	0.07
Radix Saposhnikoviae（RS）	MOL001942	isoimperatorin	RAC-beta serine/threonine-protein kinase	P31751	AKT2	0.07
Radix Saposhnikoviae（RS）	MOL001942	isoimperatorin	Acetylcholinesterase	P22303	ACHE	0.072
Radix Saposhnikoviae（RS）	MOL001942	isoimperatorin	Tubulin beta-2C chain	P68371	TUBB4B	0.072
Radix Saposhnikoviae（RS）	MOL001942	isoimperatorin	Delta-type opioid receptor	P41143	OPRD1	0.076
Radix Saposhnikoviae（RS）	MOL001942	isoimperatorin	Tryptophanyl-tRNA synthetase, mitochondrial	Q9UGM6	WARS2	0.079
Radix Saposhnikoviae（RS）	MOL001942	isoimperatorin	Potassium voltage-gated channel subfamily H member 2	Q12809	KCNH2	0.08
Radix Saposhnikoviae（RS）	MOL001942	isoimperatorin	5-hydroxytryptamine 1D receptor	P28221	HTR1D	0.081
Radix Saposhnikoviae（RS）	MOL001942	isoimperatorin	MAP kinase-activated protein kinase 2	P49137	MAPKAPK2	0.081

Radix Saposhnikoviae（RS）	MOL001942	isoimperatorin	Muscarinic acetylcholine receptor M5	P08912	CHRM5	0.084
Radix Saposhnikoviae（RS）	MOL001942	isoimperatorin	Gamma-aminobutyric-acid receptor subunit beta-3	P28472	GABRB3	0.086
Radix Saposhnikoviae（RS）	MOL001942	isoimperatorin	Carbonic anhydrase 1	P00915	CA1	0.089
Radix Saposhnikoviae（RS）	MOL001942	isoimperatorin	Beta-2 adrenergic receptor	P07550	ADRB2	0.089
Radix Saposhnikoviae（RS）	MOL001942	isoimperatorin	Carbonic anhydrase 4	P22748	CA4	0.089
Radix Saposhnikoviae（RS）	MOL001942	isoimperatorin	Death-associated protein kinase 3	O43293	DAPK3	0.093
Radix Saposhnikoviae（RS）	MOL001942	isoimperatorin	Sodium-dependent serotonin transporter	P31645	SLC6A4	0.093
Radix Saposhnikoviae（RS）	MOL001942	isoimperatorin	Tyrosyl-tRNA synthetase, cytoplasmic	P54577	YARS	0.094
Radix Saposhnikoviae（RS）	MOL001942	isoimperatorin	Gamma-aminobutyric acid receptor subunit theta	Q9UN88	GABRQ	0.096
Radix Saposhnikoviae（RS）	MOL001942	isoimperatorin	Heat shock protein HSP 90-beta	P08238	HSP90AB1	0.097
Radix Saposhnikoviae（RS）	MOL001942	isoimperatorin	Protein farnesyltransferase/geranylgeranyltransferase type I alpha subunit	P49354	FNTA	0.097
Radix Saposhnikoviae（RS）	MOL001942	isoimperatorin	DNA topoisomerase 2-alpha	P11388	TOP2A	0.099
Radix Saposhnikoviae（RS）	MOL001942	isoimperatorin	Gamma-aminobutyric-acid receptor subunit alpha-6	Q16445	GABRA6	0.099
Radix Saposhnikoviae（RS）	MOL001942	isoimperatorin	3-oxo-5-alpha-steroid 4-dehydrogenase 2	P31213	SRD5A2	0.1
Radix Saposhnikoviae（RS）	MOL001942	isoimperatorin	Inosine-5'-monophosphate dehydrogenase 1	P20839	IMPDH1	0.101
Radix Saposhnikoviae（RS）	MOL001942	isoimperatorin	Potassium channel subfamily K member 3	O14649	KCNK3	0.103
Radix Saposhnikoviae（RS）	MOL001942	isoimperatorin	Tyrosine-protein kinase HCK	P08631	HCK	0.104
Radix Saposhnikoviae（RS）	MOL001942	isoimperatorin	Tyrosine-protein phosphatase non-receptor type 1	P18031	PTPN1	0.104
Radix Saposhnikoviae（RS）	MOL001942	isoimperatorin	Peroxisome proliferator-activated receptor alpha	Q07869	PPARA	0.105
Radix Saposhnikoviae（RS）	MOL001942	isoimperatorin	Cannabinoid receptor 1	P21554	CNR1	0.106
Radix Saposhnikoviae（RS）	MOL001942	isoimperatorin	Nitric oxide synthase, inducible	P35228	NOS2	0.107
Radix Saposhnikoviae（RS）	MOL001942	isoimperatorin	Egl nine homolog 1	Q9GZT9	EGLN1	0.107
Radix Saposhnikoviae（RS）	MOL001942	isoimperatorin	Gamma-aminobutyric-acid receptor subunit alpha-4	P48169	GABRA4	0.108
Radix Saposhnikoviae（RS）	MOL001942	isoimperatorin	Dual specificity protein kinase CLK1	P49759	CLK1	0.108
Radix Saposhnikoviae（RS）	MOL001942	isoimperatorin	Cell division protein kinase 5	Q00535	CDK5	0.109
Radix Saposhnikoviae（RS）	MOL001942	isoimperatorin	Thromboxane A2 receptor	P21731	TBXA2R	0.11
Radix Saposhnikoviae（RS）	MOL001942	isoimperatorin	5-hydroxytryptamine 1B receptor	P28222	HTR1B	0.11
Radix Saposhnikoviae（RS）	MOL001942	isoimperatorin	RAC-alpha serine/threonine-protein kinase	P31749	AKT1	0.11
Radix Saposhnikoviae（RS）	MOL001942	isoimperatorin	D(4) dopamine receptor	P21917	DRD4	0.111
Radix Saposhnikoviae（RS）	MOL001942	isoimperatorin	D(1B) dopamine receptor	P21918	DRD5	0.111
Radix Saposhnikoviae（RS）	MOL001942	isoimperatorin	D(3) dopamine receptor	P35462	DRD3	0.111
Radix Saposhnikoviae（RS）	MOL001942	isoimperatorin	Ig gamma-1 chain C region	P01857	IGHG1	0.112
Radix Saposhnikoviae（RS）	MOL001942	isoimperatorin	5-hydroxytryptamine 2B receptor	P41595	HTR2B	0.113
Radix Saposhnikoviae（RS）	MOL001942	isoimperatorin	Phosphatidylinositol-4,5-bisphosphate 3-kinase catalytic subunit gamma isoform	P48736	PIK3CG	0.113
Radix Saposhnikoviae（RS）	MOL001942	isoimperatorin	DNA polymerase kappa	Q9UBT6	POLK	0.113
Radix Saposhnikoviae（RS）	MOL001942	isoimperatorin	Gamma-aminobutyric-acid receptor subunit beta-2	P47870	GABRB2	0.115
Radix Saposhnikoviae（RS）	MOL001942	isoimperatorin	Triosephosphate isomerase	P60174	TPI1	0.116
Radix Saposhnikoviae（RS）	MOL001942	isoimperatorin	Amine oxidase [flavin-containing] B	P27338	MAOB	0.118
Radix Saposhnikoviae（RS）	MOL001942	isoimperatorin	Aldose reductase	P15121	AKR1B1	0.12
Radix Saposhnikoviae（RS）	MOL001942	isoimperatorin	Hydroxyacid oxidase 1	Q9UJM8	HAO1	0.121
Radix Saposhnikoviae（RS）	MOL001942	isoimperatorin	C-jun-amino-terminal kinase-interacting protein 1	Q9UQF2	MAPK8IP1	0.122
Radix Saposhnikoviae（RS）	MOL001942	isoimperatorin	Ig kappa chain V-II region RPMI 6410	P06310	IGKV2-30	0.125
Radix Saposhnikoviae（RS）	MOL001942	isoimperatorin	Toll-like receptor 7	Q9NYK1	TLR7	0.131
Radix Saposhnikoviae（RS）	MOL001942	isoimperatorin	Carbonic anhydrase 2	P00918	CA2	0.134
Radix Saposhnikoviae（RS）	MOL001942	isoimperatorin	Trypsin-1	P07477	PRSS1	0.138
Radix Saposhnikoviae（RS）	MOL001942	isoimperatorin	Alpha-2C adrenergic receptor	P18825	ADRA2C	0.138

Radix Saposhnikoviae（RS）	MOL001942	isoimperatorin	cAMP-dependent protein kinase inhibitor alpha	P61925	PKIA	0.14
Radix Saposhnikoviae（RS）	MOL001942	isoimperatorin	Muscarinic acetylcholine receptor M2	P08172	CHRM2	0.141
Radix Saposhnikoviae（RS）	MOL001942	isoimperatorin	Muscarinic acetylcholine receptor M4	P08173	CHRM4	0.141
Radix Saposhnikoviae（RS）	MOL001942	isoimperatorin	Muscarinic acetylcholine receptor M3	P20309	CHRM3	0.141
Radix Saposhnikoviae（RS）	MOL001942	isoimperatorin	Alpha-1B adrenergic receptor	P35368	ADRA1B	0.141
Radix Saposhnikoviae（RS）	MOL001942	isoimperatorin	Progesterone receptor	P06401	PGR	0.142
Radix Saposhnikoviae（RS）	MOL001942	isoimperatorin	Gamma-aminobutyric acid receptor subunit gamma-2	P18507	GABRG2	0.145
Radix Saposhnikoviae（RS）	MOL001942	isoimperatorin	Gamma-aminobutyric acid receptor subunit gamma-1	Q8N1C3	GABRG1	0.145
Radix Saposhnikoviae（RS）	MOL001942	isoimperatorin	Tubulin alpha-3 chain	Q71U36	TUBA1A	0.146
Radix Saposhnikoviae（RS）	MOL001942	isoimperatorin	Gamma-aminobutyric acid receptor subunit rho-3	A8MPY1	GABRR3	0.155
Radix Saposhnikoviae（RS）	MOL001942	isoimperatorin	Gamma-aminobutyric acid receptor subunit pi	O00591	GABRP	0.155
Radix Saposhnikoviae（RS）	MOL001942	isoimperatorin	Gamma-aminobutyric acid receptor subunit delta	O14764	GABRD	0.155
Radix Saposhnikoviae（RS）	MOL001942	isoimperatorin	Gamma-aminobutyric-acid receptor subunit beta-1	P18505	GABRB1	0.155
Radix Saposhnikoviae（RS）	MOL001942	isoimperatorin	Gamma-aminobutyric-acid receptor subunit rho-1	P24046	GABRR1	0.155
Radix Saposhnikoviae（RS）	MOL001942	isoimperatorin	Gamma-aminobutyric acid receptor subunit rho-2	P28476	GABRR2	0.155
Radix Saposhnikoviae（RS）	MOL001942	isoimperatorin	Gamma-aminobutyric acid receptor subunit epsilon	P78334	GABRE	0.155
Radix Saposhnikoviae（RS）	MOL001942	isoimperatorin	Gamma-aminobutyric acid receptor subunit gamma-3	Q99928	GABRG3	0.155
Radix Saposhnikoviae（RS）	MOL001942	isoimperatorin	Sodium-dependent noradrenaline transporter	P23975	SLC6A2	0.16
Radix Saposhnikoviae（RS）	MOL001942	isoimperatorin	Glutamate [NMDA] receptor subunit 3A	Q8TCU5	GRIN3A	0.164
Radix Saposhnikoviae（RS）	MOL001942	isoimperatorin	Alpha-2A adrenergic receptor	P08913	ADRA2A	0.165
Radix Saposhnikoviae（RS）	MOL001942	isoimperatorin	Alpha-2B adrenergic receptor	P18089	ADRA2B	0.166
Radix Saposhnikoviae（RS）	MOL001942	isoimperatorin	5-hydroxytryptamine 2C receptor	P28335	HTR2C	0.171
Radix Saposhnikoviae（RS）	MOL001942	isoimperatorin	Gamma-aminobutyric-acid receptor subunit alpha-5	P31644	GABRA5	0.177
Radix Saposhnikoviae（RS）	MOL001942	isoimperatorin	Histamine H1 receptor	P35367	HRH1	0.178
Radix Saposhnikoviae（RS）	MOL001942	isoimperatorin	cAMP-specific 3',5'-cyclic phosphodiesterase 4A	P27815	PDE4A	0.18
Radix Saposhnikoviae（RS）	MOL001942	isoimperatorin	cAMP-specific 3',5'-cyclic phosphodiesterase 4B	Q07343	PDE4B	0.18
Radix Saposhnikoviae（RS）	MOL001942	isoimperatorin	Gamma-aminobutyric-acid receptor subunit alpha-3	P34903	GABRA3	0.187
Radix Saposhnikoviae（RS）	MOL001942	isoimperatorin	Sodium channel protein type 5 subunit alpha	Q14524	SCN5A	0.192
Radix Saposhnikoviae（RS）	MOL001942	isoimperatorin	Gamma-aminobutyric-acid receptor subunit alpha-2	P47869	GABRA2	0.208
Radix Saposhnikoviae（RS）	MOL001942	isoimperatorin	Mu-type opioid receptor	P35372	OPRM1	0.22
Radix Saposhnikoviae（RS）	MOL001942	isoimperatorin	Alpha-1A adrenergic receptor	P35348	ADRA1A	0.225
Radix Saposhnikoviae（RS）	MOL001942	isoimperatorin	Cell division protein kinase 2	P24941	CDK2	0.226
Radix Saposhnikoviae（RS）	MOL001942	isoimperatorin	Proto-oncogene tyrosine-protein kinase Src	P12931	SRC	0.227
Radix Saposhnikoviae（RS）	MOL001942	isoimperatorin	Mitogen-activated protein kinase 14	Q16539	MAPK14	0.227
Radix Saposhnikoviae（RS）	MOL001942	isoimperatorin	5-hydroxytryptamine 2A receptor	P28223	HTR2A	0.228
Radix Saposhnikoviae（RS）	MOL001942	isoimperatorin	Gamma-aminobutyric-acid receptor subunit alpha-1	P14867	GABRA1	0.229
Radix Saposhnikoviae（RS）	MOL001942	isoimperatorin	5-hydroxytryptamine 1A receptor	P08908	HTR1A	0.232
Radix Saposhnikoviae（RS）	MOL001942	isoimperatorin	D(1A) dopamine receptor	P21728	DRD1	0.251
Radix Saposhnikoviae（RS）	MOL001942	isoimperatorin	Muscarinic acetylcholine receptor M1	P11229	CHRM1	0.294
Radix Saposhnikoviae（RS）	MOL001942	isoimperatorin	Hemoglobin subunit alpha	P69905	HBA1	0.298
Radix Saposhnikoviae（RS）	MOL001942	isoimperatorin	D(2) dopamine receptor	P14416	DRD2	0.337
Radix Saposhnikoviae（RS）	MOL001942	isoimperatorin	Nuclear receptor coactivator 1	Q15788	NCOA1	0.349
Radix Saposhnikoviae（RS）	MOL001942	isoimperatorin	Prostaglandin G/H synthase 1	P23219	PTGS1	0.434
Radix Saposhnikoviae（RS）	MOL001942	isoimperatorin	Estrogen receptor beta	Q92731	ESR2	0.494
Radix Saposhnikoviae（RS）	MOL001942	isoimperatorin	Cyclin-A2	P20248	CCNA2	0.584
Radix Saposhnikoviae（RS）	MOL001942	isoimperatorin	cAMP-dependent protein kinase catalytic subunit alpha	P17612	PRKACA	0.717
Radix Saposhnikoviae（RS）	MOL001942	isoimperatorin	Prostaglandin G/H synthase 2	P35354	PTGS2	0.956

Radix Saposhnikoviae（RS）	MOL001942	isoimperatorin	Estrogen receptor	P03372	ESR1	1
Radix Saposhnikoviae（RS）	MOL001949	panaxydol	Beta-nerve growth factor	P01138	NGF	Validated
Radix Saposhnikoviae（RS）	MOL001949	panaxydol	Muscarinic acetylcholine receptor M1	P11229	CHRM1	Validated
Radix Saposhnikoviae（RS）	MOL001949	panaxydol	Brain-derived neurotrophic factor	P23560	BDNF	Validated
Radix Saposhnikoviae（RS）	MOL001949	panaxydol	Cell division protein kinase 2	P24941	CDK2	Validated
Radix Saposhnikoviae（RS）	MOL001949	panaxydol	Caspase-3	P42574	CASP3	Validated
Radix Saposhnikoviae（RS）	MOL001949	panaxydol	Microtubule-associated protein 1B	P46821	MAP1B	Validated
Radix Saposhnikoviae（RS）	MOL001949	panaxydol	Eukaryotic translation initiation factor 6	P56537	EIF6	Validated
Radix Saposhnikoviae（RS）	MOL002644	Phellopterin	Acetylcholine receptor subunit alpha	P02708	CHRNA1	0.013
Radix Saposhnikoviae（RS）	MOL002644	Phellopterin	Cholinesterase	P06276	BCHE	0.013
Radix Saposhnikoviae（RS）	MOL002644	Phellopterin	Acetylcholine receptor subunit gamma	P07510	CHRNG	0.013
Radix Saposhnikoviae（RS）	MOL002644	Phellopterin	Acetylcholine receptor subunit beta	P11230	CHRNB1	0.013
Radix Saposhnikoviae（RS）	MOL002644	Phellopterin	Neuronal acetylcholine receptor subunit beta-2	P17787	CHRNB2	0.013
Radix Saposhnikoviae（RS）	MOL002644	Phellopterin	Neuronal acetylcholine receptor subunit alpha-5	P30532	CHRNA5	0.013
Radix Saposhnikoviae（RS）	MOL002644	Phellopterin	Neuronal acetylcholine receptor subunit beta-4	P30926	CHRNB4	0.013
Radix Saposhnikoviae（RS）	MOL002644	Phellopterin	Neuronal acetylcholine receptor subunit alpha-4	P43681	CHRNA4	0.013
Radix Saposhnikoviae（RS）	MOL002644	Phellopterin	Acetylcholine receptor subunit epsilon	Q04844	CHRNE	0.013
Radix Saposhnikoviae（RS）	MOL002644	Phellopterin	Neuronal acetylcholine receptor subunit beta-3	Q05901	CHRNB3	0.013
Radix Saposhnikoviae（RS）	MOL002644	Phellopterin	Acetylcholine receptor subunit delta	Q07001	CHRND	0.013
Radix Saposhnikoviae（RS）	MOL002644	Phellopterin	Neuronal acetylcholine receptor subunit alpha-6	Q15825	KCNJ8	0.013
Radix Saposhnikoviae（RS）	MOL002644	Phellopterin	Neuronal acetylcholine receptor subunit alpha-10	Q9GZZ6	CHRNA10	0.013
Radix Saposhnikoviae（RS）	MOL002644	Phellopterin	Neuronal acetylcholine receptor subunit alpha-9	Q9UGM1	CHRNA9	0.013
Radix Saposhnikoviae（RS）	MOL002644	Phellopterin	Voltage-dependent T-type calcium channel subunit alpha-1G	O43497	CACNA1G	0.015
Radix Saposhnikoviae（RS）	MOL002644	Phellopterin	Carbonic anhydrase 12	O43570	CA12	0.015
Radix Saposhnikoviae（RS）	MOL002644	Phellopterin	Carbonic anhydrase-related protein 11	O75493	CA11	0.015
Radix Saposhnikoviae（RS）	MOL002644	Phellopterin	Carbonic anhydrase 3	P07451	CA3	0.015
Radix Saposhnikoviae（RS）	MOL002644	Phellopterin	Amine oxidase [flavin-containing] A	P21397	MAOA	0.015
Radix Saposhnikoviae（RS）	MOL002644	Phellopterin	Carbonic anhydrase 6	P23280	CA6	0.015
Radix Saposhnikoviae（RS）	MOL002644	Phellopterin	Carbonic anhydrase 5A, mitochondrial	P35218	CA5A	0.015
Radix Saposhnikoviae（RS）	MOL002644	Phellopterin	Carbonic anhydrase-related protein	P35219	CA8	0.015
Radix Saposhnikoviae（RS）	MOL002644	Phellopterin	Sodium channel protein type 4 subunit alpha	P35499	SCN4A	0.015
Radix Saposhnikoviae（RS）	MOL002644	Phellopterin	Carbonic anhydrase 7	P43166	CA7	0.015
Radix Saposhnikoviae（RS）	MOL002644	Phellopterin	Sodium channel subunit beta-1	Q07699	SCN1B	0.015
Radix Saposhnikoviae（RS）	MOL002644	Phellopterin	Sodium channel protein type 9 subunit alpha	Q15858	SCN9A	0.015
Radix Saposhnikoviae（RS）	MOL002644	Phellopterin	2,4-dienoyl-CoA reductase, mitochondrial	Q16698	DECR1	0.015
Radix Saposhnikoviae（RS）	MOL002644	Phellopterin	Carbonic anhydrase 9	Q16790	CA9	0.015
Radix Saposhnikoviae（RS）	MOL002644	Phellopterin	Sodium channel subunit beta-4	Q8IWT1	SCN4B	0.015
Radix Saposhnikoviae（RS）	MOL002644	Phellopterin	Carbonic anhydrase 13	Q8N1Q1	CA13	0.015
Radix Saposhnikoviae（RS）	MOL002644	Phellopterin	Sodium channel protein type 2 subunit alpha	Q99250	SCN2A	0.015
Radix Saposhnikoviae（RS）	MOL002644	Phellopterin	Carbonic anhydrase-related protein 10	Q9NS85	CA10	0.015
Radix Saposhnikoviae（RS）	MOL002644	Phellopterin	Sodium channel protein type 3 subunit alpha	Q9NY46	SCN3A	0.015
Radix Saposhnikoviae（RS）	MOL002644	Phellopterin	Sodium channel subunit beta-3	Q9NY72	SCN3B	0.015
Radix Saposhnikoviae（RS）	MOL002644	Phellopterin	Voltage-dependent T-type calcium channel subunit alpha-1I	Q9P0X4	CACNA1I	0.015
Radix Saposhnikoviae（RS）	MOL002644	Phellopterin	Sodium channel protein type 11 subunit alpha	Q9UI33	SCN11A	0.015
Radix Saposhnikoviae（RS）	MOL002644	Phellopterin	Carbonic anhydrase 14	Q9ULX7	CA14	0.015
Radix Saposhnikoviae（RS）	MOL002644	Phellopterin	Carbonic anhydrase 5B, mitochondrial	Q9Y2D0	CA5B	0.015
Radix Saposhnikoviae（RS）	MOL002644	Phellopterin	Sodium/potassium-transporting ATPase alpha-1 chain	P05023	ATP1A1	0.016

Radix Saposhnikoviae（RS）	MOL002644	Phellopterin	Solute carrier family 12 member 3	P55017	SLC12A3	0.016
Radix Saposhnikoviae（RS）	MOL002644	Phellopterin	ATP-sensitive inward rectifier potassium channel 11	Q14654	KCNJ11	0.016
Radix Saposhnikoviae（RS）	MOL002644	Phellopterin	Potassium channel subfamily K member 6	Q9Y257	KCNK6	0.016
Radix Saposhnikoviae（RS）	MOL002644	Phellopterin	Sodium channel protein type 10 subunit alpha	Q9Y5Y9	SCN10A	0.019
Radix Saposhnikoviae（RS）	MOL002644	Phellopterin	fMet-Leu-Phe receptor	P21462	FPR1	0.02
Radix Saposhnikoviae（RS）	MOL002644	Phellopterin	Prostaglandin D2 receptor	Q13258	PTGDR	0.02
Radix Saposhnikoviae（RS）	MOL002644	Phellopterin	Cysteinyl leukotriene receptor 2	Q9NS75	CYSLTR2	0.02
Radix Saposhnikoviae（RS）	MOL002644	Phellopterin	Cysteinyl leukotriene receptor 1	Q9Y271	CYSLTR1	0.02
Radix Saposhnikoviae（RS）	MOL002644	Phellopterin	5-hydroxytryptamine 1F receptor	P30939	HTR1F	0.021
Radix Saposhnikoviae（RS）	MOL002644	Phellopterin	Opioid receptor, sigma 1	Q5T1J1	SIGMAR1	0.021
Radix Saposhnikoviae（RS）	MOL002644	Phellopterin	Sigma 1-type opioid receptor	Q99720	SIGMAR1	0.021
Radix Saposhnikoviae（RS）	MOL002644	Phellopterin	Beta-3 adrenergic receptor	P13945	ADRB3	0.022
Radix Saposhnikoviae（RS）	MOL002644	Phellopterin	Histamine H2 receptor	P25021	HRH2	0.022
Radix Saposhnikoviae（RS）	MOL002644	Phellopterin	Glutamate receptor 2	P42262	GRIA2	0.022
Radix Saposhnikoviae（RS）	MOL002644	Phellopterin	D1 dopamine receptor-interacting protein calcyon	Q9NYX4	CALY	0.022
Radix Saposhnikoviae（RS）	MOL002644	Phellopterin	Coagulation factor IX	P00740	F9	0.023
Radix Saposhnikoviae（RS）	MOL002644	Phellopterin	Coagulation factor X	P00742	F10	0.023
Radix Saposhnikoviae（RS）	MOL002644	Phellopterin	Osteocalcin	P02818	BGLAP	0.023
Radix Saposhnikoviae（RS）	MOL002644	Phellopterin	Vitamin K-dependent protein C	P04070	PROC	0.023
Radix Saposhnikoviae（RS）	MOL002644	Phellopterin	Coagulation factor VII	P08709	F7	0.023
Radix Saposhnikoviae（RS）	MOL002644	Phellopterin	NAD(P)H dehydrogenase [quinone] 1	P15559	NQO1	0.023
Radix Saposhnikoviae（RS）	MOL002644	Phellopterin	Vitamin K-dependent protein Z	P22891	PROZ	0.023
Radix Saposhnikoviae（RS）	MOL002644	Phellopterin	Vitamin K-dependent gamma-carboxylase	P38435	GGCX	0.023
Radix Saposhnikoviae（RS）	MOL002644	Phellopterin	5-hydroxytryptamine 6 receptor	P50406	HTR6	0.023
Radix Saposhnikoviae（RS）	MOL002644	Phellopterin	Solute carrier family 12 member 2	P55011	SLC12A2	0.023
Radix Saposhnikoviae（RS）	MOL002644	Phellopterin	Sodium-dependent dopamine transporter	Q01959	SLC6A3	0.023
Radix Saposhnikoviae（RS）	MOL002644	Phellopterin	Solute carrier family 12 member 1	Q13621	SLC12A1	0.023
Radix Saposhnikoviae（RS）	MOL002644	Phellopterin	Vitamin K epoxide reductase complex subunit 1-like protein 1	Q8N0U8	VKORC1L1	0.023
Radix Saposhnikoviae（RS）	MOL002644	Phellopterin	Vitamin K epoxide reductase complex subunit 1	Q9BQB6	VKORC1	0.023
Radix Saposhnikoviae（RS）	MOL002644	Phellopterin	Solute carrier family 12 member 5	Q9H2X9	SLC12A5	0.023
Radix Saposhnikoviae（RS）	MOL002644	Phellopterin	Solute carrier family 12 member 4	Q9UP95	SLC12A4	0.023
Radix Saposhnikoviae（RS）	MOL002644	Phellopterin	Elongation factor 2	P13639	EEF2	0.024
Radix Saposhnikoviae（RS）	MOL002644	Phellopterin	Poly [ADP-ribose] polymerase 3	Q9Y6F1	PARP3	0.024
Radix Saposhnikoviae（RS）	MOL002644	Phellopterin	Sodium channel protein type 1 subunit alpha	P35498	SCN1A	0.026
Radix Saposhnikoviae（RS）	MOL002644	Phellopterin	Serine/threonine-protein kinase 17B	O94768	STK17B	0.029
Radix Saposhnikoviae（RS）	MOL002644	Phellopterin	ATP synthase subunit gamma, mitochondrial	P36542	ATP5F1C	0.029
Radix Saposhnikoviae（RS）	MOL002644	Phellopterin	UDP-glucuronosyltransferase 3A1	Q6NUS8	UGT3A1	0.029
Radix Saposhnikoviae（RS）	MOL002644	Phellopterin	Pyridoxal kinase	O00764	PDXK	0.031
Radix Saposhnikoviae（RS）	MOL002644	Phellopterin	Cystathionine beta-synthase	P35520	CBS	0.031
Radix Saposhnikoviae（RS）	MOL002644	Phellopterin	Amiloride-sensitive sodium channel subunit alpha	P37088	SCNN1A	0.031
Radix Saposhnikoviae（RS）	MOL002644	Phellopterin	Amiloride-sensitive sodium channel subunit beta	P51168	SCNN1B	0.031
Radix Saposhnikoviae（RS）	MOL002644	Phellopterin	Amiloride-sensitive sodium channel subunit gamma	P51170	SCNN1G	0.031
Radix Saposhnikoviae（RS）	MOL002644	Phellopterin	Pyridoxal phosphate phosphatase	Q96GD0	PDXP	0.031
Radix Saposhnikoviae（RS）	MOL002644	Phellopterin	Farnesyl pyrophosphate synthetase	P14324	FDPS	0.032
Radix Saposhnikoviae（RS）	MOL002644	Phellopterin	5-hydroxytryptamine 3 receptor	P46098	HTR3A	0.032
Radix Saposhnikoviae（RS）	MOL002644	Phellopterin	5-hydroxytryptamine 4 receptor	Q13639	HTR4	0.032
Radix Saposhnikoviae（RS）	MOL002644	Phellopterin	Death-associated protein kinase 3	O43293	DAPK3	0.033

Radix Saposhnikoviae（RS）	MOL002644	Phellopterin	Tyrosine-protein kinase JAK2	O60674	JAK2	0.033
Radix Saposhnikoviae（RS）	MOL002644	Phellopterin	Tyrosine-protein kinase JAK1	P23458	JAK1	0.033
Radix Saposhnikoviae（RS）	MOL002644	Phellopterin	Tyrosine-protein kinase JAK3	P52333	JAK3	0.033
Radix Saposhnikoviae（RS）	MOL002644	Phellopterin	Potassium channel subfamily K member 1	O00180	KCNK1	0.035
Radix Saposhnikoviae（RS）	MOL002644	Phellopterin	Cyclin-dependent kinase 5 activator 1	Q15078	CDK5R1	0.035
Radix Saposhnikoviae（RS）	MOL002644	Phellopterin	Acetylcholinesterase	P22303	ACHE	0.036
Radix Saposhnikoviae（RS）	MOL002644	Phellopterin	Neuronal acetylcholine receptor subunit alpha-3	P32297	CHRNA3	0.036
Radix Saposhnikoviae（RS）	MOL002644	Phellopterin	Neuronal acetylcholine receptor subunit alpha-7	P36544	CHRNA7	0.036
Radix Saposhnikoviae（RS）	MOL002644	Phellopterin	T-cell receptor alpha chain C region	P01848	TRAC	0.039
Radix Saposhnikoviae（RS）	MOL002644	Phellopterin	T-cell receptor beta chain C region	P01850	TRBC1	0.039
Radix Saposhnikoviae（RS）	MOL002644	Phellopterin	Beta-2-microglobulin	P61769	B2M	0.039
Radix Saposhnikoviae（RS）	MOL002644	Phellopterin	Tripartite motif-containing protein 13	O60858	TRIM13	0.04
Radix Saposhnikoviae（RS）	MOL002644	Phellopterin	Amine oxidase [flavin-containing] B	P27338	MAOB	0.04
Radix Saposhnikoviae（RS）	MOL002644	Phellopterin	3-phosphoinositide-dependent protein kinase 1	O15530	PDPK1	0.041
Radix Saposhnikoviae（RS）	MOL002644	Phellopterin	cGMP-specific 3',5'-cyclic phosphodiesterase	O76074	PDE5A	0.041
Radix Saposhnikoviae（RS）	MOL002644	Phellopterin	Adenosine deaminase	P00813	ADA	0.041
Radix Saposhnikoviae（RS）	MOL002644	Phellopterin	Proto-oncogene tyrosine-protein kinase LCK	P06239	LCK	0.041
Radix Saposhnikoviae（RS）	MOL002644	Phellopterin	Tyrosine-protein kinase CSK	P41240	CSK	0.041
Radix Saposhnikoviae（RS）	MOL002644	Phellopterin	Tyrosine-protein kinase ZAP-70	P43403	ZAP70	0.041
Radix Saposhnikoviae（RS）	MOL002644	Phellopterin	Tyrosine-protein kinase SYK	P43405	SYK	0.041
Radix Saposhnikoviae（RS）	MOL002644	Phellopterin	Protein kinase C theta type	Q04759	PRKCQ	0.041
Radix Saposhnikoviae（RS）	MOL002644	Phellopterin	Tyrosine-protein kinase ITK/TSK	Q08881	ITK	0.041
Radix Saposhnikoviae（RS）	MOL002644	Phellopterin	Prostaglandin reductase 1	Q14914	PTGR1	0.041
Radix Saposhnikoviae（RS）	MOL002644	Phellopterin	DNA topoisomerase 2-alpha	P11388	TOP2A	0.042
Radix Saposhnikoviae（RS）	MOL002644	Phellopterin	Calcium-activated potassium channel subunit alpha 1	Q12791	KCNMA1	0.042
Radix Saposhnikoviae（RS）	MOL002644	Phellopterin	85 kDa calcium-independent phospholipase A2	O60733	PLA2G6	0.043
Radix Saposhnikoviae（RS）	MOL002644	Phellopterin	Cytosolic phospholipase A2	P47712	PLA2G4A	0.043
Radix Saposhnikoviae（RS）	MOL002644	Phellopterin	High affinity cAMP-specific and IBMX-insensitive 3',5'-cyclic phosphodiesterase 8A	O60658	PDE8A	0.044
Radix Saposhnikoviae（RS）	MOL002644	Phellopterin	6-phosphogluconate dehydrogenase, decarboxylating	P52209	PGD	0.044
Radix Saposhnikoviae（RS）	MOL002644	Phellopterin	High-affinity cAMP-specific 3',5'-cyclic phosphodiesterase 7A	Q13946	PDE7A	0.044
Radix Saposhnikoviae（RS）	MOL002644	Phellopterin	cAMP-specific 3',5'-cyclic phosphodiesterase 7B	Q9NP56	PDE7B	0.044
Radix Saposhnikoviae（RS）	MOL002644	Phellopterin	Translocator protein	P30536	TSPO	0.045
Radix Saposhnikoviae（RS）	MOL002644	Phellopterin	cGMP-inhibited 3',5'-cyclic phosphodiesterase A	Q14432	PDE3A	0.045
Radix Saposhnikoviae（RS）	MOL002644	Phellopterin	Neuronal acetylcholine receptor subunit alpha-2	Q15822	CHRNA2	0.045
Radix Saposhnikoviae（RS）	MOL002644	Phellopterin	Gamma-aminobutyric-acid receptor subunit alpha-6	Q16445	GABRA6	0.046
Radix Saposhnikoviae（RS）	MOL002644	Phellopterin	Myeloperoxidase	P05164	MPO	0.048
Radix Saposhnikoviae（RS）	MOL002644	Phellopterin	Eosinophil peroxidase	P11678	EPX	0.048
Radix Saposhnikoviae（RS）	MOL002644	Phellopterin	Calreticulin	P27797	CALR	0.048
Radix Saposhnikoviae（RS）	MOL002644	Phellopterin	Melatonin receptor type 1B	P49286	MTNR1B	0.048
Radix Saposhnikoviae（RS）	MOL002644	Phellopterin	Calmodulin	P62158		0.048
Radix Saposhnikoviae（RS）	MOL002644	Phellopterin	Nuclear receptor ROR-beta	Q92753	RORB	0.048
Radix Saposhnikoviae（RS）	MOL002644	Phellopterin	Voltage-dependent T-type calcium channel subunit alpha-1H	O95180	CACNA1H	0.049
Radix Saposhnikoviae（RS）	MOL002644	Phellopterin	DNA topoisomerase 1	P11387	TOP1	0.051
Radix Saposhnikoviae（RS）	MOL002644	Phellopterin	Peptidyl-prolyl cis-trans isomerase, mitochondrial	P30405	PPIF	0.051
Radix Saposhnikoviae（RS）	MOL002644	Phellopterin	DNA topoisomerase I, mitochondrial	Q969P6	TOP1MT	0.051
Radix Saposhnikoviae（RS）	MOL002644	Phellopterin	4-aminobutyrate aminotransferase, mitochondrial	P80404	ABAT	0.052
Radix Saposhnikoviae（RS）	MOL002644	Phellopterin	Histone deacetylase 9	Q9UKV0	HDAC9	0.052

Radix Saposhnikoviae（RS）	MOL002644	Phellopterin	Lipoic acid synthetase, mitochondrial	O43766	LIAS	0.053
Radix Saposhnikoviae（RS）	MOL002644	Phellopterin	Tumor necrosis factor	P01375	TNF	0.053
Radix Saposhnikoviae（RS）	MOL002644	Phellopterin	Keratin, type II cytoskeletal 7	P08729	KRT7	0.053
Radix Saposhnikoviae（RS）	MOL002644	Phellopterin	Glutathione S-transferase A2	P09210	GSTA2	0.053
Radix Saposhnikoviae（RS）	MOL002644	Phellopterin	Platelet glycoprotein IX	P14770	GP9	0.053
Radix Saposhnikoviae（RS）	MOL002644	Phellopterin	Lipoyltransferase 1, mitochondrial	Q9Y234	LIPT1	0.053
Radix Saposhnikoviae（RS）	MOL002644	Phellopterin	Carboxypeptidase A1	P15085	CPA1	0.056
Radix Saposhnikoviae（RS）	MOL002644	Phellopterin	Gamma-aminobutyric-acid receptor subunit beta-3	P28472	GABRB3	0.056
Radix Saposhnikoviae（RS）	MOL002644	Phellopterin	Sodium-dependent serotonin transporter	P31645	SLC6A4	0.056
Radix Saposhnikoviae（RS）	MOL002644	Phellopterin	Gamma-aminobutyric-acid receptor subunit beta-2	P47870	GABRB2	0.056
Radix Saposhnikoviae（RS）	MOL002644	Phellopterin	Nuclear receptor coactivator 2	Q15596	NCOA2	0.056
Radix Saposhnikoviae（RS）	MOL002644	Phellopterin	Gamma-aminobutyric acid receptor subunit theta	Q9UN88	GABRQ	0.056
Radix Saposhnikoviae（RS）	MOL002644	Phellopterin	Gamma-aminobutyric-acid receptor subunit alpha-4	P48169	GABRA4	0.057
Radix Saposhnikoviae（RS）	MOL002644	Phellopterin	Alcohol dehydrogenase [NADP+]	P14550	AKR1A1	0.058
Radix Saposhnikoviae（RS）	MOL002644	Phellopterin	Phospholipase A2, membrane associated	P14555	PLA2G2A	0.059
Radix Saposhnikoviae（RS）	MOL002644	Phellopterin	Lactoylglutathione lyase	Q04760	GLO1	0.059
Radix Saposhnikoviae（RS）	MOL002644	Phellopterin	Prostaglandin reductase 2	Q8N8N7	PTGR2	0.059
Radix Saposhnikoviae（RS）	MOL002644	Phellopterin	Mineralocorticoid receptor	P08235	NR3C2	0.06
Radix Saposhnikoviae（RS）	MOL002644	Phellopterin	Mitogen-activated protein kinase 3	P27361	MAPK3	0.06
Radix Saposhnikoviae（RS）	MOL002644	Phellopterin	Peroxisome proliferator-activated receptor delta	Q03181	PPARD	0.06
Radix Saposhnikoviae（RS）	MOL002644	Phellopterin	Phospholipase A2	P04054	PLA2G1B	0.061
Radix Saposhnikoviae（RS）	MOL002644	Phellopterin	Methionine aminopeptidase 1	P53582	METAP1	0.064
Radix Saposhnikoviae（RS）	MOL002644	Phellopterin	Glutamate [NMDA] receptor subunit epsilon-4	O15399	GRIN2D	0.066
Radix Saposhnikoviae（RS）	MOL002644	Phellopterin	Glutamate [NMDA] receptor subunit 3B	O60391	GRIN3B	0.066
Radix Saposhnikoviae（RS）	MOL002644	Phellopterin	ATP synthase subunit beta, mitochondrial	P06576	ATP5F1B	0.066
Radix Saposhnikoviae（RS）	MOL002644	Phellopterin	Tyrosine-protein kinase HCK	P08631	HCK	0.066
Radix Saposhnikoviae（RS）	MOL002644	Phellopterin	Carbonic anhydrase 4	P22748	CA4	0.066
Radix Saposhnikoviae（RS）	MOL002644	Phellopterin	ATP synthase subunit alpha, mitochondrial	P25705	ATP5F1A	0.066
Radix Saposhnikoviae（RS）	MOL002644	Phellopterin	Mitogen-activated protein kinase 8	P45983	MAPK8	0.066
Radix Saposhnikoviae（RS）	MOL002644	Phellopterin	Phosphatidylinositol-4,5-bisphosphate 3-kinase catalytic subunit gamma isoform	P48736	PIK3CG	0.066
Radix Saposhnikoviae（RS）	MOL002644	Phellopterin	Mitogen-activated protein kinase 10	P53779	MAPK10	0.066
Radix Saposhnikoviae（RS）	MOL002644	Phellopterin	Prothrombin	P00734	F2	0.067
Radix Saposhnikoviae（RS）	MOL002644	Phellopterin	Prolactin receptor	P16471	PRLR	0.067
Radix Saposhnikoviae（RS）	MOL002644	Phellopterin	Tyrosyl-tRNA synthetase, cytoplasmic	P54577	YARS	0.068
Radix Saposhnikoviae（RS）	MOL002644	Phellopterin	Peptidyl-prolyl cis-trans isomerase NIMA-interacting 1	Q13526	PIN1	0.068
Radix Saposhnikoviae（RS）	MOL002644	Phellopterin	Glutamate [NMDA] receptor subunit zeta-1	Q05586	GRIN1	0.069
Radix Saposhnikoviae（RS）	MOL002644	Phellopterin	Glutamate [NMDA] receptor subunit epsilon-1	Q12879	GRIN2A	0.071
Radix Saposhnikoviae（RS）	MOL002644	Phellopterin	Glutamate [NMDA] receptor subunit epsilon-2	Q13224	GRIN2B	0.071
Radix Saposhnikoviae（RS）	MOL002644	Phellopterin	Glutamate [NMDA] receptor subunit epsilon-3	Q14957	GRIN2C	0.071
Radix Saposhnikoviae（RS）	MOL002644	Phellopterin	Proto-oncogene serine/threonine-protein kinase Pim-1	P11309	PIM1	0.079
Radix Saposhnikoviae（RS）	MOL002644	Phellopterin	D-HSCDK2	O75100	CA11	0.08
Radix Saposhnikoviae（RS）	MOL002644	Phellopterin	Cell division control protein 2 homolog	P06493	CDK1	0.08
Radix Saposhnikoviae（RS）	MOL002644	Phellopterin	Ribosyldihydronicotinamide dehydrogenase [quinone]	P16083	NQO2	0.08
Radix Saposhnikoviae（RS）	MOL002644	Phellopterin	D(4) dopamine receptor	P21917	DRD4	0.085
Radix Saposhnikoviae（RS）	MOL002644	Phellopterin	D(1B) dopamine receptor	P21918	DRD5	0.085
Radix Saposhnikoviae（RS）	MOL002644	Phellopterin	Muscarinic acetylcholine receptor M2	P08172	CHRM2	0.086
Radix Saposhnikoviae（RS）	MOL002644	Phellopterin	Alpha-1D adrenergic receptor	P25100	ADRA1D	0.086

Radix Saposhnikoviae（RS）	MOL002644	Phellopterin	Glycogen synthase kinase-3 beta	P49841	GSK3B	0.086
Radix Saposhnikoviae（RS）	MOL002644	Phellopterin	Beta-2 adrenergic receptor	P07550	ADRB2	0.087
Radix Saposhnikoviae（RS）	MOL002644	Phellopterin	Muscarinic acetylcholine receptor M5	P08912	CHRM5	0.087
Radix Saposhnikoviae（RS）	MOL002644	Phellopterin	Tryptophanyl-tRNA synthetase, mitochondrial	Q9UGM6	WARS2	0.088
Radix Saposhnikoviae（RS）	MOL002644	Phellopterin	Beta-1 adrenergic receptor	P08588	ADRB1	0.089
Radix Saposhnikoviae（RS）	MOL002644	Phellopterin	Gamma-aminobutyric acid receptor subunit gamma-2	P18507	GABRG2	0.09
Radix Saposhnikoviae（RS）	MOL002644	Phellopterin	MAP kinase-activated protein kinase 2	P49137	MAPKAPK2	0.091
Radix Saposhnikoviae（RS）	MOL002644	Phellopterin	cAMP and cAMP-inhibited cGMP 3',5'-cyclic phosphodiesterase 10A	Q9Y233	PDE10A	0.096
Radix Saposhnikoviae（RS）	MOL002644	Phellopterin	Gamma-aminobutyric acid receptor subunit rho-3	A8MPY1	GABRR3	0.101
Radix Saposhnikoviae（RS）	MOL002644	Phellopterin	Gamma-aminobutyric acid receptor subunit pi	O00591	GABRP	0.101
Radix Saposhnikoviae（RS）	MOL002644	Phellopterin	Gamma-aminobutyric-acid receptor subunit rho-1	P24046	GABRR1	0.101
Radix Saposhnikoviae（RS）	MOL002644	Phellopterin	Gamma-aminobutyric acid receptor subunit rho-2	P28476	GABRR2	0.101
Radix Saposhnikoviae（RS）	MOL002644	Phellopterin	Gamma-aminobutyric acid receptor subunit epsilon	P78334	GABRE	0.101
Radix Saposhnikoviae（RS）	MOL002644	Phellopterin	Gamma-aminobutyric acid receptor subunit gamma-1	Q8N1C3	GABRG1	0.101
Radix Saposhnikoviae（RS）	MOL002644	Phellopterin	Gamma-aminobutyric acid receptor subunit gamma-3	Q99928	GABRG3	0.101
Radix Saposhnikoviae（RS）	MOL002644	Phellopterin	Melatonin receptor type 1A	P48039	MTNR1A	0.103
Radix Saposhnikoviae（RS）	MOL002644	Phellopterin	Potassium voltage-gated channel subfamily H member 2	Q12809	KCNH2	0.107
Radix Saposhnikoviae（RS）	MOL002644	Phellopterin	Protein farnesyltransferase/geranylgeranyltransferase type I alpha subunit	P49354	FNTA	0.11
Radix Saposhnikoviae（RS）	MOL002644	Phellopterin	Gamma-aminobutyric acid receptor subunit delta	O14764	GABRD	0.112
Radix Saposhnikoviae（RS）	MOL002644	Phellopterin	Estradiol 17-beta-dehydrogenase 1	P14061	HSD17B1	0.112
Radix Saposhnikoviae（RS）	MOL002644	Phellopterin	Gamma-aminobutyric-acid receptor subunit beta-1	P18505	GABRB1	0.112
Radix Saposhnikoviae（RS）	MOL002644	Phellopterin	Endothelin-1 receptor	P25101	EDNRA	0.112
Radix Saposhnikoviae（RS）	MOL002644	Phellopterin	Gamma-aminobutyric-acid receptor subunit alpha-5	P31644	GABRA5	0.112
Radix Saposhnikoviae（RS）	MOL002644	Phellopterin	Gamma-aminobutyric-acid receptor subunit alpha-3	P34903	GABRA3	0.112
Radix Saposhnikoviae（RS）	MOL002644	Phellopterin	Oxysterols receptor LXR-alpha	Q13133	NR1H3	0.112
Radix Saposhnikoviae（RS）	MOL002644	Phellopterin	Calcium/calmodulin-dependent protein kinase type II alpha chain	Q9UQM7	CAMK2A	0.112
Radix Saposhnikoviae（RS）	MOL002644	Phellopterin	ATP-sensitive inward rectifier potassium channel 1	P48048	KCNJ1	0.114
Radix Saposhnikoviae（RS）	MOL002644	Phellopterin	Lysozyme C	P61626	LYZ	0.114
Radix Saposhnikoviae（RS）	MOL002644	Phellopterin	5-hydroxytryptamine 1D receptor	P28221	HTR1D	0.115
Radix Saposhnikoviae（RS）	MOL002644	Phellopterin	5-hydroxytryptamine 1B receptor	P28222	HTR1B	0.115
Radix Saposhnikoviae（RS）	MOL002644	Phellopterin	Cannabinoid receptor 2	P34972	CNR2	0.117
Radix Saposhnikoviae（RS）	MOL002644	Phellopterin	D(3) dopamine receptor	P35462	DRD3	0.118
Radix Saposhnikoviae（RS）	MOL002644	Phellopterin	5-hydroxytryptamine 2B receptor	P41595	HTR2B	0.118
Radix Saposhnikoviae（RS）	MOL002644	Phellopterin	Tubulin beta-1 chain	Q9H4B7	TUBB1	0.118
Radix Saposhnikoviae（RS）	MOL002644	Phellopterin	Carbonic anhydrase 1	P00915	CA1	0.119
Radix Saposhnikoviae（RS）	MOL002644	Phellopterin	Muscarinic acetylcholine receptor M4	P08173	CHRM4	0.119
Radix Saposhnikoviae（RS）	MOL002644	Phellopterin	Cannabinoid receptor 1	P21554	CNR1	0.12
Radix Saposhnikoviae（RS）	MOL002644	Phellopterin	Tubulin alpha-3 chain	Q71U36	TUBA1A	0.12
Radix Saposhnikoviae（RS）	MOL002644	Phellopterin	Dual specificity protein kinase CLK1	P49759	CLK1	0.121
Radix Saposhnikoviae（RS）	MOL002644	Phellopterin	Liver carboxylesterase 1	P23141	CES1	0.122
Radix Saposhnikoviae（RS）	MOL002644	Phellopterin	Ig gamma-1 chain C region	P01857	IGHG1	0.123
Radix Saposhnikoviae（RS）	MOL002644	Phellopterin	Hepatocyte growth factor receptor	P08581	MET	0.123
Radix Saposhnikoviae（RS）	MOL002644	Phellopterin	Cytochrome P450 19A1	P11511	CYP19A1	0.123
Radix Saposhnikoviae（RS）	MOL002644	Phellopterin	cAMP-dependent protein kinase inhibitor alpha	P61925	PKIA	0.123
Radix Saposhnikoviae（RS）	MOL002644	Phellopterin	Rho-associated protein kinase 1	Q13464	ROCK1	0.123
Radix Saposhnikoviae（RS）	MOL002644	Phellopterin	Cell division protein kinase 5	Q00535	CDK5	0.124

Radix Saposhnikoviae（RS）	MOL002644	Phellopterin	Trypsin-1	P07477	PRSS1	0.125
Radix Saposhnikoviae（RS）	MOL002644	Phellopterin	Peroxisome proliferator-activated receptor gamma	P37231	PPARG	0.125
Radix Saposhnikoviae（RS）	MOL002644	Phellopterin	Egl nine homolog 1	Q9GZT9	EGLN1	0.125
Radix Saposhnikoviae（RS）	MOL002644	Phellopterin	Serine/threonine-protein kinase 6	O14965	AURKA	0.126
Radix Saposhnikoviae（RS）	MOL002644	Phellopterin	Thromboxane A2 receptor	P21731	TBXA2R	0.126
Radix Saposhnikoviae（RS）	MOL002644	Phellopterin	S-methyl-5-thioadenosine phosphorylase	Q13126	MTAP	0.126
Radix Saposhnikoviae（RS）	MOL002644	Phellopterin	Sodium-dependent noradrenaline transporter	P23975	SLC6A2	0.127
Radix Saposhnikoviae（RS）	MOL002644	Phellopterin	Aldose reductase	P15121	AKR1B1	0.128
Radix Saposhnikoviae（RS）	MOL002644	Phellopterin	Ig kappa chain V-II region RPMI 6410	P06310	IGKV2-30	0.13
Radix Saposhnikoviae（RS）	MOL002644	Phellopterin	DNA polymerase kappa	Q9UBT6	POLK	0.131
Radix Saposhnikoviae（RS）	MOL002644	Phellopterin	Triosephosphate isomerase	P60174	TPI1	0.133
Radix Saposhnikoviae（RS）	MOL002644	Phellopterin	Glutamate [NMDA] receptor subunit 3A	Q8TCU5	GRIN3A	0.133
Radix Saposhnikoviae（RS）	MOL002644	Phellopterin	Progesterone receptor	P06401	PGR	0.134
Radix Saposhnikoviae（RS）	MOL002644	Phellopterin	Glycogen phosphorylase, muscle form	P11217	PYGM	0.134
Radix Saposhnikoviae（RS）	MOL002644	Phellopterin	Hydroxyacid oxidase 1	Q9UJM8	HAO1	0.135
Radix Saposhnikoviae（RS）	MOL002644	Phellopterin	Androgen receptor	P10275	AR	0.141
Radix Saposhnikoviae（RS）	MOL002644	Phellopterin	Gamma-aminobutyric-acid receptor subunit alpha-1	P14867	GABRA1	0.142
Radix Saposhnikoviae（RS）	MOL002644	Phellopterin	Gamma-aminobutyric-acid receptor subunit alpha-2	P47869	GABRA2	0.142
Radix Saposhnikoviae（RS）	MOL002644	Phellopterin	C-jun-amino-terminal kinase-interacting protein 1	Q9UQF2	MAPK8IP1	0.142
Radix Saposhnikoviae（RS）	MOL002644	Phellopterin	Toll-like receptor 7	Q9NYK1	TLR7	0.144
Radix Saposhnikoviae（RS）	MOL002644	Phellopterin	Carbonic anhydrase 2	P00918	CA2	0.145
Radix Saposhnikoviae（RS）	MOL002644	Phellopterin	Alpha-2A adrenergic receptor	P08913	ADRA2A	0.15
Radix Saposhnikoviae（RS）	MOL002644	Phellopterin	Alpha-2B adrenergic receptor	P18089	ADRA2B	0.15
Radix Saposhnikoviae（RS）	MOL002644	Phellopterin	Alpha-2C adrenergic receptor	P18825	ADRA2C	0.15
Radix Saposhnikoviae（RS）	MOL002644	Phellopterin	Alpha-1B adrenergic receptor	P35368	ADRA1B	0.151
Radix Saposhnikoviae（RS）	MOL002644	Phellopterin	cAMP-specific 3',5'-cyclic phosphodiesterase 4C	Q08493	PDE4C	0.157
Radix Saposhnikoviae（RS）	MOL002644	Phellopterin	Sodium channel protein type 5 subunit alpha	Q14524	SCN5A	0.17
Radix Saposhnikoviae（RS）	MOL002644	Phellopterin	5-hydroxytryptamine 2C receptor	P28335	HTR2C	0.183
Radix Saposhnikoviae（RS）	MOL002644	Phellopterin	Muscarinic acetylcholine receptor M3	P20309	CHRM3	0.184
Radix Saposhnikoviae（RS）	MOL002644	Phellopterin	Peroxisome proliferator-activated receptor alpha	Q07869	PPARA	0.201
Radix Saposhnikoviae（RS）	MOL002644	Phellopterin	Hemoglobin subunit alpha	P69905	HBA1	0.207
Radix Saposhnikoviae（RS）	MOL002644	Phellopterin	D(1A) dopamine receptor	P21728	DRD1	0.213
Radix Saposhnikoviae（RS）	MOL002644	Phellopterin	Alpha-1A adrenergic receptor	P35348	ADRA1A	0.215
Radix Saposhnikoviae（RS）	MOL002644	Phellopterin	Muscarinic acetylcholine receptor M1	P11229	CHRM1	0.218
Radix Saposhnikoviae（RS）	MOL002644	Phellopterin	Delta-type opioid receptor	P41143	OPRD1	0.241
Radix Saposhnikoviae（RS）	MOL002644	Phellopterin	Nitric oxide synthase, inducible	P35228	NOS2	0.245
Radix Saposhnikoviae（RS）	MOL002644	Phellopterin	Mitogen-activated protein kinase 14	Q16539	MAPK14	0.253
Radix Saposhnikoviae（RS）	MOL002644	Phellopterin	cAMP-dependent protein kinase catalytic subunit alpha	P17612	PRKACA	0.255
Radix Saposhnikoviae（RS）	MOL002644	Phellopterin	5-hydroxytryptamine 1A receptor	P08908	HTR1A	0.257
Radix Saposhnikoviae（RS）	MOL002644	Phellopterin	RAC-alpha serine/threonine-protein kinase	P31749	AKT1	0.258
Radix Saposhnikoviae（RS）	MOL002644	Phellopterin	Proto-oncogene tyrosine-protein kinase Src	P12931	SRC	0.266
Radix Saposhnikoviae（RS）	MOL002644	Phellopterin	cAMP-specific 3',5'-cyclic phosphodiesterase 4D	Q08499	PDE4D	0.274
Radix Saposhnikoviae（RS）	MOL002644	Phellopterin	Histamine H1 receptor	P35367	HRH1	0.291
Radix Saposhnikoviae（RS）	MOL002644	Phellopterin	5-hydroxytryptamine 2A receptor	P28223	HTR2A	0.311
Radix Saposhnikoviae（RS）	MOL002644	Phellopterin	cAMP-specific 3',5'-cyclic phosphodiesterase 4B	Q07343	PDE4B	0.329
Radix Saposhnikoviae（RS）	MOL002644	Phellopterin	Kappa-type opioid receptor	P41145	OPRK1	0.33
Radix Saposhnikoviae（RS）	MOL002644	Phellopterin	D(2) dopamine receptor	P14416	DRD2	0.343

Radix Saposhnikoviae（RS）	MOL002644	Phellopterin	Cell division protein kinase 2	P24941	CDK2	0.344
Radix Saposhnikoviae（RS）	MOL002644	Phellopterin	cAMP-specific 3',5'-cyclic phosphodiesterase 4A	P27815	PDE4A	0.382
Radix Saposhnikoviae（RS）	MOL002644	Phellopterin	Mu-type opioid receptor	P35372	OPRM1	0.48
Radix Saposhnikoviae（RS）	MOL002644	Phellopterin	Prostaglandin G/H synthase 1	P23219	PTGS1	0.482
Radix Saposhnikoviae（RS）	MOL002644	Phellopterin	Nuclear receptor coactivator 1	Q15788	NCOA1	0.514
Radix Saposhnikoviae（RS）	MOL002644	Phellopterin	Estrogen receptor beta	Q92731	ESR2	0.684
Radix Saposhnikoviae（RS）	MOL002644	Phellopterin	Cyclin-A2	P20248	CCNA2	0.828
Radix Saposhnikoviae（RS）	MOL002644	Phellopterin	Prostaglandin G/H synthase 2	P35354	PTGS2	0.909
Radix Saposhnikoviae（RS）	MOL002644	Phellopterin	Estrogen receptor	P03372	ESR1	1
Radix Saposhnikoviae（RS）	MOL003588	Prangenidin	Acetylcholine receptor subunit alpha	P02708	CHRNA1	0.001
Radix Saposhnikoviae（RS）	MOL003588	Prangenidin	Cholinesterase	P06276	BCHE	0.001
Radix Saposhnikoviae（RS）	MOL003588	Prangenidin	Acetylcholine receptor subunit gamma	P07510	CHRNG	0.001
Radix Saposhnikoviae（RS）	MOL003588	Prangenidin	Cytochrome b-c1 complex subunit 6, mitochondrial	P07919	UQCRH	0.001
Radix Saposhnikoviae（RS）	MOL003588	Prangenidin	Acetylcholine receptor subunit beta	P11230	CHRNB1	0.001
Radix Saposhnikoviae（RS）	MOL003588	Prangenidin	Neuronal acetylcholine receptor subunit beta-2	P17787	CHRNB2	0.001
Radix Saposhnikoviae（RS）	MOL003588	Prangenidin	Acetylcholinesterase	P22303	ACHE	0.001
Radix Saposhnikoviae（RS）	MOL003588	Prangenidin	Neuronal acetylcholine receptor subunit alpha-5	P30532	CHRNA5	0.001
Radix Saposhnikoviae（RS）	MOL003588	Prangenidin	Neuronal acetylcholine receptor subunit beta-4	P30926	CHRNB4	0.001
Radix Saposhnikoviae（RS）	MOL003588	Prangenidin	Acetylcholine receptor subunit epsilon	Q04844	CHRNE	0.001
Radix Saposhnikoviae（RS）	MOL003588	Prangenidin	Neuronal acetylcholine receptor subunit beta-3	Q05901	CHRNB3	0.001
Radix Saposhnikoviae（RS）	MOL003588	Prangenidin	Acetylcholine receptor subunit delta	Q07001	CHRND	0.001
Radix Saposhnikoviae（RS）	MOL003588	Prangenidin	Neuronal acetylcholine receptor subunit alpha-6	Q15825	KCNJ8	0.001
Radix Saposhnikoviae（RS）	MOL003588	Prangenidin	Neuronal acetylcholine receptor subunit alpha-10	Q9GZZ6	CHRNA10	0.001
Radix Saposhnikoviae（RS）	MOL003588	Prangenidin	Neuronal acetylcholine receptor subunit alpha-9	Q9UGM1	CHRNA9	0.001
Radix Saposhnikoviae（RS）	MOL003588	Prangenidin	Cytochrome b-c1 complex subunit 7	P14927	UQCRB	0.002
Radix Saposhnikoviae（RS）	MOL003588	Prangenidin	Succinate dehydrogenase [ubiquinone] iron-sulfur subunit, mitochondrial	P21912	SDHB	0.003
Radix Saposhnikoviae（RS）	MOL003588	Prangenidin	Succinate dehydrogenase [ubiquinone] flavoprotein subunit, mitochondrial	P31040	SDHA	0.003
Radix Saposhnikoviae（RS）	MOL003588	Prangenidin	Succinate dehydrogenase cytochrome b560 subunit, mitochondrial	Q99643	SDHC	0.003
Radix Saposhnikoviae（RS）	MOL003588	Prangenidin	Translocator protein	P30536	TSPO	0.005
Radix Saposhnikoviae（RS）	MOL003588	Prangenidin	Sodium/hydrogen exchanger 1	P19634	SLC9A1	0.006
Radix Saposhnikoviae（RS）	MOL003588	Prangenidin	Amiloride-sensitive amine oxidase [copper-containing]	P19801	AOC1	0.006
Radix Saposhnikoviae（RS）	MOL003588	Prangenidin	Amiloride-sensitive sodium channel subunit delta	P51172	SCNN1D	0.006
Radix Saposhnikoviae（RS）	MOL003588	Prangenidin	Amiloride-sensitive cation channel 2, neuronal	P78348	ASIC1	0.006
Radix Saposhnikoviae（RS）	MOL003588	Prangenidin	Amiloride-sensitive cation channel 1, neuronal	Q16515	ASIC2	0.006
Radix Saposhnikoviae（RS）	MOL003588	Prangenidin	Glutathione S-transferase P	P09211	GSTP1	0.007
Radix Saposhnikoviae（RS）	MOL003588	Prangenidin	Sphingomyelin phosphodiesterase	P17405	SMPD1	0.007
Radix Saposhnikoviae（RS）	MOL003588	Prangenidin	Histamine H2 receptor	P25021	HRH2	0.007
Radix Saposhnikoviae（RS）	MOL003588	Prangenidin	Acetyl-CoA carboxylase 2	O00763	ACACB	0.008
Radix Saposhnikoviae（RS）	MOL003588	Prangenidin	Potassium voltage-gated channel subfamily KQT member 2	O43526	KCNQ2	0.008
Radix Saposhnikoviae（RS）	MOL003588	Prangenidin	Beta-nerve growth factor	P01138	NGF	0.008
Radix Saposhnikoviae（RS）	MOL003588	Prangenidin	Protein S100-B	P04271	S100B	0.008
Radix Saposhnikoviae（RS）	MOL003588	Prangenidin	High affinity nerve growth factor receptor	P04629	NTRK1	0.008
Radix Saposhnikoviae（RS）	MOL003588	Prangenidin	Adenine phosphoribosyltransferase	P07741	APRT	0.008
Radix Saposhnikoviae（RS）	MOL003588	Prangenidin	Protein S100-A1	P23297	S100A1	0.008
Radix Saposhnikoviae（RS）	MOL003588	Prangenidin	Low molecular weight phosphotyrosine protein phosphatase	P24666	ACP1	0.008
Radix Saposhnikoviae（RS）	MOL003588	Prangenidin	Neuronal acetylcholine receptor subunit alpha-3	P32297	CHRNA3	0.008
Radix Saposhnikoviae（RS）	MOL003588	Prangenidin	Serine/threonine-protein kinase SRPK2	P78362	SRPK2	0.008

Radix Saposhnikoviae（RS）	MOL003588	Prangenidin	Potassium voltage-gated channel subfamily A member 1	Q09470	KCNA1	0.008
Radix Saposhnikoviae（RS）	MOL003588	Prangenidin	S-methyl-5-thioadenosine phosphorylase	Q13126	MTAP	0.008
Radix Saposhnikoviae（RS）	MOL003588	Prangenidin	BDNF/NT-3 growth factors receptor	Q16620	NTRK2	0.008
Radix Saposhnikoviae（RS）	MOL003588	Prangenidin	Opioid receptor, sigma 1	Q5T1J1	SIGMAR1	0.008
Radix Saposhnikoviae（RS）	MOL003588	Prangenidin	Sigma 1-type opioid receptor	Q99720	SIGMAR1	0.008
Radix Saposhnikoviae（RS）	MOL003588	Prangenidin	Peroxisomal trans-2-enoyl-CoA reductase	Q9BY49	PECR	0.008
Radix Saposhnikoviae（RS）	MOL003588	Prangenidin	Carbonic anhydrase 12	O43570	CA12	0.009
Radix Saposhnikoviae（RS）	MOL003588	Prangenidin	85 kDa calcium-independent phospholipase A2	O60733	PLA2G6	0.009
Radix Saposhnikoviae（RS）	MOL003588	Prangenidin	Interleukin-3	P08700	IL3	0.009
Radix Saposhnikoviae（RS）	MOL003588	Prangenidin	Cytosolic phospholipase A2	P47712	PLA2G4A	0.009
Radix Saposhnikoviae（RS）	MOL003588	Prangenidin	Sodium/potassium-transporting ATPase gamma chain	P54710	FXYD2	0.009
Radix Saposhnikoviae（RS）	MOL003588	Prangenidin	Solute carrier family 12 member 3	P55017	SLC12A3	0.009
Radix Saposhnikoviae（RS）	MOL003588	Prangenidin	Solute carrier family 12 member 1	Q13621	SLC12A1	0.009
Radix Saposhnikoviae（RS）	MOL003588	Prangenidin	ATP-sensitive inward rectifier potassium channel 11	Q14654	KCNJ11	0.009
Radix Saposhnikoviae（RS）	MOL003588	Prangenidin	Carbonic anhydrase 9	Q16790	CA9	0.009
Radix Saposhnikoviae（RS）	MOL003588	Prangenidin	Cytochrome b-c1 complex subunit 8	O14949	UQCRQ	0.01
Radix Saposhnikoviae（RS）	MOL003588	Prangenidin	Cytochrome b-c1 complex subunit 10	O14957	UQCR11	0.01
Radix Saposhnikoviae（RS）	MOL003588	Prangenidin	Cytochrome b	P00156	MT-CYB	0.01
Radix Saposhnikoviae（RS）	MOL003588	Prangenidin	Cytochrome c1, heme protein, mitochondrial	P08574	CYC1	0.01
Radix Saposhnikoviae（RS）	MOL003588	Prangenidin	Elongation factor 2	P13639	EEF2	0.01
Radix Saposhnikoviae（RS）	MOL003588	Prangenidin	Cytochrome b-c1 complex subunit 2, mitochondrial	P22695	UQCRC2	0.01
Radix Saposhnikoviae（RS）	MOL003588	Prangenidin	Ubiquinol-cytochrome-c reductase complex core protein 1, mitochondrial	P31930	UQCRC1	0.01
Radix Saposhnikoviae（RS）	MOL003588	Prangenidin	Cytochrome b-c1 complex subunit Rieske, mitochondrial	P47985	UQCRFS1	0.01
Radix Saposhnikoviae（RS）	MOL003588	Prangenidin	Troponin C, slow skeletal and cardiac muscles	P63316	TNNC1	0.01
Radix Saposhnikoviae（RS）	MOL003588	Prangenidin	Cytochrome b-c1 complex subunit 9	Q9UDW1	UQCR10	0.01
Radix Saposhnikoviae（RS）	MOL003588	Prangenidin	Poly [ADP-ribose] polymerase 3	Q9Y6F1	PARP3	0.01
Radix Saposhnikoviae（RS）	MOL003588	Prangenidin	Substance-P receptor	P25103	TACR1	0.011
Radix Saposhnikoviae（RS）	MOL003588	Prangenidin	6-phosphogluconate dehydrogenase, decarboxylating	P52209	PGD	0.011
Radix Saposhnikoviae（RS）	MOL003588	Prangenidin	cAMP-specific 3',5'-cyclic phosphodiesterase 7B	Q9NP56	PDE7B	0.011
Radix Saposhnikoviae（RS）	MOL003588	Prangenidin	cGMP-dependent 3',5'-cyclic phosphodiesterase	O00408	PDE2A	0.012
Radix Saposhnikoviae（RS）	MOL003588	Prangenidin	High-affinity cGMP-specific 3',5'-cyclic phosphodiesterase 9A	O76083	PDE9A	0.012
Radix Saposhnikoviae（RS）	MOL003588	Prangenidin	Adenosine deaminase	P00813	ADA	0.012
Radix Saposhnikoviae（RS）	MOL003588	Prangenidin	Gamma-aminobutyric-acid receptor subunit beta-3	P28472	GABRB3	0.012
Radix Saposhnikoviae（RS）	MOL003588	Prangenidin	Adenosine A2b receptor	P29275	ADORA2B	0.012
Radix Saposhnikoviae（RS）	MOL003588	Prangenidin	Gamma-aminobutyric-acid receptor subunit beta-2	P47870	GABRB2	0.012
Radix Saposhnikoviae（RS）	MOL003588	Prangenidin	Glutamate [NMDA] receptor subunit epsilon-1	Q12879	GRIN2A	0.012
Radix Saposhnikoviae（RS）	MOL003588	Prangenidin	Glutamate receptor, ionotropic kainate 2	Q13002	GRIK2	0.012
Radix Saposhnikoviae（RS）	MOL003588	Prangenidin	Glutamate [NMDA] receptor subunit epsilon-2	Q13224	GRIN2B	0.012
Radix Saposhnikoviae（RS）	MOL003588	Prangenidin	cGMP-inhibited 3',5'-cyclic phosphodiesterase B	Q13370	PDE3B	0.012
Radix Saposhnikoviae（RS）	MOL003588	Prangenidin	5-hydroxytryptamine 4 receptor	Q13639	HTR4	0.012
Radix Saposhnikoviae（RS）	MOL003588	Prangenidin	Gamma-aminobutyric acid receptor subunit theta	Q9UN88	GABRQ	0.012
Radix Saposhnikoviae（RS）	MOL003588	Prangenidin	cAMP and cAMP-inhibited cGMP 3',5'-cyclic phosphodiesterase 10A	Q9Y233	PDE10A	0.012
Radix Saposhnikoviae（RS）	MOL003588	Prangenidin	Serine/threonine-protein kinase 17B	O94768	STK17B	0.013
Radix Saposhnikoviae（RS）	MOL003588	Prangenidin	ATP synthase subunit gamma, mitochondrial	P36542	ATP5F1C	0.013
Radix Saposhnikoviae（RS）	MOL003588	Prangenidin	Phosphatidylinositol-4,5-bisphosphate 3-kinase catalytic subunit gamma isoform	P48736	PIK3CG	0.013
Radix Saposhnikoviae（RS）	MOL003588	Prangenidin	UDP-glucuronosyltransferase 3A1	Q6NUS8	UGT3A1	0.013
Radix Saposhnikoviae（RS）	MOL003588	Prangenidin	Succinate dehydrogenase [ubiquinone] cytochrome b small subunit, mitochondrial	O14521	SDHD	0.015

Radix Saposhnikoviae（RS）	MOL003588	Prangenidin	Tyrosine 3-monooxygenase	P07101	TH	0.015
Radix Saposhnikoviae（RS）	MOL003588	Prangenidin	Tyrosyl-tRNA synthetase, cytoplasmic	P54577	YARS	0.015
Radix Saposhnikoviae（RS）	MOL003588	Prangenidin	Tyrosyl-tRNA synthetase, mitochondrial	Q9Y2Z4	YARS2	0.015
Radix Saposhnikoviae（RS）	MOL003588	Prangenidin	Pyridoxal kinase	O00764	PDXK	0.016
Radix Saposhnikoviae（RS）	MOL003588	Prangenidin	Tripartite motif-containing protein 13	O60858	TRIM13	0.016
Radix Saposhnikoviae（RS）	MOL003588	Prangenidin	Eukaryotic translation initiation factor 4E	P06730	EIF4E	0.016
Radix Saposhnikoviae（RS）	MOL003588	Prangenidin	Carcinoembryonic antigen-related cell adhesion molecule 5	P06731	CEACAM5	0.016
Radix Saposhnikoviae（RS）	MOL003588	Prangenidin	Cystathionine beta-synthase	P35520	CBS	0.016
Radix Saposhnikoviae（RS）	MOL003588	Prangenidin	Neuronal acetylcholine receptor subunit alpha-2	Q15822	CHRNA2	0.016
Radix Saposhnikoviae（RS）	MOL003588	Prangenidin	Pyridoxal phosphate phosphatase	Q96GD0	PDXP	0.016
Radix Saposhnikoviae（RS）	MOL003588	Prangenidin	Furin	P09958	FURIN	0.017
Radix Saposhnikoviae（RS）	MOL003588	Prangenidin	2,4-dienoyl-CoA reductase, mitochondrial	Q16698	DECR1	0.017
Radix Saposhnikoviae（RS）	MOL003588	Prangenidin	Glycolipid transfer protein	Q9NZD2	GLTP	0.017
Radix Saposhnikoviae（RS）	MOL003588	Prangenidin	Actin-related protein 2/3 complex subunit 1B	O15143	ARPC1B	0.018
Radix Saposhnikoviae（RS）	MOL003588	Prangenidin	Actin-related protein 2/3 complex subunit 2	O15144	ARPC2	0.018
Radix Saposhnikoviae（RS）	MOL003588	Prangenidin	Actin-related protein 2/3 complex subunit 3	O15145	ARPC3	0.018
Radix Saposhnikoviae（RS）	MOL003588	Prangenidin	Retinoic acid receptor gamma-1	P13631	RARG	0.018
Radix Saposhnikoviae（RS）	MOL003588	Prangenidin	Actin-related protein 2/3 complex subunit 4	P59998	ARPC4	0.018
Radix Saposhnikoviae（RS）	MOL003588	Prangenidin	Actin-related protein 3	P61158	ACTR3	0.018
Radix Saposhnikoviae（RS）	MOL003588	Prangenidin	Actin-related protein 2	P61160	ACTR2	0.018
Radix Saposhnikoviae（RS）	MOL003588	Prangenidin	Bile salt sulfotransferase	Q06520	SULT2A1	0.018
Radix Saposhnikoviae（RS）	MOL003588	Prangenidin	Dehydrogenase/reductase SDR family member 8	Q8NBQ5	HSD17B11	0.018
Radix Saposhnikoviae（RS）	MOL003588	Prangenidin	Amiloride-sensitive sodium channel subunit alpha	P37088	SCNN1A	0.019
Radix Saposhnikoviae（RS）	MOL003588	Prangenidin	Neuronal acetylcholine receptor subunit alpha-4	P43681	CHRNA4	0.019
Radix Saposhnikoviae（RS）	MOL003588	Prangenidin	Amiloride-sensitive sodium channel subunit beta	P51168	SCNN1B	0.019
Radix Saposhnikoviae（RS）	MOL003588	Prangenidin	Amiloride-sensitive sodium channel subunit gamma	P51170	SCNN1G	0.019
Radix Saposhnikoviae（RS）	MOL003588	Prangenidin	3-phosphoinositide-dependent protein kinase 1	O15530	PDPK1	0.02
Radix Saposhnikoviae（RS）	MOL003588	Prangenidin	Proto-oncogene tyrosine-protein kinase LCK	P06239	LCK	0.02
Radix Saposhnikoviae（RS）	MOL003588	Prangenidin	Retinoic acid receptor beta	P10826	RARB	0.02
Radix Saposhnikoviae（RS）	MOL003588	Prangenidin	Tyrosine-protein kinase CSK	P41240	CSK	0.02
Radix Saposhnikoviae（RS）	MOL003588	Prangenidin	Tyrosine-protein kinase ZAP-70	P43403	ZAP70	0.02
Radix Saposhnikoviae（RS）	MOL003588	Prangenidin	Tyrosine-protein kinase SYK	P43405	SYK	0.02
Radix Saposhnikoviae（RS）	MOL003588	Prangenidin	Retinoic acid receptor RXR-gamma	P48443	RXRG	0.02
Radix Saposhnikoviae（RS）	MOL003588	Prangenidin	Protein kinase C theta type	Q04759	PRKCQ	0.02
Radix Saposhnikoviae（RS）	MOL003588	Prangenidin	Tyrosine-protein kinase ITK/TSK	Q08881	ITK	0.02
Radix Saposhnikoviae（RS）	MOL003588	Prangenidin	Beta-3 adrenergic receptor	P13945	ADRB3	0.022
Radix Saposhnikoviae（RS）	MOL003588	Prangenidin	Cyclin-dependent kinase 5 activator 1	Q15078	CDK5R1	0.022
Radix Saposhnikoviae（RS）	MOL003588	Prangenidin	Tyrosine-protein kinase JAK2	O60674	JAK2	0.023
Radix Saposhnikoviae（RS）	MOL003588	Prangenidin	Tyrosine-protein kinase JAK1	P23458	JAK1	0.023
Radix Saposhnikoviae（RS）	MOL003588	Prangenidin	Mitogen-activated protein kinase 8	P45983	MAPK8	0.023
Radix Saposhnikoviae（RS）	MOL003588	Prangenidin	Tyrosine-protein kinase JAK3	P52333	JAK3	0.023
Radix Saposhnikoviae（RS）	MOL003588	Prangenidin	Mitogen-activated protein kinase 10	P53779	MAPK10	0.023
Radix Saposhnikoviae（RS）	MOL003588	Prangenidin	Potassium voltage-gated channel subfamily D member 2	Q9NZV8	KCND2	0.023
Radix Saposhnikoviae（RS）	MOL003588	Prangenidin	Potassium voltage-gated channel subfamily D member 3	Q9UK17	KCND3	0.023
Radix Saposhnikoviae（RS）	MOL003588	Prangenidin	C-jun-amino-terminal kinase-interacting protein 1	Q9UQF2	MAPK8IP1	0.023
Radix Saposhnikoviae（RS）	MOL003588	Prangenidin	Protein S100-A12	P80511	S100A12	0.024
Radix Saposhnikoviae（RS）	MOL003588	Prangenidin	Protein S100-A13	Q99584	S100A13	0.024

Radix Saposhnikoviae（RS）	MOL003588	Prangenidin	Prostaglandin reductase 1	Q14914	PTGR1	0.025
Radix Saposhnikoviae（RS）	MOL003588	Prangenidin	D1 dopamine receptor-interacting protein calcyon	Q9NYX4	CALY	0.025
Radix Saposhnikoviae（RS）	MOL003588	Prangenidin	Sodium/potassium-transporting ATPase alpha-1 chain	P05023	ATP1A1	0.026
Radix Saposhnikoviae（RS）	MOL003588	Prangenidin	Carbonic anhydrase 4	P22748	CA4	0.026
Radix Saposhnikoviae（RS）	MOL003588	Prangenidin	Calcium-activated potassium channel subunit alpha 1	Q12791	KCNMA1	0.026
Radix Saposhnikoviae（RS）	MOL003588	Prangenidin	Prothrombin	P00734	F2	0.027
Radix Saposhnikoviae（RS）	MOL003588	Prangenidin	Neuronal acetylcholine receptor subunit alpha-7	P36544	CHRNA7	0.027
Radix Saposhnikoviae（RS）	MOL003588	Prangenidin	Riboflavin kinase	Q969G6	RFK	0.027
Radix Saposhnikoviae（RS）	MOL003588	Prangenidin	Histone deacetylase 8	Q9BY41	HDAC8	0.028
Radix Saposhnikoviae（RS）	MOL003588	Prangenidin	Myeloperoxidase	P05164	MPO	0.029
Radix Saposhnikoviae（RS）	MOL003588	Prangenidin	DNA topoisomerase 2-alpha	P11388	TOP2A	0.029
Radix Saposhnikoviae（RS）	MOL003588	Prangenidin	Eosinophil peroxidase	P11678	EPX	0.029
Radix Saposhnikoviae（RS）	MOL003588	Prangenidin	Calreticulin	P27797	CALR	0.029
Radix Saposhnikoviae（RS）	MOL003588	Prangenidin	RAC-beta serine/threonine-protein kinase	P31751	AKT2	0.029
Radix Saposhnikoviae（RS）	MOL003588	Prangenidin	Melatonin receptor type 1B	P49286	MTNR1B	0.029
Radix Saposhnikoviae（RS）	MOL003588	Prangenidin	Tubulin alpha-1 chain	P68366	TUBA4A	0.029
Radix Saposhnikoviae（RS）	MOL003588	Prangenidin	Rho-associated protein kinase 1	Q13464	ROCK1	0.029
Radix Saposhnikoviae（RS）	MOL003588	Prangenidin	Nuclear receptor ROR-beta	Q92753	RORB	0.029
Radix Saposhnikoviae（RS）	MOL003588	Prangenidin	High affinity cAMP-specific and IBMX-insensitive 3',5'-cyclic phosphodiesterase 8A	O60658	PDE8A	0.03
Radix Saposhnikoviae（RS）	MOL003588	Prangenidin	Gamma-aminobutyric-acid receptor subunit alpha-4	P48169	GABRA4	0.031
Radix Saposhnikoviae（RS）	MOL003588	Prangenidin	Nuclear receptor subfamily 1 group I member 3	Q14994	NR1I3	0.031
Radix Saposhnikoviae（RS）	MOL003588	Prangenidin	Gamma-aminobutyric-acid receptor subunit alpha-6	Q16445	GABRA6	0.031
Radix Saposhnikoviae（RS）	MOL003588	Prangenidin	Peptidyl-prolyl cis-trans isomerase, mitochondrial	P30405	PPIF	0.032
Radix Saposhnikoviae（RS）	MOL003588	Prangenidin	5-hydroxytryptamine 3 receptor	P46098	HTR3A	0.032
Radix Saposhnikoviae（RS）	MOL003588	Prangenidin	cGMP-inhibited 3',5'-cyclic phosphodiesterase A	Q14432	PDE3A	0.032
Radix Saposhnikoviae（RS）	MOL003588	Prangenidin	ATP synthase subunit beta, mitochondrial	P06576	ATP5F1B	0.033
Radix Saposhnikoviae（RS）	MOL003588	Prangenidin	Tyrosine-protein kinase HCK	P08631	HCK	0.033
Radix Saposhnikoviae（RS）	MOL003588	Prangenidin	ATP synthase subunit alpha, mitochondrial	P25705	ATP5F1A	0.033
Radix Saposhnikoviae（RS）	MOL003588	Prangenidin	Tumor necrosis factor	P01375	TNF	0.034
Radix Saposhnikoviae（RS）	MOL003588	Prangenidin	Keratin, type II cytoskeletal 7	P08729	KRT7	0.034
Radix Saposhnikoviae（RS）	MOL003588	Prangenidin	Glutathione S-transferase A2	P09210	GSTA2	0.034
Radix Saposhnikoviae（RS）	MOL003588	Prangenidin	Casein kinase II subunit alpha	P68400	CSNK2A1	0.034
Radix Saposhnikoviae（RS）	MOL003588	Prangenidin	Potassium voltage-gated channel subfamily H member 2	Q12809	KCNH2	0.034
Radix Saposhnikoviae（RS）	MOL003588	Prangenidin	5-hydroxytryptamine 1D receptor	P28221	HTR1D	0.035
Radix Saposhnikoviae（RS）	MOL003588	Prangenidin	5-hydroxytryptamine 1B receptor	P28222	HTR1B	0.035
Radix Saposhnikoviae（RS）	MOL003588	Prangenidin	Methionine aminopeptidase 1	P53582	METAP1	0.035
Radix Saposhnikoviae（RS）	MOL003588	Prangenidin	Alcohol dehydrogenase [NADP+]	P14550	AKR1A1	0.036
Radix Saposhnikoviae（RS）	MOL003588	Prangenidin	Gamma-aminobutyric acid receptor subunit rho-3	A8MPY1	GABRR3	0.037
Radix Saposhnikoviae（RS）	MOL003588	Prangenidin	Gamma-aminobutyric acid receptor subunit pi	O00591	GABRP	0.037
Radix Saposhnikoviae（RS）	MOL003588	Prangenidin	Gamma-aminobutyric acid receptor subunit delta	O14764	GABRD	0.037
Radix Saposhnikoviae（RS）	MOL003588	Prangenidin	Gamma-aminobutyric acid receptor subunit gamma-2	P18507	GABRG2	0.037
Radix Saposhnikoviae（RS）	MOL003588	Prangenidin	Gamma-aminobutyric-acid receptor subunit rho-1	P24046	GABRR1	0.037
Radix Saposhnikoviae（RS）	MOL003588	Prangenidin	Gamma-aminobutyric acid receptor subunit rho-2	P28476	GABRR2	0.037
Radix Saposhnikoviae（RS）	MOL003588	Prangenidin	Gamma-aminobutyric acid receptor subunit epsilon	P78334	GABRE	0.037
Radix Saposhnikoviae（RS）	MOL003588	Prangenidin	Sodium-dependent dopamine transporter	Q01959	SLC6A3	0.037
Radix Saposhnikoviae（RS）	MOL003588	Prangenidin	Gamma-aminobutyric acid receptor subunit gamma-1	Q8N1C3	GABRG1	0.037
Radix Saposhnikoviae（RS）	MOL003588	Prangenidin	Gamma-aminobutyric acid receptor subunit gamma-3	Q99928	GABRG3	0.037

Radix Saposhnikoviae（RS）	MOL003588	Prangenidin	Mitogen-activated protein kinase 3	P27361	MAPK3	0.038
Radix Saposhnikoviae（RS）	MOL003588	Prangenidin	Peroxisome proliferator-activated receptor alpha	Q07869	PPARA	0.038
Radix Saposhnikoviae（RS）	MOL003588	Prangenidin	Gonadotropin-releasing hormone receptor	P30968	GNRHR	0.041
Radix Saposhnikoviae（RS）	MOL003588	Prangenidin	Tubulin beta-2C chain	P68371	TUBB4B	0.041
Radix Saposhnikoviae（RS）	MOL003588	Prangenidin	Gonadotropin-releasing hormone II receptor	Q96P88	GNRHR2	0.041
Radix Saposhnikoviae（RS）	MOL003588	Prangenidin	Group IIE secretory phospholipase A2	Q9NZK7	PLA2G2E	0.041
Radix Saposhnikoviae（RS）	MOL003588	Prangenidin	3 beta-hydroxysteroid dehydrogenase/Delta 5-->4-isomerase type I	P14060	HSD3B1	0.042
Radix Saposhnikoviae（RS）	MOL003588	Prangenidin	3 beta-hydroxysteroid dehydrogenase/Delta 5-->4-isomerase type II	P26439	HSD3B2	0.042
Radix Saposhnikoviae（RS）	MOL003588	Prangenidin	Nitric-oxide synthase, brain	P29475	NOS1	0.043
Radix Saposhnikoviae（RS）	MOL003588	Prangenidin	Gamma-aminobutyric-acid receptor subunit beta-1	P18505	GABRB1	0.044
Radix Saposhnikoviae（RS）	MOL003588	Prangenidin	Retinoic acid receptor alpha	P10276	RARA	0.045
Radix Saposhnikoviae（RS）	MOL003588	Prangenidin	Retinoic acid receptor RXR-beta	P28702	RXRB	0.045
Radix Saposhnikoviae（RS）	MOL003588	Prangenidin	Glutamate [NMDA] receptor subunit 3A	Q8TCU5	GRIN3A	0.045
Radix Saposhnikoviae（RS）	MOL003588	Prangenidin	MAP kinase-activated protein kinase 2	P49137	MAPKAPK2	0.047
Radix Saposhnikoviae（RS）	MOL003588	Prangenidin	Calmodulin	P62158		0.047
Radix Saposhnikoviae（RS）	MOL003588	Prangenidin	Tryptophanyl-tRNA synthetase, mitochondrial	Q9UGM6	WARS2	0.048
Radix Saposhnikoviae（RS）	MOL003588	Prangenidin	Glycogen synthase kinase-3 beta	P49841	GSK3B	0.049
Radix Saposhnikoviae（RS）	MOL003588	Prangenidin	High-affinity cAMP-specific 3',5'-cyclic phosphodiesterase 7A	Q13946	PDE7A	0.049
Radix Saposhnikoviae（RS）	MOL003588	Prangenidin	Sodium channel protein type 10 subunit alpha	Q9Y5Y9	SCN10A	0.049
Radix Saposhnikoviae（RS）	MOL003588	Prangenidin	cGMP-specific 3',5'-cyclic phosphodiesterase	O76074	PDE5A	0.05
Radix Saposhnikoviae（RS）	MOL003588	Prangenidin	D(4) dopamine receptor	P21917	DRD4	0.05
Radix Saposhnikoviae（RS）	MOL003588	Prangenidin	cAMP-specific 3',5'-cyclic phosphodiesterase 4C	Q08493	PDE4C	0.05
Radix Saposhnikoviae（RS）	MOL003588	Prangenidin	D-HSCDK2	O75100	CA11	0.051
Radix Saposhnikoviae（RS）	MOL003588	Prangenidin	Beta-1 adrenergic receptor	P08588	ADRB1	0.052
Radix Saposhnikoviae（RS）	MOL003588	Prangenidin	Death-associated protein kinase 3	O43293	DAPK3	0.054
Radix Saposhnikoviae（RS）	MOL003588	Prangenidin	Gamma-aminobutyric-acid receptor subunit alpha-5	P31644	GABRA5	0.056
Radix Saposhnikoviae（RS）	MOL003588	Prangenidin	Retinoic acid receptor RXR-alpha	P19793	RXRA	0.058
Radix Saposhnikoviae（RS）	MOL003588	Prangenidin	Thiamin pyrophosphokinase 1	Q9H3S4	TPK1	0.062
Radix Saposhnikoviae（RS）	MOL003588	Prangenidin	Glucocorticoid receptor	P04150	NR3C1	0.063
Radix Saposhnikoviae（RS）	MOL003588	Prangenidin	Trypsin-1	P07477	PRSS1	0.063
Radix Saposhnikoviae（RS）	MOL003588	Prangenidin	Gamma-aminobutyric-acid receptor subunit alpha-3	P34903	GABRA3	0.063
Radix Saposhnikoviae（RS）	MOL003588	Prangenidin	Hepatocyte growth factor receptor	P08581	MET	0.064
Radix Saposhnikoviae（RS）	MOL003588	Prangenidin	Proto-oncogene serine/threonine-protein kinase Pim-1	P11309	PIM1	0.064
Radix Saposhnikoviae（RS）	MOL003588	Prangenidin	Alpha-1B adrenergic receptor	P35368	ADRA1B	0.064
Radix Saposhnikoviae（RS）	MOL003588	Prangenidin	Melatonin receptor type 1A	P48039	MTNR1A	0.064
Radix Saposhnikoviae（RS）	MOL003588	Prangenidin	Leukotriene A-4 hydrolase	P09960	LTA4H	0.065
Radix Saposhnikoviae（RS）	MOL003588	Prangenidin	Carnitine O-palmitoyltransferase I, liver isoform	P50416	CPT1A	0.065
Radix Saposhnikoviae（RS）	MOL003588	Prangenidin	Estradiol 17-beta-dehydrogenase 1	P14061	HSD17B1	0.066
Radix Saposhnikoviae（RS）	MOL003588	Prangenidin	Endothelin-1 receptor	P25101	EDNRA	0.066
Radix Saposhnikoviae（RS）	MOL003588	Prangenidin	Lysozyme C	P61626	LYZ	0.066
Radix Saposhnikoviae（RS）	MOL003588	Prangenidin	Nuclear receptor coactivator 2	Q15596	NCOA2	0.067
Radix Saposhnikoviae（RS）	MOL003588	Prangenidin	Ig kappa chain V-II region RPMI 6410	P06310	IGKV2-30	0.068
Radix Saposhnikoviae（RS）	MOL003588	Prangenidin	Phospholipase A2, membrane associated	P14555	PLA2G2A	0.068
Radix Saposhnikoviae（RS）	MOL003588	Prangenidin	Cannabinoid receptor 2	P34972	CNR2	0.068
Radix Saposhnikoviae（RS）	MOL003588	Prangenidin	Gamma-aminobutyric-acid receptor subunit alpha-1	P14867	GABRA1	0.069
Radix Saposhnikoviae（RS）	MOL003588	Prangenidin	Gamma-aminobutyric-acid receptor subunit alpha-2	P47869	GABRA2	0.069
Radix Saposhnikoviae（RS）	MOL003588	Prangenidin	Triosephosphate isomerase	P60174	TPI1	0.07

Radix Saposhnikoviae（RS）	MOL003588	Prangenidin	Ribosyldihydronicotinamide dehydrogenase [quinone]	P16083	NQO2	0.071
Radix Saposhnikoviae（RS）	MOL003588	Prangenidin	Cannabinoid receptor 1	P21554	CNR1	0.071
Radix Saposhnikoviae（RS）	MOL003588	Prangenidin	Membrane copper amine oxidase	Q16853	AOC3	0.071
Radix Saposhnikoviae（RS）	MOL003588	Prangenidin	Cytochrome P450 11B1, mitochondrial	P15538	CYP11B1	0.072
Radix Saposhnikoviae（RS）	MOL003588	Prangenidin	Mitogen-activated protein kinase 14	Q16539	MAPK14	0.072
Radix Saposhnikoviae（RS）	MOL003588	Prangenidin	DNA polymerase kappa	Q9UBT6	POLK	0.072
Radix Saposhnikoviae（RS）	MOL003588	Prangenidin	Amine oxidase [flavin-containing] B	P27338	MAOB	0.073
Radix Saposhnikoviae（RS）	MOL003588	Prangenidin	Dual specificity protein kinase CLK1	P49759	CLK1	0.075
Radix Saposhnikoviae（RS）	MOL003588	Prangenidin	Toll-like receptor 7	Q9NYK1	TLR7	0.075
Radix Saposhnikoviae（RS）	MOL003588	Prangenidin	Carbonic anhydrase 1	P00915	CA1	0.076
Radix Saposhnikoviae（RS）	MOL003588	Prangenidin	Carbonic anhydrase 2	P00918	CA2	0.076
Radix Saposhnikoviae（RS）	MOL003588	Prangenidin	Egl nine homolog 1	Q9GZT9	EGLN1	0.077
Radix Saposhnikoviae（RS）	MOL003588	Prangenidin	D(3) dopamine receptor	P35462	DRD3	0.078
Radix Saposhnikoviae（RS）	MOL003588	Prangenidin	Delta-type opioid receptor	P41143	OPRD1	0.078
Radix Saposhnikoviae（RS）	MOL003588	Prangenidin	Hydroxyacid oxidase 1	Q9UJM8	HAO1	0.079
Radix Saposhnikoviae（RS）	MOL003588	Prangenidin	Cell division control protein 2 homolog	P06493	CDK1	0.081
Radix Saposhnikoviae（RS）	MOL003588	Prangenidin	Beta-2 adrenergic receptor	P07550	ADRB2	0.081
Radix Saposhnikoviae（RS）	MOL003588	Prangenidin	Aldose reductase	P15121	AKR1B1	0.082
Radix Saposhnikoviae（RS）	MOL003588	Prangenidin	Peroxisome proliferator-activated receptor delta	Q03181	PPARD	0.083
Radix Saposhnikoviae（RS）	MOL003588	Prangenidin	cAMP-specific 3',5'-cyclic phosphodiesterase 4D	Q08499	PDE4D	0.088
Radix Saposhnikoviae（RS）	MOL003588	Prangenidin	Tubulin alpha-3 chain	Q71U36	TUBA1A	0.089
Radix Saposhnikoviae（RS）	MOL003588	Prangenidin	Mineralocorticoid receptor	P08235	NR3C2	0.09
Radix Saposhnikoviae（RS）	MOL003588	Prangenidin	Androgen receptor	P10275	AR	0.09
Radix Saposhnikoviae（RS）	MOL003588	Prangenidin	Adenosine A2a receptor	P29274	ADORA2A	0.09
Radix Saposhnikoviae（RS）	MOL003588	Prangenidin	Adenosine A1 receptor	P30542	ADORA1	0.091
Radix Saposhnikoviae（RS）	MOL003588	Prangenidin	Alpha-2C adrenergic receptor	P18825	ADRA2C	0.092
Radix Saposhnikoviae（RS）	MOL003588	Prangenidin	D(1B) dopamine receptor	P21918	DRD5	0.092
Radix Saposhnikoviae（RS）	MOL003588	Prangenidin	Nitric-oxide synthase, endothelial	P29474	NOS3	0.093
Radix Saposhnikoviae（RS）	MOL003588	Prangenidin	5-hydroxytryptamine 2B receptor	P41595	HTR2B	0.093
Radix Saposhnikoviae（RS）	MOL003588	Prangenidin	Alpha-1D adrenergic receptor	P25100	ADRA1D	0.095
Radix Saposhnikoviae（RS）	MOL003588	Prangenidin	cAMP-dependent protein kinase inhibitor alpha	P61925	PKIA	0.1
Radix Saposhnikoviae（RS）	MOL003588	Prangenidin	5-hydroxytryptamine 2C receptor	P28335	HTR2C	0.107
Radix Saposhnikoviae（RS）	MOL003588	Prangenidin	Cell division protein kinase 5	Q00535	CDK5	0.111
Radix Saposhnikoviae（RS）	MOL003588	Prangenidin	Alpha-2B adrenergic receptor	P18089	ADRA2B	0.121
Radix Saposhnikoviae（RS）	MOL003588	Prangenidin	Muscarinic acetylcholine receptor M5	P08912	CHRM5	0.124
Radix Saposhnikoviae（RS）	MOL003588	Prangenidin	Muscarinic acetylcholine receptor M2	P08172	CHRM2	0.125
Radix Saposhnikoviae（RS）	MOL003588	Prangenidin	Hemoglobin subunit alpha	P69905	HBA1	0.126
Radix Saposhnikoviae（RS）	MOL003588	Prangenidin	cAMP-specific 3',5'-cyclic phosphodiesterase 4A	P27815	PDE4A	0.128
Radix Saposhnikoviae（RS）	MOL003588	Prangenidin	Muscarinic acetylcholine receptor M3	P20309	CHRM3	0.139
Radix Saposhnikoviae（RS）	MOL003588	Prangenidin	Sodium-dependent serotonin transporter	P31645	SLC6A4	0.139
Radix Saposhnikoviae（RS）	MOL003588	Prangenidin	RAC-alpha serine/threonine-protein kinase	P31749	AKT1	0.14
Radix Saposhnikoviae（RS）	MOL003588	Prangenidin	Cyclin-A2	P20248	CCNA2	0.141
Radix Saposhnikoviae（RS）	MOL003588	Prangenidin	cAMP-specific 3',5'-cyclic phosphodiesterase 4B	Q07343	PDE4B	0.147
Radix Saposhnikoviae（RS）	MOL003588	Prangenidin	Alpha-2A adrenergic receptor	P08913	ADRA2A	0.151
Radix Saposhnikoviae（RS）	MOL003588	Prangenidin	Muscarinic acetylcholine receptor M4	P08173	CHRM4	0.153
Radix Saposhnikoviae（RS）	MOL003588	Prangenidin	5-hydroxytryptamine 1A receptor	P08908	HTR1A	0.158
Radix Saposhnikoviae（RS）	MOL003588	Prangenidin	Sodium channel protein type 5 subunit alpha	Q14524	SCN5A	0.162

Radix Saposhnikoviae（RS）	MOL003588	Prangenidin	Prostaglandin G/H synthase 1	P23219	PTGS1	0.173
Radix Saposhnikoviae（RS）	MOL003588	Prangenidin	Histamine H1 receptor	P35367	HRH1	0.174
Radix Saposhnikoviae（RS）	MOL003588	Prangenidin	Muscarinic acetylcholine receptor M1	P11229	CHRM1	0.182
Radix Saposhnikoviae（RS）	MOL003588	Prangenidin	Sodium-dependent noradrenaline transporter	P23975	SLC6A2	0.183
Radix Saposhnikoviae（RS）	MOL003588	Prangenidin	D(1A) dopamine receptor	P21728	DRD1	0.192
Radix Saposhnikoviae（RS）	MOL003588	Prangenidin	Kappa-type opioid receptor	P41145	OPRK1	0.197
Radix Saposhnikoviae（RS）	MOL003588	Prangenidin	Cell division protein kinase 2	P24941	CDK2	0.201
Radix Saposhnikoviae（RS）	MOL003588	Prangenidin	5-hydroxytryptamine 2A receptor	P28223	HTR2A	0.21
Radix Saposhnikoviae（RS）	MOL003588	Prangenidin	D(2) dopamine receptor	P14416	DRD2	0.211
Radix Saposhnikoviae（RS）	MOL003588	Prangenidin	Alpha-1A adrenergic receptor	P35348	ADRA1A	0.212
Radix Saposhnikoviae（RS）	MOL003588	Prangenidin	cAMP-dependent protein kinase catalytic subunit alpha	P17612	PRKACA	0.243
Radix Saposhnikoviae（RS）	MOL003588	Prangenidin	Nuclear receptor coactivator 1	Q15788	NCOA1	0.259
Radix Saposhnikoviae（RS）	MOL003588	Prangenidin	Mu-type opioid receptor	P35372	OPRM1	0.296
Radix Saposhnikoviae（RS）	MOL003588	Prangenidin	Progesterone receptor	P06401	PGR	0.335
Radix Saposhnikoviae（RS）	MOL003588	Prangenidin	Estrogen receptor beta	Q92731	ESR2	0.423
Radix Saposhnikoviae（RS）	MOL003588	Prangenidin	Prostaglandin G/H synthase 2	P35354	PTGS2	0.661
Radix Saposhnikoviae（RS）	MOL003588	Prangenidin	Estrogen receptor	P03372	ESR1	1
Radix Saposhnikoviae（RS）	MOL007514	methyl icosa-11,14-dienoate	SEC14-like protein 2	O76054	SEC14L2	0.024
Radix Saposhnikoviae（RS）	MOL007514	methyl icosa-11,14-dienoate	Alpha-lactalbumin	P00709	LALBA	0.024
Radix Saposhnikoviae（RS）	MOL007514	methyl icosa-11,14-dienoate	Glycodelin	P09466	PAEP	0.024
Radix Saposhnikoviae（RS）	MOL007514	methyl icosa-11,14-dienoate	Cytochrome P450 2C8	P10632	CYP2C8	0.024
Radix Saposhnikoviae（RS）	MOL007514	methyl icosa-11,14-dienoate	Hepatocyte nuclear factor 4-gamma	Q14541	HNF4G	0.024
Radix Saposhnikoviae（RS）	MOL007514	methyl icosa-11,14-dienoate	Toll-like receptor 4	O00206	TLR4	0.031
Radix Saposhnikoviae（RS）	MOL007514	methyl icosa-11,14-dienoate	Peroxisomal 3,2-trans-enoyl-CoA isomerase	O75521	ECI2	0.031
Radix Saposhnikoviae（RS）	MOL007514	methyl icosa-11,14-dienoate	Proto-oncogene tyrosine-protein kinase ABL1	P00519	ABL1	0.031
Radix Saposhnikoviae（RS）	MOL007514	methyl icosa-11,14-dienoate	Insulin	P01308	INS	0.031
Radix Saposhnikoviae（RS）	MOL007514	methyl icosa-11,14-dienoate	Poliovirus receptor	P15151	PVR	0.031
Radix Saposhnikoviae（RS）	MOL007514	methyl icosa-11,14-dienoate	cAMP-dependent protein kinase catalytic subunit alpha	P17612	PRKACA	0.031
Radix Saposhnikoviae（RS）	MOL007514	methyl icosa-11,14-dienoate	Recoverin	P35243	RCVRN	0.031
Radix Saposhnikoviae（RS）	MOL007514	methyl icosa-11,14-dienoate	Hepatocyte nuclear factor 4-alpha	P41235	HNF4A	0.031
Radix Saposhnikoviae（RS）	MOL007514	methyl icosa-11,14-dienoate	Guanylyl cyclase-activating protein 1	P43080	GUCA1A	0.031
Radix Saposhnikoviae（RS）	MOL007514	methyl icosa-11,14-dienoate	cAMP-dependent protein kinase inhibitor alpha	P61925	PKIA	0.031
Radix Saposhnikoviae（RS）	MOL007514	methyl icosa-11,14-dienoate	Calmodulin	P62158		0.031
Radix Saposhnikoviae（RS）	MOL007514	methyl icosa-11,14-dienoate	ADP-ribosylation factor 6	P62330	ARF6	0.031
Radix Saposhnikoviae（RS）	MOL007514	methyl icosa-11,14-dienoate	FK506-binding protein 1A	P62942	FKBP1A	0.031
Radix Saposhnikoviae（RS）	MOL007514	methyl icosa-11,14-dienoate	Calcineurin subunit B isoform 1	P63098	PPP3R1	0.031
Radix Saposhnikoviae（RS）	MOL007514	methyl icosa-11,14-dienoate	ADP-ribosylation factor 1	P84077	ARF1	0.031
Radix Saposhnikoviae（RS）	MOL007514	methyl icosa-11,14-dienoate	Serine/threonine-protein phosphatase 2B catalytic subunit alpha isoform	Q08209	PPP3CA	0.031
Radix Saposhnikoviae（RS）	MOL007514	methyl icosa-11,14-dienoate	Nuclear receptor coactivator 1	Q15788	NCOA1	0.031
Radix Saposhnikoviae（RS）	MOL007514	methyl icosa-11,14-dienoate	Trafficking protein particle complex subunit 6A	O75865	TRAPPC6A	0.048
Radix Saposhnikoviae（RS）	MOL007514	methyl icosa-11,14-dienoate	Trafficking protein particle complex subunit 6B	Q86SZ2	TRAPPC6B	0.048
Radix Saposhnikoviae（RS）	MOL007514	methyl icosa-11,14-dienoate	Trafficking protein particle complex subunit 5	Q8IUR0	TRAPPC5	0.048
Radix Saposhnikoviae（RS）	MOL007514	methyl icosa-11,14-dienoate	Trafficking protein particle complex subunit 4	Q9Y296	TRAPPC4	0.048
Radix Saposhnikoviae（RS）	MOL007514	methyl icosa-11,14-dienoate	Trafficking protein particle complex subunit 1	Q9Y5R8	TRAPPC1	0.048
Radix Saposhnikoviae（RS）	MOL007514	methyl icosa-11,14-dienoate	Retinoic acid receptor alpha	P10276	RARA	0.06
Radix Saposhnikoviae（RS）	MOL007514	methyl icosa-11,14-dienoate	Retinoic acid receptor beta	P10826	RARB	0.06
Radix Saposhnikoviae（RS）	MOL007514	methyl icosa-11,14-dienoate	Retinoic acid receptor RXR-alpha	P19793	RXRA	0.06

Radix Saposhnikoviae（RS）	MOL007514	methyl icosa-11,14-dienoate	Retinoic acid receptor RXR-beta	P28702	RXRB	0.06
Radix Saposhnikoviae（RS）	MOL007514	methyl icosa-11,14-dienoate	Retinoic acid receptor RXR-gamma	P48443	RXRG	0.06
Radix Saposhnikoviae（RS）	MOL007514	methyl icosa-11,14-dienoate	Dihydroorotate dehydrogenase, mitochondrial	Q02127	DHODH	0.082
Radix Saposhnikoviae（RS）	MOL007514	methyl icosa-11,14-dienoate	Furin	P09958	FURIN	0.092
Radix Saposhnikoviae（RS）	MOL007514	methyl icosa-11,14-dienoate	Myelin P2 protein	P02689	PMP2	0.095
Radix Saposhnikoviae（RS）	MOL007514	methyl icosa-11,14-dienoate	Lymphocyte antigen 96	Q9Y6Y9	LY96	0.12
Radix Saposhnikoviae（RS）	MOL007514	methyl icosa-11,14-dienoate	Ganglioside GM2 activator	P17900	GM2A	0.132
Radix Saposhnikoviae（RS）	MOL007514	methyl icosa-11,14-dienoate	Rhodopsin	P08100	RHO	0.178
Radix Saposhnikoviae（RS）	MOL007514	methyl icosa-11,14-dienoate	Trafficking protein particle complex subunit 3	O43617	TRAPPC3	0.244
Radix Saposhnikoviae（RS）	MOL007514	methyl icosa-11,14-dienoate	Gastric triacylglycerol lipase	P07098	LIPF	0.276
Radix Saposhnikoviae（RS）	MOL007514	methyl icosa-11,14-dienoate	Pancreatic triacylglycerol lipase	P16233	PNLIP	0.276
Radix Saposhnikoviae（RS）	MOL007514	methyl icosa-11,14-dienoate	Phospholipase A2	P04054	PLA2G1B	0.327
Radix Saposhnikoviae（RS）	MOL007514	methyl icosa-11,14-dienoate	Phospholipase A2, membrane associated	P14555	PLA2G2A	0.327
Radix Saposhnikoviae（RS）	MOL007514	methyl icosa-11,14-dienoate	Insulin-like growth factor IB	P05019	IGF1	0.355
Radix Saposhnikoviae（RS）	MOL007514	methyl icosa-11,14-dienoate	Glycolipid transfer protein	Q9NZD2	GLTP	0.368
Radix Saposhnikoviae（RS）	MOL007514	methyl icosa-11,14-dienoate	Hemoglobin subunit alpha	P69905	HBA1	0.582
Radix Saposhnikoviae（RS）	MOL007514	methyl icosa-11,14-dienoate	Neutrophil gelatinase-associated lipocalin	P80188	LCN2	0.712
Radix Saposhnikoviae（RS）	MOL007514	methyl icosa-11,14-dienoate	Lysozyme C	P61626	LYZ	0.794
Radix Saposhnikoviae（RS）	MOL007514	methyl icosa-11,14-dienoate	Vitamin K-dependent protein C	P04070	PROC	0.862
Radix Saposhnikoviae（RS）	MOL007514	methyl icosa-11,14-dienoate	Colipase	P04118	CLPS	0.863
Radix Saposhnikoviae（RS）	MOL011730	11-hydroxy-sec-o-beta-d-glucosylhamaudol_qt	Voltage-dependent T-type calcium channel subunit alpha-1G	O43497	CACNA1G	0.01
Radix Saposhnikoviae（RS）	MOL011730	11-hydroxy-sec-o-beta-d-glucosylhamaudol_qt	Carbonic anhydrase 12	O43570	CA12	0.01
Radix Saposhnikoviae（RS）	MOL011730	11-hydroxy-sec-o-beta-d-glucosylhamaudol_qt	Carbonic anhydrase-related protein 11	O75493	CA11	0.01
Radix Saposhnikoviae（RS）	MOL011730	11-hydroxy-sec-o-beta-d-glucosylhamaudol_qt	Voltage-dependent T-type calcium channel subunit alpha-1H	O95180	CACNA1H	0.01
Radix Saposhnikoviae（RS）	MOL011730	11-hydroxy-sec-o-beta-d-glucosylhamaudol_qt	Carbonic anhydrase 3	P07451	CA3	0.01
Radix Saposhnikoviae（RS）	MOL011730	11-hydroxy-sec-o-beta-d-glucosylhamaudol_qt	Amine oxidase [flavin-containing] A	P21397	MAOA	0.01
Radix Saposhnikoviae（RS）	MOL011730	11-hydroxy-sec-o-beta-d-glucosylhamaudol_qt	Carbonic anhydrase 6	P23280	CA6	0.01
Radix Saposhnikoviae（RS）	MOL011730	11-hydroxy-sec-o-beta-d-glucosylhamaudol_qt	Amine oxidase [flavin-containing] B	P27338	MAOB	0.01
Radix Saposhnikoviae（RS）	MOL011730	11-hydroxy-sec-o-beta-d-glucosylhamaudol_qt	Carbonic anhydrase 5A, mitochondrial	P35218	CA5A	0.01
Radix Saposhnikoviae（RS）	MOL011730	11-hydroxy-sec-o-beta-d-glucosylhamaudol_qt	Carbonic anhydrase-related protein	P35219	CA8	0.01
Radix Saposhnikoviae（RS）	MOL011730	11-hydroxy-sec-o-beta-d-glucosylhamaudol_qt	Sodium channel protein type 4 subunit alpha	P35499	SCN4A	0.01
Radix Saposhnikoviae（RS）	MOL011730	11-hydroxy-sec-o-beta-d-glucosylhamaudol_qt	Carbonic anhydrase 7	P43166	CA7	0.01
Radix Saposhnikoviae（RS）	MOL011730	11-hydroxy-sec-o-beta-d-glucosylhamaudol_qt	Sodium channel subunit beta-1	Q07699	SCN1B	0.01
Radix Saposhnikoviae（RS）	MOL011730	11-hydroxy-sec-o-beta-d-glucosylhamaudol_qt	Sodium channel protein type 9 subunit alpha	Q15858	SCN9A	0.01
Radix Saposhnikoviae（RS）	MOL011730	11-hydroxy-sec-o-beta-d-glucosylhamaudol_qt	Carbonic anhydrase 9	Q16790	CA9	0.01
Radix Saposhnikoviae（RS）	MOL011730	11-hydroxy-sec-o-beta-d-glucosylhamaudol_qt	Sodium channel subunit beta-4	Q8IWT1	SCN4B	0.01
Radix Saposhnikoviae（RS）	MOL011730	11-hydroxy-sec-o-beta-d-glucosylhamaudol_qt	Carbonic anhydrase 13	Q8N1Q1	CA13	0.01
Radix Saposhnikoviae（RS）	MOL011730	11-hydroxy-sec-o-beta-d-glucosylhamaudol_qt	Sodium channel protein type 2 subunit alpha	Q99250	SCN2A	0.01
Radix Saposhnikoviae（RS）	MOL011730	11-hydroxy-sec-o-beta-d-glucosylhamaudol_qt	Carbonic anhydrase-related protein 10	Q9NS85	CA10	0.01
Radix Saposhnikoviae（RS）	MOL011730	11-hydroxy-sec-o-beta-d-glucosylhamaudol_qt	Sodium channel protein type 3 subunit alpha	Q9NY46	SCN3A	0.01
Radix Saposhnikoviae（RS）	MOL011730	11-hydroxy-sec-o-beta-d-glucosylhamaudol_qt	Sodium channel subunit beta-3	Q9NY72	SCN3B	0.01
Radix Saposhnikoviae（RS）	MOL011730	11-hydroxy-sec-o-beta-d-glucosylhamaudol_qt	Voltage-dependent T-type calcium channel subunit alpha-1I	Q9P0X4	CACNA1I	0.01
Radix Saposhnikoviae（RS）	MOL011730	11-hydroxy-sec-o-beta-d-glucosylhamaudol_qt	Sodium channel protein type 11 subunit alpha	Q9UI33	SCN11A	0.01
Radix Saposhnikoviae（RS）	MOL011730	11-hydroxy-sec-o-beta-d-glucosylhamaudol_qt	Carbonic anhydrase 14	Q9ULX7	CA14	0.01
Radix Saposhnikoviae（RS）	MOL011730	11-hydroxy-sec-o-beta-d-glucosylhamaudol_qt	Carbonic anhydrase 5B, mitochondrial	Q9Y2D0	CA5B	0.01
Radix Saposhnikoviae（RS）	MOL011730	11-hydroxy-sec-o-beta-d-glucosylhamaudol_qt	Sodium/hydrogen exchanger 1	P19634	SLC9A1	0.012
Radix Saposhnikoviae（RS）	MOL011730	11-hydroxy-sec-o-beta-d-glucosylhamaudol_qt	Amiloride-sensitive amine oxidase [copper-containing]	P19801	AOC1	0.012

Radix Saposhnikoviae（RS）	MOL011730	11-hydroxy-sec-o-beta-d-glucosylhamaudol_qt	Amiloride-sensitive sodium channel subunit delta	P51172	SCNN1D	0.012
Radix Saposhnikoviae（RS）	MOL011730	11-hydroxy-sec-o-beta-d-glucosylhamaudol_qt	Amiloride-sensitive cation channel 2, neuronal	P78348	ASIC1	0.012
Radix Saposhnikoviae（RS）	MOL011730	11-hydroxy-sec-o-beta-d-glucosylhamaudol_qt	Amiloride-sensitive cation channel 1, neuronal	Q16515	ASIC2	0.012
Radix Saposhnikoviae（RS）	MOL011730	11-hydroxy-sec-o-beta-d-glucosylhamaudol_qt	Acetyl-CoA carboxylase 2	O00763	ACACB	0.014
Radix Saposhnikoviae（RS）	MOL011730	11-hydroxy-sec-o-beta-d-glucosylhamaudol_qt	Propionyl-CoA carboxylase alpha chain, mitochondrial	P05165	PCCA	0.014
Radix Saposhnikoviae（RS）	MOL011730	11-hydroxy-sec-o-beta-d-glucosylhamaudol_qt	Propionyl-CoA carboxylase beta chain, mitochondrial	P05166	PCCB	0.014
Radix Saposhnikoviae（RS）	MOL011730	11-hydroxy-sec-o-beta-d-glucosylhamaudol_qt	5-hydroxytryptamine 1E receptor	P28566	HTR1E	0.014
Radix Saposhnikoviae（RS）	MOL011730	11-hydroxy-sec-o-beta-d-glucosylhamaudol_qt	5-hydroxytryptamine 7 receptor	P34969	HTR7	0.014
Radix Saposhnikoviae（RS）	MOL011730	11-hydroxy-sec-o-beta-d-glucosylhamaudol_qt	Glutamate receptor 2	P42262	GRIA2	0.014
Radix Saposhnikoviae（RS）	MOL011730	11-hydroxy-sec-o-beta-d-glucosylhamaudol_qt	5-hydroxytryptamine 6 receptor	P50406	HTR6	0.014
Radix Saposhnikoviae（RS）	MOL011730	11-hydroxy-sec-o-beta-d-glucosylhamaudol_qt	Biotin--protein ligase	P50747	HLCS	0.014
Radix Saposhnikoviae（RS）	MOL011730	11-hydroxy-sec-o-beta-d-glucosylhamaudol_qt	Sodium/potassium-transporting ATPase gamma chain	P54710	FXYD2	0.014
Radix Saposhnikoviae（RS）	MOL011730	11-hydroxy-sec-o-beta-d-glucosylhamaudol_qt	Solute carrier family 12 member 2	P55011	SLC12A2	0.014
Radix Saposhnikoviae（RS）	MOL011730	11-hydroxy-sec-o-beta-d-glucosylhamaudol_qt	Solute carrier family 12 member 3	P55017	SLC12A3	0.014
Radix Saposhnikoviae（RS）	MOL011730	11-hydroxy-sec-o-beta-d-glucosylhamaudol_qt	Solute carrier family 12 member 1	Q13621	SLC12A1	0.014
Radix Saposhnikoviae（RS）	MOL011730	11-hydroxy-sec-o-beta-d-glucosylhamaudol_qt	Methylcrotonoyl-CoA carboxylase subunit alpha, mitochondrial	Q96RQ3	MCCC1	0.014
Radix Saposhnikoviae（RS）	MOL011730	11-hydroxy-sec-o-beta-d-glucosylhamaudol_qt	Methylcrotonoyl-CoA carboxylase beta chain, mitochondrial	Q9HCC0	MCCC2	0.014
Radix Saposhnikoviae（RS）	MOL011730	11-hydroxy-sec-o-beta-d-glucosylhamaudol_qt	D1 dopamine receptor-interacting protein calcyon	Q9NYX4	CALY	0.014
Radix Saposhnikoviae（RS）	MOL011730	11-hydroxy-sec-o-beta-d-glucosylhamaudol_qt	Sodium-dependent multivitamin transporter	Q9Y289	SLC5A6	0.014
Radix Saposhnikoviae（RS）	MOL011730	11-hydroxy-sec-o-beta-d-glucosylhamaudol_qt	Phospholipase A2	P04054	PLA2G1B	0.015
Radix Saposhnikoviae（RS）	MOL011730	11-hydroxy-sec-o-beta-d-glucosylhamaudol_qt	Sodium/potassium-transporting ATPase alpha-1 chain	P05023	ATP1A1	0.015
Radix Saposhnikoviae（RS）	MOL011730	11-hydroxy-sec-o-beta-d-glucosylhamaudol_qt	Calcium-activated potassium channel subunit alpha 1	Q12791	KCNMA1	0.015
Radix Saposhnikoviae（RS）	MOL011730	11-hydroxy-sec-o-beta-d-glucosylhamaudol_qt	Opioid receptor, sigma 1	Q5T1J1	SIGMAR1	0.016
Radix Saposhnikoviae（RS）	MOL011730	11-hydroxy-sec-o-beta-d-glucosylhamaudol_qt	Sigma 1-type opioid receptor	Q99720	SIGMAR1	0.016
Radix Saposhnikoviae（RS）	MOL011730	11-hydroxy-sec-o-beta-d-glucosylhamaudol_qt	Potassium channel subfamily K member 1	O00180	KCNK1	0.018
Radix Saposhnikoviae（RS）	MOL011730	11-hydroxy-sec-o-beta-d-glucosylhamaudol_qt	Sodium channel protein type 1 subunit alpha	P35498	SCN1A	0.018
Radix Saposhnikoviae（RS）	MOL011730	11-hydroxy-sec-o-beta-d-glucosylhamaudol_qt	Tyrosinase	P14679	TYR	0.019
Radix Saposhnikoviae（RS）	MOL011730	11-hydroxy-sec-o-beta-d-glucosylhamaudol_qt	fMet-Leu-Phe receptor	P21462	FPR1	0.019
Radix Saposhnikoviae（RS）	MOL011730	11-hydroxy-sec-o-beta-d-glucosylhamaudol_qt	Translocator protein	P30536	TSPO	0.019
Radix Saposhnikoviae（RS）	MOL011730	11-hydroxy-sec-o-beta-d-glucosylhamaudol_qt	3-oxo-5-alpha-steroid 4-dehydrogenase 2	P31213	SRD5A2	0.019
Radix Saposhnikoviae（RS）	MOL011730	11-hydroxy-sec-o-beta-d-glucosylhamaudol_qt	Neuronal acetylcholine receptor subunit alpha-3	P32297	CHRNA3	0.019
Radix Saposhnikoviae（RS）	MOL011730	11-hydroxy-sec-o-beta-d-glucosylhamaudol_qt	3-oxo-5-beta-steroid 4-dehydrogenase	P51857	AKR1D1	0.019
Radix Saposhnikoviae（RS）	MOL011730	11-hydroxy-sec-o-beta-d-glucosylhamaudol_qt	Troponin C, slow skeletal and cardiac muscles	P63316	TNNC1	0.019
Radix Saposhnikoviae（RS）	MOL011730	11-hydroxy-sec-o-beta-d-glucosylhamaudol_qt	Prostaglandin D2 receptor	Q13258	PTGDR	0.019
Radix Saposhnikoviae（RS）	MOL011730	11-hydroxy-sec-o-beta-d-glucosylhamaudol_qt	5-hydroxytryptamine 4 receptor	Q13639	HTR4	0.019
Radix Saposhnikoviae（RS）	MOL011730	11-hydroxy-sec-o-beta-d-glucosylhamaudol_qt	ATP-sensitive inward rectifier potassium channel 8	Q15842	KCNJ8	0.019
Radix Saposhnikoviae（RS）	MOL011730	11-hydroxy-sec-o-beta-d-glucosylhamaudol_qt	Cysteinyl leukotriene receptor 2	Q9NS75	CYSLTR2	0.019
Radix Saposhnikoviae（RS）	MOL011730	11-hydroxy-sec-o-beta-d-glucosylhamaudol_qt	Cysteinyl leukotriene receptor 1	Q9Y271	CYSLTR1	0.019
Radix Saposhnikoviae（RS）	MOL011730	11-hydroxy-sec-o-beta-d-glucosylhamaudol_qt	Glutamate receptor, ionotropic kainate 2	Q13002	GRIK2	0.02
Radix Saposhnikoviae（RS）	MOL011730	11-hydroxy-sec-o-beta-d-glucosylhamaudol_qt	Elongation factor 2	P13639	EEF2	0.021
Radix Saposhnikoviae（RS）	MOL011730	11-hydroxy-sec-o-beta-d-glucosylhamaudol_qt	Poly [ADP-ribose] polymerase 3	Q9Y6F1	PARP3	0.021
Radix Saposhnikoviae（RS）	MOL011730	11-hydroxy-sec-o-beta-d-glucosylhamaudol_qt	Acetylcholinesterase	P22303	ACHE	0.022
Radix Saposhnikoviae（RS）	MOL011730	11-hydroxy-sec-o-beta-d-glucosylhamaudol_qt	Sodium-dependent serotonin transporter	P31645	SLC6A4	0.022
Radix Saposhnikoviae（RS）	MOL011730	11-hydroxy-sec-o-beta-d-glucosylhamaudol_qt	Alpha-7 nicotinic cholinergic receptor subunit	Q693P7	CHRNA7	0.022
Radix Saposhnikoviae（RS）	MOL011730	11-hydroxy-sec-o-beta-d-glucosylhamaudol_qt	Interleukin-1 beta	P01584	IL1B	0.025
Radix Saposhnikoviae（RS）	MOL011730	11-hydroxy-sec-o-beta-d-glucosylhamaudol_qt	Matrix metalloproteinase-9	P14780	MMP9	0.025
Radix Saposhnikoviae（RS）	MOL011730	11-hydroxy-sec-o-beta-d-glucosylhamaudol_qt	Vascular endothelial growth factor A	P15692	VEGFA	0.025

Radix Saposhnikoviae（RS）	MOL011730	11-hydroxy-sec-o-beta-d-glucosylhamaudol_qt	Caspase-1	P29466	CASP1	0.025
Radix Saposhnikoviae（RS）	MOL011730	11-hydroxy-sec-o-beta-d-glucosylhamaudol_qt	Cytochrome c	P99999	CYCS	0.025
Radix Saposhnikoviae（RS）	MOL011730	11-hydroxy-sec-o-beta-d-glucosylhamaudol_qt	Transient receptor potential cation channel subfamily A member 1	O75762	TRPA1	0.026
Radix Saposhnikoviae（RS）	MOL011730	11-hydroxy-sec-o-beta-d-glucosylhamaudol_qt	Serine/threonine-protein kinase 17B	O94768	STK17B	0.026
Radix Saposhnikoviae（RS）	MOL011730	11-hydroxy-sec-o-beta-d-glucosylhamaudol_qt	cAMP response element-binding protein	P16220	CREB1	0.026
Radix Saposhnikoviae（RS）	MOL011730	11-hydroxy-sec-o-beta-d-glucosylhamaudol_qt	Phosphatidylinositol 3-kinase regulatory subunit alpha	P27986	PIK3R1	0.026
Radix Saposhnikoviae（RS）	MOL011730	11-hydroxy-sec-o-beta-d-glucosylhamaudol_qt	ATP synthase subunit gamma, mitochondrial	P36542	ATP5F1C	0.026
Radix Saposhnikoviae（RS）	MOL011730	11-hydroxy-sec-o-beta-d-glucosylhamaudol_qt	Nociceptin receptor	P41146	OPRL1	0.026
Radix Saposhnikoviae（RS）	MOL011730	11-hydroxy-sec-o-beta-d-glucosylhamaudol_qt	Serine/threonine-protein kinase PLK1	P53350	PLK1	0.026
Radix Saposhnikoviae（RS）	MOL011730	11-hydroxy-sec-o-beta-d-glucosylhamaudol_qt	UDP-glucuronosyltransferase 3A1	Q6NUS8	UGT3A1	0.026
Radix Saposhnikoviae（RS）	MOL011730	11-hydroxy-sec-o-beta-d-glucosylhamaudol_qt	Transient receptor potential cation channel subfamily M member 8	Q7Z2W7	TRPM8	0.026
Radix Saposhnikoviae（RS）	MOL011730	11-hydroxy-sec-o-beta-d-glucosylhamaudol_qt	Transient receptor potential cation channel subfamily V member 3	Q8NET8	TRPV3	0.026
Radix Saposhnikoviae（RS）	MOL011730	11-hydroxy-sec-o-beta-d-glucosylhamaudol_qt	Carbonic anhydrase 4	P22748	CA4	0.027
Radix Saposhnikoviae（RS）	MOL011730	11-hydroxy-sec-o-beta-d-glucosylhamaudol_qt	Gamma-aminobutyric-acid receptor subunit beta-3	P28472	GABRB3	0.027
Radix Saposhnikoviae（RS）	MOL011730	11-hydroxy-sec-o-beta-d-glucosylhamaudol_qt	Bile salt sulfotransferase	Q06520	SULT2A1	0.027
Radix Saposhnikoviae（RS）	MOL011730	11-hydroxy-sec-o-beta-d-glucosylhamaudol_qt	Dehydrogenase/reductase SDR family member 8	Q8NBQ5	HSD17B11	0.027
Radix Saposhnikoviae（RS）	MOL011730	11-hydroxy-sec-o-beta-d-glucosylhamaudol_qt	Ribonucleoside-diphosphate reductase M2 subunit	P31350	RRM2	0.028
Radix Saposhnikoviae（RS）	MOL011730	11-hydroxy-sec-o-beta-d-glucosylhamaudol_qt	Amiloride-sensitive sodium channel subunit alpha	P37088	SCNN1A	0.028
Radix Saposhnikoviae（RS）	MOL011730	11-hydroxy-sec-o-beta-d-glucosylhamaudol_qt	Amiloride-sensitive sodium channel subunit beta	P51168	SCNN1B	0.028
Radix Saposhnikoviae（RS）	MOL011730	11-hydroxy-sec-o-beta-d-glucosylhamaudol_qt	Amiloride-sensitive sodium channel subunit gamma	P51170	SCNN1G	0.028
Radix Saposhnikoviae（RS）	MOL011730	11-hydroxy-sec-o-beta-d-glucosylhamaudol_qt	DNA polymerase epsilon subunit 2	P56282	POLE2	0.028
Radix Saposhnikoviae（RS）	MOL011730	11-hydroxy-sec-o-beta-d-glucosylhamaudol_qt	DNA polymerase epsilon catalytic subunit A	Q07864	POLE	0.028
Radix Saposhnikoviae（RS）	MOL011730	11-hydroxy-sec-o-beta-d-glucosylhamaudol_qt	Ribonucleoside-diphosphate reductase subunit M2 B	Q7LG56	RRM2B	0.028
Radix Saposhnikoviae（RS）	MOL011730	11-hydroxy-sec-o-beta-d-glucosylhamaudol_qt	DNA polymerase epsilon subunit 3	Q9NRF9	POLE3	0.028
Radix Saposhnikoviae（RS）	MOL011730	11-hydroxy-sec-o-beta-d-glucosylhamaudol_qt	Mitogen-activated protein kinase 8	P45983	MAPK8	0.029
Radix Saposhnikoviae（RS）	MOL011730	11-hydroxy-sec-o-beta-d-glucosylhamaudol_qt	Mitogen-activated protein kinase 10	P53779	MAPK10	0.029
Radix Saposhnikoviae（RS）	MOL011730	11-hydroxy-sec-o-beta-d-glucosylhamaudol_qt	Neuronal acetylcholine receptor subunit alpha-2	Q15822	CHRNA2	0.029
Radix Saposhnikoviae（RS）	MOL011730	11-hydroxy-sec-o-beta-d-glucosylhamaudol_qt	C-jun-amino-terminal kinase-interacting protein 1	Q9UQF2	MAPK8IP1	0.029
Radix Saposhnikoviae（RS）	MOL011730	11-hydroxy-sec-o-beta-d-glucosylhamaudol_qt	Neuronal acetylcholine receptor subunit alpha-4	P43681	CHRNA4	0.03
Radix Saposhnikoviae（RS）	MOL011730	11-hydroxy-sec-o-beta-d-glucosylhamaudol_qt	Dihydrolipoyllysine-residue acetyltransferase component of pyruvate dehydrogenase complex, mitochondrial	P10515	DLAT	0.031
Radix Saposhnikoviae（RS）	MOL011730	11-hydroxy-sec-o-beta-d-glucosylhamaudol_qt	Glycine cleavage system H protein, mitochondrial	P23434	GCSH	0.031
Radix Saposhnikoviae（RS）	MOL011730	11-hydroxy-sec-o-beta-d-glucosylhamaudol_qt	Potassium voltage-gated channel subfamily H member 2	Q12809	KCNH2	0.031
Radix Saposhnikoviae（RS）	MOL011730	11-hydroxy-sec-o-beta-d-glucosylhamaudol_qt	Potassium voltage-gated channel subfamily H member 6	Q9H252	KCNH6	0.031
Radix Saposhnikoviae（RS）	MOL011730	11-hydroxy-sec-o-beta-d-glucosylhamaudol_qt	Tumor necrosis factor	P01375	TNF	0.032
Radix Saposhnikoviae（RS）	MOL011730	11-hydroxy-sec-o-beta-d-glucosylhamaudol_qt	Tyrosine-protein kinase Lyn	P07948	LYN	0.032
Radix Saposhnikoviae（RS）	MOL011730	11-hydroxy-sec-o-beta-d-glucosylhamaudol_qt	Muscarinic acetylcholine receptor M2	P08172	CHRM2	0.032
Radix Saposhnikoviae（RS）	MOL011730	11-hydroxy-sec-o-beta-d-glucosylhamaudol_qt	Muscarinic acetylcholine receptor M4	P08173	CHRM4	0.032
Radix Saposhnikoviae（RS）	MOL011730	11-hydroxy-sec-o-beta-d-glucosylhamaudol_qt	Muscarinic acetylcholine receptor M5	P08912	CHRM5	0.032
Radix Saposhnikoviae（RS）	MOL011730	11-hydroxy-sec-o-beta-d-glucosylhamaudol_qt	Muscarinic acetylcholine receptor M1	P11229	CHRM1	0.032
Radix Saposhnikoviae（RS）	MOL011730	11-hydroxy-sec-o-beta-d-glucosylhamaudol_qt	Nuclear factor NF-kappa-B p105 subunit	P19838	NFKB1	0.032
Radix Saposhnikoviae（RS）	MOL011730	11-hydroxy-sec-o-beta-d-glucosylhamaudol_qt	Fibroblast growth factor receptor 2	P21802	FGFR2	0.032
Radix Saposhnikoviae（RS）	MOL011730	11-hydroxy-sec-o-beta-d-glucosylhamaudol_qt	5-hydroxytryptamine 1F receptor	P30939	HTR1F	0.032
Radix Saposhnikoviae（RS）	MOL011730	11-hydroxy-sec-o-beta-d-glucosylhamaudol_qt	Histamine H1 receptor	P35367	HRH1	0.032
Radix Saposhnikoviae（RS）	MOL011730	11-hydroxy-sec-o-beta-d-glucosylhamaudol_qt	3-phosphoinositide-dependent protein kinase 1	O15530	PDPK1	0.033
Radix Saposhnikoviae（RS）	MOL011730	11-hydroxy-sec-o-beta-d-glucosylhamaudol_qt	85 kDa calcium-independent phospholipase A2	O60733	PLA2G6	0.033
Radix Saposhnikoviae（RS）	MOL011730	11-hydroxy-sec-o-beta-d-glucosylhamaudol_qt	Pepsin A	P00790	REN	0.033

Radix Saposhnikoviae（RS）	MOL011730	11-hydroxy-sec-o-beta-d-glucosylhamaudol_qt	Pyruvate carboxylase, mitochondrial	P11498	PC	0.033
Radix Saposhnikoviae（RS）	MOL011730	11-hydroxy-sec-o-beta-d-glucosylhamaudol_qt	Dipeptidyl peptidase 4	P27487	DPP4	0.033
Radix Saposhnikoviae（RS）	MOL011730	11-hydroxy-sec-o-beta-d-glucosylhamaudol_qt	Tyrosine-protein kinase CSK	P41240	CSK	0.033
Radix Saposhnikoviae（RS）	MOL011730	11-hydroxy-sec-o-beta-d-glucosylhamaudol_qt	5-hydroxytryptamine 2B receptor	P41595	HTR2B	0.033
Radix Saposhnikoviae（RS）	MOL011730	11-hydroxy-sec-o-beta-d-glucosylhamaudol_qt	Tyrosine-protein kinase ZAP-70	P43403	ZAP70	0.033
Radix Saposhnikoviae（RS）	MOL011730	11-hydroxy-sec-o-beta-d-glucosylhamaudol_qt	Tyrosine-protein kinase SYK	P43405	SYK	0.033
Radix Saposhnikoviae（RS）	MOL011730	11-hydroxy-sec-o-beta-d-glucosylhamaudol_qt	Cytosolic phospholipase A2	P47712	PLA2G4A	0.033
Radix Saposhnikoviae（RS）	MOL011730	11-hydroxy-sec-o-beta-d-glucosylhamaudol_qt	Protein kinase C theta type	Q04759	PRKCQ	0.033
Radix Saposhnikoviae（RS）	MOL011730	11-hydroxy-sec-o-beta-d-glucosylhamaudol_qt	Tyrosine-protein kinase ITK/TSK	Q08881	ITK	0.033
Radix Saposhnikoviae（RS）	MOL011730	11-hydroxy-sec-o-beta-d-glucosylhamaudol_qt	Prostaglandin reductase 1	Q14914	PTGR1	0.033
Radix Saposhnikoviae（RS）	MOL011730	11-hydroxy-sec-o-beta-d-glucosylhamaudol_qt	Tyrosine-protein kinase JAK2	O60674	JAK2	0.034
Radix Saposhnikoviae（RS）	MOL011730	11-hydroxy-sec-o-beta-d-glucosylhamaudol_qt	Tyrosine-protein kinase JAK1	P23458	JAK1	0.034
Radix Saposhnikoviae（RS）	MOL011730	11-hydroxy-sec-o-beta-d-glucosylhamaudol_qt	Tyrosine-protein kinase JAK3	P52333	JAK3	0.034
Radix Saposhnikoviae（RS）	MOL011730	11-hydroxy-sec-o-beta-d-glucosylhamaudol_qt	Cyclin-dependent kinase 5 activator 1	Q15078	CDK5R1	0.035
Radix Saposhnikoviae（RS）	MOL011730	11-hydroxy-sec-o-beta-d-glucosylhamaudol_qt	5-hydroxytryptamine 3 receptor	P46098	HTR3A	0.037
Radix Saposhnikoviae（RS）	MOL011730	11-hydroxy-sec-o-beta-d-glucosylhamaudol_qt	Sodium-dependent dopamine transporter	Q01959	SLC6A3	0.037
Radix Saposhnikoviae（RS）	MOL011730	11-hydroxy-sec-o-beta-d-glucosylhamaudol_qt	ATP-sensitive inward rectifier potassium channel 11	Q14654	KCNJ11	0.039
Radix Saposhnikoviae（RS）	MOL011730	11-hydroxy-sec-o-beta-d-glucosylhamaudol_qt	Alcohol dehydrogenase [NADP+]	P14550	AKR1A1	0.04
Radix Saposhnikoviae（RS）	MOL011730	11-hydroxy-sec-o-beta-d-glucosylhamaudol_qt	Aldose reductase	P15121	AKR1B1	0.04
Radix Saposhnikoviae（RS）	MOL011730	11-hydroxy-sec-o-beta-d-glucosylhamaudol_qt	cAMP-specific 3',5'-cyclic phosphodiesterase 4B	Q07343	PDE4B	0.041
Radix Saposhnikoviae（RS）	MOL011730	11-hydroxy-sec-o-beta-d-glucosylhamaudol_qt	cGMP-inhibited 3',5'-cyclic phosphodiesterase A	Q14432	PDE3A	0.041
Radix Saposhnikoviae（RS）	MOL011730	11-hydroxy-sec-o-beta-d-glucosylhamaudol_qt	Glutamate [NMDA] receptor subunit 3A	Q8TCU5	GRIN3A	0.042
Radix Saposhnikoviae（RS）	MOL011730	11-hydroxy-sec-o-beta-d-glucosylhamaudol_qt	DNA topoisomerase 1	P11387	TOP1	0.043
Radix Saposhnikoviae（RS）	MOL011730	11-hydroxy-sec-o-beta-d-glucosylhamaudol_qt	Gamma-aminobutyric acid receptor subunit gamma-2	P18507	GABRG2	0.043
Radix Saposhnikoviae（RS）	MOL011730	11-hydroxy-sec-o-beta-d-glucosylhamaudol_qt	Neuronal acetylcholine receptor subunit alpha-7	P36544	CHRNA7	0.043
Radix Saposhnikoviae（RS）	MOL011730	11-hydroxy-sec-o-beta-d-glucosylhamaudol_qt	Gamma-aminobutyric-acid receptor subunit beta-2	P47870	GABRB2	0.043
Radix Saposhnikoviae（RS）	MOL011730	11-hydroxy-sec-o-beta-d-glucosylhamaudol_qt	Gamma-aminobutyric acid receptor subunit gamma-1	Q8N1C3	GABRG1	0.043
Radix Saposhnikoviae（RS）	MOL011730	11-hydroxy-sec-o-beta-d-glucosylhamaudol_qt	DNA topoisomerase I, mitochondrial	Q969P6	TOP1MT	0.043
Radix Saposhnikoviae（RS）	MOL011730	11-hydroxy-sec-o-beta-d-glucosylhamaudol_qt	Gamma-aminobutyric acid receptor subunit theta	Q9UN88	GABRQ	0.043
Radix Saposhnikoviae（RS）	MOL011730	11-hydroxy-sec-o-beta-d-glucosylhamaudol_qt	Myeloperoxidase	P05164	MPO	0.044
Radix Saposhnikoviae（RS）	MOL011730	11-hydroxy-sec-o-beta-d-glucosylhamaudol_qt	Keratin, type II cytoskeletal 7	P08729	KRT7	0.044
Radix Saposhnikoviae（RS）	MOL011730	11-hydroxy-sec-o-beta-d-glucosylhamaudol_qt	Eosinophil peroxidase	P11678	EPX	0.044
Radix Saposhnikoviae（RS）	MOL011730	11-hydroxy-sec-o-beta-d-glucosylhamaudol_qt	Platelet glycoprotein IX	P14770	GP9	0.044
Radix Saposhnikoviae（RS）	MOL011730	11-hydroxy-sec-o-beta-d-glucosylhamaudol_qt	Sodium-dependent noradrenaline transporter	P23975	SLC6A2	0.044
Radix Saposhnikoviae（RS）	MOL011730	11-hydroxy-sec-o-beta-d-glucosylhamaudol_qt	Calreticulin	P27797	CALR	0.044
Radix Saposhnikoviae（RS）	MOL011730	11-hydroxy-sec-o-beta-d-glucosylhamaudol_qt	Melatonin receptor type 1B	P49286	MTNR1B	0.044
Radix Saposhnikoviae（RS）	MOL011730	11-hydroxy-sec-o-beta-d-glucosylhamaudol_qt	Calmodulin	P62158		0.044
Radix Saposhnikoviae（RS）	MOL011730	11-hydroxy-sec-o-beta-d-glucosylhamaudol_qt	Guanine nucleotide-binding protein G(s) subunit alpha isoforms short	P63092	GNAS	0.044
Radix Saposhnikoviae（RS）	MOL011730	11-hydroxy-sec-o-beta-d-glucosylhamaudol_qt	Adenylate cyclase type 2	Q08462	ADCY2	0.044
Radix Saposhnikoviae（RS）	MOL011730	11-hydroxy-sec-o-beta-d-glucosylhamaudol_qt	Sodium channel protein type 5 subunit alpha	Q14524	SCN5A	0.044
Radix Saposhnikoviae（RS）	MOL011730	11-hydroxy-sec-o-beta-d-glucosylhamaudol_qt	Nuclear receptor ROR-beta	Q92753	RORB	0.044
Radix Saposhnikoviae（RS）	MOL011730	11-hydroxy-sec-o-beta-d-glucosylhamaudol_qt	Group IIE secretory phospholipase A2	Q9NZK7	PLA2G2E	0.045
Radix Saposhnikoviae（RS）	MOL011730	11-hydroxy-sec-o-beta-d-glucosylhamaudol_qt	Peroxisome proliferator-activated receptor gamma	P37231	PPARG	0.046
Radix Saposhnikoviae（RS）	MOL011730	11-hydroxy-sec-o-beta-d-glucosylhamaudol_qt	Tyrosyl-tRNA synthetase, cytoplasmic	P54577	YARS	0.047
Radix Saposhnikoviae（RS）	MOL011730	11-hydroxy-sec-o-beta-d-glucosylhamaudol_qt	Casein kinase I isoform gamma-2	P78368	CSNK1G2	0.047
Radix Saposhnikoviae（RS）	MOL011730	11-hydroxy-sec-o-beta-d-glucosylhamaudol_qt	Peptidyl-prolyl cis-trans isomerase NIMA-interacting 1	Q13526	PIN1	0.047
Radix Saposhnikoviae（RS）	MOL011730	11-hydroxy-sec-o-beta-d-glucosylhamaudol_qt	Serine/threonine-protein kinase haspin	Q8TF76	HASPIN	0.047

Radix Saposhnikoviae（RS）	MOL011730	11-hydroxy-sec-o-beta-d-glucosylhamaudol_qt	Peptidyl-prolyl cis-trans isomerase, mitochondrial	P30405	PPIF	0.048
Radix Saposhnikoviae（RS）	MOL011730	11-hydroxy-sec-o-beta-d-glucosylhamaudol_qt	Alpha-1D adrenergic receptor	P25100	ADRA1D	0.05
Radix Saposhnikoviae（RS）	MOL011730	11-hydroxy-sec-o-beta-d-glucosylhamaudol_qt	Gamma-aminobutyric acid receptor subunit rho-3	A8MPY1	GABRR3	0.051
Radix Saposhnikoviae（RS）	MOL011730	11-hydroxy-sec-o-beta-d-glucosylhamaudol_qt	Gamma-aminobutyric acid receptor subunit pi	O00591	GABRP	0.051
Radix Saposhnikoviae（RS）	MOL011730	11-hydroxy-sec-o-beta-d-glucosylhamaudol_qt	Gamma-aminobutyric acid receptor subunit delta	O14764	GABRD	0.051
Radix Saposhnikoviae（RS）	MOL011730	11-hydroxy-sec-o-beta-d-glucosylhamaudol_qt	Gamma-aminobutyric-acid receptor subunit beta-1	P18505	GABRB1	0.051
Radix Saposhnikoviae（RS）	MOL011730	11-hydroxy-sec-o-beta-d-glucosylhamaudol_qt	Gamma-aminobutyric-acid receptor subunit rho-1	P24046	GABRR1	0.051
Radix Saposhnikoviae（RS）	MOL011730	11-hydroxy-sec-o-beta-d-glucosylhamaudol_qt	Gamma-aminobutyric acid receptor subunit rho-2	P28476	GABRR2	0.051
Radix Saposhnikoviae（RS）	MOL011730	11-hydroxy-sec-o-beta-d-glucosylhamaudol_qt	Gamma-aminobutyric acid receptor subunit epsilon	P78334	GABRE	0.051
Radix Saposhnikoviae（RS）	MOL011730	11-hydroxy-sec-o-beta-d-glucosylhamaudol_qt	Gamma-aminobutyric acid receptor subunit gamma-3	Q99928	GABRG3	0.051
Radix Saposhnikoviae（RS）	MOL011730	11-hydroxy-sec-o-beta-d-glucosylhamaudol_qt	3 beta-hydroxysteroid dehydrogenase/Delta 5-->4-isomerase type II	P26439	HSD3B2	0.052
Radix Saposhnikoviae（RS）	MOL011730	11-hydroxy-sec-o-beta-d-glucosylhamaudol_qt	Gamma-aminobutyric-acid receptor subunit alpha-6	Q16445	GABRA6	0.052
Radix Saposhnikoviae（RS）	MOL011730	11-hydroxy-sec-o-beta-d-glucosylhamaudol_qt	Prolactin receptor	P16471	PRLR	0.053
Radix Saposhnikoviae（RS）	MOL011730	11-hydroxy-sec-o-beta-d-glucosylhamaudol_qt	3-oxo-5-alpha-steroid 4-dehydrogenase 1	P18405	SRD5A1	0.053
Radix Saposhnikoviae（RS）	MOL011730	11-hydroxy-sec-o-beta-d-glucosylhamaudol_qt	Gonadotropin-releasing hormone receptor	P30968	GNRHR	0.053
Radix Saposhnikoviae（RS）	MOL011730	11-hydroxy-sec-o-beta-d-glucosylhamaudol_qt	Gonadotropin-releasing hormone II receptor	Q96P88	GNRHR2	0.053
Radix Saposhnikoviae（RS）	MOL011730	11-hydroxy-sec-o-beta-d-glucosylhamaudol_qt	Urokinase-type plasminogen activator	P00749	PLAU	0.054
Radix Saposhnikoviae（RS）	MOL011730	11-hydroxy-sec-o-beta-d-glucosylhamaudol_qt	Corticosteroid 11-beta-dehydrogenase isozyme 1	P28845	HSD11B1	0.054
Radix Saposhnikoviae（RS）	MOL011730	11-hydroxy-sec-o-beta-d-glucosylhamaudol_qt	ATP synthase subunit beta, mitochondrial	P06576	ATP5F1B	0.055
Radix Saposhnikoviae（RS）	MOL011730	11-hydroxy-sec-o-beta-d-glucosylhamaudol_qt	Tyrosine-protein kinase HCK	P08631	HCK	0.055
Radix Saposhnikoviae（RS）	MOL011730	11-hydroxy-sec-o-beta-d-glucosylhamaudol_qt	ATP synthase subunit alpha, mitochondrial	P25705	ATP5F1A	0.055
Radix Saposhnikoviae（RS）	MOL011730	11-hydroxy-sec-o-beta-d-glucosylhamaudol_qt	Tripartite motif-containing protein 13	O60858	TRIM13	0.056
Radix Saposhnikoviae（RS）	MOL011730	11-hydroxy-sec-o-beta-d-glucosylhamaudol_qt	Purine nucleoside phosphorylase	P00491	PNP	0.058
Radix Saposhnikoviae（RS）	MOL011730	11-hydroxy-sec-o-beta-d-glucosylhamaudol_qt	DNA polymerase alpha catalytic subunit	P09884	POLA1	0.058
Radix Saposhnikoviae（RS）	MOL011730	11-hydroxy-sec-o-beta-d-glucosylhamaudol_qt	Ribonucleoside-diphosphate reductase large subunit	P23921	RRM1	0.058
Radix Saposhnikoviae（RS）	MOL011730	11-hydroxy-sec-o-beta-d-glucosylhamaudol_qt	Tubulin beta-2C chain	P68371	TUBB4B	0.061
Radix Saposhnikoviae（RS）	MOL011730	11-hydroxy-sec-o-beta-d-glucosylhamaudol_qt	Tryptophanyl-tRNA synthetase, mitochondrial	Q9UGM6	WARS2	0.061
Radix Saposhnikoviae（RS）	MOL011730	11-hydroxy-sec-o-beta-d-glucosylhamaudol_qt	Proto-oncogene serine/threonine-protein kinase Pim-1	P11309	PIM1	0.063
Radix Saposhnikoviae（RS）	MOL011730	11-hydroxy-sec-o-beta-d-glucosylhamaudol_qt	Nitric-oxide synthase, brain	P29475	NOS1	0.065
Radix Saposhnikoviae（RS）	MOL011730	11-hydroxy-sec-o-beta-d-glucosylhamaudol_qt	Muscarinic acetylcholine receptor M3	P20309	CHRM3	0.068
Radix Saposhnikoviae（RS）	MOL011730	11-hydroxy-sec-o-beta-d-glucosylhamaudol_qt	5-hydroxytryptamine 2A receptor	P28223	HTR2A	0.068
Radix Saposhnikoviae（RS）	MOL011730	11-hydroxy-sec-o-beta-d-glucosylhamaudol_qt	Gamma-aminobutyric-acid receptor subunit alpha-4	P48169	GABRA4	0.068
Radix Saposhnikoviae（RS）	MOL011730	11-hydroxy-sec-o-beta-d-glucosylhamaudol_qt	Proto-oncogene tyrosine-protein kinase LCK	P06239	LCK	0.07
Radix Saposhnikoviae（RS）	MOL011730	11-hydroxy-sec-o-beta-d-glucosylhamaudol_qt	Death-associated protein kinase 3	O43293	DAPK3	0.072
Radix Saposhnikoviae（RS）	MOL011730	11-hydroxy-sec-o-beta-d-glucosylhamaudol_qt	Glycogen synthase kinase-3 beta	P49841	GSK3B	0.072
Radix Saposhnikoviae（RS）	MOL011730	11-hydroxy-sec-o-beta-d-glucosylhamaudol_qt	D-HSCDK2	O75100	CA11	0.074
Radix Saposhnikoviae（RS）	MOL011730	11-hydroxy-sec-o-beta-d-glucosylhamaudol_qt	5-hydroxytryptamine 1A receptor	P08908	HTR1A	0.074
Radix Saposhnikoviae（RS）	MOL011730	11-hydroxy-sec-o-beta-d-glucosylhamaudol_qt	Gamma-aminobutyric-acid receptor subunit alpha-3	P34903	GABRA3	0.076
Radix Saposhnikoviae（RS）	MOL011730	11-hydroxy-sec-o-beta-d-glucosylhamaudol_qt	cAMP-dependent protein kinase inhibitor alpha	P61925	PKIA	0.079
Radix Saposhnikoviae（RS）	MOL011730	11-hydroxy-sec-o-beta-d-glucosylhamaudol_qt	Gamma-aminobutyric-acid receptor subunit alpha-5	P31644	GABRA5	0.083
Radix Saposhnikoviae（RS）	MOL011730	11-hydroxy-sec-o-beta-d-glucosylhamaudol_qt	Glyceraldehyde-3-phosphate dehydrogenase	P04406	GAPDH	0.084
Radix Saposhnikoviae（RS）	MOL011730	11-hydroxy-sec-o-beta-d-glucosylhamaudol_qt	Thyroid hormone receptor beta-1	P10828	THRB	0.084
Radix Saposhnikoviae（RS）	MOL011730	11-hydroxy-sec-o-beta-d-glucosylhamaudol_qt	Ig kappa chain V-II region RPMI 6410	P06310	IGKV2-30	0.085
Radix Saposhnikoviae（RS）	MOL011730	11-hydroxy-sec-o-beta-d-glucosylhamaudol_qt	Phosphatidylinositol-4,5-bisphosphate 3-kinase catalytic subunit gamma isoform	P48736	PIK3CG	0.085
Radix Saposhnikoviae（RS）	MOL011730	11-hydroxy-sec-o-beta-d-glucosylhamaudol_qt	Aldo-keto reductase family 1 member C1	Q04828	AKR1C1	0.085
Radix Saposhnikoviae（RS）	MOL011730	11-hydroxy-sec-o-beta-d-glucosylhamaudol_qt	Egl nine homolog 1	Q9GZT9	EGLN1	0.085
Radix Saposhnikoviae（RS）	MOL011730	11-hydroxy-sec-o-beta-d-glucosylhamaudol_qt	Toll-like receptor 7	Q9NYK1	TLR7	0.085

Radix Saposhnikoviae（RS）	MOL011730	11-hydroxy-sec-o-beta-d-glucosylhamaudol_qt	Gamma-aminobutyric-acid receptor subunit alpha-1	P14867	GABRA1	0.086
Radix Saposhnikoviae（RS）	MOL011730	11-hydroxy-sec-o-beta-d-glucosylhamaudol_qt	Gamma-aminobutyric acid type B receptor, subunit 1	Q9UBS5	GABBR1	0.086
Radix Saposhnikoviae（RS）	MOL011730	11-hydroxy-sec-o-beta-d-glucosylhamaudol_qt	Cyclin-A2	P20248	CCNA2	0.087
Radix Saposhnikoviae（RS）	MOL011730	11-hydroxy-sec-o-beta-d-glucosylhamaudol_qt	5-hydroxytryptamine 1D receptor	P28221	HTR1D	0.087
Radix Saposhnikoviae（RS）	MOL011730	11-hydroxy-sec-o-beta-d-glucosylhamaudol_qt	5-hydroxytryptamine 1B receptor	P28222	HTR1B	0.087
Radix Saposhnikoviae（RS）	MOL011730	11-hydroxy-sec-o-beta-d-glucosylhamaudol_qt	Membrane copper amine oxidase	Q16853	AOC3	0.087
Radix Saposhnikoviae（RS）	MOL011730	11-hydroxy-sec-o-beta-d-glucosylhamaudol_qt	NADPH oxidase organizer 1	Q8NFA2	NOXO1	0.087
Radix Saposhnikoviae（RS）	MOL011730	11-hydroxy-sec-o-beta-d-glucosylhamaudol_qt	Dihydrofolate reductase	P00374	DHFR	0.088
Radix Saposhnikoviae（RS）	MOL011730	11-hydroxy-sec-o-beta-d-glucosylhamaudol_qt	L-amino-acid oxidase	Q96RQ9	IL4I1	0.088
Radix Saposhnikoviae（RS）	MOL011730	11-hydroxy-sec-o-beta-d-glucosylhamaudol_qt	Glucocorticoid receptor	P04150	NR3C1	0.089
Radix Saposhnikoviae（RS）	MOL011730	11-hydroxy-sec-o-beta-d-glucosylhamaudol_qt	Melatonin receptor type 1A	P48039	MTNR1A	0.089
Radix Saposhnikoviae（RS）	MOL011730	11-hydroxy-sec-o-beta-d-glucosylhamaudol_qt	Beta-1 adrenergic receptor	P08588	ADRB1	0.09
Radix Saposhnikoviae（RS）	MOL011730	11-hydroxy-sec-o-beta-d-glucosylhamaudol_qt	cAMP-specific 3',5'-cyclic phosphodiesterase 4A	P27815	PDE4A	0.09
Radix Saposhnikoviae（RS）	MOL011730	11-hydroxy-sec-o-beta-d-glucosylhamaudol_qt	Neutrophil gelatinase-associated lipocalin	P80188	LCN2	0.091
Radix Saposhnikoviae（RS）	MOL011730	11-hydroxy-sec-o-beta-d-glucosylhamaudol_qt	Inosine-5'-monophosphate dehydrogenase 1	P20839	IMPDH1	0.092
Radix Saposhnikoviae（RS）	MOL011730	11-hydroxy-sec-o-beta-d-glucosylhamaudol_qt	Gamma-aminobutyric-acid receptor subunit alpha-2	P47869	GABRA2	0.093
Radix Saposhnikoviae（RS）	MOL011730	11-hydroxy-sec-o-beta-d-glucosylhamaudol_qt	Nuclear receptor coactivator 2	Q15596	NCOA2	0.093
Radix Saposhnikoviae（RS）	MOL011730	11-hydroxy-sec-o-beta-d-glucosylhamaudol_qt	Hydroxyacid oxidase 1	Q9UJM8	HAO1	0.093
Radix Saposhnikoviae（RS）	MOL011730	11-hydroxy-sec-o-beta-d-glucosylhamaudol_qt	Ribosyldihydronicotinamide dehydrogenase [quinone]	P16083	NQO2	0.094
Radix Saposhnikoviae（RS）	MOL011730	11-hydroxy-sec-o-beta-d-glucosylhamaudol_qt	Casein kinase II subunit alpha	P68400	CSNK2A1	0.094
Radix Saposhnikoviae（RS）	MOL011730	11-hydroxy-sec-o-beta-d-glucosylhamaudol_qt	Inhibitor of nuclear factor kappa-B kinase subunit alpha	O15111	CHUK	0.095
Radix Saposhnikoviae（RS）	MOL011730	11-hydroxy-sec-o-beta-d-glucosylhamaudol_qt	Endothelin-1 receptor	P25101	EDNRA	0.095
Radix Saposhnikoviae（RS）	MOL011730	11-hydroxy-sec-o-beta-d-glucosylhamaudol_qt	5-hydroxytryptamine 2C receptor	P28335	HTR2C	0.095
Radix Saposhnikoviae（RS）	MOL011730	11-hydroxy-sec-o-beta-d-glucosylhamaudol_qt	Triosephosphate isomerase	P60174	TPI1	0.095
Radix Saposhnikoviae（RS）	MOL011730	11-hydroxy-sec-o-beta-d-glucosylhamaudol_qt	Nuclear receptor coactivator 5	Q9HCD5	NCOA5	0.098
Radix Saposhnikoviae（RS）	MOL011730	11-hydroxy-sec-o-beta-d-glucosylhamaudol_qt	Low molecular weight phosphotyrosine protein phosphatase	P24666	ACP1	0.099
Radix Saposhnikoviae（RS）	MOL011730	11-hydroxy-sec-o-beta-d-glucosylhamaudol_qt	Thiamin pyrophosphokinase 1	Q9H3S4	TPK1	0.099
Radix Saposhnikoviae（RS）	MOL011730	11-hydroxy-sec-o-beta-d-glucosylhamaudol_qt	Carbonic anhydrase 1	P00915	CA1	0.1
Radix Saposhnikoviae（RS）	MOL011730	11-hydroxy-sec-o-beta-d-glucosylhamaudol_qt	Ig kappa chain C region	P01834	IGKC	0.1
Radix Saposhnikoviae（RS）	MOL011730	11-hydroxy-sec-o-beta-d-glucosylhamaudol_qt	DNA polymerase kappa	Q9UBT6	POLK	0.101
Radix Saposhnikoviae（RS）	MOL011730	11-hydroxy-sec-o-beta-d-glucosylhamaudol_qt	Cannabinoid receptor 2	P34972	CNR2	0.102
Radix Saposhnikoviae（RS）	MOL011730	11-hydroxy-sec-o-beta-d-glucosylhamaudol_qt	Alpha-1B adrenergic receptor	P35368	ADRA1B	0.104
Radix Saposhnikoviae（RS）	MOL011730	11-hydroxy-sec-o-beta-d-glucosylhamaudol_qt	Alpha-2A adrenergic receptor	P08913	ADRA2A	0.105
Radix Saposhnikoviae（RS）	MOL011730	11-hydroxy-sec-o-beta-d-glucosylhamaudol_qt	Alpha-2C adrenergic receptor	P18825	ADRA2C	0.105
Radix Saposhnikoviae（RS）	MOL011730	11-hydroxy-sec-o-beta-d-glucosylhamaudol_qt	D(4) dopamine receptor	P21917	DRD4	0.106
Radix Saposhnikoviae（RS）	MOL011730	11-hydroxy-sec-o-beta-d-glucosylhamaudol_qt	D(3) dopamine receptor	P35462	DRD3	0.106
Radix Saposhnikoviae（RS）	MOL011730	11-hydroxy-sec-o-beta-d-glucosylhamaudol_qt	3 beta-hydroxysteroid dehydrogenase/Delta 5-->4-isomerase type I	P14060	HSD3B1	0.109
Radix Saposhnikoviae（RS）	MOL011730	11-hydroxy-sec-o-beta-d-glucosylhamaudol_qt	MAP kinase-activated protein kinase 2	P49137	MAPKAPK2	0.109
Radix Saposhnikoviae（RS）	MOL011730	11-hydroxy-sec-o-beta-d-glucosylhamaudol_qt	Cell division control protein 2 homolog	P06493	CDK1	0.113
Radix Saposhnikoviae（RS）	MOL011730	11-hydroxy-sec-o-beta-d-glucosylhamaudol_qt	Cannabinoid receptor 1	P21554	CNR1	0.117
Radix Saposhnikoviae（RS）	MOL011730	11-hydroxy-sec-o-beta-d-glucosylhamaudol_qt	Carbonic anhydrase 2	P00918	CA2	0.118
Radix Saposhnikoviae（RS）	MOL011730	11-hydroxy-sec-o-beta-d-glucosylhamaudol_qt	Alpha-1A adrenergic receptor	P35348	ADRA1A	0.122
Radix Saposhnikoviae（RS）	MOL011730	11-hydroxy-sec-o-beta-d-glucosylhamaudol_qt	DNA polymerase	P04293	UL30	0.123
Radix Saposhnikoviae（RS）	MOL011730	11-hydroxy-sec-o-beta-d-glucosylhamaudol_qt	Alpha-2B adrenergic receptor	P18089	ADRA2B	0.123
Radix Saposhnikoviae（RS）	MOL011730	11-hydroxy-sec-o-beta-d-glucosylhamaudol_qt	Arachidonate 5-lipoxygenase	P09917	ALOX5	0.124
Radix Saposhnikoviae（RS）	MOL011730	11-hydroxy-sec-o-beta-d-glucosylhamaudol_qt	D(1B) dopamine receptor	P21918	DRD5	0.124
Radix Saposhnikoviae（RS）	MOL011730	11-hydroxy-sec-o-beta-d-glucosylhamaudol_qt	Tubulin alpha-3 chain	Q71U36	TUBA1A	0.126

Radix Saposhnikoviae（RS）	MOL011730	11-hydroxy-sec-o-beta-d-glucosylhamaudol_qt	Cell division protein kinase 5	Q00535	CDK5	0.152
Radix Saposhnikoviae（RS）	MOL011730	11-hydroxy-sec-o-beta-d-glucosylhamaudol_qt	D(2) dopamine receptor	P14416	DRD2	0.16
Radix Saposhnikoviae（RS）	MOL011730	11-hydroxy-sec-o-beta-d-glucosylhamaudol_qt	D(1A) dopamine receptor	P21728	DRD1	0.16
Radix Saposhnikoviae（RS）	MOL011730	11-hydroxy-sec-o-beta-d-glucosylhamaudol_qt	Estradiol 17-beta-dehydrogenase 1	P14061	HSD17B1	0.167
Radix Saposhnikoviae（RS）	MOL011730	11-hydroxy-sec-o-beta-d-glucosylhamaudol_qt	Mineralocorticoid receptor	P08235	NR3C2	0.17
Radix Saposhnikoviae（RS）	MOL011730	11-hydroxy-sec-o-beta-d-glucosylhamaudol_qt	Nuclear receptor coactivator 1	Q15788	NCOA1	0.191
Radix Saposhnikoviae（RS）	MOL011730	11-hydroxy-sec-o-beta-d-glucosylhamaudol_qt	S-methyl-5-thioadenosine phosphorylase	Q13126	MTAP	0.195
Radix Saposhnikoviae（RS）	MOL011730	11-hydroxy-sec-o-beta-d-glucosylhamaudol_qt	Nitric oxide synthase, inducible	P35228	NOS2	0.197
Radix Saposhnikoviae（RS）	MOL011730	11-hydroxy-sec-o-beta-d-glucosylhamaudol_qt	Delta-type opioid receptor	P41143	OPRD1	0.201
Radix Saposhnikoviae（RS）	MOL011730	11-hydroxy-sec-o-beta-d-glucosylhamaudol_qt	Nitric-oxide synthase, endothelial	P29474	NOS3	0.203
Radix Saposhnikoviae（RS）	MOL011730	11-hydroxy-sec-o-beta-d-glucosylhamaudol_qt	Androgen receptor	P10275	AR	0.226
Radix Saposhnikoviae（RS）	MOL011730	11-hydroxy-sec-o-beta-d-glucosylhamaudol_qt	Prothrombin	P00734	F2	0.271
Radix Saposhnikoviae（RS）	MOL011730	11-hydroxy-sec-o-beta-d-glucosylhamaudol_qt	Cell division protein kinase 2	P24941	CDK2	0.275
Radix Saposhnikoviae（RS）	MOL011730	11-hydroxy-sec-o-beta-d-glucosylhamaudol_qt	Kappa-type opioid receptor	P41145	OPRK1	0.29
Radix Saposhnikoviae（RS）	MOL011730	11-hydroxy-sec-o-beta-d-glucosylhamaudol_qt	Hemoglobin subunit alpha	P69905	HBA1	0.291
Radix Saposhnikoviae（RS）	MOL011730	11-hydroxy-sec-o-beta-d-glucosylhamaudol_qt	Estrogen receptor beta	Q92731	ESR2	0.294
Radix Saposhnikoviae（RS）	MOL011730	11-hydroxy-sec-o-beta-d-glucosylhamaudol_qt	Trypsin-1	P07477	PRSS1	0.357
Radix Saposhnikoviae（RS）	MOL011730	11-hydroxy-sec-o-beta-d-glucosylhamaudol_qt	Prostaglandin G/H synthase 1	P23219	PTGS1	0.415
Radix Saposhnikoviae（RS）	MOL011730	11-hydroxy-sec-o-beta-d-glucosylhamaudol_qt	Mu-type opioid receptor	P35372	OPRM1	0.461
Radix Saposhnikoviae（RS）	MOL011730	11-hydroxy-sec-o-beta-d-glucosylhamaudol_qt	cAMP-dependent protein kinase catalytic subunit alpha	P17612	PRKACA	0.659
Radix Saposhnikoviae（RS）	MOL011730	11-hydroxy-sec-o-beta-d-glucosylhamaudol_qt	Progesterone receptor	P06401	PGR	0.693
Radix Saposhnikoviae（RS）	MOL011730	11-hydroxy-sec-o-beta-d-glucosylhamaudol_qt	Prostaglandin G/H synthase 2	P35354	PTGS2	0.798
Radix Saposhnikoviae（RS）	MOL011730	11-hydroxy-sec-o-beta-d-glucosylhamaudol_qt	Estrogen receptor	P03372	ESR1	1
Radix Saposhnikoviae（RS）	MOL011732	anomalin	Serine/threonine-protein kinase Sgk1	O00141	SGK1	0.01
Radix Saposhnikoviae（RS）	MOL011732	anomalin	Ribosomal protein S6 kinase alpha-4	O75676	RPS6KA4	0.01
Radix Saposhnikoviae（RS）	MOL011732	anomalin	NADPH--cytochrome P450 reductase	P16435	POR	0.01
Radix Saposhnikoviae（RS）	MOL011732	anomalin	Nitric-oxide synthase, brain	P29475	NOS1	0.01
Radix Saposhnikoviae（RS）	MOL011732	anomalin	Flavin reductase	P30043	BLVRB	0.01
Radix Saposhnikoviae（RS）	MOL011732	anomalin	Dihydroorotate dehydrogenase, mitochondrial	Q02127	DHODH	0.01
Radix Saposhnikoviae（RS）	MOL011732	anomalin	Dihydropyrimidine dehydrogenase [NADP+]	Q12882	DPYD	0.01
Radix Saposhnikoviae（RS）	MOL011732	anomalin	Phosphopantothenoylcysteine decarboxylase	Q96CD2	PPCDC	0.01
Radix Saposhnikoviae（RS）	MOL011732	anomalin	Acetylcholine receptor subunit alpha	P02708	CHRNA1	0.013
Radix Saposhnikoviae（RS）	MOL011732	anomalin	Cholinesterase	P06276	BCHE	0.013
Radix Saposhnikoviae（RS）	MOL011732	anomalin	Acetylcholine receptor subunit gamma	P07510	CHRNG	0.013
Radix Saposhnikoviae（RS）	MOL011732	anomalin	Acetylcholine receptor subunit beta	P11230	CHRNB1	0.013
Radix Saposhnikoviae（RS）	MOL011732	anomalin	Neuronal acetylcholine receptor subunit beta-2	P17787	CHRNB2	0.013
Radix Saposhnikoviae（RS）	MOL011732	anomalin	Acetylcholinesterase	P22303	ACHE	0.013
Radix Saposhnikoviae（RS）	MOL011732	anomalin	Neuronal acetylcholine receptor subunit alpha-5	P30532	CHRNA5	0.013
Radix Saposhnikoviae（RS）	MOL011732	anomalin	Neuronal acetylcholine receptor subunit beta-4	P30926	CHRNB4	0.013
Radix Saposhnikoviae（RS）	MOL011732	anomalin	Neuronal acetylcholine receptor subunit alpha-3	P32297	CHRNA3	0.013
Radix Saposhnikoviae（RS）	MOL011732	anomalin	Neuronal acetylcholine receptor subunit alpha-7	P36544	CHRNA7	0.013
Radix Saposhnikoviae（RS）	MOL011732	anomalin	Acetylcholine receptor subunit epsilon	Q04844	CHRNE	0.013
Radix Saposhnikoviae（RS）	MOL011732	anomalin	Neuronal acetylcholine receptor subunit beta-3	Q05901	CHRNB3	0.013
Radix Saposhnikoviae（RS）	MOL011732	anomalin	Acetylcholine receptor subunit delta	Q07001	CHRND	0.013
Radix Saposhnikoviae（RS）	MOL011732	anomalin	Neuronal acetylcholine receptor subunit alpha-2	Q15822	CHRNA2	0.013
Radix Saposhnikoviae（RS）	MOL011732	anomalin	Neuronal acetylcholine receptor subunit alpha-6	Q15825	KCNJ8	0.013
Radix Saposhnikoviae（RS）	MOL011732	anomalin	Neuronal acetylcholine receptor subunit alpha-10	Q9GZZ6	CHRNA10	0.013

Radix Saposhnikoviae（RS）	MOL011732	anomalin	Neuronal acetylcholine receptor subunit alpha-9	Q9UGM1	CHRNA9	0.013
Radix Saposhnikoviae（RS）	MOL011732	anomalin	5-hydroxytryptamine 1A receptor	P08908	HTR1A	0.014
Radix Saposhnikoviae（RS）	MOL011732	anomalin	D(2) dopamine receptor	P14416	DRD2	0.014
Radix Saposhnikoviae（RS）	MOL011732	anomalin	Alpha-2B adrenergic receptor	P18089	ADRA2B	0.014
Radix Saposhnikoviae（RS）	MOL011732	anomalin	Alpha-2C adrenergic receptor	P18825	ADRA2C	0.014
Radix Saposhnikoviae（RS）	MOL011732	anomalin	D(1A) dopamine receptor	P21728	DRD1	0.014
Radix Saposhnikoviae（RS）	MOL011732	anomalin	D(4) dopamine receptor	P21917	DRD4	0.014
Radix Saposhnikoviae（RS）	MOL011732	anomalin	D(1B) dopamine receptor	P21918	DRD5	0.014
Radix Saposhnikoviae（RS）	MOL011732	anomalin	Alpha-1D adrenergic receptor	P25100	ADRA1D	0.014
Radix Saposhnikoviae（RS）	MOL011732	anomalin	5-hydroxytryptamine 1D receptor	P28221	HTR1D	0.014
Radix Saposhnikoviae（RS）	MOL011732	anomalin	5-hydroxytryptamine 1B receptor	P28222	HTR1B	0.014
Radix Saposhnikoviae（RS）	MOL011732	anomalin	5-hydroxytryptamine 2A receptor	P28223	HTR2A	0.014
Radix Saposhnikoviae（RS）	MOL011732	anomalin	5-hydroxytryptamine 2C receptor	P28335	HTR2C	0.014
Radix Saposhnikoviae（RS）	MOL011732	anomalin	Alpha-1A adrenergic receptor	P35348	ADRA1A	0.014
Radix Saposhnikoviae（RS）	MOL011732	anomalin	Alpha-1B adrenergic receptor	P35368	ADRA1B	0.014
Radix Saposhnikoviae（RS）	MOL011732	anomalin	D(3) dopamine receptor	P35462	DRD3	0.014
Radix Saposhnikoviae（RS）	MOL011732	anomalin	5-hydroxytryptamine 2B receptor	P41595	HTR2B	0.014
Radix Saposhnikoviae（RS）	MOL011732	anomalin	Pyridoxine-5'-phosphate oxidase	Q9NVS9	PNPO	0.014
Radix Saposhnikoviae（RS）	MOL011732	anomalin	Hydroxyacid oxidase 1	Q9UJM8	HAO1	0.024
Radix Saposhnikoviae（RS）	MOL011732	anomalin	Riboflavin kinase	Q969G6	RFK	0.03
Radix Saposhnikoviae（RS）	MOL011732	anomalin	Carbonic anhydrase 1	P00915	CA1	0.052
Radix Saposhnikoviae（RS）	MOL011732	anomalin	Carbonic anhydrase 2	P00918	CA2	0.052
Radix Saposhnikoviae（RS）	MOL011732	anomalin	Sodium/potassium-transporting ATPase alpha-1 chain	P05023	ATP1A1	0.052
Radix Saposhnikoviae（RS）	MOL011732	anomalin	Calcium-activated potassium channel subunit alpha 1	Q12791	KCNMA1	0.052
Radix Saposhnikoviae（RS）	MOL011732	anomalin	ATP-sensitive inward rectifier potassium channel 11	Q14654	KCNJ11	0.052
Radix Saposhnikoviae（RS）	MOL011732	anomalin	Potassium channel subfamily K member 6	Q9Y257	KCNK6	0.055
Radix Saposhnikoviae（RS）	MOL011732	anomalin	Gamma-aminobutyric acid receptor subunit rho-3	A8MPY1	GABRR3	0.059
Radix Saposhnikoviae（RS）	MOL011732	anomalin	Gamma-aminobutyric acid receptor subunit pi	O00591	GABRP	0.059
Radix Saposhnikoviae（RS）	MOL011732	anomalin	Gamma-aminobutyric acid receptor subunit delta	O14764	GABRD	0.059
Radix Saposhnikoviae（RS）	MOL011732	anomalin	Gamma-aminobutyric-acid receptor subunit beta-1	P18505	GABRB1	0.059
Radix Saposhnikoviae（RS）	MOL011732	anomalin	Gamma-aminobutyric acid receptor subunit gamma-2	P18507	GABRG2	0.059
Radix Saposhnikoviae（RS）	MOL011732	anomalin	Gamma-aminobutyric-acid receptor subunit rho-1	P24046	GABRR1	0.059
Radix Saposhnikoviae（RS）	MOL011732	anomalin	Gamma-aminobutyric acid receptor subunit rho-2	P28476	GABRR2	0.059
Radix Saposhnikoviae（RS）	MOL011732	anomalin	Gamma-aminobutyric-acid receptor subunit alpha-5	P31644	GABRA5	0.059
Radix Saposhnikoviae（RS）	MOL011732	anomalin	Gamma-aminobutyric-acid receptor subunit alpha-3	P34903	GABRA3	0.059
Radix Saposhnikoviae（RS）	MOL011732	anomalin	Gamma-aminobutyric-acid receptor subunit beta-2	P47870	GABRB2	0.059
Radix Saposhnikoviae（RS）	MOL011732	anomalin	Gamma-aminobutyric-acid receptor subunit alpha-4	P48169	GABRA4	0.059
Radix Saposhnikoviae（RS）	MOL011732	anomalin	Gamma-aminobutyric acid receptor subunit epsilon	P78334	GABRE	0.059
Radix Saposhnikoviae（RS）	MOL011732	anomalin	Gamma-aminobutyric-acid receptor subunit alpha-6	Q16445	GABRA6	0.059
Radix Saposhnikoviae（RS）	MOL011732	anomalin	Gamma-aminobutyric acid receptor subunit gamma-1	Q8N1C3	GABRG1	0.059
Radix Saposhnikoviae（RS）	MOL011732	anomalin	Gamma-aminobutyric acid receptor subunit gamma-3	Q99928	GABRG3	0.059
Radix Saposhnikoviae（RS）	MOL011732	anomalin	Gamma-aminobutyric acid receptor subunit theta	Q9UN88	GABRQ	0.059
Radix Saposhnikoviae（RS）	MOL011732	anomalin	Voltage-dependent T-type calcium channel subunit alpha-1H	O95180	CACNA1H	0.073
Radix Saposhnikoviae（RS）	MOL011732	anomalin	Troponin C, skeletal muscle	P02585	TNNC2	0.073
Radix Saposhnikoviae（RS）	MOL011732	anomalin	Voltage-dependent calcium channel subunit alpha-2/delta-1	P54289	CACNA2D1	0.073
Radix Saposhnikoviae（RS）	MOL011732	anomalin	Calcium/calmodulin-dependent 3',5'-cyclic nucleotide phosphodiesterase 1A	P54750	PDE1A	0.073
Radix Saposhnikoviae（RS）	MOL011732	anomalin	Calmodulin	P62158		0.073

Radix Saposhnikoviae（RS）	MOL011732	anomalin	Troponin C, slow skeletal and cardiac muscles	P63316	TNNC1	0.073
Radix Saposhnikoviae（RS）	MOL011732	anomalin	Voltage-dependent L-type calcium channel subunit alpha-1D	Q01668	CACNA1D	0.073
Radix Saposhnikoviae（RS）	MOL011732	anomalin	Voltage-dependent L-type calcium channel subunit beta-2	Q08289	CACNB2	0.073
Radix Saposhnikoviae（RS）	MOL011732	anomalin	Voltage-dependent L-type calcium channel subunit alpha-1S	Q13698	CACNA1S	0.073
Radix Saposhnikoviae（RS）	MOL011732	anomalin	Voltage-dependent L-type calcium channel subunit alpha-1C	Q13936	CACNA1C	0.073
Radix Saposhnikoviae（RS）	MOL011732	anomalin	Voltage-dependent calcium channel subunit alpha-2/delta-2	Q9NY47	CACNA2D2	0.073
Radix Saposhnikoviae（RS）	MOL011732	anomalin	cAMP-dependent protein kinase catalytic subunit alpha	P17612	PRKACA	0.091
Radix Saposhnikoviae（RS）	MOL011732	anomalin	cAMP-dependent protein kinase inhibitor alpha	P61925	PKIA	0.091
Radix Saposhnikoviae（RS）	MOL011732	anomalin	Rho-associated protein kinase 1	Q13464	ROCK1	0.091
Radix Saposhnikoviae（RS）	MOL011732	anomalin	Retinoic acid receptor RXR-alpha	P19793	RXRA	0.097
Radix Saposhnikoviae（RS）	MOL011732	anomalin	Nuclear receptor subfamily 1 group I member 3	Q14994	NR1I3	0.097
Radix Saposhnikoviae（RS）	MOL011732	anomalin	Nuclear receptor coactivator 1	Q15788	NCOA1	0.097
Radix Saposhnikoviae（RS）	MOL011732	anomalin	Potassium channel subfamily K member 1	O00180	KCNK1	0.102
Radix Saposhnikoviae（RS）	MOL011732	anomalin	Gamma-aminobutyric-acid receptor subunit alpha-1	P14867	GABRA1	0.106
Radix Saposhnikoviae（RS）	MOL011732	anomalin	Gamma-aminobutyric-acid receptor subunit alpha-2	P47869	GABRA2	0.106
Radix Saposhnikoviae（RS）	MOL011732	anomalin	Bile salt sulfotransferase	Q06520	SULT2A1	0.11
Radix Saposhnikoviae（RS）	MOL011732	anomalin	Tryptophanyl-tRNA synthetase, mitochondrial	Q9UGM6	WARS2	0.137
Radix Saposhnikoviae（RS）	MOL011732	anomalin	Prostacyclin receptor	P43119	PTGIR	0.138
Radix Saposhnikoviae（RS）	MOL011732	anomalin	Peroxisome proliferator-activated receptor delta	Q03181	PPARD	0.138
Radix Saposhnikoviae（RS）	MOL011732	anomalin	Mu-type opioid receptor	P35372	OPRM1	0.14
Radix Saposhnikoviae（RS）	MOL011732	anomalin	Kappa-type opioid receptor	P41145	OPRK1	0.14
Radix Saposhnikoviae（RS）	MOL011732	anomalin	Platelet glycoprotein IX	P14770	GP9	0.158
Radix Saposhnikoviae（RS）	MOL011732	anomalin	Sodium channel protein type 5 subunit alpha	Q14524	SCN5A	0.16
Radix Saposhnikoviae（RS）	MOL011732	anomalin	Estrogen receptor beta	Q92731	ESR2	0.171
Radix Saposhnikoviae（RS）	MOL011732	anomalin	Estradiol 17-beta-dehydrogenase 1	P14061	HSD17B1	0.224
Radix Saposhnikoviae（RS）	MOL011732	anomalin	Epidermal growth factor receptor	P00533	EGFR	0.279
Radix Saposhnikoviae（RS）	MOL011732	anomalin	ATP-binding cassette transporter sub-family C member 8	Q09428	ABCC8	0.285
Radix Saposhnikoviae（RS）	MOL011732	anomalin	Toll-like receptor 7	Q9NYK1	TLR7	0.288
Radix Saposhnikoviae（RS）	MOL011732	anomalin	Death-associated protein kinase 3	O43293	DAPK3	0.289
Radix Saposhnikoviae（RS）	MOL011732	anomalin	Triosephosphate isomerase	P60174	TPI1	0.289
Radix Saposhnikoviae（RS）	MOL011732	anomalin	Protein farnesyltransferase/geranylgeranyltransferase type I alpha subunit	P49354	FNTA	0.297
Radix Saposhnikoviae（RS）	MOL011732	anomalin	Muscarinic acetylcholine receptor M1	P11229	CHRM1	0.303
Radix Saposhnikoviae（RS）	MOL011732	anomalin	DNA polymerase kappa	Q9UBT6	POLK	0.313
Radix Saposhnikoviae（RS）	MOL011732	anomalin	Dual specificity protein kinase CLK1	P49759	CLK1	0.318
Radix Saposhnikoviae（RS）	MOL011732	anomalin	Melatonin receptor type 1A	P48039	MTNR1A	0.332
Radix Saposhnikoviae（RS）	MOL011732	anomalin	Mineralocorticoid receptor	P08235	NR3C2	0.344
Radix Saposhnikoviae（RS）	MOL011732	anomalin	Progesterone receptor	P06401	PGR	0.547
Radix Saposhnikoviae（RS）	MOL011732	anomalin	Cell division protein kinase 2	P24941	CDK2	0.617
Radix Saposhnikoviae（RS）	MOL011732	anomalin	Estrogen receptor	P03372	ESR1	1
Radix Saposhnikoviae（RS）	MOL011737	divaricatacid	Serine hydroxymethyltransferase, cytosolic	P34896	SHMT1	0.012
Radix Saposhnikoviae（RS）	MOL011737	divaricatacid	Phenylalanyl-tRNA synthetase, mitochondrial	O95363	FARS2	0.013
Radix Saposhnikoviae（RS）	MOL011737	divaricatacid	Phenylalanyl-tRNA synthetase beta chain	Q9NSD9	FARSB	0.013
Radix Saposhnikoviae（RS）	MOL011737	divaricatacid	Large neutral amino acids transporter small subunit 2	Q9UHI5	SLC7A8	0.013
Radix Saposhnikoviae（RS）	MOL011737	divaricatacid	Phenylalanyl-tRNA synthetase alpha chain	Q9Y285	FARSA	0.013
Radix Saposhnikoviae（RS）	MOL011737	divaricatacid	Gamma-aminobutyric-acid receptor subunit beta-3	P28472	GABRB3	0.014
Radix Saposhnikoviae（RS）	MOL011737	divaricatacid	Acetylcholine receptor subunit alpha	P02708	CHRNA1	0.015

Radix Saposhnikoviae（RS）	MOL011737	divaricatacid	Cholinesterase	P06276	BCHE	0.015
Radix Saposhnikoviae（RS）	MOL011737	divaricatacid	Acetylcholine receptor subunit gamma	P07510	CHRNG	0.015
Radix Saposhnikoviae（RS）	MOL011737	divaricatacid	Acetylcholine receptor subunit beta	P11230	CHRNB1	0.015
Radix Saposhnikoviae（RS）	MOL011737	divaricatacid	Neuronal acetylcholine receptor subunit beta-2	P17787	CHRNB2	0.015
Radix Saposhnikoviae（RS）	MOL011737	divaricatacid	Acetylcholinesterase	P22303	ACHE	0.015
Radix Saposhnikoviae（RS）	MOL011737	divaricatacid	Neuronal acetylcholine receptor subunit alpha-5	P30532	CHRNA5	0.015
Radix Saposhnikoviae（RS）	MOL011737	divaricatacid	Neuronal acetylcholine receptor subunit beta-4	P30926	CHRNB4	0.015
Radix Saposhnikoviae（RS）	MOL011737	divaricatacid	Neuronal acetylcholine receptor subunit alpha-4	P43681	CHRNA4	0.015
Radix Saposhnikoviae（RS）	MOL011737	divaricatacid	Acetylcholine receptor subunit epsilon	Q04844	CHRNE	0.015
Radix Saposhnikoviae（RS）	MOL011737	divaricatacid	Neuronal acetylcholine receptor subunit beta-3	Q05901	CHRNB3	0.015
Radix Saposhnikoviae（RS）	MOL011737	divaricatacid	Acetylcholine receptor subunit delta	Q07001	CHRND	0.015
Radix Saposhnikoviae（RS）	MOL011737	divaricatacid	Neuronal acetylcholine receptor subunit alpha-6	Q15825	KCNJ8	0.015
Radix Saposhnikoviae（RS）	MOL011737	divaricatacid	Neuronal acetylcholine receptor subunit alpha-10	Q9GZZ6	CHRNA10	0.015
Radix Saposhnikoviae（RS）	MOL011737	divaricatacid	Neuronal acetylcholine receptor subunit alpha-9	Q9UGM1	CHRNA9	0.015
Radix Saposhnikoviae（RS）	MOL011737	divaricatacid	Opioid receptor, sigma 1	Q5T1J1	SIGMAR1	0.02
Radix Saposhnikoviae（RS）	MOL011737	divaricatacid	Glutamate [NMDA] receptor subunit 3A	Q8TCU5	GRIN3A	0.02
Radix Saposhnikoviae（RS）	MOL011737	divaricatacid	Sigma 1-type opioid receptor	Q99720	SIGMAR1	0.02
Radix Saposhnikoviae（RS）	MOL011737	divaricatacid	Tyrosine aminotransferase	P17735	TAT	0.022
Radix Saposhnikoviae（RS）	MOL011737	divaricatacid	Gamma-aminobutyric acid receptor subunit rho-3	A8MPY1	GABRR3	0.025
Radix Saposhnikoviae（RS）	MOL011737	divaricatacid	Gamma-aminobutyric acid receptor subunit pi	O00591	GABRP	0.025
Radix Saposhnikoviae（RS）	MOL011737	divaricatacid	Gamma-aminobutyric acid receptor subunit delta	O14764	GABRD	0.025
Radix Saposhnikoviae（RS）	MOL011737	divaricatacid	Gamma-aminobutyric-acid receptor subunit alpha-1	P14867	GABRA1	0.025
Radix Saposhnikoviae（RS）	MOL011737	divaricatacid	Gamma-aminobutyric-acid receptor subunit beta-1	P18505	GABRB1	0.025
Radix Saposhnikoviae（RS）	MOL011737	divaricatacid	Gamma-aminobutyric acid receptor subunit gamma-2	P18507	GABRG2	0.025
Radix Saposhnikoviae（RS）	MOL011737	divaricatacid	Gamma-aminobutyric-acid receptor subunit rho-1	P24046	GABRR1	0.025
Radix Saposhnikoviae（RS）	MOL011737	divaricatacid	Gamma-aminobutyric acid receptor subunit rho-2	P28476	GABRR2	0.025
Radix Saposhnikoviae（RS）	MOL011737	divaricatacid	Gamma-aminobutyric-acid receptor subunit alpha-5	P31644	GABRA5	0.025
Radix Saposhnikoviae（RS）	MOL011737	divaricatacid	Gamma-aminobutyric-acid receptor subunit alpha-3	P34903	GABRA3	0.025
Radix Saposhnikoviae（RS）	MOL011737	divaricatacid	Gamma-aminobutyric-acid receptor subunit alpha-2	P47869	GABRA2	0.025
Radix Saposhnikoviae（RS）	MOL011737	divaricatacid	Gamma-aminobutyric acid receptor subunit epsilon	P78334	GABRE	0.025
Radix Saposhnikoviae（RS）	MOL011737	divaricatacid	Gamma-aminobutyric acid receptor subunit gamma-1	Q8N1C3	GABRG1	0.025
Radix Saposhnikoviae（RS）	MOL011737	divaricatacid	Gamma-aminobutyric acid receptor subunit gamma-3	Q99928	GABRG3	0.025
Radix Saposhnikoviae（RS）	MOL011737	divaricatacid	Beta-2 adrenergic receptor	P07550	ADRB2	0.026
Radix Saposhnikoviae（RS）	MOL011737	divaricatacid	Muscarinic acetylcholine receptor M1	P11229	CHRM1	0.026
Radix Saposhnikoviae（RS）	MOL011737	divaricatacid	Amine oxidase [flavin-containing] A	P21397	MAOA	0.026
Radix Saposhnikoviae（RS）	MOL011737	divaricatacid	Sodium-dependent serotonin transporter	P31645	SLC6A4	0.026
Radix Saposhnikoviae（RS）	MOL011737	divaricatacid	Chromaffin granule amine transporter	P54219	SLC18A1	0.026
Radix Saposhnikoviae（RS）	MOL011737	divaricatacid	Sodium-dependent dopamine transporter	Q01959	SLC6A3	0.026
Radix Saposhnikoviae（RS）	MOL011737	divaricatacid	Synaptic vesicular amine transporter	Q05940	SLC18A2	0.026
Radix Saposhnikoviae（RS）	MOL011737	divaricatacid	5-hydroxytryptamine 6 receptor	P50406	HTR6	0.027
Radix Saposhnikoviae（RS）	MOL011737	divaricatacid	Glutamate receptor 2	P42262	GRIA2	0.028
Radix Saposhnikoviae（RS）	MOL011737	divaricatacid	5-hydroxytryptamine 2B receptor	P41595	HTR2B	0.029
Radix Saposhnikoviae（RS）	MOL011737	divaricatacid	D1 dopamine receptor-interacting protein calcyon	Q9NYX4	CALY	0.029
Radix Saposhnikoviae（RS）	MOL011737	divaricatacid	Serine/threonine-protein kinase 17B	O94768	STK17B	0.038
Radix Saposhnikoviae（RS）	MOL011737	divaricatacid	Purine nucleoside phosphorylase	P00491	PNP	0.038
Radix Saposhnikoviae（RS）	MOL011737	divaricatacid	DNA polymerase alpha catalytic subunit	P09884	POLA1	0.038
Radix Saposhnikoviae（RS）	MOL011737	divaricatacid	Proto-oncogene serine/threonine-protein kinase Pim-1	P11309	PIM1	0.038

Radix Saposhnikoviae（RS）	MOL011737	divaricatacid	Ribonucleoside-diphosphate reductase large subunit	P23921	RRM1	0.038
Radix Saposhnikoviae（RS）	MOL011737	divaricatacid	Phosphatidylinositol 3-kinase regulatory subunit alpha	P27986	PIK3R1	0.038
Radix Saposhnikoviae（RS）	MOL011737	divaricatacid	Ribonucleoside-diphosphate reductase M2 subunit	P31350	RRM2	0.038
Radix Saposhnikoviae（RS）	MOL011737	divaricatacid	ATP synthase subunit gamma, mitochondrial	P36542	ATP5F1C	0.038
Radix Saposhnikoviae（RS）	MOL011737	divaricatacid	Serine/threonine-protein kinase PLK1	P53350	PLK1	0.038
Radix Saposhnikoviae（RS）	MOL011737	divaricatacid	DNA polymerase epsilon subunit 2	P56282	POLE2	0.038
Radix Saposhnikoviae（RS）	MOL011737	divaricatacid	DNA polymerase epsilon catalytic subunit A	Q07864	POLE	0.038
Radix Saposhnikoviae（RS）	MOL011737	divaricatacid	UDP-glucuronosyltransferase 3A1	Q6NUS8	UGT3A1	0.038
Radix Saposhnikoviae（RS）	MOL011737	divaricatacid	Ribonucleoside-diphosphate reductase subunit M2 B	Q7LG56	RRM2B	0.038
Radix Saposhnikoviae（RS）	MOL011737	divaricatacid	DNA polymerase epsilon subunit 3	Q9NRF9	POLE3	0.038
Radix Saposhnikoviae（RS）	MOL011737	divaricatacid	fMet-Leu-Phe receptor	P21462	FPR1	0.039
Radix Saposhnikoviae（RS）	MOL011737	divaricatacid	Neuronal acetylcholine receptor subunit alpha-3	P32297	CHRNA3	0.039
Radix Saposhnikoviae（RS）	MOL011737	divaricatacid	Neuronal acetylcholine receptor subunit alpha-7	P36544	CHRNA7	0.039
Radix Saposhnikoviae（RS）	MOL011737	divaricatacid	Prostaglandin D2 receptor	Q13258	PTGDR	0.039
Radix Saposhnikoviae（RS）	MOL011737	divaricatacid	Cysteinyl leukotriene receptor 2	Q9NS75	CYSLTR2	0.039
Radix Saposhnikoviae（RS）	MOL011737	divaricatacid	Cysteinyl leukotriene receptor 1	Q9Y271	CYSLTR1	0.039
Radix Saposhnikoviae（RS）	MOL011737	divaricatacid	Neuronal acetylcholine receptor subunit alpha-2	Q15822	CHRNA2	0.044
Radix Saposhnikoviae（RS）	MOL011737	divaricatacid	Phenylalanine-4-hydroxylase	P00439	PAH	0.047
Radix Saposhnikoviae（RS）	MOL011737	divaricatacid	5-hydroxytryptamine 3 receptor	P46098	HTR3A	0.049
Radix Saposhnikoviae（RS）	MOL011737	divaricatacid	5-hydroxytryptamine 4 receptor	Q13639	HTR4	0.049
Radix Saposhnikoviae（RS）	MOL011737	divaricatacid	Mitogen-activated protein kinase 8	P45983	MAPK8	0.054
Radix Saposhnikoviae（RS）	MOL011737	divaricatacid	Mitogen-activated protein kinase 10	P53779	MAPK10	0.054
Radix Saposhnikoviae（RS）	MOL011737	divaricatacid	C-jun-amino-terminal kinase-interacting protein 1	Q9UQF2	MAPK8IP1	0.054
Radix Saposhnikoviae（RS）	MOL011737	divaricatacid	Dihydrolipoyllysine-residue acetyltransferase component of pyruvate dehydrogenase complex, mitochondrial	P10515	DLAT	0.057
Radix Saposhnikoviae（RS）	MOL011737	divaricatacid	Glycine cleavage system H protein, mitochondrial	P23434	GCSH	0.057
Radix Saposhnikoviae（RS）	MOL011737	divaricatacid	Beta-1 adrenergic receptor	P08588	ADRB1	0.06
Radix Saposhnikoviae（RS）	MOL011737	divaricatacid	Sodium-dependent noradrenaline transporter	P23975	SLC6A2	0.06
Radix Saposhnikoviae（RS）	MOL011737	divaricatacid	Muscarinic acetylcholine receptor M2	P08172	CHRM2	0.061
Radix Saposhnikoviae（RS）	MOL011737	divaricatacid	Muscarinic acetylcholine receptor M4	P08173	CHRM4	0.061
Radix Saposhnikoviae（RS）	MOL011737	divaricatacid	Muscarinic acetylcholine receptor M5	P08912	CHRM5	0.061
Radix Saposhnikoviae（RS）	MOL011737	divaricatacid	Muscarinic acetylcholine receptor M3	P20309	CHRM3	0.061
Radix Saposhnikoviae（RS）	MOL011737	divaricatacid	Alpha-1D adrenergic receptor	P25100	ADRA1D	0.061
Radix Saposhnikoviae（RS）	MOL011737	divaricatacid	Histamine H1 receptor	P35367	HRH1	0.061
Radix Saposhnikoviae（RS）	MOL011737	divaricatacid	Alpha-1B adrenergic receptor	P35368	ADRA1B	0.061
Radix Saposhnikoviae（RS）	MOL011737	divaricatacid	Nociceptin receptor	P41146	OPRL1	0.063
Radix Saposhnikoviae（RS）	MOL011737	divaricatacid	D(4) dopamine receptor	P21917	DRD4	0.065
Radix Saposhnikoviae（RS）	MOL011737	divaricatacid	5-hydroxytryptamine 2C receptor	P28335	HTR2C	0.065
Radix Saposhnikoviae（RS）	MOL011737	divaricatacid	Bile salt sulfotransferase	Q06520	SULT2A1	0.065
Radix Saposhnikoviae（RS）	MOL011737	divaricatacid	Prostaglandin reductase 1	Q14914	PTGR1	0.069
Radix Saposhnikoviae（RS）	MOL011737	divaricatacid	Tyrosine 3-monooxygenase	P07101	TH	0.07
Radix Saposhnikoviae（RS）	MOL011737	divaricatacid	Peptidyl-prolyl cis-trans isomerase NIMA-interacting 1	Q13526	PIN1	0.07
Radix Saposhnikoviae（RS）	MOL011737	divaricatacid	Tyrosyl-tRNA synthetase, mitochondrial	Q9Y2Z4	YARS2	0.07
Radix Saposhnikoviae（RS）	MOL011737	divaricatacid	Myeloperoxidase	P05164	MPO	0.071
Radix Saposhnikoviae（RS）	MOL011737	divaricatacid	Eosinophil peroxidase	P11678	EPX	0.071
Radix Saposhnikoviae（RS）	MOL011737	divaricatacid	Ribosyldihydronicotinamide dehydrogenase [quinone]	P16083	NQO2	0.071
Radix Saposhnikoviae（RS）	MOL011737	divaricatacid	Calreticulin	P27797	CALR	0.071

Radix Saposhnikoviae（RS）	MOL011737	divaricatacid	Melatonin receptor type 1B	P49286	MTNR1B	0.071
Radix Saposhnikoviae（RS）	MOL011737	divaricatacid	Calmodulin	P62158		0.071
Radix Saposhnikoviae（RS）	MOL011737	divaricatacid	Nuclear receptor ROR-beta	Q92753	RORB	0.071
Radix Saposhnikoviae（RS）	MOL011737	divaricatacid	5-hydroxytryptamine 1F receptor	P30939	HTR1F	0.075
Radix Saposhnikoviae（RS）	MOL011737	divaricatacid	Proto-oncogene tyrosine-protein kinase LCK	P06239	LCK	0.078
Radix Saposhnikoviae（RS）	MOL011737	divaricatacid	Tyrosine-protein kinase Lyn	P07948	LYN	0.078
Radix Saposhnikoviae（RS）	MOL011737	divaricatacid	3 beta-hydroxysteroid dehydrogenase/Delta 5-->4-isomerase type II	P26439	HSD3B2	0.08
Radix Saposhnikoviae（RS）	MOL011737	divaricatacid	Peptidyl-prolyl cis-trans isomerase, mitochondrial	P30405	PPIF	0.08
Radix Saposhnikoviae（RS）	MOL011737	divaricatacid	S-phase kinase-associated protein 1	P63208	SKP1	0.081
Radix Saposhnikoviae（RS）	MOL011737	divaricatacid	Group IIE secretory phospholipase A2	Q9NZK7	PLA2G2E	0.081
Radix Saposhnikoviae（RS）	MOL011737	divaricatacid	Casein kinase II subunit alpha	P68400	CSNK2A1	0.083
Radix Saposhnikoviae（RS）	MOL011737	divaricatacid	ATP synthase subunit beta, mitochondrial	P06576	ATP5F1B	0.084
Radix Saposhnikoviae（RS）	MOL011737	divaricatacid	Tyrosine-protein kinase HCK	P08631	HCK	0.084
Radix Saposhnikoviae（RS）	MOL011737	divaricatacid	Carbonic anhydrase 4	P22748	CA4	0.084
Radix Saposhnikoviae（RS）	MOL011737	divaricatacid	ATP synthase subunit alpha, mitochondrial	P25705	ATP5F1A	0.084
Radix Saposhnikoviae（RS）	MOL011737	divaricatacid	Phosphatidylinositol-4,5-bisphosphate 3-kinase catalytic subunit gamma isoform	P48736	PIK3CG	0.084
Radix Saposhnikoviae（RS）	MOL011737	divaricatacid	Calcium-activated potassium channel subunit alpha 1	Q12791	KCNMA1	0.084
Radix Saposhnikoviae（RS）	MOL011737	divaricatacid	ATP-sensitive inward rectifier potassium channel 11	Q14654	KCNJ11	0.084
Radix Saposhnikoviae（RS）	MOL011737	divaricatacid	cAMP-specific 3',5'-cyclic phosphodiesterase 4B	Q07343	PDE4B	0.085
Radix Saposhnikoviae（RS）	MOL011737	divaricatacid	cGMP-inhibited 3',5'-cyclic phosphodiesterase A	Q14432	PDE3A	0.085
Radix Saposhnikoviae（RS）	MOL011737	divaricatacid	Tubulin beta-2C chain	P68371	TUBB4B	0.087
Radix Saposhnikoviae（RS）	MOL011737	divaricatacid	Tubulin alpha-3 chain	Q71U36	TUBA1A	0.087
Radix Saposhnikoviae（RS）	MOL011737	divaricatacid	Prolactin receptor	P16471	PRLR	0.088
Radix Saposhnikoviae（RS）	MOL011737	divaricatacid	D-HSCDK2	O75100	CA11	0.089
Radix Saposhnikoviae（RS）	MOL011737	divaricatacid	Cell division control protein 2 homolog	P06493	CDK1	0.089
Radix Saposhnikoviae（RS）	MOL011737	divaricatacid	Sterol O-acyltransferase 2	O75908	SOAT2	0.09
Radix Saposhnikoviae（RS）	MOL011737	divaricatacid	Sterol O-acyltransferase 1	P35610	SOAT1	0.09
Radix Saposhnikoviae（RS）	MOL011737	divaricatacid	Methionine aminopeptidase 1	P53582	METAP1	0.09
Radix Saposhnikoviae（RS）	MOL011737	divaricatacid	Nitric-oxide synthase, endothelial	P29474	NOS3	0.091
Radix Saposhnikoviae（RS）	MOL011737	divaricatacid	Nitric-oxide synthase, brain	P29475	NOS1	0.091
Radix Saposhnikoviae（RS）	MOL011737	divaricatacid	Alpha-2C adrenergic receptor	P18825	ADRA2C	0.099
Radix Saposhnikoviae（RS）	MOL011737	divaricatacid	Alpha-1A adrenergic receptor	P35348	ADRA1A	0.099
Radix Saposhnikoviae（RS）	MOL011737	divaricatacid	cAMP-dependent protein kinase inhibitor alpha	P61925	PKIA	0.1
Radix Saposhnikoviae（RS）	MOL011737	divaricatacid	D(1B) dopamine receptor	P21918	DRD5	0.101
Radix Saposhnikoviae（RS）	MOL011737	divaricatacid	D(3) dopamine receptor	P35462	DRD3	0.101
Radix Saposhnikoviae（RS）	MOL011737	divaricatacid	5-hydroxytryptamine 1B receptor	P28222	HTR1B	0.112
Radix Saposhnikoviae（RS）	MOL011737	divaricatacid	Tryptophanyl-tRNA synthetase, mitochondrial	Q9UGM6	WARS2	0.121
Radix Saposhnikoviae（RS）	MOL011737	divaricatacid	5-hydroxytryptamine 1A receptor	P08908	HTR1A	0.124
Radix Saposhnikoviae（RS）	MOL011737	divaricatacid	Death-associated protein kinase 3	O43293	DAPK3	0.126
Radix Saposhnikoviae（RS）	MOL011737	divaricatacid	Tyrosine-protein kinase JAK2	O60674	JAK2	0.126
Radix Saposhnikoviae（RS）	MOL011737	divaricatacid	Tyrosine-protein kinase JAK1	P23458	JAK1	0.126
Radix Saposhnikoviae（RS）	MOL011737	divaricatacid	Tyrosine-protein kinase JAK3	P52333	JAK3	0.126
Radix Saposhnikoviae（RS）	MOL011737	divaricatacid	Tripartite motif-containing protein 13	O60858	TRIM13	0.134
Radix Saposhnikoviae（RS）	MOL011737	divaricatacid	Alpha-2A adrenergic receptor	P08913	ADRA2A	0.134
Radix Saposhnikoviae（RS）	MOL011737	divaricatacid	Alpha-2B adrenergic receptor	P18089	ADRA2B	0.134
Radix Saposhnikoviae（RS）	MOL011737	divaricatacid	Tyrosyl-tRNA synthetase, cytoplasmic	P54577	YARS	0.147
Radix Saposhnikoviae（RS）	MOL011737	divaricatacid	Melatonin receptor type 1A	P48039	MTNR1A	0.148

Radix Saposhnikoviae（RS）	MOL011737	divaricatacid	5-hydroxytryptamine 1D receptor	P28221	HTR1D	0.153
Radix Saposhnikoviae（RS）	MOL011737	divaricatacid	3 beta-hydroxysteroid dehydrogenase/Delta 5-->4-isomerase type I	P14060	HSD3B1	0.16
Radix Saposhnikoviae（RS）	MOL011737	divaricatacid	Poly [ADP-ribose] polymerase 1	P09874	PARP1	0.166
Radix Saposhnikoviae（RS）	MOL011737	divaricatacid	Cyclin-A2	P20248	CCNA2	0.166
Radix Saposhnikoviae（RS）	MOL011737	divaricatacid	5-hydroxytryptamine 2A receptor	P28223	HTR2A	0.17
Radix Saposhnikoviae（RS）	MOL011737	divaricatacid	Ig heavy chain V-I region ND	P01744	IGKC	0.173
Radix Saposhnikoviae（RS）	MOL011737	divaricatacid	Thyroid hormone receptor, alpha isoform 1 variant	Q59FW3	SIGMAR1	0.174
Radix Saposhnikoviae（RS）	MOL011737	divaricatacid	Neuropeptide Y	P01303	NPY	0.175
Radix Saposhnikoviae（RS）	MOL011737	divaricatacid	Sodium/potassium-transporting ATPase alpha-1 chain	P05023	ATP1A1	0.175
Radix Saposhnikoviae（RS）	MOL011737	divaricatacid	D(1A) dopamine receptor	P21728	DRD1	0.175
Radix Saposhnikoviae（RS）	MOL011737	divaricatacid	Glucocorticoid receptor	P04150	NR3C1	0.177
Radix Saposhnikoviae（RS）	MOL011737	divaricatacid	Cannabinoid receptor 1	P21554	CNR1	0.179
Radix Saposhnikoviae（RS）	MOL011737	divaricatacid	Phospholipase A2	P04054	PLA2G1B	0.18
Radix Saposhnikoviae（RS）	MOL011737	divaricatacid	Tyrosine-protein phosphatase non-receptor type 1	P18031	PTPN1	0.182
Radix Saposhnikoviae（RS）	MOL011737	divaricatacid	Low molecular weight phosphotyrosine protein phosphatase	P24666	ACP1	0.182
Radix Saposhnikoviae（RS）	MOL011737	divaricatacid	Ig kappa chain C region	P01834	IGKC	0.184
Radix Saposhnikoviae（RS）	MOL011737	divaricatacid	Nuclear receptor coactivator 5	Q9HCD5	NCOA5	0.184
Radix Saposhnikoviae（RS）	MOL011737	divaricatacid	Triosephosphate isomerase	P60174	TPI1	0.185
Radix Saposhnikoviae（RS）	MOL011737	divaricatacid	Cell division protein kinase 5	Q00535	CDK5	0.186
Radix Saposhnikoviae（RS）	MOL011737	divaricatacid	Androgen receptor	P10275	AR	0.188
Radix Saposhnikoviae（RS）	MOL011737	divaricatacid	MAP kinase-activated protein kinase 2	P49137	MAPKAPK2	0.189
Radix Saposhnikoviae（RS）	MOL011737	divaricatacid	Inosine-5'-monophosphate dehydrogenase 1	P20839	IMPDH1	0.191
Radix Saposhnikoviae（RS）	MOL011737	divaricatacid	DNA polymerase kappa	Q9UBT6	POLK	0.191
Radix Saposhnikoviae（RS）	MOL011737	divaricatacid	Hydroxyacid oxidase 1	Q9UJM8	HAO1	0.196
Radix Saposhnikoviae（RS）	MOL011737	divaricatacid	cAMP-dependent protein kinase catalytic subunit alpha	P17612	PRKACA	0.208
Radix Saposhnikoviae（RS）	MOL011737	divaricatacid	D(2) dopamine receptor	P14416	DRD2	0.211
Radix Saposhnikoviae（RS）	MOL011737	divaricatacid	Estradiol 17-beta-dehydrogenase 1	P14061	HSD17B1	0.22
Radix Saposhnikoviae（RS）	MOL011737	divaricatacid	Carbonic anhydrase 2	P00918	CA2	0.267
Radix Saposhnikoviae（RS）	MOL011737	divaricatacid	Delta-type opioid receptor	P41143	OPRD1	0.276
Radix Saposhnikoviae（RS）	MOL011737	divaricatacid	Mineralocorticoid receptor	P08235	NR3C2	0.294
Radix Saposhnikoviae（RS）	MOL011737	divaricatacid	Prostaglandin G/H synthase 1	P23219	PTGS1	0.326
Radix Saposhnikoviae（RS）	MOL011737	divaricatacid	cAMP-specific 3',5'-cyclic phosphodiesterase 4D	Q08499	PDE4D	0.343
Radix Saposhnikoviae（RS）	MOL011737	divaricatacid	Prothrombin	P00734	F2	0.352
Radix Saposhnikoviae（RS）	MOL011737	divaricatacid	Estrogen receptor beta	Q92731	ESR2	0.358
Radix Saposhnikoviae（RS）	MOL011737	divaricatacid	Carbonic anhydrase 1	P00915	CA1	0.359
Radix Saposhnikoviae（RS）	MOL011737	divaricatacid	Nitric oxide synthase, inducible	P35228	NOS2	0.391
Radix Saposhnikoviae（RS）	MOL011737	divaricatacid	Progesterone receptor	P06401	PGR	0.394
Radix Saposhnikoviae（RS）	MOL011737	divaricatacid	Kappa-type opioid receptor	P41145	OPRK1	0.488
Radix Saposhnikoviae（RS）	MOL011737	divaricatacid	Hemoglobin subunit alpha	P69905	HBA1	0.582
Radix Saposhnikoviae（RS）	MOL011737	divaricatacid	Trypsin-1	P07477	PRSS1	0.746
Radix Saposhnikoviae（RS）	MOL011737	divaricatacid	Cell division protein kinase 2	P24941	CDK2	0.746
Radix Saposhnikoviae（RS）	MOL011737	divaricatacid	Mu-type opioid receptor	P35372	OPRM1	0.828
Radix Saposhnikoviae（RS）	MOL011737	divaricatacid	Estrogen receptor	P03372	ESR1	0.997
Radix Saposhnikoviae（RS）	MOL011737	divaricatacid	Prostaglandin G/H synthase 2	P35354	PTGS2	1
Radix Saposhnikoviae（RS）	MOL011740	divaricatol	Kynurenine--oxoglutarate transaminase 1	Q16773	KYAT1	0.01
Radix Saposhnikoviae（RS）	MOL011740	divaricatol	Voltage-dependent T-type calcium channel subunit alpha-1G	O43497	CACNA1G	0.011
Radix Saposhnikoviae（RS）	MOL011740	divaricatol	Carbonic anhydrase 12	O43570	CA12	0.011

Radix Saposhnikoviae（RS）	MOL011740	divaricatol	Carbonic anhydrase-related protein 11	O75493	CA11	0.011
Radix Saposhnikoviae（RS）	MOL011740	divaricatol	Voltage-dependent T-type calcium channel subunit alpha-1H	O95180	CACNA1H	0.011
Radix Saposhnikoviae（RS）	MOL011740	divaricatol	Carbonic anhydrase 3	P07451	CA3	0.011
Radix Saposhnikoviae（RS）	MOL011740	divaricatol	Amine oxidase [flavin-containing] A	P21397	MAOA	0.011
Radix Saposhnikoviae（RS）	MOL011740	divaricatol	Carbonic anhydrase 6	P23280	CA6	0.011
Radix Saposhnikoviae（RS）	MOL011740	divaricatol	Amine oxidase [flavin-containing] B	P27338	MAOB	0.011
Radix Saposhnikoviae（RS）	MOL011740	divaricatol	Carbonic anhydrase 5A, mitochondrial	P35218	CA5A	0.011
Radix Saposhnikoviae（RS）	MOL011740	divaricatol	Carbonic anhydrase-related protein	P35219	CA8	0.011
Radix Saposhnikoviae（RS）	MOL011740	divaricatol	Sodium channel protein type 4 subunit alpha	P35499	SCN4A	0.011
Radix Saposhnikoviae（RS）	MOL011740	divaricatol	Carbonic anhydrase 7	P43166	CA7	0.011
Radix Saposhnikoviae（RS）	MOL011740	divaricatol	Sodium channel subunit beta-1	Q07699	SCN1B	0.011
Radix Saposhnikoviae（RS）	MOL011740	divaricatol	Sodium channel protein type 9 subunit alpha	Q15858	SCN9A	0.011
Radix Saposhnikoviae（RS）	MOL011740	divaricatol	Carbonic anhydrase 9	Q16790	CA9	0.011
Radix Saposhnikoviae（RS）	MOL011740	divaricatol	Sodium channel subunit beta-4	Q8IWT1	SCN4B	0.011
Radix Saposhnikoviae（RS）	MOL011740	divaricatol	Carbonic anhydrase 13	Q8N1Q1	CA13	0.011
Radix Saposhnikoviae（RS）	MOL011740	divaricatol	Sodium channel protein type 2 subunit alpha	Q99250	SCN2A	0.011
Radix Saposhnikoviae（RS）	MOL011740	divaricatol	Carbonic anhydrase-related protein 10	Q9NS85	CA10	0.011
Radix Saposhnikoviae（RS）	MOL011740	divaricatol	Sodium channel protein type 3 subunit alpha	Q9NY46	SCN3A	0.011
Radix Saposhnikoviae（RS）	MOL011740	divaricatol	Sodium channel subunit beta-3	Q9NY72	SCN3B	0.011
Radix Saposhnikoviae（RS）	MOL011740	divaricatol	Voltage-dependent T-type calcium channel subunit alpha-1I	Q9P0X4	CACNA1I	0.011
Radix Saposhnikoviae（RS）	MOL011740	divaricatol	Sodium channel protein type 11 subunit alpha	Q9UI33	SCN11A	0.011
Radix Saposhnikoviae（RS）	MOL011740	divaricatol	Carbonic anhydrase 14	Q9ULX7	CA14	0.011
Radix Saposhnikoviae（RS）	MOL011740	divaricatol	Carbonic anhydrase 5B, mitochondrial	Q9Y2D0	CA5B	0.011
Radix Saposhnikoviae（RS）	MOL011740	divaricatol	Potassium channel subfamily K member 6	Q9Y257	KCNK6	0.012
Radix Saposhnikoviae（RS）	MOL011740	divaricatol	Sodium/potassium-transporting ATPase alpha-1 chain	P05023	ATP1A1	0.013
Radix Saposhnikoviae（RS）	MOL011740	divaricatol	Solute carrier family 12 member 2	P55011	SLC12A2	0.013
Radix Saposhnikoviae（RS）	MOL011740	divaricatol	Calcium-activated potassium channel subunit alpha 1	Q12791	KCNMA1	0.013
Radix Saposhnikoviae（RS）	MOL011740	divaricatol	Solute carrier family 12 member 1	Q13621	SLC12A1	0.013
Radix Saposhnikoviae（RS）	MOL011740	divaricatol	ATP-sensitive inward rectifier potassium channel 11	Q14654	KCNJ11	0.013
Radix Saposhnikoviae（RS）	MOL011740	divaricatol	Acetylcholine receptor subunit alpha	P02708	CHRNA1	0.015
Radix Saposhnikoviae（RS）	MOL011740	divaricatol	Cholinesterase	P06276	BCHE	0.015
Radix Saposhnikoviae（RS）	MOL011740	divaricatol	Acetylcholine receptor subunit gamma	P07510	CHRNG	0.015
Radix Saposhnikoviae（RS）	MOL011740	divaricatol	Acetylcholine receptor subunit beta	P11230	CHRNB1	0.015
Radix Saposhnikoviae（RS）	MOL011740	divaricatol	Neuronal acetylcholine receptor subunit beta-2	P17787	CHRNB2	0.015
Radix Saposhnikoviae（RS）	MOL011740	divaricatol	Neuronal acetylcholine receptor subunit alpha-5	P30532	CHRNA5	0.015
Radix Saposhnikoviae（RS）	MOL011740	divaricatol	Neuronal acetylcholine receptor subunit beta-4	P30926	CHRNB4	0.015
Radix Saposhnikoviae（RS）	MOL011740	divaricatol	Acetylcholine receptor subunit epsilon	Q04844	CHRNE	0.015
Radix Saposhnikoviae（RS）	MOL011740	divaricatol	Neuronal acetylcholine receptor subunit beta-3	Q05901	CHRNB3	0.015
Radix Saposhnikoviae（RS）	MOL011740	divaricatol	Acetylcholine receptor subunit delta	Q07001	CHRND	0.015
Radix Saposhnikoviae（RS）	MOL011740	divaricatol	Neuronal acetylcholine receptor subunit alpha-6	Q15825	KCNJ8	0.015
Radix Saposhnikoviae（RS）	MOL011740	divaricatol	Neuronal acetylcholine receptor subunit alpha-10	Q9GZZ6	CHRNA10	0.015
Radix Saposhnikoviae（RS）	MOL011740	divaricatol	Neuronal acetylcholine receptor subunit alpha-9	Q9UGM1	CHRNA9	0.015
Radix Saposhnikoviae（RS）	MOL011740	divaricatol	5-hydroxytryptamine 1E receptor	P28566	HTR1E	0.016
Radix Saposhnikoviae（RS）	MOL011740	divaricatol	5-hydroxytryptamine 7 receptor	P34969	HTR7	0.016
Radix Saposhnikoviae（RS）	MOL011740	divaricatol	Glutamate receptor 2	P42262	GRIA2	0.016
Radix Saposhnikoviae（RS）	MOL011740	divaricatol	5-hydroxytryptamine 6 receptor	P50406	HTR6	0.016
Radix Saposhnikoviae（RS）	MOL011740	divaricatol	D1 dopamine receptor-interacting protein calcyon	Q9NYX4	CALY	0.016

Radix Saposhnikoviae（RS）	MOL011740	divaricatol	Actin-related protein 2/3 complex subunit 1B	O15143	ARPC1B	0.019
Radix Saposhnikoviae（RS）	MOL011740	divaricatol	Actin-related protein 2/3 complex subunit 2	O15144	ARPC2	0.019
Radix Saposhnikoviae（RS）	MOL011740	divaricatol	Actin-related protein 2/3 complex subunit 3	O15145	ARPC3	0.019
Radix Saposhnikoviae（RS）	MOL011740	divaricatol	Actin-related protein 2/3 complex subunit 4	P59998	ARPC4	0.019
Radix Saposhnikoviae（RS）	MOL011740	divaricatol	Actin-related protein 3	P61158	ACTR3	0.019
Radix Saposhnikoviae（RS）	MOL011740	divaricatol	Actin-related protein 2	P61160	ACTR2	0.019
Radix Saposhnikoviae（RS）	MOL011740	divaricatol	Phospholipase A2	P04054	PLA2G1B	0.021
Radix Saposhnikoviae（RS）	MOL011740	divaricatol	Retinoic acid receptor alpha	P10276	RARA	0.021
Radix Saposhnikoviae（RS）	MOL011740	divaricatol	Retinoic acid receptor beta	P10826	RARB	0.021
Radix Saposhnikoviae（RS）	MOL011740	divaricatol	Retinoic acid receptor gamma-1	P13631	RARG	0.021
Radix Saposhnikoviae（RS）	MOL011740	divaricatol	Retinoic acid receptor RXR-beta	P28702	RXRB	0.021
Radix Saposhnikoviae（RS）	MOL011740	divaricatol	Retinoic acid receptor RXR-gamma	P48443	RXRG	0.021
Radix Saposhnikoviae（RS）	MOL011740	divaricatol	Opioid receptor, sigma 1	Q5T1J1	SIGMAR1	0.021
Radix Saposhnikoviae（RS）	MOL011740	divaricatol	Sigma 1-type opioid receptor	Q99720	SIGMAR1	0.021
Radix Saposhnikoviae（RS）	MOL011740	divaricatol	5-hydroxytryptamine 4 receptor	Q13639	HTR4	0.022
Radix Saposhnikoviae（RS）	MOL011740	divaricatol	Sodium channel protein type 1 subunit alpha	P35498	SCN1A	0.023
Radix Saposhnikoviae（RS）	MOL011740	divaricatol	Neuronal acetylcholine receptor subunit alpha-4	P43681	CHRNA4	0.024
Radix Saposhnikoviae（RS）	MOL011740	divaricatol	Potassium channel subfamily K member 1	O00180	KCNK1	0.025
Radix Saposhnikoviae（RS）	MOL011740	divaricatol	Carbonic anhydrase 4	P22748	CA4	0.027
Radix Saposhnikoviae（RS）	MOL011740	divaricatol	Sodium-dependent noradrenaline transporter	P23975	SLC6A2	0.027
Radix Saposhnikoviae（RS）	MOL011740	divaricatol	Sodium-dependent serotonin transporter	P31645	SLC6A4	0.027
Radix Saposhnikoviae（RS）	MOL011740	divaricatol	Alpha-7 nicotinic cholinergic receptor subunit	Q693P7	CHRNA7	0.027
Radix Saposhnikoviae（RS）	MOL011740	divaricatol	Elongation factor 2	P13639	EEF2	0.03
Radix Saposhnikoviae（RS）	MOL011740	divaricatol	Neuronal acetylcholine receptor subunit alpha-3	P32297	CHRNA3	0.03
Radix Saposhnikoviae（RS）	MOL011740	divaricatol	Nociceptin receptor	P41146	OPRL1	0.03
Radix Saposhnikoviae（RS）	MOL011740	divaricatol	Poly [ADP-ribose] polymerase 3	Q9Y6F1	PARP3	0.03
Radix Saposhnikoviae（RS）	MOL011740	divaricatol	Gamma-aminobutyric acid receptor subunit rho-3	A8MPY1	GABRR3	0.031
Radix Saposhnikoviae（RS）	MOL011740	divaricatol	Gamma-aminobutyric acid receptor subunit pi	O00591	GABRP	0.031
Radix Saposhnikoviae（RS）	MOL011740	divaricatol	Gamma-aminobutyric acid receptor subunit delta	O14764	GABRD	0.031
Radix Saposhnikoviae（RS）	MOL011740	divaricatol	Gamma-aminobutyric-acid receptor subunit beta-1	P18505	GABRB1	0.031
Radix Saposhnikoviae（RS）	MOL011740	divaricatol	Gamma-aminobutyric acid receptor subunit gamma-2	P18507	GABRG2	0.031
Radix Saposhnikoviae（RS）	MOL011740	divaricatol	Gamma-aminobutyric-acid receptor subunit rho-1	P24046	GABRR1	0.031
Radix Saposhnikoviae（RS）	MOL011740	divaricatol	Gamma-aminobutyric acid receptor subunit rho-2	P28476	GABRR2	0.031
Radix Saposhnikoviae（RS）	MOL011740	divaricatol	Gamma-aminobutyric-acid receptor subunit beta-2	P47870	GABRB2	0.031
Radix Saposhnikoviae（RS）	MOL011740	divaricatol	Gamma-aminobutyric acid receptor subunit epsilon	P78334	GABRE	0.031
Radix Saposhnikoviae（RS）	MOL011740	divaricatol	Gamma-aminobutyric acid receptor subunit gamma-1	Q8N1C3	GABRG1	0.031
Radix Saposhnikoviae（RS）	MOL011740	divaricatol	Gamma-aminobutyric acid receptor subunit gamma-3	Q99928	GABRG3	0.031
Radix Saposhnikoviae（RS）	MOL011740	divaricatol	Gamma-aminobutyric acid receptor subunit theta	Q9UN88	GABRQ	0.031
Radix Saposhnikoviae（RS）	MOL011740	divaricatol	Acetylcholinesterase	P22303	ACHE	0.033
Radix Saposhnikoviae（RS）	MOL011740	divaricatol	Serine/threonine-protein kinase 17B	O94768	STK17B	0.034
Radix Saposhnikoviae（RS）	MOL011740	divaricatol	Proto-oncogene serine/threonine-protein kinase Pim-1	P11309	PIM1	0.034
Radix Saposhnikoviae（RS）	MOL011740	divaricatol	ATP synthase subunit gamma, mitochondrial	P36542	ATP5F1C	0.034
Radix Saposhnikoviae（RS）	MOL011740	divaricatol	UDP-glucuronosyltransferase 3A1	Q6NUS8	UGT3A1	0.034
Radix Saposhnikoviae（RS）	MOL011740	divaricatol	Potassium voltage-gated channel subfamily H member 2	Q12809	KCNH2	0.035
Radix Saposhnikoviae（RS）	MOL011740	divaricatol	Potassium voltage-gated channel subfamily H member 6	Q9H252	KCNH6	0.035
Radix Saposhnikoviae（RS）	MOL011740	divaricatol	Phosphatidylinositol 3-kinase regulatory subunit alpha	P27986	PIK3R1	0.036
Radix Saposhnikoviae（RS）	MOL011740	divaricatol	5-hydroxytryptamine 1D receptor	P28221	HTR1D	0.036

Radix Saposhnikoviae（RS）	MOL011740	divaricatol	5-hydroxytryptamine 1B receptor	P28222	HTR1B	0.036
Radix Saposhnikoviae（RS）	MOL011740	divaricatol	5-hydroxytryptamine 2B receptor	P41595	HTR2B	0.036
Radix Saposhnikoviae（RS）	MOL011740	divaricatol	Serine/threonine-protein kinase PLK1	P53350	PLK1	0.036
Radix Saposhnikoviae（RS）	MOL011740	divaricatol	Amiloride-sensitive sodium channel subunit alpha	P37088	SCNN1A	0.039
Radix Saposhnikoviae（RS）	MOL011740	divaricatol	Amiloride-sensitive sodium channel subunit beta	P51168	SCNN1B	0.039
Radix Saposhnikoviae（RS）	MOL011740	divaricatol	Amiloride-sensitive sodium channel subunit gamma	P51170	SCNN1G	0.039
Radix Saposhnikoviae（RS）	MOL011740	divaricatol	Neuronal acetylcholine receptor subunit alpha-2	Q15822	CHRNA2	0.04
Radix Saposhnikoviae（RS）	MOL011740	divaricatol	5-hydroxytryptamine 3 receptor	P46098	HTR3A	0.042
Radix Saposhnikoviae（RS）	MOL011740	divaricatol	Gamma-aminobutyric-acid receptor subunit alpha-5	P31644	GABRA5	0.043
Radix Saposhnikoviae（RS）	MOL011740	divaricatol	Gamma-aminobutyric-acid receptor subunit alpha-3	P34903	GABRA3	0.043
Radix Saposhnikoviae（RS）	MOL011740	divaricatol	Neuronal acetylcholine receptor subunit alpha-7	P36544	CHRNA7	0.043
Radix Saposhnikoviae（RS）	MOL011740	divaricatol	Gamma-aminobutyric-acid receptor subunit alpha-4	P48169	GABRA4	0.043
Radix Saposhnikoviae（RS）	MOL011740	divaricatol	Gamma-aminobutyric-acid receptor subunit alpha-6	Q16445	GABRA6	0.043
Radix Saposhnikoviae（RS）	MOL011740	divaricatol	Dehydrogenase/reductase SDR family member 8	Q8NBQ5	HSD17B11	0.043
Radix Saposhnikoviae（RS）	MOL011740	divaricatol	Tyrosine-protein kinase JAK2	O60674	JAK2	0.049
Radix Saposhnikoviae（RS）	MOL011740	divaricatol	Tyrosine-protein kinase JAK1	P23458	JAK1	0.049
Radix Saposhnikoviae（RS）	MOL011740	divaricatol	Tyrosine-protein kinase JAK3	P52333	JAK3	0.049
Radix Saposhnikoviae（RS）	MOL011740	divaricatol	Prostaglandin reductase 1	Q14914	PTGR1	0.049
Radix Saposhnikoviae（RS）	MOL011740	divaricatol	Tubulin alpha-6 chain	Q9BQE3	TUBA1C	0.051
Radix Saposhnikoviae（RS）	MOL011740	divaricatol	Tubulin beta-2B chain	Q9BVA1	TUBB2B	0.051
Radix Saposhnikoviae（RS）	MOL011740	divaricatol	Stathmin-4	Q9H169	STMN4	0.051
Radix Saposhnikoviae（RS）	MOL011740	divaricatol	Tubulin beta-2C chain	P68371	TUBB4B	0.052
Radix Saposhnikoviae（RS）	MOL011740	divaricatol	Glutamate [NMDA] receptor subunit 3A	Q8TCU5	GRIN3A	0.052
Radix Saposhnikoviae（RS）	MOL011740	divaricatol	Alpha-1D adrenergic receptor	P25100	ADRA1D	0.055
Radix Saposhnikoviae（RS）	MOL011740	divaricatol	Muscarinic acetylcholine receptor M2	P08172	CHRM2	0.056
Radix Saposhnikoviae（RS）	MOL011740	divaricatol	Alpha-2A adrenergic receptor	P08913	ADRA2A	0.056
Radix Saposhnikoviae（RS）	MOL011740	divaricatol	Sodium channel protein type 5 subunit alpha	Q14524	SCN5A	0.056
Radix Saposhnikoviae（RS）	MOL011740	divaricatol	Gamma-aminobutyric-acid receptor subunit alpha-1	P14867	GABRA1	0.057
Radix Saposhnikoviae（RS）	MOL011740	divaricatol	Gamma-aminobutyric-acid receptor subunit alpha-2	P47869	GABRA2	0.057
Radix Saposhnikoviae（RS）	MOL011740	divaricatol	Glycogen synthase kinase-3 beta	P49841	GSK3B	0.059
Radix Saposhnikoviae（RS）	MOL011740	divaricatol	Cyclin-dependent kinase 5 activator 1	Q15078	CDK5R1	0.059
Radix Saposhnikoviae（RS）	MOL011740	divaricatol	Lipoic acid synthetase, mitochondrial	O43766	LIAS	0.061
Radix Saposhnikoviae（RS）	MOL011740	divaricatol	Lipoyltransferase 1, mitochondrial	Q9Y234	LIPT1	0.061
Radix Saposhnikoviae（RS）	MOL011740	divaricatol	Carbonic anhydrase 1	P00915	CA1	0.062
Radix Saposhnikoviae（RS）	MOL011740	divaricatol	Carbonic anhydrase 2	P00918	CA2	0.062
Radix Saposhnikoviae（RS）	MOL011740	divaricatol	Mitogen-activated protein kinase 8	P45983	MAPK8	0.062
Radix Saposhnikoviae（RS）	MOL011740	divaricatol	Mitogen-activated protein kinase 10	P53779	MAPK10	0.062
Radix Saposhnikoviae（RS）	MOL011740	divaricatol	Platelet glycoprotein IX	P14770	GP9	0.063
Radix Saposhnikoviae（RS）	MOL011740	divaricatol	Proto-oncogene tyrosine-protein kinase LCK	P06239	LCK	0.064
Radix Saposhnikoviae（RS）	MOL011740	divaricatol	Tyrosine-protein kinase Lyn	P07948	LYN	0.064
Radix Saposhnikoviae（RS）	MOL011740	divaricatol	Keratin, type II cytoskeletal 7	P08729	KRT7	0.064
Radix Saposhnikoviae（RS）	MOL011740	divaricatol	Alcohol dehydrogenase [NADP+]	P14550	AKR1A1	0.064
Radix Saposhnikoviae（RS）	MOL011740	divaricatol	Aldose reductase	P15121	AKR1B1	0.064
Radix Saposhnikoviae（RS）	MOL011740	divaricatol	Peptidyl-prolyl cis-trans isomerase, mitochondrial	P30405	PPIF	0.064
Radix Saposhnikoviae（RS）	MOL011740	divaricatol	85 kDa calcium-independent phospholipase A2	O60733	PLA2G6	0.065
Radix Saposhnikoviae（RS）	MOL011740	divaricatol	Tripartite motif-containing protein 13	O60858	TRIM13	0.065
Radix Saposhnikoviae（RS）	MOL011740	divaricatol	DNA topoisomerase 1	P11387	TOP1	0.065

Radix Saposhnikoviae（RS）	MOL011740	divaricatol	Cytosolic phospholipase A2	P47712	PLA2G4A	0.065
Radix Saposhnikoviae（RS）	MOL011740	divaricatol	DNA topoisomerase I, mitochondrial	Q969P6	TOP1MT	0.065
Radix Saposhnikoviae（RS）	MOL011740	divaricatol	Tryptophanyl-tRNA synthetase, mitochondrial	Q9UGM6	WARS2	0.065
Radix Saposhnikoviae（RS）	MOL011740	divaricatol	Guanine nucleotide-binding protein G(s) subunit alpha isoforms short	P63092	GNAS	0.067
Radix Saposhnikoviae（RS）	MOL011740	divaricatol	Adenylate cyclase type 2	Q08462	ADCY2	0.067
Radix Saposhnikoviae（RS）	MOL011740	divaricatol	Methionine aminopeptidase 1	P53582	METAP1	0.068
Radix Saposhnikoviae（RS）	MOL011740	divaricatol	Ribosyldihydronicotinamide dehydrogenase [quinone]	P16083	NQO2	0.07
Radix Saposhnikoviae（RS）	MOL011740	divaricatol	Tyrosyl-tRNA synthetase, cytoplasmic	P54577	YARS	0.07
Radix Saposhnikoviae（RS）	MOL011740	divaricatol	Casein kinase II subunit alpha	P68400	CSNK2A1	0.07
Radix Saposhnikoviae（RS）	MOL011740	divaricatol	Peptidyl-prolyl cis-trans isomerase NIMA-interacting 1	Q13526	PIN1	0.07
Radix Saposhnikoviae（RS）	MOL011740	divaricatol	ATP synthase subunit beta, mitochondrial	P06576	ATP5F1B	0.071
Radix Saposhnikoviae（RS）	MOL011740	divaricatol	Tyrosine-protein kinase HCK	P08631	HCK	0.071
Radix Saposhnikoviae（RS）	MOL011740	divaricatol	ATP synthase subunit alpha, mitochondrial	P25705	ATP5F1A	0.071
Radix Saposhnikoviae（RS）	MOL011740	divaricatol	3 beta-hydroxysteroid dehydrogenase/Delta 5-->4-isomerase type II	P26439	HSD3B2	0.072
Radix Saposhnikoviae（RS）	MOL011740	divaricatol	Peroxisome proliferator-activated receptor gamma	P37231	PPARG	0.072
Radix Saposhnikoviae（RS）	MOL011740	divaricatol	Group IIE secretory phospholipase A2	Q9NZK7	PLA2G2E	0.072
Radix Saposhnikoviae（RS）	MOL011740	divaricatol	Muscarinic acetylcholine receptor M4	P08173	CHRM4	0.076
Radix Saposhnikoviae（RS）	MOL011740	divaricatol	Muscarinic acetylcholine receptor M5	P08912	CHRM5	0.076
Radix Saposhnikoviae（RS）	MOL011740	divaricatol	Muscarinic acetylcholine receptor M1	P11229	CHRM1	0.076
Radix Saposhnikoviae（RS）	MOL011740	divaricatol	Alpha-2B adrenergic receptor	P18089	ADRA2B	0.076
Radix Saposhnikoviae（RS）	MOL011740	divaricatol	Alpha-2C adrenergic receptor	P18825	ADRA2C	0.076
Radix Saposhnikoviae（RS）	MOL011740	divaricatol	D(4) dopamine receptor	P21917	DRD4	0.076
Radix Saposhnikoviae（RS）	MOL011740	divaricatol	D(1B) dopamine receptor	P21918	DRD5	0.076
Radix Saposhnikoviae（RS）	MOL011740	divaricatol	Histamine H1 receptor	P35367	HRH1	0.076
Radix Saposhnikoviae（RS）	MOL011740	divaricatol	D(3) dopamine receptor	P35462	DRD3	0.076
Radix Saposhnikoviae（RS）	MOL011740	divaricatol	Prolactin receptor	P16471	PRLR	0.08
Radix Saposhnikoviae（RS）	MOL011740	divaricatol	Nitric-oxide synthase, brain	P29475	NOS1	0.082
Radix Saposhnikoviae（RS）	MOL011740	divaricatol	5-hydroxytryptamine 1A receptor	P08908	HTR1A	0.083
Radix Saposhnikoviae（RS）	MOL011740	divaricatol	DNA topoisomerase 2-alpha	P11388	TOP2A	0.084
Radix Saposhnikoviae（RS）	MOL011740	divaricatol	Protein tyrosine kinase 2 beta	Q14289	PTK2B	0.084
Radix Saposhnikoviae（RS）	MOL011740	divaricatol	Bile salt sulfotransferase	Q06520	SULT2A1	0.095
Radix Saposhnikoviae（RS）	MOL011740	divaricatol	Muscarinic acetylcholine receptor M3	P20309	CHRM3	0.096
Radix Saposhnikoviae（RS）	MOL011740	divaricatol	cAMP-dependent protein kinase inhibitor alpha	P61925	PKIA	0.099
Radix Saposhnikoviae（RS）	MOL011740	divaricatol	Death-associated protein kinase 3	O43293	DAPK3	0.101
Radix Saposhnikoviae（RS）	MOL011740	divaricatol	Phosphatidylinositol-4,5-bisphosphate 3-kinase catalytic subunit gamma isoform	P48736	PIK3CG	0.113
Radix Saposhnikoviae（RS）	MOL011740	divaricatol	Alpha-1B adrenergic receptor	P35368	ADRA1B	0.115
Radix Saposhnikoviae（RS）	MOL011740	divaricatol	5-hydroxytryptamine 2A receptor	P28223	HTR2A	0.116
Radix Saposhnikoviae（RS）	MOL011740	divaricatol	Nuclear receptor coactivator 2	Q15596	NCOA2	0.12
Radix Saposhnikoviae（RS）	MOL011740	divaricatol	Mediator of RNA polymerase II transcription subunit 1	Q15648	MED1	0.12
Radix Saposhnikoviae（RS）	MOL011740	divaricatol	L-amino-acid oxidase	Q96RQ9	IL4I1	0.12
Radix Saposhnikoviae（RS）	MOL011740	divaricatol	Nitric oxide synthase, inducible	P35228	NOS2	0.121
Radix Saposhnikoviae（RS）	MOL011740	divaricatol	D-HSCDK2	O75100	CA11	0.123
Radix Saposhnikoviae（RS）	MOL011740	divaricatol	NADPH oxidase organizer 1	Q8NFA2	NOXO1	0.126
Radix Saposhnikoviae（RS）	MOL011740	divaricatol	5-hydroxytryptamine 2C receptor	P28335	HTR2C	0.127
Radix Saposhnikoviae（RS）	MOL011740	divaricatol	Toll-like receptor 7	Q9NYK1	TLR7	0.128
Radix Saposhnikoviae（RS）	MOL011740	divaricatol	C-jun-amino-terminal kinase-interacting protein 1	Q9UQF2	MAPK8IP1	0.128
Radix Saposhnikoviae（RS）	MOL011740	divaricatol	Nuclear receptor coactivator 5	Q9HCD5	NCOA5	0.129

Radix Saposhnikoviae（RS）	MOL011740	divaricatol	Hydroxyacid oxidase 1	Q9UJM8	HAO1	0.13
Radix Saposhnikoviae（RS）	MOL011740	divaricatol	Endothelin-1 receptor	P25101	EDNRA	0.131
Radix Saposhnikoviae（RS）	MOL011740	divaricatol	Triosephosphate isomerase	P60174	TPI1	0.131
Radix Saposhnikoviae（RS）	MOL011740	divaricatol	Dihydrofolate reductase	P00374	DHFR	0.135
Radix Saposhnikoviae（RS）	MOL011740	divaricatol	3 beta-hydroxysteroid dehydrogenase/Delta 5-->4-isomerase type I	P14060	HSD3B1	0.135
Radix Saposhnikoviae（RS）	MOL011740	divaricatol	Low molecular weight phosphotyrosine protein phosphatase	P24666	ACP1	0.135
Radix Saposhnikoviae（RS）	MOL011740	divaricatol	Alpha-1A adrenergic receptor	P35348	ADRA1A	0.135
Radix Saposhnikoviae（RS）	MOL011740	divaricatol	D(2) dopamine receptor	P14416	DRD2	0.136
Radix Saposhnikoviae（RS）	MOL011740	divaricatol	Ig kappa chain C region	P01834	IGKC	0.137
Radix Saposhnikoviae（RS）	MOL011740	divaricatol	Inosine-5'-monophosphate dehydrogenase 1	P20839	IMPDH1	0.137
Radix Saposhnikoviae（RS）	MOL011740	divaricatol	D(1A) dopamine receptor	P21728	DRD1	0.137
Radix Saposhnikoviae（RS）	MOL011740	divaricatol	S-methyl-5-thioadenosine phosphorylase	Q13126	MTAP	0.142
Radix Saposhnikoviae（RS）	MOL011740	divaricatol	DNA polymerase kappa	Q9UBT6	POLK	0.142
Radix Saposhnikoviae（RS）	MOL011740	divaricatol	Inhibitor of nuclear factor kappa-B kinase subunit alpha	O15111	CHUK	0.147
Radix Saposhnikoviae（RS）	MOL011740	divaricatol	Arachidonate 5-lipoxygenase	P09917	ALOX5	0.147
Radix Saposhnikoviae（RS）	MOL011740	divaricatol	Cannabinoid receptor 2	P34972	CNR2	0.152
Radix Saposhnikoviae（RS）	MOL011740	divaricatol	MAP kinase-activated protein kinase 2	P49137	MAPKAPK2	0.152
Radix Saposhnikoviae（RS）	MOL011740	divaricatol	Delta-type opioid receptor	P41143	OPRD1	0.163
Radix Saposhnikoviae（RS）	MOL011740	divaricatol	Tubulin alpha-3 chain	Q71U36	TUBA1A	0.163
Radix Saposhnikoviae（RS）	MOL011740	divaricatol	Mineralocorticoid receptor	P08235	NR3C2	0.164
Radix Saposhnikoviae（RS）	MOL011740	divaricatol	Estradiol 17-beta-dehydrogenase 1	P14061	HSD17B1	0.166
Radix Saposhnikoviae（RS）	MOL011740	divaricatol	Nitric-oxide synthase, endothelial	P29474	NOS3	0.168
Radix Saposhnikoviae（RS）	MOL011740	divaricatol	Cannabinoid receptor 1	P21554	CNR1	0.174
Radix Saposhnikoviae（RS）	MOL011740	divaricatol	Cell division control protein 2 homolog	P06493	CDK1	0.191
Radix Saposhnikoviae（RS）	MOL011740	divaricatol	Kappa-type opioid receptor	P41145	OPRK1	0.197
Radix Saposhnikoviae（RS）	MOL011740	divaricatol	Androgen receptor	P10275	AR	0.249
Radix Saposhnikoviae（RS）	MOL011740	divaricatol	Cell division protein kinase 5	Q00535	CDK5	0.255
Radix Saposhnikoviae（RS）	MOL011740	divaricatol	Cyclin-A2	P20248	CCNA2	0.26
Radix Saposhnikoviae（RS）	MOL011740	divaricatol	Nuclear receptor coactivator 1	Q15788	NCOA1	0.264
Radix Saposhnikoviae（RS）	MOL011740	divaricatol	cAMP-dependent protein kinase catalytic subunit alpha	P17612	PRKACA	0.304
Radix Saposhnikoviae（RS）	MOL011740	divaricatol	Prothrombin	P00734	F2	0.306
Radix Saposhnikoviae（RS）	MOL011740	divaricatol	Mu-type opioid receptor	P35372	OPRM1	0.324
Radix Saposhnikoviae（RS）	MOL011740	divaricatol	Cell division protein kinase 2	P24941	CDK2	0.364
Radix Saposhnikoviae（RS）	MOL011740	divaricatol	Progesterone receptor	P06401	PGR	0.417
Radix Saposhnikoviae（RS）	MOL011740	divaricatol	Prostaglandin G/H synthase 1	P23219	PTGS1	0.447
Radix Saposhnikoviae（RS）	MOL011740	divaricatol	Hemoglobin subunit alpha	P69905	HBA1	0.53
Radix Saposhnikoviae（RS）	MOL011740	divaricatol	Trypsin-1	P07477	PRSS1	0.547
Radix Saposhnikoviae（RS）	MOL011740	divaricatol	Estrogen receptor beta	Q92731	ESR2	0.628
Radix Saposhnikoviae（RS）	MOL011740	divaricatol	Prostaglandin G/H synthase 2	P35354	PTGS2	0.831
Radix Saposhnikoviae（RS）	MOL011740	divaricatol	Estrogen receptor	P03372	ESR1	1
Radix Saposhnikoviae（RS）	MOL011747	ledebouriellol	Gamma-aminobutyric acid receptor subunit rho-3	A8MPY1	GABRR3	0.015
Radix Saposhnikoviae（RS）	MOL011747	ledebouriellol	Gamma-aminobutyric acid receptor subunit pi	O00591	GABRP	0.015
Radix Saposhnikoviae（RS）	MOL011747	ledebouriellol	Gamma-aminobutyric acid receptor subunit delta	O14764	GABRD	0.015
Radix Saposhnikoviae（RS）	MOL011747	ledebouriellol	Gamma-aminobutyric-acid receptor subunit beta-1	P18505	GABRB1	0.015
Radix Saposhnikoviae（RS）	MOL011747	ledebouriellol	Gamma-aminobutyric acid receptor subunit gamma-2	P18507	GABRG2	0.015
Radix Saposhnikoviae（RS）	MOL011747	ledebouriellol	Gamma-aminobutyric-acid receptor subunit rho-1	P24046	GABRR1	0.015
Radix Saposhnikoviae（RS）	MOL011747	ledebouriellol	Gamma-aminobutyric acid receptor subunit rho-2	P28476	GABRR2	0.015

Radix Saposhnikoviae（RS）	MOL011747	ledebouriellol	Gamma-aminobutyric-acid receptor subunit alpha-5	P31644	GABRA5	0.015
Radix Saposhnikoviae（RS）	MOL011747	ledebouriellol	Gamma-aminobutyric-acid receptor subunit beta-2	P47870	GABRB2	0.015
Radix Saposhnikoviae（RS）	MOL011747	ledebouriellol	Gamma-aminobutyric-acid receptor subunit alpha-4	P48169	GABRA4	0.015
Radix Saposhnikoviae（RS）	MOL011747	ledebouriellol	Gamma-aminobutyric acid receptor subunit epsilon	P78334	GABRE	0.015
Radix Saposhnikoviae（RS）	MOL011747	ledebouriellol	Gamma-aminobutyric-acid receptor subunit alpha-6	Q16445	GABRA6	0.015
Radix Saposhnikoviae（RS）	MOL011747	ledebouriellol	Gamma-aminobutyric acid receptor subunit gamma-1	Q8N1C3	GABRG1	0.015
Radix Saposhnikoviae（RS）	MOL011747	ledebouriellol	Gamma-aminobutyric acid receptor subunit gamma-3	Q99928	GABRG3	0.015
Radix Saposhnikoviae（RS）	MOL011747	ledebouriellol	Gamma-aminobutyric acid receptor subunit theta	Q9UN88	GABRQ	0.015
Radix Saposhnikoviae（RS）	MOL011747	ledebouriellol	Acetylcholine receptor subunit alpha	P02708	CHRNA1	0.023
Radix Saposhnikoviae（RS）	MOL011747	ledebouriellol	Cholinesterase	P06276	BCHE	0.023
Radix Saposhnikoviae（RS）	MOL011747	ledebouriellol	Acetylcholine receptor subunit gamma	P07510	CHRNG	0.023
Radix Saposhnikoviae（RS）	MOL011747	ledebouriellol	Acetylcholine receptor subunit beta	P11230	CHRNB1	0.023
Radix Saposhnikoviae（RS）	MOL011747	ledebouriellol	Neuronal acetylcholine receptor subunit beta-2	P17787	CHRNB2	0.023
Radix Saposhnikoviae（RS）	MOL011747	ledebouriellol	Neuronal acetylcholine receptor subunit alpha-5	P30532	CHRNA5	0.023
Radix Saposhnikoviae（RS）	MOL011747	ledebouriellol	Neuronal acetylcholine receptor subunit beta-4	P30926	CHRNB4	0.023
Radix Saposhnikoviae（RS）	MOL011747	ledebouriellol	Acetylcholine receptor subunit epsilon	Q04844	CHRNE	0.023
Radix Saposhnikoviae（RS）	MOL011747	ledebouriellol	Neuronal acetylcholine receptor subunit beta-3	Q05901	CHRNB3	0.023
Radix Saposhnikoviae（RS）	MOL011747	ledebouriellol	Acetylcholine receptor subunit delta	Q07001	CHRND	0.023
Radix Saposhnikoviae（RS）	MOL011747	ledebouriellol	Neuronal acetylcholine receptor subunit alpha-2	Q15822	CHRNA2	0.023
Radix Saposhnikoviae（RS）	MOL011747	ledebouriellol	Neuronal acetylcholine receptor subunit alpha-6	Q15825	KCNJ8	0.023
Radix Saposhnikoviae（RS）	MOL011747	ledebouriellol	Neuronal acetylcholine receptor subunit alpha-10	Q9GZZ6	CHRNA10	0.023
Radix Saposhnikoviae（RS）	MOL011747	ledebouriellol	Neuronal acetylcholine receptor subunit alpha-9	Q9UGM1	CHRNA9	0.023
Radix Saposhnikoviae（RS）	MOL011747	ledebouriellol	Neuronal acetylcholine receptor subunit alpha-4	P43681	CHRNA4	0.024
Radix Saposhnikoviae（RS）	MOL011747	ledebouriellol	Alpha-2A adrenergic receptor	P08913	ADRA2A	0.028
Radix Saposhnikoviae（RS）	MOL011747	ledebouriellol	Alpha-2B adrenergic receptor	P18089	ADRA2B	0.028
Radix Saposhnikoviae（RS）	MOL011747	ledebouriellol	Alpha-2C adrenergic receptor	P18825	ADRA2C	0.028
Radix Saposhnikoviae（RS）	MOL011747	ledebouriellol	D(1B) dopamine receptor	P21918	DRD5	0.028
Radix Saposhnikoviae（RS）	MOL011747	ledebouriellol	5-hydroxytryptamine 1B receptor	P28222	HTR1B	0.028
Radix Saposhnikoviae（RS）	MOL011747	ledebouriellol	D(3) dopamine receptor	P35462	DRD3	0.028
Radix Saposhnikoviae（RS）	MOL011747	ledebouriellol	5-hydroxytryptamine 2B receptor	P41595	HTR2B	0.028
Radix Saposhnikoviae（RS）	MOL011747	ledebouriellol	D1 dopamine receptor-interacting protein calcyon	Q9NYX4	CALY	0.028
Radix Saposhnikoviae（RS）	MOL011747	ledebouriellol	Muscarinic acetylcholine receptor M5	P08912	CHRM5	0.029
Radix Saposhnikoviae（RS）	MOL011747	ledebouriellol	Histamine H1 receptor	P35367	HRH1	0.029
Radix Saposhnikoviae（RS）	MOL011747	ledebouriellol	Gamma-aminobutyric-acid receptor subunit alpha-1	P14867	GABRA1	0.032
Radix Saposhnikoviae（RS）	MOL011747	ledebouriellol	Gamma-aminobutyric-acid receptor subunit alpha-3	P34903	GABRA3	0.032
Radix Saposhnikoviae（RS）	MOL011747	ledebouriellol	Gamma-aminobutyric-acid receptor subunit alpha-2	P47869	GABRA2	0.032
Radix Saposhnikoviae（RS）	MOL011747	ledebouriellol	Death-associated protein kinase 3	O43293	DAPK3	0.041
Radix Saposhnikoviae（RS）	MOL011747	ledebouriellol	Tyrosine-protein kinase JAK2	O60674	JAK2	0.041
Radix Saposhnikoviae（RS）	MOL011747	ledebouriellol	Tyrosine-protein kinase JAK1	P23458	JAK1	0.041
Radix Saposhnikoviae（RS）	MOL011747	ledebouriellol	Tyrosine-protein kinase JAK3	P52333	JAK3	0.041
Radix Saposhnikoviae（RS）	MOL011747	ledebouriellol	Elongation factor 2	P13639	EEF2	0.042
Radix Saposhnikoviae（RS）	MOL011747	ledebouriellol	Poly [ADP-ribose] polymerase 3	Q9Y6F1	PARP3	0.042
Radix Saposhnikoviae（RS）	MOL011747	ledebouriellol	Tripartite motif-containing protein 13	O60858	TRIM13	0.048
Radix Saposhnikoviae（RS）	MOL011747	ledebouriellol	Delta-type opioid receptor	P41143	OPRD1	0.048
Radix Saposhnikoviae（RS）	MOL011747	ledebouriellol	Nociceptin receptor	P41146	OPRL1	0.048
Radix Saposhnikoviae（RS）	MOL011747	ledebouriellol	Acetylcholinesterase	P22303	ACHE	0.049
Radix Saposhnikoviae（RS）	MOL011747	ledebouriellol	Neuronal acetylcholine receptor subunit alpha-3	P32297	CHRNA3	0.049

Radix Saposhnikoviae（RS）	MOL011747	ledebouriellol	Neuronal acetylcholine receptor subunit alpha-7	P36544	CHRNA7	0.049
Radix Saposhnikoviae（RS）	MOL011747	ledebouriellol	Potassium channel subfamily K member 1	O00180	KCNK1	0.058
Radix Saposhnikoviae（RS）	MOL011747	ledebouriellol	Amiloride-sensitive sodium channel subunit alpha	P37088	SCNN1A	0.058
Radix Saposhnikoviae（RS）	MOL011747	ledebouriellol	Amiloride-sensitive sodium channel subunit beta	P51168	SCNN1B	0.058
Radix Saposhnikoviae（RS）	MOL011747	ledebouriellol	Amiloride-sensitive sodium channel subunit gamma	P51170	SCNN1G	0.058
Radix Saposhnikoviae（RS）	MOL011747	ledebouriellol	Sodium channel protein type 5 subunit alpha	Q14524	SCN5A	0.058
Radix Saposhnikoviae（RS）	MOL011747	ledebouriellol	Potassium channel subfamily K member 6	Q9Y257	KCNK6	0.058
Radix Saposhnikoviae（RS）	MOL011747	ledebouriellol	5-hydroxytryptamine 1A receptor	P08908	HTR1A	0.059
Radix Saposhnikoviae（RS）	MOL011747	ledebouriellol	D(4) dopamine receptor	P21917	DRD4	0.059
Radix Saposhnikoviae（RS）	MOL011747	ledebouriellol	5-hydroxytryptamine 1D receptor	P28221	HTR1D	0.059
Radix Saposhnikoviae（RS）	MOL011747	ledebouriellol	5-hydroxytryptamine 2C receptor	P28335	HTR2C	0.059
Radix Saposhnikoviae（RS）	MOL011747	ledebouriellol	Mitogen-activated protein kinase 8	P45983	MAPK8	0.059
Radix Saposhnikoviae（RS）	MOL011747	ledebouriellol	Mitogen-activated protein kinase 10	P53779	MAPK10	0.059
Radix Saposhnikoviae（RS）	MOL011747	ledebouriellol	C-jun-amino-terminal kinase-interacting protein 1	Q9UQF2	MAPK8IP1	0.059
Radix Saposhnikoviae（RS）	MOL011747	ledebouriellol	Muscarinic acetylcholine receptor M2	P08172	CHRM2	0.06
Radix Saposhnikoviae（RS）	MOL011747	ledebouriellol	Muscarinic acetylcholine receptor M4	P08173	CHRM4	0.06
Radix Saposhnikoviae（RS）	MOL011747	ledebouriellol	Muscarinic acetylcholine receptor M1	P11229	CHRM1	0.06
Radix Saposhnikoviae（RS）	MOL011747	ledebouriellol	Alpha-1D adrenergic receptor	P25100	ADRA1D	0.06
Radix Saposhnikoviae（RS）	MOL011747	ledebouriellol	5-hydroxytryptamine 2A receptor	P28223	HTR2A	0.06
Radix Saposhnikoviae（RS）	MOL011747	ledebouriellol	Potassium voltage-gated channel subfamily H member 2	Q12809	KCNH2	0.06
Radix Saposhnikoviae（RS）	MOL011747	ledebouriellol	Potassium voltage-gated channel subfamily H member 6	Q9H252	KCNH6	0.06
Radix Saposhnikoviae（RS）	MOL011747	ledebouriellol	Ig kappa chain C region	P01834	IGKC	0.062
Radix Saposhnikoviae（RS）	MOL011747	ledebouriellol	Ig gamma-1 chain C region	P01857	IGHG1	0.062
Radix Saposhnikoviae（RS）	MOL011747	ledebouriellol	Ig gamma-2 chain C region	P01859	IGHG2	0.062
Radix Saposhnikoviae（RS）	MOL011747	ledebouriellol	Peptidyl-prolyl cis-trans isomerase, mitochondrial	P30405	PPIF	0.086
Radix Saposhnikoviae（RS）	MOL011747	ledebouriellol	Proto-oncogene tyrosine-protein kinase LCK	P06239	LCK	0.088
Radix Saposhnikoviae（RS）	MOL011747	ledebouriellol	Tyrosine-protein kinase Lyn	P07948	LYN	0.088
Radix Saposhnikoviae（RS）	MOL011747	ledebouriellol	Keratin, type II cytoskeletal 7	P08729	KRT7	0.088
Radix Saposhnikoviae（RS）	MOL011747	ledebouriellol	Alcohol dehydrogenase [NADP+]	P14550	AKR1A1	0.088
Radix Saposhnikoviae（RS）	MOL011747	ledebouriellol	Platelet glycoprotein IX	P14770	GP9	0.088
Radix Saposhnikoviae（RS）	MOL011747	ledebouriellol	Aldose reductase	P15121	AKR1B1	0.088
Radix Saposhnikoviae（RS）	MOL011747	ledebouriellol	Peroxisome proliferator-activated receptor gamma	P37231	PPARG	0.088
Radix Saposhnikoviae（RS）	MOL011747	ledebouriellol	Nuclear receptor coactivator 2	Q15596	NCOA2	0.088
Radix Saposhnikoviae（RS）	MOL011747	ledebouriellol	D(2) dopamine receptor	P14416	DRD2	0.09
Radix Saposhnikoviae（RS）	MOL011747	ledebouriellol	D(1A) dopamine receptor	P21728	DRD1	0.09
Radix Saposhnikoviae（RS）	MOL011747	ledebouriellol	Muscarinic acetylcholine receptor M3	P20309	CHRM3	0.092
Radix Saposhnikoviae（RS）	MOL011747	ledebouriellol	Alpha-1B adrenergic receptor	P35368	ADRA1B	0.092
Radix Saposhnikoviae（RS）	MOL011747	ledebouriellol	D-HSCDK2	O75100	CA11	0.093
Radix Saposhnikoviae（RS）	MOL011747	ledebouriellol	Carbonic anhydrase 2	P00918	CA2	0.093
Radix Saposhnikoviae（RS）	MOL011747	ledebouriellol	Cell division control protein 2 homolog	P06493	CDK1	0.093
Radix Saposhnikoviae（RS）	MOL011747	ledebouriellol	Carbonic anhydrase 4	P22748	CA4	0.093
Radix Saposhnikoviae（RS）	MOL011747	ledebouriellol	Methionine aminopeptidase 1	P53582	METAP1	0.093
Radix Saposhnikoviae（RS）	MOL011747	ledebouriellol	Tyrosyl-tRNA synthetase, cytoplasmic	P54577	YARS	0.095
Radix Saposhnikoviae（RS）	MOL011747	ledebouriellol	Peptidyl-prolyl cis-trans isomerase NIMA-interacting 1	Q13526	PIN1	0.095
Radix Saposhnikoviae（RS）	MOL011747	ledebouriellol	Mu-type opioid receptor	P35372	OPRM1	0.098
Radix Saposhnikoviae（RS）	MOL011747	ledebouriellol	Kappa-type opioid receptor	P41145	OPRK1	0.098
Radix Saposhnikoviae（RS）	MOL011747	ledebouriellol	Alpha-1A adrenergic receptor	P35348	ADRA1A	0.123

Radix Saposhnikoviae（RS）	MOL011747	ledebouriellol	3-hydroxy-3-methylglutaryl-coenzyme A reductase	P04035	HMGCR	0.171
Radix Saposhnikoviae（RS）	MOL011747	ledebouriellol	NADPH oxidase organizer 1	Q8NFA2	NOXO1	0.174
Radix Saposhnikoviae（RS）	MOL011747	ledebouriellol	Nuclear receptor coactivator 5	Q9HCD5	NCOA5	0.174
Radix Saposhnikoviae（RS）	MOL011747	ledebouriellol	Toll-like receptor 7	Q9NYK1	TLR7	0.174
Radix Saposhnikoviae（RS）	MOL011747	ledebouriellol	Cyclin-A2	P20248	CCNA2	0.175
Radix Saposhnikoviae（RS）	MOL011747	ledebouriellol	cAMP-specific 3',5'-cyclic phosphodiesterase 4B	Q07343	PDE4B	0.177
Radix Saposhnikoviae（RS）	MOL011747	ledebouriellol	Tyrosine-protein kinase HCK	P08631	HCK	0.178
Radix Saposhnikoviae（RS）	MOL011747	ledebouriellol	Proto-oncogene tyrosine-protein kinase Src	P12931	SRC	0.178
Radix Saposhnikoviae（RS）	MOL011747	ledebouriellol	Estradiol 17-beta-dehydrogenase 1	P14061	HSD17B1	0.178
Radix Saposhnikoviae（RS）	MOL011747	ledebouriellol	Aldo-keto reductase family 1 member C1	Q04828	AKR1C1	0.179
Radix Saposhnikoviae（RS）	MOL011747	ledebouriellol	Prothrombin	P00734	F2	0.183
Radix Saposhnikoviae（RS）	MOL011747	ledebouriellol	Nitric-oxide synthase, endothelial	P29474	NOS3	0.183
Radix Saposhnikoviae（RS）	MOL011747	ledebouriellol	Cell division protein kinase 5	Q00535	CDK5	0.188
Radix Saposhnikoviae（RS）	MOL011747	ledebouriellol	Dihydrofolate reductase	P00374	DHFR	0.189
Radix Saposhnikoviae（RS）	MOL011747	ledebouriellol	Carbonic anhydrase 1	P00915	CA1	0.189
Radix Saposhnikoviae（RS）	MOL011747	ledebouriellol	RAC-alpha serine/threonine-protein kinase	P31749	AKT1	0.189
Radix Saposhnikoviae（RS）	MOL011747	ledebouriellol	DNA polymerase kappa	Q9UBT6	POLK	0.194
Radix Saposhnikoviae（RS）	MOL011747	ledebouriellol	cAMP-dependent protein kinase catalytic subunit alpha	P17612	PRKACA	0.195
Radix Saposhnikoviae（RS）	MOL011747	ledebouriellol	MAP kinase-activated protein kinase 2	P49137	MAPKAPK2	0.202
Radix Saposhnikoviae（RS）	MOL011747	ledebouriellol	Peroxisome proliferator-activated receptor alpha	Q07869	PPARA	0.204
Radix Saposhnikoviae（RS）	MOL011747	ledebouriellol	Cannabinoid receptor 2	P34972	CNR2	0.207
Radix Saposhnikoviae（RS）	MOL011747	ledebouriellol	Cannabinoid receptor 1	P21554	CNR1	0.235
Radix Saposhnikoviae（RS）	MOL011747	ledebouriellol	Mineralocorticoid receptor	P08235	NR3C2	0.275
Radix Saposhnikoviae（RS）	MOL011747	ledebouriellol	Trypsin-1	P07477	PRSS1	0.354
Radix Saposhnikoviae（RS）	MOL011747	ledebouriellol	Estrogen receptor beta	Q92731	ESR2	0.367
Radix Saposhnikoviae（RS）	MOL011747	ledebouriellol	Nuclear receptor coactivator 1	Q15788	NCOA1	0.373
Radix Saposhnikoviae（RS）	MOL011747	ledebouriellol	Hemoglobin subunit alpha	P69905	HBA1	0.532
Radix Saposhnikoviae（RS）	MOL011747	ledebouriellol	Progesterone receptor	P06401	PGR	0.549
Radix Saposhnikoviae（RS）	MOL011747	ledebouriellol	Prostaglandin G/H synthase 2	P35354	PTGS2	0.749
Radix Saposhnikoviae（RS）	MOL011747	ledebouriellol	Estrogen receptor	P03372	ESR1	1
Radix Saposhnikoviae（RS）	MOL011749	phelloptorin	Acetylcholine receptor subunit alpha	P02708	CHRNA1	0.012
Radix Saposhnikoviae（RS）	MOL011749	phelloptorin	Cholinesterase	P06276	BCHE	0.012
Radix Saposhnikoviae（RS）	MOL011749	phelloptorin	Acetylcholine receptor subunit gamma	P07510	CHRNG	0.012
Radix Saposhnikoviae（RS）	MOL011749	phelloptorin	Acetylcholine receptor subunit beta	P11230	CHRNB1	0.012
Radix Saposhnikoviae（RS）	MOL011749	phelloptorin	Neuronal acetylcholine receptor subunit beta-2	P17787	CHRNB2	0.012
Radix Saposhnikoviae（RS）	MOL011749	phelloptorin	Neuronal acetylcholine receptor subunit alpha-5	P30532	CHRNA5	0.012
Radix Saposhnikoviae（RS）	MOL011749	phelloptorin	Neuronal acetylcholine receptor subunit beta-4	P30926	CHRNB4	0.012
Radix Saposhnikoviae（RS）	MOL011749	phelloptorin	Neuronal acetylcholine receptor subunit alpha-4	P43681	CHRNA4	0.012
Radix Saposhnikoviae（RS）	MOL011749	phelloptorin	Acetylcholine receptor subunit epsilon	Q04844	CHRNE	0.012
Radix Saposhnikoviae（RS）	MOL011749	phelloptorin	Neuronal acetylcholine receptor subunit beta-3	Q05901	CHRNB3	0.012
Radix Saposhnikoviae（RS）	MOL011749	phelloptorin	Acetylcholine receptor subunit delta	Q07001	CHRND	0.012
Radix Saposhnikoviae（RS）	MOL011749	phelloptorin	Neuronal acetylcholine receptor subunit alpha-6	Q15825	KCNJ8	0.012
Radix Saposhnikoviae（RS）	MOL011749	phelloptorin	Neuronal acetylcholine receptor subunit alpha-10	Q9GZZ6	CHRNA10	0.012
Radix Saposhnikoviae（RS）	MOL011749	phelloptorin	Neuronal acetylcholine receptor subunit alpha-9	Q9UGM1	CHRNA9	0.012
Radix Saposhnikoviae（RS）	MOL011749	phelloptorin	D1 dopamine receptor-interacting protein calcyon	Q9NYX4	CALY	0.017
Radix Saposhnikoviae（RS）	MOL011749	phelloptorin	5-hydroxytryptamine 1E receptor	P28566	HTR1E	0.018
Radix Saposhnikoviae（RS）	MOL011749	phelloptorin	5-hydroxytryptamine 7 receptor	P34969	HTR7	0.018

Radix Saposhnikoviae（RS）	MOL011749	phelloptorin	Purine nucleoside phosphorylase	P00491	PNP	0.019
Radix Saposhnikoviae（RS）	MOL011749	phelloptorin	DNA polymerase alpha catalytic subunit	P09884	POLA1	0.019
Radix Saposhnikoviae（RS）	MOL011749	phelloptorin	Elongation factor 2	P13639	EEF2	0.019
Radix Saposhnikoviae（RS）	MOL011749	phelloptorin	Ribonucleoside-diphosphate reductase large subunit	P23921	RRM1	0.019
Radix Saposhnikoviae（RS）	MOL011749	phelloptorin	Ribonucleoside-diphosphate reductase M2 subunit	P31350	RRM2	0.019
Radix Saposhnikoviae（RS）	MOL011749	phelloptorin	DNA polymerase epsilon subunit 2	P56282	POLE2	0.019
Radix Saposhnikoviae（RS）	MOL011749	phelloptorin	DNA polymerase epsilon catalytic subunit A	Q07864	POLE	0.019
Radix Saposhnikoviae（RS）	MOL011749	phelloptorin	Opioid receptor, sigma 1	Q5T1J1	SIGMAR1	0.019
Radix Saposhnikoviae（RS）	MOL011749	phelloptorin	Ribonucleoside-diphosphate reductase subunit M2 B	Q7LG56	RRM2B	0.019
Radix Saposhnikoviae（RS）	MOL011749	phelloptorin	Sigma 1-type opioid receptor	Q99720	SIGMAR1	0.019
Radix Saposhnikoviae（RS）	MOL011749	phelloptorin	DNA polymerase epsilon subunit 3	Q9NRF9	POLE3	0.019
Radix Saposhnikoviae（RS）	MOL011749	phelloptorin	Poly [ADP-ribose] polymerase 3	Q9Y6F1	PARP3	0.019
Radix Saposhnikoviae（RS）	MOL011749	phelloptorin	Beta-3 adrenergic receptor	P13945	ADRB3	0.022
Radix Saposhnikoviae（RS）	MOL011749	phelloptorin	Coagulation factor IX	P00740	F9	0.023
Radix Saposhnikoviae（RS）	MOL011749	phelloptorin	Coagulation factor X	P00742	F10	0.023
Radix Saposhnikoviae（RS）	MOL011749	phelloptorin	Osteocalcin	P02818	BGLAP	0.023
Radix Saposhnikoviae（RS）	MOL011749	phelloptorin	Vitamin K-dependent protein C	P04070	PROC	0.023
Radix Saposhnikoviae（RS）	MOL011749	phelloptorin	Coagulation factor VII	P08709	F7	0.023
Radix Saposhnikoviae（RS）	MOL011749	phelloptorin	NAD(P)H dehydrogenase [quinone] 1	P15559	NQO1	0.023
Radix Saposhnikoviae（RS）	MOL011749	phelloptorin	Vitamin K-dependent protein Z	P22891	PROZ	0.023
Radix Saposhnikoviae（RS）	MOL011749	phelloptorin	Vitamin K-dependent gamma-carboxylase	P38435	GGCX	0.023
Radix Saposhnikoviae（RS）	MOL011749	phelloptorin	Vitamin K epoxide reductase complex subunit 1-like protein 1	Q8N0U8	VKORC1L1	0.023
Radix Saposhnikoviae（RS）	MOL011749	phelloptorin	Vitamin K epoxide reductase complex subunit 1	Q9BQB6	VKORC1	0.023
Radix Saposhnikoviae（RS）	MOL011749	phelloptorin	85 kDa calcium-independent phospholipase A2	O60733	PLA2G6	0.024
Radix Saposhnikoviae（RS）	MOL011749	phelloptorin	DNA topoisomerase 2-alpha	P11388	TOP2A	0.024
Radix Saposhnikoviae（RS）	MOL011749	phelloptorin	Cytosolic phospholipase A2	P47712	PLA2G4A	0.024
Radix Saposhnikoviae（RS）	MOL011749	phelloptorin	5-hydroxytryptamine 4 receptor	Q13639	HTR4	0.024
Radix Saposhnikoviae（RS）	MOL011749	phelloptorin	Glutamate [NMDA] receptor subunit epsilon-4	O15399	GRIN2D	0.025
Radix Saposhnikoviae（RS）	MOL011749	phelloptorin	Glutamate [NMDA] receptor subunit 3B	O60391	GRIN3B	0.025
Radix Saposhnikoviae（RS）	MOL011749	phelloptorin	Solute carrier family 12 member 5	Q9H2X9	SLC12A5	0.025
Radix Saposhnikoviae（RS）	MOL011749	phelloptorin	Solute carrier family 12 member 4	Q9UP95	SLC12A4	0.025
Radix Saposhnikoviae（RS）	MOL011749	phelloptorin	Potassium channel subfamily K member 6	Q9Y257	KCNK6	0.025
Radix Saposhnikoviae（RS）	MOL011749	phelloptorin	Sodium/potassium-transporting ATPase alpha-1 chain	P05023	ATP1A1	0.026
Radix Saposhnikoviae（RS）	MOL011749	phelloptorin	Calcium-activated potassium channel subunit alpha 1	Q12791	KCNMA1	0.026
Radix Saposhnikoviae（RS）	MOL011749	phelloptorin	ATP-sensitive inward rectifier potassium channel 11	Q14654	KCNJ11	0.026
Radix Saposhnikoviae（RS）	MOL011749	phelloptorin	Death-associated protein kinase 3	O43293	DAPK3	0.027
Radix Saposhnikoviae（RS）	MOL011749	phelloptorin	Tyrosine-protein kinase JAK2	O60674	JAK2	0.027
Radix Saposhnikoviae（RS）	MOL011749	phelloptorin	cGMP-specific 3',5'-cyclic phosphodiesterase	O76074	PDE5A	0.027
Radix Saposhnikoviae（RS）	MOL011749	phelloptorin	Adenosine deaminase	P00813	ADA	0.027
Radix Saposhnikoviae（RS）	MOL011749	phelloptorin	Tyrosine-protein kinase JAK1	P23458	JAK1	0.027
Radix Saposhnikoviae（RS）	MOL011749	phelloptorin	Amiloride-sensitive sodium channel subunit alpha	P37088	SCNN1A	0.027
Radix Saposhnikoviae（RS）	MOL011749	phelloptorin	Amiloride-sensitive sodium channel subunit beta	P51168	SCNN1B	0.027
Radix Saposhnikoviae（RS）	MOL011749	phelloptorin	Amiloride-sensitive sodium channel subunit gamma	P51170	SCNN1G	0.027
Radix Saposhnikoviae（RS）	MOL011749	phelloptorin	Tyrosine-protein kinase JAK3	P52333	JAK3	0.027
Radix Saposhnikoviae（RS）	MOL011749	phelloptorin	cAMP and cAMP-inhibited cGMP 3',5'-cyclic phosphodiesterase 10A	Q9Y233	PDE10A	0.027
Radix Saposhnikoviae（RS）	MOL011749	phelloptorin	Pyridoxal kinase	O00764	PDXK	0.028
Radix Saposhnikoviae（RS）	MOL011749	phelloptorin	Arachidonate 5-lipoxygenase	P09917	ALOX5	0.028

Radix Saposhnikoviae（RS）	MOL011749	phelloptorin	Cystathionine beta-synthase	P35520	CBS	0.028
Radix Saposhnikoviae（RS）	MOL011749	phelloptorin	Aryl hydrocarbon receptor	P35869	AHR	0.028
Radix Saposhnikoviae（RS）	MOL011749	phelloptorin	Dihydroorotate dehydrogenase, mitochondrial	Q02127	DHODH	0.028
Radix Saposhnikoviae（RS）	MOL011749	phelloptorin	Glutamate [NMDA] receptor subunit zeta-1	Q05586	GRIN1	0.028
Radix Saposhnikoviae（RS）	MOL011749	phelloptorin	Pyridoxal phosphate phosphatase	Q96GD0	PDXP	0.028
Radix Saposhnikoviae（RS）	MOL011749	phelloptorin	Transient receptor potential cation channel subfamily A member 1	O75762	TRPA1	0.029
Radix Saposhnikoviae（RS）	MOL011749	phelloptorin	Transient receptor potential cation channel subfamily M member 8	Q7Z2W7	TRPM8	0.029
Radix Saposhnikoviae（RS）	MOL011749	phelloptorin	Transient receptor potential cation channel subfamily V member 3	Q8NET8	TRPV3	0.029
Radix Saposhnikoviae（RS）	MOL011749	phelloptorin	Sodium channel protein type 10 subunit alpha	Q9Y5Y9	SCN10A	0.029
Radix Saposhnikoviae（RS）	MOL011749	phelloptorin	D-HSCDK2	O75100	CA11	0.03
Radix Saposhnikoviae（RS）	MOL011749	phelloptorin	Cell division control protein 2 homolog	P06493	CDK1	0.03
Radix Saposhnikoviae（RS）	MOL011749	phelloptorin	Cell division protein kinase 5	Q00535	CDK5	0.03
Radix Saposhnikoviae（RS）	MOL011749	phelloptorin	Acetylcholinesterase	P22303	ACHE	0.032
Radix Saposhnikoviae（RS）	MOL011749	phelloptorin	Neuronal acetylcholine receptor subunit alpha-3	P32297	CHRNA3	0.032
Radix Saposhnikoviae（RS）	MOL011749	phelloptorin	Neuronal acetylcholine receptor subunit alpha-7	P36544	CHRNA7	0.032
Radix Saposhnikoviae（RS）	MOL011749	phelloptorin	Tripartite motif-containing protein 13	O60858	TRIM13	0.033
Radix Saposhnikoviae（RS）	MOL011749	phelloptorin	Prostaglandin reductase 1	Q14914	PTGR1	0.034
Radix Saposhnikoviae（RS）	MOL011749	phelloptorin	T-cell receptor alpha chain C region	P01848	TRAC	0.035
Radix Saposhnikoviae（RS）	MOL011749	phelloptorin	T-cell receptor beta chain C region	P01850	TRBC1	0.035
Radix Saposhnikoviae（RS）	MOL011749	phelloptorin	Beta-2-microglobulin	P61769	B2M	0.035
Radix Saposhnikoviae（RS）	MOL011749	phelloptorin	Neuronal acetylcholine receptor subunit alpha-2	Q15822	CHRNA2	0.038
Radix Saposhnikoviae（RS）	MOL011749	phelloptorin	3-phosphoinositide-dependent protein kinase 1	O15530	PDPK1	0.039
Radix Saposhnikoviae（RS）	MOL011749	phelloptorin	Proto-oncogene tyrosine-protein kinase LCK	P06239	LCK	0.039
Radix Saposhnikoviae（RS）	MOL011749	phelloptorin	Proto-oncogene serine/threonine-protein kinase Pim-1	P11309	PIM1	0.039
Radix Saposhnikoviae（RS）	MOL011749	phelloptorin	Tyrosine-protein kinase CSK	P41240	CSK	0.039
Radix Saposhnikoviae（RS）	MOL011749	phelloptorin	Tyrosine-protein kinase ZAP-70	P43403	ZAP70	0.039
Radix Saposhnikoviae（RS）	MOL011749	phelloptorin	Tyrosine-protein kinase SYK	P43405	SYK	0.039
Radix Saposhnikoviae（RS）	MOL011749	phelloptorin	Glycogen synthase kinase-3 beta	P49841	GSK3B	0.039
Radix Saposhnikoviae（RS）	MOL011749	phelloptorin	Protein kinase C theta type	Q04759	PRKCQ	0.039
Radix Saposhnikoviae（RS）	MOL011749	phelloptorin	Tyrosine-protein kinase ITK/TSK	Q08881	ITK	0.039
Radix Saposhnikoviae（RS）	MOL011749	phelloptorin	5-hydroxytryptamine 1F receptor	P30939	HTR1F	0.04
Radix Saposhnikoviae（RS）	MOL011749	phelloptorin	High affinity cAMP-specific and IBMX-insensitive 3',5'-cyclic phosphodiesterase 8A	O60658	PDE8A	0.041
Radix Saposhnikoviae（RS）	MOL011749	phelloptorin	6-phosphogluconate dehydrogenase, decarboxylating	P52209	PGD	0.041
Radix Saposhnikoviae（RS）	MOL011749	phelloptorin	High-affinity cAMP-specific 3',5'-cyclic phosphodiesterase 7A	Q13946	PDE7A	0.041
Radix Saposhnikoviae（RS）	MOL011749	phelloptorin	cAMP-specific 3',5'-cyclic phosphodiesterase 7B	Q9NP56	PDE7B	0.041
Radix Saposhnikoviae（RS）	MOL011749	phelloptorin	Alpha-1D adrenergic receptor	P25100	ADRA1D	0.042
Radix Saposhnikoviae（RS）	MOL011749	phelloptorin	Myeloperoxidase	P05164	MPO	0.044
Radix Saposhnikoviae（RS）	MOL011749	phelloptorin	Eosinophil peroxidase	P11678	EPX	0.044
Radix Saposhnikoviae（RS）	MOL011749	phelloptorin	Calreticulin	P27797	CALR	0.044
Radix Saposhnikoviae（RS）	MOL011749	phelloptorin	Melatonin receptor type 1A	P48039	MTNR1A	0.044
Radix Saposhnikoviae（RS）	MOL011749	phelloptorin	Melatonin receptor type 1B	P49286	MTNR1B	0.044
Radix Saposhnikoviae（RS）	MOL011749	phelloptorin	5-hydroxytryptamine 6 receptor	P50406	HTR6	0.044
Radix Saposhnikoviae（RS）	MOL011749	phelloptorin	Calmodulin	P62158		0.044
Radix Saposhnikoviae（RS）	MOL011749	phelloptorin	Nuclear receptor ROR-beta	Q92753	RORB	0.044
Radix Saposhnikoviae（RS）	MOL011749	phelloptorin	Potassium channel subfamily K member 1	O00180	KCNK1	0.045
Radix Saposhnikoviae（RS）	MOL011749	phelloptorin	4-aminobutyrate aminotransferase, mitochondrial	P80404	ABAT	0.045
Radix Saposhnikoviae（RS）	MOL011749	phelloptorin	Histone deacetylase 9	Q9UKV0	HDAC9	0.045

Radix Saposhnikoviae（RS）	MOL011749	phelloptorin	Lipoic acid synthetase, mitochondrial	O43766	LIAS	0.046
Radix Saposhnikoviae（RS）	MOL011749	phelloptorin	Carboxypeptidase A1	P15085	CPA1	0.046
Radix Saposhnikoviae（RS）	MOL011749	phelloptorin	Lipoyltransferase 1, mitochondrial	Q9Y234	LIPT1	0.046
Radix Saposhnikoviae（RS）	MOL011749	phelloptorin	Nitric-oxide synthase, endothelial	P29474	NOS3	0.047
Radix Saposhnikoviae（RS）	MOL011749	phelloptorin	Nitric-oxide synthase, brain	P29475	NOS1	0.047
Radix Saposhnikoviae（RS）	MOL011749	phelloptorin	Keratin, type II cytoskeletal 7	P08729	KRT7	0.049
Radix Saposhnikoviae（RS）	MOL011749	phelloptorin	Platelet glycoprotein IX	P14770	GP9	0.049
Radix Saposhnikoviae（RS）	MOL011749	phelloptorin	5-hydroxytryptamine 3 receptor	P46098	HTR3A	0.049
Radix Saposhnikoviae（RS）	MOL011749	phelloptorin	Methionine aminopeptidase 1	P53582	METAP1	0.052
Radix Saposhnikoviae（RS）	MOL011749	phelloptorin	Mineralocorticoid receptor	P08235	NR3C2	0.055
Radix Saposhnikoviae（RS）	MOL011749	phelloptorin	Gamma-aminobutyric-acid receptor subunit beta-3	P28472	GABRB3	0.055
Radix Saposhnikoviae（RS）	MOL011749	phelloptorin	Translocator protein	P30536	TSPO	0.055
Radix Saposhnikoviae（RS）	MOL011749	phelloptorin	Mitogen-activated protein kinase 8	P45983	MAPK8	0.056
Radix Saposhnikoviae（RS）	MOL011749	phelloptorin	Mitogen-activated protein kinase 10	P53779	MAPK10	0.056
Radix Saposhnikoviae（RS）	MOL011749	phelloptorin	Alcohol dehydrogenase [NADP+]	P14550	AKR1A1	0.057
Radix Saposhnikoviae（RS）	MOL011749	phelloptorin	Tyrosyl-tRNA synthetase, cytoplasmic	P54577	YARS	0.058
Radix Saposhnikoviae（RS）	MOL011749	phelloptorin	Solute carrier family 12 member 2	P55011	SLC12A2	0.058
Radix Saposhnikoviae（RS）	MOL011749	phelloptorin	Peptidyl-prolyl cis-trans isomerase NIMA-interacting 1	Q13526	PIN1	0.058
Radix Saposhnikoviae（RS）	MOL011749	phelloptorin	Solute carrier family 12 member 1	Q13621	SLC12A1	0.058
Radix Saposhnikoviae（RS）	MOL011749	phelloptorin	Carbonic anhydrase 4	P22748	CA4	0.059
Radix Saposhnikoviae（RS）	MOL011749	phelloptorin	Prostacyclin receptor	P43119	PTGIR	0.06
Radix Saposhnikoviae（RS）	MOL011749	phelloptorin	Glutamate [NMDA] receptor subunit epsilon-1	Q12879	GRIN2A	0.06
Radix Saposhnikoviae（RS）	MOL011749	phelloptorin	Glutamate [NMDA] receptor subunit epsilon-2	Q13224	GRIN2B	0.06
Radix Saposhnikoviae（RS）	MOL011749	phelloptorin	Glutamate [NMDA] receptor subunit epsilon-3	Q14957	GRIN2C	0.06
Radix Saposhnikoviae（RS）	MOL011749	phelloptorin	Peroxisome proliferator-activated receptor alpha	Q07869	PPARA	0.061
Radix Saposhnikoviae（RS）	MOL011749	phelloptorin	Mitogen-activated protein kinase 3	P27361	MAPK3	0.062
Radix Saposhnikoviae（RS）	MOL011749	phelloptorin	Gamma-aminobutyric-acid receptor subunit alpha-6	Q16445	GABRA6	0.064
Radix Saposhnikoviae（RS）	MOL011749	phelloptorin	Potassium voltage-gated channel subfamily H member 2	Q12809	KCNH2	0.065
Radix Saposhnikoviae（RS）	MOL011749	phelloptorin	Muscarinic acetylcholine receptor M5	P08912	CHRM5	0.066
Radix Saposhnikoviae（RS）	MOL011749	phelloptorin	5-hydroxytryptamine 2B receptor	P41595	HTR2B	0.066
Radix Saposhnikoviae（RS）	MOL011749	phelloptorin	Group IIE secretory phospholipase A2	Q9NZK7	PLA2G2E	0.066
Radix Saposhnikoviae（RS）	MOL011749	phelloptorin	Sodium-dependent serotonin transporter	P31645	SLC6A4	0.072
Radix Saposhnikoviae（RS）	MOL011749	phelloptorin	Gamma-aminobutyric-acid receptor subunit beta-2	P47870	GABRB2	0.073
Radix Saposhnikoviae（RS）	MOL011749	phelloptorin	Gamma-aminobutyric-acid receptor subunit alpha-4	P48169	GABRA4	0.073
Radix Saposhnikoviae（RS）	MOL011749	phelloptorin	Gamma-aminobutyric acid receptor subunit theta	Q9UN88	GABRQ	0.073
Radix Saposhnikoviae（RS）	MOL011749	phelloptorin	Tryptophanyl-tRNA synthetase, mitochondrial	Q9UGM6	WARS2	0.074
Radix Saposhnikoviae（RS）	MOL011749	phelloptorin	Ribosyldihydronicotinamide dehydrogenase [quinone]	P16083	NQO2	0.075
Radix Saposhnikoviae（RS）	MOL011749	phelloptorin	Glutamate [NMDA] receptor subunit 3A	Q8TCU5	GRIN3A	0.079
Radix Saposhnikoviae（RS）	MOL011749	phelloptorin	MAP kinase-activated protein kinase 2	P49137	MAPKAPK2	0.084
Radix Saposhnikoviae（RS）	MOL011749	phelloptorin	Muscarinic acetylcholine receptor M4	P08173	CHRM4	0.09
Radix Saposhnikoviae（RS）	MOL011749	phelloptorin	Muscarinic acetylcholine receptor M3	P20309	CHRM3	0.09
Radix Saposhnikoviae（RS）	MOL011749	phelloptorin	Potassium voltage-gated channel subfamily KQT member 1	P51787	KCNQ1	0.093
Radix Saposhnikoviae（RS）	MOL011749	phelloptorin	Estradiol 17-beta-dehydrogenase 1	P14061	HSD17B1	0.094
Radix Saposhnikoviae（RS）	MOL011749	phelloptorin	ATP-sensitive inward rectifier potassium channel 1	P48048	KCNJ1	0.095
Radix Saposhnikoviae（RS）	MOL011749	phelloptorin	Prothrombin	P00734	F2	0.096
Radix Saposhnikoviae（RS）	MOL011749	phelloptorin	Lysozyme C	P61626	LYZ	0.096

Radix Saposhnikoviae（RS）	MOL011749	phelloptorin	Protein farnesyltransferase/geranylgeranyltransferase type I alpha subunit	P49354	FNTA	0.097
Radix Saposhnikoviae（RS）	MOL011749	phelloptorin	Tubulin alpha-3 chain	Q71U36	TUBA1A	0.098
Radix Saposhnikoviae（RS）	MOL011749	phelloptorin	Nitric oxide synthase, inducible	P35228	NOS2	0.099
Radix Saposhnikoviae（RS）	MOL011749	phelloptorin	Cannabinoid receptor 1	P21554	CNR1	0.1
Radix Saposhnikoviae（RS）	MOL011749	phelloptorin	Cannabinoid receptor 2	P34972	CNR2	0.103
Radix Saposhnikoviae（RS）	MOL011749	phelloptorin	Phosphatidylinositol-4,5-bisphosphate 3-kinase catalytic subunit gamma isoform	P48736	PIK3CG	0.105
Radix Saposhnikoviae（RS）	MOL011749	phelloptorin	Ig kappa chain V-II region RPMI 6410	P06310	IGKV2-30	0.107
Radix Saposhnikoviae（RS）	MOL011749	phelloptorin	Tyrosine-protein kinase HCK	P08631	HCK	0.108
Radix Saposhnikoviae（RS）	MOL011749	phelloptorin	Liver carboxylesterase 1	P23141	CES1	0.108
Radix Saposhnikoviae（RS）	MOL011749	phelloptorin	S-methyl-5-thioadenosine phosphorylase	Q13126	MTAP	0.108
Radix Saposhnikoviae（RS）	MOL011749	phelloptorin	Egl nine homolog 1	Q9GZT9	EGLN1	0.108
Radix Saposhnikoviae（RS）	MOL011749	phelloptorin	Thromboxane A2 receptor	P21731	TBXA2R	0.109
Radix Saposhnikoviae（RS）	MOL011749	phelloptorin	DNA polymerase kappa	Q9UBT6	POLK	0.112
Radix Saposhnikoviae（RS）	MOL011749	phelloptorin	Muscarinic acetylcholine receptor M1	P11229	CHRM1	0.113
Radix Saposhnikoviae（RS）	MOL011749	phelloptorin	Triosephosphate isomerase	P60174	TPI1	0.113
Radix Saposhnikoviae（RS）	MOL011749	phelloptorin	Hydroxyacid oxidase 1	Q9UJM8	HAO1	0.113
Radix Saposhnikoviae（RS）	MOL011749	phelloptorin	Muscarinic acetylcholine receptor M2	P08172	CHRM2	0.114
Radix Saposhnikoviae（RS）	MOL011749	phelloptorin	Beta-1 adrenergic receptor	P08588	ADRB1	0.114
Radix Saposhnikoviae（RS）	MOL011749	phelloptorin	D(4) dopamine receptor	P21917	DRD4	0.115
Radix Saposhnikoviae（RS）	MOL011749	phelloptorin	D(1B) dopamine receptor	P21918	DRD5	0.115
Radix Saposhnikoviae（RS）	MOL011749	phelloptorin	Amine oxidase [flavin-containing] B	P27338	MAOB	0.115
Radix Saposhnikoviae（RS）	MOL011749	phelloptorin	D(3) dopamine receptor	P35462	DRD3	0.115
Radix Saposhnikoviae（RS）	MOL011749	phelloptorin	Alpha-1B adrenergic receptor	P35368	ADRA1B	0.117
Radix Saposhnikoviae（RS）	MOL011749	phelloptorin	Toll-like receptor 7	Q9NYK1	TLR7	0.117
Radix Saposhnikoviae（RS）	MOL011749	phelloptorin	Progesterone receptor	P06401	PGR	0.118
Radix Saposhnikoviae（RS）	MOL011749	phelloptorin	Gamma-aminobutyric acid receptor subunit gamma-2	P18507	GABRG2	0.118
Radix Saposhnikoviae（RS）	MOL011749	phelloptorin	C-jun-amino-terminal kinase-interacting protein 1	Q9UQF2	MAPK8IP1	0.118
Radix Saposhnikoviae（RS）	MOL011749	phelloptorin	Androgen receptor	P10275	AR	0.119
Radix Saposhnikoviae（RS）	MOL011749	phelloptorin	Carbonic anhydrase 1	P00915	CA1	0.125
Radix Saposhnikoviae（RS）	MOL011749	phelloptorin	Trypsin-1	P07477	PRSS1	0.126
Radix Saposhnikoviae（RS）	MOL011749	phelloptorin	Aldose reductase	P15121	AKR1B1	0.126
Radix Saposhnikoviae（RS）	MOL011749	phelloptorin	Gamma-aminobutyric acid receptor subunit rho-3	A8MPY1	GABRR3	0.127
Radix Saposhnikoviae（RS）	MOL011749	phelloptorin	Gamma-aminobutyric acid receptor subunit pi	O00591	GABRP	0.127
Radix Saposhnikoviae（RS）	MOL011749	phelloptorin	Gamma-aminobutyric-acid receptor subunit rho-1	P24046	GABRR1	0.127
Radix Saposhnikoviae（RS）	MOL011749	phelloptorin	Gamma-aminobutyric acid receptor subunit rho-2	P28476	GABRR2	0.127
Radix Saposhnikoviae（RS）	MOL011749	phelloptorin	Gamma-aminobutyric-acid receptor subunit alpha-3	P34903	GABRA3	0.127
Radix Saposhnikoviae（RS）	MOL011749	phelloptorin	Gamma-aminobutyric acid receptor subunit epsilon	P78334	GABRE	0.127
Radix Saposhnikoviae（RS）	MOL011749	phelloptorin	Gamma-aminobutyric acid receptor subunit gamma-1	Q8N1C3	GABRG1	0.127
Radix Saposhnikoviae（RS）	MOL011749	phelloptorin	Gamma-aminobutyric acid receptor subunit gamma-3	Q99928	GABRG3	0.127
Radix Saposhnikoviae（RS）	MOL011749	phelloptorin	cAMP-dependent protein kinase inhibitor alpha	P61925	PKIA	0.133
Radix Saposhnikoviae（RS）	MOL011749	phelloptorin	Rho-associated protein kinase 1	Q13464	ROCK1	0.133
Radix Saposhnikoviae（RS）	MOL011749	phelloptorin	Gamma-aminobutyric acid receptor subunit delta	O14764	GABRD	0.136
Radix Saposhnikoviae（RS）	MOL011749	phelloptorin	Beta-2 adrenergic receptor	P07550	ADRB2	0.136
Radix Saposhnikoviae（RS）	MOL011749	phelloptorin	Gamma-aminobutyric-acid receptor subunit beta-1	P18505	GABRB1	0.136
Radix Saposhnikoviae（RS）	MOL011749	phelloptorin	5-hydroxytryptamine 1D receptor	P28221	HTR1D	0.136
Radix Saposhnikoviae（RS）	MOL011749	phelloptorin	5-hydroxytryptamine 1B receptor	P28222	HTR1B	0.136

Radix Saposhnikoviae（RS）	MOL011749	phelloptorin	Gamma-aminobutyric-acid receptor subunit alpha-5	P31644	GABRA5	0.136
Radix Saposhnikoviae（RS）	MOL011749	phelloptorin	Alpha-2B adrenergic receptor	P18089	ADRA2B	0.139
Radix Saposhnikoviae（RS）	MOL011749	phelloptorin	Alpha-2C adrenergic receptor	P18825	ADRA2C	0.139
Radix Saposhnikoviae（RS）	MOL011749	phelloptorin	Phospholipase A2	P04054	PLA2G1B	0.141
Radix Saposhnikoviae（RS）	MOL011749	phelloptorin	Alpha-2A adrenergic receptor	P08913	ADRA2A	0.141
Radix Saposhnikoviae（RS）	MOL011749	phelloptorin	5-hydroxytryptamine 2C receptor	P28335	HTR2C	0.141
Radix Saposhnikoviae（RS）	MOL011749	phelloptorin	cAMP-specific 3',5'-cyclic phosphodiesterase 4C	Q08493	PDE4C	0.143
Radix Saposhnikoviae（RS）	MOL011749	phelloptorin	Sodium-dependent noradrenaline transporter	P23975	SLC6A2	0.15
Radix Saposhnikoviae（RS）	MOL011749	phelloptorin	Histamine H1 receptor	P35367	HRH1	0.155
Radix Saposhnikoviae（RS）	MOL011749	phelloptorin	Gamma-aminobutyric-acid receptor subunit alpha-2	P47869	GABRA2	0.156
Radix Saposhnikoviae（RS）	MOL011749	phelloptorin	Carbonic anhydrase 2	P00918	CA2	0.158
Radix Saposhnikoviae（RS）	MOL011749	phelloptorin	Gamma-aminobutyric-acid receptor subunit alpha-1	P14867	GABRA1	0.165
Radix Saposhnikoviae（RS）	MOL011749	phelloptorin	Sodium channel protein type 5 subunit alpha	Q14524	SCN5A	0.182
Radix Saposhnikoviae（RS）	MOL011749	phelloptorin	Hemoglobin subunit alpha	P69905	HBA1	0.183
Radix Saposhnikoviae（RS）	MOL011749	phelloptorin	D(1A) dopamine receptor	P21728	DRD1	0.189
Radix Saposhnikoviae（RS）	MOL011749	phelloptorin	Alpha-1A adrenergic receptor	P35348	ADRA1A	0.193
Radix Saposhnikoviae（RS）	MOL011749	phelloptorin	Delta-type opioid receptor	P41143	OPRD1	0.193
Radix Saposhnikoviae（RS）	MOL011749	phelloptorin	Peroxisome proliferator-activated receptor delta	Q03181	PPARD	0.197
Radix Saposhnikoviae（RS）	MOL011749	phelloptorin	Prostaglandin G/H synthase 1	P23219	PTGS1	0.204
Radix Saposhnikoviae（RS）	MOL011749	phelloptorin	RAC-alpha serine/threonine-protein kinase	P31749	AKT1	0.213
Radix Saposhnikoviae（RS）	MOL011749	phelloptorin	Proto-oncogene tyrosine-protein kinase Src	P12931	SRC	0.216
Radix Saposhnikoviae（RS）	MOL011749	phelloptorin	D(2) dopamine receptor	P14416	DRD2	0.24
Radix Saposhnikoviae（RS）	MOL011749	phelloptorin	5-hydroxytryptamine 2A receptor	P28223	HTR2A	0.241
Radix Saposhnikoviae（RS）	MOL011749	phelloptorin	cAMP-specific 3',5'-cyclic phosphodiesterase 4D	Q08499	PDE4D	0.246
Radix Saposhnikoviae（RS）	MOL011749	phelloptorin	5-hydroxytryptamine 1A receptor	P08908	HTR1A	0.255
Radix Saposhnikoviae（RS）	MOL011749	phelloptorin	Kappa-type opioid receptor	P41145	OPRK1	0.265
Radix Saposhnikoviae（RS）	MOL011749	phelloptorin	Nuclear receptor coactivator 1	Q15788	NCOA1	0.269
Radix Saposhnikoviae（RS）	MOL011749	phelloptorin	cAMP-specific 3',5'-cyclic phosphodiesterase 4A	P27815	PDE4A	0.297
Radix Saposhnikoviae（RS）	MOL011749	phelloptorin	Cell division protein kinase 2	P24941	CDK2	0.305
Radix Saposhnikoviae（RS）	MOL011749	phelloptorin	Cyclin-A2	P20248	CCNA2	0.345
Radix Saposhnikoviae（RS）	MOL011749	phelloptorin	cAMP-specific 3',5'-cyclic phosphodiesterase 4B	Q07343	PDE4B	0.349
Radix Saposhnikoviae（RS）	MOL011749	phelloptorin	Mu-type opioid receptor	P35372	OPRM1	0.38
Radix Saposhnikoviae（RS）	MOL011749	phelloptorin	cAMP-dependent protein kinase catalytic subunit alpha	P17612	PRKACA	0.483
Radix Saposhnikoviae（RS）	MOL011749	phelloptorin	Estrogen receptor beta	Q92731	ESR2	0.528
Radix Saposhnikoviae（RS）	MOL011749	phelloptorin	Estrogen receptor	P03372	ESR1	0.916
Radix Saposhnikoviae（RS）	MOL011749	phelloptorin	Prostaglandin G/H synthase 2	P35354	PTGS2	1
Radix Saposhnikoviae（RS）	MOL011753	5-O-Methylvisamminol	Voltage-dependent T-type calcium channel subunit alpha-1G	O43497	CACNA1G	0.01
Radix Saposhnikoviae（RS）	MOL011753	5-O-Methylvisamminol	Carbonic anhydrase-related protein 11	O75493	CA11	0.01
Radix Saposhnikoviae（RS）	MOL011753	5-O-Methylvisamminol	Voltage-dependent T-type calcium channel subunit alpha-1H	O95180	CACNA1H	0.01
Radix Saposhnikoviae（RS）	MOL011753	5-O-Methylvisamminol	Acetylcholine receptor subunit alpha	P02708	CHRNA1	0.01
Radix Saposhnikoviae（RS）	MOL011753	5-O-Methylvisamminol	Cholinesterase	P06276	BCHE	0.01
Radix Saposhnikoviae（RS）	MOL011753	5-O-Methylvisamminol	Carbonic anhydrase 3	P07451	CA3	0.01
Radix Saposhnikoviae（RS）	MOL011753	5-O-Methylvisamminol	Acetylcholine receptor subunit gamma	P07510	CHRNG	0.01
Radix Saposhnikoviae（RS）	MOL011753	5-O-Methylvisamminol	Acetylcholine receptor subunit beta	P11230	CHRNB1	0.01
Radix Saposhnikoviae（RS）	MOL011753	5-O-Methylvisamminol	Neuronal acetylcholine receptor subunit beta-2	P17787	CHRNB2	0.01
Radix Saposhnikoviae（RS）	MOL011753	5-O-Methylvisamminol	Carbonic anhydrase 6	P23280	CA6	0.01
Radix Saposhnikoviae（RS）	MOL011753	5-O-Methylvisamminol	Amine oxidase [flavin-containing] B	P27338	MAOB	0.01

Radix Saposhnikoviae（RS）	MOL011753	5-O-Methylvisamminol	Neuronal acetylcholine receptor subunit alpha-5	P30532	CHRNA5	0.01
Radix Saposhnikoviae（RS）	MOL011753	5-O-Methylvisamminol	Neuronal acetylcholine receptor subunit beta-4	P30926	CHRNB4	0.01
Radix Saposhnikoviae（RS）	MOL011753	5-O-Methylvisamminol	Carbonic anhydrase 5A, mitochondrial	P35218	CA5A	0.01
Radix Saposhnikoviae（RS）	MOL011753	5-O-Methylvisamminol	Carbonic anhydrase-related protein	P35219	CA8	0.01
Radix Saposhnikoviae（RS）	MOL011753	5-O-Methylvisamminol	Sodium channel protein type 4 subunit alpha	P35499	SCN4A	0.01
Radix Saposhnikoviae（RS）	MOL011753	5-O-Methylvisamminol	Carbonic anhydrase 7	P43166	CA7	0.01
Radix Saposhnikoviae（RS）	MOL011753	5-O-Methylvisamminol	Neuronal acetylcholine receptor subunit alpha-4	P43681	CHRNA4	0.01
Radix Saposhnikoviae（RS）	MOL011753	5-O-Methylvisamminol	Acetylcholine receptor subunit epsilon	Q04844	CHRNE	0.01
Radix Saposhnikoviae（RS）	MOL011753	5-O-Methylvisamminol	Neuronal acetylcholine receptor subunit beta-3	Q05901	CHRNB3	0.01
Radix Saposhnikoviae（RS）	MOL011753	5-O-Methylvisamminol	Acetylcholine receptor subunit delta	Q07001	CHRND	0.01
Radix Saposhnikoviae（RS）	MOL011753	5-O-Methylvisamminol	Sodium channel subunit beta-1	Q07699	SCN1B	0.01
Radix Saposhnikoviae（RS）	MOL011753	5-O-Methylvisamminol	Neuronal acetylcholine receptor subunit alpha-6	Q15825	KCNJ8	0.01
Radix Saposhnikoviae（RS）	MOL011753	5-O-Methylvisamminol	Sodium channel protein type 9 subunit alpha	Q15858	SCN9A	0.01
Radix Saposhnikoviae（RS）	MOL011753	5-O-Methylvisamminol	Sodium channel subunit beta-4	Q8IWT1	SCN4B	0.01
Radix Saposhnikoviae（RS）	MOL011753	5-O-Methylvisamminol	Carbonic anhydrase 13	Q8N1Q1	CA13	0.01
Radix Saposhnikoviae（RS）	MOL011753	5-O-Methylvisamminol	Sodium channel protein type 2 subunit alpha	Q99250	SCN2A	0.01
Radix Saposhnikoviae（RS）	MOL011753	5-O-Methylvisamminol	Neuronal acetylcholine receptor subunit alpha-10	Q9GZZ6	CHRNA10	0.01
Radix Saposhnikoviae（RS）	MOL011753	5-O-Methylvisamminol	Carbonic anhydrase-related protein 10	Q9NS85	CA10	0.01
Radix Saposhnikoviae（RS）	MOL011753	5-O-Methylvisamminol	Sodium channel protein type 3 subunit alpha	Q9NY46	SCN3A	0.01
Radix Saposhnikoviae（RS）	MOL011753	5-O-Methylvisamminol	Sodium channel subunit beta-3	Q9NY72	SCN3B	0.01
Radix Saposhnikoviae（RS）	MOL011753	5-O-Methylvisamminol	Voltage-dependent T-type calcium channel subunit alpha-1I	Q9P0X4	CACNA1I	0.01
Radix Saposhnikoviae（RS）	MOL011753	5-O-Methylvisamminol	Neuronal acetylcholine receptor subunit alpha-9	Q9UGM1	CHRNA9	0.01
Radix Saposhnikoviae（RS）	MOL011753	5-O-Methylvisamminol	Sodium channel protein type 11 subunit alpha	Q9UI33	SCN11A	0.01
Radix Saposhnikoviae（RS）	MOL011753	5-O-Methylvisamminol	Carbonic anhydrase 14	Q9ULX7	CA14	0.01
Radix Saposhnikoviae（RS）	MOL011753	5-O-Methylvisamminol	Carbonic anhydrase 5B, mitochondrial	Q9Y2D0	CA5B	0.01
Radix Saposhnikoviae（RS）	MOL011753	5-O-Methylvisamminol	Cytochrome b	P00156	MT-CYB	0.012
Radix Saposhnikoviae（RS）	MOL011753	5-O-Methylvisamminol	Muscarinic acetylcholine receptor M2	P08172	CHRM2	0.012
Radix Saposhnikoviae（RS）	MOL011753	5-O-Methylvisamminol	Cytochrome c1, heme protein, mitochondrial	P08574	CYC1	0.012
Radix Saposhnikoviae（RS）	MOL011753	5-O-Methylvisamminol	Alpha-1D adrenergic receptor	P25100	ADRA1D	0.012
Radix Saposhnikoviae（RS）	MOL011753	5-O-Methylvisamminol	5-hydroxytryptamine 1F receptor	P30939	HTR1F	0.012
Radix Saposhnikoviae（RS）	MOL011753	5-O-Methylvisamminol	Cytochrome b-c1 complex subunit Rieske, mitochondrial	P47985	UQCRFS1	0.012
Radix Saposhnikoviae（RS）	MOL011753	5-O-Methylvisamminol	Solute carrier family 12 member 2	P55011	SLC12A2	0.012
Radix Saposhnikoviae（RS）	MOL011753	5-O-Methylvisamminol	Calcium-activated potassium channel subunit alpha 1	Q12791	KCNMA1	0.012
Radix Saposhnikoviae（RS）	MOL011753	5-O-Methylvisamminol	ATP-sensitive inward rectifier potassium channel 11	Q14654	KCNJ11	0.012
Radix Saposhnikoviae（RS）	MOL011753	5-O-Methylvisamminol	2,4-dienoyl-CoA reductase, mitochondrial	Q16698	DECR1	0.012
Radix Saposhnikoviae（RS）	MOL011753	5-O-Methylvisamminol	Chromaffin granule amine transporter	P54219	SLC18A1	0.013
Radix Saposhnikoviae（RS）	MOL011753	5-O-Methylvisamminol	Synaptic vesicular amine transporter	Q05940	SLC18A2	0.013
Radix Saposhnikoviae（RS）	MOL011753	5-O-Methylvisamminol	D1 dopamine receptor-interacting protein calcyon	Q9NYX4	CALY	0.013
Radix Saposhnikoviae（RS）	MOL011753	5-O-Methylvisamminol	Elongation factor 2	P13639	EEF2	0.014
Radix Saposhnikoviae（RS）	MOL011753	5-O-Methylvisamminol	Opioid receptor, sigma 1	Q5T1J1	SIGMAR1	0.014
Radix Saposhnikoviae（RS）	MOL011753	5-O-Methylvisamminol	Sigma 1-type opioid receptor	Q99720	SIGMAR1	0.014
Radix Saposhnikoviae（RS）	MOL011753	5-O-Methylvisamminol	Poly [ADP-ribose] polymerase 3	Q9Y6F1	PARP3	0.014
Radix Saposhnikoviae（RS）	MOL011753	5-O-Methylvisamminol	Potassium channel subfamily K member 1	O00180	KCNK1	0.016
Radix Saposhnikoviae（RS）	MOL011753	5-O-Methylvisamminol	Gamma-aminobutyric-acid receptor subunit beta-3	P28472	GABRB3	0.016
Radix Saposhnikoviae（RS）	MOL011753	5-O-Methylvisamminol	Sodium channel protein type 1 subunit alpha	P35498	SCN1A	0.017
Radix Saposhnikoviae（RS）	MOL011753	5-O-Methylvisamminol	5-hydroxytryptamine 4 receptor	Q13639	HTR4	0.017
Radix Saposhnikoviae（RS）	MOL011753	5-O-Methylvisamminol	Alpha-7 nicotinic cholinergic receptor subunit	Q693P7	CHRNA7	0.017

Radix Saposhnikoviae（RS）	MOL011753	5-O-Methylvisamminol	Coagulation factor IX	P00740	F9	0.019
Radix Saposhnikoviae（RS）	MOL011753	5-O-Methylvisamminol	Coagulation factor X	P00742	F10	0.019
Radix Saposhnikoviae（RS）	MOL011753	5-O-Methylvisamminol	Osteocalcin	P02818	BGLAP	0.019
Radix Saposhnikoviae（RS）	MOL011753	5-O-Methylvisamminol	Vitamin K-dependent protein C	P04070	PROC	0.019
Radix Saposhnikoviae（RS）	MOL011753	5-O-Methylvisamminol	Coagulation factor VII	P08709	F7	0.019
Radix Saposhnikoviae（RS）	MOL011753	5-O-Methylvisamminol	NAD(P)H dehydrogenase [quinone] 1	P15559	NQO1	0.019
Radix Saposhnikoviae（RS）	MOL011753	5-O-Methylvisamminol	Vitamin K-dependent protein Z	P22891	PROZ	0.019
Radix Saposhnikoviae（RS）	MOL011753	5-O-Methylvisamminol	Vitamin K-dependent gamma-carboxylase	P38435	GGCX	0.019
Radix Saposhnikoviae（RS）	MOL011753	5-O-Methylvisamminol	Glutamate [NMDA] receptor subunit zeta-1	Q05586	GRIN1	0.019
Radix Saposhnikoviae（RS）	MOL011753	5-O-Methylvisamminol	Glutamate [NMDA] receptor subunit epsilon-3	Q14957	GRIN2C	0.019
Radix Saposhnikoviae（RS）	MOL011753	5-O-Methylvisamminol	Cyclin-dependent kinase 5 activator 1	Q15078	CDK5R1	0.019
Radix Saposhnikoviae（RS）	MOL011753	5-O-Methylvisamminol	Vitamin K epoxide reductase complex subunit 1-like protein 1	Q8N0U8	VKORC1L1	0.019
Radix Saposhnikoviae（RS）	MOL011753	5-O-Methylvisamminol	Vitamin K epoxide reductase complex subunit 1	Q9BQB6	VKORC1	0.019
Radix Saposhnikoviae（RS）	MOL011753	5-O-Methylvisamminol	Beta-3 adrenergic receptor	P13945	ADRB3	0.02
Radix Saposhnikoviae（RS）	MOL011753	5-O-Methylvisamminol	cAMP response element-binding protein	P16220	CREB1	0.02
Radix Saposhnikoviae（RS）	MOL011753	5-O-Methylvisamminol	Nociceptin receptor	P41146	OPRL1	0.02
Radix Saposhnikoviae（RS）	MOL011753	5-O-Methylvisamminol	85 kDa calcium-independent phospholipase A2	O60733	PLA2G6	0.021
Radix Saposhnikoviae（RS）	MOL011753	5-O-Methylvisamminol	Tumor necrosis factor	P01375	TNF	0.021
Radix Saposhnikoviae（RS）	MOL011753	5-O-Methylvisamminol	Interleukin-3	P08700	IL3	0.021
Radix Saposhnikoviae（RS）	MOL011753	5-O-Methylvisamminol	DNA topoisomerase 2-alpha	P11388	TOP2A	0.021
Radix Saposhnikoviae（RS）	MOL011753	5-O-Methylvisamminol	Nuclear factor NF-kappa-B p105 subunit	P19838	NFKB1	0.021
Radix Saposhnikoviae（RS）	MOL011753	5-O-Methylvisamminol	Fibroblast growth factor receptor 2	P21802	FGFR2	0.021
Radix Saposhnikoviae（RS）	MOL011753	5-O-Methylvisamminol	DNA-(apurinic or apyrimidinic site) lyase	P27695	APEX1	0.021
Radix Saposhnikoviae（RS）	MOL011753	5-O-Methylvisamminol	Cytosolic phospholipase A2	P47712	PLA2G4A	0.021
Radix Saposhnikoviae（RS）	MOL011753	5-O-Methylvisamminol	Protein S100-A12	P80511	S100A12	0.021
Radix Saposhnikoviae（RS）	MOL011753	5-O-Methylvisamminol	Protein S100-A13	Q99584	S100A13	0.021
Radix Saposhnikoviae（RS）	MOL011753	5-O-Methylvisamminol	Ig kappa chain C region	P01834	IGKC	0.022
Radix Saposhnikoviae（RS）	MOL011753	5-O-Methylvisamminol	Ig gamma-1 chain C region	P01857	IGHG1	0.022
Radix Saposhnikoviae（RS）	MOL011753	5-O-Methylvisamminol	Ig gamma-2 chain C region	P01859	IGHG2	0.022
Radix Saposhnikoviae（RS）	MOL011753	5-O-Methylvisamminol	Translocator protein	P30536	TSPO	0.023
Radix Saposhnikoviae（RS）	MOL011753	5-O-Methylvisamminol	Gamma-aminobutyric-acid receptor subunit alpha-6	Q16445	GABRA6	0.023
Radix Saposhnikoviae（RS）	MOL011753	5-O-Methylvisamminol	Acetylcholinesterase	P22303	ACHE	0.024
Radix Saposhnikoviae（RS）	MOL011753	5-O-Methylvisamminol	Neuronal acetylcholine receptor subunit alpha-3	P32297	CHRNA3	0.024
Radix Saposhnikoviae（RS）	MOL011753	5-O-Methylvisamminol	Neuronal acetylcholine receptor subunit alpha-7	P36544	CHRNA7	0.024
Radix Saposhnikoviae（RS）	MOL011753	5-O-Methylvisamminol	Solute carrier family 22 member 6	Q4U2R8	SLC22A6	0.024
Radix Saposhnikoviae（RS）	MOL011753	5-O-Methylvisamminol	Dehydrogenase/reductase SDR family member 8	Q8NBQ5	HSD17B11	0.024
Radix Saposhnikoviae（RS）	MOL011753	5-O-Methylvisamminol	Solute carrier family 22 member 8	Q8TCC7	SLC22A8	0.024
Radix Saposhnikoviae（RS）	MOL011753	5-O-Methylvisamminol	Solute carrier family 22 member 11	Q9NSA0	SLC22A11	0.024
Radix Saposhnikoviae（RS）	MOL011753	5-O-Methylvisamminol	Carbonic anhydrase 12	O43570	CA12	0.025
Radix Saposhnikoviae（RS）	MOL011753	5-O-Methylvisamminol	Tyrosine-protein kinase Lyn	P07948	LYN	0.025
Radix Saposhnikoviae（RS）	MOL011753	5-O-Methylvisamminol	Carbonic anhydrase 9	Q16790	CA9	0.025
Radix Saposhnikoviae（RS）	MOL011753	5-O-Methylvisamminol	3-phosphoinositide-dependent protein kinase 1	O15530	PDPK1	0.026
Radix Saposhnikoviae（RS）	MOL011753	5-O-Methylvisamminol	Proto-oncogene serine/threonine-protein kinase Pim-1	P11309	PIM1	0.026
Radix Saposhnikoviae（RS）	MOL011753	5-O-Methylvisamminol	Tyrosine-protein kinase CSK	P41240	CSK	0.026
Radix Saposhnikoviae（RS）	MOL011753	5-O-Methylvisamminol	Tyrosine-protein kinase ZAP-70	P43403	ZAP70	0.026
Radix Saposhnikoviae（RS）	MOL011753	5-O-Methylvisamminol	Tyrosine-protein kinase SYK	P43405	SYK	0.026
Radix Saposhnikoviae（RS）	MOL011753	5-O-Methylvisamminol	Protein kinase C theta type	Q04759	PRKCQ	0.026

Radix Saposhnikoviae（RS）	MOL011753	5-O-Methylvisamminol	cAMP-specific 3',5'-cyclic phosphodiesterase 4B	Q07343	PDE4B	0.026
Radix Saposhnikoviae（RS）	MOL011753	5-O-Methylvisamminol	cAMP-specific 3',5'-cyclic phosphodiesterase 4C	Q08493	PDE4C	0.026
Radix Saposhnikoviae（RS）	MOL011753	5-O-Methylvisamminol	cAMP-specific 3',5'-cyclic phosphodiesterase 4D	Q08499	PDE4D	0.026
Radix Saposhnikoviae（RS）	MOL011753	5-O-Methylvisamminol	Tyrosine-protein kinase ITK/TSK	Q08881	ITK	0.026
Radix Saposhnikoviae（RS）	MOL011753	5-O-Methylvisamminol	cGMP-inhibited 3',5'-cyclic phosphodiesterase A	Q14432	PDE3A	0.026
Radix Saposhnikoviae（RS）	MOL011753	5-O-Methylvisamminol	cAMP and cAMP-inhibited cGMP 3',5'-cyclic phosphodiesterase 10A	Q9Y233	PDE10A	0.026
Radix Saposhnikoviae（RS）	MOL011753	5-O-Methylvisamminol	Amine oxidase [flavin-containing] A	P21397	MAOA	0.027
Radix Saposhnikoviae（RS）	MOL011753	5-O-Methylvisamminol	Solute carrier family 12 member 3	P55017	SLC12A3	0.027
Radix Saposhnikoviae（RS）	MOL011753	5-O-Methylvisamminol	Sodium/potassium-transporting ATPase alpha-1 chain	P05023	ATP1A1	0.029
Radix Saposhnikoviae（RS）	MOL011753	5-O-Methylvisamminol	Muscarinic acetylcholine receptor M4	P08173	CHRM4	0.029
Radix Saposhnikoviae（RS）	MOL011753	5-O-Methylvisamminol	Muscarinic acetylcholine receptor M5	P08912	CHRM5	0.029
Radix Saposhnikoviae（RS）	MOL011753	5-O-Methylvisamminol	Histamine H1 receptor	P35367	HRH1	0.029
Radix Saposhnikoviae（RS）	MOL011753	5-O-Methylvisamminol	Alpha-1B adrenergic receptor	P35368	ADRA1B	0.029
Radix Saposhnikoviae（RS）	MOL011753	5-O-Methylvisamminol	Neuronal acetylcholine receptor subunit alpha-2	Q15822	CHRNA2	0.029
Radix Saposhnikoviae（RS）	MOL011753	5-O-Methylvisamminol	Gamma-aminobutyric acid receptor subunit gamma-2	P18507	GABRG2	0.03
Radix Saposhnikoviae（RS）	MOL011753	5-O-Methylvisamminol	Plasminogen	P00747	PLG	0.032
Radix Saposhnikoviae（RS）	MOL011753	5-O-Methylvisamminol	Tissue-type plasminogen activator	P00750	PLAT	0.032
Radix Saposhnikoviae（RS）	MOL011753	5-O-Methylvisamminol	Tyrosine 3-monooxygenase	P07101	TH	0.032
Radix Saposhnikoviae（RS）	MOL011753	5-O-Methylvisamminol	Prostacyclin receptor	P43119	PTGIR	0.032
Radix Saposhnikoviae（RS）	MOL011753	5-O-Methylvisamminol	Peroxisome proliferator-activated receptor delta	Q03181	PPARD	0.032
Radix Saposhnikoviae（RS）	MOL011753	5-O-Methylvisamminol	Peptidyl-prolyl cis-trans isomerase NIMA-interacting 1	Q13526	PIN1	0.032
Radix Saposhnikoviae（RS）	MOL011753	5-O-Methylvisamminol	Tyrosyl-tRNA synthetase, mitochondrial	Q9Y2Z4	YARS2	0.032
Radix Saposhnikoviae（RS）	MOL011753	5-O-Methylvisamminol	Myeloperoxidase	P05164	MPO	0.034
Radix Saposhnikoviae（RS）	MOL011753	5-O-Methylvisamminol	ATP synthase subunit beta, mitochondrial	P06576	ATP5F1B	0.034
Radix Saposhnikoviae（RS）	MOL011753	5-O-Methylvisamminol	Eosinophil peroxidase	P11678	EPX	0.034
Radix Saposhnikoviae（RS）	MOL011753	5-O-Methylvisamminol	ATP synthase subunit alpha, mitochondrial	P25705	ATP5F1A	0.034
Radix Saposhnikoviae（RS）	MOL011753	5-O-Methylvisamminol	Calreticulin	P27797	CALR	0.034
Radix Saposhnikoviae（RS）	MOL011753	5-O-Methylvisamminol	RAC-beta serine/threonine-protein kinase	P31751	AKT2	0.034
Radix Saposhnikoviae（RS）	MOL011753	5-O-Methylvisamminol	Melatonin receptor type 1B	P49286	MTNR1B	0.034
Radix Saposhnikoviae（RS）	MOL011753	5-O-Methylvisamminol	Calmodulin	P62158		0.034
Radix Saposhnikoviae（RS）	MOL011753	5-O-Methylvisamminol	Nuclear receptor ROR-beta	Q92753	RORB	0.034
Radix Saposhnikoviae（RS）	MOL011753	5-O-Methylvisamminol	Annexin A1	P04083	ANXA1	0.035
Radix Saposhnikoviae（RS）	MOL011753	5-O-Methylvisamminol	Phosphatidylinositol 3-kinase regulatory subunit alpha	P27986	PIK3R1	0.035
Radix Saposhnikoviae（RS）	MOL011753	5-O-Methylvisamminol	Nuclear receptor 0B1	P51843	NR0B1	0.035
Radix Saposhnikoviae（RS）	MOL011753	5-O-Methylvisamminol	Serine/threonine-protein kinase PLK1	P53350	PLK1	0.035
Radix Saposhnikoviae（RS）	MOL011753	5-O-Methylvisamminol	Gamma-aminobutyric acid receptor subunit rho-3	A8MPY1	GABRR3	0.036
Radix Saposhnikoviae（RS）	MOL011753	5-O-Methylvisamminol	Gamma-aminobutyric acid receptor subunit pi	O00591	GABRP	0.036
Radix Saposhnikoviae（RS）	MOL011753	5-O-Methylvisamminol	Gamma-aminobutyric-acid receptor subunit rho-1	P24046	GABRR1	0.036
Radix Saposhnikoviae（RS）	MOL011753	5-O-Methylvisamminol	Gamma-aminobutyric acid receptor subunit rho-2	P28476	GABRR2	0.036
Radix Saposhnikoviae（RS）	MOL011753	5-O-Methylvisamminol	Gamma-aminobutyric-acid receptor subunit alpha-3	P34903	GABRA3	0.036
Radix Saposhnikoviae（RS）	MOL011753	5-O-Methylvisamminol	Gamma-aminobutyric-acid receptor subunit beta-2	P47870	GABRB2	0.036
Radix Saposhnikoviae（RS）	MOL011753	5-O-Methylvisamminol	Gamma-aminobutyric-acid receptor subunit alpha-4	P48169	GABRA4	0.036
Radix Saposhnikoviae（RS）	MOL011753	5-O-Methylvisamminol	Tubulin beta-2C chain	P68371	TUBB4B	0.036
Radix Saposhnikoviae（RS）	MOL011753	5-O-Methylvisamminol	Gamma-aminobutyric acid receptor subunit epsilon	P78334	GABRE	0.036
Radix Saposhnikoviae（RS）	MOL011753	5-O-Methylvisamminol	Tubulin alpha-3 chain	Q71U36	TUBA1A	0.036
Radix Saposhnikoviae（RS）	MOL011753	5-O-Methylvisamminol	Gamma-aminobutyric acid receptor subunit gamma-1	Q8N1C3	GABRG1	0.036
Radix Saposhnikoviae（RS）	MOL011753	5-O-Methylvisamminol	Gamma-aminobutyric acid receptor subunit gamma-3	Q99928	GABRG3	0.036

Radix Saposhnikoviae（RS）	MOL011753	5-O-Methylvisamminol	Gamma-aminobutyric acid receptor subunit theta	Q9UN88	GABRQ	0.036
Radix Saposhnikoviae（RS）	MOL011753	5-O-Methylvisamminol	Sterol O-acyltransferase 2	O75908	SOAT2	0.037
Radix Saposhnikoviae（RS）	MOL011753	5-O-Methylvisamminol	Keratin, type II cytoskeletal 7	P08729	KRT7	0.037
Radix Saposhnikoviae（RS）	MOL011753	5-O-Methylvisamminol	Alcohol dehydrogenase [NADP+]	P14550	AKR1A1	0.037
Radix Saposhnikoviae（RS）	MOL011753	5-O-Methylvisamminol	Platelet glycoprotein IX	P14770	GP9	0.037
Radix Saposhnikoviae（RS）	MOL011753	5-O-Methylvisamminol	Aldose reductase	P15121	AKR1B1	0.037
Radix Saposhnikoviae（RS）	MOL011753	5-O-Methylvisamminol	Sterol O-acyltransferase 1	P35610	SOAT1	0.037
Radix Saposhnikoviae（RS）	MOL011753	5-O-Methylvisamminol	Mitogen-activated protein kinase 8	P45983	MAPK8	0.037
Radix Saposhnikoviae（RS）	MOL011753	5-O-Methylvisamminol	Mitogen-activated protein kinase 10	P53779	MAPK10	0.037
Radix Saposhnikoviae（RS）	MOL011753	5-O-Methylvisamminol	Histone deacetylase 8	Q9BY41	HDAC8	0.037
Radix Saposhnikoviae（RS）	MOL011753	5-O-Methylvisamminol	Tryptophanyl-tRNA synthetase, mitochondrial	Q9UGM6	WARS2	0.037
Radix Saposhnikoviae（RS）	MOL011753	5-O-Methylvisamminol	Lipoic acid synthetase, mitochondrial	O43766	LIAS	0.038
Radix Saposhnikoviae（RS）	MOL011753	5-O-Methylvisamminol	Peptidyl-prolyl cis-trans isomerase, mitochondrial	P30405	PPIF	0.038
Radix Saposhnikoviae（RS）	MOL011753	5-O-Methylvisamminol	Methionine aminopeptidase 1	P53582	METAP1	0.038
Radix Saposhnikoviae（RS）	MOL011753	5-O-Methylvisamminol	S-phase kinase-associated protein 1	P63208	SKP1	0.038
Radix Saposhnikoviae（RS）	MOL011753	5-O-Methylvisamminol	Lipoyltransferase 1, mitochondrial	Q9Y234	LIPT1	0.038
Radix Saposhnikoviae（RS）	MOL011753	5-O-Methylvisamminol	Phospholipase A2, membrane associated	P14555	PLA2G2A	0.039
Radix Saposhnikoviae（RS）	MOL011753	5-O-Methylvisamminol	Lactoylglutathione lyase	Q04760	GLO1	0.039
Radix Saposhnikoviae（RS）	MOL011753	5-O-Methylvisamminol	Prostaglandin reductase 2	Q8N8N7	PTGR2	0.039
Radix Saposhnikoviae（RS）	MOL011753	5-O-Methylvisamminol	D-HSCDK2	O75100	CA11	0.041
Radix Saposhnikoviae（RS）	MOL011753	5-O-Methylvisamminol	Cell division control protein 2 homolog	P06493	CDK1	0.041
Radix Saposhnikoviae（RS）	MOL011753	5-O-Methylvisamminol	Cell division protein kinase 5	Q00535	CDK5	0.041
Radix Saposhnikoviae（RS）	MOL011753	5-O-Methylvisamminol	Glutamate [NMDA] receptor subunit epsilon-1	Q12879	GRIN2A	0.041
Radix Saposhnikoviae（RS）	MOL011753	5-O-Methylvisamminol	Glutamate [NMDA] receptor subunit epsilon-2	Q13224	GRIN2B	0.041
Radix Saposhnikoviae（RS）	MOL011753	5-O-Methylvisamminol	Sodium channel protein type 5 subunit alpha	Q14524	SCN5A	0.041
Radix Saposhnikoviae（RS）	MOL011753	5-O-Methylvisamminol	3 beta-hydroxysteroid dehydrogenase/Delta 5-->4-isomerase type II	P26439	HSD3B2	0.042
Radix Saposhnikoviae（RS）	MOL011753	5-O-Methylvisamminol	Corticosteroid 11-beta-dehydrogenase isozyme 1	P28845	HSD11B1	0.042
Radix Saposhnikoviae（RS）	MOL011753	5-O-Methylvisamminol	Gamma-aminobutyric acid receptor subunit delta	O14764	GABRD	0.043
Radix Saposhnikoviae（RS）	MOL011753	5-O-Methylvisamminol	Gamma-aminobutyric-acid receptor subunit beta-1	P18505	GABRB1	0.043
Radix Saposhnikoviae（RS）	MOL011753	5-O-Methylvisamminol	Gamma-aminobutyric-acid receptor subunit alpha-5	P31644	GABRA5	0.043
Radix Saposhnikoviae（RS）	MOL011753	5-O-Methylvisamminol	Tripartite motif-containing protein 13	O60858	TRIM13	0.045
Radix Saposhnikoviae（RS）	MOL011753	5-O-Methylvisamminol	Beta-2 adrenergic receptor	P07550	ADRB2	0.045
Radix Saposhnikoviae（RS）	MOL011753	5-O-Methylvisamminol	Beta-1 adrenergic receptor	P08588	ADRB1	0.045
Radix Saposhnikoviae（RS）	MOL011753	5-O-Methylvisamminol	Solute carrier family 12 member 1	Q13621	SLC12A1	0.045
Radix Saposhnikoviae（RS）	MOL011753	5-O-Methylvisamminol	5-hydroxytryptamine 1B receptor	P28222	HTR1B	0.046
Radix Saposhnikoviae（RS）	MOL011753	5-O-Methylvisamminol	3-oxo-5-alpha-steroid 4-dehydrogenase 1	P18405	SRD5A1	0.047
Radix Saposhnikoviae（RS）	MOL011753	5-O-Methylvisamminol	Prolactin receptor	P16471	PRLR	0.048
Radix Saposhnikoviae（RS）	MOL011753	5-O-Methylvisamminol	Nitric-oxide synthase, brain	P29475	NOS1	0.048
Radix Saposhnikoviae（RS）	MOL011753	5-O-Methylvisamminol	Sodium-dependent dopamine transporter	Q01959	SLC6A3	0.048
Radix Saposhnikoviae（RS）	MOL011753	5-O-Methylvisamminol	Glycogen synthase kinase-3 beta	P49841	GSK3B	0.049
Radix Saposhnikoviae（RS）	MOL011753	5-O-Methylvisamminol	Bile salt sulfotransferase	Q06520	SULT2A1	0.054
Radix Saposhnikoviae（RS）	MOL011753	5-O-Methylvisamminol	Proto-oncogene tyrosine-protein kinase LCK	P06239	LCK	0.055
Radix Saposhnikoviae（RS）	MOL011753	5-O-Methylvisamminol	MAP kinase-activated protein kinase 2	P49137	MAPKAPK2	0.056
Radix Saposhnikoviae（RS）	MOL011753	5-O-Methylvisamminol	Carbonic anhydrase 4	P22748	CA4	0.057
Radix Saposhnikoviae（RS）	MOL011753	5-O-Methylvisamminol	Death-associated protein kinase 3	O43293	DAPK3	0.058
Radix Saposhnikoviae（RS）	MOL011753	5-O-Methylvisamminol	Tyrosine-protein kinase JAK2	O60674	JAK2	0.058
Radix Saposhnikoviae（RS）	MOL011753	5-O-Methylvisamminol	Tyrosine-protein kinase JAK1	P23458	JAK1	0.058

Radix Saposhnikoviae（RS）	MOL011753	5-O-Methylvisamminol	Tyrosine-protein kinase JAK3	P52333	JAK3	0.058
Radix Saposhnikoviae（RS）	MOL011753	5-O-Methylvisamminol	Glutamate [NMDA] receptor subunit 3A	Q8TCU5	GRIN3A	0.058
Radix Saposhnikoviae（RS）	MOL011753	5-O-Methylvisamminol	Gamma-aminobutyric-acid receptor subunit alpha-1	P14867	GABRA1	0.059
Radix Saposhnikoviae（RS）	MOL011753	5-O-Methylvisamminol	5-hydroxytryptamine 3 receptor	P46098	HTR3A	0.059
Radix Saposhnikoviae（RS）	MOL011753	5-O-Methylvisamminol	Gamma-aminobutyric-acid receptor subunit alpha-2	P47869	GABRA2	0.059
Radix Saposhnikoviae（RS）	MOL011753	5-O-Methylvisamminol	Alpha-2C adrenergic receptor	P18825	ADRA2C	0.063
Radix Saposhnikoviae（RS）	MOL011753	5-O-Methylvisamminol	Muscarinic acetylcholine receptor M3	P20309	CHRM3	0.063
Radix Saposhnikoviae（RS）	MOL011753	5-O-Methylvisamminol	D(4) dopamine receptor	P21917	DRD4	0.063
Radix Saposhnikoviae（RS）	MOL011753	5-O-Methylvisamminol	5-hydroxytryptamine 1D receptor	P28221	HTR1D	0.063
Radix Saposhnikoviae（RS）	MOL011753	5-O-Methylvisamminol	5-hydroxytryptamine 2B receptor	P41595	HTR2B	0.063
Radix Saposhnikoviae（RS）	MOL011753	5-O-Methylvisamminol	Muscarinic acetylcholine receptor M1	P11229	CHRM1	0.064
Radix Saposhnikoviae（RS）	MOL011753	5-O-Methylvisamminol	Hepatocyte growth factor receptor	P08581	MET	0.068
Radix Saposhnikoviae（RS）	MOL011753	5-O-Methylvisamminol	Tyrosyl-tRNA synthetase, cytoplasmic	P54577	YARS	0.068
Radix Saposhnikoviae（RS）	MOL011753	5-O-Methylvisamminol	Casein kinase II subunit alpha	P68400	CSNK2A1	0.068
Radix Saposhnikoviae（RS）	MOL011753	5-O-Methylvisamminol	Neuropeptide Y	P01303	NPY	0.069
Radix Saposhnikoviae（RS）	MOL011753	5-O-Methylvisamminol	Ig kappa chain V-II region RPMI 6410	P06310	IGKV2-30	0.069
Radix Saposhnikoviae（RS）	MOL011753	5-O-Methylvisamminol	Low molecular weight phosphotyrosine protein phosphatase	P24666	ACP1	0.07
Radix Saposhnikoviae（RS）	MOL011753	5-O-Methylvisamminol	Lysozyme C	P61626	LYZ	0.07
Radix Saposhnikoviae（RS）	MOL011753	5-O-Methylvisamminol	cAMP-dependent protein kinase inhibitor alpha	P61925	PKIA	0.07
Radix Saposhnikoviae（RS）	MOL011753	5-O-Methylvisamminol	Nuclear receptor coactivator 1	Q15788	NCOA1	0.07
Radix Saposhnikoviae（RS）	MOL011753	5-O-Methylvisamminol	Gamma-aminobutyric acid type B receptor, subunit 1	Q9UBS5	GABBR1	0.07
Radix Saposhnikoviae（RS）	MOL011753	5-O-Methylvisamminol	Dihydrofolate reductase	P00374	DHFR	0.072
Radix Saposhnikoviae（RS）	MOL011753	5-O-Methylvisamminol	Melatonin receptor type 1A	P48039	MTNR1A	0.072
Radix Saposhnikoviae（RS）	MOL011753	5-O-Methylvisamminol	Activated CDC42 kinase 1	Q07912	TNK2	0.072
Radix Saposhnikoviae（RS）	MOL011753	5-O-Methylvisamminol	Nuclear receptor coactivator 2	Q15596	NCOA2	0.072
Radix Saposhnikoviae（RS）	MOL011753	5-O-Methylvisamminol	Membrane copper amine oxidase	Q16853	AOC3	0.072
Radix Saposhnikoviae（RS）	MOL011753	5-O-Methylvisamminol	Cytohesin-2	Q99418	CYTH2	0.072
Radix Saposhnikoviae（RS）	MOL011753	5-O-Methylvisamminol	Aldo-keto reductase family 1 member C1	Q04828	AKR1C1	0.073
Radix Saposhnikoviae（RS）	MOL011753	5-O-Methylvisamminol	Prothrombin	P00734	F2	0.074
Radix Saposhnikoviae（RS）	MOL011753	5-O-Methylvisamminol	Endothelin-1 receptor	P25101	EDNRA	0.074
Radix Saposhnikoviae（RS）	MOL011753	5-O-Methylvisamminol	Phosphatidylinositol-4,5-bisphosphate 3-kinase catalytic subunit gamma isoform	P48736	PIK3CG	0.074
Radix Saposhnikoviae（RS）	MOL011753	5-O-Methylvisamminol	Tubulin beta-1 chain	Q9H4B7	TUBB1	0.074
Radix Saposhnikoviae（RS）	MOL011753	5-O-Methylvisamminol	Nuclear receptor coactivator 5	Q9HCD5	NCOA5	0.074
Radix Saposhnikoviae（RS）	MOL011753	5-O-Methylvisamminol	Glyceraldehyde-3-phosphate dehydrogenase	P04406	GAPDH	0.075
Radix Saposhnikoviae（RS）	MOL011753	5-O-Methylvisamminol	Toll-like receptor 7	Q9NYK1	TLR7	0.075
Radix Saposhnikoviae（RS）	MOL011753	5-O-Methylvisamminol	Lactotransferrin	P02788	LTF	0.076
Radix Saposhnikoviae（RS）	MOL011753	5-O-Methylvisamminol	Tyrosine-protein phosphatase non-receptor type 1	P18031	PTPN1	0.076
Radix Saposhnikoviae（RS）	MOL011753	5-O-Methylvisamminol	Thymidylate synthase	P04818	TYMS	0.077
Radix Saposhnikoviae（RS）	MOL011753	5-O-Methylvisamminol	S-methyl-5-thioadenosine phosphorylase	Q13126	MTAP	0.077
Radix Saposhnikoviae（RS）	MOL011753	5-O-Methylvisamminol	DNA polymerase kappa	Q9UBT6	POLK	0.077
Radix Saposhnikoviae（RS）	MOL011753	5-O-Methylvisamminol	Cannabinoid receptor 2	P34972	CNR2	0.078
Radix Saposhnikoviae（RS）	MOL011753	5-O-Methylvisamminol	Triosephosphate isomerase	P60174	TPI1	0.078
Radix Saposhnikoviae（RS）	MOL011753	5-O-Methylvisamminol	C-jun-amino-terminal kinase-interacting protein 1	Q9UQF2	MAPK8IP1	0.078
Radix Saposhnikoviae（RS）	MOL011753	5-O-Methylvisamminol	Glucocorticoid receptor	P04150	NR3C1	0.079
Radix Saposhnikoviae（RS）	MOL011753	5-O-Methylvisamminol	Hydroxyacid oxidase 1	Q9UJM8	HAO1	0.079
Radix Saposhnikoviae（RS）	MOL011753	5-O-Methylvisamminol	3 beta-hydroxysteroid dehydrogenase/Delta 5-->4-isomerase type I	P14060	HSD3B1	0.08
Radix Saposhnikoviae（RS）	MOL011753	5-O-Methylvisamminol	Alpha-2B adrenergic receptor	P18089	ADRA2B	0.08

Radix Saposhnikoviae（RS）	MOL011753	5-O-Methylvisamminol	Group IIE secretory phospholipase A2	Q9NZK7	PLA2G2E	0.081
Radix Saposhnikoviae（RS）	MOL011753	5-O-Methylvisamminol	Alpha-1A adrenergic receptor	P35348	ADRA1A	0.082
Radix Saposhnikoviae（RS）	MOL011753	5-O-Methylvisamminol	Inhibitor of nuclear factor kappa-B kinase subunit alpha	O15111	CHUK	0.083
Radix Saposhnikoviae（RS）	MOL011753	5-O-Methylvisamminol	Alpha-2A adrenergic receptor	P08913	ADRA2A	0.083
Radix Saposhnikoviae（RS）	MOL011753	5-O-Methylvisamminol	Arachidonate 5-lipoxygenase	P09917	ALOX5	0.083
Radix Saposhnikoviae（RS）	MOL011753	5-O-Methylvisamminol	Peroxisome proliferator-activated receptor gamma	P37231	PPARG	0.083
Radix Saposhnikoviae（RS）	MOL011753	5-O-Methylvisamminol	Thiamin pyrophosphokinase 1	Q9H3S4	TPK1	0.083
Radix Saposhnikoviae（RS）	MOL011753	5-O-Methylvisamminol	Tyrosine-protein kinase HCK	P08631	HCK	0.085
Radix Saposhnikoviae（RS）	MOL011753	5-O-Methylvisamminol	Cannabinoid receptor 1	P21554	CNR1	0.086
Radix Saposhnikoviae（RS）	MOL011753	5-O-Methylvisamminol	cAMP-specific 3',5'-cyclic phosphodiesterase 4A	P27815	PDE4A	0.086
Radix Saposhnikoviae（RS）	MOL011753	5-O-Methylvisamminol	Phospholipase A2	P04054	PLA2G1B	0.09
Radix Saposhnikoviae（RS）	MOL011753	5-O-Methylvisamminol	Sodium-dependent serotonin transporter	P31645	SLC6A4	0.092
Radix Saposhnikoviae（RS）	MOL011753	5-O-Methylvisamminol	5-hydroxytryptamine 1A receptor	P08908	HTR1A	0.093
Radix Saposhnikoviae（RS）	MOL011753	5-O-Methylvisamminol	Trypsin-1	P07477	PRSS1	0.094
Radix Saposhnikoviae（RS）	MOL011753	5-O-Methylvisamminol	Ribosyldihydronicotinamide dehydrogenase [quinone]	P16083	NQO2	0.095
Radix Saposhnikoviae（RS）	MOL011753	5-O-Methylvisamminol	D(1B) dopamine receptor	P21918	DRD5	0.097
Radix Saposhnikoviae（RS）	MOL011753	5-O-Methylvisamminol	D(3) dopamine receptor	P35462	DRD3	0.097
Radix Saposhnikoviae（RS）	MOL011753	5-O-Methylvisamminol	Estradiol 17-beta-dehydrogenase 1	P14061	HSD17B1	0.098
Radix Saposhnikoviae（RS）	MOL011753	5-O-Methylvisamminol	Androgen receptor	P10275	AR	0.1
Radix Saposhnikoviae（RS）	MOL011753	5-O-Methylvisamminol	Nitric-oxide synthase, endothelial	P29474	NOS3	0.101
Radix Saposhnikoviae（RS）	MOL011753	5-O-Methylvisamminol	5-hydroxytryptamine 2C receptor	P28335	HTR2C	0.105
Radix Saposhnikoviae（RS）	MOL011753	5-O-Methylvisamminol	Sodium-dependent noradrenaline transporter	P23975	SLC6A2	0.111
Radix Saposhnikoviae（RS）	MOL011753	5-O-Methylvisamminol	5-hydroxytryptamine 2A receptor	P28223	HTR2A	0.118
Radix Saposhnikoviae（RS）	MOL011753	5-O-Methylvisamminol	Mitogen-activated protein kinase 14	Q16539	MAPK14	0.144
Radix Saposhnikoviae（RS）	MOL011753	5-O-Methylvisamminol	Carbonic anhydrase 1	P00915	CA1	0.156
Radix Saposhnikoviae（RS）	MOL011753	5-O-Methylvisamminol	Carbonic anhydrase 2	P00918	CA2	0.156
Radix Saposhnikoviae（RS）	MOL011753	5-O-Methylvisamminol	Cytochrome P450 11B1, mitochondrial	P15538	CYP11B1	0.159
Radix Saposhnikoviae（RS）	MOL011753	5-O-Methylvisamminol	Cell division protein kinase 2	P24941	CDK2	0.162
Radix Saposhnikoviae（RS）	MOL011753	5-O-Methylvisamminol	Delta-type opioid receptor	P41143	OPRD1	0.166
Radix Saposhnikoviae（RS）	MOL011753	5-O-Methylvisamminol	Nitric oxide synthase, inducible	P35228	NOS2	0.172
Radix Saposhnikoviae（RS）	MOL011753	5-O-Methylvisamminol	D(2) dopamine receptor	P14416	DRD2	0.182
Radix Saposhnikoviae（RS）	MOL011753	5-O-Methylvisamminol	Mineralocorticoid receptor	P08235	NR3C2	0.183
Radix Saposhnikoviae（RS）	MOL011753	5-O-Methylvisamminol	cAMP-dependent protein kinase catalytic subunit alpha	P17612	PRKACA	0.185
Radix Saposhnikoviae（RS）	MOL011753	5-O-Methylvisamminol	D(1A) dopamine receptor	P21728	DRD1	0.199
Radix Saposhnikoviae（RS）	MOL011753	5-O-Methylvisamminol	Cyclin-A2	P20248	CCNA2	0.226
Radix Saposhnikoviae（RS）	MOL011753	5-O-Methylvisamminol	Hemoglobin subunit alpha	P69905	HBA1	0.239
Radix Saposhnikoviae（RS）	MOL011753	5-O-Methylvisamminol	Estrogen receptor beta	Q92731	ESR2	0.293
Radix Saposhnikoviae（RS）	MOL011753	5-O-Methylvisamminol	Kappa-type opioid receptor	P41145	OPRK1	0.306
Radix Saposhnikoviae（RS）	MOL011753	5-O-Methylvisamminol	Prostaglandin G/H synthase 1	P23219	PTGS1	0.379
Radix Saposhnikoviae（RS）	MOL011753	5-O-Methylvisamminol	Mu-type opioid receptor	P35372	OPRM1	0.384
Radix Saposhnikoviae（RS）	MOL011753	5-O-Methylvisamminol	Progesterone receptor	P06401	PGR	0.413
Radix Saposhnikoviae（RS）	MOL011753	5-O-Methylvisamminol	Prostaglandin G/H synthase 2	P35354	PTGS2	0.797
Radix Saposhnikoviae（RS）	MOL011753	5-O-Methylvisamminol	Estrogen receptor	P03372	ESR1	1
Radix Saposhnikoviae（RS）	MOL013077	Decursin	Glutamate receptor 1	P42261	GRIA1	0.001
Radix Saposhnikoviae（RS）	MOL013077	Decursin	Acetyl-CoA carboxylase 2	O00763	ACACB	0.004
Radix Saposhnikoviae（RS）	MOL013077	Decursin	Acetylcholine receptor subunit alpha	P02708	CHRNA1	0.004
Radix Saposhnikoviae（RS）	MOL013077	Decursin	Propionyl-CoA carboxylase alpha chain, mitochondrial	P05165	PCCA	0.004

Radix Saposhnikoviae（RS）	MOL013077	Decursin	Propionyl-CoA carboxylase beta chain, mitochondrial	P05166	PCCB	0.004
Radix Saposhnikoviae（RS）	MOL013077	Decursin	Cholinesterase	P06276	BCHE	0.004
Radix Saposhnikoviae（RS）	MOL013077	Decursin	Acetylcholine receptor subunit gamma	P07510	CHRNG	0.004
Radix Saposhnikoviae（RS）	MOL013077	Decursin	Acetylcholine receptor subunit beta	P11230	CHRNB1	0.004
Radix Saposhnikoviae（RS）	MOL013077	Decursin	Pyruvate carboxylase, mitochondrial	P11498	PC	0.004
Radix Saposhnikoviae（RS）	MOL013077	Decursin	Neuronal acetylcholine receptor subunit beta-2	P17787	CHRNB2	0.004
Radix Saposhnikoviae（RS）	MOL013077	Decursin	Acetylcholinesterase	P22303	ACHE	0.004
Radix Saposhnikoviae（RS）	MOL013077	Decursin	Neuronal acetylcholine receptor subunit alpha-5	P30532	CHRNA5	0.004
Radix Saposhnikoviae（RS）	MOL013077	Decursin	Neuronal acetylcholine receptor subunit beta-4	P30926	CHRNB4	0.004
Radix Saposhnikoviae（RS）	MOL013077	Decursin	Biotin--protein ligase	P50747	HLCS	0.004
Radix Saposhnikoviae（RS）	MOL013077	Decursin	Acetylcholine receptor subunit epsilon	Q04844	CHRNE	0.004
Radix Saposhnikoviae（RS）	MOL013077	Decursin	Neuronal acetylcholine receptor subunit beta-3	Q05901	CHRNB3	0.004
Radix Saposhnikoviae（RS）	MOL013077	Decursin	Acetylcholine receptor subunit delta	Q07001	CHRND	0.004
Radix Saposhnikoviae（RS）	MOL013077	Decursin	Neuronal acetylcholine receptor subunit alpha-6	Q15825	KCNJ8	0.004
Radix Saposhnikoviae（RS）	MOL013077	Decursin	Methylcrotonoyl-CoA carboxylase subunit alpha, mitochondrial	Q96RQ3	MCCC1	0.004
Radix Saposhnikoviae（RS）	MOL013077	Decursin	Neuronal acetylcholine receptor subunit alpha-10	Q9GZZ6	CHRNA10	0.004
Radix Saposhnikoviae（RS）	MOL013077	Decursin	Methylcrotonoyl-CoA carboxylase beta chain, mitochondrial	Q9HCC0	MCCC2	0.004
Radix Saposhnikoviae（RS）	MOL013077	Decursin	Neuronal acetylcholine receptor subunit alpha-9	Q9UGM1	CHRNA9	0.004
Radix Saposhnikoviae（RS）	MOL013077	Decursin	Sodium-dependent multivitamin transporter	Q9Y289	SLC5A6	0.004
Radix Saposhnikoviae（RS）	MOL013077	Decursin	Elongation factor Tu GTP-binding domain-containing protein 1	Q7Z2Z2	EFL1	0.006
Radix Saposhnikoviae（RS）	MOL013077	Decursin	D(2) dopamine receptor	P14416	DRD2	0.009
Radix Saposhnikoviae（RS）	MOL013077	Decursin	Alpha-2B adrenergic receptor	P18089	ADRA2B	0.009
Radix Saposhnikoviae（RS）	MOL013077	Decursin	Alpha-2C adrenergic receptor	P18825	ADRA2C	0.009
Radix Saposhnikoviae（RS）	MOL013077	Decursin	D(1A) dopamine receptor	P21728	DRD1	0.009
Radix Saposhnikoviae（RS）	MOL013077	Decursin	D(4) dopamine receptor	P21917	DRD4	0.009
Radix Saposhnikoviae（RS）	MOL013077	Decursin	D(1B) dopamine receptor	P21918	DRD5	0.009
Radix Saposhnikoviae（RS）	MOL013077	Decursin	Alpha-1B adrenergic receptor	P35368	ADRA1B	0.009
Radix Saposhnikoviae（RS）	MOL013077	Decursin	D(3) dopamine receptor	P35462	DRD3	0.009
Radix Saposhnikoviae（RS）	MOL013077	Decursin	Sodium-dependent dopamine transporter	Q01959	SLC6A3	0.009
Radix Saposhnikoviae（RS）	MOL013077	Decursin	Potassium voltage-gated channel subfamily KQT member 2	O43526	KCNQ2	0.01
Radix Saposhnikoviae（RS）	MOL013077	Decursin	High affinity nerve growth factor receptor	P04629	NTRK1	0.01
Radix Saposhnikoviae（RS）	MOL013077	Decursin	5-hydroxytryptamine 3 receptor	P46098	HTR3A	0.01
Radix Saposhnikoviae（RS）	MOL013077	Decursin	Potassium voltage-gated channel subfamily A member 1	Q09470	KCNA1	0.01
Radix Saposhnikoviae（RS）	MOL013077	Decursin	BDNF/NT-3 growth factors receptor	Q16620	NTRK2	0.01
Radix Saposhnikoviae（RS）	MOL013077	Decursin	Potassium voltage-gated channel subfamily D member 2	Q9NZV8	KCND2	0.01
Radix Saposhnikoviae（RS）	MOL013077	Decursin	Potassium voltage-gated channel subfamily D member 3	Q9UK17	KCND3	0.01
Radix Saposhnikoviae（RS）	MOL013077	Decursin	Opioid receptor, sigma 1	Q5T1J1	SIGMAR1	0.012
Radix Saposhnikoviae（RS）	MOL013077	Decursin	Sigma 1-type opioid receptor	Q99720	SIGMAR1	0.012
Radix Saposhnikoviae（RS）	MOL013077	Decursin	Tripartite motif-containing protein 13	O60858	TRIM13	0.013
Radix Saposhnikoviae（RS）	MOL013077	Decursin	Neuronal acetylcholine receptor subunit alpha-3	P32297	CHRNA3	0.013
Radix Saposhnikoviae（RS）	MOL013077	Decursin	2,4-dienoyl-CoA reductase, mitochondrial	Q16698	DECR1	0.013
Radix Saposhnikoviae（RS）	MOL013077	Decursin	Substance-P receptor	P25103	TACR1	0.015
Radix Saposhnikoviae（RS）	MOL013077	Decursin	Death-associated protein kinase 3	O43293	DAPK3	0.016
Radix Saposhnikoviae（RS）	MOL013077	Decursin	Tyrosine-protein kinase JAK2	O60674	JAK2	0.016
Radix Saposhnikoviae（RS）	MOL013077	Decursin	Gamma-aminobutyric acid receptor subunit gamma-2	P18507	GABRG2	0.016
Radix Saposhnikoviae（RS）	MOL013077	Decursin	Tyrosine-protein kinase JAK1	P23458	JAK1	0.016
Radix Saposhnikoviae（RS）	MOL013077	Decursin	Gamma-aminobutyric-acid receptor subunit beta-3	P28472	GABRB3	0.016

Radix Saposhnikoviae（RS）	MOL013077	Decursin	Translocator protein	P30536	TSPO	0.016
Radix Saposhnikoviae（RS）	MOL013077	Decursin	Gamma-aminobutyric-acid receptor subunit beta-2	P47870	GABRB2	0.016
Radix Saposhnikoviae（RS）	MOL013077	Decursin	Tyrosine-protein kinase JAK3	P52333	JAK3	0.016
Radix Saposhnikoviae（RS）	MOL013077	Decursin	Gamma-aminobutyric acid receptor subunit theta	Q9UN88	GABRQ	0.016
Radix Saposhnikoviae（RS）	MOL013077	Decursin	Glutamate receptor, ionotropic kainate 2	Q13002	GRIK2	0.017
Radix Saposhnikoviae（RS）	MOL013077	Decursin	Serine/threonine-protein phosphatase 2A 65 kDa regulatory subunit A alpha isoform	P30153	PPP2R1A	0.019
Radix Saposhnikoviae（RS）	MOL013077	Decursin	Serine/threonine-protein phosphatase PP1-alpha catalytic subunit	P62136	PPP1CA	0.019
Radix Saposhnikoviae（RS）	MOL013077	Decursin	Serine/threonine-protein phosphatase 2A catalytic subunit alpha isoform	P67775	PPP2CA	0.019
Radix Saposhnikoviae（RS）	MOL013077	Decursin	Serine/threonine-protein phosphatase 2A 56 kDa regulatory subunit gamma isoform	Q13362	PPP2R5C	0.019
Radix Saposhnikoviae（RS）	MOL013077	Decursin	Neuronal acetylcholine receptor subunit alpha-2	Q15822	CHRNA2	0.023
Radix Saposhnikoviae（RS）	MOL013077	Decursin	Gamma-aminobutyric acid receptor subunit rho-3	A8MPY1	GABRR3	0.024
Radix Saposhnikoviae（RS）	MOL013077	Decursin	Gamma-aminobutyric acid receptor subunit pi	O00591	GABRP	0.024
Radix Saposhnikoviae（RS）	MOL013077	Decursin	Gamma-aminobutyric-acid receptor subunit rho-1	P24046	GABRR1	0.024
Radix Saposhnikoviae（RS）	MOL013077	Decursin	Gamma-aminobutyric acid receptor subunit rho-2	P28476	GABRR2	0.024
Radix Saposhnikoviae（RS）	MOL013077	Decursin	Gamma-aminobutyric acid receptor subunit epsilon	P78334	GABRE	0.024
Radix Saposhnikoviae（RS）	MOL013077	Decursin	Gamma-aminobutyric acid receptor subunit gamma-1	Q8N1C3	GABRG1	0.024
Radix Saposhnikoviae（RS）	MOL013077	Decursin	Gamma-aminobutyric acid receptor subunit gamma-3	Q99928	GABRG3	0.024
Radix Saposhnikoviae（RS）	MOL013077	Decursin	Gamma-aminobutyric-acid receptor subunit alpha-6	Q16445	GABRA6	0.025
Radix Saposhnikoviae（RS）	MOL013077	Decursin	5-hydroxytryptamine 1A receptor	P08908	HTR1A	0.026
Radix Saposhnikoviae（RS）	MOL013077	Decursin	Alpha-1A adrenergic receptor	P35348	ADRA1A	0.026
Radix Saposhnikoviae（RS）	MOL013077	Decursin	Neuronal acetylcholine receptor subunit alpha-4	P43681	CHRNA4	0.026
Radix Saposhnikoviae（RS）	MOL013077	Decursin	Troponin C, slow skeletal and cardiac muscles	P63316	TNNC1	0.026
Radix Saposhnikoviae（RS）	MOL013077	Decursin	Bile salt sulfotransferase	Q06520	SULT2A1	0.026
Radix Saposhnikoviae（RS）	MOL013077	Decursin	ATP-sensitive inward rectifier potassium channel 11	Q14654	KCNJ11	0.026
Radix Saposhnikoviae（RS）	MOL013077	Decursin	ATP-sensitive inward rectifier potassium channel 8	Q15842	KCNJ8	0.026
Radix Saposhnikoviae（RS）	MOL013077	Decursin	Dehydrogenase/reductase SDR family member 8	Q8NBQ5	HSD17B11	0.026
Radix Saposhnikoviae（RS）	MOL013077	Decursin	Ig gamma-2 chain C region	P01859	IGHG2	0.031
Radix Saposhnikoviae（RS）	MOL013077	Decursin	Gamma-aminobutyric acid receptor subunit delta	O14764	GABRD	0.032
Radix Saposhnikoviae（RS）	MOL013077	Decursin	Gamma-aminobutyric-acid receptor subunit beta-1	P18505	GABRB1	0.032
Radix Saposhnikoviae（RS）	MOL013077	Decursin	Gamma-aminobutyric-acid receptor subunit alpha-4	P48169	GABRA4	0.033
Radix Saposhnikoviae（RS）	MOL013077	Decursin	Glutamate [NMDA] receptor subunit 3A	Q8TCU5	GRIN3A	0.034
Radix Saposhnikoviae（RS）	MOL013077	Decursin	Succinate dehydrogenase [ubiquinone] cytochrome b small subunit, mitochondrial	O14521	SDHD	0.037
Radix Saposhnikoviae（RS）	MOL013077	Decursin	Proto-oncogene tyrosine-protein kinase LCK	P06239	LCK	0.037
Radix Saposhnikoviae（RS）	MOL013077	Decursin	Tyrosine-protein kinase Lyn	P07948	LYN	0.037
Radix Saposhnikoviae（RS）	MOL013077	Decursin	Nitric-oxide synthase, endothelial	P29474	NOS3	0.037
Radix Saposhnikoviae（RS）	MOL013077	Decursin	Nitric-oxide synthase, brain	P29475	NOS1	0.037
Radix Saposhnikoviae（RS）	MOL013077	Decursin	Neuronal acetylcholine receptor subunit alpha-7	P36544	CHRNA7	0.037
Radix Saposhnikoviae（RS）	MOL013077	Decursin	Guanine nucleotide-binding protein G(s) subunit alpha isoforms short	P63092	GNAS	0.037
Radix Saposhnikoviae（RS）	MOL013077	Decursin	S-phase kinase-associated protein 1	P63208	SKP1	0.037
Radix Saposhnikoviae（RS）	MOL013077	Decursin	Adenylate cyclase type 2	Q08462	ADCY2	0.037
Radix Saposhnikoviae（RS）	MOL013077	Decursin	Group IIE secretory phospholipase A2	Q9NZK7	PLA2G2E	0.037
Radix Saposhnikoviae（RS）	MOL013077	Decursin	Methionine aminopeptidase 1	P53582	METAP1	0.04
Radix Saposhnikoviae（RS）	MOL013077	Decursin	Tubulin beta-2C chain	P68371	TUBB4B	0.04
Radix Saposhnikoviae（RS）	MOL013077	Decursin	Tubulin alpha-3 chain	Q71U36	TUBA1A	0.04
Radix Saposhnikoviae（RS）	MOL013077	Decursin	Potassium channel subfamily K member 1	O00180	KCNK1	0.041
Radix Saposhnikoviae（RS）	MOL013077	Decursin	Macrophage migration inhibitory factor	P14174	MIF	0.041
Radix Saposhnikoviae（RS）	MOL013077	Decursin	Potassium channel subfamily K member 6	Q9Y257	KCNK6	0.041

Radix Saposhnikoviae（RS）	MOL013077	Decursin	Tryptophanyl-tRNA synthetase, mitochondrial	Q9UGM6	WARS2	0.042
Radix Saposhnikoviae（RS）	MOL013077	Decursin	Alpha-1D adrenergic receptor	P25100	ADRA1D	0.043
Radix Saposhnikoviae（RS）	MOL013077	Decursin	5-hydroxytryptamine 2C receptor	P28335	HTR2C	0.043
Radix Saposhnikoviae（RS）	MOL013077	Decursin	Peptidyl-prolyl cis-trans isomerase, mitochondrial	P30405	PPIF	0.043
Radix Saposhnikoviae（RS）	MOL013077	Decursin	Sodium-dependent serotonin transporter	P31645	SLC6A4	0.043
Radix Saposhnikoviae（RS）	MOL013077	Decursin	Lipoic acid synthetase, mitochondrial	O43766	LIAS	0.044
Radix Saposhnikoviae（RS）	MOL013077	Decursin	Alpha-2A adrenergic receptor	P08913	ADRA2A	0.044
Radix Saposhnikoviae（RS）	MOL013077	Decursin	Lipoyltransferase 1, mitochondrial	Q9Y234	LIPT1	0.044
Radix Saposhnikoviae（RS）	MOL013077	Decursin	Tyrosyl-tRNA synthetase, cytoplasmic	P54577	YARS	0.046
Radix Saposhnikoviae（RS）	MOL013077	Decursin	Peptidyl-prolyl cis-trans isomerase NIMA-interacting 1	Q13526	PIN1	0.046
Radix Saposhnikoviae（RS）	MOL013077	Decursin	Platelet glycoprotein IX	P14770	GP9	0.048
Radix Saposhnikoviae（RS）	MOL013077	Decursin	Gamma-aminobutyric-acid receptor subunit alpha-5	P31644	GABRA5	0.048
Radix Saposhnikoviae（RS）	MOL013077	Decursin	Gamma-aminobutyric-acid receptor subunit alpha-3	P34903	GABRA3	0.048
Radix Saposhnikoviae（RS）	MOL013077	Decursin	Gamma-aminobutyric-acid receptor subunit alpha-2	P47869	GABRA2	0.048
Radix Saposhnikoviae（RS）	MOL013077	Decursin	Delta-type opioid receptor	P41143	OPRD1	0.05
Radix Saposhnikoviae（RS）	MOL013077	Decursin	Retinoic acid receptor RXR-alpha	P19793	RXRA	0.053
Radix Saposhnikoviae（RS）	MOL013077	Decursin	Nuclear receptor subfamily 1 group I member 3	Q14994	NR1I3	0.053
Radix Saposhnikoviae（RS）	MOL013077	Decursin	3-oxo-5-alpha-steroid 4-dehydrogenase 2	P31213	SRD5A2	0.055
Radix Saposhnikoviae（RS）	MOL013077	Decursin	Gamma-aminobutyric-acid receptor subunit alpha-1	P14867	GABRA1	0.056
Radix Saposhnikoviae（RS）	MOL013077	Decursin	cGMP-inhibited 3',5'-cyclic phosphodiesterase A	Q14432	PDE3A	0.056
Radix Saposhnikoviae（RS）	MOL013077	Decursin	Hydroxyacid oxidase 1	Q9UJM8	HAO1	0.056
Radix Saposhnikoviae（RS）	MOL013077	Decursin	Carbonic anhydrase 4	P22748	CA4	0.057
Radix Saposhnikoviae（RS）	MOL013077	Decursin	Corticosteroid 11-beta-dehydrogenase isozyme 1	P28845	HSD11B1	0.057
Radix Saposhnikoviae（RS）	MOL013077	Decursin	Muscarinic acetylcholine receptor M5	P08912	CHRM5	0.059
Radix Saposhnikoviae（RS）	MOL013077	Decursin	3 beta-hydroxysteroid dehydrogenase/Delta 5-->4-isomerase type I	P14060	HSD3B1	0.06
Radix Saposhnikoviae（RS）	MOL013077	Decursin	3 beta-hydroxysteroid dehydrogenase/Delta 5-->4-isomerase type II	P26439	HSD3B2	0.06
Radix Saposhnikoviae（RS）	MOL013077	Decursin	RAC-beta serine/threonine-protein kinase	P31751	AKT2	0.061
Radix Saposhnikoviae（RS）	MOL013077	Decursin	cAMP-dependent protein kinase inhibitor alpha	P61925	PKIA	0.061
Radix Saposhnikoviae（RS）	MOL013077	Decursin	Ig kappa chain C region	P01834	IGKC	0.07
Radix Saposhnikoviae（RS）	MOL013077	Decursin	Ig gamma-1 chain C region	P01857	IGHG1	0.07
Radix Saposhnikoviae（RS）	MOL013077	Decursin	Sodium-dependent noradrenaline transporter	P23975	SLC6A2	0.076
Radix Saposhnikoviae（RS）	MOL013077	Decursin	Prostaglandin G/H synthase 1	P23219	PTGS1	0.081
Radix Saposhnikoviae（RS）	MOL013077	Decursin	NADPH oxidase organizer 1	Q8NFA2	NOXO1	0.083
Radix Saposhnikoviae（RS）	MOL013077	Decursin	Sodium/potassium-transporting ATPase alpha-1 chain	P05023	ATP1A1	0.084
Radix Saposhnikoviae（RS）	MOL013077	Decursin	MAP kinase-activated protein kinase 2	P49137	MAPKAPK2	0.084
Radix Saposhnikoviae（RS）	MOL013077	Decursin	Cytochrome P450 11B1, mitochondrial	P15538	CYP11B1	0.086
Radix Saposhnikoviae（RS）	MOL013077	Decursin	Nuclear receptor coactivator 2	Q15596	NCOA2	0.086
Radix Saposhnikoviae（RS）	MOL013077	Decursin	Cytochrome P450 19A1	P11511	CYP19A1	0.087
Radix Saposhnikoviae（RS）	MOL013077	Decursin	Lysozyme C	P61626	LYZ	0.087
Radix Saposhnikoviae（RS）	MOL013077	Decursin	Glucocorticoid receptor	P04150	NR3C1	0.089
Radix Saposhnikoviae（RS）	MOL013077	Decursin	Dual specificity protein kinase CLK1	P49759	CLK1	0.089
Radix Saposhnikoviae（RS）	MOL013077	Decursin	Triosephosphate isomerase	P60174	TPI1	0.09
Radix Saposhnikoviae（RS）	MOL013077	Decursin	Sodium channel protein type 5 subunit alpha	Q14524	SCN5A	0.09
Radix Saposhnikoviae（RS）	MOL013077	Decursin	Mitogen-activated protein kinase 14	Q16539	MAPK14	0.091
Radix Saposhnikoviae（RS）	MOL013077	Decursin	Muscarinic acetylcholine receptor M2	P08172	CHRM2	0.092
Radix Saposhnikoviae（RS）	MOL013077	Decursin	Muscarinic acetylcholine receptor M4	P08173	CHRM4	0.092
Radix Saposhnikoviae（RS）	MOL013077	Decursin	Tyrosine-protein phosphatase non-receptor type 1	P18031	PTPN1	0.092

Radix Saposhnikoviae（RS）	MOL013077	Decursin	Histamine H1 receptor	P35367	HRH1	0.092
Radix Saposhnikoviae（RS）	MOL013077	Decursin	Nuclear receptor coactivator 5	Q9HCD5	NCOA5	0.092
Radix Saposhnikoviae（RS）	MOL013077	Decursin	Tyrosine-protein kinase HCK	P08631	HCK	0.093
Radix Saposhnikoviae（RS）	MOL013077	Decursin	5-hydroxytryptamine 2A receptor	P28223	HTR2A	0.093
Radix Saposhnikoviae（RS）	MOL013077	Decursin	Neutrophil gelatinase-associated lipocalin	P80188	LCN2	0.093
Radix Saposhnikoviae（RS）	MOL013077	Decursin	DNA polymerase kappa	Q9UBT6	POLK	0.094
Radix Saposhnikoviae（RS）	MOL013077	Decursin	Mu-type opioid receptor	P35372	OPRM1	0.095
Radix Saposhnikoviae（RS）	MOL013077	Decursin	Gamma-aminobutyric acid type B receptor, subunit 1	Q9UBS5	GABBR1	0.095
Radix Saposhnikoviae（RS）	MOL013077	Decursin	Aldo-keto reductase family 1 member B10	O60218	AKR1B10	0.099
Radix Saposhnikoviae（RS）	MOL013077	Decursin	Cannabinoid receptor 1	P21554	CNR1	0.099
Radix Saposhnikoviae（RS）	MOL013077	Decursin	Phosphatidylinositol-4,5-bisphosphate 3-kinase catalytic subunit gamma isoform	P48736	PIK3CG	0.102
Radix Saposhnikoviae（RS）	MOL013077	Decursin	Hemoglobin subunit alpha	P69905	HBA1	0.103
Radix Saposhnikoviae（RS）	MOL013077	Decursin	Muscarinic acetylcholine receptor M1	P11229	CHRM1	0.109
Radix Saposhnikoviae（RS）	MOL013077	Decursin	Muscarinic acetylcholine receptor M3	P20309	CHRM3	0.109
Radix Saposhnikoviae（RS）	MOL013077	Decursin	Kappa-type opioid receptor	P41145	OPRK1	0.109
Radix Saposhnikoviae（RS）	MOL013077	Decursin	cAMP-specific 3',5'-cyclic phosphodiesterase 4B	Q07343	PDE4B	0.12
Radix Saposhnikoviae（RS）	MOL013077	Decursin	Carbonic anhydrase 2	P00918	CA2	0.121
Radix Saposhnikoviae（RS）	MOL013077	Decursin	3-oxo-5-alpha-steroid 4-dehydrogenase 1	P18405	SRD5A1	0.121
Radix Saposhnikoviae（RS）	MOL013077	Decursin	Androgen receptor	P10275	AR	0.123
Radix Saposhnikoviae（RS）	MOL013077	Decursin	Prothrombin	P00734	F2	0.139
Radix Saposhnikoviae（RS）	MOL013077	Decursin	Estradiol 17-beta-dehydrogenase 1	P14061	HSD17B1	0.179
Radix Saposhnikoviae（RS）	MOL013077	Decursin	Nuclear receptor coactivator 1	Q15788	NCOA1	0.181
Radix Saposhnikoviae（RS）	MOL013077	Decursin	ATP-binding cassette transporter sub-family C member 8	Q09428	ABCC8	0.184
Radix Saposhnikoviae（RS）	MOL013077	Decursin	Carbonic anhydrase 1	P00915	CA1	0.185
Radix Saposhnikoviae（RS）	MOL013077	Decursin	Mineralocorticoid receptor	P08235	NR3C2	0.187
Radix Saposhnikoviae（RS）	MOL013077	Decursin	Cyclin-A2	P20248	CCNA2	0.192
Radix Saposhnikoviae（RS）	MOL013077	Decursin	Trypsin-1	P07477	PRSS1	0.201
Radix Saposhnikoviae（RS）	MOL013077	Decursin	cAMP-dependent protein kinase catalytic subunit alpha	P17612	PRKACA	0.333
Radix Saposhnikoviae（RS）	MOL013077	Decursin	Progesterone receptor	P06401	PGR	0.513
Radix Saposhnikoviae（RS）	MOL013077	Decursin	Estrogen receptor beta	Q92731	ESR2	0.543
Radix Saposhnikoviae（RS）	MOL013077	Decursin	Prostaglandin G/H synthase 2	P35354	PTGS2	0.613
Radix Saposhnikoviae（RS）	MOL013077	Decursin	Estrogen receptor	P03372	ESR1	1
CassiaTwig（CT）	MOL000073	ent-Epicatechin	Glucose-6-phosphate isomerase	P06744	GPI	0.01
CassiaTwig（CT）	MOL000073	ent-Epicatechin	Cation-independent mannose-6-phosphate receptor	P11717	IGF2R	0.01
CassiaTwig（CT）	MOL000073	ent-Epicatechin	Hexokinase-1	P19367	HK1	0.01
CassiaTwig（CT）	MOL000073	ent-Epicatechin	Gamma-aminobutyric-acid receptor subunit beta-3	P28472	GABRB3	0.01
CassiaTwig（CT）	MOL000073	ent-Epicatechin	Translocator protein	P30536	TSPO	0.01
CassiaTwig（CT）	MOL000073	ent-Epicatechin	Phosphatidylinositol 3-kinase regulatory subunit beta	O00459	PIK3R2	0.011
CassiaTwig（CT）	MOL000073	ent-Epicatechin	Beta-nerve growth factor	P01138	NGF	0.011
CassiaTwig（CT）	MOL000073	ent-Epicatechin	Phosphatidylinositol 3-kinase regulatory subunit alpha	P27986	PIK3R1	0.011
CassiaTwig（CT）	MOL000073	ent-Epicatechin	Mitogen-activated protein kinase 1	P28482	MAPK1	0.011
CassiaTwig（CT）	MOL000073	ent-Epicatechin	Potassium voltage-gated channel subfamily H member 2	Q12809	KCNH2	0.011
CassiaTwig（CT）	MOL000073	ent-Epicatechin	Potassium voltage-gated channel subfamily H member 6	Q9H252	KCNH6	0.011
CassiaTwig（CT）	MOL000073	ent-Epicatechin	D1 dopamine receptor-interacting protein calcyon	Q9NYX4	CALY	0.011
CassiaTwig（CT）	MOL000073	ent-Epicatechin	cAMP-dependent protein kinase type I-alpha regulatory subunit	P10644	PRKAR1A	0.014
CassiaTwig（CT）	MOL000073	ent-Epicatechin	Amine oxidase [flavin-containing] A	P21397	MAOA	0.014
CassiaTwig（CT）	MOL000073	ent-Epicatechin	cAMP-dependent protein kinase type II-beta regulatory subunit	P31323	PRKAR2B	0.014
CassiaTwig（CT）	MOL000073	ent-Epicatechin	Neuronal acetylcholine receptor subunit alpha-4	P43681	CHRNA4	0.014

CassiaTwig（CT）	MOL000073	ent-Epicatechin	5-hydroxytryptamine 3 receptor	P46098	HTR3A	0.014
CassiaTwig（CT）	MOL000073	ent-Epicatechin	Chromaffin granule amine transporter	P54219	SLC18A1	0.014
CassiaTwig（CT）	MOL000073	ent-Epicatechin	5-hydroxytryptamine 4 receptor	Q13639	HTR4	0.014
CassiaTwig（CT）	MOL000073	ent-Epicatechin	Opioid receptor, sigma 1	Q5T1J1	SIGMAR1	0.014
CassiaTwig（CT）	MOL000073	ent-Epicatechin	Sigma 1-type opioid receptor	Q99720	SIGMAR1	0.014
CassiaTwig（CT）	MOL000073	ent-Epicatechin	cGMP-dependent 3',5'-cyclic phosphodiesterase	O00408	PDE2A	0.015
CassiaTwig（CT）	MOL000073	ent-Epicatechin	Serine/threonine-protein kinase 17B	O94768	STK17B	0.015
CassiaTwig（CT）	MOL000073	ent-Epicatechin	Proto-oncogene serine/threonine-protein kinase Pim-1	P11309	PIM1	0.015
CassiaTwig（CT）	MOL000073	ent-Epicatechin	ATP synthase subunit gamma, mitochondrial	P36542	ATP5F1C	0.015
CassiaTwig（CT）	MOL000073	ent-Epicatechin	Phosphatidylinositol-4,5-bisphosphate 3-kinase catalytic subunit gamma isoform	P48736	PIK3CG	0.015
CassiaTwig（CT）	MOL000073	ent-Epicatechin	Alpha-7 nicotinic cholinergic receptor subunit	Q693P7	CHRNA7	0.015
CassiaTwig（CT）	MOL000073	ent-Epicatechin	UDP-glucuronosyltransferase 3A1	Q6NUS8	UGT3A1	0.015
CassiaTwig（CT）	MOL000073	ent-Epicatechin	Substance-P receptor	P25103	TACR1	0.016
CassiaTwig（CT）	MOL000073	ent-Epicatechin	Dihydrolipoyllysine-residue acetyltransferase component of pyruvate dehydrogenase complex, mitochondrial	P10515	DLAT	0.017
CassiaTwig（CT）	MOL000073	ent-Epicatechin	Glycine cleavage system H protein, mitochondrial	P23434	GCSH	0.017
CassiaTwig（CT）	MOL000073	ent-Epicatechin	Gamma-aminobutyric-acid receptor subunit beta-2	P47870	GABRB2	0.017
CassiaTwig（CT）	MOL000073	ent-Epicatechin	Gamma-aminobutyric-acid receptor subunit alpha-6	Q16445	GABRA6	0.017
CassiaTwig（CT）	MOL000073	ent-Epicatechin	Gamma-aminobutyric acid receptor subunit gamma-1	Q8N1C3	GABRG1	0.017
CassiaTwig（CT）	MOL000073	ent-Epicatechin	Gamma-aminobutyric acid receptor subunit theta	Q9UN88	GABRQ	0.017
CassiaTwig（CT）	MOL000073	ent-Epicatechin	Potassium channel subfamily K member 1	O00180	KCNK1	0.018
CassiaTwig（CT）	MOL000073	ent-Epicatechin	Interleukin-3	P08700	IL3	0.018
CassiaTwig（CT）	MOL000073	ent-Epicatechin	Cell division protein kinase 4	P11802	CDK4	0.018
CassiaTwig（CT）	MOL000073	ent-Epicatechin	Neuronal acetylcholine receptor subunit alpha-3	P32297	CHRNA3	0.018
CassiaTwig（CT）	MOL000073	ent-Epicatechin	Cell division protein kinase 7	P50613	CDK7	0.018
CassiaTwig（CT）	MOL000073	ent-Epicatechin	Cell division protein kinase 9	P50750	CDK9	0.018
CassiaTwig（CT）	MOL000073	ent-Epicatechin	Protein S100-A12	P80511	S100A12	0.018
CassiaTwig（CT）	MOL000073	ent-Epicatechin	Solute carrier family 22 member 6	Q4U2R8	SLC22A6	0.018
CassiaTwig（CT）	MOL000073	ent-Epicatechin	Solute carrier family 22 member 8	Q8TCC7	SLC22A8	0.018
CassiaTwig（CT）	MOL000073	ent-Epicatechin	Protein S100-A13	Q99584	S100A13	0.018
CassiaTwig（CT）	MOL000073	ent-Epicatechin	Solute carrier family 22 member 11	Q9NSA0	SLC22A11	0.018
CassiaTwig（CT）	MOL000073	ent-Epicatechin	Amiloride-sensitive sodium channel subunit alpha	P37088	SCNN1A	0.019
CassiaTwig（CT）	MOL000073	ent-Epicatechin	Amiloride-sensitive sodium channel subunit beta	P51168	SCNN1B	0.019
CassiaTwig（CT）	MOL000073	ent-Epicatechin	Amiloride-sensitive sodium channel subunit gamma	P51170	SCNN1G	0.019
CassiaTwig（CT）	MOL000073	ent-Epicatechin	Carbonic anhydrase 4	P22748	CA4	0.02
CassiaTwig（CT）	MOL000073	ent-Epicatechin	Glycogen synthase kinase-3 beta	P49841	GSK3B	0.02
CassiaTwig（CT）	MOL000073	ent-Epicatechin	Sodium/potassium-transporting ATPase gamma chain	P54710	FXYD2	0.02
CassiaTwig（CT）	MOL000073	ent-Epicatechin	Solute carrier family 12 member 3	P55017	SLC12A3	0.02
CassiaTwig（CT）	MOL000073	ent-Epicatechin	Calcium-activated potassium channel subunit alpha 1	Q12791	KCNMA1	0.02
CassiaTwig（CT）	MOL000073	ent-Epicatechin	Cyclin-dependent kinase 5 activator 1	Q15078	CDK5R1	0.02
CassiaTwig（CT）	MOL000073	ent-Epicatechin	Retinoic acid receptor alpha	P10276	RARA	0.021
CassiaTwig（CT）	MOL000073	ent-Epicatechin	Retinoic acid receptor beta	P10826	RARB	0.021
CassiaTwig（CT）	MOL000073	ent-Epicatechin	Retinoic acid receptor gamma-1	P13631	RARG	0.021
CassiaTwig（CT）	MOL000073	ent-Epicatechin	Retinoic acid receptor RXR-gamma	P48443	RXRG	0.021
CassiaTwig（CT）	MOL000073	ent-Epicatechin	Tyrosine 3-monooxygenase	P07101	TH	0.022
CassiaTwig（CT）	MOL000073	ent-Epicatechin	Peptidyl-prolyl cis-trans isomerase NIMA-interacting 1	Q13526	PIN1	0.022
CassiaTwig（CT）	MOL000073	ent-Epicatechin	Tyrosyl-tRNA synthetase, mitochondrial	Q9Y2Z4	YARS2	0.022
CassiaTwig（CT）	MOL000073	ent-Epicatechin	Gamma-aminobutyric acid receptor subunit rho-3	A8MPY1	GABRR3	0.023
CassiaTwig（CT）	MOL000073	ent-Epicatechin	Gamma-aminobutyric acid receptor subunit pi	O00591	GABRP	0.023
CassiaTwig（CT）	MOL000073	ent-Epicatechin	Gamma-aminobutyric acid receptor subunit delta	O14764	GABRD	0.023
CassiaTwig（CT）	MOL000073	ent-Epicatechin	Gamma-aminobutyric-acid receptor subunit beta-1	P18505	GABRB1	0.023
CassiaTwig（CT）	MOL000073	ent-Epicatechin	Gamma-aminobutyric acid receptor subunit gamma-2	P18507	GABRG2	0.023
CassiaTwig（CT）	MOL000073	ent-Epicatechin	Gamma-aminobutyric-acid receptor subunit rho-1	P24046	GABRR1	0.023

CassiaTwig（CT）	MOL000073	ent-Epicatechin	Gamma-aminobutyric acid receptor subunit rho-2	P28476	GABRR2	0.023
CassiaTwig（CT）	MOL000073	ent-Epicatechin	Aryl hydrocarbon receptor	P35869	AHR	0.023
CassiaTwig（CT）	MOL000073	ent-Epicatechin	Gamma-aminobutyric acid receptor subunit epsilon	P78334	GABRE	0.023
CassiaTwig（CT）	MOL000073	ent-Epicatechin	Dihydroorotate dehydrogenase, mitochondrial	Q02127	DHODH	0.023
CassiaTwig（CT）	MOL000073	ent-Epicatechin	Gamma-aminobutyric acid receptor subunit gamma-3	Q99928	GABRG3	0.023
CassiaTwig（CT）	MOL000073	ent-Epicatechin	Gamma-aminobutyric-acid receptor subunit alpha-4	P48169	GABRA4	0.024
CassiaTwig（CT）	MOL000073	ent-Epicatechin	S-phase kinase-associated protein 1	P63208	SKP1	0.024
CassiaTwig（CT）	MOL000073	ent-Epicatechin	Inhibitor of nuclear factor kappa-B kinase subunit beta	O14920	IKBKB	0.025
CassiaTwig（CT）	MOL000073	ent-Epicatechin	Phenylalanine-4-hydroxylase	P00439	PAH	0.025
CassiaTwig（CT）	MOL000073	ent-Epicatechin	Beta-3 adrenergic receptor	P13945	ADRB3	0.025
CassiaTwig（CT）	MOL000073	ent-Epicatechin	Neuronal acetylcholine receptor subunit alpha-7	P36544	CHRNA7	0.025
CassiaTwig（CT）	MOL000073	ent-Epicatechin	Neuronal acetylcholine receptor subunit alpha-2	Q15822	CHRNA2	0.025
CassiaTwig（CT）	MOL000073	ent-Epicatechin	Cystine/glutamate transporter	Q9UPY5	SLC7A11	0.025
CassiaTwig（CT）	MOL000073	ent-Epicatechin	Casein kinase II subunit alpha	P68400	CSNK2A1	0.026
CassiaTwig（CT）	MOL000073	ent-Epicatechin	Methionine aminopeptidase 1	P53582	METAP1	0.027
CassiaTwig（CT）	MOL000073	ent-Epicatechin	Purine nucleoside phosphorylase	P00491	PNP	0.028
CassiaTwig（CT）	MOL000073	ent-Epicatechin	Lipoic acid synthetase, mitochondrial	O43766	LIAS	0.029
CassiaTwig（CT）	MOL000073	ent-Epicatechin	Muscarinic acetylcholine receptor M2	P08172	CHRM2	0.029
CassiaTwig（CT）	MOL000073	ent-Epicatechin	Muscarinic acetylcholine receptor M4	P08173	CHRM4	0.029
CassiaTwig（CT）	MOL000073	ent-Epicatechin	Synaptic vesicular amine transporter	Q05940	SLC18A2	0.029
CassiaTwig（CT）	MOL000073	ent-Epicatechin	Lipoyltransferase 1, mitochondrial	Q9Y234	LIPT1	0.029
CassiaTwig（CT）	MOL000073	ent-Epicatechin	Ig kappa chain C region	P01834	IGKC	0.03
CassiaTwig（CT）	MOL000073	ent-Epicatechin	Ig gamma-1 chain C region	P01857	IGHG1	0.03
CassiaTwig（CT）	MOL000073	ent-Epicatechin	Myeloperoxidase	P05164	MPO	0.03
CassiaTwig（CT）	MOL000073	ent-Epicatechin	Eosinophil peroxidase	P11678	EPX	0.03
CassiaTwig（CT）	MOL000073	ent-Epicatechin	Calreticulin	P27797	CALR	0.03
CassiaTwig（CT）	MOL000073	ent-Epicatechin	Gamma-aminobutyric-acid receptor subunit alpha-5	P31644	GABRA5	0.03
CassiaTwig（CT）	MOL000073	ent-Epicatechin	Gamma-aminobutyric-acid receptor subunit alpha-3	P34903	GABRA3	0.03
CassiaTwig（CT）	MOL000073	ent-Epicatechin	Prostacyclin receptor	P43119	PTGIR	0.03
CassiaTwig（CT）	MOL000073	ent-Epicatechin	Melatonin receptor type 1A	P48039	MTNR1A	0.03
CassiaTwig（CT）	MOL000073	ent-Epicatechin	Melatonin receptor type 1B	P49286	MTNR1B	0.03
CassiaTwig（CT）	MOL000073	ent-Epicatechin	Oxysterols receptor LXR-beta	P55055	NR1H2	0.03
CassiaTwig（CT）	MOL000073	ent-Epicatechin	Calmodulin	P62158		0.03
CassiaTwig（CT）	MOL000073	ent-Epicatechin	Peroxisome proliferator-activated receptor delta	Q03181	PPARD	0.03
CassiaTwig（CT）	MOL000073	ent-Epicatechin	Oxysterols receptor LXR-alpha	Q13133	NR1H3	0.03
CassiaTwig（CT）	MOL000073	ent-Epicatechin	Sodium channel protein type 5 subunit alpha	Q14524	SCN5A	0.03
CassiaTwig（CT）	MOL000073	ent-Epicatechin	Nuclear receptor ROR-beta	Q92753	RORB	0.03
CassiaTwig（CT）	MOL000073	ent-Epicatechin	cGMP-inhibited 3',5'-cyclic phosphodiesterase A	Q14432	PDE3A	0.031
CassiaTwig（CT）	MOL000073	ent-Epicatechin	cAMP and cAMP-inhibited cGMP 3',5'-cyclic phosphodiesterase 10A	Q9Y233	PDE10A	0.031
CassiaTwig（CT）	MOL000073	ent-Epicatechin	Macrophage migration inhibitory factor	P14174	MIF	0.032
CassiaTwig（CT）	MOL000073	ent-Epicatechin	Peptidyl-prolyl cis-trans isomerase, mitochondrial	P30405	PPIF	0.032
CassiaTwig（CT）	MOL000073	ent-Epicatechin	Potassium/sodium hyperpolarization-activated cyclic nucleotide-gated channel 2	Q9UL51	HCN2	0.032
CassiaTwig（CT）	MOL000073	ent-Epicatechin	ATP synthase subunit beta, mitochondrial	P06576	ATP5F1B	0.033
CassiaTwig（CT）	MOL000073	ent-Epicatechin	Tyrosine-protein kinase HCK	P08631	HCK	0.033
CassiaTwig（CT）	MOL000073	ent-Epicatechin	Acetylcholinesterase	P22303	ACHE	0.033
CassiaTwig（CT）	MOL000073	ent-Epicatechin	ATP synthase subunit alpha, mitochondrial	P25705	ATP5F1A	0.033
CassiaTwig（CT）	MOL000073	ent-Epicatechin	Glycogen phosphorylase, muscle form	P11217	PYGM	0.034
CassiaTwig（CT）	MOL000073	ent-Epicatechin	Sterol O-acyltransferase 2	O75908	SOAT2	0.037
CassiaTwig（CT）	MOL000073	ent-Epicatechin	Prolactin receptor	P16471	PRLR	0.037
CassiaTwig（CT）	MOL000073	ent-Epicatechin	Gonadotropin-releasing hormone receptor	P30968	GNRHR	0.037
CassiaTwig（CT）	MOL000073	ent-Epicatechin	Sterol O-acyltransferase 1	P35610	SOAT1	0.037
CassiaTwig（CT）	MOL000073	ent-Epicatechin	Gonadotropin-releasing hormone II receptor	Q96P88	GNRHR2	0.037
CassiaTwig（CT）	MOL000073	ent-Epicatechin	3 beta-hydroxysteroid dehydrogenase/Delta 5-->4-isomerase type I	P14060	HSD3B1	0.038

CassiaTwig（CT）	MOL000073	ent-Epicatechin	3 beta-hydroxysteroid dehydrogenase/Delta 5-->4-isomerase type II	P26439	HSD3B2	0.038
CassiaTwig（CT）	MOL000073	ent-Epicatechin	Estrogen-related receptor gamma	P62508	ESRRG	0.038
CassiaTwig（CT）	MOL000073	ent-Epicatechin	Epidermal growth factor receptor	P00533	EGFR	0.039
CassiaTwig（CT）	MOL000073	ent-Epicatechin	DNA topoisomerase 2-alpha	P11388	TOP2A	0.039
CassiaTwig（CT）	MOL000073	ent-Epicatechin	5-hydroxytryptamine 1D receptor	P28221	HTR1D	0.039
CassiaTwig（CT）	MOL000073	ent-Epicatechin	5-hydroxytryptamine 1B receptor	P28222	HTR1B	0.039
CassiaTwig（CT）	MOL000073	ent-Epicatechin	Tubulin beta-2C chain	P68371	TUBB4B	0.039
CassiaTwig（CT）	MOL000073	ent-Epicatechin	Cell division protein kinase 6	Q00534	CDK6	0.039
CassiaTwig（CT）	MOL000073	ent-Epicatechin	Protein tyrosine kinase 2 beta	Q14289	PTK2B	0.039
CassiaTwig（CT）	MOL000073	ent-Epicatechin	Gamma-aminobutyric-acid receptor subunit alpha-2	P47869	GABRA2	0.04
CassiaTwig（CT）	MOL000073	ent-Epicatechin	5-hydroxytryptamine 2B receptor	P41595	HTR2B	0.041
CassiaTwig（CT）	MOL000073	ent-Epicatechin	5-hydroxytryptamine 2A receptor	P28223	HTR2A	0.044
CassiaTwig（CT）	MOL000073	ent-Epicatechin	Platelet glycoprotein IX	P14770	GP9	0.045
CassiaTwig（CT）	MOL000073	ent-Epicatechin	Gamma-aminobutyric-acid receptor subunit alpha-1	P14867	GABRA1	0.047
CassiaTwig（CT）	MOL000073	ent-Epicatechin	Group IIE secretory phospholipase A2	Q9NZK7	PLA2G2E	0.051
CassiaTwig（CT）	MOL000073	ent-Epicatechin	Inhibitor of nuclear factor kappa-B kinase subunit alpha	O15111	CHUK	0.052
CassiaTwig（CT）	MOL000073	ent-Epicatechin	D(4) dopamine receptor	P21917	DRD4	0.053
CassiaTwig（CT）	MOL000073	ent-Epicatechin	Peroxisome proliferator-activated receptor gamma	P37231	PPARG	0.053
CassiaTwig（CT）	MOL000073	ent-Epicatechin	Delta-type opioid receptor	P41143	OPRD1	0.053
CassiaTwig（CT）	MOL000073	ent-Epicatechin	Kappa-type opioid receptor	P41145	OPRK1	0.053
CassiaTwig（CT）	MOL000073	ent-Epicatechin	Retinoic acid receptor RXR-beta	P28702	RXRB	0.055
CassiaTwig（CT）	MOL000073	ent-Epicatechin	5-hydroxytryptamine 1A receptor	P08908	HTR1A	0.056
CassiaTwig（CT）	MOL000073	ent-Epicatechin	Tubulin beta-1 chain	Q9H4B7	TUBB1	0.056
CassiaTwig（CT）	MOL000073	ent-Epicatechin	Egl nine homolog 1	Q9GZT9	EGLN1	0.057
CassiaTwig（CT）	MOL000073	ent-Epicatechin	Cytochrome P450 19A1	P11511	CYP19A1	0.058
CassiaTwig（CT）	MOL000073	ent-Epicatechin	Ribosyldihydronicotinamide dehydrogenase [quinone]	P16083	NQO2	0.058
CassiaTwig（CT）	MOL000073	ent-Epicatechin	Mitogen-activated protein kinase 10	P53779	MAPK10	0.058
CassiaTwig（CT）	MOL000073	ent-Epicatechin	Histamine H1 receptor	P35367	HRH1	0.059
CassiaTwig（CT）	MOL000073	ent-Epicatechin	cAMP-specific 3',5'-cyclic phosphodiesterase 4B	Q07343	PDE4B	0.059
CassiaTwig（CT）	MOL000073	ent-Epicatechin	Cannabinoid receptor 1	P21554	CNR1	0.06
CassiaTwig（CT）	MOL000073	ent-Epicatechin	Geranylgeranyl pyrophosphate synthetase	O95749	GGPS1	0.061
CassiaTwig（CT）	MOL000073	ent-Epicatechin	Dihydrofolate reductase	P00374	DHFR	0.062
CassiaTwig（CT）	MOL000073	ent-Epicatechin	Proto-oncogene tyrosine-protein kinase Src	P12931	SRC	0.063
CassiaTwig（CT）	MOL000073	ent-Epicatechin	S-methyl-5-thioadenosine phosphorylase	Q13126	MTAP	0.063
CassiaTwig（CT）	MOL000073	ent-Epicatechin	Nuclear receptor coactivator 2	Q15596	NCOA2	0.063
CassiaTwig（CT）	MOL000073	ent-Epicatechin	Toll-like receptor 7	Q9NYK1	TLR7	0.063
CassiaTwig（CT）	MOL000073	ent-Epicatechin	DNA polymerase kappa	Q9UBT6	POLK	0.063
CassiaTwig（CT）	MOL000073	ent-Epicatechin	Neuropeptide Y	P01303	NPY	0.064
CassiaTwig（CT）	MOL000073	ent-Epicatechin	cAMP-specific 3',5'-cyclic phosphodiesterase 4A	P27815	PDE4A	0.064
CassiaTwig（CT）	MOL000073	ent-Epicatechin	Thyroid hormone receptor beta-1	P10828	THRB	0.065
CassiaTwig（CT）	MOL000073	ent-Epicatechin	Alpha-1D adrenergic receptor	P25100	ADRA1D	0.066
CassiaTwig（CT）	MOL000073	ent-Epicatechin	ATP-sensitive inward rectifier potassium channel 1	P48048	KCNJ1	0.066
CassiaTwig（CT）	MOL000073	ent-Epicatechin	Nuclear receptor coactivator 5	Q9HCD5	NCOA5	0.066
CassiaTwig（CT）	MOL000073	ent-Epicatechin	Carbonic anhydrase 1	P00915	CA1	0.067
CassiaTwig（CT）	MOL000073	ent-Epicatechin	Alpha-2C adrenergic receptor	P18825	ADRA2C	0.067
CassiaTwig（CT）	MOL000073	ent-Epicatechin	D(1A) dopamine receptor	P21728	DRD1	0.067
CassiaTwig（CT）	MOL000073	ent-Epicatechin	D(1B) dopamine receptor	P21918	DRD5	0.067
CassiaTwig（CT）	MOL000073	ent-Epicatechin	RAC-alpha serine/threonine-protein kinase	P31749	AKT1	0.067
CassiaTwig（CT）	MOL000073	ent-Epicatechin	Cytochrome P450 11B1, mitochondrial	P15538	CYP11B1	0.068
CassiaTwig（CT）	MOL000073	ent-Epicatechin	MAP kinase-activated protein kinase 2	P49137	MAPKAPK2	0.068
CassiaTwig（CT）	MOL000073	ent-Epicatechin	cAMP-dependent protein kinase inhibitor alpha	P61925	PKIA	0.069
CassiaTwig（CT）	MOL000073	ent-Epicatechin	Rho-associated protein kinase 1	Q13464	ROCK1	0.069
CassiaTwig（CT）	MOL000073	ent-Epicatechin	Glutamate [NMDA] receptor subunit 3A	Q8TCU5	GRIN3A	0.069

CassiaTwig（CT）	MOL000073	ent-Epicatechin	Interferon gamma	P01579	IFNG	0.07
CassiaTwig（CT）	MOL000073	ent-Epicatechin	Mitogen-activated protein kinase 14	Q16539	MAPK14	0.07
CassiaTwig（CT）	MOL000073	ent-Epicatechin	Tyrosyl-tRNA synthetase, cytoplasmic	P54577	YARS	0.072
CassiaTwig（CT）	MOL000073	ent-Epicatechin	5-hydroxytryptamine 2C receptor	P28335	HTR2C	0.073
CassiaTwig（CT）	MOL000073	ent-Epicatechin	Glucocorticoid receptor	P04150	NR3C1	0.076
CassiaTwig（CT）	MOL000073	ent-Epicatechin	Sodium-dependent dopamine transporter	Q01959	SLC6A3	0.077
CassiaTwig（CT）	MOL000073	ent-Epicatechin	Arachidonate 5-lipoxygenase	P09917	ALOX5	0.078
CassiaTwig（CT）	MOL000073	ent-Epicatechin	Alpha-1B adrenergic receptor	P35368	ADRA1B	0.08
CassiaTwig（CT）	MOL000073	ent-Epicatechin	Beta-1 adrenergic receptor	P08588	ADRB1	0.081
CassiaTwig（CT）	MOL000073	ent-Epicatechin	Alpha-2A adrenergic receptor	P08913	ADRA2A	0.081
CassiaTwig（CT）	MOL000073	ent-Epicatechin	D(2) dopamine receptor	P14416	DRD2	0.081
CassiaTwig（CT）	MOL000073	ent-Epicatechin	Alpha-2B adrenergic receptor	P18089	ADRA2B	0.081
CassiaTwig（CT）	MOL000073	ent-Epicatechin	D(3) dopamine receptor	P35462	DRD3	0.081
CassiaTwig（CT）	MOL000073	ent-Epicatechin	Tubulin alpha-3 chain	Q71U36	TUBA1A	0.081
CassiaTwig（CT）	MOL000073	ent-Epicatechin	D-HSCDK2	O75100	CA11	0.085
CassiaTwig（CT）	MOL000073	ent-Epicatechin	Mu-type opioid receptor	P35372	OPRM1	0.088
CassiaTwig（CT）	MOL000073	ent-Epicatechin	Carbonic anhydrase 2	P00918	CA2	0.091
CassiaTwig（CT）	MOL000073	ent-Epicatechin	Muscarinic acetylcholine receptor M1	P11229	CHRM1	0.093
CassiaTwig（CT）	MOL000073	ent-Epicatechin	Muscarinic acetylcholine receptor M3	P20309	CHRM3	0.093
CassiaTwig（CT）	MOL000073	ent-Epicatechin	Beta-2 adrenergic receptor	P07550	ADRB2	0.095
CassiaTwig（CT）	MOL000073	ent-Epicatechin	Sodium-dependent serotonin transporter	P31645	SLC6A4	0.099
CassiaTwig（CT）	MOL000073	ent-Epicatechin	Prothrombin	P00734	F2	0.1
CassiaTwig（CT）	MOL000073	ent-Epicatechin	cAMP-dependent protein kinase catalytic subunit alpha	P17612	PRKACA	0.105
CassiaTwig（CT）	MOL000073	ent-Epicatechin	Cell division control protein 2 homolog	P06493	CDK1	0.108
CassiaTwig（CT）	MOL000073	ent-Epicatechin	Alpha-1A adrenergic receptor	P35348	ADRA1A	0.108
CassiaTwig（CT）	MOL000073	ent-Epicatechin	Cell division protein kinase 5	Q00535	CDK5	0.108
CassiaTwig（CT）	MOL000073	ent-Epicatechin	Mineralocorticoid receptor	P08235	NR3C2	0.116
CassiaTwig（CT）	MOL000073	ent-Epicatechin	Sodium-dependent noradrenaline transporter	P23975	SLC6A2	0.117
CassiaTwig（CT）	MOL000073	ent-Epicatechin	cAMP-specific 3',5'-cyclic phosphodiesterase 4D	Q08499	PDE4D	0.125
CassiaTwig（CT）	MOL000073	ent-Epicatechin	Nitric oxide synthase, inducible	P35228	NOS2	0.128
CassiaTwig（CT）	MOL000073	ent-Epicatechin	Androgen receptor	P10275	AR	0.157
CassiaTwig（CT）	MOL000073	ent-Epicatechin	Prostaglandin G/H synthase 1	P23219	PTGS1	0.165
CassiaTwig（CT）	MOL000073	ent-Epicatechin	Cell division protein kinase 2	P24941	CDK2	0.178
CassiaTwig（CT）	MOL000073	ent-Epicatechin	Nuclear receptor coactivator 1	Q15788	NCOA1	0.193
CassiaTwig（CT）	MOL000073	ent-Epicatechin	Hemoglobin subunit alpha	P69905	HBA1	0.209
CassiaTwig（CT）	MOL000073	ent-Epicatechin	Trypsin-1	P07477	PRSS1	0.21
CassiaTwig（CT）	MOL000073	ent-Epicatechin	Prostaglandin G/H synthase 2	P35354	PTGS2	0.219
CassiaTwig（CT）	MOL000073	ent-Epicatechin	Progesterone receptor	P06401	PGR	0.275
CassiaTwig（CT）	MOL000073	ent-Epicatechin	Cyclin-A2	P20248	CCNA2	0.32
CassiaTwig（CT）	MOL000073	ent-Epicatechin	Estrogen receptor beta	Q92731	ESR2	0.388
CassiaTwig（CT）	MOL000073	ent-Epicatechin	Estrogen receptor	P03372	ESR1	1
CassiaTwig（CT）	MOL000105	protocatechuic acid	Maltase-glucoamylase, intestinal	O43451	MGAM	Validated
CassiaTwig（CT）	MOL000105	protocatechuic acid	Protein kinase C gamma type	P05129	PRKCG	Validated
CassiaTwig（CT）	MOL000105	protocatechuic acid	Protein kinase C beta type	P05771	PRKCB	Validated
CassiaTwig（CT）	MOL000105	protocatechuic acid	Protein kinase C alpha type	P17252	PRKCA	Validated
CassiaTwig（CT）	MOL000105	protocatechuic acid	Protein kinase C zeta type	Q05513	PRKCZ	Validated
CassiaTwig（CT）	MOL000358	beta-sitosterol	Retinoic acid receptor RXR-gamma	P48443	RXRG	0.01
CassiaTwig（CT）	MOL000358	beta-sitosterol	Nuclear receptor 0B1	P51843	NR0B1	0.01
CassiaTwig（CT）	MOL000358	beta-sitosterol	Retinoic acid-induced protein 3	Q8NFJ5	GPRC5A	0.01
CassiaTwig（CT）	MOL000358	beta-sitosterol	Retinoic acid receptor alpha	P10276	RARA	0.023
CassiaTwig（CT）	MOL000358	beta-sitosterol	Retinoic acid receptor beta	P10826	RARB	0.023
CassiaTwig（CT）	MOL000358	beta-sitosterol	Retinal dehydrogenase 2	O94788	ALDH1A2	0.029
CassiaTwig（CT）	MOL000358	beta-sitosterol	Retinal dehydrogenase 1	P00352	ALDH1A1	0.029

CassiaTwig（CT）	MOL000358	beta-sitosterol	Potassium channel subfamily K member 1	O00180	KCNK1	0.036
CassiaTwig（CT）	MOL000358	beta-sitosterol	Sodium channel protein type 5 subunit alpha	Q14524	SCN5A	0.036
CassiaTwig（CT）	MOL000358	beta-sitosterol	Potassium channel subfamily K member 6	Q9Y257	KCNK6	0.036
CassiaTwig（CT）	MOL000358	beta-sitosterol	Serine/threonine-protein phosphatase 2A 65 kDa regulatory subunit A alpha isoform	P30153	PPP2R1A	0.038
CassiaTwig（CT）	MOL000358	beta-sitosterol	Serine/threonine-protein phosphatase PP1-alpha catalytic subunit	P62136	PPP1CA	0.038
CassiaTwig（CT）	MOL000358	beta-sitosterol	Ig kappa chain C region	P01834	IGKC	0.043
CassiaTwig（CT）	MOL000358	beta-sitosterol	Ig gamma-1 chain C region	P01857	IGHG1	0.043
CassiaTwig（CT）	MOL000358	beta-sitosterol	Ig gamma-2 chain C region	P01859	IGHG2	0.043
CassiaTwig（CT）	MOL000358	beta-sitosterol	Nuclear receptor subfamily 1 group I member 3	Q14994	NR1I3	0.05
CassiaTwig（CT）	MOL000358	beta-sitosterol	Retinoic acid receptor gamma-1	P13631	RARG	0.052
CassiaTwig（CT）	MOL000358	beta-sitosterol	Retinoic acid receptor RXR-beta	P28702	RXRB	0.052
CassiaTwig（CT）	MOL000358	beta-sitosterol	Bile salt sulfotransferase	Q06520	SULT2A1	0.056
CassiaTwig（CT）	MOL000358	beta-sitosterol	Dehydrogenase/reductase SDR family member 8	Q8NBQ5	HSD17B11	0.056
CassiaTwig（CT）	MOL000358	beta-sitosterol	Platelet glycoprotein IX	P14770	GP9	0.063
CassiaTwig（CT）	MOL000358	beta-sitosterol	Phospholipase A2	P04054	PLA2G1B	0.069
CassiaTwig（CT）	MOL000358	beta-sitosterol	Phospholipase A2, membrane associated	P14555	PLA2G2A	0.069
CassiaTwig（CT）	MOL000358	beta-sitosterol	3-oxo-5-alpha-steroid 4-dehydrogenase 1	P18405	SRD5A1	0.074
CassiaTwig（CT）	MOL000358	beta-sitosterol	3-oxo-5-alpha-steroid 4-dehydrogenase 2	P31213	SRD5A2	0.074
CassiaTwig（CT）	MOL000358	beta-sitosterol	3 beta-hydroxysteroid dehydrogenase/Delta 5-->4-isomerase type II	P26439	HSD3B2	0.079
CassiaTwig（CT）	MOL000358	beta-sitosterol	Microtubule-associated protein 2	P11137	MAP2	0.08
CassiaTwig（CT）	MOL000358	beta-sitosterol	Microtubule-associated protein 1A	P78559	MAP1A	0.08
CassiaTwig（CT）	MOL000358	beta-sitosterol	Gonadotropin-releasing hormone receptor	P30968	GNRHR	0.082
CassiaTwig（CT）	MOL000358	beta-sitosterol	Gonadotropin-releasing hormone II receptor	Q96P88	GNRHR2	0.082
CassiaTwig（CT）	MOL000358	beta-sitosterol	Corticosteroid 11-beta-dehydrogenase isozyme 1	P28845	HSD11B1	0.084
CassiaTwig（CT）	MOL000358	beta-sitosterol	Cytosolic phospholipase A2	P47712	PLA2G4A	0.084
CassiaTwig（CT）	MOL000358	beta-sitosterol	Retinoic acid receptor RXR-alpha	P19793	RXRA	0.092
CassiaTwig（CT）	MOL000358	beta-sitosterol	Serine/threonine-protein phosphatase 2A catalytic subunit alpha isoform	P67775	PPP2CA	0.095
CassiaTwig（CT）	MOL000358	beta-sitosterol	Serine/threonine-protein phosphatase 2A 56 kDa regulatory subunit gamma isoform	Q13362	PPP2R5C	0.095
CassiaTwig（CT）	MOL000358	beta-sitosterol	Sodium-dependent serotonin transporter	P31645	SLC6A4	0.146
CassiaTwig（CT）	MOL000358	beta-sitosterol	Aldo-keto reductase family 1 member C1	Q04828	AKR1C1	0.147
CassiaTwig（CT）	MOL000358	beta-sitosterol	Prostaglandin G/H synthase 2	P35354	PTGS2	0.15
CassiaTwig（CT）	MOL000358	beta-sitosterol	Estrogen receptor beta	Q92731	ESR2	0.163
CassiaTwig（CT）	MOL000358	beta-sitosterol	Mediator of RNA polymerase II transcription subunit 1	Q15648	MED1	0.164
CassiaTwig（CT）	MOL000358	beta-sitosterol	Estradiol 17-beta-dehydrogenase 1	P14061	HSD17B1	0.173
CassiaTwig（CT）	MOL000358	beta-sitosterol	3 beta-hydroxysteroid dehydrogenase/Delta 5-->4-isomerase type I	P14060	HSD3B1	0.175
CassiaTwig（CT）	MOL000358	beta-sitosterol	Nuclear receptor coactivator 5	Q9HCD5	NCOA5	0.178
CassiaTwig（CT）	MOL000358	beta-sitosterol	Androgen receptor	P10275	AR	0.184
CassiaTwig（CT）	MOL000358	beta-sitosterol	Glucocorticoid receptor	P04150	NR3C1	0.187
CassiaTwig（CT）	MOL000358	beta-sitosterol	Cannabinoid receptor 2	P34972	CNR2	0.191
CassiaTwig（CT）	MOL000358	beta-sitosterol	Nuclear receptor coactivator 1	Q15788	NCOA1	0.296
CassiaTwig（CT）	MOL000358	beta-sitosterol	Mineralocorticoid receptor	P08235	NR3C2	0.39
CassiaTwig（CT）	MOL000358	beta-sitosterol	Estrogen receptor	P03372	ESR1	0.971
CassiaTwig（CT）	MOL000358	beta-sitosterol	Progesterone receptor	P06401	PGR	1
CassiaTwig（CT）	MOL000359	sitosterol	Retinoic acid receptor RXR-alpha	P19793	RXRA	0.016
CassiaTwig（CT）	MOL000359	sitosterol	Nuclear receptor subfamily 1 group I member 3	Q14994	NR1I3	0.016
CassiaTwig（CT）	MOL000359	sitosterol	Elongation factor Tu GTP-binding domain-containing protein 1	Q7Z2Z2	EFL1	0.026
CassiaTwig（CT）	MOL000359	sitosterol	Potassium channel subfamily K member 1	O00180	KCNK1	0.032
CassiaTwig（CT）	MOL000359	sitosterol	D-HSCDK2	O75100	CA11	0.032
CassiaTwig（CT）	MOL000359	sitosterol	Cell division control protein 2 homolog	P06493	CDK1	0.032
CassiaTwig（CT）	MOL000359	sitosterol	Cell division protein kinase 5	Q00535	CDK5	0.032
CassiaTwig（CT）	MOL000359	sitosterol	Sodium channel protein type 5 subunit alpha	Q14524	SCN5A	0.032
CassiaTwig（CT）	MOL000359	sitosterol	Potassium channel subfamily K member 6	Q9Y257	KCNK6	0.032

CassiaTwig（CT）	MOL000359	sitosterol	Neuronal acetylcholine receptor subunit alpha-3	P32297	CHRNA3	0.033
CassiaTwig（CT）	MOL000359	sitosterol	Neuronal acetylcholine receptor subunit alpha-7	P36544	CHRNA7	0.033
CassiaTwig（CT）	MOL000359	sitosterol	Neuronal acetylcholine receptor subunit alpha-4	P43681	CHRNA4	0.033
CassiaTwig（CT）	MOL000359	sitosterol	Phosphatidylinositol 3-kinase regulatory subunit alpha	P27986	PIK3R1	0.034
CassiaTwig（CT）	MOL000359	sitosterol	Phosphatidylinositol-4,5-bisphosphate 3-kinase catalytic subunit gamma isoform	P48736	PIK3CG	0.034
CassiaTwig（CT）	MOL000359	sitosterol	Serine/threonine-protein kinase PLK1	P53350	PLK1	0.034
CassiaTwig（CT）	MOL000359	sitosterol	Dehydrogenase/reductase SDR family member 8	Q8NBQ5	HSD17B11	0.041
CassiaTwig（CT）	MOL000359	sitosterol	Ig kappa chain C region	P01834	IGKC	0.042
CassiaTwig（CT）	MOL000359	sitosterol	Ig gamma-1 chain C region	P01857	IGHG1	0.042
CassiaTwig（CT）	MOL000359	sitosterol	Ig gamma-2 chain C region	P01859	IGHG2	0.042
CassiaTwig（CT）	MOL000359	sitosterol	Platelet glycoprotein IX	P14770	GP9	0.054
CassiaTwig（CT）	MOL000359	sitosterol	Phospholipase A2	P04054	PLA2G1B	0.057
CassiaTwig（CT）	MOL000359	sitosterol	Phospholipase A2, membrane associated	P14555	PLA2G2A	0.057
CassiaTwig（CT）	MOL000359	sitosterol	Annexin A1	P04083	ANXA1	0.062
CassiaTwig（CT）	MOL000359	sitosterol	Nuclear receptor 0B1	P51843	NR0B1	0.062
CassiaTwig（CT）	MOL000359	sitosterol	3 beta-hydroxysteroid dehydrogenase/Delta 5-->4-isomerase type II	P26439	HSD3B2	0.064
CassiaTwig（CT）	MOL000359	sitosterol	Cytosolic phospholipase A2	P47712	PLA2G4A	0.066
CassiaTwig（CT）	MOL000359	sitosterol	Corticosteroid 11-beta-dehydrogenase isozyme 1	P28845	HSD11B1	0.067
CassiaTwig（CT）	MOL000359	sitosterol	3-oxo-5-alpha-steroid 4-dehydrogenase 2	P31213	SRD5A2	0.067
CassiaTwig（CT）	MOL000359	sitosterol	Microtubule-associated protein 2	P11137	MAP2	0.068
CassiaTwig（CT）	MOL000359	sitosterol	Prolactin receptor	P16471	PRLR	0.068
CassiaTwig（CT）	MOL000359	sitosterol	Gonadotropin-releasing hormone receptor	P30968	GNRHR	0.068
CassiaTwig（CT）	MOL000359	sitosterol	Microtubule-associated protein 1A	P78559	MAP1A	0.068
CassiaTwig（CT）	MOL000359	sitosterol	Gonadotropin-releasing hormone II receptor	Q96P88	GNRHR2	0.068
CassiaTwig（CT）	MOL000359	sitosterol	Nuclear receptor coactivator 1	Q15788	NCOA1	0.069
CassiaTwig（CT）	MOL000359	sitosterol	Bile salt sulfotransferase	Q06520	SULT2A1	0.091
CassiaTwig（CT）	MOL000359	sitosterol	Prostaglandin G/H synthase 2	P35354	PTGS2	0.115
CassiaTwig（CT）	MOL000359	sitosterol	DNA polymerase kappa	Q9UBT6	POLK	0.115
CassiaTwig（CT）	MOL000359	sitosterol	Nuclear receptor coactivator 2	Q15596	NCOA2	0.116
CassiaTwig（CT）	MOL000359	sitosterol	Aldo-keto reductase family 1 member C1	Q04828	AKR1C1	0.125
CassiaTwig（CT）	MOL000359	sitosterol	ATP-binding cassette transporter sub-family C member 8	Q09428	ABCC8	0.127
CassiaTwig（CT）	MOL000359	sitosterol	3 beta-hydroxysteroid dehydrogenase/Delta 5-->4-isomerase type I	P14060	HSD3B1	0.128
CassiaTwig（CT）	MOL000359	sitosterol	Nitric-oxide synthase, endothelial	P29474	NOS3	0.134
CassiaTwig（CT）	MOL000359	sitosterol	Cannabinoid receptor 2	P34972	CNR2	0.136
CassiaTwig（CT）	MOL000359	sitosterol	3-oxo-5-alpha-steroid 4-dehydrogenase 1	P18405	SRD5A1	0.145
CassiaTwig（CT）	MOL000359	sitosterol	Nuclear receptor coactivator 5	Q9HCD5	NCOA5	0.15
CassiaTwig（CT）	MOL000359	sitosterol	Estradiol 17-beta-dehydrogenase 1	P14061	HSD17B1	0.221
CassiaTwig（CT）	MOL000359	sitosterol	Androgen receptor	P10275	AR	0.224
CassiaTwig（CT）	MOL000359	sitosterol	Estrogen receptor beta	Q92731	ESR2	0.246
CassiaTwig（CT）	MOL000359	sitosterol	Glucocorticoid receptor	P04150	NR3C1	0.288
CassiaTwig（CT）	MOL000359	sitosterol	Mineralocorticoid receptor	P08235	NR3C2	0.376
CassiaTwig（CT）	MOL000359	sitosterol	Estrogen receptor	P03372	ESR1	0.93
CassiaTwig（CT）	MOL000359	sitosterol	Progesterone receptor	P06401	PGR	1
CassiaTwig（CT）	MOL000475	anethole	Transcription factor AP-1	P05412	JUN	Validated
CassiaTwig（CT）	MOL000475	anethole	NF-kappa-B inhibitor alpha	P25963	NFKBIA	Validated
CassiaTwig（CT）	MOL000475	anethole	Interleukin-2	P60568	IL2	Validated
CassiaTwig（CT）	MOL000475	anethole	Transcription factor p65	Q04206	RELA	Validated
CassiaTwig（CT）	MOL000704	styrene	Myc proto-oncogene protein	P01106	MYC	Validated
CassiaTwig（CT）	MOL000704	styrene	Prolactin	P01236	PRL	Validated
CassiaTwig（CT）	MOL000704	styrene	Metallothionein-2	P02795	MT2A	Validated
CassiaTwig（CT）	MOL000704	styrene	Interleukin-4	P05112	IL4	Validated
CassiaTwig（CT）	MOL000704	styrene	Interleukin-5	P05113	IL5	Validated
CassiaTwig（CT）	MOL000704	styrene	Cytochrome P450 1A2	P05177	CYP1A2	Validated

CassiaTwig（CT）	MOL000704	styrene	Cytochrome P450 2E1	P05181	CYP2E1	Validated
CassiaTwig（CT）	MOL000704	styrene	Low affinity immunoglobulin epsilon Fc receptor	P06734	FCER2	Validated
CassiaTwig（CT）	MOL000704	styrene	Cytochrome P450 3A4	P08684	CYP3A4	Validated
CassiaTwig（CT）	MOL000704	styrene	Transforming growth factor beta-3	P10600	TGFB3	Validated
CassiaTwig（CT）	MOL000704	styrene	Interleukin-13	P35225	IL13	Validated
CassiaTwig（CT）	MOL000704	styrene	Transforming growth factor beta-2	P61812	TGFB2	Validated
CassiaTwig（CT）	MOL000704	styrene	Apoptosis regulator BAX	Q07812	BAX	Validated
CassiaTwig（CT）	MOL000704	styrene	Bcl-2-like protein 1	Q07817	BCL2L1	Validated
CassiaTwig（CT）	MOL000704	styrene	Sialin	Q9NRA2	SLC17A5	Validated
CassiaTwig（CT）	MOL000991	cinnamaldehyde	C5a anaphylatoxin chemotactic receptor	P21730	C5AR1	Validated
CassiaTwig（CT）	MOL001736	(-)-taxifolin	Gamma-aminobutyric acid receptor subunit rho-3	A8MPY1	GABRR3	0.011
CassiaTwig（CT）	MOL001736	(-)-taxifolin	Gamma-aminobutyric acid receptor subunit pi	O00591	GABRP	0.011
CassiaTwig（CT）	MOL001736	(-)-taxifolin	Gamma-aminobutyric acid receptor subunit delta	O14764	GABRD	0.011
CassiaTwig（CT）	MOL001736	(-)-taxifolin	Cytochrome b-c1 complex subunit 8	O14949	UQCRQ	0.011
CassiaTwig（CT）	MOL001736	(-)-taxifolin	Cytochrome b-c1 complex subunit 10	O14957	UQCR11	0.011
CassiaTwig（CT）	MOL001736	(-)-taxifolin	Cytochrome b-c1 complex subunit 6, mitochondrial	P07919	UQCRH	0.011
CassiaTwig（CT）	MOL001736	(-)-taxifolin	Cytochrome b-c1 complex subunit 7	P14927	UQCRB	0.011
CassiaTwig（CT）	MOL001736	(-)-taxifolin	Gamma-aminobutyric-acid receptor subunit beta-1	P18505	GABRB1	0.011
CassiaTwig（CT）	MOL001736	(-)-taxifolin	Gamma-aminobutyric acid receptor subunit gamma-2	P18507	GABRG2	0.011
CassiaTwig（CT）	MOL001736	(-)-taxifolin	Cytochrome b-c1 complex subunit 2, mitochondrial	P22695	UQCRC2	0.011
CassiaTwig（CT）	MOL001736	(-)-taxifolin	Gamma-aminobutyric-acid receptor subunit rho-1	P24046	GABRR1	0.011
CassiaTwig（CT）	MOL001736	(-)-taxifolin	Gamma-aminobutyric acid receptor subunit rho-2	P28476	GABRR2	0.011
CassiaTwig（CT）	MOL001736	(-)-taxifolin	Gamma-aminobutyric-acid receptor subunit alpha-5	P31644	GABRA5	0.011
CassiaTwig（CT）	MOL001736	(-)-taxifolin	Ubiquinol-cytochrome-c reductase complex core protein 1, mitochondrial	P31930	UQCRC1	0.011
CassiaTwig（CT）	MOL001736	(-)-taxifolin	Gamma-aminobutyric-acid receptor subunit alpha-3	P34903	GABRA3	0.011
CassiaTwig（CT）	MOL001736	(-)-taxifolin	Gamma-aminobutyric-acid receptor subunit beta-2	P47870	GABRB2	0.011
CassiaTwig（CT）	MOL001736	(-)-taxifolin	Gamma-aminobutyric-acid receptor subunit alpha-4	P48169	GABRA4	0.011
CassiaTwig（CT）	MOL001736	(-)-taxifolin	Gamma-aminobutyric acid receptor subunit epsilon	P78334	GABRE	0.011
CassiaTwig（CT）	MOL001736	(-)-taxifolin	Gamma-aminobutyric-acid receptor subunit alpha-6	Q16445	GABRA6	0.011
CassiaTwig（CT）	MOL001736	(-)-taxifolin	Gamma-aminobutyric acid receptor subunit gamma-1	Q8N1C3	GABRG1	0.011
CassiaTwig（CT）	MOL001736	(-)-taxifolin	Gamma-aminobutyric acid receptor subunit gamma-3	Q99928	GABRG3	0.011
CassiaTwig（CT）	MOL001736	(-)-taxifolin	Cytochrome b-c1 complex subunit 9	Q9UDW1	UQCR10	0.011
CassiaTwig（CT）	MOL001736	(-)-taxifolin	Gamma-aminobutyric acid receptor subunit theta	Q9UN88	GABRQ	0.011
CassiaTwig（CT）	MOL001736	(-)-taxifolin	5-hydroxytryptamine 1A receptor	P08908	HTR1A	0.016
CassiaTwig（CT）	MOL001736	(-)-taxifolin	5-hydroxytryptamine 1D receptor	P28221	HTR1D	0.016
CassiaTwig（CT）	MOL001736	(-)-taxifolin	5-hydroxytryptamine 1B receptor	P28222	HTR1B	0.016
CassiaTwig（CT）	MOL001736	(-)-taxifolin	5-hydroxytryptamine 2C receptor	P28335	HTR2C	0.016
CassiaTwig（CT）	MOL001736	(-)-taxifolin	5-hydroxytryptamine 2B receptor	P41595	HTR2B	0.016
CassiaTwig（CT）	MOL001736	(-)-taxifolin	Calmodulin	P62158		0.016
CassiaTwig（CT）	MOL001736	(-)-taxifolin	D1 dopamine receptor-interacting protein calcyon	Q9NYX4	CALY	0.016
CassiaTwig（CT）	MOL001736	(-)-taxifolin	Phosphatidylinositol 3-kinase regulatory subunit beta	O00459	PIK3R2	0.017
CassiaTwig（CT）	MOL001736	(-)-taxifolin	Beta-3 adrenergic receptor	P13945	ADRB3	0.017
CassiaTwig（CT）	MOL001736	(-)-taxifolin	Sodium/hydrogen exchanger 1	P19634	SLC9A1	0.017
CassiaTwig（CT）	MOL001736	(-)-taxifolin	Amiloride-sensitive amine oxidase [copper-containing]	P19801	AOC1	0.017
CassiaTwig（CT）	MOL001736	(-)-taxifolin	Phosphatidylinositol 3-kinase regulatory subunit alpha	P27986	PIK3R1	0.017
CassiaTwig（CT）	MOL001736	(-)-taxifolin	5-hydroxytryptamine 2A receptor	P28223	HTR2A	0.017
CassiaTwig（CT）	MOL001736	(-)-taxifolin	Mitogen-activated protein kinase 1	P28482	MAPK1	0.017
CassiaTwig（CT）	MOL001736	(-)-taxifolin	Glutamate receptor 2	P42262	GRIA2	0.017
CassiaTwig（CT）	MOL001736	(-)-taxifolin	Amiloride-sensitive sodium channel subunit delta	P51172	SCNN1D	0.017
CassiaTwig（CT）	MOL001736	(-)-taxifolin	Amiloride-sensitive cation channel 2, neuronal	P78348	ASIC1	0.017
CassiaTwig（CT）	MOL001736	(-)-taxifolin	Potassium voltage-gated channel subfamily H member 2	Q12809	KCNH2	0.017
CassiaTwig（CT）	MOL001736	(-)-taxifolin	Amiloride-sensitive cation channel 1, neuronal	Q16515	ASIC2	0.017

CassiaTwig（CT）	MOL001736	(-)-taxifolin	Potassium channel subfamily K member 6	Q9Y257	KCNK6	0.02
CassiaTwig（CT）	MOL001736	(-)-taxifolin	5-hydroxytryptamine 3 receptor	P46098	HTR3A	0.021
CassiaTwig（CT）	MOL001736	(-)-taxifolin	Glutamate [NMDA] receptor subunit 3A	Q8TCU5	GRIN3A	0.021
CassiaTwig（CT）	MOL001736	(-)-taxifolin	Death-associated protein kinase 3	O43293	DAPK3	0.022
CassiaTwig（CT）	MOL001736	(-)-taxifolin	Tyrosine-protein kinase JAK2	O60674	JAK2	0.022
CassiaTwig（CT）	MOL001736	(-)-taxifolin	Tyrosine-protein kinase JAK1	P23458	JAK1	0.022
CassiaTwig（CT）	MOL001736	(-)-taxifolin	Tyrosine-protein kinase JAK3	P52333	JAK3	0.022
CassiaTwig（CT）	MOL001736	(-)-taxifolin	Pepsin A	P00790	REN	0.027
CassiaTwig（CT）	MOL001736	(-)-taxifolin	Dipeptidyl peptidase 4	P27487	DPP4	0.027
CassiaTwig（CT）	MOL001736	(-)-taxifolin	Cytochrome b	P00156	MT-CYB	0.028
CassiaTwig（CT）	MOL001736	(-)-taxifolin	Cytochrome c1, heme protein, mitochondrial	P08574	CYC1	0.028
CassiaTwig（CT）	MOL001736	(-)-taxifolin	Cytochrome b-c1 complex subunit Rieske, mitochondrial	P47985	UQCRFS1	0.028
CassiaTwig（CT）	MOL001736	(-)-taxifolin	Cell division protein kinase 4	P11802	CDK4	0.029
CassiaTwig（CT）	MOL001736	(-)-taxifolin	Cell division protein kinase 7	P50613	CDK7	0.029
CassiaTwig（CT）	MOL001736	(-)-taxifolin	Cell division protein kinase 9	P50750	CDK9	0.029
CassiaTwig（CT）	MOL001736	(-)-taxifolin	Cell division protein kinase 6	Q00534	CDK6	0.029
CassiaTwig（CT）	MOL001736	(-)-taxifolin	Tyrosine 3-monooxygenase	P07101	TH	0.03
CassiaTwig（CT）	MOL001736	(-)-taxifolin	Tyrosyl-tRNA synthetase, mitochondrial	Q9Y2Z4	YARS2	0.03
CassiaTwig（CT）	MOL001736	(-)-taxifolin	Adenosine A2b receptor	P29275	ADORA2B	0.033
CassiaTwig（CT）	MOL001736	(-)-taxifolin	Adenosine A3 receptor	P33765	SHMT1	0.033
CassiaTwig（CT）	MOL001736	(-)-taxifolin	Gamma-aminobutyric-acid receptor subunit alpha-2	P47869	GABRA2	0.033
CassiaTwig（CT）	MOL001736	(-)-taxifolin	Cyclin-dependent kinase 5 activator 1	Q15078	CDK5R1	0.033
CassiaTwig（CT）	MOL001736	(-)-taxifolin	cGMP-specific 3',5'-cyclic phosphodiesterase	O76074	PDE5A	0.034
CassiaTwig（CT）	MOL001736	(-)-taxifolin	Adenosine A1 receptor	P30542	ADORA1	0.034
CassiaTwig（CT）	MOL001736	(-)-taxifolin	cAMP-specific 3',5'-cyclic phosphodiesterase 4C	Q08493	PDE4C	0.034
CassiaTwig（CT）	MOL001736	(-)-taxifolin	Phenylalanine-4-hydroxylase	P00439	PAH	0.038
CassiaTwig（CT）	MOL001736	(-)-taxifolin	D(4) dopamine receptor	P21917	DRD4	0.038
CassiaTwig（CT）	MOL001736	(-)-taxifolin	Amiloride-sensitive sodium channel subunit alpha	P37088	SCNN1A	0.039
CassiaTwig（CT）	MOL001736	(-)-taxifolin	Amiloride-sensitive sodium channel subunit beta	P51168	SCNN1B	0.039
CassiaTwig（CT）	MOL001736	(-)-taxifolin	Amiloride-sensitive sodium channel subunit gamma	P51170	SCNN1G	0.039
CassiaTwig（CT）	MOL001736	(-)-taxifolin	Potassium channel subfamily K member 1	O00180	KCNK1	0.042
CassiaTwig（CT）	MOL001736	(-)-taxifolin	Carbonic anhydrase 4	P22748	CA4	0.043
CassiaTwig（CT）	MOL001736	(-)-taxifolin	Sodium/potassium-transporting ATPase gamma chain	P54710	FXYD2	0.043
CassiaTwig（CT）	MOL001736	(-)-taxifolin	Sodium-dependent dopamine transporter	Q01959	SLC6A3	0.043
CassiaTwig（CT）	MOL001736	(-)-taxifolin	Macrophage migration inhibitory factor	P14174	MIF	0.044
CassiaTwig（CT）	MOL001736	(-)-taxifolin	Serine/threonine-protein kinase 17B	O94768	STK17B	0.045
CassiaTwig（CT）	MOL001736	(-)-taxifolin	Proto-oncogene serine/threonine-protein kinase Pim-1	P11309	PIM1	0.045
CassiaTwig（CT）	MOL001736	(-)-taxifolin	ATP synthase subunit gamma, mitochondrial	P36542	ATP5F1C	0.045
CassiaTwig（CT）	MOL001736	(-)-taxifolin	UDP-glucuronosyltransferase 3A1	Q6NUS8	UGT3A1	0.045
CassiaTwig（CT）	MOL001736	(-)-taxifolin	cAMP-dependent protein kinase inhibitor alpha	P61925	PKIA	0.047
CassiaTwig（CT）	MOL001736	(-)-taxifolin	Sodium channel protein type 10 subunit alpha	Q9Y5Y9	SCN10A	0.047
CassiaTwig（CT）	MOL001736	(-)-taxifolin	Rho-associated protein kinase 1	Q13464	ROCK1	0.048
CassiaTwig（CT）	MOL001736	(-)-taxifolin	Ig kappa chain C region	P01834	IGKC	0.051
CassiaTwig（CT）	MOL001736	(-)-taxifolin	Ig gamma-1 chain C region	P01857	IGHG1	0.051
CassiaTwig（CT）	MOL001736	(-)-taxifolin	Carboxypeptidase A1	P15085	CPA1	0.051
CassiaTwig（CT）	MOL001736	(-)-taxifolin	Chymase	P23946	CMA1	0.051
CassiaTwig（CT）	MOL001736	(-)-taxifolin	Mitogen-activated protein kinase 8	P45983	MAPK8	0.051
CassiaTwig（CT）	MOL001736	(-)-taxifolin	Mitogen-activated protein kinase 10	P53779	MAPK10	0.051
CassiaTwig（CT）	MOL001736	(-)-taxifolin	Peptidyl-prolyl cis-trans isomerase, mitochondrial	P30405	PPIF	0.052
CassiaTwig（CT）	MOL001736	(-)-taxifolin	Proto-oncogene tyrosine-protein kinase LCK	P06239	LCK	0.053
CassiaTwig（CT）	MOL001736	(-)-taxifolin	Tyrosine-protein kinase Lyn	P07948	LYN	0.053
CassiaTwig（CT）	MOL001736	(-)-taxifolin	Muscarinic acetylcholine receptor M4	P08173	CHRM4	0.053
CassiaTwig（CT）	MOL001736	(-)-taxifolin	Gamma-aminobutyric-acid receptor subunit alpha-1	P14867	GABRA1	0.053

CassiaTwig（CT）	MOL001736	(-)-taxifolin	Aryl hydrocarbon receptor	P35869	AHR	0.054
CassiaTwig（CT）	MOL001736	(-)-taxifolin	Dihydroorotate dehydrogenase, mitochondrial	Q02127	DHODH	0.054
CassiaTwig（CT）	MOL001736	(-)-taxifolin	Glycogen phosphorylase, muscle form	P11217	PYGM	0.055
CassiaTwig（CT）	MOL001736	(-)-taxifolin	Group IIE secretory phospholipase A2	Q9NZK7	PLA2G2E	0.058
CassiaTwig（CT）	MOL001736	(-)-taxifolin	Ribosyldihydronicotinamide dehydrogenase [quinone]	P16083	NQO2	0.059
CassiaTwig（CT）	MOL001736	(-)-taxifolin	Alpha-1D adrenergic receptor	P25100	ADRA1D	0.059
CassiaTwig（CT）	MOL001736	(-)-taxifolin	Alpha-1B adrenergic receptor	P35368	ADRA1B	0.059
CassiaTwig（CT）	MOL001736	(-)-taxifolin	Peroxisome proliferator-activated receptor gamma	P37231	PPARG	0.059
CassiaTwig（CT）	MOL001736	(-)-taxifolin	D(1A) dopamine receptor	P21728	DRD1	0.06
CassiaTwig（CT）	MOL001736	(-)-taxifolin	D(1B) dopamine receptor	P21918	DRD5	0.06
CassiaTwig（CT）	MOL001736	(-)-taxifolin	D(3) dopamine receptor	P35462	DRD3	0.06
CassiaTwig（CT）	MOL001736	(-)-taxifolin	Epidermal growth factor receptor	P00533	EGFR	0.065
CassiaTwig（CT）	MOL001736	(-)-taxifolin	Retinoic acid receptor RXR-beta	P28702	RXRB	0.065
CassiaTwig（CT）	MOL001736	(-)-taxifolin	Tyrosyl-tRNA synthetase, cytoplasmic	P54577	YARS	0.065
CassiaTwig（CT）	MOL001736	(-)-taxifolin	Oxysterols receptor LXR-beta	P55055	NR1H2	0.065
CassiaTwig（CT）	MOL001736	(-)-taxifolin	Oxysterols receptor LXR-alpha	Q13133	NR1H3	0.065
CassiaTwig（CT）	MOL001736	(-)-taxifolin	Nuclear receptor coactivator 2	Q15596	NCOA2	0.065
CassiaTwig（CT）	MOL001736	(-)-taxifolin	Sodium channel protein type 5 subunit alpha	Q14524	SCN5A	0.068
CassiaTwig（CT）	MOL001736	(-)-taxifolin	Adenosine A2a receptor	P29274	ADORA2A	0.073
CassiaTwig（CT）	MOL001736	(-)-taxifolin	Glycogen synthase kinase-3 beta	P49841	GSK3B	0.073
CassiaTwig（CT）	MOL001736	(-)-taxifolin	cAMP-specific 3',5'-cyclic phosphodiesterase 4B	Q07343	PDE4B	0.073
CassiaTwig（CT）	MOL001736	(-)-taxifolin	Platelet glycoprotein IX	P14770	GP9	0.074
CassiaTwig（CT）	MOL001736	(-)-taxifolin	Sterol O-acyltransferase 2	O75908	SOAT2	0.08
CassiaTwig（CT）	MOL001736	(-)-taxifolin	DNA topoisomerase 2-alpha	P11388	TOP2A	0.08
CassiaTwig（CT）	MOL001736	(-)-taxifolin	Sterol O-acyltransferase 1	P35610	SOAT1	0.08
CassiaTwig（CT）	MOL001736	(-)-taxifolin	Protein tyrosine kinase 2 beta	Q14289	PTK2B	0.08
CassiaTwig（CT）	MOL001736	(-)-taxifolin	Alpha-2C adrenergic receptor	P18825	ADRA2C	0.081
CassiaTwig（CT）	MOL001736	(-)-taxifolin	D(2) dopamine receptor	P14416	DRD2	0.082
CassiaTwig（CT）	MOL001736	(-)-taxifolin	Estrogen-related receptor gamma	P62508	ESRRG	0.087
CassiaTwig（CT）	MOL001736	(-)-taxifolin	Carbonic anhydrase 1	P00915	CA1	0.092
CassiaTwig（CT）	MOL001736	(-)-taxifolin	ATP synthase subunit beta, mitochondrial	P06576	ATP5F1B	0.094
CassiaTwig（CT）	MOL001736	(-)-taxifolin	ATP synthase subunit alpha, mitochondrial	P25705	ATP5F1A	0.094
CassiaTwig（CT）	MOL001736	(-)-taxifolin	Heat shock protein HSP 90-beta	P08238	HSP90AB1	0.102
CassiaTwig（CT）	MOL001736	(-)-taxifolin	Alpha-2A adrenergic receptor	P08913	ADRA2A	0.102
CassiaTwig（CT）	MOL001736	(-)-taxifolin	Alpha-2B adrenergic receptor	P18089	ADRA2B	0.102
CassiaTwig（CT）	MOL001736	(-)-taxifolin	Alpha-1A adrenergic receptor	P35348	ADRA1A	0.102
CassiaTwig（CT）	MOL001736	(-)-taxifolin	DNA polymerase kappa	Q9UBT6	POLK	0.102
CassiaTwig（CT）	MOL001736	(-)-taxifolin	Beta-2 adrenergic receptor	P07550	ADRB2	0.103
CassiaTwig（CT）	MOL001736	(-)-taxifolin	Beta-1 adrenergic receptor	P08588	ADRB1	0.103
CassiaTwig（CT）	MOL001736	(-)-taxifolin	Cannabinoid receptor 1	P21554	CNR1	0.103
CassiaTwig（CT）	MOL001736	(-)-taxifolin	Mu-type opioid receptor	P35372	OPRM1	0.103
CassiaTwig（CT）	MOL001736	(-)-taxifolin	D-HSCDK2	O75100	CA11	0.106
CassiaTwig（CT）	MOL001736	(-)-taxifolin	Proto-oncogene tyrosine-protein kinase Src	P12931	SRC	0.107
CassiaTwig（CT）	MOL001736	(-)-taxifolin	Leukotriene A-4 hydrolase	P09960	LTA4H	0.108
CassiaTwig（CT）	MOL001736	(-)-taxifolin	Retinoic acid receptor RXR-alpha	P19793	RXRA	0.108
CassiaTwig（CT）	MOL001736	(-)-taxifolin	Cyclin-A2	P20248	CCNA2	0.108
CassiaTwig（CT）	MOL001736	(-)-taxifolin	Egl nine homolog 1	Q9GZT9	EGLN1	0.108
CassiaTwig（CT）	MOL001736	(-)-taxifolin	C-jun-amino-terminal kinase-interacting protein 1	Q9UQF2	MAPK8IP1	0.108
CassiaTwig（CT）	MOL001736	(-)-taxifolin	Dihydrofolate reductase	P00374	DHFR	0.109
CassiaTwig（CT）	MOL001736	(-)-taxifolin	RAC-alpha serine/threonine-protein kinase	P31749	AKT1	0.109
CassiaTwig（CT）	MOL001736	(-)-taxifolin	Calcium/calmodulin-dependent protein kinase type II alpha chain	Q9UQM7	CAMK2A	0.109
CassiaTwig（CT）	MOL001736	(-)-taxifolin	Muscarinic acetylcholine receptor M1	P11229	CHRM1	0.111
CassiaTwig（CT）	MOL001736	(-)-taxifolin	Solute carrier family 12 member 1	Q13621	SLC12A1	0.111

CassiaTwig（CT）	MOL001736	(-)-taxifolin	cAMP-specific 3',5'-cyclic phosphodiesterase 4A	P27815	PDE4A	0.113
CassiaTwig（CT）	MOL001736	(-)-taxifolin	Tubulin alpha-3 chain	Q71U36	TUBA1A	0.113
CassiaTwig（CT）	MOL001736	(-)-taxifolin	MAP kinase-activated protein kinase 2	P49137	MAPKAPK2	0.114
CassiaTwig（CT）	MOL001736	(-)-taxifolin	Fibroblast growth factor receptor 2	P21802	FGFR2	0.115
CassiaTwig（CT）	MOL001736	(-)-taxifolin	Mitogen-activated protein kinase 14	Q16539	MAPK14	0.115
CassiaTwig（CT）	MOL001736	(-)-taxifolin	Inhibitor of nuclear factor kappa-B kinase subunit alpha	O15111	CHUK	0.122
CassiaTwig（CT）	MOL001736	(-)-taxifolin	Casein kinase II subunit alpha	P68400	CSNK2A1	0.122
CassiaTwig（CT）	MOL001736	(-)-taxifolin	Interferon gamma	P01579	IFNG	0.13
CassiaTwig（CT）	MOL001736	(-)-taxifolin	Carbonic anhydrase 2	P00918	CA2	0.141
CassiaTwig（CT）	MOL001736	(-)-taxifolin	Cell division control protein 2 homolog	P06493	CDK1	0.143
CassiaTwig（CT）	MOL001736	(-)-taxifolin	Phosphatidylinositol-4,5-bisphosphate 3-kinase catalytic subunit gamma isoform	P48736	PIK3CG	0.144
CassiaTwig（CT）	MOL001736	(-)-taxifolin	Tyrosine-protein kinase HCK	P08631	HCK	0.153
CassiaTwig（CT）	MOL001736	(-)-taxifolin	cAMP-dependent protein kinase catalytic subunit alpha	P17612	PRKACA	0.154
CassiaTwig（CT）	MOL001736	(-)-taxifolin	Muscarinic acetylcholine receptor M2	P08172	CHRM2	0.169
CassiaTwig（CT）	MOL001736	(-)-taxifolin	Cell division protein kinase 2	P24941	CDK2	0.181
CassiaTwig（CT）	MOL001736	(-)-taxifolin	Cell division protein kinase 5	Q00535	CDK5	0.181
CassiaTwig（CT）	MOL001736	(-)-taxifolin	Arachidonate 5-lipoxygenase	P09917	ALOX5	0.182
CassiaTwig（CT）	MOL001736	(-)-taxifolin	Prothrombin	P00734	F2	0.188
CassiaTwig（CT）	MOL001736	(-)-taxifolin	Nitric oxide synthase, inducible	P35228	NOS2	0.213
CassiaTwig（CT）	MOL001736	(-)-taxifolin	Trypsin-1	P07477	PRSS1	0.251
CassiaTwig（CT）	MOL001736	(-)-taxifolin	Hemoglobin subunit alpha	P69905	HBA1	0.375
CassiaTwig（CT）	MOL001736	(-)-taxifolin	Prostaglandin G/H synthase 1	P23219	PTGS1	0.381
CassiaTwig（CT）	MOL001736	(-)-taxifolin	Nuclear receptor coactivator 1	Q15788	NCOA1	0.389
CassiaTwig（CT）	MOL001736	(-)-taxifolin	Prostaglandin G/H synthase 2	P35354	PTGS2	0.645
CassiaTwig（CT）	MOL001736	(-)-taxifolin	Estrogen receptor beta	Q92731	ESR2	0.681
CassiaTwig（CT）	MOL001736	(-)-taxifolin	Estrogen receptor	P03372	ESR1	1
CassiaTwig（CT）	MOL002295	trans-cinnamic acid	Maltase-glucoamylase, intestinal	O43451	MGAM	Validated
CassiaTwig（CT）	MOL002295	trans-cinnamic acid	Trans-cinnamate 4-monooxygenase	P92994	CYP73A5	Validated
CassiaTwig（CT）	MOL004480	acetic acid	Transcription factor p65	Q04206	RELA	Validated
CassiaTwig（CT）	MOL004480	acetic acid	Toll-like receptor 4	O00206	TLR4	Validated
CassiaTwig（CT）	MOL004480	acetic acid	Interferon beta	P01574	IFNB1	Validated
CassiaTwig（CT）	MOL004480	acetic acid	NF-kappa-B inhibitor alpha	P25963	NFKBIA	Validated
CassiaTwig（CT）	MOL004480	acetic acid	Prostaglandin G/H synthase 2	P35354	PTGS2	Validated
CassiaTwig（CT）	MOL004480	acetic acid	Interferon regulatory factor 3	Q14653	IRF3	Validated
CassiaTwig（CT）	MOL004480	acetic acid	Nuclear factor erythroid 2-related factor 2	Q16236	NFE2L2	Validated
CassiaTwig（CT）	MOL004480	acetic acid	Thioredoxin reductase 1, cytoplasmic	Q16881	TXNRD1	Validated
CassiaTwig（CT）	MOL004480	acetic acid	Transient receptor potential cation channel subfamily V member 1	Q8NER1	TRPV1	Validated
CassiaTwig（CT）	MOL004480	acetic acid	Transient receptor potential cation channel subfamily V member 4	Q9HBA0	TRPV4	Validated
CassiaTwig（CT）	MOL004576	taxifolin	Retinoic acid receptor alpha	P10276	RARA	0.01
CassiaTwig（CT）	MOL004576	taxifolin	Retinoic acid receptor beta	P10826	RARB	0.01
CassiaTwig（CT）	MOL004576	taxifolin	Retinoic acid receptor gamma-1	P13631	RARG	0.01
CassiaTwig（CT）	MOL004576	taxifolin	Sucrase-isomaltase, intestinal	P14410	SI	0.01
CassiaTwig（CT）	MOL004576	taxifolin	Retinoic acid receptor RXR-beta	P28702	RXRB	0.01
CassiaTwig（CT）	MOL004576	taxifolin	Retinoic acid receptor RXR-gamma	P48443	RXRG	0.01
CassiaTwig（CT）	MOL004576	taxifolin	2,4-dienoyl-CoA reductase, mitochondrial	Q16698	DECR1	0.01
CassiaTwig（CT）	MOL004576	taxifolin	5-hydroxytryptamine 4 receptor	Q13639	HTR4	0.012
CassiaTwig（CT）	MOL004576	taxifolin	Epidermal growth factor receptor	P00533	EGFR	0.013
CassiaTwig（CT）	MOL004576	taxifolin	Trypsin-2	P07478	PRSS2	0.013
CassiaTwig（CT）	MOL004576	taxifolin	Cell division protein kinase 4	P11802	CDK4	0.013
CassiaTwig（CT）	MOL004576	taxifolin	Elongation factor 2	P13639	EEF2	0.013
CassiaTwig（CT）	MOL004576	taxifolin	Estradiol 17-beta-dehydrogenase 1	P14061	HSD17B1	0.013
CassiaTwig（CT）	MOL004576	taxifolin	Cell division protein kinase 7	P50613	CDK7	0.013
CassiaTwig（CT）	MOL004576	taxifolin	Cell division protein kinase 9	P50750	CDK9	0.013

CassiaTwig（CT）	MOL004576	taxifolin	Cell division protein kinase 6	Q00534	CDK6	0.013
CassiaTwig（CT）	MOL004576	taxifolin	Poly [ADP-ribose] polymerase 3	Q9Y6F1	PARP3	0.013
CassiaTwig（CT）	MOL004576	taxifolin	Dehydrogenase/reductase SDR family member 8	Q8NBQ5	HSD17B11	0.014
CassiaTwig（CT）	MOL004576	taxifolin	Acetylcholinesterase	P22303	ACHE	0.015
CassiaTwig（CT）	MOL004576	taxifolin	Neuronal acetylcholine receptor subunit alpha-3	P32297	CHRNA3	0.015
CassiaTwig（CT）	MOL004576	taxifolin	Neuronal acetylcholine receptor subunit alpha-7	P36544	CHRNA7	0.015
CassiaTwig（CT）	MOL004576	taxifolin	Glycogen synthase kinase-3 beta	P49841	GSK3B	0.015
CassiaTwig（CT）	MOL004576	taxifolin	Cyclin-dependent kinase 5 activator 1	Q15078	CDK5R1	0.015
CassiaTwig（CT）	MOL004576	taxifolin	ADP-ribosyl cyclase 2	Q10588	BST1	0.016
CassiaTwig（CT）	MOL004576	taxifolin	Nicotinamide mononucleotide adenylyltransferase 3	Q96T66	NMNAT3	0.016
CassiaTwig（CT）	MOL004576	taxifolin	Nicotinamide mononucleotide adenylyltransferase 1	Q9HAN9	NMNAT1	0.016
CassiaTwig（CT）	MOL004576	taxifolin	Adenosine A2a receptor	P29274	ADORA2A	0.017
CassiaTwig（CT）	MOL004576	taxifolin	Adenosine A2b receptor	P29275	ADORA2B	0.017
CassiaTwig（CT）	MOL004576	taxifolin	Adenosine A3 receptor	P33765	SHMT1	0.017
CassiaTwig（CT）	MOL004576	taxifolin	Delta-type opioid receptor	P41143	OPRD1	0.017
CassiaTwig（CT）	MOL004576	taxifolin	Kappa-type opioid receptor	P41145	OPRK1	0.017
CassiaTwig（CT）	MOL004576	taxifolin	Neuronal acetylcholine receptor subunit alpha-2	Q15822	CHRNA2	0.017
CassiaTwig（CT）	MOL004576	taxifolin	Phenylalanine-4-hydroxylase	P00439	PAH	0.019
CassiaTwig（CT）	MOL004576	taxifolin	5-hydroxytryptamine 2A receptor	P28223	HTR2A	0.019
CassiaTwig（CT）	MOL004576	taxifolin	Tyrosyl-tRNA synthetase, cytoplasmic	P54577	YARS	0.019
CassiaTwig（CT）	MOL004576	taxifolin	Tyrosine-protein kinase JAK2	O60674	JAK2	0.02
CassiaTwig（CT）	MOL004576	taxifolin	Tyrosine-protein kinase JAK1	P23458	JAK1	0.02
CassiaTwig（CT）	MOL004576	taxifolin	Sodium-dependent serotonin transporter	P31645	SLC6A4	0.02
CassiaTwig（CT）	MOL004576	taxifolin	Tyrosine-protein kinase JAK3	P52333	JAK3	0.02
CassiaTwig（CT）	MOL004576	taxifolin	Glutamate [NMDA] receptor subunit 3A	Q8TCU5	GRIN3A	0.02
CassiaTwig（CT）	MOL004576	taxifolin	T-cell receptor alpha chain C region	P01848	TRAC	0.021
CassiaTwig（CT）	MOL004576	taxifolin	T-cell receptor beta chain C region	P01850	TRBC1	0.021
CassiaTwig（CT）	MOL004576	taxifolin	Amiloride-sensitive sodium channel subunit alpha	P37088	SCNN1A	0.021
CassiaTwig（CT）	MOL004576	taxifolin	Amiloride-sensitive sodium channel subunit beta	P51168	SCNN1B	0.021
CassiaTwig（CT）	MOL004576	taxifolin	Amiloride-sensitive sodium channel subunit gamma	P51170	SCNN1G	0.021
CassiaTwig（CT）	MOL004576	taxifolin	Beta-2-microglobulin	P61769	B2M	0.021
CassiaTwig（CT）	MOL004576	taxifolin	Prostaglandin reductase 1	Q14914	PTGR1	0.021
CassiaTwig（CT）	MOL004576	taxifolin	Serine/threonine-protein kinase 17B	O94768	STK17B	0.022
CassiaTwig（CT）	MOL004576	taxifolin	ATP synthase subunit gamma, mitochondrial	P36542	ATP5F1C	0.022
CassiaTwig（CT）	MOL004576	taxifolin	UDP-glucuronosyltransferase 3A1	Q6NUS8	UGT3A1	0.022
CassiaTwig（CT）	MOL004576	taxifolin	Carbonic anhydrase 4	P22748	CA4	0.024
CassiaTwig（CT）	MOL004576	taxifolin	Sodium/potassium-transporting ATPase gamma chain	P54710	FXYD2	0.024
CassiaTwig（CT）	MOL004576	taxifolin	cAMP-specific 3',5'-cyclic phosphodiesterase 4C	Q08493	PDE4C	0.024
CassiaTwig（CT）	MOL004576	taxifolin	cGMP-inhibited 3',5'-cyclic phosphodiesterase A	Q14432	PDE3A	0.024
CassiaTwig（CT）	MOL004576	taxifolin	cAMP and cAMP-inhibited cGMP 3',5'-cyclic phosphodiesterase 10A	Q9Y233	PDE10A	0.024
CassiaTwig（CT）	MOL004576	taxifolin	Guanine nucleotide-binding protein G(s) subunit alpha isoforms short	P63092	GNAS	0.025
CassiaTwig（CT）	MOL004576	taxifolin	Adenylate cyclase type 2	Q08462	ADCY2	0.025
CassiaTwig（CT）	MOL004576	taxifolin	Ig kappa chain C region	P01834	IGKC	0.027
CassiaTwig（CT）	MOL004576	taxifolin	Ig gamma-1 chain C region	P01857	IGHG1	0.027
CassiaTwig（CT）	MOL004576	taxifolin	Glycogen phosphorylase, muscle form	P11217	PYGM	0.027
CassiaTwig（CT）	MOL004576	taxifolin	Prostacyclin receptor	P43119	PTGIR	0.027
CassiaTwig（CT）	MOL004576	taxifolin	Casein kinase I isoform gamma-2	P78368	CSNK1G2	0.027
CassiaTwig（CT）	MOL004576	taxifolin	Peroxisome proliferator-activated receptor delta	Q03181	PPARD	0.027
CassiaTwig（CT）	MOL004576	taxifolin	Serine/threonine-protein kinase haspin	Q8TF76	HASPIN	0.027
CassiaTwig（CT）	MOL004576	taxifolin	Inhibitor of nuclear factor kappa-B kinase subunit beta	O14920	IKBKB	0.028
CassiaTwig（CT）	MOL004576	taxifolin	Ribosyldihydronicotinamide dehydrogenase [quinone]	P16083	NQO2	0.028
CassiaTwig（CT）	MOL004576	taxifolin	Cystine/glutamate transporter	Q9UPY5	SLC7A11	0.028
CassiaTwig（CT）	MOL004576	taxifolin	Phospholipase A2	P04054	PLA2G1B	0.029

CassiaTwig（CT）	MOL004576	taxifolin	Proto-oncogene tyrosine-protein kinase LCK	P06239	LCK	0.029
CassiaTwig（CT）	MOL004576	taxifolin	Tyrosine-protein kinase Lyn	P07948	LYN	0.029
CassiaTwig（CT）	MOL004576	taxifolin	5-hydroxytryptamine 1D receptor	P28221	HTR1D	0.029
CassiaTwig（CT）	MOL004576	taxifolin	Peptidyl-prolyl cis-trans isomerase, mitochondrial	P30405	PPIF	0.029
CassiaTwig（CT）	MOL004576	taxifolin	5-hydroxytryptamine 2B receptor	P41595	HTR2B	0.029
CassiaTwig（CT）	MOL004576	taxifolin	Aldo-keto reductase family 1 member C3	P42330	AKR1C3	0.029
CassiaTwig（CT）	MOL004576	taxifolin	Bile salt sulfotransferase	Q06520	SULT2A1	0.029
CassiaTwig（CT）	MOL004576	taxifolin	Macrophage migration inhibitory factor	P14174	MIF	0.031
CassiaTwig（CT）	MOL004576	taxifolin	Sodium-dependent dopamine transporter	Q01959	SLC6A3	0.031
CassiaTwig（CT）	MOL004576	taxifolin	Muscarinic acetylcholine receptor M4	P08173	CHRM4	0.032
CassiaTwig（CT）	MOL004576	taxifolin	Mitogen-activated protein kinase 8	P45983	MAPK8	0.034
CassiaTwig（CT）	MOL004576	taxifolin	5-hydroxytryptamine 3 receptor	P46098	HTR3A	0.034
CassiaTwig（CT）	MOL004576	taxifolin	Mitogen-activated protein kinase 10	P53779	MAPK10	0.034
CassiaTwig（CT）	MOL004576	taxifolin	Nitric-oxide synthase, brain	P29475	NOS1	0.035
CassiaTwig（CT）	MOL004576	taxifolin	RAC-beta serine/threonine-protein kinase	P31751	AKT2	0.036
CassiaTwig（CT）	MOL004576	taxifolin	Rho-associated protein kinase 1	Q13464	ROCK1	0.036
CassiaTwig（CT）	MOL004576	taxifolin	Beta-2 adrenergic receptor	P07550	ADRB2	0.04
CassiaTwig（CT）	MOL004576	taxifolin	Alpha-1D adrenergic receptor	P25100	ADRA1D	0.04
CassiaTwig（CT）	MOL004576	taxifolin	5-hydroxytryptamine 1B receptor	P28222	HTR1B	0.04
CassiaTwig（CT）	MOL004576	taxifolin	5-hydroxytryptamine 2C receptor	P28335	HTR2C	0.04
CassiaTwig（CT）	MOL004576	taxifolin	Death-associated protein kinase 3	O43293	DAPK3	0.042
CassiaTwig（CT）	MOL004576	taxifolin	Sterol O-acyltransferase 2	O75908	SOAT2	0.042
CassiaTwig（CT）	MOL004576	taxifolin	Sterol O-acyltransferase 1	P35610	SOAT1	0.042
CassiaTwig（CT）	MOL004576	taxifolin	Retinoic acid receptor RXR-alpha	P19793	RXRA	0.043
CassiaTwig（CT）	MOL004576	taxifolin	Nuclear receptor subfamily 1 group I member 3	Q14994	NR1I3	0.043
CassiaTwig（CT）	MOL004576	taxifolin	ATP synthase subunit beta, mitochondrial	P06576	ATP5F1B	0.046
CassiaTwig（CT）	MOL004576	taxifolin	Tyrosine-protein kinase HCK	P08631	HCK	0.046
CassiaTwig（CT）	MOL004576	taxifolin	Proto-oncogene serine/threonine-protein kinase Pim-1	P11309	PIM1	0.046
CassiaTwig（CT）	MOL004576	taxifolin	DNA topoisomerase 2-alpha	P11388	TOP2A	0.046
CassiaTwig（CT）	MOL004576	taxifolin	ATP synthase subunit alpha, mitochondrial	P25705	ATP5F1A	0.046
CassiaTwig（CT）	MOL004576	taxifolin	Phosphatidylinositol-4,5-bisphosphate 3-kinase catalytic subunit gamma isoform	P48736	PIK3CG	0.046
CassiaTwig（CT）	MOL004576	taxifolin	Protein tyrosine kinase 2 beta	Q14289	PTK2B	0.046
CassiaTwig（CT）	MOL004576	taxifolin	Androgen receptor	P10275	AR	0.048
CassiaTwig（CT）	MOL004576	taxifolin	Prolactin receptor	P16471	PRLR	0.048
CassiaTwig（CT）	MOL004576	taxifolin	Estrogen-related receptor gamma	P62508	ESRRG	0.048
CassiaTwig（CT）	MOL004576	taxifolin	Carbonic anhydrase 1	P00915	CA1	0.049
CassiaTwig（CT）	MOL004576	taxifolin	cAMP-specific 3',5'-cyclic phosphodiesterase 4B	Q07343	PDE4B	0.049
CassiaTwig（CT）	MOL004576	taxifolin	Beta-1 adrenergic receptor	P08588	ADRB1	0.05
CassiaTwig（CT）	MOL004576	taxifolin	D(4) dopamine receptor	P21917	DRD4	0.051
CassiaTwig（CT）	MOL004576	taxifolin	Cannabinoid receptor 2	P34972	CNR2	0.052
CassiaTwig（CT）	MOL004576	taxifolin	Nitric oxide synthase, inducible	P35228	NOS2	0.052
CassiaTwig（CT）	MOL004576	taxifolin	Aldo-keto reductase family 1 member C1	Q04828	AKR1C1	0.052
CassiaTwig（CT）	MOL004576	taxifolin	Tubulin alpha-3 chain	Q71U36	TUBA1A	0.052
CassiaTwig（CT）	MOL004576	taxifolin	cGMP-specific 3',5'-cyclic phosphodiesterase	O76074	PDE5A	0.053
CassiaTwig（CT）	MOL004576	taxifolin	Coagulation factor VII	P08709	F7	0.053
CassiaTwig（CT）	MOL004576	taxifolin	Bifunctional purine biosynthesis protein PURH [Includes: Phosphoribosylaminoimidazolecarboxamide formyltransferase	P31939	ATIC	0.053
CassiaTwig（CT）	MOL004576	taxifolin	Membrane copper amine oxidase	Q16853	AOC3	0.053
CassiaTwig（CT）	MOL004576	taxifolin	Casein kinase I isoform gamma-1	Q9HCP0	CSNK1G1	0.053
CassiaTwig（CT）	MOL004576	taxifolin	5-hydroxytryptamine 1A receptor	P08908	HTR1A	0.054
CassiaTwig（CT）	MOL004576	taxifolin	ATP-sensitive inward rectifier potassium channel 1	P48048	KCNJ1	0.054
CassiaTwig（CT）	MOL004576	taxifolin	Solute carrier family 12 member 1	Q13621	SLC12A1	0.055
CassiaTwig（CT）	MOL004576	taxifolin	Toll-like receptor 7	Q9NYK1	TLR7	0.055

CassiaTwig（CT）	MOL004576	taxifolin	Cytochrome P450 19A1	P11511	CYP19A1	0.056
CassiaTwig（CT）	MOL004576	taxifolin	Muscarinic acetylcholine receptor M3	P20309	CHRM3	0.056
CassiaTwig（CT）	MOL004576	taxifolin	Mediator of RNA polymerase II transcription subunit 1	Q15648	MED1	0.056
CassiaTwig（CT）	MOL004576	taxifolin	Geranylgeranyl pyrophosphate synthetase	O95749	GGPS1	0.057
CassiaTwig（CT）	MOL004576	taxifolin	Cannabinoid receptor 1	P21554	CNR1	0.057
CassiaTwig（CT）	MOL004576	taxifolin	Calmodulin	P62158		0.057
CassiaTwig（CT）	MOL004576	taxifolin	Casein kinase II subunit alpha	P68400	CSNK2A1	0.057
CassiaTwig（CT）	MOL004576	taxifolin	Oxysterols receptor LXR-alpha	Q13133	NR1H3	0.057
CassiaTwig（CT）	MOL004576	taxifolin	Sodium channel protein type 5 subunit alpha	Q14524	SCN5A	0.057
CassiaTwig（CT）	MOL004576	taxifolin	Thiamin pyrophosphokinase 1	Q9H3S4	TPK1	0.057
CassiaTwig（CT）	MOL004576	taxifolin	Glucocorticoid receptor	P04150	NR3C1	0.058
CassiaTwig（CT）	MOL004576	taxifolin	Proto-oncogene tyrosine-protein kinase Src	P12931	SRC	0.058
CassiaTwig（CT）	MOL004576	taxifolin	Peroxisome proliferator-activated receptor gamma	P37231	PPARG	0.059
CassiaTwig（CT）	MOL004576	taxifolin	Group IIE secretory phospholipase A2	Q9NZK7	PLA2G2E	0.059
CassiaTwig（CT）	MOL004576	taxifolin	Fibroblast growth factor receptor 2	P21802	FGFR2	0.06
CassiaTwig（CT）	MOL004576	taxifolin	D(2) dopamine receptor	P14416	DRD2	0.061
CassiaTwig（CT）	MOL004576	taxifolin	Alpha-1B adrenergic receptor	P35368	ADRA1B	0.061
CassiaTwig（CT）	MOL004576	taxifolin	D(3) dopamine receptor	P35462	DRD3	0.061
CassiaTwig（CT）	MOL004576	taxifolin	S-methyl-5-thioadenosine phosphorylase	Q13126	MTAP	0.061
CassiaTwig（CT）	MOL004576	taxifolin	Dihydrofolate reductase	P00374	DHFR	0.062
CassiaTwig（CT）	MOL004576	taxifolin	Mitogen-activated protein kinase 14	Q16539	MAPK14	0.063
CassiaTwig（CT）	MOL004576	taxifolin	DNA polymerase kappa	Q9UBT6	POLK	0.063
CassiaTwig（CT）	MOL004576	taxifolin	D-HSCDK2	O75100	CA11	0.064
CassiaTwig（CT）	MOL004576	taxifolin	Nuclear receptor coactivator 5	Q9HCD5	NCOA5	0.064
CassiaTwig（CT）	MOL004576	taxifolin	Neuropeptide Y	P01303	NPY	0.065
CassiaTwig（CT）	MOL004576	taxifolin	Muscarinic acetylcholine receptor M2	P08172	CHRM2	0.067
CassiaTwig（CT）	MOL004576	taxifolin	Muscarinic acetylcholine receptor M1	P11229	CHRM1	0.067
CassiaTwig（CT）	MOL004576	taxifolin	Interferon gamma	P01579	IFNG	0.068
CassiaTwig（CT）	MOL004576	taxifolin	Mu-type opioid receptor	P35372	OPRM1	0.068
CassiaTwig（CT）	MOL004576	taxifolin	Alpha-2A adrenergic receptor	P08913	ADRA2A	0.072
CassiaTwig（CT）	MOL004576	taxifolin	Alpha-2C adrenergic receptor	P18825	ADRA2C	0.072
CassiaTwig（CT）	MOL004576	taxifolin	D(1A) dopamine receptor	P21728	DRD1	0.072
CassiaTwig（CT）	MOL004576	taxifolin	D(1B) dopamine receptor	P21918	DRD5	0.072
CassiaTwig（CT）	MOL004576	taxifolin	Nitric-oxide synthase, endothelial	P29474	NOS3	0.073
CassiaTwig（CT）	MOL004576	taxifolin	cAMP-dependent protein kinase inhibitor alpha	P61925	PKIA	0.074
CassiaTwig（CT）	MOL004576	taxifolin	Carbonic anhydrase 2	P00918	CA2	0.075
CassiaTwig（CT）	MOL004576	taxifolin	Cell division protein kinase 2	P24941	CDK2	0.08
CassiaTwig（CT）	MOL004576	taxifolin	Cell division control protein 2 homolog	P06493	CDK1	0.081
CassiaTwig（CT）	MOL004576	taxifolin	Alpha-2B adrenergic receptor	P18089	ADRA2B	0.082
CassiaTwig（CT）	MOL004576	taxifolin	Inhibitor of nuclear factor kappa-B kinase subunit alpha	O15111	CHUK	0.09
CassiaTwig（CT）	MOL004576	taxifolin	Arachidonate 5-lipoxygenase	P09917	ALOX5	0.09
CassiaTwig（CT）	MOL004576	taxifolin	Prothrombin	P00734	F2	0.097
CassiaTwig（CT）	MOL004576	taxifolin	Cell division protein kinase 5	Q00535	CDK5	0.097
CassiaTwig（CT）	MOL004576	taxifolin	cAMP-specific 3',5'-cyclic phosphodiesterase 4A	P27815	PDE4A	0.101
CassiaTwig（CT）	MOL004576	taxifolin	Alpha-1A adrenergic receptor	P35348	ADRA1A	0.104
CassiaTwig（CT）	MOL004576	taxifolin	C-jun-amino-terminal kinase-interacting protein 1	Q9UQF2	MAPK8IP1	0.106
CassiaTwig（CT）	MOL004576	taxifolin	Sodium-dependent noradrenaline transporter	P23975	SLC6A2	0.108
CassiaTwig（CT）	MOL004576	taxifolin	MAP kinase-activated protein kinase 2	P49137	MAPKAPK2	0.114
CassiaTwig（CT）	MOL004576	taxifolin	RAC-alpha serine/threonine-protein kinase	P31749	AKT1	0.116
CassiaTwig（CT）	MOL004576	taxifolin	Tyrosine-protein phosphatase non-receptor type 1	P18031	PTPN1	0.119
CassiaTwig（CT）	MOL004576	taxifolin	Hemoglobin subunit alpha	P69905	HBA1	0.137
CassiaTwig（CT）	MOL004576	taxifolin	Cyclin-A2	P20248	CCNA2	0.174
CassiaTwig（CT）	MOL004576	taxifolin	cAMP-dependent protein kinase catalytic subunit alpha	P17612	PRKACA	0.19

CassiaTwig（CT）	MOL004576	taxifolin	Trypsin-1	P07477	PRSS1	0.2
CassiaTwig（CT）	MOL004576	taxifolin	Prostaglandin G/H synthase 1	P23219	PTGS1	0.242
CassiaTwig（CT）	MOL004576	taxifolin	Progesterone receptor	P06401	PGR	0.278
CassiaTwig（CT）	MOL004576	taxifolin	Nuclear receptor coactivator 1	Q15788	NCOA1	0.278
CassiaTwig（CT）	MOL004576	taxifolin	Estrogen receptor beta	Q92731	ESR2	0.437
CassiaTwig（CT）	MOL004576	taxifolin	Prostaglandin G/H synthase 2	P35354	PTGS2	0.522
CassiaTwig（CT）	MOL004576	taxifolin	Estrogen receptor	P03372	ESR1	1
CassiaTwig（CT）	MOL011169	Peroxyergosterol	Muscarinic acetylcholine receptor M2	P08172	CHRM2	0.011
CassiaTwig（CT）	MOL011169	Peroxyergosterol	Muscarinic acetylcholine receptor M4	P08173	CHRM4	0.011
CassiaTwig（CT）	MOL011169	Peroxyergosterol	Muscarinic acetylcholine receptor M5	P08912	CHRM5	0.011
CassiaTwig（CT）	MOL011169	Peroxyergosterol	Muscarinic acetylcholine receptor M1	P11229	CHRM1	0.011
CassiaTwig（CT）	MOL011169	Peroxyergosterol	Muscarinic acetylcholine receptor M3	P20309	CHRM3	0.011
CassiaTwig（CT）	MOL011169	Peroxyergosterol	Sodium-dependent noradrenaline transporter	P23975	SLC6A2	0.011
CassiaTwig（CT）	MOL011169	Peroxyergosterol	Histamine H1 receptor	P35367	HRH1	0.011
CassiaTwig（CT）	MOL011169	Peroxyergosterol	Aldose reductase	P15121	AKR1B1	0.014
CassiaTwig（CT）	MOL011169	Peroxyergosterol	Prostaglandin G/H synthase 1	P23219	PTGS1	0.014
CassiaTwig（CT）	MOL011169	Peroxyergosterol	Mitogen-activated protein kinase 3	P27361	MAPK3	0.014
CassiaTwig（CT）	MOL011169	Peroxyergosterol	Prostaglandin G/H synthase 2	P35354	PTGS2	0.014
CassiaTwig（CT）	MOL011169	Peroxyergosterol	Peroxisome proliferator-activated receptor delta	Q03181	PPARD	0.014
CassiaTwig（CT）	MOL011169	Peroxyergosterol	Nociceptin receptor	P41146	OPRL1	0.027
CassiaTwig（CT）	MOL011169	Peroxyergosterol	Ig kappa chain C region	P01834	IGKC	0.033
CassiaTwig（CT）	MOL011169	Peroxyergosterol	Ig gamma-1 chain C region	P01857	IGHG1	0.033
CassiaTwig（CT）	MOL011169	Peroxyergosterol	Ig gamma-2 chain C region	P01859	IGHG2	0.033
CassiaTwig（CT）	MOL011169	Peroxyergosterol	Dehydrogenase/reductase SDR family member 8	Q8NBQ5	HSD17B11	0.037
CassiaTwig（CT）	MOL011169	Peroxyergosterol	Proto-oncogene tyrosine-protein kinase LCK	P06239	LCK	0.04
CassiaTwig（CT）	MOL011169	Peroxyergosterol	Tyrosine-protein kinase Lyn	P07948	LYN	0.04
CassiaTwig（CT）	MOL011169	Peroxyergosterol	cAMP-dependent protein kinase catalytic subunit alpha	P17612	PRKACA	0.04
CassiaTwig（CT）	MOL011169	Peroxyergosterol	cAMP-dependent protein kinase inhibitor alpha	P61925	PKIA	0.04
CassiaTwig（CT）	MOL011169	Peroxyergosterol	3 beta-hydroxysteroid dehydrogenase/Delta 5-->4-isomerase type II	P26439	HSD3B2	0.054
CassiaTwig（CT）	MOL011169	Peroxyergosterol	Retinoic acid receptor RXR-alpha	P19793	RXRA	0.056
CassiaTwig（CT）	MOL011169	Peroxyergosterol	Nuclear receptor subfamily 1 group I member 3	Q14994	NR1I3	0.056
CassiaTwig（CT）	MOL011169	Peroxyergosterol	Tripartite motif-containing protein 13	O60858	TRIM13	0.058
CassiaTwig（CT）	MOL011169	Peroxyergosterol	Microtubule-associated protein 2	P11137	MAP2	0.058
CassiaTwig（CT）	MOL011169	Peroxyergosterol	Prolactin receptor	P16471	PRLR	0.058
CassiaTwig（CT）	MOL011169	Peroxyergosterol	Gonadotropin-releasing hormone receptor	P30968	GNRHR	0.058
CassiaTwig（CT）	MOL011169	Peroxyergosterol	Delta-type opioid receptor	P41143	OPRD1	0.058
CassiaTwig（CT）	MOL011169	Peroxyergosterol	Microtubule-associated protein 1A	P78559	MAP1A	0.058
CassiaTwig（CT）	MOL011169	Peroxyergosterol	Gonadotropin-releasing hormone II receptor	Q96P88	GNRHR2	0.058
CassiaTwig（CT）	MOL011169	Peroxyergosterol	Corticosteroid 11-beta-dehydrogenase isozyme 1	P28845	HSD11B1	0.059
CassiaTwig（CT）	MOL011169	Peroxyergosterol	Bile salt sulfotransferase	Q06520	SULT2A1	0.083
CassiaTwig（CT）	MOL011169	Peroxyergosterol	Tyrosine-protein phosphatase non-receptor type 1	P18031	PTPN1	0.087
CassiaTwig（CT）	MOL011169	Peroxyergosterol	Nitric oxide synthase, inducible	P35228	NOS2	0.087
CassiaTwig（CT）	MOL011169	Peroxyergosterol	Mediator of RNA polymerase II transcription subunit 1	Q15648	MED1	0.088
CassiaTwig（CT）	MOL011169	Peroxyergosterol	NADPH oxidase organizer 1	Q8NFA2	NOXO1	0.089
CassiaTwig（CT）	MOL011169	Peroxyergosterol	Acetylcholinesterase	P22303	ACHE	0.097
CassiaTwig（CT）	MOL011169	Peroxyergosterol	Aldo-keto reductase family 1 member C1	Q04828	AKR1C1	0.098
CassiaTwig（CT）	MOL011169	Peroxyergosterol	3 beta-hydroxysteroid dehydrogenase/Delta 5-->4-isomerase type I	P14060	HSD3B1	0.106
CassiaTwig（CT）	MOL011169	Peroxyergosterol	Cannabinoid receptor 2	P34972	CNR2	0.106
CassiaTwig（CT）	MOL011169	Peroxyergosterol	Nuclear receptor coactivator 5	Q9HCD5	NCOA5	0.109
CassiaTwig（CT）	MOL011169	Peroxyergosterol	3-oxo-5-alpha-steroid 4-dehydrogenase 1	P18405	SRD5A1	0.124
CassiaTwig（CT）	MOL011169	Peroxyergosterol	3-oxo-5-alpha-steroid 4-dehydrogenase 2	P31213	SRD5A2	0.126
CassiaTwig（CT）	MOL011169	Peroxyergosterol	Mu-type opioid receptor	P35372	OPRM1	0.152
CassiaTwig（CT）	MOL011169	Peroxyergosterol	Kappa-type opioid receptor	P41145	OPRK1	0.152

CassiaTwig（CT）	MOL011169	Peroxyergosterol	Nuclear receptor coactivator 1	Q15788	NCOA1	0.178
CassiaTwig（CT）	MOL011169	Peroxyergosterol	Glucocorticoid receptor	P04150	NR3C1	0.181
CassiaTwig（CT）	MOL011169	Peroxyergosterol	Androgen receptor	P10275	AR	0.185
CassiaTwig（CT）	MOL011169	Peroxyergosterol	Estradiol 17-beta-dehydrogenase 1	P14061	HSD17B1	0.188
CassiaTwig（CT）	MOL011169	Peroxyergosterol	Estrogen receptor beta	Q92731	ESR2	0.195
CassiaTwig（CT）	MOL011169	Peroxyergosterol	Mineralocorticoid receptor	P08235	NR3C2	0.25
CassiaTwig（CT）	MOL011169	Peroxyergosterol	Progesterone receptor	P06401	PGR	0.821
CassiaTwig（CT）	MOL011169	Peroxyergosterol	Estrogen receptor	P03372	ESR1	1
Sargassum pallidum（SP）	MOL000098	quercetin	Adenosine A2b receptor	P29275	ADORA2B	0.011
Sargassum pallidum（SP）	MOL000098	quercetin	Adenosine A3 receptor	P33765	SHMT1	0.011
Sargassum pallidum（SP）	MOL000098	quercetin	cGMP-specific 3',5'-cyclic phosphodiesterase	O76074	PDE5A	0.012
Sargassum pallidum（SP）	MOL000098	quercetin	Adenosine A1 receptor	P30542	ADORA1	0.012
Sargassum pallidum（SP）	MOL000098	quercetin	cAMP-specific 3',5'-cyclic phosphodiesterase 4B	Q07343	PDE4B	0.012
Sargassum pallidum（SP）	MOL000098	quercetin	Neuronal acetylcholine receptor subunit alpha-2	Q15822	CHRNA2	0.012
Sargassum pallidum（SP）	MOL000098	quercetin	Potassium channel subfamily K member 1	O00180	KCNK1	0.013
Sargassum pallidum（SP）	MOL000098	quercetin	Tyrosine 3-monooxygenase	P07101	TH	0.013
Sargassum pallidum（SP）	MOL000098	quercetin	cGMP-inhibited 3',5'-cyclic phosphodiesterase A	Q14432	PDE3A	0.013
Sargassum pallidum（SP）	MOL000098	quercetin	cAMP and cAMP-inhibited cGMP 3',5'-cyclic phosphodiesterase 10A	Q9Y233	PDE10A	0.013
Sargassum pallidum（SP）	MOL000098	quercetin	Tyrosyl-tRNA synthetase, mitochondrial	Q9Y2Z4	YARS2	0.013
Sargassum pallidum（SP）	MOL000098	quercetin	3-phosphoinositide-dependent protein kinase 1	O15530	PDPK1	0.014
Sargassum pallidum（SP）	MOL000098	quercetin	Serine/threonine-protein kinase 17B	O94768	STK17B	0.014
Sargassum pallidum（SP）	MOL000098	quercetin	Pepsin A	P00790	REN	0.014
Sargassum pallidum（SP）	MOL000098	quercetin	Tyrosine-protein kinase Lyn	P07948	LYN	0.014
Sargassum pallidum（SP）	MOL000098	quercetin	Muscarinic acetylcholine receptor M5	P08912	CHRM5	0.014
Sargassum pallidum（SP）	MOL000098	quercetin	Cell division protein kinase 4	P11802	CDK4	0.014
Sargassum pallidum（SP）	MOL000098	quercetin	Gamma-aminobutyric-acid receptor subunit alpha-1	P14867	GABRA1	0.014
Sargassum pallidum（SP）	MOL000098	quercetin	Dipeptidyl peptidase 4	P27487	DPP4	0.014
Sargassum pallidum（SP）	MOL000098	quercetin	ATP synthase subunit gamma, mitochondrial	P36542	ATP5F1C	0.014
Sargassum pallidum（SP）	MOL000098	quercetin	Tyrosine-protein kinase CSK	P41240	CSK	0.014
Sargassum pallidum（SP）	MOL000098	quercetin	Tyrosine-protein kinase ZAP-70	P43403	ZAP70	0.014
Sargassum pallidum（SP）	MOL000098	quercetin	Tyrosine-protein kinase SYK	P43405	SYK	0.014
Sargassum pallidum（SP）	MOL000098	quercetin	Gamma-aminobutyric-acid receptor subunit alpha-2	P47869	GABRA2	0.014
Sargassum pallidum（SP）	MOL000098	quercetin	Cell division protein kinase 7	P50613	CDK7	0.014
Sargassum pallidum（SP）	MOL000098	quercetin	Cell division protein kinase 9	P50750	CDK9	0.014
Sargassum pallidum（SP）	MOL000098	quercetin	Cell division protein kinase 6	Q00534	CDK6	0.014
Sargassum pallidum（SP）	MOL000098	quercetin	Protein kinase C theta type	Q04759	PRKCQ	0.014
Sargassum pallidum（SP）	MOL000098	quercetin	Tyrosine-protein kinase ITK/TSK	Q08881	ITK	0.014
Sargassum pallidum（SP）	MOL000098	quercetin	Cyclin-dependent kinase 5 activator 1	Q15078	CDK5R1	0.014
Sargassum pallidum（SP）	MOL000098	quercetin	UDP-glucuronosyltransferase 3A1	Q6NUS8	UGT3A1	0.014
Sargassum pallidum（SP）	MOL000098	quercetin	Dehydrogenase/reductase SDR family member 8	Q8NBQ5	HSD17B11	0.014
Sargassum pallidum（SP）	MOL000098	quercetin	85 kDa calcium-independent phospholipase A2	O60733	PLA2G6	0.015
Sargassum pallidum（SP）	MOL000098	quercetin	Ig gamma-2 chain C region	P01859	IGHG2	0.015
Sargassum pallidum（SP）	MOL000098	quercetin	Retinoic acid receptor alpha	P10276	RARA	0.015
Sargassum pallidum（SP）	MOL000098	quercetin	Retinoic acid receptor beta	P10826	RARB	0.015
Sargassum pallidum（SP）	MOL000098	quercetin	Retinoic acid receptor gamma-1	P13631	RARG	0.015
Sargassum pallidum（SP）	MOL000098	quercetin	Beta-3 adrenergic receptor	P13945	ADRB3	0.015
Sargassum pallidum（SP）	MOL000098	quercetin	5-hydroxytryptamine 1D receptor	P28221	HTR1D	0.015
Sargassum pallidum（SP）	MOL000098	quercetin	5-hydroxytryptamine 1B receptor	P28222	HTR1B	0.015

Sargassum pallidum（SP）	MOL000098	quercetin	5-hydroxytryptamine 2C receptor	P28335	HTR2C	0.015
Sargassum pallidum（SP）	MOL000098	quercetin	Amiloride-sensitive sodium channel subunit alpha	P37088	SCNN1A	0.015
Sargassum pallidum（SP）	MOL000098	quercetin	Cytosolic phospholipase A2	P47712	PLA2G4A	0.015
Sargassum pallidum（SP）	MOL000098	quercetin	Retinoic acid receptor RXR-gamma	P48443	RXRG	0.015
Sargassum pallidum（SP）	MOL000098	quercetin	Amiloride-sensitive sodium channel subunit beta	P51168	SCNN1B	0.015
Sargassum pallidum（SP）	MOL000098	quercetin	Amiloride-sensitive sodium channel subunit gamma	P51170	SCNN1G	0.015
Sargassum pallidum（SP）	MOL000098	quercetin	Sodium/potassium-transporting ATPase gamma chain	P54710	FXYD2	0.015
Sargassum pallidum（SP）	MOL000098	quercetin	Solute carrier family 12 member 3	P55017	SLC12A3	0.015
Sargassum pallidum（SP）	MOL000098	quercetin	Calcium-activated potassium channel subunit alpha 1	Q12791	KCNMA1	0.015
Sargassum pallidum（SP）	MOL000098	quercetin	5-hydroxytryptamine 3 receptor	P46098	HTR3A	0.017
Sargassum pallidum（SP）	MOL000098	quercetin	Prostaglandin reductase 1	Q14914	PTGR1	0.017
Sargassum pallidum（SP）	MOL000098	quercetin	Glutamate [NMDA] receptor subunit 3A	Q8TCU5	GRIN3A	0.017
Sargassum pallidum（SP）	MOL000098	quercetin	DNA topoisomerase 1	P11387	TOP1	0.02
Sargassum pallidum（SP）	MOL000098	quercetin	Macrophage migration inhibitory factor	P14174	MIF	0.02
Sargassum pallidum（SP）	MOL000098	quercetin	Calcium/calmodulin-dependent protein kinase type II subunit delta	Q13557	CAMK2D	0.02
Sargassum pallidum（SP）	MOL000098	quercetin	DNA topoisomerase I, mitochondrial	Q969P6	TOP1MT	0.02
Sargassum pallidum（SP）	MOL000098	quercetin	STE20-like serine/threonine-protein kinase	Q9H2G2	SLK	0.02
Sargassum pallidum（SP）	MOL000098	quercetin	Tripartite motif-containing protein 13	O60858	TRIM13	0.021
Sargassum pallidum（SP）	MOL000098	quercetin	Chymase	P23946	CMA1	0.021
Sargassum pallidum（SP）	MOL000098	quercetin	DNA-(apurinic or apyrimidinic site) lyase	P27695	APEX1	0.021
Sargassum pallidum（SP）	MOL000098	quercetin	Succinate dehydrogenase [ubiquinone] cytochrome b small subunit, mitochondrial	O14521	SDHD	0.022
Sargassum pallidum（SP）	MOL000098	quercetin	Inhibitor of nuclear factor kappa-B kinase subunit beta	O14920	IKBKB	0.022
Sargassum pallidum（SP）	MOL000098	quercetin	Ribosyldihydronicotinamide dehydrogenase [quinone]	P16083	NQO2	0.022
Sargassum pallidum（SP）	MOL000098	quercetin	Peptidyl-prolyl cis-trans isomerase, mitochondrial	P30405	PPIF	0.022
Sargassum pallidum（SP）	MOL000098	quercetin	Prostacyclin receptor	P43119	PTGIR	0.022
Sargassum pallidum（SP）	MOL000098	quercetin	Mitogen-activated protein kinase 8	P45983	MAPK8	0.022
Sargassum pallidum（SP）	MOL000098	quercetin	Mitogen-activated protein kinase 10	P53779	MAPK10	0.022
Sargassum pallidum（SP）	MOL000098	quercetin	Peroxisome proliferator-activated receptor delta	Q03181	PPARD	0.022
Sargassum pallidum（SP）	MOL000098	quercetin	Group IIE secretory phospholipase A2	Q9NZK7	PLA2G2E	0.022
Sargassum pallidum（SP）	MOL000098	quercetin	Cystine/glutamate transporter	Q9UPY5	SLC7A11	0.022
Sargassum pallidum（SP）	MOL000098	quercetin	Oxysterols receptor LXR-beta	P55055	NR1H2	0.023
Sargassum pallidum（SP）	MOL000098	quercetin	Oxysterols receptor LXR-alpha	Q13133	NR1H3	0.023
Sargassum pallidum（SP）	MOL000098	quercetin	Sodium channel protein type 5 subunit alpha	Q14524	SCN5A	0.023
Sargassum pallidum（SP）	MOL000098	quercetin	Phenylalanine-4-hydroxylase	P00439	PAH	0.024
Sargassum pallidum（SP）	MOL000098	quercetin	Rho-associated protein kinase 1	Q13464	ROCK1	0.024
Sargassum pallidum（SP）	MOL000098	quercetin	Alpha-1D adrenergic receptor	P25100	ADRA1D	0.025
Sargassum pallidum（SP）	MOL000098	quercetin	D(4) dopamine receptor	P21917	DRD4	0.026
Sargassum pallidum（SP）	MOL000098	quercetin	Keratin, type II cytoskeletal 7	P08729	KRT7	0.028
Sargassum pallidum（SP）	MOL000098	quercetin	Platelet glycoprotein IX	P14770	GP9	0.028
Sargassum pallidum（SP）	MOL000098	quercetin	Adenosine A2a receptor	P29274	ADORA2A	0.029
Sargassum pallidum（SP）	MOL000098	quercetin	Sterol O-acyltransferase 2	O75908	SOAT2	0.031
Sargassum pallidum（SP）	MOL000098	quercetin	Sterol O-acyltransferase 1	P35610	SOAT1	0.031
Sargassum pallidum（SP）	MOL000098	quercetin	Tubulin beta-2C chain	P68371	TUBB4B	0.031
Sargassum pallidum（SP）	MOL000098	quercetin	3 beta-hydroxysteroid dehydrogenase/Delta 5-->4-isomerase type II	P26439	HSD3B2	0.032
Sargassum pallidum（SP）	MOL000098	quercetin	Protein tyrosine kinase 2 beta	Q14289	PTK2B	0.032
Sargassum pallidum（SP）	MOL000098	quercetin	Tyrosyl-tRNA synthetase, cytoplasmic	P54577	YARS	0.033
Sargassum pallidum（SP）	MOL000098	quercetin	ATP synthase subunit beta, mitochondrial	P06576	ATP5F1B	0.034

Sargassum pallidum（SP）	MOL000098	quercetin	Muscarinic acetylcholine receptor M4	P08173	CHRM4	0.034
Sargassum pallidum（SP）	MOL000098	quercetin	Tyrosine-protein kinase HCK	P08631	HCK	0.034
Sargassum pallidum（SP）	MOL000098	quercetin	Proto-oncogene serine/threonine-protein kinase Pim-1	P11309	PIM1	0.034
Sargassum pallidum（SP）	MOL000098	quercetin	Muscarinic acetylcholine receptor M3	P20309	CHRM3	0.034
Sargassum pallidum（SP）	MOL000098	quercetin	ATP synthase subunit alpha, mitochondrial	P25705	ATP5F1A	0.034
Sargassum pallidum（SP）	MOL000098	quercetin	Phosphatidylinositol-4,5-bisphosphate 3-kinase catalytic subunit gamma isoform	P48736	PIK3CG	0.034
Sargassum pallidum（SP）	MOL000098	quercetin	Epidermal growth factor receptor	P00533	EGFR	0.035
Sargassum pallidum（SP）	MOL000098	quercetin	Carbonic anhydrase 1	P00915	CA1	0.035
Sargassum pallidum（SP）	MOL000098	quercetin	Proto-oncogene tyrosine-protein kinase LCK	P06239	LCK	0.035
Sargassum pallidum（SP）	MOL000098	quercetin	5-hydroxytryptamine 1A receptor	P08908	HTR1A	0.035
Sargassum pallidum（SP）	MOL000098	quercetin	Glycogen phosphorylase, muscle form	P11217	PYGM	0.035
Sargassum pallidum（SP）	MOL000098	quercetin	Alpha-1B adrenergic receptor	P35368	ADRA1B	0.035
Sargassum pallidum（SP）	MOL000098	quercetin	Bile salt sulfotransferase	Q06520	SULT2A1	0.036
Sargassum pallidum（SP）	MOL000098	quercetin	Ig kappa chain C region	P01834	IGKC	0.037
Sargassum pallidum（SP）	MOL000098	quercetin	Ig gamma-1 chain C region	P01857	IGHG1	0.037
Sargassum pallidum（SP）	MOL000098	quercetin	D(2) dopamine receptor	P14416	DRD2	0.037
Sargassum pallidum（SP）	MOL000098	quercetin	D(3) dopamine receptor	P35462	DRD3	0.037
Sargassum pallidum（SP）	MOL000098	quercetin	Estrogen-related receptor gamma	P62508	ESRRG	0.037
Sargassum pallidum（SP）	MOL000098	quercetin	Muscarinic acetylcholine receptor M2	P08172	CHRM2	0.044
Sargassum pallidum（SP）	MOL000098	quercetin	Muscarinic acetylcholine receptor M1	P11229	CHRM1	0.044
Sargassum pallidum（SP）	MOL000098	quercetin	Retinoic acid receptor RXR-beta	P28702	RXRB	0.044
Sargassum pallidum（SP）	MOL000098	quercetin	Aldo-keto reductase family 1 member C1	Q04828	AKR1C1	0.044
Sargassum pallidum（SP）	MOL000098	quercetin	Alpha-2A adrenergic receptor	P08913	ADRA2A	0.045
Sargassum pallidum（SP）	MOL000098	quercetin	Cytochrome P450 19A1	P11511	CYP19A1	0.045
Sargassum pallidum（SP）	MOL000098	quercetin	Alpha-2C adrenergic receptor	P18825	ADRA2C	0.045
Sargassum pallidum（SP）	MOL000098	quercetin	Alpha-1A adrenergic receptor	P35348	ADRA1A	0.045
Sargassum pallidum（SP）	MOL000098	quercetin	Kynurenine--oxoglutarate transaminase 1	Q16773	KYAT1	0.045
Sargassum pallidum（SP）	MOL000098	quercetin	Membrane copper amine oxidase	Q16853	AOC3	0.045
Sargassum pallidum（SP）	MOL000098	quercetin	NADPH oxidase organizer 1	Q8NFA2	NOXO1	0.045
Sargassum pallidum（SP）	MOL000098	quercetin	Serine/threonine-protein kinase 6	O14965	AURKA	0.046
Sargassum pallidum（SP）	MOL000098	quercetin	Thyroid hormone receptor beta-1	P10828	THRB	0.046
Sargassum pallidum（SP）	MOL000098	quercetin	Egl nine homolog 1	Q9GZT9	EGLN1	0.046
Sargassum pallidum（SP）	MOL000098	quercetin	Tubulin beta-1 chain	Q9H4B7	TUBB1	0.046
Sargassum pallidum（SP）	MOL000098	quercetin	Cannabinoid receptor 1	P21554	CNR1	0.047
Sargassum pallidum（SP）	MOL000098	quercetin	D(1A) dopamine receptor	P21728	DRD1	0.047
Sargassum pallidum（SP）	MOL000098	quercetin	D(1B) dopamine receptor	P21918	DRD5	0.047
Sargassum pallidum（SP）	MOL000098	quercetin	Endothelin-1 receptor	P25101	EDNRA	0.047
Sargassum pallidum（SP）	MOL000098	quercetin	Thiamin pyrophosphokinase 1	Q9H3S4	TPK1	0.047
Sargassum pallidum（SP）	MOL000098	quercetin	Glucocorticoid receptor	P04150	NR3C1	0.048
Sargassum pallidum（SP）	MOL000098	quercetin	Ig kappa chain V-II region RPMI 6410	P06310	IGKV2-30	0.048
Sargassum pallidum（SP）	MOL000098	quercetin	Leukotriene A-4 hydrolase	P09960	LTA4H	0.048
Sargassum pallidum（SP）	MOL000098	quercetin	RAC-alpha serine/threonine-protein kinase	P31749	AKT1	0.048
Sargassum pallidum（SP）	MOL000098	quercetin	Nitric oxide synthase, inducible	P35228	NOS2	0.048
Sargassum pallidum（SP）	MOL000098	quercetin	S-methyl-5-thioadenosine phosphorylase	Q13126	MTAP	0.048
Sargassum pallidum（SP）	MOL000098	quercetin	cAMP-specific 3',5'-cyclic phosphodiesterase 4A	P27815	PDE4A	0.049
Sargassum pallidum（SP）	MOL000098	quercetin	Triosephosphate isomerase	P60174	TPI1	0.049
Sargassum pallidum（SP）	MOL000098	quercetin	Solute carrier family 12 member 1	Q13621	SLC12A1	0.049

Sargassum pallidum（SP）	MOL000098	quercetin	Thyroid hormone receptor alpha	P10827	THRA	0.05
Sargassum pallidum（SP）	MOL000098	quercetin	Calmodulin	P62158		0.05
Sargassum pallidum（SP）	MOL000098	quercetin	Casein kinase II subunit alpha	P68400	CSNK2A1	0.05
Sargassum pallidum（SP）	MOL000098	quercetin	Neuropeptide Y	P01303	NPY	0.051
Sargassum pallidum（SP）	MOL000098	quercetin	Peroxisome proliferator-activated receptor gamma	P37231	PPARG	0.051
Sargassum pallidum（SP）	MOL000098	quercetin	Nuclear receptor coactivator 2	Q15596	NCOA2	0.051
Sargassum pallidum（SP）	MOL000098	quercetin	Toll-like receptor 7	Q9NYK1	TLR7	0.051
Sargassum pallidum（SP）	MOL000098	quercetin	C-jun-amino-terminal kinase-interacting protein 1	Q9UQF2	MAPK8IP1	0.051
Sargassum pallidum（SP）	MOL000098	quercetin	cAMP-dependent protein kinase inhibitor alpha	P61925	PKIA	0.053
Sargassum pallidum（SP）	MOL000098	quercetin	Interferon gamma	P01579	IFNG	0.055
Sargassum pallidum（SP）	MOL000098	quercetin	Fibroblast growth factor receptor 2	P21802	FGFR2	0.055
Sargassum pallidum（SP）	MOL000098	quercetin	Sodium-dependent noradrenaline transporter	P23975	SLC6A2	0.055
Sargassum pallidum（SP）	MOL000098	quercetin	MAP kinase-activated protein kinase 2	P49137	MAPKAPK2	0.055
Sargassum pallidum（SP）	MOL000098	quercetin	Glycogen synthase kinase-3 beta	P49841	GSK3B	0.055
Sargassum pallidum（SP）	MOL000098	quercetin	Sodium-dependent dopamine transporter	Q01959	SLC6A3	0.055
Sargassum pallidum（SP）	MOL000098	quercetin	Nuclear receptor coactivator 5	Q9HCD5	NCOA5	0.055
Sargassum pallidum（SP）	MOL000098	quercetin	Carbonic anhydrase 2	P00918	CA2	0.056
Sargassum pallidum（SP）	MOL000098	quercetin	Alpha-2B adrenergic receptor	P18089	ADRA2B	0.056
Sargassum pallidum（SP）	MOL000098	quercetin	Proto-oncogene tyrosine-protein kinase Src	P12931	SRC	0.057
Sargassum pallidum（SP）	MOL000098	quercetin	DNA polymerase kappa	Q9UBT6	POLK	0.058
Sargassum pallidum（SP）	MOL000098	quercetin	DNA topoisomerase 2-alpha	P11388	TOP2A	0.059
Sargassum pallidum（SP）	MOL000098	quercetin	3 beta-hydroxysteroid dehydrogenase/Delta 5-->4-isomerase type I	P14060	HSD3B1	0.061
Sargassum pallidum（SP）	MOL000098	quercetin	Beta-2 adrenergic receptor	P07550	ADRB2	0.066
Sargassum pallidum（SP）	MOL000098	quercetin	Beta-1 adrenergic receptor	P08588	ADRB1	0.066
Sargassum pallidum（SP）	MOL000098	quercetin	Tubulin alpha-3 chain	Q71U36	TUBA1A	0.067
Sargassum pallidum（SP）	MOL000098	quercetin	Prothrombin	P00734	F2	0.07
Sargassum pallidum（SP）	MOL000098	quercetin	Estradiol 17-beta-dehydrogenase 1	P14061	HSD17B1	0.071
Sargassum pallidum（SP）	MOL000098	quercetin	Delta-type opioid receptor	P41143	OPRD1	0.073
Sargassum pallidum（SP）	MOL000098	quercetin	Kappa-type opioid receptor	P41145	OPRK1	0.073
Sargassum pallidum（SP）	MOL000098	quercetin	D-HSCDK2	O75100	CA11	0.075
Sargassum pallidum（SP）	MOL000098	quercetin	Sodium-dependent serotonin transporter	P31645	SLC6A4	0.075
Sargassum pallidum（SP）	MOL000098	quercetin	Mineralocorticoid receptor	P08235	NR3C2	0.077
Sargassum pallidum（SP）	MOL000098	quercetin	Inhibitor of nuclear factor kappa-B kinase subunit alpha	O15111	CHUK	0.079
Sargassum pallidum（SP）	MOL000098	quercetin	Arachidonate 5-lipoxygenase	P09917	ALOX5	0.079
Sargassum pallidum（SP）	MOL000098	quercetin	cAMP-dependent protein kinase catalytic subunit alpha	P17612	PRKACA	0.083
Sargassum pallidum（SP）	MOL000098	quercetin	Dihydrofolate reductase	P00374	DHFR	0.091
Sargassum pallidum（SP）	MOL000098	quercetin	Cell division control protein 2 homolog	P06493	CDK1	0.095
Sargassum pallidum（SP）	MOL000098	quercetin	Cell division protein kinase 5	Q00535	CDK5	0.115
Sargassum pallidum（SP）	MOL000098	quercetin	Trypsin-1	P07477	PRSS1	0.138
Sargassum pallidum（SP）	MOL000098	quercetin	Cyclin-A2	P20248	CCNA2	0.151
Sargassum pallidum（SP）	MOL000098	quercetin	Cell division protein kinase 2	P24941	CDK2	0.156
Sargassum pallidum（SP）	MOL000098	quercetin	Hemoglobin subunit alpha	P69905	HBA1	0.169
Sargassum pallidum（SP）	MOL000098	quercetin	Mu-type opioid receptor	P35372	OPRM1	0.179
Sargassum pallidum（SP）	MOL000098	quercetin	Progesterone receptor	P06401	PGR	0.2
Sargassum pallidum（SP）	MOL000098	quercetin	Nuclear receptor coactivator 1	Q15788	NCOA1	0.205
Sargassum pallidum（SP）	MOL000098	quercetin	Prostaglandin G/H synthase 1	P23219	PTGS1	0.22
Sargassum pallidum（SP）	MOL000098	quercetin	Estrogen receptor beta	Q92731	ESR2	0.357

Sargassum pallidum（SP）	MOL000098	quercetin	Prostaglandin G/H synthase 2	P35354	PTGS2	0.39
Sargassum pallidum（SP）	MOL000098	quercetin	Estrogen receptor	P03372	ESR1	1
Sargassum pallidum（SP）	MOL005440	Isofucosterol	Ig kappa chain C region	P01834	IGKC	0.012
Sargassum pallidum（SP）	MOL005440	Isofucosterol	Ig gamma-1 chain C region	P01857	IGHG1	0.012
Sargassum pallidum（SP）	MOL005440	Isofucosterol	Ig gamma-2 chain C region	P01859	IGHG2	0.012
Sargassum pallidum（SP）	MOL005440	Isofucosterol	Bile salt sulfotransferase	Q06520	SULT2A1	0.02
Sargassum pallidum（SP）	MOL005440	Isofucosterol	Dehydrogenase/reductase SDR family member 8	Q8NBQ5	HSD17B11	0.02
Sargassum pallidum（SP）	MOL005440	Isofucosterol	Muscarinic acetylcholine receptor M2	P08172	CHRM2	0.025
Sargassum pallidum（SP）	MOL005440	Isofucosterol	Neuronal acetylcholine receptor subunit alpha-2	Q15822	CHRNA2	0.025
Sargassum pallidum（SP）	MOL005440	Isofucosterol	Serine/threonine-protein phosphatase 2A catalytic subunit alpha isoform	P67775	PPP2CA	0.033
Sargassum pallidum（SP）	MOL005440	Isofucosterol	Serine/threonine-protein phosphatase 2A 56 kDa regulatory subunit gamma isoform	Q13362	PPP2R5C	0.033
Sargassum pallidum（SP）	MOL005440	Isofucosterol	Microtubule-associated protein 2	P11137	MAP2	0.038
Sargassum pallidum（SP）	MOL005440	Isofucosterol	Microtubule-associated protein 1A	P78559	MAP1A	0.038
Sargassum pallidum（SP）	MOL005440	Isofucosterol	Prolactin receptor	P16471	PRLR	0.04
Sargassum pallidum（SP）	MOL005440	Isofucosterol	Gonadotropin-releasing hormone receptor	P30968	GNRHR	0.041
Sargassum pallidum（SP）	MOL005440	Isofucosterol	Gonadotropin-releasing hormone II receptor	Q96P88	GNRHR2	0.041
Sargassum pallidum（SP）	MOL005440	Isofucosterol	3 beta-hydroxysteroid dehydrogenase/Delta 5-->4-isomerase type II	P26439	HSD3B2	0.042
Sargassum pallidum（SP）	MOL005440	Isofucosterol	Corticosteroid 11-beta-dehydrogenase isozyme 1	P28845	HSD11B1	0.044
Sargassum pallidum（SP）	MOL005440	Isofucosterol	Cytosolic phospholipase A2	P47712	PLA2G4A	0.044
Sargassum pallidum（SP）	MOL005440	Isofucosterol	Retinoic acid receptor RXR-alpha	P19793	RXRA	0.055
Sargassum pallidum（SP）	MOL005440	Isofucosterol	Nuclear receptor subfamily 1 group I member 3	Q14994	NR1I3	0.055
Sargassum pallidum（SP）	MOL005440	Isofucosterol	3-oxo-5-alpha-steroid 4-dehydrogenase 1	P18405	SRD5A1	0.068
Sargassum pallidum（SP）	MOL005440	Isofucosterol	Prostaglandin G/H synthase 2	P35354	PTGS2	0.104
Sargassum pallidum（SP）	MOL005440	Isofucosterol	Aldo-keto reductase family 1 member C1	Q04828	AKR1C1	0.106
Sargassum pallidum（SP）	MOL005440	Isofucosterol	DNA polymerase kappa	Q9UBT6	POLK	0.107
Sargassum pallidum（SP）	MOL005440	Isofucosterol	NADPH oxidase organizer 1	Q8NFA2	NOXO1	0.116
Sargassum pallidum（SP）	MOL005440	Isofucosterol	Mediator of RNA polymerase II transcription subunit 1	Q15648	MED1	0.121
Sargassum pallidum（SP）	MOL005440	Isofucosterol	Nuclear receptor coactivator 5	Q9HCD5	NCOA5	0.127
Sargassum pallidum（SP）	MOL005440	Isofucosterol	Cannabinoid receptor 2	P34972	CNR2	0.138
Sargassum pallidum（SP）	MOL005440	Isofucosterol	3 beta-hydroxysteroid dehydrogenase/Delta 5-->4-isomerase type I	P14060	HSD3B1	0.142
Sargassum pallidum（SP）	MOL005440	Isofucosterol	Estrogen receptor beta	Q92731	ESR2	0.168
Sargassum pallidum（SP）	MOL005440	Isofucosterol	3-oxo-5-alpha-steroid 4-dehydrogenase 2	P31213	SRD5A2	0.19
Sargassum pallidum（SP）	MOL005440	Isofucosterol	Androgen receptor	P10275	AR	0.229
Sargassum pallidum（SP）	MOL005440	Isofucosterol	Glucocorticoid receptor	P04150	NR3C1	0.239
Sargassum pallidum（SP）	MOL005440	Isofucosterol	Estradiol 17-beta-dehydrogenase 1	P14061	HSD17B1	0.251
Sargassum pallidum（SP）	MOL005440	Isofucosterol	Nuclear receptor coactivator 1	Q15788	NCOA1	0.384
Sargassum pallidum（SP）	MOL005440	Isofucosterol	Mineralocorticoid receptor	P08235	NR3C2	0.43
Sargassum pallidum（SP）	MOL005440	Isofucosterol	Estrogen receptor	P03372	ESR1	0.789
Sargassum pallidum（SP）	MOL005440	Isofucosterol	Progesterone receptor	P06401	PGR	1
Sargassum pallidum（SP）	MOL010578	N-[(1S)-1-(benzyl)-2-[[(1S)-1-(benzyl)-2-hydroxy-ethyl]amino]-2-keto-ethyl]benzamide	Cytochrome b-c1 complex subunit 8	O14949	UQCRQ	0.034
Sargassum pallidum（SP）	MOL010578	N-[(1S)-1-(benzyl)-2-[[(1S)-1-(benzyl)-2-hydroxy-ethyl]amino]-2-keto-ethyl]benzamide	Cytochrome b-c1 complex subunit 10	O14957	UQCR11	0.034
Sargassum pallidum（SP）	MOL010578	N-[(1S)-1-(benzyl)-2-[[(1S)-1-(benzyl)-2-hydroxy-ethyl]amino]-2-keto-ethyl]benzamide	Cytochrome b	P00156	MT-CYB	0.034
Sargassum pallidum（SP）	MOL010578	N-[(1S)-1-(benzyl)-2-[[(1S)-1-(benzyl)-2-hydroxy-ethyl]amino]-2-keto-ethyl]benzamide	Cytochrome b-c1 complex subunit 6, mitochondrial	P07919	UQCRH	0.034
Sargassum pallidum（SP）	MOL010578	N-[(1S)-1-(benzyl)-2-[[(1S)-1-(benzyl)-2-hydroxy-ethyl]amino]-2-keto-ethyl]benzamide	Cytochrome c1, heme protein, mitochondrial	P08574	CYC1	0.034

Sargassum pallidum（SP）	MOL010578	N-[(1S)-1-(benzyl)-2-[[(1S)-1-(benzyl)-2-hydroxy-ethyl]amino]-2-keto-ethyl]benzamide	Cytochrome b-c1 complex subunit 2, mitochondrial	P22695	UQCRC2	0.034
Sargassum pallidum（SP）	MOL010578	N-[(1S)-1-(benzyl)-2-[[(1S)-1-(benzyl)-2-hydroxy-ethyl]amino]-2-keto-ethyl]benzamide	Ubiquinol-cytochrome-c reductase complex core protein 1, mitochondrial	P31930	UQCRC1	0.034
Sargassum pallidum（SP）	MOL010578	N-[(1S)-1-(benzyl)-2-[[(1S)-1-(benzyl)-2-hydroxy-ethyl]amino]-2-keto-ethyl]benzamide	Cytochrome b-c1 complex subunit Rieske, mitochondrial	P47985	UQCRFS1	0.034
Sargassum pallidum（SP）	MOL010578	N-[(1S)-1-(benzyl)-2-[[(1S)-1-(benzyl)-2-hydroxy-ethyl]amino]-2-keto-ethyl]benzamide	Cytochrome b-c1 complex subunit 9	Q9UDW1	UQCR10	0.034
Sargassum pallidum（SP）	MOL010578	N-[(1S)-1-(benzyl)-2-[[(1S)-1-(benzyl)-2-hydroxy-ethyl]amino]-2-keto-ethyl]benzamide	Potassium channel subfamily K member 1	O00180	KCNK1	0.346
Sargassum pallidum（SP）	MOL010578	N-[(1S)-1-(benzyl)-2-[[(1S)-1-(benzyl)-2-hydroxy-ethyl]amino]-2-keto-ethyl]benzamide	Potassium channel subfamily K member 6	Q9Y257	KCNK6	0.346
Sargassum pallidum（SP）	MOL010578	N-[(1S)-1-(benzyl)-2-[[(1S)-1-(benzyl)-2-hydroxy-ethyl]amino]-2-keto-ethyl]benzamide	Platelet glycoprotein IX	P14770	GP9	0.354
Sargassum pallidum（SP）	MOL010578	N-[(1S)-1-(benzyl)-2-[[(1S)-1-(benzyl)-2-hydroxy-ethyl]amino]-2-keto-ethyl]benzamide	Muscarinic acetylcholine receptor M2	P08172	CHRM2	0.649
Sargassum pallidum（SP）	MOL010578	N-[(1S)-1-(benzyl)-2-[[(1S)-1-(benzyl)-2-hydroxy-ethyl]amino]-2-keto-ethyl]benzamide	Muscarinic acetylcholine receptor M4	P08173	CHRM4	0.649
Sargassum pallidum（SP）	MOL010578	N-[(1S)-1-(benzyl)-2-[[(1S)-1-(benzyl)-2-hydroxy-ethyl]amino]-2-keto-ethyl]benzamide	cGMP-specific 3',5'-cyclic phosphodiesterase	O76074	PDE5A	0.7
Sargassum pallidum（SP）	MOL010578	N-[(1S)-1-(benzyl)-2-[[(1S)-1-(benzyl)-2-hydroxy-ethyl]amino]-2-keto-ethyl]benzamide	Sodium channel protein type 5 subunit alpha	Q14524	SCN5A	0.743
Sargassum pallidum（SP）	MOL010578	N-[(1S)-1-(benzyl)-2-[[(1S)-1-(benzyl)-2-hydroxy-ethyl]amino]-2-keto-ethyl]benzamide	Mitogen-activated protein kinase 14	Q16539	MAPK14	0.813
Sargassum pallidum（SP）	MOL010578	N-[(1S)-1-(benzyl)-2-[[(1S)-1-(benzyl)-2-hydroxy-ethyl]amino]-2-keto-ethyl]benzamide	Muscarinic acetylcholine receptor M1	P11229	CHRM1	1
Sargassum pallidum（SP）	MOL010580	Diglycol dibenzoate	Nitric oxide synthase, inducible	P35228	NOS2	0.965
Sargassum pallidum（SP）	MOL010580	Diglycol dibenzoate	L-amino-acid oxidase	Q96RQ9	IL4I1	0.995
Sargassum pallidum（SP）	MOL010580	Diglycol dibenzoate	Serine/threonine-protein kinase 6	O14965	AURKA	1
Polygonatum sibiricum(PGS)	MOL000358	beta-sitosterol	Retinoic acid receptor RXR-gamma	P48443	RXRG	0.01
Polygonatum sibiricum(PGS)	MOL000358	beta-sitosterol	Nuclear receptor 0B1	P51843	NR0B1	0.01
Polygonatum sibiricum(PGS)	MOL000358	beta-sitosterol	Retinoic acid-induced protein 3	Q8NFJ5	GPRC5A	0.01
Polygonatum sibiricum(PGS)	MOL000358	beta-sitosterol	Retinoic acid receptor alpha	P10276	RARA	0.023
Polygonatum sibiricum(PGS)	MOL000358	beta-sitosterol	Retinoic acid receptor beta	P10826	RARB	0.023
Polygonatum sibiricum(PGS)	MOL000358	beta-sitosterol	Retinal dehydrogenase 2	O94788	ALDH1A2	0.029
Polygonatum sibiricum(PGS)	MOL000358	beta-sitosterol	Retinal dehydrogenase 1	P00352	ALDH1A1	0.029
Polygonatum sibiricum(PGS)	MOL000358	beta-sitosterol	Potassium channel subfamily K member 1	O00180	KCNK1	0.036
Polygonatum sibiricum(PGS)	MOL000358	beta-sitosterol	Sodium channel protein type 5 subunit alpha	Q14524	SCN5A	0.036
Polygonatum sibiricum(PGS)	MOL000358	beta-sitosterol	Potassium channel subfamily K member 6	Q9Y257	KCNK6	0.036
Polygonatum sibiricum(PGS)	MOL000358	beta-sitosterol	Serine/threonine-protein phosphatase 2A 65 kDa regulatory subunit A alpha isoform	P30153	PPP2R1A	0.038
Polygonatum sibiricum(PGS)	MOL000358	beta-sitosterol	Serine/threonine-protein phosphatase PP1-alpha catalytic subunit	P62136	PPP1CA	0.038
Polygonatum sibiricum(PGS)	MOL000358	beta-sitosterol	Ig kappa chain C region	P01834	IGKC	0.043
Polygonatum sibiricum(PGS)	MOL000358	beta-sitosterol	Ig gamma-1 chain C region	P01857	IGHG1	0.043
Polygonatum sibiricum(PGS)	MOL000358	beta-sitosterol	Ig gamma-2 chain C region	P01859	IGHG2	0.043
Polygonatum sibiricum(PGS)	MOL000358	beta-sitosterol	Nuclear receptor subfamily 1 group I member 3	Q14994	NR1I3	0.05
Polygonatum sibiricum(PGS)	MOL000358	beta-sitosterol	Retinoic acid receptor gamma-1	P13631	RARG	0.052
Polygonatum sibiricum(PGS)	MOL000358	beta-sitosterol	Retinoic acid receptor RXR-beta	P28702	RXRB	0.052
Polygonatum sibiricum(PGS)	MOL000358	beta-sitosterol	Bile salt sulfotransferase	Q06520	SULT2A1	0.056
Polygonatum sibiricum(PGS)	MOL000358	beta-sitosterol	Dehydrogenase/reductase SDR family member 8	Q8NBQ5	HSD17B11	0.056
Polygonatum sibiricum(PGS)	MOL000358	beta-sitosterol	Platelet glycoprotein IX	P14770	GP9	0.063
Polygonatum sibiricum(PGS)	MOL000358	beta-sitosterol	Phospholipase A2	P04054	PLA2G1B	0.069
Polygonatum sibiricum(PGS)	MOL000358	beta-sitosterol	Phospholipase A2, membrane associated	P14555	PLA2G2A	0.069
Polygonatum sibiricum(PGS)	MOL000358	beta-sitosterol	3-oxo-5-alpha-steroid 4-dehydrogenase 1	P18405	SRD5A1	0.074

Polygonatum sibiricum(PGS)	MOL000358	beta-sitosterol	3-oxo-5-alpha-steroid 4-dehydrogenase 2	P31213	SRD5A2	0.074
Polygonatum sibiricum(PGS)	MOL000358	beta-sitosterol	3 beta-hydroxysteroid dehydrogenase/Delta 5-->4-isomerase type II	P26439	HSD3B2	0.079
Polygonatum sibiricum(PGS)	MOL000358	beta-sitosterol	Microtubule-associated protein 2	P11137	MAP2	0.08
Polygonatum sibiricum(PGS)	MOL000358	beta-sitosterol	Microtubule-associated protein 1A	P78559	MAP1A	0.08
Polygonatum sibiricum(PGS)	MOL000358	beta-sitosterol	Gonadotropin-releasing hormone receptor	P30968	GNRHR	0.082
Polygonatum sibiricum(PGS)	MOL000358	beta-sitosterol	Gonadotropin-releasing hormone II receptor	Q96P88	GNRHR2	0.082
Polygonatum sibiricum(PGS)	MOL000358	beta-sitosterol	Corticosteroid 11-beta-dehydrogenase isozyme 1	P28845	HSD11B1	0.084
Polygonatum sibiricum(PGS)	MOL000358	beta-sitosterol	Cytosolic phospholipase A2	P47712	PLA2G4A	0.084
Polygonatum sibiricum(PGS)	MOL000358	beta-sitosterol	Retinoic acid receptor RXR-alpha	P19793	RXRA	0.092
Polygonatum sibiricum(PGS)	MOL000358	beta-sitosterol	Serine/threonine-protein phosphatase 2A catalytic subunit alpha isoform	P67775	PPP2CA	0.095
Polygonatum sibiricum(PGS)	MOL000358	beta-sitosterol	Serine/threonine-protein phosphatase 2A 56 kDa regulatory subunit gamma isoform	Q13362	PPP2R5C	0.095
Polygonatum sibiricum(PGS)	MOL000358	beta-sitosterol	Sodium-dependent serotonin transporter	P31645	SLC6A4	0.146
Polygonatum sibiricum(PGS)	MOL000358	beta-sitosterol	Aldo-keto reductase family 1 member C1	Q04828	AKR1C1	0.147
Polygonatum sibiricum(PGS)	MOL000358	beta-sitosterol	Prostaglandin G/H synthase 2	P35354	PTGS2	0.15
Polygonatum sibiricum(PGS)	MOL000358	beta-sitosterol	Estrogen receptor beta	Q92731	ESR2	0.163
Polygonatum sibiricum(PGS)	MOL000358	beta-sitosterol	Mediator of RNA polymerase II transcription subunit 1	Q15648	MED1	0.164
Polygonatum sibiricum(PGS)	MOL000358	beta-sitosterol	Estradiol 17-beta-dehydrogenase 1	P14061	HSD17B1	0.173
Polygonatum sibiricum(PGS)	MOL000358	beta-sitosterol	3 beta-hydroxysteroid dehydrogenase/Delta 5-->4-isomerase type I	P14060	HSD3B1	0.175
Polygonatum sibiricum(PGS)	MOL000358	beta-sitosterol	Nuclear receptor coactivator 5	Q9HCD5	NCOA5	0.178
Polygonatum sibiricum(PGS)	MOL000358	beta-sitosterol	Androgen receptor	P10275	AR	0.184
Polygonatum sibiricum(PGS)	MOL000358	beta-sitosterol	Glucocorticoid receptor	P04150	NR3C1	0.187
Polygonatum sibiricum(PGS)	MOL000358	beta-sitosterol	Cannabinoid receptor 2	P34972	CNR2	0.191
Polygonatum sibiricum(PGS)	MOL000358	beta-sitosterol	Nuclear receptor coactivator 1	Q15788	NCOA1	0.296
Polygonatum sibiricum(PGS)	MOL000358	beta-sitosterol	Mineralocorticoid receptor	P08235	NR3C2	0.39
Polygonatum sibiricum(PGS)	MOL000358	beta-sitosterol	Estrogen receptor	P03372	ESR1	0.971
Polygonatum sibiricum(PGS)	MOL000358	beta-sitosterol	Progesterone receptor	P06401	PGR	1
Polygonatum sibiricum(PGS)	MOL000359	sitosterol	Retinoic acid receptor RXR-alpha	P19793	RXRA	0.016
Polygonatum sibiricum(PGS)	MOL000359	sitosterol	Nuclear receptor subfamily 1 group I member 3	Q14994	NR1I3	0.016
Polygonatum sibiricum(PGS)	MOL000359	sitosterol	Elongation factor Tu GTP-binding domain-containing protein 1	Q7Z2Z2	EFL1	0.026
Polygonatum sibiricum(PGS)	MOL000359	sitosterol	Potassium channel subfamily K member 1	O00180	KCNK1	0.032
Polygonatum sibiricum(PGS)	MOL000359	sitosterol	D-HSCDK2	O75100	CA11	0.032
Polygonatum sibiricum(PGS)	MOL000359	sitosterol	Cell division control protein 2 homolog	P06493	CDK1	0.032
Polygonatum sibiricum(PGS)	MOL000359	sitosterol	Cell division protein kinase 5	Q00535	CDK5	0.032
Polygonatum sibiricum(PGS)	MOL000359	sitosterol	Sodium channel protein type 5 subunit alpha	Q14524	SCN5A	0.032
Polygonatum sibiricum(PGS)	MOL000359	sitosterol	Potassium channel subfamily K member 6	Q9Y257	KCNK6	0.032
Polygonatum sibiricum(PGS)	MOL000359	sitosterol	Neuronal acetylcholine receptor subunit alpha-3	P32297	CHRNA3	0.033
Polygonatum sibiricum(PGS)	MOL000359	sitosterol	Neuronal acetylcholine receptor subunit alpha-7	P36544	CHRNA7	0.033
Polygonatum sibiricum(PGS)	MOL000359	sitosterol	Neuronal acetylcholine receptor subunit alpha-4	P43681	CHRNA4	0.033
Polygonatum sibiricum(PGS)	MOL000359	sitosterol	Phosphatidylinositol 3-kinase regulatory subunit alpha	P27986	PIK3R1	0.034
Polygonatum sibiricum(PGS)	MOL000359	sitosterol	Phosphatidylinositol-4,5-bisphosphate 3-kinase catalytic subunit gamma isoform	P48736	PIK3CG	0.034
Polygonatum sibiricum(PGS)	MOL000359	sitosterol	Serine/threonine-protein kinase PLK1	P53350	PLK1	0.034
Polygonatum sibiricum(PGS)	MOL000359	sitosterol	Dehydrogenase/reductase SDR family member 8	Q8NBQ5	HSD17B11	0.041
Polygonatum sibiricum(PGS)	MOL000359	sitosterol	Ig kappa chain C region	P01834	IGKC	0.042
Polygonatum sibiricum(PGS)	MOL000359	sitosterol	Ig gamma-1 chain C region	P01857	IGHG1	0.042
Polygonatum sibiricum(PGS)	MOL000359	sitosterol	Ig gamma-2 chain C region	P01859	IGHG2	0.042
Polygonatum sibiricum(PGS)	MOL000359	sitosterol	Platelet glycoprotein IX	P14770	GP9	0.054
Polygonatum sibiricum(PGS)	MOL000359	sitosterol	Phospholipase A2	P04054	PLA2G1B	0.057

Polygonatum sibiricum(PGS)	MOL000359	sitosterol	Phospholipase A2, membrane associated	P14555	PLA2G2A	0.057
Polygonatum sibiricum(PGS)	MOL000359	sitosterol	Annexin A1	P04083	ANXA1	0.062
Polygonatum sibiricum(PGS)	MOL000359	sitosterol	Nuclear receptor 0B1	P51843	NR0B1	0.062
Polygonatum sibiricum(PGS)	MOL000359	sitosterol	3 beta-hydroxysteroid dehydrogenase/Delta 5-->4-isomerase type II	P26439	HSD3B2	0.064
Polygonatum sibiricum(PGS)	MOL000359	sitosterol	Cytosolic phospholipase A2	P47712	PLA2G4A	0.066
Polygonatum sibiricum(PGS)	MOL000359	sitosterol	Corticosteroid 11-beta-dehydrogenase isozyme 1	P28845	HSD11B1	0.067
Polygonatum sibiricum(PGS)	MOL000359	sitosterol	3-oxo-5-alpha-steroid 4-dehydrogenase 2	P31213	SRD5A2	0.067
Polygonatum sibiricum(PGS)	MOL000359	sitosterol	Microtubule-associated protein 2	P11137	MAP2	0.068
Polygonatum sibiricum(PGS)	MOL000359	sitosterol	Prolactin receptor	P16471	PRLR	0.068
Polygonatum sibiricum(PGS)	MOL000359	sitosterol	Gonadotropin-releasing hormone receptor	P30968	GNRHR	0.068
Polygonatum sibiricum(PGS)	MOL000359	sitosterol	Microtubule-associated protein 1A	P78559	MAP1A	0.068
Polygonatum sibiricum(PGS)	MOL000359	sitosterol	Gonadotropin-releasing hormone II receptor	Q96P88	GNRHR2	0.068
Polygonatum sibiricum(PGS)	MOL000359	sitosterol	Nuclear receptor coactivator 1	Q15788	NCOA1	0.069
Polygonatum sibiricum(PGS)	MOL000359	sitosterol	Bile salt sulfotransferase	Q06520	SULT2A1	0.091
Polygonatum sibiricum(PGS)	MOL000359	sitosterol	Prostaglandin G/H synthase 2	P35354	PTGS2	0.115
Polygonatum sibiricum(PGS)	MOL000359	sitosterol	DNA polymerase kappa	Q9UBT6	POLK	0.115
Polygonatum sibiricum(PGS)	MOL000359	sitosterol	Nuclear receptor coactivator 2	Q15596	NCOA2	0.116
Polygonatum sibiricum(PGS)	MOL000359	sitosterol	Aldo-keto reductase family 1 member C1	Q04828	AKR1C1	0.125
Polygonatum sibiricum(PGS)	MOL000359	sitosterol	ATP-binding cassette transporter sub-family C member 8	Q09428	ABCC8	0.127
Polygonatum sibiricum(PGS)	MOL000359	sitosterol	3 beta-hydroxysteroid dehydrogenase/Delta 5-->4-isomerase type I	P14060	HSD3B1	0.128
Polygonatum sibiricum(PGS)	MOL000359	sitosterol	Nitric-oxide synthase, endothelial	P29474	NOS3	0.134
Polygonatum sibiricum(PGS)	MOL000359	sitosterol	Cannabinoid receptor 2	P34972	CNR2	0.136
Polygonatum sibiricum(PGS)	MOL000359	sitosterol	3-oxo-5-alpha-steroid 4-dehydrogenase 1	P18405	SRD5A1	0.145
Polygonatum sibiricum(PGS)	MOL000359	sitosterol	Nuclear receptor coactivator 5	Q9HCD5	NCOA5	0.15
Polygonatum sibiricum(PGS)	MOL000359	sitosterol	Estradiol 17-beta-dehydrogenase 1	P14061	HSD17B1	0.221
Polygonatum sibiricum(PGS)	MOL000359	sitosterol	Androgen receptor	P10275	AR	0.224
Polygonatum sibiricum(PGS)	MOL000359	sitosterol	Estrogen receptor beta	Q92731	ESR2	0.246
Polygonatum sibiricum(PGS)	MOL000359	sitosterol	Glucocorticoid receptor	P04150	NR3C1	0.288
Polygonatum sibiricum(PGS)	MOL000359	sitosterol	Mineralocorticoid receptor	P08235	NR3C2	0.376
Polygonatum sibiricum(PGS)	MOL000359	sitosterol	Estrogen receptor	P03372	ESR1	0.93
Polygonatum sibiricum(PGS)	MOL000359	sitosterol	Progesterone receptor	P06401	PGR	1
Polygonatum sibiricum(PGS)	MOL000546	diosgenin	Elongation factor Tu GTP-binding domain-containing protein 1	Q7Z2Z2	EFL1	0.012
Polygonatum sibiricum(PGS)	MOL000546	diosgenin	Muscarinic acetylcholine receptor M2	P08172	CHRM2	0.016
Polygonatum sibiricum(PGS)	MOL000546	diosgenin	Ribosyldihydronicotinamide dehydrogenase [quinone]	P16083	NQO2	0.016
Polygonatum sibiricum(PGS)	MOL000546	diosgenin	Prostaglandin G/H synthase 1	P23219	PTGS1	0.016
Polygonatum sibiricum(PGS)	MOL000546	diosgenin	Neuronal acetylcholine receptor subunit alpha-3	P32297	CHRNA3	0.016
Polygonatum sibiricum(PGS)	MOL000546	diosgenin	Neuronal acetylcholine receptor subunit alpha-7	P36544	CHRNA7	0.016
Polygonatum sibiricum(PGS)	MOL000546	diosgenin	Neuronal acetylcholine receptor subunit alpha-4	P43681	CHRNA4	0.016
Polygonatum sibiricum(PGS)	MOL000546	diosgenin	Casein kinase II subunit alpha	P68400	CSNK2A1	0.016
Polygonatum sibiricum(PGS)	MOL000546	diosgenin	Opioid receptor, sigma 1	Q5T1J1	SIGMAR1	0.016
Polygonatum sibiricum(PGS)	MOL000546	diosgenin	Glutamate [NMDA] receptor subunit 3A	Q8TCU5	GRIN3A	0.016
Polygonatum sibiricum(PGS)	MOL000546	diosgenin	Sigma 1-type opioid receptor	Q99720	SIGMAR1	0.016
Polygonatum sibiricum(PGS)	MOL000546	diosgenin	Potassium channel subfamily K member 1	O00180	KCNK1	0.019
Polygonatum sibiricum(PGS)	MOL000546	diosgenin	Sodium channel protein type 5 subunit alpha	Q14524	SCN5A	0.019
Polygonatum sibiricum(PGS)	MOL000546	diosgenin	Potassium channel subfamily K member 6	Q9Y257	KCNK6	0.019
Polygonatum sibiricum(PGS)	MOL000546	diosgenin	Nuclear receptor coactivator 2	Q15596	NCOA2	0.02
Polygonatum sibiricum(PGS)	MOL000546	diosgenin	Ig kappa chain C region	P01834	IGKC	0.021

Polygonatum sibiricum(PGS)	MOL000546	diosgenin	Ig gamma-1 chain C region	P01857	IGHG1	0.021
Polygonatum sibiricum(PGS)	MOL000546	diosgenin	Ig gamma-2 chain C region	P01859	IGHG2	0.021
Polygonatum sibiricum(PGS)	MOL000546	diosgenin	Retinoic acid receptor alpha	P10276	RARA	0.021
Polygonatum sibiricum(PGS)	MOL000546	diosgenin	Retinoic acid receptor beta	P10826	RARB	0.021
Polygonatum sibiricum(PGS)	MOL000546	diosgenin	Retinoic acid receptor gamma-1	P13631	RARG	0.021
Polygonatum sibiricum(PGS)	MOL000546	diosgenin	Retinoic acid receptor RXR-gamma	P48443	RXRG	0.021
Polygonatum sibiricum(PGS)	MOL000546	diosgenin	Dehydrogenase/reductase SDR family member 8	Q8NBQ5	HSD17B11	0.023
Polygonatum sibiricum(PGS)	MOL000546	diosgenin	Nuclear receptor subfamily 1 group I member 3	Q14994	NR1I3	0.026
Polygonatum sibiricum(PGS)	MOL000546	diosgenin	Serine/threonine-protein phosphatase 2A catalytic subunit alpha isoform	P67775	PPP2CA	0.031
Polygonatum sibiricum(PGS)	MOL000546	diosgenin	Serine/threonine-protein phosphatase 2A 56 kDa regulatory subunit gamma isoform	Q13362	PPP2R5C	0.031
Polygonatum sibiricum(PGS)	MOL000546	diosgenin	Platelet glycoprotein IX	P14770	GP9	0.033
Polygonatum sibiricum(PGS)	MOL000546	diosgenin	3 beta-hydroxysteroid dehydrogenase/Delta 5-->4-isomerase type II	P26439	HSD3B2	0.041
Polygonatum sibiricum(PGS)	MOL000546	diosgenin	Annexin A1	P04083	ANXA1	0.042
Polygonatum sibiricum(PGS)	MOL000546	diosgenin	Nuclear receptor 0B1	P51843	NR0B1	0.042
Polygonatum sibiricum(PGS)	MOL000546	diosgenin	Neuronal acetylcholine receptor subunit alpha-2	Q15822	CHRNA2	0.042
Polygonatum sibiricum(PGS)	MOL000546	diosgenin	Cytosolic phospholipase A2	P47712	PLA2G4A	0.044
Polygonatum sibiricum(PGS)	MOL000546	diosgenin	Microtubule-associated protein 2	P11137	MAP2	0.045
Polygonatum sibiricum(PGS)	MOL000546	diosgenin	Prolactin receptor	P16471	PRLR	0.045
Polygonatum sibiricum(PGS)	MOL000546	diosgenin	Gonadotropin-releasing hormone receptor	P30968	GNRHR	0.045
Polygonatum sibiricum(PGS)	MOL000546	diosgenin	Microtubule-associated protein 1A	P78559	MAP1A	0.045
Polygonatum sibiricum(PGS)	MOL000546	diosgenin	Gonadotropin-releasing hormone II receptor	Q96P88	GNRHR2	0.045
Polygonatum sibiricum(PGS)	MOL000546	diosgenin	Bile salt sulfotransferase	Q06520	SULT2A1	0.055
Polygonatum sibiricum(PGS)	MOL000546	diosgenin	S-methyl-5-thioadenosine phosphorylase	Q13126	MTAP	0.068
Polygonatum sibiricum(PGS)	MOL000546	diosgenin	Dihydrofolate reductase	P00374	DHFR	0.069
Polygonatum sibiricum(PGS)	MOL000546	diosgenin	cAMP-specific 3',5'-cyclic phosphodiesterase 4A	P27815	PDE4A	0.07
Polygonatum sibiricum(PGS)	MOL000546	diosgenin	NADPH oxidase organizer 1	Q8NFA2	NOXO1	0.072
Polygonatum sibiricum(PGS)	MOL000546	diosgenin	DNA polymerase kappa	Q9UBT6	POLK	0.072
Polygonatum sibiricum(PGS)	MOL000546	diosgenin	Mediator of RNA polymerase II transcription subunit 1	Q15648	MED1	0.074
Polygonatum sibiricum(PGS)	MOL000546	diosgenin	Cannabinoid receptor 2	P34972	CNR2	0.08
Polygonatum sibiricum(PGS)	MOL000546	diosgenin	Nuclear receptor coactivator 5	Q9HCD5	NCOA5	0.08
Polygonatum sibiricum(PGS)	MOL000546	diosgenin	3 beta-hydroxysteroid dehydrogenase/Delta 5-->4-isomerase type I	P14060	HSD3B1	0.084
Polygonatum sibiricum(PGS)	MOL000546	diosgenin	Retinoic acid receptor RXR-beta	P28702	RXRB	0.085
Polygonatum sibiricum(PGS)	MOL000546	diosgenin	Retinoic acid receptor RXR-alpha	P19793	RXRA	0.091
Polygonatum sibiricum(PGS)	MOL000546	diosgenin	3-oxo-5-alpha-steroid 4-dehydrogenase 1	P18405	SRD5A1	0.103
Polygonatum sibiricum(PGS)	MOL000546	diosgenin	3-oxo-5-alpha-steroid 4-dehydrogenase 2	P31213	SRD5A2	0.105
Polygonatum sibiricum(PGS)	MOL000546	diosgenin	Nuclear receptor coactivator 1	Q15788	NCOA1	0.105
Polygonatum sibiricum(PGS)	MOL000546	diosgenin	Estradiol 17-beta-dehydrogenase 1	P14061	HSD17B1	0.141
Polygonatum sibiricum(PGS)	MOL000546	diosgenin	Mineralocorticoid receptor	P08235	NR3C2	0.158
Polygonatum sibiricum(PGS)	MOL000546	diosgenin	Androgen receptor	P10275	AR	0.213
Polygonatum sibiricum(PGS)	MOL000546	diosgenin	Glucocorticoid receptor	P04150	NR3C1	0.257
Polygonatum sibiricum(PGS)	MOL000546	diosgenin	Estrogen receptor beta	Q92731	ESR2	0.31
Polygonatum sibiricum(PGS)	MOL000546	diosgenin	Progesterone receptor	P06401	PGR	0.772
Polygonatum sibiricum(PGS)	MOL000546	diosgenin	Estrogen receptor	P03372	ESR1	1
Polygonatum sibiricum(PGS)	MOL001792	DFV	Interleukin-3	P08700	IL3	0.011
Polygonatum sibiricum(PGS)	MOL001792	DFV	Protein S100-A12	P80511	S100A12	0.011
Polygonatum sibiricum(PGS)	MOL001792	DFV	Protein S100-A13	Q99584	S100A13	0.011
Polygonatum sibiricum(PGS)	MOL001792	DFV	Troponin C, slow skeletal and cardiac muscles	P63316	TNNC1	0.012

Polygonatum sibiricum(PGS)	MOL001792	DFV	ATP-sensitive inward rectifier potassium channel 11	Q14654	KCNJ11	0.012
Polygonatum sibiricum(PGS)	MOL001792	DFV	ATP-sensitive inward rectifier potassium channel 8	Q15842	KCNJ8	0.012
Polygonatum sibiricum(PGS)	MOL001792	DFV	Epidermal growth factor receptor	P00533	EGFR	0.014
Polygonatum sibiricum(PGS)	MOL001792	DFV	Cell division protein kinase 4	P11802	CDK4	0.014
Polygonatum sibiricum(PGS)	MOL001792	DFV	Glycogen synthase kinase-3 beta	P49841	GSK3B	0.014
Polygonatum sibiricum(PGS)	MOL001792	DFV	Cell division protein kinase 7	P50613	CDK7	0.014
Polygonatum sibiricum(PGS)	MOL001792	DFV	Cell division protein kinase 9	P50750	CDK9	0.014
Polygonatum sibiricum(PGS)	MOL001792	DFV	Cell division protein kinase 6	Q00534	CDK6	0.014
Polygonatum sibiricum(PGS)	MOL001792	DFV	Prostaglandin reductase 1	Q14914	PTGR1	0.014
Polygonatum sibiricum(PGS)	MOL001792	DFV	Cyclin-dependent kinase 5 activator 1	Q15078	CDK5R1	0.014
Polygonatum sibiricum(PGS)	MOL001792	DFV	Serine/threonine-protein kinase 17B	O94768	STK17B	0.015
Polygonatum sibiricum(PGS)	MOL001792	DFV	Dihydrofolate reductase	P00374	DHFR	0.015
Polygonatum sibiricum(PGS)	MOL001792	DFV	Stromelysin-1	P08254	MMP3	0.015
Polygonatum sibiricum(PGS)	MOL001792	DFV	Stromelysin-2	P09238	MMP10	0.015
Polygonatum sibiricum(PGS)	MOL001792	DFV	Proto-oncogene serine/threonine-protein kinase Pim-1	P11309	PIM1	0.015
Polygonatum sibiricum(PGS)	MOL001792	DFV	ATP synthase subunit gamma, mitochondrial	P36542	ATP5F1C	0.015
Polygonatum sibiricum(PGS)	MOL001792	DFV	Amiloride-sensitive sodium channel subunit alpha	P37088	SCNN1A	0.015
Polygonatum sibiricum(PGS)	MOL001792	DFV	Macrophage metalloelastase	P39900	MMP12	0.015
Polygonatum sibiricum(PGS)	MOL001792	DFV	Amiloride-sensitive sodium channel subunit beta	P51168	SCNN1B	0.015
Polygonatum sibiricum(PGS)	MOL001792	DFV	Amiloride-sensitive sodium channel subunit gamma	P51170	SCNN1G	0.015
Polygonatum sibiricum(PGS)	MOL001792	DFV	UDP-glucuronosyltransferase 3A1	Q6NUS8	UGT3A1	0.015
Polygonatum sibiricum(PGS)	MOL001792	DFV	Phenylalanine-4-hydroxylase	P00439	PAH	0.016
Polygonatum sibiricum(PGS)	MOL001792	DFV	Beta-3 adrenergic receptor	P13945	ADRB3	0.016
Polygonatum sibiricum(PGS)	MOL001792	DFV	Tyrosine 3-monooxygenase	P07101	TH	0.017
Polygonatum sibiricum(PGS)	MOL001792	DFV	Retinoic acid receptor alpha	P10276	RARA	0.017
Polygonatum sibiricum(PGS)	MOL001792	DFV	Retinoic acid receptor beta	P10826	RARB	0.017
Polygonatum sibiricum(PGS)	MOL001792	DFV	Retinoic acid receptor gamma-1	P13631	RARG	0.017
Polygonatum sibiricum(PGS)	MOL001792	DFV	5-hydroxytryptamine 1D receptor	P28221	HTR1D	0.017
Polygonatum sibiricum(PGS)	MOL001792	DFV	5-hydroxytryptamine 2B receptor	P41595	HTR2B	0.017
Polygonatum sibiricum(PGS)	MOL001792	DFV	Retinoic acid receptor RXR-gamma	P48443	RXRG	0.017
Polygonatum sibiricum(PGS)	MOL001792	DFV	Tyrosyl-tRNA synthetase, mitochondrial	Q9Y2Z4	YARS2	0.017
Polygonatum sibiricum(PGS)	MOL001792	DFV	5-hydroxytryptamine 6 receptor	P50406	HTR6	0.018
Polygonatum sibiricum(PGS)	MOL001792	DFV	Aryl hydrocarbon receptor	P35869	AHR	0.019
Polygonatum sibiricum(PGS)	MOL001792	DFV	Dihydroorotate dehydrogenase, mitochondrial	Q02127	DHODH	0.019
Polygonatum sibiricum(PGS)	MOL001792	DFV	Group IIE secretory phospholipase A2	Q9NZK7	PLA2G2E	0.02
Polygonatum sibiricum(PGS)	MOL001792	DFV	Inhibitor of nuclear factor kappa-B kinase subunit beta	O14920	IKBKB	0.021
Polygonatum sibiricum(PGS)	MOL001792	DFV	Muscarinic acetylcholine receptor M3	P20309	CHRM3	0.021
Polygonatum sibiricum(PGS)	MOL001792	DFV	Cystine/glutamate transporter	Q9UPY5	SLC7A11	0.021
Polygonatum sibiricum(PGS)	MOL001792	DFV	Ribosyldihydronicotinamide dehydrogenase [quinone]	P16083	NQO2	0.022
Polygonatum sibiricum(PGS)	MOL001792	DFV	Carbonic anhydrase 4	P22748	CA4	0.022
Polygonatum sibiricum(PGS)	MOL001792	DFV	Sodium/potassium-transporting ATPase gamma chain	P54710	FXYD2	0.022
Polygonatum sibiricum(PGS)	MOL001792	DFV	Guanine nucleotide-binding protein G(s) subunit alpha isoforms short	P63092	GNAS	0.023
Polygonatum sibiricum(PGS)	MOL001792	DFV	Adenylate cyclase type 2	Q08462	ADCY2	0.023
Polygonatum sibiricum(PGS)	MOL001792	DFV	Casein kinase I isoform gamma-2	P78368	CSNK1G2	0.024
Polygonatum sibiricum(PGS)	MOL001792	DFV	Serine/threonine-protein kinase haspin	Q8TF76	HASPIN	0.024
Polygonatum sibiricum(PGS)	MOL001792	DFV	RAC-beta serine/threonine-protein kinase	P31751	AKT2	0.025
Polygonatum sibiricum(PGS)	MOL001792	DFV	Sepiapterin reductase	P35270	SPR	0.025

Polygonatum sibiricum(PGS)	MOL001792	DFV	6-pyruvoyl tetrahydrobiopterin synthase	Q03393	PTS	0.025
Polygonatum sibiricum(PGS)	MOL001792	DFV	Rho-associated protein kinase 1	Q13464	ROCK1	0.025
Polygonatum sibiricum(PGS)	MOL001792	DFV	cGMP-inhibited 3',5'-cyclic phosphodiesterase A	Q14432	PDE3A	0.026
Polygonatum sibiricum(PGS)	MOL001792	DFV	cAMP and cAMP-inhibited cGMP 3',5'-cyclic phosphodiesterase 10A	Q9Y233	PDE10A	0.026
Polygonatum sibiricum(PGS)	MOL001792	DFV	Potassium channel subfamily K member 1	O00180	KCNK1	0.027
Polygonatum sibiricum(PGS)	MOL001792	DFV	cGMP-specific 3',5'-cyclic phosphodiesterase	O76074	PDE5A	0.027
Polygonatum sibiricum(PGS)	MOL001792	DFV	Proto-oncogene tyrosine-protein kinase LCK	P06239	LCK	0.027
Polygonatum sibiricum(PGS)	MOL001792	DFV	Tyrosine-protein kinase Lyn	P07948	LYN	0.027
Polygonatum sibiricum(PGS)	MOL001792	DFV	Adenosine A2a receptor	P29274	ADORA2A	0.027
Polygonatum sibiricum(PGS)	MOL001792	DFV	Adenosine A1 receptor	P30542	ADORA1	0.027
Polygonatum sibiricum(PGS)	MOL001792	DFV	Platelet glycoprotein IX	P14770	GP9	0.028
Polygonatum sibiricum(PGS)	MOL001792	DFV	Neuronal acetylcholine receptor subunit alpha-7	P36544	CHRNA7	0.028
Polygonatum sibiricum(PGS)	MOL001792	DFV	Neuronal acetylcholine receptor subunit alpha-4	P43681	CHRNA4	0.028
Polygonatum sibiricum(PGS)	MOL001792	DFV	Gamma-aminobutyric-acid receptor subunit alpha-4	P48169	GABRA4	0.028
Polygonatum sibiricum(PGS)	MOL001792	DFV	Glutamate receptor, ionotropic kainate 2	Q13002	GRIK2	0.028
Polygonatum sibiricum(PGS)	MOL001792	DFV	Gamma-aminobutyric-acid receptor subunit alpha-6	Q16445	GABRA6	0.028
Polygonatum sibiricum(PGS)	MOL001792	DFV	Oxysterols receptor LXR-beta	P55055	NR1H2	0.031
Polygonatum sibiricum(PGS)	MOL001792	DFV	Nuclear receptor coactivator 2	Q15596	NCOA2	0.031
Polygonatum sibiricum(PGS)	MOL001792	DFV	Macrophage migration inhibitory factor	P14174	MIF	0.032
Polygonatum sibiricum(PGS)	MOL001792	DFV	D(4) dopamine receptor	P21917	DRD4	0.032
Polygonatum sibiricum(PGS)	MOL001792	DFV	DNA-(apurinic or apyrimidinic site) lyase	P27695	APEX1	0.032
Polygonatum sibiricum(PGS)	MOL001792	DFV	5-hydroxytryptamine 1B receptor	P28222	HTR1B	0.032
Polygonatum sibiricum(PGS)	MOL001792	DFV	Peptidyl-prolyl cis-trans isomerase, mitochondrial	P30405	PPIF	0.032
Polygonatum sibiricum(PGS)	MOL001792	DFV	Urokinase-type plasminogen activator	P00749	PLAU	0.033
Polygonatum sibiricum(PGS)	MOL001792	DFV	Muscarinic acetylcholine receptor M4	P08173	CHRM4	0.038
Polygonatum sibiricum(PGS)	MOL001792	DFV	5-hydroxytryptamine 3 receptor	P46098	HTR3A	0.039
Polygonatum sibiricum(PGS)	MOL001792	DFV	Tubulin beta-2C chain	P68371	TUBB4B	0.04
Polygonatum sibiricum(PGS)	MOL001792	DFV	Gamma-aminobutyric-acid receptor subunit alpha-5	P31644	GABRA5	0.041
Polygonatum sibiricum(PGS)	MOL001792	DFV	Gamma-aminobutyric-acid receptor subunit alpha-3	P34903	GABRA3	0.041
Polygonatum sibiricum(PGS)	MOL001792	DFV	Ig gamma-1 chain C region	P01857	IGHG1	0.042
Polygonatum sibiricum(PGS)	MOL001792	DFV	ATP synthase subunit beta, mitochondrial	P06576	ATP5F1B	0.042
Polygonatum sibiricum(PGS)	MOL001792	DFV	Tyrosine-protein kinase HCK	P08631	HCK	0.042
Polygonatum sibiricum(PGS)	MOL001792	DFV	Glycogen phosphorylase, muscle form	P11217	PYGM	0.042
Polygonatum sibiricum(PGS)	MOL001792	DFV	ATP synthase subunit alpha, mitochondrial	P25705	ATP5F1A	0.042
Polygonatum sibiricum(PGS)	MOL001792	DFV	Phosphatidylinositol-4,5-bisphosphate 3-kinase catalytic subunit gamma isoform	P48736	PIK3CG	0.042
Polygonatum sibiricum(PGS)	MOL001792	DFV	Alpha-1D adrenergic receptor	P25100	ADRA1D	0.046
Polygonatum sibiricum(PGS)	MOL001792	DFV	Tyrosyl-tRNA synthetase, cytoplasmic	P54577	YARS	0.046
Polygonatum sibiricum(PGS)	MOL001792	DFV	Protein tyrosine kinase 2 beta	Q14289	PTK2B	0.046
Polygonatum sibiricum(PGS)	MOL001792	DFV	Sterol O-acyltransferase 2	O75908	SOAT2	0.047
Polygonatum sibiricum(PGS)	MOL001792	DFV	D(1A) dopamine receptor	P21728	DRD1	0.047
Polygonatum sibiricum(PGS)	MOL001792	DFV	D(1B) dopamine receptor	P21918	DRD5	0.047
Polygonatum sibiricum(PGS)	MOL001792	DFV	5-hydroxytryptamine 2C receptor	P28335	HTR2C	0.047
Polygonatum sibiricum(PGS)	MOL001792	DFV	D(3) dopamine receptor	P35462	DRD3	0.047
Polygonatum sibiricum(PGS)	MOL001792	DFV	Sterol O-acyltransferase 1	P35610	SOAT1	0.047
Polygonatum sibiricum(PGS)	MOL001792	DFV	Sodium-dependent dopamine transporter	Q01959	SLC6A3	0.052
Polygonatum sibiricum(PGS)	MOL001792	DFV	Peroxisome proliferator-activated receptor gamma	P37231	PPARG	0.054
Polygonatum sibiricum(PGS)	MOL001792	DFV	Muscarinic acetylcholine receptor M1	P11229	CHRM1	0.055

Polygonatum sibiricum(PGS)	MOL001792	DFV	Estrogen-related receptor gamma	P62508	ESRRG	0.056
Polygonatum sibiricum(PGS)	MOL001792	DFV	Casein kinase II subunit alpha	P68400	CSNK2A1	0.056
Polygonatum sibiricum(PGS)	MOL001792	DFV	Carbonic anhydrase 1	P00915	CA1	0.058
Polygonatum sibiricum(PGS)	MOL001792	DFV	Ornithine aminotransferase, mitochondrial	P04181	OAT	0.059
Polygonatum sibiricum(PGS)	MOL001792	DFV	Nitric-oxide synthase, endothelial	P29474	NOS3	0.059
Polygonatum sibiricum(PGS)	MOL001792	DFV	Serine/threonine-protein kinase 6	O14965	AURKA	0.06
Polygonatum sibiricum(PGS)	MOL001792	DFV	Phospholipase A2	P04054	PLA2G1B	0.06
Polygonatum sibiricum(PGS)	MOL001792	DFV	Alpha-2C adrenergic receptor	P18825	ADRA2C	0.061
Polygonatum sibiricum(PGS)	MOL001792	DFV	Retinoic acid receptor RXR-beta	P28702	RXRB	0.061
Polygonatum sibiricum(PGS)	MOL001792	DFV	Endothelin-1 receptor	P25101	EDNRA	0.062
Polygonatum sibiricum(PGS)	MOL001792	DFV	Geranylgeranyl pyrophosphate synthetase	O95749	GGPS1	0.063
Polygonatum sibiricum(PGS)	MOL001792	DFV	Low molecular weight phosphotyrosine protein phosphatase	P24666	ACP1	0.063
Polygonatum sibiricum(PGS)	MOL001792	DFV	RAC-alpha serine/threonine-protein kinase	P31749	AKT1	0.063
Polygonatum sibiricum(PGS)	MOL001792	DFV	cAMP-dependent protein kinase inhibitor alpha	P61925	PKIA	0.063
Polygonatum sibiricum(PGS)	MOL001792	DFV	Egl nine homolog 1	Q9GZT9	EGLN1	0.063
Polygonatum sibiricum(PGS)	MOL001792	DFV	Casein kinase I isoform gamma-1	Q9HCP0	CSNK1G1	0.063
Polygonatum sibiricum(PGS)	MOL001792	DFV	Calcium/calmodulin-dependent protein kinase type II alpha chain	Q9UQM7	CAMK2A	0.063
Polygonatum sibiricum(PGS)	MOL001792	DFV	Androgen receptor	P10275	AR	0.064
Polygonatum sibiricum(PGS)	MOL001792	DFV	Thyroid hormone receptor alpha	P10827	THRA	0.065
Polygonatum sibiricum(PGS)	MOL001792	DFV	5-hydroxytryptamine 2A receptor	P28223	HTR2A	0.066
Polygonatum sibiricum(PGS)	MOL001792	DFV	S-methyl-5-thioadenosine phosphorylase	Q13126	MTAP	0.066
Polygonatum sibiricum(PGS)	MOL001792	DFV	Cytochrome P450 19A1	P11511	CYP19A1	0.067
Polygonatum sibiricum(PGS)	MOL001792	DFV	Prolyl endopeptidase	P48147	PREP	0.068
Polygonatum sibiricum(PGS)	MOL001792	DFV	D-HSCDK2	O75100	CA11	0.069
Polygonatum sibiricum(PGS)	MOL001792	DFV	Retinoic acid receptor RXR-alpha	P19793	RXRA	0.07
Polygonatum sibiricum(PGS)	MOL001792	DFV	Sodium-dependent serotonin transporter	P31645	SLC6A4	0.071
Polygonatum sibiricum(PGS)	MOL001792	DFV	Tyrosine-protein phosphatase non-receptor type 1	P18031	PTPN1	0.072
Polygonatum sibiricum(PGS)	MOL001792	DFV	ATP-sensitive inward rectifier potassium channel 1	P48048	KCNJ1	0.072
Polygonatum sibiricum(PGS)	MOL001792	DFV	Sodium channel protein type 5 subunit alpha	Q14524	SCN5A	0.072
Polygonatum sibiricum(PGS)	MOL001792	DFV	Muscarinic acetylcholine receptor M2	P08172	CHRM2	0.073
Polygonatum sibiricum(PGS)	MOL001792	DFV	Proto-oncogene tyrosine-protein kinase Src	P12931	SRC	0.073
Polygonatum sibiricum(PGS)	MOL001792	DFV	Oxysterols receptor LXR-alpha	Q13133	NR1H3	0.073
Polygonatum sibiricum(PGS)	MOL001792	DFV	Beta-1 adrenergic receptor	P08588	ADRB1	0.074
Polygonatum sibiricum(PGS)	MOL001792	DFV	Gamma-aminobutyric-acid receptor subunit alpha-2	P47869	GABRA2	0.074
Polygonatum sibiricum(PGS)	MOL001792	DFV	Alpha-1B adrenergic receptor	P35368	ADRA1B	0.076
Polygonatum sibiricum(PGS)	MOL001792	DFV	D(2) dopamine receptor	P14416	DRD2	0.077
Polygonatum sibiricum(PGS)	MOL001792	DFV	5-hydroxytryptamine 1A receptor	P08908	HTR1A	0.078
Polygonatum sibiricum(PGS)	MOL001792	DFV	Neuropeptide Y	P01303	NPY	0.081
Polygonatum sibiricum(PGS)	MOL001792	DFV	Interferon gamma	P01579	IFNG	0.084
Polygonatum sibiricum(PGS)	MOL001792	DFV	Inhibitor of nuclear factor kappa-B kinase subunit alpha	O15111	CHUK	0.087
Polygonatum sibiricum(PGS)	MOL001792	DFV	Gamma-aminobutyric-acid receptor subunit alpha-1	P14867	GABRA1	0.088
Polygonatum sibiricum(PGS)	MOL001792	DFV	Alpha-2A adrenergic receptor	P08913	ADRA2A	0.09
Polygonatum sibiricum(PGS)	MOL001792	DFV	DNA topoisomerase 2-alpha	P11388	TOP2A	0.091
Polygonatum sibiricum(PGS)	MOL001792	DFV	Alpha-2B adrenergic receptor	P18089	ADRA2B	0.091
Polygonatum sibiricum(PGS)	MOL001792	DFV	Carbonic anhydrase 2	P00918	CA2	0.093
Polygonatum sibiricum(PGS)	MOL001792	DFV	Sodium-dependent noradrenaline transporter	P23975	SLC6A2	0.093
Polygonatum sibiricum(PGS)	MOL001792	DFV	Tubulin alpha-3 chain	Q71U36	TUBA1A	0.094

Polygonatum sibiricum(PGS)	MOL001792	DFV	Cell division control protein 2 homolog	P06493	CDK1	0.096
Polygonatum sibiricum(PGS)	MOL001792	DFV	Ig kappa chain C region	P01834	IGKC	0.097
Polygonatum sibiricum(PGS)	MOL001792	DFV	Beta-2 adrenergic receptor	P07550	ADRB2	0.103
Polygonatum sibiricum(PGS)	MOL001792	DFV	cAMP-specific 3',5'-cyclic phosphodiesterase 4B	Q07343	PDE4B	0.107
Polygonatum sibiricum(PGS)	MOL001792	DFV	Arachidonate 5-lipoxygenase	P09917	ALOX5	0.119
Polygonatum sibiricum(PGS)	MOL001792	DFV	Alpha-1A adrenergic receptor	P35348	ADRA1A	0.12
Polygonatum sibiricum(PGS)	MOL001792	DFV	Cell division protein kinase 2	P24941	CDK2	0.123
Polygonatum sibiricum(PGS)	MOL001792	DFV	Cell division protein kinase 5	Q00535	CDK5	0.123
Polygonatum sibiricum(PGS)	MOL001792	DFV	cAMP-dependent protein kinase catalytic subunit alpha	P17612	PRKACA	0.138
Polygonatum sibiricum(PGS)	MOL001792	DFV	cAMP-specific 3',5'-cyclic phosphodiesterase 4A	P27815	PDE4A	0.144
Polygonatum sibiricum(PGS)	MOL001792	DFV	Nitric oxide synthase, inducible	P35228	NOS2	0.146
Polygonatum sibiricum(PGS)	MOL001792	DFV	Mitogen-activated protein kinase 14	Q16539	MAPK14	0.167
Polygonatum sibiricum(PGS)	MOL001792	DFV	Hemoglobin subunit alpha	P69905	HBA1	0.187
Polygonatum sibiricum(PGS)	MOL001792	DFV	Prostaglandin G/H synthase 1	P23219	PTGS1	0.19
Polygonatum sibiricum(PGS)	MOL001792	DFV	Coagulation factor VII	P08709	F7	0.209
Polygonatum sibiricum(PGS)	MOL001792	DFV	Prothrombin	P00734	F2	0.21
Polygonatum sibiricum(PGS)	MOL001792	DFV	Trypsin-1	P07477	PRSS1	0.22
Polygonatum sibiricum(PGS)	MOL001792	DFV	C-jun-amino-terminal kinase-interacting protein 1	Q9UQF2	MAPK8IP1	0.228
Polygonatum sibiricum(PGS)	MOL001792	DFV	Nuclear receptor coactivator 1	Q15788	NCOA1	0.254
Polygonatum sibiricum(PGS)	MOL001792	DFV	Prostaglandin G/H synthase 2	P35354	PTGS2	0.293
Polygonatum sibiricum(PGS)	MOL001792	DFV	Cyclin-A2	P20248	CCNA2	0.45
Polygonatum sibiricum(PGS)	MOL001792	DFV	Estrogen receptor beta	Q92731	ESR2	0.475
Polygonatum sibiricum(PGS)	MOL001792	DFV	Estrogen receptor	P03372	ESR1	1
Polygonatum sibiricum(PGS)	MOL002714	baicalein	Voltage-dependent T-type calcium channel subunit alpha-1G	O43497	CACNA1G	0.01
Polygonatum sibiricum(PGS)	MOL002714	baicalein	Carbonic anhydrase 12	O43570	CA12	0.01
Polygonatum sibiricum(PGS)	MOL002714	baicalein	Carbonic anhydrase-related protein 11	O75493	CA11	0.01
Polygonatum sibiricum(PGS)	MOL002714	baicalein	Voltage-dependent T-type calcium channel subunit alpha-1H	O95180	CACNA1H	0.01
Polygonatum sibiricum(PGS)	MOL002714	baicalein	Carbonic anhydrase 3	P07451	CA3	0.01
Polygonatum sibiricum(PGS)	MOL002714	baicalein	Sodium/hydrogen exchanger 1	P19634	SLC9A1	0.01
Polygonatum sibiricum(PGS)	MOL002714	baicalein	Amiloride-sensitive amine oxidase [copper-containing]	P19801	AOC1	0.01
Polygonatum sibiricum(PGS)	MOL002714	baicalein	Carbonic anhydrase 6	P23280	CA6	0.01
Polygonatum sibiricum(PGS)	MOL002714	baicalein	Amine oxidase [flavin-containing] B	P27338	MAOB	0.01
Polygonatum sibiricum(PGS)	MOL002714	baicalein	Carbonic anhydrase 5A, mitochondrial	P35218	CA5A	0.01
Polygonatum sibiricum(PGS)	MOL002714	baicalein	Carbonic anhydrase-related protein	P35219	CA8	0.01
Polygonatum sibiricum(PGS)	MOL002714	baicalein	Sodium channel protein type 1 subunit alpha	P35498	SCN1A	0.01
Polygonatum sibiricum(PGS)	MOL002714	baicalein	Sodium channel protein type 4 subunit alpha	P35499	SCN4A	0.01
Polygonatum sibiricum(PGS)	MOL002714	baicalein	Carbonic anhydrase 7	P43166	CA7	0.01
Polygonatum sibiricum(PGS)	MOL002714	baicalein	Amiloride-sensitive sodium channel subunit delta	P51172	SCNN1D	0.01
Polygonatum sibiricum(PGS)	MOL002714	baicalein	Amiloride-sensitive cation channel 2, neuronal	P78348	ASIC1	0.01
Polygonatum sibiricum(PGS)	MOL002714	baicalein	Sodium channel subunit beta-1	Q07699	SCN1B	0.01
Polygonatum sibiricum(PGS)	MOL002714	baicalein	Sodium channel protein type 9 subunit alpha	Q15858	SCN9A	0.01
Polygonatum sibiricum(PGS)	MOL002714	baicalein	Amiloride-sensitive cation channel 1, neuronal	Q16515	ASIC2	0.01
Polygonatum sibiricum(PGS)	MOL002714	baicalein	Carbonic anhydrase 9	Q16790	CA9	0.01
Polygonatum sibiricum(PGS)	MOL002714	baicalein	Opioid receptor, sigma 1	Q5T1J1	SIGMAR1	0.01
Polygonatum sibiricum(PGS)	MOL002714	baicalein	Sodium channel subunit beta-4	Q8IWT1	SCN4B	0.01
Polygonatum sibiricum(PGS)	MOL002714	baicalein	Carbonic anhydrase 13	Q8N1Q1	CA13	0.01
Polygonatum sibiricum(PGS)	MOL002714	baicalein	Sodium channel protein type 2 subunit alpha	Q99250	SCN2A	0.01

Polygonatum sibiricum(PGS)	MOL002714	baicalein	Sigma 1-type opioid receptor	Q99720	SIGMAR1	0.01
Polygonatum sibiricum(PGS)	MOL002714	baicalein	Carbonic anhydrase-related protein 10	Q9NS85	CA10	0.01
Polygonatum sibiricum(PGS)	MOL002714	baicalein	Sodium channel protein type 3 subunit alpha	Q9NY46	SCN3A	0.01
Polygonatum sibiricum(PGS)	MOL002714	baicalein	Sodium channel subunit beta-3	Q9NY72	SCN3B	0.01
Polygonatum sibiricum(PGS)	MOL002714	baicalein	Voltage-dependent T-type calcium channel subunit alpha-1I	Q9P0X4	CACNA1I	0.01
Polygonatum sibiricum(PGS)	MOL002714	baicalein	Sodium channel protein type 11 subunit alpha	Q9UI33	SCN11A	0.01
Polygonatum sibiricum(PGS)	MOL002714	baicalein	Carbonic anhydrase 14	Q9ULX7	CA14	0.01
Polygonatum sibiricum(PGS)	MOL002714	baicalein	Potassium channel subfamily K member 6	Q9Y257	KCNK6	0.01
Polygonatum sibiricum(PGS)	MOL002714	baicalein	Carbonic anhydrase 5B, mitochondrial	Q9Y2D0	CA5B	0.01
Polygonatum sibiricum(PGS)	MOL002714	baicalein	Retinoic acid receptor alpha	P10276	RARA	0.011
Polygonatum sibiricum(PGS)	MOL002714	baicalein	Retinoic acid receptor beta	P10826	RARB	0.011
Polygonatum sibiricum(PGS)	MOL002714	baicalein	Retinoic acid receptor gamma-1	P13631	RARG	0.011
Polygonatum sibiricum(PGS)	MOL002714	baicalein	Retinoic acid receptor RXR-beta	P28702	RXRB	0.011
Polygonatum sibiricum(PGS)	MOL002714	baicalein	Retinoic acid receptor RXR-gamma	P48443	RXRG	0.011
Polygonatum sibiricum(PGS)	MOL002714	baicalein	Furin	P09958	FURIN	0.012
Polygonatum sibiricum(PGS)	MOL002714	baicalein	Sodium/potassium-transporting ATPase gamma chain	P54710	FXYD2	0.012
Polygonatum sibiricum(PGS)	MOL002714	baicalein	D1 dopamine receptor-interacting protein calcyon	Q9NYX4	CALY	0.012
Polygonatum sibiricum(PGS)	MOL002714	baicalein	Glycolipid transfer protein	Q9NZD2	GLTP	0.012
Polygonatum sibiricum(PGS)	MOL002714	baicalein	Gamma-aminobutyric acid receptor subunit rho-3	A8MPY1	GABRR3	0.013
Polygonatum sibiricum(PGS)	MOL002714	baicalein	Phosphatidylinositol 3-kinase regulatory subunit beta	O00459	PIK3R2	0.013
Polygonatum sibiricum(PGS)	MOL002714	baicalein	Gamma-aminobutyric acid receptor subunit pi	O00591	GABRP	0.013
Polygonatum sibiricum(PGS)	MOL002714	baicalein	Gamma-aminobutyric acid receptor subunit delta	O14764	GABRD	0.013
Polygonatum sibiricum(PGS)	MOL002714	baicalein	Beta-3 adrenergic receptor	P13945	ADRB3	0.013
Polygonatum sibiricum(PGS)	MOL002714	baicalein	Gamma-aminobutyric-acid receptor subunit beta-1	P18505	GABRB1	0.013
Polygonatum sibiricum(PGS)	MOL002714	baicalein	Gamma-aminobutyric acid receptor subunit gamma-2	P18507	GABRG2	0.013
Polygonatum sibiricum(PGS)	MOL002714	baicalein	Gamma-aminobutyric-acid receptor subunit rho-1	P24046	GABRR1	0.013
Polygonatum sibiricum(PGS)	MOL002714	baicalein	Phosphatidylinositol 3-kinase regulatory subunit alpha	P27986	PIK3R1	0.013
Polygonatum sibiricum(PGS)	MOL002714	baicalein	Gamma-aminobutyric acid receptor subunit rho-2	P28476	GABRR2	0.013
Polygonatum sibiricum(PGS)	MOL002714	baicalein	Mitogen-activated protein kinase 1	P28482	MAPK1	0.013
Polygonatum sibiricum(PGS)	MOL002714	baicalein	Solute carrier family 12 member 2	P55011	SLC12A2	0.013
Polygonatum sibiricum(PGS)	MOL002714	baicalein	Gamma-aminobutyric acid receptor subunit epsilon	P78334	GABRE	0.013
Polygonatum sibiricum(PGS)	MOL002714	baicalein	Gamma-aminobutyric acid receptor subunit gamma-1	Q8N1C3	GABRG1	0.013
Polygonatum sibiricum(PGS)	MOL002714	baicalein	Gamma-aminobutyric acid receptor subunit gamma-3	Q99928	GABRG3	0.013
Polygonatum sibiricum(PGS)	MOL002714	baicalein	Neuronal acetylcholine receptor subunit alpha-3	P32297	CHRNA3	0.014
Polygonatum sibiricum(PGS)	MOL002714	baicalein	Neuronal acetylcholine receptor subunit alpha-4	P43681	CHRNA4	0.014
Polygonatum sibiricum(PGS)	MOL002714	baicalein	Gamma-aminobutyric-acid receptor subunit alpha-4	P48169	GABRA4	0.014
Polygonatum sibiricum(PGS)	MOL002714	baicalein	Chromaffin granule amine transporter	P54219	SLC18A1	0.014
Polygonatum sibiricum(PGS)	MOL002714	baicalein	Synaptic vesicular amine transporter	Q05940	SLC18A2	0.014
Polygonatum sibiricum(PGS)	MOL002714	baicalein	Gamma-aminobutyric-acid receptor subunit alpha-6	Q16445	GABRA6	0.014
Polygonatum sibiricum(PGS)	MOL002714	baicalein	Protein S100-B	P04271	S100B	0.015
Polygonatum sibiricum(PGS)	MOL002714	baicalein	Trypsin-2	P07478	PRSS2	0.015
Polygonatum sibiricum(PGS)	MOL002714	baicalein	Interleukin-3	P08700	IL3	0.015
Polygonatum sibiricum(PGS)	MOL002714	baicalein	Protein S100-A1	P23297	S100A1	0.015
Polygonatum sibiricum(PGS)	MOL002714	baicalein	Histamine H1 receptor	P35367	HRH1	0.015
Polygonatum sibiricum(PGS)	MOL002714	baicalein	Muscarinic acetylcholine receptor M4	P08173	CHRM4	0.016
Polygonatum sibiricum(PGS)	MOL002714	baicalein	Muscarinic acetylcholine receptor M1	P11229	CHRM1	0.016
Polygonatum sibiricum(PGS)	MOL002714	baicalein	Elongation factor 2	P13639	EEF2	0.016

Polygonatum sibiricum(PGS)	MOL002714	baicalein	Muscarinic acetylcholine receptor M3	P20309	CHRM3	0.016
Polygonatum sibiricum(PGS)	MOL002714	baicalein	Poly [ADP-ribose] polymerase 3	Q9Y6F1	PARP3	0.016
Polygonatum sibiricum(PGS)	MOL002714	baicalein	Purine nucleoside phosphorylase	P00491	PNP	0.018
Polygonatum sibiricum(PGS)	MOL002714	baicalein	DNA polymerase alpha catalytic subunit	P09884	POLA1	0.018
Polygonatum sibiricum(PGS)	MOL002714	baicalein	Ribonucleoside-diphosphate reductase large subunit	P23921	RRM1	0.018
Polygonatum sibiricum(PGS)	MOL002714	baicalein	Ribonucleoside-diphosphate reductase M2 subunit	P31350	RRM2	0.018
Polygonatum sibiricum(PGS)	MOL002714	baicalein	DNA polymerase epsilon subunit 2	P56282	POLE2	0.018
Polygonatum sibiricum(PGS)	MOL002714	baicalein	DNA polymerase epsilon catalytic subunit A	Q07864	POLE	0.018
Polygonatum sibiricum(PGS)	MOL002714	baicalein	Ribonucleoside-diphosphate reductase subunit M2 B	Q7LG56	RRM2B	0.018
Polygonatum sibiricum(PGS)	MOL002714	baicalein	DNA polymerase epsilon subunit 3	Q9NRF9	POLE3	0.018
Polygonatum sibiricum(PGS)	MOL002714	baicalein	Cytochrome b	P00156	MT-CYB	0.019
Polygonatum sibiricum(PGS)	MOL002714	baicalein	Cytochrome c1, heme protein, mitochondrial	P08574	CYC1	0.019
Polygonatum sibiricum(PGS)	MOL002714	baicalein	Cytochrome b-c1 complex subunit Rieske, mitochondrial	P47985	UQCRFS1	0.019
Polygonatum sibiricum(PGS)	MOL002714	baicalein	Neuronal acetylcholine receptor subunit alpha-2	Q15822	CHRNA2	0.019
Polygonatum sibiricum(PGS)	MOL002714	baicalein	5-hydroxytryptamine 4 receptor	Q13639	HTR4	0.02
Polygonatum sibiricum(PGS)	MOL002714	baicalein	Cell division protein kinase 4	P11802	CDK4	0.021
Polygonatum sibiricum(PGS)	MOL002714	baicalein	Cell division protein kinase 7	P50613	CDK7	0.021
Polygonatum sibiricum(PGS)	MOL002714	baicalein	Cell division protein kinase 9	P50750	CDK9	0.021
Polygonatum sibiricum(PGS)	MOL002714	baicalein	Cell division protein kinase 6	Q00534	CDK6	0.021
Polygonatum sibiricum(PGS)	MOL002714	baicalein	Bile salt sulfotransferase	Q06520	SULT2A1	0.021
Polygonatum sibiricum(PGS)	MOL002714	baicalein	Dehydrogenase/reductase SDR family member 8	Q8NBQ5	HSD17B11	0.021
Polygonatum sibiricum(PGS)	MOL002714	baicalein	Potassium channel subfamily K member 1	O00180	KCNK1	0.022
Polygonatum sibiricum(PGS)	MOL002714	baicalein	Serine/threonine-protein kinase 17B	O94768	STK17B	0.022
Polygonatum sibiricum(PGS)	MOL002714	baicalein	Gamma-aminobutyric-acid receptor subunit alpha-5	P31644	GABRA5	0.022
Polygonatum sibiricum(PGS)	MOL002714	baicalein	Gamma-aminobutyric-acid receptor subunit alpha-3	P34903	GABRA3	0.022
Polygonatum sibiricum(PGS)	MOL002714	baicalein	ATP synthase subunit gamma, mitochondrial	P36542	ATP5F1C	0.022
Polygonatum sibiricum(PGS)	MOL002714	baicalein	UDP-glucuronosyltransferase 3A1	Q6NUS8	UGT3A1	0.022
Polygonatum sibiricum(PGS)	MOL002714	baicalein	Neuronal acetylcholine receptor subunit alpha-7	P36544	CHRNA7	0.023
Polygonatum sibiricum(PGS)	MOL002714	baicalein	Cyclin-dependent kinase 5 activator 1	Q15078	CDK5R1	0.023
Polygonatum sibiricum(PGS)	MOL002714	baicalein	3-phosphoinositide-dependent protein kinase 1	O15530	PDPK1	0.024
Polygonatum sibiricum(PGS)	MOL002714	baicalein	Tyrosine-protein kinase Lyn	P07948	LYN	0.024
Polygonatum sibiricum(PGS)	MOL002714	baicalein	Tyrosine-protein kinase CSK	P41240	CSK	0.024
Polygonatum sibiricum(PGS)	MOL002714	baicalein	Tyrosine-protein kinase ZAP-70	P43403	ZAP70	0.024
Polygonatum sibiricum(PGS)	MOL002714	baicalein	Tyrosine-protein kinase SYK	P43405	SYK	0.024
Polygonatum sibiricum(PGS)	MOL002714	baicalein	Protein kinase C theta type	Q04759	PRKCQ	0.024
Polygonatum sibiricum(PGS)	MOL002714	baicalein	Tyrosine-protein kinase ITK/TSK	Q08881	ITK	0.024
Polygonatum sibiricum(PGS)	MOL002714	baicalein	85 kDa calcium-independent phospholipase A2	O60733	PLA2G6	0.025
Polygonatum sibiricum(PGS)	MOL002714	baicalein	Amiloride-sensitive sodium channel subunit alpha	P37088	SCNN1A	0.025
Polygonatum sibiricum(PGS)	MOL002714	baicalein	Cytosolic phospholipase A2	P47712	PLA2G4A	0.025
Polygonatum sibiricum(PGS)	MOL002714	baicalein	Amiloride-sensitive sodium channel subunit beta	P51168	SCNN1B	0.025
Polygonatum sibiricum(PGS)	MOL002714	baicalein	Amiloride-sensitive sodium channel subunit gamma	P51170	SCNN1G	0.025
Polygonatum sibiricum(PGS)	MOL002714	baicalein	Carbonic anhydrase 4	P22748	CA4	0.026
Polygonatum sibiricum(PGS)	MOL002714	baicalein	Tyrosine-protein kinase JAK2	O60674	JAK2	0.027
Polygonatum sibiricum(PGS)	MOL002714	baicalein	Tyrosine-protein kinase JAK1	P23458	JAK1	0.027
Polygonatum sibiricum(PGS)	MOL002714	baicalein	Tyrosine-protein kinase JAK3	P52333	JAK3	0.027
Polygonatum sibiricum(PGS)	MOL002714	baicalein	Tripartite motif-containing protein 13	O60858	TRIM13	0.028
Polygonatum sibiricum(PGS)	MOL002714	baicalein	cAMP response element-binding protein	P16220	CREB1	0.028

Polygonatum sibiricum(PGS)	MOL002714	baicalein	Glutamate [NMDA] receptor subunit 3A	Q8TCU5	GRIN3A	0.028
Polygonatum sibiricum(PGS)	MOL002714	baicalein	Phenylalanine-4-hydroxylase	P00439	PAH	0.029
Polygonatum sibiricum(PGS)	MOL002714	baicalein	Amine oxidase [flavin-containing] A	P21397	MAOA	0.029
Polygonatum sibiricum(PGS)	MOL002714	baicalein	Potassium voltage-gated channel subfamily H member 2	Q12809	KCNH2	0.029
Polygonatum sibiricum(PGS)	MOL002714	baicalein	Potassium voltage-gated channel subfamily H member 6	Q9H252	KCNH6	0.029
Polygonatum sibiricum(PGS)	MOL002714	baicalein	Sodium channel protein type 10 subunit alpha	Q9Y5Y9	SCN10A	0.03
Polygonatum sibiricum(PGS)	MOL002714	baicalein	Solute carrier family 12 member 1	Q13621	SLC12A1	0.031
Polygonatum sibiricum(PGS)	MOL002714	baicalein	Methionine aminopeptidase 1	P53582	METAP1	0.032
Polygonatum sibiricum(PGS)	MOL002714	baicalein	Calcium/calmodulin-dependent protein kinase type II subunit delta	Q13557	CAMK2D	0.032
Polygonatum sibiricum(PGS)	MOL002714	baicalein	STE20-like serine/threonine-protein kinase	Q9H2G2	SLK	0.032
Polygonatum sibiricum(PGS)	MOL002714	baicalein	DNA-(apurinic or apyrimidinic site) lyase	P27695	APEX1	0.033
Polygonatum sibiricum(PGS)	MOL002714	baicalein	Gamma-aminobutyric-acid receptor subunit alpha-1	P14867	GABRA1	0.034
Polygonatum sibiricum(PGS)	MOL002714	baicalein	Mitogen-activated protein kinase 8	P45983	MAPK8	0.034
Polygonatum sibiricum(PGS)	MOL002714	baicalein	Gamma-aminobutyric-acid receptor subunit alpha-2	P47869	GABRA2	0.034
Polygonatum sibiricum(PGS)	MOL002714	baicalein	Mitogen-activated protein kinase 10	P53779	MAPK10	0.034
Polygonatum sibiricum(PGS)	MOL002714	baicalein	Inhibitor of nuclear factor kappa-B kinase subunit beta	O14920	IKBKB	0.035
Polygonatum sibiricum(PGS)	MOL002714	baicalein	Phospholipase A2, membrane associated	P14555	PLA2G2A	0.035
Polygonatum sibiricum(PGS)	MOL002714	baicalein	Casein kinase I isoform gamma-2	P78368	CSNK1G2	0.035
Polygonatum sibiricum(PGS)	MOL002714	baicalein	Protein S100-A12	P80511	S100A12	0.035
Polygonatum sibiricum(PGS)	MOL002714	baicalein	Lactoylglutathione lyase	Q04760	GLO1	0.035
Polygonatum sibiricum(PGS)	MOL002714	baicalein	Prostaglandin reductase 2	Q8N8N7	PTGR2	0.035
Polygonatum sibiricum(PGS)	MOL002714	baicalein	Serine/threonine-protein kinase haspin	Q8TF76	HASPIN	0.035
Polygonatum sibiricum(PGS)	MOL002714	baicalein	Protein S100-A13	Q99584	S100A13	0.035
Polygonatum sibiricum(PGS)	MOL002714	baicalein	Group IIE secretory phospholipase A2	Q9NZK7	PLA2G2E	0.035
Polygonatum sibiricum(PGS)	MOL002714	baicalein	Cystine/glutamate transporter	Q9UPY5	SLC7A11	0.035
Polygonatum sibiricum(PGS)	MOL002714	baicalein	Ig kappa chain C region	P01834	IGKC	0.036
Polygonatum sibiricum(PGS)	MOL002714	baicalein	Ig gamma-1 chain C region	P01857	IGHG1	0.036
Polygonatum sibiricum(PGS)	MOL002714	baicalein	Myeloperoxidase	P05164	MPO	0.036
Polygonatum sibiricum(PGS)	MOL002714	baicalein	Muscarinic acetylcholine receptor M2	P08172	CHRM2	0.036
Polygonatum sibiricum(PGS)	MOL002714	baicalein	DNA topoisomerase 1	P11387	TOP1	0.036
Polygonatum sibiricum(PGS)	MOL002714	baicalein	Eosinophil peroxidase	P11678	EPX	0.036
Polygonatum sibiricum(PGS)	MOL002714	baicalein	Calreticulin	P27797	CALR	0.036
Polygonatum sibiricum(PGS)	MOL002714	baicalein	Sepiapterin reductase	P35270	SPR	0.036
Polygonatum sibiricum(PGS)	MOL002714	baicalein	Melatonin receptor type 1B	P49286	MTNR1B	0.036
Polygonatum sibiricum(PGS)	MOL002714	baicalein	Calmodulin	P62158		0.036
Polygonatum sibiricum(PGS)	MOL002714	baicalein	6-pyruvoyl tetrahydrobiopterin synthase	Q03393	PTS	0.036
Polygonatum sibiricum(PGS)	MOL002714	baicalein	Nuclear receptor ROR-beta	Q92753	RORB	0.036
Polygonatum sibiricum(PGS)	MOL002714	baicalein	DNA topoisomerase I, mitochondrial	Q969P6	TOP1MT	0.036
Polygonatum sibiricum(PGS)	MOL002714	baicalein	Tyrosyl-tRNA synthetase, cytoplasmic	P54577	YARS	0.037
Polygonatum sibiricum(PGS)	MOL002714	baicalein	Peptidyl-prolyl cis-trans isomerase NIMA-interacting 1	Q13526	PIN1	0.037
Polygonatum sibiricum(PGS)	MOL002714	baicalein	Urokinase-type plasminogen activator	P00749	PLAU	0.038
Polygonatum sibiricum(PGS)	MOL002714	baicalein	5-hydroxytryptamine 3 receptor	P46098	HTR3A	0.038
Polygonatum sibiricum(PGS)	MOL002714	baicalein	Glycogen phosphorylase, muscle form	P11217	PYGM	0.039
Polygonatum sibiricum(PGS)	MOL002714	baicalein	Peptidyl-prolyl cis-trans isomerase, mitochondrial	P30405	PPIF	0.039
Polygonatum sibiricum(PGS)	MOL002714	baicalein	Tubulin alpha-1 chain	P68366	TUBA4A	0.04
Polygonatum sibiricum(PGS)	MOL002714	baicalein	3-oxo-5-alpha-steroid 4-dehydrogenase 2	P31213	SRD5A2	0.041
Polygonatum sibiricum(PGS)	MOL002714	baicalein	Sterol O-acyltransferase 2	O75908	SOAT2	0.043

Polygonatum sibiricum(PGS)	MOL002714	baicalein	Gonadotropin-releasing hormone receptor	P30968	GNRHR	0.043
Polygonatum sibiricum(PGS)	MOL002714	baicalein	Sterol O-acyltransferase 1	P35610	SOAT1	0.043
Polygonatum sibiricum(PGS)	MOL002714	baicalein	Gonadotropin-releasing hormone II receptor	Q96P88	GNRHR2	0.043
Polygonatum sibiricum(PGS)	MOL002714	baicalein	Microtubule-associated protein 2	P11137	MAP2	0.044
Polygonatum sibiricum(PGS)	MOL002714	baicalein	3 beta-hydroxysteroid dehydrogenase/Delta 5-->4-isomerase type I	P14060	HSD3B1	0.044
Polygonatum sibiricum(PGS)	MOL002714	baicalein	3 beta-hydroxysteroid dehydrogenase/Delta 5-->4-isomerase type II	P26439	HSD3B2	0.044
Polygonatum sibiricum(PGS)	MOL002714	baicalein	Nitric-oxide synthase, brain	P29475	NOS1	0.044
Polygonatum sibiricum(PGS)	MOL002714	baicalein	Estrogen-related receptor gamma	P62508	ESRRG	0.044
Polygonatum sibiricum(PGS)	MOL002714	baicalein	Microtubule-associated protein 1A	P78559	MAP1A	0.044
Polygonatum sibiricum(PGS)	MOL002714	baicalein	Keratin, type II cytoskeletal 7	P08729	KRT7	0.045
Polygonatum sibiricum(PGS)	MOL002714	baicalein	Platelet glycoprotein IX	P14770	GP9	0.045
Polygonatum sibiricum(PGS)	MOL002714	baicalein	cAMP-specific 3',5'-cyclic phosphodiesterase 4C	Q08493	PDE4C	0.045
Polygonatum sibiricum(PGS)	MOL002714	baicalein	cGMP-inhibited 3',5'-cyclic phosphodiesterase A	Q14432	PDE3A	0.045
Polygonatum sibiricum(PGS)	MOL002714	baicalein	Epidermal growth factor receptor	P00533	EGFR	0.046
Polygonatum sibiricum(PGS)	MOL002714	baicalein	5-hydroxytryptamine 1B receptor	P28222	HTR1B	0.046
Polygonatum sibiricum(PGS)	MOL002714	baicalein	5-hydroxytryptamine 2C receptor	P28335	HTR2C	0.046
Polygonatum sibiricum(PGS)	MOL002714	baicalein	5-hydroxytryptamine 2B receptor	P41595	HTR2B	0.046
Polygonatum sibiricum(PGS)	MOL002714	baicalein	ATP synthase subunit beta, mitochondrial	P06576	ATP5F1B	0.047
Polygonatum sibiricum(PGS)	MOL002714	baicalein	Tyrosine-protein kinase HCK	P08631	HCK	0.047
Polygonatum sibiricum(PGS)	MOL002714	baicalein	ATP synthase subunit alpha, mitochondrial	P25705	ATP5F1A	0.047
Polygonatum sibiricum(PGS)	MOL002714	baicalein	Phosphatidylinositol-4,5-bisphosphate 3-kinase catalytic subunit gamma isoform	P48736	PIK3CG	0.047
Polygonatum sibiricum(PGS)	MOL002714	baicalein	Protein tyrosine kinase 2 beta	Q14289	PTK2B	0.047
Polygonatum sibiricum(PGS)	MOL002714	baicalein	Proto-oncogene serine/threonine-protein kinase Pim-1	P11309	PIM1	0.049
Polygonatum sibiricum(PGS)	MOL002714	baicalein	Sodium-dependent dopamine transporter	Q01959	SLC6A3	0.049
Polygonatum sibiricum(PGS)	MOL002714	baicalein	Glycogen synthase kinase-3 beta	P49841	GSK3B	0.05
Polygonatum sibiricum(PGS)	MOL002714	baicalein	Tubulin beta-2C chain	P68371	TUBB4B	0.05
Polygonatum sibiricum(PGS)	MOL002714	baicalein	Proto-oncogene tyrosine-protein kinase LCK	P06239	LCK	0.051
Polygonatum sibiricum(PGS)	MOL002714	baicalein	DNA (cytosine-5)-methyltransferase 1	P26358	DNMT1	0.056
Polygonatum sibiricum(PGS)	MOL002714	baicalein	Toll-like receptor 7	Q9NYK1	TLR7	0.057
Polygonatum sibiricum(PGS)	MOL002714	baicalein	Death-associated protein kinase 3	O43293	DAPK3	0.058
Polygonatum sibiricum(PGS)	MOL002714	baicalein	Carbonic anhydrase 1	P00915	CA1	0.058
Polygonatum sibiricum(PGS)	MOL002714	baicalein	5-hydroxytryptamine 1D receptor	P28221	HTR1D	0.062
Polygonatum sibiricum(PGS)	MOL002714	baicalein	Serine/threonine-protein kinase 6	O14965	AURKA	0.067
Polygonatum sibiricum(PGS)	MOL002714	baicalein	Hepatocyte growth factor receptor	P08581	MET	0.067
Polygonatum sibiricum(PGS)	MOL002714	baicalein	Mannose-binding protein C	P11226	MBL2	0.068
Polygonatum sibiricum(PGS)	MOL002714	baicalein	5-hydroxytryptamine 2A receptor	P28223	HTR2A	0.068
Polygonatum sibiricum(PGS)	MOL002714	baicalein	Coagulation factor VII	P08709	F7	0.069
Polygonatum sibiricum(PGS)	MOL002714	baicalein	Tyrosine-protein phosphatase non-receptor type 1	P18031	PTPN1	0.069
Polygonatum sibiricum(PGS)	MOL002714	baicalein	Aldo-keto reductase family 1 member C1	Q04828	AKR1C1	0.069
Polygonatum sibiricum(PGS)	MOL002714	baicalein	5-hydroxytryptamine 1A receptor	P08908	HTR1A	0.07
Polygonatum sibiricum(PGS)	MOL002714	baicalein	Dihydrofolate reductase	P00374	DHFR	0.071
Polygonatum sibiricum(PGS)	MOL002714	baicalein	Ig kappa chain V-II region RPMI 6410	P06310	IGKV2-30	0.071
Polygonatum sibiricum(PGS)	MOL002714	baicalein	Thyroid hormone receptor alpha	P10827	THRA	0.071
Polygonatum sibiricum(PGS)	MOL002714	baicalein	Casein kinase II subunit alpha	P68400	CSNK2A1	0.071
Polygonatum sibiricum(PGS)	MOL002714	baicalein	cAMP-specific 3',5'-cyclic phosphodiesterase 4D	Q08499	PDE4D	0.071
Polygonatum sibiricum(PGS)	MOL002714	baicalein	L-amino-acid oxidase	Q96RQ9	IL4I1	0.071
Polygonatum sibiricum(PGS)	MOL002714	baicalein	Cannabinoid receptor 1	P21554	CNR1	0.073

Polygonatum sibiricum(PGS)	MOL002714	baicalein	Endothelin-1 receptor	P25101	EDNRA	0.073
Polygonatum sibiricum(PGS)	MOL002714	baicalein	C-jun-amino-terminal kinase-interacting protein 1	Q9UQF2	MAPK8IP1	0.073
Polygonatum sibiricum(PGS)	MOL002714	baicalein	Calcium/calmodulin-dependent protein kinase type II alpha chain	Q9UQM7	CAMK2A	0.073
Polygonatum sibiricum(PGS)	MOL002714	baicalein	Carbonic anhydrase 2	P00918	CA2	0.074
Polygonatum sibiricum(PGS)	MOL002714	baicalein	Ribosyldihydronicotinamide dehydrogenase [quinone]	P16083	NQO2	0.074
Polygonatum sibiricum(PGS)	MOL002714	baicalein	Sodium-dependent noradrenaline transporter	P23975	SLC6A2	0.074
Polygonatum sibiricum(PGS)	MOL002714	baicalein	Melatonin receptor type 1A	P48039	MTNR1A	0.074
Polygonatum sibiricum(PGS)	MOL002714	baicalein	Triosephosphate isomerase	P60174	TPI1	0.074
Polygonatum sibiricum(PGS)	MOL002714	baicalein	S-methyl-5-thioadenosine phosphorylase	Q13126	MTAP	0.074
Polygonatum sibiricum(PGS)	MOL002714	baicalein	Membrane copper amine oxidase	Q16853	AOC3	0.075
Polygonatum sibiricum(PGS)	MOL002714	baicalein	Phospholipase A2	P04054	PLA2G1B	0.078
Polygonatum sibiricum(PGS)	MOL002714	baicalein	Beta-2 adrenergic receptor	P07550	ADRB2	0.079
Polygonatum sibiricum(PGS)	MOL002714	baicalein	D(4) dopamine receptor	P21917	DRD4	0.079
Polygonatum sibiricum(PGS)	MOL002714	baicalein	MAP kinase-activated protein kinase 2	P49137	MAPKAPK2	0.08
Polygonatum sibiricum(PGS)	MOL002714	baicalein	Sodium channel protein type 5 subunit alpha	Q14524	SCN5A	0.082
Polygonatum sibiricum(PGS)	MOL002714	baicalein	Nuclear receptor coactivator 5	Q9HCD5	NCOA5	0.083
Polygonatum sibiricum(PGS)	MOL002714	baicalein	Interferon gamma	P01579	IFNG	0.085
Polygonatum sibiricum(PGS)	MOL002714	baicalein	3-oxo-5-alpha-steroid 4-dehydrogenase 1	P18405	SRD5A1	0.087
Polygonatum sibiricum(PGS)	MOL002714	baicalein	Androgen receptor	P10275	AR	0.089
Polygonatum sibiricum(PGS)	MOL002714	baicalein	Proto-oncogene tyrosine-protein kinase Src	P12931	SRC	0.089
Polygonatum sibiricum(PGS)	MOL002714	baicalein	Nitric-oxide synthase, endothelial	P29474	NOS3	0.092
Polygonatum sibiricum(PGS)	MOL002714	baicalein	Sodium-dependent serotonin transporter	P31645	SLC6A4	0.093
Polygonatum sibiricum(PGS)	MOL002714	baicalein	cAMP-specific 3',5'-cyclic phosphodiesterase 4A	P27815	PDE4A	0.094
Polygonatum sibiricum(PGS)	MOL002714	baicalein	Alpha-2A adrenergic receptor	P08913	ADRA2A	0.095
Polygonatum sibiricum(PGS)	MOL002714	baicalein	Alpha-2C adrenergic receptor	P18825	ADRA2C	0.095
Polygonatum sibiricum(PGS)	MOL002714	baicalein	D(3) dopamine receptor	P35462	DRD3	0.095
Polygonatum sibiricum(PGS)	MOL002714	baicalein	DNA polymerase kappa	Q9UBT6	POLK	0.095
Polygonatum sibiricum(PGS)	MOL002714	baicalein	Beta-1 adrenergic receptor	P08588	ADRB1	0.096
Polygonatum sibiricum(PGS)	MOL002714	baicalein	Alpha-1D adrenergic receptor	P25100	ADRA1D	0.096
Polygonatum sibiricum(PGS)	MOL002714	baicalein	D-HSCDK2	O75100	CA11	0.1
Polygonatum sibiricum(PGS)	MOL002714	baicalein	Cell division control protein 2 homolog	P06493	CDK1	0.1
Polygonatum sibiricum(PGS)	MOL002714	baicalein	Tubulin alpha-3 chain	Q71U36	TUBA1A	0.103
Polygonatum sibiricum(PGS)	MOL002714	baicalein	cAMP-dependent protein kinase inhibitor alpha	P61925	PKIA	0.107
Polygonatum sibiricum(PGS)	MOL002714	baicalein	Rho-associated protein kinase 1	Q13464	ROCK1	0.107
Polygonatum sibiricum(PGS)	MOL002714	baicalein	Inhibitor of nuclear factor kappa-B kinase subunit alpha	O15111	CHUK	0.112
Polygonatum sibiricum(PGS)	MOL002714	baicalein	Arachidonate 5-lipoxygenase	P09917	ALOX5	0.112
Polygonatum sibiricum(PGS)	MOL002714	baicalein	Alpha-2B adrenergic receptor	P18089	ADRA2B	0.112
Polygonatum sibiricum(PGS)	MOL002714	baicalein	D(1B) dopamine receptor	P21918	DRD5	0.112
Polygonatum sibiricum(PGS)	MOL002714	baicalein	Cell division protein kinase 5	Q00535	CDK5	0.127
Polygonatum sibiricum(PGS)	MOL002714	baicalein	DNA topoisomerase 2-alpha	P11388	TOP2A	0.128
Polygonatum sibiricum(PGS)	MOL002714	baicalein	Alpha-1B adrenergic receptor	P35368	ADRA1B	0.129
Polygonatum sibiricum(PGS)	MOL002714	baicalein	Progesterone receptor	P06401	PGR	0.136
Polygonatum sibiricum(PGS)	MOL002714	baicalein	Estradiol 17-beta-dehydrogenase 1	P14061	HSD17B1	0.142
Polygonatum sibiricum(PGS)	MOL002714	baicalein	D(2) dopamine receptor	P14416	DRD2	0.145
Polygonatum sibiricum(PGS)	MOL002714	baicalein	Hemoglobin subunit alpha	P69905	HBA1	0.146
Polygonatum sibiricum(PGS)	MOL002714	baicalein	Peroxisome proliferator-activated receptor gamma	P37231	PPARG	0.151
Polygonatum sibiricum(PGS)	MOL002714	baicalein	Delta-type opioid receptor	P41143	OPRD1	0.156

Polygonatum sibiricum(PGS)	MOL002714	baicalein	RAC-alpha serine/threonine-protein kinase	P31749	AKT1	0.158
Polygonatum sibiricum(PGS)	MOL002714	baicalein	Alpha-1A adrenergic receptor	P35348	ADRA1A	0.162
Polygonatum sibiricum(PGS)	MOL002714	baicalein	D(1A) dopamine receptor	P21728	DRD1	0.178
Polygonatum sibiricum(PGS)	MOL002714	baicalein	cAMP-specific 3',5'-cyclic phosphodiesterase 4B	Q07343	PDE4B	0.192
Polygonatum sibiricum(PGS)	MOL002714	baicalein	Prothrombin	P00734	F2	0.197
Polygonatum sibiricum(PGS)	MOL002714	baicalein	Kappa-type opioid receptor	P41145	OPRK1	0.22
Polygonatum sibiricum(PGS)	MOL002714	baicalein	Nitric oxide synthase, inducible	P35228	NOS2	0.237
Polygonatum sibiricum(PGS)	MOL002714	baicalein	Cell division protein kinase 2	P24941	CDK2	0.241
Polygonatum sibiricum(PGS)	MOL002714	baicalein	Nuclear receptor coactivator 1	Q15788	NCOA1	0.271
Polygonatum sibiricum(PGS)	MOL002714	baicalein	Trypsin-1	P07477	PRSS1	0.332
Polygonatum sibiricum(PGS)	MOL002714	baicalein	Mu-type opioid receptor	P35372	OPRM1	0.402
Polygonatum sibiricum(PGS)	MOL002714	baicalein	Prostaglandin G/H synthase 1	P23219	PTGS1	0.422
Polygonatum sibiricum(PGS)	MOL002714	baicalein	cAMP-dependent protein kinase catalytic subunit alpha	P17612	PRKACA	0.441
Polygonatum sibiricum(PGS)	MOL002714	baicalein	Estrogen receptor beta	Q92731	ESR2	0.491
Polygonatum sibiricum(PGS)	MOL002714	baicalein	Cyclin-A2	P20248	CCNA2	0.677
Polygonatum sibiricum(PGS)	MOL002714	baicalein	Prostaglandin G/H synthase 2	P35354	PTGS2	0.778
Polygonatum sibiricum(PGS)	MOL002714	baicalein	Estrogen receptor	P03372	ESR1	1
Polygonatum sibiricum(PGS)	MOL002959	3'-Methoxydaidzein	Serine/threonine-protein kinase 17B	O94768	STK17B	0.012
Polygonatum sibiricum(PGS)	MOL002959	3'-Methoxydaidzein	Proto-oncogene serine/threonine-protein kinase Pim-1	P11309	PIM1	0.012
Polygonatum sibiricum(PGS)	MOL002959	3'-Methoxydaidzein	ATP synthase subunit gamma, mitochondrial	P36542	ATP5F1C	0.012
Polygonatum sibiricum(PGS)	MOL002959	3'-Methoxydaidzein	5-hydroxytryptamine 3 receptor	P46098	HTR3A	0.012
Polygonatum sibiricum(PGS)	MOL002959	3'-Methoxydaidzein	Troponin C, slow skeletal and cardiac muscles	P63316	TNNC1	0.012
Polygonatum sibiricum(PGS)	MOL002959	3'-Methoxydaidzein	ATP-sensitive inward rectifier potassium channel 11	Q14654	KCNJ11	0.012
Polygonatum sibiricum(PGS)	MOL002959	3'-Methoxydaidzein	ATP-sensitive inward rectifier potassium channel 8	Q15842	KCNJ8	0.012
Polygonatum sibiricum(PGS)	MOL002959	3'-Methoxydaidzein	UDP-glucuronosyltransferase 3A1	Q6NUS8	UGT3A1	0.012
Polygonatum sibiricum(PGS)	MOL002959	3'-Methoxydaidzein	Complement factor B	P00751	CFB	0.013
Polygonatum sibiricum(PGS)	MOL002959	3'-Methoxydaidzein	Carbonic anhydrase 1	P00915	CA1	0.013
Polygonatum sibiricum(PGS)	MOL002959	3'-Methoxydaidzein	Carbonic anhydrase 2	P00918	CA2	0.013
Polygonatum sibiricum(PGS)	MOL002959	3'-Methoxydaidzein	Interleukin-3	P08700	IL3	0.013
Polygonatum sibiricum(PGS)	MOL002959	3'-Methoxydaidzein	Furin	P09958	FURIN	0.013
Polygonatum sibiricum(PGS)	MOL002959	3'-Methoxydaidzein	Carbonic anhydrase 4	P22748	CA4	0.013
Polygonatum sibiricum(PGS)	MOL002959	3'-Methoxydaidzein	Protein S100-A12	P80511	S100A12	0.013
Polygonatum sibiricum(PGS)	MOL002959	3'-Methoxydaidzein	Protein S100-A13	Q99584	S100A13	0.013
Polygonatum sibiricum(PGS)	MOL002959	3'-Methoxydaidzein	Glycolipid transfer protein	Q9NZD2	GLTP	0.013
Polygonatum sibiricum(PGS)	MOL002959	3'-Methoxydaidzein	Coagulation factor IX	P00740	F9	0.014
Polygonatum sibiricum(PGS)	MOL002959	3'-Methoxydaidzein	Coagulation factor X	P00742	F10	0.014
Polygonatum sibiricum(PGS)	MOL002959	3'-Methoxydaidzein	Pepsin A	P00790	REN	0.014
Polygonatum sibiricum(PGS)	MOL002959	3'-Methoxydaidzein	Tumor necrosis factor	P01375	TNF	0.014
Polygonatum sibiricum(PGS)	MOL002959	3'-Methoxydaidzein	Osteocalcin	P02818	BGLAP	0.014
Polygonatum sibiricum(PGS)	MOL002959	3'-Methoxydaidzein	Vitamin K-dependent protein C	P04070	PROC	0.014
Polygonatum sibiricum(PGS)	MOL002959	3'-Methoxydaidzein	NAD(P)H dehydrogenase [quinone] 1	P15559	NQO1	0.014
Polygonatum sibiricum(PGS)	MOL002959	3'-Methoxydaidzein	Nuclear factor NF-kappa-B p105 subunit	P19838	NFKB1	0.014
Polygonatum sibiricum(PGS)	MOL002959	3'-Methoxydaidzein	Vitamin K-dependent protein Z	P22891	PROZ	0.014
Polygonatum sibiricum(PGS)	MOL002959	3'-Methoxydaidzein	Amiloride-sensitive sodium channel subunit alpha	P37088	SCNN1A	0.014
Polygonatum sibiricum(PGS)	MOL002959	3'-Methoxydaidzein	Vitamin K-dependent gamma-carboxylase	P38435	GGCX	0.014
Polygonatum sibiricum(PGS)	MOL002959	3'-Methoxydaidzein	Mitogen-activated protein kinase 8	P45983	MAPK8	0.014
Polygonatum sibiricum(PGS)	MOL002959	3'-Methoxydaidzein	Amiloride-sensitive sodium channel subunit beta	P51168	SCNN1B	0.014

Polygonatum sibiricum(PGS)	MOL002959	3'-Methoxydaidzein	Amiloride-sensitive sodium channel subunit gamma	P51170	SCNN1G	0.014
Polygonatum sibiricum(PGS)	MOL002959	3'-Methoxydaidzein	Mitogen-activated protein kinase 10	P53779	MAPK10	0.014
Polygonatum sibiricum(PGS)	MOL002959	3'-Methoxydaidzein	Vitamin K epoxide reductase complex subunit 1-like protein 1	Q8N0U8	VKORC1L1	0.014
Polygonatum sibiricum(PGS)	MOL002959	3'-Methoxydaidzein	Vitamin K epoxide reductase complex subunit 1	Q9BQB6	VKORC1	0.014
Polygonatum sibiricum(PGS)	MOL002959	3'-Methoxydaidzein	C-jun-amino-terminal kinase-interacting protein 1	Q9UQF2	MAPK8IP1	0.014
Polygonatum sibiricum(PGS)	MOL002959	3'-Methoxydaidzein	Phenylalanine-4-hydroxylase	P00439	PAH	0.015
Polygonatum sibiricum(PGS)	MOL002959	3'-Methoxydaidzein	Urokinase-type plasminogen activator	P00749	PLAU	0.015
Polygonatum sibiricum(PGS)	MOL002959	3'-Methoxydaidzein	Pyruvate carboxylase, mitochondrial	P11498	PC	0.015
Polygonatum sibiricum(PGS)	MOL002959	3'-Methoxydaidzein	Peroxiredoxin-5, mitochondrial	P30044	PRDX5	0.015
Polygonatum sibiricum(PGS)	MOL002959	3'-Methoxydaidzein	Potassium voltage-gated channel subfamily H member 2	Q12809	KCNH2	0.015
Polygonatum sibiricum(PGS)	MOL002959	3'-Methoxydaidzein	Potassium voltage-gated channel subfamily H member 6	Q9H252	KCNH6	0.015
Polygonatum sibiricum(PGS)	MOL002959	3'-Methoxydaidzein	Gamma-aminobutyric-acid receptor subunit beta-3	P28472	GABRB3	0.016
Polygonatum sibiricum(PGS)	MOL002959	3'-Methoxydaidzein	Gamma-aminobutyric-acid receptor subunit alpha-4	P48169	GABRA4	0.016
Polygonatum sibiricum(PGS)	MOL002959	3'-Methoxydaidzein	Neuronal acetylcholine receptor subunit alpha-2	Q15822	CHRNA2	0.016
Polygonatum sibiricum(PGS)	MOL002959	3'-Methoxydaidzein	Gamma-aminobutyric-acid receptor subunit alpha-6	Q16445	GABRA6	0.016
Polygonatum sibiricum(PGS)	MOL002959	3'-Methoxydaidzein	cGMP-specific 3',5'-cyclic phosphodiesterase	O76074	PDE5A	0.017
Polygonatum sibiricum(PGS)	MOL002959	3'-Methoxydaidzein	D(4) dopamine receptor	P21917	DRD4	0.017
Polygonatum sibiricum(PGS)	MOL002959	3'-Methoxydaidzein	5-hydroxytryptamine 1D receptor	P28221	HTR1D	0.017
Polygonatum sibiricum(PGS)	MOL002959	3'-Methoxydaidzein	Adenosine A2a receptor	P29274	ADORA2A	0.017
Polygonatum sibiricum(PGS)	MOL002959	3'-Methoxydaidzein	Adenosine A1 receptor	P30542	ADORA1	0.017
Polygonatum sibiricum(PGS)	MOL002959	3'-Methoxydaidzein	5-hydroxytryptamine 2B receptor	P41595	HTR2B	0.017
Polygonatum sibiricum(PGS)	MOL002959	3'-Methoxydaidzein	Bile salt sulfotransferase	Q06520	SULT2A1	0.017
Polygonatum sibiricum(PGS)	MOL002959	3'-Methoxydaidzein	cAMP-specific 3',5'-cyclic phosphodiesterase 4C	Q08493	PDE4C	0.017
Polygonatum sibiricum(PGS)	MOL002959	3'-Methoxydaidzein	cGMP-inhibited 3',5'-cyclic phosphodiesterase A	Q14432	PDE3A	0.017
Polygonatum sibiricum(PGS)	MOL002959	3'-Methoxydaidzein	cAMP and cAMP-inhibited cGMP 3',5'-cyclic phosphodiesterase 10A	Q9Y233	PDE10A	0.017
Polygonatum sibiricum(PGS)	MOL002959	3'-Methoxydaidzein	DNA topoisomerase 1	P11387	TOP1	0.019
Polygonatum sibiricum(PGS)	MOL002959	3'-Methoxydaidzein	4-aminobutyrate aminotransferase, mitochondrial	P80404	ABAT	0.019
Polygonatum sibiricum(PGS)	MOL002959	3'-Methoxydaidzein	DNA topoisomerase I, mitochondrial	Q969P6	TOP1MT	0.019
Polygonatum sibiricum(PGS)	MOL002959	3'-Methoxydaidzein	Histone deacetylase 9	Q9UKV0	HDAC9	0.019
Polygonatum sibiricum(PGS)	MOL002959	3'-Methoxydaidzein	Muscarinic acetylcholine receptor M2	P08172	CHRM2	0.02
Polygonatum sibiricum(PGS)	MOL002959	3'-Methoxydaidzein	Muscarinic acetylcholine receptor M4	P08173	CHRM4	0.02
Polygonatum sibiricum(PGS)	MOL002959	3'-Methoxydaidzein	Muscarinic acetylcholine receptor M1	P11229	CHRM1	0.02
Polygonatum sibiricum(PGS)	MOL002959	3'-Methoxydaidzein	Muscarinic acetylcholine receptor M3	P20309	CHRM3	0.02
Polygonatum sibiricum(PGS)	MOL002959	3'-Methoxydaidzein	Sodium-dependent noradrenaline transporter	P23975	SLC6A2	0.02
Polygonatum sibiricum(PGS)	MOL002959	3'-Methoxydaidzein	Peptidyl-prolyl cis-trans isomerase, mitochondrial	P30405	PPIF	0.02
Polygonatum sibiricum(PGS)	MOL002959	3'-Methoxydaidzein	Mu-type opioid receptor	P35372	OPRM1	0.02
Polygonatum sibiricum(PGS)	MOL002959	3'-Methoxydaidzein	Kappa-type opioid receptor	P41145	OPRK1	0.02
Polygonatum sibiricum(PGS)	MOL002959	3'-Methoxydaidzein	Gamma-aminobutyric acid receptor subunit theta	Q9UN88	GABRQ	0.02
Polygonatum sibiricum(PGS)	MOL002959	3'-Methoxydaidzein	Retinoic acid receptor gamma-1	P13631	RARG	0.021
Polygonatum sibiricum(PGS)	MOL002959	3'-Methoxydaidzein	Retinoic acid receptor RXR-alpha	P19793	RXRA	0.021
Polygonatum sibiricum(PGS)	MOL002959	3'-Methoxydaidzein	Tubulin beta-2C chain	P68371	TUBB4B	0.022
Polygonatum sibiricum(PGS)	MOL002959	3'-Methoxydaidzein	Tubulin alpha-3 chain	Q71U36	TUBA1A	0.022
Polygonatum sibiricum(PGS)	MOL002959	3'-Methoxydaidzein	Sterol O-acyltransferase 2	O75908	SOAT2	0.023
Polygonatum sibiricum(PGS)	MOL002959	3'-Methoxydaidzein	Sterol O-acyltransferase 1	P35610	SOAT1	0.023
Polygonatum sibiricum(PGS)	MOL002959	3'-Methoxydaidzein	Sodium-dependent dopamine transporter	Q01959	SLC6A3	0.023
Polygonatum sibiricum(PGS)	MOL002959	3'-Methoxydaidzein	Gamma-aminobutyric-acid receptor subunit beta-2	P47870	GABRB2	0.024
Polygonatum sibiricum(PGS)	MOL002959	3'-Methoxydaidzein	Glutamate [NMDA] receptor subunit 3A	Q8TCU5	GRIN3A	0.024

Polygonatum sibiricum(PGS)	MOL002959	3'-Methoxydaidzein	Aryl hydrocarbon receptor	P35869	AHR	0.025
Polygonatum sibiricum(PGS)	MOL002959	3'-Methoxydaidzein	Dihydroorotate dehydrogenase, mitochondrial	Q02127	DHODH	0.025
Polygonatum sibiricum(PGS)	MOL002959	3'-Methoxydaidzein	Putative uncharacterized protein	Q7Z3Y4	TRAPPC6B	0.026
Polygonatum sibiricum(PGS)	MOL002959	3'-Methoxydaidzein	5-hydroxytryptamine 1A receptor	P08908	HTR1A	0.027
Polygonatum sibiricum(PGS)	MOL002959	3'-Methoxydaidzein	Gamma-aminobutyric acid receptor subunit gamma-2	P18507	GABRG2	0.027
Polygonatum sibiricum(PGS)	MOL002959	3'-Methoxydaidzein	5-hydroxytryptamine 1B receptor	P28222	HTR1B	0.027
Polygonatum sibiricum(PGS)	MOL002959	3'-Methoxydaidzein	5-hydroxytryptamine 2C receptor	P28335	HTR2C	0.027
Polygonatum sibiricum(PGS)	MOL002959	3'-Methoxydaidzein	ATP synthase subunit beta, mitochondrial	P06576	ATP5F1B	0.028
Polygonatum sibiricum(PGS)	MOL002959	3'-Methoxydaidzein	Tyrosine-protein kinase HCK	P08631	HCK	0.028
Polygonatum sibiricum(PGS)	MOL002959	3'-Methoxydaidzein	Phospholipase A2, membrane associated	P14555	PLA2G2A	0.028
Polygonatum sibiricum(PGS)	MOL002959	3'-Methoxydaidzein	ATP synthase subunit alpha, mitochondrial	P25705	ATP5F1A	0.028
Polygonatum sibiricum(PGS)	MOL002959	3'-Methoxydaidzein	Phosphatidylinositol-4,5-bisphosphate 3-kinase catalytic subunit gamma isoform	P48736	PIK3CG	0.028
Polygonatum sibiricum(PGS)	MOL002959	3'-Methoxydaidzein	Keratin, type II cytoskeletal 7	P08729	KRT7	0.029
Polygonatum sibiricum(PGS)	MOL002959	3'-Methoxydaidzein	Inhibitor of nuclear factor kappa-B kinase subunit alpha	O15111	CHUK	0.03
Polygonatum sibiricum(PGS)	MOL002959	3'-Methoxydaidzein	D-HSCDK2	O75100	CA11	0.03
Polygonatum sibiricum(PGS)	MOL002959	3'-Methoxydaidzein	Group IIE secretory phospholipase A2	Q9NZK7	PLA2G2E	0.03
Polygonatum sibiricum(PGS)	MOL002959	3'-Methoxydaidzein	Gamma-aminobutyric acid receptor subunit rho-3	A8MPY1	GABRR3	0.031
Polygonatum sibiricum(PGS)	MOL002959	3'-Methoxydaidzein	Gamma-aminobutyric acid receptor subunit pi	O00591	GABRP	0.031
Polygonatum sibiricum(PGS)	MOL002959	3'-Methoxydaidzein	Gamma-aminobutyric acid receptor subunit delta	O14764	GABRD	0.031
Polygonatum sibiricum(PGS)	MOL002959	3'-Methoxydaidzein	Gamma-aminobutyric-acid receptor subunit beta-1	P18505	GABRB1	0.031
Polygonatum sibiricum(PGS)	MOL002959	3'-Methoxydaidzein	Fibroblast growth factor receptor 2	P21802	FGFR2	0.031
Polygonatum sibiricum(PGS)	MOL002959	3'-Methoxydaidzein	Gamma-aminobutyric-acid receptor subunit rho-1	P24046	GABRR1	0.031
Polygonatum sibiricum(PGS)	MOL002959	3'-Methoxydaidzein	Dipeptidyl peptidase 4	P27487	DPP4	0.031
Polygonatum sibiricum(PGS)	MOL002959	3'-Methoxydaidzein	Gamma-aminobutyric acid receptor subunit rho-2	P28476	GABRR2	0.031
Polygonatum sibiricum(PGS)	MOL002959	3'-Methoxydaidzein	cAMP-dependent protein kinase inhibitor alpha	P61925	PKIA	0.031
Polygonatum sibiricum(PGS)	MOL002959	3'-Methoxydaidzein	Gamma-aminobutyric acid receptor subunit epsilon	P78334	GABRE	0.031
Polygonatum sibiricum(PGS)	MOL002959	3'-Methoxydaidzein	Gamma-aminobutyric acid receptor subunit gamma-1	Q8N1C3	GABRG1	0.031
Polygonatum sibiricum(PGS)	MOL002959	3'-Methoxydaidzein	Gamma-aminobutyric acid receptor subunit gamma-3	Q99928	GABRG3	0.031
Polygonatum sibiricum(PGS)	MOL002959	3'-Methoxydaidzein	3 beta-hydroxysteroid dehydrogenase/Delta 5-->4-isomerase type II	P26439	HSD3B2	0.032
Polygonatum sibiricum(PGS)	MOL002959	3'-Methoxydaidzein	Retinoic acid receptor beta	P10826	RARB	0.033
Polygonatum sibiricum(PGS)	MOL002959	3'-Methoxydaidzein	Acetylcholinesterase	P22303	ACHE	0.033
Polygonatum sibiricum(PGS)	MOL002959	3'-Methoxydaidzein	Retinoic acid receptor RXR-beta	P28702	RXRB	0.033
Polygonatum sibiricum(PGS)	MOL002959	3'-Methoxydaidzein	Retinoic acid receptor RXR-gamma	P48443	RXRG	0.033
Polygonatum sibiricum(PGS)	MOL002959	3'-Methoxydaidzein	Beta-2 adrenergic receptor	P07550	ADRB2	0.034
Polygonatum sibiricum(PGS)	MOL002959	3'-Methoxydaidzein	Beta-1 adrenergic receptor	P08588	ADRB1	0.034
Polygonatum sibiricum(PGS)	MOL002959	3'-Methoxydaidzein	Mineralocorticoid receptor	P08235	NR3C2	0.036
Polygonatum sibiricum(PGS)	MOL002959	3'-Methoxydaidzein	Prolactin receptor	P16471	PRLR	0.036
Polygonatum sibiricum(PGS)	MOL002959	3'-Methoxydaidzein	Sodium channel protein type 10 subunit alpha	Q9Y5Y9	SCN10A	0.036
Polygonatum sibiricum(PGS)	MOL002959	3'-Methoxydaidzein	D(1B) dopamine receptor	P21918	DRD5	0.038
Polygonatum sibiricum(PGS)	MOL002959	3'-Methoxydaidzein	DNA topoisomerase 2-alpha	P11388	TOP2A	0.039
Polygonatum sibiricum(PGS)	MOL002959	3'-Methoxydaidzein	Ribosyldihydronicotinamide dehydrogenase [quinone]	P16083	NQO2	0.039
Polygonatum sibiricum(PGS)	MOL002959	3'-Methoxydaidzein	Gamma-aminobutyric-acid receptor subunit alpha-5	P31644	GABRA5	0.039
Polygonatum sibiricum(PGS)	MOL002959	3'-Methoxydaidzein	Protein tyrosine kinase 2 beta	Q14289	PTK2B	0.039
Polygonatum sibiricum(PGS)	MOL002959	3'-Methoxydaidzein	5-hydroxytryptamine 2A receptor	P28223	HTR2A	0.04
Polygonatum sibiricum(PGS)	MOL002959	3'-Methoxydaidzein	Geranylgeranyl pyrophosphate synthetase	O95749	GGPS1	0.041
Polygonatum sibiricum(PGS)	MOL002959	3'-Methoxydaidzein	Sodium-dependent serotonin transporter	P31645	SLC6A4	0.041
Polygonatum sibiricum(PGS)	MOL002959	3'-Methoxydaidzein	Aldo-keto reductase family 1 member C1	Q04828	AKR1C1	0.041

Polygonatum sibiricum(PGS)	MOL002959	3'-Methoxydaidzein	Casein kinase I isoform gamma-1	Q9HCP0	CSNK1G1	0.042
Polygonatum sibiricum(PGS)	MOL002959	3'-Methoxydaidzein	Epidermal growth factor receptor	P00533	EGFR	0.043
Polygonatum sibiricum(PGS)	MOL002959	3'-Methoxydaidzein	Glyceraldehyde-3-phosphate dehydrogenase	P04406	GAPDH	0.043
Polygonatum sibiricum(PGS)	MOL002959	3'-Methoxydaidzein	Cytochrome P450 19A1	P11511	CYP19A1	0.043
Polygonatum sibiricum(PGS)	MOL002959	3'-Methoxydaidzein	RAC-alpha serine/threonine-protein kinase	P31749	AKT1	0.043
Polygonatum sibiricum(PGS)	MOL002959	3'-Methoxydaidzein	Gamma-aminobutyric-acid receptor subunit alpha-3	P34903	GABRA3	0.043
Polygonatum sibiricum(PGS)	MOL002959	3'-Methoxydaidzein	Toll-like receptor 7	Q9NYK1	TLR7	0.043
Polygonatum sibiricum(PGS)	MOL002959	3'-Methoxydaidzein	Ig kappa chain V-II region RPMI 6410	P06310	IGKV2-30	0.044
Polygonatum sibiricum(PGS)	MOL002959	3'-Methoxydaidzein	Alpha-1D adrenergic receptor	P25100	ADRA1D	0.044
Polygonatum sibiricum(PGS)	MOL002959	3'-Methoxydaidzein	Delta-type opioid receptor	P41143	OPRD1	0.044
Polygonatum sibiricum(PGS)	MOL002959	3'-Methoxydaidzein	Hydroxyacid oxidase 1	Q9UJM8	HAO1	0.044
Polygonatum sibiricum(PGS)	MOL002959	3'-Methoxydaidzein	Alpha-2A adrenergic receptor	P08913	ADRA2A	0.045
Polygonatum sibiricum(PGS)	MOL002959	3'-Methoxydaidzein	Retinoic acid receptor alpha	P10276	RARA	0.045
Polygonatum sibiricum(PGS)	MOL002959	3'-Methoxydaidzein	Cannabinoid receptor 1	P21554	CNR1	0.045
Polygonatum sibiricum(PGS)	MOL002959	3'-Methoxydaidzein	Melatonin receptor type 1A	P48039	MTNR1A	0.045
Polygonatum sibiricum(PGS)	MOL002959	3'-Methoxydaidzein	Interferon gamma	P01579	IFNG	0.046
Polygonatum sibiricum(PGS)	MOL002959	3'-Methoxydaidzein	Alpha-2C adrenergic receptor	P18825	ADRA2C	0.046
Polygonatum sibiricum(PGS)	MOL002959	3'-Methoxydaidzein	Casein kinase II subunit alpha	P68400	CSNK2A1	0.046
Polygonatum sibiricum(PGS)	MOL002959	3'-Methoxydaidzein	Coagulation factor VII	P08709	F7	0.047
Polygonatum sibiricum(PGS)	MOL002959	3'-Methoxydaidzein	Thyroid hormone receptor alpha	P10827	THRA	0.047
Polygonatum sibiricum(PGS)	MOL002959	3'-Methoxydaidzein	Gamma-aminobutyric-acid receptor subunit alpha-1	P14867	GABRA1	0.047
Polygonatum sibiricum(PGS)	MOL002959	3'-Methoxydaidzein	Gamma-aminobutyric-acid receptor subunit alpha-2	P47869	GABRA2	0.047
Polygonatum sibiricum(PGS)	MOL002959	3'-Methoxydaidzein	Calmodulin	P62158		0.047
Polygonatum sibiricum(PGS)	MOL002959	3'-Methoxydaidzein	Activin receptor type-1	Q04771	ACVR1	0.047
Polygonatum sibiricum(PGS)	MOL002959	3'-Methoxydaidzein	Solute carrier family 12 member 1	Q13621	SLC12A1	0.047
Polygonatum sibiricum(PGS)	MOL002959	3'-Methoxydaidzein	Colipase	P04118	CLPS	0.048
Polygonatum sibiricum(PGS)	MOL002959	3'-Methoxydaidzein	Thyroid hormone receptor beta-1	P10828	THRB	0.048
Polygonatum sibiricum(PGS)	MOL002959	3'-Methoxydaidzein	D(1A) dopamine receptor	P21728	DRD1	0.048
Polygonatum sibiricum(PGS)	MOL002959	3'-Methoxydaidzein	D(3) dopamine receptor	P35462	DRD3	0.048
Polygonatum sibiricum(PGS)	MOL002959	3'-Methoxydaidzein	cAMP-specific 3',5'-cyclic phosphodiesterase 4D	Q08499	PDE4D	0.048
Polygonatum sibiricum(PGS)	MOL002959	3'-Methoxydaidzein	Dihydrofolate reductase	P00374	DHFR	0.049
Polygonatum sibiricum(PGS)	MOL002959	3'-Methoxydaidzein	Proto-oncogene tyrosine-protein kinase Src	P12931	SRC	0.049
Polygonatum sibiricum(PGS)	MOL002959	3'-Methoxydaidzein	Neutrophil gelatinase-associated lipocalin	P80188	LCN2	0.049
Polygonatum sibiricum(PGS)	MOL002959	3'-Methoxydaidzein	Nuclear receptor coactivator 5	Q9HCD5	NCOA5	0.049
Polygonatum sibiricum(PGS)	MOL002959	3'-Methoxydaidzein	Alpha-1B adrenergic receptor	P35368	ADRA1B	0.053
Polygonatum sibiricum(PGS)	MOL002959	3'-Methoxydaidzein	Alpha-2B adrenergic receptor	P18089	ADRA2B	0.055
Polygonatum sibiricum(PGS)	MOL002959	3'-Methoxydaidzein	Ig gamma-1 chain C region	P01857	IGHG1	0.056
Polygonatum sibiricum(PGS)	MOL002959	3'-Methoxydaidzein	Ig kappa chain C region	P01834	IGKC	0.057
Polygonatum sibiricum(PGS)	MOL002959	3'-Methoxydaidzein	cAMP-specific 3',5'-cyclic phosphodiesterase 4B	Q07343	PDE4B	0.057
Polygonatum sibiricum(PGS)	MOL002959	3'-Methoxydaidzein	3 beta-hydroxysteroid dehydrogenase/Delta 5-->4-isomerase type I	P14060	HSD3B1	0.058
Polygonatum sibiricum(PGS)	MOL002959	3'-Methoxydaidzein	Arachidonate 5-lipoxygenase	P09917	ALOX5	0.059
Polygonatum sibiricum(PGS)	MOL002959	3'-Methoxydaidzein	Peroxisome proliferator-activated receptor gamma	P37231	PPARG	0.059
Polygonatum sibiricum(PGS)	MOL002959	3'-Methoxydaidzein	Phospholipase A2	P04054	PLA2G1B	0.061
Polygonatum sibiricum(PGS)	MOL002959	3'-Methoxydaidzein	Alpha-1A adrenergic receptor	P35348	ADRA1A	0.063
Polygonatum sibiricum(PGS)	MOL002959	3'-Methoxydaidzein	Cell division control protein 2 homolog	P06493	CDK1	0.065
Polygonatum sibiricum(PGS)	MOL002959	3'-Methoxydaidzein	Estradiol 17-beta-dehydrogenase 1	P14061	HSD17B1	0.066
Polygonatum sibiricum(PGS)	MOL002959	3'-Methoxydaidzein	Rho-associated protein kinase 1	Q13464	ROCK1	0.066

Polygonatum sibiricum(PGS)	MOL002959	3'-Methoxydaidzein	Prothrombin	P00734	F2	0.068
Polygonatum sibiricum(PGS)	MOL002959	3'-Methoxydaidzein	D(2) dopamine receptor	P14416	DRD2	0.069
Polygonatum sibiricum(PGS)	MOL002959	3'-Methoxydaidzein	Sodium channel protein type 5 subunit alpha	Q14524	SCN5A	0.073
Polygonatum sibiricum(PGS)	MOL002959	3'-Methoxydaidzein	Progesterone receptor	P06401	PGR	0.075
Polygonatum sibiricum(PGS)	MOL002959	3'-Methoxydaidzein	Androgen receptor	P10275	AR	0.075
Polygonatum sibiricum(PGS)	MOL002959	3'-Methoxydaidzein	Estrogen-related receptor gamma	P62508	ESRRG	0.078
Polygonatum sibiricum(PGS)	MOL002959	3'-Methoxydaidzein	Prostaglandin G/H synthase 1	P23219	PTGS1	0.083
Polygonatum sibiricum(PGS)	MOL002959	3'-Methoxydaidzein	Trypsin-1	P07477	PRSS1	0.089
Polygonatum sibiricum(PGS)	MOL002959	3'-Methoxydaidzein	Nuclear receptor coactivator 2	Q15596	NCOA2	0.09
Polygonatum sibiricum(PGS)	MOL002959	3'-Methoxydaidzein	Nitric oxide synthase, inducible	P35228	NOS2	0.092
Polygonatum sibiricum(PGS)	MOL002959	3'-Methoxydaidzein	Mitogen-activated protein kinase 14	Q16539	MAPK14	0.093
Polygonatum sibiricum(PGS)	MOL002959	3'-Methoxydaidzein	Serine/threonine-protein kinase 6	O14965	AURKA	0.094
Polygonatum sibiricum(PGS)	MOL002959	3'-Methoxydaidzein	ATP-sensitive inward rectifier potassium channel 1	P48048	KCNJ1	0.096
Polygonatum sibiricum(PGS)	MOL002959	3'-Methoxydaidzein	Cell division protein kinase 5	Q00535	CDK5	0.099
Polygonatum sibiricum(PGS)	MOL002959	3'-Methoxydaidzein	cAMP-specific 3',5'-cyclic phosphodiesterase 4A	P27815	PDE4A	0.118
Polygonatum sibiricum(PGS)	MOL002959	3'-Methoxydaidzein	MAP kinase-activated protein kinase 2	P49137	MAPKAPK2	0.15
Polygonatum sibiricum(PGS)	MOL002959	3'-Methoxydaidzein	Hemoglobin subunit alpha	P69905	HBA1	0.196
Polygonatum sibiricum(PGS)	MOL002959	3'-Methoxydaidzein	Cyclin-A2	P20248	CCNA2	0.232
Polygonatum sibiricum(PGS)	MOL002959	3'-Methoxydaidzein	Cell division protein kinase 2	P24941	CDK2	0.251
Polygonatum sibiricum(PGS)	MOL002959	3'-Methoxydaidzein	Nuclear receptor coactivator 1	Q15788	NCOA1	0.258
Polygonatum sibiricum(PGS)	MOL002959	3'-Methoxydaidzein	cAMP-dependent protein kinase catalytic subunit alpha	P17612	PRKACA	0.277
Polygonatum sibiricum(PGS)	MOL002959	3'-Methoxydaidzein	Prostaglandin G/H synthase 2	P35354	PTGS2	0.423
Polygonatum sibiricum(PGS)	MOL002959	3'-Methoxydaidzein	Estrogen receptor beta	Q92731	ESR2	0.43
Polygonatum sibiricum(PGS)	MOL002959	3'-Methoxydaidzein	Estrogen receptor	P03372	ESR1	1
Polygonatum sibiricum(PGS)	MOL003889	methylprotodioscin_qt	Elongation factor Tu GTP-binding domain-containing protein 1	Q7Z2Z2	EFL1	0.029
Polygonatum sibiricum(PGS)	MOL003889	methylprotodioscin_qt	Muscarinic acetylcholine receptor M4	P08173	CHRM4	0.037
Polygonatum sibiricum(PGS)	MOL003889	methylprotodioscin_qt	Muscarinic acetylcholine receptor M5	P08912	CHRM5	0.037
Polygonatum sibiricum(PGS)	MOL003889	methylprotodioscin_qt	Muscarinic acetylcholine receptor M1	P11229	CHRM1	0.037
Polygonatum sibiricum(PGS)	MOL003889	methylprotodioscin_qt	Histamine H1 receptor	P35367	HRH1	0.037
Polygonatum sibiricum(PGS)	MOL003889	methylprotodioscin_qt	Opioid receptor, sigma 1	Q5T1J1	SIGMAR1	0.039
Polygonatum sibiricum(PGS)	MOL003889	methylprotodioscin_qt	Glutamate [NMDA] receptor subunit 3A	Q8TCU5	GRIN3A	0.039
Polygonatum sibiricum(PGS)	MOL003889	methylprotodioscin_qt	Sigma 1-type opioid receptor	Q99720	SIGMAR1	0.039
Polygonatum sibiricum(PGS)	MOL003889	methylprotodioscin_qt	Retinoic acid receptor RXR-alpha	P19793	RXRA	0.04
Polygonatum sibiricum(PGS)	MOL003889	methylprotodioscin_qt	Nuclear receptor subfamily 1 group I member 3	Q14994	NR1I3	0.04
Polygonatum sibiricum(PGS)	MOL003889	methylprotodioscin_qt	Nuclear receptor coactivator 1	Q15788	NCOA1	0.04
Polygonatum sibiricum(PGS)	MOL003889	methylprotodioscin_qt	Ig kappa chain C region	P01834	IGKC	0.044
Polygonatum sibiricum(PGS)	MOL003889	methylprotodioscin_qt	Ig gamma-1 chain C region	P01857	IGHG1	0.044
Polygonatum sibiricum(PGS)	MOL003889	methylprotodioscin_qt	Ig gamma-2 chain C region	P01859	IGHG2	0.044
Polygonatum sibiricum(PGS)	MOL003889	methylprotodioscin_qt	Dehydrogenase/reductase SDR family member 8	Q8NBQ5	HSD17B11	0.045
Polygonatum sibiricum(PGS)	MOL003889	methylprotodioscin_qt	3 beta-hydroxysteroid dehydrogenase/Delta 5-->4-isomerase type II	P26439	HSD3B2	0.064
Polygonatum sibiricum(PGS)	MOL003889	methylprotodioscin_qt	Annexin A1	P04083	ANXA1	0.068
Polygonatum sibiricum(PGS)	MOL003889	methylprotodioscin_qt	Microtubule-associated protein 2	P11137	MAP2	0.068
Polygonatum sibiricum(PGS)	MOL003889	methylprotodioscin_qt	3-oxo-5-alpha-steroid 4-dehydrogenase 2	P31213	SRD5A2	0.068
Polygonatum sibiricum(PGS)	MOL003889	methylprotodioscin_qt	Nuclear receptor 0B1	P51843	NR0B1	0.068
Polygonatum sibiricum(PGS)	MOL003889	methylprotodioscin_qt	Microtubule-associated protein 1A	P78559	MAP1A	0.068
Polygonatum sibiricum(PGS)	MOL003889	methylprotodioscin_qt	Estrogen receptor beta	Q92731	ESR2	0.068
Polygonatum sibiricum(PGS)	MOL003889	methylprotodioscin_qt	Prolactin receptor	P16471	PRLR	0.069

Polygonatum sibiricum(PGS)	MOL003889	methylprotodioscin_qt	Gonadotropin-releasing hormone receptor	P30968	GNRHR	0.069
Polygonatum sibiricum(PGS)	MOL003889	methylprotodioscin_qt	Cytosolic phospholipase A2	P47712	PLA2G4A	0.069
Polygonatum sibiricum(PGS)	MOL003889	methylprotodioscin_qt	Gonadotropin-releasing hormone II receptor	Q96P88	GNRHR2	0.069
Polygonatum sibiricum(PGS)	MOL003889	methylprotodioscin_qt	Bile salt sulfotransferase	Q06520	SULT2A1	0.096
Polygonatum sibiricum(PGS)	MOL003889	methylprotodioscin_qt	3 beta-hydroxysteroid dehydrogenase/Delta 5-->4-isomerase type I	P14060	HSD3B1	0.128
Polygonatum sibiricum(PGS)	MOL003889	methylprotodioscin_qt	Mediator of RNA polymerase II transcription subunit 1	Q15648	MED1	0.129
Polygonatum sibiricum(PGS)	MOL003889	methylprotodioscin_qt	NADPH oxidase organizer 1	Q8NFA2	NOXO1	0.131
Polygonatum sibiricum(PGS)	MOL003889	methylprotodioscin_qt	3-oxo-5-alpha-steroid 4-dehydrogenase 1	P18405	SRD5A1	0.145
Polygonatum sibiricum(PGS)	MOL003889	methylprotodioscin_qt	Aldo-keto reductase family 1 member C1	Q04828	AKR1C1	0.146
Polygonatum sibiricum(PGS)	MOL003889	methylprotodioscin_qt	Nuclear receptor coactivator 5	Q9HCD5	NCOA5	0.146
Polygonatum sibiricum(PGS)	MOL003889	methylprotodioscin_qt	Muscarinic acetylcholine receptor M2	P08172	CHRM2	0.176
Polygonatum sibiricum(PGS)	MOL003889	methylprotodioscin_qt	Neuronal acetylcholine receptor subunit alpha-2	Q15822	CHRNA2	0.178
Polygonatum sibiricum(PGS)	MOL003889	methylprotodioscin_qt	Androgen receptor	P10275	AR	0.223
Polygonatum sibiricum(PGS)	MOL003889	methylprotodioscin_qt	Estradiol 17-beta-dehydrogenase 1	P14061	HSD17B1	0.224
Polygonatum sibiricum(PGS)	MOL003889	methylprotodioscin_qt	Glucocorticoid receptor	P04150	NR3C1	0.298
Polygonatum sibiricum(PGS)	MOL003889	methylprotodioscin_qt	Mineralocorticoid receptor	P08235	NR3C2	0.302
Polygonatum sibiricum(PGS)	MOL003889	methylprotodioscin_qt	Estrogen receptor	P03372	ESR1	0.903
Polygonatum sibiricum(PGS)	MOL003889	methylprotodioscin_qt	Progesterone receptor	P06401	PGR	1
Polygonatum sibiricum(PGS)	MOL004941	(2R)-7-hydroxy-2-(4-hydroxyphenyl)chroman-4-one	DNA polymerase alpha catalytic subunit	P09884	POLA1	0.01
Polygonatum sibiricum(PGS)	MOL004941	(2R)-7-hydroxy-2-(4-hydroxyphenyl)chroman-4-one	Ribonucleoside-diphosphate reductase large subunit	P23921	RRM1	0.01
Polygonatum sibiricum(PGS)	MOL004941	(2R)-7-hydroxy-2-(4-hydroxyphenyl)chroman-4-one	Ribonucleoside-diphosphate reductase M2 subunit	P31350	RRM2	0.01
Polygonatum sibiricum(PGS)	MOL004941	(2R)-7-hydroxy-2-(4-hydroxyphenyl)chroman-4-one	Sodium channel protein type 1 subunit alpha	P35498	SCN1A	0.01
Polygonatum sibiricum(PGS)	MOL004941	(2R)-7-hydroxy-2-(4-hydroxyphenyl)chroman-4-one	DNA polymerase epsilon subunit 2	P56282	POLE2	0.01
Polygonatum sibiricum(PGS)	MOL004941	(2R)-7-hydroxy-2-(4-hydroxyphenyl)chroman-4-one	DNA polymerase epsilon catalytic subunit A	Q07864	POLE	0.01
Polygonatum sibiricum(PGS)	MOL004941	(2R)-7-hydroxy-2-(4-hydroxyphenyl)chroman-4-one	Ribonucleoside-diphosphate reductase subunit M2 B	Q7LG56	RRM2B	0.01
Polygonatum sibiricum(PGS)	MOL004941	(2R)-7-hydroxy-2-(4-hydroxyphenyl)chroman-4-one	DNA polymerase epsilon subunit 3	Q9NRF9	POLE3	0.01
Polygonatum sibiricum(PGS)	MOL004941	(2R)-7-hydroxy-2-(4-hydroxyphenyl)chroman-4-one	Serine/threonine-protein kinase 17B	O94768	STK17B	0.012
Polygonatum sibiricum(PGS)	MOL004941	(2R)-7-hydroxy-2-(4-hydroxyphenyl)chroman-4-one	Tyrosine-protein kinase HCK	P08631	HCK	0.012
Polygonatum sibiricum(PGS)	MOL004941	(2R)-7-hydroxy-2-(4-hydroxyphenyl)chroman-4-one	Proto-oncogene serine/threonine-protein kinase Pim-1	P11309	PIM1	0.012
Polygonatum sibiricum(PGS)	MOL004941	(2R)-7-hydroxy-2-(4-hydroxyphenyl)chroman-4-one	D(4) dopamine receptor	P21917	DRD4	0.012
Polygonatum sibiricum(PGS)	MOL004941	(2R)-7-hydroxy-2-(4-hydroxyphenyl)chroman-4-one	5-hydroxytryptamine 1D receptor	P28221	HTR1D	0.012
Polygonatum sibiricum(PGS)	MOL004941	(2R)-7-hydroxy-2-(4-hydroxyphenyl)chroman-4-one	5-hydroxytryptamine 1B receptor	P28222	HTR1B	0.012
Polygonatum sibiricum(PGS)	MOL004941	(2R)-7-hydroxy-2-(4-hydroxyphenyl)chroman-4-one	ATP synthase subunit gamma, mitochondrial	P36542	ATP5F1C	0.012
Polygonatum sibiricum(PGS)	MOL004941	(2R)-7-hydroxy-2-(4-hydroxyphenyl)chroman-4-one	Glutamate receptor 2	P42262	GRIA2	0.012
Polygonatum sibiricum(PGS)	MOL004941	(2R)-7-hydroxy-2-(4-hydroxyphenyl)chroman-4-one	UDP-glucuronosyltransferase 3A1	Q6NUS8	UGT3A1	0.012
Polygonatum sibiricum(PGS)	MOL004941	(2R)-7-hydroxy-2-(4-hydroxyphenyl)chroman-4-one	D1 dopamine receptor-interacting protein calcyon	Q9NYX4	CALY	0.012
Polygonatum sibiricum(PGS)	MOL004941	(2R)-7-hydroxy-2-(4-hydroxyphenyl)chroman-4-one	Prothrombin	P00734	F2	0.014
Polygonatum sibiricum(PGS)	MOL004941	(2R)-7-hydroxy-2-(4-hydroxyphenyl)chroman-4-one	Interleukin-3	P08700	IL3	0.014
Polygonatum sibiricum(PGS)	MOL004941	(2R)-7-hydroxy-2-(4-hydroxyphenyl)chroman-4-one	Muscarinic acetylcholine receptor M5	P08912	CHRM5	0.014
Polygonatum sibiricum(PGS)	MOL004941	(2R)-7-hydroxy-2-(4-hydroxyphenyl)chroman-4-one	Histamine H2 receptor	P25021	HRH2	0.014
Polygonatum sibiricum(PGS)	MOL004941	(2R)-7-hydroxy-2-(4-hydroxyphenyl)chroman-4-one	Protein S100-B	P04271	S100B	0.015
Polygonatum sibiricum(PGS)	MOL004941	(2R)-7-hydroxy-2-(4-hydroxyphenyl)chroman-4-one	Protein S100-A1	P23297	S100A1	0.015
Polygonatum sibiricum(PGS)	MOL004941	(2R)-7-hydroxy-2-(4-hydroxyphenyl)chroman-4-one	Sodium/potassium-transporting ATPase alpha-1 chain	P05023	ATP1A1	0.016
Polygonatum sibiricum(PGS)	MOL004941	(2R)-7-hydroxy-2-(4-hydroxyphenyl)chroman-4-one	Dehydrogenase/reductase SDR family member 8	Q8NBQ5	HSD17B11	0.016
Polygonatum sibiricum(PGS)	MOL004941	(2R)-7-hydroxy-2-(4-hydroxyphenyl)chroman-4-one	Glutamate [NMDA] receptor subunit epsilon-4	O15399	GRIN2D	0.017
Polygonatum sibiricum(PGS)	MOL004941	(2R)-7-hydroxy-2-(4-hydroxyphenyl)chroman-4-one	Glutamate [NMDA] receptor subunit 3B	O60391	GRIN3B	0.017
Polygonatum sibiricum(PGS)	MOL004941	(2R)-7-hydroxy-2-(4-hydroxyphenyl)chroman-4-one	3 beta-hydroxysteroid dehydrogenase/Delta 5-->4-isomerase type I	P14060	HSD3B1	0.017

Polygonatum sibiricum(PGS)	MOL004941	(2R)-7-hydroxy-2-(4-hydroxyphenyl)chroman-4-one	Mitogen-activated protein kinase 8	P45983	MAPK8	0.017
Polygonatum sibiricum(PGS)	MOL004941	(2R)-7-hydroxy-2-(4-hydroxyphenyl)chroman-4-one	Mitogen-activated protein kinase 10	P53779	MAPK10	0.017
Polygonatum sibiricum(PGS)	MOL004941	(2R)-7-hydroxy-2-(4-hydroxyphenyl)chroman-4-one	Glutamate [NMDA] receptor subunit zeta-1	Q05586	GRIN1	0.017
Polygonatum sibiricum(PGS)	MOL004941	(2R)-7-hydroxy-2-(4-hydroxyphenyl)chroman-4-one	Alpha-7 nicotinic cholinergic receptor subunit	Q693P7	CHRNA7	0.017
Polygonatum sibiricum(PGS)	MOL004941	(2R)-7-hydroxy-2-(4-hydroxyphenyl)chroman-4-one	C-jun-amino-terminal kinase-interacting protein 1	Q9UQF2	MAPK8IP1	0.017
Polygonatum sibiricum(PGS)	MOL004941	(2R)-7-hydroxy-2-(4-hydroxyphenyl)chroman-4-one	High affinity cAMP-specific and IBMX-insensitive 3',5'-cyclic phosphodiesterase 8A	O60658	PDE8A	0.018
Polygonatum sibiricum(PGS)	MOL004941	(2R)-7-hydroxy-2-(4-hydroxyphenyl)chroman-4-one	6-phosphogluconate dehydrogenase, decarboxylating	P52209	PGD	0.018
Polygonatum sibiricum(PGS)	MOL004941	(2R)-7-hydroxy-2-(4-hydroxyphenyl)chroman-4-one	cAMP-specific 3',5'-cyclic phosphodiesterase 4C	Q08493	PDE4C	0.018
Polygonatum sibiricum(PGS)	MOL004941	(2R)-7-hydroxy-2-(4-hydroxyphenyl)chroman-4-one	High-affinity cAMP-specific 3',5'-cyclic phosphodiesterase 7A	Q13946	PDE7A	0.018
Polygonatum sibiricum(PGS)	MOL004941	(2R)-7-hydroxy-2-(4-hydroxyphenyl)chroman-4-one	cGMP-inhibited 3',5'-cyclic phosphodiesterase A	Q14432	PDE3A	0.018
Polygonatum sibiricum(PGS)	MOL004941	(2R)-7-hydroxy-2-(4-hydroxyphenyl)chroman-4-one	Solute carrier family 22 member 6	Q4U2R8	SLC22A6	0.018
Polygonatum sibiricum(PGS)	MOL004941	(2R)-7-hydroxy-2-(4-hydroxyphenyl)chroman-4-one	Solute carrier family 22 member 8	Q8TCC7	SLC22A8	0.018
Polygonatum sibiricum(PGS)	MOL004941	(2R)-7-hydroxy-2-(4-hydroxyphenyl)chroman-4-one	cAMP-specific 3',5'-cyclic phosphodiesterase 7B	Q9NP56	PDE7B	0.018
Polygonatum sibiricum(PGS)	MOL004941	(2R)-7-hydroxy-2-(4-hydroxyphenyl)chroman-4-one	Solute carrier family 22 member 11	Q9NSA0	SLC22A11	0.018
Polygonatum sibiricum(PGS)	MOL004941	(2R)-7-hydroxy-2-(4-hydroxyphenyl)chroman-4-one	cAMP and cAMP-inhibited cGMP 3',5'-cyclic phosphodiesterase 10A	Q9Y233	PDE10A	0.018
Polygonatum sibiricum(PGS)	MOL004941	(2R)-7-hydroxy-2-(4-hydroxyphenyl)chroman-4-one	Substance-P receptor	P25103	TACR1	0.019
Polygonatum sibiricum(PGS)	MOL004941	(2R)-7-hydroxy-2-(4-hydroxyphenyl)chroman-4-one	5-hydroxytryptamine 3 receptor	P46098	HTR3A	0.019
Polygonatum sibiricum(PGS)	MOL004941	(2R)-7-hydroxy-2-(4-hydroxyphenyl)chroman-4-one	Neuronal acetylcholine receptor subunit alpha-10	Q9GZZ6	CHRNA10	0.019
Polygonatum sibiricum(PGS)	MOL004941	(2R)-7-hydroxy-2-(4-hydroxyphenyl)chroman-4-one	Sodium channel protein type 10 subunit alpha	Q9Y5Y9	SCN10A	0.019
Polygonatum sibiricum(PGS)	MOL004941	(2R)-7-hydroxy-2-(4-hydroxyphenyl)chroman-4-one	Stromelysin-1	P08254	MMP3	0.021
Polygonatum sibiricum(PGS)	MOL004941	(2R)-7-hydroxy-2-(4-hydroxyphenyl)chroman-4-one	Stromelysin-2	P09238	MMP10	0.021
Polygonatum sibiricum(PGS)	MOL004941	(2R)-7-hydroxy-2-(4-hydroxyphenyl)chroman-4-one	Macrophage metalloelastase	P39900	MMP12	0.021
Polygonatum sibiricum(PGS)	MOL004941	(2R)-7-hydroxy-2-(4-hydroxyphenyl)chroman-4-one	Purine nucleoside phosphorylase	P00491	PNP	0.024
Polygonatum sibiricum(PGS)	MOL004941	(2R)-7-hydroxy-2-(4-hydroxyphenyl)chroman-4-one	Mineralocorticoid receptor	P08235	NR3C2	0.025
Polygonatum sibiricum(PGS)	MOL004941	(2R)-7-hydroxy-2-(4-hydroxyphenyl)chroman-4-one	Corticosteroid 11-beta-dehydrogenase isozyme 1	P28845	HSD11B1	0.025
Polygonatum sibiricum(PGS)	MOL004941	(2R)-7-hydroxy-2-(4-hydroxyphenyl)chroman-4-one	Translocator protein	P30536	TSPO	0.025
Polygonatum sibiricum(PGS)	MOL004941	(2R)-7-hydroxy-2-(4-hydroxyphenyl)chroman-4-one	Thymidine kinase 2, mitochondrial	O00142	TK2	0.026
Polygonatum sibiricum(PGS)	MOL004941	(2R)-7-hydroxy-2-(4-hydroxyphenyl)chroman-4-one	Carbonic anhydrase 1	P00915	CA1	0.026
Polygonatum sibiricum(PGS)	MOL004941	(2R)-7-hydroxy-2-(4-hydroxyphenyl)chroman-4-one	Sepiapterin reductase	P35270	SPR	0.026
Polygonatum sibiricum(PGS)	MOL004941	(2R)-7-hydroxy-2-(4-hydroxyphenyl)chroman-4-one	6-pyruvoyl tetrahydrobiopterin synthase	Q03393	PTS	0.026
Polygonatum sibiricum(PGS)	MOL004941	(2R)-7-hydroxy-2-(4-hydroxyphenyl)chroman-4-one	72 kDa type IV collagenase	P08253	MMP2	0.027
Polygonatum sibiricum(PGS)	MOL004941	(2R)-7-hydroxy-2-(4-hydroxyphenyl)chroman-4-one	Angiotensin-converting enzyme	P12821	ACE	0.027
Polygonatum sibiricum(PGS)	MOL004941	(2R)-7-hydroxy-2-(4-hydroxyphenyl)chroman-4-one	Succinate dehydrogenase [ubiquinone] cytochrome b small subunit, mitochondrial	O14521	SDHD	0.029
Polygonatum sibiricum(PGS)	MOL004941	(2R)-7-hydroxy-2-(4-hydroxyphenyl)chroman-4-one	Phenylalanine-4-hydroxylase	P00439	PAH	0.029
Polygonatum sibiricum(PGS)	MOL004941	(2R)-7-hydroxy-2-(4-hydroxyphenyl)chroman-4-one	Ribosyldihydronicotinamide dehydrogenase [quinone]	P16083	NQO2	0.029
Polygonatum sibiricum(PGS)	MOL004941	(2R)-7-hydroxy-2-(4-hydroxyphenyl)chroman-4-one	D(3) dopamine receptor	P35462	DRD3	0.029
Polygonatum sibiricum(PGS)	MOL004941	(2R)-7-hydroxy-2-(4-hydroxyphenyl)chroman-4-one	Tyrosyl-tRNA synthetase, cytoplasmic	P54577	YARS	0.029
Polygonatum sibiricum(PGS)	MOL004941	(2R)-7-hydroxy-2-(4-hydroxyphenyl)chroman-4-one	Peptidyl-prolyl cis-trans isomerase NIMA-interacting 1	Q13526	PIN1	0.029
Polygonatum sibiricum(PGS)	MOL004941	(2R)-7-hydroxy-2-(4-hydroxyphenyl)chroman-4-one	Sterol O-acyltransferase 2	O75908	SOAT2	0.03
Polygonatum sibiricum(PGS)	MOL004941	(2R)-7-hydroxy-2-(4-hydroxyphenyl)chroman-4-one	ATP synthase subunit beta, mitochondrial	P06576	ATP5F1B	0.03
Polygonatum sibiricum(PGS)	MOL004941	(2R)-7-hydroxy-2-(4-hydroxyphenyl)chroman-4-one	Phospholipase A2, membrane associated	P14555	PLA2G2A	0.03
Polygonatum sibiricum(PGS)	MOL004941	(2R)-7-hydroxy-2-(4-hydroxyphenyl)chroman-4-one	ATP synthase subunit alpha, mitochondrial	P25705	ATP5F1A	0.03
Polygonatum sibiricum(PGS)	MOL004941	(2R)-7-hydroxy-2-(4-hydroxyphenyl)chroman-4-one	Aldo-keto reductase family 1 member C3	P42330	AKR1C3	0.03
Polygonatum sibiricum(PGS)	MOL004941	(2R)-7-hydroxy-2-(4-hydroxyphenyl)chroman-4-one	Phosphatidylinositol-4,5-bisphosphate 3-kinase catalytic subunit gamma isoform	P48736	PIK3CG	0.03
Polygonatum sibiricum(PGS)	MOL004941	(2R)-7-hydroxy-2-(4-hydroxyphenyl)chroman-4-one	Lactoylglutathione lyase	Q04760	GLO1	0.03
Polygonatum sibiricum(PGS)	MOL004941	(2R)-7-hydroxy-2-(4-hydroxyphenyl)chroman-4-one	Prostaglandin reductase 2	Q8N8N7	PTGR2	0.03
Polygonatum sibiricum(PGS)	MOL004941	(2R)-7-hydroxy-2-(4-hydroxyphenyl)chroman-4-one	Niemann-Pick C1-like protein 1	Q9UHC9	NPC1L1	0.03

Polygonatum sibiricum(PGS)	MOL004941	(2R)-7-hydroxy-2-(4-hydroxyphenyl)chroman-4-one	Gamma-aminobutyric-acid receptor subunit beta-3	P28472	GABRB3	0.031
Polygonatum sibiricum(PGS)	MOL004941	(2R)-7-hydroxy-2-(4-hydroxyphenyl)chroman-4-one	Tubulin alpha-1 chain	P68366	TUBA4A	0.031
Polygonatum sibiricum(PGS)	MOL004941	(2R)-7-hydroxy-2-(4-hydroxyphenyl)chroman-4-one	Keratin, type II cytoskeletal 7	P08729	KRT7	0.032
Polygonatum sibiricum(PGS)	MOL004941	(2R)-7-hydroxy-2-(4-hydroxyphenyl)chroman-4-one	5-hydroxytryptamine 1A receptor	P08908	HTR1A	0.032
Polygonatum sibiricum(PGS)	MOL004941	(2R)-7-hydroxy-2-(4-hydroxyphenyl)chroman-4-one	5-hydroxytryptamine 2A receptor	P28223	HTR2A	0.032
Polygonatum sibiricum(PGS)	MOL004941	(2R)-7-hydroxy-2-(4-hydroxyphenyl)chroman-4-one	Retinoic acid receptor RXR-beta	P28702	RXRB	0.032
Polygonatum sibiricum(PGS)	MOL004941	(2R)-7-hydroxy-2-(4-hydroxyphenyl)chroman-4-one	Peptidyl-prolyl cis-trans isomerase, mitochondrial	P30405	PPIF	0.032
Polygonatum sibiricum(PGS)	MOL004941	(2R)-7-hydroxy-2-(4-hydroxyphenyl)chroman-4-one	5-hydroxytryptamine 2B receptor	P41595	HTR2B	0.032
Polygonatum sibiricum(PGS)	MOL004941	(2R)-7-hydroxy-2-(4-hydroxyphenyl)chroman-4-one	Oxysterols receptor LXR-beta	P55055	NR1H2	0.032
Polygonatum sibiricum(PGS)	MOL004941	(2R)-7-hydroxy-2-(4-hydroxyphenyl)chroman-4-one	Oxysterols receptor LXR-alpha	Q13133	NR1H3	0.032
Polygonatum sibiricum(PGS)	MOL004941	(2R)-7-hydroxy-2-(4-hydroxyphenyl)chroman-4-one	Ig gamma-2 chain C region	P01859	IGHG2	0.034
Polygonatum sibiricum(PGS)	MOL004941	(2R)-7-hydroxy-2-(4-hydroxyphenyl)chroman-4-one	Peroxisome proliferator-activated receptor delta	Q03181	PPARD	0.034
Polygonatum sibiricum(PGS)	MOL004941	(2R)-7-hydroxy-2-(4-hydroxyphenyl)chroman-4-one	Protein S100-A12	P80511	S100A12	0.035
Polygonatum sibiricum(PGS)	MOL004941	(2R)-7-hydroxy-2-(4-hydroxyphenyl)chroman-4-one	Protein S100-A13	Q99584	S100A13	0.035
Polygonatum sibiricum(PGS)	MOL004941	(2R)-7-hydroxy-2-(4-hydroxyphenyl)chroman-4-one	Gamma-aminobutyric-acid receptor subunit beta-2	P47870	GABRB2	0.037
Polygonatum sibiricum(PGS)	MOL004941	(2R)-7-hydroxy-2-(4-hydroxyphenyl)chroman-4-one	Gamma-aminobutyric acid receptor subunit theta	Q9UN88	GABRQ	0.037
Polygonatum sibiricum(PGS)	MOL004941	(2R)-7-hydroxy-2-(4-hydroxyphenyl)chroman-4-one	Carbonic anhydrase 2	P00918	CA2	0.038
Polygonatum sibiricum(PGS)	MOL004941	(2R)-7-hydroxy-2-(4-hydroxyphenyl)chroman-4-one	Bile salt sulfotransferase	Q06520	SULT2A1	0.039
Polygonatum sibiricum(PGS)	MOL004941	(2R)-7-hydroxy-2-(4-hydroxyphenyl)chroman-4-one	cAMP-dependent protein kinase inhibitor alpha	P61925	PKIA	0.04
Polygonatum sibiricum(PGS)	MOL004941	(2R)-7-hydroxy-2-(4-hydroxyphenyl)chroman-4-one	Protein tyrosine kinase 2 beta	Q14289	PTK2B	0.04
Polygonatum sibiricum(PGS)	MOL004941	(2R)-7-hydroxy-2-(4-hydroxyphenyl)chroman-4-one	cAMP-specific 3',5'-cyclic phosphodiesterase 4A	P27815	PDE4A	0.042
Polygonatum sibiricum(PGS)	MOL004941	(2R)-7-hydroxy-2-(4-hydroxyphenyl)chroman-4-one	Delta-type opioid receptor	P41143	OPRD1	0.042
Polygonatum sibiricum(PGS)	MOL004941	(2R)-7-hydroxy-2-(4-hydroxyphenyl)chroman-4-one	cAMP-specific 3',5'-cyclic phosphodiesterase 4B	Q07343	PDE4B	0.042
Polygonatum sibiricum(PGS)	MOL004941	(2R)-7-hydroxy-2-(4-hydroxyphenyl)chroman-4-one	D(1A) dopamine receptor	P21728	DRD1	0.047
Polygonatum sibiricum(PGS)	MOL004941	(2R)-7-hydroxy-2-(4-hydroxyphenyl)chroman-4-one	D(1B) dopamine receptor	P21918	DRD5	0.047
Polygonatum sibiricum(PGS)	MOL004941	(2R)-7-hydroxy-2-(4-hydroxyphenyl)chroman-4-one	Gamma-aminobutyric acid receptor subunit gamma-2	P18507	GABRG2	0.049
Polygonatum sibiricum(PGS)	MOL004941	(2R)-7-hydroxy-2-(4-hydroxyphenyl)chroman-4-one	Gamma-aminobutyric acid receptor subunit gamma-1	Q8N1C3	GABRG1	0.049
Polygonatum sibiricum(PGS)	MOL004941	(2R)-7-hydroxy-2-(4-hydroxyphenyl)chroman-4-one	5-hydroxytryptamine 2C receptor	P28335	HTR2C	0.055
Polygonatum sibiricum(PGS)	MOL004941	(2R)-7-hydroxy-2-(4-hydroxyphenyl)chroman-4-one	Sodium channel protein type 5 subunit alpha	Q14524	SCN5A	0.055
Polygonatum sibiricum(PGS)	MOL004941	(2R)-7-hydroxy-2-(4-hydroxyphenyl)chroman-4-one	Gamma-aminobutyric acid receptor subunit rho-3	A8MPY1	GABRR3	0.056
Polygonatum sibiricum(PGS)	MOL004941	(2R)-7-hydroxy-2-(4-hydroxyphenyl)chroman-4-one	Gamma-aminobutyric acid receptor subunit pi	O00591	GABRP	0.056
Polygonatum sibiricum(PGS)	MOL004941	(2R)-7-hydroxy-2-(4-hydroxyphenyl)chroman-4-one	Gamma-aminobutyric acid receptor subunit delta	O14764	GABRD	0.056
Polygonatum sibiricum(PGS)	MOL004941	(2R)-7-hydroxy-2-(4-hydroxyphenyl)chroman-4-one	Gamma-aminobutyric-acid receptor subunit beta-1	P18505	GABRB1	0.056
Polygonatum sibiricum(PGS)	MOL004941	(2R)-7-hydroxy-2-(4-hydroxyphenyl)chroman-4-one	Gamma-aminobutyric-acid receptor subunit rho-1	P24046	GABRR1	0.056
Polygonatum sibiricum(PGS)	MOL004941	(2R)-7-hydroxy-2-(4-hydroxyphenyl)chroman-4-one	Gamma-aminobutyric acid receptor subunit rho-2	P28476	GABRR2	0.056
Polygonatum sibiricum(PGS)	MOL004941	(2R)-7-hydroxy-2-(4-hydroxyphenyl)chroman-4-one	Gamma-aminobutyric acid receptor subunit epsilon	P78334	GABRE	0.056
Polygonatum sibiricum(PGS)	MOL004941	(2R)-7-hydroxy-2-(4-hydroxyphenyl)chroman-4-one	Gamma-aminobutyric acid receptor subunit gamma-3	Q99928	GABRG3	0.056
Polygonatum sibiricum(PGS)	MOL004941	(2R)-7-hydroxy-2-(4-hydroxyphenyl)chroman-4-one	Ornithine aminotransferase, mitochondrial	P04181	OAT	0.057
Polygonatum sibiricum(PGS)	MOL004941	(2R)-7-hydroxy-2-(4-hydroxyphenyl)chroman-4-one	Tubulin beta-1 chain	Q9H4B7	TUBB1	0.059
Polygonatum sibiricum(PGS)	MOL004941	(2R)-7-hydroxy-2-(4-hydroxyphenyl)chroman-4-one	Muscarinic acetylcholine receptor M4	P08173	CHRM4	0.06
Polygonatum sibiricum(PGS)	MOL004941	(2R)-7-hydroxy-2-(4-hydroxyphenyl)chroman-4-one	Low molecular weight phosphotyrosine protein phosphatase	P24666	ACP1	0.06
Polygonatum sibiricum(PGS)	MOL004941	(2R)-7-hydroxy-2-(4-hydroxyphenyl)chroman-4-one	Nuclear receptor coactivator 5	Q9HCD5	NCOA5	0.06
Polygonatum sibiricum(PGS)	MOL004941	(2R)-7-hydroxy-2-(4-hydroxyphenyl)chroman-4-one	DNA polymerase kappa	Q9UBT6	POLK	0.06
Polygonatum sibiricum(PGS)	MOL004941	(2R)-7-hydroxy-2-(4-hydroxyphenyl)chroman-4-one	Beta-2 adrenergic receptor	P07550	ADRB2	0.062
Polygonatum sibiricum(PGS)	MOL004941	(2R)-7-hydroxy-2-(4-hydroxyphenyl)chroman-4-one	Beta-1 adrenergic receptor	P08588	ADRB1	0.062
Polygonatum sibiricum(PGS)	MOL004941	(2R)-7-hydroxy-2-(4-hydroxyphenyl)chroman-4-one	Androgen receptor	P10275	AR	0.062
Polygonatum sibiricum(PGS)	MOL004941	(2R)-7-hydroxy-2-(4-hydroxyphenyl)chroman-4-one	RAC-alpha serine/threonine-protein kinase	P31749	AKT1	0.062

Polygonatum sibiricum(PGS)	MOL004941	(2R)-7-hydroxy-2-(4-hydroxyphenyl)chroman-4-one	Neuronal acetylcholine receptor subunit alpha-7	P36544	CHRNA7	0.062
Polygonatum sibiricum(PGS)	MOL004941	(2R)-7-hydroxy-2-(4-hydroxyphenyl)chroman-4-one	Neuronal acetylcholine receptor subunit alpha-4	P43681	CHRNA4	0.062
Polygonatum sibiricum(PGS)	MOL004941	(2R)-7-hydroxy-2-(4-hydroxyphenyl)chroman-4-one	Glutamate receptor, ionotropic kainate 2	Q13002	GRIK2	0.062
Polygonatum sibiricum(PGS)	MOL004941	(2R)-7-hydroxy-2-(4-hydroxyphenyl)chroman-4-one	Inosine-5'-monophosphate dehydrogenase 1	P20839	IMPDH1	0.063
Polygonatum sibiricum(PGS)	MOL004941	(2R)-7-hydroxy-2-(4-hydroxyphenyl)chroman-4-one	Casein kinase II subunit alpha	P68400	CSNK2A1	0.063
Polygonatum sibiricum(PGS)	MOL004941	(2R)-7-hydroxy-2-(4-hydroxyphenyl)chroman-4-one	Muscarinic acetylcholine receptor M2	P08172	CHRM2	0.064
Polygonatum sibiricum(PGS)	MOL004941	(2R)-7-hydroxy-2-(4-hydroxyphenyl)chroman-4-one	Estradiol 17-beta-dehydrogenase 1	P14061	HSD17B1	0.064
Polygonatum sibiricum(PGS)	MOL004941	(2R)-7-hydroxy-2-(4-hydroxyphenyl)chroman-4-one	Inhibitor of nuclear factor kappa-B kinase subunit alpha	O15111	CHUK	0.066
Polygonatum sibiricum(PGS)	MOL004941	(2R)-7-hydroxy-2-(4-hydroxyphenyl)chroman-4-one	Arachidonate 5-lipoxygenase	P09917	ALOX5	0.066
Polygonatum sibiricum(PGS)	MOL004941	(2R)-7-hydroxy-2-(4-hydroxyphenyl)chroman-4-one	Peroxisome proliferator-activated receptor gamma	P37231	PPARG	0.066
Polygonatum sibiricum(PGS)	MOL004941	(2R)-7-hydroxy-2-(4-hydroxyphenyl)chroman-4-one	Alpha-1D adrenergic receptor	P25100	ADRA1D	0.067
Polygonatum sibiricum(PGS)	MOL004941	(2R)-7-hydroxy-2-(4-hydroxyphenyl)chroman-4-one	Sterol O-acyltransferase 1	P35610	SOAT1	0.067
Polygonatum sibiricum(PGS)	MOL004941	(2R)-7-hydroxy-2-(4-hydroxyphenyl)chroman-4-one	Kappa-type opioid receptor	P41145	OPRK1	0.067
Polygonatum sibiricum(PGS)	MOL004941	(2R)-7-hydroxy-2-(4-hydroxyphenyl)chroman-4-one	Group IIE secretory phospholipase A2	Q9NZK7	PLA2G2E	0.067
Polygonatum sibiricum(PGS)	MOL004941	(2R)-7-hydroxy-2-(4-hydroxyphenyl)chroman-4-one	cGMP-specific 3',5'-cyclic phosphodiesterase	O76074	PDE5A	0.068
Polygonatum sibiricum(PGS)	MOL004941	(2R)-7-hydroxy-2-(4-hydroxyphenyl)chroman-4-one	Thymidylate synthase	P04818	TYMS	0.068
Polygonatum sibiricum(PGS)	MOL004941	(2R)-7-hydroxy-2-(4-hydroxyphenyl)chroman-4-one	D(2) dopamine receptor	P14416	DRD2	0.068
Polygonatum sibiricum(PGS)	MOL004941	(2R)-7-hydroxy-2-(4-hydroxyphenyl)chroman-4-one	cAMP-specific 3',5'-cyclic phosphodiesterase 4D	Q08499	PDE4D	0.068
Polygonatum sibiricum(PGS)	MOL004941	(2R)-7-hydroxy-2-(4-hydroxyphenyl)chroman-4-one	Nuclear receptor coactivator 2	Q15596	NCOA2	0.068
Polygonatum sibiricum(PGS)	MOL004941	(2R)-7-hydroxy-2-(4-hydroxyphenyl)chroman-4-one	Tubulin alpha-3 chain	Q71U36	TUBA1A	0.069
Polygonatum sibiricum(PGS)	MOL004941	(2R)-7-hydroxy-2-(4-hydroxyphenyl)chroman-4-one	Phospholipase A2	P04054	PLA2G1B	0.07
Polygonatum sibiricum(PGS)	MOL004941	(2R)-7-hydroxy-2-(4-hydroxyphenyl)chroman-4-one	Cytochrome P450 19A1	P11511	CYP19A1	0.07
Polygonatum sibiricum(PGS)	MOL004941	(2R)-7-hydroxy-2-(4-hydroxyphenyl)chroman-4-one	Thyroid hormone receptor alpha	P10827	THRA	0.073
Polygonatum sibiricum(PGS)	MOL004941	(2R)-7-hydroxy-2-(4-hydroxyphenyl)chroman-4-one	Peroxisome proliferator-activated receptor alpha	Q07869	PPARA	0.073
Polygonatum sibiricum(PGS)	MOL004941	(2R)-7-hydroxy-2-(4-hydroxyphenyl)chroman-4-one	Thyroid hormone receptor, alpha isoform 1 variant	Q59FW3	SIGMAR1	0.073
Polygonatum sibiricum(PGS)	MOL004941	(2R)-7-hydroxy-2-(4-hydroxyphenyl)chroman-4-one	Ig kappa chain C region	P01834	IGKC	0.074
Polygonatum sibiricum(PGS)	MOL004941	(2R)-7-hydroxy-2-(4-hydroxyphenyl)chroman-4-one	Ig gamma-1 chain C region	P01857	IGHG1	0.074
Polygonatum sibiricum(PGS)	MOL004941	(2R)-7-hydroxy-2-(4-hydroxyphenyl)chroman-4-one	Ig kappa chain V-II region RPMI 6410	P06310	IGKV2-30	0.074
Polygonatum sibiricum(PGS)	MOL004941	(2R)-7-hydroxy-2-(4-hydroxyphenyl)chroman-4-one	Thyroid hormone receptor beta-1	P10828	THRB	0.075
Polygonatum sibiricum(PGS)	MOL004941	(2R)-7-hydroxy-2-(4-hydroxyphenyl)chroman-4-one	Tyrosine-protein phosphatase non-receptor type 1	P18031	PTPN1	0.075
Polygonatum sibiricum(PGS)	MOL004941	(2R)-7-hydroxy-2-(4-hydroxyphenyl)chroman-4-one	Cytochrome P450 11B1, mitochondrial	P15538	CYP11B1	0.076
Polygonatum sibiricum(PGS)	MOL004941	(2R)-7-hydroxy-2-(4-hydroxyphenyl)chroman-4-one	Hemoglobin subunit alpha	P69905	HBA1	0.081
Polygonatum sibiricum(PGS)	MOL004941	(2R)-7-hydroxy-2-(4-hydroxyphenyl)chroman-4-one	Alpha-2C adrenergic receptor	P18825	ADRA2C	0.085
Polygonatum sibiricum(PGS)	MOL004941	(2R)-7-hydroxy-2-(4-hydroxyphenyl)chroman-4-one	Alpha-1B adrenergic receptor	P35368	ADRA1B	0.085
Polygonatum sibiricum(PGS)	MOL004941	(2R)-7-hydroxy-2-(4-hydroxyphenyl)chroman-4-one	Rho-associated protein kinase 1	Q13464	ROCK1	0.087
Polygonatum sibiricum(PGS)	MOL004941	(2R)-7-hydroxy-2-(4-hydroxyphenyl)chroman-4-one	Mu-type opioid receptor	P35372	OPRM1	0.092
Polygonatum sibiricum(PGS)	MOL004941	(2R)-7-hydroxy-2-(4-hydroxyphenyl)chroman-4-one	Gamma-aminobutyric-acid receptor subunit alpha-6	Q16445	GABRA6	0.093
Polygonatum sibiricum(PGS)	MOL004941	(2R)-7-hydroxy-2-(4-hydroxyphenyl)chroman-4-one	Alpha-2A adrenergic receptor	P08913	ADRA2A	0.103
Polygonatum sibiricum(PGS)	MOL004941	(2R)-7-hydroxy-2-(4-hydroxyphenyl)chroman-4-one	Histamine H1 receptor	P35367	HRH1	0.104
Polygonatum sibiricum(PGS)	MOL004941	(2R)-7-hydroxy-2-(4-hydroxyphenyl)chroman-4-one	Gamma-aminobutyric-acid receptor subunit alpha-4	P48169	GABRA4	0.105
Polygonatum sibiricum(PGS)	MOL004941	(2R)-7-hydroxy-2-(4-hydroxyphenyl)chroman-4-one	DNA topoisomerase 2-alpha	P11388	TOP2A	0.107
Polygonatum sibiricum(PGS)	MOL004941	(2R)-7-hydroxy-2-(4-hydroxyphenyl)chroman-4-one	Sodium-dependent dopamine transporter	Q01959	SLC6A3	0.113
Polygonatum sibiricum(PGS)	MOL004941	(2R)-7-hydroxy-2-(4-hydroxyphenyl)chroman-4-one	Glutamate [NMDA] receptor subunit 3A	Q8TCU5	GRIN3A	0.116
Polygonatum sibiricum(PGS)	MOL004941	(2R)-7-hydroxy-2-(4-hydroxyphenyl)chroman-4-one	Alpha-2B adrenergic receptor	P18089	ADRA2B	0.12
Polygonatum sibiricum(PGS)	MOL004941	(2R)-7-hydroxy-2-(4-hydroxyphenyl)chroman-4-one	Gamma-aminobutyric-acid receptor subunit alpha-3	P34903	GABRA3	0.124
Polygonatum sibiricum(PGS)	MOL004941	(2R)-7-hydroxy-2-(4-hydroxyphenyl)chroman-4-one	Alpha-1A adrenergic receptor	P35348	ADRA1A	0.126
Polygonatum sibiricum(PGS)	MOL004941	(2R)-7-hydroxy-2-(4-hydroxyphenyl)chroman-4-one	Gamma-aminobutyric-acid receptor subunit alpha-5	P31644	GABRA5	0.13

Polygonatum sibiricum(PGS)	MOL004941	(2R)-7-hydroxy-2-(4-hydroxyphenyl)chroman-4-one	Gamma-aminobutyric-acid receptor subunit alpha-1	P14867	GABRA1	0.138
Polygonatum sibiricum(PGS)	MOL004941	(2R)-7-hydroxy-2-(4-hydroxyphenyl)chroman-4-one	Gamma-aminobutyric-acid receptor subunit alpha-2	P47869	GABRA2	0.138
Polygonatum sibiricum(PGS)	MOL004941	(2R)-7-hydroxy-2-(4-hydroxyphenyl)chroman-4-one	Mitogen-activated protein kinase 14	Q16539	MAPK14	0.139
Polygonatum sibiricum(PGS)	MOL004941	(2R)-7-hydroxy-2-(4-hydroxyphenyl)chroman-4-one	Heat shock protein HSP 90-beta	P08238	HSP90AB1	0.142
Polygonatum sibiricum(PGS)	MOL004941	(2R)-7-hydroxy-2-(4-hydroxyphenyl)chroman-4-one	Nuclear receptor coactivator 1	Q15788	NCOA1	0.159
Polygonatum sibiricum(PGS)	MOL004941	(2R)-7-hydroxy-2-(4-hydroxyphenyl)chroman-4-one	Sodium-dependent serotonin transporter	P31645	SLC6A4	0.174
Polygonatum sibiricum(PGS)	MOL004941	(2R)-7-hydroxy-2-(4-hydroxyphenyl)chroman-4-one	Muscarinic acetylcholine receptor M3	P20309	CHRM3	0.177
Polygonatum sibiricum(PGS)	MOL004941	(2R)-7-hydroxy-2-(4-hydroxyphenyl)chroman-4-one	Nitric oxide synthase, inducible	P35228	NOS2	0.194
Polygonatum sibiricum(PGS)	MOL004941	(2R)-7-hydroxy-2-(4-hydroxyphenyl)chroman-4-one	Sodium-dependent noradrenaline transporter	P23975	SLC6A2	0.199
Polygonatum sibiricum(PGS)	MOL004941	(2R)-7-hydroxy-2-(4-hydroxyphenyl)chroman-4-one	Muscarinic acetylcholine receptor M1	P11229	CHRM1	0.247
Polygonatum sibiricum(PGS)	MOL004941	(2R)-7-hydroxy-2-(4-hydroxyphenyl)chroman-4-one	Estrogen receptor beta	Q92731	ESR2	0.247
Polygonatum sibiricum(PGS)	MOL004941	(2R)-7-hydroxy-2-(4-hydroxyphenyl)chroman-4-one	cAMP-dependent protein kinase catalytic subunit alpha	P17612	PRKACA	0.272
Polygonatum sibiricum(PGS)	MOL004941	(2R)-7-hydroxy-2-(4-hydroxyphenyl)chroman-4-one	Prostaglandin G/H synthase 1	P23219	PTGS1	0.281
Polygonatum sibiricum(PGS)	MOL004941	(2R)-7-hydroxy-2-(4-hydroxyphenyl)chroman-4-one	Prostaglandin G/H synthase 2	P35354	PTGS2	0.54
Polygonatum sibiricum(PGS)	MOL004941	(2R)-7-hydroxy-2-(4-hydroxyphenyl)chroman-4-one	Estrogen receptor	P03372	ESR1	1
Polygonatum sibiricum(PGS)	MOL006331	4',5-Dihydroxyflavone	Trypsin-2	P07478	PRSS2	0.01
Polygonatum sibiricum(PGS)	MOL006331	4',5-Dihydroxyflavone	Acetylcholinesterase	P22303	ACHE	0.01
Polygonatum sibiricum(PGS)	MOL006331	4',5-Dihydroxyflavone	Sodium channel protein type 10 subunit alpha	Q9Y5Y9	SCN10A	0.01
Polygonatum sibiricum(PGS)	MOL006331	4',5-Dihydroxyflavone	Neuronal acetylcholine receptor subunit alpha-2	Q15822	CHRNA2	0.011
Polygonatum sibiricum(PGS)	MOL006331	4',5-Dihydroxyflavone	Farnesyl pyrophosphate synthetase	P14324	FDPS	0.012
Polygonatum sibiricum(PGS)	MOL006331	4',5-Dihydroxyflavone	Coagulation factor IX	P00740	F9	0.013
Polygonatum sibiricum(PGS)	MOL006331	4',5-Dihydroxyflavone	Coagulation factor X	P00742	F10	0.013
Polygonatum sibiricum(PGS)	MOL006331	4',5-Dihydroxyflavone	Osteocalcin	P02818	BGLAP	0.013
Polygonatum sibiricum(PGS)	MOL006331	4',5-Dihydroxyflavone	Vitamin K-dependent protein C	P04070	PROC	0.013
Polygonatum sibiricum(PGS)	MOL006331	4',5-Dihydroxyflavone	Sodium/potassium-transporting ATPase alpha-1 chain	P05023	ATP1A1	0.013
Polygonatum sibiricum(PGS)	MOL006331	4',5-Dihydroxyflavone	Tyrosine 3-monooxygenase	P07101	TH	0.013
Polygonatum sibiricum(PGS)	MOL006331	4',5-Dihydroxyflavone	Coagulation factor VII	P08709	F7	0.013
Polygonatum sibiricum(PGS)	MOL006331	4',5-Dihydroxyflavone	Furin	P09958	FURIN	0.013
Polygonatum sibiricum(PGS)	MOL006331	4',5-Dihydroxyflavone	NAD(P)H dehydrogenase [quinone] 1	P15559	NQO1	0.013
Polygonatum sibiricum(PGS)	MOL006331	4',5-Dihydroxyflavone	Carbonic anhydrase 4	P22748	CA4	0.013
Polygonatum sibiricum(PGS)	MOL006331	4',5-Dihydroxyflavone	Vitamin K-dependent protein Z	P22891	PROZ	0.013
Polygonatum sibiricum(PGS)	MOL006331	4',5-Dihydroxyflavone	Vitamin K-dependent gamma-carboxylase	P38435	GGCX	0.013
Polygonatum sibiricum(PGS)	MOL006331	4',5-Dihydroxyflavone	Nociceptin receptor	P41146	OPRL1	0.013
Polygonatum sibiricum(PGS)	MOL006331	4',5-Dihydroxyflavone	Gamma-aminobutyric-acid receptor subunit alpha-4	P48169	GABRA4	0.013
Polygonatum sibiricum(PGS)	MOL006331	4',5-Dihydroxyflavone	Tyrosyl-tRNA synthetase, cytoplasmic	P54577	YARS	0.013
Polygonatum sibiricum(PGS)	MOL006331	4',5-Dihydroxyflavone	Sodium/potassium-transporting ATPase gamma chain	P54710	FXYD2	0.013
Polygonatum sibiricum(PGS)	MOL006331	4',5-Dihydroxyflavone	Solute carrier family 12 member 3	P55017	SLC12A3	0.013
Polygonatum sibiricum(PGS)	MOL006331	4',5-Dihydroxyflavone	ATP-sensitive inward rectifier potassium channel 11	Q14654	KCNJ11	0.013
Polygonatum sibiricum(PGS)	MOL006331	4',5-Dihydroxyflavone	Gamma-aminobutyric-acid receptor subunit alpha-6	Q16445	GABRA6	0.013
Polygonatum sibiricum(PGS)	MOL006331	4',5-Dihydroxyflavone	Vitamin K epoxide reductase complex subunit 1-like protein 1	Q8N0U8	VKORC1L1	0.013
Polygonatum sibiricum(PGS)	MOL006331	4',5-Dihydroxyflavone	Vitamin K epoxide reductase complex subunit 1	Q9BQB6	VKORC1	0.013
Polygonatum sibiricum(PGS)	MOL006331	4',5-Dihydroxyflavone	Glycolipid transfer protein	Q9NZD2	GLTP	0.013
Polygonatum sibiricum(PGS)	MOL006331	4',5-Dihydroxyflavone	Gamma-aminobutyric acid receptor subunit theta	Q9UN88	GABRQ	0.013
Polygonatum sibiricum(PGS)	MOL006331	4',5-Dihydroxyflavone	Tyrosyl-tRNA synthetase, mitochondrial	Q9Y2Z4	YARS2	0.013
Polygonatum sibiricum(PGS)	MOL006331	4',5-Dihydroxyflavone	Muscarinic acetylcholine receptor M4	P08173	CHRM4	0.014
Polygonatum sibiricum(PGS)	MOL006331	4',5-Dihydroxyflavone	Muscarinic acetylcholine receptor M5	P08912	CHRM5	0.014

Polygonatum sibiricum(PGS)	MOL006331	4',5-Dihydroxyflavone	Dihydrolipoyllysine-residue acetyltransferase component of pyruvate dehydrogenase complex, mitochondrial	P10515	DLAT	0.014
Polygonatum sibiricum(PGS)	MOL006331	4',5-Dihydroxyflavone	Muscarinic acetylcholine receptor M1	P11229	CHRM1	0.014
Polygonatum sibiricum(PGS)	MOL006331	4',5-Dihydroxyflavone	Cell division protein kinase 4	P11802	CDK4	0.014
Polygonatum sibiricum(PGS)	MOL006331	4',5-Dihydroxyflavone	Glycine cleavage system H protein, mitochondrial	P23434	GCSH	0.014
Polygonatum sibiricum(PGS)	MOL006331	4',5-Dihydroxyflavone	Histamine H1 receptor	P35367	HRH1	0.014
Polygonatum sibiricum(PGS)	MOL006331	4',5-Dihydroxyflavone	Amiloride-sensitive sodium channel subunit alpha	P37088	SCNN1A	0.014
Polygonatum sibiricum(PGS)	MOL006331	4',5-Dihydroxyflavone	Cell division protein kinase 7	P50613	CDK7	0.014
Polygonatum sibiricum(PGS)	MOL006331	4',5-Dihydroxyflavone	Cell division protein kinase 9	P50750	CDK9	0.014
Polygonatum sibiricum(PGS)	MOL006331	4',5-Dihydroxyflavone	Amiloride-sensitive sodium channel subunit beta	P51168	SCNN1B	0.014
Polygonatum sibiricum(PGS)	MOL006331	4',5-Dihydroxyflavone	Amiloride-sensitive sodium channel subunit gamma	P51170	SCNN1G	0.014
Polygonatum sibiricum(PGS)	MOL006331	4',5-Dihydroxyflavone	Serine/threonine-protein kinase 17B	O94768	STK17B	0.015
Polygonatum sibiricum(PGS)	MOL006331	4',5-Dihydroxyflavone	ATP synthase subunit gamma, mitochondrial	P36542	ATP5F1C	0.015
Polygonatum sibiricum(PGS)	MOL006331	4',5-Dihydroxyflavone	UDP-glucuronosyltransferase 3A1	Q6NUS8	UGT3A1	0.015
Polygonatum sibiricum(PGS)	MOL006331	4',5-Dihydroxyflavone	Dehydrogenase/reductase SDR family member 8	Q8NBQ5	HSD17B11	0.015
Polygonatum sibiricum(PGS)	MOL006331	4',5-Dihydroxyflavone	3-phosphoinositide-dependent protein kinase 1	O15530	PDPK1	0.016
Polygonatum sibiricum(PGS)	MOL006331	4',5-Dihydroxyflavone	Tyrosine-protein kinase Lyn	P07948	LYN	0.016
Polygonatum sibiricum(PGS)	MOL006331	4',5-Dihydroxyflavone	Tyrosine-protein kinase CSK	P41240	CSK	0.016
Polygonatum sibiricum(PGS)	MOL006331	4',5-Dihydroxyflavone	Tyrosine-protein kinase ZAP-70	P43403	ZAP70	0.016
Polygonatum sibiricum(PGS)	MOL006331	4',5-Dihydroxyflavone	Tyrosine-protein kinase SYK	P43405	SYK	0.016
Polygonatum sibiricum(PGS)	MOL006331	4',5-Dihydroxyflavone	Protein kinase C theta type	Q04759	PRKCQ	0.016
Polygonatum sibiricum(PGS)	MOL006331	4',5-Dihydroxyflavone	Tyrosine-protein kinase ITK/TSK	Q08881	ITK	0.016
Polygonatum sibiricum(PGS)	MOL006331	4',5-Dihydroxyflavone	Gamma-aminobutyric acid receptor subunit rho-3	A8MPY1	GABRR3	0.017
Polygonatum sibiricum(PGS)	MOL006331	4',5-Dihydroxyflavone	Potassium channel subfamily K member 1	O00180	KCNK1	0.017
Polygonatum sibiricum(PGS)	MOL006331	4',5-Dihydroxyflavone	Gamma-aminobutyric acid receptor subunit pi	O00591	GABRP	0.017
Polygonatum sibiricum(PGS)	MOL006331	4',5-Dihydroxyflavone	Gamma-aminobutyric acid receptor subunit delta	O14764	GABRD	0.017
Polygonatum sibiricum(PGS)	MOL006331	4',5-Dihydroxyflavone	Urokinase-type plasminogen activator	P00749	PLAU	0.017
Polygonatum sibiricum(PGS)	MOL006331	4',5-Dihydroxyflavone	Ig gamma-2 chain C region	P01859	IGHG2	0.017
Polygonatum sibiricum(PGS)	MOL006331	4',5-Dihydroxyflavone	Macrophage migration inhibitory factor	P14174	MIF	0.017
Polygonatum sibiricum(PGS)	MOL006331	4',5-Dihydroxyflavone	Gamma-aminobutyric-acid receptor subunit beta-1	P18505	GABRB1	0.017
Polygonatum sibiricum(PGS)	MOL006331	4',5-Dihydroxyflavone	Gamma-aminobutyric acid receptor subunit gamma-2	P18507	GABRG2	0.017
Polygonatum sibiricum(PGS)	MOL006331	4',5-Dihydroxyflavone	Gamma-aminobutyric-acid receptor subunit rho-1	P24046	GABRR1	0.017
Polygonatum sibiricum(PGS)	MOL006331	4',5-Dihydroxyflavone	Alpha-1D adrenergic receptor	P25100	ADRA1D	0.017
Polygonatum sibiricum(PGS)	MOL006331	4',5-Dihydroxyflavone	5-hydroxytryptamine 2A receptor	P28223	HTR2A	0.017
Polygonatum sibiricum(PGS)	MOL006331	4',5-Dihydroxyflavone	Gamma-aminobutyric acid receptor subunit rho-2	P28476	GABRR2	0.017
Polygonatum sibiricum(PGS)	MOL006331	4',5-Dihydroxyflavone	Gamma-aminobutyric-acid receptor subunit alpha-5	P31644	GABRA5	0.017
Polygonatum sibiricum(PGS)	MOL006331	4',5-Dihydroxyflavone	Gamma-aminobutyric-acid receptor subunit alpha-3	P34903	GABRA3	0.017
Polygonatum sibiricum(PGS)	MOL006331	4',5-Dihydroxyflavone	Gamma-aminobutyric-acid receptor subunit beta-2	P47870	GABRB2	0.017
Polygonatum sibiricum(PGS)	MOL006331	4',5-Dihydroxyflavone	Gamma-aminobutyric acid receptor subunit epsilon	P78334	GABRE	0.017
Polygonatum sibiricum(PGS)	MOL006331	4',5-Dihydroxyflavone	Gamma-aminobutyric acid receptor subunit gamma-1	Q8N1C3	GABRG1	0.017
Polygonatum sibiricum(PGS)	MOL006331	4',5-Dihydroxyflavone	Gamma-aminobutyric acid receptor subunit gamma-3	Q99928	GABRG3	0.017
Polygonatum sibiricum(PGS)	MOL006331	4',5-Dihydroxyflavone	cAMP-specific 3',5'-cyclic phosphodiesterase 4C	Q08493	PDE4C	0.018
Polygonatum sibiricum(PGS)	MOL006331	4',5-Dihydroxyflavone	cGMP-inhibited 3',5'-cyclic phosphodiesterase A	Q14432	PDE3A	0.018
Polygonatum sibiricum(PGS)	MOL006331	4',5-Dihydroxyflavone	Prostaglandin reductase 1	Q14914	PTGR1	0.018
Polygonatum sibiricum(PGS)	MOL006331	4',5-Dihydroxyflavone	cAMP and cAMP-inhibited cGMP 3',5'-cyclic phosphodiesterase 10A	Q9Y233	PDE10A	0.018
Polygonatum sibiricum(PGS)	MOL006331	4',5-Dihydroxyflavone	T-cell receptor alpha chain C region	P01848	TRAC	0.019
Polygonatum sibiricum(PGS)	MOL006331	4',5-Dihydroxyflavone	T-cell receptor beta chain C region	P01850	TRBC1	0.019

Polygonatum sibiricum(PGS)	MOL006331	4',5-Dihydroxyflavone	Sodium-dependent serotonin transporter	P31645	SLC6A4	0.019
Polygonatum sibiricum(PGS)	MOL006331	4',5-Dihydroxyflavone	Beta-2-microglobulin	P61769	B2M	0.019
Polygonatum sibiricum(PGS)	MOL006331	4',5-Dihydroxyflavone	Casein kinase II subunit alpha	P68400	CSNK2A1	0.019
Polygonatum sibiricum(PGS)	MOL006331	4',5-Dihydroxyflavone	DNA topoisomerase 1	P11387	TOP1	0.02
Polygonatum sibiricum(PGS)	MOL006331	4',5-Dihydroxyflavone	Pyruvate carboxylase, mitochondrial	P11498	PC	0.02
Polygonatum sibiricum(PGS)	MOL006331	4',5-Dihydroxyflavone	Delta-aminolevulinic acid dehydratase	P13716	ALAD	0.02
Polygonatum sibiricum(PGS)	MOL006331	4',5-Dihydroxyflavone	Alcohol dehydrogenase [NADP+]	P14550	AKR1A1	0.02
Polygonatum sibiricum(PGS)	MOL006331	4',5-Dihydroxyflavone	Aldose reductase	P15121	AKR1B1	0.02
Polygonatum sibiricum(PGS)	MOL006331	4',5-Dihydroxyflavone	Peptidyl-prolyl cis-trans isomerase, mitochondrial	P30405	PPIF	0.02
Polygonatum sibiricum(PGS)	MOL006331	4',5-Dihydroxyflavone	DNA topoisomerase I, mitochondrial	Q969P6	TOP1MT	0.02
Polygonatum sibiricum(PGS)	MOL006331	4',5-Dihydroxyflavone	85 kDa calcium-independent phospholipase A2	O60733	PLA2G6	0.021
Polygonatum sibiricum(PGS)	MOL006331	4',5-Dihydroxyflavone	Carboxypeptidase A1	P15085	CPA1	0.021
Polygonatum sibiricum(PGS)	MOL006331	4',5-Dihydroxyflavone	Cytosolic phospholipase A2	P47712	PLA2G4A	0.021
Polygonatum sibiricum(PGS)	MOL006331	4',5-Dihydroxyflavone	DNA polymerase subunit alpha B	Q14181	POLA2	0.021
Polygonatum sibiricum(PGS)	MOL006331	4',5-Dihydroxyflavone	Inhibitor of nuclear factor kappa-B kinase subunit beta	O14920	IKBKB	0.023
Polygonatum sibiricum(PGS)	MOL006331	4',5-Dihydroxyflavone	Mitogen-activated protein kinase 8	P45983	MAPK8	0.023
Polygonatum sibiricum(PGS)	MOL006331	4',5-Dihydroxyflavone	Mitogen-activated protein kinase 10	P53779	MAPK10	0.023
Polygonatum sibiricum(PGS)	MOL006331	4',5-Dihydroxyflavone	Group IIE secretory phospholipase A2	Q9NZK7	PLA2G2E	0.023
Polygonatum sibiricum(PGS)	MOL006331	4',5-Dihydroxyflavone	Cystine/glutamate transporter	Q9UPY5	SLC7A11	0.023
Polygonatum sibiricum(PGS)	MOL006331	4',5-Dihydroxyflavone	Prostacyclin receptor	P43119	PTGIR	0.024
Polygonatum sibiricum(PGS)	MOL006331	4',5-Dihydroxyflavone	Peroxisome proliferator-activated receptor delta	Q03181	PPARD	0.024
Polygonatum sibiricum(PGS)	MOL006331	4',5-Dihydroxyflavone	Glycogen phosphorylase, muscle form	P11217	PYGM	0.025
Polygonatum sibiricum(PGS)	MOL006331	4',5-Dihydroxyflavone	5-hydroxytryptamine 2B receptor	P41595	HTR2B	0.025
Polygonatum sibiricum(PGS)	MOL006331	4',5-Dihydroxyflavone	Gamma-aminobutyric-acid receptor subunit alpha-2	P47869	GABRA2	0.025
Polygonatum sibiricum(PGS)	MOL006331	4',5-Dihydroxyflavone	Alpha-1B adrenergic receptor	P35368	ADRA1B	0.026
Polygonatum sibiricum(PGS)	MOL006331	4',5-Dihydroxyflavone	Tripartite motif-containing protein 13	O60858	TRIM13	0.027
Polygonatum sibiricum(PGS)	MOL006331	4',5-Dihydroxyflavone	DNA-(apurinic or apyrimidinic site) lyase	P27695	APEX1	0.027
Polygonatum sibiricum(PGS)	MOL006331	4',5-Dihydroxyflavone	Calcium-activated potassium channel subunit alpha 1	Q12791	KCNMA1	0.027
Polygonatum sibiricum(PGS)	MOL006331	4',5-Dihydroxyflavone	Sterol O-acyltransferase 2	O75908	SOAT2	0.028
Polygonatum sibiricum(PGS)	MOL006331	4',5-Dihydroxyflavone	Sterol O-acyltransferase 1	P35610	SOAT1	0.028
Polygonatum sibiricum(PGS)	MOL006331	4',5-Dihydroxyflavone	Keratin, type II cytoskeletal 7	P08729	KRT7	0.029
Polygonatum sibiricum(PGS)	MOL006331	4',5-Dihydroxyflavone	Platelet glycoprotein IX	P14770	GP9	0.029
Polygonatum sibiricum(PGS)	MOL006331	4',5-Dihydroxyflavone	Retinal rod rhodopsin-sensitive cGMP 3',5'-cyclic phosphodiesterase subunit gamma	P18545	PDE6G	0.029
Polygonatum sibiricum(PGS)	MOL006331	4',5-Dihydroxyflavone	Nitric-oxide synthase, brain	P29475	NOS1	0.029
Polygonatum sibiricum(PGS)	MOL006331	4',5-Dihydroxyflavone	RAC-beta serine/threonine-protein kinase	P31751	AKT2	0.029
Polygonatum sibiricum(PGS)	MOL006331	4',5-Dihydroxyflavone	Tubulin beta-2C chain	P68371	TUBB4B	0.029
Polygonatum sibiricum(PGS)	MOL006331	4',5-Dihydroxyflavone	Rho-associated protein kinase 1	Q13464	ROCK1	0.029
Polygonatum sibiricum(PGS)	MOL006331	4',5-Dihydroxyflavone	Epidermal growth factor receptor	P00533	EGFR	0.03
Polygonatum sibiricum(PGS)	MOL006331	4',5-Dihydroxyflavone	Muscarinic acetylcholine receptor M2	P08172	CHRM2	0.03
Polygonatum sibiricum(PGS)	MOL006331	4',5-Dihydroxyflavone	Cell division protein kinase 6	Q00534	CDK6	0.03
Polygonatum sibiricum(PGS)	MOL006331	4',5-Dihydroxyflavone	ATP synthase subunit beta, mitochondrial	P06576	ATP5F1B	0.031
Polygonatum sibiricum(PGS)	MOL006331	4',5-Dihydroxyflavone	Tyrosine-protein kinase HCK	P08631	HCK	0.031
Polygonatum sibiricum(PGS)	MOL006331	4',5-Dihydroxyflavone	Gamma-aminobutyric-acid receptor subunit alpha-1	P14867	GABRA1	0.031
Polygonatum sibiricum(PGS)	MOL006331	4',5-Dihydroxyflavone	ATP synthase subunit alpha, mitochondrial	P25705	ATP5F1A	0.031
Polygonatum sibiricum(PGS)	MOL006331	4',5-Dihydroxyflavone	Phosphatidylinositol-4,5-bisphosphate 3-kinase catalytic subunit gamma isoform	P48736	PIK3CG	0.031
Polygonatum sibiricum(PGS)	MOL006331	4',5-Dihydroxyflavone	Proto-oncogene serine/threonine-protein kinase Pim-1	P11309	PIM1	0.032
Polygonatum sibiricum(PGS)	MOL006331	4',5-Dihydroxyflavone	Proto-oncogene tyrosine-protein kinase LCK	P06239	LCK	0.033

Polygonatum sibiricum(PGS)	MOL006331	4',5-Dihydroxyflavone	Glycogen synthase kinase-3 beta	P49841	GSK3B	0.033
Polygonatum sibiricum(PGS)	MOL006331	4',5-Dihydroxyflavone	Ribosyldihydronicotinamide dehydrogenase [quinone]	P16083	NQO2	0.034
Polygonatum sibiricum(PGS)	MOL006331	4',5-Dihydroxyflavone	D(4) dopamine receptor	P21917	DRD4	0.034
Polygonatum sibiricum(PGS)	MOL006331	4',5-Dihydroxyflavone	3 beta-hydroxysteroid dehydrogenase/Delta 5-->4-isomerase type II	P26439	HSD3B2	0.034
Polygonatum sibiricum(PGS)	MOL006331	4',5-Dihydroxyflavone	5-hydroxytryptamine 1B receptor	P28222	HTR1B	0.034
Polygonatum sibiricum(PGS)	MOL006331	4',5-Dihydroxyflavone	5-hydroxytryptamine 2C receptor	P28335	HTR2C	0.034
Polygonatum sibiricum(PGS)	MOL006331	4',5-Dihydroxyflavone	Bile salt sulfotransferase	Q06520	SULT2A1	0.035
Polygonatum sibiricum(PGS)	MOL006331	4',5-Dihydroxyflavone	Ig kappa chain C region	P01834	IGKC	0.037
Polygonatum sibiricum(PGS)	MOL006331	4',5-Dihydroxyflavone	Ig gamma-1 chain C region	P01857	IGHG1	0.037
Polygonatum sibiricum(PGS)	MOL006331	4',5-Dihydroxyflavone	Mineralocorticoid receptor	P08235	NR3C2	0.037
Polygonatum sibiricum(PGS)	MOL006331	4',5-Dihydroxyflavone	Protein tyrosine kinase 2 beta	Q14289	PTK2B	0.037
Polygonatum sibiricum(PGS)	MOL006331	4',5-Dihydroxyflavone	3-oxo-5-alpha-steroid 4-dehydrogenase 1	P18405	SRD5A1	0.038
Polygonatum sibiricum(PGS)	MOL006331	4',5-Dihydroxyflavone	Sodium-dependent dopamine transporter	Q01959	SLC6A3	0.038
Polygonatum sibiricum(PGS)	MOL006331	4',5-Dihydroxyflavone	cAMP-specific 3',5'-cyclic phosphodiesterase 4B	Q07343	PDE4B	0.038
Polygonatum sibiricum(PGS)	MOL006331	4',5-Dihydroxyflavone	Estrogen-related receptor gamma	P62508	ESRRG	0.039
Polygonatum sibiricum(PGS)	MOL006331	4',5-Dihydroxyflavone	Sodium channel protein type 5 subunit alpha	Q14524	SCN5A	0.039
Polygonatum sibiricum(PGS)	MOL006331	4',5-Dihydroxyflavone	Phospholipase A2	P04054	PLA2G1B	0.04
Polygonatum sibiricum(PGS)	MOL006331	4',5-Dihydroxyflavone	Sodium-dependent noradrenaline transporter	P23975	SLC6A2	0.04
Polygonatum sibiricum(PGS)	MOL006331	4',5-Dihydroxyflavone	Mediator of RNA polymerase II transcription subunit 1	Q15648	MED1	0.04
Polygonatum sibiricum(PGS)	MOL006331	4',5-Dihydroxyflavone	Hepatocyte growth factor receptor	P08581	MET	0.041
Polygonatum sibiricum(PGS)	MOL006331	4',5-Dihydroxyflavone	Muscarinic acetylcholine receptor M3	P20309	CHRM3	0.041
Polygonatum sibiricum(PGS)	MOL006331	4',5-Dihydroxyflavone	Cannabinoid receptor 2	P34972	CNR2	0.041
Polygonatum sibiricum(PGS)	MOL006331	4',5-Dihydroxyflavone	Serine/threonine-protein kinase 12	Q96GD4	AURKB	0.041
Polygonatum sibiricum(PGS)	MOL006331	4',5-Dihydroxyflavone	Lactotransferrin	P02788	LTF	0.042
Polygonatum sibiricum(PGS)	MOL006331	4',5-Dihydroxyflavone	Leukotriene A-4 hydrolase	P09960	LTA4H	0.042
Polygonatum sibiricum(PGS)	MOL006331	4',5-Dihydroxyflavone	Retinoic acid receptor RXR-alpha	P19793	RXRA	0.042
Polygonatum sibiricum(PGS)	MOL006331	4',5-Dihydroxyflavone	Delta-type opioid receptor	P41143	OPRD1	0.042
Polygonatum sibiricum(PGS)	MOL006331	4',5-Dihydroxyflavone	Kappa-type opioid receptor	P41145	OPRK1	0.042
Polygonatum sibiricum(PGS)	MOL006331	4',5-Dihydroxyflavone	NADPH oxidase organizer 1	Q8NFA2	NOXO1	0.042
Polygonatum sibiricum(PGS)	MOL006331	4',5-Dihydroxyflavone	Thyroid hormone receptor beta-1	P10828	THRB	0.043
Polygonatum sibiricum(PGS)	MOL006331	4',5-Dihydroxyflavone	5-hydroxytryptamine 1D receptor	P28221	HTR1D	0.043
Polygonatum sibiricum(PGS)	MOL006331	4',5-Dihydroxyflavone	cAMP-specific 3',5'-cyclic phosphodiesterase 4D	Q08499	PDE4D	0.043
Polygonatum sibiricum(PGS)	MOL006331	4',5-Dihydroxyflavone	Membrane copper amine oxidase	Q16853	AOC3	0.043
Polygonatum sibiricum(PGS)	MOL006331	4',5-Dihydroxyflavone	Tubulin beta-1 chain	Q9H4B7	TUBB1	0.043
Polygonatum sibiricum(PGS)	MOL006331	4',5-Dihydroxyflavone	Serine/threonine-protein kinase 6	O14965	AURKA	0.044
Polygonatum sibiricum(PGS)	MOL006331	4',5-Dihydroxyflavone	5-hydroxytryptamine 1A receptor	P08908	HTR1A	0.044
Polygonatum sibiricum(PGS)	MOL006331	4',5-Dihydroxyflavone	Alpha-2A adrenergic receptor	P08913	ADRA2A	0.044
Polygonatum sibiricum(PGS)	MOL006331	4',5-Dihydroxyflavone	Cannabinoid receptor 1	P21554	CNR1	0.044
Polygonatum sibiricum(PGS)	MOL006331	4',5-Dihydroxyflavone	Endothelin-1 receptor	P25101	EDNRA	0.044
Polygonatum sibiricum(PGS)	MOL006331	4',5-Dihydroxyflavone	Melatonin receptor type 1A	P48039	MTNR1A	0.044
Polygonatum sibiricum(PGS)	MOL006331	4',5-Dihydroxyflavone	Thyroid hormone receptor alpha	P10827	THRA	0.045
Polygonatum sibiricum(PGS)	MOL006331	4',5-Dihydroxyflavone	Alpha-1A adrenergic receptor	P35348	ADRA1A	0.045
Polygonatum sibiricum(PGS)	MOL006331	4',5-Dihydroxyflavone	Aldo-keto reductase family 1 member C1	Q04828	AKR1C1	0.045
Polygonatum sibiricum(PGS)	MOL006331	4',5-Dihydroxyflavone	S-methyl-5-thioadenosine phosphorylase	Q13126	MTAP	0.045
Polygonatum sibiricum(PGS)	MOL006331	4',5-Dihydroxyflavone	Thiamin pyrophosphokinase 1	Q9H3S4	TPK1	0.045
Polygonatum sibiricum(PGS)	MOL006331	4',5-Dihydroxyflavone	Toll-like receptor 7	Q9NYK1	TLR7	0.045
Polygonatum sibiricum(PGS)	MOL006331	4',5-Dihydroxyflavone	Cytochrome P450 19A1	P11511	CYP19A1	0.046

Polygonatum sibiricum(PGS)	MOL006331	4',5-Dihydroxyflavone	Nitric oxide synthase, inducible	P35228	NOS2	0.046
Polygonatum sibiricum(PGS)	MOL006331	4',5-Dihydroxyflavone	L-amino-acid oxidase	Q96RQ9	IL4I1	0.046
Polygonatum sibiricum(PGS)	MOL006331	4',5-Dihydroxyflavone	Tyrosine-protein phosphatase non-receptor type 1	P18031	PTPN1	0.047
Polygonatum sibiricum(PGS)	MOL006331	4',5-Dihydroxyflavone	DNA polymerase kappa	Q9UBT6	POLK	0.047
Polygonatum sibiricum(PGS)	MOL006331	4',5-Dihydroxyflavone	D-HSCDK2	O75100	CA11	0.048
Polygonatum sibiricum(PGS)	MOL006331	4',5-Dihydroxyflavone	Beta-2 adrenergic receptor	P07550	ADRB2	0.048
Polygonatum sibiricum(PGS)	MOL006331	4',5-Dihydroxyflavone	Solute carrier family 12 member 1	Q13621	SLC12A1	0.048
Polygonatum sibiricum(PGS)	MOL006331	4',5-Dihydroxyflavone	Nuclear receptor coactivator 5	Q9HCD5	NCOA5	0.048
Polygonatum sibiricum(PGS)	MOL006331	4',5-Dihydroxyflavone	C-jun-amino-terminal kinase-interacting protein 1	Q9UQF2	MAPK8IP1	0.048
Polygonatum sibiricum(PGS)	MOL006331	4',5-Dihydroxyflavone	Proto-oncogene tyrosine-protein kinase Src	P12931	SRC	0.049
Polygonatum sibiricum(PGS)	MOL006331	4',5-Dihydroxyflavone	Nuclear receptor coactivator 2	Q15596	NCOA2	0.049
Polygonatum sibiricum(PGS)	MOL006331	4',5-Dihydroxyflavone	D(2) dopamine receptor	P14416	DRD2	0.052
Polygonatum sibiricum(PGS)	MOL006331	4',5-Dihydroxyflavone	Alpha-2B adrenergic receptor	P18089	ADRA2B	0.052
Polygonatum sibiricum(PGS)	MOL006331	4',5-Dihydroxyflavone	Alpha-2C adrenergic receptor	P18825	ADRA2C	0.052
Polygonatum sibiricum(PGS)	MOL006331	4',5-Dihydroxyflavone	D(1A) dopamine receptor	P21728	DRD1	0.052
Polygonatum sibiricum(PGS)	MOL006331	4',5-Dihydroxyflavone	D(1B) dopamine receptor	P21918	DRD5	0.052
Polygonatum sibiricum(PGS)	MOL006331	4',5-Dihydroxyflavone	D(3) dopamine receptor	P35462	DRD3	0.052
Polygonatum sibiricum(PGS)	MOL006331	4',5-Dihydroxyflavone	MAP kinase-activated protein kinase 2	P49137	MAPKAPK2	0.052
Polygonatum sibiricum(PGS)	MOL006331	4',5-Dihydroxyflavone	Interferon gamma	P01579	IFNG	0.053
Polygonatum sibiricum(PGS)	MOL006331	4',5-Dihydroxyflavone	Carbonic anhydrase 1	P00915	CA1	0.056
Polygonatum sibiricum(PGS)	MOL006331	4',5-Dihydroxyflavone	Mu-type opioid receptor	P35372	OPRM1	0.056
Polygonatum sibiricum(PGS)	MOL006331	4',5-Dihydroxyflavone	Hemoglobin subunit alpha	P69905	HBA1	0.056
Polygonatum sibiricum(PGS)	MOL006331	4',5-Dihydroxyflavone	cGMP-specific 3',5'-cyclic phosphodiesterase	O76074	PDE5A	0.059
Polygonatum sibiricum(PGS)	MOL006331	4',5-Dihydroxyflavone	3 beta-hydroxysteroid dehydrogenase/Delta 5-->4-isomerase type I	P14060	HSD3B1	0.06
Polygonatum sibiricum(PGS)	MOL006331	4',5-Dihydroxyflavone	cAMP-dependent protein kinase inhibitor alpha	P61925	PKIA	0.06
Polygonatum sibiricum(PGS)	MOL006331	4',5-Dihydroxyflavone	Beta-1 adrenergic receptor	P08588	ADRB1	0.061
Polygonatum sibiricum(PGS)	MOL006331	4',5-Dihydroxyflavone	Nitric-oxide synthase, endothelial	P29474	NOS3	0.061
Polygonatum sibiricum(PGS)	MOL006331	4',5-Dihydroxyflavone	Tubulin alpha-3 chain	Q71U36	TUBA1A	0.061
Polygonatum sibiricum(PGS)	MOL006331	4',5-Dihydroxyflavone	Cell division control protein 2 homolog	P06493	CDK1	0.065
Polygonatum sibiricum(PGS)	MOL006331	4',5-Dihydroxyflavone	DNA topoisomerase 2-alpha	P11388	TOP2A	0.066
Polygonatum sibiricum(PGS)	MOL006331	4',5-Dihydroxyflavone	Estradiol 17-beta-dehydrogenase 1	P14061	HSD17B1	0.067
Polygonatum sibiricum(PGS)	MOL006331	4',5-Dihydroxyflavone	Carbonic anhydrase 2	P00918	CA2	0.071
Polygonatum sibiricum(PGS)	MOL006331	4',5-Dihydroxyflavone	Inhibitor of nuclear factor kappa-B kinase subunit alpha	O15111	CHUK	0.073
Polygonatum sibiricum(PGS)	MOL006331	4',5-Dihydroxyflavone	Arachidonate 5-lipoxygenase	P09917	ALOX5	0.073
Polygonatum sibiricum(PGS)	MOL006331	4',5-Dihydroxyflavone	cAMP-specific 3',5'-cyclic phosphodiesterase 4A	P27815	PDE4A	0.078
Polygonatum sibiricum(PGS)	MOL006331	4',5-Dihydroxyflavone	Cell division protein kinase 5	Q00535	CDK5	0.082
Polygonatum sibiricum(PGS)	MOL006331	4',5-Dihydroxyflavone	Dihydrofolate reductase	P00374	DHFR	0.084
Polygonatum sibiricum(PGS)	MOL006331	4',5-Dihydroxyflavone	RAC-alpha serine/threonine-protein kinase	P31749	AKT1	0.091
Polygonatum sibiricum(PGS)	MOL006331	4',5-Dihydroxyflavone	Trypsin-1	P07477	PRSS1	0.095
Polygonatum sibiricum(PGS)	MOL006331	4',5-Dihydroxyflavone	Peroxisome proliferator-activated receptor gamma	P37231	PPARG	0.098
Polygonatum sibiricum(PGS)	MOL006331	4',5-Dihydroxyflavone	Prothrombin	P00734	F2	0.112
Polygonatum sibiricum(PGS)	MOL006331	4',5-Dihydroxyflavone	Cell division protein kinase 2	P24941	CDK2	0.135
Polygonatum sibiricum(PGS)	MOL006331	4',5-Dihydroxyflavone	cAMP-dependent protein kinase catalytic subunit alpha	P17612	PRKACA	0.153
Polygonatum sibiricum(PGS)	MOL006331	4',5-Dihydroxyflavone	Nuclear receptor coactivator 1	Q15788	NCOA1	0.199
Polygonatum sibiricum(PGS)	MOL006331	4',5-Dihydroxyflavone	Prostaglandin G/H synthase 1	P23219	PTGS1	0.215
Polygonatum sibiricum(PGS)	MOL006331	4',5-Dihydroxyflavone	Estrogen receptor beta	Q92731	ESR2	0.365
Polygonatum sibiricum(PGS)	MOL006331	4',5-Dihydroxyflavone	Progesterone receptor	P06401	PGR	0.391

Polygonatum sibiricum(PGS)	MOL006331	4',5-Dihydroxyflavone	Prostaglandin G/H synthase 2	P35354	PTGS2	0.398
Polygonatum sibiricum(PGS)	MOL006331	4',5-Dihydroxyflavone	Cyclin-A2	P20248	CCNA2	0.411
Polygonatum sibiricum(PGS)	MOL006331	4',5-Dihydroxyflavone	Estrogen receptor	P03372	ESR1	1
Polygonatum sibiricum(PGS)	MOL006670	aucubigenin	DNA-directed RNA polymerase II subunit RPB1	P24928	POLR2A	Validated
Polygonatum sibiricum(PGS)	MOL009760	sibiricoside A_qt	Aldose reductase	P15121	AKR1B1	0.014
Polygonatum sibiricum(PGS)	MOL009760	sibiricoside A_qt	Prostaglandin G/H synthase 1	P23219	PTGS1	0.014
Polygonatum sibiricum(PGS)	MOL009760	sibiricoside A_qt	Mitogen-activated protein kinase 3	P27361	MAPK3	0.014
Polygonatum sibiricum(PGS)	MOL009760	sibiricoside A_qt	Prostaglandin G/H synthase 2	P35354	PTGS2	0.014
Polygonatum sibiricum(PGS)	MOL009760	sibiricoside A_qt	Peroxisome proliferator-activated receptor delta	Q03181	PPARD	0.014
Polygonatum sibiricum(PGS)	MOL009760	sibiricoside A_qt	Elongation factor Tu GTP-binding domain-containing protein 1	Q7Z2Z2	EFL1	0.015
Polygonatum sibiricum(PGS)	MOL009760	sibiricoside A_qt	Gamma-aminobutyric-acid receptor subunit alpha-1	P14867	GABRA1	0.016
Polygonatum sibiricum(PGS)	MOL009760	sibiricoside A_qt	Gamma-aminobutyric-acid receptor subunit alpha-2	P47869	GABRA2	0.016
Polygonatum sibiricum(PGS)	MOL009760	sibiricoside A_qt	Muscarinic acetylcholine receptor M4	P08173	CHRM4	0.023
Polygonatum sibiricum(PGS)	MOL009760	sibiricoside A_qt	Muscarinic acetylcholine receptor M5	P08912	CHRM5	0.023
Polygonatum sibiricum(PGS)	MOL009760	sibiricoside A_qt	Histamine H1 receptor	P35367	HRH1	0.023
Polygonatum sibiricum(PGS)	MOL009760	sibiricoside A_qt	Retinoic acid receptor RXR-gamma	P48443	RXRG	0.023
Polygonatum sibiricum(PGS)	MOL009760	sibiricoside A_qt	Retinoic acid-induced protein 3	Q8NFJ5	GPRC5A	0.023
Polygonatum sibiricum(PGS)	MOL009760	sibiricoside A_qt	D(2) dopamine receptor	P14416	DRD2	0.026
Polygonatum sibiricum(PGS)	MOL009760	sibiricoside A_qt	D(1A) dopamine receptor	P21728	DRD1	0.026
Polygonatum sibiricum(PGS)	MOL009760	sibiricoside A_qt	5-hydroxytryptamine 2B receptor	P41595	HTR2B	0.026
Polygonatum sibiricum(PGS)	MOL009760	sibiricoside A_qt	Nuclear receptor coactivator 2	Q15596	NCOA2	0.026
Polygonatum sibiricum(PGS)	MOL009760	sibiricoside A_qt	Neuronal acetylcholine receptor subunit alpha-3	P32297	CHRNA3	0.03
Polygonatum sibiricum(PGS)	MOL009760	sibiricoside A_qt	Neuronal acetylcholine receptor subunit alpha-7	P36544	CHRNA7	0.03
Polygonatum sibiricum(PGS)	MOL009760	sibiricoside A_qt	Neuronal acetylcholine receptor subunit alpha-4	P43681	CHRNA4	0.03
Polygonatum sibiricum(PGS)	MOL009760	sibiricoside A_qt	Retinoic acid receptor alpha	P10276	RARA	0.031
Polygonatum sibiricum(PGS)	MOL009760	sibiricoside A_qt	Retinoic acid receptor beta	P10826	RARB	0.031
Polygonatum sibiricum(PGS)	MOL009760	sibiricoside A_qt	Opioid receptor, sigma 1	Q5T1J1	SIGMAR1	0.032
Polygonatum sibiricum(PGS)	MOL009760	sibiricoside A_qt	Glutamate [NMDA] receptor subunit 3A	Q8TCU5	GRIN3A	0.032
Polygonatum sibiricum(PGS)	MOL009760	sibiricoside A_qt	Sigma 1-type opioid receptor	Q99720	SIGMAR1	0.032
Polygonatum sibiricum(PGS)	MOL009760	sibiricoside A_qt	Dehydrogenase/reductase SDR family member 8	Q8NBQ5	HSD17B11	0.036
Polygonatum sibiricum(PGS)	MOL009760	sibiricoside A_qt	Ig kappa chain C region	P01834	IGKC	0.038
Polygonatum sibiricum(PGS)	MOL009760	sibiricoside A_qt	Ig gamma-1 chain C region	P01857	IGHG1	0.038
Polygonatum sibiricum(PGS)	MOL009760	sibiricoside A_qt	Ig gamma-2 chain C region	P01859	IGHG2	0.038
Polygonatum sibiricum(PGS)	MOL009760	sibiricoside A_qt	Retinal dehydrogenase 2	O94788	ALDH1A2	0.042
Polygonatum sibiricum(PGS)	MOL009760	sibiricoside A_qt	Retinal dehydrogenase 1	P00352	ALDH1A1	0.042
Polygonatum sibiricum(PGS)	MOL009760	sibiricoside A_qt	Muscarinic acetylcholine receptor M1	P11229	CHRM1	0.043
Polygonatum sibiricum(PGS)	MOL009760	sibiricoside A_qt	Annexin A1	P04083	ANXA1	0.046
Polygonatum sibiricum(PGS)	MOL009760	sibiricoside A_qt	Nuclear receptor subfamily 1 group I member 3	Q14994	NR1I3	0.049
Polygonatum sibiricum(PGS)	MOL009760	sibiricoside A_qt	Serine/threonine-protein phosphatase 2A catalytic subunit alpha isoform	P67775	PPP2CA	0.051
Polygonatum sibiricum(PGS)	MOL009760	sibiricoside A_qt	Serine/threonine-protein phosphatase 2A 56 kDa regulatory subunit gamma isoform	Q13362	PPP2R5C	0.051
Polygonatum sibiricum(PGS)	MOL009760	sibiricoside A_qt	3 beta-hydroxysteroid dehydrogenase/Delta 5-->4-isomerase type II	P26439	HSD3B2	0.058
Polygonatum sibiricum(PGS)	MOL009760	sibiricoside A_qt	Cytosolic phospholipase A2	P47712	PLA2G4A	0.06
Polygonatum sibiricum(PGS)	MOL009760	sibiricoside A_qt	Microtubule-associated protein 2	P11137	MAP2	0.061
Polygonatum sibiricum(PGS)	MOL009760	sibiricoside A_qt	Microtubule-associated protein 1A	P78559	MAP1A	0.061
Polygonatum sibiricum(PGS)	MOL009760	sibiricoside A_qt	Prolactin receptor	P16471	PRLR	0.062
Polygonatum sibiricum(PGS)	MOL009760	sibiricoside A_qt	3-oxo-5-alpha-steroid 4-dehydrogenase 2	P31213	SRD5A2	0.062
Polygonatum sibiricum(PGS)	MOL009760	sibiricoside A_qt	Estrogen receptor beta	Q92731	ESR2	0.062

Polygonatum sibiricum(PGS)	MOL009760	sibiricoside A_qt	Gonadotropin-releasing hormone receptor	P30968	GNRHR	0.063
Polygonatum sibiricum(PGS)	MOL009760	sibiricoside A_qt	Gonadotropin-releasing hormone II receptor	Q96P88	GNRHR2	0.063
Polygonatum sibiricum(PGS)	MOL009760	sibiricoside A_qt	Corticosteroid 11-beta-dehydrogenase isozyme 1	P28845	HSD11B1	0.064
Polygonatum sibiricum(PGS)	MOL009760	sibiricoside A_qt	Retinoic acid receptor gamma-1	P13631	RARG	0.065
Polygonatum sibiricum(PGS)	MOL009760	sibiricoside A_qt	Nuclear receptor 0B1	P51843	NR0B1	0.079
Polygonatum sibiricum(PGS)	MOL009760	sibiricoside A_qt	Bile salt sulfotransferase	Q06520	SULT2A1	0.081
Polygonatum sibiricum(PGS)	MOL009760	sibiricoside A_qt	Retinoic acid receptor RXR-alpha	P19793	RXRA	0.091
Polygonatum sibiricum(PGS)	MOL009760	sibiricoside A_qt	Retinoic acid receptor RXR-beta	P28702	RXRB	0.101
Polygonatum sibiricum(PGS)	MOL009760	sibiricoside A_qt	DNA polymerase kappa	Q9UBT6	POLK	0.11
Polygonatum sibiricum(PGS)	MOL009760	sibiricoside A_qt	Mediator of RNA polymerase II transcription subunit 1	Q15648	MED1	0.115
Polygonatum sibiricum(PGS)	MOL009760	sibiricoside A_qt	3 beta-hydroxysteroid dehydrogenase/Delta 5-->4-isomerase type I	P14060	HSD3B1	0.116
Polygonatum sibiricum(PGS)	MOL009760	sibiricoside A_qt	Cannabinoid receptor 2	P34972	CNR2	0.116
Polygonatum sibiricum(PGS)	MOL009760	sibiricoside A_qt	NADPH oxidase organizer 1	Q8NFA2	NOXO1	0.118
Polygonatum sibiricum(PGS)	MOL009760	sibiricoside A_qt	Thiamin pyrophosphokinase 1	Q9H3S4	TPK1	0.118
Polygonatum sibiricum(PGS)	MOL009760	sibiricoside A_qt	Nuclear receptor coactivator 5	Q9HCD5	NCOA5	0.123
Polygonatum sibiricum(PGS)	MOL009760	sibiricoside A_qt	3-oxo-5-alpha-steroid 4-dehydrogenase 1	P18405	SRD5A1	0.134
Polygonatum sibiricum(PGS)	MOL009760	sibiricoside A_qt	Aldo-keto reductase family 1 member C1	Q04828	AKR1C1	0.137
Polygonatum sibiricum(PGS)	MOL009760	sibiricoside A_qt	Muscarinic acetylcholine receptor M2	P08172	CHRM2	0.142
Polygonatum sibiricum(PGS)	MOL009760	sibiricoside A_qt	Neuronal acetylcholine receptor subunit alpha-2	Q15822	CHRNA2	0.151
Polygonatum sibiricum(PGS)	MOL009760	sibiricoside A_qt	Estradiol 17-beta-dehydrogenase 1	P14061	HSD17B1	0.199
Polygonatum sibiricum(PGS)	MOL009760	sibiricoside A_qt	Androgen receptor	P10275	AR	0.209
Polygonatum sibiricum(PGS)	MOL009760	sibiricoside A_qt	Nuclear receptor coactivator 1	Q15788	NCOA1	0.251
Polygonatum sibiricum(PGS)	MOL009760	sibiricoside A_qt	Mineralocorticoid receptor	P08235	NR3C2	0.355
Polygonatum sibiricum(PGS)	MOL009760	sibiricoside A_qt	Glucocorticoid receptor	P04150	NR3C1	0.388
Polygonatum sibiricum(PGS)	MOL009760	sibiricoside A_qt	Progesterone receptor	P06401	PGR	0.943
Polygonatum sibiricum(PGS)	MOL009760	sibiricoside A_qt	Estrogen receptor	P03372	ESR1	1
Polygonatum sibiricum(PGS)	MOL009766	zhonghualiaoine 1	Bile salt sulfotransferase	Q06520	SULT2A1	0.12
Polygonatum sibiricum(PGS)	MOL009766	zhonghualiaoine 1	Dehydrogenase/reductase SDR family member 8	Q8NBQ5	HSD17B11	0.12
Polygonatum sibiricum(PGS)	MOL009766	zhonghualiaoine 1	Ig kappa chain C region	P01834	IGKC	0.124
Polygonatum sibiricum(PGS)	MOL009766	zhonghualiaoine 1	Ig gamma-1 chain C region	P01857	IGHG1	0.124
Polygonatum sibiricum(PGS)	MOL009766	zhonghualiaoine 1	Ig gamma-2 chain C region	P01859	IGHG2	0.124
Polygonatum sibiricum(PGS)	MOL009766	zhonghualiaoine 1	Androgen receptor	P10275	AR	0.181
Polygonatum sibiricum(PGS)	MOL009766	zhonghualiaoine 1	Microtubule-associated protein 2	P11137	MAP2	0.181
Polygonatum sibiricum(PGS)	MOL009766	zhonghualiaoine 1	Prolactin receptor	P16471	PRLR	0.181
Polygonatum sibiricum(PGS)	MOL009766	zhonghualiaoine 1	Microtubule-associated protein 1A	P78559	MAP1A	0.181
Polygonatum sibiricum(PGS)	MOL009766	zhonghualiaoine 1	3 beta-hydroxysteroid dehydrogenase/Delta 5-->4-isomerase type I	P14060	HSD3B1	0.222
Polygonatum sibiricum(PGS)	MOL009766	zhonghualiaoine 1	3-oxo-5-alpha-steroid 4-dehydrogenase 1	P18405	SRD5A1	0.234
Polygonatum sibiricum(PGS)	MOL009766	zhonghualiaoine 1	3-oxo-5-alpha-steroid 4-dehydrogenase 2	P31213	SRD5A2	0.234
Polygonatum sibiricum(PGS)	MOL009766	zhonghualiaoine 1	Progesterone receptor	P06401	PGR	0.458
Polygonatum sibiricum(PGS)	MOL009766	zhonghualiaoine 1	Mineralocorticoid receptor	P08235	NR3C2	0.458
Polygonatum sibiricum(PGS)	MOL009766	zhonghualiaoine 1	Prostaglandin G/H synthase 2	P35354	PTGS2	0.52
Polygonatum sibiricum(PGS)	MOL009766	zhonghualiaoine 1	Mediator of RNA polymerase II transcription subunit 1	Q15648	MED1	0.521
Polygonatum sibiricum(PGS)	MOL009766	zhonghualiaoine 1	NADPH oxidase organizer 1	Q8NFA2	NOXO1	0.527
Polygonatum sibiricum(PGS)	MOL009766	zhonghualiaoine 1	Estradiol 17-beta-dehydrogenase 1	P14061	HSD17B1	0.534
Polygonatum sibiricum(PGS)	MOL009766	zhonghualiaoine 1	Nuclear receptor coactivator 1	Q15788	NCOA1	0.534
Polygonatum sibiricum(PGS)	MOL009766	zhonghualiaoine 1	Epidermal growth factor receptor	P00533	EGFR	0.543
Polygonatum sibiricum(PGS)	MOL009766	zhonghualiaoine 1	Cannabinoid receptor 2	P34972	CNR2	0.549

Polygonatum sibiricum(PGS)	MOL009766	zhonghualiaoine 1	Nuclear receptor coactivator 5	Q9HCD5	NCOA5	0.601
Polygonatum sibiricum(PGS)	MOL009766	zhonghualiaoine 1	Estrogen receptor	P03372	ESR1	1
Astragali Radix（AgR）	MOL000033	(3S,8S,9S,10R,13R,14S,17R)-10,13-dimethyl-17-[(2R,5S)-5-propan-2-yloctan-2-yl]-2,3,4,7,8,9,11,12,14,15,16,17-dodecahydro-1H-cyclopenta[a]phenanthren-3-ol	Retinal dehydrogenase 2	O94788	ALDH1A2	0.02
Astragali Radix（AgR）	MOL000033	(3S,8S,9S,10R,13R,14S,17R)-10,13-dimethyl-17-[(2R,5S)-5-propan-2-yloctan-2-yl]-2,3,4,7,8,9,11,12,14,15,16,17-dodecahydro-1H-cyclopenta[a]phenanthren-3-ol	Retinal dehydrogenase 1	P00352	ALDH1A1	0.02
Astragali Radix（AgR）	MOL000033	(3S,8S,9S,10R,13R,14S,17R)-10,13-dimethyl-17-[(2R,5S)-5-propan-2-yloctan-2-yl]-2,3,4,7,8,9,11,12,14,15,16,17-dodecahydro-1H-cyclopenta[a]phenanthren-3-ol	Nuclear receptor 0B1	P51843	NR0B1	0.02
Astragali Radix（AgR）	MOL000033	(3S,8S,9S,10R,13R,14S,17R)-10,13-dimethyl-17-[(2R,5S)-5-propan-2-yloctan-2-yl]-2,3,4,7,8,9,11,12,14,15,16,17-dodecahydro-1H-cyclopenta[a]phenanthren-3-ol	Retinoic acid-induced protein 3	Q8NFJ5	GPRC5A	0.02
Astragali Radix（AgR）	MOL000033	(3S,8S,9S,10R,13R,14S,17R)-10,13-dimethyl-17-[(2R,5S)-5-propan-2-yloctan-2-yl]-2,3,4,7,8,9,11,12,14,15,16,17-dodecahydro-1H-cyclopenta[a]phenanthren-3-ol	Nuclear receptor coactivator 2	Q15596	NCOA2	0.021
Astragali Radix（AgR）	MOL000033	(3S,8S,9S,10R,13R,14S,17R)-10,13-dimethyl-17-[(2R,5S)-5-propan-2-yloctan-2-yl]-2,3,4,7,8,9,11,12,14,15,16,17-dodecahydro-1H-cyclopenta[a]phenanthren-3-ol	Neuronal acetylcholine receptor subunit alpha-3	P32297	CHRNA3	0.027
Astragali Radix（AgR）	MOL000033	(3S,8S,9S,10R,13R,14S,17R)-10,13-dimethyl-17-[(2R,5S)-5-propan-2-yloctan-2-yl]-2,3,4,7,8,9,11,12,14,15,16,17-dodecahydro-1H-cyclopenta[a]phenanthren-3-ol	Neuronal acetylcholine receptor subunit alpha-7	P36544	CHRNA7	0.027
Astragali Radix（AgR）	MOL000033	(3S,8S,9S,10R,13R,14S,17R)-10,13-dimethyl-17-[(2R,5S)-5-propan-2-yloctan-2-yl]-2,3,4,7,8,9,11,12,14,15,16,17-dodecahydro-1H-cyclopenta[a]phenanthren-3-ol	Neuronal acetylcholine receptor subunit alpha-4	P43681	CHRNA4	0.027
Astragali Radix（AgR）	MOL000033	(3S,8S,9S,10R,13R,14S,17R)-10,13-dimethyl-17-[(2R,5S)-5-propan-2-yloctan-2-yl]-2,3,4,7,8,9,11,12,14,15,16,17-dodecahydro-1H-cyclopenta[a]phenanthren-3-ol	Ig kappa chain C region	P01834	IGKC	0.035
Astragali Radix（AgR）	MOL000033	(3S,8S,9S,10R,13R,14S,17R)-10,13-dimethyl-17-[(2R,5S)-5-propan-2-yloctan-2-yl]-2,3,4,7,8,9,11,12,14,15,16,17-dodecahydro-1H-cyclopenta[a]phenanthren-3-ol	Ig gamma-1 chain C region	P01857	IGHG1	0.035
Astragali Radix（AgR）	MOL000033	(3S,8S,9S,10R,13R,14S,17R)-10,13-dimethyl-17-[(2R,5S)-5-propan-2-yloctan-2-yl]-2,3,4,7,8,9,11,12,14,15,16,17-dodecahydro-1H-cyclopenta[a]phenanthren-3-ol	Ig gamma-2 chain C region	P01859	IGHG2	0.035
Astragali Radix（AgR）	MOL000033	(3S,8S,9S,10R,13R,14S,17R)-10,13-dimethyl-17-[(2R,5S)-5-propan-2-yloctan-2-yl]-2,3,4,7,8,9,11,12,14,15,16,17-dodecahydro-1H-cyclopenta[a]phenanthren-3-ol	Nuclear receptor subfamily 1 group I member 3	Q14994	NR1I3	0.039
Astragali Radix（AgR）	MOL000033	(3S,8S,9S,10R,13R,14S,17R)-10,13-dimethyl-17-[(2R,5S)-5-propan-2-yloctan-2-yl]-2,3,4,7,8,9,11,12,14,15,16,17-dodecahydro-1H-cyclopenta[a]phenanthren-3-ol	Dehydrogenase/reductase SDR family member 8	Q8NBQ5	HSD17B11	0.041
Astragali Radix（AgR）	MOL000033	(3S,8S,9S,10R,13R,14S,17R)-10,13-dimethyl-17-[(2R,5S)-5-propan-2-yloctan-2-yl]-2,3,4,7,8,9,11,12,14,15,16,17-dodecahydro-1H-cyclopenta[a]phenanthren-3-ol	3 beta-hydroxysteroid dehydrogenase/Delta 5-->4-isomerase type II	P26439	HSD3B2	0.056
Astragali Radix（AgR）	MOL000033	(3S,8S,9S,10R,13R,14S,17R)-10,13-dimethyl-17-[(2R,5S)-5-propan-2-yloctan-2-yl]-2,3,4,7,8,9,11,12,14,15,16,17-dodecahydro-1H-cyclopenta[a]phenanthren-3-ol	Retinoic acid receptor gamma-1	P13631	RARG	0.057

Astragali Radix（AgR）	MOL000033	(3S,8S,9S,10R,13R,14S,17R)-10,13-dimethyl-17-[(2R,5S)-5-propan-2-yloctan-2-yl]-2,3,4,7,8,9,11,12,14,15,16,17-dodecahydro-1H-cyclopenta[a]phenanthren-3-ol	Retinoic acid receptor RXR-gamma	P48443	RXRG	0.057
Astragali Radix（AgR）	MOL000033	(3S,8S,9S,10R,13R,14S,17R)-10,13-dimethyl-17-[(2R,5S)-5-propan-2-yloctan-2-yl]-2,3,4,7,8,9,11,12,14,15,16,17-dodecahydro-1H-cyclopenta[a]phenanthren-3-ol	Microtubule-associated protein 2	P11137	MAP2	0.059
Astragali Radix（AgR）	MOL000033	(3S,8S,9S,10R,13R,14S,17R)-10,13-dimethyl-17-[(2R,5S)-5-propan-2-yloctan-2-yl]-2,3,4,7,8,9,11,12,14,15,16,17-dodecahydro-1H-cyclopenta[a]phenanthren-3-ol	Prolactin receptor	P16471	PRLR	0.059
Astragali Radix（AgR）	MOL000033	(3S,8S,9S,10R,13R,14S,17R)-10,13-dimethyl-17-[(2R,5S)-5-propan-2-yloctan-2-yl]-2,3,4,7,8,9,11,12,14,15,16,17-dodecahydro-1H-cyclopenta[a]phenanthren-3-ol	3-oxo-5-alpha-steroid 4-dehydrogenase 2	P31213	SRD5A2	0.059
Astragali Radix（AgR）	MOL000033	(3S,8S,9S,10R,13R,14S,17R)-10,13-dimethyl-17-[(2R,5S)-5-propan-2-yloctan-2-yl]-2,3,4,7,8,9,11,12,14,15,16,17-dodecahydro-1H-cyclopenta[a]phenanthren-3-ol	Microtubule-associated protein 1A	P78559	MAP1A	0.059
Astragali Radix（AgR）	MOL000033	(3S,8S,9S,10R,13R,14S,17R)-10,13-dimethyl-17-[(2R,5S)-5-propan-2-yloctan-2-yl]-2,3,4,7,8,9,11,12,14,15,16,17-dodecahydro-1H-cyclopenta[a]phenanthren-3-ol	Estrogen receptor beta	Q92731	ESR2	0.059
Astragali Radix（AgR）	MOL000033	(3S,8S,9S,10R,13R,14S,17R)-10,13-dimethyl-17-[(2R,5S)-5-propan-2-yloctan-2-yl]-2,3,4,7,8,9,11,12,14,15,16,17-dodecahydro-1H-cyclopenta[a]phenanthren-3-ol	Gonadotropin-releasing hormone receptor	P30968	GNRHR	0.06
Astragali Radix（AgR）	MOL000033	(3S,8S,9S,10R,13R,14S,17R)-10,13-dimethyl-17-[(2R,5S)-5-propan-2-yloctan-2-yl]-2,3,4,7,8,9,11,12,14,15,16,17-dodecahydro-1H-cyclopenta[a]phenanthren-3-ol	Gonadotropin-releasing hormone II receptor	Q96P88	GNRHR2	0.06
Astragali Radix（AgR）	MOL000033	(3S,8S,9S,10R,13R,14S,17R)-10,13-dimethyl-17-[(2R,5S)-5-propan-2-yloctan-2-yl]-2,3,4,7,8,9,11,12,14,15,16,17-dodecahydro-1H-cyclopenta[a]phenanthren-3-ol	Retinoic acid receptor alpha	P10276	RARA	0.061
Astragali Radix（AgR）	MOL000033	(3S,8S,9S,10R,13R,14S,17R)-10,13-dimethyl-17-[(2R,5S)-5-propan-2-yloctan-2-yl]-2,3,4,7,8,9,11,12,14,15,16,17-dodecahydro-1H-cyclopenta[a]phenanthren-3-ol	Retinoic acid receptor beta	P10826	RARB	0.061
Astragali Radix（AgR）	MOL000033	(3S,8S,9S,10R,13R,14S,17R)-10,13-dimethyl-17-[(2R,5S)-5-propan-2-yloctan-2-yl]-2,3,4,7,8,9,11,12,14,15,16,17-dodecahydro-1H-cyclopenta[a]phenanthren-3-ol	Corticosteroid 11-beta-dehydrogenase isozyme 1	P28845	HSD11B1	0.061
Astragali Radix（AgR）	MOL000033	(3S,8S,9S,10R,13R,14S,17R)-10,13-dimethyl-17-[(2R,5S)-5-propan-2-yloctan-2-yl]-2,3,4,7,8,9,11,12,14,15,16,17-dodecahydro-1H-cyclopenta[a]phenanthren-3-ol	Cytosolic phospholipase A2	P47712	PLA2G4A	0.062
Astragali Radix（AgR）	MOL000033	(3S,8S,9S,10R,13R,14S,17R)-10,13-dimethyl-17-[(2R,5S)-5-propan-2-yloctan-2-yl]-2,3,4,7,8,9,11,12,14,15,16,17-dodecahydro-1H-cyclopenta[a]phenanthren-3-ol	Bile salt sulfotransferase	Q06520	SULT2A1	0.099
Astragali Radix（AgR）	MOL000033	(3S,8S,9S,10R,13R,14S,17R)-10,13-dimethyl-17-[(2R,5S)-5-propan-2-yloctan-2-yl]-2,3,4,7,8,9,11,12,14,15,16,17-dodecahydro-1H-cyclopenta[a]phenanthren-3-ol	DNA polymerase kappa	Q9UBT6	POLK	0.106
Astragali Radix（AgR）	MOL000033	(3S,8S,9S,10R,13R,14S,17R)-10,13-dimethyl-17-[(2R,5S)-5-propan-2-yloctan-2-yl]-2,3,4,7,8,9,11,12,14,15,16,17-dodecahydro-1H-cyclopenta[a]phenanthren-3-ol	Prostaglandin G/H synthase 2	P35354	PTGS2	0.112

Astragali Radix（AgR）	MOL000033	(3S,8S,9S,10R,13R,14S,17R)-10,13-dimethyl-17-[(2R,5S)-5-propan-2-yloctan-2-yl]-2,3,4,7,8,9,11,12,14,15,16,17-dodecahydro-1H-cyclopenta[a]phenanthren-3-ol	Retinoic acid receptor RXR-alpha	P19793	RXRA	0.113
Astragali Radix（AgR）	MOL000033	(3S,8S,9S,10R,13R,14S,17R)-10,13-dimethyl-17-[(2R,5S)-5-propan-2-yloctan-2-yl]-2,3,4,7,8,9,11,12,14,15,16,17-dodecahydro-1H-cyclopenta[a]phenanthren-3-ol	Aldo-keto reductase family 1 member C1	Q04828	AKR1C1	0.114
Astragali Radix（AgR）	MOL000033	(3S,8S,9S,10R,13R,14S,17R)-10,13-dimethyl-17-[(2R,5S)-5-propan-2-yloctan-2-yl]-2,3,4,7,8,9,11,12,14,15,16,17-dodecahydro-1H-cyclopenta[a]phenanthren-3-ol	ATP-binding cassette transporter sub-family C member 8	Q09428	ABCC8	0.114
Astragali Radix（AgR）	MOL000033	(3S,8S,9S,10R,13R,14S,17R)-10,13-dimethyl-17-[(2R,5S)-5-propan-2-yloctan-2-yl]-2,3,4,7,8,9,11,12,14,15,16,17-dodecahydro-1H-cyclopenta[a]phenanthren-3-ol	3 beta-hydroxysteroid dehydrogenase/Delta 5-->4-isomerase type I	P14060	HSD3B1	0.12
Astragali Radix（AgR）	MOL000033	(3S,8S,9S,10R,13R,14S,17R)-10,13-dimethyl-17-[(2R,5S)-5-propan-2-yloctan-2-yl]-2,3,4,7,8,9,11,12,14,15,16,17-dodecahydro-1H-cyclopenta[a]phenanthren-3-ol	Nuclear receptor coactivator 5	Q9HCD5	NCOA5	0.122
Astragali Radix（AgR）	MOL000033	(3S,8S,9S,10R,13R,14S,17R)-10,13-dimethyl-17-[(2R,5S)-5-propan-2-yloctan-2-yl]-2,3,4,7,8,9,11,12,14,15,16,17-dodecahydro-1H-cyclopenta[a]phenanthren-3-ol	Cannabinoid receptor 2	P34972	CNR2	0.124
Astragali Radix（AgR）	MOL000033	(3S,8S,9S,10R,13R,14S,17R)-10,13-dimethyl-17-[(2R,5S)-5-propan-2-yloctan-2-yl]-2,3,4,7,8,9,11,12,14,15,16,17-dodecahydro-1H-cyclopenta[a]phenanthren-3-ol	Retinoic acid receptor RXR-beta	P28702	RXRB	0.128
Astragali Radix（AgR）	MOL000033	(3S,8S,9S,10R,13R,14S,17R)-10,13-dimethyl-17-[(2R,5S)-5-propan-2-yloctan-2-yl]-2,3,4,7,8,9,11,12,14,15,16,17-dodecahydro-1H-cyclopenta[a]phenanthren-3-ol	3-oxo-5-alpha-steroid 4-dehydrogenase 1	P18405	SRD5A1	0.132
Astragali Radix（AgR）	MOL000033	(3S,8S,9S,10R,13R,14S,17R)-10,13-dimethyl-17-[(2R,5S)-5-propan-2-yloctan-2-yl]-2,3,4,7,8,9,11,12,14,15,16,17-dodecahydro-1H-cyclopenta[a]phenanthren-3-ol	Glucocorticoid receptor	P04150	NR3C1	0.137
Astragali Radix（AgR）	MOL000033	(3S,8S,9S,10R,13R,14S,17R)-10,13-dimethyl-17-[(2R,5S)-5-propan-2-yloctan-2-yl]-2,3,4,7,8,9,11,12,14,15,16,17-dodecahydro-1H-cyclopenta[a]phenanthren-3-ol	Androgen receptor	P10275	AR	0.206
Astragali Radix（AgR）	MOL000033	(3S,8S,9S,10R,13R,14S,17R)-10,13-dimethyl-17-[(2R,5S)-5-propan-2-yloctan-2-yl]-2,3,4,7,8,9,11,12,14,15,16,17-dodecahydro-1H-cyclopenta[a]phenanthren-3-ol	Mineralocorticoid receptor	P08235	NR3C2	0.208
Astragali Radix（AgR）	MOL000033	(3S,8S,9S,10R,13R,14S,17R)-10,13-dimethyl-17-[(2R,5S)-5-propan-2-yloctan-2-yl]-2,3,4,7,8,9,11,12,14,15,16,17-dodecahydro-1H-cyclopenta[a]phenanthren-3-ol	Nuclear receptor coactivator 1	Q15788	NCOA1	0.224
Astragali Radix（AgR）	MOL000033	(3S,8S,9S,10R,13R,14S,17R)-10,13-dimethyl-17-[(2R,5S)-5-propan-2-yloctan-2-yl]-2,3,4,7,8,9,11,12,14,15,16,17-dodecahydro-1H-cyclopenta[a]phenanthren-3-ol	Estradiol 17-beta-dehydrogenase 1	P14061	HSD17B1	0.23
Astragali Radix（AgR）	MOL000033	(3S,8S,9S,10R,13R,14S,17R)-10,13-dimethyl-17-[(2R,5S)-5-propan-2-yloctan-2-yl]-2,3,4,7,8,9,11,12,14,15,16,17-dodecahydro-1H-cyclopenta[a]phenanthren-3-ol	Progesterone receptor	P06401	PGR	0.948
Astragali Radix（AgR）	MOL000033	(3S,8S,9S,10R,13R,14S,17R)-10,13-dimethyl-17-[(2R,5S)-5-propan-2-yloctan-2-yl]-2,3,4,7,8,9,11,12,14,15,16,17-dodecahydro-1H-cyclopenta[a]phenanthren-3-ol	Estrogen receptor	P03372	ESR1	1
Astragali Radix（AgR）	MOL000098	quercetin	Adenosine A2b receptor	P29275	ADORA2B	0.011
Astragali Radix（AgR）	MOL000098	quercetin	Adenosine A3 receptor	P33765	SHMT1	0.011

Astragali Radix（AgR）	MOL000098	quercetin	cGMP-specific 3',5'-cyclic phosphodiesterase	O76074	PDE5A	0.012
Astragali Radix（AgR）	MOL000098	quercetin	Adenosine A1 receptor	P30542	ADORA1	0.012
Astragali Radix（AgR）	MOL000098	quercetin	cAMP-specific 3',5'-cyclic phosphodiesterase 4B	Q07343	PDE4B	0.012
Astragali Radix（AgR）	MOL000098	quercetin	Neuronal acetylcholine receptor subunit alpha-2	Q15822	CHRNA2	0.012
Astragali Radix（AgR）	MOL000098	quercetin	Potassium channel subfamily K member 1	O00180	KCNK1	0.013
Astragali Radix（AgR）	MOL000098	quercetin	Tyrosine 3-monooxygenase	P07101	TH	0.013
Astragali Radix（AgR）	MOL000098	quercetin	cGMP-inhibited 3',5'-cyclic phosphodiesterase A	Q14432	PDE3A	0.013
Astragali Radix（AgR）	MOL000098	quercetin	cAMP and cAMP-inhibited cGMP 3',5'-cyclic phosphodiesterase 10A	Q9Y233	PDE10A	0.013
Astragali Radix（AgR）	MOL000098	quercetin	Tyrosyl-tRNA synthetase, mitochondrial	Q9Y2Z4	YARS2	0.013
Astragali Radix（AgR）	MOL000098	quercetin	3-phosphoinositide-dependent protein kinase 1	O15530	PDPK1	0.014
Astragali Radix（AgR）	MOL000098	quercetin	Serine/threonine-protein kinase 17B	O94768	STK17B	0.014
Astragali Radix（AgR）	MOL000098	quercetin	Pepsin A	P00790	REN	0.014
Astragali Radix（AgR）	MOL000098	quercetin	Tyrosine-protein kinase Lyn	P07948	LYN	0.014
Astragali Radix（AgR）	MOL000098	quercetin	Muscarinic acetylcholine receptor M5	P08912	CHRM5	0.014
Astragali Radix（AgR）	MOL000098	quercetin	Cell division protein kinase 4	P11802	CDK4	0.014
Astragali Radix（AgR）	MOL000098	quercetin	Gamma-aminobutyric-acid receptor subunit alpha-1	P14867	GABRA1	0.014
Astragali Radix（AgR）	MOL000098	quercetin	Dipeptidyl peptidase 4	P27487	DPP4	0.014
Astragali Radix（AgR）	MOL000098	quercetin	ATP synthase subunit gamma, mitochondrial	P36542	ATP5F1C	0.014
Astragali Radix（AgR）	MOL000098	quercetin	Tyrosine-protein kinase CSK	P41240	CSK	0.014
Astragali Radix（AgR）	MOL000098	quercetin	Tyrosine-protein kinase ZAP-70	P43403	ZAP70	0.014
Astragali Radix（AgR）	MOL000098	quercetin	Tyrosine-protein kinase SYK	P43405	SYK	0.014
Astragali Radix（AgR）	MOL000098	quercetin	Gamma-aminobutyric-acid receptor subunit alpha-2	P47869	GABRA2	0.014
Astragali Radix（AgR）	MOL000098	quercetin	Cell division protein kinase 7	P50613	CDK7	0.014
Astragali Radix（AgR）	MOL000098	quercetin	Cell division protein kinase 9	P50750	CDK9	0.014
Astragali Radix（AgR）	MOL000098	quercetin	Cell division protein kinase 6	Q00534	CDK6	0.014
Astragali Radix（AgR）	MOL000098	quercetin	Protein kinase C theta type	Q04759	PRKCQ	0.014
Astragali Radix（AgR）	MOL000098	quercetin	Tyrosine-protein kinase ITK/TSK	Q08881	ITK	0.014
Astragali Radix（AgR）	MOL000098	quercetin	Cyclin-dependent kinase 5 activator 1	Q15078	CDK5R1	0.014
Astragali Radix（AgR）	MOL000098	quercetin	UDP-glucuronosyltransferase 3A1	Q6NUS8	UGT3A1	0.014
Astragali Radix（AgR）	MOL000098	quercetin	Dehydrogenase/reductase SDR family member 8	Q8NBQ5	HSD17B11	0.014
Astragali Radix（AgR）	MOL000098	quercetin	85 kDa calcium-independent phospholipase A2	O60733	PLA2G6	0.015
Astragali Radix（AgR）	MOL000098	quercetin	Ig gamma-2 chain C region	P01859	IGHG2	0.015
Astragali Radix（AgR）	MOL000098	quercetin	Retinoic acid receptor alpha	P10276	RARA	0.015
Astragali Radix（AgR）	MOL000098	quercetin	Retinoic acid receptor beta	P10826	RARB	0.015
Astragali Radix（AgR）	MOL000098	quercetin	Retinoic acid receptor gamma-1	P13631	RARG	0.015
Astragali Radix（AgR）	MOL000098	quercetin	Beta-3 adrenergic receptor	P13945	ADRB3	0.015
Astragali Radix（AgR）	MOL000098	quercetin	5-hydroxytryptamine 1D receptor	P28221	HTR1D	0.015
Astragali Radix（AgR）	MOL000098	quercetin	5-hydroxytryptamine 1B receptor	P28222	HTR1B	0.015
Astragali Radix（AgR）	MOL000098	quercetin	5-hydroxytryptamine 2C receptor	P28335	HTR2C	0.015
Astragali Radix（AgR）	MOL000098	quercetin	Amiloride-sensitive sodium channel subunit alpha	P37088	SCNN1A	0.015
Astragali Radix（AgR）	MOL000098	quercetin	Cytosolic phospholipase A2	P47712	PLA2G4A	0.015
Astragali Radix（AgR）	MOL000098	quercetin	Retinoic acid receptor RXR-gamma	P48443	RXRG	0.015
Astragali Radix（AgR）	MOL000098	quercetin	Amiloride-sensitive sodium channel subunit beta	P51168	SCNN1B	0.015
Astragali Radix（AgR）	MOL000098	quercetin	Amiloride-sensitive sodium channel subunit gamma	P51170	SCNN1G	0.015
Astragali Radix（AgR）	MOL000098	quercetin	Sodium/potassium-transporting ATPase gamma chain	P54710	FXYD2	0.015
Astragali Radix（AgR）	MOL000098	quercetin	Solute carrier family 12 member 3	P55017	SLC12A3	0.015
Astragali Radix（AgR）	MOL000098	quercetin	Calcium-activated potassium channel subunit alpha 1	Q12791	KCNMA1	0.015

Astragali Radix（AgR）	MOL000098	quercetin	5-hydroxytryptamine 3 receptor	P46098	HTR3A	0.017
Astragali Radix（AgR）	MOL000098	quercetin	Prostaglandin reductase 1	Q14914	PTGR1	0.017
Astragali Radix（AgR）	MOL000098	quercetin	Glutamate [NMDA] receptor subunit 3A	Q8TCU5	GRIN3A	0.017
Astragali Radix（AgR）	MOL000098	quercetin	DNA topoisomerase 1	P11387	TOP1	0.02
Astragali Radix（AgR）	MOL000098	quercetin	Macrophage migration inhibitory factor	P14174	MIF	0.02
Astragali Radix（AgR）	MOL000098	quercetin	Calcium/calmodulin-dependent protein kinase type II subunit delta	Q13557	CAMK2D	0.02
Astragali Radix（AgR）	MOL000098	quercetin	DNA topoisomerase I, mitochondrial	Q969P6	TOP1MT	0.02
Astragali Radix（AgR）	MOL000098	quercetin	STE20-like serine/threonine-protein kinase	Q9H2G2	SLK	0.02
Astragali Radix（AgR）	MOL000098	quercetin	Tripartite motif-containing protein 13	O60858	TRIM13	0.021
Astragali Radix（AgR）	MOL000098	quercetin	Chymase	P23946	CMA1	0.021
Astragali Radix（AgR）	MOL000098	quercetin	DNA-(apurinic or apyrimidinic site) lyase	P27695	APEX1	0.021
Astragali Radix（AgR）	MOL000098	quercetin	Succinate dehydrogenase [ubiquinone] cytochrome b small subunit, mitochondrial	O14521	SDHD	0.022
Astragali Radix（AgR）	MOL000098	quercetin	Inhibitor of nuclear factor kappa-B kinase subunit beta	O14920	IKBKB	0.022
Astragali Radix（AgR）	MOL000098	quercetin	Ribosyldihydronicotinamide dehydrogenase [quinone]	P16083	NQO2	0.022
Astragali Radix（AgR）	MOL000098	quercetin	Peptidyl-prolyl cis-trans isomerase, mitochondrial	P30405	PPIF	0.022
Astragali Radix（AgR）	MOL000098	quercetin	Prostacyclin receptor	P43119	PTGIR	0.022
Astragali Radix（AgR）	MOL000098	quercetin	Mitogen-activated protein kinase 8	P45983	MAPK8	0.022
Astragali Radix（AgR）	MOL000098	quercetin	Mitogen-activated protein kinase 10	P53779	MAPK10	0.022
Astragali Radix（AgR）	MOL000098	quercetin	Peroxisome proliferator-activated receptor delta	Q03181	PPARD	0.022
Astragali Radix（AgR）	MOL000098	quercetin	Group IIE secretory phospholipase A2	Q9NZK7	PLA2G2E	0.022
Astragali Radix（AgR）	MOL000098	quercetin	Cystine/glutamate transporter	Q9UPY5	SLC7A11	0.022
Astragali Radix（AgR）	MOL000098	quercetin	Oxysterols receptor LXR-beta	P55055	NR1H2	0.023
Astragali Radix（AgR）	MOL000098	quercetin	Oxysterols receptor LXR-alpha	Q13133	NR1H3	0.023
Astragali Radix（AgR）	MOL000098	quercetin	Sodium channel protein type 5 subunit alpha	Q14524	SCN5A	0.023
Astragali Radix（AgR）	MOL000098	quercetin	Phenylalanine-4-hydroxylase	P00439	PAH	0.024
Astragali Radix（AgR）	MOL000098	quercetin	Rho-associated protein kinase 1	Q13464	ROCK1	0.024
Astragali Radix（AgR）	MOL000098	quercetin	Alpha-1D adrenergic receptor	P25100	ADRA1D	0.025
Astragali Radix（AgR）	MOL000098	quercetin	D(4) dopamine receptor	P21917	DRD4	0.026
Astragali Radix（AgR）	MOL000098	quercetin	Keratin, type II cytoskeletal 7	P08729	KRT7	0.028
Astragali Radix（AgR）	MOL000098	quercetin	Platelet glycoprotein IX	P14770	GP9	0.028
Astragali Radix（AgR）	MOL000098	quercetin	Adenosine A2a receptor	P29274	ADORA2A	0.029
Astragali Radix（AgR）	MOL000098	quercetin	Sterol O-acyltransferase 2	O75908	SOAT2	0.031
Astragali Radix（AgR）	MOL000098	quercetin	Sterol O-acyltransferase 1	P35610	SOAT1	0.031
Astragali Radix（AgR）	MOL000098	quercetin	Tubulin beta-2C chain	P68371	TUBB4B	0.031
Astragali Radix（AgR）	MOL000098	quercetin	3 beta-hydroxysteroid dehydrogenase/Delta 5-->4-isomerase type II	P26439	HSD3B2	0.032
Astragali Radix（AgR）	MOL000098	quercetin	Protein tyrosine kinase 2 beta	Q14289	PTK2B	0.032
Astragali Radix（AgR）	MOL000098	quercetin	Tyrosyl-tRNA synthetase, cytoplasmic	P54577	YARS	0.033
Astragali Radix（AgR）	MOL000098	quercetin	ATP synthase subunit beta, mitochondrial	P06576	ATP5F1B	0.034
Astragali Radix（AgR）	MOL000098	quercetin	Muscarinic acetylcholine receptor M4	P08173	CHRM4	0.034
Astragali Radix（AgR）	MOL000098	quercetin	Tyrosine-protein kinase HCK	P08631	HCK	0.034
Astragali Radix（AgR）	MOL000098	quercetin	Proto-oncogene serine/threonine-protein kinase Pim-1	P11309	PIM1	0.034
Astragali Radix（AgR）	MOL000098	quercetin	Muscarinic acetylcholine receptor M3	P20309	CHRM3	0.034
Astragali Radix（AgR）	MOL000098	quercetin	ATP synthase subunit alpha, mitochondrial	P25705	ATP5F1A	0.034
Astragali Radix（AgR）	MOL000098	quercetin	Phosphatidylinositol-4,5-bisphosphate 3-kinase catalytic subunit gamma isoform	P48736	PIK3CG	0.034
Astragali Radix（AgR）	MOL000098	quercetin	Epidermal growth factor receptor	P00533	EGFR	0.035
Astragali Radix（AgR）	MOL000098	quercetin	Carbonic anhydrase 1	P00915	CA1	0.035
Astragali Radix（AgR）	MOL000098	quercetin	Proto-oncogene tyrosine-protein kinase LCK	P06239	LCK	0.035

Astragali Radix（AgR）	MOL000098	quercetin	5-hydroxytryptamine 1A receptor	P08908	HTR1A	0.035
Astragali Radix（AgR）	MOL000098	quercetin	Glycogen phosphorylase, muscle form	P11217	PYGM	0.035
Astragali Radix（AgR）	MOL000098	quercetin	Alpha-1B adrenergic receptor	P35368	ADRA1B	0.035
Astragali Radix（AgR）	MOL000098	quercetin	Bile salt sulfotransferase	Q06520	SULT2A1	0.036
Astragali Radix（AgR）	MOL000098	quercetin	Ig kappa chain C region	P01834	IGKC	0.037
Astragali Radix（AgR）	MOL000098	quercetin	Ig gamma-1 chain C region	P01857	IGHG1	0.037
Astragali Radix（AgR）	MOL000098	quercetin	D(2) dopamine receptor	P14416	DRD2	0.037
Astragali Radix（AgR）	MOL000098	quercetin	D(3) dopamine receptor	P35462	DRD3	0.037
Astragali Radix（AgR）	MOL000098	quercetin	Estrogen-related receptor gamma	P62508	ESRRG	0.037
Astragali Radix（AgR）	MOL000098	quercetin	Muscarinic acetylcholine receptor M2	P08172	CHRM2	0.044
Astragali Radix（AgR）	MOL000098	quercetin	Muscarinic acetylcholine receptor M1	P11229	CHRM1	0.044
Astragali Radix（AgR）	MOL000098	quercetin	Retinoic acid receptor RXR-beta	P28702	RXRB	0.044
Astragali Radix（AgR）	MOL000098	quercetin	Aldo-keto reductase family 1 member C1	Q04828	AKR1C1	0.044
Astragali Radix（AgR）	MOL000098	quercetin	Alpha-2A adrenergic receptor	P08913	ADRA2A	0.045
Astragali Radix（AgR）	MOL000098	quercetin	Cytochrome P450 19A1	P11511	CYP19A1	0.045
Astragali Radix（AgR）	MOL000098	quercetin	Alpha-2C adrenergic receptor	P18825	ADRA2C	0.045
Astragali Radix（AgR）	MOL000098	quercetin	Alpha-1A adrenergic receptor	P35348	ADRA1A	0.045
Astragali Radix（AgR）	MOL000098	quercetin	Kynurenine--oxoglutarate transaminase 1	Q16773	KYAT1	0.045
Astragali Radix（AgR）	MOL000098	quercetin	Membrane copper amine oxidase	Q16853	AOC3	0.045
Astragali Radix（AgR）	MOL000098	quercetin	NADPH oxidase organizer 1	Q8NFA2	NOXO1	0.045
Astragali Radix（AgR）	MOL000098	quercetin	Serine/threonine-protein kinase 6	O14965	AURKA	0.046
Astragali Radix（AgR）	MOL000098	quercetin	Thyroid hormone receptor beta-1	P10828	THRB	0.046
Astragali Radix（AgR）	MOL000098	quercetin	Egl nine homolog 1	Q9GZT9	EGLN1	0.046
Astragali Radix（AgR）	MOL000098	quercetin	Tubulin beta-1 chain	Q9H4B7	TUBB1	0.046
Astragali Radix（AgR）	MOL000098	quercetin	Cannabinoid receptor 1	P21554	CNR1	0.047
Astragali Radix（AgR）	MOL000098	quercetin	D(1A) dopamine receptor	P21728	DRD1	0.047
Astragali Radix（AgR）	MOL000098	quercetin	D(1B) dopamine receptor	P21918	DRD5	0.047
Astragali Radix（AgR）	MOL000098	quercetin	Endothelin-1 receptor	P25101	EDNRA	0.047
Astragali Radix（AgR）	MOL000098	quercetin	Thiamin pyrophosphokinase 1	Q9H3S4	TPK1	0.047
Astragali Radix（AgR）	MOL000098	quercetin	Glucocorticoid receptor	P04150	NR3C1	0.048
Astragali Radix（AgR）	MOL000098	quercetin	Ig kappa chain V-II region RPMI 6410	P06310	IGKV2-30	0.048
Astragali Radix（AgR）	MOL000098	quercetin	Leukotriene A-4 hydrolase	P09960	LTA4H	0.048
Astragali Radix（AgR）	MOL000098	quercetin	RAC-alpha serine/threonine-protein kinase	P31749	AKT1	0.048
Astragali Radix（AgR）	MOL000098	quercetin	Nitric oxide synthase, inducible	P35228	NOS2	0.048
Astragali Radix（AgR）	MOL000098	quercetin	S-methyl-5-thioadenosine phosphorylase	Q13126	MTAP	0.048
Astragali Radix（AgR）	MOL000098	quercetin	cAMP-specific 3',5'-cyclic phosphodiesterase 4A	P27815	PDE4A	0.049
Astragali Radix（AgR）	MOL000098	quercetin	Triosephosphate isomerase	P60174	TPI1	0.049
Astragali Radix（AgR）	MOL000098	quercetin	Solute carrier family 12 member 1	Q13621	SLC12A1	0.049
Astragali Radix（AgR）	MOL000098	quercetin	Thyroid hormone receptor alpha	P10827	THRA	0.05
Astragali Radix（AgR）	MOL000098	quercetin	Calmodulin	P62158		0.05
Astragali Radix（AgR）	MOL000098	quercetin	Casein kinase II subunit alpha	P68400	CSNK2A1	0.05
Astragali Radix（AgR）	MOL000098	quercetin	Neuropeptide Y	P01303	NPY	0.051
Astragali Radix（AgR）	MOL000098	quercetin	Peroxisome proliferator-activated receptor gamma	P37231	PPARG	0.051
Astragali Radix（AgR）	MOL000098	quercetin	Nuclear receptor coactivator 2	Q15596	NCOA2	0.051
Astragali Radix（AgR）	MOL000098	quercetin	Toll-like receptor 7	Q9NYK1	TLR7	0.051
Astragali Radix（AgR）	MOL000098	quercetin	C-jun-amino-terminal kinase-interacting protein 1	Q9UQF2	MAPK8IP1	0.051
Astragali Radix（AgR）	MOL000098	quercetin	cAMP-dependent protein kinase inhibitor alpha	P61925	PKIA	0.053

Astragali Radix（AgR）	MOL000098	quercetin	Interferon gamma	P01579	IFNG	0.055
Astragali Radix（AgR）	MOL000098	quercetin	Fibroblast growth factor receptor 2	P21802	FGFR2	0.055
Astragali Radix（AgR）	MOL000098	quercetin	Sodium-dependent noradrenaline transporter	P23975	SLC6A2	0.055
Astragali Radix（AgR）	MOL000098	quercetin	MAP kinase-activated protein kinase 2	P49137	MAPKAPK2	0.055
Astragali Radix（AgR）	MOL000098	quercetin	Glycogen synthase kinase-3 beta	P49841	GSK3B	0.055
Astragali Radix（AgR）	MOL000098	quercetin	Sodium-dependent dopamine transporter	Q01959	SLC6A3	0.055
Astragali Radix（AgR）	MOL000098	quercetin	Nuclear receptor coactivator 5	Q9HCD5	NCOA5	0.055
Astragali Radix（AgR）	MOL000098	quercetin	Carbonic anhydrase 2	P00918	CA2	0.056
Astragali Radix（AgR）	MOL000098	quercetin	Alpha-2B adrenergic receptor	P18089	ADRA2B	0.056
Astragali Radix（AgR）	MOL000098	quercetin	Proto-oncogene tyrosine-protein kinase Src	P12931	SRC	0.057
Astragali Radix（AgR）	MOL000098	quercetin	DNA polymerase kappa	Q9UBT6	POLK	0.058
Astragali Radix（AgR）	MOL000098	quercetin	DNA topoisomerase 2-alpha	P11388	TOP2A	0.059
Astragali Radix（AgR）	MOL000098	quercetin	3 beta-hydroxysteroid dehydrogenase/Delta 5-->4-isomerase type I	P14060	HSD3B1	0.061
Astragali Radix（AgR）	MOL000098	quercetin	Beta-2 adrenergic receptor	P07550	ADRB2	0.066
Astragali Radix（AgR）	MOL000098	quercetin	Beta-1 adrenergic receptor	P08588	ADRB1	0.066
Astragali Radix（AgR）	MOL000098	quercetin	Tubulin alpha-3 chain	Q71U36	TUBA1A	0.067
Astragali Radix（AgR）	MOL000098	quercetin	Prothrombin	P00734	F2	0.07
Astragali Radix（AgR）	MOL000098	quercetin	Estradiol 17-beta-dehydrogenase 1	P14061	HSD17B1	0.071
Astragali Radix（AgR）	MOL000098	quercetin	Delta-type opioid receptor	P41143	OPRD1	0.073
Astragali Radix（AgR）	MOL000098	quercetin	Kappa-type opioid receptor	P41145	OPRK1	0.073
Astragali Radix（AgR）	MOL000098	quercetin	D-HSCDK2	O75100	CA11	0.075
Astragali Radix（AgR）	MOL000098	quercetin	Sodium-dependent serotonin transporter	P31645	SLC6A4	0.075
Astragali Radix（AgR）	MOL000098	quercetin	Mineralocorticoid receptor	P08235	NR3C2	0.077
Astragali Radix（AgR）	MOL000098	quercetin	Inhibitor of nuclear factor kappa-B kinase subunit alpha	O15111	CHUK	0.079
Astragali Radix（AgR）	MOL000098	quercetin	Arachidonate 5-lipoxygenase	P09917	ALOX5	0.079
Astragali Radix（AgR）	MOL000098	quercetin	cAMP-dependent protein kinase catalytic subunit alpha	P17612	PRKACA	0.083
Astragali Radix（AgR）	MOL000098	quercetin	Dihydrofolate reductase	P00374	DHFR	0.091
Astragali Radix（AgR）	MOL000098	quercetin	Cell division control protein 2 homolog	P06493	CDK1	0.095
Astragali Radix（AgR）	MOL000098	quercetin	Cell division protein kinase 5	Q00535	CDK5	0.115
Astragali Radix（AgR）	MOL000098	quercetin	Trypsin-1	P07477	PRSS1	0.138
Astragali Radix（AgR）	MOL000098	quercetin	Cyclin-A2	P20248	CCNA2	0.151
Astragali Radix（AgR）	MOL000098	quercetin	Cell division protein kinase 2	P24941	CDK2	0.156
Astragali Radix（AgR）	MOL000098	quercetin	Hemoglobin subunit alpha	P69905	HBA1	0.169
Astragali Radix（AgR）	MOL000098	quercetin	Mu-type opioid receptor	P35372	OPRM1	0.179
Astragali Radix（AgR）	MOL000098	quercetin	Progesterone receptor	P06401	PGR	0.2
Astragali Radix（AgR）	MOL000098	quercetin	Nuclear receptor coactivator 1	Q15788	NCOA1	0.205
Astragali Radix（AgR）	MOL000098	quercetin	Prostaglandin G/H synthase 1	P23219	PTGS1	0.22
Astragali Radix（AgR）	MOL000098	quercetin	Estrogen receptor beta	Q92731	ESR2	0.357
Astragali Radix（AgR）	MOL000098	quercetin	Prostaglandin G/H synthase 2	P35354	PTGS2	0.39
Astragali Radix（AgR）	MOL000098	quercetin	Estrogen receptor	P03372	ESR1	1
Astragali Radix（AgR）	MOL000211	Mairin	Potassium channel subfamily K member 1	O00180	KCNK1	0.03
Astragali Radix（AgR）	MOL000211	Mairin	Gamma-aminobutyric-acid receptor subunit alpha-1	P14867	GABRA1	0.03
Astragali Radix（AgR）	MOL000211	Mairin	Gamma-aminobutyric-acid receptor subunit alpha-2	P47869	GABRA2	0.03
Astragali Radix（AgR）	MOL000211	Mairin	Sodium channel protein type 5 subunit alpha	Q14524	SCN5A	0.03
Astragali Radix（AgR）	MOL000211	Mairin	Ig kappa chain C region	P01834	IGKC	0.046
Astragali Radix（AgR）	MOL000211	Mairin	Ig gamma-1 chain C region	P01857	IGHG1	0.046
Astragali Radix（AgR）	MOL000211	Mairin	Ig gamma-2 chain C region	P01859	IGHG2	0.046

Astragali Radix（AgR）	MOL000211	Mairin	3-oxo-5-alpha-steroid 4-dehydrogenase 1	P18405	SRD5A1	0.075
Astragali Radix（AgR）	MOL000211	Mairin	3-oxo-5-alpha-steroid 4-dehydrogenase 2	P31213	SRD5A2	0.075
Astragali Radix（AgR）	MOL000211	Mairin	Bile salt sulfotransferase	Q06520	SULT2A1	0.075
Astragali Radix（AgR）	MOL000211	Mairin	Prolactin receptor	P16471	PRLR	0.092
Astragali Radix（AgR）	MOL000211	Mairin	Retinoic acid receptor RXR-alpha	P19793	RXRA	0.092
Astragali Radix（AgR）	MOL000211	Mairin	Gonadotropin-releasing hormone receptor	P30968	GNRHR	0.092
Astragali Radix（AgR）	MOL000211	Mairin	Nuclear receptor subfamily 1 group I member 3	Q14994	NR1I3	0.092
Astragali Radix（AgR）	MOL000211	Mairin	Gonadotropin-releasing hormone II receptor	Q96P88	GNRHR2	0.092
Astragali Radix（AgR）	MOL000211	Mairin	Nuclear receptor coactivator 5	Q9HCD5	NCOA5	0.145
Astragali Radix（AgR）	MOL000211	Mairin	Mediator of RNA polymerase II transcription subunit 1	Q15648	MED1	0.147
Astragali Radix（AgR）	MOL000211	Mairin	Estrogen receptor beta	Q92731	ESR2	0.194
Astragali Radix（AgR）	MOL000211	Mairin	Glucocorticoid receptor	P04150	NR3C1	0.197
Astragali Radix（AgR）	MOL000211	Mairin	Estradiol 17-beta-dehydrogenase 1	P14061	HSD17B1	0.242
Astragali Radix（AgR）	MOL000211	Mairin	Androgen receptor	P10275	AR	0.293
Astragali Radix（AgR）	MOL000211	Mairin	Nuclear receptor coactivator 1	Q15788	NCOA1	0.293
Astragali Radix（AgR）	MOL000211	Mairin	Mineralocorticoid receptor	P08235	NR3C2	0.296
Astragali Radix（AgR）	MOL000211	Mairin	Estrogen receptor	P03372	ESR1	0.897
Astragali Radix（AgR）	MOL000211	Mairin	Progesterone receptor	P06401	PGR	1
Astragali Radix（AgR）	MOL000239	Jaranol	Alpha-2A adrenergic receptor	P08913	ADRA2A	0.01
Astragali Radix（AgR）	MOL000239	Jaranol	D1 dopamine receptor-interacting protein calcyon	Q9NYX4	CALY	0.01
Astragali Radix（AgR）	MOL000239	Jaranol	T-cell receptor alpha chain C region	P01848	TRAC	0.011
Astragali Radix（AgR）	MOL000239	Jaranol	T-cell receptor beta chain C region	P01850	TRBC1	0.011
Astragali Radix（AgR）	MOL000239	Jaranol	Glutamate receptor 2	P42262	GRIA2	0.011
Astragali Radix（AgR）	MOL000239	Jaranol	Beta-2-microglobulin	P61769	B2M	0.011
Astragali Radix（AgR）	MOL000239	Jaranol	Opioid receptor, sigma 1	Q5T1J1	SIGMAR1	0.011
Astragali Radix（AgR）	MOL000239	Jaranol	Sigma 1-type opioid receptor	Q99720	SIGMAR1	0.011
Astragali Radix（AgR）	MOL000239	Jaranol	Death-associated protein kinase 3	O43293	DAPK3	0.012
Astragali Radix（AgR）	MOL000239	Jaranol	Trypsin-2	P07478	PRSS2	0.012
Astragali Radix（AgR）	MOL000239	Jaranol	Gamma-aminobutyric acid receptor subunit gamma-2	P18507	GABRG2	0.012
Astragali Radix（AgR）	MOL000239	Jaranol	Gamma-aminobutyric-acid receptor subunit beta-3	P28472	GABRB3	0.012
Astragali Radix（AgR）	MOL000239	Jaranol	Gamma-aminobutyric-acid receptor subunit beta-2	P47870	GABRB2	0.012
Astragali Radix（AgR）	MOL000239	Jaranol	Chromaffin granule amine transporter	P54219	SLC18A1	0.012
Astragali Radix（AgR）	MOL000239	Jaranol	Synaptic vesicular amine transporter	Q05940	SLC18A2	0.012
Astragali Radix（AgR）	MOL000239	Jaranol	Gamma-aminobutyric acid receptor subunit theta	Q9UN88	GABRQ	0.012
Astragali Radix（AgR）	MOL000239	Jaranol	Potassium channel subfamily K member 6	Q9Y257	KCNK6	0.012
Astragali Radix（AgR）	MOL000239	Jaranol	Glutamate [NMDA] receptor subunit epsilon-4	O15399	GRIN2D	0.013
Astragali Radix（AgR）	MOL000239	Jaranol	Glutamate [NMDA] receptor subunit 3B	O60391	GRIN3B	0.013
Astragali Radix（AgR）	MOL000239	Jaranol	Muscarinic acetylcholine receptor M4	P08173	CHRM4	0.013
Astragali Radix（AgR）	MOL000239	Jaranol	Substance-P receptor	P25103	TACR1	0.013
Astragali Radix（AgR）	MOL000239	Jaranol	Histamine H1 receptor	P35367	HRH1	0.013
Astragali Radix（AgR）	MOL000239	Jaranol	Glutamate [NMDA] receptor subunit zeta-1	Q05586	GRIN1	0.013
Astragali Radix（AgR）	MOL000239	Jaranol	5-hydroxytryptamine 4 receptor	Q13639	HTR4	0.013
Astragali Radix（AgR）	MOL000239	Jaranol	Cell division protein kinase 4	P11802	CDK4	0.014
Astragali Radix（AgR）	MOL000239	Jaranol	Cell division protein kinase 7	P50613	CDK7	0.014
Astragali Radix（AgR）	MOL000239	Jaranol	Cell division protein kinase 9	P50750	CDK9	0.014
Astragali Radix（AgR）	MOL000239	Jaranol	Cell division protein kinase 6	Q00534	CDK6	0.014
Astragali Radix（AgR）	MOL000239	Jaranol	Neuronal acetylcholine receptor subunit alpha-3	P32297	CHRNA3	0.015

Astragali Radix（AgR）	MOL000239	Jaranol	Neuronal acetylcholine receptor subunit alpha-7	P36544	CHRNA7	0.015
Astragali Radix（AgR）	MOL000239	Jaranol	Amiloride-sensitive sodium channel subunit alpha	P37088	SCNN1A	0.015
Astragali Radix（AgR）	MOL000239	Jaranol	Amiloride-sensitive sodium channel subunit beta	P51168	SCNN1B	0.015
Astragali Radix（AgR）	MOL000239	Jaranol	Amiloride-sensitive sodium channel subunit gamma	P51170	SCNN1G	0.015
Astragali Radix（AgR）	MOL000239	Jaranol	Tyrosine 3-monooxygenase	P07101	TH	0.016
Astragali Radix（AgR）	MOL000239	Jaranol	cAMP response element-binding protein	P16220	CREB1	0.016
Astragali Radix（AgR）	MOL000239	Jaranol	Nociceptin receptor	P41146	OPRL1	0.016
Astragali Radix（AgR）	MOL000239	Jaranol	Cyclin-dependent kinase 5 activator 1	Q15078	CDK5R1	0.016
Astragali Radix（AgR）	MOL000239	Jaranol	Tyrosyl-tRNA synthetase, mitochondrial	Q9Y2Z4	YARS2	0.016
Astragali Radix（AgR）	MOL000239	Jaranol	Serine/threonine-protein kinase 17B	O94768	STK17B	0.017
Astragali Radix（AgR）	MOL000239	Jaranol	Carbonic anhydrase 4	P22748	CA4	0.017
Astragali Radix（AgR）	MOL000239	Jaranol	ATP synthase subunit gamma, mitochondrial	P36542	ATP5F1C	0.017
Astragali Radix（AgR）	MOL000239	Jaranol	Sodium/potassium-transporting ATPase gamma chain	P54710	FXYD2	0.017
Astragali Radix（AgR）	MOL000239	Jaranol	Solute carrier family 12 member 3	P55017	SLC12A3	0.017
Astragali Radix（AgR）	MOL000239	Jaranol	Calcium-activated potassium channel subunit alpha 1	Q12791	KCNMA1	0.017
Astragali Radix（AgR）	MOL000239	Jaranol	UDP-glucuronosyltransferase 3A1	Q6NUS8	UGT3A1	0.017
Astragali Radix（AgR）	MOL000239	Jaranol	3-phosphoinositide-dependent protein kinase 1	O15530	PDPK1	0.018
Astragali Radix（AgR）	MOL000239	Jaranol	Tyrosine-protein kinase Lyn	P07948	LYN	0.018
Astragali Radix（AgR）	MOL000239	Jaranol	Tyrosine-protein kinase CSK	P41240	CSK	0.018
Astragali Radix（AgR）	MOL000239	Jaranol	Tyrosine-protein kinase ZAP-70	P43403	ZAP70	0.018
Astragali Radix（AgR）	MOL000239	Jaranol	Tyrosine-protein kinase SYK	P43405	SYK	0.018
Astragali Radix（AgR）	MOL000239	Jaranol	Protein kinase C theta type	Q04759	PRKCQ	0.018
Astragali Radix（AgR）	MOL000239	Jaranol	Tyrosine-protein kinase ITK/TSK	Q08881	ITK	0.018
Astragali Radix（AgR）	MOL000239	Jaranol	Gamma-aminobutyric acid receptor subunit rho-3	A8MPY1	GABRR3	0.019
Astragali Radix（AgR）	MOL000239	Jaranol	Gamma-aminobutyric acid receptor subunit pi	O00591	GABRP	0.019
Astragali Radix（AgR）	MOL000239	Jaranol	Gamma-aminobutyric acid receptor subunit delta	O14764	GABRD	0.019
Astragali Radix（AgR）	MOL000239	Jaranol	Retinoic acid receptor alpha	P10276	RARA	0.019
Astragali Radix（AgR）	MOL000239	Jaranol	Retinoic acid receptor beta	P10826	RARB	0.019
Astragali Radix（AgR）	MOL000239	Jaranol	Retinoic acid receptor gamma-1	P13631	RARG	0.019
Astragali Radix（AgR）	MOL000239	Jaranol	Gamma-aminobutyric-acid receptor subunit beta-1	P18505	GABRB1	0.019
Astragali Radix（AgR）	MOL000239	Jaranol	Gamma-aminobutyric-acid receptor subunit rho-1	P24046	GABRR1	0.019
Astragali Radix（AgR）	MOL000239	Jaranol	Gamma-aminobutyric acid receptor subunit rho-2	P28476	GABRR2	0.019
Astragali Radix（AgR）	MOL000239	Jaranol	Retinoic acid receptor RXR-gamma	P48443	RXRG	0.019
Astragali Radix（AgR）	MOL000239	Jaranol	Gamma-aminobutyric acid receptor subunit epsilon	P78334	GABRE	0.019
Astragali Radix（AgR）	MOL000239	Jaranol	Prostaglandin reductase 1	Q14914	PTGR1	0.019
Astragali Radix（AgR）	MOL000239	Jaranol	Gamma-aminobutyric acid receptor subunit gamma-1	Q8N1C3	GABRG1	0.019
Astragali Radix（AgR）	MOL000239	Jaranol	Gamma-aminobutyric acid receptor subunit gamma-3	Q99928	GABRG3	0.019
Astragali Radix（AgR）	MOL000239	Jaranol	Neuronal acetylcholine receptor subunit alpha-2	Q15822	CHRNA2	0.02
Astragali Radix（AgR）	MOL000239	Jaranol	Retinoic acid receptor RXR-alpha	P19793	RXRA	0.021
Astragali Radix（AgR）	MOL000239	Jaranol	D(4) dopamine receptor	P21917	DRD4	0.022
Astragali Radix（AgR）	MOL000239	Jaranol	Potassium voltage-gated channel subfamily H member 2	Q12809	KCNH2	0.022
Astragali Radix（AgR）	MOL000239	Jaranol	Potassium voltage-gated channel subfamily H member 6	Q9H252	KCNH6	0.022
Astragali Radix（AgR）	MOL000239	Jaranol	Potassium channel subfamily K member 1	O00180	KCNK1	0.023
Astragali Radix（AgR）	MOL000239	Jaranol	Succinate dehydrogenase [ubiquinone] cytochrome b small subunit, mitochondrial	O14521	SDHD	0.023
Astragali Radix（AgR）	MOL000239	Jaranol	Myosin light chain 6B	P14649	MYL6B	0.023
Astragali Radix（AgR）	MOL000239	Jaranol	Myosin regulatory light chain 12A	P19105	MYL12A	0.023
Astragali Radix（AgR）	MOL000239	Jaranol	Adenosine A2a receptor	P29274	ADORA2A	0.023

Astragali Radix（AgR）	MOL000239	Jaranol	Nitric-oxide synthase, endothelial	P29474	NOS3	0.023
Astragali Radix（AgR）	MOL000239	Jaranol	Nitric-oxide synthase, brain	P29475	NOS1	0.023
Astragali Radix（AgR）	MOL000239	Jaranol	Adenosine A1 receptor	P30542	ADORA1	0.023
Astragali Radix（AgR）	MOL000239	Jaranol	Calcium/calmodulin-dependent protein kinase type II subunit delta	Q13557	CAMK2D	0.023
Astragali Radix（AgR）	MOL000239	Jaranol	cGMP-inhibited 3',5'-cyclic phosphodiesterase A	Q14432	PDE3A	0.023
Astragali Radix（AgR）	MOL000239	Jaranol	STE20-like serine/threonine-protein kinase	Q9H2G2	SLK	0.023
Astragali Radix（AgR）	MOL000239	Jaranol	cAMP and cAMP-inhibited cGMP 3',5'-cyclic phosphodiesterase 10A	Q9Y233	PDE10A	0.023
Astragali Radix（AgR）	MOL000239	Jaranol	Tumor necrosis factor	P01375	TNF	0.024
Astragali Radix（AgR）	MOL000239	Jaranol	Nuclear factor NF-kappa-B p105 subunit	P19838	NFKB1	0.024
Astragali Radix（AgR）	MOL000239	Jaranol	Peptidyl-prolyl cis-trans isomerase, mitochondrial	P30405	PPIF	0.024
Astragali Radix（AgR）	MOL000239	Jaranol	Methionine aminopeptidase 1	P53582	METAP1	0.024
Astragali Radix（AgR）	MOL000239	Jaranol	85 kDa calcium-independent phospholipase A2	O60733	PLA2G6	0.025
Astragali Radix（AgR）	MOL000239	Jaranol	Ig kappa chain C region	P01834	IGKC	0.025
Astragali Radix（AgR）	MOL000239	Jaranol	Ig gamma-1 chain C region	P01857	IGHG1	0.025
Astragali Radix（AgR）	MOL000239	Jaranol	Amine oxidase [flavin-containing] A	P21397	MAOA	0.025
Astragali Radix（AgR）	MOL000239	Jaranol	Prostacyclin receptor	P43119	PTGIR	0.025
Astragali Radix（AgR）	MOL000239	Jaranol	Cytosolic phospholipase A2	P47712	PLA2G4A	0.025
Astragali Radix（AgR）	MOL000239	Jaranol	Peroxisome proliferator-activated receptor delta	Q03181	PPARD	0.025
Astragali Radix（AgR）	MOL000239	Jaranol	Glycogen phosphorylase, muscle form	P11217	PYGM	0.026
Astragali Radix（AgR）	MOL000239	Jaranol	Mitogen-activated protein kinase 8	P45983	MAPK8	0.026
Astragali Radix（AgR）	MOL000239	Jaranol	Mitogen-activated protein kinase 10	P53779	MAPK10	0.026
Astragali Radix（AgR）	MOL000239	Jaranol	Tyrosine-protein kinase JAK2	O60674	JAK2	0.027
Astragali Radix（AgR）	MOL000239	Jaranol	Tyrosine-protein kinase JAK1	P23458	JAK1	0.027
Astragali Radix（AgR）	MOL000239	Jaranol	Tyrosine-protein kinase JAK3	P52333	JAK3	0.027
Astragali Radix（AgR）	MOL000239	Jaranol	Oxysterols receptor LXR-beta	P55055	NR1H2	0.027
Astragali Radix（AgR）	MOL000239	Jaranol	Oxysterols receptor LXR-alpha	Q13133	NR1H3	0.027
Astragali Radix（AgR）	MOL000239	Jaranol	Ribosyldihydronicotinamide dehydrogenase [quinone]	P16083	NQO2	0.028
Astragali Radix（AgR）	MOL000239	Jaranol	DNA-(apurinic or apyrimidinic site) lyase	P27695	APEX1	0.028
Astragali Radix（AgR）	MOL000239	Jaranol	Sodium channel protein type 10 subunit alpha	Q9Y5Y9	SCN10A	0.028
Astragali Radix（AgR）	MOL000239	Jaranol	Inhibitor of nuclear factor kappa-B kinase subunit beta	O14920	IKBKB	0.029
Astragali Radix（AgR）	MOL000239	Jaranol	Muscarinic acetylcholine receptor M2	P08172	CHRM2	0.029
Astragali Radix（AgR）	MOL000239	Jaranol	Mineralocorticoid receptor	P08235	NR3C2	0.029
Astragali Radix（AgR）	MOL000239	Jaranol	Muscarinic acetylcholine receptor M3	P20309	CHRM3	0.029
Astragali Radix（AgR）	MOL000239	Jaranol	5-hydroxytryptamine 3 receptor	P46098	HTR3A	0.029
Astragali Radix（AgR）	MOL000239	Jaranol	Cystine/glutamate transporter	Q9UPY5	SLC7A11	0.029
Astragali Radix（AgR）	MOL000239	Jaranol	Sterol O-acyltransferase 2	O75908	SOAT2	0.03
Astragali Radix（AgR）	MOL000239	Jaranol	Macrophage migration inhibitory factor	P14174	MIF	0.03
Astragali Radix（AgR）	MOL000239	Jaranol	Acetylcholinesterase	P22303	ACHE	0.03
Astragali Radix（AgR）	MOL000239	Jaranol	Sterol O-acyltransferase 1	P35610	SOAT1	0.03
Astragali Radix（AgR）	MOL000239	Jaranol	Epidermal growth factor receptor	P00533	EGFR	0.031
Astragali Radix（AgR）	MOL000239	Jaranol	Tubulin alpha-1 chain	P68366	TUBA4A	0.031
Astragali Radix（AgR）	MOL000239	Jaranol	Keratin, type II cytoskeletal 7	P08729	KRT7	0.033
Astragali Radix（AgR）	MOL000239	Jaranol	Platelet glycoprotein IX	P14770	GP9	0.033
Astragali Radix（AgR）	MOL000239	Jaranol	Gonadotropin-releasing hormone receptor	P30968	GNRHR	0.033
Astragali Radix（AgR）	MOL000239	Jaranol	RAC-beta serine/threonine-protein kinase	P31751	AKT2	0.033
Astragali Radix（AgR）	MOL000239	Jaranol	Gonadotropin-releasing hormone II receptor	Q96P88	GNRHR2	0.033
Astragali Radix（AgR）	MOL000239	Jaranol	Tripartite motif-containing protein 13	O60858	TRIM13	0.034

Astragali Radix（AgR）	MOL000239	Jaranol	Alpha-2B adrenergic receptor	P18089	ADRA2B	0.034
Astragali Radix（AgR）	MOL000239	Jaranol	Alpha-2C adrenergic receptor	P18825	ADRA2C	0.034
Astragali Radix（AgR）	MOL000239	Jaranol	Alpha-1D adrenergic receptor	P25100	ADRA1D	0.034
Astragali Radix（AgR）	MOL000239	Jaranol	5-hydroxytryptamine 1D receptor	P28221	HTR1D	0.034
Astragali Radix（AgR）	MOL000239	Jaranol	5-hydroxytryptamine 1B receptor	P28222	HTR1B	0.034
Astragali Radix（AgR）	MOL000239	Jaranol	5-hydroxytryptamine 2C receptor	P28335	HTR2C	0.034
Astragali Radix（AgR）	MOL000239	Jaranol	Gamma-aminobutyric-acid receptor subunit alpha-5	P31644	GABRA5	0.034
Astragali Radix（AgR）	MOL000239	Jaranol	Gamma-aminobutyric-acid receptor subunit alpha-3	P34903	GABRA3	0.034
Astragali Radix（AgR）	MOL000239	Jaranol	D(3) dopamine receptor	P35462	DRD3	0.034
Astragali Radix（AgR）	MOL000239	Jaranol	5-hydroxytryptamine 2B receptor	P41595	HTR2B	0.034
Astragali Radix（AgR）	MOL000239	Jaranol	ATP synthase subunit beta, mitochondrial	P06576	ATP5F1B	0.035
Astragali Radix（AgR）	MOL000239	Jaranol	ATP synthase subunit alpha, mitochondrial	P25705	ATP5F1A	0.035
Astragali Radix（AgR）	MOL000239	Jaranol	Tyrosyl-tRNA synthetase, cytoplasmic	P54577	YARS	0.035
Astragali Radix（AgR）	MOL000239	Jaranol	Protein tyrosine kinase 2 beta	Q14289	PTK2B	0.035
Astragali Radix（AgR）	MOL000239	Jaranol	Tyrosine-protein kinase HCK	P08631	HCK	0.036
Astragali Radix（AgR）	MOL000239	Jaranol	Phosphatidylinositol-4,5-bisphosphate 3-kinase catalytic subunit gamma isoform	P48736	PIK3CG	0.036
Astragali Radix（AgR）	MOL000239	Jaranol	Proto-oncogene serine/threonine-protein kinase Pim-1	P11309	PIM1	0.037
Astragali Radix（AgR）	MOL000239	Jaranol	Proto-oncogene tyrosine-protein kinase LCK	P06239	LCK	0.038
Astragali Radix（AgR）	MOL000239	Jaranol	Microtubule-associated protein 2	P11137	MAP2	0.039
Astragali Radix（AgR）	MOL000239	Jaranol	3 beta-hydroxysteroid dehydrogenase/Delta 5-->4-isomerase type I	P14060	HSD3B1	0.039
Astragali Radix（AgR）	MOL000239	Jaranol	3 beta-hydroxysteroid dehydrogenase/Delta 5-->4-isomerase type II	P26439	HSD3B2	0.039
Astragali Radix（AgR）	MOL000239	Jaranol	Estrogen-related receptor gamma	P62508	ESRRG	0.039
Astragali Radix（AgR）	MOL000239	Jaranol	Microtubule-associated protein 1A	P78559	MAP1A	0.039
Astragali Radix（AgR）	MOL000239	Jaranol	Gamma-aminobutyric-acid receptor subunit alpha-2	P47869	GABRA2	0.045
Astragali Radix（AgR）	MOL000239	Jaranol	D(1B) dopamine receptor	P21918	DRD5	0.046
Astragali Radix（AgR）	MOL000239	Jaranol	Alpha-1B adrenergic receptor	P35368	ADRA1B	0.047
Astragali Radix（AgR）	MOL000239	Jaranol	cGMP-specific 3',5'-cyclic phosphodiesterase	O76074	PDE5A	0.049
Astragali Radix（AgR）	MOL000239	Jaranol	Glucocorticoid receptor	P04150	NR3C1	0.049
Astragali Radix（AgR）	MOL000239	Jaranol	Ig kappa chain V-II region RPMI 6410	P06310	IGKV2-30	0.049
Astragali Radix（AgR）	MOL000239	Jaranol	Beta-1 adrenergic receptor	P08588	ADRB1	0.049
Astragali Radix（AgR）	MOL000239	Jaranol	L-amino-acid oxidase	Q96RQ9	IL4I1	0.049
Astragali Radix（AgR）	MOL000239	Jaranol	Serine/threonine-protein kinase 6	O14965	AURKA	0.05
Astragali Radix（AgR）	MOL000239	Jaranol	5-hydroxytryptamine 1A receptor	P08908	HTR1A	0.05
Astragali Radix（AgR）	MOL000239	Jaranol	Cytochrome P450 19A1	P11511	CYP19A1	0.05
Astragali Radix（AgR）	MOL000239	Jaranol	Fibroblast growth factor receptor 2	P21802	FGFR2	0.05
Astragali Radix（AgR）	MOL000239	Jaranol	RAC-alpha serine/threonine-protein kinase	P31749	AKT1	0.05
Astragali Radix（AgR）	MOL000239	Jaranol	Triosephosphate isomerase	P60174	TPI1	0.05
Astragali Radix（AgR）	MOL000239	Jaranol	Aldo-keto reductase family 1 member C1	Q04828	AKR1C1	0.05
Astragali Radix（AgR）	MOL000239	Jaranol	cAMP-specific 3',5'-cyclic phosphodiesterase 4C	Q08493	PDE4C	0.05
Astragali Radix（AgR）	MOL000239	Jaranol	Egl nine homolog 1	Q9GZT9	EGLN1	0.05
Astragali Radix（AgR）	MOL000239	Jaranol	Cannabinoid receptor 1	P21554	CNR1	0.051
Astragali Radix（AgR）	MOL000239	Jaranol	Nitric oxide synthase, inducible	P35228	NOS2	0.051
Astragali Radix（AgR）	MOL000239	Jaranol	Membrane copper amine oxidase	Q16853	AOC3	0.051
Astragali Radix（AgR）	MOL000239	Jaranol	Thiamin pyrophosphokinase 1	Q9H3S4	TPK1	0.051
Astragali Radix（AgR）	MOL000239	Jaranol	Dihydrofolate reductase	P00374	DHFR	0.052
Astragali Radix（AgR）	MOL000239	Jaranol	Thyroid hormone receptor beta-1	P10828	THRB	0.052
Astragali Radix（AgR）	MOL000239	Jaranol	Peroxisome proliferator-activated receptor alpha	Q07869	PPARA	0.052

Astragali Radix（AgR）	MOL000239	Jaranol	Tubulin beta-1 chain	Q9H4B7	TUBB1	0.052
Astragali Radix（AgR）	MOL000239	Jaranol	Toll-like receptor 7	Q9NYK1	TLR7	0.052
Astragali Radix（AgR）	MOL000239	Jaranol	Estradiol 17-beta-dehydrogenase 1	P14061	HSD17B1	0.053
Astragali Radix（AgR）	MOL000239	Jaranol	Gamma-aminobutyric-acid receptor subunit alpha-1	P14867	GABRA1	0.053
Astragali Radix（AgR）	MOL000239	Jaranol	Thyroid hormone receptor alpha	P10827	THRA	0.054
Astragali Radix（AgR）	MOL000239	Jaranol	Calmodulin	P62158		0.054
Astragali Radix（AgR）	MOL000239	Jaranol	Solute carrier family 12 member 1	Q13621	SLC12A1	0.054
Astragali Radix（AgR）	MOL000239	Jaranol	Neuropeptide Y	P01303	NPY	0.055
Astragali Radix（AgR）	MOL000239	Jaranol	Liver carboxylesterase 1	P23141	CES1	0.055
Astragali Radix（AgR）	MOL000239	Jaranol	Glycogen synthase kinase-3 beta	P49841	GSK3B	0.055
Astragali Radix（AgR）	MOL000239	Jaranol	C-jun-amino-terminal kinase-interacting protein 1	Q9UQF2	MAPK8IP1	0.055
Astragali Radix（AgR）	MOL000239	Jaranol	Calcium/calmodulin-dependent protein kinase type II alpha chain	Q9UQM7	CAMK2A	0.055
Astragali Radix（AgR）	MOL000239	Jaranol	Melatonin receptor type 1A	P48039	MTNR1A	0.056
Astragali Radix（AgR）	MOL000239	Jaranol	Tubulin alpha-3 chain	Q71U36	TUBA1A	0.056
Astragali Radix（AgR）	MOL000239	Jaranol	Glutamate [NMDA] receptor subunit 3A	Q8TCU5	GRIN3A	0.056
Astragali Radix（AgR）	MOL000239	Jaranol	Carbonic anhydrase 1	P00915	CA1	0.057
Astragali Radix（AgR）	MOL000239	Jaranol	Interferon gamma	P01579	IFNG	0.058
Astragali Radix（AgR）	MOL000239	Jaranol	ATP-sensitive inward rectifier potassium channel 1	P48048	KCNJ1	0.058
Astragali Radix（AgR）	MOL000239	Jaranol	Casein kinase II subunit alpha	P68400	CSNK2A1	0.058
Astragali Radix（AgR）	MOL000239	Jaranol	Nuclear receptor coactivator 5	Q9HCD5	NCOA5	0.059
Astragali Radix（AgR）	MOL000239	Jaranol	Muscarinic acetylcholine receptor M1	P11229	CHRM1	0.06
Astragali Radix（AgR）	MOL000239	Jaranol	Proto-oncogene tyrosine-protein kinase Src	P12931	SRC	0.061
Astragali Radix（AgR）	MOL000239	Jaranol	Group IIE secretory phospholipase A2	Q9NZK7	PLA2G2E	0.061
Astragali Radix（AgR）	MOL000239	Jaranol	DNA polymerase kappa	Q9UBT6	POLK	0.062
Astragali Radix（AgR）	MOL000239	Jaranol	Phospholipase A2	P04054	PLA2G1B	0.064
Astragali Radix（AgR）	MOL000239	Jaranol	Androgen receptor	P10275	AR	0.066
Astragali Radix（AgR）	MOL000239	Jaranol	Sodium channel protein type 5 subunit alpha	Q14524	SCN5A	0.066
Astragali Radix（AgR）	MOL000239	Jaranol	D-HSCDK2	O75100	CA11	0.068
Astragali Radix（AgR）	MOL000239	Jaranol	cAMP-dependent protein kinase inhibitor alpha	P61925	PKIA	0.068
Astragali Radix（AgR）	MOL000239	Jaranol	D(1A) dopamine receptor	P21728	DRD1	0.071
Astragali Radix（AgR）	MOL000239	Jaranol	D(2) dopamine receptor	P14416	DRD2	0.072
Astragali Radix（AgR）	MOL000239	Jaranol	Retinoic acid receptor RXR-beta	P28702	RXRB	0.072
Astragali Radix（AgR）	MOL000239	Jaranol	Sodium-dependent dopamine transporter	Q01959	SLC6A3	0.073
Astragali Radix（AgR）	MOL000239	Jaranol	Carbonic anhydrase 2	P00918	CA2	0.076
Astragali Radix（AgR）	MOL000239	Jaranol	cAMP-specific 3',5'-cyclic phosphodiesterase 4D	Q08499	PDE4D	0.077
Astragali Radix（AgR）	MOL000239	Jaranol	5-hydroxytryptamine 2A receptor	P28223	HTR2A	0.078
Astragali Radix（AgR）	MOL000239	Jaranol	MAP kinase-activated protein kinase 2	P49137	MAPKAPK2	0.082
Astragali Radix（AgR）	MOL000239	Jaranol	Cell division control protein 2 homolog	P06493	CDK1	0.086
Astragali Radix（AgR）	MOL000239	Jaranol	Nuclear receptor coactivator 2	Q15596	NCOA2	0.086
Astragali Radix（AgR）	MOL000239	Jaranol	Alpha-1A adrenergic receptor	P35348	ADRA1A	0.087
Astragali Radix（AgR）	MOL000239	Jaranol	Peroxisome proliferator-activated receptor gamma	P37231	PPARG	0.09
Astragali Radix（AgR）	MOL000239	Jaranol	Inhibitor of nuclear factor kappa-B kinase subunit alpha	O15111	CHUK	0.091
Astragali Radix（AgR）	MOL000239	Jaranol	Arachidonate 5-lipoxygenase	P09917	ALOX5	0.091
Astragali Radix（AgR）	MOL000239	Jaranol	Prothrombin	P00734	F2	0.093
Astragali Radix（AgR）	MOL000239	Jaranol	Progesterone receptor	P06401	PGR	0.097
Astragali Radix（AgR）	MOL000239	Jaranol	DNA topoisomerase 2-alpha	P11388	TOP2A	0.099
Astragali Radix（AgR）	MOL000239	Jaranol	cAMP-dependent protein kinase catalytic subunit alpha	P17612	PRKACA	0.103

Astragali Radix（AgR）	MOL000239	Jaranol	Cell division protein kinase 5	Q00535	CDK5	0.103
Astragali Radix（AgR）	MOL000239	Jaranol	Sodium-dependent serotonin transporter	P31645	SLC6A4	0.115
Astragali Radix（AgR）	MOL000239	Jaranol	Cell division protein kinase 2	P24941	CDK2	0.124
Astragali Radix（AgR）	MOL000239	Jaranol	Delta-type opioid receptor	P41143	OPRD1	0.125
Astragali Radix（AgR）	MOL000239	Jaranol	Sodium-dependent noradrenaline transporter	P23975	SLC6A2	0.134
Astragali Radix（AgR）	MOL000239	Jaranol	Trypsin-1	P07477	PRSS1	0.141
Astragali Radix（AgR）	MOL000239	Jaranol	Kappa-type opioid receptor	P41145	OPRK1	0.143
Astragali Radix（AgR）	MOL000239	Jaranol	cAMP-specific 3',5'-cyclic phosphodiesterase 4A	P27815	PDE4A	0.152
Astragali Radix（AgR）	MOL000239	Jaranol	cAMP-specific 3',5'-cyclic phosphodiesterase 4B	Q07343	PDE4B	0.152
Astragali Radix（AgR）	MOL000239	Jaranol	Hemoglobin subunit alpha	P69905	HBA1	0.18
Astragali Radix（AgR）	MOL000239	Jaranol	Nuclear receptor coactivator 1	Q15788	NCOA1	0.208
Astragali Radix（AgR）	MOL000239	Jaranol	Mu-type opioid receptor	P35372	OPRM1	0.213
Astragali Radix（AgR）	MOL000239	Jaranol	Cyclin-A2	P20248	CCNA2	0.26
Astragali Radix（AgR）	MOL000239	Jaranol	Prostaglandin G/H synthase 1	P23219	PTGS1	0.277
Astragali Radix（AgR）	MOL000239	Jaranol	Estrogen receptor beta	Q92731	ESR2	0.355
Astragali Radix（AgR）	MOL000239	Jaranol	Prostaglandin G/H synthase 2	P35354	PTGS2	0.6
Astragali Radix（AgR）	MOL000239	Jaranol	Estrogen receptor	P03372	ESR1	1
Astragali Radix（AgR）	MOL000296	hederagenin	Nuclear receptor 0B1	P51843	NR0B1	0.013
Astragali Radix（AgR）	MOL000296	hederagenin	Retinoic acid-induced protein 3	Q8NFJ5	GPRC5A	0.013
Astragali Radix（AgR）	MOL000296	hederagenin	Elongation factor Tu GTP-binding domain-containing protein 1	Q7Z2Z2	EFL1	0.016
Astragali Radix（AgR）	MOL000296	hederagenin	Tripartite motif-containing protein 13	O60858	TRIM13	0.027
Astragali Radix（AgR）	MOL000296	hederagenin	Nociceptin receptor	P41146	OPRL1	0.027
Astragali Radix（AgR）	MOL000296	hederagenin	Retinal dehydrogenase 2	O94788	ALDH1A2	0.028
Astragali Radix（AgR）	MOL000296	hederagenin	Retinal dehydrogenase 1	P00352	ALDH1A1	0.028
Astragali Radix（AgR）	MOL000296	hederagenin	Neuronal acetylcholine receptor subunit alpha-3	P32297	CHRNA3	0.03
Astragali Radix（AgR）	MOL000296	hederagenin	Neuronal acetylcholine receptor subunit alpha-7	P36544	CHRNA7	0.03
Astragali Radix（AgR）	MOL000296	hederagenin	Neuronal acetylcholine receptor subunit alpha-4	P43681	CHRNA4	0.03
Astragali Radix（AgR）	MOL000296	hederagenin	Dehydrogenase/reductase SDR family member 8	Q8NBQ5	HSD17B11	0.036
Astragali Radix（AgR）	MOL000296	hederagenin	Ig kappa chain C region	P01834	IGKC	0.04
Astragali Radix（AgR）	MOL000296	hederagenin	Ig gamma-1 chain C region	P01857	IGHG1	0.04
Astragali Radix（AgR）	MOL000296	hederagenin	Ig gamma-2 chain C region	P01859	IGHG2	0.04
Astragali Radix（AgR）	MOL000296	hederagenin	Retinoic acid receptor RXR-gamma	P48443	RXRG	0.043
Astragali Radix（AgR）	MOL000296	hederagenin	Nuclear receptor subfamily 1 group I member 3	Q14994	NR1I3	0.043
Astragali Radix（AgR）	MOL000296	hederagenin	Retinoic acid receptor alpha	P10276	RARA	0.051
Astragali Radix（AgR）	MOL000296	hederagenin	Retinoic acid receptor beta	P10826	RARB	0.051
Astragali Radix（AgR）	MOL000296	hederagenin	Muscarinic acetylcholine receptor M2	P08172	CHRM2	0.056
Astragali Radix（AgR）	MOL000296	hederagenin	Neuronal acetylcholine receptor subunit alpha-2	Q15822	CHRNA2	0.056
Astragali Radix（AgR）	MOL000296	hederagenin	3 beta-hydroxysteroid dehydrogenase/Delta 5-->4-isomerase type II	P26439	HSD3B2	0.057
Astragali Radix（AgR）	MOL000296	hederagenin	Microtubule-associated protein 2	P11137	MAP2	0.061
Astragali Radix（AgR）	MOL000296	hederagenin	Microtubule-associated protein 1A	P78559	MAP1A	0.061
Astragali Radix（AgR）	MOL000296	hederagenin	Prolactin receptor	P16471	PRLR	0.062
Astragali Radix（AgR）	MOL000296	hederagenin	3-oxo-5-alpha-steroid 4-dehydrogenase 2	P31213	SRD5A2	0.062
Astragali Radix（AgR）	MOL000296	hederagenin	Estrogen receptor beta	Q92731	ESR2	0.062
Astragali Radix（AgR）	MOL000296	hederagenin	Gonadotropin-releasing hormone receptor	P30968	GNRHR	0.063
Astragali Radix（AgR）	MOL000296	hederagenin	Gonadotropin-releasing hormone II receptor	Q96P88	GNRHR2	0.063
Astragali Radix（AgR）	MOL000296	hederagenin	Corticosteroid 11-beta-dehydrogenase isozyme 1	P28845	HSD11B1	0.065
Astragali Radix（AgR）	MOL000296	hederagenin	Delta-type opioid receptor	P41143	OPRD1	0.065

Astragali Radix（AgR）	MOL000296	hederagenin	Retinoic acid receptor gamma-1	P13631	RARG	0.074
Astragali Radix（AgR）	MOL000296	hederagenin	Retinoic acid receptor RXR-beta	P28702	RXRB	0.074
Astragali Radix（AgR）	MOL000296	hederagenin	Bile salt sulfotransferase	Q06520	SULT2A1	0.081
Astragali Radix（AgR）	MOL000296	hederagenin	Mu-type opioid receptor	P35372	OPRM1	0.103
Astragali Radix（AgR）	MOL000296	hederagenin	Kappa-type opioid receptor	P41145	OPRK1	0.103
Astragali Radix（AgR）	MOL000296	hederagenin	Retinoic acid receptor RXR-alpha	P19793	RXRA	0.104
Astragali Radix（AgR）	MOL000296	hederagenin	DNA polymerase kappa	Q9UBT6	POLK	0.112
Astragali Radix（AgR）	MOL000296	hederagenin	NADPH oxidase organizer 1	Q8NFA2	NOXO1	0.114
Astragali Radix（AgR）	MOL000296	hederagenin	3 beta-hydroxysteroid dehydrogenase/Delta 5-->4-isomerase type I	P14060	HSD3B1	0.115
Astragali Radix（AgR）	MOL000296	hederagenin	ATP-binding cassette transporter sub-family C member 8	Q09428	ABCC8	0.115
Astragali Radix（AgR）	MOL000296	hederagenin	Glucocorticoid receptor	P04150	NR3C1	0.116
Astragali Radix（AgR）	MOL000296	hederagenin	Mediator of RNA polymerase II transcription subunit 1	Q15648	MED1	0.121
Astragali Radix（AgR）	MOL000296	hederagenin	Nuclear receptor coactivator 5	Q9HCD5	NCOA5	0.125
Astragali Radix（AgR）	MOL000296	hederagenin	Cannabinoid receptor 2	P34972	CNR2	0.128
Astragali Radix（AgR）	MOL000296	hederagenin	Aldo-keto reductase family 1 member C1	Q04828	AKR1C1	0.131
Astragali Radix（AgR）	MOL000296	hederagenin	Androgen receptor	P10275	AR	0.135
Astragali Radix（AgR）	MOL000296	hederagenin	3-oxo-5-alpha-steroid 4-dehydrogenase 1	P18405	SRD5A1	0.135
Astragali Radix（AgR）	MOL000296	hederagenin	Estradiol 17-beta-dehydrogenase 1	P14061	HSD17B1	0.198
Astragali Radix（AgR）	MOL000296	hederagenin	Mineralocorticoid receptor	P08235	NR3C2	0.215
Astragali Radix（AgR）	MOL000296	hederagenin	Prostaglandin G/H synthase 2	P35354	PTGS2	0.236
Astragali Radix（AgR）	MOL000296	hederagenin	Nuclear receptor coactivator 1	Q15788	NCOA1	0.307
Astragali Radix（AgR）	MOL000296	hederagenin	Progesterone receptor	P06401	PGR	0.811
Astragali Radix（AgR）	MOL000296	hederagenin	Estrogen receptor	P03372	ESR1	1
Astragali Radix（AgR）	MOL000354	isorhamnetin	Sodium/hydrogen exchanger 1	P19634	SLC9A1	0.01
Astragali Radix（AgR）	MOL000354	isorhamnetin	Amiloride-sensitive amine oxidase [copper-containing]	P19801	AOC1	0.01
Astragali Radix（AgR）	MOL000354	isorhamnetin	Glutamate receptor 2	P42262	GRIA2	0.01
Astragali Radix（AgR）	MOL000354	isorhamnetin	5-hydroxytryptamine 6 receptor	P50406	HTR6	0.01
Astragali Radix（AgR）	MOL000354	isorhamnetin	Amiloride-sensitive sodium channel subunit delta	P51172	SCNN1D	0.01
Astragali Radix（AgR）	MOL000354	isorhamnetin	Chromaffin granule amine transporter	P54219	SLC18A1	0.01
Astragali Radix（AgR）	MOL000354	isorhamnetin	Amiloride-sensitive cation channel 2, neuronal	P78348	ASIC1	0.01
Astragali Radix（AgR）	MOL000354	isorhamnetin	Amiloride-sensitive cation channel 1, neuronal	Q16515	ASIC2	0.01
Astragali Radix（AgR）	MOL000354	isorhamnetin	Opioid receptor, sigma 1	Q5T1J1	SIGMAR1	0.01
Astragali Radix（AgR）	MOL000354	isorhamnetin	Sigma 1-type opioid receptor	Q99720	SIGMAR1	0.01
Astragali Radix（AgR）	MOL000354	isorhamnetin	Potassium channel subfamily K member 6	Q9Y257	KCNK6	0.01
Astragali Radix（AgR）	MOL000354	isorhamnetin	Sucrase-isomaltase, intestinal	P14410	SI	0.011
Astragali Radix（AgR）	MOL000354	isorhamnetin	2,4-dienoyl-CoA reductase, mitochondrial	Q16698	DECR1	0.011
Astragali Radix（AgR）	MOL000354	isorhamnetin	Sodium channel protein type 10 subunit alpha	Q9Y5Y9	SCN10A	0.011
Astragali Radix（AgR）	MOL000354	isorhamnetin	Gamma-aminobutyric acid receptor subunit rho-3	A8MPY1	GABRR3	0.012
Astragali Radix（AgR）	MOL000354	isorhamnetin	Gamma-aminobutyric acid receptor subunit pi	O00591	GABRP	0.012
Astragali Radix（AgR）	MOL000354	isorhamnetin	Gamma-aminobutyric acid receptor subunit delta	O14764	GABRD	0.012
Astragali Radix（AgR）	MOL000354	isorhamnetin	Retinoic acid receptor alpha	P10276	RARA	0.012
Astragali Radix（AgR）	MOL000354	isorhamnetin	Retinoic acid receptor beta	P10826	RARB	0.012
Astragali Radix（AgR）	MOL000354	isorhamnetin	Retinoic acid receptor gamma-1	P13631	RARG	0.012
Astragali Radix（AgR）	MOL000354	isorhamnetin	Gamma-aminobutyric-acid receptor subunit beta-1	P18505	GABRB1	0.012
Astragali Radix（AgR）	MOL000354	isorhamnetin	Gamma-aminobutyric acid receptor subunit gamma-2	P18507	GABRG2	0.012
Astragali Radix（AgR）	MOL000354	isorhamnetin	Gamma-aminobutyric-acid receptor subunit rho-1	P24046	GABRR1	0.012
Astragali Radix（AgR）	MOL000354	isorhamnetin	Gamma-aminobutyric acid receptor subunit rho-2	P28476	GABRR2	0.012

Astragali Radix（AgR）	MOL000354	isorhamnetin	Retinoic acid receptor RXR-beta	P28702	RXRB	0.012
Astragali Radix（AgR）	MOL000354	isorhamnetin	Gamma-aminobutyric-acid receptor subunit alpha-5	P31644	GABRA5	0.012
Astragali Radix（AgR）	MOL000354	isorhamnetin	Gamma-aminobutyric-acid receptor subunit alpha-3	P34903	GABRA3	0.012
Astragali Radix（AgR）	MOL000354	isorhamnetin	Retinoic acid receptor RXR-gamma	P48443	RXRG	0.012
Astragali Radix（AgR）	MOL000354	isorhamnetin	Gamma-aminobutyric acid receptor subunit epsilon	P78334	GABRE	0.012
Astragali Radix（AgR）	MOL000354	isorhamnetin	5-hydroxytryptamine 4 receptor	Q13639	HTR4	0.012
Astragali Radix（AgR）	MOL000354	isorhamnetin	Alpha-7 nicotinic cholinergic receptor subunit	Q693P7	CHRNA7	0.012
Astragali Radix（AgR）	MOL000354	isorhamnetin	Gamma-aminobutyric acid receptor subunit gamma-1	Q8N1C3	GABRG1	0.012
Astragali Radix（AgR）	MOL000354	isorhamnetin	Gamma-aminobutyric acid receptor subunit gamma-3	Q99928	GABRG3	0.012
Astragali Radix（AgR）	MOL000354	isorhamnetin	3-phosphoinositide-dependent protein kinase 1	O15530	PDPK1	0.014
Astragali Radix（AgR）	MOL000354	isorhamnetin	Alcohol dehydrogenase 1C	P00326	ADH1C	0.014
Astragali Radix（AgR）	MOL000354	isorhamnetin	Cytochrome c oxidase subunit 1	P00395	MT-CO1	0.014
Astragali Radix（AgR）	MOL000354	isorhamnetin	Cytochrome c oxidase subunit 2	P00403	MT-CO2	0.014
Astragali Radix（AgR）	MOL000354	isorhamnetin	Cytochrome c oxidase subunit 3	P00414	MT-CO3	0.014
Astragali Radix（AgR）	MOL000354	isorhamnetin	Cytochrome c oxidase subunit 6C	P09669	COX6C	0.014
Astragali Radix（AgR）	MOL000354	isorhamnetin	Cytochrome c oxidase subunit 8A, mitochondrial	P10176	COX8A	0.014
Astragali Radix（AgR）	MOL000354	isorhamnetin	Cytochrome c oxidase subunit 5B, mitochondrial	P10606	COX5B	0.014
Astragali Radix（AgR）	MOL000354	isorhamnetin	Cytochrome c oxidase subunit 4 isoform 1, mitochondrial	P13073	COX4I1	0.014
Astragali Radix（AgR）	MOL000354	isorhamnetin	Cytochrome c oxidase subunit 6B1	P14854	COX6B1	0.014
Astragali Radix（AgR）	MOL000354	isorhamnetin	Cytochrome c oxidase subunit 7C, mitochondrial	P15954	COX7C	0.014
Astragali Radix（AgR）	MOL000354	isorhamnetin	Ferrochelatase, mitochondrial	P22830	FECH	0.014
Astragali Radix（AgR）	MOL000354	isorhamnetin	Liver carboxylesterase 1	P23141	CES1	0.014
Astragali Radix（AgR）	MOL000354	isorhamnetin	Cytochrome c oxidase polypeptide 7A1, mitochondrial	P24310	COX7A1	0.014
Astragali Radix（AgR）	MOL000354	isorhamnetin	Cytochrome c oxidase subunit 7B, mitochondrial	P24311	COX7B	0.014
Astragali Radix（AgR）	MOL000354	isorhamnetin	Tyrosine-protein kinase CSK	P41240	CSK	0.014
Astragali Radix（AgR）	MOL000354	isorhamnetin	Tyrosine-protein kinase ZAP-70	P43403	ZAP70	0.014
Astragali Radix（AgR）	MOL000354	isorhamnetin	Tyrosine-protein kinase SYK	P43405	SYK	0.014
Astragali Radix（AgR）	MOL000354	isorhamnetin	Gastrotropin	P51161	FABP6	0.014
Astragali Radix（AgR）	MOL000354	isorhamnetin	Cytochrome c oxidase subunit 6A2, mitochondrial	Q02221	COX6A2	0.014
Astragali Radix（AgR）	MOL000354	isorhamnetin	Protein kinase C theta type	Q04759	PRKCQ	0.014
Astragali Radix（AgR）	MOL000354	isorhamnetin	Tyrosine-protein kinase ITK/TSK	Q08881	ITK	0.014
Astragali Radix（AgR）	MOL000354	isorhamnetin	Ferrochelatase	Q7KZA3	DKFZp686P18130	0.014
Astragali Radix（AgR）	MOL000354	isorhamnetin	Adenylosuccinate synthetase isozyme 1	Q8N142	ADSSL1	0.014
Astragali Radix（AgR）	MOL000354	isorhamnetin	Trypsin-2	P07478	PRSS2	0.015
Astragali Radix（AgR）	MOL000354	isorhamnetin	Elongation factor 2	P13639	EEF2	0.015
Astragali Radix（AgR）	MOL000354	isorhamnetin	Cyclin-dependent kinase 5 activator 1	Q15078	CDK5R1	0.015
Astragali Radix（AgR）	MOL000354	isorhamnetin	Poly [ADP-ribose] polymerase 3	Q9Y6F1	PARP3	0.015
Astragali Radix（AgR）	MOL000354	isorhamnetin	Tyrosine-protein kinase Lyn	P07948	LYN	0.016
Astragali Radix（AgR）	MOL000354	isorhamnetin	Death-associated protein kinase 3	O43293	DAPK3	0.017
Astragali Radix（AgR）	MOL000354	isorhamnetin	Cell division protein kinase 4	P11802	CDK4	0.017
Astragali Radix（AgR）	MOL000354	isorhamnetin	Cell division protein kinase 7	P50613	CDK7	0.017
Astragali Radix（AgR）	MOL000354	isorhamnetin	Cell division protein kinase 9	P50750	CDK9	0.017
Astragali Radix（AgR）	MOL000354	isorhamnetin	Cell division protein kinase 6	Q00534	CDK6	0.017
Astragali Radix（AgR）	MOL000354	isorhamnetin	Serine/threonine-protein kinase 17B	O94768	STK17B	0.021
Astragali Radix（AgR）	MOL000354	isorhamnetin	ATP synthase subunit gamma, mitochondrial	P36542	ATP5F1C	0.021
Astragali Radix（AgR）	MOL000354	isorhamnetin	Neuronal acetylcholine receptor subunit alpha-2	Q15822	CHRNA2	0.021
Astragali Radix（AgR）	MOL000354	isorhamnetin	Solute carrier family 22 member 6	Q4U2R8	SLC22A6	0.021

Astragali Radix（AgR）	MOL000354	isorhamnetin	UDP-glucuronosyltransferase 3A1	Q6NUS8	UGT3A1	0.021
Astragali Radix（AgR）	MOL000354	isorhamnetin	Solute carrier family 22 member 8	Q8TCC7	SLC22A8	0.021
Astragali Radix（AgR）	MOL000354	isorhamnetin	Solute carrier family 22 member 11	Q9NSA0	SLC22A11	0.021
Astragali Radix（AgR）	MOL000354	isorhamnetin	Potassium channel subfamily K member 1	O00180	KCNK1	0.022
Astragali Radix（AgR）	MOL000354	isorhamnetin	Beta-1 adrenergic receptor	P08588	ADRB1	0.022
Astragali Radix（AgR）	MOL000354	isorhamnetin	Tyrosine 3-monooxygenase	P07101	TH	0.023
Astragali Radix（AgR）	MOL000354	isorhamnetin	Gamma-aminobutyric-acid receptor subunit alpha-1	P14867	GABRA1	0.023
Astragali Radix（AgR）	MOL000354	isorhamnetin	Amiloride-sensitive sodium channel subunit alpha	P37088	SCNN1A	0.023
Astragali Radix（AgR）	MOL000354	isorhamnetin	Gamma-aminobutyric-acid receptor subunit alpha-2	P47869	GABRA2	0.023
Astragali Radix（AgR）	MOL000354	isorhamnetin	Amiloride-sensitive sodium channel subunit beta	P51168	SCNN1B	0.023
Astragali Radix（AgR）	MOL000354	isorhamnetin	Amiloride-sensitive sodium channel subunit gamma	P51170	SCNN1G	0.023
Astragali Radix（AgR）	MOL000354	isorhamnetin	Potassium voltage-gated channel subfamily H member 6	Q9H252	KCNH6	0.023
Astragali Radix（AgR）	MOL000354	isorhamnetin	Tyrosyl-tRNA synthetase, mitochondrial	Q9Y2Z4	YARS2	0.023
Astragali Radix（AgR）	MOL000354	isorhamnetin	Muscarinic acetylcholine receptor M5	P08912	CHRM5	0.024
Astragali Radix（AgR）	MOL000354	isorhamnetin	Amine oxidase [flavin-containing] A	P21397	MAOA	0.024
Astragali Radix（AgR）	MOL000354	isorhamnetin	Histamine H1 receptor	P35367	HRH1	0.024
Astragali Radix（AgR）	MOL000354	isorhamnetin	Synaptic vesicular amine transporter	Q05940	SLC18A2	0.024
Astragali Radix（AgR）	MOL000354	isorhamnetin	Tubulin alpha-6 chain	Q9BQE3	TUBA1C	0.025
Astragali Radix（AgR）	MOL000354	isorhamnetin	Tubulin beta-2B chain	Q9BVA1	TUBB2B	0.025
Astragali Radix（AgR）	MOL000354	isorhamnetin	Stathmin-4	Q9H169	STMN4	0.025
Astragali Radix（AgR）	MOL000354	isorhamnetin	Retinal rod rhodopsin-sensitive cGMP 3',5'-cyclic phosphodiesterase subunit gamma	P18545	PDE6G	0.026
Astragali Radix（AgR）	MOL000354	isorhamnetin	5-hydroxytryptamine 3 receptor	P46098	HTR3A	0.026
Astragali Radix（AgR）	MOL000354	isorhamnetin	Prostaglandin reductase 1	Q14914	PTGR1	0.026
Astragali Radix（AgR）	MOL000354	isorhamnetin	Adenosine A2a receptor	P29274	ADORA2A	0.027
Astragali Radix（AgR）	MOL000354	isorhamnetin	Adenosine A1 receptor	P30542	ADORA1	0.027
Astragali Radix（AgR）	MOL000354	isorhamnetin	cAMP-specific 3',5'-cyclic phosphodiesterase 4C	Q08493	PDE4C	0.027
Astragali Radix（AgR）	MOL000354	isorhamnetin	cGMP-inhibited 3',5'-cyclic phosphodiesterase A	Q14432	PDE3A	0.027
Astragali Radix（AgR）	MOL000354	isorhamnetin	cAMP and cAMP-inhibited cGMP 3',5'-cyclic phosphodiesterase 10A	Q9Y233	PDE10A	0.027
Astragali Radix（AgR）	MOL000354	isorhamnetin	Carbonic anhydrase 4	P22748	CA4	0.028
Astragali Radix（AgR）	MOL000354	isorhamnetin	Kappa-type opioid receptor	P41145	OPRK1	0.028
Astragali Radix（AgR）	MOL000354	isorhamnetin	Sodium/potassium-transporting ATPase gamma chain	P54710	FXYD2	0.028
Astragali Radix（AgR）	MOL000354	isorhamnetin	Calcium/calmodulin-dependent protein kinase type II subunit delta	Q13557	CAMK2D	0.03
Astragali Radix（AgR）	MOL000354	isorhamnetin	STE20-like serine/threonine-protein kinase	Q9H2G2	SLK	0.03
Astragali Radix（AgR）	MOL000354	isorhamnetin	Succinate dehydrogenase [ubiquinone] cytochrome b small subunit, mitochondrial	O14521	SDHD	0.033
Astragali Radix（AgR）	MOL000354	isorhamnetin	DNA topoisomerase 1	P11387	TOP1	0.033
Astragali Radix（AgR）	MOL000354	isorhamnetin	Phospholipase A2, membrane associated	P14555	PLA2G2A	0.033
Astragali Radix（AgR）	MOL000354	isorhamnetin	Peptidyl-prolyl cis-trans isomerase, mitochondrial	P30405	PPIF	0.033
Astragali Radix（AgR）	MOL000354	isorhamnetin	Casein kinase I isoform gamma-2	P78368	CSNK1G2	0.033
Astragali Radix（AgR）	MOL000354	isorhamnetin	Lactoylglutathione lyase	Q04760	GLO1	0.033
Astragali Radix（AgR）	MOL000354	isorhamnetin	Prostaglandin reductase 2	Q8N8N7	PTGR2	0.033
Astragali Radix（AgR）	MOL000354	isorhamnetin	Serine/threonine-protein kinase haspin	Q8TF76	HASPIN	0.033
Astragali Radix（AgR）	MOL000354	isorhamnetin	DNA topoisomerase I, mitochondrial	Q969P6	TOP1MT	0.033
Astragali Radix（AgR）	MOL000354	isorhamnetin	Ig kappa chain C region	P01834	IGKC	0.034
Astragali Radix（AgR）	MOL000354	isorhamnetin	Ig gamma-1 chain C region	P01857	IGHG1	0.034
Astragali Radix（AgR）	MOL000354	isorhamnetin	Proto-oncogene tyrosine-protein kinase LCK	P06239	LCK	0.034
Astragali Radix（AgR）	MOL000354	isorhamnetin	Ribosyldihydronicotinamide dehydrogenase [quinone]	P16083	NQO2	0.034
Astragali Radix（AgR）	MOL000354	isorhamnetin	Mitogen-activated protein kinase 8	P45983	MAPK8	0.034

Astragali Radix（AgR）	MOL000354	isorhamnetin	Mitogen-activated protein kinase 10	P53779	MAPK10	0.034
Astragali Radix（AgR）	MOL000354	isorhamnetin	Sodium channel protein type 5 subunit alpha	Q14524	SCN5A	0.035
Astragali Radix（AgR）	MOL000354	isorhamnetin	Glycogen synthase kinase-3 beta	P49841	GSK3B	0.036
Astragali Radix（AgR）	MOL000354	isorhamnetin	Potassium voltage-gated channel subfamily H member 2	Q12809	KCNH2	0.036
Astragali Radix（AgR）	MOL000354	isorhamnetin	Urokinase-type plasminogen activator	P00749	PLAU	0.037
Astragali Radix（AgR）	MOL000354	isorhamnetin	Macrophage migration inhibitory factor	P14174	MIF	0.037
Astragali Radix（AgR）	MOL000354	isorhamnetin	Tyrosine-protein kinase JAK2	O60674	JAK2	0.038
Astragali Radix（AgR）	MOL000354	isorhamnetin	Epidermal growth factor receptor	P00533	EGFR	0.038
Astragali Radix（AgR）	MOL000354	isorhamnetin	Tyrosine-protein kinase JAK1	P23458	JAK1	0.038
Astragali Radix（AgR）	MOL000354	isorhamnetin	Tyrosine-protein kinase JAK3	P52333	JAK3	0.038
Astragali Radix（AgR）	MOL000354	isorhamnetin	Glutamate [NMDA] receptor subunit 3A	Q8TCU5	GRIN3A	0.04
Astragali Radix（AgR）	MOL000354	isorhamnetin	Sterol O-acyltransferase 2	O75908	SOAT2	0.042
Astragali Radix（AgR）	MOL000354	isorhamnetin	DNA-(apurinic or apyrimidinic site) lyase	P27695	APEX1	0.042
Astragali Radix（AgR）	MOL000354	isorhamnetin	Sterol O-acyltransferase 1	P35610	SOAT1	0.042
Astragali Radix（AgR）	MOL000354	isorhamnetin	Mineralocorticoid receptor	P08235	NR3C2	0.043
Astragali Radix（AgR）	MOL000354	isorhamnetin	3 beta-hydroxysteroid dehydrogenase/Delta 5-->4-isomerase type II	P26439	HSD3B2	0.043
Astragali Radix（AgR）	MOL000354	isorhamnetin	Prostacyclin receptor	P43119	PTGIR	0.043
Astragali Radix（AgR）	MOL000354	isorhamnetin	Keratin, type II cytoskeletal 7	P08729	KRT7	0.044
Astragali Radix（AgR）	MOL000354	isorhamnetin	Platelet glycoprotein IX	P14770	GP9	0.044
Astragali Radix（AgR）	MOL000354	isorhamnetin	3-oxo-5-alpha-steroid 4-dehydrogenase 1	P18405	SRD5A1	0.044
Astragali Radix（AgR）	MOL000354	isorhamnetin	Acetylcholinesterase	P22303	ACHE	0.044
Astragali Radix（AgR）	MOL000354	isorhamnetin	Gonadotropin-releasing hormone receptor	P30968	GNRHR	0.044
Astragali Radix（AgR）	MOL000354	isorhamnetin	Gonadotropin-releasing hormone II receptor	Q96P88	GNRHR2	0.044
Astragali Radix（AgR）	MOL000354	isorhamnetin	Microtubule-associated protein 2	P11137	MAP2	0.045
Astragali Radix（AgR）	MOL000354	isorhamnetin	Delta-type opioid receptor	P41143	OPRD1	0.045
Astragali Radix（AgR）	MOL000354	isorhamnetin	Microtubule-associated protein 1A	P78559	MAP1A	0.045
Astragali Radix（AgR）	MOL000354	isorhamnetin	Peroxisome proliferator-activated receptor alpha	Q07869	PPARA	0.046
Astragali Radix（AgR）	MOL000354	isorhamnetin	ATP synthase subunit beta, mitochondrial	P06576	ATP5F1B	0.047
Astragali Radix（AgR）	MOL000354	isorhamnetin	Tyrosine-protein kinase HCK	P08631	HCK	0.047
Astragali Radix（AgR）	MOL000354	isorhamnetin	ATP synthase subunit alpha, mitochondrial	P25705	ATP5F1A	0.047
Astragali Radix（AgR）	MOL000354	isorhamnetin	Phosphatidylinositol-4,5-bisphosphate 3-kinase catalytic subunit gamma isoform	P48736	PIK3CG	0.047
Astragali Radix（AgR）	MOL000354	isorhamnetin	Protein tyrosine kinase 2 beta	Q14289	PTK2B	0.047
Astragali Radix（AgR）	MOL000354	isorhamnetin	Alpha-1D adrenergic receptor	P25100	ADRA1D	0.048
Astragali Radix（AgR）	MOL000354	isorhamnetin	Tyrosyl-tRNA synthetase, cytoplasmic	P54577	YARS	0.049
Astragali Radix（AgR）	MOL000354	isorhamnetin	MAP kinase-activated protein kinase 2	P49137	MAPKAPK2	0.051
Astragali Radix（AgR）	MOL000354	isorhamnetin	Estrogen-related receptor gamma	P62508	ESRRG	0.051
Astragali Radix（AgR）	MOL000354	isorhamnetin	Tubulin alpha-3 chain	Q71U36	TUBA1A	0.053
Astragali Radix（AgR）	MOL000354	isorhamnetin	Glycogen phosphorylase, muscle form	P11217	PYGM	0.054
Astragali Radix（AgR）	MOL000354	isorhamnetin	cGMP-specific 3',5'-cyclic phosphodiesterase	O76074	PDE5A	0.057
Astragali Radix（AgR）	MOL000354	isorhamnetin	Carbonic anhydrase 1	P00915	CA1	0.06
Astragali Radix（AgR）	MOL000354	isorhamnetin	Alpha-2A adrenergic receptor	P08913	ADRA2A	0.061
Astragali Radix（AgR）	MOL000354	isorhamnetin	Proto-oncogene serine/threonine-protein kinase Pim-1	P11309	PIM1	0.061
Astragali Radix（AgR）	MOL000354	isorhamnetin	5-hydroxytryptamine 1D receptor	P28221	HTR1D	0.061
Astragali Radix（AgR）	MOL000354	isorhamnetin	5-hydroxytryptamine 1B receptor	P28222	HTR1B	0.061
Astragali Radix（AgR）	MOL000354	isorhamnetin	Hepatocyte growth factor receptor	P08581	MET	0.063
Astragali Radix（AgR）	MOL000354	isorhamnetin	Oxysterols receptor LXR-alpha	Q13133	NR1H3	0.064
Astragali Radix（AgR）	MOL000354	isorhamnetin	Phospholipase A2	P04054	PLA2G1B	0.065

Astragali Radix（AgR）	MOL000354	isorhamnetin	Nitric oxide synthase, inducible	P35228	NOS2	0.065
Astragali Radix（AgR）	MOL000354	isorhamnetin	Egl nine homolog 1	Q9GZT9	EGLN1	0.065
Astragali Radix（AgR）	MOL000354	isorhamnetin	cAMP-dependent protein kinase inhibitor alpha	P61925	PKIA	0.066
Astragali Radix（AgR）	MOL000354	isorhamnetin	Sodium-dependent dopamine transporter	Q01959	SLC6A3	0.066
Astragali Radix（AgR）	MOL000354	isorhamnetin	Membrane copper amine oxidase	Q16853	AOC3	0.066
Astragali Radix（AgR）	MOL000354	isorhamnetin	Muscarinic acetylcholine receptor M4	P08173	CHRM4	0.067
Astragali Radix（AgR）	MOL000354	isorhamnetin	Aldo-keto reductase family 1 member C1	Q04828	AKR1C1	0.067
Astragali Radix（AgR）	MOL000354	isorhamnetin	Cytochrome P450 19A1	P11511	CYP19A1	0.068
Astragali Radix（AgR）	MOL000354	isorhamnetin	Sodium-dependent noradrenaline transporter	P23975	SLC6A2	0.068
Astragali Radix（AgR）	MOL000354	isorhamnetin	Calcium/calmodulin-dependent protein kinase type II alpha chain	Q9UQM7	CAMK2A	0.068
Astragali Radix（AgR）	MOL000354	isorhamnetin	Inhibitor of nuclear factor kappa-B kinase subunit alpha	O15111	CHUK	0.069
Astragali Radix（AgR）	MOL000354	isorhamnetin	Ig kappa chain V-II region RPMI 6410	P06310	IGKV2-30	0.069
Astragali Radix（AgR）	MOL000354	isorhamnetin	Arachidonate 5-lipoxygenase	P09917	ALOX5	0.069
Astragali Radix（AgR）	MOL000354	isorhamnetin	Leukotriene A-4 hydrolase	P09960	LTA4H	0.069
Astragali Radix（AgR）	MOL000354	isorhamnetin	Tyrosine-protein phosphatase non-receptor type 1	P18031	PTPN1	0.069
Astragali Radix（AgR）	MOL000354	isorhamnetin	Peroxisome proliferator-activated receptor gamma	P37231	PPARG	0.069
Astragali Radix（AgR）	MOL000354	isorhamnetin	Group IIE secretory phospholipase A2	Q9NZK7	PLA2G2E	0.069
Astragali Radix（AgR）	MOL000354	isorhamnetin	Calmodulin	P62158		0.07
Astragali Radix（AgR）	MOL000354	isorhamnetin	Thymidylate synthase	P04818	TYMS	0.071
Astragali Radix（AgR）	MOL000354	isorhamnetin	Casein kinase II subunit alpha	P68400	CSNK2A1	0.071
Astragali Radix（AgR）	MOL000354	isorhamnetin	Solute carrier family 12 member 1	Q13621	SLC12A1	0.071
Astragali Radix（AgR）	MOL000354	isorhamnetin	C-jun-amino-terminal kinase-interacting protein 1	Q9UQF2	MAPK8IP1	0.071
Astragali Radix（AgR）	MOL000354	isorhamnetin	Glucocorticoid receptor	P04150	NR3C1	0.072
Astragali Radix（AgR）	MOL000354	isorhamnetin	S-methyl-5-thioadenosine phosphorylase	Q13126	MTAP	0.072
Astragali Radix（AgR）	MOL000354	isorhamnetin	Melatonin receptor type 1A	P48039	MTNR1A	0.073
Astragali Radix（AgR）	MOL000354	isorhamnetin	Dihydrofolate reductase	P00374	DHFR	0.074
Astragali Radix（AgR）	MOL000354	isorhamnetin	Neuropeptide Y	P01303	NPY	0.075
Astragali Radix（AgR）	MOL000354	isorhamnetin	D(4) dopamine receptor	P21917	DRD4	0.075
Astragali Radix（AgR）	MOL000354	isorhamnetin	Alpha-1B adrenergic receptor	P35368	ADRA1B	0.075
Astragali Radix（AgR）	MOL000354	isorhamnetin	D-HSCDK2	O75100	CA11	0.076
Astragali Radix（AgR）	MOL000354	isorhamnetin	Proto-oncogene tyrosine-protein kinase Src	P12931	SRC	0.077
Astragali Radix（AgR）	MOL000354	isorhamnetin	Fibroblast growth factor receptor 2	P21802	FGFR2	0.077
Astragali Radix（AgR）	MOL000354	isorhamnetin	Interferon gamma	P01579	IFNG	0.081
Astragali Radix（AgR）	MOL000354	isorhamnetin	Muscarinic acetylcholine receptor M3	P20309	CHRM3	0.081
Astragali Radix（AgR）	MOL000354	isorhamnetin	Toll-like receptor 7	Q9NYK1	TLR7	0.081
Astragali Radix（AgR）	MOL000354	isorhamnetin	Estradiol 17-beta-dehydrogenase 1	P14061	HSD17B1	0.082
Astragali Radix（AgR）	MOL000354	isorhamnetin	Nuclear receptor coactivator 5	Q9HCD5	NCOA5	0.082
Astragali Radix（AgR）	MOL000354	isorhamnetin	3 beta-hydroxysteroid dehydrogenase/Delta 5-->4-isomerase type I	P14060	HSD3B1	0.086
Astragali Radix（AgR）	MOL000354	isorhamnetin	Alpha-2B adrenergic receptor	P18089	ADRA2B	0.087
Astragali Radix（AgR）	MOL000354	isorhamnetin	Alpha-2C adrenergic receptor	P18825	ADRA2C	0.087
Astragali Radix（AgR）	MOL000354	isorhamnetin	D(1B) dopamine receptor	P21918	DRD5	0.087
Astragali Radix（AgR）	MOL000354	isorhamnetin	D(3) dopamine receptor	P35462	DRD3	0.087
Astragali Radix（AgR）	MOL000354	isorhamnetin	DNA polymerase kappa	Q9UBT6	POLK	0.087
Astragali Radix（AgR）	MOL000354	isorhamnetin	5-hydroxytryptamine 2B receptor	P41595	HTR2B	0.088
Astragali Radix（AgR）	MOL000354	isorhamnetin	Carbonic anhydrase 2	P00918	CA2	0.091
Astragali Radix（AgR）	MOL000354	isorhamnetin	Androgen receptor	P10275	AR	0.091
Astragali Radix（AgR）	MOL000354	isorhamnetin	DNA topoisomerase 2-alpha	P11388	TOP2A	0.093

Astragali Radix（AgR）	MOL000354	isorhamnetin	Mu-type opioid receptor	P35372	OPRM1	0.093
Astragali Radix（AgR）	MOL000354	isorhamnetin	Peroxisome proliferator-activated receptor delta	Q03181	PPARD	0.093
Astragali Radix（AgR）	MOL000354	isorhamnetin	Muscarinic acetylcholine receptor M2	P08172	CHRM2	0.095
Astragali Radix（AgR）	MOL000354	isorhamnetin	Muscarinic acetylcholine receptor M1	P11229	CHRM1	0.095
Astragali Radix（AgR）	MOL000354	isorhamnetin	Cell division control protein 2 homolog	P06493	CDK1	0.096
Astragali Radix（AgR）	MOL000354	isorhamnetin	5-hydroxytryptamine 1A receptor	P08908	HTR1A	0.102
Astragali Radix（AgR）	MOL000354	isorhamnetin	Sodium-dependent serotonin transporter	P31645	SLC6A4	0.109
Astragali Radix（AgR）	MOL000354	isorhamnetin	Cell division protein kinase 2	P24941	CDK2	0.115
Astragali Radix（AgR）	MOL000354	isorhamnetin	Cell division protein kinase 5	Q00535	CDK5	0.116
Astragali Radix（AgR）	MOL000354	isorhamnetin	cAMP-specific 3',5'-cyclic phosphodiesterase 4B	Q07343	PDE4B	0.117
Astragali Radix（AgR）	MOL000354	isorhamnetin	D(1A) dopamine receptor	P21728	DRD1	0.126
Astragali Radix（AgR）	MOL000354	isorhamnetin	Alpha-1A adrenergic receptor	P35348	ADRA1A	0.128
Astragali Radix（AgR）	MOL000354	isorhamnetin	5-hydroxytryptamine 2A receptor	P28223	HTR2A	0.13
Astragali Radix（AgR）	MOL000354	isorhamnetin	Coagulation factor VII	P08709	F7	0.138
Astragali Radix（AgR）	MOL000354	isorhamnetin	5-hydroxytryptamine 2C receptor	P28335	HTR2C	0.143
Astragali Radix（AgR）	MOL000354	isorhamnetin	RAC-alpha serine/threonine-protein kinase	P31749	AKT1	0.146
Astragali Radix（AgR）	MOL000354	isorhamnetin	cAMP-specific 3',5'-cyclic phosphodiesterase 4A	P27815	PDE4A	0.147
Astragali Radix（AgR）	MOL000354	isorhamnetin	D(2) dopamine receptor	P14416	DRD2	0.153
Astragali Radix（AgR）	MOL000354	isorhamnetin	Hemoglobin subunit alpha	P69905	HBA1	0.162
Astragali Radix（AgR）	MOL000354	isorhamnetin	Trypsin-1	P07477	PRSS1	0.197
Astragali Radix（AgR）	MOL000354	isorhamnetin	Prothrombin	P00734	F2	0.199
Astragali Radix（AgR）	MOL000354	isorhamnetin	Cyclin-A2	P20248	CCNA2	0.205
Astragali Radix（AgR）	MOL000354	isorhamnetin	cAMP-dependent protein kinase catalytic subunit alpha	P17612	PRKACA	0.217
Astragali Radix（AgR）	MOL000354	isorhamnetin	Prostaglandin G/H synthase 1	P23219	PTGS1	0.324
Astragali Radix（AgR）	MOL000354	isorhamnetin	Nuclear receptor coactivator 1	Q15788	NCOA1	0.357
Astragali Radix（AgR）	MOL000354	isorhamnetin	Progesterone receptor	P06401	PGR	0.372
Astragali Radix（AgR）	MOL000354	isorhamnetin	Estrogen receptor beta	Q92731	ESR2	0.475
Astragali Radix（AgR）	MOL000354	isorhamnetin	Prostaglandin G/H synthase 2	P35354	PTGS2	0.562
Astragali Radix（AgR）	MOL000354	isorhamnetin	Estrogen receptor	P03372	ESR1	1
Astragali Radix（AgR）	MOL000354	isorhamnetin	Neutrophil cytosol factor 1	P14598	NCF1	Validated
Astragali Radix（AgR）	MOL000354	isorhamnetin	Nitric oxide synthase, inducible	P35228	NOS2	Validated
Astragali Radix（AgR）	MOL000354	isorhamnetin	Xanthine dehydrogenase/oxidase	P47989	XDH	Validated
Astragali Radix（AgR）	MOL000354	isorhamnetin	Oxidized low-density lipoprotein receptor 1	P78380	OLR1	Validated
Astragali Radix（AgR）	MOL000354	isorhamnetin	Transcription factor p65	Q04206	RELA	Validated
Astragali Radix（AgR）	MOL000356	lupeol	Superoxide dismutase [Cu-Zn]	P00441	SOD1	Validated
Astragali Radix（AgR）	MOL000356	lupeol	Apoptotic protease-activating factor 1	O14727	APAF1	Validated
Astragali Radix（AgR）	MOL000356	lupeol	Estrogen receptor	P03372	ESR1	Validated
Astragali Radix（AgR）	MOL000356	lupeol	Cell division control protein 2 homolog	P06493	CDC2	Validated
Astragali Radix（AgR）	MOL000356	lupeol	Apoptosis regulator Bcl-2	P10415	BCL2	Validated
Astragali Radix（AgR）	MOL000356	lupeol	G2/mitotic-specific cyclin-B1	P14635	CCNB1	Validated
Astragali Radix（AgR）	MOL000356	lupeol	M-phase inducer phosphatase 3	P30307	CDC25C	Validated
Astragali Radix（AgR）	MOL000356	lupeol	14-3-3 protein sigma	P31947	SFN	Validated
Astragali Radix（AgR）	MOL000356	lupeol	Caspase-3	P42574	CASP3	Validated
Astragali Radix（AgR）	MOL000356	lupeol	Carnitine O-acetyltransferase	P43155	CRAT	Validated
Astragali Radix（AgR）	MOL000356	lupeol	Serine/threonine-protein kinase PLK1	P53350	PLK1	Validated
Astragali Radix（AgR）	MOL000356	lupeol	Caspase-9	P55211	CASP9	Validated
Astragali Radix（AgR）	MOL000356	lupeol	Apoptosis regulator BAX	Q07812	BAX	Validated

Astragali Radix（AgR）	MOL000371	3,9-di-O-methylnissolin	Vascular endothelial growth factor	A2A2V4	VEGFA	0.01
Astragali Radix（AgR）	MOL000371	3,9-di-O-methylnissolin	Tumor necrosis factor ligand superfamily member 11	O14788	TNFSF11	0.01
Astragali Radix（AgR）	MOL000371	3,9-di-O-methylnissolin	3-phosphoinositide-dependent protein kinase 1	O15530	PDPK1	0.01
Astragali Radix（AgR）	MOL000371	3,9-di-O-methylnissolin	Cytochrome b	P00156	MT-CYB	0.01
Astragali Radix（AgR）	MOL000371	3,9-di-O-methylnissolin	Proto-oncogene tyrosine-protein kinase LCK	P06239	LCK	0.01
Astragali Radix（AgR）	MOL000371	3,9-di-O-methylnissolin	Cytochrome c1, heme protein, mitochondrial	P08574	CYC1	0.01
Astragali Radix（AgR）	MOL000371	3,9-di-O-methylnissolin	Interleukin-3	P08700	IL3	0.01
Astragali Radix（AgR）	MOL000371	3,9-di-O-methylnissolin	Tyrosinase	P14679	TYR	0.01
Astragali Radix（AgR）	MOL000371	3,9-di-O-methylnissolin	3-oxo-5-alpha-steroid 4-dehydrogenase 1	P18405	SRD5A1	0.01
Astragali Radix（AgR）	MOL000371	3,9-di-O-methylnissolin	Phosphatidylinositol 3-kinase regulatory subunit alpha	P27986	PIK3R1	0.01
Astragali Radix（AgR）	MOL000371	3,9-di-O-methylnissolin	Cadherin-5	P33151	CDH5	0.01
Astragali Radix（AgR）	MOL000371	3,9-di-O-methylnissolin	Tyrosine-protein kinase CSK	P41240	CSK	0.01
Astragali Radix（AgR）	MOL000371	3,9-di-O-methylnissolin	Tyrosine-protein kinase ZAP-70	P43403	ZAP70	0.01
Astragali Radix（AgR）	MOL000371	3,9-di-O-methylnissolin	Tyrosine-protein kinase SYK	P43405	SYK	0.01
Astragali Radix（AgR）	MOL000371	3,9-di-O-methylnissolin	Cytochrome b-c1 complex subunit Rieske, mitochondrial	P47985	UQCRFS1	0.01
Astragali Radix（AgR）	MOL000371	3,9-di-O-methylnissolin	Glycogen synthase kinase-3 beta	P49841	GSK3B	0.01
Astragali Radix（AgR）	MOL000371	3,9-di-O-methylnissolin	3-oxo-5-beta-steroid 4-dehydrogenase	P51857	AKR1D1	0.01
Astragali Radix（AgR）	MOL000371	3,9-di-O-methylnissolin	Serine/threonine-protein kinase PLK1	P53350	PLK1	0.01
Astragali Radix（AgR）	MOL000371	3,9-di-O-methylnissolin	Protein S100-A12	P80511	S100A12	0.01
Astragali Radix（AgR）	MOL000371	3,9-di-O-methylnissolin	Protein kinase C theta type	Q04759	PRKCQ	0.01
Astragali Radix（AgR）	MOL000371	3,9-di-O-methylnissolin	Tyrosine-protein kinase ITK/TSK	Q08881	ITK	0.01
Astragali Radix（AgR）	MOL000371	3,9-di-O-methylnissolin	Neuronal acetylcholine receptor subunit alpha-2	Q15822	CHRNA2	0.01
Astragali Radix（AgR）	MOL000371	3,9-di-O-methylnissolin	Protein S100-A13	Q99584	S100A13	0.01
Astragali Radix（AgR）	MOL000371	3,9-di-O-methylnissolin	Serine/threonine-protein kinase 17B	O94768	STK17B	0.011
Astragali Radix（AgR）	MOL000371	3,9-di-O-methylnissolin	ATP synthase subunit gamma, mitochondrial	P36542	ATP5F1C	0.011
Astragali Radix（AgR）	MOL000371	3,9-di-O-methylnissolin	Amiloride-sensitive sodium channel subunit alpha	P37088	SCNN1A	0.011
Astragali Radix（AgR）	MOL000371	3,9-di-O-methylnissolin	Nociceptin receptor	P41146	OPRL1	0.011
Astragali Radix（AgR）	MOL000371	3,9-di-O-methylnissolin	Amiloride-sensitive sodium channel subunit beta	P51168	SCNN1B	0.011
Astragali Radix（AgR）	MOL000371	3,9-di-O-methylnissolin	Amiloride-sensitive sodium channel subunit gamma	P51170	SCNN1G	0.011
Astragali Radix（AgR）	MOL000371	3,9-di-O-methylnissolin	UDP-glucuronosyltransferase 3A1	Q6NUS8	UGT3A1	0.011
Astragali Radix（AgR）	MOL000371	3,9-di-O-methylnissolin	Dihydrofolate reductase	P00374	DHFR	0.012
Astragali Radix（AgR）	MOL000371	3,9-di-O-methylnissolin	T-cell receptor alpha chain C region	P01848	TRAC	0.012
Astragali Radix（AgR）	MOL000371	3,9-di-O-methylnissolin	T-cell receptor beta chain C region	P01850	TRBC1	0.012
Astragali Radix（AgR）	MOL000371	3,9-di-O-methylnissolin	Muscarinic acetylcholine receptor M5	P08912	CHRM5	0.012
Astragali Radix（AgR）	MOL000371	3,9-di-O-methylnissolin	Retinoic acid receptor alpha	P10276	RARA	0.012
Astragali Radix（AgR）	MOL000371	3,9-di-O-methylnissolin	Retinoic acid receptor beta	P10826	RARB	0.012
Astragali Radix（AgR）	MOL000371	3,9-di-O-methylnissolin	Retinoic acid receptor gamma-1	P13631	RARG	0.012
Astragali Radix（AgR）	MOL000371	3,9-di-O-methylnissolin	Amine oxidase [flavin-containing] A	P21397	MAOA	0.012
Astragali Radix（AgR）	MOL000371	3,9-di-O-methylnissolin	D(4) dopamine receptor	P21917	DRD4	0.012
Astragali Radix（AgR）	MOL000371	3,9-di-O-methylnissolin	5-hydroxytryptamine 1D receptor	P28221	HTR1D	0.012
Astragali Radix（AgR）	MOL000371	3,9-di-O-methylnissolin	Histamine H1 receptor	P35367	HRH1	0.012
Astragali Radix（AgR）	MOL000371	3,9-di-O-methylnissolin	D(3) dopamine receptor	P35462	DRD3	0.012
Astragali Radix（AgR）	MOL000371	3,9-di-O-methylnissolin	Retinoic acid receptor RXR-gamma	P48443	RXRG	0.012
Astragali Radix（AgR）	MOL000371	3,9-di-O-methylnissolin	Beta-2-microglobulin	P61769	B2M	0.012
Astragali Radix（AgR）	MOL000371	3,9-di-O-methylnissolin	Potassium voltage-gated channel subfamily H member 2	Q12809	KCNH2	0.012
Astragali Radix（AgR）	MOL000371	3,9-di-O-methylnissolin	Potassium voltage-gated channel subfamily H member 6	Q9H252	KCNH6	0.012
Astragali Radix（AgR）	MOL000371	3,9-di-O-methylnissolin	Pepsin A	P00790	REN	0.013

Astragali Radix（AgR）	MOL000371	3,9-di-O-methylnissolin	Dipeptidyl peptidase 4	P27487	DPP4	0.013
Astragali Radix（AgR）	MOL000371	3,9-di-O-methylnissolin	Bile salt sulfotransferase	Q06520	SULT2A1	0.013
Astragali Radix（AgR）	MOL000371	3,9-di-O-methylnissolin	Glycogen phosphorylase, muscle form	P11217	PYGM	0.014
Astragali Radix（AgR）	MOL000371	3,9-di-O-methylnissolin	Cell division protein kinase 4	P11802	CDK4	0.014
Astragali Radix（AgR）	MOL000371	3,9-di-O-methylnissolin	Cell division protein kinase 7	P50613	CDK7	0.014
Astragali Radix（AgR）	MOL000371	3,9-di-O-methylnissolin	Cell division protein kinase 9	P50750	CDK9	0.014
Astragali Radix（AgR）	MOL000371	3,9-di-O-methylnissolin	Tyrosine 3-monooxygenase	P07101	TH	0.015
Astragali Radix（AgR）	MOL000371	3,9-di-O-methylnissolin	Gamma-aminobutyric-acid receptor subunit beta-3	P28472	GABRB3	0.015
Astragali Radix（AgR）	MOL000371	3,9-di-O-methylnissolin	Gamma-aminobutyric-acid receptor subunit beta-2	P47870	GABRB2	0.015
Astragali Radix（AgR）	MOL000371	3,9-di-O-methylnissolin	Peptidyl-prolyl cis-trans isomerase NIMA-interacting 1	Q13526	PIN1	0.015
Astragali Radix（AgR）	MOL000371	3,9-di-O-methylnissolin	Tyrosyl-tRNA synthetase, mitochondrial	Q9Y2Z4	YARS2	0.015
Astragali Radix（AgR）	MOL000371	3,9-di-O-methylnissolin	Gamma-aminobutyric acid receptor subunit rho-3	A8MPY1	GABRR3	0.016
Astragali Radix（AgR）	MOL000371	3,9-di-O-methylnissolin	Gamma-aminobutyric acid receptor subunit pi	O00591	GABRP	0.016
Astragali Radix（AgR）	MOL000371	3,9-di-O-methylnissolin	Gamma-aminobutyric acid receptor subunit delta	O14764	GABRD	0.016
Astragali Radix（AgR）	MOL000371	3,9-di-O-methylnissolin	Gamma-aminobutyric-acid receptor subunit beta-1	P18505	GABRB1	0.016
Astragali Radix（AgR）	MOL000371	3,9-di-O-methylnissolin	Gamma-aminobutyric acid receptor subunit gamma-2	P18507	GABRG2	0.016
Astragali Radix（AgR）	MOL000371	3,9-di-O-methylnissolin	Gamma-aminobutyric-acid receptor subunit rho-1	P24046	GABRR1	0.016
Astragali Radix（AgR）	MOL000371	3,9-di-O-methylnissolin	Gamma-aminobutyric acid receptor subunit rho-2	P28476	GABRR2	0.016
Astragali Radix（AgR）	MOL000371	3,9-di-O-methylnissolin	Neuronal acetylcholine receptor subunit alpha-7	P36544	CHRNA7	0.016
Astragali Radix（AgR）	MOL000371	3,9-di-O-methylnissolin	5-hydroxytryptamine 3 receptor	P46098	HTR3A	0.016
Astragali Radix（AgR）	MOL000371	3,9-di-O-methylnissolin	Gamma-aminobutyric acid receptor subunit epsilon	P78334	GABRE	0.016
Astragali Radix（AgR）	MOL000371	3,9-di-O-methylnissolin	Gamma-aminobutyric-acid receptor subunit alpha-6	Q16445	GABRA6	0.016
Astragali Radix（AgR）	MOL000371	3,9-di-O-methylnissolin	Gamma-aminobutyric acid receptor subunit gamma-3	Q99928	GABRG3	0.016
Astragali Radix（AgR）	MOL000371	3,9-di-O-methylnissolin	Potassium channel subfamily K member 1	O00180	KCNK1	0.017
Astragali Radix（AgR）	MOL000371	3,9-di-O-methylnissolin	Aryl hydrocarbon receptor	P35869	AHR	0.017
Astragali Radix（AgR）	MOL000371	3,9-di-O-methylnissolin	Dihydroorotate dehydrogenase, mitochondrial	Q02127	DHODH	0.017
Astragali Radix（AgR）	MOL000371	3,9-di-O-methylnissolin	DNA topoisomerase 1	P11387	TOP1	0.018
Astragali Radix（AgR）	MOL000371	3,9-di-O-methylnissolin	Alcohol dehydrogenase [NADP+]	P14550	AKR1A1	0.018
Astragali Radix（AgR）	MOL000371	3,9-di-O-methylnissolin	Aldose reductase	P15121	AKR1B1	0.018
Astragali Radix（AgR）	MOL000371	3,9-di-O-methylnissolin	Methionine aminopeptidase 1	P53582	METAP1	0.018
Astragali Radix（AgR）	MOL000371	3,9-di-O-methylnissolin	DNA topoisomerase I, mitochondrial	Q969P6	TOP1MT	0.018
Astragali Radix（AgR）	MOL000371	3,9-di-O-methylnissolin	Tumor necrosis factor	P01375	TNF	0.019
Astragali Radix（AgR）	MOL000371	3,9-di-O-methylnissolin	Nuclear factor NF-kappa-B p105 subunit	P19838	NFKB1	0.019
Astragali Radix（AgR）	MOL000371	3,9-di-O-methylnissolin	Acetylcholinesterase	P22303	ACHE	0.019
Astragali Radix（AgR）	MOL000371	3,9-di-O-methylnissolin	Tubulin beta-2C chain	P68371	TUBB4B	0.019
Astragali Radix（AgR）	MOL000371	3,9-di-O-methylnissolin	Tubulin alpha-3 chain	Q71U36	TUBA1A	0.019
Astragali Radix（AgR）	MOL000371	3,9-di-O-methylnissolin	Casein kinase I isoform gamma-3	Q9Y6M4	CSNK1G3	0.019
Astragali Radix（AgR）	MOL000371	3,9-di-O-methylnissolin	Inhibitor of nuclear factor kappa-B kinase subunit beta	O14920	IKBKB	0.02
Astragali Radix（AgR）	MOL000371	3,9-di-O-methylnissolin	Inhibitor of nuclear factor kappa-B kinase subunit alpha	O15111	CHUK	0.02
Astragali Radix（AgR）	MOL000371	3,9-di-O-methylnissolin	Lipoic acid synthetase, mitochondrial	O43766	LIAS	0.02
Astragali Radix（AgR）	MOL000371	3,9-di-O-methylnissolin	Adenosine A2a receptor	P29274	ADORA2A	0.02
Astragali Radix（AgR）	MOL000371	3,9-di-O-methylnissolin	Peptidyl-prolyl cis-trans isomerase, mitochondrial	P30405	PPIF	0.02
Astragali Radix（AgR）	MOL000371	3,9-di-O-methylnissolin	Adenosine A1 receptor	P30542	ADORA1	0.02
Astragali Radix（AgR）	MOL000371	3,9-di-O-methylnissolin	Mitogen-activated protein kinase 8	P45983	MAPK8	0.02
Astragali Radix（AgR）	MOL000371	3,9-di-O-methylnissolin	Mitogen-activated protein kinase 10	P53779	MAPK10	0.02
Astragali Radix（AgR）	MOL000371	3,9-di-O-methylnissolin	cAMP-specific 3',5'-cyclic phosphodiesterase 4C	Q08493	PDE4C	0.02
Astragali Radix（AgR）	MOL000371	3,9-di-O-methylnissolin	cGMP-inhibited 3',5'-cyclic phosphodiesterase A	Q14432	PDE3A	0.02

Astragali Radix（AgR）	MOL000371	3,9-di-O-methylnissolin	Cystine/glutamate transporter	Q9UPY5	SLC7A11	0.02
Astragali Radix（AgR）	MOL000371	3,9-di-O-methylnissolin	cAMP and cAMP-inhibited cGMP 3',5'-cyclic phosphodiesterase 10A	Q9Y233	PDE10A	0.02
Astragali Radix（AgR）	MOL000371	3,9-di-O-methylnissolin	Lipoyltransferase 1, mitochondrial	Q9Y234	LIPT1	0.02
Astragali Radix（AgR）	MOL000371	3,9-di-O-methylnissolin	Lactoylglutathione lyase	Q04760	GLO1	0.021
Astragali Radix（AgR）	MOL000371	3,9-di-O-methylnissolin	Prostaglandin reductase 2	Q8N8N7	PTGR2	0.021
Astragali Radix（AgR）	MOL000371	3,9-di-O-methylnissolin	Tyrosine-protein kinase JAK2	O60674	JAK2	0.022
Astragali Radix（AgR）	MOL000371	3,9-di-O-methylnissolin	Muscarinic acetylcholine receptor M2	P08172	CHRM2	0.022
Astragali Radix（AgR）	MOL000371	3,9-di-O-methylnissolin	Muscarinic acetylcholine receptor M4	P08173	CHRM4	0.022
Astragali Radix（AgR）	MOL000371	3,9-di-O-methylnissolin	D(1B) dopamine receptor	P21918	DRD5	0.022
Astragali Radix（AgR）	MOL000371	3,9-di-O-methylnissolin	Tyrosine-protein kinase JAK1	P23458	JAK1	0.022
Astragali Radix（AgR）	MOL000371	3,9-di-O-methylnissolin	Sodium-dependent noradrenaline transporter	P23975	SLC6A2	0.022
Astragali Radix（AgR）	MOL000371	3,9-di-O-methylnissolin	Alpha-1D adrenergic receptor	P25100	ADRA1D	0.022
Astragali Radix（AgR）	MOL000371	3,9-di-O-methylnissolin	5-hydroxytryptamine 1B receptor	P28222	HTR1B	0.022
Astragali Radix（AgR）	MOL000371	3,9-di-O-methylnissolin	5-hydroxytryptamine 2B receptor	P41595	HTR2B	0.022
Astragali Radix（AgR）	MOL000371	3,9-di-O-methylnissolin	Tyrosine-protein kinase JAK3	P52333	JAK3	0.022
Astragali Radix（AgR）	MOL000371	3,9-di-O-methylnissolin	Myeloperoxidase	P05164	MPO	0.023
Astragali Radix（AgR）	MOL000371	3,9-di-O-methylnissolin	Eosinophil peroxidase	P11678	EPX	0.023
Astragali Radix（AgR）	MOL000371	3,9-di-O-methylnissolin	Calreticulin	P27797	CALR	0.023
Astragali Radix（AgR）	MOL000371	3,9-di-O-methylnissolin	Melatonin receptor type 1B	P49286	MTNR1B	0.023
Astragali Radix（AgR）	MOL000371	3,9-di-O-methylnissolin	Nuclear receptor ROR-beta	Q92753	RORB	0.023
Astragali Radix（AgR）	MOL000371	3,9-di-O-methylnissolin	Sterol O-acyltransferase 2	O75908	SOAT2	0.024
Astragali Radix（AgR）	MOL000371	3,9-di-O-methylnissolin	Urokinase-type plasminogen activator	P00749	PLAU	0.024
Astragali Radix（AgR）	MOL000371	3,9-di-O-methylnissolin	Peroxiredoxin-5, mitochondrial	P30044	PRDX5	0.024
Astragali Radix（AgR）	MOL000371	3,9-di-O-methylnissolin	Sterol O-acyltransferase 1	P35610	SOAT1	0.024
Astragali Radix（AgR）	MOL000371	3,9-di-O-methylnissolin	Gamma-aminobutyric-acid receptor subunit alpha-4	P48169	GABRA4	0.024
Astragali Radix（AgR）	MOL000371	3,9-di-O-methylnissolin	Corticosteroid 11-beta-dehydrogenase isozyme 1	P28845	HSD11B1	0.026
Astragali Radix（AgR）	MOL000371	3,9-di-O-methylnissolin	Gonadotropin-releasing hormone receptor	P30968	GNRHR	0.027
Astragali Radix（AgR）	MOL000371	3,9-di-O-methylnissolin	3-oxo-5-alpha-steroid 4-dehydrogenase 2	P31213	SRD5A2	0.027
Astragali Radix（AgR）	MOL000371	3,9-di-O-methylnissolin	Gonadotropin-releasing hormone II receptor	Q96P88	GNRHR2	0.027
Astragali Radix（AgR）	MOL000371	3,9-di-O-methylnissolin	MAP kinase-activated protein kinase 2	P49137	MAPKAPK2	0.028
Astragali Radix（AgR）	MOL000371	3,9-di-O-methylnissolin	Tripartite motif-containing protein 13	O60858	TRIM13	0.029
Astragali Radix（AgR）	MOL000371	3,9-di-O-methylnissolin	ATP synthase subunit beta, mitochondrial	P06576	ATP5F1B	0.029
Astragali Radix（AgR）	MOL000371	3,9-di-O-methylnissolin	Microtubule-associated protein 2	P11137	MAP2	0.029
Astragali Radix（AgR）	MOL000371	3,9-di-O-methylnissolin	Proto-oncogene serine/threonine-protein kinase Pim-1	P11309	PIM1	0.029
Astragali Radix（AgR）	MOL000371	3,9-di-O-methylnissolin	3 beta-hydroxysteroid dehydrogenase/Delta 5-->4-isomerase type I	P14060	HSD3B1	0.029
Astragali Radix（AgR）	MOL000371	3,9-di-O-methylnissolin	ATP synthase subunit alpha, mitochondrial	P25705	ATP5F1A	0.029
Astragali Radix（AgR）	MOL000371	3,9-di-O-methylnissolin	3 beta-hydroxysteroid dehydrogenase/Delta 5-->4-isomerase type II	P26439	HSD3B2	0.029
Astragali Radix（AgR）	MOL000371	3,9-di-O-methylnissolin	Estrogen-related receptor gamma	P62508	ESRRG	0.029
Astragali Radix（AgR）	MOL000371	3,9-di-O-methylnissolin	Microtubule-associated protein 1A	P78559	MAP1A	0.029
Astragali Radix（AgR）	MOL000371	3,9-di-O-methylnissolin	Retinoic acid receptor RXR-alpha	P19793	RXRA	0.03
Astragali Radix（AgR）	MOL000371	3,9-di-O-methylnissolin	Nuclear receptor subfamily 1 group I member 3	Q14994	NR1I3	0.03
Astragali Radix（AgR）	MOL000371	3,9-di-O-methylnissolin	Tryptophanyl-tRNA synthetase, mitochondrial	Q9UGM6	WARS2	0.03
Astragali Radix（AgR）	MOL000371	3,9-di-O-methylnissolin	DNA topoisomerase 2-alpha	P11388	TOP2A	0.031
Astragali Radix（AgR）	MOL000371	3,9-di-O-methylnissolin	Platelet glycoprotein IX	P14770	GP9	0.031
Astragali Radix（AgR）	MOL000371	3,9-di-O-methylnissolin	Sodium-dependent serotonin transporter	P31645	SLC6A4	0.031
Astragali Radix（AgR）	MOL000371	3,9-di-O-methylnissolin	Protein tyrosine kinase 2 beta	Q14289	PTK2B	0.031
Astragali Radix（AgR）	MOL000371	3,9-di-O-methylnissolin	Alpha-2A adrenergic receptor	P08913	ADRA2A	0.032

Astragali Radix（AgR）	MOL000371	3,9-di-O-methylnissolin	Alpha-2B adrenergic receptor	P18089	ADRA2B	0.032
Astragali Radix（AgR）	MOL000371	3,9-di-O-methylnissolin	Alpha-2C adrenergic receptor	P18825	ADRA2C	0.032
Astragali Radix（AgR）	MOL000371	3,9-di-O-methylnissolin	5-hydroxytryptamine 2C receptor	P28335	HTR2C	0.032
Astragali Radix（AgR）	MOL000371	3,9-di-O-methylnissolin	Estradiol 17-beta-dehydrogenase 1	P14061	HSD17B1	0.033
Astragali Radix（AgR）	MOL000371	3,9-di-O-methylnissolin	Retinoic acid receptor RXR-beta	P28702	RXRB	0.034
Astragali Radix（AgR）	MOL000371	3,9-di-O-methylnissolin	D-HSCDK2	O75100	CA11	0.035
Astragali Radix（AgR）	MOL000371	3,9-di-O-methylnissolin	Epidermal growth factor receptor	P00533	EGFR	0.035
Astragali Radix（AgR）	MOL000371	3,9-di-O-methylnissolin	Cell division protein kinase 6	Q00534	CDK6	0.035
Astragali Radix（AgR）	MOL000371	3,9-di-O-methylnissolin	Tyrosyl-tRNA synthetase, cytoplasmic	P54577	YARS	0.037
Astragali Radix（AgR）	MOL000371	3,9-di-O-methylnissolin	Nuclear receptor coactivator 2	Q15596	NCOA2	0.037
Astragali Radix（AgR）	MOL000371	3,9-di-O-methylnissolin	Gamma-aminobutyric-acid receptor subunit alpha-3	P34903	GABRA3	0.04
Astragali Radix（AgR）	MOL000371	3,9-di-O-methylnissolin	Muscarinic acetylcholine receptor M1	P11229	CHRM1	0.041
Astragali Radix（AgR）	MOL000371	3,9-di-O-methylnissolin	Muscarinic acetylcholine receptor M3	P20309	CHRM3	0.041
Astragali Radix（AgR）	MOL000371	3,9-di-O-methylnissolin	Alpha-1B adrenergic receptor	P35368	ADRA1B	0.041
Astragali Radix（AgR）	MOL000371	3,9-di-O-methylnissolin	Thiamin pyrophosphokinase 1	Q9H3S4	TPK1	0.042
Astragali Radix（AgR）	MOL000371	3,9-di-O-methylnissolin	Ig kappa chain V-II region RPMI 6410	P06310	IGKV2-30	0.043
Astragali Radix（AgR）	MOL000371	3,9-di-O-methylnissolin	Thyroid hormone receptor alpha	P10827	THRA	0.043
Astragali Radix（AgR）	MOL000371	3,9-di-O-methylnissolin	Gamma-aminobutyric acid type B receptor, subunit 1	Q9UBS5	GABBR1	0.043
Astragali Radix（AgR）	MOL000371	3,9-di-O-methylnissolin	Thyroid hormone receptor beta-1	P10828	THRB	0.044
Astragali Radix（AgR）	MOL000371	3,9-di-O-methylnissolin	Egl nine homolog 1	Q9GZT9	EGLN1	0.044
Astragali Radix（AgR）	MOL000371	3,9-di-O-methylnissolin	Toll-like receptor 7	Q9NYK1	TLR7	0.044
Astragali Radix（AgR）	MOL000371	3,9-di-O-methylnissolin	Hepatocyte growth factor receptor	P08581	MET	0.045
Astragali Radix（AgR）	MOL000371	3,9-di-O-methylnissolin	5-hydroxytryptamine 1A receptor	P08908	HTR1A	0.045
Astragali Radix（AgR）	MOL000371	3,9-di-O-methylnissolin	Arachidonate 5-lipoxygenase	P09917	ALOX5	0.045
Astragali Radix（AgR）	MOL000371	3,9-di-O-methylnissolin	Leukotriene A-4 hydrolase	P09960	LTA4H	0.045
Astragali Radix（AgR）	MOL000371	3,9-di-O-methylnissolin	Fibroblast growth factor receptor 2	P21802	FGFR2	0.045
Astragali Radix（AgR）	MOL000371	3,9-di-O-methylnissolin	Activin receptor type-1	Q04771	ACVR1	0.045
Astragali Radix（AgR）	MOL000371	3,9-di-O-methylnissolin	Sodium channel protein type 5 subunit alpha	Q14524	SCN5A	0.045
Astragali Radix（AgR）	MOL000371	3,9-di-O-methylnissolin	Casein kinase I isoform gamma-1	Q9HCP0	CSNK1G1	0.045
Astragali Radix（AgR）	MOL000371	3,9-di-O-methylnissolin	Geranylgeranyl pyrophosphate synthetase	O95749	GGPS1	0.046
Astragali Radix（AgR）	MOL000371	3,9-di-O-methylnissolin	ATP-sensitive inward rectifier potassium channel 1	P48048	KCNJ1	0.046
Astragali Radix（AgR）	MOL000371	3,9-di-O-methylnissolin	Casein kinase II subunit alpha	P68400	CSNK2A1	0.046
Astragali Radix（AgR）	MOL000371	3,9-di-O-methylnissolin	S-methyl-5-thioadenosine phosphorylase	Q13126	MTAP	0.046
Astragali Radix（AgR）	MOL000371	3,9-di-O-methylnissolin	Tyrosine-protein phosphatase non-receptor type 1	P18031	PTPN1	0.047
Astragali Radix（AgR）	MOL000371	3,9-di-O-methylnissolin	Inosine-5'-monophosphate dehydrogenase 1	P20839	IMPDH1	0.047
Astragali Radix（AgR）	MOL000371	3,9-di-O-methylnissolin	Gamma-aminobutyric-acid receptor subunit alpha-5	P31644	GABRA5	0.047
Astragali Radix（AgR）	MOL000371	3,9-di-O-methylnissolin	Phosphatidylinositol-4,5-bisphosphate 3-kinase catalytic subunit gamma isoform	P48736	PIK3CG	0.047
Astragali Radix（AgR）	MOL000371	3,9-di-O-methylnissolin	Tubulin beta-1 chain	Q9H4B7	TUBB1	0.047
Astragali Radix（AgR）	MOL000371	3,9-di-O-methylnissolin	C-jun-amino-terminal kinase-interacting protein 1	Q9UQF2	MAPK8IP1	0.047
Astragali Radix（AgR）	MOL000371	3,9-di-O-methylnissolin	cGMP-specific 3',5'-cyclic phosphodiesterase	O76074	PDE5A	0.048
Astragali Radix（AgR）	MOL000371	3,9-di-O-methylnissolin	Heat shock protein HSP 90-beta	P08238	HSP90AB1	0.048
Astragali Radix（AgR）	MOL000371	3,9-di-O-methylnissolin	Cannabinoid receptor 1	P21554	CNR1	0.048
Astragali Radix（AgR）	MOL000371	3,9-di-O-methylnissolin	DNA polymerase kappa	Q9UBT6	POLK	0.048
Astragali Radix（AgR）	MOL000371	3,9-di-O-methylnissolin	Peroxisome proliferator-activated receptor alpha	Q07869	PPARA	0.049
Astragali Radix（AgR）	MOL000371	3,9-di-O-methylnissolin	Interferon gamma	P01579	IFNG	0.05
Astragali Radix（AgR）	MOL000371	3,9-di-O-methylnissolin	Ribosyldihydronicotinamide dehydrogenase [quinone]	P16083	NQO2	0.05
Astragali Radix（AgR）	MOL000371	3,9-di-O-methylnissolin	D(2) dopamine receptor	P14416	DRD2	0.051

Astragali Radix（AgR）	MOL000371	3,9-di-O-methylnissolin	5-hydroxytryptamine 2A receptor	P28223	HTR2A	0.051
Astragali Radix（AgR）	MOL000371	3,9-di-O-methylnissolin	Triosephosphate isomerase	P60174	TPI1	0.051
Astragali Radix（AgR）	MOL000371	3,9-di-O-methylnissolin	cAMP-specific 3',5'-cyclic phosphodiesterase 4D	Q08499	PDE4D	0.051
Astragali Radix（AgR）	MOL000371	3,9-di-O-methylnissolin	Nuclear receptor coactivator 5	Q9HCD5	NCOA5	0.051
Astragali Radix（AgR）	MOL000371	3,9-di-O-methylnissolin	Phospholipase A2	P04054	PLA2G1B	0.052
Astragali Radix（AgR）	MOL000371	3,9-di-O-methylnissolin	Melatonin receptor type 1A	P48039	MTNR1A	0.052
Astragali Radix（AgR）	MOL000371	3,9-di-O-methylnissolin	Calmodulin	P62158		0.052
Astragali Radix（AgR）	MOL000371	3,9-di-O-methylnissolin	Thymidylate synthase	P04818	TYMS	0.054
Astragali Radix（AgR）	MOL000371	3,9-di-O-methylnissolin	Tyrosine-protein kinase HCK	P08631	HCK	0.055
Astragali Radix（AgR）	MOL000371	3,9-di-O-methylnissolin	Cell division control protein 2 homolog	P06493	CDK1	0.056
Astragali Radix（AgR）	MOL000371	3,9-di-O-methylnissolin	Mitogen-activated protein kinase 14	Q16539	MAPK14	0.057
Astragali Radix（AgR）	MOL000371	3,9-di-O-methylnissolin	Gamma-aminobutyric-acid receptor subunit alpha-2	P47869	GABRA2	0.058
Astragali Radix（AgR）	MOL000371	3,9-di-O-methylnissolin	D(1A) dopamine receptor	P21728	DRD1	0.061
Astragali Radix（AgR）	MOL000371	3,9-di-O-methylnissolin	Alpha-1A adrenergic receptor	P35348	ADRA1A	0.061
Astragali Radix（AgR）	MOL000371	3,9-di-O-methylnissolin	Proto-oncogene tyrosine-protein kinase Src	P12931	SRC	0.067
Astragali Radix（AgR）	MOL000371	3,9-di-O-methylnissolin	Gamma-aminobutyric-acid receptor subunit alpha-1	P14867	GABRA1	0.067
Astragali Radix（AgR）	MOL000371	3,9-di-O-methylnissolin	Peroxisome proliferator-activated receptor gamma	P37231	PPARG	0.077
Astragali Radix（AgR）	MOL000371	3,9-di-O-methylnissolin	Cell division protein kinase 5	Q00535	CDK5	0.078
Astragali Radix（AgR）	MOL000371	3,9-di-O-methylnissolin	Phospholipase A2, membrane associated	P14555	PLA2G2A	0.08
Astragali Radix（AgR）	MOL000371	3,9-di-O-methylnissolin	Delta-type opioid receptor	P41143	OPRD1	0.083
Astragali Radix（AgR）	MOL000371	3,9-di-O-methylnissolin	Mineralocorticoid receptor	P08235	NR3C2	0.095
Astragali Radix（AgR）	MOL000371	3,9-di-O-methylnissolin	Androgen receptor	P10275	AR	0.095
Astragali Radix（AgR）	MOL000371	3,9-di-O-methylnissolin	Coagulation factor VII	P08709	F7	0.101
Astragali Radix（AgR）	MOL000371	3,9-di-O-methylnissolin	RAC-alpha serine/threonine-protein kinase	P31749	AKT1	0.104
Astragali Radix（AgR）	MOL000371	3,9-di-O-methylnissolin	Hydroxyacid oxidase 1	Q9UJM8	HAO1	0.105
Astragali Radix（AgR）	MOL000371	3,9-di-O-methylnissolin	cAMP-specific 3',5'-cyclic phosphodiesterase 4B	Q07343	PDE4B	0.131
Astragali Radix（AgR）	MOL000371	3,9-di-O-methylnissolin	Prostaglandin G/H synthase 1	P23219	PTGS1	0.133
Astragali Radix（AgR）	MOL000371	3,9-di-O-methylnissolin	Kappa-type opioid receptor	P41145	OPRK1	0.137
Astragali Radix（AgR）	MOL000371	3,9-di-O-methylnissolin	Prothrombin	P00734	F2	0.15
Astragali Radix（AgR）	MOL000371	3,9-di-O-methylnissolin	Cell division protein kinase 2	P24941	CDK2	0.154
Astragali Radix（AgR）	MOL000371	3,9-di-O-methylnissolin	cAMP-specific 3',5'-cyclic phosphodiesterase 4A	P27815	PDE4A	0.158
Astragali Radix（AgR）	MOL000371	3,9-di-O-methylnissolin	Hemoglobin subunit alpha	P69905	HBA1	0.174
Astragali Radix（AgR）	MOL000371	3,9-di-O-methylnissolin	Mu-type opioid receptor	P35372	OPRM1	0.186
Astragali Radix（AgR）	MOL000371	3,9-di-O-methylnissolin	Progesterone receptor	P06401	PGR	0.198
Astragali Radix（AgR）	MOL000371	3,9-di-O-methylnissolin	Nuclear receptor coactivator 1	Q15788	NCOA1	0.222
Astragali Radix（AgR）	MOL000371	3,9-di-O-methylnissolin	cAMP-dependent protein kinase catalytic subunit alpha	P17612	PRKACA	0.262
Astragali Radix（AgR）	MOL000371	3,9-di-O-methylnissolin	Trypsin-1	P07477	PRSS1	0.281
Astragali Radix（AgR）	MOL000371	3,9-di-O-methylnissolin	Estrogen receptor beta	Q92731	ESR2	0.344
Astragali Radix（AgR）	MOL000371	3,9-di-O-methylnissolin	Prostaglandin G/H synthase 2	P35354	PTGS2	0.372
Astragali Radix（AgR）	MOL000371	3,9-di-O-methylnissolin	Cyclin-A2	P20248	CCNA2	0.47
Astragali Radix（AgR）	MOL000371	3,9-di-O-methylnissolin	Estrogen receptor	P03372	ESR1	1
Astragali Radix（AgR）	MOL000378	7-O-methylisomucronulatol	Cytochrome b-c1 complex subunit 8	O14949	UQCRQ	0.011
Astragali Radix（AgR）	MOL000378	7-O-methylisomucronulatol	Cytochrome b-c1 complex subunit 10	O14957	UQCR11	0.011
Astragali Radix（AgR）	MOL000378	7-O-methylisomucronulatol	Cytochrome b	P00156	MT-CYB	0.011
Astragali Radix（AgR）	MOL000378	7-O-methylisomucronulatol	Cytochrome b-c1 complex subunit 6, mitochondrial	P07919	UQCRH	0.011
Astragali Radix（AgR）	MOL000378	7-O-methylisomucronulatol	Cytochrome c1, heme protein, mitochondrial	P08574	CYC1	0.011
Astragali Radix（AgR）	MOL000378	7-O-methylisomucronulatol	Cytochrome b-c1 complex subunit 2, mitochondrial	P22695	UQCRC2	0.011

Astragali Radix（AgR）	MOL000378	7-O-methylisomucronulatol	Ubiquinol-cytochrome-c reductase complex core protein 1, mitochondrial	P31930	UQCRC1	0.011
Astragali Radix（AgR）	MOL000378	7-O-methylisomucronulatol	Cytochrome b-c1 complex subunit Rieske, mitochondrial	P47985	UQCRFS1	0.011
Astragali Radix（AgR）	MOL000378	7-O-methylisomucronulatol	Cytochrome b-c1 complex subunit 9	Q9UDW1	UQCR10	0.011
Astragali Radix（AgR）	MOL000378	7-O-methylisomucronulatol	GTPase KRas	P01116	KRAS	0.013
Astragali Radix（AgR）	MOL000378	7-O-methylisomucronulatol	Phosducin	P20941	PDC	0.013
Astragali Radix（AgR）	MOL000378	7-O-methylisomucronulatol	Protein farnesyltransferase/geranylgeranyltransferase type I alpha subunit	P49354	FNTA	0.013
Astragali Radix（AgR）	MOL000378	7-O-methylisomucronulatol	Protein farnesyltransferase subunit beta	P49356	FNTB	0.013
Astragali Radix（AgR）	MOL000378	7-O-methylisomucronulatol	Geranylgeranyl transferase type-2 subunit beta	P53611	RABGGTB	0.013
Astragali Radix（AgR）	MOL000378	7-O-methylisomucronulatol	Guanine nucleotide-binding protein G(I)/G(S)/G(T) subunit beta-1	P62873	GNB1	0.013
Astragali Radix（AgR）	MOL000378	7-O-methylisomucronulatol	Guanine nucleotide-binding protein G(T) subunit gamma-T1	P63211	GNGT1	0.013
Astragali Radix（AgR）	MOL000378	7-O-methylisomucronulatol	Geranylgeranyl transferase type-2 subunit alpha	Q92696	RABGGTA	0.013
Astragali Radix（AgR）	MOL000378	7-O-methylisomucronulatol	Acetylcholine receptor subunit alpha	P02708	CHRNA1	0.016
Astragali Radix（AgR）	MOL000378	7-O-methylisomucronulatol	Cholinesterase	P06276	BCHE	0.016
Astragali Radix（AgR）	MOL000378	7-O-methylisomucronulatol	Acetylcholine receptor subunit gamma	P07510	CHRNG	0.016
Astragali Radix（AgR）	MOL000378	7-O-methylisomucronulatol	Muscarinic acetylcholine receptor M2	P08172	CHRM2	0.016
Astragali Radix（AgR）	MOL000378	7-O-methylisomucronulatol	Muscarinic acetylcholine receptor M4	P08173	CHRM4	0.016
Astragali Radix（AgR）	MOL000378	7-O-methylisomucronulatol	Muscarinic acetylcholine receptor M5	P08912	CHRM5	0.016
Astragali Radix（AgR）	MOL000378	7-O-methylisomucronulatol	Alpha-2A adrenergic receptor	P08913	ADRA2A	0.016
Astragali Radix（AgR）	MOL000378	7-O-methylisomucronulatol	Acetylcholine receptor subunit beta	P11230	CHRNB1	0.016
Astragali Radix（AgR）	MOL000378	7-O-methylisomucronulatol	D(2) dopamine receptor	P14416	DRD2	0.016
Astragali Radix（AgR）	MOL000378	7-O-methylisomucronulatol	Neuronal acetylcholine receptor subunit beta-2	P17787	CHRNB2	0.016
Astragali Radix（AgR）	MOL000378	7-O-methylisomucronulatol	Alpha-2B adrenergic receptor	P18089	ADRA2B	0.016
Astragali Radix（AgR）	MOL000378	7-O-methylisomucronulatol	Alpha-2C adrenergic receptor	P18825	ADRA2C	0.016
Astragali Radix（AgR）	MOL000378	7-O-methylisomucronulatol	Muscarinic acetylcholine receptor M3	P20309	CHRM3	0.016
Astragali Radix（AgR）	MOL000378	7-O-methylisomucronulatol	D(1A) dopamine receptor	P21728	DRD1	0.016
Astragali Radix（AgR）	MOL000378	7-O-methylisomucronulatol	D(4) dopamine receptor	P21917	DRD4	0.016
Astragali Radix（AgR）	MOL000378	7-O-methylisomucronulatol	D(1B) dopamine receptor	P21918	DRD5	0.016
Astragali Radix（AgR）	MOL000378	7-O-methylisomucronulatol	Alpha-1D adrenergic receptor	P25100	ADRA1D	0.016
Astragali Radix（AgR）	MOL000378	7-O-methylisomucronulatol	5-hydroxytryptamine 2A receptor	P28223	HTR2A	0.016
Astragali Radix（AgR）	MOL000378	7-O-methylisomucronulatol	5-hydroxytryptamine 2C receptor	P28335	HTR2C	0.016
Astragali Radix（AgR）	MOL000378	7-O-methylisomucronulatol	Neuronal acetylcholine receptor subunit alpha-5	P30532	CHRNA5	0.016
Astragali Radix（AgR）	MOL000378	7-O-methylisomucronulatol	Neuronal acetylcholine receptor subunit beta-4	P30926	CHRNB4	0.016
Astragali Radix（AgR）	MOL000378	7-O-methylisomucronulatol	Histamine H1 receptor	P35367	HRH1	0.016
Astragali Radix（AgR）	MOL000378	7-O-methylisomucronulatol	Alpha-1B adrenergic receptor	P35368	ADRA1B	0.016
Astragali Radix（AgR）	MOL000378	7-O-methylisomucronulatol	D(3) dopamine receptor	P35462	DRD3	0.016
Astragali Radix（AgR）	MOL000378	7-O-methylisomucronulatol	Neuronal acetylcholine receptor subunit alpha-4	P43681	CHRNA4	0.016
Astragali Radix（AgR）	MOL000378	7-O-methylisomucronulatol	Acetylcholine receptor subunit epsilon	Q04844	CHRNE	0.016
Astragali Radix（AgR）	MOL000378	7-O-methylisomucronulatol	Neuronal acetylcholine receptor subunit beta-3	Q05901	CHRNB3	0.016
Astragali Radix（AgR）	MOL000378	7-O-methylisomucronulatol	Acetylcholine receptor subunit delta	Q07001	CHRND	0.016
Astragali Radix（AgR）	MOL000378	7-O-methylisomucronulatol	Neuronal acetylcholine receptor subunit alpha-6	Q15825	KCNJ8	0.016
Astragali Radix（AgR）	MOL000378	7-O-methylisomucronulatol	Neuronal acetylcholine receptor subunit alpha-10	Q9GZZ6	CHRNA10	0.016
Astragali Radix（AgR）	MOL000378	7-O-methylisomucronulatol	Neuronal acetylcholine receptor subunit alpha-9	Q9UGM1	CHRNA9	0.016
Astragali Radix（AgR）	MOL000378	7-O-methylisomucronulatol	Opioid receptor, sigma 1	Q5T1J1	SIGMAR1	0.018
Astragali Radix（AgR）	MOL000378	7-O-methylisomucronulatol	Glutamate [NMDA] receptor subunit 3A	Q8TCU5	GRIN3A	0.018
Astragali Radix（AgR）	MOL000378	7-O-methylisomucronulatol	Sigma 1-type opioid receptor	Q99720	SIGMAR1	0.018
Astragali Radix（AgR）	MOL000378	7-O-methylisomucronulatol	Gamma-aminobutyric acid receptor subunit rho-3	A8MPY1	GABRR3	0.019

Astragali Radix（AgR）	MOL000378	7-O-methylisomucronulatol	Gamma-aminobutyric acid receptor subunit pi	O00591	GABRP	0.019
Astragali Radix（AgR）	MOL000378	7-O-methylisomucronulatol	Gamma-aminobutyric acid receptor subunit delta	O14764	GABRD	0.019
Astragali Radix（AgR）	MOL000378	7-O-methylisomucronulatol	Gamma-aminobutyric-acid receptor subunit beta-1	P18505	GABRB1	0.019
Astragali Radix（AgR）	MOL000378	7-O-methylisomucronulatol	Gamma-aminobutyric acid receptor subunit gamma-2	P18507	GABRG2	0.019
Astragali Radix（AgR）	MOL000378	7-O-methylisomucronulatol	Gamma-aminobutyric-acid receptor subunit rho-1	P24046	GABRR1	0.019
Astragali Radix（AgR）	MOL000378	7-O-methylisomucronulatol	Gamma-aminobutyric-acid receptor subunit beta-3	P28472	GABRB3	0.019
Astragali Radix（AgR）	MOL000378	7-O-methylisomucronulatol	Gamma-aminobutyric acid receptor subunit rho-2	P28476	GABRR2	0.019
Astragali Radix（AgR）	MOL000378	7-O-methylisomucronulatol	Translocator protein	P30536	TSPO	0.019
Astragali Radix（AgR）	MOL000378	7-O-methylisomucronulatol	Gamma-aminobutyric-acid receptor subunit alpha-5	P31644	GABRA5	0.019
Astragali Radix（AgR）	MOL000378	7-O-methylisomucronulatol	Gamma-aminobutyric-acid receptor subunit alpha-3	P34903	GABRA3	0.019
Astragali Radix（AgR）	MOL000378	7-O-methylisomucronulatol	Gamma-aminobutyric-acid receptor subunit beta-2	P47870	GABRB2	0.019
Astragali Radix（AgR）	MOL000378	7-O-methylisomucronulatol	Gamma-aminobutyric-acid receptor subunit alpha-4	P48169	GABRA4	0.019
Astragali Radix（AgR）	MOL000378	7-O-methylisomucronulatol	Gamma-aminobutyric acid receptor subunit epsilon	P78334	GABRE	0.019
Astragali Radix（AgR）	MOL000378	7-O-methylisomucronulatol	Gamma-aminobutyric acid receptor subunit gamma-3	Q99928	GABRG3	0.019
Astragali Radix（AgR）	MOL000378	7-O-methylisomucronulatol	Gamma-aminobutyric acid receptor subunit theta	Q9UN88	GABRQ	0.019
Astragali Radix（AgR）	MOL000378	7-O-methylisomucronulatol	Potassium channel subfamily K member 1	O00180	KCNK1	0.023
Astragali Radix（AgR）	MOL000378	7-O-methylisomucronulatol	2,4-dienoyl-CoA reductase, mitochondrial	Q16698	DECR1	0.028
Astragali Radix（AgR）	MOL000378	7-O-methylisomucronulatol	Sodium/potassium-transporting ATPase alpha-1 chain	P05023	ATP1A1	0.029
Astragali Radix（AgR）	MOL000378	7-O-methylisomucronulatol	Sodium/potassium-transporting ATPase gamma chain	P54710	FXYD2	0.029
Astragali Radix（AgR）	MOL000378	7-O-methylisomucronulatol	Calcium-activated potassium channel subunit alpha 1	Q12791	KCNMA1	0.029
Astragali Radix（AgR）	MOL000378	7-O-methylisomucronulatol	ATP-sensitive inward rectifier potassium channel 11	Q14654	KCNJ11	0.029
Astragali Radix（AgR）	MOL000378	7-O-methylisomucronulatol	Acetylcholinesterase	P22303	ACHE	0.036
Astragali Radix（AgR）	MOL000378	7-O-methylisomucronulatol	Neuronal acetylcholine receptor subunit alpha-3	P32297	CHRNA3	0.036
Astragali Radix（AgR）	MOL000378	7-O-methylisomucronulatol	Neuronal acetylcholine receptor subunit alpha-7	P36544	CHRNA7	0.036
Astragali Radix（AgR）	MOL000378	7-O-methylisomucronulatol	Tyrosine-protein kinase JAK2	O60674	JAK2	0.037
Astragali Radix（AgR）	MOL000378	7-O-methylisomucronulatol	Tyrosine-protein kinase JAK1	P23458	JAK1	0.037
Astragali Radix（AgR）	MOL000378	7-O-methylisomucronulatol	Tyrosine-protein kinase JAK3	P52333	JAK3	0.037
Astragali Radix（AgR）	MOL000378	7-O-methylisomucronulatol	Neuronal acetylcholine receptor subunit alpha-2	Q15822	CHRNA2	0.038
Astragali Radix（AgR）	MOL000378	7-O-methylisomucronulatol	Bile salt sulfotransferase	Q06520	SULT2A1	0.04
Astragali Radix（AgR）	MOL000378	7-O-methylisomucronulatol	Dehydrogenase/reductase SDR family member 8	Q8NBQ5	HSD17B11	0.04
Astragali Radix（AgR）	MOL000378	7-O-methylisomucronulatol	Solute carrier family 22 member 6	Q4U2R8	SLC22A6	0.042
Astragali Radix（AgR）	MOL000378	7-O-methylisomucronulatol	Solute carrier family 22 member 8	Q8TCC7	SLC22A8	0.042
Astragali Radix（AgR）	MOL000378	7-O-methylisomucronulatol	Solute carrier family 22 member 11	Q9NSA0	SLC22A11	0.042
Astragali Radix（AgR）	MOL000378	7-O-methylisomucronulatol	Gamma-aminobutyric-acid receptor subunit alpha-2	P47869	GABRA2	0.045
Astragali Radix（AgR）	MOL000378	7-O-methylisomucronulatol	Cell division protein kinase 2	P24941	CDK2	0.046
Astragali Radix（AgR）	MOL000378	7-O-methylisomucronulatol	Sodium channel protein type 5 subunit alpha	Q14524	SCN5A	0.052
Astragali Radix（AgR）	MOL000378	7-O-methylisomucronulatol	Alpha-1A adrenergic receptor	P35348	ADRA1A	0.057
Astragali Radix（AgR）	MOL000378	7-O-methylisomucronulatol	Myeloperoxidase	P05164	MPO	0.058
Astragali Radix（AgR）	MOL000378	7-O-methylisomucronulatol	Eosinophil peroxidase	P11678	EPX	0.058
Astragali Radix（AgR）	MOL000378	7-O-methylisomucronulatol	Ribosyldihydronicotinamide dehydrogenase [quinone]	P16083	NQO2	0.058
Astragali Radix（AgR）	MOL000378	7-O-methylisomucronulatol	Calreticulin	P27797	CALR	0.058
Astragali Radix（AgR）	MOL000378	7-O-methylisomucronulatol	Melatonin receptor type 1A	P48039	MTNR1A	0.058
Astragali Radix（AgR）	MOL000378	7-O-methylisomucronulatol	Melatonin receptor type 1B	P49286	MTNR1B	0.058
Astragali Radix（AgR）	MOL000378	7-O-methylisomucronulatol	Calmodulin	P62158		0.058
Astragali Radix（AgR）	MOL000378	7-O-methylisomucronulatol	Tubulin beta-2C chain	P68371	TUBB4B	0.058
Astragali Radix（AgR）	MOL000378	7-O-methylisomucronulatol	Tubulin alpha-3 chain	Q71U36	TUBA1A	0.058
Astragali Radix（AgR）	MOL000378	7-O-methylisomucronulatol	Nuclear receptor ROR-beta	Q92753	RORB	0.058

Astragali Radix（AgR）	MOL000378	7-O-methylisomucronulatol	Sterol O-acyltransferase 2	O75908	SOAT2	0.059
Astragali Radix（AgR）	MOL000378	7-O-methylisomucronulatol	cAMP-dependent protein kinase catalytic subunit alpha	P17612	PRKACA	0.059
Astragali Radix（AgR）	MOL000378	7-O-methylisomucronulatol	Sterol O-acyltransferase 1	P35610	SOAT1	0.059
Astragali Radix（AgR）	MOL000378	7-O-methylisomucronulatol	Rho-associated protein kinase 1	Q13464	ROCK1	0.059
Astragali Radix（AgR）	MOL000378	7-O-methylisomucronulatol	Carbonic anhydrase 1	P00915	CA1	0.06
Astragali Radix（AgR）	MOL000378	7-O-methylisomucronulatol	Carbonic anhydrase 2	P00918	CA2	0.06
Astragali Radix（AgR）	MOL000378	7-O-methylisomucronulatol	Stromelysin-1	P08254	MMP3	0.062
Astragali Radix（AgR）	MOL000378	7-O-methylisomucronulatol	Stromelysin-2	P09238	MMP10	0.062
Astragali Radix（AgR）	MOL000378	7-O-methylisomucronulatol	DNA topoisomerase 2-alpha	P11388	TOP2A	0.062
Astragali Radix（AgR）	MOL000378	7-O-methylisomucronulatol	Prothrombin	P00734	F2	0.063
Astragali Radix（AgR）	MOL000378	7-O-methylisomucronulatol	Trypsin-1	P07477	PRSS1	0.063
Astragali Radix（AgR）	MOL000378	7-O-methylisomucronulatol	Nitric-oxide synthase, endothelial	P29474	NOS3	0.063
Astragali Radix（AgR）	MOL000378	7-O-methylisomucronulatol	Nitric-oxide synthase, brain	P29475	NOS1	0.063
Astragali Radix（AgR）	MOL000378	7-O-methylisomucronulatol	Gamma-aminobutyric-acid receptor subunit alpha-1	P14867	GABRA1	0.07
Astragali Radix（AgR）	MOL000378	7-O-methylisomucronulatol	Succinate dehydrogenase [ubiquinone] cytochrome b small subunit, mitochondrial	O14521	SDHD	0.071
Astragali Radix（AgR）	MOL000378	7-O-methylisomucronulatol	3 beta-hydroxysteroid dehydrogenase/Delta 5-->4-isomerase type I	P14060	HSD3B1	0.075
Astragali Radix（AgR）	MOL000378	7-O-methylisomucronulatol	3 beta-hydroxysteroid dehydrogenase/Delta 5-->4-isomerase type II	P26439	HSD3B2	0.075
Astragali Radix（AgR）	MOL000378	7-O-methylisomucronulatol	Kappa-type opioid receptor	P41145	OPRK1	0.089
Astragali Radix（AgR）	MOL000378	7-O-methylisomucronulatol	D-HSCDK2	O75100	CA11	0.096
Astragali Radix（AgR）	MOL000378	7-O-methylisomucronulatol	Cell division control protein 2 homolog	P06493	CDK1	0.096
Astragali Radix（AgR）	MOL000378	7-O-methylisomucronulatol	Phospholipase A2	P04054	PLA2G1B	0.099
Astragali Radix（AgR）	MOL000378	7-O-methylisomucronulatol	Triosephosphate isomerase	P60174	TPI1	0.118
Astragali Radix（AgR）	MOL000378	7-O-methylisomucronulatol	Glyceraldehyde-3-phosphate dehydrogenase	P04406	GAPDH	0.119
Astragali Radix（AgR）	MOL000378	7-O-methylisomucronulatol	Ig kappa chain V-II region RPMI 6410	P06310	IGKV2-30	0.12
Astragali Radix（AgR）	MOL000378	7-O-methylisomucronulatol	Tyrosine-protein kinase HCK	P08631	HCK	0.121
Astragali Radix（AgR）	MOL000378	7-O-methylisomucronulatol	MAP kinase-activated protein kinase 2	P49137	MAPKAPK2	0.122
Astragali Radix（AgR）	MOL000378	7-O-methylisomucronulatol	NADPH oxidase organizer 1	Q8NFA2	NOXO1	0.122
Astragali Radix（AgR）	MOL000378	7-O-methylisomucronulatol	Phospholipase A2, membrane associated	P14555	PLA2G2A	0.124
Astragali Radix（AgR）	MOL000378	7-O-methylisomucronulatol	Macrophage metalloelastase	P39900	MMP12	0.127
Astragali Radix（AgR）	MOL000378	7-O-methylisomucronulatol	Nuclear receptor coactivator 2	Q15596	NCOA2	0.127
Astragali Radix（AgR）	MOL000378	7-O-methylisomucronulatol	Liver carboxylesterase 1	P23141	CES1	0.132
Astragali Radix（AgR）	MOL000378	7-O-methylisomucronulatol	Endothelin-1 receptor	P25101	EDNRA	0.134
Astragali Radix（AgR）	MOL000378	7-O-methylisomucronulatol	Mitogen-activated protein kinase 14	Q16539	MAPK14	0.135
Astragali Radix（AgR）	MOL000378	7-O-methylisomucronulatol	Cytochrome P450 11B1, mitochondrial	P15538	CYP11B1	0.138
Astragali Radix（AgR）	MOL000378	7-O-methylisomucronulatol	Thyroid hormone receptor alpha	P10827	THRA	0.141
Astragali Radix（AgR）	MOL000378	7-O-methylisomucronulatol	Cell division protein kinase 5	Q00535	CDK5	0.145
Astragali Radix（AgR）	MOL000378	7-O-methylisomucronulatol	cAMP-specific 3',5'-cyclic phosphodiesterase 4B	Q07343	PDE4B	0.149
Astragali Radix（AgR）	MOL000378	7-O-methylisomucronulatol	Androgen receptor	P10275	AR	0.171
Astragali Radix（AgR）	MOL000378	7-O-methylisomucronulatol	Mineralocorticoid receptor	P08235	NR3C2	0.248
Astragali Radix（AgR）	MOL000378	7-O-methylisomucronulatol	Mu-type opioid receptor	P35372	OPRM1	0.273
Astragali Radix（AgR）	MOL000378	7-O-methylisomucronulatol	Hemoglobin subunit alpha	P69905	HBA1	0.291
Astragali Radix（AgR）	MOL000378	7-O-methylisomucronulatol	Nuclear receptor coactivator 1	Q15788	NCOA1	0.296
Astragali Radix（AgR）	MOL000378	7-O-methylisomucronulatol	Sodium-dependent noradrenaline transporter	P23975	SLC6A2	0.324
Astragali Radix（AgR）	MOL000378	7-O-methylisomucronulatol	Sodium-dependent serotonin transporter	P31645	SLC6A4	0.324
Astragali Radix（AgR）	MOL000378	7-O-methylisomucronulatol	Prostaglandin G/H synthase 1	P23219	PTGS1	0.407
Astragali Radix（AgR）	MOL000378	7-O-methylisomucronulatol	Progesterone receptor	P06401	PGR	0.422
Astragali Radix（AgR）	MOL000378	7-O-methylisomucronulatol	Estrogen receptor beta	Q92731	ESR2	0.464

Astragali Radix（AgR）	MOL000378	7-O-methylisomucronulatol	Prostaglandin G/H synthase 2	P35354	PTGS2	0.817
Astragali Radix（AgR）	MOL000378	7-O-methylisomucronulatol	Estrogen receptor	P03372	ESR1	1
Astragali Radix（AgR）	MOL000379	9,10-dimethoxypterocarpan-3-O-β-D-glucoside	Trypsin-1	P07477	PRSS1	0.092
Astragali Radix（AgR）	MOL000379	9,10-dimethoxypterocarpan-3-O-β-D-glucoside	Acetylcholinesterase	P22303	ACHE	0.106
Astragali Radix（AgR）	MOL000379	9,10-dimethoxypterocarpan-3-O-β-D-glucoside	Nuclear receptor coactivator 1	Q15788	NCOA1	0.192
Astragali Radix（AgR）	MOL000379	9,10-dimethoxypterocarpan-3-O-β-D-glucoside	Estrogen receptor beta	Q92731	ESR2	0.45
Astragali Radix（AgR）	MOL000379	9,10-dimethoxypterocarpan-3-O-β-D-glucoside	Estrogen receptor	P03372	ESR1	1
Astragali Radix（AgR）	MOL000380	(6aR,11aR)-9,10-dimethoxy-6a,11a-dihydro-6H-benzofurano[3,2-c]chromen-3-ol	Death-associated protein kinase 3	O43293	DAPK3	0.01
Astragali Radix（AgR）	MOL000380	(6aR,11aR)-9,10-dimethoxy-6a,11a-dihydro-6H-benzofurano[3,2-c]chromen-3-ol	Neuronal acetylcholine receptor subunit alpha-3	P32297	CHRNA3	0.011
Astragali Radix（AgR）	MOL000380	(6aR,11aR)-9,10-dimethoxy-6a,11a-dihydro-6H-benzofurano[3,2-c]chromen-3-ol	Serine/threonine-protein kinase 17B	O94768	STK17B	0.012
Astragali Radix（AgR）	MOL000380	(6aR,11aR)-9,10-dimethoxy-6a,11a-dihydro-6H-benzofurano[3,2-c]chromen-3-ol	ATP synthase subunit gamma, mitochondrial	P36542	ATP5F1C	0.012
Astragali Radix（AgR）	MOL000380	(6aR,11aR)-9,10-dimethoxy-6a,11a-dihydro-6H-benzofurano[3,2-c]chromen-3-ol	UDP-glucuronosyltransferase 3A1	Q6NUS8	UGT3A1	0.012
Astragali Radix（AgR）	MOL000380	(6aR,11aR)-9,10-dimethoxy-6a,11a-dihydro-6H-benzofurano[3,2-c]chromen-3-ol	Cytochrome b	P00156	MT-CYB	0.013
Astragali Radix（AgR）	MOL000380	(6aR,11aR)-9,10-dimethoxy-6a,11a-dihydro-6H-benzofurano[3,2-c]chromen-3-ol	Cytochrome c1, heme protein, mitochondrial	P08574	CYC1	0.013
Astragali Radix（AgR）	MOL000380	(6aR,11aR)-9,10-dimethoxy-6a,11a-dihydro-6H-benzofurano[3,2-c]chromen-3-ol	Cytochrome b-c1 complex subunit Rieske, mitochondrial	P47985	UQCRFS1	0.013
Astragali Radix（AgR）	MOL000380	(6aR,11aR)-9,10-dimethoxy-6a,11a-dihydro-6H-benzofurano[3,2-c]chromen-3-ol	Neuronal acetylcholine receptor subunit alpha-2	Q15822	CHRNA2	0.013
Astragali Radix（AgR）	MOL000380	(6aR,11aR)-9,10-dimethoxy-6a,11a-dihydro-6H-benzofurano[3,2-c]chromen-3-ol	Gamma-aminobutyric acid receptor subunit rho-3	A8MPY1	GABRR3	0.014
Astragali Radix（AgR）	MOL000380	(6aR,11aR)-9,10-dimethoxy-6a,11a-dihydro-6H-benzofurano[3,2-c]chromen-3-ol	Gamma-aminobutyric acid receptor subunit pi	O00591	GABRP	0.014
Astragali Radix（AgR）	MOL000380	(6aR,11aR)-9,10-dimethoxy-6a,11a-dihydro-6H-benzofurano[3,2-c]chromen-3-ol	Gamma-aminobutyric acid receptor subunit delta	O14764	GABRD	0.014
Astragali Radix（AgR）	MOL000380	(6aR,11aR)-9,10-dimethoxy-6a,11a-dihydro-6H-benzofurano[3,2-c]chromen-3-ol	Muscarinic acetylcholine receptor M5	P08912	CHRM5	0.014
Astragali Radix（AgR）	MOL000380	(6aR,11aR)-9,10-dimethoxy-6a,11a-dihydro-6H-benzofurano[3,2-c]chromen-3-ol	Gamma-aminobutyric-acid receptor subunit beta-1	P18505	GABRB1	0.014
Astragali Radix（AgR）	MOL000380	(6aR,11aR)-9,10-dimethoxy-6a,11a-dihydro-6H-benzofurano[3,2-c]chromen-3-ol	Gamma-aminobutyric acid receptor subunit gamma-2	P18507	GABRG2	0.014
Astragali Radix（AgR）	MOL000380	(6aR,11aR)-9,10-dimethoxy-6a,11a-dihydro-6H-benzofurano[3,2-c]chromen-3-ol	D(4) dopamine receptor	P21917	DRD4	0.014
Astragali Radix（AgR）	MOL000380	(6aR,11aR)-9,10-dimethoxy-6a,11a-dihydro-6H-benzofurano[3,2-c]chromen-3-ol	Gamma-aminobutyric-acid receptor subunit rho-1	P24046	GABRR1	0.014
Astragali Radix（AgR）	MOL000380	(6aR,11aR)-9,10-dimethoxy-6a,11a-dihydro-6H-benzofurano[3,2-c]chromen-3-ol	5-hydroxytryptamine 1B receptor	P28222	HTR1B	0.014
Astragali Radix（AgR）	MOL000380	(6aR,11aR)-9,10-dimethoxy-6a,11a-dihydro-6H-benzofurano[3,2-c]chromen-3-ol	Gamma-aminobutyric-acid receptor subunit beta-3	P28472	GABRB3	0.014
Astragali Radix（AgR）	MOL000380	(6aR,11aR)-9,10-dimethoxy-6a,11a-dihydro-6H-benzofurano[3,2-c]chromen-3-ol	Gamma-aminobutyric acid receptor subunit rho-2	P28476	GABRR2	0.014
Astragali Radix（AgR）	MOL000380	(6aR,11aR)-9,10-dimethoxy-6a,11a-dihydro-6H-benzofurano[3,2-c]chromen-3-ol	Histamine H1 receptor	P35367	HRH1	0.014
Astragali Radix（AgR）	MOL000380	(6aR,11aR)-9,10-dimethoxy-6a,11a-dihydro-6H-benzofurano[3,2-c]chromen-3-ol	D(3) dopamine receptor	P35462	DRD3	0.014
Astragali Radix（AgR）	MOL000380	(6aR,11aR)-9,10-dimethoxy-6a,11a-dihydro-6H-benzofurano[3,2-c]chromen-3-ol	Amiloride-sensitive sodium channel subunit alpha	P37088	SCNN1A	0.014
Astragali Radix（AgR）	MOL000380	(6aR,11aR)-9,10-dimethoxy-6a,11a-dihydro-6H-benzofurano[3,2-c]chromen-3-ol	Nociceptin receptor	P41146	OPRL1	0.014
Astragali Radix（AgR）	MOL000380	(6aR,11aR)-9,10-dimethoxy-6a,11a-dihydro-6H-benzofurano[3,2-c]chromen-3-ol	Gamma-aminobutyric-acid receptor subunit beta-2	P47870	GABRB2	0.014
Astragali Radix（AgR）	MOL000380	(6aR,11aR)-9,10-dimethoxy-6a,11a-dihydro-6H-benzofurano[3,2-c]chromen-3-ol	Amiloride-sensitive sodium channel subunit beta	P51168	SCNN1B	0.014

Astragali Radix（AgR）	MOL000380	(6aR,11aR)-9,10-dimethoxy-6a,11a-dihydro-6H-benzofurano[3,2-c]chromen-3-ol	Amiloride-sensitive sodium channel subunit gamma	P51170	SCNN1G	0.014
Astragali Radix（AgR）	MOL000380	(6aR,11aR)-9,10-dimethoxy-6a,11a-dihydro-6H-benzofurano[3,2-c]chromen-3-ol	Gamma-aminobutyric acid receptor subunit epsilon	P78334	GABRE	0.014
Astragali Radix（AgR）	MOL000380	(6aR,11aR)-9,10-dimethoxy-6a,11a-dihydro-6H-benzofurano[3,2-c]chromen-3-ol	Potassium voltage-gated channel subfamily H member 2	Q12809	KCNH2	0.014
Astragali Radix（AgR）	MOL000380	(6aR,11aR)-9,10-dimethoxy-6a,11a-dihydro-6H-benzofurano[3,2-c]chromen-3-ol	Gamma-aminobutyric-acid receptor subunit alpha-6	Q16445	GABRA6	0.014
Astragali Radix（AgR）	MOL000380	(6aR,11aR)-9,10-dimethoxy-6a,11a-dihydro-6H-benzofurano[3,2-c]chromen-3-ol	Gamma-aminobutyric acid receptor subunit gamma-3	Q99928	GABRG3	0.014
Astragali Radix（AgR）	MOL000380	(6aR,11aR)-9,10-dimethoxy-6a,11a-dihydro-6H-benzofurano[3,2-c]chromen-3-ol	Potassium voltage-gated channel subfamily H member 6	Q9H252	KCNH6	0.014
Astragali Radix（AgR）	MOL000380	(6aR,11aR)-9,10-dimethoxy-6a,11a-dihydro-6H-benzofurano[3,2-c]chromen-3-ol	3-phosphoinositide-dependent protein kinase 1	O15530	PDPK1	0.015
Astragali Radix（AgR）	MOL000380	(6aR,11aR)-9,10-dimethoxy-6a,11a-dihydro-6H-benzofurano[3,2-c]chromen-3-ol	T-cell receptor alpha chain C region	P01848	TRAC	0.015
Astragali Radix（AgR）	MOL000380	(6aR,11aR)-9,10-dimethoxy-6a,11a-dihydro-6H-benzofurano[3,2-c]chromen-3-ol	T-cell receptor beta chain C region	P01850	TRBC1	0.015
Astragali Radix（AgR）	MOL000380	(6aR,11aR)-9,10-dimethoxy-6a,11a-dihydro-6H-benzofurano[3,2-c]chromen-3-ol	Proto-oncogene tyrosine-protein kinase LCK	P06239	LCK	0.015
Astragali Radix（AgR）	MOL000380	(6aR,11aR)-9,10-dimethoxy-6a,11a-dihydro-6H-benzofurano[3,2-c]chromen-3-ol	Dihydrolipoyllysine-residue acetyltransferase component of pyruvate dehydrogenase complex, mitochondrial	P10515	DLAT	0.015
Astragali Radix（AgR）	MOL000380	(6aR,11aR)-9,10-dimethoxy-6a,11a-dihydro-6H-benzofurano[3,2-c]chromen-3-ol	Glycine cleavage system H protein, mitochondrial	P23434	GCSH	0.015
Astragali Radix（AgR）	MOL000380	(6aR,11aR)-9,10-dimethoxy-6a,11a-dihydro-6H-benzofurano[3,2-c]chromen-3-ol	Tyrosine-protein kinase CSK	P41240	CSK	0.015
Astragali Radix（AgR）	MOL000380	(6aR,11aR)-9,10-dimethoxy-6a,11a-dihydro-6H-benzofurano[3,2-c]chromen-3-ol	Tyrosine-protein kinase ZAP-70	P43403	ZAP70	0.015
Astragali Radix（AgR）	MOL000380	(6aR,11aR)-9,10-dimethoxy-6a,11a-dihydro-6H-benzofurano[3,2-c]chromen-3-ol	Tyrosine-protein kinase SYK	P43405	SYK	0.015
Astragali Radix（AgR）	MOL000380	(6aR,11aR)-9,10-dimethoxy-6a,11a-dihydro-6H-benzofurano[3,2-c]chromen-3-ol	Glycogen synthase kinase-3 beta	P49841	GSK3B	0.015
Astragali Radix（AgR）	MOL000380	(6aR,11aR)-9,10-dimethoxy-6a,11a-dihydro-6H-benzofurano[3,2-c]chromen-3-ol	Beta-2-microglobulin	P61769	B2M	0.015
Astragali Radix（AgR）	MOL000380	(6aR,11aR)-9,10-dimethoxy-6a,11a-dihydro-6H-benzofurano[3,2-c]chromen-3-ol	Sodium-dependent dopamine transporter	Q01959	SLC6A3	0.015
Astragali Radix（AgR）	MOL000380	(6aR,11aR)-9,10-dimethoxy-6a,11a-dihydro-6H-benzofurano[3,2-c]chromen-3-ol	Protein kinase C theta type	Q04759	PRKCQ	0.015
Astragali Radix（AgR）	MOL000380	(6aR,11aR)-9,10-dimethoxy-6a,11a-dihydro-6H-benzofurano[3,2-c]chromen-3-ol	Tyrosine-protein kinase ITK/TSK	Q08881	ITK	0.015
Astragali Radix（AgR）	MOL000380	(6aR,11aR)-9,10-dimethoxy-6a,11a-dihydro-6H-benzofurano[3,2-c]chromen-3-ol	Dehydrogenase/reductase SDR family member 8	Q8NBQ5	HSD17B11	0.015
Astragali Radix（AgR）	MOL000380	(6aR,11aR)-9,10-dimethoxy-6a,11a-dihydro-6H-benzofurano[3,2-c]chromen-3-ol	Glutamate [NMDA] receptor subunit 3A	Q8TCU5	GRIN3A	0.015
Astragali Radix（AgR）	MOL000380	(6aR,11aR)-9,10-dimethoxy-6a,11a-dihydro-6H-benzofurano[3,2-c]chromen-3-ol	Vascular endothelial growth factor	A2A2V4	VEGFA	0.016
Astragali Radix（AgR）	MOL000380	(6aR,11aR)-9,10-dimethoxy-6a,11a-dihydro-6H-benzofurano[3,2-c]chromen-3-ol	Tumor necrosis factor ligand superfamily member 11	O14788	TNFSF11	0.016
Astragali Radix（AgR）	MOL000380	(6aR,11aR)-9,10-dimethoxy-6a,11a-dihydro-6H-benzofurano[3,2-c]chromen-3-ol	Tyrosine 3-monooxygenase	P07101	TH	0.016
Astragali Radix（AgR）	MOL000380	(6aR,11aR)-9,10-dimethoxy-6a,11a-dihydro-6H-benzofurano[3,2-c]chromen-3-ol	Cadherin-5	P33151	CDH5	0.016
Astragali Radix（AgR）	MOL000380	(6aR,11aR)-9,10-dimethoxy-6a,11a-dihydro-6H-benzofurano[3,2-c]chromen-3-ol	Peptidyl-prolyl cis-trans isomerase NIMA-interacting 1	Q13526	PIN1	0.016
Astragali Radix（AgR）	MOL000380	(6aR,11aR)-9,10-dimethoxy-6a,11a-dihydro-6H-benzofurano[3,2-c]chromen-3-ol	Tyrosyl-tRNA synthetase, mitochondrial	Q9Y2Z4	YARS2	0.016
Astragali Radix（AgR）	MOL000380	(6aR,11aR)-9,10-dimethoxy-6a,11a-dihydro-6H-benzofurano[3,2-c]chromen-3-ol	Glycogen phosphorylase, muscle form	P11217	PYGM	0.017
Astragali Radix（AgR）	MOL000380	(6aR,11aR)-9,10-dimethoxy-6a,11a-dihydro-6H-benzofurano[3,2-c]chromen-3-ol	Cell division protein kinase 4	P11802	CDK4	0.017
Astragali Radix（AgR）	MOL000380	(6aR,11aR)-9,10-dimethoxy-6a,11a-dihydro-6H-benzofurano[3,2-c]chromen-3-ol	Neuronal acetylcholine receptor subunit alpha-7	P36544	CHRNA7	0.017

Astragali Radix（AgR）	MOL000380	(6aR,11aR)-9,10-dimethoxy-6a,11a-dihydro-6H-benzofurano[3,2-c]chromen-3-ol	Cell division protein kinase 7	P50613	CDK7	0.017
Astragali Radix（AgR）	MOL000380	(6aR,11aR)-9,10-dimethoxy-6a,11a-dihydro-6H-benzofurano[3,2-c]chromen-3-ol	Cell division protein kinase 9	P50750	CDK9	0.017
Astragali Radix（AgR）	MOL000380	(6aR,11aR)-9,10-dimethoxy-6a,11a-dihydro-6H-benzofurano[3,2-c]chromen-3-ol	cGMP-specific 3',5'-cyclic phosphodiesterase	O76074	PDE5A	0.018
Astragali Radix（AgR）	MOL000380	(6aR,11aR)-9,10-dimethoxy-6a,11a-dihydro-6H-benzofurano[3,2-c]chromen-3-ol	Adenosine A2a receptor	P29274	ADORA2A	0.018
Astragali Radix（AgR）	MOL000380	(6aR,11aR)-9,10-dimethoxy-6a,11a-dihydro-6H-benzofurano[3,2-c]chromen-3-ol	Adenosine A1 receptor	P30542	ADORA1	0.018
Astragali Radix（AgR）	MOL000380	(6aR,11aR)-9,10-dimethoxy-6a,11a-dihydro-6H-benzofurano[3,2-c]chromen-3-ol	cAMP-specific 3',5'-cyclic phosphodiesterase 4C	Q08493	PDE4C	0.018
Astragali Radix（AgR）	MOL000380	(6aR,11aR)-9,10-dimethoxy-6a,11a-dihydro-6H-benzofurano[3,2-c]chromen-3-ol	cGMP-inhibited 3',5'-cyclic phosphodiesterase A	Q14432	PDE3A	0.018
Astragali Radix（AgR）	MOL000380	(6aR,11aR)-9,10-dimethoxy-6a,11a-dihydro-6H-benzofurano[3,2-c]chromen-3-ol	cAMP and cAMP-inhibited cGMP 3',5'-cyclic phosphodiesterase 10A	Q9Y233	PDE10A	0.018
Astragali Radix（AgR）	MOL000380	(6aR,11aR)-9,10-dimethoxy-6a,11a-dihydro-6H-benzofurano[3,2-c]chromen-3-ol	Gamma-aminobutyric-acid receptor subunit alpha-4	P48169	GABRA4	0.02
Astragali Radix（AgR）	MOL000380	(6aR,11aR)-9,10-dimethoxy-6a,11a-dihydro-6H-benzofurano[3,2-c]chromen-3-ol	Carbonic anhydrase 1	P00915	CA1	0.021
Astragali Radix（AgR）	MOL000380	(6aR,11aR)-9,10-dimethoxy-6a,11a-dihydro-6H-benzofurano[3,2-c]chromen-3-ol	Tumor necrosis factor	P01375	TNF	0.021
Astragali Radix（AgR）	MOL000380	(6aR,11aR)-9,10-dimethoxy-6a,11a-dihydro-6H-benzofurano[3,2-c]chromen-3-ol	Alcohol dehydrogenase [NADP+]	P14550	AKR1A1	0.021
Astragali Radix（AgR）	MOL000380	(6aR,11aR)-9,10-dimethoxy-6a,11a-dihydro-6H-benzofurano[3,2-c]chromen-3-ol	Aldose reductase	P15121	AKR1B1	0.021
Astragali Radix（AgR）	MOL000380	(6aR,11aR)-9,10-dimethoxy-6a,11a-dihydro-6H-benzofurano[3,2-c]chromen-3-ol	Nuclear factor NF-kappa-B p105 subunit	P19838	NFKB1	0.021
Astragali Radix（AgR）	MOL000380	(6aR,11aR)-9,10-dimethoxy-6a,11a-dihydro-6H-benzofurano[3,2-c]chromen-3-ol	Carbonic anhydrase 4	P22748	CA4	0.021
Astragali Radix（AgR）	MOL000380	(6aR,11aR)-9,10-dimethoxy-6a,11a-dihydro-6H-benzofurano[3,2-c]chromen-3-ol	Sepiapterin reductase	P35270	SPR	0.021
Astragali Radix（AgR）	MOL000380	(6aR,11aR)-9,10-dimethoxy-6a,11a-dihydro-6H-benzofurano[3,2-c]chromen-3-ol	Aryl hydrocarbon receptor	P35869	AHR	0.021
Astragali Radix（AgR）	MOL000380	(6aR,11aR)-9,10-dimethoxy-6a,11a-dihydro-6H-benzofurano[3,2-c]chromen-3-ol	Methionine aminopeptidase 1	P53582	METAP1	0.021
Astragali Radix（AgR）	MOL000380	(6aR,11aR)-9,10-dimethoxy-6a,11a-dihydro-6H-benzofurano[3,2-c]chromen-3-ol	Dihydroorotate dehydrogenase, mitochondrial	Q02127	DHODH	0.021
Astragali Radix（AgR）	MOL000380	(6aR,11aR)-9,10-dimethoxy-6a,11a-dihydro-6H-benzofurano[3,2-c]chromen-3-ol	6-pyruvoyl tetrahydrobiopterin synthase	Q03393	PTS	0.021
Astragali Radix（AgR）	MOL000380	(6aR,11aR)-9,10-dimethoxy-6a,11a-dihydro-6H-benzofurano[3,2-c]chromen-3-ol	Inhibitor of nuclear factor kappa-B kinase subunit beta	O14920	IKBKB	0.022
Astragali Radix（AgR）	MOL000380	(6aR,11aR)-9,10-dimethoxy-6a,11a-dihydro-6H-benzofurano[3,2-c]chromen-3-ol	Ig kappa chain C region	P01834	IGKC	0.022
Astragali Radix（AgR）	MOL000380	(6aR,11aR)-9,10-dimethoxy-6a,11a-dihydro-6H-benzofurano[3,2-c]chromen-3-ol	Ig gamma-1 chain C region	P01857	IGHG1	0.022
Astragali Radix（AgR）	MOL000380	(6aR,11aR)-9,10-dimethoxy-6a,11a-dihydro-6H-benzofurano[3,2-c]chromen-3-ol	Peroxisome proliferator-activated receptor gamma	P37231	PPARG	0.022
Astragali Radix（AgR）	MOL000380	(6aR,11aR)-9,10-dimethoxy-6a,11a-dihydro-6H-benzofurano[3,2-c]chromen-3-ol	Group IIE secretory phospholipase A2	Q9NZK7	PLA2G2E	0.022
Astragali Radix（AgR）	MOL000380	(6aR,11aR)-9,10-dimethoxy-6a,11a-dihydro-6H-benzofurano[3,2-c]chromen-3-ol	Cystine/glutamate transporter	Q9UPY5	SLC7A11	0.022
Astragali Radix（AgR）	MOL000380	(6aR,11aR)-9,10-dimethoxy-6a,11a-dihydro-6H-benzofurano[3,2-c]chromen-3-ol	Muscarinic acetylcholine receptor M4	P08173	CHRM4	0.023
Astragali Radix（AgR）	MOL000380	(6aR,11aR)-9,10-dimethoxy-6a,11a-dihydro-6H-benzofurano[3,2-c]chromen-3-ol	D(1B) dopamine receptor	P21918	DRD5	0.023
Astragali Radix（AgR）	MOL000380	(6aR,11aR)-9,10-dimethoxy-6a,11a-dihydro-6H-benzofurano[3,2-c]chromen-3-ol	Sodium-dependent noradrenaline transporter	P23975	SLC6A2	0.023
Astragali Radix（AgR）	MOL000380	(6aR,11aR)-9,10-dimethoxy-6a,11a-dihydro-6H-benzofurano[3,2-c]chromen-3-ol	Alpha-1D adrenergic receptor	P25100	ADRA1D	0.023
Astragali Radix（AgR）	MOL000380	(6aR,11aR)-9,10-dimethoxy-6a,11a-dihydro-6H-benzofurano[3,2-c]chromen-3-ol	Peptidyl-prolyl cis-trans isomerase, mitochondrial	P30405	PPIF	0.023

Astragali Radix（AgR）	MOL000380	(6aR,11aR)-9,10-dimethoxy-6a,11a-dihydro-6H-benzofurano[3,2-c]chromen-3-ol	5-hydroxytryptamine 2B receptor	P41595	HTR2B	0.023
Astragali Radix（AgR）	MOL000380	(6aR,11aR)-9,10-dimethoxy-6a,11a-dihydro-6H-benzofurano[3,2-c]chromen-3-ol	Mitogen-activated protein kinase 8	P45983	MAPK8	0.023
Astragali Radix（AgR）	MOL000380	(6aR,11aR)-9,10-dimethoxy-6a,11a-dihydro-6H-benzofurano[3,2-c]chromen-3-ol	Mitogen-activated protein kinase 10	P53779	MAPK10	0.023
Astragali Radix（AgR）	MOL000380	(6aR,11aR)-9,10-dimethoxy-6a,11a-dihydro-6H-benzofurano[3,2-c]chromen-3-ol	Casein kinase II subunit alpha	P68400	CSNK2A1	0.023
Astragali Radix（AgR）	MOL000380	(6aR,11aR)-9,10-dimethoxy-6a,11a-dihydro-6H-benzofurano[3,2-c]chromen-3-ol	Tyrosine-protein kinase JAK2	O60674	JAK2	0.024
Astragali Radix（AgR）	MOL000380	(6aR,11aR)-9,10-dimethoxy-6a,11a-dihydro-6H-benzofurano[3,2-c]chromen-3-ol	Tyrosine-protein kinase JAK1	P23458	JAK1	0.024
Astragali Radix（AgR）	MOL000380	(6aR,11aR)-9,10-dimethoxy-6a,11a-dihydro-6H-benzofurano[3,2-c]chromen-3-ol	Tyrosine-protein kinase JAK3	P52333	JAK3	0.024
Astragali Radix（AgR）	MOL000380	(6aR,11aR)-9,10-dimethoxy-6a,11a-dihydro-6H-benzofurano[3,2-c]chromen-3-ol	Urokinase-type plasminogen activator	P00749	PLAU	0.025
Astragali Radix（AgR）	MOL000380	(6aR,11aR)-9,10-dimethoxy-6a,11a-dihydro-6H-benzofurano[3,2-c]chromen-3-ol	Myeloperoxidase	P05164	MPO	0.025
Astragali Radix（AgR）	MOL000380	(6aR,11aR)-9,10-dimethoxy-6a,11a-dihydro-6H-benzofurano[3,2-c]chromen-3-ol	Eosinophil peroxidase	P11678	EPX	0.025
Astragali Radix（AgR）	MOL000380	(6aR,11aR)-9,10-dimethoxy-6a,11a-dihydro-6H-benzofurano[3,2-c]chromen-3-ol	Macrophage migration inhibitory factor	P14174	MIF	0.025
Astragali Radix（AgR）	MOL000380	(6aR,11aR)-9,10-dimethoxy-6a,11a-dihydro-6H-benzofurano[3,2-c]chromen-3-ol	Calreticulin	P27797	CALR	0.025
Astragali Radix（AgR）	MOL000380	(6aR,11aR)-9,10-dimethoxy-6a,11a-dihydro-6H-benzofurano[3,2-c]chromen-3-ol	Peroxiredoxin-5, mitochondrial	P30044	PRDX5	0.025
Astragali Radix（AgR）	MOL000380	(6aR,11aR)-9,10-dimethoxy-6a,11a-dihydro-6H-benzofurano[3,2-c]chromen-3-ol	Melatonin receptor type 1B	P49286	MTNR1B	0.025
Astragali Radix（AgR）	MOL000380	(6aR,11aR)-9,10-dimethoxy-6a,11a-dihydro-6H-benzofurano[3,2-c]chromen-3-ol	Nuclear receptor ROR-beta	Q92753	RORB	0.025
Astragali Radix（AgR）	MOL000380	(6aR,11aR)-9,10-dimethoxy-6a,11a-dihydro-6H-benzofurano[3,2-c]chromen-3-ol	Sterol O-acyltransferase 2	O75908	SOAT2	0.027
Astragali Radix（AgR）	MOL000380	(6aR,11aR)-9,10-dimethoxy-6a,11a-dihydro-6H-benzofurano[3,2-c]chromen-3-ol	Sterol O-acyltransferase 1	P35610	SOAT1	0.027
Astragali Radix（AgR）	MOL000380	(6aR,11aR)-9,10-dimethoxy-6a,11a-dihydro-6H-benzofurano[3,2-c]chromen-3-ol	ATP synthase subunit beta, mitochondrial	P06576	ATP5F1B	0.028
Astragali Radix（AgR）	MOL000380	(6aR,11aR)-9,10-dimethoxy-6a,11a-dihydro-6H-benzofurano[3,2-c]chromen-3-ol	Tyrosine-protein kinase HCK	P08631	HCK	0.028
Astragali Radix（AgR）	MOL000380	(6aR,11aR)-9,10-dimethoxy-6a,11a-dihydro-6H-benzofurano[3,2-c]chromen-3-ol	ATP synthase subunit alpha, mitochondrial	P25705	ATP5F1A	0.028
Astragali Radix（AgR）	MOL000380	(6aR,11aR)-9,10-dimethoxy-6a,11a-dihydro-6H-benzofurano[3,2-c]chromen-3-ol	5-hydroxytryptamine 3 receptor	P46098	HTR3A	0.028
Astragali Radix（AgR）	MOL000380	(6aR,11aR)-9,10-dimethoxy-6a,11a-dihydro-6H-benzofurano[3,2-c]chromen-3-ol	Phosphatidylinositol-4,5-bisphosphate 3-kinase catalytic subunit gamma isoform	P48736	PIK3CG	0.028
Astragali Radix（AgR）	MOL000380	(6aR,11aR)-9,10-dimethoxy-6a,11a-dihydro-6H-benzofurano[3,2-c]chromen-3-ol	Potassium channel subfamily K member 1	O00180	KCNK1	0.029
Astragali Radix（AgR）	MOL000380	(6aR,11aR)-9,10-dimethoxy-6a,11a-dihydro-6H-benzofurano[3,2-c]chromen-3-ol	Corticosteroid 11-beta-dehydrogenase isozyme 1	P28845	HSD11B1	0.029
Astragali Radix（AgR）	MOL000380	(6aR,11aR)-9,10-dimethoxy-6a,11a-dihydro-6H-benzofurano[3,2-c]chromen-3-ol	Tubulin beta-2C chain	P68371	TUBB4B	0.029
Astragali Radix（AgR）	MOL000380	(6aR,11aR)-9,10-dimethoxy-6a,11a-dihydro-6H-benzofurano[3,2-c]chromen-3-ol	Potassium channel subfamily K member 6	Q9Y257	KCNK6	0.029
Astragali Radix（AgR）	MOL000380	(6aR,11aR)-9,10-dimethoxy-6a,11a-dihydro-6H-benzofurano[3,2-c]chromen-3-ol	Acetylcholinesterase	P22303	ACHE	0.03
Astragali Radix（AgR）	MOL000380	(6aR,11aR)-9,10-dimethoxy-6a,11a-dihydro-6H-benzofurano[3,2-c]chromen-3-ol	Gonadotropin-releasing hormone receptor	P30968	GNRHR	0.03
Astragali Radix（AgR）	MOL000380	(6aR,11aR)-9,10-dimethoxy-6a,11a-dihydro-6H-benzofurano[3,2-c]chromen-3-ol	Gonadotropin-releasing hormone II receptor	Q96P88	GNRHR2	0.03
Astragali Radix（AgR）	MOL000380	(6aR,11aR)-9,10-dimethoxy-6a,11a-dihydro-6H-benzofurano[3,2-c]chromen-3-ol	Microtubule-associated protein 2	P11137	MAP2	0.031
Astragali Radix（AgR）	MOL000380	(6aR,11aR)-9,10-dimethoxy-6a,11a-dihydro-6H-benzofurano[3,2-c]chromen-3-ol	Proto-oncogene serine/threonine-protein kinase Pim-1	P11309	PIM1	0.031

Astragali Radix（AgR）	MOL000380	(6aR,11aR)-9,10-dimethoxy-6a,11a-dihydro-6H-benzofurano[3,2-c]chromen-3-ol	3 beta-hydroxysteroid dehydrogenase/Delta 5-->4-isomerase type I	P14060	HSD3B1	0.031
Astragali Radix（AgR）	MOL000380	(6aR,11aR)-9,10-dimethoxy-6a,11a-dihydro-6H-benzofurano[3,2-c]chromen-3-ol	3 beta-hydroxysteroid dehydrogenase/Delta 5-->4-isomerase type II	P26439	HSD3B2	0.031
Astragali Radix（AgR）	MOL000380	(6aR,11aR)-9,10-dimethoxy-6a,11a-dihydro-6H-benzofurano[3,2-c]chromen-3-ol	Microtubule-associated protein 1A	P78559	MAP1A	0.031
Astragali Radix（AgR）	MOL000380	(6aR,11aR)-9,10-dimethoxy-6a,11a-dihydro-6H-benzofurano[3,2-c]chromen-3-ol	Casein kinase I isoform gamma-3	Q9Y6M4	CSNK1G3	0.031
Astragali Radix（AgR）	MOL000380	(6aR,11aR)-9,10-dimethoxy-6a,11a-dihydro-6H-benzofurano[3,2-c]chromen-3-ol	Tripartite motif-containing protein 13	O60858	TRIM13	0.032
Astragali Radix（AgR）	MOL000380	(6aR,11aR)-9,10-dimethoxy-6a,11a-dihydro-6H-benzofurano[3,2-c]chromen-3-ol	Muscarinic acetylcholine receptor M2	P08172	CHRM2	0.032
Astragali Radix（AgR）	MOL000380	(6aR,11aR)-9,10-dimethoxy-6a,11a-dihydro-6H-benzofurano[3,2-c]chromen-3-ol	Alpha-2A adrenergic receptor	P08913	ADRA2A	0.032
Astragali Radix（AgR）	MOL000380	(6aR,11aR)-9,10-dimethoxy-6a,11a-dihydro-6H-benzofurano[3,2-c]chromen-3-ol	Alpha-2B adrenergic receptor	P18089	ADRA2B	0.032
Astragali Radix（AgR）	MOL000380	(6aR,11aR)-9,10-dimethoxy-6a,11a-dihydro-6H-benzofurano[3,2-c]chromen-3-ol	Alpha-2C adrenergic receptor	P18825	ADRA2C	0.032
Astragali Radix（AgR）	MOL000380	(6aR,11aR)-9,10-dimethoxy-6a,11a-dihydro-6H-benzofurano[3,2-c]chromen-3-ol	5-hydroxytryptamine 2C receptor	P28335	HTR2C	0.032
Astragali Radix（AgR）	MOL000380	(6aR,11aR)-9,10-dimethoxy-6a,11a-dihydro-6H-benzofurano[3,2-c]chromen-3-ol	Gamma-aminobutyric-acid receptor subunit alpha-3	P34903	GABRA3	0.032
Astragali Radix（AgR）	MOL000380	(6aR,11aR)-9,10-dimethoxy-6a,11a-dihydro-6H-benzofurano[3,2-c]chromen-3-ol	Tryptophanyl-tRNA synthetase, mitochondrial	Q9UGM6	WARS2	0.033
Astragali Radix（AgR）	MOL000380	(6aR,11aR)-9,10-dimethoxy-6a,11a-dihydro-6H-benzofurano[3,2-c]chromen-3-ol	DNA topoisomerase 2-alpha	P11388	TOP2A	0.034
Astragali Radix（AgR）	MOL000380	(6aR,11aR)-9,10-dimethoxy-6a,11a-dihydro-6H-benzofurano[3,2-c]chromen-3-ol	Retinoic acid receptor RXR-alpha	P19793	RXRA	0.034
Astragali Radix（AgR）	MOL000380	(6aR,11aR)-9,10-dimethoxy-6a,11a-dihydro-6H-benzofurano[3,2-c]chromen-3-ol	Bile salt sulfotransferase	Q06520	SULT2A1	0.034
Astragali Radix（AgR）	MOL000380	(6aR,11aR)-9,10-dimethoxy-6a,11a-dihydro-6H-benzofurano[3,2-c]chromen-3-ol	Protein tyrosine kinase 2 beta	Q14289	PTK2B	0.034
Astragali Radix（AgR）	MOL000380	(6aR,11aR)-9,10-dimethoxy-6a,11a-dihydro-6H-benzofurano[3,2-c]chromen-3-ol	Nuclear receptor subfamily 1 group I member 3	Q14994	NR1I3	0.034
Astragali Radix（AgR）	MOL000380	(6aR,11aR)-9,10-dimethoxy-6a,11a-dihydro-6H-benzofurano[3,2-c]chromen-3-ol	Estradiol 17-beta-dehydrogenase 1	P14061	HSD17B1	0.035
Astragali Radix（AgR）	MOL000380	(6aR,11aR)-9,10-dimethoxy-6a,11a-dihydro-6H-benzofurano[3,2-c]chromen-3-ol	cAMP-dependent protein kinase inhibitor alpha	P61925	PKIA	0.035
Astragali Radix（AgR）	MOL000380	(6aR,11aR)-9,10-dimethoxy-6a,11a-dihydro-6H-benzofurano[3,2-c]chromen-3-ol	Rho-associated protein kinase 1	Q13464	ROCK1	0.035
Astragali Radix（AgR）	MOL000380	(6aR,11aR)-9,10-dimethoxy-6a,11a-dihydro-6H-benzofurano[3,2-c]chromen-3-ol	5-hydroxytryptamine 1A receptor	P08908	HTR1A	0.036
Astragali Radix（AgR）	MOL000380	(6aR,11aR)-9,10-dimethoxy-6a,11a-dihydro-6H-benzofurano[3,2-c]chromen-3-ol	Phospholipase A2, membrane associated	P14555	PLA2G2A	0.036
Astragali Radix（AgR）	MOL000380	(6aR,11aR)-9,10-dimethoxy-6a,11a-dihydro-6H-benzofurano[3,2-c]chromen-3-ol	Platelet glycoprotein IX	P14770	GP9	0.037
Astragali Radix（AgR）	MOL000380	(6aR,11aR)-9,10-dimethoxy-6a,11a-dihydro-6H-benzofurano[3,2-c]chromen-3-ol	Estrogen-related receptor gamma	P62508	ESRRG	0.037
Astragali Radix（AgR）	MOL000380	(6aR,11aR)-9,10-dimethoxy-6a,11a-dihydro-6H-benzofurano[3,2-c]chromen-3-ol	D-HSCDK2	O75100	CA11	0.038
Astragali Radix（AgR）	MOL000380	(6aR,11aR)-9,10-dimethoxy-6a,11a-dihydro-6H-benzofurano[3,2-c]chromen-3-ol	Epidermal growth factor receptor	P00533	EGFR	0.038
Astragali Radix（AgR）	MOL000380	(6aR,11aR)-9,10-dimethoxy-6a,11a-dihydro-6H-benzofurano[3,2-c]chromen-3-ol	Gamma-aminobutyric-acid receptor subunit alpha-1	P14867	GABRA1	0.038
Astragali Radix（AgR）	MOL000380	(6aR,11aR)-9,10-dimethoxy-6a,11a-dihydro-6H-benzofurano[3,2-c]chromen-3-ol	Gamma-aminobutyric-acid receptor subunit alpha-5	P31644	GABRA5	0.038
Astragali Radix（AgR）	MOL000380	(6aR,11aR)-9,10-dimethoxy-6a,11a-dihydro-6H-benzofurano[3,2-c]chromen-3-ol	Cell division protein kinase 6	Q00534	CDK6	0.038
Astragali Radix（AgR）	MOL000380	(6aR,11aR)-9,10-dimethoxy-6a,11a-dihydro-6H-benzofurano[3,2-c]chromen-3-ol	Muscarinic acetylcholine receptor M1	P11229	CHRM1	0.041
Astragali Radix（AgR）	MOL000380	(6aR,11aR)-9,10-dimethoxy-6a,11a-dihydro-6H-benzofurano[3,2-c]chromen-3-ol	Muscarinic acetylcholine receptor M3	P20309	CHRM3	0.041

Astragali Radix（AgR）	MOL000380	(6aR,11aR)-9,10-dimethoxy-6a,11a-dihydro-6H-benzofurano[3,2-c]chromen-3-ol	5-hydroxytryptamine 2A receptor	P28223	HTR2A	0.041
Astragali Radix（AgR）	MOL000380	(6aR,11aR)-9,10-dimethoxy-6a,11a-dihydro-6H-benzofurano[3,2-c]chromen-3-ol	Sodium-dependent serotonin transporter	P31645	SLC6A4	0.041
Astragali Radix（AgR）	MOL000380	(6aR,11aR)-9,10-dimethoxy-6a,11a-dihydro-6H-benzofurano[3,2-c]chromen-3-ol	Alpha-1B adrenergic receptor	P35368	ADRA1B	0.041
Astragali Radix（AgR）	MOL000380	(6aR,11aR)-9,10-dimethoxy-6a,11a-dihydro-6H-benzofurano[3,2-c]chromen-3-ol	Gamma-aminobutyric-acid receptor subunit alpha-2	P47869	GABRA2	0.044
Astragali Radix（AgR）	MOL000380	(6aR,11aR)-9,10-dimethoxy-6a,11a-dihydro-6H-benzofurano[3,2-c]chromen-3-ol	Ig kappa chain V-II region RPMI 6410	P06310	IGKV2-30	0.045
Astragali Radix（AgR）	MOL000380	(6aR,11aR)-9,10-dimethoxy-6a,11a-dihydro-6H-benzofurano[3,2-c]chromen-3-ol	Fibroblast growth factor receptor 2	P21802	FGFR2	0.045
Astragali Radix（AgR）	MOL000380	(6aR,11aR)-9,10-dimethoxy-6a,11a-dihydro-6H-benzofurano[3,2-c]chromen-3-ol	Activin receptor type-1	Q04771	ACVR1	0.045
Astragali Radix（AgR）	MOL000380	(6aR,11aR)-9,10-dimethoxy-6a,11a-dihydro-6H-benzofurano[3,2-c]chromen-3-ol	Oxysterols receptor LXR-alpha	Q13133	NR1H3	0.045
Astragali Radix（AgR）	MOL000380	(6aR,11aR)-9,10-dimethoxy-6a,11a-dihydro-6H-benzofurano[3,2-c]chromen-3-ol	Geranylgeranyl pyrophosphate synthetase	O95749	GGPS1	0.046
Astragali Radix（AgR）	MOL000380	(6aR,11aR)-9,10-dimethoxy-6a,11a-dihydro-6H-benzofurano[3,2-c]chromen-3-ol	Carbonic anhydrase 2	P00918	CA2	0.046
Astragali Radix（AgR）	MOL000380	(6aR,11aR)-9,10-dimethoxy-6a,11a-dihydro-6H-benzofurano[3,2-c]chromen-3-ol	Hepatocyte growth factor receptor	P08581	MET	0.046
Astragali Radix（AgR）	MOL000380	(6aR,11aR)-9,10-dimethoxy-6a,11a-dihydro-6H-benzofurano[3,2-c]chromen-3-ol	Leukotriene A-4 hydrolase	P09960	LTA4H	0.046
Astragali Radix（AgR）	MOL000380	(6aR,11aR)-9,10-dimethoxy-6a,11a-dihydro-6H-benzofurano[3,2-c]chromen-3-ol	Thyroid hormone receptor alpha	P10827	THRA	0.046
Astragali Radix（AgR）	MOL000380	(6aR,11aR)-9,10-dimethoxy-6a,11a-dihydro-6H-benzofurano[3,2-c]chromen-3-ol	Cytochrome P450 19A1	P11511	CYP19A1	0.046
Astragali Radix（AgR）	MOL000380	(6aR,11aR)-9,10-dimethoxy-6a,11a-dihydro-6H-benzofurano[3,2-c]chromen-3-ol	Nitric oxide synthase, inducible	P35228	NOS2	0.046
Astragali Radix（AgR）	MOL000380	(6aR,11aR)-9,10-dimethoxy-6a,11a-dihydro-6H-benzofurano[3,2-c]chromen-3-ol	ATP-sensitive inward rectifier potassium channel 1	P48048	KCNJ1	0.047
Astragali Radix（AgR）	MOL000380	(6aR,11aR)-9,10-dimethoxy-6a,11a-dihydro-6H-benzofurano[3,2-c]chromen-3-ol	Thyroid hormone receptor, alpha isoform 1 variant	Q59FW3	SIGMAR1	0.047
Astragali Radix（AgR）	MOL000380	(6aR,11aR)-9,10-dimethoxy-6a,11a-dihydro-6H-benzofurano[3,2-c]chromen-3-ol	Casein kinase I isoform gamma-1	Q9HCP0	CSNK1G1	0.047
Astragali Radix（AgR）	MOL000380	(6aR,11aR)-9,10-dimethoxy-6a,11a-dihydro-6H-benzofurano[3,2-c]chromen-3-ol	Toll-like receptor 7	Q9NYK1	TLR7	0.047
Astragali Radix（AgR）	MOL000380	(6aR,11aR)-9,10-dimethoxy-6a,11a-dihydro-6H-benzofurano[3,2-c]chromen-3-ol	Inhibitor of nuclear factor kappa-B kinase subunit alpha	O15111	CHUK	0.048
Astragali Radix（AgR）	MOL000380	(6aR,11aR)-9,10-dimethoxy-6a,11a-dihydro-6H-benzofurano[3,2-c]chromen-3-ol	Glucocorticoid receptor	P04150	NR3C1	0.048
Astragali Radix（AgR）	MOL000380	(6aR,11aR)-9,10-dimethoxy-6a,11a-dihydro-6H-benzofurano[3,2-c]chromen-3-ol	Cannabinoid receptor 1	P21554	CNR1	0.048
Astragali Radix（AgR）	MOL000380	(6aR,11aR)-9,10-dimethoxy-6a,11a-dihydro-6H-benzofurano[3,2-c]chromen-3-ol	Tubulin beta-1 chain	Q9H4B7	TUBB1	0.048
Astragali Radix（AgR）	MOL000380	(6aR,11aR)-9,10-dimethoxy-6a,11a-dihydro-6H-benzofurano[3,2-c]chromen-3-ol	DNA polymerase kappa	Q9UBT6	POLK	0.048
Astragali Radix（AgR）	MOL000380	(6aR,11aR)-9,10-dimethoxy-6a,11a-dihydro-6H-benzofurano[3,2-c]chromen-3-ol	Aldo-keto reductase family 1 member C1	Q04828	AKR1C1	0.049
Astragali Radix（AgR）	MOL000380	(6aR,11aR)-9,10-dimethoxy-6a,11a-dihydro-6H-benzofurano[3,2-c]chromen-3-ol	C-jun-amino-terminal kinase-interacting protein 1	Q9UQF2	MAPK8IP1	0.049
Astragali Radix（AgR）	MOL000380	(6aR,11aR)-9,10-dimethoxy-6a,11a-dihydro-6H-benzofurano[3,2-c]chromen-3-ol	Dihydrofolate reductase	P00374	DHFR	0.05
Astragali Radix（AgR）	MOL000380	(6aR,11aR)-9,10-dimethoxy-6a,11a-dihydro-6H-benzofurano[3,2-c]chromen-3-ol	Heat shock protein HSP 90-beta	P08238	HSP90AB1	0.05
Astragali Radix（AgR）	MOL000380	(6aR,11aR)-9,10-dimethoxy-6a,11a-dihydro-6H-benzofurano[3,2-c]chromen-3-ol	D(2) dopamine receptor	P14416	DRD2	0.05
Astragali Radix（AgR）	MOL000380	(6aR,11aR)-9,10-dimethoxy-6a,11a-dihydro-6H-benzofurano[3,2-c]chromen-3-ol	Inosine-5'-monophosphate dehydrogenase 1	P20839	IMPDH1	0.05
Astragali Radix（AgR）	MOL000380	(6aR,11aR)-9,10-dimethoxy-6a,11a-dihydro-6H-benzofurano[3,2-c]chromen-3-ol	Alpha-1A adrenergic receptor	P35348	ADRA1A	0.05

Astragali Radix（AgR）	MOL000380	(6aR,11aR)-9,10-dimethoxy-6a,11a-dihydro-6H-benzofurano[3,2-c]chromen-3-ol	Thiamin pyrophosphokinase 1	Q9H3S4	TPK1	0.05
Astragali Radix（AgR）	MOL000380	(6aR,11aR)-9,10-dimethoxy-6a,11a-dihydro-6H-benzofurano[3,2-c]chromen-3-ol	Phospholipase A2	P04054	PLA2G1B	0.051
Astragali Radix（AgR）	MOL000380	(6aR,11aR)-9,10-dimethoxy-6a,11a-dihydro-6H-benzofurano[3,2-c]chromen-3-ol	Neutrophil gelatinase-associated lipocalin	P80188	LCN2	0.051
Astragali Radix（AgR）	MOL000380	(6aR,11aR)-9,10-dimethoxy-6a,11a-dihydro-6H-benzofurano[3,2-c]chromen-3-ol	Peroxisome proliferator-activated receptor alpha	Q07869	PPARA	0.051
Astragali Radix（AgR）	MOL000380	(6aR,11aR)-9,10-dimethoxy-6a,11a-dihydro-6H-benzofurano[3,2-c]chromen-3-ol	Ribosyldihydronicotinamide dehydrogenase [quinone]	P16083	NQO2	0.052
Astragali Radix（AgR）	MOL000380	(6aR,11aR)-9,10-dimethoxy-6a,11a-dihydro-6H-benzofurano[3,2-c]chromen-3-ol	MAP kinase-activated protein kinase 2	P49137	MAPKAPK2	0.052
Astragali Radix（AgR）	MOL000380	(6aR,11aR)-9,10-dimethoxy-6a,11a-dihydro-6H-benzofurano[3,2-c]chromen-3-ol	Interferon gamma	P01579	IFNG	0.053
Astragali Radix（AgR）	MOL000380	(6aR,11aR)-9,10-dimethoxy-6a,11a-dihydro-6H-benzofurano[3,2-c]chromen-3-ol	Triosephosphate isomerase	P60174	TPI1	0.053
Astragali Radix（AgR）	MOL000380	(6aR,11aR)-9,10-dimethoxy-6a,11a-dihydro-6H-benzofurano[3,2-c]chromen-3-ol	cAMP-specific 3',5'-cyclic phosphodiesterase 4D	Q08499	PDE4D	0.053
Astragali Radix（AgR）	MOL000380	(6aR,11aR)-9,10-dimethoxy-6a,11a-dihydro-6H-benzofurano[3,2-c]chromen-3-ol	Melatonin receptor type 1A	P48039	MTNR1A	0.054
Astragali Radix（AgR）	MOL000380	(6aR,11aR)-9,10-dimethoxy-6a,11a-dihydro-6H-benzofurano[3,2-c]chromen-3-ol	Calmodulin	P62158		0.054
Astragali Radix（AgR）	MOL000380	(6aR,11aR)-9,10-dimethoxy-6a,11a-dihydro-6H-benzofurano[3,2-c]chromen-3-ol	Nuclear receptor coactivator 2	Q15596	NCOA2	0.054
Astragali Radix（AgR）	MOL000380	(6aR,11aR)-9,10-dimethoxy-6a,11a-dihydro-6H-benzofurano[3,2-c]chromen-3-ol	Thymidylate synthase	P04818	TYMS	0.055
Astragali Radix（AgR）	MOL000380	(6aR,11aR)-9,10-dimethoxy-6a,11a-dihydro-6H-benzofurano[3,2-c]chromen-3-ol	Tyrosyl-tRNA synthetase, cytoplasmic	P54577	YARS	0.055
Astragali Radix（AgR）	MOL000380	(6aR,11aR)-9,10-dimethoxy-6a,11a-dihydro-6H-benzofurano[3,2-c]chromen-3-ol	Nuclear receptor coactivator 5	Q9HCD5	NCOA5	0.056
Astragali Radix（AgR）	MOL000380	(6aR,11aR)-9,10-dimethoxy-6a,11a-dihydro-6H-benzofurano[3,2-c]chromen-3-ol	Mitogen-activated protein kinase 14	Q16539	MAPK14	0.058
Astragali Radix（AgR）	MOL000380	(6aR,11aR)-9,10-dimethoxy-6a,11a-dihydro-6H-benzofurano[3,2-c]chromen-3-ol	Cell division control protein 2 homolog	P06493	CDK1	0.059
Astragali Radix（AgR）	MOL000380	(6aR,11aR)-9,10-dimethoxy-6a,11a-dihydro-6H-benzofurano[3,2-c]chromen-3-ol	D(1A) dopamine receptor	P21728	DRD1	0.059
Astragali Radix（AgR）	MOL000380	(6aR,11aR)-9,10-dimethoxy-6a,11a-dihydro-6H-benzofurano[3,2-c]chromen-3-ol	Tubulin alpha-3 chain	Q71U36	TUBA1A	0.062
Astragali Radix（AgR）	MOL000380	(6aR,11aR)-9,10-dimethoxy-6a,11a-dihydro-6H-benzofurano[3,2-c]chromen-3-ol	Proto-oncogene tyrosine-protein kinase Src	P12931	SRC	0.066
Astragali Radix（AgR）	MOL000380	(6aR,11aR)-9,10-dimethoxy-6a,11a-dihydro-6H-benzofurano[3,2-c]chromen-3-ol	Arachidonate 5-lipoxygenase	P09917	ALOX5	0.073
Astragali Radix（AgR）	MOL000380	(6aR,11aR)-9,10-dimethoxy-6a,11a-dihydro-6H-benzofurano[3,2-c]chromen-3-ol	Cell division protein kinase 5	Q00535	CDK5	0.081
Astragali Radix（AgR）	MOL000380	(6aR,11aR)-9,10-dimethoxy-6a,11a-dihydro-6H-benzofurano[3,2-c]chromen-3-ol	cAMP-specific 3',5'-cyclic phosphodiesterase 4B	Q07343	PDE4B	0.083
Astragali Radix（AgR）	MOL000380	(6aR,11aR)-9,10-dimethoxy-6a,11a-dihydro-6H-benzofurano[3,2-c]chromen-3-ol	Delta-type opioid receptor	P41143	OPRD1	0.087
Astragali Radix（AgR）	MOL000380	(6aR,11aR)-9,10-dimethoxy-6a,11a-dihydro-6H-benzofurano[3,2-c]chromen-3-ol	Sodium channel protein type 5 subunit alpha	Q14524	SCN5A	0.096
Astragali Radix（AgR）	MOL000380	(6aR,11aR)-9,10-dimethoxy-6a,11a-dihydro-6H-benzofurano[3,2-c]chromen-3-ol	Mineralocorticoid receptor	P08235	NR3C2	0.097
Astragali Radix（AgR）	MOL000380	(6aR,11aR)-9,10-dimethoxy-6a,11a-dihydro-6H-benzofurano[3,2-c]chromen-3-ol	Tyrosine-protein phosphatase non-receptor type 1	P18031	PTPN1	0.097
Astragali Radix（AgR）	MOL000380	(6aR,11aR)-9,10-dimethoxy-6a,11a-dihydro-6H-benzofurano[3,2-c]chromen-3-ol	Coagulation factor VII	P08709	F7	0.098
Astragali Radix（AgR）	MOL000380	(6aR,11aR)-9,10-dimethoxy-6a,11a-dihydro-6H-benzofurano[3,2-c]chromen-3-ol	Androgen receptor	P10275	AR	0.098
Astragali Radix（AgR）	MOL000380	(6aR,11aR)-9,10-dimethoxy-6a,11a-dihydro-6H-benzofurano[3,2-c]chromen-3-ol	Prostaglandin G/H synthase 1	P23219	PTGS1	0.102
Astragali Radix（AgR）	MOL000380	(6aR,11aR)-9,10-dimethoxy-6a,11a-dihydro-6H-benzofurano[3,2-c]chromen-3-ol	cAMP-specific 3',5'-cyclic phosphodiesterase 4A	P27815	PDE4A	0.105

Astragali Radix（AgR）	MOL000380	(6aR,11aR)-9,10-dimethoxy-6a,11a-dihydro-6H-benzofurano[3,2-c]chromen-3-ol	RAC-alpha serine/threonine-protein kinase	P31749	AKT1	0.105
Astragali Radix（AgR）	MOL000380	(6aR,11aR)-9,10-dimethoxy-6a,11a-dihydro-6H-benzofurano[3,2-c]chromen-3-ol	Hydroxyacid oxidase 1	Q9UJM8	HAO1	0.106
Astragali Radix（AgR）	MOL000380	(6aR,11aR)-9,10-dimethoxy-6a,11a-dihydro-6H-benzofurano[3,2-c]chromen-3-ol	Hemoglobin subunit alpha	P69905	HBA1	0.121
Astragali Radix（AgR）	MOL000380	(6aR,11aR)-9,10-dimethoxy-6a,11a-dihydro-6H-benzofurano[3,2-c]chromen-3-ol	Kappa-type opioid receptor	P41145	OPRK1	0.123
Astragali Radix（AgR）	MOL000380	(6aR,11aR)-9,10-dimethoxy-6a,11a-dihydro-6H-benzofurano[3,2-c]chromen-3-ol	Cell division protein kinase 2	P24941	CDK2	0.16
Astragali Radix（AgR）	MOL000380	(6aR,11aR)-9,10-dimethoxy-6a,11a-dihydro-6H-benzofurano[3,2-c]chromen-3-ol	Prothrombin	P00734	F2	0.169
Astragali Radix（AgR）	MOL000380	(6aR,11aR)-9,10-dimethoxy-6a,11a-dihydro-6H-benzofurano[3,2-c]chromen-3-ol	Mu-type opioid receptor	P35372	OPRM1	0.19
Astragali Radix（AgR）	MOL000380	(6aR,11aR)-9,10-dimethoxy-6a,11a-dihydro-6H-benzofurano[3,2-c]chromen-3-ol	Nuclear receptor coactivator 1	Q15788	NCOA1	0.227
Astragali Radix（AgR）	MOL000380	(6aR,11aR)-9,10-dimethoxy-6a,11a-dihydro-6H-benzofurano[3,2-c]chromen-3-ol	Trypsin-1	P07477	PRSS1	0.23
Astragali Radix（AgR）	MOL000380	(6aR,11aR)-9,10-dimethoxy-6a,11a-dihydro-6H-benzofurano[3,2-c]chromen-3-ol	cAMP-dependent protein kinase catalytic subunit alpha	P17612	PRKACA	0.233
Astragali Radix（AgR）	MOL000380	(6aR,11aR)-9,10-dimethoxy-6a,11a-dihydro-6H-benzofurano[3,2-c]chromen-3-ol	Progesterone receptor	P06401	PGR	0.234
Astragali Radix（AgR）	MOL000380	(6aR,11aR)-9,10-dimethoxy-6a,11a-dihydro-6H-benzofurano[3,2-c]chromen-3-ol	Prostaglandin G/H synthase 2	P35354	PTGS2	0.335
Astragali Radix（AgR）	MOL000380	(6aR,11aR)-9,10-dimethoxy-6a,11a-dihydro-6H-benzofurano[3,2-c]chromen-3-ol	Estrogen receptor beta	Q92731	ESR2	0.391
Astragali Radix（AgR）	MOL000380	(6aR,11aR)-9,10-dimethoxy-6a,11a-dihydro-6H-benzofurano[3,2-c]chromen-3-ol	Cyclin-A2	P20248	CCNA2	0.403
Astragali Radix（AgR）	MOL000380	(6aR,11aR)-9,10-dimethoxy-6a,11a-dihydro-6H-benzofurano[3,2-c]chromen-3-ol	Estrogen receptor	P03372	ESR1	1
Astragali Radix（AgR）	MOL000387	Bifendate	Muscarinic acetylcholine receptor M1	P11229	CHRM1	0.014
Astragali Radix（AgR）	MOL000387	Bifendate	Amine oxidase [flavin-containing] A	P21397	MAOA	0.014
Astragali Radix（AgR）	MOL000387	Bifendate	5-hydroxytryptamine 2A receptor	P28223	HTR2A	0.014
Astragali Radix（AgR）	MOL000387	Bifendate	Alpha-1A adrenergic receptor	P35348	ADRA1A	0.014
Astragali Radix（AgR）	MOL000387	Bifendate	Chromaffin granule amine transporter	P54219	SLC18A1	0.014
Astragali Radix（AgR）	MOL000387	Bifendate	Synaptic vesicular amine transporter	Q05940	SLC18A2	0.014
Astragali Radix（AgR）	MOL000387	Bifendate	2,4-dienoyl-CoA reductase, mitochondrial	Q16698	DECR1	0.02
Astragali Radix（AgR）	MOL000387	Bifendate	Alpha-2B adrenergic receptor	P18089	ADRA2B	0.023
Astragali Radix（AgR）	MOL000387	Bifendate	Alpha-2C adrenergic receptor	P18825	ADRA2C	0.023
Astragali Radix（AgR）	MOL000387	Bifendate	D(1A) dopamine receptor	P21728	DRD1	0.023
Astragali Radix（AgR）	MOL000387	Bifendate	D(1B) dopamine receptor	P21918	DRD5	0.023
Astragali Radix（AgR）	MOL000387	Bifendate	Elongation factor 2	P13639	EEF2	0.024
Astragali Radix（AgR）	MOL000387	Bifendate	Poly [ADP-ribose] polymerase 3	Q9Y6F1	PARP3	0.024
Astragali Radix（AgR）	MOL000387	Bifendate	Gamma-aminobutyric-acid receptor subunit beta-3	P28472	GABRB3	0.027
Astragali Radix（AgR）	MOL000387	Bifendate	Tubulin alpha-3 chain	Q71U36	TUBA1A	0.027
Astragali Radix（AgR）	MOL000387	Bifendate	Tubulin alpha-6 chain	Q9BQE3	TUBA1C	0.027
Astragali Radix（AgR）	MOL000387	Bifendate	Tubulin beta-2B chain	Q9BVA1	TUBB2B	0.027
Astragali Radix（AgR）	MOL000387	Bifendate	Stathmin-4	Q9H169	STMN4	0.027
Astragali Radix（AgR）	MOL000387	Bifendate	Translocator protein	P30536	TSPO	0.036
Astragali Radix（AgR）	MOL000387	Bifendate	Sodium-dependent noradrenaline transporter	P23975	SLC6A2	0.037
Astragali Radix（AgR）	MOL000387	Bifendate	Sodium-dependent serotonin transporter	P31645	SLC6A4	0.037
Astragali Radix（AgR）	MOL000387	Bifendate	Sodium-dependent dopamine transporter	Q01959	SLC6A3	0.037
Astragali Radix（AgR）	MOL000387	Bifendate	Mitogen-activated protein kinase 8	P45983	MAPK8	0.038
Astragali Radix（AgR）	MOL000387	Bifendate	Mitogen-activated protein kinase 10	P53779	MAPK10	0.038

Astragali Radix（AgR）	MOL000387	Bifendate	C-jun-amino-terminal kinase-interacting protein 1	Q9UQF2	MAPK8IP1	0.038
Astragali Radix（AgR）	MOL000387	Bifendate	cAMP-specific 3',5'-cyclic phosphodiesterase 4A	P27815	PDE4A	0.041
Astragali Radix（AgR）	MOL000387	Bifendate	cGMP-inhibited 3',5'-cyclic phosphodiesterase A	Q14432	PDE3A	0.041
Astragali Radix（AgR）	MOL000387	Bifendate	cAMP and cAMP-inhibited cGMP 3',5'-cyclic phosphodiesterase 10A	Q9Y233	PDE10A	0.041
Astragali Radix（AgR）	MOL000387	Bifendate	Gamma-aminobutyric-acid receptor subunit alpha-6	Q16445	GABRA6	0.054
Astragali Radix（AgR）	MOL000387	Bifendate	Substance-P receptor	P25103	TACR1	0.057
Astragali Radix（AgR）	MOL000387	Bifendate	Glutamate [NMDA] receptor subunit 3A	Q8TCU5	GRIN3A	0.057
Astragali Radix（AgR）	MOL000387	Bifendate	Mu-type opioid receptor	P35372	OPRM1	0.058
Astragali Radix（AgR）	MOL000387	Bifendate	Delta-type opioid receptor	P41143	OPRD1	0.058
Astragali Radix（AgR）	MOL000387	Bifendate	Sterol O-acyltransferase 2	O75908	SOAT2	0.061
Astragali Radix（AgR）	MOL000387	Bifendate	Sterol O-acyltransferase 1	P35610	SOAT1	0.061
Astragali Radix（AgR）	MOL000387	Bifendate	Prothrombin	P00734	F2	0.062
Astragali Radix（AgR）	MOL000387	Bifendate	Macrophage migration inhibitory factor	P14174	MIF	0.062
Astragali Radix（AgR）	MOL000387	Bifendate	Gamma-aminobutyric-acid receptor subunit beta-2	P47870	GABRB2	0.062
Astragali Radix（AgR）	MOL000387	Bifendate	Gamma-aminobutyric-acid receptor subunit alpha-4	P48169	GABRA4	0.062
Astragali Radix（AgR）	MOL000387	Bifendate	Gamma-aminobutyric acid receptor subunit theta	Q9UN88	GABRQ	0.062
Astragali Radix（AgR）	MOL000387	Bifendate	Proto-oncogene tyrosine-protein kinase LCK	P06239	LCK	0.064
Astragali Radix（AgR）	MOL000387	Bifendate	Tyrosine-protein kinase Lyn	P07948	LYN	0.064
Astragali Radix（AgR）	MOL000387	Bifendate	Gamma-aminobutyric acid receptor subunit gamma-2	P18507	GABRG2	0.08
Astragali Radix（AgR）	MOL000387	Bifendate	Phospholipase A2	P04054	PLA2G1B	0.081
Astragali Radix（AgR）	MOL000387	Bifendate	Aldo-keto reductase family 1 member C3	P42330	AKR1C3	0.081
Astragali Radix（AgR）	MOL000387	Bifendate	Group IIE secretory phospholipase A2	Q9NZK7	PLA2G2E	0.081
Astragali Radix（AgR）	MOL000387	Bifendate	Gamma-aminobutyric acid receptor subunit rho-3	A8MPY1	GABRR3	0.089
Astragali Radix（AgR）	MOL000387	Bifendate	Gamma-aminobutyric acid receptor subunit pi	O00591	GABRP	0.089
Astragali Radix（AgR）	MOL000387	Bifendate	Gamma-aminobutyric acid receptor subunit delta	O14764	GABRD	0.089
Astragali Radix（AgR）	MOL000387	Bifendate	Gamma-aminobutyric-acid receptor subunit beta-1	P18505	GABRB1	0.089
Astragali Radix（AgR）	MOL000387	Bifendate	Gamma-aminobutyric-acid receptor subunit rho-1	P24046	GABRR1	0.089
Astragali Radix（AgR）	MOL000387	Bifendate	Gamma-aminobutyric acid receptor subunit rho-2	P28476	GABRR2	0.089
Astragali Radix（AgR）	MOL000387	Bifendate	Gamma-aminobutyric acid receptor subunit epsilon	P78334	GABRE	0.089
Astragali Radix（AgR）	MOL000387	Bifendate	Gamma-aminobutyric acid receptor subunit gamma-1	Q8N1C3	GABRG1	0.089
Astragali Radix（AgR）	MOL000387	Bifendate	Gamma-aminobutyric acid receptor subunit gamma-3	Q99928	GABRG3	0.089
Astragali Radix（AgR）	MOL000387	Bifendate	Gamma-aminobutyric-acid receptor subunit alpha-1	P14867	GABRA1	0.098
Astragali Radix（AgR）	MOL000387	Bifendate	Gamma-aminobutyric-acid receptor subunit alpha-5	P31644	GABRA5	0.098
Astragali Radix（AgR）	MOL000387	Bifendate	Gamma-aminobutyric-acid receptor subunit alpha-3	P34903	GABRA3	0.098
Astragali Radix（AgR）	MOL000387	Bifendate	DNA topoisomerase 2-alpha	P11388	TOP2A	0.099
Astragali Radix（AgR）	MOL000387	Bifendate	Protein tyrosine kinase 2 beta	Q14289	PTK2B	0.099
Astragali Radix（AgR）	MOL000387	Bifendate	Gamma-aminobutyric-acid receptor subunit alpha-2	P47869	GABRA2	0.107
Astragali Radix（AgR）	MOL000387	Bifendate	Thyroid hormone receptor beta-1	P10828	THRB	0.122
Astragali Radix（AgR）	MOL000387	Bifendate	Coagulation factor VII	P08709	F7	0.123
Astragali Radix（AgR）	MOL000387	Bifendate	Sodium channel protein type 5 subunit alpha	Q14524	SCN5A	0.123
Astragali Radix（AgR）	MOL000387	Bifendate	Nuclear receptor coactivator 5	Q9HCD5	NCOA5	0.124
Astragali Radix（AgR）	MOL000387	Bifendate	Cytochrome P450 11B1, mitochondrial	P15538	CYP11B1	0.128
Astragali Radix（AgR）	MOL000387	Bifendate	Cell division protein kinase 2	P24941	CDK2	0.13
Astragali Radix（AgR）	MOL000387	Bifendate	Thymidylate synthase	P04818	TYMS	0.132
Astragali Radix（AgR）	MOL000387	Bifendate	cAMP-specific 3',5'-cyclic phosphodiesterase 4B	Q07343	PDE4B	0.137
Astragali Radix（AgR）	MOL000387	Bifendate	cAMP-specific 3',5'-cyclic phosphodiesterase 4D	Q08499	PDE4D	0.137
Astragali Radix（AgR）	MOL000387	Bifendate	RAC-alpha serine/threonine-protein kinase	P31749	AKT1	0.138

Astragali Radix（AgR）	MOL000387	Bifendate	Proto-oncogene serine/threonine-protein kinase Pim-1	P11309	PIM1	0.139
Astragali Radix（AgR）	MOL000387	Bifendate	Tyrosine-protein kinase HCK	P08631	HCK	0.14
Astragali Radix（AgR）	MOL000387	Bifendate	Cell division protein kinase 5	Q00535	CDK5	0.142
Astragali Radix（AgR）	MOL000387	Bifendate	Tubulin beta-1 chain	Q9H4B7	TUBB1	0.149
Astragali Radix（AgR）	MOL000387	Bifendate	Phosphatidylinositol-4,5-bisphosphate 3-kinase catalytic subunit gamma isoform	P48736	PIK3CG	0.151
Astragali Radix（AgR）	MOL000387	Bifendate	Prostaglandin G/H synthase 1	P23219	PTGS1	0.259
Astragali Radix（AgR）	MOL000387	Bifendate	Cyclin-A2	P20248	CCNA2	0.278
Astragali Radix（AgR）	MOL000387	Bifendate	Nuclear receptor coactivator 1	Q15788	NCOA1	0.413
Astragali Radix（AgR）	MOL000387	Bifendate	cAMP-dependent protein kinase catalytic subunit alpha	P17612	PRKACA	0.546
Astragali Radix（AgR）	MOL000387	Bifendate	Estrogen receptor beta	Q92731	ESR2	0.621
Astragali Radix（AgR）	MOL000387	Bifendate	Prostaglandin G/H synthase 2	P35354	PTGS2	0.706
Astragali Radix（AgR）	MOL000387	Bifendate	Estrogen receptor	P03372	ESR1	1
Astragali Radix（AgR）	MOL000392	formononetin	Carbonic anhydrase 4	P22748	CA4	0.01
Astragali Radix（AgR）	MOL000392	formononetin	Carbonic anhydrase 9	Q16790	CA9	0.01
Astragali Radix（AgR）	MOL000392	formononetin	Sodium channel protein type 10 subunit alpha	Q9Y5Y9	SCN10A	0.01
Astragali Radix（AgR）	MOL000392	formononetin	Glutathione S-transferase P	P09211	GSTP1	0.011
Astragali Radix（AgR）	MOL000392	formononetin	Delta-type opioid receptor	P41143	OPRD1	0.011
Astragali Radix（AgR）	MOL000392	formononetin	Kappa-type opioid receptor	P41145	OPRK1	0.011
Astragali Radix（AgR）	MOL000392	formononetin	Alpha-7 nicotinic cholinergic receptor subunit	Q693P7	CHRNA7	0.011
Astragali Radix（AgR）	MOL000392	formononetin	Histamine H2 receptor	P25021	HRH2	0.012
Astragali Radix（AgR）	MOL000392	formononetin	Alpha-1D adrenergic receptor	P25100	ADRA1D	0.012
Astragali Radix（AgR）	MOL000392	formononetin	5-hydroxytryptamine 7 receptor	P34969	HTR7	0.012
Astragali Radix（AgR）	MOL000392	formononetin	Alpha-1B adrenergic receptor	P35368	ADRA1B	0.012
Astragali Radix（AgR）	MOL000392	formononetin	Glutamate receptor 2	P42262	GRIA2	0.012
Astragali Radix（AgR）	MOL000392	formononetin	5-hydroxytryptamine 4 receptor	Q13639	HTR4	0.012
Astragali Radix（AgR）	MOL000392	formononetin	2,4-dienoyl-CoA reductase, mitochondrial	Q16698	DECR1	0.012
Astragali Radix（AgR）	MOL000392	formononetin	Retinal dehydrogenase 2	O94788	ALDH1A2	0.013
Astragali Radix（AgR）	MOL000392	formononetin	Retinal dehydrogenase 1	P00352	ALDH1A1	0.013
Astragali Radix（AgR）	MOL000392	formononetin	Nuclear receptor 0B1	P51843	NR0B1	0.013
Astragali Radix（AgR）	MOL000392	formononetin	Troponin C, slow skeletal and cardiac muscles	P63316	TNNC1	0.013
Astragali Radix（AgR）	MOL000392	formononetin	Potassium voltage-gated channel subfamily H member 2	Q12809	KCNH2	0.013
Astragali Radix（AgR）	MOL000392	formononetin	Calcium-activated potassium channel subunit beta-1	Q16558	KCNMB1	0.013
Astragali Radix（AgR）	MOL000392	formononetin	Calcium-activated potassium channel subunit beta-4	Q86W47	KCNMB4	0.013
Astragali Radix（AgR）	MOL000392	formononetin	Retinoic acid-induced protein 3	Q8NFJ5	GPRC5A	0.013
Astragali Radix（AgR）	MOL000392	formononetin	Small conductance calcium-activated potassium channel protein 1	Q92952	KCNN1	0.013
Astragali Radix（AgR）	MOL000392	formononetin	Potassium voltage-gated channel subfamily H member 6	Q9H252	KCNH6	0.013
Astragali Radix（AgR）	MOL000392	formononetin	Small conductance calcium-activated potassium channel protein 2	Q9H2S1	KCNN2	0.013
Astragali Radix（AgR）	MOL000392	formononetin	Calcium-activated potassium channel subunit beta-3	Q9NPA1	KCNMB3	0.013
Astragali Radix（AgR）	MOL000392	formononetin	Potassium voltage-gated channel subfamily H member 7	Q9NS40	KCNH7	0.013
Astragali Radix（AgR）	MOL000392	formononetin	D1 dopamine receptor-interacting protein calcyon	Q9NYX4	CALY	0.013
Astragali Radix（AgR）	MOL000392	formononetin	Small conductance calcium-activated potassium channel protein 3	Q9UGI6	KCNN3	0.013
Astragali Radix（AgR）	MOL000392	formononetin	Calcium-activated potassium channel subunit beta 2	Q9Y691	KCNMB2	0.013
Astragali Radix（AgR）	MOL000392	formononetin	Nitric-oxide synthase, brain	P29475	NOS1	0.016
Astragali Radix（AgR）	MOL000392	formononetin	Cholinesterase	P06276	BCHE	0.018
Astragali Radix（AgR）	MOL000392	formononetin	Amine oxidase [flavin-containing] A	P21397	MAOA	0.018
Astragali Radix（AgR）	MOL000392	formononetin	Serine/threonine-protein kinase 17B	O94768	STK17B	0.019
Astragali Radix（AgR）	MOL000392	formononetin	ATP synthase subunit beta, mitochondrial	P06576	ATP5F1B	0.019

Astragali Radix（AgR）	MOL000392	formononetin	ATP synthase subunit alpha, mitochondrial	P25705	ATP5F1A	0.019
Astragali Radix（AgR）	MOL000392	formononetin	ATP synthase subunit gamma, mitochondrial	P36542	ATP5F1C	0.019
Astragali Radix（AgR）	MOL000392	formononetin	UDP-glucuronosyltransferase 3A1	Q6NUS8	UGT3A1	0.019
Astragali Radix（AgR）	MOL000392	formononetin	Vascular endothelial growth factor	A2A2V4	VEGFA	0.02
Astragali Radix（AgR）	MOL000392	formononetin	Tumor necrosis factor ligand superfamily member 11	O14788	TNFSF11	0.02
Astragali Radix（AgR）	MOL000392	formononetin	Cadherin-5	P33151	CDH5	0.02
Astragali Radix（AgR）	MOL000392	formononetin	Amiloride-sensitive sodium channel subunit alpha	P37088	SCNN1A	0.02
Astragali Radix（AgR）	MOL000392	formononetin	Amiloride-sensitive sodium channel subunit beta	P51168	SCNN1B	0.02
Astragali Radix（AgR）	MOL000392	formononetin	Amiloride-sensitive sodium channel subunit gamma	P51170	SCNN1G	0.02
Astragali Radix（AgR）	MOL000392	formononetin	Prostaglandin reductase 1	Q14914	PTGR1	0.02
Astragali Radix（AgR）	MOL000392	formononetin	Carbonic anhydrase 1	P00915	CA1	0.021
Astragali Radix（AgR）	MOL000392	formononetin	Carbonic anhydrase 2	P00918	CA2	0.021
Astragali Radix（AgR）	MOL000392	formononetin	Stromelysin-1	P08254	MMP3	0.021
Astragali Radix（AgR）	MOL000392	formononetin	Stromelysin-2	P09238	MMP10	0.021
Astragali Radix（AgR）	MOL000392	formononetin	Dihydrolipoyllysine-residue acetyltransferase component of pyruvate dehydrogenase complex, mitochondrial	P10515	DLAT	0.021
Astragali Radix（AgR）	MOL000392	formononetin	Glycine cleavage system H protein, mitochondrial	P23434	GCSH	0.021
Astragali Radix（AgR）	MOL000392	formononetin	Macrophage metalloelastase	P39900	MMP12	0.021
Astragali Radix（AgR）	MOL000392	formononetin	Interleukin-3	P08700	IL3	0.022
Astragali Radix（AgR）	MOL000392	formononetin	Protein S100-A12	P80511	S100A12	0.022
Astragali Radix（AgR）	MOL000392	formononetin	Protein S100-A13	Q99584	S100A13	0.022
Astragali Radix（AgR）	MOL000392	formononetin	Neuronal acetylcholine receptor subunit alpha-10	Q9GZZ6	CHRNA10	0.022
Astragali Radix（AgR）	MOL000392	formononetin	Gamma-aminobutyric acid receptor subunit gamma-2	P18507	GABRG2	0.023
Astragali Radix（AgR）	MOL000392	formononetin	Acetylcholinesterase	P22303	ACHE	0.023
Astragali Radix（AgR）	MOL000392	formononetin	Calcium-activated potassium channel subunit alpha 1	Q12791	KCNMA1	0.024
Astragali Radix（AgR）	MOL000392	formononetin	Tyrosine-protein kinase Lyn	P07948	LYN	0.027
Astragali Radix（AgR）	MOL000392	formononetin	Glutamate [NMDA] receptor subunit 3A	Q8TCU5	GRIN3A	0.027
Astragali Radix（AgR）	MOL000392	formononetin	3-phosphoinositide-dependent protein kinase 1	O15530	PDPK1	0.028
Astragali Radix（AgR）	MOL000392	formononetin	Tyrosine-protein kinase CSK	P41240	CSK	0.028
Astragali Radix（AgR）	MOL000392	formononetin	Tyrosine-protein kinase ZAP-70	P43403	ZAP70	0.028
Astragali Radix（AgR）	MOL000392	formononetin	Tyrosine-protein kinase SYK	P43405	SYK	0.028
Astragali Radix（AgR）	MOL000392	formononetin	5-hydroxytryptamine 3 receptor	P46098	HTR3A	0.028
Astragali Radix（AgR）	MOL000392	formononetin	Glycogen synthase kinase-3 beta	P49841	GSK3B	0.028
Astragali Radix（AgR）	MOL000392	formononetin	Protein kinase C theta type	Q04759	PRKCQ	0.028
Astragali Radix（AgR）	MOL000392	formononetin	cAMP-specific 3',5'-cyclic phosphodiesterase 4B	Q07343	PDE4B	0.028
Astragali Radix（AgR）	MOL000392	formononetin	Tyrosine-protein kinase ITK/TSK	Q08881	ITK	0.028
Astragali Radix（AgR）	MOL000392	formononetin	cGMP-inhibited 3',5'-cyclic phosphodiesterase A	Q14432	PDE3A	0.028
Astragali Radix（AgR）	MOL000392	formononetin	cAMP and cAMP-inhibited cGMP 3',5'-cyclic phosphodiesterase 10A	Q9Y233	PDE10A	0.028
Astragali Radix（AgR）	MOL000392	formononetin	Muscarinic acetylcholine receptor M2	P08172	CHRM2	0.029
Astragali Radix（AgR）	MOL000392	formononetin	Alpha-2B adrenergic receptor	P18089	ADRA2B	0.03
Astragali Radix（AgR）	MOL000392	formononetin	Alpha-2C adrenergic receptor	P18825	ADRA2C	0.03
Astragali Radix（AgR）	MOL000392	formononetin	D(1B) dopamine receptor	P21918	DRD5	0.03
Astragali Radix（AgR）	MOL000392	formononetin	Mu-type opioid receptor	P35372	OPRM1	0.03
Astragali Radix（AgR）	MOL000392	formononetin	D(3) dopamine receptor	P35462	DRD3	0.03
Astragali Radix（AgR）	MOL000392	formononetin	Gamma-aminobutyric acid receptor subunit rho-3	A8MPY1	GABRR3	0.031
Astragali Radix（AgR）	MOL000392	formononetin	Gamma-aminobutyric acid receptor subunit pi	O00591	GABRP	0.031
Astragali Radix（AgR）	MOL000392	formononetin	Gamma-aminobutyric acid receptor subunit delta	O14764	GABRD	0.031

Astragali Radix（AgR）	MOL000392	formononetin	Gamma-aminobutyric-acid receptor subunit beta-1	P18505	GABRB1	0.031
Astragali Radix（AgR）	MOL000392	formononetin	Gamma-aminobutyric-acid receptor subunit rho-1	P24046	GABRR1	0.031
Astragali Radix（AgR）	MOL000392	formononetin	Gamma-aminobutyric-acid receptor subunit beta-3	P28472	GABRB3	0.031
Astragali Radix（AgR）	MOL000392	formononetin	Gamma-aminobutyric acid receptor subunit rho-2	P28476	GABRR2	0.031
Astragali Radix（AgR）	MOL000392	formononetin	Gamma-aminobutyric-acid receptor subunit alpha-5	P31644	GABRA5	0.031
Astragali Radix（AgR）	MOL000392	formononetin	Gamma-aminobutyric acid receptor subunit epsilon	P78334	GABRE	0.031
Astragali Radix（AgR）	MOL000392	formononetin	Gamma-aminobutyric acid receptor subunit gamma-1	Q8N1C3	GABRG1	0.031
Astragali Radix（AgR）	MOL000392	formononetin	Gamma-aminobutyric acid receptor subunit gamma-3	Q99928	GABRG3	0.031
Astragali Radix（AgR）	MOL000392	formononetin	Retinoic acid receptor beta	P10826	RARB	0.032
Astragali Radix（AgR）	MOL000392	formononetin	Retinoic acid receptor RXR-gamma	P48443	RXRG	0.032
Astragali Radix（AgR）	MOL000392	formononetin	Calmodulin	P62158		0.033
Astragali Radix（AgR）	MOL000392	formononetin	Sterol O-acyltransferase 2	O75908	SOAT2	0.034
Astragali Radix（AgR）	MOL000392	formononetin	Sterol O-acyltransferase 1	P35610	SOAT1	0.034
Astragali Radix（AgR）	MOL000392	formononetin	Casein kinase II subunit alpha	P68400	CSNK2A1	0.034
Astragali Radix（AgR）	MOL000392	formononetin	Lipoic acid synthetase, mitochondrial	O43766	LIAS	0.035
Astragali Radix（AgR）	MOL000392	formononetin	Peptidyl-prolyl cis-trans isomerase, mitochondrial	P30405	PPIF	0.035
Astragali Radix（AgR）	MOL000392	formononetin	Lipoyltransferase 1, mitochondrial	Q9Y234	LIPT1	0.035
Astragali Radix（AgR）	MOL000392	formononetin	Myosin light chain 6B	P14649	MYL6B	0.036
Astragali Radix（AgR）	MOL000392	formononetin	Myosin regulatory light chain 12A	P19105	MYL12A	0.036
Astragali Radix（AgR）	MOL000392	formononetin	Gamma-aminobutyric-acid receptor subunit alpha-3	P34903	GABRA3	0.038
Astragali Radix（AgR）	MOL000392	formononetin	Tyrosine-protein kinase HCK	P08631	HCK	0.043
Astragali Radix（AgR）	MOL000392	formononetin	Phosphatidylinositol-4,5-bisphosphate 3-kinase catalytic subunit gamma isoform	P48736	PIK3CG	0.043
Astragali Radix（AgR）	MOL000392	formononetin	Beta-2 adrenergic receptor	P07550	ADRB2	0.045
Astragali Radix（AgR）	MOL000392	formononetin	Urokinase-type plasminogen activator	P00749	PLAU	0.047
Astragali Radix（AgR）	MOL000392	formononetin	Muscarinic acetylcholine receptor M5	P08912	CHRM5	0.047
Astragali Radix（AgR）	MOL000392	formononetin	Alpha-2A adrenergic receptor	P08913	ADRA2A	0.048
Astragali Radix（AgR）	MOL000392	formononetin	Androgen receptor	P10275	AR	0.052
Astragali Radix（AgR）	MOL000392	formononetin	Retinoic acid receptor alpha	P10276	RARA	0.052
Astragali Radix（AgR）	MOL000392	formononetin	Retinoic acid receptor gamma-1	P13631	RARG	0.052
Astragali Radix（AgR）	MOL000392	formononetin	Retinoic acid receptor RXR-alpha	P19793	RXRA	0.052
Astragali Radix（AgR）	MOL000392	formononetin	Peroxisome proliferator-activated receptor gamma	P37231	PPARG	0.052
Astragali Radix（AgR）	MOL000392	formononetin	Gamma-aminobutyric-acid receptor subunit alpha-1	P14867	GABRA1	0.053
Astragali Radix（AgR）	MOL000392	formononetin	Gamma-aminobutyric-acid receptor subunit alpha-2	P47869	GABRA2	0.053
Astragali Radix（AgR）	MOL000392	formononetin	Proto-oncogene serine/threonine-protein kinase Pim-1	P11309	PIM1	0.054
Astragali Radix（AgR）	MOL000392	formononetin	Nitric oxide synthase, inducible	P35228	NOS2	0.055
Astragali Radix（AgR）	MOL000392	formononetin	Nitric-oxide synthase, endothelial	P29474	NOS3	0.057
Astragali Radix（AgR）	MOL000392	formononetin	Aldo-keto reductase family 1 member C3	P42330	AKR1C3	0.061
Astragali Radix（AgR）	MOL000392	formononetin	Proto-oncogene tyrosine-protein kinase LCK	P06239	LCK	0.062
Astragali Radix（AgR）	MOL000392	formononetin	cAMP-specific 3',5'-cyclic phosphodiesterase 4A	P27815	PDE4A	0.063
Astragali Radix（AgR）	MOL000392	formononetin	Muscarinic acetylcholine receptor M4	P08173	CHRM4	0.065
Astragali Radix（AgR）	MOL000392	formononetin	Muscarinic acetylcholine receptor M3	P20309	CHRM3	0.065
Astragali Radix（AgR）	MOL000392	formononetin	Rho-associated protein kinase 1	Q13464	ROCK1	0.067
Astragali Radix（AgR）	MOL000392	formononetin	DNA topoisomerase 2-alpha	P11388	TOP2A	0.069
Astragali Radix（AgR）	MOL000392	formononetin	Activin receptor type-1	Q04771	ACVR1	0.069
Astragali Radix（AgR）	MOL000392	formononetin	Protein tyrosine kinase 2 beta	Q14289	PTK2B	0.069
Astragali Radix（AgR）	MOL000392	formononetin	DNA polymerase kappa	Q9UBT6	POLK	0.069
Astragali Radix（AgR）	MOL000392	formononetin	Epidermal growth factor receptor	P00533	EGFR	0.07

Astragali Radix（AgR）	MOL000392	formononetin	Retinoic acid receptor RXR-beta	P28702	RXRB	0.071
Astragali Radix（AgR）	MOL000392	formononetin	Phospholipase A2, membrane associated	P14555	PLA2G2A	0.072
Astragali Radix（AgR）	MOL000392	formononetin	Beta-1 adrenergic receptor	P08588	ADRB1	0.073
Astragali Radix（AgR）	MOL000392	formononetin	Dihydrofolate reductase	P00374	DHFR	0.074
Astragali Radix（AgR）	MOL000392	formononetin	Heat shock protein HSP 90-beta	P08238	HSP90AB1	0.074
Astragali Radix（AgR）	MOL000392	formononetin	5-hydroxytryptamine 1A receptor	P08908	HTR1A	0.074
Astragali Radix（AgR）	MOL000392	formononetin	Vitamin K-dependent protein C	P04070	PROC	0.075
Astragali Radix（AgR）	MOL000392	formononetin	Glyceraldehyde-3-phosphate dehydrogenase	P04406	GAPDH	0.075
Astragali Radix（AgR）	MOL000392	formononetin	Mitogen-activated protein kinase 14	Q16539	MAPK14	0.075
Astragali Radix（AgR）	MOL000392	formononetin	Serine/threonine-protein kinase 6	O14965	AURKA	0.076
Astragali Radix（AgR）	MOL000392	formononetin	Coagulation factor VII	P08709	F7	0.076
Astragali Radix（AgR）	MOL000392	formononetin	Low molecular weight phosphotyrosine protein phosphatase	P24666	ACP1	0.076
Astragali Radix（AgR）	MOL000392	formononetin	RAC-alpha serine/threonine-protein kinase	P31749	AKT1	0.077
Astragali Radix（AgR）	MOL000392	formononetin	Ig kappa chain C region	P01834	IGKC	0.078
Astragali Radix（AgR）	MOL000392	formononetin	Proto-oncogene tyrosine-protein kinase Src	P12931	SRC	0.078
Astragali Radix（AgR）	MOL000392	formononetin	Cannabinoid receptor 2	P34972	CNR2	0.078
Astragali Radix（AgR）	MOL000392	formononetin	Lysozyme C	P61626	LYZ	0.079
Astragali Radix（AgR）	MOL000392	formononetin	ATP-binding cassette transporter sub-family C member 8	Q09428	ABCC8	0.079
Astragali Radix（AgR）	MOL000392	formononetin	C-jun-amino-terminal kinase-interacting protein 1	Q9UQF2	MAPK8IP1	0.079
Astragali Radix（AgR）	MOL000392	formononetin	Cannabinoid receptor 1	P21554	CNR1	0.08
Astragali Radix（AgR）	MOL000392	formononetin	Estrogen-related receptor gamma	P62508	ESRRG	0.08
Astragali Radix（AgR）	MOL000392	formononetin	Thyroid hormone receptor beta-1	P10828	THRB	0.081
Astragali Radix（AgR）	MOL000392	formononetin	5-hydroxytryptamine 1B receptor	P28222	HTR1B	0.081
Astragali Radix（AgR）	MOL000392	formononetin	5-hydroxytryptamine 2C receptor	P28335	HTR2C	0.081
Astragali Radix（AgR）	MOL000392	formononetin	Sodium-dependent noradrenaline transporter	P23975	SLC6A2	0.082
Astragali Radix（AgR）	MOL000392	formononetin	5-hydroxytryptamine 2B receptor	P41595	HTR2B	0.082
Astragali Radix（AgR）	MOL000392	formononetin	Nuclear receptor coactivator 5	Q9HCD5	NCOA5	0.082
Astragali Radix（AgR）	MOL000392	formononetin	Histamine H1 receptor	P35367	HRH1	0.083
Astragali Radix（AgR）	MOL000392	formononetin	Alpha-1A adrenergic receptor	P35348	ADRA1A	0.086
Astragali Radix（AgR）	MOL000392	formononetin	Fibroblast growth factor receptor 2	P21802	FGFR2	0.087
Astragali Radix（AgR）	MOL000392	formononetin	Liver carboxylesterase 1	P23141	CES1	0.093
Astragali Radix（AgR）	MOL000392	formononetin	MAP kinase-activated protein kinase 2	P49137	MAPKAPK2	0.097
Astragali Radix（AgR）	MOL000392	formononetin	Muscarinic acetylcholine receptor M1	P11229	CHRM1	0.101
Astragali Radix（AgR）	MOL000392	formononetin	Nuclear receptor coactivator 2	Q15596	NCOA2	0.11
Astragali Radix（AgR）	MOL000392	formononetin	Sodium-dependent serotonin transporter	P31645	SLC6A4	0.116
Astragali Radix（AgR）	MOL000392	formononetin	5-hydroxytryptamine 2A receptor	P28223	HTR2A	0.118
Astragali Radix（AgR）	MOL000392	formononetin	Phospholipase A2	P04054	PLA2G1B	0.128
Astragali Radix（AgR）	MOL000392	formononetin	Prothrombin	P00734	F2	0.152
Astragali Radix（AgR）	MOL000392	formononetin	D(1A) dopamine receptor	P21728	DRD1	0.155
Astragali Radix（AgR）	MOL000392	formononetin	Cytochrome P450 11B1, mitochondrial	P15538	CYP11B1	0.162
Astragali Radix（AgR）	MOL000392	formononetin	ATP-sensitive inward rectifier potassium channel 1	P48048	KCNJ1	0.163
Astragali Radix（AgR）	MOL000392	formononetin	Progesterone receptor	P06401	PGR	0.17
Astragali Radix（AgR）	MOL000392	formononetin	Prostaglandin G/H synthase 1	P23219	PTGS1	0.195
Astragali Radix（AgR）	MOL000392	formononetin	D(2) dopamine receptor	P14416	DRD2	0.228
Astragali Radix（AgR）	MOL000392	formononetin	Cell division protein kinase 2	P24941	CDK2	0.231
Astragali Radix（AgR）	MOL000392	formononetin	Trypsin-1	P07477	PRSS1	0.258
Astragali Radix（AgR）	MOL000392	formononetin	Hemoglobin subunit alpha	P69905	HBA1	0.342

Astragali Radix（AgR）	MOL000392	formononetin	Nuclear receptor coactivator 1	Q15788	NCOA1	0.362
Astragali Radix（AgR）	MOL000392	formononetin	cAMP-dependent protein kinase catalytic subunit alpha	P17612	PRKACA	0.434
Astragali Radix（AgR）	MOL000392	formononetin	Cyclin-A2	P20248	CCNA2	0.567
Astragali Radix（AgR）	MOL000392	formononetin	Estrogen receptor beta	Q92731	ESR2	0.583
Astragali Radix（AgR）	MOL000392	formononetin	Prostaglandin G/H synthase 2	P35354	PTGS2	0.8
Astragali Radix（AgR）	MOL000392	formononetin	Estrogen receptor	P03372	ESR1	1
Astragali Radix（AgR）	MOL000398	isoflavanone	Cytochrome b-c1 complex subunit 8	O14949	UQCRQ	0.01
Astragali Radix（AgR）	MOL000398	isoflavanone	Cytochrome b-c1 complex subunit 10	O14957	UQCR11	0.01
Astragali Radix（AgR）	MOL000398	isoflavanone	Cytochrome b-c1 complex subunit 6, mitochondrial	P07919	UQCRH	0.01
Astragali Radix（AgR）	MOL000398	isoflavanone	Cytochrome b-c1 complex subunit 7	P14927	UQCRB	0.01
Astragali Radix（AgR）	MOL000398	isoflavanone	Cytochrome b-c1 complex subunit 2, mitochondrial	P22695	UQCRC2	0.01
Astragali Radix（AgR）	MOL000398	isoflavanone	Ubiquinol-cytochrome-c reductase complex core protein 1, mitochondrial	P31930	UQCRC1	0.01
Astragali Radix（AgR）	MOL000398	isoflavanone	Cytochrome b-c1 complex subunit 9	Q9UDW1	UQCR10	0.01
Astragali Radix（AgR）	MOL000398	isoflavanone	Gamma-aminobutyric acid receptor subunit rho-3	A8MPY1	GABRR3	0.011
Astragali Radix（AgR）	MOL000398	isoflavanone	Gamma-aminobutyric acid receptor subunit pi	O00591	GABRP	0.011
Astragali Radix（AgR）	MOL000398	isoflavanone	Gamma-aminobutyric acid receptor subunit delta	O14764	GABRD	0.011
Astragali Radix（AgR）	MOL000398	isoflavanone	Gamma-aminobutyric-acid receptor subunit beta-1	P18505	GABRB1	0.011
Astragali Radix（AgR）	MOL000398	isoflavanone	Gamma-aminobutyric acid receptor subunit gamma-2	P18507	GABRG2	0.011
Astragali Radix（AgR）	MOL000398	isoflavanone	Gamma-aminobutyric-acid receptor subunit rho-1	P24046	GABRR1	0.011
Astragali Radix（AgR）	MOL000398	isoflavanone	Gamma-aminobutyric-acid receptor subunit beta-3	P28472	GABRB3	0.011
Astragali Radix（AgR）	MOL000398	isoflavanone	Gamma-aminobutyric acid receptor subunit rho-2	P28476	GABRR2	0.011
Astragali Radix（AgR）	MOL000398	isoflavanone	Gamma-aminobutyric-acid receptor subunit alpha-5	P31644	GABRA5	0.011
Astragali Radix（AgR）	MOL000398	isoflavanone	Gamma-aminobutyric-acid receptor subunit alpha-3	P34903	GABRA3	0.011
Astragali Radix（AgR）	MOL000398	isoflavanone	Gamma-aminobutyric acid receptor subunit epsilon	P78334	GABRE	0.011
Astragali Radix（AgR）	MOL000398	isoflavanone	Gamma-aminobutyric acid receptor subunit gamma-1	Q8N1C3	GABRG1	0.011
Astragali Radix（AgR）	MOL000398	isoflavanone	Gamma-aminobutyric acid receptor subunit gamma-3	Q99928	GABRG3	0.011
Astragali Radix（AgR）	MOL000398	isoflavanone	Hydroxyacid oxidase 1	Q9UJM8	HAO1	0.015
Astragali Radix（AgR）	MOL000398	isoflavanone	cAMP-dependent protein kinase type I-alpha regulatory subunit	P10644	PRKAR1A	0.016
Astragali Radix（AgR）	MOL000398	isoflavanone	cAMP-dependent protein kinase type II-beta regulatory subunit	P31323	PRKAR2B	0.016
Astragali Radix（AgR）	MOL000398	isoflavanone	Potassium/sodium hyperpolarization-activated cyclic nucleotide-gated channel 2	Q9UL51	HCN2	0.016
Astragali Radix（AgR）	MOL000398	isoflavanone	Potassium channel subfamily K member 6	Q9Y257	KCNK6	0.018
Astragali Radix（AgR）	MOL000398	isoflavanone	D(1A) dopamine receptor	P21728	DRD1	0.021
Astragali Radix（AgR）	MOL000398	isoflavanone	D(1B) dopamine receptor	P21918	DRD5	0.021
Astragali Radix（AgR）	MOL000398	isoflavanone	5-hydroxytryptamine 1A receptor	P08908	HTR1A	0.022
Astragali Radix（AgR）	MOL000398	isoflavanone	Alpha-2A adrenergic receptor	P08913	ADRA2A	0.022
Astragali Radix（AgR）	MOL000398	isoflavanone	5-hydroxytryptamine 1D receptor	P28221	HTR1D	0.022
Astragali Radix（AgR）	MOL000398	isoflavanone	5-hydroxytryptamine 2C receptor	P28335	HTR2C	0.022
Astragali Radix（AgR）	MOL000398	isoflavanone	D(4) dopamine receptor	P21917	DRD4	0.023
Astragali Radix（AgR）	MOL000398	isoflavanone	Glutamate receptor 2	P42262	GRIA2	0.023
Astragali Radix（AgR）	MOL000398	isoflavanone	Sodium-dependent noradrenaline transporter	P23975	SLC6A2	0.025
Astragali Radix（AgR）	MOL000398	isoflavanone	Sodium-dependent serotonin transporter	P31645	SLC6A4	0.025
Astragali Radix（AgR）	MOL000398	isoflavanone	cGMP-specific 3',5'-cyclic phosphodiesterase	O76074	PDE5A	0.027
Astragali Radix（AgR）	MOL000398	isoflavanone	Adenosine A2b receptor	P29275	ADORA2B	0.027
Astragali Radix（AgR）	MOL000398	isoflavanone	cAMP-specific 3',5'-cyclic phosphodiesterase 4D	Q08499	PDE4D	0.027
Astragali Radix（AgR）	MOL000398	isoflavanone	High-affinity cAMP-specific 3',5'-cyclic phosphodiesterase 7A	Q13946	PDE7A	0.027
Astragali Radix（AgR）	MOL000398	isoflavanone	cAMP and cAMP-inhibited cGMP 3',5'-cyclic phosphodiesterase 10A	Q9Y233	PDE10A	0.027
Astragali Radix（AgR）	MOL000398	isoflavanone	Cytochrome b	P00156	MT-CYB	0.031

Astragali Radix（AgR）	MOL000398	isoflavanone	Cytochrome c1, heme protein, mitochondrial	P08574	CYC1	0.031
Astragali Radix（AgR）	MOL000398	isoflavanone	Cytochrome b-c1 complex subunit Rieske, mitochondrial	P47985	UQCRFS1	0.031
Astragali Radix（AgR）	MOL000398	isoflavanone	Tumor necrosis factor	P01375	TNF	0.033
Astragali Radix（AgR）	MOL000398	isoflavanone	Nuclear factor NF-kappa-B p105 subunit	P19838	NFKB1	0.033
Astragali Radix（AgR）	MOL000398	isoflavanone	Retinoic acid receptor beta	P10826	RARB	0.034
Astragali Radix（AgR）	MOL000398	isoflavanone	Retinoic acid receptor gamma-1	P13631	RARG	0.034
Astragali Radix（AgR）	MOL000398	isoflavanone	Retinoic acid receptor RXR-beta	P28702	RXRB	0.034
Astragali Radix（AgR）	MOL000398	isoflavanone	Retinoic acid receptor RXR-gamma	P48443	RXRG	0.034
Astragali Radix（AgR）	MOL000398	isoflavanone	Tyrosine 3-monooxygenase	P07101	TH	0.036
Astragali Radix（AgR）	MOL000398	isoflavanone	Tyrosyl-tRNA synthetase, cytoplasmic	P54577	YARS	0.036
Astragali Radix（AgR）	MOL000398	isoflavanone	Tyrosyl-tRNA synthetase, mitochondrial	Q9Y2Z4	YARS2	0.036
Astragali Radix（AgR）	MOL000398	isoflavanone	Serine/threonine-protein kinase 17B	O94768	STK17B	0.037
Astragali Radix（AgR）	MOL000398	isoflavanone	Proto-oncogene serine/threonine-protein kinase Pim-1	P11309	PIM1	0.037
Astragali Radix（AgR）	MOL000398	isoflavanone	ATP synthase subunit gamma, mitochondrial	P36542	ATP5F1C	0.037
Astragali Radix（AgR）	MOL000398	isoflavanone	UDP-glucuronosyltransferase 3A1	Q6NUS8	UGT3A1	0.037
Astragali Radix（AgR）	MOL000398	isoflavanone	Tripartite motif-containing protein 13	O60858	TRIM13	0.039
Astragali Radix（AgR）	MOL000398	isoflavanone	Amiloride-sensitive sodium channel subunit alpha	P37088	SCNN1A	0.039
Astragali Radix（AgR）	MOL000398	isoflavanone	Nociceptin receptor	P41146	OPRL1	0.039
Astragali Radix（AgR）	MOL000398	isoflavanone	Amiloride-sensitive sodium channel subunit beta	P51168	SCNN1B	0.039
Astragali Radix（AgR）	MOL000398	isoflavanone	Amiloride-sensitive sodium channel subunit gamma	P51170	SCNN1G	0.039
Astragali Radix（AgR）	MOL000398	isoflavanone	Vascular endothelial growth factor	A2A2V4	VEGFA	0.041
Astragali Radix（AgR）	MOL000398	isoflavanone	Tumor necrosis factor ligand superfamily member 11	O14788	TNFSF11	0.041
Astragali Radix（AgR）	MOL000398	isoflavanone	Cadherin-5	P33151	CDH5	0.041
Astragali Radix（AgR）	MOL000398	isoflavanone	Glycogen phosphorylase, muscle form	P11217	PYGM	0.042
Astragali Radix（AgR）	MOL000398	isoflavanone	Cell division protein kinase 7	P50613	CDK7	0.042
Astragali Radix（AgR）	MOL000398	isoflavanone	Cell division protein kinase 9	P50750	CDK9	0.042
Astragali Radix（AgR）	MOL000398	isoflavanone	Cyclin-dependent kinase 5 activator 1	Q15078	CDK5R1	0.042
Astragali Radix（AgR）	MOL000398	isoflavanone	Mitogen-activated protein kinase 3	P27361	MAPK3	0.043
Astragali Radix（AgR）	MOL000398	isoflavanone	Mitogen-activated protein kinase 1	P28482	MAPK1	0.043
Astragali Radix（AgR）	MOL000398	isoflavanone	Potassium channel subfamily K member 1	O00180	KCNK1	0.044
Astragali Radix（AgR）	MOL000398	isoflavanone	Sodium channel protein type 5 subunit alpha	Q14524	SCN5A	0.044
Astragali Radix（AgR）	MOL000398	isoflavanone	cAMP-dependent protein kinase catalytic subunit alpha	P17612	PRKACA	0.051
Astragali Radix（AgR）	MOL000398	isoflavanone	Peptidyl-prolyl cis-trans isomerase, mitochondrial	P30405	PPIF	0.051
Astragali Radix（AgR）	MOL000398	isoflavanone	Rho-associated protein kinase 1	Q13464	ROCK1	0.051
Astragali Radix（AgR）	MOL000398	isoflavanone	Sepiapterin reductase	P35270	SPR	0.052
Astragali Radix（AgR）	MOL000398	isoflavanone	6-pyruvoyl tetrahydrobiopterin synthase	Q03393	PTS	0.052
Astragali Radix（AgR）	MOL000398	isoflavanone	Alpha-2B adrenergic receptor	P18089	ADRA2B	0.054
Astragali Radix（AgR）	MOL000398	isoflavanone	Alpha-2C adrenergic receptor	P18825	ADRA2C	0.054
Astragali Radix（AgR）	MOL000398	isoflavanone	5-hydroxytryptamine 1B receptor	P28222	HTR1B	0.054
Astragali Radix（AgR）	MOL000398	isoflavanone	D(3) dopamine receptor	P35462	DRD3	0.055
Astragali Radix（AgR）	MOL000398	isoflavanone	Thymidine kinase 2, mitochondrial	O00142	TK2	0.056
Astragali Radix（AgR）	MOL000398	isoflavanone	Tubulin beta-2C chain	P68371	TUBB4B	0.056
Astragali Radix（AgR）	MOL000398	isoflavanone	Tubulin alpha-3 chain	Q71U36	TUBA1A	0.056
Astragali Radix（AgR）	MOL000398	isoflavanone	Sterol O-acyltransferase 2	O75908	SOAT2	0.057
Astragali Radix（AgR）	MOL000398	isoflavanone	Sterol O-acyltransferase 1	P35610	SOAT1	0.057
Astragali Radix（AgR）	MOL000398	isoflavanone	Proto-oncogene tyrosine-protein kinase LCK	P06239	LCK	0.058
Astragali Radix（AgR）	MOL000398	isoflavanone	Tyrosine-protein kinase Lyn	P07948	LYN	0.058

Astragali Radix（AgR）	MOL000398	isoflavanone	Ribosyldihydronicotinamide dehydrogenase [quinone]	P16083	NQO2	0.058
Astragali Radix（AgR）	MOL000398	isoflavanone	Casein kinase II subunit alpha	P68400	CSNK2A1	0.058
Astragali Radix（AgR）	MOL000398	isoflavanone	Carbonic anhydrase 1	P00915	CA1	0.059
Astragali Radix（AgR）	MOL000398	isoflavanone	Sodium/potassium-transporting ATPase gamma chain	P54710	FXYD2	0.059
Astragali Radix（AgR）	MOL000398	isoflavanone	Gamma-aminobutyric-acid receptor subunit alpha-1	P14867	GABRA1	0.06
Astragali Radix（AgR）	MOL000398	isoflavanone	Gamma-aminobutyric-acid receptor subunit alpha-2	P47869	GABRA2	0.06
Astragali Radix（AgR）	MOL000398	isoflavanone	Muscarinic acetylcholine receptor M4	P08173	CHRM4	0.061
Astragali Radix（AgR）	MOL000398	isoflavanone	Muscarinic acetylcholine receptor M1	P11229	CHRM1	0.061
Astragali Radix（AgR）	MOL000398	isoflavanone	Muscarinic acetylcholine receptor M3	P20309	CHRM3	0.061
Astragali Radix（AgR）	MOL000398	isoflavanone	Beta-2 adrenergic receptor	P07550	ADRB2	0.065
Astragali Radix（AgR）	MOL000398	isoflavanone	Beta-1 adrenergic receptor	P08588	ADRB1	0.065
Astragali Radix（AgR）	MOL000398	isoflavanone	cAMP-specific 3',5'-cyclic phosphodiesterase 4C	Q08493	PDE4C	0.065
Astragali Radix（AgR）	MOL000398	isoflavanone	Aryl hydrocarbon receptor	P35869	AHR	0.066
Astragali Radix（AgR）	MOL000398	isoflavanone	Dihydroorotate dehydrogenase, mitochondrial	Q02127	DHODH	0.066
Astragali Radix（AgR）	MOL000398	isoflavanone	Urokinase-type plasminogen activator	P00749	PLAU	0.068
Astragali Radix（AgR）	MOL000398	isoflavanone	Macrophage migration inhibitory factor	P14174	MIF	0.068
Astragali Radix（AgR）	MOL000398	isoflavanone	Peroxiredoxin-5, mitochondrial	P30044	PRDX5	0.069
Astragali Radix（AgR）	MOL000398	isoflavanone	Inhibitor of nuclear factor kappa-B kinase subunit alpha	O15111	CHUK	0.076
Astragali Radix（AgR）	MOL000398	isoflavanone	Group IIE secretory phospholipase A2	Q9NZK7	PLA2G2E	0.076
Astragali Radix（AgR）	MOL000398	isoflavanone	Fibroblast growth factor receptor 2	P21802	FGFR2	0.078
Astragali Radix（AgR）	MOL000398	isoflavanone	Retinoic acid receptor alpha	P10276	RARA	0.079
Astragali Radix（AgR）	MOL000398	isoflavanone	ATP synthase subunit beta, mitochondrial	P06576	ATP5F1B	0.084
Astragali Radix（AgR）	MOL000398	isoflavanone	Tyrosine-protein kinase HCK	P08631	HCK	0.084
Astragali Radix（AgR）	MOL000398	isoflavanone	Platelet glycoprotein IX	P14770	GP9	0.084
Astragali Radix（AgR）	MOL000398	isoflavanone	ATP synthase subunit alpha, mitochondrial	P25705	ATP5F1A	0.084
Astragali Radix（AgR）	MOL000398	isoflavanone	Casein kinase I isoform gamma-3	Q9Y6M4	CSNK1G3	0.084
Astragali Radix（AgR）	MOL000398	isoflavanone	D(2) dopamine receptor	P14416	DRD2	0.089
Astragali Radix（AgR）	MOL000398	isoflavanone	Delta-type opioid receptor	P41143	OPRD1	0.089
Astragali Radix（AgR）	MOL000398	isoflavanone	Kappa-type opioid receptor	P41145	OPRK1	0.089
Astragali Radix（AgR）	MOL000398	isoflavanone	5-hydroxytryptamine 2A receptor	P28223	HTR2A	0.092
Astragali Radix（AgR）	MOL000398	isoflavanone	Glycogen synthase kinase-3 beta	P49841	GSK3B	0.094
Astragali Radix（AgR）	MOL000398	isoflavanone	Epidermal growth factor receptor	P00533	EGFR	0.095
Astragali Radix（AgR）	MOL000398	isoflavanone	Cell division protein kinase 6	Q00534	CDK6	0.095
Astragali Radix（AgR）	MOL000398	isoflavanone	Muscarinic acetylcholine receptor M2	P08172	CHRM2	0.096
Astragali Radix（AgR）	MOL000398	isoflavanone	Cell division protein kinase 4	P11802	CDK4	0.096
Astragali Radix（AgR）	MOL000398	isoflavanone	DNA topoisomerase 2-alpha	P11388	TOP2A	0.101
Astragali Radix（AgR）	MOL000398	isoflavanone	Protein tyrosine kinase 2 beta	Q14289	PTK2B	0.101
Astragali Radix（AgR）	MOL000398	isoflavanone	Estrogen-related receptor gamma	P62508	ESRRG	0.102
Astragali Radix（AgR）	MOL000398	isoflavanone	cGMP-inhibited 3',5'-cyclic phosphodiesterase A	Q14432	PDE3A	0.103
Astragali Radix（AgR）	MOL000398	isoflavanone	Progesterone receptor	P06401	PGR	0.116
Astragali Radix（AgR）	MOL000398	isoflavanone	Thyroid hormone receptor alpha	P10827	THRA	0.117
Astragali Radix（AgR）	MOL000398	isoflavanone	Toll-like receptor 7	Q9NYK1	TLR7	0.119
Astragali Radix（AgR）	MOL000398	isoflavanone	Sodium channel protein type 10 subunit alpha	Q9Y5Y9	SCN10A	0.119
Astragali Radix（AgR）	MOL000398	isoflavanone	Prolyl endopeptidase	P48147	PREP	0.123
Astragali Radix（AgR）	MOL000398	isoflavanone	Activin receptor type-1	Q04771	ACVR1	0.124
Astragali Radix（AgR）	MOL000398	isoflavanone	Thyroid hormone receptor beta-1	P10828	THRB	0.125
Astragali Radix（AgR）	MOL000398	isoflavanone	Cytochrome P450 19A1	P11511	CYP19A1	0.125

Astragali Radix（AgR）	MOL000398	isoflavanone	ATP-sensitive inward rectifier potassium channel 1	P48048	KCNJ1	0.125
Astragali Radix（AgR）	MOL000398	isoflavanone	MAP kinase-activated protein kinase 2	P49137	MAPKAPK2	0.125
Astragali Radix（AgR）	MOL000398	isoflavanone	Prostaglandin G/H synthase 1	P23219	PTGS1	0.127
Astragali Radix（AgR）	MOL000398	isoflavanone	Nitric-oxide synthase, endothelial	P29474	NOS3	0.127
Astragali Radix（AgR）	MOL000398	isoflavanone	Carbonic anhydrase 2	P00918	CA2	0.128
Astragali Radix（AgR）	MOL000398	isoflavanone	Endothelin-1 receptor	P25101	EDNRA	0.128
Astragali Radix（AgR）	MOL000398	isoflavanone	Casein kinase I isoform gamma-1	Q9HCP0	CSNK1G1	0.128
Astragali Radix（AgR）	MOL000398	isoflavanone	Coagulation factor VII	P08709	F7	0.129
Astragali Radix（AgR）	MOL000398	isoflavanone	Dihydrofolate reductase	P00374	DHFR	0.131
Astragali Radix（AgR）	MOL000398	isoflavanone	Potassium voltage-gated channel subfamily KQT member 1	P51787	KCNQ1	0.131
Astragali Radix（AgR）	MOL000398	isoflavanone	Calmodulin	P62158		0.132
Astragali Radix（AgR）	MOL000398	isoflavanone	Phosphatidylinositol-4,5-bisphosphate 3-kinase catalytic subunit gamma isoform	P48736	PIK3CG	0.134
Astragali Radix（AgR）	MOL000398	isoflavanone	Peroxisome proliferator-activated receptor gamma	P37231	PPARG	0.135
Astragali Radix（AgR）	MOL000398	isoflavanone	Nuclear receptor coactivator 2	Q15596	NCOA2	0.135
Astragali Radix（AgR）	MOL000398	isoflavanone	Liver carboxylesterase 1	P23141	CES1	0.137
Astragali Radix（AgR）	MOL000398	isoflavanone	Adenosine A2a receptor	P29274	ADORA2A	0.14
Astragali Radix（AgR）	MOL000398	isoflavanone	Adenosine A1 receptor	P30542	ADORA1	0.14
Astragali Radix（AgR）	MOL000398	isoflavanone	cAMP-specific 3',5'-cyclic phosphodiesterase 4B	Q07343	PDE4B	0.14
Astragali Radix（AgR）	MOL000398	isoflavanone	Cytochrome P450 11B1, mitochondrial	P15538	CYP11B1	0.142
Astragali Radix（AgR）	MOL000398	isoflavanone	Arachidonate 5-lipoxygenase	P09917	ALOX5	0.152
Astragali Radix（AgR）	MOL000398	isoflavanone	Proto-oncogene tyrosine-protein kinase Src	P12931	SRC	0.179
Astragali Radix（AgR）	MOL000398	isoflavanone	Mu-type opioid receptor	P35372	OPRM1	0.189
Astragali Radix（AgR）	MOL000398	isoflavanone	D-HSCDK2	O75100	CA11	0.2
Astragali Radix（AgR）	MOL000398	isoflavanone	cAMP-specific 3',5'-cyclic phosphodiesterase 4A	P27815	PDE4A	0.216
Astragali Radix（AgR）	MOL000398	isoflavanone	Prothrombin	P00734	F2	0.226
Astragali Radix（AgR）	MOL000398	isoflavanone	Cell division control protein 2 homolog	P06493	CDK1	0.253
Astragali Radix（AgR）	MOL000398	isoflavanone	Cell division protein kinase 5	Q00535	CDK5	0.253
Astragali Radix（AgR）	MOL000398	isoflavanone	Heat shock protein HSP 90-beta	P08238	HSP90AB1	0.265
Astragali Radix（AgR）	MOL000398	isoflavanone	Acetylcholinesterase	P22303	ACHE	0.267
Astragali Radix（AgR）	MOL000398	isoflavanone	RAC-alpha serine/threonine-protein kinase	P31749	AKT1	0.274
Astragali Radix（AgR）	MOL000398	isoflavanone	Hemoglobin subunit alpha	P69905	HBA1	0.283
Astragali Radix（AgR）	MOL000398	isoflavanone	C-jun-amino-terminal kinase-interacting protein 1	Q9UQF2	MAPK8IP1	0.295
Astragali Radix（AgR）	MOL000398	isoflavanone	Nuclear receptor coactivator 1	Q15788	NCOA1	0.432
Astragali Radix（AgR）	MOL000398	isoflavanone	Trypsin-1	P07477	PRSS1	0.463
Astragali Radix（AgR）	MOL000398	isoflavanone	Cell division protein kinase 2	P24941	CDK2	0.521
Astragali Radix（AgR）	MOL000398	isoflavanone	Prostaglandin G/H synthase 2	P35354	PTGS2	0.64
Astragali Radix（AgR）	MOL000398	isoflavanone	Cyclin-A2	P20248	CCNA2	0.668
Astragali Radix（AgR）	MOL000398	isoflavanone	Estrogen receptor beta	Q92731	ESR2	0.876
Astragali Radix（AgR）	MOL000398	isoflavanone	Estrogen receptor	P03372	ESR1	1
Astragali Radix（AgR）	MOL000417	Calycosin	Neuronal acetylcholine receptor subunit alpha-2	Q15822	CHRNA2	0.01
Astragali Radix（AgR）	MOL000417	Calycosin	Opioid receptor, sigma 1	Q5T1J1	SIGMAR1	0.01
Astragali Radix（AgR）	MOL000417	Calycosin	Sigma 1-type opioid receptor	Q99720	SIGMAR1	0.01
Astragali Radix（AgR）	MOL000417	Calycosin	Sodium channel protein type 10 subunit alpha	Q9Y5Y9	SCN10A	0.01
Astragali Radix（AgR）	MOL000417	Calycosin	Potassium channel subfamily K member 6	Q9Y257	KCNK6	0.011
Astragali Radix（AgR）	MOL000417	Calycosin	Retinal dehydrogenase 2	O94788	ALDH1A2	0.012
Astragali Radix（AgR）	MOL000417	Calycosin	Retinal dehydrogenase 1	P00352	ALDH1A1	0.012
Astragali Radix（AgR）	MOL000417	Calycosin	Rhodopsin	P08100	RHO	0.012

Astragali Radix（AgR）	MOL000417	Calycosin	Dihydrolipoyllysine-residue acetyltransferase component of pyruvate dehydrogenase complex, mitochondrial	P10515	DLAT	0.012
Astragali Radix（AgR）	MOL000417	Calycosin	Glycine cleavage system H protein, mitochondrial	P23434	GCSH	0.012
Astragali Radix（AgR）	MOL000417	Calycosin	Nuclear receptor 0B1	P51843	NR0B1	0.012
Astragali Radix（AgR）	MOL000417	Calycosin	5-hydroxytryptamine 4 receptor	Q13639	HTR4	0.012
Astragali Radix（AgR）	MOL000417	Calycosin	Retinoic acid-induced protein 3	Q8NFJ5	GPRC5A	0.012
Astragali Radix（AgR）	MOL000417	Calycosin	Carbonic anhydrase 1	P00915	CA1	0.013
Astragali Radix（AgR）	MOL000417	Calycosin	Carbonic anhydrase 2	P00918	CA2	0.013
Astragali Radix（AgR）	MOL000417	Calycosin	Interleukin-3	P08700	IL3	0.013
Astragali Radix（AgR）	MOL000417	Calycosin	Glycogen phosphorylase, muscle form	P11217	PYGM	0.013
Astragali Radix（AgR）	MOL000417	Calycosin	Cell division protein kinase 4	P11802	CDK4	0.013
Astragali Radix（AgR）	MOL000417	Calycosin	Carbonic anhydrase 4	P22748	CA4	0.013
Astragali Radix（AgR）	MOL000417	Calycosin	Glycogen synthase kinase-3 beta	P49841	GSK3B	0.013
Astragali Radix（AgR）	MOL000417	Calycosin	Cell division protein kinase 7	P50613	CDK7	0.013
Astragali Radix（AgR）	MOL000417	Calycosin	Cell division protein kinase 9	P50750	CDK9	0.013
Astragali Radix（AgR）	MOL000417	Calycosin	Tubulin beta-2C chain	P68371	TUBB4B	0.013
Astragali Radix（AgR）	MOL000417	Calycosin	Protein S100-A12	P80511	S100A12	0.013
Astragali Radix（AgR）	MOL000417	Calycosin	Cell division protein kinase 6	Q00534	CDK6	0.013
Astragali Radix（AgR）	MOL000417	Calycosin	Cyclin-dependent kinase 5 activator 1	Q15078	CDK5R1	0.013
Astragali Radix（AgR）	MOL000417	Calycosin	Protein S100-A13	Q99584	S100A13	0.013
Astragali Radix（AgR）	MOL000417	Calycosin	Tubulin alpha-6 chain	Q9BQE3	TUBA1C	0.013
Astragali Radix（AgR）	MOL000417	Calycosin	Tubulin beta-2B chain	Q9BVA1	TUBB2B	0.013
Astragali Radix（AgR）	MOL000417	Calycosin	Stathmin-4	Q9H169	STMN4	0.013
Astragali Radix（AgR）	MOL000417	Calycosin	Gamma-aminobutyric acid receptor subunit rho-3	A8MPY1	GABRR3	0.014
Astragali Radix（AgR）	MOL000417	Calycosin	Gamma-aminobutyric acid receptor subunit pi	O00591	GABRP	0.014
Astragali Radix（AgR）	MOL000417	Calycosin	Gamma-aminobutyric acid receptor subunit delta	O14764	GABRD	0.014
Astragali Radix（AgR）	MOL000417	Calycosin	Tyrosine-protein kinase JAK2	O60674	JAK2	0.014
Astragali Radix（AgR）	MOL000417	Calycosin	Gamma-aminobutyric-acid receptor subunit beta-1	P18505	GABRB1	0.014
Astragali Radix（AgR）	MOL000417	Calycosin	Tyrosine-protein kinase JAK1	P23458	JAK1	0.014
Astragali Radix（AgR）	MOL000417	Calycosin	Gamma-aminobutyric-acid receptor subunit rho-1	P24046	GABRR1	0.014
Astragali Radix（AgR）	MOL000417	Calycosin	Gamma-aminobutyric acid receptor subunit rho-2	P28476	GABRR2	0.014
Astragali Radix（AgR）	MOL000417	Calycosin	Amiloride-sensitive sodium channel subunit alpha	P37088	SCNN1A	0.014
Astragali Radix（AgR）	MOL000417	Calycosin	Gamma-aminobutyric-acid receptor subunit beta-2	P47870	GABRB2	0.014
Astragali Radix（AgR）	MOL000417	Calycosin	Amiloride-sensitive sodium channel subunit beta	P51168	SCNN1B	0.014
Astragali Radix（AgR）	MOL000417	Calycosin	Amiloride-sensitive sodium channel subunit gamma	P51170	SCNN1G	0.014
Astragali Radix（AgR）	MOL000417	Calycosin	Tyrosine-protein kinase JAK3	P52333	JAK3	0.014
Astragali Radix（AgR）	MOL000417	Calycosin	Gamma-aminobutyric acid receptor subunit epsilon	P78334	GABRE	0.014
Astragali Radix（AgR）	MOL000417	Calycosin	Gamma-aminobutyric acid receptor subunit gamma-1	Q8N1C3	GABRG1	0.014
Astragali Radix（AgR）	MOL000417	Calycosin	Gamma-aminobutyric acid receptor subunit gamma-3	Q99928	GABRG3	0.014
Astragali Radix（AgR）	MOL000417	Calycosin	Serine/threonine-protein kinase 17B	O94768	STK17B	0.015
Astragali Radix（AgR）	MOL000417	Calycosin	Dihydrofolate reductase	P00374	DHFR	0.015
Astragali Radix（AgR）	MOL000417	Calycosin	Tumor necrosis factor	P01375	TNF	0.015
Astragali Radix（AgR）	MOL000417	Calycosin	Proto-oncogene serine/threonine-protein kinase Pim-1	P11309	PIM1	0.015
Astragali Radix（AgR）	MOL000417	Calycosin	Nuclear factor NF-kappa-B p105 subunit	P19838	NFKB1	0.015
Astragali Radix（AgR）	MOL000417	Calycosin	Fibroblast growth factor receptor 2	P21802	FGFR2	0.015
Astragali Radix（AgR）	MOL000417	Calycosin	Translocator protein	P30536	TSPO	0.015
Astragali Radix（AgR）	MOL000417	Calycosin	ATP synthase subunit gamma, mitochondrial	P36542	ATP5F1C	0.015

Astragali Radix（AgR）	MOL000417	Calycosin	UDP-glucuronosyltransferase 3A1	Q6NUS8	UGT3A1	0.015
Astragali Radix（AgR）	MOL000417	Calycosin	Adenosine A2a receptor	P29274	ADORA2A	0.017
Astragali Radix（AgR）	MOL000417	Calycosin	Adenosine A1 receptor	P30542	ADORA1	0.017
Astragali Radix（AgR）	MOL000417	Calycosin	cAMP-specific 3',5'-cyclic phosphodiesterase 4C	Q08493	PDE4C	0.017
Astragali Radix（AgR）	MOL000417	Calycosin	cGMP-inhibited 3',5'-cyclic phosphodiesterase A	Q14432	PDE3A	0.017
Astragali Radix（AgR）	MOL000417	Calycosin	cAMP and cAMP-inhibited cGMP 3',5'-cyclic phosphodiesterase 10A	Q9Y233	PDE10A	0.017
Astragali Radix（AgR）	MOL000417	Calycosin	Phenylalanine-4-hydroxylase	P00439	PAH	0.019
Astragali Radix（AgR）	MOL000417	Calycosin	DNA topoisomerase 1	P11387	TOP1	0.019
Astragali Radix（AgR）	MOL000417	Calycosin	D(4) dopamine receptor	P21917	DRD4	0.019
Astragali Radix（AgR）	MOL000417	Calycosin	5-hydroxytryptamine 1D receptor	P28221	HTR1D	0.019
Astragali Radix（AgR）	MOL000417	Calycosin	DNA topoisomerase I, mitochondrial	Q969P6	TOP1MT	0.019
Astragali Radix（AgR）	MOL000417	Calycosin	Lipoic acid synthetase, mitochondrial	O43766	LIAS	0.02
Astragali Radix（AgR）	MOL000417	Calycosin	Muscarinic acetylcholine receptor M1	P11229	CHRM1	0.02
Astragali Radix（AgR）	MOL000417	Calycosin	4-aminobutyrate aminotransferase, mitochondrial	P80404	ABAT	0.02
Astragali Radix（AgR）	MOL000417	Calycosin	Peroxisome proliferator-activated receptor delta	Q03181	PPARD	0.02
Astragali Radix（AgR）	MOL000417	Calycosin	Peroxisome proliferator-activated receptor alpha	Q07869	PPARA	0.02
Astragali Radix（AgR）	MOL000417	Calycosin	Histone deacetylase 9	Q9UKV0	HDAC9	0.02
Astragali Radix（AgR）	MOL000417	Calycosin	Lipoyltransferase 1, mitochondrial	Q9Y234	LIPT1	0.02
Astragali Radix（AgR）	MOL000417	Calycosin	Macrophage migration inhibitory factor	P14174	MIF	0.021
Astragali Radix（AgR）	MOL000417	Calycosin	Urokinase-type plasminogen activator	P00749	PLAU	0.022
Astragali Radix（AgR）	MOL000417	Calycosin	Peroxiredoxin-5, mitochondrial	P30044	PRDX5	0.022
Astragali Radix（AgR）	MOL000417	Calycosin	Peptidyl-prolyl cis-trans isomerase, mitochondrial	P30405	PPIF	0.022
Astragali Radix（AgR）	MOL000417	Calycosin	Potassium channel subfamily K member 1	O00180	KCNK1	0.023
Astragali Radix（AgR）	MOL000417	Calycosin	Gamma-aminobutyric-acid receptor subunit beta-3	P28472	GABRB3	0.023
Astragali Radix（AgR）	MOL000417	Calycosin	Oxysterols receptor LXR-beta	P55055	NR1H2	0.023
Astragali Radix（AgR）	MOL000417	Calycosin	Oxysterols receptor LXR-alpha	Q13133	NR1H3	0.023
Astragali Radix（AgR）	MOL000417	Calycosin	Glutamate [NMDA] receptor subunit 3A	Q8TCU5	GRIN3A	0.023
Astragali Radix（AgR）	MOL000417	Calycosin	Sterol O-acyltransferase 2	O75908	SOAT2	0.024
Astragali Radix（AgR）	MOL000417	Calycosin	Ribosyldihydronicotinamide dehydrogenase [quinone]	P16083	NQO2	0.024
Astragali Radix（AgR）	MOL000417	Calycosin	Aryl hydrocarbon receptor	P35869	AHR	0.024
Astragali Radix（AgR）	MOL000417	Calycosin	Dihydroorotate dehydrogenase, mitochondrial	Q02127	DHODH	0.024
Astragali Radix（AgR）	MOL000417	Calycosin	Niemann-Pick C1-like protein 1	Q9UHC9	NPC1L1	0.024
Astragali Radix（AgR）	MOL000417	Calycosin	5-hydroxytryptamine 3 receptor	P46098	HTR3A	0.025
Astragali Radix（AgR）	MOL000417	Calycosin	Inhibitor of nuclear factor kappa-B kinase subunit beta	O14920	IKBKB	0.026
Astragali Radix（AgR）	MOL000417	Calycosin	Cystine/glutamate transporter	Q9UPY5	SLC7A11	0.026
Astragali Radix（AgR）	MOL000417	Calycosin	Mitogen-activated protein kinase 8	P45983	MAPK8	0.027
Astragali Radix（AgR）	MOL000417	Calycosin	Mitogen-activated protein kinase 10	P53779	MAPK10	0.027
Astragali Radix（AgR）	MOL000417	Calycosin	Group IIE secretory phospholipase A2	Q9NZK7	PLA2G2E	0.027
Astragali Radix（AgR）	MOL000417	Calycosin	Estradiol 17-beta-dehydrogenase 1	P14061	HSD17B1	0.028
Astragali Radix（AgR）	MOL000417	Calycosin	RAC-beta serine/threonine-protein kinase	P31751	AKT2	0.028
Astragali Radix（AgR）	MOL000417	Calycosin	Epidermal growth factor receptor	P00533	EGFR	0.029
Astragali Radix（AgR）	MOL000417	Calycosin	Retinoic acid receptor beta	P10826	RARB	0.029
Astragali Radix（AgR）	MOL000417	Calycosin	Phospholipase A2, membrane associated	P14555	PLA2G2A	0.029
Astragali Radix（AgR）	MOL000417	Calycosin	Retinoic acid receptor RXR-gamma	P48443	RXRG	0.029
Astragali Radix（AgR）	MOL000417	Calycosin	Retinoic acid receptor RXR-alpha	P19793	RXRA	0.03
Astragali Radix（AgR）	MOL000417	Calycosin	Potassium voltage-gated channel subfamily H member 2	Q12809	KCNH2	0.03
Astragali Radix（AgR）	MOL000417	Calycosin	Tubulin alpha-3 chain	Q71U36	TUBA1A	0.03

Astragali Radix（AgR）	MOL000417	Calycosin	Potassium voltage-gated channel subfamily H member 6	Q9H252	KCNH6	0.03
Astragali Radix（AgR）	MOL000417	Calycosin	Ig gamma-1 chain C region	P01857	IGHG1	0.031
Astragali Radix（AgR）	MOL000417	Calycosin	5-hydroxytryptamine 1B receptor	P28222	HTR1B	0.031
Astragali Radix（AgR）	MOL000417	Calycosin	5-hydroxytryptamine 2A receptor	P28223	HTR2A	0.031
Astragali Radix（AgR）	MOL000417	Calycosin	5-hydroxytryptamine 2B receptor	P41595	HTR2B	0.031
Astragali Radix（AgR）	MOL000417	Calycosin	Platelet glycoprotein IX	P14770	GP9	0.032
Astragali Radix（AgR）	MOL000417	Calycosin	Acetylcholinesterase	P22303	ACHE	0.033
Astragali Radix（AgR）	MOL000417	Calycosin	ATP synthase subunit beta, mitochondrial	P06576	ATP5F1B	0.034
Astragali Radix（AgR）	MOL000417	Calycosin	Tyrosine-protein kinase HCK	P08631	HCK	0.034
Astragali Radix（AgR）	MOL000417	Calycosin	ATP synthase subunit alpha, mitochondrial	P25705	ATP5F1A	0.034
Astragali Radix（AgR）	MOL000417	Calycosin	3 beta-hydroxysteroid dehydrogenase/Delta 5-->4-isomerase type II	P26439	HSD3B2	0.034
Astragali Radix（AgR）	MOL000417	Calycosin	DNA topoisomerase 2-alpha	P11388	TOP2A	0.036
Astragali Radix（AgR）	MOL000417	Calycosin	Protein tyrosine kinase 2 beta	Q14289	PTK2B	0.036
Astragali Radix（AgR）	MOL000417	Calycosin	Delta-type opioid receptor	P41143	OPRD1	0.037
Astragali Radix（AgR）	MOL000417	Calycosin	Kappa-type opioid receptor	P41145	OPRK1	0.037
Astragali Radix（AgR）	MOL000417	Calycosin	cGMP-specific 3',5'-cyclic phosphodiesterase	O76074	PDE5A	0.038
Astragali Radix（AgR）	MOL000417	Calycosin	Prolactin receptor	P16471	PRLR	0.038
Astragali Radix（AgR）	MOL000417	Calycosin	Muscarinic acetylcholine receptor M2	P08172	CHRM2	0.042
Astragali Radix（AgR）	MOL000417	Calycosin	D(1B) dopamine receptor	P21918	DRD5	0.042
Astragali Radix（AgR）	MOL000417	Calycosin	Glyceraldehyde-3-phosphate dehydrogenase	P04406	GAPDH	0.043
Astragali Radix（AgR）	MOL000417	Calycosin	5-hydroxytryptamine 2C receptor	P28335	HTR2C	0.043
Astragali Radix（AgR）	MOL000417	Calycosin	Gamma-aminobutyric-acid receptor subunit alpha-5	P31644	GABRA5	0.043
Astragali Radix（AgR）	MOL000417	Calycosin	Gamma-aminobutyric-acid receptor subunit alpha-3	P34903	GABRA3	0.043
Astragali Radix（AgR）	MOL000417	Calycosin	Potassium voltage-gated channel subfamily KQT member 1	P51787	KCNQ1	0.043
Astragali Radix（AgR）	MOL000417	Calycosin	Toll-like receptor 7	Q9NYK1	TLR7	0.043
Astragali Radix（AgR）	MOL000417	Calycosin	Ig kappa chain V-II region RPMI 6410	P06310	IGKV2-30	0.044
Astragali Radix（AgR）	MOL000417	Calycosin	Thyroid hormone receptor alpha	P10827	THRA	0.044
Astragali Radix（AgR）	MOL000417	Calycosin	Cytochrome P450 19A1	P11511	CYP19A1	0.044
Astragali Radix（AgR）	MOL000417	Calycosin	Cannabinoid receptor 1	P21554	CNR1	0.044
Astragali Radix（AgR）	MOL000417	Calycosin	Melatonin receptor type 1A	P48039	MTNR1A	0.044
Astragali Radix（AgR）	MOL000417	Calycosin	Serine/threonine-protein kinase 6	O14965	AURKA	0.045
Astragali Radix（AgR）	MOL000417	Calycosin	Retinoic acid receptor alpha	P10276	RARA	0.045
Astragali Radix（AgR）	MOL000417	Calycosin	Retinoic acid receptor gamma-1	P13631	RARG	0.045
Astragali Radix（AgR）	MOL000417	Calycosin	ATP-binding cassette transporter sub-family C member 8	Q09428	ABCC8	0.045
Astragali Radix（AgR）	MOL000417	Calycosin	Casein kinase I isoform gamma-1	Q9HCP0	CSNK1G1	0.045
Astragali Radix（AgR）	MOL000417	Calycosin	Glucocorticoid receptor	P04150	NR3C1	0.046
Astragali Radix（AgR）	MOL000417	Calycosin	5-hydroxytryptamine 1A receptor	P08908	HTR1A	0.046
Astragali Radix（AgR）	MOL000417	Calycosin	Thyroid hormone receptor beta-1	P10828	THRB	0.046
Astragali Radix（AgR）	MOL000417	Calycosin	Aldo-keto reductase family 1 member C1	Q04828	AKR1C1	0.046
Astragali Radix（AgR）	MOL000417	Calycosin	S-methyl-5-thioadenosine phosphorylase	Q13126	MTAP	0.047
Astragali Radix（AgR）	MOL000417	Calycosin	Neutrophil gelatinase-associated lipocalin	P80188	LCN2	0.048
Astragali Radix（AgR）	MOL000417	Calycosin	cAMP-specific 3',5'-cyclic phosphodiesterase 4D	Q08499	PDE4D	0.048
Astragali Radix（AgR）	MOL000417	Calycosin	Tyrosine-protein phosphatase non-receptor type 1	P18031	PTPN1	0.049
Astragali Radix（AgR）	MOL000417	Calycosin	Casein kinase II subunit alpha	P68400	CSNK2A1	0.05
Astragali Radix（AgR）	MOL000417	Calycosin	Proto-oncogene tyrosine-protein kinase Src	P12931	SRC	0.051
Astragali Radix（AgR）	MOL000417	Calycosin	Beta-1 adrenergic receptor	P08588	ADRB1	0.052
Astragali Radix（AgR）	MOL000417	Calycosin	Sterol O-acyltransferase 1	P35610	SOAT1	0.052

Astragali Radix（AgR）	MOL000417	Calycosin	Nuclear receptor coactivator 5	Q9HCD5	NCOA5	0.052
Astragali Radix（AgR）	MOL000417	Calycosin	Phosphatidylinositol-4,5-bisphosphate 3-kinase catalytic subunit gamma isoform	P48736	PIK3CG	0.053
Astragali Radix（AgR）	MOL000417	Calycosin	Sodium channel protein type 5 subunit alpha	Q14524	SCN5A	0.053
Astragali Radix（AgR）	MOL000417	Calycosin	Interferon gamma	P01579	IFNG	0.054
Astragali Radix（AgR）	MOL000417	Calycosin	D(1A) dopamine receptor	P21728	DRD1	0.054
Astragali Radix（AgR）	MOL000417	Calycosin	D(3) dopamine receptor	P35462	DRD3	0.054
Astragali Radix（AgR）	MOL000417	Calycosin	Sodium-dependent serotonin transporter	P31645	SLC6A4	0.055
Astragali Radix（AgR）	MOL000417	Calycosin	Inhibitor of nuclear factor kappa-B kinase subunit alpha	O15111	CHUK	0.056
Astragali Radix（AgR）	MOL000417	Calycosin	Sodium-dependent dopamine transporter	Q01959	SLC6A3	0.057
Astragali Radix（AgR）	MOL000417	Calycosin	cAMP-specific 3',5'-cyclic phosphodiesterase 4B	Q07343	PDE4B	0.059
Astragali Radix（AgR）	MOL000417	Calycosin	cAMP-dependent protein kinase inhibitor alpha	P61925	PKIA	0.06
Astragali Radix（AgR）	MOL000417	Calycosin	Rho-associated protein kinase 1	Q13464	ROCK1	0.06
Astragali Radix（AgR）	MOL000417	Calycosin	D-HSCDK2	O75100	CA11	0.062
Astragali Radix（AgR）	MOL000417	Calycosin	Phospholipase A2	P04054	PLA2G1B	0.062
Astragali Radix（AgR）	MOL000417	Calycosin	Beta-2 adrenergic receptor	P07550	ADRB2	0.063
Astragali Radix（AgR）	MOL000417	Calycosin	Ig kappa chain C region	P01834	IGKC	0.065
Astragali Radix（AgR）	MOL000417	Calycosin	Gamma-aminobutyric-acid receptor subunit alpha-1	P14867	GABRA1	0.065
Astragali Radix（AgR）	MOL000417	Calycosin	Alpha-2C adrenergic receptor	P18825	ADRA2C	0.065
Astragali Radix（AgR）	MOL000417	Calycosin	3 beta-hydroxysteroid dehydrogenase/Delta 5-->4-isomerase type I	P14060	HSD3B1	0.066
Astragali Radix（AgR）	MOL000417	Calycosin	Mu-type opioid receptor	P35372	OPRM1	0.07
Astragali Radix（AgR）	MOL000417	Calycosin	Gamma-aminobutyric-acid receptor subunit alpha-2	P47869	GABRA2	0.074
Astragali Radix（AgR）	MOL000417	Calycosin	Alpha-1D adrenergic receptor	P25100	ADRA1D	0.075
Astragali Radix（AgR）	MOL000417	Calycosin	Alpha-2A adrenergic receptor	P08913	ADRA2A	0.076
Astragali Radix（AgR）	MOL000417	Calycosin	Alpha-2B adrenergic receptor	P18089	ADRA2B	0.076
Astragali Radix（AgR）	MOL000417	Calycosin	Cell division control protein 2 homolog	P06493	CDK1	0.078
Astragali Radix（AgR）	MOL000417	Calycosin	Cell division protein kinase 2	P24941	CDK2	0.078
Astragali Radix（AgR）	MOL000417	Calycosin	Sodium-dependent noradrenaline transporter	P23975	SLC6A2	0.079
Astragali Radix（AgR）	MOL000417	Calycosin	Peroxisome proliferator-activated receptor gamma	P37231	PPARG	0.08
Astragali Radix（AgR）	MOL000417	Calycosin	Estrogen-related receptor gamma	P62508	ESRRG	0.082
Astragali Radix（AgR）	MOL000417	Calycosin	Arachidonate 5-lipoxygenase	P09917	ALOX5	0.084
Astragali Radix（AgR）	MOL000417	Calycosin	C-jun-amino-terminal kinase-interacting protein 1	Q9UQF2	MAPK8IP1	0.087
Astragali Radix（AgR）	MOL000417	Calycosin	D(2) dopamine receptor	P14416	DRD2	0.089
Astragali Radix（AgR）	MOL000417	Calycosin	Retinoic acid receptor RXR-beta	P28702	RXRB	0.089
Astragali Radix（AgR）	MOL000417	Calycosin	Cyclin-A2	P20248	CCNA2	0.092
Astragali Radix（AgR）	MOL000417	Calycosin	Coagulation factor VII	P08709	F7	0.093
Astragali Radix（AgR）	MOL000417	Calycosin	Nitric oxide synthase, inducible	P35228	NOS2	0.094
Astragali Radix（AgR）	MOL000417	Calycosin	Cell division protein kinase 5	Q00535	CDK5	0.094
Astragali Radix（AgR）	MOL000417	Calycosin	Prothrombin	P00734	F2	0.096
Astragali Radix（AgR）	MOL000417	Calycosin	RAC-alpha serine/threonine-protein kinase	P31749	AKT1	0.096
Astragali Radix（AgR）	MOL000417	Calycosin	Alpha-1B adrenergic receptor	P35368	ADRA1B	0.097
Astragali Radix（AgR）	MOL000417	Calycosin	MAP kinase-activated protein kinase 2	P49137	MAPKAPK2	0.099
Astragali Radix（AgR）	MOL000417	Calycosin	cAMP-specific 3',5'-cyclic phosphodiesterase 4A	P27815	PDE4A	0.1
Astragali Radix（AgR）	MOL000417	Calycosin	ATP-sensitive inward rectifier potassium channel 1	P48048	KCNJ1	0.1
Astragali Radix（AgR）	MOL000417	Calycosin	Hemoglobin subunit alpha	P69905	HBA1	0.101
Astragali Radix（AgR）	MOL000417	Calycosin	Nuclear receptor coactivator 2	Q15596	NCOA2	0.104
Astragali Radix（AgR）	MOL000417	Calycosin	Prostaglandin G/H synthase 1	P23219	PTGS1	0.109
Astragali Radix（AgR）	MOL000417	Calycosin	Alpha-1A adrenergic receptor	P35348	ADRA1A	0.12

Astragali Radix（AgR）	MOL000417	Calycosin	Androgen receptor	P10275	AR	0.124
Astragali Radix（AgR）	MOL000417	Calycosin	Mitogen-activated protein kinase 14	Q16539	MAPK14	0.144
Astragali Radix（AgR）	MOL000417	Calycosin	Trypsin-1	P07477	PRSS1	0.178
Astragali Radix（AgR）	MOL000417	Calycosin	Nuclear receptor coactivator 1	Q15788	NCOA1	0.245
Astragali Radix（AgR）	MOL000417	Calycosin	cAMP-dependent protein kinase catalytic subunit alpha	P17612	PRKACA	0.284
Astragali Radix（AgR）	MOL000417	Calycosin	Progesterone receptor	P06401	PGR	0.297
Astragali Radix（AgR）	MOL000417	Calycosin	Estrogen receptor beta	Q92731	ESR2	0.412
Astragali Radix（AgR）	MOL000417	Calycosin	Prostaglandin G/H synthase 2	P35354	PTGS2	0.415
Astragali Radix（AgR）	MOL000417	Calycosin	Estrogen receptor	P03372	ESR1	1
Astragali Radix（AgR）	MOL000422	kaempferol	Substance-P receptor	P25103	TACR1	0.01
Astragali Radix（AgR）	MOL000422	kaempferol	5-hydroxytryptamine 3 receptor	P46098	HTR3A	0.01
Astragali Radix（AgR）	MOL000422	kaempferol	Sodium channel protein type 10 subunit alpha	Q9Y5Y9	SCN10A	0.01
Astragali Radix（AgR）	MOL000422	kaempferol	Phosphatidylinositol 3-kinase regulatory subunit beta	O00459	PIK3R2	0.011
Astragali Radix（AgR）	MOL000422	kaempferol	Death-associated protein kinase 3	O43293	DAPK3	0.011
Astragali Radix（AgR）	MOL000422	kaempferol	Tyrosine-protein kinase JAK2	O60674	JAK2	0.011
Astragali Radix（AgR）	MOL000422	kaempferol	T-cell receptor alpha chain C region	P01848	TRAC	0.011
Astragali Radix（AgR）	MOL000422	kaempferol	T-cell receptor beta chain C region	P01850	TRBC1	0.011
Astragali Radix（AgR）	MOL000422	kaempferol	Trypsin-2	P07478	PRSS2	0.011
Astragali Radix（AgR）	MOL000422	kaempferol	Elongation factor 2	P13639	EEF2	0.011
Astragali Radix（AgR）	MOL000422	kaempferol	Beta-3 adrenergic receptor	P13945	ADRB3	0.011
Astragali Radix（AgR）	MOL000422	kaempferol	Tyrosine-protein kinase JAK1	P23458	JAK1	0.011
Astragali Radix（AgR）	MOL000422	kaempferol	Phosphatidylinositol 3-kinase regulatory subunit alpha	P27986	PIK3R1	0.011
Astragali Radix（AgR）	MOL000422	kaempferol	Mitogen-activated protein kinase 1	P28482	MAPK1	0.011
Astragali Radix（AgR）	MOL000422	kaempferol	Tyrosine-protein kinase JAK3	P52333	JAK3	0.011
Astragali Radix（AgR）	MOL000422	kaempferol	Beta-2-microglobulin	P61769	B2M	0.011
Astragali Radix（AgR）	MOL000422	kaempferol	Poly [ADP-ribose] polymerase 3	Q9Y6F1	PARP3	0.011
Astragali Radix（AgR）	MOL000422	kaempferol	Potassium channel subfamily K member 6	Q9Y257	KCNK6	0.012
Astragali Radix（AgR）	MOL000422	kaempferol	Gamma-aminobutyric acid receptor subunit rho-3	A8MPY1	GABRR3	0.013
Astragali Radix（AgR）	MOL000422	kaempferol	Gamma-aminobutyric acid receptor subunit pi	O00591	GABRP	0.013
Astragali Radix（AgR）	MOL000422	kaempferol	Gamma-aminobutyric acid receptor subunit delta	O14764	GABRD	0.013
Astragali Radix（AgR）	MOL000422	kaempferol	Gamma-aminobutyric-acid receptor subunit beta-1	P18505	GABRB1	0.013
Astragali Radix（AgR）	MOL000422	kaempferol	Gamma-aminobutyric acid receptor subunit gamma-2	P18507	GABRG2	0.013
Astragali Radix（AgR）	MOL000422	kaempferol	Gamma-aminobutyric-acid receptor subunit rho-1	P24046	GABRR1	0.013
Astragali Radix（AgR）	MOL000422	kaempferol	Gamma-aminobutyric acid receptor subunit rho-2	P28476	GABRR2	0.013
Astragali Radix（AgR）	MOL000422	kaempferol	Gamma-aminobutyric-acid receptor subunit alpha-5	P31644	GABRA5	0.013
Astragali Radix（AgR）	MOL000422	kaempferol	Gamma-aminobutyric-acid receptor subunit alpha-3	P34903	GABRA3	0.013
Astragali Radix（AgR）	MOL000422	kaempferol	Gamma-aminobutyric acid receptor subunit epsilon	P78334	GABRE	0.013
Astragali Radix（AgR）	MOL000422	kaempferol	Gamma-aminobutyric acid receptor subunit gamma-1	Q8N1C3	GABRG1	0.013
Astragali Radix（AgR）	MOL000422	kaempferol	Gamma-aminobutyric acid receptor subunit gamma-3	Q99928	GABRG3	0.013
Astragali Radix（AgR）	MOL000422	kaempferol	Sucrase-isomaltase, intestinal	P14410	SI	0.014
Astragali Radix（AgR）	MOL000422	kaempferol	Interleukin-3	P08700	IL3	0.015
Astragali Radix（AgR）	MOL000422	kaempferol	Protein S100-A12	P80511	S100A12	0.015
Astragali Radix（AgR）	MOL000422	kaempferol	Protein S100-A13	Q99584	S100A13	0.015
Astragali Radix（AgR）	MOL000422	kaempferol	Phenylalanine-4-hydroxylase	P00439	PAH	0.016
Astragali Radix（AgR）	MOL000422	kaempferol	Cell division protein kinase 4	P11802	CDK4	0.017
Astragali Radix（AgR）	MOL000422	kaempferol	Cell division protein kinase 7	P50613	CDK7	0.017
Astragali Radix（AgR）	MOL000422	kaempferol	Cell division protein kinase 9	P50750	CDK9	0.017

Astragali Radix（AgR）	MOL000422	kaempferol	Cell division protein kinase 6	Q00534	CDK6	0.017
Astragali Radix（AgR）	MOL000422	kaempferol	Serine/threonine-protein kinase 17B	O94768	STK17B	0.018
Astragali Radix（AgR）	MOL000422	kaempferol	Proto-oncogene serine/threonine-protein kinase Pim-1	P11309	PIM1	0.018
Astragali Radix（AgR）	MOL000422	kaempferol	ATP synthase subunit gamma, mitochondrial	P36542	ATP5F1C	0.018
Astragali Radix（AgR）	MOL000422	kaempferol	Neuronal acetylcholine receptor subunit alpha-2	Q15822	CHRNA2	0.018
Astragali Radix（AgR）	MOL000422	kaempferol	UDP-glucuronosyltransferase 3A1	Q6NUS8	UGT3A1	0.018
Astragali Radix（AgR）	MOL000422	kaempferol	Pepsin A	P00790	REN	0.019
Astragali Radix（AgR）	MOL000422	kaempferol	Tyrosine 3-monooxygenase	P07101	TH	0.019
Astragali Radix（AgR）	MOL000422	kaempferol	Retinoic acid receptor alpha	P10276	RARA	0.019
Astragali Radix（AgR）	MOL000422	kaempferol	Retinoic acid receptor beta	P10826	RARB	0.019
Astragali Radix（AgR）	MOL000422	kaempferol	Retinoic acid receptor gamma-1	P13631	RARG	0.019
Astragali Radix（AgR）	MOL000422	kaempferol	Dipeptidyl peptidase 4	P27487	DPP4	0.019
Astragali Radix（AgR）	MOL000422	kaempferol	Amiloride-sensitive sodium channel subunit alpha	P37088	SCNN1A	0.019
Astragali Radix（AgR）	MOL000422	kaempferol	Retinoic acid receptor RXR-gamma	P48443	RXRG	0.019
Astragali Radix（AgR）	MOL000422	kaempferol	Amiloride-sensitive sodium channel subunit beta	P51168	SCNN1B	0.019
Astragali Radix（AgR）	MOL000422	kaempferol	Amiloride-sensitive sodium channel subunit gamma	P51170	SCNN1G	0.019
Astragali Radix（AgR）	MOL000422	kaempferol	Peptidyl-prolyl cis-trans isomerase NIMA-interacting 1	Q13526	PIN1	0.019
Astragali Radix（AgR）	MOL000422	kaempferol	Cyclin-dependent kinase 5 activator 1	Q15078	CDK5R1	0.019
Astragali Radix（AgR）	MOL000422	kaempferol	Tyrosyl-tRNA synthetase, mitochondrial	Q9Y2Z4	YARS2	0.019
Astragali Radix（AgR）	MOL000422	kaempferol	Adenosine A2b receptor	P29275	ADORA2B	0.02
Astragali Radix（AgR）	MOL000422	kaempferol	Adenosine A3 receptor	P33765	SHMT1	0.02
Astragali Radix（AgR）	MOL000422	kaempferol	Prostaglandin reductase 1	Q14914	PTGR1	0.02
Astragali Radix（AgR）	MOL000422	kaempferol	Potassium voltage-gated channel subfamily H member 2	Q12809	KCNH2	0.021
Astragali Radix（AgR）	MOL000422	kaempferol	Potassium voltage-gated channel subfamily H member 6	Q9H252	KCNH6	0.021
Astragali Radix（AgR）	MOL000422	kaempferol	cGMP-specific 3',5'-cyclic phosphodiesterase	O76074	PDE5A	0.022
Astragali Radix（AgR）	MOL000422	kaempferol	Carbonic anhydrase 4	P22748	CA4	0.022
Astragali Radix（AgR）	MOL000422	kaempferol	Adenosine A1 receptor	P30542	ADORA1	0.022
Astragali Radix（AgR）	MOL000422	kaempferol	Sodium/potassium-transporting ATPase gamma chain	P54710	FXYD2	0.022
Astragali Radix（AgR）	MOL000422	kaempferol	cAMP and cAMP-inhibited cGMP 3',5'-cyclic phosphodiesterase 10A	Q9Y233	PDE10A	0.023
Astragali Radix（AgR）	MOL000422	kaempferol	Tryptophanyl-tRNA synthetase, mitochondrial	Q9UGM6	WARS2	0.024
Astragali Radix（AgR）	MOL000422	kaempferol	Potassium channel subfamily K member 1	O00180	KCNK1	0.025
Astragali Radix（AgR）	MOL000422	kaempferol	Tripartite motif-containing protein 13	O60858	TRIM13	0.025
Astragali Radix（AgR）	MOL000422	kaempferol	Prostacyclin receptor	P43119	PTGIR	0.025
Astragali Radix（AgR）	MOL000422	kaempferol	Methionine aminopeptidase 1	P53582	METAP1	0.025
Astragali Radix（AgR）	MOL000422	kaempferol	Peroxisome proliferator-activated receptor delta	Q03181	PPARD	0.025
Astragali Radix（AgR）	MOL000422	kaempferol	Succinate dehydrogenase [ubiquinone] cytochrome b small subunit, mitochondrial	O14521	SDHD	0.026
Astragali Radix（AgR）	MOL000422	kaempferol	Ig kappa chain C region	P01834	IGKC	0.026
Astragali Radix（AgR）	MOL000422	kaempferol	Ig gamma-1 chain C region	P01857	IGHG1	0.026
Astragali Radix（AgR）	MOL000422	kaempferol	Chymase	P23946	CMA1	0.026
Astragali Radix（AgR）	MOL000422	kaempferol	Gamma-aminobutyric-acid receptor subunit alpha-2	P47869	GABRA2	0.026
Astragali Radix（AgR）	MOL000422	kaempferol	Ribosyldihydronicotinamide dehydrogenase [quinone]	P16083	NQO2	0.027
Astragali Radix（AgR）	MOL000422	kaempferol	Peptidyl-prolyl cis-trans isomerase, mitochondrial	P30405	PPIF	0.027
Astragali Radix（AgR）	MOL000422	kaempferol	Mitogen-activated protein kinase 8	P45983	MAPK8	0.027
Astragali Radix（AgR）	MOL000422	kaempferol	Mitogen-activated protein kinase 10	P53779	MAPK10	0.027
Astragali Radix（AgR）	MOL000422	kaempferol	Inhibitor of nuclear factor kappa-B kinase subunit beta	O14920	IKBKB	0.028
Astragali Radix（AgR）	MOL000422	kaempferol	Oxysterols receptor LXR-beta	P55055	NR1H2	0.028
Astragali Radix（AgR）	MOL000422	kaempferol	Oxysterols receptor LXR-alpha	Q13133	NR1H3	0.028

Astragali Radix（AgR）	MOL000422	kaempferol	Cystine/glutamate transporter	Q9UPY5	SLC7A11	0.028
Astragali Radix（AgR）	MOL000422	kaempferol	Proto-oncogene tyrosine-protein kinase LCK	P06239	LCK	0.031
Astragali Radix（AgR）	MOL000422	kaempferol	Tyrosine-protein kinase Lyn	P07948	LYN	0.031
Astragali Radix（AgR）	MOL000422	kaempferol	Alpha-2A adrenergic receptor	P08913	ADRA2A	0.032
Astragali Radix（AgR）	MOL000422	kaempferol	Macrophage migration inhibitory factor	P14174	MIF	0.032
Astragali Radix（AgR）	MOL000422	kaempferol	DNA-(apurinic or apyrimidinic site) lyase	P27695	APEX1	0.032
Astragali Radix（AgR）	MOL000422	kaempferol	5-hydroxytryptamine 1D receptor	P28221	HTR1D	0.032
Astragali Radix（AgR）	MOL000422	kaempferol	5-hydroxytryptamine 1B receptor	P28222	HTR1B	0.032
Astragali Radix（AgR）	MOL000422	kaempferol	Sterol O-acyltransferase 2	O75908	SOAT2	0.033
Astragali Radix（AgR）	MOL000422	kaempferol	Glycogen phosphorylase, muscle form	P11217	PYGM	0.033
Astragali Radix（AgR）	MOL000422	kaempferol	Sterol O-acyltransferase 1	P35610	SOAT1	0.033
Astragali Radix（AgR）	MOL000422	kaempferol	Keratin, type II cytoskeletal 7	P08729	KRT7	0.034
Astragali Radix（AgR）	MOL000422	kaempferol	Platelet glycoprotein IX	P14770	GP9	0.034
Astragali Radix（AgR）	MOL000422	kaempferol	Gonadotropin-releasing hormone receptor	P30968	GNRHR	0.035
Astragali Radix（AgR）	MOL000422	kaempferol	Gonadotropin-releasing hormone II receptor	Q96P88	GNRHR2	0.035
Astragali Radix（AgR）	MOL000422	kaempferol	Gamma-aminobutyric-acid receptor subunit alpha-1	P14867	GABRA1	0.036
Astragali Radix（AgR）	MOL000422	kaempferol	Prolactin receptor	P16471	PRLR	0.036
Astragali Radix（AgR）	MOL000422	kaempferol	Tubulin beta-2C chain	P68371	TUBB4B	0.036
Astragali Radix（AgR）	MOL000422	kaempferol	Beta-1 adrenergic receptor	P08588	ADRB1	0.037
Astragali Radix（AgR）	MOL000422	kaempferol	Protein tyrosine kinase 2 beta	Q14289	PTK2B	0.037
Astragali Radix（AgR）	MOL000422	kaempferol	Glutamate [NMDA] receptor subunit 3A	Q8TCU5	GRIN3A	0.037
Astragali Radix（AgR）	MOL000422	kaempferol	Epidermal growth factor receptor	P00533	EGFR	0.038
Astragali Radix（AgR）	MOL000422	kaempferol	Microtubule-associated protein 2	P11137	MAP2	0.038
Astragali Radix（AgR）	MOL000422	kaempferol	3 beta-hydroxysteroid dehydrogenase/Delta 5-->4-isomerase type I	P14060	HSD3B1	0.038
Astragali Radix（AgR）	MOL000422	kaempferol	3 beta-hydroxysteroid dehydrogenase/Delta 5-->4-isomerase type II	P26439	HSD3B2	0.038
Astragali Radix（AgR）	MOL000422	kaempferol	Estrogen-related receptor gamma	P62508	ESRRG	0.038
Astragali Radix（AgR）	MOL000422	kaempferol	Microtubule-associated protein 1A	P78559	MAP1A	0.038
Astragali Radix（AgR）	MOL000422	kaempferol	Sodium-dependent dopamine transporter	Q01959	SLC6A3	0.038
Astragali Radix（AgR）	MOL000422	kaempferol	ATP synthase subunit beta, mitochondrial	P06576	ATP5F1B	0.04
Astragali Radix（AgR）	MOL000422	kaempferol	Tyrosine-protein kinase HCK	P08631	HCK	0.04
Astragali Radix（AgR）	MOL000422	kaempferol	Acetylcholinesterase	P22303	ACHE	0.04
Astragali Radix（AgR）	MOL000422	kaempferol	ATP synthase subunit alpha, mitochondrial	P25705	ATP5F1A	0.04
Astragali Radix（AgR）	MOL000422	kaempferol	Phosphatidylinositol-4,5-bisphosphate 3-kinase catalytic subunit gamma isoform	P48736	PIK3CG	0.04
Astragali Radix（AgR）	MOL000422	kaempferol	Glycogen synthase kinase-3 beta	P49841	GSK3B	0.042
Astragali Radix（AgR）	MOL000422	kaempferol	Alpha-2B adrenergic receptor	P18089	ADRA2B	0.044
Astragali Radix（AgR）	MOL000422	kaempferol	Alpha-2C adrenergic receptor	P18825	ADRA2C	0.044
Astragali Radix（AgR）	MOL000422	kaempferol	D(4) dopamine receptor	P21917	DRD4	0.044
Astragali Radix（AgR）	MOL000422	kaempferol	Alpha-1D adrenergic receptor	P25100	ADRA1D	0.045
Astragali Radix（AgR）	MOL000422	kaempferol	Adenosine A2a receptor	P29274	ADORA2A	0.047
Astragali Radix（AgR）	MOL000422	kaempferol	Muscarinic acetylcholine receptor M4	P08173	CHRM4	0.048
Astragali Radix（AgR）	MOL000422	kaempferol	Carbonic anhydrase 1	P00915	CA1	0.049
Astragali Radix（AgR）	MOL000422	kaempferol	cGMP-inhibited 3',5'-cyclic phosphodiesterase A	Q14432	PDE3A	0.05
Astragali Radix（AgR）	MOL000422	kaempferol	Beta-2 adrenergic receptor	P07550	ADRB2	0.051
Astragali Radix（AgR）	MOL000422	kaempferol	Cannabinoid receptor 1	P21554	CNR1	0.052
Astragali Radix（AgR）	MOL000422	kaempferol	Retinoic acid receptor RXR-beta	P28702	RXRB	0.052
Astragali Radix（AgR）	MOL000422	kaempferol	Dual specificity protein kinase CLK1	P49759	CLK1	0.052
Astragali Radix（AgR）	MOL000422	kaempferol	cAMP-dependent protein kinase inhibitor alpha	P61925	PKIA	0.052

Astragali Radix（AgR）	MOL000422	kaempferol	Aldo-keto reductase family 1 member C1	Q04828	AKR1C1	0.052
Astragali Radix（AgR）	MOL000422	kaempferol	Tubulin beta-1 chain	Q9H4B7	TUBB1	0.052
Astragali Radix（AgR）	MOL000422	kaempferol	Serine/threonine-protein kinase 6	O14965	AURKA	0.053
Astragali Radix（AgR）	MOL000422	kaempferol	Cytochrome P450 19A1	P11511	CYP19A1	0.053
Astragali Radix（AgR）	MOL000422	kaempferol	Egl nine homolog 1	Q9GZT9	EGLN1	0.053
Astragali Radix（AgR）	MOL000422	kaempferol	Leukotriene A-4 hydrolase	P09960	LTA4H	0.054
Astragali Radix（AgR）	MOL000422	kaempferol	RAC-alpha serine/threonine-protein kinase	P31749	AKT1	0.054
Astragali Radix（AgR）	MOL000422	kaempferol	Membrane copper amine oxidase	Q16853	AOC3	0.054
Astragali Radix（AgR）	MOL000422	kaempferol	Dihydrofolate reductase	P00374	DHFR	0.055
Astragali Radix（AgR）	MOL000422	kaempferol	Glucocorticoid receptor	P04150	NR3C1	0.055
Astragali Radix（AgR）	MOL000422	kaempferol	Thymidylate synthase	P04818	TYMS	0.055
Astragali Radix（AgR）	MOL000422	kaempferol	Thyroid hormone receptor beta-1	P10828	THRB	0.055
Astragali Radix（AgR）	MOL000422	kaempferol	Ig kappa chain V-II region RPMI 6410	P06310	IGKV2-30	0.056
Astragali Radix（AgR）	MOL000422	kaempferol	5-hydroxytryptamine 1A receptor	P08908	HTR1A	0.056
Astragali Radix（AgR）	MOL000422	kaempferol	D(1B) dopamine receptor	P21918	DRD5	0.056
Astragali Radix（AgR）	MOL000422	kaempferol	D(3) dopamine receptor	P35462	DRD3	0.056
Astragali Radix（AgR）	MOL000422	kaempferol	Triosephosphate isomerase	P60174	TPI1	0.056
Astragali Radix（AgR）	MOL000422	kaempferol	Calmodulin	P62158		0.056
Astragali Radix（AgR）	MOL000422	kaempferol	S-methyl-5-thioadenosine phosphorylase	Q13126	MTAP	0.056
Astragali Radix（AgR）	MOL000422	kaempferol	Thyroid hormone receptor alpha	P10827	THRA	0.057
Astragali Radix（AgR）	MOL000422	kaempferol	5-hydroxytryptamine 2C receptor	P28335	HTR2C	0.057
Astragali Radix（AgR）	MOL000422	kaempferol	5-hydroxytryptamine 2B receptor	P41595	HTR2B	0.057
Astragali Radix（AgR）	MOL000422	kaempferol	Solute carrier family 12 member 1	Q13621	SLC12A1	0.057
Astragali Radix（AgR）	MOL000422	kaempferol	Sodium channel protein type 5 subunit alpha	Q14524	SCN5A	0.057
Astragali Radix（AgR）	MOL000422	kaempferol	Toll-like receptor 7	Q9NYK1	TLR7	0.057
Astragali Radix（AgR）	MOL000422	kaempferol	Calcium/calmodulin-dependent protein kinase type II alpha chain	Q9UQM7	CAMK2A	0.057
Astragali Radix（AgR）	MOL000422	kaempferol	Alpha-1B adrenergic receptor	P35368	ADRA1B	0.058
Astragali Radix（AgR）	MOL000422	kaempferol	Casein kinase II subunit alpha	P68400	CSNK2A1	0.058
Astragali Radix（AgR）	MOL000422	kaempferol	Thiamin pyrophosphokinase 1	Q9H3S4	TPK1	0.058
Astragali Radix（AgR）	MOL000422	kaempferol	C-jun-amino-terminal kinase-interacting protein 1	Q9UQF2	MAPK8IP1	0.058
Astragali Radix（AgR）	MOL000422	kaempferol	Peroxisome proliferator-activated receptor gamma	P37231	PPARG	0.059
Astragali Radix（AgR）	MOL000422	kaempferol	Neuropeptide Y	P01303	NPY	0.06
Astragali Radix（AgR）	MOL000422	kaempferol	Nuclear receptor coactivator 2	Q15596	NCOA2	0.06
Astragali Radix（AgR）	MOL000422	kaempferol	Group IIE secretory phospholipase A2	Q9NZK7	PLA2G2E	0.06
Astragali Radix（AgR）	MOL000422	kaempferol	Nuclear receptor coactivator 5	Q9HCD5	NCOA5	0.062
Astragali Radix（AgR）	MOL000422	kaempferol	Interferon gamma	P01579	IFNG	0.063
Astragali Radix（AgR）	MOL000422	kaempferol	Phospholipase A2	P04054	PLA2G1B	0.063
Astragali Radix（AgR）	MOL000422	kaempferol	Fibroblast growth factor receptor 2	P21802	FGFR2	0.063
Astragali Radix（AgR）	MOL000422	kaempferol	Proto-oncogene tyrosine-protein kinase Src	P12931	SRC	0.066
Astragali Radix（AgR）	MOL000422	kaempferol	Muscarinic acetylcholine receptor M3	P20309	CHRM3	0.066
Astragali Radix（AgR）	MOL000422	kaempferol	Tyrosyl-tRNA synthetase, cytoplasmic	P54577	YARS	0.066
Astragali Radix（AgR）	MOL000422	kaempferol	DNA polymerase kappa	Q9UBT6	POLK	0.068
Astragali Radix（AgR）	MOL000422	kaempferol	DNA topoisomerase 2-alpha	P11388	TOP2A	0.072
Astragali Radix（AgR）	MOL000422	kaempferol	Progesterone receptor	P06401	PGR	0.074
Astragali Radix（AgR）	MOL000422	kaempferol	5-hydroxytryptamine 2A receptor	P28223	HTR2A	0.074
Astragali Radix（AgR）	MOL000422	kaempferol	Carbonic anhydrase 2	P00918	CA2	0.075
Astragali Radix（AgR）	MOL000422	kaempferol	Tubulin alpha-3 chain	Q71U36	TUBA1A	0.077

Astragali Radix（AgR）	MOL000422	kaempferol	D(1A) dopamine receptor	P21728	DRD1	0.081
Astragali Radix（AgR）	MOL000422	kaempferol	D-HSCDK2	O75100	CA11	0.084
Astragali Radix（AgR）	MOL000422	kaempferol	Muscarinic acetylcholine receptor M2	P08172	CHRM2	0.084
Astragali Radix（AgR）	MOL000422	kaempferol	Delta-type opioid receptor	P41143	OPRD1	0.085
Astragali Radix（AgR）	MOL000422	kaempferol	Kappa-type opioid receptor	P41145	OPRK1	0.085
Astragali Radix（AgR）	MOL000422	kaempferol	Inhibitor of nuclear factor kappa-B kinase subunit alpha	O15111	CHUK	0.091
Astragali Radix（AgR）	MOL000422	kaempferol	Arachidonate 5-lipoxygenase	P09917	ALOX5	0.091
Astragali Radix（AgR）	MOL000422	kaempferol	D(2) dopamine receptor	P14416	DRD2	0.093
Astragali Radix（AgR）	MOL000422	kaempferol	Sodium-dependent noradrenaline transporter	P23975	SLC6A2	0.094
Astragali Radix（AgR）	MOL000422	kaempferol	Alpha-1A adrenergic receptor	P35348	ADRA1A	0.098
Astragali Radix（AgR）	MOL000422	kaempferol	Muscarinic acetylcholine receptor M1	P11229	CHRM1	0.099
Astragali Radix（AgR）	MOL000422	kaempferol	cAMP-specific 3',5'-cyclic phosphodiesterase 4A	P27815	PDE4A	0.104
Astragali Radix（AgR）	MOL000422	kaempferol	Sodium-dependent serotonin transporter	P31645	SLC6A4	0.105
Astragali Radix（AgR）	MOL000422	kaempferol	Prothrombin	P00734	F2	0.106
Astragali Radix（AgR）	MOL000422	kaempferol	Cell division control protein 2 homolog	P06493	CDK1	0.107
Astragali Radix（AgR）	MOL000422	kaempferol	Nitric oxide synthase, inducible	P35228	NOS2	0.113
Astragali Radix（AgR）	MOL000422	kaempferol	cAMP-specific 3',5'-cyclic phosphodiesterase 4D	Q08499	PDE4D	0.113
Astragali Radix（AgR）	MOL000422	kaempferol	Androgen receptor	P10275	AR	0.115
Astragali Radix（AgR）	MOL000422	kaempferol	cAMP-dependent protein kinase catalytic subunit alpha	P17612	PRKACA	0.116
Astragali Radix（AgR）	MOL000422	kaempferol	Cell division protein kinase 5	Q00535	CDK5	0.129
Astragali Radix（AgR）	MOL000422	kaempferol	cAMP-specific 3',5'-cyclic phosphodiesterase 4B	Q07343	PDE4B	0.131
Astragali Radix（AgR）	MOL000422	kaempferol	Cell division protein kinase 2	P24941	CDK2	0.153
Astragali Radix（AgR）	MOL000422	kaempferol	Trypsin-1	P07477	PRSS1	0.157
Astragali Radix（AgR）	MOL000422	kaempferol	MAP kinase-activated protein kinase 2	P49137	MAPKAPK2	0.174
Astragali Radix（AgR）	MOL000422	kaempferol	Hemoglobin subunit alpha	P69905	HBA1	0.191
Astragali Radix（AgR）	MOL000422	kaempferol	Mu-type opioid receptor	P35372	OPRM1	0.203
Astragali Radix（AgR）	MOL000422	kaempferol	Prostaglandin G/H synthase 1	P23219	PTGS1	0.25
Astragali Radix（AgR）	MOL000422	kaempferol	Nuclear receptor coactivator 1	Q15788	NCOA1	0.265
Astragali Radix（AgR）	MOL000422	kaempferol	Cyclin-A2	P20248	CCNA2	0.342
Astragali Radix（AgR）	MOL000422	kaempferol	Estrogen receptor beta	Q92731	ESR2	0.383
Astragali Radix（AgR）	MOL000422	kaempferol	Prostaglandin G/H synthase 2	P35354	PTGS2	0.573
Astragali Radix（AgR）	MOL000422	kaempferol	Estrogen receptor	P03372	ESR1	1
Astragali Radix（AgR）	MOL000422	kaempferol	Peroxidase C1A	P00433	PRXC1A	Validated
Astragali Radix（AgR）	MOL000422	kaempferol	Tumor necrosis factor	P01375	TNF	Validated
Astragali Radix（AgR）	MOL000422	kaempferol	Estrogen receptor	P03372	ESR1	Validated
Astragali Radix（AgR）	MOL000422	kaempferol	Interstitial collagenase	P03956	MMP1	Validated
Astragali Radix（AgR）	MOL000422	kaempferol	Cytochrome P450 1A1	P04798	CYP1A1	Validated
Astragali Radix（AgR）	MOL000422	kaempferol	Cytochrome P450 1A2	P05177	CYP1A2	Validated
Astragali Radix（AgR）	MOL000422	kaempferol	Nitric oxide synthase, inducible	P35228	NOS2	Validated
Astragali Radix（AgR）	MOL000422	kaempferol	Peroxisome proliferator-activated receptor gamma	P37231	PPARG	Validated
Astragali Radix（AgR）	MOL000422	kaempferol	Transcription factor p65	Q04206	RELA	Validated
Astragali Radix（AgR）	MOL000422	kaempferol	Serine/threonine-protein phosphatase 2B catalytic subunit alpha isoform	Q08209	PPP3CA	Validated
Astragali Radix（AgR）	MOL000422	kaempferol	Cytochrome P450 1B1	Q16678	CYP1B1	Validated
Astragali Radix（AgR）	MOL000422	kaempferol	Estrogen receptor beta	Q92731	ESR2	Validated
Astragali Radix（AgR）	MOL000422	kaempferol	Inhibitor of nuclear factor kappa-B kinase subunit beta	O14920	IKBKB	Validated
Astragali Radix（AgR）	MOL000422	kaempferol	26S proteasome non-ATPase regulatory subunit 3	O43242	PSMD3	Validated
Astragali Radix（AgR）	MOL000422	kaempferol	Nuclear receptor subfamily 1 group I member 2	O75469	NR1I2	Validated

Astragali Radix（AgR）	MOL000422	kaempferol	Activator of 90 kDa heat shock protein ATPase homolog 1	O95433	AHSA1	Validated
Astragali Radix（AgR）	MOL000422	kaempferol	Antileukoproteinase	P03973	SLPI	Validated
Astragali Radix（AgR）	MOL000422	kaempferol	Intercellular adhesion molecule 1	P05362	ICAM1	Validated
Astragali Radix（AgR）	MOL000422	kaempferol	Transcription factor AP-1	P05412	JUN	Validated
Astragali Radix（AgR）	MOL000422	kaempferol	Insulin receptor	P06213	INSR	Validated
Astragali Radix（AgR）	MOL000422	kaempferol	Cell division control protein 2 homolog	P06493	CDC2	Validated
Astragali Radix（AgR）	MOL000422	kaempferol	Cytochrome P450 3A4	P08684	CYP3A4	Validated
Astragali Radix（AgR）	MOL000422	kaempferol	Glutathione S-transferase P	P09211	GSTP1	Validated
Astragali Radix（AgR）	MOL000422	kaempferol	Glutathione S-transferase Mu 1	P09488	GSTM1	Validated
Astragali Radix（AgR）	MOL000422	kaempferol	Heme oxygenase 1	P09601	HMOX1	Validated
Astragali Radix（AgR）	MOL000422	kaempferol	Arachidonate 5-lipoxygenase	P09917	ALOX5	Validated
Astragali Radix（AgR）	MOL000422	kaempferol	Apoptosis regulator Bcl-2	P10415	BCL2	Validated
Astragali Radix（AgR）	MOL000422	kaempferol	Solute carrier family 2, facilitated glucose transporter member 4	P14672	SLC2A4	Validated
Astragali Radix（AgR）	MOL000422	kaempferol	E-selectin	P16581	SELE	Validated
Astragali Radix（AgR）	MOL000422	kaempferol	Vascular cell adhesion protein 1	P19320	VCAM1	Validated
Astragali Radix（AgR）	MOL000422	kaempferol	Glutathione S-transferase Mu 2	P28161	GSTM2	Validated
Astragali Radix（AgR）	MOL000422	kaempferol	RAC-alpha serine/threonine-protein kinase	P31749	AKT1	Validated
Astragali Radix（AgR）	MOL000422	kaempferol	Prostaglandin G/H synthase 2	P35354	PTGS2	Validated
Astragali Radix（AgR）	MOL000422	kaempferol	Aryl hydrocarbon receptor	P35869	AHR	Validated
Astragali Radix（AgR）	MOL000422	kaempferol	Signal transducer and activator of transcription 1-alpha/beta	P42224	STAT1	Validated
Astragali Radix（AgR）	MOL000422	kaempferol	Aldo-keto reductase family 1 member C3	P42330	AKR1C3	Validated
Astragali Radix（AgR）	MOL000422	kaempferol	Caspase-3	P42574	CASP3	Validated
Astragali Radix（AgR）	MOL000422	kaempferol	Mitogen-activated protein kinase 8	P45983	MAPK8	Validated
Astragali Radix（AgR）	MOL000422	kaempferol	Xanthine dehydrogenase/oxidase	P47989	XDH	Validated
Astragali Radix（AgR）	MOL000422	kaempferol	Type I iodothyronine deiodinase	P49895	DIO1	Validated
Astragali Radix（AgR）	MOL000422	kaempferol	Apoptosis regulator BAX	Q07812	BAX	Validated
Astragali Radix（AgR）	MOL000422	kaempferol	Nuclear receptor subfamily 1 group I member 3	Q14994	NR1I3	Validated
Astragali Radix（AgR）	MOL000422	kaempferol	Hyaluronan synthase 2	Q92819	HAS2	Validated
Astragali Radix（AgR）	MOL000433	FA	Prothrombin	P00734	F2	0.08
Astragali Radix（AgR）	MOL000433	FA	Trypsin-1	P07477	PRSS1	0.08
Astragali Radix（AgR）	MOL000433	FA	Sepiapterin reductase	P35270	SPR	0.104
Astragali Radix（AgR）	MOL000433	FA	6-pyruvoyl tetrahydrobiopterin synthase	Q03393	PTS	0.104
Astragali Radix（AgR）	MOL000433	FA	Folate receptor beta	P14207	FOLR2	0.47
Astragali Radix（AgR）	MOL000433	FA	Cyclin-A2	P20248	CCNA2	1
Astragali Radix（AgR）	MOL000438	(3R)-3-(2-hydroxy-3,4-dimethoxyphenyl)chroman-7-ol	Sodium-dependent noradrenaline transporter	P23975	SLC6A2	0.01
Astragali Radix（AgR）	MOL000438	(3R)-3-(2-hydroxy-3,4-dimethoxyphenyl)chroman-7-ol	5-hydroxytryptamine 2A receptor	P28223	HTR2A	0.01
Astragali Radix（AgR）	MOL000438	(3R)-3-(2-hydroxy-3,4-dimethoxyphenyl)chroman-7-ol	Sodium-dependent serotonin transporter	P31645	SLC6A4	0.01
Astragali Radix（AgR）	MOL000438	(3R)-3-(2-hydroxy-3,4-dimethoxyphenyl)chroman-7-ol	Retinoic acid receptor alpha	P10276	RARA	0.011
Astragali Radix（AgR）	MOL000438	(3R)-3-(2-hydroxy-3,4-dimethoxyphenyl)chroman-7-ol	Retinoic acid receptor beta	P10826	RARB	0.011
Astragali Radix（AgR）	MOL000438	(3R)-3-(2-hydroxy-3,4-dimethoxyphenyl)chroman-7-ol	Retinoic acid receptor gamma-1	P13631	RARG	0.011
Astragali Radix（AgR）	MOL000438	(3R)-3-(2-hydroxy-3,4-dimethoxyphenyl)chroman-7-ol	Retinoic acid receptor RXR-beta	P28702	RXRB	0.011
Astragali Radix（AgR）	MOL000438	(3R)-3-(2-hydroxy-3,4-dimethoxyphenyl)chroman-7-ol	Retinoic acid receptor RXR-gamma	P48443	RXRG	0.011
Astragali Radix（AgR）	MOL000438	(3R)-3-(2-hydroxy-3,4-dimethoxyphenyl)chroman-7-ol	2,4-dienoyl-CoA reductase, mitochondrial	Q16698	DECR1	0.011
Astragali Radix（AgR）	MOL000438	(3R)-3-(2-hydroxy-3,4-dimethoxyphenyl)chroman-7-ol	Pepsin A	P00790	REN	0.012
Astragali Radix（AgR）	MOL000438	(3R)-3-(2-hydroxy-3,4-dimethoxyphenyl)chroman-7-ol	Protein S100-B	P04271	S100B	0.012
Astragali Radix（AgR）	MOL000438	(3R)-3-(2-hydroxy-3,4-dimethoxyphenyl)chroman-7-ol	Interleukin-3	P08700	IL3	0.012
Astragali Radix（AgR）	MOL000438	(3R)-3-(2-hydroxy-3,4-dimethoxyphenyl)chroman-7-ol	Protein S100-A1	P23297	S100A1	0.012

Astragali Radix（AgR）	MOL000438	(3R)-3-(2-hydroxy-3,4-dimethoxyphenyl)chroman-7-ol	Dipeptidyl peptidase 4	P27487	DPP4	0.012
Astragali Radix（AgR）	MOL000438	(3R)-3-(2-hydroxy-3,4-dimethoxyphenyl)chroman-7-ol	Histamine H1 receptor	P35367	HRH1	0.012
Astragali Radix（AgR）	MOL000438	(3R)-3-(2-hydroxy-3,4-dimethoxyphenyl)chroman-7-ol	Glutamate [NMDA] receptor subunit zeta-1	Q05586	GRIN1	0.012
Astragali Radix（AgR）	MOL000438	(3R)-3-(2-hydroxy-3,4-dimethoxyphenyl)chroman-7-ol	Glutamate [NMDA] receptor subunit epsilon-1	Q12879	GRIN2A	0.012
Astragali Radix（AgR）	MOL000438	(3R)-3-(2-hydroxy-3,4-dimethoxyphenyl)chroman-7-ol	Glutamate [NMDA] receptor subunit epsilon-2	Q13224	GRIN2B	0.012
Astragali Radix（AgR）	MOL000438	(3R)-3-(2-hydroxy-3,4-dimethoxyphenyl)chroman-7-ol	Glutamate [NMDA] receptor subunit epsilon-3	Q14957	GRIN2C	0.012
Astragali Radix（AgR）	MOL000438	(3R)-3-(2-hydroxy-3,4-dimethoxyphenyl)chroman-7-ol	Tripartite motif-containing protein 13	O60858	TRIM13	0.013
Astragali Radix（AgR）	MOL000438	(3R)-3-(2-hydroxy-3,4-dimethoxyphenyl)chroman-7-ol	Neuronal acetylcholine receptor subunit alpha-3	P32297	CHRNA3	0.013
Astragali Radix（AgR）	MOL000438	(3R)-3-(2-hydroxy-3,4-dimethoxyphenyl)chroman-7-ol	Neuronal acetylcholine receptor subunit alpha-7	P36544	CHRNA7	0.013
Astragali Radix（AgR）	MOL000438	(3R)-3-(2-hydroxy-3,4-dimethoxyphenyl)chroman-7-ol	Nociceptin receptor	P41146	OPRL1	0.013
Astragali Radix（AgR）	MOL000438	(3R)-3-(2-hydroxy-3,4-dimethoxyphenyl)chroman-7-ol	Neuronal acetylcholine receptor subunit alpha-2	Q15822	CHRNA2	0.015
Astragali Radix（AgR）	MOL000438	(3R)-3-(2-hydroxy-3,4-dimethoxyphenyl)chroman-7-ol	Phenylalanine-4-hydroxylase	P00439	PAH	0.018
Astragali Radix（AgR）	MOL000438	(3R)-3-(2-hydroxy-3,4-dimethoxyphenyl)chroman-7-ol	D(4) dopamine receptor	P21917	DRD4	0.018
Astragali Radix（AgR）	MOL000438	(3R)-3-(2-hydroxy-3,4-dimethoxyphenyl)chroman-7-ol	5-hydroxytryptamine 1D receptor	P28221	HTR1D	0.018
Astragali Radix（AgR）	MOL000438	(3R)-3-(2-hydroxy-3,4-dimethoxyphenyl)chroman-7-ol	5-hydroxytryptamine 1B receptor	P28222	HTR1B	0.018
Astragali Radix（AgR）	MOL000438	(3R)-3-(2-hydroxy-3,4-dimethoxyphenyl)chroman-7-ol	Dihydrofolate reductase	P00374	DHFR	0.02
Astragali Radix（AgR）	MOL000438	(3R)-3-(2-hydroxy-3,4-dimethoxyphenyl)chroman-7-ol	T-cell receptor alpha chain C region	P01848	TRAC	0.02
Astragali Radix（AgR）	MOL000438	(3R)-3-(2-hydroxy-3,4-dimethoxyphenyl)chroman-7-ol	T-cell receptor beta chain C region	P01850	TRBC1	0.02
Astragali Radix（AgR）	MOL000438	(3R)-3-(2-hydroxy-3,4-dimethoxyphenyl)chroman-7-ol	Dihydrolipoyllysine-residue acetyltransferase component of pyruvate dehydrogenase complex, mitochondrial	P10515	DLAT	0.02
Astragali Radix（AgR）	MOL000438	(3R)-3-(2-hydroxy-3,4-dimethoxyphenyl)chroman-7-ol	Glycine cleavage system H protein, mitochondrial	P23434	GCSH	0.02
Astragali Radix（AgR）	MOL000438	(3R)-3-(2-hydroxy-3,4-dimethoxyphenyl)chroman-7-ol	Beta-2-microglobulin	P61769	B2M	0.02
Astragali Radix（AgR）	MOL000438	(3R)-3-(2-hydroxy-3,4-dimethoxyphenyl)chroman-7-ol	cGMP-specific 3',5'-cyclic phosphodiesterase	O76074	PDE5A	0.021
Astragali Radix（AgR）	MOL000438	(3R)-3-(2-hydroxy-3,4-dimethoxyphenyl)chroman-7-ol	Serine/threonine-protein kinase 17B	O94768	STK17B	0.021
Astragali Radix（AgR）	MOL000438	(3R)-3-(2-hydroxy-3,4-dimethoxyphenyl)chroman-7-ol	Adenosine A2a receptor	P29274	ADORA2A	0.021
Astragali Radix（AgR）	MOL000438	(3R)-3-(2-hydroxy-3,4-dimethoxyphenyl)chroman-7-ol	Adenosine A1 receptor	P30542	ADORA1	0.021
Astragali Radix（AgR）	MOL000438	(3R)-3-(2-hydroxy-3,4-dimethoxyphenyl)chroman-7-ol	ATP synthase subunit gamma, mitochondrial	P36542	ATP5F1C	0.021
Astragali Radix（AgR）	MOL000438	(3R)-3-(2-hydroxy-3,4-dimethoxyphenyl)chroman-7-ol	Glycogen synthase kinase-3 beta	P49841	GSK3B	0.021
Astragali Radix（AgR）	MOL000438	(3R)-3-(2-hydroxy-3,4-dimethoxyphenyl)chroman-7-ol	cAMP-specific 3',5'-cyclic phosphodiesterase 4B	Q07343	PDE4B	0.021
Astragali Radix（AgR）	MOL000438	(3R)-3-(2-hydroxy-3,4-dimethoxyphenyl)chroman-7-ol	cGMP-inhibited 3',5'-cyclic phosphodiesterase A	Q14432	PDE3A	0.021
Astragali Radix（AgR）	MOL000438	(3R)-3-(2-hydroxy-3,4-dimethoxyphenyl)chroman-7-ol	Cyclin-dependent kinase 5 activator 1	Q15078	CDK5R1	0.021
Astragali Radix（AgR）	MOL000438	(3R)-3-(2-hydroxy-3,4-dimethoxyphenyl)chroman-7-ol	UDP-glucuronosyltransferase 3A1	Q6NUS8	UGT3A1	0.021
Astragali Radix（AgR）	MOL000438	(3R)-3-(2-hydroxy-3,4-dimethoxyphenyl)chroman-7-ol	cAMP and cAMP-inhibited cGMP 3',5'-cyclic phosphodiesterase 10A	Q9Y233	PDE10A	0.021
Astragali Radix（AgR）	MOL000438	(3R)-3-(2-hydroxy-3,4-dimethoxyphenyl)chroman-7-ol	Vascular endothelial growth factor	A2A2V4	VEGFA	0.022
Astragali Radix（AgR）	MOL000438	(3R)-3-(2-hydroxy-3,4-dimethoxyphenyl)chroman-7-ol	Tumor necrosis factor ligand superfamily member 11	O14788	TNFSF11	0.022
Astragali Radix（AgR）	MOL000438	(3R)-3-(2-hydroxy-3,4-dimethoxyphenyl)chroman-7-ol	Cadherin-5	P33151	CDH5	0.022
Astragali Radix（AgR）	MOL000438	(3R)-3-(2-hydroxy-3,4-dimethoxyphenyl)chroman-7-ol	Amiloride-sensitive sodium channel subunit alpha	P37088	SCNN1A	0.024
Astragali Radix（AgR）	MOL000438	(3R)-3-(2-hydroxy-3,4-dimethoxyphenyl)chroman-7-ol	Amiloride-sensitive sodium channel subunit beta	P51168	SCNN1B	0.024
Astragali Radix（AgR）	MOL000438	(3R)-3-(2-hydroxy-3,4-dimethoxyphenyl)chroman-7-ol	Amiloride-sensitive sodium channel subunit gamma	P51170	SCNN1G	0.024
Astragali Radix（AgR）	MOL000438	(3R)-3-(2-hydroxy-3,4-dimethoxyphenyl)chroman-7-ol	Bile salt sulfotransferase	Q06520	SULT2A1	0.024
Astragali Radix（AgR）	MOL000438	(3R)-3-(2-hydroxy-3,4-dimethoxyphenyl)chroman-7-ol	Muscarinic acetylcholine receptor M4	P08173	CHRM4	0.026
Astragali Radix（AgR）	MOL000438	(3R)-3-(2-hydroxy-3,4-dimethoxyphenyl)chroman-7-ol	Gamma-aminobutyric-acid receptor subunit alpha-3	P34903	GABRA3	0.027
Astragali Radix（AgR）	MOL000438	(3R)-3-(2-hydroxy-3,4-dimethoxyphenyl)chroman-7-ol	Potassium channel subfamily K member 6	Q9Y257	KCNK6	0.027
Astragali Radix（AgR）	MOL000438	(3R)-3-(2-hydroxy-3,4-dimethoxyphenyl)chroman-7-ol	Beta-2 adrenergic receptor	P07550	ADRB2	0.029
Astragali Radix（AgR）	MOL000438	(3R)-3-(2-hydroxy-3,4-dimethoxyphenyl)chroman-7-ol	D(2) dopamine receptor	P14416	DRD2	0.029
Astragali Radix（AgR）	MOL000438	(3R)-3-(2-hydroxy-3,4-dimethoxyphenyl)chroman-7-ol	D(3) dopamine receptor	P35462	DRD3	0.029
Astragali Radix（AgR）	MOL000438	(3R)-3-(2-hydroxy-3,4-dimethoxyphenyl)chroman-7-ol	Protein S100-A12	P80511	S100A12	0.029

Astragali Radix（AgR）	MOL000438	(3R)-3-(2-hydroxy-3,4-dimethoxyphenyl)chroman-7-ol	Protein S100-A13	Q99584	S100A13	0.029
Astragali Radix（AgR）	MOL000438	(3R)-3-(2-hydroxy-3,4-dimethoxyphenyl)chroman-7-ol	Phospholipase A2	P04054	PLA2G1B	0.03
Astragali Radix（AgR）	MOL000438	(3R)-3-(2-hydroxy-3,4-dimethoxyphenyl)chroman-7-ol	Phospholipase A2, membrane associated	P14555	PLA2G2A	0.03
Astragali Radix（AgR）	MOL000438	(3R)-3-(2-hydroxy-3,4-dimethoxyphenyl)chroman-7-ol	Kappa-type opioid receptor	P41145	OPRK1	0.03
Astragali Radix（AgR）	MOL000438	(3R)-3-(2-hydroxy-3,4-dimethoxyphenyl)chroman-7-ol	Methionine aminopeptidase 1	P53582	METAP1	0.03
Astragali Radix（AgR）	MOL000438	(3R)-3-(2-hydroxy-3,4-dimethoxyphenyl)chroman-7-ol	Tubulin beta-2C chain	P68371	TUBB4B	0.03
Astragali Radix（AgR）	MOL000438	(3R)-3-(2-hydroxy-3,4-dimethoxyphenyl)chroman-7-ol	Peroxisome proliferator-activated receptor delta	Q03181	PPARD	0.03
Astragali Radix（AgR）	MOL000438	(3R)-3-(2-hydroxy-3,4-dimethoxyphenyl)chroman-7-ol	Peroxisome proliferator-activated receptor alpha	Q07869	PPARA	0.03
Astragali Radix（AgR）	MOL000438	(3R)-3-(2-hydroxy-3,4-dimethoxyphenyl)chroman-7-ol	Tubulin alpha-3 chain	Q71U36	TUBA1A	0.03
Astragali Radix（AgR）	MOL000438	(3R)-3-(2-hydroxy-3,4-dimethoxyphenyl)chroman-7-ol	Mu-type opioid receptor	P35372	OPRM1	0.031
Astragali Radix（AgR）	MOL000438	(3R)-3-(2-hydroxy-3,4-dimethoxyphenyl)chroman-7-ol	Delta-type opioid receptor	P41143	OPRD1	0.031
Astragali Radix（AgR）	MOL000438	(3R)-3-(2-hydroxy-3,4-dimethoxyphenyl)chroman-7-ol	Mitogen-activated protein kinase 8	P45983	MAPK8	0.032
Astragali Radix（AgR）	MOL000438	(3R)-3-(2-hydroxy-3,4-dimethoxyphenyl)chroman-7-ol	Mitogen-activated protein kinase 10	P53779	MAPK10	0.032
Astragali Radix（AgR）	MOL000438	(3R)-3-(2-hydroxy-3,4-dimethoxyphenyl)chroman-7-ol	Tryptophanyl-tRNA synthetase, mitochondrial	Q9UGM6	WARS2	0.032
Astragali Radix（AgR）	MOL000438	(3R)-3-(2-hydroxy-3,4-dimethoxyphenyl)chroman-7-ol	Ig kappa chain C region	P01834	IGKC	0.033
Astragali Radix（AgR）	MOL000438	(3R)-3-(2-hydroxy-3,4-dimethoxyphenyl)chroman-7-ol	Ig gamma-1 chain C region	P01857	IGHG1	0.033
Astragali Radix（AgR）	MOL000438	(3R)-3-(2-hydroxy-3,4-dimethoxyphenyl)chroman-7-ol	Proto-oncogene tyrosine-protein kinase LCK	P06239	LCK	0.033
Astragali Radix（AgR）	MOL000438	(3R)-3-(2-hydroxy-3,4-dimethoxyphenyl)chroman-7-ol	Tyrosine-protein kinase Lyn	P07948	LYN	0.033
Astragali Radix（AgR）	MOL000438	(3R)-3-(2-hydroxy-3,4-dimethoxyphenyl)chroman-7-ol	Sterol O-acyltransferase 2	O75908	SOAT2	0.034
Astragali Radix（AgR）	MOL000438	(3R)-3-(2-hydroxy-3,4-dimethoxyphenyl)chroman-7-ol	Peptidyl-prolyl cis-trans isomerase, mitochondrial	P30405	PPIF	0.034
Astragali Radix（AgR）	MOL000438	(3R)-3-(2-hydroxy-3,4-dimethoxyphenyl)chroman-7-ol	Sterol O-acyltransferase 1	P35610	SOAT1	0.034
Astragali Radix（AgR）	MOL000438	(3R)-3-(2-hydroxy-3,4-dimethoxyphenyl)chroman-7-ol	Tyrosyl-tRNA synthetase, cytoplasmic	P54577	YARS	0.034
Astragali Radix（AgR）	MOL000438	(3R)-3-(2-hydroxy-3,4-dimethoxyphenyl)chroman-7-ol	Peptidyl-prolyl cis-trans isomerase NIMA-interacting 1	Q13526	PIN1	0.034
Astragali Radix（AgR）	MOL000438	(3R)-3-(2-hydroxy-3,4-dimethoxyphenyl)chroman-7-ol	Myeloperoxidase	P05164	MPO	0.035
Astragali Radix（AgR）	MOL000438	(3R)-3-(2-hydroxy-3,4-dimethoxyphenyl)chroman-7-ol	Eosinophil peroxidase	P11678	EPX	0.035
Astragali Radix（AgR）	MOL000438	(3R)-3-(2-hydroxy-3,4-dimethoxyphenyl)chroman-7-ol	Calreticulin	P27797	CALR	0.035
Astragali Radix（AgR）	MOL000438	(3R)-3-(2-hydroxy-3,4-dimethoxyphenyl)chroman-7-ol	Melatonin receptor type 1A	P48039	MTNR1A	0.035
Astragali Radix（AgR）	MOL000438	(3R)-3-(2-hydroxy-3,4-dimethoxyphenyl)chroman-7-ol	Melatonin receptor type 1B	P49286	MTNR1B	0.035
Astragali Radix（AgR）	MOL000438	(3R)-3-(2-hydroxy-3,4-dimethoxyphenyl)chroman-7-ol	Casein kinase II subunit alpha	P68400	CSNK2A1	0.035
Astragali Radix（AgR）	MOL000438	(3R)-3-(2-hydroxy-3,4-dimethoxyphenyl)chroman-7-ol	Nuclear receptor ROR-beta	Q92753	RORB	0.035
Astragali Radix（AgR）	MOL000438	(3R)-3-(2-hydroxy-3,4-dimethoxyphenyl)chroman-7-ol	Urokinase-type plasminogen activator	P00749	PLAU	0.037
Astragali Radix（AgR）	MOL000438	(3R)-3-(2-hydroxy-3,4-dimethoxyphenyl)chroman-7-ol	Peroxiredoxin-5, mitochondrial	P30044	PRDX5	0.037
Astragali Radix（AgR）	MOL000438	(3R)-3-(2-hydroxy-3,4-dimethoxyphenyl)chroman-7-ol	Beta-1 adrenergic receptor	P08588	ADRB1	0.04
Astragali Radix（AgR）	MOL000438	(3R)-3-(2-hydroxy-3,4-dimethoxyphenyl)chroman-7-ol	Alpha-2A adrenergic receptor	P08913	ADRA2A	0.04
Astragali Radix（AgR）	MOL000438	(3R)-3-(2-hydroxy-3,4-dimethoxyphenyl)chroman-7-ol	Alpha-2C adrenergic receptor	P18825	ADRA2C	0.04
Astragali Radix（AgR）	MOL000438	(3R)-3-(2-hydroxy-3,4-dimethoxyphenyl)chroman-7-ol	D(1A) dopamine receptor	P21728	DRD1	0.04
Astragali Radix（AgR）	MOL000438	(3R)-3-(2-hydroxy-3,4-dimethoxyphenyl)chroman-7-ol	D(1B) dopamine receptor	P21918	DRD5	0.04
Astragali Radix（AgR）	MOL000438	(3R)-3-(2-hydroxy-3,4-dimethoxyphenyl)chroman-7-ol	Alpha-1D adrenergic receptor	P25100	ADRA1D	0.04
Astragali Radix（AgR）	MOL000438	(3R)-3-(2-hydroxy-3,4-dimethoxyphenyl)chroman-7-ol	Alpha-1A adrenergic receptor	P35348	ADRA1A	0.04
Astragali Radix（AgR）	MOL000438	(3R)-3-(2-hydroxy-3,4-dimethoxyphenyl)chroman-7-ol	Alpha-1B adrenergic receptor	P35368	ADRA1B	0.04
Astragali Radix（AgR）	MOL000438	(3R)-3-(2-hydroxy-3,4-dimethoxyphenyl)chroman-7-ol	S-phase kinase-associated protein 1	P63208	SKP1	0.041
Astragali Radix（AgR）	MOL000438	(3R)-3-(2-hydroxy-3,4-dimethoxyphenyl)chroman-7-ol	Muscarinic acetylcholine receptor M2	P08172	CHRM2	0.042
Astragali Radix（AgR）	MOL000438	(3R)-3-(2-hydroxy-3,4-dimethoxyphenyl)chroman-7-ol	Muscarinic acetylcholine receptor M1	P11229	CHRM1	0.042
Astragali Radix（AgR）	MOL000438	(3R)-3-(2-hydroxy-3,4-dimethoxyphenyl)chroman-7-ol	Muscarinic acetylcholine receptor M3	P20309	CHRM3	0.042
Astragali Radix（AgR）	MOL000438	(3R)-3-(2-hydroxy-3,4-dimethoxyphenyl)chroman-7-ol	Mineralocorticoid receptor	P08235	NR3C2	0.043
Astragali Radix（AgR）	MOL000438	(3R)-3-(2-hydroxy-3,4-dimethoxyphenyl)chroman-7-ol	Androgen receptor	P10275	AR	0.043
Astragali Radix（AgR）	MOL000438	(3R)-3-(2-hydroxy-3,4-dimethoxyphenyl)chroman-7-ol	3-oxo-5-alpha-steroid 4-dehydrogenase 1	P18405	SRD5A1	0.043

Astragali Radix（AgR）	MOL000438	(3R)-3-(2-hydroxy-3,4-dimethoxyphenyl)chroman-7-ol	Gonadotropin-releasing hormone receptor	P30968	GNRHR	0.043
Astragali Radix（AgR）	MOL000438	(3R)-3-(2-hydroxy-3,4-dimethoxyphenyl)chroman-7-ol	Estrogen-related receptor gamma	P62508	ESRRG	0.043
Astragali Radix（AgR）	MOL000438	(3R)-3-(2-hydroxy-3,4-dimethoxyphenyl)chroman-7-ol	Gonadotropin-releasing hormone II receptor	Q96P88	GNRHR2	0.043
Astragali Radix（AgR）	MOL000438	(3R)-3-(2-hydroxy-3,4-dimethoxyphenyl)chroman-7-ol	Inhibitor of nuclear factor kappa-B kinase subunit alpha	O15111	CHUK	0.045
Astragali Radix（AgR）	MOL000438	(3R)-3-(2-hydroxy-3,4-dimethoxyphenyl)chroman-7-ol	Arachidonate 5-lipoxygenase	P09917	ALOX5	0.045
Astragali Radix（AgR）	MOL000438	(3R)-3-(2-hydroxy-3,4-dimethoxyphenyl)chroman-7-ol	Protein tyrosine kinase 2 beta	Q14289	PTK2B	0.045
Astragali Radix（AgR）	MOL000438	(3R)-3-(2-hydroxy-3,4-dimethoxyphenyl)chroman-7-ol	ATP synthase subunit beta, mitochondrial	P06576	ATP5F1B	0.046
Astragali Radix（AgR）	MOL000438	(3R)-3-(2-hydroxy-3,4-dimethoxyphenyl)chroman-7-ol	Tyrosine-protein kinase HCK	P08631	HCK	0.046
Astragali Radix（AgR）	MOL000438	(3R)-3-(2-hydroxy-3,4-dimethoxyphenyl)chroman-7-ol	Proto-oncogene serine/threonine-protein kinase Pim-1	P11309	PIM1	0.046
Astragali Radix（AgR）	MOL000438	(3R)-3-(2-hydroxy-3,4-dimethoxyphenyl)chroman-7-ol	ATP synthase subunit alpha, mitochondrial	P25705	ATP5F1A	0.046
Astragali Radix（AgR）	MOL000438	(3R)-3-(2-hydroxy-3,4-dimethoxyphenyl)chroman-7-ol	Phosphatidylinositol-4,5-bisphosphate 3-kinase catalytic subunit gamma isoform	P48736	PIK3CG	0.046
Astragali Radix（AgR）	MOL000438	(3R)-3-(2-hydroxy-3,4-dimethoxyphenyl)chroman-7-ol	Retinoic acid receptor RXR-alpha	P19793	RXRA	0.047
Astragali Radix（AgR）	MOL000438	(3R)-3-(2-hydroxy-3,4-dimethoxyphenyl)chroman-7-ol	Nuclear receptor subfamily 1 group I member 3	Q14994	NR1I3	0.047
Astragali Radix（AgR）	MOL000438	(3R)-3-(2-hydroxy-3,4-dimethoxyphenyl)chroman-7-ol	Platelet glycoprotein IX	P14770	GP9	0.05
Astragali Radix（AgR）	MOL000438	(3R)-3-(2-hydroxy-3,4-dimethoxyphenyl)chroman-7-ol	Alpha-2B adrenergic receptor	P18089	ADRA2B	0.051
Astragali Radix（AgR）	MOL000438	(3R)-3-(2-hydroxy-3,4-dimethoxyphenyl)chroman-7-ol	Estradiol 17-beta-dehydrogenase 1	P14061	HSD17B1	0.054
Astragali Radix（AgR）	MOL000438	(3R)-3-(2-hydroxy-3,4-dimethoxyphenyl)chroman-7-ol	Potassium channel subfamily K member 1	O00180	KCNK1	0.059
Astragali Radix（AgR）	MOL000438	(3R)-3-(2-hydroxy-3,4-dimethoxyphenyl)chroman-7-ol	Gamma-aminobutyric-acid receptor subunit alpha-2	P47869	GABRA2	0.059
Astragali Radix（AgR）	MOL000438	(3R)-3-(2-hydroxy-3,4-dimethoxyphenyl)chroman-7-ol	Tubulin beta-1 chain	Q9H4B7	TUBB1	0.063
Astragali Radix（AgR）	MOL000438	(3R)-3-(2-hydroxy-3,4-dimethoxyphenyl)chroman-7-ol	Cyclin-A2	P20248	CCNA2	0.064
Astragali Radix（AgR）	MOL000438	(3R)-3-(2-hydroxy-3,4-dimethoxyphenyl)chroman-7-ol	Nitric oxide synthase, inducible	P35228	NOS2	0.064
Astragali Radix（AgR）	MOL000438	(3R)-3-(2-hydroxy-3,4-dimethoxyphenyl)chroman-7-ol	Ig heavy chain V-I region ND	P01744	IGKC	0.065
Astragali Radix（AgR）	MOL000438	(3R)-3-(2-hydroxy-3,4-dimethoxyphenyl)chroman-7-ol	Carbonic anhydrase 1	P00915	CA1	0.066
Astragali Radix（AgR）	MOL000438	(3R)-3-(2-hydroxy-3,4-dimethoxyphenyl)chroman-7-ol	Neuropeptide Y	P01303	NPY	0.066
Astragali Radix（AgR）	MOL000438	(3R)-3-(2-hydroxy-3,4-dimethoxyphenyl)chroman-7-ol	Epidermal growth factor receptor	P00533	EGFR	0.067
Astragali Radix（AgR）	MOL000438	(3R)-3-(2-hydroxy-3,4-dimethoxyphenyl)chroman-7-ol	Trypsin-2	P07478	PRSS2	0.067
Astragali Radix（AgR）	MOL000438	(3R)-3-(2-hydroxy-3,4-dimethoxyphenyl)chroman-7-ol	Proto-oncogene tyrosine-protein kinase Src	P12931	SRC	0.067
Astragali Radix（AgR）	MOL000438	(3R)-3-(2-hydroxy-3,4-dimethoxyphenyl)chroman-7-ol	Cannabinoid receptor 1	P21554	CNR1	0.067
Astragali Radix（AgR）	MOL000438	(3R)-3-(2-hydroxy-3,4-dimethoxyphenyl)chroman-7-ol	Endothelin-1 receptor	P25101	EDNRA	0.067
Astragali Radix（AgR）	MOL000438	(3R)-3-(2-hydroxy-3,4-dimethoxyphenyl)chroman-7-ol	Casein kinase I isoform gamma-1	Q9HCP0	CSNK1G1	0.067
Astragali Radix（AgR）	MOL000438	(3R)-3-(2-hydroxy-3,4-dimethoxyphenyl)chroman-7-ol	Triosephosphate isomerase	P60174	TPI1	0.068
Astragali Radix（AgR）	MOL000438	(3R)-3-(2-hydroxy-3,4-dimethoxyphenyl)chroman-7-ol	ATP-binding cassette transporter sub-family C member 8	Q09428	ABCC8	0.068
Astragali Radix（AgR）	MOL000438	(3R)-3-(2-hydroxy-3,4-dimethoxyphenyl)chroman-7-ol	Gamma-aminobutyric acid type B receptor, subunit 1	Q9UBS5	GABBR1	0.068
Astragali Radix（AgR）	MOL000438	(3R)-3-(2-hydroxy-3,4-dimethoxyphenyl)chroman-7-ol	C-jun-amino-terminal kinase-interacting protein 1	Q9UQF2	MAPK8IP1	0.068
Astragali Radix（AgR）	MOL000438	(3R)-3-(2-hydroxy-3,4-dimethoxyphenyl)chroman-7-ol	RAC-alpha serine/threonine-protein kinase	P31749	AKT1	0.069
Astragali Radix（AgR）	MOL000438	(3R)-3-(2-hydroxy-3,4-dimethoxyphenyl)chroman-7-ol	Activin receptor type-1	Q04771	ACVR1	0.069
Astragali Radix（AgR）	MOL000438	(3R)-3-(2-hydroxy-3,4-dimethoxyphenyl)chroman-7-ol	Tyrosine-protein phosphatase non-receptor type 1	P18031	PTPN1	0.07
Astragali Radix（AgR）	MOL000438	(3R)-3-(2-hydroxy-3,4-dimethoxyphenyl)chroman-7-ol	Neutrophil gelatinase-associated lipocalin	P80188	LCN2	0.07
Astragali Radix（AgR）	MOL000438	(3R)-3-(2-hydroxy-3,4-dimethoxyphenyl)chroman-7-ol	5'-AMP-activated protein kinase catalytic subunit alpha-1	Q13131	PRKAA1	0.071
Astragali Radix（AgR）	MOL000438	(3R)-3-(2-hydroxy-3,4-dimethoxyphenyl)chroman-7-ol	Carbonic anhydrase 2	P00918	CA2	0.072
Astragali Radix（AgR）	MOL000438	(3R)-3-(2-hydroxy-3,4-dimethoxyphenyl)chroman-7-ol	Cannabinoid receptor 2	P34972	CNR2	0.072
Astragali Radix（AgR）	MOL000438	(3R)-3-(2-hydroxy-3,4-dimethoxyphenyl)chroman-7-ol	D-HSCDK2	O75100	CA11	0.073
Astragali Radix（AgR）	MOL000438	(3R)-3-(2-hydroxy-3,4-dimethoxyphenyl)chroman-7-ol	Cell division protein kinase 2	P24941	CDK2	0.073
Astragali Radix（AgR）	MOL000438	(3R)-3-(2-hydroxy-3,4-dimethoxyphenyl)chroman-7-ol	cAMP-specific 3',5'-cyclic phosphodiesterase 4A	P27815	PDE4A	0.073
Astragali Radix（AgR）	MOL000438	(3R)-3-(2-hydroxy-3,4-dimethoxyphenyl)chroman-7-ol	Nuclear receptor coactivator 5	Q9HCD5	NCOA5	0.073
Astragali Radix（AgR）	MOL000438	(3R)-3-(2-hydroxy-3,4-dimethoxyphenyl)chroman-7-ol	Fibroblast growth factor receptor 2	P21802	FGFR2	0.074
Astragali Radix（AgR）	MOL000438	(3R)-3-(2-hydroxy-3,4-dimethoxyphenyl)chroman-7-ol	ATP-sensitive inward rectifier potassium channel 1	P48048	KCNJ1	0.074

Astragali Radix（AgR）	MOL000438	(3R)-3-(2-hydroxy-3,4-dimethoxyphenyl)chroman-7-ol	Calmodulin	P62158		0.074
Astragali Radix（AgR）	MOL000438	(3R)-3-(2-hydroxy-3,4-dimethoxyphenyl)chroman-7-ol	Ribosyldihydronicotinamide dehydrogenase [quinone]	P16083	NQO2	0.075
Astragali Radix（AgR）	MOL000438	(3R)-3-(2-hydroxy-3,4-dimethoxyphenyl)chroman-7-ol	MAP kinase-activated protein kinase 2	P49137	MAPKAPK2	0.075
Astragali Radix（AgR）	MOL000438	(3R)-3-(2-hydroxy-3,4-dimethoxyphenyl)chroman-7-ol	Interferon gamma	P01579	IFNG	0.081
Astragali Radix（AgR）	MOL000438	(3R)-3-(2-hydroxy-3,4-dimethoxyphenyl)chroman-7-ol	Hemoglobin subunit alpha	P69905	HBA1	0.083
Astragali Radix（AgR）	MOL000438	(3R)-3-(2-hydroxy-3,4-dimethoxyphenyl)chroman-7-ol	DNA topoisomerase 2-alpha	P11388	TOP2A	0.087
Astragali Radix（AgR）	MOL000438	(3R)-3-(2-hydroxy-3,4-dimethoxyphenyl)chroman-7-ol	Mitogen-activated protein kinase 14	Q16539	MAPK14	0.087
Astragali Radix（AgR）	MOL000438	(3R)-3-(2-hydroxy-3,4-dimethoxyphenyl)chroman-7-ol	Gamma-aminobutyric-acid receptor subunit alpha-1	P14867	GABRA1	0.091
Astragali Radix（AgR）	MOL000438	(3R)-3-(2-hydroxy-3,4-dimethoxyphenyl)chroman-7-ol	Sodium channel protein type 5 subunit alpha	Q14524	SCN5A	0.091
Astragali Radix（AgR）	MOL000438	(3R)-3-(2-hydroxy-3,4-dimethoxyphenyl)chroman-7-ol	Group IIE secretory phospholipase A2	Q9NZK7	PLA2G2E	0.091
Astragali Radix（AgR）	MOL000438	(3R)-3-(2-hydroxy-3,4-dimethoxyphenyl)chroman-7-ol	Cell division control protein 2 homolog	P06493	CDK1	0.099
Astragali Radix（AgR）	MOL000438	(3R)-3-(2-hydroxy-3,4-dimethoxyphenyl)chroman-7-ol	Prostaglandin G/H synthase 1	P23219	PTGS1	0.115
Astragali Radix（AgR）	MOL000438	(3R)-3-(2-hydroxy-3,4-dimethoxyphenyl)chroman-7-ol	Cell division protein kinase 5	Q00535	CDK5	0.125
Astragali Radix（AgR）	MOL000438	(3R)-3-(2-hydroxy-3,4-dimethoxyphenyl)chroman-7-ol	Heat shock protein HSP 90-beta	P08238	HSP90AB1	0.139
Astragali Radix（AgR）	MOL000438	(3R)-3-(2-hydroxy-3,4-dimethoxyphenyl)chroman-7-ol	Coagulation factor VII	P08709	F7	0.142
Astragali Radix（AgR）	MOL000438	(3R)-3-(2-hydroxy-3,4-dimethoxyphenyl)chroman-7-ol	Prothrombin	P00734	F2	0.161
Astragali Radix（AgR）	MOL000438	(3R)-3-(2-hydroxy-3,4-dimethoxyphenyl)chroman-7-ol	Trypsin-1	P07477	PRSS1	0.247
Astragali Radix（AgR）	MOL000438	(3R)-3-(2-hydroxy-3,4-dimethoxyphenyl)chroman-7-ol	Progesterone receptor	P06401	PGR	0.33
Astragali Radix（AgR）	MOL000438	(3R)-3-(2-hydroxy-3,4-dimethoxyphenyl)chroman-7-ol	Nuclear receptor coactivator 1	Q15788	NCOA1	0.357
Astragali Radix（AgR）	MOL000438	(3R)-3-(2-hydroxy-3,4-dimethoxyphenyl)chroman-7-ol	Prostaglandin G/H synthase 2	P35354	PTGS2	0.358
Astragali Radix（AgR）	MOL000438	(3R)-3-(2-hydroxy-3,4-dimethoxyphenyl)chroman-7-ol	cAMP-dependent protein kinase catalytic subunit alpha	P17612	PRKACA	0.426
Astragali Radix（AgR）	MOL000438	(3R)-3-(2-hydroxy-3,4-dimethoxyphenyl)chroman-7-ol	Estrogen receptor beta	Q92731	ESR2	0.465
Astragali Radix（AgR）	MOL000438	(3R)-3-(2-hydroxy-3,4-dimethoxyphenyl)chroman-7-ol	Estrogen receptor	P03372	ESR1	1
Astragali Radix（AgR）	MOL000442	1,7-Dihydroxy-3,9-dimethoxy pterocarpene	Elongation factor 2	P13639	EEF2	0.01
Astragali Radix（AgR）	MOL000442	1,7-Dihydroxy-3,9-dimethoxy pterocarpene	Tyrosinase	P14679	TYR	0.01
Astragali Radix（AgR）	MOL000442	1,7-Dihydroxy-3,9-dimethoxy pterocarpene	3-oxo-5-alpha-steroid 4-dehydrogenase 2	P31213	SRD5A2	0.01
Astragali Radix（AgR）	MOL000442	1,7-Dihydroxy-3,9-dimethoxy pterocarpene	3-oxo-5-beta-steroid 4-dehydrogenase	P51857	AKR1D1	0.01
Astragali Radix（AgR）	MOL000442	1,7-Dihydroxy-3,9-dimethoxy pterocarpene	Poly [ADP-ribose] polymerase 3	Q9Y6F1	PARP3	0.01
Astragali Radix（AgR）	MOL000442	1,7-Dihydroxy-3,9-dimethoxy pterocarpene	Acetylcholinesterase	P22303	ACHE	0.011
Astragali Radix（AgR）	MOL000442	1,7-Dihydroxy-3,9-dimethoxy pterocarpene	Neuronal acetylcholine receptor subunit alpha-3	P32297	CHRNA3	0.011
Astragali Radix（AgR）	MOL000442	1,7-Dihydroxy-3,9-dimethoxy pterocarpene	Neuronal acetylcholine receptor subunit alpha-7	P36544	CHRNA7	0.011
Astragali Radix（AgR）	MOL000442	1,7-Dihydroxy-3,9-dimethoxy pterocarpene	Sodium-dependent serotonin transporter	P31645	SLC6A4	0.012
Astragali Radix（AgR）	MOL000442	1,7-Dihydroxy-3,9-dimethoxy pterocarpene	5-hydroxytryptamine 2B receptor	P41595	HTR2B	0.012
Astragali Radix（AgR）	MOL000442	1,7-Dihydroxy-3,9-dimethoxy pterocarpene	Potassium voltage-gated channel subfamily H member 2	Q12809	KCNH2	0.012
Astragali Radix（AgR）	MOL000442	1,7-Dihydroxy-3,9-dimethoxy pterocarpene	Potassium voltage-gated channel subfamily H member 6	Q9H252	KCNH6	0.012
Astragali Radix（AgR）	MOL000442	1,7-Dihydroxy-3,9-dimethoxy pterocarpene	Gamma-aminobutyric-acid receptor subunit beta-3	P28472	GABRB3	0.013
Astragali Radix（AgR）	MOL000442	1,7-Dihydroxy-3,9-dimethoxy pterocarpene	Gamma-aminobutyric-acid receptor subunit beta-2	P47870	GABRB2	0.013
Astragali Radix（AgR）	MOL000442	1,7-Dihydroxy-3,9-dimethoxy pterocarpene	Neuronal acetylcholine receptor subunit alpha-2	Q15822	CHRNA2	0.013
Astragali Radix（AgR）	MOL000442	1,7-Dihydroxy-3,9-dimethoxy pterocarpene	Tripartite motif-containing protein 13	O60858	TRIM13	0.014
Astragali Radix（AgR）	MOL000442	1,7-Dihydroxy-3,9-dimethoxy pterocarpene	Cytochrome b	P00156	MT-CYB	0.014
Astragali Radix（AgR）	MOL000442	1,7-Dihydroxy-3,9-dimethoxy pterocarpene	Cytochrome c1, heme protein, mitochondrial	P08574	CYC1	0.014
Astragali Radix（AgR）	MOL000442	1,7-Dihydroxy-3,9-dimethoxy pterocarpene	Cytochrome b-c1 complex subunit Rieske, mitochondrial	P47985	UQCRFS1	0.014
Astragali Radix（AgR）	MOL000442	1,7-Dihydroxy-3,9-dimethoxy pterocarpene	Bile salt sulfotransferase	Q06520	SULT2A1	0.014
Astragali Radix（AgR）	MOL000442	1,7-Dihydroxy-3,9-dimethoxy pterocarpene	Dehydrogenase/reductase SDR family member 8	Q8NBQ5	HSD17B11	0.014
Astragali Radix（AgR）	MOL000442	1,7-Dihydroxy-3,9-dimethoxy pterocarpene	Pyridoxal kinase	O00764	PDXK	0.015
Astragali Radix（AgR）	MOL000442	1,7-Dihydroxy-3,9-dimethoxy pterocarpene	Interleukin-3	P08700	IL3	0.015
Astragali Radix（AgR）	MOL000442	1,7-Dihydroxy-3,9-dimethoxy pterocarpene	5-hydroxytryptamine 1F receptor	P30939	HTR1F	0.015

Astragali Radix（AgR）	MOL000442	1,7-Dihydroxy-3,9-dimethoxy pterocarpene	Cystathionine beta-synthase	P35520	CBS	0.015
Astragali Radix（AgR）	MOL000442	1,7-Dihydroxy-3,9-dimethoxy pterocarpene	Protein S100-A12	P80511	S100A12	0.015
Astragali Radix（AgR）	MOL000442	1,7-Dihydroxy-3,9-dimethoxy pterocarpene	2,4-dienoyl-CoA reductase, mitochondrial	Q16698	DECR1	0.015
Astragali Radix（AgR）	MOL000442	1,7-Dihydroxy-3,9-dimethoxy pterocarpene	Pyridoxal phosphate phosphatase	Q96GD0	PDXP	0.015
Astragali Radix（AgR）	MOL000442	1,7-Dihydroxy-3,9-dimethoxy pterocarpene	Protein S100-A13	Q99584	S100A13	0.015
Astragali Radix（AgR）	MOL000442	1,7-Dihydroxy-3,9-dimethoxy pterocarpene	Carbonic anhydrase 12	O43570	CA12	0.016
Astragali Radix（AgR）	MOL000442	1,7-Dihydroxy-3,9-dimethoxy pterocarpene	Serine/threonine-protein kinase 17B	O94768	STK17B	0.016
Astragali Radix（AgR）	MOL000442	1,7-Dihydroxy-3,9-dimethoxy pterocarpene	Sodium/potassium-transporting ATPase alpha-1 chain	P05023	ATP1A1	0.016
Astragali Radix（AgR）	MOL000442	1,7-Dihydroxy-3,9-dimethoxy pterocarpene	ATP synthase subunit gamma, mitochondrial	P36542	ATP5F1C	0.016
Astragali Radix（AgR）	MOL000442	1,7-Dihydroxy-3,9-dimethoxy pterocarpene	Solute carrier family 12 member 1	Q13621	SLC12A1	0.016
Astragali Radix（AgR）	MOL000442	1,7-Dihydroxy-3,9-dimethoxy pterocarpene	Carbonic anhydrase 9	Q16790	CA9	0.016
Astragali Radix（AgR）	MOL000442	1,7-Dihydroxy-3,9-dimethoxy pterocarpene	UDP-glucuronosyltransferase 3A1	Q6NUS8	UGT3A1	0.016
Astragali Radix（AgR）	MOL000442	1,7-Dihydroxy-3,9-dimethoxy pterocarpene	T-cell receptor alpha chain C region	P01848	TRAC	0.017
Astragali Radix（AgR）	MOL000442	1,7-Dihydroxy-3,9-dimethoxy pterocarpene	T-cell receptor beta chain C region	P01850	TRBC1	0.017
Astragali Radix（AgR）	MOL000442	1,7-Dihydroxy-3,9-dimethoxy pterocarpene	Dihydrolipoyllysine-residue acetyltransferase component of pyruvate dehydrogenase complex, mitochondrial	P10515	DLAT	0.017
Astragali Radix（AgR）	MOL000442	1,7-Dihydroxy-3,9-dimethoxy pterocarpene	Glycine cleavage system H protein, mitochondrial	P23434	GCSH	0.017
Astragali Radix（AgR）	MOL000442	1,7-Dihydroxy-3,9-dimethoxy pterocarpene	Amiloride-sensitive sodium channel subunit alpha	P37088	SCNN1A	0.017
Astragali Radix（AgR）	MOL000442	1,7-Dihydroxy-3,9-dimethoxy pterocarpene	5-hydroxytryptamine 3 receptor	P46098	HTR3A	0.017
Astragali Radix（AgR）	MOL000442	1,7-Dihydroxy-3,9-dimethoxy pterocarpene	Amiloride-sensitive sodium channel subunit beta	P51168	SCNN1B	0.017
Astragali Radix（AgR）	MOL000442	1,7-Dihydroxy-3,9-dimethoxy pterocarpene	Amiloride-sensitive sodium channel subunit gamma	P51170	SCNN1G	0.017
Astragali Radix（AgR）	MOL000442	1,7-Dihydroxy-3,9-dimethoxy pterocarpene	Beta-2-microglobulin	P61769	B2M	0.017
Astragali Radix（AgR）	MOL000442	1,7-Dihydroxy-3,9-dimethoxy pterocarpene	Retinoic acid receptor alpha	P10276	RARA	0.019
Astragali Radix（AgR）	MOL000442	1,7-Dihydroxy-3,9-dimethoxy pterocarpene	Retinoic acid receptor beta	P10826	RARB	0.019
Astragali Radix（AgR）	MOL000442	1,7-Dihydroxy-3,9-dimethoxy pterocarpene	Retinoic acid receptor gamma-1	P13631	RARG	0.019
Astragali Radix（AgR）	MOL000442	1,7-Dihydroxy-3,9-dimethoxy pterocarpene	Retinoic acid receptor RXR-gamma	P48443	RXRG	0.019
Astragali Radix（AgR）	MOL000442	1,7-Dihydroxy-3,9-dimethoxy pterocarpene	Gamma-aminobutyric acid receptor subunit rho-3	A8MPY1	GABRR3	0.02
Astragali Radix（AgR）	MOL000442	1,7-Dihydroxy-3,9-dimethoxy pterocarpene	Gamma-aminobutyric acid receptor subunit pi	O00591	GABRP	0.02
Astragali Radix（AgR）	MOL000442	1,7-Dihydroxy-3,9-dimethoxy pterocarpene	Gamma-aminobutyric acid receptor subunit delta	O14764	GABRD	0.02
Astragali Radix（AgR）	MOL000442	1,7-Dihydroxy-3,9-dimethoxy pterocarpene	Pepsin A	P00790	REN	0.02
Astragali Radix（AgR）	MOL000442	1,7-Dihydroxy-3,9-dimethoxy pterocarpene	Pyruvate carboxylase, mitochondrial	P11498	PC	0.02
Astragali Radix（AgR）	MOL000442	1,7-Dihydroxy-3,9-dimethoxy pterocarpene	Gamma-aminobutyric-acid receptor subunit beta-1	P18505	GABRB1	0.02
Astragali Radix（AgR）	MOL000442	1,7-Dihydroxy-3,9-dimethoxy pterocarpene	Gamma-aminobutyric acid receptor subunit gamma-2	P18507	GABRG2	0.02
Astragali Radix（AgR）	MOL000442	1,7-Dihydroxy-3,9-dimethoxy pterocarpene	Gamma-aminobutyric-acid receptor subunit rho-1	P24046	GABRR1	0.02
Astragali Radix（AgR）	MOL000442	1,7-Dihydroxy-3,9-dimethoxy pterocarpene	Dipeptidyl peptidase 4	P27487	DPP4	0.02
Astragali Radix（AgR）	MOL000442	1,7-Dihydroxy-3,9-dimethoxy pterocarpene	Gamma-aminobutyric acid receptor subunit rho-2	P28476	GABRR2	0.02
Astragali Radix（AgR）	MOL000442	1,7-Dihydroxy-3,9-dimethoxy pterocarpene	Gamma-aminobutyric-acid receptor subunit alpha-5	P31644	GABRA5	0.02
Astragali Radix（AgR）	MOL000442	1,7-Dihydroxy-3,9-dimethoxy pterocarpene	Gamma-aminobutyric acid receptor subunit epsilon	P78334	GABRE	0.02
Astragali Radix（AgR）	MOL000442	1,7-Dihydroxy-3,9-dimethoxy pterocarpene	Gamma-aminobutyric acid receptor subunit gamma-1	Q8N1C3	GABRG1	0.02
Astragali Radix（AgR）	MOL000442	1,7-Dihydroxy-3,9-dimethoxy pterocarpene	Gamma-aminobutyric acid receptor subunit gamma-3	Q99928	GABRG3	0.02
Astragali Radix（AgR）	MOL000442	1,7-Dihydroxy-3,9-dimethoxy pterocarpene	Tyrosine-protein kinase JAK2	O60674	JAK2	0.021
Astragali Radix（AgR）	MOL000442	1,7-Dihydroxy-3,9-dimethoxy pterocarpene	Tyrosine-protein kinase Lyn	P07948	LYN	0.021
Astragali Radix（AgR）	MOL000442	1,7-Dihydroxy-3,9-dimethoxy pterocarpene	Tyrosine-protein kinase JAK1	P23458	JAK1	0.021
Astragali Radix（AgR）	MOL000442	1,7-Dihydroxy-3,9-dimethoxy pterocarpene	Alpha-1D adrenergic receptor	P25100	ADRA1D	0.021
Astragali Radix（AgR）	MOL000442	1,7-Dihydroxy-3,9-dimethoxy pterocarpene	Tyrosine-protein kinase JAK3	P52333	JAK3	0.021
Astragali Radix（AgR）	MOL000442	1,7-Dihydroxy-3,9-dimethoxy pterocarpene	3-phosphoinositide-dependent protein kinase 1	O15530	PDPK1	0.022
Astragali Radix（AgR）	MOL000442	1,7-Dihydroxy-3,9-dimethoxy pterocarpene	Muscarinic acetylcholine receptor M5	P08912	CHRM5	0.022

Astragali Radix（AgR）	MOL000442	1,7-Dihydroxy-3,9-dimethoxy pterocarpene	Alpha-2A adrenergic receptor	P08913	ADRA2A	0.022
Astragali Radix（AgR）	MOL000442	1,7-Dihydroxy-3,9-dimethoxy pterocarpene	Muscarinic acetylcholine receptor M1	P11229	CHRM1	0.022
Astragali Radix（AgR）	MOL000442	1,7-Dihydroxy-3,9-dimethoxy pterocarpene	Histamine H1 receptor	P35367	HRH1	0.022
Astragali Radix（AgR）	MOL000442	1,7-Dihydroxy-3,9-dimethoxy pterocarpene	D(3) dopamine receptor	P35462	DRD3	0.022
Astragali Radix（AgR）	MOL000442	1,7-Dihydroxy-3,9-dimethoxy pterocarpene	Tyrosine-protein kinase CSK	P41240	CSK	0.022
Astragali Radix（AgR）	MOL000442	1,7-Dihydroxy-3,9-dimethoxy pterocarpene	Tyrosine-protein kinase ZAP-70	P43403	ZAP70	0.022
Astragali Radix（AgR）	MOL000442	1,7-Dihydroxy-3,9-dimethoxy pterocarpene	Tyrosine-protein kinase SYK	P43405	SYK	0.022
Astragali Radix（AgR）	MOL000442	1,7-Dihydroxy-3,9-dimethoxy pterocarpene	Protein kinase C theta type	Q04759	PRKCQ	0.022
Astragali Radix（AgR）	MOL000442	1,7-Dihydroxy-3,9-dimethoxy pterocarpene	Tyrosine-protein kinase ITK/TSK	Q08881	ITK	0.022
Astragali Radix（AgR）	MOL000442	1,7-Dihydroxy-3,9-dimethoxy pterocarpene	Cyclin-dependent kinase 5 activator 1	Q15078	CDK5R1	0.022
Astragali Radix（AgR）	MOL000442	1,7-Dihydroxy-3,9-dimethoxy pterocarpene	Tumor necrosis factor	P01375	TNF	0.024
Astragali Radix（AgR）	MOL000442	1,7-Dihydroxy-3,9-dimethoxy pterocarpene	Nuclear factor NF-kappa-B p105 subunit	P19838	NFKB1	0.024
Astragali Radix（AgR）	MOL000442	1,7-Dihydroxy-3,9-dimethoxy pterocarpene	85 kDa calcium-independent phospholipase A2	O60733	PLA2G6	0.025
Astragali Radix（AgR）	MOL000442	1,7-Dihydroxy-3,9-dimethoxy pterocarpene	Coagulation factor IX	P00740	F9	0.025
Astragali Radix（AgR）	MOL000442	1,7-Dihydroxy-3,9-dimethoxy pterocarpene	Coagulation factor X	P00742	F10	0.025
Astragali Radix（AgR）	MOL000442	1,7-Dihydroxy-3,9-dimethoxy pterocarpene	Osteocalcin	P02818	BGLAP	0.025
Astragali Radix（AgR）	MOL000442	1,7-Dihydroxy-3,9-dimethoxy pterocarpene	Vitamin K-dependent protein C	P04070	PROC	0.025
Astragali Radix（AgR）	MOL000442	1,7-Dihydroxy-3,9-dimethoxy pterocarpene	NAD(P)H dehydrogenase [quinone] 1	P15559	NQO1	0.025
Astragali Radix（AgR）	MOL000442	1,7-Dihydroxy-3,9-dimethoxy pterocarpene	Vitamin K-dependent protein Z	P22891	PROZ	0.025
Astragali Radix（AgR）	MOL000442	1,7-Dihydroxy-3,9-dimethoxy pterocarpene	Vitamin K-dependent gamma-carboxylase	P38435	GGCX	0.025
Astragali Radix（AgR）	MOL000442	1,7-Dihydroxy-3,9-dimethoxy pterocarpene	Cytosolic phospholipase A2	P47712	PLA2G4A	0.025
Astragali Radix（AgR）	MOL000442	1,7-Dihydroxy-3,9-dimethoxy pterocarpene	Vitamin K epoxide reductase complex subunit 1-like protein 1	Q8N0U8	VKORC1L1	0.025
Astragali Radix（AgR）	MOL000442	1,7-Dihydroxy-3,9-dimethoxy pterocarpene	Vitamin K epoxide reductase complex subunit 1	Q9BQB6	VKORC1	0.025
Astragali Radix（AgR）	MOL000442	1,7-Dihydroxy-3,9-dimethoxy pterocarpene	DNA topoisomerase 1	P11387	TOP1	0.026
Astragali Radix（AgR）	MOL000442	1,7-Dihydroxy-3,9-dimethoxy pterocarpene	Methionine aminopeptidase 1	P53582	METAP1	0.026
Astragali Radix（AgR）	MOL000442	1,7-Dihydroxy-3,9-dimethoxy pterocarpene	DNA topoisomerase I, mitochondrial	Q969P6	TOP1MT	0.026
Astragali Radix（AgR）	MOL000442	1,7-Dihydroxy-3,9-dimethoxy pterocarpene	Peptidyl-prolyl cis-trans isomerase, mitochondrial	P30405	PPIF	0.027
Astragali Radix（AgR）	MOL000442	1,7-Dihydroxy-3,9-dimethoxy pterocarpene	Gamma-aminobutyric-acid receptor subunit alpha-3	P34903	GABRA3	0.027
Astragali Radix（AgR）	MOL000442	1,7-Dihydroxy-3,9-dimethoxy pterocarpene	Oxysterols receptor LXR-beta	P55055	NR1H2	0.027
Astragali Radix（AgR）	MOL000442	1,7-Dihydroxy-3,9-dimethoxy pterocarpene	Oxysterols receptor LXR-alpha	Q13133	NR1H3	0.027
Astragali Radix（AgR）	MOL000442	1,7-Dihydroxy-3,9-dimethoxy pterocarpene	Myeloperoxidase	P05164	MPO	0.028
Astragali Radix（AgR）	MOL000442	1,7-Dihydroxy-3,9-dimethoxy pterocarpene	Eosinophil peroxidase	P11678	EPX	0.028
Astragali Radix（AgR）	MOL000442	1,7-Dihydroxy-3,9-dimethoxy pterocarpene	Calreticulin	P27797	CALR	0.028
Astragali Radix（AgR）	MOL000442	1,7-Dihydroxy-3,9-dimethoxy pterocarpene	Sepiapterin reductase	P35270	SPR	0.028
Astragali Radix（AgR）	MOL000442	1,7-Dihydroxy-3,9-dimethoxy pterocarpene	Melatonin receptor type 1B	P49286	MTNR1B	0.028
Astragali Radix（AgR）	MOL000442	1,7-Dihydroxy-3,9-dimethoxy pterocarpene	6-pyruvoyl tetrahydrobiopterin synthase	Q03393	PTS	0.028
Astragali Radix（AgR）	MOL000442	1,7-Dihydroxy-3,9-dimethoxy pterocarpene	Nuclear receptor ROR-beta	Q92753	RORB	0.028
Astragali Radix（AgR）	MOL000442	1,7-Dihydroxy-3,9-dimethoxy pterocarpene	Urokinase-type plasminogen activator	P00749	PLAU	0.029
Astragali Radix（AgR）	MOL000442	1,7-Dihydroxy-3,9-dimethoxy pterocarpene	DNA-(apurinic or apyrimidinic site) lyase	P27695	APEX1	0.029
Astragali Radix（AgR）	MOL000442	1,7-Dihydroxy-3,9-dimethoxy pterocarpene	Peroxiredoxin-5, mitochondrial	P30044	PRDX5	0.029
Astragali Radix（AgR）	MOL000442	1,7-Dihydroxy-3,9-dimethoxy pterocarpene	Sterol O-acyltransferase 2	O75908	SOAT2	0.03
Astragali Radix（AgR）	MOL000442	1,7-Dihydroxy-3,9-dimethoxy pterocarpene	Myosin light chain 6B	P14649	MYL6B	0.03
Astragali Radix（AgR）	MOL000442	1,7-Dihydroxy-3,9-dimethoxy pterocarpene	Myosin regulatory light chain 12A	P19105	MYL12A	0.03
Astragali Radix（AgR）	MOL000442	1,7-Dihydroxy-3,9-dimethoxy pterocarpene	Sterol O-acyltransferase 1	P35610	SOAT1	0.03
Astragali Radix（AgR）	MOL000442	1,7-Dihydroxy-3,9-dimethoxy pterocarpene	Tyrosyl-tRNA synthetase, cytoplasmic	P54577	YARS	0.03
Astragali Radix（AgR）	MOL000442	1,7-Dihydroxy-3,9-dimethoxy pterocarpene	Tubulin beta-2C chain	P68371	TUBB4B	0.03
Astragali Radix（AgR）	MOL000442	1,7-Dihydroxy-3,9-dimethoxy pterocarpene	Peptidyl-prolyl cis-trans isomerase NIMA-interacting 1	Q13526	PIN1	0.03

Astragali Radix（AgR）	MOL000442	1,7-Dihydroxy-3,9-dimethoxy pterocarpene	Tubulin alpha-3 chain	Q71U36	TUBA1A	0.03
Astragali Radix（AgR）	MOL000442	1,7-Dihydroxy-3,9-dimethoxy pterocarpene	Muscarinic acetylcholine receptor M2	P08172	CHRM2	0.031
Astragali Radix（AgR）	MOL000442	1,7-Dihydroxy-3,9-dimethoxy pterocarpene	Muscarinic acetylcholine receptor M4	P08173	CHRM4	0.031
Astragali Radix（AgR）	MOL000442	1,7-Dihydroxy-3,9-dimethoxy pterocarpene	Muscarinic acetylcholine receptor M3	P20309	CHRM3	0.031
Astragali Radix（AgR）	MOL000442	1,7-Dihydroxy-3,9-dimethoxy pterocarpene	D(4) dopamine receptor	P21917	DRD4	0.031
Astragali Radix（AgR）	MOL000442	1,7-Dihydroxy-3,9-dimethoxy pterocarpene	Alpha-2B adrenergic receptor	P18089	ADRA2B	0.032
Astragali Radix（AgR）	MOL000442	1,7-Dihydroxy-3,9-dimethoxy pterocarpene	Alpha-2C adrenergic receptor	P18825	ADRA2C	0.032
Astragali Radix（AgR）	MOL000442	1,7-Dihydroxy-3,9-dimethoxy pterocarpene	D(1B) dopamine receptor	P21918	DRD5	0.032
Astragali Radix（AgR）	MOL000442	1,7-Dihydroxy-3,9-dimethoxy pterocarpene	Mitogen-activated protein kinase 8	P45983	MAPK8	0.032
Astragali Radix（AgR）	MOL000442	1,7-Dihydroxy-3,9-dimethoxy pterocarpene	Mitogen-activated protein kinase 10	P53779	MAPK10	0.032
Astragali Radix（AgR）	MOL000442	1,7-Dihydroxy-3,9-dimethoxy pterocarpene	3 beta-hydroxysteroid dehydrogenase/Delta 5-->4-isomerase type II	P26439	HSD3B2	0.033
Astragali Radix（AgR）	MOL000442	1,7-Dihydroxy-3,9-dimethoxy pterocarpene	Microtubule-associated protein 2	P11137	MAP2	0.034
Astragali Radix（AgR）	MOL000442	1,7-Dihydroxy-3,9-dimethoxy pterocarpene	Gamma-aminobutyric-acid receptor subunit alpha-1	P14867	GABRA1	0.034
Astragali Radix（AgR）	MOL000442	1,7-Dihydroxy-3,9-dimethoxy pterocarpene	Prolactin receptor	P16471	PRLR	0.034
Astragali Radix（AgR）	MOL000442	1,7-Dihydroxy-3,9-dimethoxy pterocarpene	3-oxo-5-alpha-steroid 4-dehydrogenase 1	P18405	SRD5A1	0.034
Astragali Radix（AgR）	MOL000442	1,7-Dihydroxy-3,9-dimethoxy pterocarpene	Gonadotropin-releasing hormone receptor	P30968	GNRHR	0.034
Astragali Radix（AgR）	MOL000442	1,7-Dihydroxy-3,9-dimethoxy pterocarpene	Gamma-aminobutyric-acid receptor subunit alpha-2	P47869	GABRA2	0.034
Astragali Radix（AgR）	MOL000442	1,7-Dihydroxy-3,9-dimethoxy pterocarpene	Estrogen-related receptor gamma	P62508	ESRRG	0.034
Astragali Radix（AgR）	MOL000442	1,7-Dihydroxy-3,9-dimethoxy pterocarpene	Microtubule-associated protein 1A	P78559	MAP1A	0.034
Astragali Radix（AgR）	MOL000442	1,7-Dihydroxy-3,9-dimethoxy pterocarpene	Protein tyrosine kinase 2 beta	Q14289	PTK2B	0.034
Astragali Radix（AgR）	MOL000442	1,7-Dihydroxy-3,9-dimethoxy pterocarpene	Gonadotropin-releasing hormone II receptor	Q96P88	GNRHR2	0.034
Astragali Radix（AgR）	MOL000442	1,7-Dihydroxy-3,9-dimethoxy pterocarpene	5-hydroxytryptamine 1A receptor	P08908	HTR1A	0.036
Astragali Radix（AgR）	MOL000442	1,7-Dihydroxy-3,9-dimethoxy pterocarpene	5-hydroxytryptamine 1D receptor	P28221	HTR1D	0.036
Astragali Radix（AgR）	MOL000442	1,7-Dihydroxy-3,9-dimethoxy pterocarpene	ATP synthase subunit beta, mitochondrial	P06576	ATP5F1B	0.037
Astragali Radix（AgR）	MOL000442	1,7-Dihydroxy-3,9-dimethoxy pterocarpene	Tyrosine-protein kinase HCK	P08631	HCK	0.037
Astragali Radix（AgR）	MOL000442	1,7-Dihydroxy-3,9-dimethoxy pterocarpene	ATP synthase subunit alpha, mitochondrial	P25705	ATP5F1A	0.037
Astragali Radix（AgR）	MOL000442	1,7-Dihydroxy-3,9-dimethoxy pterocarpene	Phosphatidylinositol-4,5-bisphosphate 3-kinase catalytic subunit gamma isoform	P48736	PIK3CG	0.037
Astragali Radix（AgR）	MOL000442	1,7-Dihydroxy-3,9-dimethoxy pterocarpene	Carbonic anhydrase 4	P22748	CA4	0.038
Astragali Radix（AgR）	MOL000442	1,7-Dihydroxy-3,9-dimethoxy pterocarpene	Nitric-oxide synthase, brain	P29475	NOS1	0.039
Astragali Radix（AgR）	MOL000442	1,7-Dihydroxy-3,9-dimethoxy pterocarpene	Tryptophanyl-tRNA synthetase, mitochondrial	Q9UGM6	WARS2	0.039
Astragali Radix（AgR）	MOL000442	1,7-Dihydroxy-3,9-dimethoxy pterocarpene	5-hydroxytryptamine 2C receptor	P28335	HTR2C	0.041
Astragali Radix（AgR）	MOL000442	1,7-Dihydroxy-3,9-dimethoxy pterocarpene	Proto-oncogene serine/threonine-protein kinase Pim-1	P11309	PIM1	0.044
Astragali Radix（AgR）	MOL000442	1,7-Dihydroxy-3,9-dimethoxy pterocarpene	5-hydroxytryptamine 1B receptor	P28222	HTR1B	0.045
Astragali Radix（AgR）	MOL000442	1,7-Dihydroxy-3,9-dimethoxy pterocarpene	RAC-beta serine/threonine-protein kinase	P31751	AKT2	0.045
Astragali Radix（AgR）	MOL000442	1,7-Dihydroxy-3,9-dimethoxy pterocarpene	cAMP-dependent protein kinase inhibitor alpha	P61925	PKIA	0.045
Astragali Radix（AgR）	MOL000442	1,7-Dihydroxy-3,9-dimethoxy pterocarpene	Death-associated protein kinase 3	O43293	DAPK3	0.048
Astragali Radix（AgR）	MOL000442	1,7-Dihydroxy-3,9-dimethoxy pterocarpene	Proto-oncogene tyrosine-protein kinase LCK	P06239	LCK	0.049
Astragali Radix（AgR）	MOL000442	1,7-Dihydroxy-3,9-dimethoxy pterocarpene	Alpha-1B adrenergic receptor	P35368	ADRA1B	0.049
Astragali Radix（AgR）	MOL000442	1,7-Dihydroxy-3,9-dimethoxy pterocarpene	D(2) dopamine receptor	P14416	DRD2	0.05
Astragali Radix（AgR）	MOL000442	1,7-Dihydroxy-3,9-dimethoxy pterocarpene	5-hydroxytryptamine 2A receptor	P28223	HTR2A	0.05
Astragali Radix（AgR）	MOL000442	1,7-Dihydroxy-3,9-dimethoxy pterocarpene	Glycogen synthase kinase-3 beta	P49841	GSK3B	0.05
Astragali Radix（AgR）	MOL000442	1,7-Dihydroxy-3,9-dimethoxy pterocarpene	D-HSCDK2	O75100	CA11	0.051
Astragali Radix（AgR）	MOL000442	1,7-Dihydroxy-3,9-dimethoxy pterocarpene	Retinoic acid receptor RXR-beta	P28702	RXRB	0.053
Astragali Radix（AgR）	MOL000442	1,7-Dihydroxy-3,9-dimethoxy pterocarpene	Delta-type opioid receptor	P41143	OPRD1	0.053
Astragali Radix（AgR）	MOL000442	1,7-Dihydroxy-3,9-dimethoxy pterocarpene	Fibroblast growth factor receptor 2	P21802	FGFR2	0.054
Astragali Radix（AgR）	MOL000442	1,7-Dihydroxy-3,9-dimethoxy pterocarpene	Serine/threonine-protein kinase 6	O14965	AURKA	0.056
Astragali Radix（AgR）	MOL000442	1,7-Dihydroxy-3,9-dimethoxy pterocarpene	cGMP-specific 3',5'-cyclic phosphodiesterase	O76074	PDE5A	0.057

Astragali Radix（AgR）	MOL000442	1,7-Dihydroxy-3,9-dimethoxy pterocarpene	Leukotriene A-4 hydrolase	P09960	LTA4H	0.057
Astragali Radix（AgR）	MOL000442	1,7-Dihydroxy-3,9-dimethoxy pterocarpene	Lysozyme C	P61626	LYZ	0.057
Astragali Radix（AgR）	MOL000442	1,7-Dihydroxy-3,9-dimethoxy pterocarpene	RAC-alpha serine/threonine-protein kinase	P31749	AKT1	0.058
Astragali Radix（AgR）	MOL000442	1,7-Dihydroxy-3,9-dimethoxy pterocarpene	Epidermal growth factor receptor	P00533	EGFR	0.059
Astragali Radix（AgR）	MOL000442	1,7-Dihydroxy-3,9-dimethoxy pterocarpene	Heat shock protein HSP 90-beta	P08238	HSP90AB1	0.059
Astragali Radix（AgR）	MOL000442	1,7-Dihydroxy-3,9-dimethoxy pterocarpene	Proto-oncogene tyrosine-protein kinase Src	P12931	SRC	0.059
Astragali Radix（AgR）	MOL000442	1,7-Dihydroxy-3,9-dimethoxy pterocarpene	Cannabinoid receptor 2	P34972	CNR2	0.059
Astragali Radix（AgR）	MOL000442	1,7-Dihydroxy-3,9-dimethoxy pterocarpene	Alpha-1A adrenergic receptor	P35348	ADRA1A	0.059
Astragali Radix（AgR）	MOL000442	1,7-Dihydroxy-3,9-dimethoxy pterocarpene	ATP-sensitive inward rectifier potassium channel 1	P48048	KCNJ1	0.059
Astragali Radix（AgR）	MOL000442	1,7-Dihydroxy-3,9-dimethoxy pterocarpene	cAMP-specific 3',5'-cyclic phosphodiesterase 4D	Q08499	PDE4D	0.059
Astragali Radix（AgR）	MOL000442	1,7-Dihydroxy-3,9-dimethoxy pterocarpene	S-methyl-5-thioadenosine phosphorylase	Q13126	MTAP	0.059
Astragali Radix（AgR）	MOL000442	1,7-Dihydroxy-3,9-dimethoxy pterocarpene	Egl nine homolog 1	Q9GZT9	EGLN1	0.059
Astragali Radix（AgR）	MOL000442	1,7-Dihydroxy-3,9-dimethoxy pterocarpene	Thiamin pyrophosphokinase 1	Q9H3S4	TPK1	0.059
Astragali Radix（AgR）	MOL000442	1,7-Dihydroxy-3,9-dimethoxy pterocarpene	D(1A) dopamine receptor	P21728	DRD1	0.06
Astragali Radix（AgR）	MOL000442	1,7-Dihydroxy-3,9-dimethoxy pterocarpene	Liver carboxylesterase 1	P23141	CES1	0.06
Astragali Radix（AgR）	MOL000442	1,7-Dihydroxy-3,9-dimethoxy pterocarpene	Membrane copper amine oxidase	Q16853	AOC3	0.06
Astragali Radix（AgR）	MOL000442	1,7-Dihydroxy-3,9-dimethoxy pterocarpene	Tubulin beta-1 chain	Q9H4B7	TUBB1	0.06
Astragali Radix（AgR）	MOL000442	1,7-Dihydroxy-3,9-dimethoxy pterocarpene	Carbonic anhydrase 1	P00915	CA1	0.061
Astragali Radix（AgR）	MOL000442	1,7-Dihydroxy-3,9-dimethoxy pterocarpene	Carbonic anhydrase 2	P00918	CA2	0.061
Astragali Radix（AgR）	MOL000442	1,7-Dihydroxy-3,9-dimethoxy pterocarpene	Melatonin receptor type 1A	P48039	MTNR1A	0.061
Astragali Radix（AgR）	MOL000442	1,7-Dihydroxy-3,9-dimethoxy pterocarpene	Triosephosphate isomerase	P60174	TPI1	0.061
Astragali Radix（AgR）	MOL000442	1,7-Dihydroxy-3,9-dimethoxy pterocarpene	Calmodulin	P62158		0.061
Astragali Radix（AgR）	MOL000442	1,7-Dihydroxy-3,9-dimethoxy pterocarpene	Neutrophil gelatinase-associated lipocalin	P80188	LCN2	0.061
Astragali Radix（AgR）	MOL000442	1,7-Dihydroxy-3,9-dimethoxy pterocarpene	Nuclear receptor coactivator 2	Q15596	NCOA2	0.061
Astragali Radix（AgR）	MOL000442	1,7-Dihydroxy-3,9-dimethoxy pterocarpene	Hydroxyacid oxidase 1	Q9UJM8	HAO1	0.061
Astragali Radix（AgR）	MOL000442	1,7-Dihydroxy-3,9-dimethoxy pterocarpene	Hepatocyte growth factor receptor	P08581	MET	0.062
Astragali Radix（AgR）	MOL000442	1,7-Dihydroxy-3,9-dimethoxy pterocarpene	Toll-like receptor 7	Q9NYK1	TLR7	0.062
Astragali Radix（AgR）	MOL000442	1,7-Dihydroxy-3,9-dimethoxy pterocarpene	Dihydrofolate reductase	P00374	DHFR	0.063
Astragali Radix（AgR）	MOL000442	1,7-Dihydroxy-3,9-dimethoxy pterocarpene	Activin receptor type-1	Q04771	ACVR1	0.063
Astragali Radix（AgR）	MOL000442	1,7-Dihydroxy-3,9-dimethoxy pterocarpene	Interferon gamma	P01579	IFNG	0.064
Astragali Radix（AgR）	MOL000442	1,7-Dihydroxy-3,9-dimethoxy pterocarpene	Nuclear receptor coactivator 5	Q9HCD5	NCOA5	0.064
Astragali Radix（AgR）	MOL000442	1,7-Dihydroxy-3,9-dimethoxy pterocarpene	Tyrosine-protein phosphatase non-receptor type 1	P18031	PTPN1	0.065
Astragali Radix（AgR）	MOL000442	1,7-Dihydroxy-3,9-dimethoxy pterocarpene	Cannabinoid receptor 1	P21554	CNR1	0.066
Astragali Radix（AgR）	MOL000442	1,7-Dihydroxy-3,9-dimethoxy pterocarpene	Estradiol 17-beta-dehydrogenase 1	P14061	HSD17B1	0.068
Astragali Radix（AgR）	MOL000442	1,7-Dihydroxy-3,9-dimethoxy pterocarpene	DNA topoisomerase 2-alpha	P11388	TOP2A	0.07
Astragali Radix（AgR）	MOL000442	1,7-Dihydroxy-3,9-dimethoxy pterocarpene	Casein kinase II subunit alpha	P68400	CSNK2A1	0.07
Astragali Radix（AgR）	MOL000442	1,7-Dihydroxy-3,9-dimethoxy pterocarpene	DNA polymerase kappa	Q9UBT6	POLK	0.07
Astragali Radix（AgR）	MOL000442	1,7-Dihydroxy-3,9-dimethoxy pterocarpene	3 beta-hydroxysteroid dehydrogenase/Delta 5-->4-isomerase type I	P14060	HSD3B1	0.071
Astragali Radix（AgR）	MOL000442	1,7-Dihydroxy-3,9-dimethoxy pterocarpene	C-jun-amino-terminal kinase-interacting protein 1	Q9UQF2	MAPK8IP1	0.071
Astragali Radix（AgR）	MOL000442	1,7-Dihydroxy-3,9-dimethoxy pterocarpene	Kappa-type opioid receptor	P41145	OPRK1	0.073
Astragali Radix（AgR）	MOL000442	1,7-Dihydroxy-3,9-dimethoxy pterocarpene	Mineralocorticoid receptor	P08235	NR3C2	0.074
Astragali Radix（AgR）	MOL000442	1,7-Dihydroxy-3,9-dimethoxy pterocarpene	Cell division control protein 2 homolog	P06493	CDK1	0.081
Astragali Radix（AgR）	MOL000442	1,7-Dihydroxy-3,9-dimethoxy pterocarpene	Nitric-oxide synthase, endothelial	P29474	NOS3	0.084
Astragali Radix（AgR）	MOL000442	1,7-Dihydroxy-3,9-dimethoxy pterocarpene	Phospholipase A2, membrane associated	P14555	PLA2G2A	0.098
Astragali Radix（AgR）	MOL000442	1,7-Dihydroxy-3,9-dimethoxy pterocarpene	Ribosyldihydronicotinamide dehydrogenase [quinone]	P16083	NQO2	0.098
Astragali Radix（AgR）	MOL000442	1,7-Dihydroxy-3,9-dimethoxy pterocarpene	Phospholipase A2	P04054	PLA2G1B	0.099
Astragali Radix（AgR）	MOL000442	1,7-Dihydroxy-3,9-dimethoxy pterocarpene	cAMP-specific 3',5'-cyclic phosphodiesterase 4B	Q07343	PDE4B	0.099

Astragali Radix（AgR）	MOL000442	1,7-Dihydroxy-3,9-dimethoxy pterocarpene	Mu-type opioid receptor	P35372	OPRM1	0.107
Astragali Radix（AgR）	MOL000442	1,7-Dihydroxy-3,9-dimethoxy pterocarpene	MAP kinase-activated protein kinase 2	P49137	MAPKAPK2	0.11
Astragali Radix（AgR）	MOL000442	1,7-Dihydroxy-3,9-dimethoxy pterocarpene	Cell division protein kinase 5	Q00535	CDK5	0.11
Astragali Radix（AgR）	MOL000442	1,7-Dihydroxy-3,9-dimethoxy pterocarpene	Coagulation factor VII	P08709	F7	0.114
Astragali Radix（AgR）	MOL000442	1,7-Dihydroxy-3,9-dimethoxy pterocarpene	Androgen receptor	P10275	AR	0.115
Astragali Radix（AgR）	MOL000442	1,7-Dihydroxy-3,9-dimethoxy pterocarpene	Nitric oxide synthase, inducible	P35228	NOS2	0.13
Astragali Radix（AgR）	MOL000442	1,7-Dihydroxy-3,9-dimethoxy pterocarpene	Mitogen-activated protein kinase 14	Q16539	MAPK14	0.133
Astragali Radix（AgR）	MOL000442	1,7-Dihydroxy-3,9-dimethoxy pterocarpene	cAMP-specific 3',5'-cyclic phosphodiesterase 4A	P27815	PDE4A	0.152
Astragali Radix（AgR）	MOL000442	1,7-Dihydroxy-3,9-dimethoxy pterocarpene	Cell division protein kinase 2	P24941	CDK2	0.201
Astragali Radix（AgR）	MOL000442	1,7-Dihydroxy-3,9-dimethoxy pterocarpene	Prostaglandin G/H synthase 1	P23219	PTGS1	0.215
Astragali Radix（AgR）	MOL000442	1,7-Dihydroxy-3,9-dimethoxy pterocarpene	Nuclear receptor coactivator 1	Q15788	NCOA1	0.23
Astragali Radix（AgR）	MOL000442	1,7-Dihydroxy-3,9-dimethoxy pterocarpene	Cyclin-A2	P20248	CCNA2	0.253
Astragali Radix（AgR）	MOL000442	1,7-Dihydroxy-3,9-dimethoxy pterocarpene	Hemoglobin subunit alpha	P69905	HBA1	0.274
Astragali Radix（AgR）	MOL000442	1,7-Dihydroxy-3,9-dimethoxy pterocarpene	Prothrombin	P00734	F2	0.301
Astragali Radix（AgR）	MOL000442	1,7-Dihydroxy-3,9-dimethoxy pterocarpene	Trypsin-1	P07477	PRSS1	0.311
Astragali Radix（AgR）	MOL000442	1,7-Dihydroxy-3,9-dimethoxy pterocarpene	Progesterone receptor	P06401	PGR	0.357
Astragali Radix（AgR）	MOL000442	1,7-Dihydroxy-3,9-dimethoxy pterocarpene	Estrogen receptor beta	Q92731	ESR2	0.386
Astragali Radix（AgR）	MOL000442	1,7-Dihydroxy-3,9-dimethoxy pterocarpene	cAMP-dependent protein kinase catalytic subunit alpha	P17612	PRKACA	0.41
Astragali Radix（AgR）	MOL000442	1,7-Dihydroxy-3,9-dimethoxy pterocarpene	Prostaglandin G/H synthase 2	P35354	PTGS2	0.476
Astragali Radix（AgR）	MOL000442	1,7-Dihydroxy-3,9-dimethoxy pterocarpene	Estrogen receptor	P03372	ESR1	1
Astragali Radix（AgR）	MOL004480	acetic acid	Trehalase	O43280	TREH	Validated
Astragali Radix（AgR）	MOL004480	acetic acid	Maltase-glucoamylase, intestinal	O43451	MGAM	Validated
Astragali Radix（AgR）	MOL004480	acetic acid	Superoxide dismutase [Cu-Zn]	P00441	SOD1	Validated
Astragali Radix（AgR）	MOL004480	acetic acid	Renin	P00797	REN	Validated
Astragali Radix（AgR）	MOL004480	acetic acid	Proto-oncogene c-Fos	P01100	FOS	Validated
Astragali Radix（AgR）	MOL004480	acetic acid	Tumor necrosis factor	P01375	TNF	Validated
Astragali Radix（AgR）	MOL004480	acetic acid	Myeloperoxidase	P05164	MPO	Validated
Astragali Radix（AgR）	MOL004480	acetic acid	6-phosphofructokinase, muscle type	P08237	PFKM	Validated
Astragali Radix（AgR）	MOL004480	acetic acid	Lactase-phlorizin hydrolase	P09848	LCT	Validated
Astragali Radix（AgR）	MOL004480	acetic acid	Angiotensin-converting enzyme	P12821	ACE	Validated
Astragali Radix（AgR）	MOL004480	acetic acid	Sucrase-isomaltase, intestinal	P14410	SI	Validated
Astragali Radix（AgR）	MOL004480	acetic acid	P-selectin	P16109	SELP	Validated
Astragali Radix（AgR）	MOL004480	acetic acid	Interleukin-10	P22301	IL10	Validated
Astragali Radix（AgR）	MOL004480	acetic acid	Nitric oxide synthase, inducible	P35228	NOS2	Validated
Astragali Radix（AgR）	MOL004480	acetic acid	Prostaglandin G/H synthase 2	P35354	PTGS2	Validated
Astragali Radix（AgR）	MOL004480	acetic acid	Gamma-glutamyltransferase 5	P36269	GGT5	Validated
Astragali Radix（AgR）	MOL004480	acetic acid	Prostaglandin E2 receptor EP3 subtype	P43115	PTGER3	Validated
Astragali Radix（AgR）	MOL004480	acetic acid	Aquaporin-4	P55087	AQP4	Validated
Astragali Radix（AgR）	MOL004480	acetic acid	Transcription factor p65	Q04206	RELA	Validated
Astragali Radix（AgR）	MOL004480	acetic acid	Lysine-specific demethylase NO66	Q9H6W3	NO66	Validated
Astragali Radix（AgR）	MOL004480	acetic acid	Non-lysosomal glucosylceramidase	Q9HCG7	GBA2	Validated
Astragali Radix（AgR）	MOL004480	acetic acid	Heparan sulfate glucosamine 3-O-sulfotransferase 3A1	Q9Y663	HS3ST3A1	Validated
Ramulus Mori(RM)	MOL000422	kaempferol	Substance-P receptor	P25103	TACR1	0.01
Ramulus Mori(RM)	MOL000422	kaempferol	5-hydroxytryptamine 3 receptor	P46098	HTR3A	0.01
Ramulus Mori(RM)	MOL000422	kaempferol	Sodium channel protein type 10 subunit alpha	Q9Y5Y9	SCN10A	0.01
Ramulus Mori(RM)	MOL000422	kaempferol	Phosphatidylinositol 3-kinase regulatory subunit beta	O00459	PIK3R2	0.011
Ramulus Mori(RM)	MOL000422	kaempferol	Death-associated protein kinase 3	O43293	DAPK3	0.011

Ramulus Mori(RM)	MOL000422	kaempferol	Tyrosine-protein kinase JAK2	O60674	JAK2	0.011
Ramulus Mori(RM)	MOL000422	kaempferol	T-cell receptor alpha chain C region	P01848	TRAC	0.011
Ramulus Mori(RM)	MOL000422	kaempferol	T-cell receptor beta chain C region	P01850	TRBC1	0.011
Ramulus Mori(RM)	MOL000422	kaempferol	Trypsin-2	P07478	PRSS2	0.011
Ramulus Mori(RM)	MOL000422	kaempferol	Elongation factor 2	P13639	EEF2	0.011
Ramulus Mori(RM)	MOL000422	kaempferol	Beta-3 adrenergic receptor	P13945	ADRB3	0.011
Ramulus Mori(RM)	MOL000422	kaempferol	Tyrosine-protein kinase JAK1	P23458	JAK1	0.011
Ramulus Mori(RM)	MOL000422	kaempferol	Phosphatidylinositol 3-kinase regulatory subunit alpha	P27986	PIK3R1	0.011
Ramulus Mori(RM)	MOL000422	kaempferol	Mitogen-activated protein kinase 1	P28482	MAPK1	0.011
Ramulus Mori(RM)	MOL000422	kaempferol	Tyrosine-protein kinase JAK3	P52333	JAK3	0.011
Ramulus Mori(RM)	MOL000422	kaempferol	Beta-2-microglobulin	P61769	B2M	0.011
Ramulus Mori(RM)	MOL000422	kaempferol	Poly [ADP-ribose] polymerase 3	Q9Y6F1	PARP3	0.011
Ramulus Mori(RM)	MOL000422	kaempferol	Potassium channel subfamily K member 6	Q9Y257	KCNK6	0.012
Ramulus Mori(RM)	MOL000422	kaempferol	Gamma-aminobutyric acid receptor subunit rho-3	A8MPY1	GABRR3	0.013
Ramulus Mori(RM)	MOL000422	kaempferol	Gamma-aminobutyric acid receptor subunit pi	O00591	GABRP	0.013
Ramulus Mori(RM)	MOL000422	kaempferol	Gamma-aminobutyric acid receptor subunit delta	O14764	GABRD	0.013
Ramulus Mori(RM)	MOL000422	kaempferol	Gamma-aminobutyric-acid receptor subunit beta-1	P18505	GABRB1	0.013
Ramulus Mori(RM)	MOL000422	kaempferol	Gamma-aminobutyric acid receptor subunit gamma-2	P18507	GABRG2	0.013
Ramulus Mori(RM)	MOL000422	kaempferol	Gamma-aminobutyric-acid receptor subunit rho-1	P24046	GABRR1	0.013
Ramulus Mori(RM)	MOL000422	kaempferol	Gamma-aminobutyric acid receptor subunit rho-2	P28476	GABRR2	0.013
Ramulus Mori(RM)	MOL000422	kaempferol	Gamma-aminobutyric-acid receptor subunit alpha-5	P31644	GABRA5	0.013
Ramulus Mori(RM)	MOL000422	kaempferol	Gamma-aminobutyric-acid receptor subunit alpha-3	P34903	GABRA3	0.013
Ramulus Mori(RM)	MOL000422	kaempferol	Gamma-aminobutyric acid receptor subunit epsilon	P78334	GABRE	0.013
Ramulus Mori(RM)	MOL000422	kaempferol	Gamma-aminobutyric acid receptor subunit gamma-1	Q8N1C3	GABRG1	0.013
Ramulus Mori(RM)	MOL000422	kaempferol	Gamma-aminobutyric acid receptor subunit gamma-3	Q99928	GABRG3	0.013
Ramulus Mori(RM)	MOL000422	kaempferol	Sucrase-isomaltase, intestinal	P14410	SI	0.014
Ramulus Mori(RM)	MOL000422	kaempferol	Interleukin-3	P08700	IL3	0.015
Ramulus Mori(RM)	MOL000422	kaempferol	Protein S100-A12	P80511	S100A12	0.015
Ramulus Mori(RM)	MOL000422	kaempferol	Protein S100-A13	Q99584	S100A13	0.015
Ramulus Mori(RM)	MOL000422	kaempferol	Phenylalanine-4-hydroxylase	P00439	PAH	0.016
Ramulus Mori(RM)	MOL000422	kaempferol	Cell division protein kinase 4	P11802	CDK4	0.017
Ramulus Mori(RM)	MOL000422	kaempferol	Cell division protein kinase 7	P50613	CDK7	0.017
Ramulus Mori(RM)	MOL000422	kaempferol	Cell division protein kinase 9	P50750	CDK9	0.017
Ramulus Mori(RM)	MOL000422	kaempferol	Cell division protein kinase 6	Q00534	CDK6	0.017
Ramulus Mori(RM)	MOL000422	kaempferol	Serine/threonine-protein kinase 17B	O94768	STK17B	0.018
Ramulus Mori(RM)	MOL000422	kaempferol	Proto-oncogene serine/threonine-protein kinase Pim-1	P11309	PIM1	0.018
Ramulus Mori(RM)	MOL000422	kaempferol	ATP synthase subunit gamma, mitochondrial	P36542	ATP5F1C	0.018
Ramulus Mori(RM)	MOL000422	kaempferol	Neuronal acetylcholine receptor subunit alpha-2	Q15822	CHRNA2	0.018
Ramulus Mori(RM)	MOL000422	kaempferol	UDP-glucuronosyltransferase 3A1	Q6NUS8	UGT3A1	0.018
Ramulus Mori(RM)	MOL000422	kaempferol	Pepsin A	P00790	REN	0.019
Ramulus Mori(RM)	MOL000422	kaempferol	Tyrosine 3-monooxygenase	P07101	TH	0.019
Ramulus Mori(RM)	MOL000422	kaempferol	Retinoic acid receptor alpha	P10276	RARA	0.019
Ramulus Mori(RM)	MOL000422	kaempferol	Retinoic acid receptor beta	P10826	RARB	0.019
Ramulus Mori(RM)	MOL000422	kaempferol	Retinoic acid receptor gamma-1	P13631	RARG	0.019
Ramulus Mori(RM)	MOL000422	kaempferol	Dipeptidyl peptidase 4	P27487	DPP4	0.019
Ramulus Mori(RM)	MOL000422	kaempferol	Amiloride-sensitive sodium channel subunit alpha	P37088	SCNN1A	0.019
Ramulus Mori(RM)	MOL000422	kaempferol	Retinoic acid receptor RXR-gamma	P48443	RXRG	0.019
Ramulus Mori(RM)	MOL000422	kaempferol	Amiloride-sensitive sodium channel subunit beta	P51168	SCNN1B	0.019
Ramulus Mori(RM)	MOL000422	kaempferol	Amiloride-sensitive sodium channel subunit gamma	P51170	SCNN1G	0.019
Ramulus Mori(RM)	MOL000422	kaempferol	Peptidyl-prolyl cis-trans isomerase NIMA-interacting 1	Q13526	PIN1	0.019
Ramulus Mori(RM)	MOL000422	kaempferol	Cyclin-dependent kinase 5 activator 1	Q15078	CDK5R1	0.019
Ramulus Mori(RM)	MOL000422	kaempferol	Tyrosyl-tRNA synthetase, mitochondrial	Q9Y2Z4	YARS2	0.019
Ramulus Mori(RM)	MOL000422	kaempferol	Adenosine A2b receptor	P29275	ADORA2B	0.02

Ramulus Mori(RM)	MOL000422	kaempferol	Adenosine A3 receptor	P33765	SHMT1	0.02
Ramulus Mori(RM)	MOL000422	kaempferol	Prostaglandin reductase 1	Q14914	PTGR1	0.02
Ramulus Mori(RM)	MOL000422	kaempferol	Potassium voltage-gated channel subfamily H member 2	Q12809	KCNH2	0.021
Ramulus Mori(RM)	MOL000422	kaempferol	Potassium voltage-gated channel subfamily H member 6	Q9H252	KCNH6	0.021
Ramulus Mori(RM)	MOL000422	kaempferol	cGMP-specific 3',5'-cyclic phosphodiesterase	O76074	PDE5A	0.022
Ramulus Mori(RM)	MOL000422	kaempferol	Carbonic anhydrase 4	P22748	CA4	0.022
Ramulus Mori(RM)	MOL000422	kaempferol	Adenosine A1 receptor	P30542	ADORA1	0.022
Ramulus Mori(RM)	MOL000422	kaempferol	Sodium/potassium-transporting ATPase gamma chain	P54710	FXYD2	0.022
Ramulus Mori(RM)	MOL000422	kaempferol	cAMP and cAMP-inhibited cGMP 3',5'-cyclic phosphodiesterase 10A	Q9Y233	PDE10A	0.023
Ramulus Mori(RM)	MOL000422	kaempferol	Tryptophanyl-tRNA synthetase, mitochondrial	Q9UGM6	WARS2	0.024
Ramulus Mori(RM)	MOL000422	kaempferol	Potassium channel subfamily K member 1	O00180	KCNK1	0.025
Ramulus Mori(RM)	MOL000422	kaempferol	Tripartite motif-containing protein 13	O60858	TRIM13	0.025
Ramulus Mori(RM)	MOL000422	kaempferol	Prostacyclin receptor	P43119	PTGIR	0.025
Ramulus Mori(RM)	MOL000422	kaempferol	Methionine aminopeptidase 1	P53582	METAP1	0.025
Ramulus Mori(RM)	MOL000422	kaempferol	Peroxisome proliferator-activated receptor delta	Q03181	PPARD	0.025
Ramulus Mori(RM)	MOL000422	kaempferol	Succinate dehydrogenase [ubiquinone] cytochrome b small subunit, mitochondrial	O14521	SDHD	0.026
Ramulus Mori(RM)	MOL000422	kaempferol	Ig kappa chain C region	P01834	IGKC	0.026
Ramulus Mori(RM)	MOL000422	kaempferol	Ig gamma-1 chain C region	P01857	IGHG1	0.026
Ramulus Mori(RM)	MOL000422	kaempferol	Chymase	P23946	CMA1	0.026
Ramulus Mori(RM)	MOL000422	kaempferol	Gamma-aminobutyric-acid receptor subunit alpha-2	P47869	GABRA2	0.026
Ramulus Mori(RM)	MOL000422	kaempferol	Ribosyldihydronicotinamide dehydrogenase [quinone]	P16083	NQO2	0.027
Ramulus Mori(RM)	MOL000422	kaempferol	Peptidyl-prolyl cis-trans isomerase, mitochondrial	P30405	PPIF	0.027
Ramulus Mori(RM)	MOL000422	kaempferol	Mitogen-activated protein kinase 8	P45983	MAPK8	0.027
Ramulus Mori(RM)	MOL000422	kaempferol	Mitogen-activated protein kinase 10	P53779	MAPK10	0.027
Ramulus Mori(RM)	MOL000422	kaempferol	Inhibitor of nuclear factor kappa-B kinase subunit beta	O14920	IKBKB	0.028
Ramulus Mori(RM)	MOL000422	kaempferol	Oxysterols receptor LXR-beta	P55055	NR1H2	0.028
Ramulus Mori(RM)	MOL000422	kaempferol	Oxysterols receptor LXR-alpha	Q13133	NR1H3	0.028
Ramulus Mori(RM)	MOL000422	kaempferol	Cystine/glutamate transporter	Q9UPY5	SLC7A11	0.028
Ramulus Mori(RM)	MOL000422	kaempferol	Proto-oncogene tyrosine-protein kinase LCK	P06239	LCK	0.031
Ramulus Mori(RM)	MOL000422	kaempferol	Tyrosine-protein kinase Lyn	P07948	LYN	0.031
Ramulus Mori(RM)	MOL000422	kaempferol	Alpha-2A adrenergic receptor	P08913	ADRA2A	0.032
Ramulus Mori(RM)	MOL000422	kaempferol	Macrophage migration inhibitory factor	P14174	MIF	0.032
Ramulus Mori(RM)	MOL000422	kaempferol	DNA-(apurinic or apyrimidinic site) lyase	P27695	APEX1	0.032
Ramulus Mori(RM)	MOL000422	kaempferol	5-hydroxytryptamine 1D receptor	P28221	HTR1D	0.032
Ramulus Mori(RM)	MOL000422	kaempferol	5-hydroxytryptamine 1B receptor	P28222	HTR1B	0.032
Ramulus Mori(RM)	MOL000422	kaempferol	Sterol O-acyltransferase 2	O75908	SOAT2	0.033
Ramulus Mori(RM)	MOL000422	kaempferol	Glycogen phosphorylase, muscle form	P11217	PYGM	0.033
Ramulus Mori(RM)	MOL000422	kaempferol	Sterol O-acyltransferase 1	P35610	SOAT1	0.033
Ramulus Mori(RM)	MOL000422	kaempferol	Keratin, type II cytoskeletal 7	P08729	KRT7	0.034
Ramulus Mori(RM)	MOL000422	kaempferol	Platelet glycoprotein IX	P14770	GP9	0.034
Ramulus Mori(RM)	MOL000422	kaempferol	Gonadotropin-releasing hormone receptor	P30968	GNRHR	0.035
Ramulus Mori(RM)	MOL000422	kaempferol	Gonadotropin-releasing hormone II receptor	Q96P88	GNRHR2	0.035
Ramulus Mori(RM)	MOL000422	kaempferol	Gamma-aminobutyric-acid receptor subunit alpha-1	P14867	GABRA1	0.036
Ramulus Mori(RM)	MOL000422	kaempferol	Prolactin receptor	P16471	PRLR	0.036
Ramulus Mori(RM)	MOL000422	kaempferol	Tubulin beta-2C chain	P68371	TUBB4B	0.036
Ramulus Mori(RM)	MOL000422	kaempferol	Beta-1 adrenergic receptor	P08588	ADRB1	0.037
Ramulus Mori(RM)	MOL000422	kaempferol	Protein tyrosine kinase 2 beta	Q14289	PTK2B	0.037
Ramulus Mori(RM)	MOL000422	kaempferol	Glutamate [NMDA] receptor subunit 3A	Q8TCU5	GRIN3A	0.037
Ramulus Mori(RM)	MOL000422	kaempferol	Epidermal growth factor receptor	P00533	EGFR	0.038
Ramulus Mori(RM)	MOL000422	kaempferol	Microtubule-associated protein 2	P11137	MAP2	0.038
Ramulus Mori(RM)	MOL000422	kaempferol	3 beta-hydroxysteroid dehydrogenase/Delta 5-->4-isomerase type I	P14060	HSD3B1	0.038
Ramulus Mori(RM)	MOL000422	kaempferol	3 beta-hydroxysteroid dehydrogenase/Delta 5-->4-isomerase type II	P26439	HSD3B2	0.038

Ramulus Mori(RM)	MOL000422	kaempferol	Estrogen-related receptor gamma	P62508	ESRRG	0.038
Ramulus Mori(RM)	MOL000422	kaempferol	Microtubule-associated protein 1A	P78559	MAP1A	0.038
Ramulus Mori(RM)	MOL000422	kaempferol	Sodium-dependent dopamine transporter	Q01959	SLC6A3	0.038
Ramulus Mori(RM)	MOL000422	kaempferol	ATP synthase subunit beta, mitochondrial	P06576	ATP5F1B	0.04
Ramulus Mori(RM)	MOL000422	kaempferol	Tyrosine-protein kinase HCK	P08631	HCK	0.04
Ramulus Mori(RM)	MOL000422	kaempferol	Acetylcholinesterase	P22303	ACHE	0.04
Ramulus Mori(RM)	MOL000422	kaempferol	ATP synthase subunit alpha, mitochondrial	P25705	ATP5F1A	0.04
Ramulus Mori(RM)	MOL000422	kaempferol	Phosphatidylinositol-4,5-bisphosphate 3-kinase catalytic subunit gamma isoform	P48736	PIK3CG	0.04
Ramulus Mori(RM)	MOL000422	kaempferol	Glycogen synthase kinase-3 beta	P49841	GSK3B	0.042
Ramulus Mori(RM)	MOL000422	kaempferol	Alpha-2B adrenergic receptor	P18089	ADRA2B	0.044
Ramulus Mori(RM)	MOL000422	kaempferol	Alpha-2C adrenergic receptor	P18825	ADRA2C	0.044
Ramulus Mori(RM)	MOL000422	kaempferol	D(4) dopamine receptor	P21917	DRD4	0.044
Ramulus Mori(RM)	MOL000422	kaempferol	Alpha-1D adrenergic receptor	P25100	ADRA1D	0.045
Ramulus Mori(RM)	MOL000422	kaempferol	Adenosine A2a receptor	P29274	ADORA2A	0.047
Ramulus Mori(RM)	MOL000422	kaempferol	Muscarinic acetylcholine receptor M4	P08173	CHRM4	0.048
Ramulus Mori(RM)	MOL000422	kaempferol	Carbonic anhydrase 1	P00915	CA1	0.049
Ramulus Mori(RM)	MOL000422	kaempferol	cGMP-inhibited 3',5'-cyclic phosphodiesterase A	Q14432	PDE3A	0.05
Ramulus Mori(RM)	MOL000422	kaempferol	Beta-2 adrenergic receptor	P07550	ADRB2	0.051
Ramulus Mori(RM)	MOL000422	kaempferol	Cannabinoid receptor 1	P21554	CNR1	0.052
Ramulus Mori(RM)	MOL000422	kaempferol	Retinoic acid receptor RXR-beta	P28702	RXRB	0.052
Ramulus Mori(RM)	MOL000422	kaempferol	Dual specificity protein kinase CLK1	P49759	CLK1	0.052
Ramulus Mori(RM)	MOL000422	kaempferol	cAMP-dependent protein kinase inhibitor alpha	P61925	PKIA	0.052
Ramulus Mori(RM)	MOL000422	kaempferol	Aldo-keto reductase family 1 member C1	Q04828	AKR1C1	0.052
Ramulus Mori(RM)	MOL000422	kaempferol	Tubulin beta-1 chain	Q9H4B7	TUBB1	0.052
Ramulus Mori(RM)	MOL000422	kaempferol	Serine/threonine-protein kinase 6	O14965	AURKA	0.053
Ramulus Mori(RM)	MOL000422	kaempferol	Cytochrome P450 19A1	P11511	CYP19A1	0.053
Ramulus Mori(RM)	MOL000422	kaempferol	Egl nine homolog 1	Q9GZT9	EGLN1	0.053
Ramulus Mori(RM)	MOL000422	kaempferol	Leukotriene A-4 hydrolase	P09960	LTA4H	0.054
Ramulus Mori(RM)	MOL000422	kaempferol	RAC-alpha serine/threonine-protein kinase	P31749	AKT1	0.054
Ramulus Mori(RM)	MOL000422	kaempferol	Membrane copper amine oxidase	Q16853	AOC3	0.054
Ramulus Mori(RM)	MOL000422	kaempferol	Dihydrofolate reductase	P00374	DHFR	0.055
Ramulus Mori(RM)	MOL000422	kaempferol	Glucocorticoid receptor	P04150	NR3C1	0.055
Ramulus Mori(RM)	MOL000422	kaempferol	Thymidylate synthase	P04818	TYMS	0.055
Ramulus Mori(RM)	MOL000422	kaempferol	Thyroid hormone receptor beta-1	P10828	THRB	0.055
Ramulus Mori(RM)	MOL000422	kaempferol	Ig kappa chain V-II region RPMI 6410	P06310	IGKV2-30	0.056
Ramulus Mori(RM)	MOL000422	kaempferol	5-hydroxytryptamine 1A receptor	P08908	HTR1A	0.056
Ramulus Mori(RM)	MOL000422	kaempferol	D(1B) dopamine receptor	P21918	DRD5	0.056
Ramulus Mori(RM)	MOL000422	kaempferol	D(3) dopamine receptor	P35462	DRD3	0.056
Ramulus Mori(RM)	MOL000422	kaempferol	Triosephosphate isomerase	P60174	TPI1	0.056
Ramulus Mori(RM)	MOL000422	kaempferol	Calmodulin	P62158		0.056
Ramulus Mori(RM)	MOL000422	kaempferol	S-methyl-5-thioadenosine phosphorylase	Q13126	MTAP	0.056
Ramulus Mori(RM)	MOL000422	kaempferol	Thyroid hormone receptor alpha	P10827	THRA	0.057
Ramulus Mori(RM)	MOL000422	kaempferol	5-hydroxytryptamine 2C receptor	P28335	HTR2C	0.057
Ramulus Mori(RM)	MOL000422	kaempferol	5-hydroxytryptamine 2B receptor	P41595	HTR2B	0.057
Ramulus Mori(RM)	MOL000422	kaempferol	Solute carrier family 12 member 1	Q13621	SLC12A1	0.057
Ramulus Mori(RM)	MOL000422	kaempferol	Sodium channel protein type 5 subunit alpha	Q14524	SCN5A	0.057
Ramulus Mori(RM)	MOL000422	kaempferol	Toll-like receptor 7	Q9NYK1	TLR7	0.057
Ramulus Mori(RM)	MOL000422	kaempferol	Calcium/calmodulin-dependent protein kinase type II alpha chain	Q9UQM7	CAMK2A	0.057
Ramulus Mori(RM)	MOL000422	kaempferol	Alpha-1B adrenergic receptor	P35368	ADRA1B	0.058
Ramulus Mori(RM)	MOL000422	kaempferol	Casein kinase II subunit alpha	P68400	CSNK2A1	0.058
Ramulus Mori(RM)	MOL000422	kaempferol	Thiamin pyrophosphokinase 1	Q9H3S4	TPK1	0.058
Ramulus Mori(RM)	MOL000422	kaempferol	C-jun-amino-terminal kinase-interacting protein 1	Q9UQF2	MAPK8IP1	0.058
Ramulus Mori(RM)	MOL000422	kaempferol	Peroxisome proliferator-activated receptor gamma	P37231	PPARG	0.059

Ramulus Mori(RM)	MOL000422	kaempferol	Neuropeptide Y	P01303	NPY	0.06
Ramulus Mori(RM)	MOL000422	kaempferol	Nuclear receptor coactivator 2	Q15596	NCOA2	0.06
Ramulus Mori(RM)	MOL000422	kaempferol	Group IIE secretory phospholipase A2	Q9NZK7	PLA2G2E	0.06
Ramulus Mori(RM)	MOL000422	kaempferol	Nuclear receptor coactivator 5	Q9HCD5	NCOA5	0.062
Ramulus Mori(RM)	MOL000422	kaempferol	Interferon gamma	P01579	IFNG	0.063
Ramulus Mori(RM)	MOL000422	kaempferol	Phospholipase A2	P04054	PLA2G1B	0.063
Ramulus Mori(RM)	MOL000422	kaempferol	Fibroblast growth factor receptor 2	P21802	FGFR2	0.063
Ramulus Mori(RM)	MOL000422	kaempferol	Proto-oncogene tyrosine-protein kinase Src	P12931	SRC	0.066
Ramulus Mori(RM)	MOL000422	kaempferol	Muscarinic acetylcholine receptor M3	P20309	CHRM3	0.066
Ramulus Mori(RM)	MOL000422	kaempferol	Tyrosyl-tRNA synthetase, cytoplasmic	P54577	YARS	0.066
Ramulus Mori(RM)	MOL000422	kaempferol	DNA polymerase kappa	Q9UBT6	POLK	0.068
Ramulus Mori(RM)	MOL000422	kaempferol	DNA topoisomerase 2-alpha	P11388	TOP2A	0.072
Ramulus Mori(RM)	MOL000422	kaempferol	Progesterone receptor	P06401	PGR	0.074
Ramulus Mori(RM)	MOL000422	kaempferol	5-hydroxytryptamine 2A receptor	P28223	HTR2A	0.074
Ramulus Mori(RM)	MOL000422	kaempferol	Carbonic anhydrase 2	P00918	CA2	0.075
Ramulus Mori(RM)	MOL000422	kaempferol	Tubulin alpha-3 chain	Q71U36	TUBA1A	0.077
Ramulus Mori(RM)	MOL000422	kaempferol	D(1A) dopamine receptor	P21728	DRD1	0.081
Ramulus Mori(RM)	MOL000422	kaempferol	D-HSCDK2	O75100	CA11	0.084
Ramulus Mori(RM)	MOL000422	kaempferol	Muscarinic acetylcholine receptor M2	P08172	CHRM2	0.084
Ramulus Mori(RM)	MOL000422	kaempferol	Delta-type opioid receptor	P41143	OPRD1	0.085
Ramulus Mori(RM)	MOL000422	kaempferol	Kappa-type opioid receptor	P41145	OPRK1	0.085
Ramulus Mori(RM)	MOL000422	kaempferol	Inhibitor of nuclear factor kappa-B kinase subunit alpha	O15111	CHUK	0.091
Ramulus Mori(RM)	MOL000422	kaempferol	Arachidonate 5-lipoxygenase	P09917	ALOX5	0.091
Ramulus Mori(RM)	MOL000422	kaempferol	D(2) dopamine receptor	P14416	DRD2	0.093
Ramulus Mori(RM)	MOL000422	kaempferol	Sodium-dependent noradrenaline transporter	P23975	SLC6A2	0.094
Ramulus Mori(RM)	MOL000422	kaempferol	Alpha-1A adrenergic receptor	P35348	ADRA1A	0.098
Ramulus Mori(RM)	MOL000422	kaempferol	Muscarinic acetylcholine receptor M1	P11229	CHRM1	0.099
Ramulus Mori(RM)	MOL000422	kaempferol	cAMP-specific 3',5'-cyclic phosphodiesterase 4A	P27815	PDE4A	0.104
Ramulus Mori(RM)	MOL000422	kaempferol	Sodium-dependent serotonin transporter	P31645	SLC6A4	0.105
Ramulus Mori(RM)	MOL000422	kaempferol	Prothrombin	P00734	F2	0.106
Ramulus Mori(RM)	MOL000422	kaempferol	Cell division control protein 2 homolog	P06493	CDK1	0.107
Ramulus Mori(RM)	MOL000422	kaempferol	Nitric oxide synthase, inducible	P35228	NOS2	0.113
Ramulus Mori(RM)	MOL000422	kaempferol	cAMP-specific 3',5'-cyclic phosphodiesterase 4D	Q08499	PDE4D	0.113
Ramulus Mori(RM)	MOL000422	kaempferol	Androgen receptor	P10275	AR	0.115
Ramulus Mori(RM)	MOL000422	kaempferol	cAMP-dependent protein kinase catalytic subunit alpha	P17612	PRKACA	0.116
Ramulus Mori(RM)	MOL000422	kaempferol	Cell division protein kinase 5	Q00535	CDK5	0.129
Ramulus Mori(RM)	MOL000422	kaempferol	cAMP-specific 3',5'-cyclic phosphodiesterase 4B	Q07343	PDE4B	0.131
Ramulus Mori(RM)	MOL000422	kaempferol	Cell division protein kinase 2	P24941	CDK2	0.153
Ramulus Mori(RM)	MOL000422	kaempferol	Trypsin-1	P07477	PRSS1	0.157
Ramulus Mori(RM)	MOL000422	kaempferol	MAP kinase-activated protein kinase 2	P49137	MAPKAPK2	0.174
Ramulus Mori(RM)	MOL000422	kaempferol	Hemoglobin subunit alpha	P69905	HBA1	0.191
Ramulus Mori(RM)	MOL000422	kaempferol	Mu-type opioid receptor	P35372	OPRM1	0.203
Ramulus Mori(RM)	MOL000422	kaempferol	Prostaglandin G/H synthase 1	P23219	PTGS1	0.25
Ramulus Mori(RM)	MOL000422	kaempferol	Nuclear receptor coactivator 1	Q15788	NCOA1	0.265
Ramulus Mori(RM)	MOL000422	kaempferol	Cyclin-A2	P20248	CCNA2	0.342
Ramulus Mori(RM)	MOL000422	kaempferol	Estrogen receptor beta	Q92731	ESR2	0.383
Ramulus Mori(RM)	MOL000422	kaempferol	Prostaglandin G/H synthase 2	P35354	PTGS2	0.573
Ramulus Mori(RM)	MOL000422	kaempferol	Estrogen receptor	P03372	ESR1	1
Ramulus Mori(RM)	MOL000729	Oxysanguinarine	Beta-3 adrenergic receptor	P13945	ADRB3	0.01
Ramulus Mori(RM)	MOL000729	Oxysanguinarine	Tyrosinase	P14679	TYR	0.01
Ramulus Mori(RM)	MOL000729	Oxysanguinarine	3-oxo-5-alpha-steroid 4-dehydrogenase 2	P31213	SRD5A2	0.01
Ramulus Mori(RM)	MOL000729	Oxysanguinarine	3-oxo-5-beta-steroid 4-dehydrogenase	P51857	AKR1D1	0.01
Ramulus Mori(RM)	MOL000729	Oxysanguinarine	Retinal dehydrogenase 2	O94788	ALDH1A2	0.011

Ramulus Mori(RM)	MOL000729	Oxysanguinarine	Retinal dehydrogenase 1	P00352	ALDH1A1	0.011
Ramulus Mori(RM)	MOL000729	Oxysanguinarine	Elongation factor 2	P13639	EEF2	0.011
Ramulus Mori(RM)	MOL000729	Oxysanguinarine	Nuclear receptor 0B1	P51843	NR0B1	0.011
Ramulus Mori(RM)	MOL000729	Oxysanguinarine	Retinoic acid-induced protein 3	Q8NFJ5	GPRC5A	0.011
Ramulus Mori(RM)	MOL000729	Oxysanguinarine	Poly [ADP-ribose] polymerase 3	Q9Y6F1	PARP3	0.011
Ramulus Mori(RM)	MOL000729	Oxysanguinarine	5-hydroxytryptamine 3 receptor	P46098	HTR3A	0.012
Ramulus Mori(RM)	MOL000729	Oxysanguinarine	5-hydroxytryptamine 4 receptor	Q13639	HTR4	0.012
Ramulus Mori(RM)	MOL000729	Oxysanguinarine	High affinity cAMP-specific and IBMX-insensitive 3',5'-cyclic phosphodiesterase 8A	O60658	PDE8A	0.014
Ramulus Mori(RM)	MOL000729	Oxysanguinarine	6-phosphogluconate dehydrogenase, decarboxylating	P52209	PGD	0.014
Ramulus Mori(RM)	MOL000729	Oxysanguinarine	cAMP-specific 3',5'-cyclic phosphodiesterase 4C	Q08493	PDE4C	0.014
Ramulus Mori(RM)	MOL000729	Oxysanguinarine	High-affinity cAMP-specific 3',5'-cyclic phosphodiesterase 7A	Q13946	PDE7A	0.014
Ramulus Mori(RM)	MOL000729	Oxysanguinarine	cAMP-specific 3',5'-cyclic phosphodiesterase 7B	Q9NP56	PDE7B	0.014
Ramulus Mori(RM)	MOL000729	Oxysanguinarine	Delta-type opioid receptor	P41143	OPRD1	0.015
Ramulus Mori(RM)	MOL000729	Oxysanguinarine	Kappa-type opioid receptor	P41145	OPRK1	0.015
Ramulus Mori(RM)	MOL000729	Oxysanguinarine	Serine/threonine-protein kinase 17B	O94768	STK17B	0.016
Ramulus Mori(RM)	MOL000729	Oxysanguinarine	Furin	P09958	FURIN	0.016
Ramulus Mori(RM)	MOL000729	Oxysanguinarine	Phosphatidylinositol 3-kinase regulatory subunit alpha	P27986	PIK3R1	0.016
Ramulus Mori(RM)	MOL000729	Oxysanguinarine	ATP synthase subunit gamma, mitochondrial	P36542	ATP5F1C	0.016
Ramulus Mori(RM)	MOL000729	Oxysanguinarine	Amiloride-sensitive sodium channel subunit alpha	P37088	SCNN1A	0.016
Ramulus Mori(RM)	MOL000729	Oxysanguinarine	Amiloride-sensitive sodium channel subunit beta	P51168	SCNN1B	0.016
Ramulus Mori(RM)	MOL000729	Oxysanguinarine	Amiloride-sensitive sodium channel subunit gamma	P51170	SCNN1G	0.016
Ramulus Mori(RM)	MOL000729	Oxysanguinarine	Serine/threonine-protein kinase PLK1	P53350	PLK1	0.016
Ramulus Mori(RM)	MOL000729	Oxysanguinarine	UDP-glucuronosyltransferase 3A1	Q6NUS8	UGT3A1	0.016
Ramulus Mori(RM)	MOL000729	Oxysanguinarine	Glycolipid transfer protein	Q9NZD2	GLTP	0.016
Ramulus Mori(RM)	MOL000729	Oxysanguinarine	Potassium channel subfamily K member 6	Q9Y257	KCNK6	0.016
Ramulus Mori(RM)	MOL000729	Oxysanguinarine	Dihydrofolate reductase	P00374	DHFR	0.017
Ramulus Mori(RM)	MOL000729	Oxysanguinarine	cGMP-inhibited 3',5'-cyclic phosphodiesterase A	Q14432	PDE3A	0.017
Ramulus Mori(RM)	MOL000729	Oxysanguinarine	cAMP and cAMP-inhibited cGMP 3',5'-cyclic phosphodiesterase 10A	Q9Y233	PDE10A	0.017
Ramulus Mori(RM)	MOL000729	Oxysanguinarine	Tyrosine-protein kinase JAK2	O60674	JAK2	0.02
Ramulus Mori(RM)	MOL000729	Oxysanguinarine	Tyrosine-protein kinase Lyn	P07948	LYN	0.02
Ramulus Mori(RM)	MOL000729	Oxysanguinarine	Tyrosine-protein kinase JAK1	P23458	JAK1	0.02
Ramulus Mori(RM)	MOL000729	Oxysanguinarine	Tyrosine-protein kinase JAK3	P52333	JAK3	0.02
Ramulus Mori(RM)	MOL000729	Oxysanguinarine	Cyclin-dependent kinase 5 activator 1	Q15078	CDK5R1	0.02
Ramulus Mori(RM)	MOL000729	Oxysanguinarine	3-phosphoinositide-dependent protein kinase 1	O15530	PDPK1	0.021
Ramulus Mori(RM)	MOL000729	Oxysanguinarine	Glycogen phosphorylase, muscle form	P11217	PYGM	0.021
Ramulus Mori(RM)	MOL000729	Oxysanguinarine	Cell division protein kinase 4	P11802	CDK4	0.021
Ramulus Mori(RM)	MOL000729	Oxysanguinarine	Neuronal acetylcholine receptor subunit alpha-3	P32297	CHRNA3	0.021
Ramulus Mori(RM)	MOL000729	Oxysanguinarine	Neuronal acetylcholine receptor subunit alpha-7	P36544	CHRNA7	0.021
Ramulus Mori(RM)	MOL000729	Oxysanguinarine	Tyrosine-protein kinase CSK	P41240	CSK	0.021
Ramulus Mori(RM)	MOL000729	Oxysanguinarine	Tyrosine-protein kinase ZAP-70	P43403	ZAP70	0.021
Ramulus Mori(RM)	MOL000729	Oxysanguinarine	Tyrosine-protein kinase SYK	P43405	SYK	0.021
Ramulus Mori(RM)	MOL000729	Oxysanguinarine	Cell division protein kinase 7	P50613	CDK7	0.021
Ramulus Mori(RM)	MOL000729	Oxysanguinarine	Cell division protein kinase 9	P50750	CDK9	0.021
Ramulus Mori(RM)	MOL000729	Oxysanguinarine	Cell division protein kinase 6	Q00534	CDK6	0.021
Ramulus Mori(RM)	MOL000729	Oxysanguinarine	Protein kinase C theta type	Q04759	PRKCQ	0.021
Ramulus Mori(RM)	MOL000729	Oxysanguinarine	Tyrosine-protein kinase ITK/TSK	Q08881	ITK	0.021
Ramulus Mori(RM)	MOL000729	Oxysanguinarine	Sodium-dependent dopamine transporter	Q01959	SLC6A3	0.022
Ramulus Mori(RM)	MOL000729	Oxysanguinarine	Synaptic vesicular amine transporter	Q05940	SLC18A2	0.022
Ramulus Mori(RM)	MOL000729	Oxysanguinarine	Neuronal acetylcholine receptor subunit alpha-2	Q15822	CHRNA2	0.022
Ramulus Mori(RM)	MOL000729	Oxysanguinarine	Dehydrogenase/reductase SDR family member 8	Q8NBQ5	HSD17B11	0.022
Ramulus Mori(RM)	MOL000729	Oxysanguinarine	D(1B) dopamine receptor	P21918	DRD5	0.023
Ramulus Mori(RM)	MOL000729	Oxysanguinarine	Glutamate [NMDA] receptor subunit 3A	Q8TCU5	GRIN3A	0.023
Ramulus Mori(RM)	MOL000729	Oxysanguinarine	Gamma-aminobutyric-acid receptor subunit alpha-3	P34903	GABRA3	0.024

Ramulus Mori(RM)	MOL000729	Oxysanguinarine	Lipoic acid synthetase, mitochondrial	O43766	LIAS	0.025
Ramulus Mori(RM)	MOL000729	Oxysanguinarine	T-cell receptor alpha chain C region	P01848	TRAC	0.025
Ramulus Mori(RM)	MOL000729	Oxysanguinarine	T-cell receptor beta chain C region	P01850	TRBC1	0.025
Ramulus Mori(RM)	MOL000729	Oxysanguinarine	Myeloperoxidase	P05164	MPO	0.025
Ramulus Mori(RM)	MOL000729	Oxysanguinarine	Eosinophil peroxidase	P11678	EPX	0.025
Ramulus Mori(RM)	MOL000729	Oxysanguinarine	Calreticulin	P27797	CALR	0.025
Ramulus Mori(RM)	MOL000729	Oxysanguinarine	Melatonin receptor type 1B	P49286	MTNR1B	0.025
Ramulus Mori(RM)	MOL000729	Oxysanguinarine	Beta-2-microglobulin	P61769	B2M	0.025
Ramulus Mori(RM)	MOL000729	Oxysanguinarine	Protein S100-A12	P80511	S100A12	0.025
Ramulus Mori(RM)	MOL000729	Oxysanguinarine	Nuclear receptor subfamily 1 group I member 3	Q14994	NR1I3	0.025
Ramulus Mori(RM)	MOL000729	Oxysanguinarine	Nuclear receptor ROR-beta	Q92753	RORB	0.025
Ramulus Mori(RM)	MOL000729	Oxysanguinarine	Protein S100-A13	Q99584	S100A13	0.025
Ramulus Mori(RM)	MOL000729	Oxysanguinarine	Lipoyltransferase 1, mitochondrial	Q9Y234	LIPT1	0.025
Ramulus Mori(RM)	MOL000729	Oxysanguinarine	85 kDa calcium-independent phospholipase A2	O60733	PLA2G6	0.026
Ramulus Mori(RM)	MOL000729	Oxysanguinarine	Cytosolic phospholipase A2	P47712	PLA2G4A	0.026
Ramulus Mori(RM)	MOL000729	Oxysanguinarine	S-phase kinase-associated protein 1	P63208	SKP1	0.026
Ramulus Mori(RM)	MOL000729	Oxysanguinarine	Group IIE secretory phospholipase A2	Q9NZK7	PLA2G2E	0.026
Ramulus Mori(RM)	MOL000729	Oxysanguinarine	Beta-1 adrenergic receptor	P08588	ADRB1	0.027
Ramulus Mori(RM)	MOL000729	Oxysanguinarine	DNA topoisomerase 1	P11387	TOP1	0.028
Ramulus Mori(RM)	MOL000729	Oxysanguinarine	Carbonic anhydrase 4	P22748	CA4	0.028
Ramulus Mori(RM)	MOL000729	Oxysanguinarine	Mitogen-activated protein kinase 8	P45983	MAPK8	0.028
Ramulus Mori(RM)	MOL000729	Oxysanguinarine	Mitogen-activated protein kinase 10	P53779	MAPK10	0.028
Ramulus Mori(RM)	MOL000729	Oxysanguinarine	Sodium/potassium-transporting ATPase gamma chain	P54710	FXYD2	0.028
Ramulus Mori(RM)	MOL000729	Oxysanguinarine	DNA topoisomerase I, mitochondrial	Q969P6	TOP1MT	0.028
Ramulus Mori(RM)	MOL000729	Oxysanguinarine	Ig kappa chain C region	P01834	IGKC	0.029
Ramulus Mori(RM)	MOL000729	Oxysanguinarine	Ig gamma-2 chain C region	P01859	IGHG2	0.029
Ramulus Mori(RM)	MOL000729	Oxysanguinarine	DNA-(apurinic or apyrimidinic site) lyase	P27695	APEX1	0.029
Ramulus Mori(RM)	MOL000729	Oxysanguinarine	Retinoic acid receptor RXR-gamma	P48443	RXRG	0.029
Ramulus Mori(RM)	MOL000729	Oxysanguinarine	Retinoic acid receptor alpha	P10276	RARA	0.031
Ramulus Mori(RM)	MOL000729	Oxysanguinarine	Retinoic acid receptor beta	P10826	RARB	0.031
Ramulus Mori(RM)	MOL000729	Oxysanguinarine	Tubulin alpha-1 chain	P68366	TUBA4A	0.031
Ramulus Mori(RM)	MOL000729	Oxysanguinarine	Tyrosyl-tRNA synthetase, cytoplasmic	P54577	YARS	0.032
Ramulus Mori(RM)	MOL000729	Oxysanguinarine	Peptidyl-prolyl cis-trans isomerase NIMA-interacting 1	Q13526	PIN1	0.032
Ramulus Mori(RM)	MOL000729	Oxysanguinarine	Sterol O-acyltransferase 2	O75908	SOAT2	0.033
Ramulus Mori(RM)	MOL000729	Oxysanguinarine	Urokinase-type plasminogen activator	P00749	PLAU	0.033
Ramulus Mori(RM)	MOL000729	Oxysanguinarine	Alcohol dehydrogenase [NADP+]	P14550	AKR1A1	0.033
Ramulus Mori(RM)	MOL000729	Oxysanguinarine	3 beta-hydroxysteroid dehydrogenase/Delta 5-->4-isomerase type II	P26439	HSD3B2	0.033
Ramulus Mori(RM)	MOL000729	Oxysanguinarine	Sterol O-acyltransferase 1	P35610	SOAT1	0.033
Ramulus Mori(RM)	MOL000729	Oxysanguinarine	Prostacyclin receptor	P43119	PTGIR	0.033
Ramulus Mori(RM)	MOL000729	Oxysanguinarine	Mitogen-activated protein kinase 3	P27361	MAPK3	0.034
Ramulus Mori(RM)	MOL000729	Oxysanguinarine	Protein tyrosine kinase 2 beta	Q14289	PTK2B	0.034
Ramulus Mori(RM)	MOL000729	Oxysanguinarine	Microtubule-associated protein 2	P11137	MAP2	0.035
Ramulus Mori(RM)	MOL000729	Oxysanguinarine	Estrogen-related receptor gamma	P62508	ESRRG	0.035
Ramulus Mori(RM)	MOL000729	Oxysanguinarine	Microtubule-associated protein 1A	P78559	MAP1A	0.035
Ramulus Mori(RM)	MOL000729	Oxysanguinarine	Potassium channel subfamily K member 1	O00180	KCNK1	0.036
Ramulus Mori(RM)	MOL000729	Oxysanguinarine	Prolactin receptor	P16471	PRLR	0.036
Ramulus Mori(RM)	MOL000729	Oxysanguinarine	3-oxo-5-alpha-steroid 4-dehydrogenase 1	P18405	SRD5A1	0.036
Ramulus Mori(RM)	MOL000729	Oxysanguinarine	Acetylcholinesterase	P22303	ACHE	0.036
Ramulus Mori(RM)	MOL000729	Oxysanguinarine	Nitric-oxide synthase, brain	P29475	NOS1	0.036
Ramulus Mori(RM)	MOL000729	Oxysanguinarine	Gonadotropin-releasing hormone receptor	P30968	GNRHR	0.036
Ramulus Mori(RM)	MOL000729	Oxysanguinarine	cAMP-specific 3',5'-cyclic phosphodiesterase 4D	Q08499	PDE4D	0.036
Ramulus Mori(RM)	MOL000729	Oxysanguinarine	Gonadotropin-releasing hormone II receptor	Q96P88	GNRHR2	0.036
Ramulus Mori(RM)	MOL000729	Oxysanguinarine	Corticosteroid 11-beta-dehydrogenase isozyme 1	P28845	HSD11B1	0.037

Ramulus Mori(RM)	MOL000729	Oxysanguinarine	Potassium voltage-gated channel subfamily H member 2	Q12809	KCNH2	0.037
Ramulus Mori(RM)	MOL000729	Oxysanguinarine	Potassium voltage-gated channel subfamily H member 6	Q9H252	KCNH6	0.037
Ramulus Mori(RM)	MOL000729	Oxysanguinarine	Alpha-2B adrenergic receptor	P18089	ADRA2B	0.038
Ramulus Mori(RM)	MOL000729	Oxysanguinarine	Alpha-2C adrenergic receptor	P18825	ADRA2C	0.038
Ramulus Mori(RM)	MOL000729	Oxysanguinarine	5-hydroxytryptamine 1F receptor	P30939	HTR1F	0.038
Ramulus Mori(RM)	MOL000729	Oxysanguinarine	D(3) dopamine receptor	P35462	DRD3	0.038
Ramulus Mori(RM)	MOL000729	Oxysanguinarine	Tubulin beta-2C chain	P68371	TUBB4B	0.038
Ramulus Mori(RM)	MOL000729	Oxysanguinarine	ATP synthase subunit beta, mitochondrial	P06576	ATP5F1B	0.039
Ramulus Mori(RM)	MOL000729	Oxysanguinarine	Tyrosine-protein kinase HCK	P08631	HCK	0.039
Ramulus Mori(RM)	MOL000729	Oxysanguinarine	ATP synthase subunit alpha, mitochondrial	P25705	ATP5F1A	0.039
Ramulus Mori(RM)	MOL000729	Oxysanguinarine	Platelet glycoprotein IX	P14770	GP9	0.04
Ramulus Mori(RM)	MOL000729	Oxysanguinarine	Tryptophanyl-tRNA synthetase, mitochondrial	Q9UGM6	WARS2	0.04
Ramulus Mori(RM)	MOL000729	Oxysanguinarine	Calmodulin	P62158		0.041
Ramulus Mori(RM)	MOL000729	Oxysanguinarine	Beta-2 adrenergic receptor	P07550	ADRB2	0.044
Ramulus Mori(RM)	MOL000729	Oxysanguinarine	Proto-oncogene serine/threonine-protein kinase Pim-1	P11309	PIM1	0.044
Ramulus Mori(RM)	MOL000729	Oxysanguinarine	Death-associated protein kinase 3	O43293	DAPK3	0.048
Ramulus Mori(RM)	MOL000729	Oxysanguinarine	Epidermal growth factor receptor	P00533	EGFR	0.048
Ramulus Mori(RM)	MOL000729	Oxysanguinarine	Proto-oncogene tyrosine-protein kinase LCK	P06239	LCK	0.048
Ramulus Mori(RM)	MOL000729	Oxysanguinarine	Glycogen synthase kinase-3 beta	P49841	GSK3B	0.048
Ramulus Mori(RM)	MOL000729	Oxysanguinarine	Retinoic acid receptor gamma-1	P13631	RARG	0.049
Ramulus Mori(RM)	MOL000729	Oxysanguinarine	RAC-beta serine/threonine-protein kinase	P31751	AKT2	0.051
Ramulus Mori(RM)	MOL000729	Oxysanguinarine	Sodium-dependent noradrenaline transporter	P23975	SLC6A2	0.052
Ramulus Mori(RM)	MOL000729	Oxysanguinarine	Alpha-2A adrenergic receptor	P08913	ADRA2A	0.053
Ramulus Mori(RM)	MOL000729	Oxysanguinarine	D(4) dopamine receptor	P21917	DRD4	0.053
Ramulus Mori(RM)	MOL000729	Oxysanguinarine	Bile salt sulfotransferase	Q06520	SULT2A1	0.054
Ramulus Mori(RM)	MOL000729	Oxysanguinarine	Serine/threonine-protein kinase 6	O14965	AURKA	0.057
Ramulus Mori(RM)	MOL000729	Oxysanguinarine	Mu-type opioid receptor	P35372	OPRM1	0.057
Ramulus Mori(RM)	MOL000729	Oxysanguinarine	Melatonin receptor type 1A	P48039	MTNR1A	0.057
Ramulus Mori(RM)	MOL000729	Oxysanguinarine	Dual specificity protein kinase CLK1	P49759	CLK1	0.057
Ramulus Mori(RM)	MOL000729	Oxysanguinarine	NADPH oxidase organizer 1	Q8NFA2	NOXO1	0.057
Ramulus Mori(RM)	MOL000729	Oxysanguinarine	Protein farnesyltransferase/geranylgeranyltransferase type I alpha subunit	P49354	FNTA	0.058
Ramulus Mori(RM)	MOL000729	Oxysanguinarine	Thiamin pyrophosphokinase 1	Q9H3S4	TPK1	0.058
Ramulus Mori(RM)	MOL000729	Oxysanguinarine	Thyroid hormone receptor beta-1	P10828	THRB	0.059
Ramulus Mori(RM)	MOL000729	Oxysanguinarine	Cannabinoid receptor 2	P34972	CNR2	0.059
Ramulus Mori(RM)	MOL000729	Oxysanguinarine	cAMP-specific 3',5'-cyclic phosphodiesterase 4B	Q07343	PDE4B	0.059
Ramulus Mori(RM)	MOL000729	Oxysanguinarine	Mitogen-activated protein kinase 14	Q16539	MAPK14	0.059
Ramulus Mori(RM)	MOL000729	Oxysanguinarine	Calcium/calmodulin-dependent protein kinase type II alpha chain	Q9UQM7	CAMK2A	0.059
Ramulus Mori(RM)	MOL000729	Oxysanguinarine	Lysozyme C	P61626	LYZ	0.06
Ramulus Mori(RM)	MOL000729	Oxysanguinarine	Glucocorticoid receptor	P04150	NR3C1	0.061
Ramulus Mori(RM)	MOL000729	Oxysanguinarine	Ig kappa chain V-II region RPMI 6410	P06310	IGKV2-30	0.061
Ramulus Mori(RM)	MOL000729	Oxysanguinarine	Thyroid hormone receptor alpha	P10827	THRA	0.061
Ramulus Mori(RM)	MOL000729	Oxysanguinarine	Gamma-aminobutyric-acid receptor subunit alpha-2	P47869	GABRA2	0.061
Ramulus Mori(RM)	MOL000729	Oxysanguinarine	Colipase	P04118	CLPS	0.062
Ramulus Mori(RM)	MOL000729	Oxysanguinarine	Ribosyldihydronicotinamide dehydrogenase [quinone]	P16083	NQO2	0.062
Ramulus Mori(RM)	MOL000729	Oxysanguinarine	Phosphatidylinositol-4,5-bisphosphate 3-kinase catalytic subunit gamma isoform	P48736	PIK3CG	0.062
Ramulus Mori(RM)	MOL000729	Oxysanguinarine	Tubulin beta-1 chain	Q9H4B7	TUBB1	0.063
Ramulus Mori(RM)	MOL000729	Oxysanguinarine	Casein kinase II subunit alpha	P68400	CSNK2A1	0.064
Ramulus Mori(RM)	MOL000729	Oxysanguinarine	Neutrophil gelatinase-associated lipocalin	P80188	LCN2	0.064
Ramulus Mori(RM)	MOL000729	Oxysanguinarine	C-jun-amino-terminal kinase-interacting protein 1	Q9UQF2	MAPK8IP1	0.064
Ramulus Mori(RM)	MOL000729	Oxysanguinarine	Ig gamma-1 chain C region	P01857	IGHG1	0.066
Ramulus Mori(RM)	MOL000729	Oxysanguinarine	Hepatocyte growth factor receptor	P08581	MET	0.066
Ramulus Mori(RM)	MOL000729	Oxysanguinarine	Alpha-1D adrenergic receptor	P25100	ADRA1D	0.066

Ramulus Mori(RM)	MOL000729	Oxysanguinarine	Aldo-keto reductase family 1 member C1	Q04828	AKR1C1	0.066
Ramulus Mori(RM)	MOL000729	Oxysanguinarine	Hydroxyacid oxidase 1	Q9UJM8	HAO1	0.066
Ramulus Mori(RM)	MOL000729	Oxysanguinarine	Retinoic acid receptor RXR-alpha	P19793	RXRA	0.067
Ramulus Mori(RM)	MOL000729	Oxysanguinarine	Sodium-dependent serotonin transporter	P31645	SLC6A4	0.067
Ramulus Mori(RM)	MOL000729	Oxysanguinarine	Triosephosphate isomerase	P60174	TPI1	0.067
Ramulus Mori(RM)	MOL000729	Oxysanguinarine	Interferon gamma	P01579	IFNG	0.068
Ramulus Mori(RM)	MOL000729	Oxysanguinarine	5-hydroxytryptamine 1B receptor	P28222	HTR1B	0.068
Ramulus Mori(RM)	MOL000729	Oxysanguinarine	5-hydroxytryptamine 2B receptor	P41595	HTR2B	0.068
Ramulus Mori(RM)	MOL000729	Oxysanguinarine	Muscarinic acetylcholine receptor M5	P08912	CHRM5	0.069
Ramulus Mori(RM)	MOL000729	Oxysanguinarine	Proto-oncogene tyrosine-protein kinase Src	P12931	SRC	0.069
Ramulus Mori(RM)	MOL000729	Oxysanguinarine	3 beta-hydroxysteroid dehydrogenase/Delta 5-->4-isomerase type I	P14060	HSD3B1	0.07
Ramulus Mori(RM)	MOL000729	Oxysanguinarine	Retinoic acid receptor RXR-beta	P28702	RXRB	0.071
Ramulus Mori(RM)	MOL000729	Oxysanguinarine	Nuclear receptor coactivator 2	Q15596	NCOA2	0.073
Ramulus Mori(RM)	MOL000729	Oxysanguinarine	Nuclear receptor coactivator 5	Q9HCD5	NCOA5	0.073
Ramulus Mori(RM)	MOL000729	Oxysanguinarine	Peroxisome proliferator-activated receptor alpha	Q07869	PPARA	0.074
Ramulus Mori(RM)	MOL000729	Oxysanguinarine	DNA polymerase kappa	Q9UBT6	POLK	0.074
Ramulus Mori(RM)	MOL000729	Oxysanguinarine	Aldose reductase	P15121	AKR1B1	0.075
Ramulus Mori(RM)	MOL000729	Oxysanguinarine	D-HSCDK2	O75100	CA11	0.076
Ramulus Mori(RM)	MOL000729	Oxysanguinarine	Gamma-aminobutyric-acid receptor subunit alpha-1	P14867	GABRA1	0.079
Ramulus Mori(RM)	MOL000729	Oxysanguinarine	Nitric-oxide synthase, endothelial	P29474	NOS3	0.08
Ramulus Mori(RM)	MOL000729	Oxysanguinarine	Sodium channel protein type 5 subunit alpha	Q14524	SCN5A	0.082
Ramulus Mori(RM)	MOL000729	Oxysanguinarine	5-hydroxytryptamine 1D receptor	P28221	HTR1D	0.083
Ramulus Mori(RM)	MOL000729	Oxysanguinarine	Tubulin alpha-3 chain	Q71U36	TUBA1A	0.083
Ramulus Mori(RM)	MOL000729	Oxysanguinarine	Muscarinic acetylcholine receptor M4	P08173	CHRM4	0.084
Ramulus Mori(RM)	MOL000729	Oxysanguinarine	Muscarinic acetylcholine receptor M3	P20309	CHRM3	0.084
Ramulus Mori(RM)	MOL000729	Oxysanguinarine	cAMP-specific 3',5'-cyclic phosphodiesterase 4A	P27815	PDE4A	0.084
Ramulus Mori(RM)	MOL000729	Oxysanguinarine	Phospholipase A2	P04054	PLA2G1B	0.085
Ramulus Mori(RM)	MOL000729	Oxysanguinarine	Phospholipase A2, membrane associated	P14555	PLA2G2A	0.087
Ramulus Mori(RM)	MOL000729	Oxysanguinarine	Carbonic anhydrase 1	P00915	CA1	0.098
Ramulus Mori(RM)	MOL000729	Oxysanguinarine	Carbonic anhydrase 2	P00918	CA2	0.098
Ramulus Mori(RM)	MOL000729	Oxysanguinarine	5-hydroxytryptamine 2C receptor	P28335	HTR2C	0.098
Ramulus Mori(RM)	MOL000729	Oxysanguinarine	Muscarinic acetylcholine receptor M2	P08172	CHRM2	0.099
Ramulus Mori(RM)	MOL000729	Oxysanguinarine	Muscarinic acetylcholine receptor M1	P11229	CHRM1	0.099
Ramulus Mori(RM)	MOL000729	Oxysanguinarine	Cell division control protein 2 homolog	P06493	CDK1	0.104
Ramulus Mori(RM)	MOL000729	Oxysanguinarine	Histamine H1 receptor	P35367	HRH1	0.106
Ramulus Mori(RM)	MOL000729	Oxysanguinarine	cAMP-dependent protein kinase inhibitor alpha	P61925	PKIA	0.107
Ramulus Mori(RM)	MOL000729	Oxysanguinarine	DNA topoisomerase 2-alpha	P11388	TOP2A	0.109
Ramulus Mori(RM)	MOL000729	Oxysanguinarine	MAP kinase-activated protein kinase 2	P49137	MAPKAPK2	0.11
Ramulus Mori(RM)	MOL000729	Oxysanguinarine	Alpha-1B adrenergic receptor	P35368	ADRA1B	0.111
Ramulus Mori(RM)	MOL000729	Oxysanguinarine	Peroxisome proliferator-activated receptor gamma	P37231	PPARG	0.115
Ramulus Mori(RM)	MOL000729	Oxysanguinarine	Peroxisome proliferator-activated receptor delta	Q03181	PPARD	0.115
Ramulus Mori(RM)	MOL000729	Oxysanguinarine	Mineralocorticoid receptor	P08235	NR3C2	0.124
Ramulus Mori(RM)	MOL000729	Oxysanguinarine	Type-1 angiotensin II receptor	P30556	AGTR1	0.127
Ramulus Mori(RM)	MOL000729	Oxysanguinarine	Estradiol 17-beta-dehydrogenase 1	P14061	HSD17B1	0.129
Ramulus Mori(RM)	MOL000729	Oxysanguinarine	Cell division protein kinase 5	Q00535	CDK5	0.131
Ramulus Mori(RM)	MOL000729	Oxysanguinarine	Hemoglobin subunit alpha	P69905	HBA1	0.147
Ramulus Mori(RM)	MOL000729	Oxysanguinarine	Prothrombin	P00734	F2	0.149
Ramulus Mori(RM)	MOL000729	Oxysanguinarine	5-hydroxytryptamine 1A receptor	P08908	HTR1A	0.151
Ramulus Mori(RM)	MOL000729	Oxysanguinarine	Androgen receptor	P10275	AR	0.167
Ramulus Mori(RM)	MOL000729	Oxysanguinarine	D(1A) dopamine receptor	P21728	DRD1	0.172
Ramulus Mori(RM)	MOL000729	Oxysanguinarine	5-hydroxytryptamine 2A receptor	P28223	HTR2A	0.172
Ramulus Mori(RM)	MOL000729	Oxysanguinarine	Alpha-1A adrenergic receptor	P35348	ADRA1A	0.172
Ramulus Mori(RM)	MOL000729	Oxysanguinarine	Trypsin-1	P07477	PRSS1	0.196

Ramulus Mori(RM)	MOL000729	Oxysanguinarine	Nitric oxide synthase, inducible	P35228	NOS2	0.202
Ramulus Mori(RM)	MOL000729	Oxysanguinarine	D(2) dopamine receptor	P14416	DRD2	0.204
Ramulus Mori(RM)	MOL000729	Oxysanguinarine	Prostaglandin G/H synthase 1	P23219	PTGS1	0.237
Ramulus Mori(RM)	MOL000729	Oxysanguinarine	Nuclear receptor coactivator 1	Q15788	NCOA1	0.268
Ramulus Mori(RM)	MOL000729	Oxysanguinarine	Cell division protein kinase 2	P24941	CDK2	0.278
Ramulus Mori(RM)	MOL000729	Oxysanguinarine	Estrogen receptor beta	Q92731	ESR2	0.391
Ramulus Mori(RM)	MOL000729	Oxysanguinarine	Progesterone receptor	P06401	PGR	0.519
Ramulus Mori(RM)	MOL000729	Oxysanguinarine	cAMP-dependent protein kinase catalytic subunit alpha	P17612	PRKACA	0.578
Ramulus Mori(RM)	MOL000729	Oxysanguinarine	Prostaglandin G/H synthase 2	P35354	PTGS2	0.606
Ramulus Mori(RM)	MOL000729	Oxysanguinarine	Cyclin-A2	P20248	CCNA2	0.83
Ramulus Mori(RM)	MOL000729	Oxysanguinarine	Estrogen receptor	P03372	ESR1	1
Ramulus Mori(RM)	MOL000737	morin	D1 dopamine receptor-interacting protein calcyon	Q9NYX4	CALY	0.01
Ramulus Mori(RM)	MOL000737	morin	Sodium/hydrogen exchanger 1	P19634	SLC9A1	0.011
Ramulus Mori(RM)	MOL000737	morin	Amiloride-sensitive amine oxidase [copper-containing]	P19801	AOC1	0.011
Ramulus Mori(RM)	MOL000737	morin	Glutamate receptor 2	P42262	GRIA2	0.011
Ramulus Mori(RM)	MOL000737	morin	Amiloride-sensitive sodium channel subunit delta	P51172	SCNN1D	0.011
Ramulus Mori(RM)	MOL000737	morin	Amiloride-sensitive cation channel 2, neuronal	P78348	ASIC1	0.011
Ramulus Mori(RM)	MOL000737	morin	Amiloride-sensitive cation channel 1, neuronal	Q16515	ASIC2	0.011
Ramulus Mori(RM)	MOL000737	morin	Phenylalanine-4-hydroxylase	P00439	PAH	0.012
Ramulus Mori(RM)	MOL000737	morin	2,4-dienoyl-CoA reductase, mitochondrial	Q16698	DECR1	0.013
Ramulus Mori(RM)	MOL000737	morin	Potassium channel subfamily K member 1	O00180	KCNK1	0.014
Ramulus Mori(RM)	MOL000737	morin	Alcohol dehydrogenase 1C	P00326	ADH1C	0.014
Ramulus Mori(RM)	MOL000737	morin	Cytochrome c oxidase subunit 1	P00395	MT-CO1	0.014
Ramulus Mori(RM)	MOL000737	morin	Cytochrome c oxidase subunit 2	P00403	MT-CO2	0.014
Ramulus Mori(RM)	MOL000737	morin	Cytochrome c oxidase subunit 3	P00414	MT-CO3	0.014
Ramulus Mori(RM)	MOL000737	morin	Cytochrome c oxidase subunit 6C	P09669	COX6C	0.014
Ramulus Mori(RM)	MOL000737	morin	Cytochrome c oxidase subunit 8A, mitochondrial	P10176	COX8A	0.014
Ramulus Mori(RM)	MOL000737	morin	Cytochrome c oxidase subunit 5B, mitochondrial	P10606	COX5B	0.014
Ramulus Mori(RM)	MOL000737	morin	Cytochrome c oxidase subunit 4 isoform 1, mitochondrial	P13073	COX4I1	0.014
Ramulus Mori(RM)	MOL000737	morin	Cytochrome c oxidase subunit 6B1	P14854	COX6B1	0.014
Ramulus Mori(RM)	MOL000737	morin	Cytochrome c oxidase subunit 7C, mitochondrial	P15954	COX7C	0.014
Ramulus Mori(RM)	MOL000737	morin	Ferrochelatase, mitochondrial	P22830	FECH	0.014
Ramulus Mori(RM)	MOL000737	morin	Liver carboxylesterase 1	P23141	CES1	0.014
Ramulus Mori(RM)	MOL000737	morin	Cytochrome c oxidase polypeptide 7A1, mitochondrial	P24310	COX7A1	0.014
Ramulus Mori(RM)	MOL000737	morin	Cytochrome c oxidase subunit 7B, mitochondrial	P24311	COX7B	0.014
Ramulus Mori(RM)	MOL000737	morin	Gastrotropin	P51161	FABP6	0.014
Ramulus Mori(RM)	MOL000737	morin	Cytochrome c oxidase subunit 6A2, mitochondrial	Q02221	COX6A2	0.014
Ramulus Mori(RM)	MOL000737	morin	Ferrochelatase	Q7KZA3	DKFZp686P18130	0.014
Ramulus Mori(RM)	MOL000737	morin	Elongation factor 2	P13639	EEF2	0.015
Ramulus Mori(RM)	MOL000737	morin	Poly [ADP-ribose] polymerase 3	Q9Y6F1	PARP3	0.015
Ramulus Mori(RM)	MOL000737	morin	Phosphatidylinositol 3-kinase regulatory subunit beta	O00459	PIK3R2	0.016
Ramulus Mori(RM)	MOL000737	morin	T-cell receptor alpha chain C region	P01848	TRAC	0.016
Ramulus Mori(RM)	MOL000737	morin	T-cell receptor beta chain C region	P01850	TRBC1	0.016
Ramulus Mori(RM)	MOL000737	morin	Trypsin-2	P07478	PRSS2	0.016
Ramulus Mori(RM)	MOL000737	morin	Beta-3 adrenergic receptor	P13945	ADRB3	0.016
Ramulus Mori(RM)	MOL000737	morin	Phosphatidylinositol 3-kinase regulatory subunit alpha	P27986	PIK3R1	0.016
Ramulus Mori(RM)	MOL000737	morin	Mitogen-activated protein kinase 1	P28482	MAPK1	0.016
Ramulus Mori(RM)	MOL000737	morin	Beta-2-microglobulin	P61769	B2M	0.016
Ramulus Mori(RM)	MOL000737	morin	Death-associated protein kinase 3	O43293	DAPK3	0.017
Ramulus Mori(RM)	MOL000737	morin	Tyrosine-protein kinase JAK2	O60674	JAK2	0.017
Ramulus Mori(RM)	MOL000737	morin	Tyrosine-protein kinase JAK1	P23458	JAK1	0.017
Ramulus Mori(RM)	MOL000737	morin	Tyrosine-protein kinase JAK3	P52333	JAK3	0.017
Ramulus Mori(RM)	MOL000737	morin	Carbonic anhydrase 4	P22748	CA4	0.018

Ramulus Mori(RM)	MOL000737	morin	Muscarinic acetylcholine receptor M5	P08912	CHRM5	0.019
Ramulus Mori(RM)	MOL000737	morin	Sucrase-isomaltase, intestinal	P14410	SI	0.019
Ramulus Mori(RM)	MOL000737	morin	Histamine H1 receptor	P35367	HRH1	0.019
Ramulus Mori(RM)	MOL000737	morin	Pepsin A	P00790	REN	0.021
Ramulus Mori(RM)	MOL000737	morin	Dipeptidyl peptidase 4	P27487	DPP4	0.021
Ramulus Mori(RM)	MOL000737	morin	Interleukin-3	P08700	IL3	0.022
Ramulus Mori(RM)	MOL000737	morin	Gamma-aminobutyric-acid receptor subunit alpha-4	P48169	GABRA4	0.022
Ramulus Mori(RM)	MOL000737	morin	Protein S100-A12	P80511	S100A12	0.022
Ramulus Mori(RM)	MOL000737	morin	Gamma-aminobutyric-acid receptor subunit alpha-6	Q16445	GABRA6	0.022
Ramulus Mori(RM)	MOL000737	morin	Protein S100-A13	Q99584	S100A13	0.022
Ramulus Mori(RM)	MOL000737	morin	Solute carrier family 22 member 6	Q4U2R8	SLC22A6	0.023
Ramulus Mori(RM)	MOL000737	morin	Solute carrier family 22 member 8	Q8TCC7	SLC22A8	0.023
Ramulus Mori(RM)	MOL000737	morin	Solute carrier family 22 member 11	Q9NSA0	SLC22A11	0.023
Ramulus Mori(RM)	MOL000737	morin	Serine/threonine-protein kinase 17B	O94768	STK17B	0.024
Ramulus Mori(RM)	MOL000737	morin	Proto-oncogene serine/threonine-protein kinase Pim-1	P11309	PIM1	0.024
Ramulus Mori(RM)	MOL000737	morin	ATP synthase subunit gamma, mitochondrial	P36542	ATP5F1C	0.024
Ramulus Mori(RM)	MOL000737	morin	UDP-glucuronosyltransferase 3A1	Q6NUS8	UGT3A1	0.024
Ramulus Mori(RM)	MOL000737	morin	Tyrosine 3-monooxygenase	P07101	TH	0.025
Ramulus Mori(RM)	MOL000737	morin	5-hydroxytryptamine 3 receptor	P46098	HTR3A	0.025
Ramulus Mori(RM)	MOL000737	morin	Peptidyl-prolyl cis-trans isomerase NIMA-interacting 1	Q13526	PIN1	0.025
Ramulus Mori(RM)	MOL000737	morin	Tyrosyl-tRNA synthetase, mitochondrial	Q9Y2Z4	YARS2	0.025
Ramulus Mori(RM)	MOL000737	morin	Sodium channel protein type 10 subunit alpha	Q9Y5Y9	SCN10A	0.025
Ramulus Mori(RM)	MOL000737	morin	Cell division protein kinase 4	P11802	CDK4	0.026
Ramulus Mori(RM)	MOL000737	morin	Substance-P receptor	P25103	TACR1	0.026
Ramulus Mori(RM)	MOL000737	morin	Amiloride-sensitive sodium channel subunit alpha	P37088	SCNN1A	0.026
Ramulus Mori(RM)	MOL000737	morin	Cell division protein kinase 7	P50613	CDK7	0.026
Ramulus Mori(RM)	MOL000737	morin	Cell division protein kinase 9	P50750	CDK9	0.026
Ramulus Mori(RM)	MOL000737	morin	Amiloride-sensitive sodium channel subunit beta	P51168	SCNN1B	0.026
Ramulus Mori(RM)	MOL000737	morin	Amiloride-sensitive sodium channel subunit gamma	P51170	SCNN1G	0.026
Ramulus Mori(RM)	MOL000737	morin	Potassium voltage-gated channel subfamily H member 2	Q12809	KCNH2	0.027
Ramulus Mori(RM)	MOL000737	morin	Potassium voltage-gated channel subfamily H member 6	Q9H252	KCNH6	0.027
Ramulus Mori(RM)	MOL000737	morin	Gamma-aminobutyric-acid receptor subunit beta-3	P28472	GABRB3	0.028
Ramulus Mori(RM)	MOL000737	morin	Gamma-aminobutyric-acid receptor subunit beta-2	P47870	GABRB2	0.028
Ramulus Mori(RM)	MOL000737	morin	Prostaglandin reductase 1	Q14914	PTGR1	0.028
Ramulus Mori(RM)	MOL000737	morin	Gamma-aminobutyric acid receptor subunit theta	Q9UN88	GABRQ	0.028
Ramulus Mori(RM)	MOL000737	morin	Carbonic anhydrase 1	P00915	CA1	0.029
Ramulus Mori(RM)	MOL000737	morin	Cyclin-dependent kinase 5 activator 1	Q15078	CDK5R1	0.029
Ramulus Mori(RM)	MOL000737	morin	Tripartite motif-containing protein 13	O60858	TRIM13	0.03
Ramulus Mori(RM)	MOL000737	morin	cAMP response element-binding protein	P16220	CREB1	0.03
Ramulus Mori(RM)	MOL000737	morin	Nociceptin receptor	P41146	OPRL1	0.03
Ramulus Mori(RM)	MOL000737	morin	cGMP-inhibited 3',5'-cyclic phosphodiesterase A	Q14432	PDE3A	0.033
Ramulus Mori(RM)	MOL000737	morin	cAMP and cAMP-inhibited cGMP 3',5'-cyclic phosphodiesterase 10A	Q9Y233	PDE10A	0.033
Ramulus Mori(RM)	MOL000737	morin	cGMP-specific 3',5'-cyclic phosphodiesterase	O76074	PDE5A	0.034
Ramulus Mori(RM)	MOL000737	morin	DNA topoisomerase 1	P11387	TOP1	0.034
Ramulus Mori(RM)	MOL000737	morin	Adenosine A2a receptor	P29274	ADORA2A	0.034
Ramulus Mori(RM)	MOL000737	morin	Adenosine A1 receptor	P30542	ADORA1	0.034
Ramulus Mori(RM)	MOL000737	morin	DNA topoisomerase I, mitochondrial	Q969P6	TOP1MT	0.034
Ramulus Mori(RM)	MOL000737	morin	Inhibitor of nuclear factor kappa-B kinase subunit beta	O14920	IKBKB	0.035
Ramulus Mori(RM)	MOL000737	morin	Casein kinase I isoform gamma-2	P78368	CSNK1G2	0.035
Ramulus Mori(RM)	MOL000737	morin	cAMP-specific 3',5'-cyclic phosphodiesterase 4C	Q08493	PDE4C	0.035
Ramulus Mori(RM)	MOL000737	morin	Sodium channel protein type 5 subunit alpha	Q14524	SCN5A	0.035
Ramulus Mori(RM)	MOL000737	morin	Serine/threonine-protein kinase haspin	Q8TF76	HASPIN	0.035
Ramulus Mori(RM)	MOL000737	morin	Cystine/glutamate transporter	Q9UPY5	SLC7A11	0.035

Ramulus Mori(RM)	MOL000737	morin	Thymidine kinase 2, mitochondrial	O00142	TK2	0.036
Ramulus Mori(RM)	MOL000737	morin	Succinate dehydrogenase [ubiquinone] cytochrome b small subunit, mitochondrial	O14521	SDHD	0.036
Ramulus Mori(RM)	MOL000737	morin	Ribosyldihydronicotinamide dehydrogenase [quinone]	P16083	NQO2	0.036
Ramulus Mori(RM)	MOL000737	morin	Chymase	P23946	CMA1	0.036
Ramulus Mori(RM)	MOL000737	morin	Ig kappa chain C region	P01834	IGKC	0.037
Ramulus Mori(RM)	MOL000737	morin	Ig gamma-1 chain C region	P01857	IGHG1	0.037
Ramulus Mori(RM)	MOL000737	morin	Peptidyl-prolyl cis-trans isomerase, mitochondrial	P30405	PPIF	0.037
Ramulus Mori(RM)	MOL000737	morin	Mitogen-activated protein kinase 8	P45983	MAPK8	0.037
Ramulus Mori(RM)	MOL000737	morin	Mitogen-activated protein kinase 10	P53779	MAPK10	0.037
Ramulus Mori(RM)	MOL000737	morin	Tumor necrosis factor	P01375	TNF	0.039
Ramulus Mori(RM)	MOL000737	morin	Nuclear factor NF-kappa-B p105 subunit	P19838	NFKB1	0.039
Ramulus Mori(RM)	MOL000737	morin	Carbonic anhydrase 2	P00918	CA2	0.04
Ramulus Mori(RM)	MOL000737	morin	Ribonucleoside-diphosphate reductase large subunit	P23921	RRM1	0.04
Ramulus Mori(RM)	MOL000737	morin	Proto-oncogene tyrosine-protein kinase LCK	P06239	LCK	0.041
Ramulus Mori(RM)	MOL000737	morin	Tyrosine-protein kinase Lyn	P07948	LYN	0.041
Ramulus Mori(RM)	MOL000737	morin	Retinoic acid receptor RXR-beta	P28702	RXRB	0.043
Ramulus Mori(RM)	MOL000737	morin	Oxysterols receptor LXR-beta	P55055	NR1H2	0.043
Ramulus Mori(RM)	MOL000737	morin	Oxysterols receptor LXR-alpha	Q13133	NR1H3	0.043
Ramulus Mori(RM)	MOL000737	morin	Phospholipase A2	P04054	PLA2G1B	0.045
Ramulus Mori(RM)	MOL000737	morin	Sterol O-acyltransferase 2	O75908	SOAT2	0.046
Ramulus Mori(RM)	MOL000737	morin	Macrophage migration inhibitory factor	P14174	MIF	0.046
Ramulus Mori(RM)	MOL000737	morin	Sterol O-acyltransferase 1	P35610	SOAT1	0.046
Ramulus Mori(RM)	MOL000737	morin	Urokinase-type plasminogen activator	P00749	PLAU	0.047
Ramulus Mori(RM)	MOL000737	morin	Gamma-aminobutyric acid receptor subunit gamma-2	P18507	GABRG2	0.047
Ramulus Mori(RM)	MOL000737	morin	Tryptophanyl-tRNA synthetase, mitochondrial	Q9UGM6	WARS2	0.047
Ramulus Mori(RM)	MOL000737	morin	Glycogen phosphorylase, muscle form	P11217	PYGM	0.048
Ramulus Mori(RM)	MOL000737	morin	Beta-1 adrenergic receptor	P08588	ADRB1	0.049
Ramulus Mori(RM)	MOL000737	morin	Tubulin alpha-1 chain	P68366	TUBA4A	0.051
Ramulus Mori(RM)	MOL000737	morin	ATP synthase subunit beta, mitochondrial	P06576	ATP5F1B	0.052
Ramulus Mori(RM)	MOL000737	morin	Tyrosine-protein kinase HCK	P08631	HCK	0.052
Ramulus Mori(RM)	MOL000737	morin	ATP synthase subunit alpha, mitochondrial	P25705	ATP5F1A	0.052
Ramulus Mori(RM)	MOL000737	morin	Phosphatidylinositol-4,5-bisphosphate 3-kinase catalytic subunit gamma isoform	P48736	PIK3CG	0.052
Ramulus Mori(RM)	MOL000737	morin	Protein tyrosine kinase 2 beta	Q14289	PTK2B	0.052
Ramulus Mori(RM)	MOL000737	morin	Gamma-aminobutyric acid receptor subunit rho-3	A8MPY1	GABRR3	0.053
Ramulus Mori(RM)	MOL000737	morin	Gamma-aminobutyric acid receptor subunit pi	O00591	GABRP	0.053
Ramulus Mori(RM)	MOL000737	morin	Gamma-aminobutyric acid receptor subunit delta	O14764	GABRD	0.053
Ramulus Mori(RM)	MOL000737	morin	Platelet glycoprotein IX	P14770	GP9	0.053
Ramulus Mori(RM)	MOL000737	morin	Gamma-aminobutyric-acid receptor subunit beta-1	P18505	GABRB1	0.053
Ramulus Mori(RM)	MOL000737	morin	Gamma-aminobutyric-acid receptor subunit rho-1	P24046	GABRR1	0.053
Ramulus Mori(RM)	MOL000737	morin	Gamma-aminobutyric acid receptor subunit rho-2	P28476	GABRR2	0.053
Ramulus Mori(RM)	MOL000737	morin	Gamma-aminobutyric-acid receptor subunit alpha-5	P31644	GABRA5	0.053
Ramulus Mori(RM)	MOL000737	morin	Gamma-aminobutyric-acid receptor subunit alpha-3	P34903	GABRA3	0.053
Ramulus Mori(RM)	MOL000737	morin	Gamma-aminobutyric acid receptor subunit epsilon	P78334	GABRE	0.053
Ramulus Mori(RM)	MOL000737	morin	Gamma-aminobutyric acid receptor subunit gamma-1	Q8N1C3	GABRG1	0.053
Ramulus Mori(RM)	MOL000737	morin	Gamma-aminobutyric acid receptor subunit gamma-3	Q99928	GABRG3	0.053
Ramulus Mori(RM)	MOL000737	morin	Microtubule-associated protein 2	P11137	MAP2	0.054
Ramulus Mori(RM)	MOL000737	morin	3 beta-hydroxysteroid dehydrogenase/Delta 5-->4-isomerase type I	P14060	HSD3B1	0.054
Ramulus Mori(RM)	MOL000737	morin	3 beta-hydroxysteroid dehydrogenase/Delta 5-->4-isomerase type II	P26439	HSD3B2	0.054
Ramulus Mori(RM)	MOL000737	morin	Microtubule-associated protein 1A	P78559	MAP1A	0.054
Ramulus Mori(RM)	MOL000737	morin	Glutamate [NMDA] receptor subunit 3A	Q8TCU5	GRIN3A	0.054
Ramulus Mori(RM)	MOL000737	morin	Alpha-1D adrenergic receptor	P25100	ADRA1D	0.056
Ramulus Mori(RM)	MOL000737	morin	Epidermal growth factor receptor	P00533	EGFR	0.057

Ramulus Mori(RM)	MOL000737	morin	Cell division protein kinase 6	Q00534	CDK6	0.057
Ramulus Mori(RM)	MOL000737	morin	Estrogen-related receptor gamma	P62508	ESRRG	0.058
Ramulus Mori(RM)	MOL000737	morin	Gamma-aminobutyric-acid receptor subunit alpha-2	P47869	GABRA2	0.061
Ramulus Mori(RM)	MOL000737	morin	5-hydroxytryptamine 2A receptor	P28223	HTR2A	0.062
Ramulus Mori(RM)	MOL000737	morin	Glycogen synthase kinase-3 beta	P49841	GSK3B	0.062
Ramulus Mori(RM)	MOL000737	morin	D(4) dopamine receptor	P21917	DRD4	0.065
Ramulus Mori(RM)	MOL000737	morin	5-hydroxytryptamine 1B receptor	P28222	HTR1B	0.065
Ramulus Mori(RM)	MOL000737	morin	5-hydroxytryptamine 2B receptor	P41595	HTR2B	0.065
Ramulus Mori(RM)	MOL000737	morin	Sodium-dependent serotonin transporter	P31645	SLC6A4	0.066
Ramulus Mori(RM)	MOL000737	morin	Beta-2 adrenergic receptor	P07550	ADRB2	0.067
Ramulus Mori(RM)	MOL000737	morin	Serine/threonine-protein kinase 6	O14965	AURKA	0.071
Ramulus Mori(RM)	MOL000737	morin	Cytochrome P450 19A1	P11511	CYP19A1	0.071
Ramulus Mori(RM)	MOL000737	morin	Alpha-1B adrenergic receptor	P35368	ADRA1B	0.071
Ramulus Mori(RM)	MOL000737	morin	Thromboxane A2 receptor	P21731	TBXA2R	0.072
Ramulus Mori(RM)	MOL000737	morin	cAMP-dependent protein kinase inhibitor alpha	P61925	PKIA	0.072
Ramulus Mori(RM)	MOL000737	morin	Egl nine homolog 1	Q9GZT9	EGLN1	0.072
Ramulus Mori(RM)	MOL000737	morin	Thyroid hormone receptor alpha	P10827	THRA	0.073
Ramulus Mori(RM)	MOL000737	morin	Triosephosphate isomerase	P60174	TPI1	0.073
Ramulus Mori(RM)	MOL000737	morin	Peroxisome proliferator-activated receptor alpha	Q07869	PPARA	0.073
Ramulus Mori(RM)	MOL000737	morin	Group IIE secretory phospholipase A2	Q9NZK7	PLA2G2E	0.073
Ramulus Mori(RM)	MOL000737	morin	Gamma-aminobutyric-acid receptor subunit alpha-1	P14867	GABRA1	0.074
Ramulus Mori(RM)	MOL000737	morin	Glucocorticoid receptor	P04150	NR3C1	0.075
Ramulus Mori(RM)	MOL000737	morin	Dihydrofolate reductase	P00374	DHFR	0.076
Ramulus Mori(RM)	MOL000737	morin	Androgen receptor	P10275	AR	0.076
Ramulus Mori(RM)	MOL000737	morin	Tyrosine-protein phosphatase non-receptor type 1	P18031	PTPN1	0.076
Ramulus Mori(RM)	MOL000737	morin	Membrane copper amine oxidase	Q16853	AOC3	0.076
Ramulus Mori(RM)	MOL000737	morin	Hydroxyacid oxidase 1	Q9UJM8	HAO1	0.076
Ramulus Mori(RM)	MOL000737	morin	Casein kinase II subunit alpha	P68400	CSNK2A1	0.077
Ramulus Mori(RM)	MOL000737	morin	Sodium-dependent dopamine transporter	Q01959	SLC6A3	0.077
Ramulus Mori(RM)	MOL000737	morin	S-methyl-5-thioadenosine phosphorylase	Q13126	MTAP	0.077
Ramulus Mori(RM)	MOL000737	morin	Toll-like receptor 7	Q9NYK1	TLR7	0.077
Ramulus Mori(RM)	MOL000737	morin	Leukotriene A-4 hydrolase	P09960	LTA4H	0.078
Ramulus Mori(RM)	MOL000737	morin	D(1B) dopamine receptor	P21918	DRD5	0.078
Ramulus Mori(RM)	MOL000737	morin	5-hydroxytryptamine 1D receptor	P28221	HTR1D	0.078
Ramulus Mori(RM)	MOL000737	morin	D(3) dopamine receptor	P35462	DRD3	0.078
Ramulus Mori(RM)	MOL000737	morin	Mitogen-activated protein kinase 14	Q16539	MAPK14	0.078
Ramulus Mori(RM)	MOL000737	morin	C-jun-amino-terminal kinase-interacting protein 1	Q9UQF2	MAPK8IP1	0.078
Ramulus Mori(RM)	MOL000737	morin	Calcium/calmodulin-dependent protein kinase type II alpha chain	Q9UQM7	CAMK2A	0.078
Ramulus Mori(RM)	MOL000737	morin	Neuropeptide Y	P01303	NPY	0.079
Ramulus Mori(RM)	MOL000737	morin	5-hydroxytryptamine 1A receptor	P08908	HTR1A	0.079
Ramulus Mori(RM)	MOL000737	morin	5-hydroxytryptamine 2C receptor	P28335	HTR2C	0.079
Ramulus Mori(RM)	MOL000737	morin	Calmodulin	P62158		0.079
Ramulus Mori(RM)	MOL000737	morin	Alpha-2A adrenergic receptor	P08913	ADRA2A	0.08
Ramulus Mori(RM)	MOL000737	morin	Tyrosyl-tRNA synthetase, cytoplasmic	P54577	YARS	0.08
Ramulus Mori(RM)	MOL000737	morin	Solute carrier family 12 member 1	Q13621	SLC12A1	0.08
Ramulus Mori(RM)	MOL000737	morin	Fibroblast growth factor receptor 2	P21802	FGFR2	0.081
Ramulus Mori(RM)	MOL000737	morin	Tubulin alpha-3 chain	Q71U36	TUBA1A	0.081
Ramulus Mori(RM)	MOL000737	morin	Thymidylate synthase	P04818	TYMS	0.083
Ramulus Mori(RM)	MOL000737	morin	Acetylcholinesterase	P22303	ACHE	0.083
Ramulus Mori(RM)	MOL000737	morin	Nuclear receptor coactivator 5	Q9HCD5	NCOA5	0.083
Ramulus Mori(RM)	MOL000737	morin	Nuclear receptor coactivator 2	Q15596	NCOA2	0.084
Ramulus Mori(RM)	MOL000737	morin	Muscarinic acetylcholine receptor M4	P08173	CHRM4	0.086
Ramulus Mori(RM)	MOL000737	morin	Alpha-1A adrenergic receptor	P35348	ADRA1A	0.086

Ramulus Mori(RM)	MOL000737	morin	Interferon gamma	P01579	IFNG	0.087
Ramulus Mori(RM)	MOL000737	morin	Proto-oncogene tyrosine-protein kinase Src	P12931	SRC	0.09
Ramulus Mori(RM)	MOL000737	morin	DNA polymerase kappa	Q9UBT6	POLK	0.091
Ramulus Mori(RM)	MOL000737	morin	D(1A) dopamine receptor	P21728	DRD1	0.092
Ramulus Mori(RM)	MOL000737	morin	D(2) dopamine receptor	P14416	DRD2	0.093
Ramulus Mori(RM)	MOL000737	morin	Alpha-2B adrenergic receptor	P18089	ADRA2B	0.094
Ramulus Mori(RM)	MOL000737	morin	Alpha-2C adrenergic receptor	P18825	ADRA2C	0.094
Ramulus Mori(RM)	MOL000737	morin	Sodium-dependent noradrenaline transporter	P23975	SLC6A2	0.095
Ramulus Mori(RM)	MOL000737	morin	DNA topoisomerase 2-alpha	P11388	TOP2A	0.107
Ramulus Mori(RM)	MOL000737	morin	Muscarinic acetylcholine receptor M2	P08172	CHRM2	0.109
Ramulus Mori(RM)	MOL000737	morin	Muscarinic acetylcholine receptor M3	P20309	CHRM3	0.109
Ramulus Mori(RM)	MOL000737	morin	Inhibitor of nuclear factor kappa-B kinase subunit alpha	O15111	CHUK	0.113
Ramulus Mori(RM)	MOL000737	morin	Arachidonate 5-lipoxygenase	P09917	ALOX5	0.113
Ramulus Mori(RM)	MOL000737	morin	cAMP-specific 3',5'-cyclic phosphodiesterase 4D	Q08499	PDE4D	0.114
Ramulus Mori(RM)	MOL000737	morin	D-HSCDK2	O75100	CA11	0.123
Ramulus Mori(RM)	MOL000737	morin	cAMP-specific 3',5'-cyclic phosphodiesterase 4A	P27815	PDE4A	0.146
Ramulus Mori(RM)	MOL000737	morin	Nitric oxide synthase, inducible	P35228	NOS2	0.148
Ramulus Mori(RM)	MOL000737	morin	cAMP-specific 3',5'-cyclic phosphodiesterase 4B	Q07343	PDE4B	0.148
Ramulus Mori(RM)	MOL000737	morin	Muscarinic acetylcholine receptor M1	P11229	CHRM1	0.154
Ramulus Mori(RM)	MOL000737	morin	cAMP-dependent protein kinase catalytic subunit alpha	P17612	PRKACA	0.155
Ramulus Mori(RM)	MOL000737	morin	Peroxisome proliferator-activated receptor gamma	P37231	PPARG	0.155
Ramulus Mori(RM)	MOL000737	morin	Cell division control protein 2 homolog	P06493	CDK1	0.156
Ramulus Mori(RM)	MOL000737	morin	Delta-type opioid receptor	P41143	OPRD1	0.166
Ramulus Mori(RM)	MOL000737	morin	Kappa-type opioid receptor	P41145	OPRK1	0.166
Ramulus Mori(RM)	MOL000737	morin	RAC-alpha serine/threonine-protein kinase	P31749	AKT1	0.167
Ramulus Mori(RM)	MOL000737	morin	Hemoglobin subunit alpha	P69905	HBA1	0.179
Ramulus Mori(RM)	MOL000737	morin	Cell division protein kinase 5	Q00535	CDK5	0.188
Ramulus Mori(RM)	MOL000737	morin	MAP kinase-activated protein kinase 2	P49137	MAPKAPK2	0.237
Ramulus Mori(RM)	MOL000737	morin	Cell division protein kinase 2	P24941	CDK2	0.256
Ramulus Mori(RM)	MOL000737	morin	Nuclear receptor coactivator 1	Q15788	NCOA1	0.258
Ramulus Mori(RM)	MOL000737	morin	Mu-type opioid receptor	P35372	OPRM1	0.268
Ramulus Mori(RM)	MOL000737	morin	Prostaglandin G/H synthase 1	P23219	PTGS1	0.386
Ramulus Mori(RM)	MOL000737	morin	Prothrombin	P00734	F2	0.392
Ramulus Mori(RM)	MOL000737	morin	Trypsin-1	P07477	PRSS1	0.398
Ramulus Mori(RM)	MOL000737	morin	Estrogen receptor beta	Q92731	ESR2	0.514
Ramulus Mori(RM)	MOL000737	morin	Cyclin-A2	P20248	CCNA2	0.534
Ramulus Mori(RM)	MOL000737	morin	Prostaglandin G/H synthase 2	P35354	PTGS2	0.68
Ramulus Mori(RM)	MOL000737	morin	Estrogen receptor	P03372	ESR1	1
Ramulus Mori(RM)	MOL000737	morin	Arachidonate 5-lipoxygenase	P09917	ALOX5	Validated
Ramulus Mori(RM)	MOL000737	morin	Xanthine dehydrogenase/oxidase	P47989	XDH	Validated
Ramulus Mori(RM)	MOL000737	morin	Phosphatidylinositol-5-phosphate 4-kinase type-2 alpha	P48426	PIP4K2A	Validated
Ramulus Mori(RM)	MOL000737	morin	Glutathione reductase, mitochondrial	P00390	GSR	Validated
Ramulus Mori(RM)	MOL000737	morin	Endothelin-1	P05305	EDN1	Validated
Ramulus Mori(RM)	MOL000737	morin	Multidrug resistance protein 1	P08183	ABCB1	Validated
Ramulus Mori(RM)	MOL000737	morin	DNA topoisomerase 1	P11387	TOP1	Validated
Ramulus Mori(RM)	MOL000737	morin	Platelet glycoprotein 4	P16671	D36	Validated
Ramulus Mori(RM)	MOL000737	morin	Type I iodothyronine deiodinase	P49895	DIO1	Validated
Ramulus Mori(RM)	MOL000737	morin	Basic leucine zipper transcriptional factor ATF-like 3	Q9NR55	BATF3	Validated
Ramulus Mori(RM)	MOL000738	kuwanon c	Tyrosinase	P14679	TYR	Validated
Ramulus Mori(RM)	MOL001788	adenine	Putative adenosylhomocysteinase 2	O43865	AHCYL1	Validated
Ramulus Mori(RM)	MOL002212	ecdysterone	Superoxide dismutase [Cu-Zn]	P00441	SOD1	Validated
Ramulus Mori(RM)	MOL005570	myoinositol	Inositol oxygenase	Q9UGB7	MIOX	Validated
Silybum marianum(SM)	MOL000098	quercetin	Adenosine A2b receptor	P29275	ADORA2B	0.011

Silybum marianum(SM)	MOL000098	quercetin	Adenosine A3 receptor	P33765	SHMT1	0.011
Silybum marianum(SM)	MOL000098	quercetin	cGMP-specific 3',5'-cyclic phosphodiesterase	O76074	PDE5A	0.012
Silybum marianum(SM)	MOL000098	quercetin	Adenosine A1 receptor	P30542	ADORA1	0.012
Silybum marianum(SM)	MOL000098	quercetin	cAMP-specific 3',5'-cyclic phosphodiesterase 4B	Q07343	PDE4B	0.012
Silybum marianum(SM)	MOL000098	quercetin	Neuronal acetylcholine receptor subunit alpha-2	Q15822	CHRNA2	0.012
Silybum marianum(SM)	MOL000098	quercetin	Potassium channel subfamily K member 1	O00180	KCNK1	0.013
Silybum marianum(SM)	MOL000098	quercetin	Tyrosine 3-monooxygenase	P07101	TH	0.013
Silybum marianum(SM)	MOL000098	quercetin	cGMP-inhibited 3',5'-cyclic phosphodiesterase A	Q14432	PDE3A	0.013
Silybum marianum(SM)	MOL000098	quercetin	cAMP and cAMP-inhibited cGMP 3',5'-cyclic phosphodiesterase 10A	Q9Y233	PDE10A	0.013
Silybum marianum(SM)	MOL000098	quercetin	Tyrosyl-tRNA synthetase, mitochondrial	Q9Y2Z4	YARS2	0.013
Silybum marianum(SM)	MOL000098	quercetin	3-phosphoinositide-dependent protein kinase 1	O15530	PDPK1	0.014
Silybum marianum(SM)	MOL000098	quercetin	Serine/threonine-protein kinase 17B	O94768	STK17B	0.014
Silybum marianum(SM)	MOL000098	quercetin	Pepsin A	P00790	REN	0.014
Silybum marianum(SM)	MOL000098	quercetin	Tyrosine-protein kinase Lyn	P07948	LYN	0.014
Silybum marianum(SM)	MOL000098	quercetin	Muscarinic acetylcholine receptor M5	P08912	CHRM5	0.014
Silybum marianum(SM)	MOL000098	quercetin	Cell division protein kinase 4	P11802	CDK4	0.014
Silybum marianum(SM)	MOL000098	quercetin	Gamma-aminobutyric-acid receptor subunit alpha-1	P14867	GABRA1	0.014
Silybum marianum(SM)	MOL000098	quercetin	Dipeptidyl peptidase 4	P27487	DPP4	0.014
Silybum marianum(SM)	MOL000098	quercetin	ATP synthase subunit gamma, mitochondrial	P36542	ATP5F1C	0.014
Silybum marianum(SM)	MOL000098	quercetin	Tyrosine-protein kinase CSK	P41240	CSK	0.014
Silybum marianum(SM)	MOL000098	quercetin	Tyrosine-protein kinase ZAP-70	P43403	ZAP70	0.014
Silybum marianum(SM)	MOL000098	quercetin	Tyrosine-protein kinase SYK	P43405	SYK	0.014
Silybum marianum(SM)	MOL000098	quercetin	Gamma-aminobutyric-acid receptor subunit alpha-2	P47869	GABRA2	0.014
Silybum marianum(SM)	MOL000098	quercetin	Cell division protein kinase 7	P50613	CDK7	0.014
Silybum marianum(SM)	MOL000098	quercetin	Cell division protein kinase 9	P50750	CDK9	0.014
Silybum marianum(SM)	MOL000098	quercetin	Cell division protein kinase 6	Q00534	CDK6	0.014
Silybum marianum(SM)	MOL000098	quercetin	Protein kinase C theta type	Q04759	PRKCQ	0.014
Silybum marianum(SM)	MOL000098	quercetin	Tyrosine-protein kinase ITK/TSK	Q08881	ITK	0.014
Silybum marianum(SM)	MOL000098	quercetin	Cyclin-dependent kinase 5 activator 1	Q15078	CDK5R1	0.014
Silybum marianum(SM)	MOL000098	quercetin	UDP-glucuronosyltransferase 3A1	Q6NUS8	UGT3A1	0.014
Silybum marianum(SM)	MOL000098	quercetin	Dehydrogenase/reductase SDR family member 8	Q8NBQ5	HSD17B11	0.014
Silybum marianum(SM)	MOL000098	quercetin	85 kDa calcium-independent phospholipase A2	O60733	PLA2G6	0.015
Silybum marianum(SM)	MOL000098	quercetin	Ig gamma-2 chain C region	P01859	IGHG2	0.015
Silybum marianum(SM)	MOL000098	quercetin	Retinoic acid receptor alpha	P10276	RARA	0.015
Silybum marianum(SM)	MOL000098	quercetin	Retinoic acid receptor beta	P10826	RARB	0.015
Silybum marianum(SM)	MOL000098	quercetin	Retinoic acid receptor gamma-1	P13631	RARG	0.015
Silybum marianum(SM)	MOL000098	quercetin	Beta-3 adrenergic receptor	P13945	ADRB3	0.015
Silybum marianum(SM)	MOL000098	quercetin	5-hydroxytryptamine 1D receptor	P28221	HTR1D	0.015
Silybum marianum(SM)	MOL000098	quercetin	5-hydroxytryptamine 1B receptor	P28222	HTR1B	0.015
Silybum marianum(SM)	MOL000098	quercetin	5-hydroxytryptamine 2C receptor	P28335	HTR2C	0.015
Silybum marianum(SM)	MOL000098	quercetin	Amiloride-sensitive sodium channel subunit alpha	P37088	SCNN1A	0.015
Silybum marianum(SM)	MOL000098	quercetin	Cytosolic phospholipase A2	P47712	PLA2G4A	0.015
Silybum marianum(SM)	MOL000098	quercetin	Retinoic acid receptor RXR-gamma	P48443	RXRG	0.015
Silybum marianum(SM)	MOL000098	quercetin	Amiloride-sensitive sodium channel subunit beta	P51168	SCNN1B	0.015
Silybum marianum(SM)	MOL000098	quercetin	Amiloride-sensitive sodium channel subunit gamma	P51170	SCNN1G	0.015
Silybum marianum(SM)	MOL000098	quercetin	Sodium/potassium-transporting ATPase gamma chain	P54710	FXYD2	0.015
Silybum marianum(SM)	MOL000098	quercetin	Solute carrier family 12 member 3	P55017	SLC12A3	0.015

Silybum marianum(SM)	MOL000098	quercetin	Calcium-activated potassium channel subunit alpha 1	Q12791	KCNMA1	0.015
Silybum marianum(SM)	MOL000098	quercetin	5-hydroxytryptamine 3 receptor	P46098	HTR3A	0.017
Silybum marianum(SM)	MOL000098	quercetin	Prostaglandin reductase 1	Q14914	PTGR1	0.017
Silybum marianum(SM)	MOL000098	quercetin	Glutamate [NMDA] receptor subunit 3A	Q8TCU5	GRIN3A	0.017
Silybum marianum(SM)	MOL000098	quercetin	DNA topoisomerase 1	P11387	TOP1	0.02
Silybum marianum(SM)	MOL000098	quercetin	Macrophage migration inhibitory factor	P14174	MIF	0.02
Silybum marianum(SM)	MOL000098	quercetin	Calcium/calmodulin-dependent protein kinase type II subunit delta	Q13557	CAMK2D	0.02
Silybum marianum(SM)	MOL000098	quercetin	DNA topoisomerase I, mitochondrial	Q969P6	TOP1MT	0.02
Silybum marianum(SM)	MOL000098	quercetin	STE20-like serine/threonine-protein kinase	Q9H2G2	SLK	0.02
Silybum marianum(SM)	MOL000098	quercetin	Tripartite motif-containing protein 13	O60858	TRIM13	0.021
Silybum marianum(SM)	MOL000098	quercetin	Chymase	P23946	CMA1	0.021
Silybum marianum(SM)	MOL000098	quercetin	DNA-(apurinic or apyrimidinic site) lyase	P27695	APEX1	0.021
Silybum marianum(SM)	MOL000098	quercetin	Succinate dehydrogenase [ubiquinone] cytochrome b small subunit, mitochondrial	O14521	SDHD	0.022
Silybum marianum(SM)	MOL000098	quercetin	Inhibitor of nuclear factor kappa-B kinase subunit beta	O14920	IKBKB	0.022
Silybum marianum(SM)	MOL000098	quercetin	Ribosyldihydronicotinamide dehydrogenase [quinone]	P16083	NQO2	0.022
Silybum marianum(SM)	MOL000098	quercetin	Peptidyl-prolyl cis-trans isomerase, mitochondrial	P30405	PPIF	0.022
Silybum marianum(SM)	MOL000098	quercetin	Prostacyclin receptor	P43119	PTGIR	0.022
Silybum marianum(SM)	MOL000098	quercetin	Mitogen-activated protein kinase 8	P45983	MAPK8	0.022
Silybum marianum(SM)	MOL000098	quercetin	Mitogen-activated protein kinase 10	P53779	MAPK10	0.022
Silybum marianum(SM)	MOL000098	quercetin	Peroxisome proliferator-activated receptor delta	Q03181	PPARD	0.022
Silybum marianum(SM)	MOL000098	quercetin	Group IIE secretory phospholipase A2	Q9NZK7	PLA2G2E	0.022
Silybum marianum(SM)	MOL000098	quercetin	Cystine/glutamate transporter	Q9UPY5	SLC7A11	0.022
Silybum marianum(SM)	MOL000098	quercetin	Oxysterols receptor LXR-beta	P55055	NR1H2	0.023
Silybum marianum(SM)	MOL000098	quercetin	Oxysterols receptor LXR-alpha	Q13133	NR1H3	0.023
Silybum marianum(SM)	MOL000098	quercetin	Sodium channel protein type 5 subunit alpha	Q14524	SCN5A	0.023
Silybum marianum(SM)	MOL000098	quercetin	Phenylalanine-4-hydroxylase	P00439	PAH	0.024
Silybum marianum(SM)	MOL000098	quercetin	Rho-associated protein kinase 1	Q13464	ROCK1	0.024
Silybum marianum(SM)	MOL000098	quercetin	Alpha-1D adrenergic receptor	P25100	ADRA1D	0.025
Silybum marianum(SM)	MOL000098	quercetin	D(4) dopamine receptor	P21917	DRD4	0.026
Silybum marianum(SM)	MOL000098	quercetin	Keratin, type II cytoskeletal 7	P08729	KRT7	0.028
Silybum marianum(SM)	MOL000098	quercetin	Platelet glycoprotein IX	P14770	GP9	0.028
Silybum marianum(SM)	MOL000098	quercetin	Adenosine A2a receptor	P29274	ADORA2A	0.029
Silybum marianum(SM)	MOL000098	quercetin	Sterol O-acyltransferase 2	O75908	SOAT2	0.031
Silybum marianum(SM)	MOL000098	quercetin	Sterol O-acyltransferase 1	P35610	SOAT1	0.031
Silybum marianum(SM)	MOL000098	quercetin	Tubulin beta-2C chain	P68371	TUBB4B	0.031
Silybum marianum(SM)	MOL000098	quercetin	3 beta-hydroxysteroid dehydrogenase/Delta 5-->4-isomerase type II	P26439	HSD3B2	0.032
Silybum marianum(SM)	MOL000098	quercetin	Protein tyrosine kinase 2 beta	Q14289	PTK2B	0.032
Silybum marianum(SM)	MOL000098	quercetin	Tyrosyl-tRNA synthetase, cytoplasmic	P54577	YARS	0.033
Silybum marianum(SM)	MOL000098	quercetin	ATP synthase subunit beta, mitochondrial	P06576	ATP5F1B	0.034
Silybum marianum(SM)	MOL000098	quercetin	Muscarinic acetylcholine receptor M4	P08173	CHRM4	0.034
Silybum marianum(SM)	MOL000098	quercetin	Tyrosine-protein kinase HCK	P08631	HCK	0.034
Silybum marianum(SM)	MOL000098	quercetin	Proto-oncogene serine/threonine-protein kinase Pim-1	P11309	PIM1	0.034
Silybum marianum(SM)	MOL000098	quercetin	Muscarinic acetylcholine receptor M3	P20309	CHRM3	0.034
Silybum marianum(SM)	MOL000098	quercetin	ATP synthase subunit alpha, mitochondrial	P25705	ATP5F1A	0.034
Silybum marianum(SM)	MOL000098	quercetin	Phosphatidylinositol-4,5-bisphosphate 3-kinase catalytic subunit gamma isoform	P48736	PIK3CG	0.034
Silybum marianum(SM)	MOL000098	quercetin	Epidermal growth factor receptor	P00533	EGFR	0.035
Silybum marianum(SM)	MOL000098	quercetin	Carbonic anhydrase 1	P00915	CA1	0.035

Silybum marianum(SM)	MOL000098	quercetin	Proto-oncogene tyrosine-protein kinase LCK	P06239	LCK	0.035
Silybum marianum(SM)	MOL000098	quercetin	5-hydroxytryptamine 1A receptor	P08908	HTR1A	0.035
Silybum marianum(SM)	MOL000098	quercetin	Glycogen phosphorylase, muscle form	P11217	PYGM	0.035
Silybum marianum(SM)	MOL000098	quercetin	Alpha-1B adrenergic receptor	P35368	ADRA1B	0.035
Silybum marianum(SM)	MOL000098	quercetin	Bile salt sulfotransferase	Q06520	SULT2A1	0.036
Silybum marianum(SM)	MOL000098	quercetin	Ig kappa chain C region	P01834	IGKC	0.037
Silybum marianum(SM)	MOL000098	quercetin	Ig gamma-1 chain C region	P01857	IGHG1	0.037
Silybum marianum(SM)	MOL000098	quercetin	D(2) dopamine receptor	P14416	DRD2	0.037
Silybum marianum(SM)	MOL000098	quercetin	D(3) dopamine receptor	P35462	DRD3	0.037
Silybum marianum(SM)	MOL000098	quercetin	Estrogen-related receptor gamma	P62508	ESRRG	0.037
Silybum marianum(SM)	MOL000098	quercetin	Muscarinic acetylcholine receptor M2	P08172	CHRM2	0.044
Silybum marianum(SM)	MOL000098	quercetin	Muscarinic acetylcholine receptor M1	P11229	CHRM1	0.044
Silybum marianum(SM)	MOL000098	quercetin	Retinoic acid receptor RXR-beta	P28702	RXRB	0.044
Silybum marianum(SM)	MOL000098	quercetin	Aldo-keto reductase family 1 member C1	Q04828	AKR1C1	0.044
Silybum marianum(SM)	MOL000098	quercetin	Alpha-2A adrenergic receptor	P08913	ADRA2A	0.045
Silybum marianum(SM)	MOL000098	quercetin	Cytochrome P450 19A1	P11511	CYP19A1	0.045
Silybum marianum(SM)	MOL000098	quercetin	Alpha-2C adrenergic receptor	P18825	ADRA2C	0.045
Silybum marianum(SM)	MOL000098	quercetin	Alpha-1A adrenergic receptor	P35348	ADRA1A	0.045
Silybum marianum(SM)	MOL000098	quercetin	Kynurenine--oxoglutarate transaminase 1	Q16773	KYAT1	0.045
Silybum marianum(SM)	MOL000098	quercetin	Membrane copper amine oxidase	Q16853	AOC3	0.045
Silybum marianum(SM)	MOL000098	quercetin	NADPH oxidase organizer 1	Q8NFA2	NOXO1	0.045
Silybum marianum(SM)	MOL000098	quercetin	Serine/threonine-protein kinase 6	O14965	AURKA	0.046
Silybum marianum(SM)	MOL000098	quercetin	Thyroid hormone receptor beta-1	P10828	THRB	0.046
Silybum marianum(SM)	MOL000098	quercetin	Egl nine homolog 1	Q9GZT9	EGLN1	0.046
Silybum marianum(SM)	MOL000098	quercetin	Tubulin beta-1 chain	Q9H4B7	TUBB1	0.046
Silybum marianum(SM)	MOL000098	quercetin	Cannabinoid receptor 1	P21554	CNR1	0.047
Silybum marianum(SM)	MOL000098	quercetin	D(1A) dopamine receptor	P21728	DRD1	0.047
Silybum marianum(SM)	MOL000098	quercetin	D(1B) dopamine receptor	P21918	DRD5	0.047
Silybum marianum(SM)	MOL000098	quercetin	Endothelin-1 receptor	P25101	EDNRA	0.047
Silybum marianum(SM)	MOL000098	quercetin	Thiamin pyrophosphokinase 1	Q9H3S4	TPK1	0.047
Silybum marianum(SM)	MOL000098	quercetin	Glucocorticoid receptor	P04150	NR3C1	0.048
Silybum marianum(SM)	MOL000098	quercetin	Ig kappa chain V-II region RPMI 6410	P06310	IGKV2-30	0.048
Silybum marianum(SM)	MOL000098	quercetin	Leukotriene A-4 hydrolase	P09960	LTA4H	0.048
Silybum marianum(SM)	MOL000098	quercetin	RAC-alpha serine/threonine-protein kinase	P31749	AKT1	0.048
Silybum marianum(SM)	MOL000098	quercetin	Nitric oxide synthase, inducible	P35228	NOS2	0.048
Silybum marianum(SM)	MOL000098	quercetin	S-methyl-5-thioadenosine phosphorylase	Q13126	MTAP	0.048
Silybum marianum(SM)	MOL000098	quercetin	cAMP-specific 3',5'-cyclic phosphodiesterase 4A	P27815	PDE4A	0.049
Silybum marianum(SM)	MOL000098	quercetin	Triosephosphate isomerase	P60174	TPI1	0.049
Silybum marianum(SM)	MOL000098	quercetin	Solute carrier family 12 member 1	Q13621	SLC12A1	0.049
Silybum marianum(SM)	MOL000098	quercetin	Thyroid hormone receptor alpha	P10827	THRA	0.05
Silybum marianum(SM)	MOL000098	quercetin	Calmodulin	P62158		0.05
Silybum marianum(SM)	MOL000098	quercetin	Casein kinase II subunit alpha	P68400	CSNK2A1	0.05
Silybum marianum(SM)	MOL000098	quercetin	Neuropeptide Y	P01303	NPY	0.051
Silybum marianum(SM)	MOL000098	quercetin	Peroxisome proliferator-activated receptor gamma	P37231	PPARG	0.051
Silybum marianum(SM)	MOL000098	quercetin	Nuclear receptor coactivator 2	Q15596	NCOA2	0.051
Silybum marianum(SM)	MOL000098	quercetin	Toll-like receptor 7	Q9NYK1	TLR7	0.051
Silybum marianum(SM)	MOL000098	quercetin	C-jun-amino-terminal kinase-interacting protein 1	Q9UQF2	MAPK8IP1	0.051

Silybum marianum(SM)	MOL000098	quercetin	cAMP-dependent protein kinase inhibitor alpha	P61925	PKIA	0.053
Silybum marianum(SM)	MOL000098	quercetin	Interferon gamma	P01579	IFNG	0.055
Silybum marianum(SM)	MOL000098	quercetin	Fibroblast growth factor receptor 2	P21802	FGFR2	0.055
Silybum marianum(SM)	MOL000098	quercetin	Sodium-dependent noradrenaline transporter	P23975	SLC6A2	0.055
Silybum marianum(SM)	MOL000098	quercetin	MAP kinase-activated protein kinase 2	P49137	MAPKAPK2	0.055
Silybum marianum(SM)	MOL000098	quercetin	Glycogen synthase kinase-3 beta	P49841	GSK3B	0.055
Silybum marianum(SM)	MOL000098	quercetin	Sodium-dependent dopamine transporter	Q01959	SLC6A3	0.055
Silybum marianum(SM)	MOL000098	quercetin	Nuclear receptor coactivator 5	Q9HCD5	NCOA5	0.055
Silybum marianum(SM)	MOL000098	quercetin	Carbonic anhydrase 2	P00918	CA2	0.056
Silybum marianum(SM)	MOL000098	quercetin	Alpha-2B adrenergic receptor	P18089	ADRA2B	0.056
Silybum marianum(SM)	MOL000098	quercetin	Proto-oncogene tyrosine-protein kinase Src	P12931	SRC	0.057
Silybum marianum(SM)	MOL000098	quercetin	DNA polymerase kappa	Q9UBT6	POLK	0.058
Silybum marianum(SM)	MOL000098	quercetin	DNA topoisomerase 2-alpha	P11388	TOP2A	0.059
Silybum marianum(SM)	MOL000098	quercetin	3 beta-hydroxysteroid dehydrogenase/Delta 5-->4-isomerase type I	P14060	HSD3B1	0.061
Silybum marianum(SM)	MOL000098	quercetin	Beta-2 adrenergic receptor	P07550	ADRB2	0.066
Silybum marianum(SM)	MOL000098	quercetin	Beta-1 adrenergic receptor	P08588	ADRB1	0.066
Silybum marianum(SM)	MOL000098	quercetin	Tubulin alpha-3 chain	Q71U36	TUBA1A	0.067
Silybum marianum(SM)	MOL000098	quercetin	Prothrombin	P00734	F2	0.07
Silybum marianum(SM)	MOL000098	quercetin	Estradiol 17-beta-dehydrogenase 1	P14061	HSD17B1	0.071
Silybum marianum(SM)	MOL000098	quercetin	Delta-type opioid receptor	P41143	OPRD1	0.073
Silybum marianum(SM)	MOL000098	quercetin	Kappa-type opioid receptor	P41145	OPRK1	0.073
Silybum marianum(SM)	MOL000098	quercetin	D-HSCDK2	O75100	CA11	0.075
Silybum marianum(SM)	MOL000098	quercetin	Sodium-dependent serotonin transporter	P31645	SLC6A4	0.075
Silybum marianum(SM)	MOL000098	quercetin	Mineralocorticoid receptor	P08235	NR3C2	0.077
Silybum marianum(SM)	MOL000098	quercetin	Inhibitor of nuclear factor kappa-B kinase subunit alpha	O15111	CHUK	0.079
Silybum marianum(SM)	MOL000098	quercetin	Arachidonate 5-lipoxygenase	P09917	ALOX5	0.079
Silybum marianum(SM)	MOL000098	quercetin	cAMP-dependent protein kinase catalytic subunit alpha	P17612	PRKACA	0.083
Silybum marianum(SM)	MOL000098	quercetin	Dihydrofolate reductase	P00374	DHFR	0.091
Silybum marianum(SM)	MOL000098	quercetin	Cell division control protein 2 homolog	P06493	CDK1	0.095
Silybum marianum(SM)	MOL000098	quercetin	Cell division protein kinase 5	Q00535	CDK5	0.115
Silybum marianum(SM)	MOL000098	quercetin	Trypsin-1	P07477	PRSS1	0.138
Silybum marianum(SM)	MOL000098	quercetin	Cyclin-A2	P20248	CCNA2	0.151
Silybum marianum(SM)	MOL000098	quercetin	Cell division protein kinase 2	P24941	CDK2	0.156
Silybum marianum(SM)	MOL000098	quercetin	Hemoglobin subunit alpha	P69905	HBA1	0.169
Silybum marianum(SM)	MOL000098	quercetin	Mu-type opioid receptor	P35372	OPRM1	0.179
Silybum marianum(SM)	MOL000098	quercetin	Progesterone receptor	P06401	PGR	0.2
Silybum marianum(SM)	MOL000098	quercetin	Nuclear receptor coactivator 1	Q15788	NCOA1	0.205
Silybum marianum(SM)	MOL000098	quercetin	Prostaglandin G/H synthase 1	P23219	PTGS1	0.22
Silybum marianum(SM)	MOL000098	quercetin	Estrogen receptor beta	Q92731	ESR2	0.357
Silybum marianum(SM)	MOL000098	quercetin	Prostaglandin G/H synthase 2	P35354	PTGS2	0.39
Silybum marianum(SM)	MOL000098	quercetin	Estrogen receptor	P03372	ESR1	1
Silybum marianum(SM)	MOL000449	Stigmasterol	5-hydroxytryptamine 1A receptor	P08908	HTR1A	0.012
Silybum marianum(SM)	MOL000449	Stigmasterol	Alpha-2A adrenergic receptor	P08913	ADRA2A	0.012
Silybum marianum(SM)	MOL000449	Stigmasterol	D(2) dopamine receptor	P14416	DRD2	0.012
Silybum marianum(SM)	MOL000449	Stigmasterol	Alpha-2B adrenergic receptor	P18089	ADRA2B	0.012
Silybum marianum(SM)	MOL000449	Stigmasterol	Alpha-2C adrenergic receptor	P18825	ADRA2C	0.012
Silybum marianum(SM)	MOL000449	Stigmasterol	5-hydroxytryptamine 1D receptor	P28221	HTR1D	0.012

Silybum marianum(SM)	MOL000449	Stigmasterol	5-hydroxytryptamine 1B receptor	P28222	HTR1B	0.012
Silybum marianum(SM)	MOL000449	Stigmasterol	5-hydroxytryptamine 2C receptor	P28335	HTR2C	0.012
Silybum marianum(SM)	MOL000449	Stigmasterol	D(3) dopamine receptor	P35462	DRD3	0.012
Silybum marianum(SM)	MOL000449	Stigmasterol	Elongation factor Tu GTP-binding domain-containing protein 1	Q7Z2Z2	EFL1	0.024
Silybum marianum(SM)	MOL000449	Stigmasterol	Phosphatidylinositol 3-kinase regulatory subunit alpha	P27986	PIK3R1	0.031
Silybum marianum(SM)	MOL000449	Stigmasterol	Neuronal acetylcholine receptor subunit alpha-3	P32297	CHRNA3	0.031
Silybum marianum(SM)	MOL000449	Stigmasterol	Neuronal acetylcholine receptor subunit alpha-7	P36544	CHRNA7	0.031
Silybum marianum(SM)	MOL000449	Stigmasterol	Neuronal acetylcholine receptor subunit alpha-4	P43681	CHRNA4	0.031
Silybum marianum(SM)	MOL000449	Stigmasterol	Phosphatidylinositol-4,5-bisphosphate 3-kinase catalytic subunit gamma isoform	P48736	PIK3CG	0.031
Silybum marianum(SM)	MOL000449	Stigmasterol	Serine/threonine-protein kinase PLK1	P53350	PLK1	0.031
Silybum marianum(SM)	MOL000449	Stigmasterol	Dehydrogenase/reductase SDR family member 8	Q8NBQ5	HSD17B11	0.037
Silybum marianum(SM)	MOL000449	Stigmasterol	Ig kappa chain C region	P01834	IGKC	0.04
Silybum marianum(SM)	MOL000449	Stigmasterol	Ig gamma-1 chain C region	P01857	IGHG1	0.04
Silybum marianum(SM)	MOL000449	Stigmasterol	Ig gamma-2 chain C region	P01859	IGHG2	0.04
Silybum marianum(SM)	MOL000449	Stigmasterol	Retinoic acid receptor alpha	P10276	RARA	0.041
Silybum marianum(SM)	MOL000449	Stigmasterol	Retinoic acid receptor gamma-1	P13631	RARG	0.041
Silybum marianum(SM)	MOL000449	Stigmasterol	Nuclear receptor coactivator 2	Q15596	NCOA2	0.041
Silybum marianum(SM)	MOL000449	Stigmasterol	3 beta-hydroxysteroid dehydrogenase/Delta 5-->4-isomerase type II	P26439	HSD3B2	0.06
Silybum marianum(SM)	MOL000449	Stigmasterol	Annexin A1	P04083	ANXA1	0.063
Silybum marianum(SM)	MOL000449	Stigmasterol	Nuclear receptor 0B1	P51843	NR0B1	0.063
Silybum marianum(SM)	MOL000449	Stigmasterol	Microtubule-associated protein 2	P11137	MAP2	0.064
Silybum marianum(SM)	MOL000449	Stigmasterol	Microtubule-associated protein 1A	P78559	MAP1A	0.064
Silybum marianum(SM)	MOL000449	Stigmasterol	Prolactin receptor	P16471	PRLR	0.065
Silybum marianum(SM)	MOL000449	Stigmasterol	3-oxo-5-alpha-steroid 4-dehydrogenase 2	P31213	SRD5A2	0.065
Silybum marianum(SM)	MOL000449	Stigmasterol	Cytosolic phospholipase A2	P47712	PLA2G4A	0.065
Silybum marianum(SM)	MOL000449	Stigmasterol	Estrogen receptor beta	Q92731	ESR2	0.065
Silybum marianum(SM)	MOL000449	Stigmasterol	Gonadotropin-releasing hormone receptor	P30968	GNRHR	0.066
Silybum marianum(SM)	MOL000449	Stigmasterol	Gonadotropin-releasing hormone II receptor	Q96P88	GNRHR2	0.066
Silybum marianum(SM)	MOL000449	Stigmasterol	Corticosteroid 11-beta-dehydrogenase isozyme 1	P28845	HSD11B1	0.067
Silybum marianum(SM)	MOL000449	Stigmasterol	Retinoic acid receptor RXR-alpha	P19793	RXRA	0.072
Silybum marianum(SM)	MOL000449	Stigmasterol	Nuclear receptor subfamily 1 group I member 3	Q14994	NR1I3	0.072
Silybum marianum(SM)	MOL000449	Stigmasterol	Bile salt sulfotransferase	Q06520	SULT2A1	0.087
Silybum marianum(SM)	MOL000449	Stigmasterol	Retinoic acid receptor RXR-beta	P28702	RXRB	0.094
Silybum marianum(SM)	MOL000449	Stigmasterol	cAMP-dependent protein kinase catalytic subunit alpha	P17612	PRKACA	0.115
Silybum marianum(SM)	MOL000449	Stigmasterol	DNA polymerase kappa	Q9UBT6	POLK	0.118
Silybum marianum(SM)	MOL000449	Stigmasterol	NADPH oxidase organizer 1	Q8NFA2	NOXO1	0.122
Silybum marianum(SM)	MOL000449	Stigmasterol	3 beta-hydroxysteroid dehydrogenase/Delta 5-->4-isomerase type I	P14060	HSD3B1	0.123
Silybum marianum(SM)	MOL000449	Stigmasterol	Aldo-keto reductase family 1 member C1	Q04828	AKR1C1	0.128
Silybum marianum(SM)	MOL000449	Stigmasterol	Mediator of RNA polymerase II transcription subunit 1	Q15648	MED1	0.129
Silybum marianum(SM)	MOL000449	Stigmasterol	Cannabinoid receptor 1	P21554	CNR1	0.13
Silybum marianum(SM)	MOL000449	Stigmasterol	Cannabinoid receptor 2	P34972	CNR2	0.137
Silybum marianum(SM)	MOL000449	Stigmasterol	Nuclear receptor coactivator 5	Q9HCD5	NCOA5	0.137
Silybum marianum(SM)	MOL000449	Stigmasterol	3-oxo-5-alpha-steroid 4-dehydrogenase 1	P18405	SRD5A1	0.142
Silybum marianum(SM)	MOL000449	Stigmasterol	Estradiol 17-beta-dehydrogenase 1	P14061	HSD17B1	0.214
Silybum marianum(SM)	MOL000449	Stigmasterol	Androgen receptor	P10275	AR	0.221
Silybum marianum(SM)	MOL000449	Stigmasterol	Nuclear receptor coactivator 1	Q15788	NCOA1	0.241
Silybum marianum(SM)	MOL000449	Stigmasterol	Glucocorticoid receptor	P04150	NR3C1	0.292

Silybum marianum(SM)	MOL000449	Stigmasterol	Mineralocorticoid receptor	P08235	NR3C2	0.377
Silybum marianum(SM)	MOL000449	Stigmasterol	Estrogen receptor	P03372	ESR1	0.983
Silybum marianum(SM)	MOL000449	Stigmasterol	Progesterone receptor	P06401	PGR	1
Silybum marianum(SM)	MOL000953	CLR	Elongation factor Tu GTP-binding domain-containing protein 1	Q7Z2Z2	EFL1	0.013
Silybum marianum(SM)	MOL000953	CLR	Retinoic acid-induced protein 3	Q8NFJ5	GPRC5A	0.016
Silybum marianum(SM)	MOL000953	CLR	5-hydroxytryptamine 1A receptor	P08908	HTR1A	0.017
Silybum marianum(SM)	MOL000953	CLR	5-hydroxytryptamine 2A receptor	P28223	HTR2A	0.018
Silybum marianum(SM)	MOL000953	CLR	5-hydroxytryptamine 2C receptor	P28335	HTR2C	0.018
Silybum marianum(SM)	MOL000953	CLR	D(3) dopamine receptor	P35462	DRD3	0.018
Silybum marianum(SM)	MOL000953	CLR	Muscarinic acetylcholine receptor M4	P08173	CHRM4	0.019
Silybum marianum(SM)	MOL000953	CLR	Muscarinic acetylcholine receptor M5	P08912	CHRM5	0.019
Silybum marianum(SM)	MOL000953	CLR	Muscarinic acetylcholine receptor M3	P20309	CHRM3	0.019
Silybum marianum(SM)	MOL000953	CLR	Histamine H1 receptor	P35367	HRH1	0.019
Silybum marianum(SM)	MOL000953	CLR	Tyrosine-protein kinase JAK2	O60674	JAK2	0.023
Silybum marianum(SM)	MOL000953	CLR	Muscarinic acetylcholine receptor M2	P08172	CHRM2	0.023
Silybum marianum(SM)	MOL000953	CLR	Tyrosine-protein kinase JAK1	P23458	JAK1	0.023
Silybum marianum(SM)	MOL000953	CLR	Tyrosine-protein kinase JAK3	P52333	JAK3	0.023
Silybum marianum(SM)	MOL000953	CLR	Neuronal acetylcholine receptor subunit alpha-3	P32297	CHRNA3	0.024
Silybum marianum(SM)	MOL000953	CLR	Neuronal acetylcholine receptor subunit alpha-7	P36544	CHRNA7	0.024
Silybum marianum(SM)	MOL000953	CLR	Neuronal acetylcholine receptor subunit alpha-4	P43681	CHRNA4	0.024
Silybum marianum(SM)	MOL000953	CLR	Nuclear receptor subfamily 1 group I member 3	Q14994	NR1I3	0.027
Silybum marianum(SM)	MOL000953	CLR	Retinal dehydrogenase 2	O94788	ALDH1A2	0.03
Silybum marianum(SM)	MOL000953	CLR	Retinal dehydrogenase 1	P00352	ALDH1A1	0.03
Silybum marianum(SM)	MOL000953	CLR	Dehydrogenase/reductase SDR family member 8	Q8NBQ5	HSD17B11	0.03
Silybum marianum(SM)	MOL000953	CLR	5-hydroxytryptamine 2B receptor	P41595	HTR2B	0.031
Silybum marianum(SM)	MOL000953	CLR	Ig kappa chain C region	P01834	IGKC	0.032
Silybum marianum(SM)	MOL000953	CLR	Ig gamma-1 chain C region	P01857	IGHG1	0.032
Silybum marianum(SM)	MOL000953	CLR	Ig gamma-2 chain C region	P01859	IGHG2	0.032
Silybum marianum(SM)	MOL000953	CLR	Muscarinic acetylcholine receptor M1	P11229	CHRM1	0.032
Silybum marianum(SM)	MOL000953	CLR	Annexin A1	P04083	ANXA1	0.034
Silybum marianum(SM)	MOL000953	CLR	Gamma-aminobutyric-acid receptor subunit alpha-1	P14867	GABRA1	0.039
Silybum marianum(SM)	MOL000953	CLR	Gamma-aminobutyric-acid receptor subunit alpha-2	P47869	GABRA2	0.039
Silybum marianum(SM)	MOL000953	CLR	D(2) dopamine receptor	P14416	DRD2	0.044
Silybum marianum(SM)	MOL000953	CLR	D(1A) dopamine receptor	P21728	DRD1	0.044
Silybum marianum(SM)	MOL000953	CLR	Cytosolic phospholipase A2	P47712	PLA2G4A	0.048
Silybum marianum(SM)	MOL000953	CLR	3 beta-hydroxysteroid dehydrogenase/Delta 5-->4-isomerase type II	P26439	HSD3B2	0.051
Silybum marianum(SM)	MOL000953	CLR	Corticosteroid 11-beta-dehydrogenase isozyme 1	P28845	HSD11B1	0.052
Silybum marianum(SM)	MOL000953	CLR	Gonadotropin-releasing hormone receptor	P30968	GNRHR	0.054
Silybum marianum(SM)	MOL000953	CLR	Gonadotropin-releasing hormone II receptor	Q96P88	GNRHR2	0.054
Silybum marianum(SM)	MOL000953	CLR	Microtubule-associated protein 2	P11137	MAP2	0.055
Silybum marianum(SM)	MOL000953	CLR	Prolactin receptor	P16471	PRLR	0.055
Silybum marianum(SM)	MOL000953	CLR	Microtubule-associated protein 1A	P78559	MAP1A	0.055
Silybum marianum(SM)	MOL000953	CLR	Nuclear receptor 0B1	P51843	NR0B1	0.058
Silybum marianum(SM)	MOL000953	CLR	Bile salt sulfotransferase	Q06520	SULT2A1	0.066
Silybum marianum(SM)	MOL000953	CLR	cAMP-dependent protein kinase catalytic subunit alpha	P17612	PRKACA	0.084
Silybum marianum(SM)	MOL000953	CLR	Mu-type opioid receptor	P35372	OPRM1	0.084
Silybum marianum(SM)	MOL000953	CLR	Retinoic acid receptor gamma-1	P13631	RARG	0.093

Silybum marianum(SM)	MOL000953	CLR	Retinoic acid receptor RXR-gamma	P48443	RXRG	0.093
Silybum marianum(SM)	MOL000953	CLR	Lysozyme C	P61626	LYZ	0.093
Silybum marianum(SM)	MOL000953	CLR	ATP-binding cassette transporter sub-family C member 8	Q09428	ABCC8	0.093
Silybum marianum(SM)	MOL000953	CLR	DNA polymerase kappa	Q9UBT6	POLK	0.093
Silybum marianum(SM)	MOL000953	CLR	Retinoic acid receptor beta	P10826	RARB	0.095
Silybum marianum(SM)	MOL000953	CLR	Aldo-keto reductase family 1 member C1	Q04828	AKR1C1	0.095
Silybum marianum(SM)	MOL000953	CLR	Mediator of RNA polymerase II transcription subunit 1	Q15648	MED1	0.096
Silybum marianum(SM)	MOL000953	CLR	3 beta-hydroxysteroid dehydrogenase/Delta 5-->4-isomerase type I	P14060	HSD3B1	0.1
Silybum marianum(SM)	MOL000953	CLR	Nuclear receptor coactivator 5	Q9HCD5	NCOA5	0.102
Silybum marianum(SM)	MOL000953	CLR	Retinoic acid receptor RXR-alpha	P19793	RXRA	0.104
Silybum marianum(SM)	MOL000953	CLR	Cannabinoid receptor 2	P34972	CNR2	0.105
Silybum marianum(SM)	MOL000953	CLR	Nuclear receptor coactivator 1	Q15788	NCOA1	0.108
Silybum marianum(SM)	MOL000953	CLR	3-oxo-5-alpha-steroid 4-dehydrogenase 1	P18405	SRD5A1	0.118
Silybum marianum(SM)	MOL000953	CLR	Retinoic acid receptor RXR-beta	P28702	RXRB	0.119
Silybum marianum(SM)	MOL000953	CLR	3-oxo-5-alpha-steroid 4-dehydrogenase 2	P31213	SRD5A2	0.119
Silybum marianum(SM)	MOL000953	CLR	Retinoic acid receptor alpha	P10276	RARA	0.12
Silybum marianum(SM)	MOL000953	CLR	Estradiol 17-beta-dehydrogenase 1	P14061	HSD17B1	0.165
Silybum marianum(SM)	MOL000953	CLR	Androgen receptor	P10275	AR	0.179
Silybum marianum(SM)	MOL000953	CLR	Prostaglandin G/H synthase 2	P35354	PTGS2	0.189
Silybum marianum(SM)	MOL000953	CLR	Glucocorticoid receptor	P04150	NR3C1	0.196
Silybum marianum(SM)	MOL000953	CLR	Mineralocorticoid receptor	P08235	NR3C2	0.288
Silybum marianum(SM)	MOL000953	CLR	Estrogen receptor beta	Q92731	ESR2	0.444
Silybum marianum(SM)	MOL000953	CLR	Progesterone receptor	P06401	PGR	0.844
Silybum marianum(SM)	MOL000953	CLR	Estrogen receptor	P03372	ESR1	1
Silybum marianum(SM)	MOL001285	octanol	Protein kinase C beta type	P05771	PRKCB	Validated
Silybum marianum(SM)	MOL001477	silymarin	Apoptotic protease-activating factor 1	O14727	APAF1	Validated
Silybum marianum(SM)	MOL001477	silymarin	Baculoviral IAP repeat-containing protein 5	O15392	BIRC5	Validated
Silybum marianum(SM)	MOL001477	silymarin	Superoxide dismutase [Cu-Zn]	P00441	SOD1	Validated
Silybum marianum(SM)	MOL001477	silymarin	Tumor necrosis factor	P01375	TNF	Validated
Silybum marianum(SM)	MOL001477	silymarin	Interleukin-1 beta	P01584	IL1B	Validated
Silybum marianum(SM)	MOL001477	silymarin	Catalase	P04040	CAT	Validated
Silybum marianum(SM)	MOL001477	silymarin	Interleukin-4	P05112	IL4	Validated
Silybum marianum(SM)	MOL001477	silymarin	Myeloperoxidase	P05164	MPO	Validated
Silybum marianum(SM)	MOL001477	silymarin	Intercellular adhesion molecule 1	P05362	ICAM1	Validated
Silybum marianum(SM)	MOL001477	silymarin	Cell division control protein 2 homolog	P06493	CDC2	Validated
Silybum marianum(SM)	MOL001477	silymarin	Low affinity immunoglobulin epsilon Fc receptor	P06734	FCER2	Validated
Silybum marianum(SM)	MOL001477	silymarin	Cytochrome P450 3A4	P08684	CYP3A4	Validated
Silybum marianum(SM)	MOL001477	silymarin	Interleukin-8	P10145	IL8	Validated
Silybum marianum(SM)	MOL001477	silymarin	Apoptosis regulator Bcl-2	P10415	BCL2	Validated
Silybum marianum(SM)	MOL001477	silymarin	G2/mitotic-specific cyclin-B1	P14635	CCNB1	Validated
Silybum marianum(SM)	MOL001477	silymarin	Vascular endothelial growth factor A	P15692	VEGFA	Validated
Silybum marianum(SM)	MOL001477	silymarin	E-selectin	P16581	SELE	Validated
Silybum marianum(SM)	MOL001477	silymarin	Cyclin-A2	P20248	CCNA2	Validated
Silybum marianum(SM)	MOL001477	silymarin	UDP-glucuronosyltransferase 1-1	P22309	UGT1A1	Validated
Silybum marianum(SM)	MOL001477	tyramine	Sodium-dependent noradrenaline transporter	P23975	SLC6A2	Validated
Silybum marianum(SM)	MOL001477	silymarin	Alanine aminotransferase 1	P24298	GPT	Validated
Silybum marianum(SM)	MOL001477	silymarin	G1/S-specific cyclin-D1	P24385	CCND1	Validated

Silybum marianum(SM)	MOL001477	silymarin	Prostaglandin G/H synthase 2	P35354	PTGS2	Validated
Silybum marianum(SM)	MOL001477	silymarin	Caspase-3	P42574	CASP3	Validated
Silybum marianum(SM)	MOL001477	silymarin	Carnitine O-acetyltransferase	P43155	CRAT	Validated
Silybum marianum(SM)	MOL001477	silymarin	Mitogen-activated protein kinase 8	P45983	MAPK8	Validated
Silybum marianum(SM)	MOL001477	silymarin	Casein kinase I isoform alpha	P48729	CSNK1A1	Validated
Silybum marianum(SM)	MOL001477	silymarin	Interleukin-2	P60568	IL2	Validated
Silybum marianum(SM)	MOL001477	silymarin	Transcription factor p65	Q04206	RELA	Validated
Silybum marianum(SM)	MOL001477	silymarin	Apoptosis regulator BAX	Q07812	BAX	Validated
Silybum marianum(SM)	MOL001477	silymarin	Bcl-2-like protein 1	Q07817	BCL2L2	Validated
Silybum marianum(SM)	MOL001477	silymarin	ATP-binding cassette sub-family B member 5	Q2M3G0	ABCB5	Validated
Silybum marianum(SM)	MOL001477	silymarin	NAD-dependent deacetylase sirtuin-1	Q96EB6	SIRT1	Validated
Silybum marianum(SM)	MOL001477	silymarin	Protocadherin alpha-C2	Q9Y5I4	PCDHAC2	Validated
Silybum marianum(SM)	MOL001736	(-)-taxifolin	Gamma-aminobutyric acid receptor subunit rho-3	A8MPY1	GABRR3	0.011
Silybum marianum(SM)	MOL001736	(-)-taxifolin	Gamma-aminobutyric acid receptor subunit pi	O00591	GABRP	0.011
Silybum marianum(SM)	MOL001736	(-)-taxifolin	Gamma-aminobutyric acid receptor subunit delta	O14764	GABRD	0.011
Silybum marianum(SM)	MOL001736	(-)-taxifolin	Cytochrome b-c1 complex subunit 8	O14949	UQCRQ	0.011
Silybum marianum(SM)	MOL001736	(-)-taxifolin	Cytochrome b-c1 complex subunit 10	O14957	UQCR11	0.011
Silybum marianum(SM)	MOL001736	(-)-taxifolin	Cytochrome b-c1 complex subunit 6, mitochondrial	P07919	UQCRH	0.011
Silybum marianum(SM)	MOL001736	(-)-taxifolin	Cytochrome b-c1 complex subunit 7	P14927	UQCRB	0.011
Silybum marianum(SM)	MOL001736	(-)-taxifolin	Gamma-aminobutyric-acid receptor subunit beta-1	P18505	GABRB1	0.011
Silybum marianum(SM)	MOL001736	(-)-taxifolin	Gamma-aminobutyric acid receptor subunit gamma-2	P18507	GABRG2	0.011
Silybum marianum(SM)	MOL001736	(-)-taxifolin	Cytochrome b-c1 complex subunit 2, mitochondrial	P22695	UQCRC2	0.011
Silybum marianum(SM)	MOL001736	(-)-taxifolin	Gamma-aminobutyric-acid receptor subunit rho-1	P24046	GABRR1	0.011
Silybum marianum(SM)	MOL001736	(-)-taxifolin	Gamma-aminobutyric acid receptor subunit rho-2	P28476	GABRR2	0.011
Silybum marianum(SM)	MOL001736	(-)-taxifolin	Gamma-aminobutyric-acid receptor subunit alpha-5	P31644	GABRA5	0.011
Silybum marianum(SM)	MOL001736	(-)-taxifolin	Ubiquinol-cytochrome-c reductase complex core protein 1, mitochondrial	P31930	UQCRC1	0.011
Silybum marianum(SM)	MOL001736	(-)-taxifolin	Gamma-aminobutyric-acid receptor subunit alpha-3	P34903	GABRA3	0.011
Silybum marianum(SM)	MOL001736	(-)-taxifolin	Gamma-aminobutyric-acid receptor subunit beta-2	P47870	GABRB2	0.011
Silybum marianum(SM)	MOL001736	(-)-taxifolin	Gamma-aminobutyric-acid receptor subunit alpha-4	P48169	GABRA4	0.011
Silybum marianum(SM)	MOL001736	(-)-taxifolin	Gamma-aminobutyric acid receptor subunit epsilon	P78334	GABRE	0.011
Silybum marianum(SM)	MOL001736	(-)-taxifolin	Gamma-aminobutyric-acid receptor subunit alpha-6	Q16445	GABRA6	0.011
Silybum marianum(SM)	MOL001736	(-)-taxifolin	Gamma-aminobutyric acid receptor subunit gamma-1	Q8N1C3	GABRG1	0.011
Silybum marianum(SM)	MOL001736	(-)-taxifolin	Gamma-aminobutyric acid receptor subunit gamma-3	Q99928	GABRG3	0.011
Silybum marianum(SM)	MOL001736	(-)-taxifolin	Cytochrome b-c1 complex subunit 9	Q9UDW1	UQCR10	0.011
Silybum marianum(SM)	MOL001736	(-)-taxifolin	Gamma-aminobutyric acid receptor subunit theta	Q9UN88	GABRQ	0.011
Silybum marianum(SM)	MOL001736	(-)-taxifolin	5-hydroxytryptamine 1A receptor	P08908	HTR1A	0.016
Silybum marianum(SM)	MOL001736	(-)-taxifolin	5-hydroxytryptamine 1D receptor	P28221	HTR1D	0.016
Silybum marianum(SM)	MOL001736	(-)-taxifolin	5-hydroxytryptamine 1B receptor	P28222	HTR1B	0.016
Silybum marianum(SM)	MOL001736	(-)-taxifolin	5-hydroxytryptamine 2C receptor	P28335	HTR2C	0.016
Silybum marianum(SM)	MOL001736	(-)-taxifolin	5-hydroxytryptamine 2B receptor	P41595	HTR2B	0.016
Silybum marianum(SM)	MOL001736	(-)-taxifolin	Calmodulin	P62158		0.016
Silybum marianum(SM)	MOL001736	(-)-taxifolin	D1 dopamine receptor-interacting protein calcyon	Q9NYX4	CALY	0.016
Silybum marianum(SM)	MOL001736	(-)-taxifolin	Phosphatidylinositol 3-kinase regulatory subunit beta	O00459	PIK3R2	0.017
Silybum marianum(SM)	MOL001736	(-)-taxifolin	Beta-3 adrenergic receptor	P13945	ADRB3	0.017
Silybum marianum(SM)	MOL001736	(-)-taxifolin	Sodium/hydrogen exchanger 1	P19634	SLC9A1	0.017
Silybum marianum(SM)	MOL001736	(-)-taxifolin	Amiloride-sensitive amine oxidase [copper-containing]	P19801	AOC1	0.017
Silybum marianum(SM)	MOL001736	(-)-taxifolin	Phosphatidylinositol 3-kinase regulatory subunit alpha	P27986	PIK3R1	0.017

Silybum marianum(SM)	MOL001736	(-)-taxifolin	5-hydroxytryptamine 2A receptor	P28223	HTR2A	0.017
Silybum marianum(SM)	MOL001736	(-)-taxifolin	Mitogen-activated protein kinase 1	P28482	MAPK1	0.017
Silybum marianum(SM)	MOL001736	(-)-taxifolin	Glutamate receptor 2	P42262	GRIA2	0.017
Silybum marianum(SM)	MOL001736	(-)-taxifolin	Amiloride-sensitive sodium channel subunit delta	P51172	SCNN1D	0.017
Silybum marianum(SM)	MOL001736	(-)-taxifolin	Amiloride-sensitive cation channel 2, neuronal	P78348	ASIC1	0.017
Silybum marianum(SM)	MOL001736	(-)-taxifolin	Potassium voltage-gated channel subfamily H member 2	Q12809	KCNH2	0.017
Silybum marianum(SM)	MOL001736	(-)-taxifolin	Amiloride-sensitive cation channel 1, neuronal	Q16515	ASIC2	0.017
Silybum marianum(SM)	MOL001736	(-)-taxifolin	Potassium channel subfamily K member 6	Q9Y257	KCNK6	0.02
Silybum marianum(SM)	MOL001736	(-)-taxifolin	5-hydroxytryptamine 3 receptor	P46098	HTR3A	0.021
Silybum marianum(SM)	MOL001736	(-)-taxifolin	Glutamate [NMDA] receptor subunit 3A	Q8TCU5	GRIN3A	0.021
Silybum marianum(SM)	MOL001736	(-)-taxifolin	Death-associated protein kinase 3	O43293	DAPK3	0.022
Silybum marianum(SM)	MOL001736	(-)-taxifolin	Tyrosine-protein kinase JAK2	O60674	JAK2	0.022
Silybum marianum(SM)	MOL001736	(-)-taxifolin	Tyrosine-protein kinase JAK1	P23458	JAK1	0.022
Silybum marianum(SM)	MOL001736	(-)-taxifolin	Tyrosine-protein kinase JAK3	P52333	JAK3	0.022
Silybum marianum(SM)	MOL001736	(-)-taxifolin	Pepsin A	P00790	REN	0.027
Silybum marianum(SM)	MOL001736	(-)-taxifolin	Dipeptidyl peptidase 4	P27487	DPP4	0.027
Silybum marianum(SM)	MOL001736	(-)-taxifolin	Cytochrome b	P00156	MT-CYB	0.028
Silybum marianum(SM)	MOL001736	(-)-taxifolin	Cytochrome c1, heme protein, mitochondrial	P08574	CYC1	0.028
Silybum marianum(SM)	MOL001736	(-)-taxifolin	Cytochrome b-c1 complex subunit Rieske, mitochondrial	P47985	UQCRFS1	0.028
Silybum marianum(SM)	MOL001736	(-)-taxifolin	Cell division protein kinase 4	P11802	CDK4	0.029
Silybum marianum(SM)	MOL001736	(-)-taxifolin	Cell division protein kinase 7	P50613	CDK7	0.029
Silybum marianum(SM)	MOL001736	(-)-taxifolin	Cell division protein kinase 9	P50750	CDK9	0.029
Silybum marianum(SM)	MOL001736	(-)-taxifolin	Cell division protein kinase 6	Q00534	CDK6	0.029
Silybum marianum(SM)	MOL001736	(-)-taxifolin	Tyrosine 3-monooxygenase	P07101	TH	0.03
Silybum marianum(SM)	MOL001736	(-)-taxifolin	Tyrosyl-tRNA synthetase, mitochondrial	Q9Y2Z4	YARS2	0.03
Silybum marianum(SM)	MOL001736	(-)-taxifolin	Adenosine A2b receptor	P29275	ADORA2B	0.033
Silybum marianum(SM)	MOL001736	(-)-taxifolin	Adenosine A3 receptor	P33765	SHMT1	0.033
Silybum marianum(SM)	MOL001736	(-)-taxifolin	Gamma-aminobutyric-acid receptor subunit alpha-2	P47869	GABRA2	0.033
Silybum marianum(SM)	MOL001736	(-)-taxifolin	Cyclin-dependent kinase 5 activator 1	Q15078	CDK5R1	0.033
Silybum marianum(SM)	MOL001736	(-)-taxifolin	cGMP-specific 3',5'-cyclic phosphodiesterase	O76074	PDE5A	0.034
Silybum marianum(SM)	MOL001736	(-)-taxifolin	Adenosine A1 receptor	P30542	ADORA1	0.034
Silybum marianum(SM)	MOL001736	(-)-taxifolin	cAMP-specific 3',5'-cyclic phosphodiesterase 4C	Q08493	PDE4C	0.034
Silybum marianum(SM)	MOL001736	(-)-taxifolin	Phenylalanine-4-hydroxylase	P00439	PAH	0.038
Silybum marianum(SM)	MOL001736	(-)-taxifolin	D(4) dopamine receptor	P21917	DRD4	0.038
Silybum marianum(SM)	MOL001736	(-)-taxifolin	Amiloride-sensitive sodium channel subunit alpha	P37088	SCNN1A	0.039
Silybum marianum(SM)	MOL001736	(-)-taxifolin	Amiloride-sensitive sodium channel subunit beta	P51168	SCNN1B	0.039
Silybum marianum(SM)	MOL001736	(-)-taxifolin	Amiloride-sensitive sodium channel subunit gamma	P51170	SCNN1G	0.039
Silybum marianum(SM)	MOL001736	(-)-taxifolin	Potassium channel subfamily K member 1	O00180	KCNK1	0.042
Silybum marianum(SM)	MOL001736	(-)-taxifolin	Carbonic anhydrase 4	P22748	CA4	0.043
Silybum marianum(SM)	MOL001736	(-)-taxifolin	Sodium/potassium-transporting ATPase gamma chain	P54710	FXYD2	0.043
Silybum marianum(SM)	MOL001736	(-)-taxifolin	Sodium-dependent dopamine transporter	Q01959	SLC6A3	0.043
Silybum marianum(SM)	MOL001736	(-)-taxifolin	Macrophage migration inhibitory factor	P14174	MIF	0.044
Silybum marianum(SM)	MOL001736	(-)-taxifolin	Serine/threonine-protein kinase 17B	O94768	STK17B	0.045
Silybum marianum(SM)	MOL001736	(-)-taxifolin	Proto-oncogene serine/threonine-protein kinase Pim-1	P11309	PIM1	0.045
Silybum marianum(SM)	MOL001736	(-)-taxifolin	ATP synthase subunit gamma, mitochondrial	P36542	ATP5F1C	0.045
Silybum marianum(SM)	MOL001736	(-)-taxifolin	UDP-glucuronosyltransferase 3A1	Q6NUS8	UGT3A1	0.045
Silybum marianum(SM)	MOL001736	(-)-taxifolin	cAMP-dependent protein kinase inhibitor alpha	P61925	PKIA	0.047

Silybum marianum(SM)	MOL001736	(-)-taxifolin	Sodium channel protein type 10 subunit alpha	Q9Y5Y9	SCN10A	0.047
Silybum marianum(SM)	MOL001736	(-)-taxifolin	Rho-associated protein kinase 1	Q13464	ROCK1	0.048
Silybum marianum(SM)	MOL001736	(-)-taxifolin	Ig kappa chain C region	P01834	IGKC	0.051
Silybum marianum(SM)	MOL001736	(-)-taxifolin	Ig gamma-1 chain C region	P01857	IGHG1	0.051
Silybum marianum(SM)	MOL001736	(-)-taxifolin	Carboxypeptidase A1	P15085	CPA1	0.051
Silybum marianum(SM)	MOL001736	(-)-taxifolin	Chymase	P23946	CMA1	0.051
Silybum marianum(SM)	MOL001736	(-)-taxifolin	Mitogen-activated protein kinase 8	P45983	MAPK8	0.051
Silybum marianum(SM)	MOL001736	(-)-taxifolin	Mitogen-activated protein kinase 10	P53779	MAPK10	0.051
Silybum marianum(SM)	MOL001736	(-)-taxifolin	Peptidyl-prolyl cis-trans isomerase, mitochondrial	P30405	PPIF	0.052
Silybum marianum(SM)	MOL001736	(-)-taxifolin	Proto-oncogene tyrosine-protein kinase LCK	P06239	LCK	0.053
Silybum marianum(SM)	MOL001736	(-)-taxifolin	Tyrosine-protein kinase Lyn	P07948	LYN	0.053
Silybum marianum(SM)	MOL001736	(-)-taxifolin	Muscarinic acetylcholine receptor M4	P08173	CHRM4	0.053
Silybum marianum(SM)	MOL001736	(-)-taxifolin	Gamma-aminobutyric-acid receptor subunit alpha-1	P14867	GABRA1	0.053
Silybum marianum(SM)	MOL001736	(-)-taxifolin	Aryl hydrocarbon receptor	P35869	AHR	0.054
Silybum marianum(SM)	MOL001736	(-)-taxifolin	Dihydroorotate dehydrogenase, mitochondrial	Q02127	DHODH	0.054
Silybum marianum(SM)	MOL001736	(-)-taxifolin	Glycogen phosphorylase, muscle form	P11217	PYGM	0.055
Silybum marianum(SM)	MOL001736	(-)-taxifolin	Group IIE secretory phospholipase A2	Q9NZK7	PLA2G2E	0.058
Silybum marianum(SM)	MOL001736	(-)-taxifolin	Ribosyldihydronicotinamide dehydrogenase [quinone]	P16083	NQO2	0.059
Silybum marianum(SM)	MOL001736	(-)-taxifolin	Alpha-1D adrenergic receptor	P25100	ADRA1D	0.059
Silybum marianum(SM)	MOL001736	(-)-taxifolin	Alpha-1B adrenergic receptor	P35368	ADRA1B	0.059
Silybum marianum(SM)	MOL001736	(-)-taxifolin	Peroxisome proliferator-activated receptor gamma	P37231	PPARG	0.059
Silybum marianum(SM)	MOL001736	(-)-taxifolin	D(1A) dopamine receptor	P21728	DRD1	0.06
Silybum marianum(SM)	MOL001736	(-)-taxifolin	D(1B) dopamine receptor	P21918	DRD5	0.06
Silybum marianum(SM)	MOL001736	(-)-taxifolin	D(3) dopamine receptor	P35462	DRD3	0.06
Silybum marianum(SM)	MOL001736	(-)-taxifolin	Epidermal growth factor receptor	P00533	EGFR	0.065
Silybum marianum(SM)	MOL001736	(-)-taxifolin	Retinoic acid receptor RXR-beta	P28702	RXRB	0.065
Silybum marianum(SM)	MOL001736	(-)-taxifolin	Tyrosyl-tRNA synthetase, cytoplasmic	P54577	YARS	0.065
Silybum marianum(SM)	MOL001736	(-)-taxifolin	Oxysterols receptor LXR-beta	P55055	NR1H2	0.065
Silybum marianum(SM)	MOL001736	(-)-taxifolin	Oxysterols receptor LXR-alpha	Q13133	NR1H3	0.065
Silybum marianum(SM)	MOL001736	(-)-taxifolin	Nuclear receptor coactivator 2	Q15596	NCOA2	0.065
Silybum marianum(SM)	MOL001736	(-)-taxifolin	Sodium channel protein type 5 subunit alpha	Q14524	SCN5A	0.068
Silybum marianum(SM)	MOL001736	(-)-taxifolin	Adenosine A2a receptor	P29274	ADORA2A	0.073
Silybum marianum(SM)	MOL001736	(-)-taxifolin	Glycogen synthase kinase-3 beta	P49841	GSK3B	0.073
Silybum marianum(SM)	MOL001736	(-)-taxifolin	cAMP-specific 3',5'-cyclic phosphodiesterase 4B	Q07343	PDE4B	0.073
Silybum marianum(SM)	MOL001736	(-)-taxifolin	Platelet glycoprotein IX	P14770	GP9	0.074
Silybum marianum(SM)	MOL001736	(-)-taxifolin	Sterol O-acyltransferase 2	O75908	SOAT2	0.08
Silybum marianum(SM)	MOL001736	(-)-taxifolin	DNA topoisomerase 2-alpha	P11388	TOP2A	0.08
Silybum marianum(SM)	MOL001736	(-)-taxifolin	Sterol O-acyltransferase 1	P35610	SOAT1	0.08
Silybum marianum(SM)	MOL001736	(-)-taxifolin	Protein tyrosine kinase 2 beta	Q14289	PTK2B	0.08
Silybum marianum(SM)	MOL001736	(-)-taxifolin	Alpha-2C adrenergic receptor	P18825	ADRA2C	0.081
Silybum marianum(SM)	MOL001736	(-)-taxifolin	D(2) dopamine receptor	P14416	DRD2	0.082
Silybum marianum(SM)	MOL001736	(-)-taxifolin	Estrogen-related receptor gamma	P62508	ESRRG	0.087
Silybum marianum(SM)	MOL001736	(-)-taxifolin	Carbonic anhydrase 1	P00915	CA1	0.092
Silybum marianum(SM)	MOL001736	(-)-taxifolin	ATP synthase subunit beta, mitochondrial	P06576	ATP5F1B	0.094
Silybum marianum(SM)	MOL001736	(-)-taxifolin	ATP synthase subunit alpha, mitochondrial	P25705	ATP5F1A	0.094
Silybum marianum(SM)	MOL001736	(-)-taxifolin	Heat shock protein HSP 90-beta	P08238	HSP90AB1	0.102
Silybum marianum(SM)	MOL001736	(-)-taxifolin	Alpha-2A adrenergic receptor	P08913	ADRA2A	0.102

Silybum marianum(SM)	MOL001736	(-)-taxifolin	Alpha-2B adrenergic receptor	P18089	ADRA2B	0.102
Silybum marianum(SM)	MOL001736	(-)-taxifolin	Alpha-1A adrenergic receptor	P35348	ADRA1A	0.102
Silybum marianum(SM)	MOL001736	(-)-taxifolin	DNA polymerase kappa	Q9UBT6	POLK	0.102
Silybum marianum(SM)	MOL001736	(-)-taxifolin	Beta-2 adrenergic receptor	P07550	ADRB2	0.103
Silybum marianum(SM)	MOL001736	(-)-taxifolin	Beta-1 adrenergic receptor	P08588	ADRB1	0.103
Silybum marianum(SM)	MOL001736	(-)-taxifolin	Cannabinoid receptor 1	P21554	CNR1	0.103
Silybum marianum(SM)	MOL001736	(-)-taxifolin	Mu-type opioid receptor	P35372	OPRM1	0.103
Silybum marianum(SM)	MOL001736	(-)-taxifolin	D-HSCDK2	O75100	CA11	0.106
Silybum marianum(SM)	MOL001736	(-)-taxifolin	Proto-oncogene tyrosine-protein kinase Src	P12931	SRC	0.107
Silybum marianum(SM)	MOL001736	(-)-taxifolin	Leukotriene A-4 hydrolase	P09960	LTA4H	0.108
Silybum marianum(SM)	MOL001736	(-)-taxifolin	Retinoic acid receptor RXR-alpha	P19793	RXRA	0.108
Silybum marianum(SM)	MOL001736	(-)-taxifolin	Cyclin-A2	P20248	CCNA2	0.108
Silybum marianum(SM)	MOL001736	(-)-taxifolin	Egl nine homolog 1	Q9GZT9	EGLN1	0.108
Silybum marianum(SM)	MOL001736	(-)-taxifolin	C-jun-amino-terminal kinase-interacting protein 1	Q9UQF2	MAPK8IP1	0.108
Silybum marianum(SM)	MOL001736	(-)-taxifolin	Dihydrofolate reductase	P00374	DHFR	0.109
Silybum marianum(SM)	MOL001736	(-)-taxifolin	RAC-alpha serine/threonine-protein kinase	P31749	AKT1	0.109
Silybum marianum(SM)	MOL001736	(-)-taxifolin	Calcium/calmodulin-dependent protein kinase type II alpha chain	Q9UQM7	CAMK2A	0.109
Silybum marianum(SM)	MOL001736	(-)-taxifolin	Muscarinic acetylcholine receptor M1	P11229	CHRM1	0.111
Silybum marianum(SM)	MOL001736	(-)-taxifolin	Solute carrier family 12 member 1	Q13621	SLC12A1	0.111
Silybum marianum(SM)	MOL001736	(-)-taxifolin	cAMP-specific 3',5'-cyclic phosphodiesterase 4A	P27815	PDE4A	0.113
Silybum marianum(SM)	MOL001736	(-)-taxifolin	Tubulin alpha-3 chain	Q71U36	TUBA1A	0.113
Silybum marianum(SM)	MOL001736	(-)-taxifolin	MAP kinase-activated protein kinase 2	P49137	MAPKAPK2	0.114
Silybum marianum(SM)	MOL001736	(-)-taxifolin	Fibroblast growth factor receptor 2	P21802	FGFR2	0.115
Silybum marianum(SM)	MOL001736	(-)-taxifolin	Mitogen-activated protein kinase 14	Q16539	MAPK14	0.115
Silybum marianum(SM)	MOL001736	(-)-taxifolin	Inhibitor of nuclear factor kappa-B kinase subunit alpha	O15111	CHUK	0.122
Silybum marianum(SM)	MOL001736	(-)-taxifolin	Casein kinase II subunit alpha	P68400	CSNK2A1	0.122
Silybum marianum(SM)	MOL001736	(-)-taxifolin	Interferon gamma	P01579	IFNG	0.13
Silybum marianum(SM)	MOL001736	(-)-taxifolin	Carbonic anhydrase 2	P00918	CA2	0.141
Silybum marianum(SM)	MOL001736	(-)-taxifolin	Cell division control protein 2 homolog	P06493	CDK1	0.143
Silybum marianum(SM)	MOL001736	(-)-taxifolin	Phosphatidylinositol-4,5-bisphosphate 3-kinase catalytic subunit gamma isoform	P48736	PIK3CG	0.144
Silybum marianum(SM)	MOL001736	(-)-taxifolin	Tyrosine-protein kinase HCK	P08631	HCK	0.153
Silybum marianum(SM)	MOL001736	(-)-taxifolin	cAMP-dependent protein kinase catalytic subunit alpha	P17612	PRKACA	0.154
Silybum marianum(SM)	MOL001736	(-)-taxifolin	Muscarinic acetylcholine receptor M2	P08172	CHRM2	0.169
Silybum marianum(SM)	MOL001736	(-)-taxifolin	Cell division protein kinase 2	P24941	CDK2	0.181
Silybum marianum(SM)	MOL001736	(-)-taxifolin	Cell division protein kinase 5	Q00535	CDK5	0.181
Silybum marianum(SM)	MOL001736	(-)-taxifolin	Arachidonate 5-lipoxygenase	P09917	ALOX5	0.182
Silybum marianum(SM)	MOL001736	(-)-taxifolin	Prothrombin	P00734	F2	0.188
Silybum marianum(SM)	MOL001736	(-)-taxifolin	Nitric oxide synthase, inducible	P35228	NOS2	0.213
Silybum marianum(SM)	MOL001736	(-)-taxifolin	Trypsin-1	P07477	PRSS1	0.251
Silybum marianum(SM)	MOL001736	(-)-taxifolin	Hemoglobin subunit alpha	P69905	HBA1	0.375
Silybum marianum(SM)	MOL001736	(-)-taxifolin	Prostaglandin G/H synthase 1	P23219	PTGS1	0.381
Silybum marianum(SM)	MOL001736	(-)-taxifolin	Nuclear receptor coactivator 1	Q15788	NCOA1	0.389
Silybum marianum(SM)	MOL001736	(-)-taxifolin	Prostaglandin G/H synthase 2	P35354	PTGS2	0.645
Silybum marianum(SM)	MOL001736	(-)-taxifolin	Estrogen receptor beta	Q92731	ESR2	0.681
Silybum marianum(SM)	MOL001736	(-)-taxifolin	Estrogen receptor	P03372	ESR1	1
Silybum marianum(SM)	MOL007180	vitamin-e	Cyclin-A2	P20248	CCNA2	1
Silybum marianum(SM)	MOL007180	vitamin-e	Cyclin-A2	P20248	CCNA2	1

Silybum marianum(SM)	MOL007180	vitamin-e	Cyclin-A2	P20248	CCNA2	1
Silybum marianum(SM)	MOL007180	vitamin-e	Cyclin-A2	P20248	CCNA2	1
Silybum marianum(SM)	MOL007180	vitamin-e	Cyclin-A2	P20248	CCNA2	1
Silybum marianum(SM)	MOL007449	24-methylidenelophenol	D-HSCDK2	O75100	CA11	0.004
Silybum marianum(SM)	MOL007449	24-methylidenelophenol	Cell division control protein 2 homolog	P06493	CDK1	0.004
Silybum marianum(SM)	MOL007449	24-methylidenelophenol	Cell division protein kinase 5	Q00535	CDK5	0.004
Silybum marianum(SM)	MOL007449	24-methylidenelophenol	Dehydrogenase/reductase SDR family member 8	Q8NBQ5	HSD17B11	0.022
Silybum marianum(SM)	MOL007449	24-methylidenelophenol	Nuclear receptor subfamily 1 group I member 3	Q14994	NR1I3	0.027
Silybum marianum(SM)	MOL007449	24-methylidenelophenol	Proto-oncogene tyrosine-protein kinase LCK	P06239	LCK	0.028
Silybum marianum(SM)	MOL007449	24-methylidenelophenol	Tyrosine-protein kinase Lyn	P07948	LYN	0.028
Silybum marianum(SM)	MOL007449	24-methylidenelophenol	Phospholipase A2	P04054	PLA2G1B	0.031
Silybum marianum(SM)	MOL007449	24-methylidenelophenol	Phospholipase A2, membrane associated	P14555	PLA2G2A	0.031
Silybum marianum(SM)	MOL007449	24-methylidenelophenol	ATP synthase subunit beta, mitochondrial	P06576	ATP5F1B	0.032
Silybum marianum(SM)	MOL007449	24-methylidenelophenol	ATP synthase subunit alpha, mitochondrial	P25705	ATP5F1A	0.032
Silybum marianum(SM)	MOL007449	24-methylidenelophenol	3 beta-hydroxysteroid dehydrogenase/Delta 5-->4-isomerase type II	P26439	HSD3B2	0.045
Silybum marianum(SM)	MOL007449	24-methylidenelophenol	Retinoic acid receptor alpha	P10276	RARA	0.048
Silybum marianum(SM)	MOL007449	24-methylidenelophenol	Retinoic acid receptor beta	P10826	RARB	0.048
Silybum marianum(SM)	MOL007449	24-methylidenelophenol	Retinoic acid receptor RXR-beta	P28702	RXRB	0.048
Silybum marianum(SM)	MOL007449	24-methylidenelophenol	Retinoic acid receptor RXR-gamma	P48443	RXRG	0.048
Silybum marianum(SM)	MOL007449	24-methylidenelophenol	Microtubule-associated protein 2	P11137	MAP2	0.05
Silybum marianum(SM)	MOL007449	24-methylidenelophenol	Microtubule-associated protein 1A	P78559	MAP1A	0.05
Silybum marianum(SM)	MOL007449	24-methylidenelophenol	Gonadotropin-releasing hormone receptor	P30968	GNRHR	0.052
Silybum marianum(SM)	MOL007449	24-methylidenelophenol	Gonadotropin-releasing hormone II receptor	Q96P88	GNRHR2	0.052
Silybum marianum(SM)	MOL007449	24-methylidenelophenol	Retinoic acid receptor RXR-alpha	P19793	RXRA	0.075
Silybum marianum(SM)	MOL007449	24-methylidenelophenol	Bile salt sulfotransferase	Q06520	SULT2A1	0.093
Silybum marianum(SM)	MOL007449	24-methylidenelophenol	Sodium-dependent serotonin transporter	P31645	SLC6A4	0.1
Silybum marianum(SM)	MOL007449	24-methylidenelophenol	DNA polymerase kappa	Q9UBT6	POLK	0.104
Silybum marianum(SM)	MOL007449	24-methylidenelophenol	Glucocorticoid receptor	P04150	NR3C1	0.105
Silybum marianum(SM)	MOL007449	24-methylidenelophenol	Prostaglandin G/H synthase 2	P35354	PTGS2	0.105
Silybum marianum(SM)	MOL007449	24-methylidenelophenol	Estrogen-related receptor gamma	P62508	ESRRG	0.108
Silybum marianum(SM)	MOL007449	24-methylidenelophenol	Mediator of RNA polymerase II transcription subunit 1	Q15648	MED1	0.112
Silybum marianum(SM)	MOL007449	24-methylidenelophenol	Aldo-keto reductase family 1 member C1	Q04828	AKR1C1	0.116
Silybum marianum(SM)	MOL007449	24-methylidenelophenol	Cannabinoid receptor 2	P34972	CNR2	0.122
Silybum marianum(SM)	MOL007449	24-methylidenelophenol	3 beta-hydroxysteroid dehydrogenase/Delta 5-->4-isomerase type I	P14060	HSD3B1	0.124
Silybum marianum(SM)	MOL007449	24-methylidenelophenol	Nuclear receptor coactivator 5	Q9HCD5	NCOA5	0.13
Silybum marianum(SM)	MOL007449	24-methylidenelophenol	Androgen receptor	P10275	AR	0.149
Silybum marianum(SM)	MOL007449	24-methylidenelophenol	Mineralocorticoid receptor	P08235	NR3C2	0.247
Silybum marianum(SM)	MOL007449	24-methylidenelophenol	Estradiol 17-beta-dehydrogenase 1	P14061	HSD17B1	0.253
Silybum marianum(SM)	MOL007449	24-methylidenelophenol	Nuclear receptor coactivator 1	Q15788	NCOA1	0.284
Silybum marianum(SM)	MOL007449	24-methylidenelophenol	Estrogen receptor beta	Q92731	ESR2	0.331
Silybum marianum(SM)	MOL007449	24-methylidenelophenol	Progesterone receptor	P06401	PGR	0.831
Silybum marianum(SM)	MOL007449	24-methylidenelophenol	Estrogen receptor	P03372	ESR1	1
Silybum marianum(SM)	MOL007451	silydianin	Neuronal acetylcholine receptor subunit alpha-3	P32297	CHRNA3	0.054
Silybum marianum(SM)	MOL007451	silydianin	Neuronal acetylcholine receptor subunit alpha-7	P36544	CHRNA7	0.054
Silybum marianum(SM)	MOL007451	silydianin	Retinoic acid receptor alpha	P10276	RARA	0.061
Silybum marianum(SM)	MOL007451	silydianin	Retinoic acid receptor beta	P10826	RARB	0.061
Silybum marianum(SM)	MOL007451	silydianin	Retinoic acid receptor gamma-1	P13631	RARG	0.061

Silybum marianum(SM)	MOL007451	silydianin	Retinoic acid receptor RXR-beta	P28702	RXRB	0.061
Silybum marianum(SM)	MOL007451	silydianin	Retinoic acid receptor RXR-gamma	P48443	RXRG	0.061
Silybum marianum(SM)	MOL007451	silydianin	Potassium channel subfamily K member 1	O00180	KCNK1	0.094
Silybum marianum(SM)	MOL007451	silydianin	Gamma-aminobutyric-acid receptor subunit alpha-1	P14867	GABRA1	0.094
Silybum marianum(SM)	MOL007451	silydianin	Gamma-aminobutyric-acid receptor subunit alpha-2	P47869	GABRA2	0.094
Silybum marianum(SM)	MOL007451	silydianin	Sodium channel protein type 5 subunit alpha	Q14524	SCN5A	0.094
Silybum marianum(SM)	MOL007451	silydianin	Carbonic anhydrase 1	P00915	CA1	0.143
Silybum marianum(SM)	MOL007451	silydianin	Carbonic anhydrase 2	P00918	CA2	0.143
Silybum marianum(SM)	MOL007451	silydianin	Sodium/potassium-transporting ATPase gamma chain	P54710	FXYD2	0.143
Silybum marianum(SM)	MOL007451	silydianin	Progesterone receptor	P06401	PGR	0.224
Silybum marianum(SM)	MOL007451	silydianin	Mineralocorticoid receptor	P08235	NR3C2	0.224
Silybum marianum(SM)	MOL007451	silydianin	Proto-oncogene tyrosine-protein kinase LCK	P06239	LCK	0.246
Silybum marianum(SM)	MOL007451	silydianin	Tyrosine-protein kinase Lyn	P07948	LYN	0.246
Silybum marianum(SM)	MOL007451	silydianin	Phosphatidylinositol-4,5-bisphosphate 3-kinase catalytic subunit gamma isoform	P48736	PIK3CG	0.47
Silybum marianum(SM)	MOL007451	silydianin	Muscarinic acetylcholine receptor M1	P11229	CHRM1	0.488
Silybum marianum(SM)	MOL007451	silydianin	Tyrosine-protein kinase HCK	P08631	HCK	0.534
Silybum marianum(SM)	MOL007451	silydianin	Estrogen receptor	P03372	ESR1	1
Silybum marianum(SM)	MOL007451	silydianin	Cell division control protein 2 homolog	P06493	CDC2	Validated
Silybum marianum(SM)	MOL007451	silydianin	Cell division protein kinase 4	P11802	CDK4	Validated
Silybum marianum(SM)	MOL007451	silydianin	G2/mitotic-specific cyclin-B1	P14635	CCNB1	Validated
Silybum marianum(SM)	MOL007451	silydianin	Cyclin-A2	P20248	CCNA2	Validated
Silybum marianum(SM)	MOL007451	silydianin	G1/S-specific cyclin-D1	P24385	CCND1	Validated
Silybum marianum(SM)	MOL007451	silydianin	Cell division protein kinase 2	P24941	CDK2	Validated
Silybum marianum(SM)	MOL007451	silydianin	G1/S-specific cyclin-D3	P30281	CCND3	Validated
Silybum marianum(SM)	MOL007451	silydianin	M-phase inducer phosphatase 2	P30305	CDC25B	Validated
Silybum marianum(SM)	MOL007451	silydianin	M-phase inducer phosphatase 3	P30307	CDC25C	Validated
Silybum marianum(SM)	MOL007451	silydianin	Cyclin-dependent kinase inhibitor 1	P38936	CDKN1A	Validated
Silybum marianum(SM)	MOL007451	silydianin	Caspase-3	P42574	CASP3	Validated
Silybum marianum(SM)	MOL007451	silydianin	Eukaryotic translation initiation factor 6	P56537	EIF6	Validated
Silybum marianum(SM)	MOL007451	silydianin	Cell division protein kinase 6	Q00534	CDK6	Validated
Silybum marianum(SM)	MOL007451	silydianin	Fanconi anemia group D2 protein	Q9BXW9	FANCD2	Validated
Silybum marianum(SM)	MOL007451	silydianin	Probable E3 ubiquitin-protein ligase HERC5	Q9UII4	HERC5	Validated
Silybum marianum(SM)	MOL007451	silydianin	Protocadherin alpha-C2	Q9Y5I4	PCDHAC2	Validated
Silybum marianum(SM)	MOL007454	silymonin	Tripartite motif-containing protein 13	O60858	TRIM13	0.074
Silybum marianum(SM)	MOL007454	silymonin	Nociceptin receptor	P41146	OPRL1	0.074
Silybum marianum(SM)	MOL007454	silymonin	Phosphatidylinositol 3-kinase regulatory subunit alpha	P27986	PIK3R1	0.164
Silybum marianum(SM)	MOL007454	silymonin	Phosphatidylinositol-4,5-bisphosphate 3-kinase catalytic subunit gamma isoform	P48736	PIK3CG	0.164
Silybum marianum(SM)	MOL007454	silymonin	Serine/threonine-protein kinase PLK1	P53350	PLK1	0.164
Silybum marianum(SM)	MOL007454	silymonin	Mu-type opioid receptor	P35372	OPRM1	0.392
Silybum marianum(SM)	MOL007454	silymonin	Delta-type opioid receptor	P41143	OPRD1	0.392
Silybum marianum(SM)	MOL007454	silymonin	Kappa-type opioid receptor	P41145	OPRK1	0.392
Silybum marianum(SM)	MOL007454	silymonin	Estrogen receptor	P03372	ESR1	0.999
Silybum marianum(SM)	MOL007454	silymonin	Tyrosine-protein kinase HCK	P08631	HCK	1
Silybum marianum(SM)	MOL007455	silandrin	Retinoic acid receptor alpha	P10276	RARA	0.011
Silybum marianum(SM)	MOL007455	silandrin	Retinoic acid receptor beta	P10826	RARB	0.011
Silybum marianum(SM)	MOL007455	silandrin	Retinoic acid receptor gamma-1	P13631	RARG	0.011
Silybum marianum(SM)	MOL007455	silandrin	Retinoic acid receptor RXR-beta	P28702	RXRB	0.011

Silybum marianum(SM)	MOL007455	silandrin	Retinoic acid receptor RXR-gamma	P48443	RXRG	0.011
Silybum marianum(SM)	MOL007455	silandrin	ATP synthase subunit beta, mitochondrial	P06576	ATP5F1B	0.063
Silybum marianum(SM)	MOL007455	silandrin	ATP synthase subunit alpha, mitochondrial	P25705	ATP5F1A	0.063
Silybum marianum(SM)	MOL007455	silandrin	Sterol O-acyltransferase 2	O75908	SOAT2	0.066
Silybum marianum(SM)	MOL007455	silandrin	Sterol O-acyltransferase 1	P35610	SOAT1	0.066
Silybum marianum(SM)	MOL007455	silandrin	Interferon gamma	P01579	IFNG	0.162
Silybum marianum(SM)	MOL007455	silandrin	Nuclear receptor coactivator 1	Q15788	NCOA1	0.311
Silybum marianum(SM)	MOL007455	silandrin	Estrogen receptor	P03372	ESR1	0.556
Silybum marianum(SM)	MOL007455	silandrin	Estrogen receptor beta	Q92731	ESR2	1
Orostachys fimbriata（OF）	MOL000098	quercetin	Adenosine A2b receptor	P29275	ADORA2B	0.011
Orostachys fimbriata（OF）	MOL000098	quercetin	Adenosine A3 receptor	P33765	SHMT1	0.011
Orostachys fimbriata（OF）	MOL000098	quercetin	cGMP-specific 3',5'-cyclic phosphodiesterase	O76074	PDE5A	0.012
Orostachys fimbriata（OF）	MOL000098	quercetin	Adenosine A1 receptor	P30542	ADORA1	0.012
Orostachys fimbriata（OF）	MOL000098	quercetin	cAMP-specific 3',5'-cyclic phosphodiesterase 4B	Q07343	PDE4B	0.012
Orostachys fimbriata（OF）	MOL000098	quercetin	Neuronal acetylcholine receptor subunit alpha-2	Q15822	CHRNA2	0.012
Orostachys fimbriata（OF）	MOL000098	quercetin	Potassium channel subfamily K member 1	O00180	KCNK1	0.013
Orostachys fimbriata（OF）	MOL000098	quercetin	Tyrosine 3-monooxygenase	P07101	TH	0.013
Orostachys fimbriata（OF）	MOL000098	quercetin	cGMP-inhibited 3',5'-cyclic phosphodiesterase A	Q14432	PDE3A	0.013
Orostachys fimbriata（OF）	MOL000098	quercetin	cAMP and cAMP-inhibited cGMP 3',5'-cyclic phosphodiesterase 10A	Q9Y233	PDE10A	0.013
Orostachys fimbriata（OF）	MOL000098	quercetin	Tyrosyl-tRNA synthetase, mitochondrial	Q9Y2Z4	YARS2	0.013
Orostachys fimbriata（OF）	MOL000098	quercetin	3-phosphoinositide-dependent protein kinase 1	O15530	PDPK1	0.014
Orostachys fimbriata（OF）	MOL000098	quercetin	Serine/threonine-protein kinase 17B	O94768	STK17B	0.014
Orostachys fimbriata（OF）	MOL000098	quercetin	Pepsin A	P00790	REN	0.014
Orostachys fimbriata（OF）	MOL000098	quercetin	Tyrosine-protein kinase Lyn	P07948	LYN	0.014
Orostachys fimbriata（OF）	MOL000098	quercetin	Muscarinic acetylcholine receptor M5	P08912	CHRM5	0.014
Orostachys fimbriata（OF）	MOL000098	quercetin	Cell division protein kinase 4	P11802	CDK4	0.014
Orostachys fimbriata（OF）	MOL000098	quercetin	Gamma-aminobutyric-acid receptor subunit alpha-1	P14867	GABRA1	0.014
Orostachys fimbriata（OF）	MOL000098	quercetin	Dipeptidyl peptidase 4	P27487	DPP4	0.014
Orostachys fimbriata（OF）	MOL000098	quercetin	ATP synthase subunit gamma, mitochondrial	P36542	ATP5F1C	0.014
Orostachys fimbriata（OF）	MOL000098	quercetin	Tyrosine-protein kinase CSK	P41240	CSK	0.014
Orostachys fimbriata（OF）	MOL000098	quercetin	Tyrosine-protein kinase ZAP-70	P43403	ZAP70	0.014
Orostachys fimbriata（OF）	MOL000098	quercetin	Tyrosine-protein kinase SYK	P43405	SYK	0.014
Orostachys fimbriata（OF）	MOL000098	quercetin	Gamma-aminobutyric-acid receptor subunit alpha-2	P47869	GABRA2	0.014
Orostachys fimbriata（OF）	MOL000098	quercetin	Cell division protein kinase 7	P50613	CDK7	0.014
Orostachys fimbriata（OF）	MOL000098	quercetin	Cell division protein kinase 9	P50750	CDK9	0.014
Orostachys fimbriata（OF）	MOL000098	quercetin	Cell division protein kinase 6	Q00534	CDK6	0.014
Orostachys fimbriata（OF）	MOL000098	quercetin	Protein kinase C theta type	Q04759	PRKCQ	0.014
Orostachys fimbriata（OF）	MOL000098	quercetin	Tyrosine-protein kinase ITK/TSK	Q08881	ITK	0.014
Orostachys fimbriata（OF）	MOL000098	quercetin	Cyclin-dependent kinase 5 activator 1	Q15078	CDK5R1	0.014
Orostachys fimbriata（OF）	MOL000098	quercetin	UDP-glucuronosyltransferase 3A1	Q6NUS8	UGT3A1	0.014
Orostachys fimbriata（OF）	MOL000098	quercetin	Dehydrogenase/reductase SDR family member 8	Q8NBQ5	HSD17B11	0.014
Orostachys fimbriata（OF）	MOL000098	quercetin	85 kDa calcium-independent phospholipase A2	O60733	PLA2G6	0.015
Orostachys fimbriata（OF）	MOL000098	quercetin	Ig gamma-2 chain C region	P01859	IGHG2	0.015
Orostachys fimbriata（OF）	MOL000098	quercetin	Retinoic acid receptor alpha	P10276	RARA	0.015
Orostachys fimbriata（OF）	MOL000098	quercetin	Retinoic acid receptor beta	P10826	RARB	0.015
Orostachys fimbriata（OF）	MOL000098	quercetin	Retinoic acid receptor gamma-1	P13631	RARG	0.015
Orostachys fimbriata（OF）	MOL000098	quercetin	Beta-3 adrenergic receptor	P13945	ADRB3	0.015

Orostachys fimbriata（OF）	MOL000098	quercetin	5-hydroxytryptamine 1D receptor	P28221	HTR1D	0.015
Orostachys fimbriata（OF）	MOL000098	quercetin	5-hydroxytryptamine 1B receptor	P28222	HTR1B	0.015
Orostachys fimbriata（OF）	MOL000098	quercetin	5-hydroxytryptamine 2C receptor	P28335	HTR2C	0.015
Orostachys fimbriata（OF）	MOL000098	quercetin	Amiloride-sensitive sodium channel subunit alpha	P37088	SCNN1A	0.015
Orostachys fimbriata（OF）	MOL000098	quercetin	Cytosolic phospholipase A2	P47712	PLA2G4A	0.015
Orostachys fimbriata（OF）	MOL000098	quercetin	Retinoic acid receptor RXR-gamma	P48443	RXRG	0.015
Orostachys fimbriata（OF）	MOL000098	quercetin	Amiloride-sensitive sodium channel subunit beta	P51168	SCNN1B	0.015
Orostachys fimbriata（OF）	MOL000098	quercetin	Amiloride-sensitive sodium channel subunit gamma	P51170	SCNN1G	0.015
Orostachys fimbriata（OF）	MOL000098	quercetin	Sodium/potassium-transporting ATPase gamma chain	P54710	FXYD2	0.015
Orostachys fimbriata（OF）	MOL000098	quercetin	Solute carrier family 12 member 3	P55017	SLC12A3	0.015
Orostachys fimbriata（OF）	MOL000098	quercetin	Calcium-activated potassium channel subunit alpha 1	Q12791	KCNMA1	0.015
Orostachys fimbriata（OF）	MOL000098	quercetin	5-hydroxytryptamine 3 receptor	P46098	HTR3A	0.017
Orostachys fimbriata（OF）	MOL000098	quercetin	Prostaglandin reductase 1	Q14914	PTGR1	0.017
Orostachys fimbriata（OF）	MOL000098	quercetin	Glutamate [NMDA] receptor subunit 3A	Q8TCU5	GRIN3A	0.017
Orostachys fimbriata（OF）	MOL000098	quercetin	DNA topoisomerase 1	P11387	TOP1	0.02
Orostachys fimbriata（OF）	MOL000098	quercetin	Macrophage migration inhibitory factor	P14174	MIF	0.02
Orostachys fimbriata（OF）	MOL000098	quercetin	Calcium/calmodulin-dependent protein kinase type II subunit delta	Q13557	CAMK2D	0.02
Orostachys fimbriata（OF）	MOL000098	quercetin	DNA topoisomerase I, mitochondrial	Q969P6	TOP1MT	0.02
Orostachys fimbriata（OF）	MOL000098	quercetin	STE20-like serine/threonine-protein kinase	Q9H2G2	SLK	0.02
Orostachys fimbriata（OF）	MOL000098	quercetin	Tripartite motif-containing protein 13	O60858	TRIM13	0.021
Orostachys fimbriata（OF）	MOL000098	quercetin	Chymase	P23946	CMA1	0.021
Orostachys fimbriata（OF）	MOL000098	quercetin	DNA-(apurinic or apyrimidinic site) lyase	P27695	APEX1	0.021
Orostachys fimbriata（OF）	MOL000098	quercetin	Succinate dehydrogenase [ubiquinone] cytochrome b small subunit, mitochondrial	O14521	SDHD	0.022
Orostachys fimbriata（OF）	MOL000098	quercetin	Inhibitor of nuclear factor kappa-B kinase subunit beta	O14920	IKBKB	0.022
Orostachys fimbriata（OF）	MOL000098	quercetin	Ribosyldihydronicotinamide dehydrogenase [quinone]	P16083	NQO2	0.022
Orostachys fimbriata（OF）	MOL000098	quercetin	Peptidyl-prolyl cis-trans isomerase, mitochondrial	P30405	PPIF	0.022
Orostachys fimbriata（OF）	MOL000098	quercetin	Prostacyclin receptor	P43119	PTGIR	0.022
Orostachys fimbriata（OF）	MOL000098	quercetin	Mitogen-activated protein kinase 8	P45983	MAPK8	0.022
Orostachys fimbriata（OF）	MOL000098	quercetin	Mitogen-activated protein kinase 10	P53779	MAPK10	0.022
Orostachys fimbriata（OF）	MOL000098	quercetin	Peroxisome proliferator-activated receptor delta	Q03181	PPARD	0.022
Orostachys fimbriata（OF）	MOL000098	quercetin	Group IIE secretory phospholipase A2	Q9NZK7	PLA2G2E	0.022
Orostachys fimbriata（OF）	MOL000098	quercetin	Cystine/glutamate transporter	Q9UPY5	SLC7A11	0.022
Orostachys fimbriata（OF）	MOL000098	quercetin	Oxysterols receptor LXR-beta	P55055	NR1H2	0.023
Orostachys fimbriata（OF）	MOL000098	quercetin	Oxysterols receptor LXR-alpha	Q13133	NR1H3	0.023
Orostachys fimbriata（OF）	MOL000098	quercetin	Sodium channel protein type 5 subunit alpha	Q14524	SCN5A	0.023
Orostachys fimbriata（OF）	MOL000098	quercetin	Phenylalanine-4-hydroxylase	P00439	PAH	0.024
Orostachys fimbriata（OF）	MOL000098	quercetin	Rho-associated protein kinase 1	Q13464	ROCK1	0.024
Orostachys fimbriata（OF）	MOL000098	quercetin	Alpha-1D adrenergic receptor	P25100	ADRA1D	0.025
Orostachys fimbriata（OF）	MOL000098	quercetin	D(4) dopamine receptor	P21917	DRD4	0.026
Orostachys fimbriata（OF）	MOL000098	quercetin	Keratin, type II cytoskeletal 7	P08729	KRT7	0.028
Orostachys fimbriata（OF）	MOL000098	quercetin	Platelet glycoprotein IX	P14770	GP9	0.028
Orostachys fimbriata（OF）	MOL000098	quercetin	Adenosine A2a receptor	P29274	ADORA2A	0.029
Orostachys fimbriata（OF）	MOL000098	quercetin	Sterol O-acyltransferase 2	O75908	SOAT2	0.031
Orostachys fimbriata（OF）	MOL000098	quercetin	Sterol O-acyltransferase 1	P35610	SOAT1	0.031
Orostachys fimbriata（OF）	MOL000098	quercetin	Tubulin beta-2C chain	P68371	TUBB4B	0.031
Orostachys fimbriata（OF）	MOL000098	quercetin	3 beta-hydroxysteroid dehydrogenase/Delta 5-->4-isomerase type II	P26439	HSD3B2	0.032
Orostachys fimbriata（OF）	MOL000098	quercetin	Protein tyrosine kinase 2 beta	Q14289	PTK2B	0.032

Orostachys fimbriata（OF）	MOL000098	quercetin	Tyrosyl-tRNA synthetase, cytoplasmic	P54577	YARS	0.033
Orostachys fimbriata（OF）	MOL000098	quercetin	ATP synthase subunit beta, mitochondrial	P06576	ATP5F1B	0.034
Orostachys fimbriata（OF）	MOL000098	quercetin	Muscarinic acetylcholine receptor M4	P08173	CHRM4	0.034
Orostachys fimbriata（OF）	MOL000098	quercetin	Tyrosine-protein kinase HCK	P08631	HCK	0.034
Orostachys fimbriata（OF）	MOL000098	quercetin	Proto-oncogene serine/threonine-protein kinase Pim-1	P11309	PIM1	0.034
Orostachys fimbriata（OF）	MOL000098	quercetin	Muscarinic acetylcholine receptor M3	P20309	CHRM3	0.034
Orostachys fimbriata（OF）	MOL000098	quercetin	ATP synthase subunit alpha, mitochondrial	P25705	ATP5F1A	0.034
Orostachys fimbriata（OF）	MOL000098	quercetin	Phosphatidylinositol-4,5-bisphosphate 3-kinase catalytic subunit gamma isoform	P48736	PIK3CG	0.034
Orostachys fimbriata（OF）	MOL000098	quercetin	Epidermal growth factor receptor	P00533	EGFR	0.035
Orostachys fimbriata（OF）	MOL000098	quercetin	Carbonic anhydrase 1	P00915	CA1	0.035
Orostachys fimbriata（OF）	MOL000098	quercetin	Proto-oncogene tyrosine-protein kinase LCK	P06239	LCK	0.035
Orostachys fimbriata（OF）	MOL000098	quercetin	5-hydroxytryptamine 1A receptor	P08908	HTR1A	0.035
Orostachys fimbriata（OF）	MOL000098	quercetin	Glycogen phosphorylase, muscle form	P11217	PYGM	0.035
Orostachys fimbriata（OF）	MOL000098	quercetin	Alpha-1B adrenergic receptor	P35368	ADRA1B	0.035
Orostachys fimbriata（OF）	MOL000098	quercetin	Bile salt sulfotransferase	Q06520	SULT2A1	0.036
Orostachys fimbriata（OF）	MOL000098	quercetin	Ig kappa chain C region	P01834	IGKC	0.037
Orostachys fimbriata（OF）	MOL000098	quercetin	Ig gamma-1 chain C region	P01857	IGHG1	0.037
Orostachys fimbriata（OF）	MOL000098	quercetin	D(2) dopamine receptor	P14416	DRD2	0.037
Orostachys fimbriata（OF）	MOL000098	quercetin	D(3) dopamine receptor	P35462	DRD3	0.037
Orostachys fimbriata（OF）	MOL000098	quercetin	Estrogen-related receptor gamma	P62508	ESRRG	0.037
Orostachys fimbriata（OF）	MOL000098	quercetin	Muscarinic acetylcholine receptor M2	P08172	CHRM2	0.044
Orostachys fimbriata（OF）	MOL000098	quercetin	Muscarinic acetylcholine receptor M1	P11229	CHRM1	0.044
Orostachys fimbriata（OF）	MOL000098	quercetin	Retinoic acid receptor RXR-beta	P28702	RXRB	0.044
Orostachys fimbriata（OF）	MOL000098	quercetin	Aldo-keto reductase family 1 member C1	Q04828	AKR1C1	0.044
Orostachys fimbriata（OF）	MOL000098	quercetin	Alpha-2A adrenergic receptor	P08913	ADRA2A	0.045
Orostachys fimbriata（OF）	MOL000098	quercetin	Cytochrome P450 19A1	P11511	CYP19A1	0.045
Orostachys fimbriata（OF）	MOL000098	quercetin	Alpha-2C adrenergic receptor	P18825	ADRA2C	0.045
Orostachys fimbriata（OF）	MOL000098	quercetin	Alpha-1A adrenergic receptor	P35348	ADRA1A	0.045
Orostachys fimbriata（OF）	MOL000098	quercetin	Kynurenine--oxoglutarate transaminase 1	Q16773	KYAT1	0.045
Orostachys fimbriata（OF）	MOL000098	quercetin	Membrane copper amine oxidase	Q16853	AOC3	0.045
Orostachys fimbriata（OF）	MOL000098	quercetin	NADPH oxidase organizer 1	Q8NFA2	NOXO1	0.045
Orostachys fimbriata（OF）	MOL000098	quercetin	Serine/threonine-protein kinase 6	O14965	AURKA	0.046
Orostachys fimbriata（OF）	MOL000098	quercetin	Thyroid hormone receptor beta-1	P10828	THRB	0.046
Orostachys fimbriata（OF）	MOL000098	quercetin	Egl nine homolog 1	Q9GZT9	EGLN1	0.046
Orostachys fimbriata（OF）	MOL000098	quercetin	Tubulin beta-1 chain	Q9H4B7	TUBB1	0.046
Orostachys fimbriata（OF）	MOL000098	quercetin	Cannabinoid receptor 1	P21554	CNR1	0.047
Orostachys fimbriata（OF）	MOL000098	quercetin	D(1A) dopamine receptor	P21728	DRD1	0.047
Orostachys fimbriata（OF）	MOL000098	quercetin	D(1B) dopamine receptor	P21918	DRD5	0.047
Orostachys fimbriata（OF）	MOL000098	quercetin	Endothelin-1 receptor	P25101	EDNRA	0.047
Orostachys fimbriata（OF）	MOL000098	quercetin	Thiamin pyrophosphokinase 1	Q9H3S4	TPK1	0.047
Orostachys fimbriata（OF）	MOL000098	quercetin	Glucocorticoid receptor	P04150	NR3C1	0.048
Orostachys fimbriata（OF）	MOL000098	quercetin	Ig kappa chain V-II region RPMI 6410	P06310	IGKV2-30	0.048
Orostachys fimbriata（OF）	MOL000098	quercetin	Leukotriene A-4 hydrolase	P09960	LTA4H	0.048
Orostachys fimbriata（OF）	MOL000098	quercetin	RAC-alpha serine/threonine-protein kinase	P31749	AKT1	0.048
Orostachys fimbriata（OF）	MOL000098	quercetin	Nitric oxide synthase, inducible	P35228	NOS2	0.048
Orostachys fimbriata（OF）	MOL000098	quercetin	S-methyl-5-thioadenosine phosphorylase	Q13126	MTAP	0.048
Orostachys fimbriata（OF）	MOL000098	quercetin	cAMP-specific 3',5'-cyclic phosphodiesterase 4A	P27815	PDE4A	0.049

Orostachys fimbriata（OF）	MOL000098	quercetin	Triosephosphate isomerase	P60174	TPI1	0.049
Orostachys fimbriata（OF）	MOL000098	quercetin	Solute carrier family 12 member 1	Q13621	SLC12A1	0.049
Orostachys fimbriata（OF）	MOL000098	quercetin	Thyroid hormone receptor alpha	P10827	THRA	0.05
Orostachys fimbriata（OF）	MOL000098	quercetin	Calmodulin	P62158		0.05
Orostachys fimbriata（OF）	MOL000098	quercetin	Casein kinase II subunit alpha	P68400	CSNK2A1	0.05
Orostachys fimbriata（OF）	MOL000098	quercetin	Neuropeptide Y	P01303	NPY	0.051
Orostachys fimbriata（OF）	MOL000098	quercetin	Peroxisome proliferator-activated receptor gamma	P37231	PPARG	0.051
Orostachys fimbriata（OF）	MOL000098	quercetin	Nuclear receptor coactivator 2	Q15596	NCOA2	0.051
Orostachys fimbriata（OF）	MOL000098	quercetin	Toll-like receptor 7	Q9NYK1	TLR7	0.051
Orostachys fimbriata（OF）	MOL000098	quercetin	C-jun-amino-terminal kinase-interacting protein 1	Q9UQF2	MAPK8IP1	0.051
Orostachys fimbriata（OF）	MOL000098	quercetin	cAMP-dependent protein kinase inhibitor alpha	P61925	PKIA	0.053
Orostachys fimbriata（OF）	MOL000098	quercetin	Interferon gamma	P01579	IFNG	0.055
Orostachys fimbriata（OF）	MOL000098	quercetin	Fibroblast growth factor receptor 2	P21802	FGFR2	0.055
Orostachys fimbriata（OF）	MOL000098	quercetin	Sodium-dependent noradrenaline transporter	P23975	SLC6A2	0.055
Orostachys fimbriata（OF）	MOL000098	quercetin	MAP kinase-activated protein kinase 2	P49137	MAPKAPK2	0.055
Orostachys fimbriata（OF）	MOL000098	quercetin	Glycogen synthase kinase-3 beta	P49841	GSK3B	0.055
Orostachys fimbriata（OF）	MOL000098	quercetin	Sodium-dependent dopamine transporter	Q01959	SLC6A3	0.055
Orostachys fimbriata（OF）	MOL000098	quercetin	Nuclear receptor coactivator 5	Q9HCD5	NCOA5	0.055
Orostachys fimbriata（OF）	MOL000098	quercetin	Carbonic anhydrase 2	P00918	CA2	0.056
Orostachys fimbriata（OF）	MOL000098	quercetin	Alpha-2B adrenergic receptor	P18089	ADRA2B	0.056
Orostachys fimbriata（OF）	MOL000098	quercetin	Proto-oncogene tyrosine-protein kinase Src	P12931	SRC	0.057
Orostachys fimbriata（OF）	MOL000098	quercetin	DNA polymerase kappa	Q9UBT6	POLK	0.058
Orostachys fimbriata（OF）	MOL000098	quercetin	DNA topoisomerase 2-alpha	P11388	TOP2A	0.059
Orostachys fimbriata（OF）	MOL000098	quercetin	3 beta-hydroxysteroid dehydrogenase/Delta 5-->4-isomerase type I	P14060	HSD3B1	0.061
Orostachys fimbriata（OF）	MOL000098	quercetin	Beta-2 adrenergic receptor	P07550	ADRB2	0.066
Orostachys fimbriata（OF）	MOL000098	quercetin	Beta-1 adrenergic receptor	P08588	ADRB1	0.066
Orostachys fimbriata（OF）	MOL000098	quercetin	Tubulin alpha-3 chain	Q71U36	TUBA1A	0.067
Orostachys fimbriata（OF）	MOL000098	quercetin	Prothrombin	P00734	F2	0.07
Orostachys fimbriata（OF）	MOL000098	quercetin	Estradiol 17-beta-dehydrogenase 1	P14061	HSD17B1	0.071
Orostachys fimbriata（OF）	MOL000098	quercetin	Delta-type opioid receptor	P41143	OPRD1	0.073
Orostachys fimbriata（OF）	MOL000098	quercetin	Kappa-type opioid receptor	P41145	OPRK1	0.073
Orostachys fimbriata（OF）	MOL000098	quercetin	D-HSCDK2	O75100	CA11	0.075
Orostachys fimbriata（OF）	MOL000098	quercetin	Sodium-dependent serotonin transporter	P31645	SLC6A4	0.075
Orostachys fimbriata（OF）	MOL000098	quercetin	Mineralocorticoid receptor	P08235	NR3C2	0.077
Orostachys fimbriata（OF）	MOL000098	quercetin	Inhibitor of nuclear factor kappa-B kinase subunit alpha	O15111	CHUK	0.079
Orostachys fimbriata（OF）	MOL000098	quercetin	Arachidonate 5-lipoxygenase	P09917	ALOX5	0.079
Orostachys fimbriata（OF）	MOL000098	quercetin	cAMP-dependent protein kinase catalytic subunit alpha	P17612	PRKACA	0.083
Orostachys fimbriata（OF）	MOL000098	quercetin	Dihydrofolate reductase	P00374	DHFR	0.091
Orostachys fimbriata（OF）	MOL000098	quercetin	Cell division control protein 2 homolog	P06493	CDK1	0.095
Orostachys fimbriata（OF）	MOL000098	quercetin	Cell division protein kinase 5	Q00535	CDK5	0.115
Orostachys fimbriata（OF）	MOL000098	quercetin	Trypsin-1	P07477	PRSS1	0.138
Orostachys fimbriata（OF）	MOL000098	quercetin	Cyclin-A2	P20248	CCNA2	0.151
Orostachys fimbriata（OF）	MOL000098	quercetin	Cell division protein kinase 2	P24941	CDK2	0.156
Orostachys fimbriata（OF）	MOL000098	quercetin	Hemoglobin subunit alpha	P69905	HBA1	0.169
Orostachys fimbriata（OF）	MOL000098	quercetin	Mu-type opioid receptor	P35372	OPRM1	0.179
Orostachys fimbriata（OF）	MOL000098	quercetin	Progesterone receptor	P06401	PGR	0.2
Orostachys fimbriata（OF）	MOL000098	quercetin	Nuclear receptor coactivator 1	Q15788	NCOA1	0.205

Orostachys fimbriata（OF）	MOL000098	quercetin	Prostaglandin G/H synthase 1	P23219	PTGS1	0.22
Orostachys fimbriata（OF）	MOL000098	quercetin	Estrogen receptor beta	Q92731	ESR2	0.357
Orostachys fimbriata（OF）	MOL000098	quercetin	Prostaglandin G/H synthase 2	P35354	PTGS2	0.39
Orostachys fimbriata（OF）	MOL000098	quercetin	Estrogen receptor	P03372	ESR1	1
Orostachys fimbriata（OF）	MOL000358	beta-sitosterol	Retinoic acid receptor RXR-gamma	P48443	RXRG	0.01
Orostachys fimbriata（OF）	MOL000358	beta-sitosterol	Nuclear receptor 0B1	P51843	NR0B1	0.01
Orostachys fimbriata（OF）	MOL000358	beta-sitosterol	Retinoic acid-induced protein 3	Q8NFJ5	GPRC5A	0.01
Orostachys fimbriata（OF）	MOL000358	beta-sitosterol	Retinoic acid receptor alpha	P10276	RARA	0.023
Orostachys fimbriata（OF）	MOL000358	beta-sitosterol	Retinoic acid receptor beta	P10826	RARB	0.023
Orostachys fimbriata（OF）	MOL000358	beta-sitosterol	Retinal dehydrogenase 2	O94788	ALDH1A2	0.029
Orostachys fimbriata（OF）	MOL000358	beta-sitosterol	Retinal dehydrogenase 1	P00352	ALDH1A1	0.029
Orostachys fimbriata（OF）	MOL000358	beta-sitosterol	Potassium channel subfamily K member 1	O00180	KCNK1	0.036
Orostachys fimbriata（OF）	MOL000358	beta-sitosterol	Sodium channel protein type 5 subunit alpha	Q14524	SCN5A	0.036
Orostachys fimbriata（OF）	MOL000358	beta-sitosterol	Potassium channel subfamily K member 6	Q9Y257	KCNK6	0.036
Orostachys fimbriata（OF）	MOL000358	beta-sitosterol	Serine/threonine-protein phosphatase 2A 65 kDa regulatory subunit A alpha isoform	P30153	PPP2R1A	0.038
Orostachys fimbriata（OF）	MOL000358	beta-sitosterol	Serine/threonine-protein phosphatase PP1-alpha catalytic subunit	P62136	PPP1CA	0.038
Orostachys fimbriata（OF）	MOL000358	beta-sitosterol	Ig kappa chain C region	P01834	IGKC	0.043
Orostachys fimbriata（OF）	MOL000358	beta-sitosterol	Ig gamma-1 chain C region	P01857	IGHG1	0.043
Orostachys fimbriata（OF）	MOL000358	beta-sitosterol	Ig gamma-2 chain C region	P01859	IGHG2	0.043
Orostachys fimbriata（OF）	MOL000358	beta-sitosterol	Nuclear receptor subfamily 1 group I member 3	Q14994	NR1I3	0.05
Orostachys fimbriata（OF）	MOL000358	beta-sitosterol	Retinoic acid receptor gamma-1	P13631	RARG	0.052
Orostachys fimbriata（OF）	MOL000358	beta-sitosterol	Retinoic acid receptor RXR-beta	P28702	RXRB	0.052
Orostachys fimbriata（OF）	MOL000358	beta-sitosterol	Bile salt sulfotransferase	Q06520	SULT2A1	0.056
Orostachys fimbriata（OF）	MOL000358	beta-sitosterol	Dehydrogenase/reductase SDR family member 8	Q8NBQ5	HSD17B11	0.056
Orostachys fimbriata（OF）	MOL000358	beta-sitosterol	Platelet glycoprotein IX	P14770	GP9	0.063
Orostachys fimbriata（OF）	MOL000358	beta-sitosterol	Phospholipase A2	P04054	PLA2G1B	0.069
Orostachys fimbriata（OF）	MOL000358	beta-sitosterol	Phospholipase A2, membrane associated	P14555	PLA2G2A	0.069
Orostachys fimbriata（OF）	MOL000358	beta-sitosterol	3-oxo-5-alpha-steroid 4-dehydrogenase 1	P18405	SRD5A1	0.074
Orostachys fimbriata（OF）	MOL000358	beta-sitosterol	3-oxo-5-alpha-steroid 4-dehydrogenase 2	P31213	SRD5A2	0.074
Orostachys fimbriata（OF）	MOL000358	beta-sitosterol	3 beta-hydroxysteroid dehydrogenase/Delta 5-->4-isomerase type II	P26439	HSD3B2	0.079
Orostachys fimbriata（OF）	MOL000358	beta-sitosterol	Microtubule-associated protein 2	P11137	MAP2	0.08
Orostachys fimbriata（OF）	MOL000358	beta-sitosterol	Microtubule-associated protein 1A	P78559	MAP1A	0.08
Orostachys fimbriata（OF）	MOL000358	beta-sitosterol	Gonadotropin-releasing hormone receptor	P30968	GNRHR	0.082
Orostachys fimbriata（OF）	MOL000358	beta-sitosterol	Gonadotropin-releasing hormone II receptor	Q96P88	GNRHR2	0.082
Orostachys fimbriata（OF）	MOL000358	beta-sitosterol	Corticosteroid 11-beta-dehydrogenase isozyme 1	P28845	HSD11B1	0.084
Orostachys fimbriata（OF）	MOL000358	beta-sitosterol	Cytosolic phospholipase A2	P47712	PLA2G4A	0.084
Orostachys fimbriata（OF）	MOL000358	beta-sitosterol	Retinoic acid receptor RXR-alpha	P19793	RXRA	0.092
Orostachys fimbriata（OF）	MOL000358	beta-sitosterol	Serine/threonine-protein phosphatase 2A catalytic subunit alpha isoform	P67775	PPP2CA	0.095
Orostachys fimbriata（OF）	MOL000358	beta-sitosterol	Serine/threonine-protein phosphatase 2A 56 kDa regulatory subunit gamma isoform	Q13362	PPP2R5C	0.095
Orostachys fimbriata（OF）	MOL000358	beta-sitosterol	Sodium-dependent serotonin transporter	P31645	SLC6A4	0.146
Orostachys fimbriata（OF）	MOL000358	beta-sitosterol	Aldo-keto reductase family 1 member C1	Q04828	AKR1C1	0.147
Orostachys fimbriata（OF）	MOL000358	beta-sitosterol	Prostaglandin G/H synthase 2	P35354	PTGS2	0.15
Orostachys fimbriata（OF）	MOL000358	beta-sitosterol	Estrogen receptor beta	Q92731	ESR2	0.163
Orostachys fimbriata（OF）	MOL000358	beta-sitosterol	Mediator of RNA polymerase II transcription subunit 1	Q15648	MED1	0.164
Orostachys fimbriata（OF）	MOL000358	beta-sitosterol	Estradiol 17-beta-dehydrogenase 1	P14061	HSD17B1	0.173
Orostachys fimbriata（OF）	MOL000358	beta-sitosterol	3 beta-hydroxysteroid dehydrogenase/Delta 5-->4-isomerase type I	P14060	HSD3B1	0.175
Orostachys fimbriata（OF）	MOL000358	beta-sitosterol	Nuclear receptor coactivator 5	Q9HCD5	NCOA5	0.178

Orostachys fimbriata（OF）	MOL000358	beta-sitosterol	Androgen receptor	P10275	AR	0.184
Orostachys fimbriata（OF）	MOL000358	beta-sitosterol	Glucocorticoid receptor	P04150	NR3C1	0.187
Orostachys fimbriata（OF）	MOL000358	beta-sitosterol	Cannabinoid receptor 2	P34972	CNR2	0.191
Orostachys fimbriata（OF）	MOL000358	beta-sitosterol	Nuclear receptor coactivator 1	Q15788	NCOA1	0.296
Orostachys fimbriata（OF）	MOL000358	beta-sitosterol	Mineralocorticoid receptor	P08235	NR3C2	0.39
Orostachys fimbriata（OF）	MOL000358	beta-sitosterol	Estrogen receptor	P03372	ESR1	0.971
Orostachys fimbriata（OF）	MOL000358	beta-sitosterol	Progesterone receptor	P06401	PGR	1
Orostachys fimbriata（OF）	MOL000359	sitosterol	Retinoic acid receptor RXR-alpha	P19793	RXRA	0.016
Orostachys fimbriata（OF）	MOL000359	sitosterol	Nuclear receptor subfamily 1 group I member 3	Q14994	NR1I3	0.016
Orostachys fimbriata（OF）	MOL000359	sitosterol	Elongation factor Tu GTP-binding domain-containing protein 1	Q7Z2Z2	EFL1	0.026
Orostachys fimbriata（OF）	MOL000359	sitosterol	Potassium channel subfamily K member 1	O00180	KCNK1	0.032
Orostachys fimbriata（OF）	MOL000359	sitosterol	D-HSCDK2	O75100	CA11	0.032
Orostachys fimbriata（OF）	MOL000359	sitosterol	Cell division control protein 2 homolog	P06493	CDK1	0.032
Orostachys fimbriata（OF）	MOL000359	sitosterol	Cell division protein kinase 5	Q00535	CDK5	0.032
Orostachys fimbriata（OF）	MOL000359	sitosterol	Sodium channel protein type 5 subunit alpha	Q14524	SCN5A	0.032
Orostachys fimbriata（OF）	MOL000359	sitosterol	Potassium channel subfamily K member 6	Q9Y257	KCNK6	0.032
Orostachys fimbriata（OF）	MOL000359	sitosterol	Neuronal acetylcholine receptor subunit alpha-3	P32297	CHRNA3	0.033
Orostachys fimbriata（OF）	MOL000359	sitosterol	Neuronal acetylcholine receptor subunit alpha-7	P36544	CHRNA7	0.033
Orostachys fimbriata（OF）	MOL000359	sitosterol	Neuronal acetylcholine receptor subunit alpha-4	P43681	CHRNA4	0.033
Orostachys fimbriata（OF）	MOL000359	sitosterol	Phosphatidylinositol 3-kinase regulatory subunit alpha	P27986	PIK3R1	0.034
Orostachys fimbriata（OF）	MOL000359	sitosterol	Phosphatidylinositol-4,5-bisphosphate 3-kinase catalytic subunit gamma isoform	P48736	PIK3CG	0.034
Orostachys fimbriata（OF）	MOL000359	sitosterol	Serine/threonine-protein kinase PLK1	P53350	PLK1	0.034
Orostachys fimbriata（OF）	MOL000359	sitosterol	Dehydrogenase/reductase SDR family member 8	Q8NBQ5	HSD17B11	0.041
Orostachys fimbriata（OF）	MOL000359	sitosterol	Ig kappa chain C region	P01834	IGKC	0.042
Orostachys fimbriata（OF）	MOL000359	sitosterol	Ig gamma-1 chain C region	P01857	IGHG1	0.042
Orostachys fimbriata（OF）	MOL000359	sitosterol	Ig gamma-2 chain C region	P01859	IGHG2	0.042
Orostachys fimbriata（OF）	MOL000359	sitosterol	Platelet glycoprotein IX	P14770	GP9	0.054
Orostachys fimbriata（OF）	MOL000359	sitosterol	Phospholipase A2	P04054	PLA2G1B	0.057
Orostachys fimbriata（OF）	MOL000359	sitosterol	Phospholipase A2, membrane associated	P14555	PLA2G2A	0.057
Orostachys fimbriata（OF）	MOL000359	sitosterol	Annexin A1	P04083	ANXA1	0.062
Orostachys fimbriata（OF）	MOL000359	sitosterol	Nuclear receptor 0B1	P51843	NR0B1	0.062
Orostachys fimbriata（OF）	MOL000359	sitosterol	3 beta-hydroxysteroid dehydrogenase/Delta 5-->4-isomerase type II	P26439	HSD3B2	0.064
Orostachys fimbriata（OF）	MOL000359	sitosterol	Cytosolic phospholipase A2	P47712	PLA2G4A	0.066
Orostachys fimbriata（OF）	MOL000359	sitosterol	Corticosteroid 11-beta-dehydrogenase isozyme 1	P28845	HSD11B1	0.067
Orostachys fimbriata（OF）	MOL000359	sitosterol	3-oxo-5-alpha-steroid 4-dehydrogenase 2	P31213	SRD5A2	0.067
Orostachys fimbriata（OF）	MOL000359	sitosterol	Microtubule-associated protein 2	P11137	MAP2	0.068
Orostachys fimbriata（OF）	MOL000359	sitosterol	Prolactin receptor	P16471	PRLR	0.068
Orostachys fimbriata（OF）	MOL000359	sitosterol	Gonadotropin-releasing hormone receptor	P30968	GNRHR	0.068
Orostachys fimbriata（OF）	MOL000359	sitosterol	Microtubule-associated protein 1A	P78559	MAP1A	0.068
Orostachys fimbriata（OF）	MOL000359	sitosterol	Gonadotropin-releasing hormone II receptor	Q96P88	GNRHR2	0.068
Orostachys fimbriata（OF）	MOL000359	sitosterol	Nuclear receptor coactivator 1	Q15788	NCOA1	0.069
Orostachys fimbriata（OF）	MOL000359	sitosterol	Bile salt sulfotransferase	Q06520	SULT2A1	0.091
Orostachys fimbriata（OF）	MOL000359	sitosterol	Prostaglandin G/H synthase 2	P35354	PTGS2	0.115
Orostachys fimbriata（OF）	MOL000359	sitosterol	DNA polymerase kappa	Q9UBT6	POLK	0.115
Orostachys fimbriata（OF）	MOL000359	sitosterol	Nuclear receptor coactivator 2	Q15596	NCOA2	0.116
Orostachys fimbriata（OF）	MOL000359	sitosterol	Aldo-keto reductase family 1 member C1	Q04828	AKR1C1	0.125
Orostachys fimbriata（OF）	MOL000359	sitosterol	ATP-binding cassette transporter sub-family C member 8	Q09428	ABCC8	0.127

Orostachys fimbriata（OF）	MOL000359	sitosterol	3 beta-hydroxysteroid dehydrogenase/Delta 5-->4-isomerase type I	P14060	HSD3B1	0.128
Orostachys fimbriata（OF）	MOL000359	sitosterol	Nitric-oxide synthase, endothelial	P29474	NOS3	0.134
Orostachys fimbriata（OF）	MOL000359	sitosterol	Cannabinoid receptor 2	P34972	CNR2	0.136
Orostachys fimbriata（OF）	MOL000359	sitosterol	3-oxo-5-alpha-steroid 4-dehydrogenase 1	P18405	SRD5A1	0.145
Orostachys fimbriata（OF）	MOL000359	sitosterol	Nuclear receptor coactivator 5	Q9HCD5	NCOA5	0.15
Orostachys fimbriata（OF）	MOL000359	sitosterol	Estradiol 17-beta-dehydrogenase 1	P14061	HSD17B1	0.221
Orostachys fimbriata（OF）	MOL000359	sitosterol	Androgen receptor	P10275	AR	0.224
Orostachys fimbriata（OF）	MOL000359	sitosterol	Estrogen receptor beta	Q92731	ESR2	0.246
Orostachys fimbriata（OF）	MOL000359	sitosterol	Glucocorticoid receptor	P04150	NR3C1	0.288
Orostachys fimbriata（OF）	MOL000359	sitosterol	Mineralocorticoid receptor	P08235	NR3C2	0.376
Orostachys fimbriata（OF）	MOL000359	sitosterol	Estrogen receptor	P03372	ESR1	0.93
Orostachys fimbriata（OF）	MOL000359	sitosterol	Progesterone receptor	P06401	PGR	1
Orostachys fimbriata（OF）	MOL000422	kaempferol	Substance-P receptor	P25103	TACR1	0.01
Orostachys fimbriata（OF）	MOL000422	kaempferol	5-hydroxytryptamine 3 receptor	P46098	HTR3A	0.01
Orostachys fimbriata（OF）	MOL000422	kaempferol	Sodium channel protein type 10 subunit alpha	Q9Y5Y9	SCN10A	0.01
Orostachys fimbriata（OF）	MOL000422	kaempferol	Phosphatidylinositol 3-kinase regulatory subunit beta	O00459	PIK3R2	0.011
Orostachys fimbriata（OF）	MOL000422	kaempferol	Death-associated protein kinase 3	O43293	DAPK3	0.011
Orostachys fimbriata（OF）	MOL000422	kaempferol	Tyrosine-protein kinase JAK2	O60674	JAK2	0.011
Orostachys fimbriata（OF）	MOL000422	kaempferol	T-cell receptor alpha chain C region	P01848	TRAC	0.011
Orostachys fimbriata（OF）	MOL000422	kaempferol	T-cell receptor beta chain C region	P01850	TRBC1	0.011
Orostachys fimbriata（OF）	MOL000422	kaempferol	Trypsin-2	P07478	PRSS2	0.011
Orostachys fimbriata（OF）	MOL000422	kaempferol	Elongation factor 2	P13639	EEF2	0.011
Orostachys fimbriata（OF）	MOL000422	kaempferol	Beta-3 adrenergic receptor	P13945	ADRB3	0.011
Orostachys fimbriata（OF）	MOL000422	kaempferol	Tyrosine-protein kinase JAK1	P23458	JAK1	0.011
Orostachys fimbriata（OF）	MOL000422	kaempferol	Phosphatidylinositol 3-kinase regulatory subunit alpha	P27986	PIK3R1	0.011
Orostachys fimbriata（OF）	MOL000422	kaempferol	Mitogen-activated protein kinase 1	P28482	MAPK1	0.011
Orostachys fimbriata（OF）	MOL000422	kaempferol	Tyrosine-protein kinase JAK3	P52333	JAK3	0.011
Orostachys fimbriata（OF）	MOL000422	kaempferol	Beta-2-microglobulin	P61769	B2M	0.011
Orostachys fimbriata（OF）	MOL000422	kaempferol	Poly [ADP-ribose] polymerase 3	Q9Y6F1	PARP3	0.011
Orostachys fimbriata（OF）	MOL000422	kaempferol	Potassium channel subfamily K member 6	Q9Y257	KCNK6	0.012
Orostachys fimbriata（OF）	MOL000422	kaempferol	Gamma-aminobutyric acid receptor subunit rho-3	A8MPY1	GABRR3	0.013
Orostachys fimbriata（OF）	MOL000422	kaempferol	Gamma-aminobutyric acid receptor subunit pi	O00591	GABRP	0.013
Orostachys fimbriata（OF）	MOL000422	kaempferol	Gamma-aminobutyric acid receptor subunit delta	O14764	GABRD	0.013
Orostachys fimbriata（OF）	MOL000422	kaempferol	Gamma-aminobutyric-acid receptor subunit beta-1	P18505	GABRB1	0.013
Orostachys fimbriata（OF）	MOL000422	kaempferol	Gamma-aminobutyric acid receptor subunit gamma-2	P18507	GABRG2	0.013
Orostachys fimbriata（OF）	MOL000422	kaempferol	Gamma-aminobutyric-acid receptor subunit rho-1	P24046	GABRR1	0.013
Orostachys fimbriata（OF）	MOL000422	kaempferol	Gamma-aminobutyric acid receptor subunit rho-2	P28476	GABRR2	0.013
Orostachys fimbriata（OF）	MOL000422	kaempferol	Gamma-aminobutyric-acid receptor subunit alpha-5	P31644	GABRA5	0.013
Orostachys fimbriata（OF）	MOL000422	kaempferol	Gamma-aminobutyric-acid receptor subunit alpha-3	P34903	GABRA3	0.013
Orostachys fimbriata（OF）	MOL000422	kaempferol	Gamma-aminobutyric acid receptor subunit epsilon	P78334	GABRE	0.013
Orostachys fimbriata（OF）	MOL000422	kaempferol	Gamma-aminobutyric acid receptor subunit gamma-1	Q8N1C3	GABRG1	0.013
Orostachys fimbriata（OF）	MOL000422	kaempferol	Gamma-aminobutyric acid receptor subunit gamma-3	Q99928	GABRG3	0.013
Orostachys fimbriata（OF）	MOL000422	kaempferol	Sucrase-isomaltase, intestinal	P14410	SI	0.014
Orostachys fimbriata（OF）	MOL000422	kaempferol	Interleukin-3	P08700	IL3	0.015
Orostachys fimbriata（OF）	MOL000422	kaempferol	Protein S100-A12	P80511	S100A12	0.015
Orostachys fimbriata（OF）	MOL000422	kaempferol	Protein S100-A13	Q99584	S100A13	0.015
Orostachys fimbriata（OF）	MOL000422	kaempferol	Phenylalanine-4-hydroxylase	P00439	PAH	0.016

Orostachys fimbriata（OF）	MOL000422	kaempferol	Cell division protein kinase 4	P11802	CDK4	0.017
Orostachys fimbriata（OF）	MOL000422	kaempferol	Cell division protein kinase 7	P50613	CDK7	0.017
Orostachys fimbriata（OF）	MOL000422	kaempferol	Cell division protein kinase 9	P50750	CDK9	0.017
Orostachys fimbriata（OF）	MOL000422	kaempferol	Cell division protein kinase 6	Q00534	CDK6	0.017
Orostachys fimbriata（OF）	MOL000422	kaempferol	Serine/threonine-protein kinase 17B	O94768	STK17B	0.018
Orostachys fimbriata（OF）	MOL000422	kaempferol	Proto-oncogene serine/threonine-protein kinase Pim-1	P11309	PIM1	0.018
Orostachys fimbriata（OF）	MOL000422	kaempferol	ATP synthase subunit gamma, mitochondrial	P36542	ATP5F1C	0.018
Orostachys fimbriata（OF）	MOL000422	kaempferol	Neuronal acetylcholine receptor subunit alpha-2	Q15822	CHRNA2	0.018
Orostachys fimbriata（OF）	MOL000422	kaempferol	UDP-glucuronosyltransferase 3A1	Q6NUS8	UGT3A1	0.018
Orostachys fimbriata（OF）	MOL000422	kaempferol	Pepsin A	P00790	REN	0.019
Orostachys fimbriata（OF）	MOL000422	kaempferol	Tyrosine 3-monooxygenase	P07101	TH	0.019
Orostachys fimbriata（OF）	MOL000422	kaempferol	Retinoic acid receptor alpha	P10276	RARA	0.019
Orostachys fimbriata（OF）	MOL000422	kaempferol	Retinoic acid receptor beta	P10826	RARB	0.019
Orostachys fimbriata（OF）	MOL000422	kaempferol	Retinoic acid receptor gamma-1	P13631	RARG	0.019
Orostachys fimbriata（OF）	MOL000422	kaempferol	Dipeptidyl peptidase 4	P27487	DPP4	0.019
Orostachys fimbriata（OF）	MOL000422	kaempferol	Amiloride-sensitive sodium channel subunit alpha	P37088	SCNN1A	0.019
Orostachys fimbriata（OF）	MOL000422	kaempferol	Retinoic acid receptor RXR-gamma	P48443	RXRG	0.019
Orostachys fimbriata（OF）	MOL000422	kaempferol	Amiloride-sensitive sodium channel subunit beta	P51168	SCNN1B	0.019
Orostachys fimbriata（OF）	MOL000422	kaempferol	Amiloride-sensitive sodium channel subunit gamma	P51170	SCNN1G	0.019
Orostachys fimbriata（OF）	MOL000422	kaempferol	Peptidyl-prolyl cis-trans isomerase NIMA-interacting 1	Q13526	PIN1	0.019
Orostachys fimbriata（OF）	MOL000422	kaempferol	Cyclin-dependent kinase 5 activator 1	Q15078	CDK5R1	0.019
Orostachys fimbriata（OF）	MOL000422	kaempferol	Tyrosyl-tRNA synthetase, mitochondrial	Q9Y2Z4	YARS2	0.019
Orostachys fimbriata（OF）	MOL000422	kaempferol	Adenosine A2b receptor	P29275	ADORA2B	0.02
Orostachys fimbriata（OF）	MOL000422	kaempferol	Adenosine A3 receptor	P33765	SHMT1	0.02
Orostachys fimbriata（OF）	MOL000422	kaempferol	Prostaglandin reductase 1	Q14914	PTGR1	0.02
Orostachys fimbriata（OF）	MOL000422	kaempferol	Potassium voltage-gated channel subfamily H member 2	Q12809	KCNH2	0.021
Orostachys fimbriata（OF）	MOL000422	kaempferol	Potassium voltage-gated channel subfamily H member 6	Q9H252	KCNH6	0.021
Orostachys fimbriata（OF）	MOL000422	kaempferol	cGMP-specific 3',5'-cyclic phosphodiesterase	O76074	PDE5A	0.022
Orostachys fimbriata（OF）	MOL000422	kaempferol	Carbonic anhydrase 4	P22748	CA4	0.022
Orostachys fimbriata（OF）	MOL000422	kaempferol	Adenosine A1 receptor	P30542	ADORA1	0.022
Orostachys fimbriata（OF）	MOL000422	kaempferol	Sodium/potassium-transporting ATPase gamma chain	P54710	FXYD2	0.022
Orostachys fimbriata（OF）	MOL000422	kaempferol	cAMP and cAMP-inhibited cGMP 3',5'-cyclic phosphodiesterase 10A	Q9Y233	PDE10A	0.023
Orostachys fimbriata（OF）	MOL000422	kaempferol	Tryptophanyl-tRNA synthetase, mitochondrial	Q9UGM6	WARS2	0.024
Orostachys fimbriata（OF）	MOL000422	kaempferol	Potassium channel subfamily K member 1	O00180	KCNK1	0.025
Orostachys fimbriata（OF）	MOL000422	kaempferol	Tripartite motif-containing protein 13	O60858	TRIM13	0.025
Orostachys fimbriata（OF）	MOL000422	kaempferol	Prostacyclin receptor	P43119	PTGIR	0.025
Orostachys fimbriata（OF）	MOL000422	kaempferol	Methionine aminopeptidase 1	P53582	METAP1	0.025
Orostachys fimbriata（OF）	MOL000422	kaempferol	Peroxisome proliferator-activated receptor delta	Q03181	PPARD	0.025
Orostachys fimbriata（OF）	MOL000422	kaempferol	Succinate dehydrogenase [ubiquinone] cytochrome b small subunit, mitochondrial	O14521	SDHD	0.026
Orostachys fimbriata（OF）	MOL000422	kaempferol	Ig kappa chain C region	P01834	IGKC	0.026
Orostachys fimbriata（OF）	MOL000422	kaempferol	Ig gamma-1 chain C region	P01857	IGHG1	0.026
Orostachys fimbriata（OF）	MOL000422	kaempferol	Chymase	P23946	CMA1	0.026
Orostachys fimbriata（OF）	MOL000422	kaempferol	Gamma-aminobutyric-acid receptor subunit alpha-2	P47869	GABRA2	0.026
Orostachys fimbriata（OF）	MOL000422	kaempferol	Ribosyldihydronicotinamide dehydrogenase [quinone]	P16083	NQO2	0.027
Orostachys fimbriata（OF）	MOL000422	kaempferol	Peptidyl-prolyl cis-trans isomerase, mitochondrial	P30405	PPIF	0.027
Orostachys fimbriata（OF）	MOL000422	kaempferol	Mitogen-activated protein kinase 8	P45983	MAPK8	0.027
Orostachys fimbriata（OF）	MOL000422	kaempferol	Mitogen-activated protein kinase 10	P53779	MAPK10	0.027

Orostachys fimbriata（OF）	MOL000422	kaempferol	Inhibitor of nuclear factor kappa-B kinase subunit beta	O14920	IKBKB	0.028
Orostachys fimbriata（OF）	MOL000422	kaempferol	Oxysterols receptor LXR-beta	P55055	NR1H2	0.028
Orostachys fimbriata（OF）	MOL000422	kaempferol	Oxysterols receptor LXR-alpha	Q13133	NR1H3	0.028
Orostachys fimbriata（OF）	MOL000422	kaempferol	Cystine/glutamate transporter	Q9UPY5	SLC7A11	0.028
Orostachys fimbriata（OF）	MOL000422	kaempferol	Proto-oncogene tyrosine-protein kinase LCK	P06239	LCK	0.031
Orostachys fimbriata（OF）	MOL000422	kaempferol	Tyrosine-protein kinase Lyn	P07948	LYN	0.031
Orostachys fimbriata（OF）	MOL000422	kaempferol	Alpha-2A adrenergic receptor	P08913	ADRA2A	0.032
Orostachys fimbriata（OF）	MOL000422	kaempferol	Macrophage migration inhibitory factor	P14174	MIF	0.032
Orostachys fimbriata（OF）	MOL000422	kaempferol	DNA-(apurinic or apyrimidinic site) lyase	P27695	APEX1	0.032
Orostachys fimbriata（OF）	MOL000422	kaempferol	5-hydroxytryptamine 1D receptor	P28221	HTR1D	0.032
Orostachys fimbriata（OF）	MOL000422	kaempferol	5-hydroxytryptamine 1B receptor	P28222	HTR1B	0.032
Orostachys fimbriata（OF）	MOL000422	kaempferol	Sterol O-acyltransferase 2	O75908	SOAT2	0.033
Orostachys fimbriata（OF）	MOL000422	kaempferol	Glycogen phosphorylase, muscle form	P11217	PYGM	0.033
Orostachys fimbriata（OF）	MOL000422	kaempferol	Sterol O-acyltransferase 1	P35610	SOAT1	0.033
Orostachys fimbriata（OF）	MOL000422	kaempferol	Keratin, type II cytoskeletal 7	P08729	KRT7	0.034
Orostachys fimbriata（OF）	MOL000422	kaempferol	Platelet glycoprotein IX	P14770	GP9	0.034
Orostachys fimbriata（OF）	MOL000422	kaempferol	Gonadotropin-releasing hormone receptor	P30968	GNRHR	0.035
Orostachys fimbriata（OF）	MOL000422	kaempferol	Gonadotropin-releasing hormone II receptor	Q96P88	GNRHR2	0.035
Orostachys fimbriata（OF）	MOL000422	kaempferol	Gamma-aminobutyric-acid receptor subunit alpha-1	P14867	GABRA1	0.036
Orostachys fimbriata（OF）	MOL000422	kaempferol	Prolactin receptor	P16471	PRLR	0.036
Orostachys fimbriata（OF）	MOL000422	kaempferol	Tubulin beta-2C chain	P68371	TUBB4B	0.036
Orostachys fimbriata（OF）	MOL000422	kaempferol	Beta-1 adrenergic receptor	P08588	ADRB1	0.037
Orostachys fimbriata（OF）	MOL000422	kaempferol	Protein tyrosine kinase 2 beta	Q14289	PTK2B	0.037
Orostachys fimbriata（OF）	MOL000422	kaempferol	Glutamate [NMDA] receptor subunit 3A	Q8TCU5	GRIN3A	0.037
Orostachys fimbriata（OF）	MOL000422	kaempferol	Epidermal growth factor receptor	P00533	EGFR	0.038
Orostachys fimbriata（OF）	MOL000422	kaempferol	Microtubule-associated protein 2	P11137	MAP2	0.038
Orostachys fimbriata（OF）	MOL000422	kaempferol	3 beta-hydroxysteroid dehydrogenase/Delta 5-->4-isomerase type I	P14060	HSD3B1	0.038
Orostachys fimbriata（OF）	MOL000422	kaempferol	3 beta-hydroxysteroid dehydrogenase/Delta 5-->4-isomerase type II	P26439	HSD3B2	0.038
Orostachys fimbriata（OF）	MOL000422	kaempferol	Estrogen-related receptor gamma	P62508	ESRRG	0.038
Orostachys fimbriata（OF）	MOL000422	kaempferol	Microtubule-associated protein 1A	P78559	MAP1A	0.038
Orostachys fimbriata（OF）	MOL000422	kaempferol	Sodium-dependent dopamine transporter	Q01959	SLC6A3	0.038
Orostachys fimbriata（OF）	MOL000422	kaempferol	ATP synthase subunit beta, mitochondrial	P06576	ATP5F1B	0.04
Orostachys fimbriata（OF）	MOL000422	kaempferol	Tyrosine-protein kinase HCK	P08631	HCK	0.04
Orostachys fimbriata（OF）	MOL000422	kaempferol	Acetylcholinesterase	P22303	ACHE	0.04
Orostachys fimbriata（OF）	MOL000422	kaempferol	ATP synthase subunit alpha, mitochondrial	P25705	ATP5F1A	0.04
Orostachys fimbriata（OF）	MOL000422	kaempferol	Phosphatidylinositol-4,5-bisphosphate 3-kinase catalytic subunit gamma isoform	P48736	PIK3CG	0.04
Orostachys fimbriata（OF）	MOL000422	kaempferol	Glycogen synthase kinase-3 beta	P49841	GSK3B	0.042
Orostachys fimbriata（OF）	MOL000422	kaempferol	Alpha-2B adrenergic receptor	P18089	ADRA2B	0.044
Orostachys fimbriata（OF）	MOL000422	kaempferol	Alpha-2C adrenergic receptor	P18825	ADRA2C	0.044
Orostachys fimbriata（OF）	MOL000422	kaempferol	D(4) dopamine receptor	P21917	DRD4	0.044
Orostachys fimbriata（OF）	MOL000422	kaempferol	Alpha-1D adrenergic receptor	P25100	ADRA1D	0.045
Orostachys fimbriata（OF）	MOL000422	kaempferol	Adenosine A2a receptor	P29274	ADORA2A	0.047
Orostachys fimbriata（OF）	MOL000422	kaempferol	Muscarinic acetylcholine receptor M4	P08173	CHRM4	0.048
Orostachys fimbriata（OF）	MOL000422	kaempferol	Carbonic anhydrase 1	P00915	CA1	0.049
Orostachys fimbriata（OF）	MOL000422	kaempferol	cGMP-inhibited 3',5'-cyclic phosphodiesterase A	Q14432	PDE3A	0.05
Orostachys fimbriata（OF）	MOL000422	kaempferol	Beta-2 adrenergic receptor	P07550	ADRB2	0.051
Orostachys fimbriata（OF）	MOL000422	kaempferol	Cannabinoid receptor 1	P21554	CNR1	0.052

Orostachys fimbriata（OF）	MOL000422	kaempferol	Retinoic acid receptor RXR-beta	P28702	RXRB	0.052
Orostachys fimbriata（OF）	MOL000422	kaempferol	Dual specificity protein kinase CLK1	P49759	CLK1	0.052
Orostachys fimbriata（OF）	MOL000422	kaempferol	cAMP-dependent protein kinase inhibitor alpha	P61925	PKIA	0.052
Orostachys fimbriata（OF）	MOL000422	kaempferol	Aldo-keto reductase family 1 member C1	Q04828	AKR1C1	0.052
Orostachys fimbriata（OF）	MOL000422	kaempferol	Tubulin beta-1 chain	Q9H4B7	TUBB1	0.052
Orostachys fimbriata（OF）	MOL000422	kaempferol	Serine/threonine-protein kinase 6	O14965	AURKA	0.053
Orostachys fimbriata（OF）	MOL000422	kaempferol	Cytochrome P450 19A1	P11511	CYP19A1	0.053
Orostachys fimbriata（OF）	MOL000422	kaempferol	Egl nine homolog 1	Q9GZT9	EGLN1	0.053
Orostachys fimbriata（OF）	MOL000422	kaempferol	Leukotriene A-4 hydrolase	P09960	LTA4H	0.054
Orostachys fimbriata（OF）	MOL000422	kaempferol	RAC-alpha serine/threonine-protein kinase	P31749	AKT1	0.054
Orostachys fimbriata（OF）	MOL000422	kaempferol	Membrane copper amine oxidase	Q16853	AOC3	0.054
Orostachys fimbriata（OF）	MOL000422	kaempferol	Dihydrofolate reductase	P00374	DHFR	0.055
Orostachys fimbriata（OF）	MOL000422	kaempferol	Glucocorticoid receptor	P04150	NR3C1	0.055
Orostachys fimbriata（OF）	MOL000422	kaempferol	Thymidylate synthase	P04818	TYMS	0.055
Orostachys fimbriata（OF）	MOL000422	kaempferol	Thyroid hormone receptor beta-1	P10828	THRB	0.055
Orostachys fimbriata（OF）	MOL000422	kaempferol	Ig kappa chain V-II region RPMI 6410	P06310	IGKV2-30	0.056
Orostachys fimbriata（OF）	MOL000422	kaempferol	5-hydroxytryptamine 1A receptor	P08908	HTR1A	0.056
Orostachys fimbriata（OF）	MOL000422	kaempferol	D(1B) dopamine receptor	P21918	DRD5	0.056
Orostachys fimbriata（OF）	MOL000422	kaempferol	D(3) dopamine receptor	P35462	DRD3	0.056
Orostachys fimbriata（OF）	MOL000422	kaempferol	Triosephosphate isomerase	P60174	TPI1	0.056
Orostachys fimbriata（OF）	MOL000422	kaempferol	Calmodulin	P62158		0.056
Orostachys fimbriata（OF）	MOL000422	kaempferol	S-methyl-5-thioadenosine phosphorylase	Q13126	MTAP	0.056
Orostachys fimbriata（OF）	MOL000422	kaempferol	Thyroid hormone receptor alpha	P10827	THRA	0.057
Orostachys fimbriata（OF）	MOL000422	kaempferol	5-hydroxytryptamine 2C receptor	P28335	HTR2C	0.057
Orostachys fimbriata（OF）	MOL000422	kaempferol	5-hydroxytryptamine 2B receptor	P41595	HTR2B	0.057
Orostachys fimbriata（OF）	MOL000422	kaempferol	Solute carrier family 12 member 1	Q13621	SLC12A1	0.057
Orostachys fimbriata（OF）	MOL000422	kaempferol	Sodium channel protein type 5 subunit alpha	Q14524	SCN5A	0.057
Orostachys fimbriata（OF）	MOL000422	kaempferol	Toll-like receptor 7	Q9NYK1	TLR7	0.057
Orostachys fimbriata（OF）	MOL000422	kaempferol	Calcium/calmodulin-dependent protein kinase type II alpha chain	Q9UQM7	CAMK2A	0.057
Orostachys fimbriata（OF）	MOL000422	kaempferol	Alpha-1B adrenergic receptor	P35368	ADRA1B	0.058
Orostachys fimbriata（OF）	MOL000422	kaempferol	Casein kinase II subunit alpha	P68400	CSNK2A1	0.058
Orostachys fimbriata（OF）	MOL000422	kaempferol	Thiamin pyrophosphokinase 1	Q9H3S4	TPK1	0.058
Orostachys fimbriata（OF）	MOL000422	kaempferol	C-jun-amino-terminal kinase-interacting protein 1	Q9UQF2	MAPK8IP1	0.058
Orostachys fimbriata（OF）	MOL000422	kaempferol	Peroxisome proliferator-activated receptor gamma	P37231	PPARG	0.059
Orostachys fimbriata（OF）	MOL000422	kaempferol	Neuropeptide Y	P01303	NPY	0.06
Orostachys fimbriata（OF）	MOL000422	kaempferol	Nuclear receptor coactivator 2	Q15596	NCOA2	0.06
Orostachys fimbriata（OF）	MOL000422	kaempferol	Group IIE secretory phospholipase A2	Q9NZK7	PLA2G2E	0.06
Orostachys fimbriata（OF）	MOL000422	kaempferol	Nuclear receptor coactivator 5	Q9HCD5	NCOA5	0.062
Orostachys fimbriata（OF）	MOL000422	kaempferol	Interferon gamma	P01579	IFNG	0.063
Orostachys fimbriata（OF）	MOL000422	kaempferol	Phospholipase A2	P04054	PLA2G1B	0.063
Orostachys fimbriata（OF）	MOL000422	kaempferol	Fibroblast growth factor receptor 2	P21802	FGFR2	0.063
Orostachys fimbriata（OF）	MOL000422	kaempferol	Proto-oncogene tyrosine-protein kinase Src	P12931	SRC	0.066
Orostachys fimbriata（OF）	MOL000422	kaempferol	Muscarinic acetylcholine receptor M3	P20309	CHRM3	0.066
Orostachys fimbriata（OF）	MOL000422	kaempferol	Tyrosyl-tRNA synthetase, cytoplasmic	P54577	YARS	0.066
Orostachys fimbriata（OF）	MOL000422	kaempferol	DNA polymerase kappa	Q9UBT6	POLK	0.068
Orostachys fimbriata（OF）	MOL000422	kaempferol	DNA topoisomerase 2-alpha	P11388	TOP2A	0.072
Orostachys fimbriata（OF）	MOL000422	kaempferol	Progesterone receptor	P06401	PGR	0.074

Orostachys fimbriata（OF）	MOL000422	kaempferol	5-hydroxytryptamine 2A receptor	P28223	HTR2A	0.074
Orostachys fimbriata（OF）	MOL000422	kaempferol	Carbonic anhydrase 2	P00918	CA2	0.075
Orostachys fimbriata（OF）	MOL000422	kaempferol	Tubulin alpha-3 chain	Q71U36	TUBA1A	0.077
Orostachys fimbriata（OF）	MOL000422	kaempferol	D(1A) dopamine receptor	P21728	DRD1	0.081
Orostachys fimbriata（OF）	MOL000422	kaempferol	D-HSCDK2	O75100	CA11	0.084
Orostachys fimbriata（OF）	MOL000422	kaempferol	Muscarinic acetylcholine receptor M2	P08172	CHRM2	0.084
Orostachys fimbriata（OF）	MOL000422	kaempferol	Delta-type opioid receptor	P41143	OPRD1	0.085
Orostachys fimbriata（OF）	MOL000422	kaempferol	Kappa-type opioid receptor	P41145	OPRK1	0.085
Orostachys fimbriata（OF）	MOL000422	kaempferol	Inhibitor of nuclear factor kappa-B kinase subunit alpha	O15111	CHUK	0.091
Orostachys fimbriata（OF）	MOL000422	kaempferol	Arachidonate 5-lipoxygenase	P09917	ALOX5	0.091
Orostachys fimbriata（OF）	MOL000422	kaempferol	D(2) dopamine receptor	P14416	DRD2	0.093
Orostachys fimbriata（OF）	MOL000422	kaempferol	Alpha-1A adrenergic receptor	P35348	ADRA1A	0.098
Orostachys fimbriata（OF）	MOL000422	kaempferol	Muscarinic acetylcholine receptor M1	P11229	CHRM1	0.099
Orostachys fimbriata（OF）	MOL000422	kaempferol	cAMP-specific 3',5'-cyclic phosphodiesterase 4A	P27815	PDE4A	0.104
Orostachys fimbriata（OF）	MOL000422	kaempferol	Sodium-dependent serotonin transporter	P31645	SLC6A4	0.105
Orostachys fimbriata（OF）	MOL000422	kaempferol	Prothrombin	P00734	F2	0.106
Orostachys fimbriata（OF）	MOL000422	kaempferol	Cell division control protein 2 homolog	P06493	CDK1	0.107
Orostachys fimbriata（OF）	MOL000422	kaempferol	Nitric oxide synthase, inducible	P35228	NOS2	0.113
Orostachys fimbriata（OF）	MOL000422	kaempferol	cAMP-specific 3',5'-cyclic phosphodiesterase 4D	Q08499	PDE4D	0.113
Orostachys fimbriata（OF）	MOL000422	kaempferol	Androgen receptor	P10275	AR	0.115
Orostachys fimbriata（OF）	MOL000422	kaempferol	cAMP-dependent protein kinase catalytic subunit alpha	P17612	PRKACA	0.116
Orostachys fimbriata（OF）	MOL000422	kaempferol	Cell division protein kinase 5	Q00535	CDK5	0.129
Orostachys fimbriata（OF）	MOL000422	kaempferol	cAMP-specific 3',5'-cyclic phosphodiesterase 4B	Q07343	PDE4B	0.131
Orostachys fimbriata（OF）	MOL000422	kaempferol	Cell division protein kinase 2	P24941	CDK2	0.153
Orostachys fimbriata（OF）	MOL000422	kaempferol	Trypsin-1	P07477	PRSS1	0.157
Orostachys fimbriata（OF）	MOL000422	kaempferol	MAP kinase-activated protein kinase 2	P49137	MAPKAPK2	0.174
Orostachys fimbriata（OF）	MOL000422	kaempferol	Hemoglobin subunit alpha	P69905	HBA1	0.191
Orostachys fimbriata（OF）	MOL000422	kaempferol	Mu-type opioid receptor	P35372	OPRM1	0.203
Orostachys fimbriata（OF）	MOL000422	kaempferol	Prostaglandin G/H synthase 1	P23219	PTGS1	0.25
Orostachys fimbriata（OF）	MOL000422	kaempferol	Nuclear receptor coactivator 1	Q15788	NCOA1	0.265
Orostachys fimbriata（OF）	MOL000422	kaempferol	Cyclin-A2	P20248	CCNA2	0.342
Orostachys fimbriata（OF）	MOL000422	kaempferol	Estrogen receptor beta	Q92731	ESR2	0.383
Orostachys fimbriata（OF）	MOL000422	kaempferol	Prostaglandin G/H synthase 2	P35354	PTGS2	0.573
Orostachys fimbriata（OF）	MOL000422	kaempferol	Estrogen receptor	P03372	ESR1	1
Orostachys fimbriata（OF）	MOL002823	Herbacetin	Gamma-aminobutyric acid receptor subunit rho-3	A8MPY1	GABRR3	0.016
Orostachys fimbriata（OF）	MOL002823	Herbacetin	Gamma-aminobutyric acid receptor subunit pi	O00591	GABRP	0.016
Orostachys fimbriata（OF）	MOL002823	Herbacetin	Gamma-aminobutyric acid receptor subunit delta	O14764	GABRD	0.016
Orostachys fimbriata（OF）	MOL002823	Herbacetin	Gamma-aminobutyric-acid receptor subunit beta-1	P18505	GABRB1	0.016
Orostachys fimbriata（OF）	MOL002823	Herbacetin	Gamma-aminobutyric acid receptor subunit gamma-2	P18507	GABRG2	0.016
Orostachys fimbriata（OF）	MOL002823	Herbacetin	Gamma-aminobutyric-acid receptor subunit rho-1	P24046	GABRR1	0.016
Orostachys fimbriata（OF）	MOL002823	Herbacetin	Gamma-aminobutyric acid receptor subunit rho-2	P28476	GABRR2	0.016
Orostachys fimbriata（OF）	MOL002823	Herbacetin	Gamma-aminobutyric-acid receptor subunit alpha-5	P31644	GABRA5	0.016
Orostachys fimbriata（OF）	MOL002823	Herbacetin	Gamma-aminobutyric-acid receptor subunit alpha-3	P34903	GABRA3	0.016
Orostachys fimbriata（OF）	MOL002823	Herbacetin	Gamma-aminobutyric-acid receptor subunit beta-2	P47870	GABRB2	0.016
Orostachys fimbriata（OF）	MOL002823	Herbacetin	Gamma-aminobutyric-acid receptor subunit alpha-4	P48169	GABRA4	0.016
Orostachys fimbriata（OF）	MOL002823	Herbacetin	Gamma-aminobutyric acid receptor subunit epsilon	P78334	GABRE	0.016
Orostachys fimbriata（OF）	MOL002823	Herbacetin	Gamma-aminobutyric-acid receptor subunit alpha-6	Q16445	GABRA6	0.016

Orostachys fimbriata（OF）	MOL002823	Herbacetin	Gamma-aminobutyric acid receptor subunit gamma-1	Q8N1C3	GABRG1	0.016
Orostachys fimbriata（OF）	MOL002823	Herbacetin	Gamma-aminobutyric acid receptor subunit gamma-3	Q99928	GABRG3	0.016
Orostachys fimbriata（OF）	MOL002823	Herbacetin	Gamma-aminobutyric acid receptor subunit theta	Q9UN88	GABRQ	0.016
Orostachys fimbriata（OF）	MOL002823	Herbacetin	Phosphatidylinositol 3-kinase regulatory subunit beta	O00459	PIK3R2	0.022
Orostachys fimbriata（OF）	MOL002823	Herbacetin	Beta-3 adrenergic receptor	P13945	ADRB3	0.022
Orostachys fimbriata（OF）	MOL002823	Herbacetin	Sodium/hydrogen exchanger 1	P19634	SLC9A1	0.022
Orostachys fimbriata（OF）	MOL002823	Herbacetin	Amiloride-sensitive amine oxidase [copper-containing]	P19801	AOC1	0.022
Orostachys fimbriata（OF）	MOL002823	Herbacetin	Phosphatidylinositol 3-kinase regulatory subunit alpha	P27986	PIK3R1	0.022
Orostachys fimbriata（OF）	MOL002823	Herbacetin	Mitogen-activated protein kinase 1	P28482	MAPK1	0.022
Orostachys fimbriata（OF）	MOL002823	Herbacetin	Amiloride-sensitive sodium channel subunit delta	P51172	SCNN1D	0.022
Orostachys fimbriata（OF）	MOL002823	Herbacetin	Amiloride-sensitive cation channel 2, neuronal	P78348	ASIC1	0.022
Orostachys fimbriata（OF）	MOL002823	Herbacetin	Amiloride-sensitive cation channel 1, neuronal	Q16515	ASIC2	0.022
Orostachys fimbriata（OF）	MOL002823	Herbacetin	5-hydroxytryptamine 1A receptor	P08908	HTR1A	0.023
Orostachys fimbriata（OF）	MOL002823	Herbacetin	5-hydroxytryptamine 1D receptor	P28221	HTR1D	0.023
Orostachys fimbriata（OF）	MOL002823	Herbacetin	5-hydroxytryptamine 1B receptor	P28222	HTR1B	0.023
Orostachys fimbriata（OF）	MOL002823	Herbacetin	5-hydroxytryptamine 2C receptor	P28335	HTR2C	0.023
Orostachys fimbriata（OF）	MOL002823	Herbacetin	5-hydroxytryptamine 2B receptor	P41595	HTR2B	0.023
Orostachys fimbriata（OF）	MOL002823	Herbacetin	D1 dopamine receptor-interacting protein calcyon	Q9NYX4	CALY	0.023
Orostachys fimbriata（OF）	MOL002823	Herbacetin	5-hydroxytryptamine 2A receptor	P28223	HTR2A	0.026
Orostachys fimbriata（OF）	MOL002823	Herbacetin	Glutamate receptor 2	P42262	GRIA2	0.026
Orostachys fimbriata（OF）	MOL002823	Herbacetin	5-hydroxytryptamine 3 receptor	P46098	HTR3A	0.027
Orostachys fimbriata（OF）	MOL002823	Herbacetin	Glutamate [NMDA] receptor subunit 3A	Q8TCU5	GRIN3A	0.027
Orostachys fimbriata（OF）	MOL002823	Herbacetin	Sodium channel protein type 10 subunit alpha	Q9Y5Y9	SCN10A	0.027
Orostachys fimbriata（OF）	MOL002823	Herbacetin	Tumor necrosis factor	P01375	TNF	0.028
Orostachys fimbriata（OF）	MOL002823	Herbacetin	Nuclear factor NF-kappa-B p105 subunit	P19838	NFKB1	0.028
Orostachys fimbriata（OF）	MOL002823	Herbacetin	Fibroblast growth factor receptor 2	P21802	FGFR2	0.028
Orostachys fimbriata（OF）	MOL002823	Herbacetin	Death-associated protein kinase 3	O43293	DAPK3	0.031
Orostachys fimbriata（OF）	MOL002823	Herbacetin	Tyrosine-protein kinase JAK2	O60674	JAK2	0.031
Orostachys fimbriata（OF）	MOL002823	Herbacetin	Tyrosine-protein kinase JAK1	P23458	JAK1	0.031
Orostachys fimbriata（OF）	MOL002823	Herbacetin	Tyrosine-protein kinase JAK3	P52333	JAK3	0.031
Orostachys fimbriata（OF）	MOL002823	Herbacetin	Cytochrome b	P00156	MT-CYB	0.035
Orostachys fimbriata（OF）	MOL002823	Herbacetin	Muscarinic acetylcholine receptor M2	P08172	CHRM2	0.035
Orostachys fimbriata（OF）	MOL002823	Herbacetin	Muscarinic acetylcholine receptor M4	P08173	CHRM4	0.035
Orostachys fimbriata（OF）	MOL002823	Herbacetin	Cytochrome c1, heme protein, mitochondrial	P08574	CYC1	0.035
Orostachys fimbriata（OF）	MOL002823	Herbacetin	Muscarinic acetylcholine receptor M1	P11229	CHRM1	0.035
Orostachys fimbriata（OF）	MOL002823	Herbacetin	Cytochrome b-c1 complex subunit 7	P14927	UQCRB	0.035
Orostachys fimbriata（OF）	MOL002823	Herbacetin	Muscarinic acetylcholine receptor M3	P20309	CHRM3	0.035
Orostachys fimbriata（OF）	MOL002823	Herbacetin	Cytochrome b-c1 complex subunit Rieske, mitochondrial	P47985	UQCRFS1	0.035
Orostachys fimbriata（OF）	MOL002823	Herbacetin	Potassium channel subfamily K member 6	Q9Y257	KCNK6	0.035
Orostachys fimbriata（OF）	MOL002823	Herbacetin	Serine/threonine-protein kinase 17B	O94768	STK17B	0.048
Orostachys fimbriata（OF）	MOL002823	Herbacetin	Tyrosine 3-monooxygenase	P07101	TH	0.048
Orostachys fimbriata（OF）	MOL002823	Herbacetin	Proto-oncogene serine/threonine-protein kinase Pim-1	P11309	PIM1	0.048
Orostachys fimbriata（OF）	MOL002823	Herbacetin	ATP synthase subunit gamma, mitochondrial	P36542	ATP5F1C	0.048
Orostachys fimbriata（OF）	MOL002823	Herbacetin	Tyrosyl-tRNA synthetase, cytoplasmic	P54577	YARS	0.048
Orostachys fimbriata（OF）	MOL002823	Herbacetin	UDP-glucuronosyltransferase 3A1	Q6NUS8	UGT3A1	0.048
Orostachys fimbriata（OF）	MOL002823	Herbacetin	Tyrosyl-tRNA synthetase, mitochondrial	Q9Y2Z4	YARS2	0.048
Orostachys fimbriata（OF）	MOL002823	Herbacetin	Tripartite motif-containing protein 13	O60858	TRIM13	0.052

Orostachys fimbriata（OF）	MOL002823	Herbacetin	Kappa-type opioid receptor	P41145	OPRK1	0.052
Orostachys fimbriata（OF）	MOL002823	Herbacetin	Nociceptin receptor	P41146	OPRL1	0.052
Orostachys fimbriata（OF）	MOL002823	Herbacetin	Phenylalanine-4-hydroxylase	P00439	PAH	0.058
Orostachys fimbriata（OF）	MOL002823	Herbacetin	Amiloride-sensitive sodium channel subunit alpha	P37088	SCNN1A	0.059
Orostachys fimbriata（OF）	MOL002823	Herbacetin	Amiloride-sensitive sodium channel subunit beta	P51168	SCNN1B	0.059
Orostachys fimbriata（OF）	MOL002823	Herbacetin	Amiloride-sensitive sodium channel subunit gamma	P51170	SCNN1G	0.059
Orostachys fimbriata（OF）	MOL002823	Herbacetin	Prostaglandin reductase 1	Q14914	PTGR1	0.06
Orostachys fimbriata（OF）	MOL002823	Herbacetin	D(4) dopamine receptor	P21917	DRD4	0.061
Orostachys fimbriata（OF）	MOL002823	Herbacetin	Glycogen synthase kinase-3 beta	P49841	GSK3B	0.062
Orostachys fimbriata（OF）	MOL002823	Herbacetin	Cyclin-dependent kinase 5 activator 1	Q15078	CDK5R1	0.062
Orostachys fimbriata（OF）	MOL002823	Herbacetin	Sodium-dependent dopamine transporter	Q01959	SLC6A3	0.065
Orostachys fimbriata（OF）	MOL002823	Herbacetin	Cell division protein kinase 4	P11802	CDK4	0.067
Orostachys fimbriata（OF）	MOL002823	Herbacetin	Cell division protein kinase 7	P50613	CDK7	0.067
Orostachys fimbriata（OF）	MOL002823	Herbacetin	Cell division protein kinase 9	P50750	CDK9	0.067
Orostachys fimbriata（OF）	MOL002823	Herbacetin	Cell division protein kinase 6	Q00534	CDK6	0.067
Orostachys fimbriata（OF）	MOL002823	Herbacetin	Aryl hydrocarbon receptor	P35869	AHR	0.073
Orostachys fimbriata（OF）	MOL002823	Herbacetin	Dihydroorotate dehydrogenase, mitochondrial	Q02127	DHODH	0.073
Orostachys fimbriata（OF）	MOL002823	Herbacetin	cAMP-dependent protein kinase catalytic subunit alpha	P17612	PRKACA	0.075
Orostachys fimbriata（OF）	MOL002823	Herbacetin	Rho-associated protein kinase 1	Q13464	ROCK1	0.075
Orostachys fimbriata（OF）	MOL002823	Herbacetin	Potassium channel subfamily K member 1	O00180	KCNK1	0.076
Orostachys fimbriata（OF）	MOL002823	Herbacetin	Proto-oncogene tyrosine-protein kinase LCK	P06239	LCK	0.076
Orostachys fimbriata（OF）	MOL002823	Herbacetin	Tyrosine-protein kinase Lyn	P07948	LYN	0.076
Orostachys fimbriata（OF）	MOL002823	Herbacetin	Nuclear receptor coactivator 2	Q15596	NCOA2	0.076
Orostachys fimbriata（OF）	MOL002823	Herbacetin	Ribosyldihydronicotinamide dehydrogenase [quinone]	P16083	NQO2	0.08
Orostachys fimbriata（OF）	MOL002823	Herbacetin	DNA (cytosine-5)-methyltransferase 1	P26358	DNMT1	0.08
Orostachys fimbriata（OF）	MOL002823	Herbacetin	Progesterone receptor	P06401	PGR	0.082
Orostachys fimbriata（OF）	MOL002823	Herbacetin	Androgen receptor	P10275	AR	0.082
Orostachys fimbriata（OF）	MOL002823	Herbacetin	Gonadotropin-releasing hormone receptor	P30968	GNRHR	0.082
Orostachys fimbriata（OF）	MOL002823	Herbacetin	Gonadotropin-releasing hormone II receptor	Q96P88	GNRHR2	0.082
Orostachys fimbriata（OF）	MOL002823	Herbacetin	3 beta-hydroxysteroid dehydrogenase/Delta 5-->4-isomerase type I	P14060	HSD3B1	0.088
Orostachys fimbriata（OF）	MOL002823	Herbacetin	3 beta-hydroxysteroid dehydrogenase/Delta 5-->4-isomerase type II	P26439	HSD3B2	0.088
Orostachys fimbriata（OF）	MOL002823	Herbacetin	Estrogen-related receptor gamma	P62508	ESRRG	0.088
Orostachys fimbriata（OF）	MOL002823	Herbacetin	Gamma-aminobutyric-acid receptor subunit alpha-1	P14867	GABRA1	0.093
Orostachys fimbriata（OF）	MOL002823	Herbacetin	Gamma-aminobutyric-acid receptor subunit alpha-2	P47869	GABRA2	0.093
Orostachys fimbriata（OF）	MOL002823	Herbacetin	Alpha-1D adrenergic receptor	P25100	ADRA1D	0.094
Orostachys fimbriata（OF）	MOL002823	Herbacetin	Alpha-1B adrenergic receptor	P35368	ADRA1B	0.094
Orostachys fimbriata（OF）	MOL002823	Herbacetin	D(3) dopamine receptor	P35462	DRD3	0.099
Orostachys fimbriata（OF）	MOL002823	Herbacetin	DNA topoisomerase 2-alpha	P11388	TOP2A	0.101
Orostachys fimbriata（OF）	MOL002823	Herbacetin	Protein tyrosine kinase 2 beta	Q14289	PTK2B	0.101
Orostachys fimbriata（OF）	MOL002823	Herbacetin	Mitogen-activated protein kinase 8	P45983	MAPK8	0.103
Orostachys fimbriata（OF）	MOL002823	Herbacetin	Mitogen-activated protein kinase 10	P53779	MAPK10	0.103
Orostachys fimbriata（OF）	MOL002823	Herbacetin	Prothrombin	P00734	F2	0.107
Orostachys fimbriata（OF）	MOL002823	Herbacetin	ATP synthase subunit beta, mitochondrial	P06576	ATP5F1B	0.109
Orostachys fimbriata（OF）	MOL002823	Herbacetin	Tyrosine-protein kinase HCK	P08631	HCK	0.109
Orostachys fimbriata（OF）	MOL002823	Herbacetin	ATP synthase subunit alpha, mitochondrial	P25705	ATP5F1A	0.109
Orostachys fimbriata（OF）	MOL002823	Herbacetin	Phosphatidylinositol-4,5-bisphosphate 3-kinase catalytic subunit gamma isoform	P48736	PIK3CG	0.109
Orostachys fimbriata（OF）	MOL002823	Herbacetin	Delta-type opioid receptor	P41143	OPRD1	0.119

Orostachys fimbriata（OF）	MOL002823	Herbacetin	Sodium channel protein type 5 subunit alpha	Q14524	SCN5A	0.124
Orostachys fimbriata（OF）	MOL002823	Herbacetin	Platelet glycoprotein IX	P14770	GP9	0.125
Orostachys fimbriata（OF）	MOL002823	Herbacetin	Beta-1 adrenergic receptor	P08588	ADRB1	0.13
Orostachys fimbriata（OF）	MOL002823	Herbacetin	Alpha-1A adrenergic receptor	P35348	ADRA1A	0.13
Orostachys fimbriata（OF）	MOL002823	Herbacetin	Alpha-2A adrenergic receptor	P08913	ADRA2A	0.131
Orostachys fimbriata（OF）	MOL002823	Herbacetin	Alpha-2C adrenergic receptor	P18825	ADRA2C	0.132
Orostachys fimbriata（OF）	MOL002823	Herbacetin	D(1A) dopamine receptor	P21728	DRD1	0.136
Orostachys fimbriata（OF）	MOL002823	Herbacetin	D(1B) dopamine receptor	P21918	DRD5	0.136
Orostachys fimbriata（OF）	MOL002823	Herbacetin	Epidermal growth factor receptor	P00533	EGFR	0.147
Orostachys fimbriata（OF）	MOL002823	Herbacetin	Glycogen phosphorylase, muscle form	P11217	PYGM	0.147
Orostachys fimbriata（OF）	MOL002823	Herbacetin	Nitric oxide synthase, inducible	P35228	NOS2	0.16
Orostachys fimbriata（OF）	MOL002823	Herbacetin	Phospholipase A2	P04054	PLA2G1B	0.162
Orostachys fimbriata（OF）	MOL002823	Herbacetin	Ornithine aminotransferase, mitochondrial	P04181	OAT	0.164
Orostachys fimbriata（OF）	MOL002823	Herbacetin	Beta-2 adrenergic receptor	P07550	ADRB2	0.166
Orostachys fimbriata（OF）	MOL002823	Herbacetin	Peroxisome proliferator-activated receptor gamma	P37231	PPARG	0.166
Orostachys fimbriata（OF）	MOL002823	Herbacetin	Inhibitor of nuclear factor kappa-B kinase subunit alpha	O15111	CHUK	0.168
Orostachys fimbriata（OF）	MOL002823	Herbacetin	Alpha-2B adrenergic receptor	P18089	ADRA2B	0.168
Orostachys fimbriata（OF）	MOL002823	Herbacetin	Egl nine homolog 1	Q9GZT9	EGLN1	0.168
Orostachys fimbriata（OF）	MOL002823	Herbacetin	Leukotriene A-4 hydrolase	P09960	LTA4H	0.169
Orostachys fimbriata（OF）	MOL002823	Herbacetin	Group IIE secretory phospholipase A2	Q9NZK7	PLA2G2E	0.169
Orostachys fimbriata（OF）	MOL002823	Herbacetin	Membrane copper amine oxidase	Q16853	AOC3	0.172
Orostachys fimbriata（OF）	MOL002823	Herbacetin	Tubulin alpha-3 chain	Q71U36	TUBA1A	0.172
Orostachys fimbriata（OF）	MOL002823	Herbacetin	Calcium/calmodulin-dependent protein kinase type II alpha chain	Q9UQM7	CAMK2A	0.173
Orostachys fimbriata（OF）	MOL002823	Herbacetin	Casein kinase II subunit alpha	P68400	CSNK2A1	0.174
Orostachys fimbriata（OF）	MOL002823	Herbacetin	D(2) dopamine receptor	P14416	DRD2	0.177
Orostachys fimbriata（OF）	MOL002823	Herbacetin	DNA polymerase kappa	Q9UBT6	POLK	0.18
Orostachys fimbriata（OF）	MOL002823	Herbacetin	Mitogen-activated protein kinase 14	Q16539	MAPK14	0.181
Orostachys fimbriata（OF）	MOL002823	Herbacetin	MAP kinase-activated protein kinase 2	P49137	MAPKAPK2	0.182
Orostachys fimbriata（OF）	MOL002823	Herbacetin	Toll-like receptor 7	Q9NYK1	TLR7	0.183
Orostachys fimbriata（OF）	MOL002823	Herbacetin	Potassium voltage-gated channel subfamily KQT member 1	P51787	KCNQ1	0.184
Orostachys fimbriata（OF）	MOL002823	Herbacetin	Mu-type opioid receptor	P35372	OPRM1	0.185
Orostachys fimbriata（OF）	MOL002823	Herbacetin	Thyroid hormone receptor alpha	P10827	THRA	0.188
Orostachys fimbriata（OF）	MOL002823	Herbacetin	Proto-oncogene tyrosine-protein kinase Src	P12931	SRC	0.19
Orostachys fimbriata（OF）	MOL002823	Herbacetin	Tyrosine-protein phosphatase non-receptor type 1	P18031	PTPN1	0.192
Orostachys fimbriata（OF）	MOL002823	Herbacetin	D-HSCDK2	O75100	CA11	0.219
Orostachys fimbriata（OF）	MOL002823	Herbacetin	Cell division control protein 2 homolog	P06493	CDK1	0.219
Orostachys fimbriata（OF）	MOL002823	Herbacetin	Trypsin-1	P07477	PRSS1	0.227
Orostachys fimbriata（OF）	MOL002823	Herbacetin	Arachidonate 5-lipoxygenase	P09917	ALOX5	0.254
Orostachys fimbriata（OF）	MOL002823	Herbacetin	Cell division protein kinase 5	Q00535	CDK5	0.295
Orostachys fimbriata（OF）	MOL002823	Herbacetin	C-jun-amino-terminal kinase-interacting protein 1	Q9UQF2	MAPK8IP1	0.335
Orostachys fimbriata（OF）	MOL002823	Herbacetin	Nuclear receptor coactivator 1	Q15788	NCOA1	0.342
Orostachys fimbriata（OF）	MOL002823	Herbacetin	cAMP-specific 3',5'-cyclic phosphodiesterase 4B	Q07343	PDE4B	0.371
Orostachys fimbriata（OF）	MOL002823	Herbacetin	Cyclin-A2	P20248	CCNA2	0.375
Orostachys fimbriata（OF）	MOL002823	Herbacetin	Prostaglandin G/H synthase 1	P23219	PTGS1	0.541
Orostachys fimbriata（OF）	MOL002823	Herbacetin	Cell division protein kinase 2	P24941	CDK2	0.658
Orostachys fimbriata（OF）	MOL002823	Herbacetin	Estrogen receptor beta	Q92731	ESR2	0.71
Orostachys fimbriata（OF）	MOL002823	Herbacetin	Estrogen receptor	P03372	ESR1	0.914

Orostachys fimbriata（OF）	MOL002823	Herbacetin	Prostaglandin G/H synthase 2	P35354	PTGS2	1

Supplementary Tab.3 110 DPN-related genes

Gene	Uniprot	Description	Database
ABCC8	Q09428	ATP-binding cassette sub-family C member 8	# 606176 OMIM Updated June 30, 2018
GCK	P35557	Glucokinase	# 606176 OMIM Updated June 30, 2018
INS	P01308	Insulin	# 606176 OMIM Updated June 30, 2018
KCNJ11	Q14654	ATP-sensitive inward rectifier potassium channel 11	# 606176 OMIM Updated June 30, 2018
AP1AR	Q63HQ0	AP-1 complex-associated regulatory protein	GSE95849
ASF1A	Q9Y294	Histone chaperone ASF1A	GSE95849
CCDC19	Q9UL16	Cilia- and flagella-associated protein 45	GSE95849
CCR2	P41597	C-C chemokine receptor type 2	GSE95849
CDRT1	O95170	CMT1A duplicated region transcript 1 protein	GSE95849
CLC	Q9UBD9	Cardiotrophin-like cytokine factor 1	GSE95849
DBX1	A6NMT0	Homeobox protein DBX1	GSE95849
DCAF7	P61962	DDB1- and CUL4-associated factor 7	GSE95849
DDX60	Q8IY21	Probable ATP-dependent RNA helicase DDX60	GSE95849
DIRC1	Q969H9	Disrupted in renal carcinoma protein 1	GSE95849
DMRTC2	Q8IXT2	Doublesex- and mab-3-related transcription factor C2	GSE95849
DTX3L	Q8TDB6	E3 ubiquitin-protein ligase DTX3L	GSE95849
DUSP6	Q16828	Dual specificity protein phosphatase 6	GSE95849
EVC	P57679	Ellis-van Creveld syndrome protein	GSE95849
FAM111A	Q96PZ2	Protein FAM111A	GSE95849
FAM228A	Q86W67	Protein FAM228A	GSE95849
FCER1A	P12319	High affinity immunoglobulin epsilon receptor subunit	GSE95849
FGF1	P05230	Fibroblast growth factor 1	GSE95849
FGL2	Q14314	Fibroleukin	GSE95849
FLI1	Q01543	Friend leukemia integration 1 transcription factor	GSE95849
FLRT3	Q9NZU0	Leucine-rich repeat transmembrane protein FLRT3	GSE95849
FOS	P01100	Proto-oncogene c-Fos	GSE95849
GIMAP2	Q9UG22	GTPase IMAP family member 2	GSE95849
GIMAP4	Q9NUV9	GTPase IMAP family member 4	GSE95849
GIMAP8	Q8ND71	GTPase IMAP family member 8	GSE95849
GRASP	Q7Z6J2	General receptor for phosphoinositides 1-associated sca	GSE95849
HEXB	P07686	Beta-hexosaminidase subunit beta	GSE95849
HIC1	Q14526	Hypermethylated in cancer 1 protein	GSE95849
IAPP	P10997	Islet amyloid polypeptide	GSE95849
IER3	P46695	Radiation-inducible immediate-early gene IEX-1	GSE95849
IGJ	P01591	Immunoglobulin J chain	GSE95849
LAMB3	Q13751	Laminin subunit beta-3	GSE95849
MAP1LC3A	Q9H492	Microtubule-associated proteins 1A/1B light chain 3A	GSE95849
MAT2B	Q9NZL9	Methionine adenosyltransferase 2 subunit beta	GSE95849
PLAU	P00749	Urokinase-type plasminogen activator	GSE95849
PPP4R1L	Q9P1A2	Putative serine	GSE95849
SGCG	Q13326	Gamma-sarcoglycan	GSE95849
SLC25A24	Q6NUK1	Calcium-binding mitochondrial carrier protein SCaMC-	GSE95849
SLCO4A1	Q96BD0	Solute carrier organic anion transporter family member	GSE95849
SPDYE1	Q8NFV5	Speedy protein E1	GSE95849
SPDYE3	A6NKU9	Speedy protein E3	GSE95849
SPDYE5	A6NIY4	Speedy protein E5	GSE95849
SPDYE6	P0CI01	Speedy protein E6	GSE95849
SYT13	Q7L8C5	Synaptotagmin-13	GSE95849
TFAP2C	Q92754	Transcription factor AP-2 gamma	GSE95849
TMEM132I	Q14C87	Transmembrane protein 132D	GSE95849

TMEM253	P0C7T8	Transmembrane protein 253	GSE95849
TRAF4	Q9BUZ4	TNF receptor-associated factor 4	GSE95849
TRIM22	Q8IYM9	E3 ubiquitin-protein ligase TRIM22	GSE95849
TRIM64	A6NGJ6	Tripartite motif-containing protein 64	GSE95849
TRIM64B	A6NI03	Putative tripartite motif-containing protein 64B	GSE95849
TUBB1	Q9H4B7	Tubulin beta-1 chain	GSE95849
ZC3H12A	Q5D1E8	Endoribonuclease ZC3H12A	GSE95849
AKT1	P31749	RAC-alpha serine/threonine-protein kinase	KEGG H01459
BAX	Q07812	Apoptosis regulator BAX	KEGG H01459
BCL2	P10415	Apoptosis regulator Bcl-2	KEGG H01459
CASP3	P42574	Caspase-3	KEGG H01459
CCL2	P13500	C-C motif chemokine 2	KEGG H01459
CCND1	P24385	G1/S-specific cyclin-D1	KEGG H01459
COL1A1	P02452	Collagen alpha-1(I) chain	KEGG H01459
COL3A1	P02461	Collagen alpha-1(III) chain	KEGG H01459
CXCL8	P10145	Interleukin-8	KEGG H01459
EDN1	P05305	Endothelin-1	KEGG H01459
F3	P13726	Tissue factor	KEGG H01459
FN1	P02751	Fibronectin	KEGG H01459
ICAM1	P05362	Intercellular adhesion molecule 1	KEGG H01459
IL1A	P01583	Interleukin-1 alpha	KEGG H01459
IL1B	P01584	Interleukin-1 beta	KEGG H01459
IL6	P05231	Interleukin-6	KEGG H01459
JUN	P05412	Transcription factor AP-1	KEGG H01459
MAPK1	P28482	Mitogen-activated protein kinase 1	KEGG H01459
MAPK14	Q16539	Mitogen-activated protein kinase 14	KEGG H01459
MAPK8	P45983	Mitogen-activated protein kinase 8	KEGG H01459
MMP2	P08253	72 kDa type IV collagenase	KEGG H01459
NFATC1	O95644	Nuclear factor of activated T-cells	KEGG H01459
NOS3	P29474	Nitric oxide synthase	KEGG H01459
OPRM1	P35372	Mu-type opioid receptor	KEGG H01459
PRKCA	P17252	Protein kinase C alpha type	KEGG H01459
PRKCB	P05771	Protein kinase C beta type	KEGG H01459
RELA	Q04206	Transcription factor p65	KEGG H01459
SELE	P16581	E-selectin	KEGG H01459
SERPINE1	P05121	Plasminogen activator inhibitor 1	KEGG H01459
SNAI1	O95863	Zinc finger protein SNAI1	KEGG H01459
TGFB1	P01137	Transforming growth factor beta-1	KEGG H01459
THBD	P07204	Thrombomodulin	KEGG H01459
TNF	P01375	Tumor necrosis factor	KEGG H01459
VEGFA	P15692	Vascular endothelial growth factor A	KEGG H01459
STAT1	P42224	Signal transducer and activator of transcription 1-alpha/	KEGG H01459
ACE	P12821	angiotensin I converting enzyme	May, 2018: DisGeNET app version v5.1
ADIPOQ	Q15848	adiponectin, C1Q and collagen domain containing	May, 2018: DisGeNET app version v5.1
HSPB3	Q12988	heat shock protein family B (small) member 3	May, 2018: DisGeNET app version v5.1
FCN3	O75636	ficolin 3	May, 2018: DisGeNET app version v5.1
PNPLA3	Q9NST1	patatin like phospholipase domain containing 3	May, 2018: DisGeNET app version v5.1
SREBF1	P36956	sterol regulatory element binding transcription factor 1	May, 2018: DisGeNET app version v5.1
SCN9A	Q15858	sodium voltage-gated channel alpha subunit 9	May, 2018: DisGeNET app version v5.1
IMPACT	Q9P2X3	impact RWD domain protein	May, 2018: DisGeNET app version v5.1
LEP	P41159	leptin	May, 2018: DisGeNET app version v5.1
HSPB2	Q16082	heat shock protein family B (small) member 2	May, 2018: DisGeNET app version v5.1
HSPB1	P04792	heat shock protein family B (small) member 1	May, 2018: DisGeNET app version v5.1
HGF	P14210	hepatocyte growth factor	May, 2018: DisGeNET app version v5.1

NOS1AP	O75052	nitric oxide synthase 1 adaptor protein	May, 2018: DisGeNET app version v5.1		
SLC6A4	P31645	Sodium-dependent serotonin transporter	drugbank	Version 5.1.0	7.02
SLC6A3	Q01959	Sodium-dependent dopamine transporter	drugbank	Version 5.1.0	7.02
SLC6A2	P23975	Sodium-dependent noradrenaline transporter	drugbank	Version 5.1.0	7.02
CYP1A2	P05177	Cytochrome P450 1A2	drugbank	Version 5.1.0	7.02
CYP2D6	P10635	Cytochrome P450 2D6	drugbank	Version 5.1.0	7.02
